# A rapid and stable spontaneous reprogramming system of Spermatogonial stem cells to Pluripotent State

**DOI:** 10.1186/s13578-023-01150-z

**Published:** 2023-12-01

**Authors:** Rui Wei, Xiaoyu Zhang, Xiaoxiao Li, Jian Wen, Hongyang Liu, Jiqiang Fu, Li Li, Wenyi Zhang, Zhen Liu, Yang Yang, Kang Zou

**Affiliations:** 1 Germline Stem Cells and Microenvironment Lab, College of Animal Science and Technology, Nanjing Agricultural University, Nanjing, 210095 China; 2 Stem Cell Research and Translation Center, Nanjing Agricultural University, Nanjing, 210095 China; 3 Institute of Neuroscience, State Key Laboratory of Neuroscience, Center for Excellence in Brain Science & Intelligence Technology, Chinese Academy of Sciences, 320 Yue-Yang Road, Shanghai, 200031 China; 4 State Key Laboratory of Reproductive Medicine and Offspring Health, Nanjing Medical University, Nanjing, 211166 China

**Keywords:** Cell fate, Feeder layer, Germline, Pluripotency, Reprogramming

## Abstract

**Background:**

The scarcity of pluripotent stem cells poses a major challenge to the clinical application, given ethical and biosafety considerations. While germline stem cells commit to gamete differentiation throughout life, studies demonstrated the spontaneous acquisition of pluripotency by spermatogonial stem cells (SSCs) from neonatal testes at a low frequency (1 in 1.5 × 10^7^). Notably, this process occurs without exogenous oncogenes or chemical supplementation. However, while knockout of the *p53* gene accelerates the transformation of SSCs, it also increases risk and hampers their clinical use.

**Results:**

We report a transformation system that efficiently and stably convert SSCs into pluripotent stem cells around 10 passages with the morphology similar to that of epiblast stem cells, which convert to embryonic stem (ES) cell-like colonies after change with ES medium. Epidermal growth factor (EGF), leukemia inhibitory factor (LIF) and fresh mouse embryonic fibroblast feeder (MEF) are essential for transformation, and addition of 2i (CHIR99021 and PD0325901) further enhanced the pluripotency. Transcriptome analysis revealed that EGF activated the RAS signaling pathway and inhibited p38 to initiate transformation, and synergically cooperated with LIF to promote the transformation.

**Conclusion:**

This system established an efficient and safe resource of pluripotent cells from autologous germline, and provide new avenues for regenerative medicine and animal cloning.

**Supplementary Information:**

The online version contains supplementary material available at 10.1186/s13578-023-01150-z.

## Introduction

Spermatogonial stem cells (SSCs) are germline stem cells residing in the seminiferous tubules of the testis, and essential for the maintenance of the male fertility throughout of reproductive life. These cells possess the remarkable capacities for self-renewal and differentiation into spermatozoa through a highly orchestrated process [1]. Accordingly, SSCs are traditionally defined as unipotent stem cells. However, evidence suggests that germ cells may possess pluripotent potential, as they are known to form teratomas [2, 3]. Testicular germ cell tumors, one of the most common solid tumors in young adults [4], are typically accompanied by p53 dysfunction and ectopic expression of the pluripotency marker NANOG [5, 6]. While the fate of SSCs is thought to be closely tied to their niche, no direct evidence of their pluripotency outside this environment has been reported, until a landmark study demonstrated that long-term cultured SSCs could be induced to adopt a pluripotent state, albeit at a very low frequency [7]. These colonies exhibited faster growth than SSCs and were maintained stably when the culture medium was replaced with ES cell medium containing fetal bovine serum (FBS) and LIF. These SSCs derived pluripotent cells exhibited similar characteristics to ESCs, including colony morphology, expression profile of marker genes, and capacity to generate germline chimeras after blastocyst injection. However, it is worth noting that the genomic imprinting pattern of ESCs and embryonic stem-like (ESL) cells differs. Notably, the transformation efficiency of testicular cells from *p53* knockout mice was found to be remarkably increased, and further investigation indicated that the transformation process was related to changes in chromosome stability caused by *p53* deletion and epigenetic modifications such as methylation [8], which is consistent with the observation that suppression of *p53* or its target gene *p21* increases the reprogramming efficiency in establishment of induced pluripotent stem cells (iPSCs) [9]. Compared with iPSCs technology, derivation of ES-like state from SSCs could be spontaneously achieved by modification of culture medium, rather than overexpression of exogenous factors. However, low efficiency of transformation limites the application of SSCs derived pluripotent stem cells in research and clinic. Knockout of *p53* increases transformation efficiency, but simultaneously increases risk in clinic use.

Although the molecular mechanism of SSCs spontaneous transformation was totally unknown at that time, many growth factors in the medium, such as glial cell derived neurotrophic factor (GDNF), basic fibroblast growth factor (bFGF), epidermal growth factor (EGF), and LIF, were believed to be essential for the formation of ESL colonies from neonatal testis cells [7]. Subsequently, different conditions for SSCs long-term culture, including IMDM/FBS culture condition with MEF feeder [10], and IMDM/SFM culture condition without feeder [11, 12], were established, and SSCs lines can be maintained stably in vitro for 2 years with a low mutation rate, typical male imprinting characteristics, and capacities of self-renewal and spermatogenesis. Importantly, these long-term cultured SSCs do not transform into pluripotent state during the process of culture, probably because some growth factors, including EGF and LIF, were removed [13]. In past years, we established a SSCs culture system with minor modification of IMDM/FBS medium [10]. SSCs cultured in this medium with MEF feeder layers were stably maintained for more than 40 passages, and effectively retained the normal expression profile and the capacity of spermatogenesis. However, a few colonies similar to ES colony frequently appeared around 25 passages, albeit that EGF and LIF are not included in the medium. In this culture system, the transformation efficiency of *p53* deficient SSCs was also higher than that of wild type SSCs, consistent with reported observations [7]. Through ATAC-seq and RNA-seq analysis, we recently demonstrated that SMAD3 was activated and played an important role in the late stage of transformation, but in the initiation step the expression and the phosphorylation of SMAD3 were in relatively low levels in *p53* deficient SSCs, implying that a prerequisite is required to activate SMAD3 to drive SSCs transformation [14].

In this study, we conclude that four key factors (stable maintenance in our modified SSCs culture medium, EGF, LIF, and fresh MEF feeder) are crucial for the reprogramming of SSCs into pluripotent cells. Notably, primary SSCs failed to convert under this condition, and only SSCs cultured in our modified SSCs medium for at least five passages were able to efficiently transform after culturing in transformation medium for another five passages. These transformed cells grew stably and rapidly on MEF feeders and formed colonies morphologically similar to epiblast stem cell colonies. They highly expressed pluripotent marker genes such as *Nanog* and *Sox2*, and could generate teratoma in nude mice and participate in embryonic development. As a result, we called them as germline stem cells derived pluripotent cells (GSPCs). Interestingly, after replacement with standard ES medium (DMEM + FBS), GSPCs converted into ES-like colonies. These ES-like cells proliferated rapidly, highly expressed pluripotent markers, and generated teratoma more efficiently. Transcriptomic analysis revealed that the gene expression profiles of GSPCs and ES-like cells were also very similar to that of ESCs. Furthermore, the “2i” medium which maintains ESC in the naïve state [15] further enhanced the expression level of NANOG in both GSPCs and ES-like cells. Transcriptome analysis and molecular assays were employed to investigate the biological characteristics of these two types of pluripotent cells, and to explore the molecular underpinning of transformation.

In summary, we established a rapid and stable transformation system to derive pluripotent stem cells from germline stem cells, and demonstrated pivotal signaling pathways, which raises the potential in translational medicine and animal science.

## Results

### SSCs spontaneously transform into ES-like state in the modified culture system

**Fig. 1:**
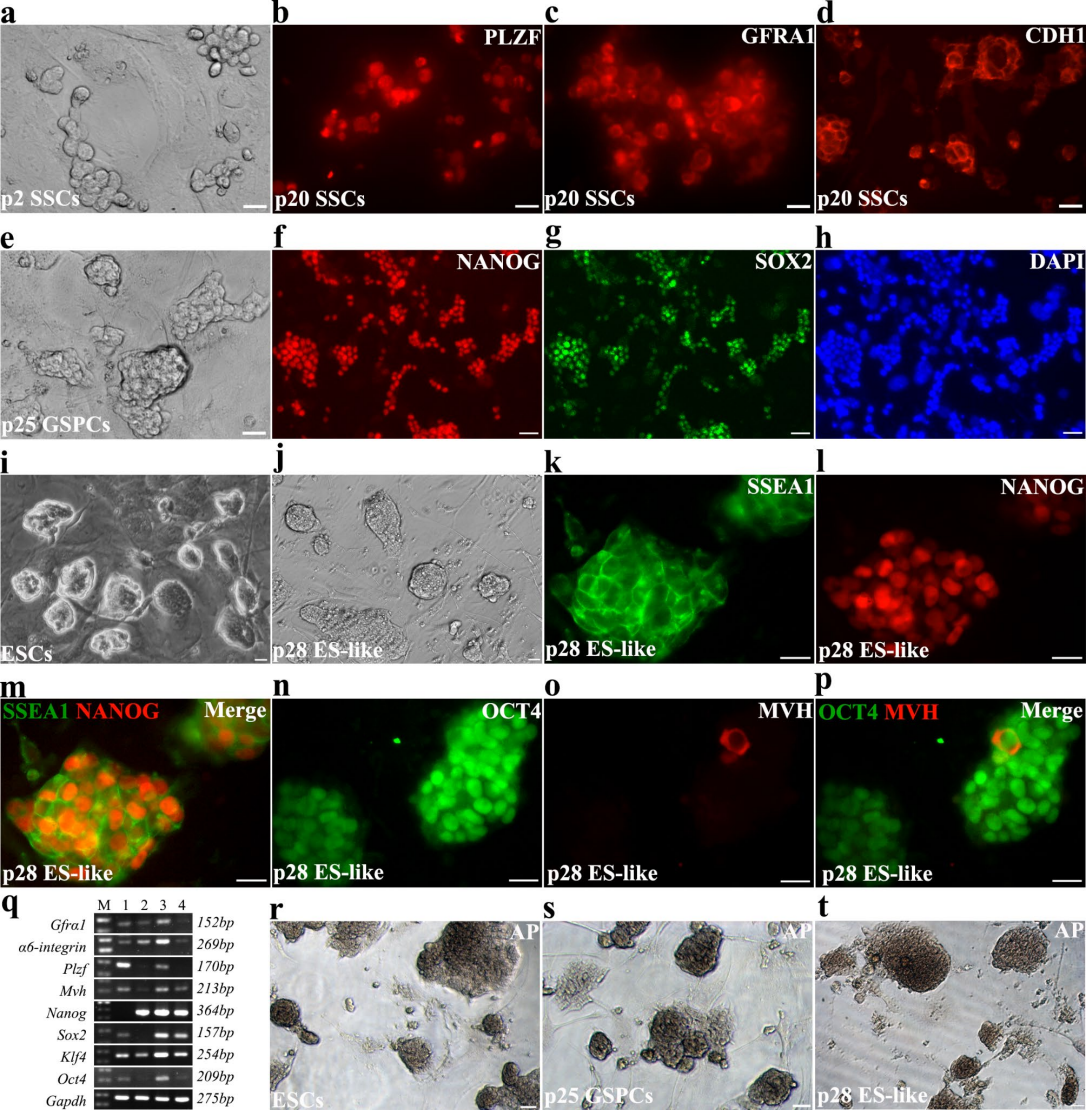
Newly isolated SSCs transformed into pluripotent state during long-term culture. **a-d** Newly isolated SSCs were purified and plated on MEF feeder (**a**), and were identified with IF staining using antibodies against PLZF (**b**), GFRA1 (**c**) and CDH1 (**d**). **e** Typical colonies of transformed cells from long-term cultured SSCs were exhibited. **f-h** Identification of transformed pluripotent cells using dual IF staining of OCT4 (**f**), SOX2 (**g**) and DAPI (**h**). **i-j** The morphology of ESCs (**i**) and ES-like cells derived from transformed pluripotent cells (**j**) on MEF feeder were exhibited. **k-p** The expression of pluripotent and germline markers (**k**. SSEA1, **l**. NANOG, **m**. merge; **n**. OCT4, **o**. MVH, **p**. merge) in ES-like cells were detected using IF staining. **q** The expression of germline and pluripotent markers were determined using RT-PCR (M. marker, 1. newly isolated SSCs, 2. GSPCs derived from long-term culture, 3. ES-like cells derived from GSPCs, 4. H_2_O). **r-t** The alkaline phosphatase activity was detected in ESCs (**r**), GSPCs (**s**) and ES-like cells (**t**). Scale bar = 20 μm

We established several mouse SSC lines from neonatal testis using IMDM condition [10] with minor modification (medium 1, see component in Table S1). SSCs isolated from testes of 5 days mice stably grew on MEF feeder layer, and formed grape-like colonies within 2–3 passages (Fig. 1a). They could be passaged in vitro for more than 40 passages and stably expressed SSCs markers such as PLZF, GFRA1 and CDH1 (Fig. 1b-d). Primary SSCs in this medium were passaged every 5–7 days, and after 20 generations could be passaged 3–4 days (in a ratio of 1:3 − 1:5). During in vitro culture, typical SSCs colonies were stably maintained (cellular boundary is relatively clear and colonies are relatively loose) (Figure S1a-b), verified by the capacity of germline reconstitution through testis transplantation (Figure S1c-e). Interestingly, a few of compact colonies (less than 10% of total SSCs colonies) were occasionally observed after long-term culture (usually need more than 25 passages), and this type of colonies became dominant after another 4–5 passages (Fig. 1e). Immunofluorescence staining demonstrated that they expressed NANOG and SOX2 (Fig. 1f-h), reflecting that they were in pluripotent state. We subsequently picked these colonies to MEF feeder and cultured them in ESCs medium. Gradually, colonies became compact with high density, the boundary of each cell could hardly be distinguished (Fig. 1j), which were very similar to typical ESCs colonies (Fig. 1i). The expression of pluripotent markers SSEA1 and NANOG indicated these ES-like cells as pluripotent cells (Fig. 1k-m). Majority of these cells highly expressed OCT4, and only a very small number of cells expressed germline marker MVH (Fig. 1n-p), indicating that most cells have lost the characteristics of germline cells. Subcutaneous injection of these cells into nude mice induced teratoma (Figure S2a-b). Both pluripotent cells generated after long-term culture and ES-like cells derived from transformed cells highly expressed pluripotent markers, *Nanog*, *Sox2*, *Klf4* and *Oct4* (Fig. 1q), and had high alkaline phosphatase activity (Fig. 1r-t), similar to ESCs. Based on these observations, we confirmed that SSCs have transformed into pluripotent state, and named them as germline stem cells derived pluripotent cells (GSPCs). However, expression of very low levels of germline markers, such as *Gfrα1*, *α6-integrin*, *Plzf* and *Mvh* (Fig. 1q), indicated that a few germ cells were still not transformed, yet.

This phenomenon is consistent with reported observation [7], which is interesting, since no exogenous gene or chemical molecule is required to be introduced in. Germline stem cells are believed to retain the potential of pluripotency [2], and GSPCs occasionally appeared in culture for unknown reasons. Therefore, we proposed that some key factors in the culture medium induced the pluripotency of SSCs during culture in vitro.

### Supplementation of EGF/LIF increased the efficiency of spontaneous reprogramming of SSCs

**Fig. 2:**
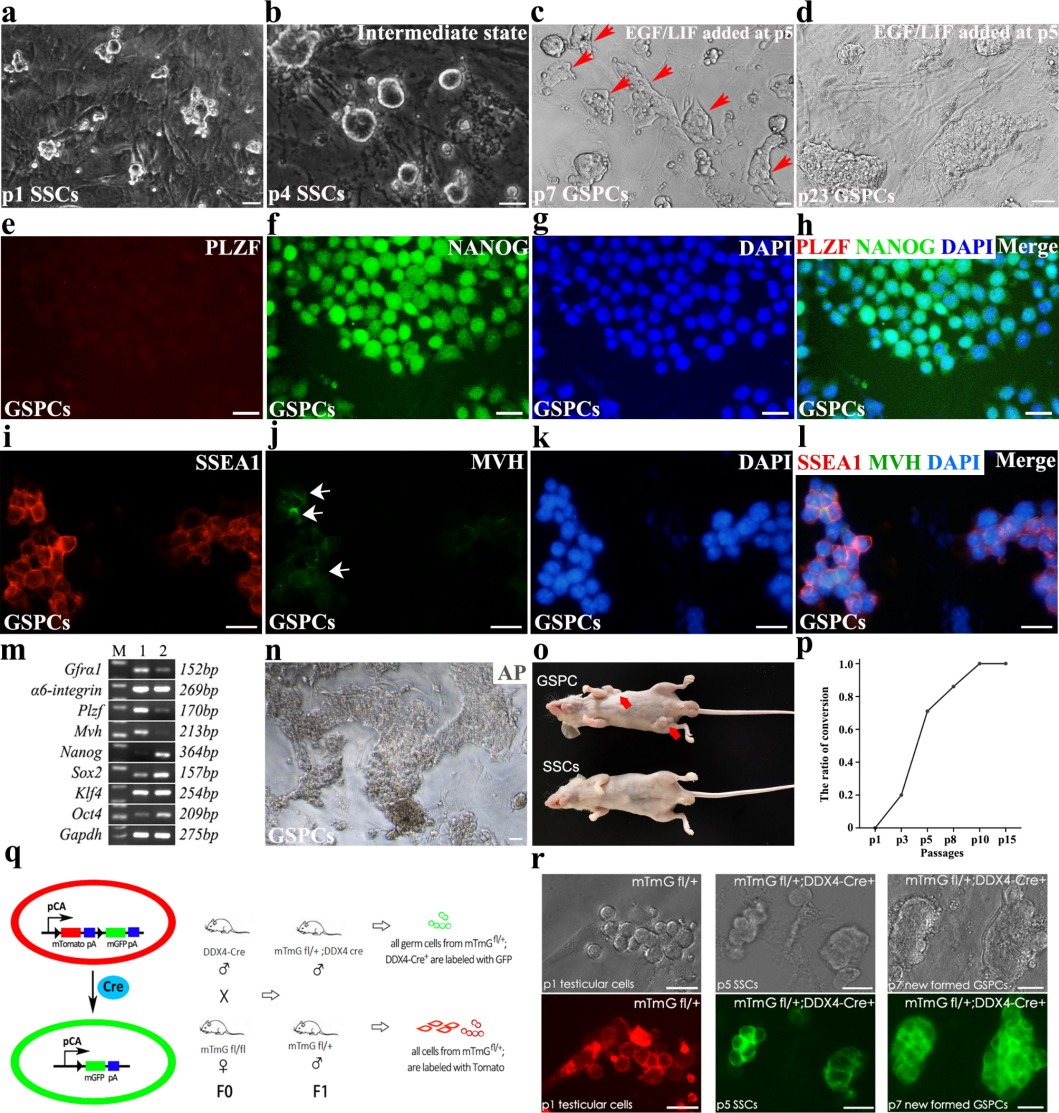
Addition of EGF and LIF effectively accelerated SSCs transformation. **a** The morphology of newly isolated SSCs maintained on MEF feeder with medium1 after 1 passage was exhibited. **b**, SSCs colonies on MEF feeder with medium 1 for 4 passages, and medium 1 was replaced with medium 2, were exhibited. **c** After another 3 passages in medium 2, some GSPCs colonies appeared (red arrow heads). **d** The representative GSPCs colonies after long-term culture were exhibited. **e-l** The expression of NANOG, SSEA1 and MVH in stable GSPCs was detected using IF (**e**. PLZF, **f**. NANOG, **g**. DAPI, **h**. merge; **i**. SSEA1, **j**. MVH (arrows indicate a few MVH^+^ cells), **k**. DAPI, **l**. merge). **m** The expression levels of *Gfrα1*, *α6-integrin*, *Plzf, Mvh*, *Nanog*, *Sox2*, *Klf4, Oct4* and *Gapdh* in SSCs and GSPCs were determined using RT-PCR (M. marker, 1. SSCs, 2. GSPCs). **n** Alkaline phosphatase activity of stable GSPCs derived from SSCs cultured in medium 2 was detected. **o** GSPCs derived in medium 2 induced teratoma in nude mice as early as 8–10 days, while SSCs cultured in medium 1 could not induce teratoma. **p** The conversion ratio of SSCs cultured in medium 1 was statistically analyzed. **q** The strategy to make germline specific GFP mice and isolate GFP labeled SSCs. **r** Tracing the formation of GFP labeled GSPCs under medium 2 condition. The total cells from mTmG^fl/+^ mice indiscriminately expressed tomato (left), while SSCs from mTmG^fl/fl^;*Ddx4*-Cre^+^ mice specifically expressed GFP (middle), and when they transformed in medium 2, GFP signal was only observed in GSPCs (right). Scale bar = 20 μm

Since spontaneous transformation into pluripotent state was associated with medium components, and previous study revealed that *p53* deficiency accelerates transformation [7], we therefore hypothesized that some factors may affect the transformation efficiency. Based on the published papers [7, 10, 12, 16] and our experience, we screened many candidate growth factors, and after several rounds of screening finally found that the addition of 10 ng /ml LIF and 20 ng /ml EGF to medium 1 (medium 2) was able to effectively cultivate the SSCs cultured on fresh MEF feeder layers to transform into pluripotent state. Importantly, primary SSCs are required to be stably maintained on fresh MEF feeder in medium 1 for at least 5 passages (about 35–40 days) to adapt to the in vitro culture condition, and then the medium was replaced with medium 2 containing LIF and EGF. About 3 passages later (around 15 days), around 30% colonies distinct to normal SSCs morphology could be observed (Fig. 2c), which were identical to those derived from long-term culture in medium 1 (Fig. 1e). However, they became dominant colonies after 2–3 passages, which were more efficient than those derived from long-term cultured cells in medium 1 (less than 10% colonies transformed after 25 passages, and took another 4–5 passages to become dominant). The adhesion to both neighbor cells and feeder layers increased, and cell boundary was indistinguishable. After further culture, the majority of cell clusters gradually converted into compact colonies (Fig. 2d), which resembled the epiblast cell colonies. Similar to the GSPCs derived from long-term culture in medium 1, these GSPCs highly expressed pluripotent markers including NANOG and SSEA1, and expressed a very low ratio of MVH and hardly expressed PLZF (Fig. 2e-l). Intensive expression of pluripotent markers, *Nanog*, *Sox2*, *Klf4* and *Oct4*, further verified the pluripotent characteristics of GSPCs, whereas weak expression levels of SSC and germline markers (*Gfrα1*, *α6-integrin*, *Plzf* and *Mvh*) suggested that a few untransformed germ cells were probably remained (Fig. 2m). Both SSCs and GSPCs have normal diploid karyotype (Figure S4a), excluding the fates of meiosis or tumorisation. GSPCs derived in medium 2 also expressed alkaline phosphatase (Fig. 2n), and induced teratoma in nude mice after intraperitoneal injection (Fig. 2o). GSPCs derived in medium 2 were able to be stably maintained in vitro for more than 40 passages with a sharper growth curve, compared to GSPCs derived from long-term culture (Figure S2c), probably due to the faster proliferation rate (1:5 − 1:10 subculture every 48–72 h).

Notably, we found that not only the passages of MEF (less than three passages) were pivotal for SSCs transformation, but also the passages of SSCs remarkably affected the transformation efficiency. Newly isolated SSCs on fresh MEF feeder layer hardly yield GSPCs colony in medium 2 (0 of 12 attempts), whereas SSCs cultured on fresh MEF feeder layer for 5 passages in medium 1, could transform within 3–4 passages after replacement of medium2 with a successful rate of over 70% (10 of 14 attempts), and p8 (8 passages) SSCs cultured in medium 1 transformed even more efficiently within p5 SSCs (12 of 14 attempts). It was worth noting that all attempts of SSCs more than 10 passages in medium 1 transformed into GSPCs within 5 passages in medium 2 (Fig. 2p). These observations indicate that stable maintenance in medium 1 is a prerequisite for SSCs transformation in medium 2, and longer culture in medium 1 leads to higher transformation efficiency, which is in agreement with the conclusion that acquirement of the indefinitely proliferative capacity is important for the self-renewal of pluripotent stem cells [17]. Collectively, the transformation efficiency and transformation time of each condition in study were summarized, demonstrating a higher efficiency than reported transformation systems (Table 1).

**Table 1:** Comparison of the transformation time and efficiency

Conditions	Total time of culture	Transformation frequency
Primary testicular cells	appear in 1-2 M, dominant in 2-3 M	occasional [7]
SSCs cell line from neonatal testis	5 M	not observed [7]
p53 KO SSCs from neonatal testis	6 M	2 out of some experiments [7]
p53 KO SSCs from mature testis	6 M	50% (4 out of 8 experiments) [7]
SSCs from neonatal testis in med 1	4 M (25 passages)	27.5% (11 out of 40 experiments)
SSCs from neonatal testis in med 1 for 5 passages and changed med 2	1.5 M dominant (10 passages: 5 in med 1, 5 in med 2)	71.4% (10 of 14 experiments)
SSCs from neonatal testis in med 1 for 8 passages were changed med 2	1.8 M dominant (13 passages: 8 in med 1, and 5 in med 2)	85.7% (12 of 14 experiments)
SSCs from neonatal testis in med 1 for 10 passages were changed med 2	dominant in 2 M (15 passages: 10 in med 1, and 5 in med 2)	100% (6 of 6 experiments)

M: months; Med: medium

All the culture conditions above require MEF feeder layers

To exclude the possibility that GSPCs were derived from MEF, fresh MEF were cultured in medium 2 for more than 6 passages, or mitomycin-C treated MEF were cultured with medium 2 for more than 20 days, until cell death, no transformed pluripotent stem cells was observed. Moreover, SSCs from mTmG^fl/fl^;*Ddx4*-Cre^+^ mice, which specifically express GFP in germ cells and express Tomato in somatic cells (Fig. 2q), were used to trace the fate of SSCs during culture. The results exhibited that all the colonies were GFP-labelled (Fig. 2r), excluding the possibility of somatic cells or MEFs reprogramming. Thus, we confirmed that addition of EGF/LIF to our modified IMDM medium remarkably increased the transformation efficiency of SSCs into pluripotent state.

### Conversion of GSPCs to ES-like state under standard ESC culture condition

**Fig. 3:**
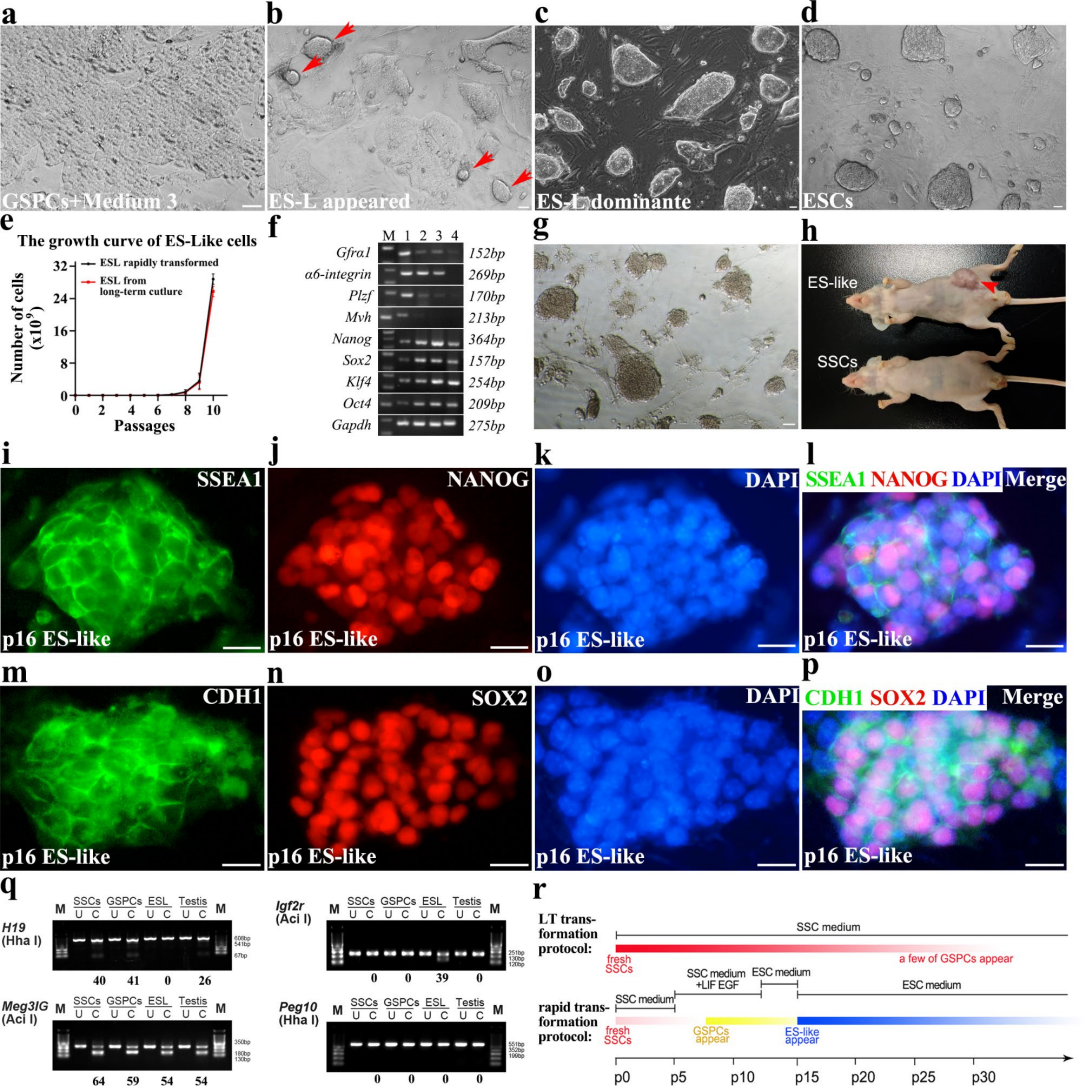
ES medium induced GSPCs to transform into ES-like state. **a** The morphology of GSPCs cultured in medium 3 for 1 passage was exhibited. **b** ES-like colonies appeared in GSPCs cultured in medium 3 for 3–4 passages. **c** ES-like colonies became dominant after a few passages. **d** The typical ES colonies were exhibited. **e** The growth curve of ES-like cells was exhibited. **f** The expression levels of *Gfrα1*, *α6-integrin*, *Plzf, Mvh*, *Nanog*, *Sox2*, *Klf4*, *Oct4* and *Gapdh* were determined using RT-PCR (M. marker, (1) SSCs, (2) GSPCs, (3) ES-like cells, (4) ESCs). **g** Alkaline phosphatase activity was determined in ES-like cells derived from GSPCs. **h** ES-like cells derived from GSPCs induced teratoma in nude mice, while SSCs cultured in medium 1 could not induce teratoma. **i-p** IF staining assays detected the expression of pluripotent markers in ES-like cells derived from GSPCs (**i**, SSEA1. **j**, NANOG. **k**, DAPI. **l**, merge; **m**, CDH1. **n**, SOX2. **o**, DAPI. **p**, merge). **q** COBRA demonstrated the parental imprinting characteristics of SSCs, GSPCs and ES-like cells. **r** The schematic procedure of SSCs transformation into GSPCs and ES-like was summarized. Scale bar = 20 μm

Interestingly, ES-like cells transformed by Shinohara’s system could be maintained in vitro only in standard ESCs medium [7]. Therefore, stable GSPCs (usually need another 6–7 passages in medium 2 from the appearance of GSPCs colonies) were transferred into standard ESC medium (medium 3) on MEF feeder to see what happen. In the new condition, a small number of cells underwent apoptosis within the first 2–3 passages, and simultaneously some compact colonies similar to typical ES colonies formed (Fig. 3a-b). ES-like colonies usually became dominant after another 3–4 passages, and could be stably maintained under this condition (Fig. 3c), with the morphology indistinguishable from ESCs colonies (Fig. 3d). These ES-like cells proliferated rapidly, with an average subculture time of 3 days, identical to those generated using Shinohara’s protocol (Fig. 3e). Moreover, their expression profiles of marker genes (Fig. 3f), karyotype (Figure S4a) and alkaline phosphatase activity (Fig. 3g) were identical to ES-like derived from long-term culture condition. ES-like cells also generated teratomas in nude mice (Fig. 3h), confirming their pluripotency. Intensive expression of SSEA1, NANOG, CDH1 and SOX2 further confirmed their pluripotency identity (Fig. 3i-p). A very low proportion of MVH^+^ cells were detected in colonies (Figure S3a-c), which probably resulted from a few of untransformed SSCs in the colonies.

To analyze the imprinting pattern of GSPCs and ES-like cells, two paternally imprinted regions (*H19* and *Meg3 IG* regions) and two maternally imprinted regions (*Igf2r* and *Peg10* regions) were examined by combined bisulfite restriction analysis (COBRA). GSPCs possessed a similar imprinting pattern with SSCs, which exhibited a typical paternally methylation status in differentially methylated regions (DMRs), whereas ES-like cells had both paternal and maternal imprinting patterns (*Meg3 IG* and *Igf2r* regions) (Fig. 3q). These results implied that GSPCs maintained the paternal imprinting characteristics of SSCs, which were remarkably changed in ES-like cells.

It was worth noting that ES-like colony was not observed after directly replacing SSCs medium with ESCs medium in primary or long-term cultured SSCs, since SSCs failed to survive in ESC medium. This indicates that transformation into GSPCs state probably is an essential step for ES-like formation. Based on these observations, we summarized the schematic procedure of SSCs transformation (Fig. 3r).

### Comparison of the pluripotency of GSPCs and ES-like cells

**Fig. 4:**
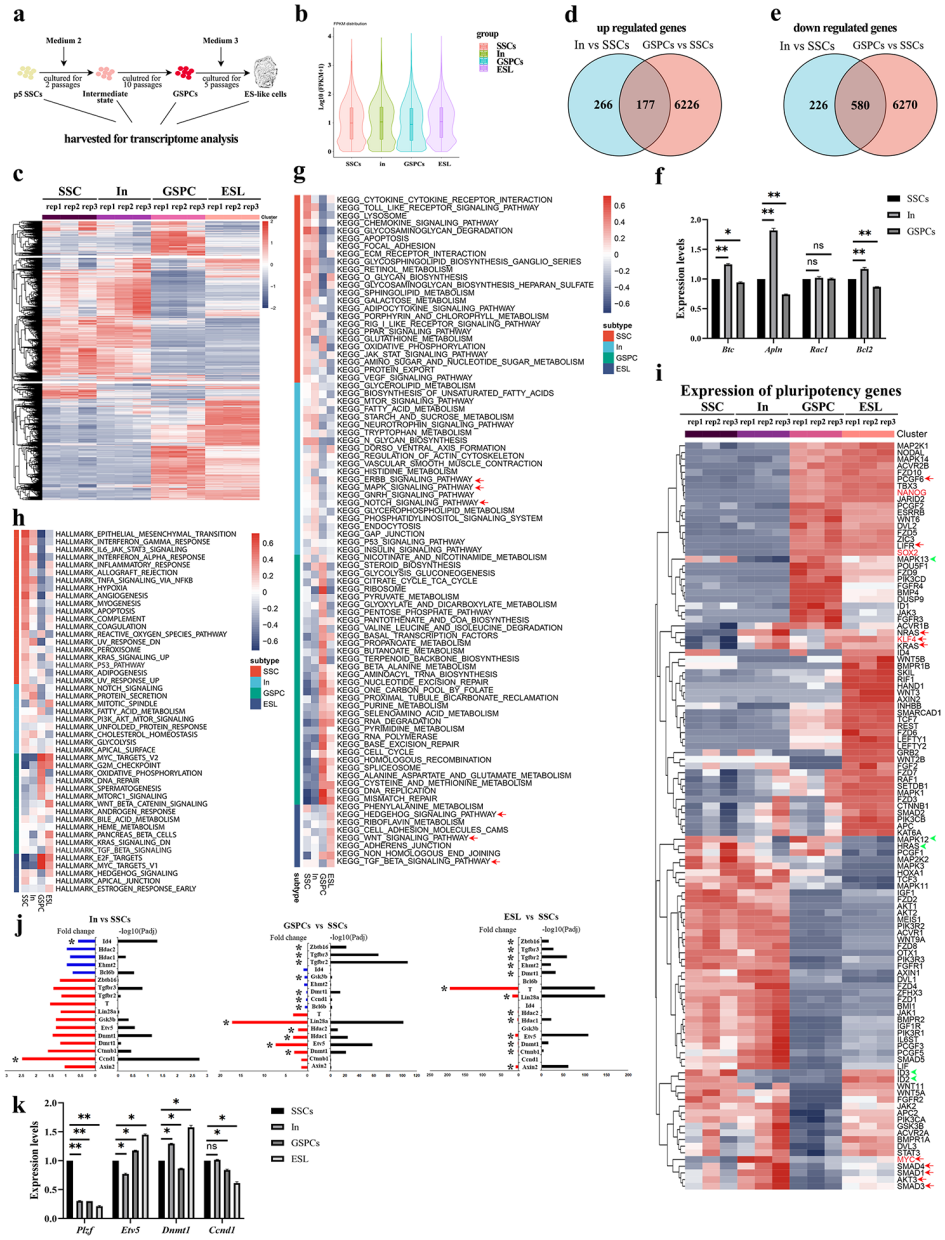
Analysis of transcriptomic characteristics of SSCs, intermediate state cells, GSPCs and ES-like cells. **a** The illustration of sample collection for RNA-sequencing. **b-c** Violin Plot (**b**) and heatmap (**c**) of DEGs identified from the SSCs, In, GSPCs and ES-like cells. **d-e** Venn diagrams summarized the up-regulated genes (**d**) and down-regulated genes (**e**) in In vs. SSCs and GSPCs vs. SSCs. **f** The relative expression levels of *Btc*, *Apln*, *Rac1* and *Bcl2* genes in SSCs, In state cells and GSPCs were determined using RT-PCR, and were statistically analyzed, *p < 0.05; **p < 0.01. **g** KEGG of differential genes in SSCs, In state cells, GSPCs, ES-like cells and shared by two datasets. **h** Hallmarks of differential genes in SSCs, In state cells, GSPCs and ES-like cells. **i** The relative expression levels of pluripotency associated genes in SSCs, In state cells, GSPCs and ES-like cells were exhibited. **j** Representative DEGs in SSCs, In, GSPCs and ES-like cells were selected. Left, fold change. Blue, downregulated genes; red, upregulated genes. Right, false discovery rate (FDR)-adjusted *p* values determined using DESeq2 (–log10-transformed). * represents a remarkable difference. **k** The relative expression levels of *Plzf*, *Etv5*, *Dnmt1* and *Ccnd1* genes in SSCs, In state cells, GSPCs and ES-like cells were determined using RT-PCR, and were statistically analyzed, *p < 0.05; **p < 0.01

Although GSPCs and ES-like cells exhibited characteristics of pluripotent stem cells, it’s not clear whether they were different in hierarchy of pluripotency, or just morphologically distinct induced by culture medium. To determine their pluripotency levels, we first compared the growth condition of GSPCs and ES-like cells. Both of these cells could be stably maintained in vitro for more than 30 passages, and the average subculture time was 2–3 days for GSPCs and ES-like cells. Similar to ESCs, GSPCs and ES-like also highly expressed pluripotent markers, including OCT4, NANOG and SOX2. To further determine the pluripotency of these cells, ESCs, ES-like, GSPCs and SSCs were subcutaneously injected into nude mice to test the efficiency of teratoma formation. Notably, the capacity to generate teratomas of GSPCs and ES-like cells was remarkably higher than that of ESCs. Teratomas could be observed 7–10 days post GSPCs or ES-like cells injection, while ESCs need at least 15–20 days to generate visible teratomas (Table 2). This observation indicated a higher homogeneity of GSPCs and ES-like cells than ESCs [18]. Finally, we tested the potential of blastocyst development. ESCs, GSPCs and ES-like were labeled with GFP and sorted for establishment of GFP + clones in vitro, and were microinjected into 8-cell stage blastocysts (2.5 d.p.c). The results demonstrated that GSPCs, ES-like and ESCs cells could participate in embryo development (Fig. 4Sb-d), further confirming their pluripotency.

**Table 2:** Comparison of teratoma formation efficiency

Cell types	Culture conditions	Average days to observe teratoma (n = 5)
SSCs	medium 1	
GSPCs	medium 2	8–10 days
ES-Like cells	medium 3	7–8 days
ESCs	medium 3	15–20 days

### Comparison of gene expression profiles of SSCs, transforming SSCs, GSPCs and ES-like cells through transcriptomic analysis

To reveal the connection between EGF/LIF signals and SSCs spontaneous reprogramming, transcriptome analysis was conducted. Differential expression genes (DEGs) of SSCs (5 passages in medium 1), intermediate state cells (5 passages in medium 1, and 2 passages in medium 2, referred to as “In”), GSPCs (5 passages in medium 1, and 10 passages in medium 2), and ES-like cells (GSPCs cultured for 10 passages in medium 3) were screened to reveal this relationship (Fig. 4a). The results of gene expression analysis indicated that SSCs and In state cells have similar transcriptomic profiles, which are distinctly different from those of GSPCs and ES-like cells (Fig. 4b-c). This analysis revealed that In state cells might be in the initiating stage of SSCs transformation, and transformed into a completely different cell type (GSPCs) after eight passages in LIF/EGF medium. Furthermore, the gene expression profile partially changed when ESC medium was replaced.

Using these transcriptome data, we further compared the gene expression profiles of GSPCs and ES-like cells with ESCs, the well characterized pluripotent cells. We obtained RNA-seq datasets of wild-type mouse embryonic stem cells from the Gene Expression Omnibus (GEO) database [19] and performed a comparative analysis with our study’s dataset. After eliminating batch effects among the data, principal component analysis (PCA) was conducted on individual samples (Figure S5a). The results revealed distinct clustering of the four groups, with the SSCs group significantly separated from the other three, suggesting a unique expression pattern in the SSCs group. The samples of the ESCs group were positioned between the GSPCs and ES-like cells groups, indicating a close similarity of their expression pattern. Furthermore, the gene expression heatmap among samples (Figure S5b) demonstrated a high overlap in expression patterns between the ESCs and GSPCs groups, with hierarchical clustering showing a closer similarity between ESCs and GSPCs. The sample correlation heatmap (Figure S5c) indicated a significant positive correlation between the ESCs group and ES-like cells and GSPCs groups, with correlation coefficients reaching average values of 0.960 and 0.968, respectively, but a lower correlation with the SSCs group.

Furthermore, we compared the expression levels of some representative genes associated with pluripotency in SSCs, GSPCs, ES-like cells and ESCs (Figure S5d), and observed that typical genes for pluripotency, e.g., *Nanog*, *Klf4*, *Sox2*, *Esrrb* and *Lifr*, were remarkably activated in all of these cells, confirming their pluripotent state. However, we also noticed that *Bmp4*, *Mapk15* and *Pou5f1* were mainly highly expressed in GSPCs, while many genes in Wnt signaling pathways (*Wnt3a*, *Axin2*, *Apc*, *Gsk3b*, *Tcf7* etc.,) and in MAPK signaling pathway (*Mapk3k3*, *Mapk6*, *Mapk8*, *Mapk9* etc.,) were strongly expressed in ES-like cells. Most of these genes were also expressed in ESCs, but the expression levels were lower than in ES-like cells. The gene expression heatmap revealed that, compared to GSPCs, ES-like cells are closer to ESCs in the expression pattern of pluripotency associated genes.

Collectively, these results indicate that gene expression characteristics of GSPCs and ES-like cells are similar to pluripotent stem cells.

### Analysis of the transcriptomic changes during SSCs transformation

To reveal the molecular events during SSCs transformation, we compared the DEGs of In state cells with SSCs and GSPCs. The Venn diagram further highlighted the similarities and differences of gene expression profiles of SSCs, In state cells, and GSPCs. Notably, the number of differentially expressed genes in In state cells was remarkably less than that in GSPCs (443 up-regulated in In state vs. 6403 up-regulated in GSPCs, 1:14.45; 806 down-regulated in In vs. 6850 down-regulated in GSPCs, 1:8.49) (Fig. 4d and e). Among them, genes that were uniquely differentially expressed in In state cells (266 up-regulated and 226 down-regulated) probably represent key genes for the initiation of transformation (refer to Table S4 and S5). The enhanced expression of EGF and EGFR-associated genes (*Areg*, *Btc*, *Eps8*, and *Ereg*) [20] in In state cells suggested that EGF might function earlier than LIF signal in SSCs transformation. Additionally, several GTPase or G-protein receptor-associated genes (*Apln*, *Appl2*, *Arhgap6*, *Arhgap18*, *Arhgap22*, *Dock8*, *Gpr137b*, *Stard13*, and *Vav3*) [21–25] were up-regulated in In state cells, while the expression levels of *Rgs1* (the activator of GTPase activity) [26] and *Arhgap30* (stimulate GTP-hydrolysis on RAC1 to inactivate RAC1 activity) [27] were decreased. These findings indicate the potential role of the G-protein family in the onset of SSCs transformation, with *RAC1* being a probable key gene involved in transformation. Our transcriptome analysis revealed that *Smad3* and *Ltbp1*, the regulators for TGF-β activation [28], were up-regulated. These findings are consistent with our previous study, which demonstrated that SMAD3 plays a crucial role in the transformation of SSCs. Furthermore, we observed an increase in the expression of genes associated with cell survival, such as *Nabp1* [29], *Bcl2* [30], *Dclre1b* [31], *Rrm2b* [32], and *Styk1* [30], and a decrease in the expression of apoptotic or death-associated genes, including *Card9* [33], *Dapk1* [34], *Klf15* [35], *Tnfrsf21* [36], and *Usp13* [37]. Moreover, we selectively detected the expression levels of these genes (*Btc*, *Apln*, *Rac1* and *Bcl2*) using RT-PCR [Fig. 4f], and confirmed the consistency with expression change of transcriptomic analysis. From these results we concluded that the increased proliferative capacity and anti-apoptotic capacity are essential for SSCs transformation.

We also observed an up-regulation of genes promoting proliferation, such as *Arid5a* [38], *Ccnd1* [39], *Foxm1* [40], and *Prkca* [41], and a down-regulation of cyclin inhibitor *Cdkn1c* [42], suggesting that increased proliferation is a key feature of SSCs transformation. Additionally, we found that the expression levels of many members in the MAPK and Wnt signaling pathways were significantly altered. For instance, *Lgr6*, *Mapk6*, *Map3k15*, and *Mapk13* were up-regulated, while *Dact1*, *Gpc3*, *Lefty1*, *Lyn*, *Ptk2b*, *Six2*, and *Wnt5b* were down-regulated. These results suggest that the MAPK and Wnt signaling pathways play a role in the initiation of SSCs transformation. Finally, we identified 177 up-regulated genes in In state cells compared to SSCs and GSPCs compared to SSCs, which may be pivotal for the late stage of transformation or GSPCs maintenance. These genes include *Brca2*, *Cbx3*, *Ccdc18*, *Ccnb1*, *Ccne2*, *Cdc7*, *Klf4*, and *Sirt1*, which are well-known factors associated with pluripotency.

To gain further insights into the molecular mechanisms underlying SSCs transformation, we analyzed the FPKM values of genes in SSCs, In state cells, GSPCs and ES-like cells (Table S6), and identified enriched gene functions through KEGG analysis (Fig. 4g). Gene categories that were highly expressed in SSCs, such as cytokine-cytokine receptor interaction and chemokine signaling pathway, were down-regulated in In state cells and further decreased in GSPCs, suggesting a change in essential growth factors during the transformation process. We then focused on the potential signaling pathways that may play a pivotal role in the transformation process, and found that the average expression levels of genes belonging to mTOR, ERBB (also known as EGFR), MAPK, GnRH, and insulin signaling pathways were up-regulated in In state cells, indicating their involvement in the initiation of SSCs transformation. In GSPCs, transcriptional activation of signaling pathways related to carbohydrate metabolism, amino acid metabolism, DNA repair, cell cycle, and ribosome, indicated that metabolic changes might be essential in this phase. Furthermore, we observed a significant increase in the average expression levels of genes in Hedgehog, Wnt, and TGF-β signaling pathways in ES-like cells. The compact structure of ES-like cell colonies might be attributed to the up-regulation of genes associated with cell adhesion molecules CAMs and adherens junction.

The molecular characteristics of cells at different stages of transformation were analyzed, and the differentially expressed genes (DEGs) were identified (Fig. 4h). In SSCs, genes associated with Epithelial-mesenchymal transition (EMT), IL6-JAK-STAT signaling, hypoxia, apoptosis, p53 signaling pathway, Notch signaling pathway, and Glycolysis were detected. The expression levels of these genes gradually decreased from In state cells to GSPCs, and to ES-like cells. The PI3K-AKT-mTOR signaling pathway was activated in the intermediate state, indicating its involvement in the initiation of SSCs transformation. In GSPCs, the dominant categories were associated with pluripotency, cell cycle (G2M checkpoint), DNA repair, and spermatogenesis. In ES-like cells, the Wnt and TGF-β signaling pathways were activated, and the up-regulation of genes associated with cell adhesion molecules CAMs and adherens junction likely caused the compact structure of ES-like cells colonies. The expression patterns of the DEGs demonstrated the pivotal signaling pathways of each stage in the transformation processes. Interestingly, the trends of gene expression changes were consistent with observations from *p53* knockout model, suggesting that the different transformation models of SSCs (*p53* knockout or LIF/EGF stimulation) probably shared similar signaling pathways.

To investigate the key signaling pathways involved in the initiation of SSCs transformation, we compared the expression patterns of pluripotency-associated signaling pathways, including Wnt, Ras, TGF-β, and JAK-STAT signaling pathways (Figure S5). We analyzed the expression changes of genes associated with pluripotency from SSCs to In state cells and found that genes in the Wnt and TGF-β signaling pathways were activated, accompanied by transcriptional activation of pluripotent genes such as *Sox2*, *Nodal*, and *Esrrb*, while the expression levels of genes in the JAK-STAT signaling pathway were down-regulated. Additionally, some genes in the RAS signaling pathway were up-regulated, while others were down-regulated. These observations suggest that Wnt, TGF-β, and RAS signaling pathways may be involved in the initiation of SSCs transformation. Although JAK-STAT is essential for SSCs proliferation [43], it becomes dispensable for the initiation of SSCs transformation, GSPCs, and ES-like maintenance, suggesting that other signaling pathways, such as Wnt and TGF-β signaling pathways, may promote proliferation activity of GSPCs and ES-like cells.

Furthermore, the expression levels of genes associated with pluripotency were analyzed to seek the clues of transformation. The expression difference among SSCs, transforming cells (In state) and transformed cells (GSPCs, ESL) was significant (Fig. 4 g-i). Although SSCs and In state cells exhibited similar expression profiles of these pluripotent genes, we noticed that several genes were specifically activated in In state, including *Nras*, *Klf4*, *Kras*, *GSK3*β, *Myc*, *Akt3*, *Smad1*, *Smad3* and *Smad4* (Fig. 4i), implying they were pivotal genes for the initiation of SSCs transformation. Notably, *Myc* and *Klf4* were highly expressed in In state, and remarkably down-regulated in GSPCs, indicating that they are essential for SSCs transformation, but dispensable for GSPCs maintenance. Since *Myc* [44] and *Klf4* [45] have been identified as the targets of RAS, and RAS is the downstream molecule of EGF signaling pathway [46], there is a possibility that *Myc* and *Klf4* were activated by *Nras* and *Kras* through EGF-RAS-MEK signaling pathway in the initiation of SSCs transformation. Combined with the KEGG results showing that EGFR and MAPK signaling pathways were predominantly activated in In state (Fig. 4g), we proposed that EGF-RAS-MAPK and PI3K-AKT signaling pathways were decisive for the initiation of SSCs transformation. Although the expression levels of *Smad1*, *Smad3* and *Smad4* were remarkably enhanced in In state, our previous study revealed that activation of SMAD3 promoted SSCs transformation in the late stage, rather than driving the initiation of SSCs transformation, and revealed *Nanog* as a direct target of SMAD3 in SSCs [14]. Therefore, we hypothesized that enhanced expression levels of *Smad3* and *Smad4* were a prerequisite for the transition from In state to GSPCs. Notably, *Myc* is also a target of the FGF signaling pathway [47], and both SSCs medium (Medium 1) and GSPCs medium (Medium 2) contain 10 ng/ml bFGF. However, *Myc* was not activated in primary SSCs, indicating that it was activated by EGF in Medium 2, rather than bFGF.

Additionally, we analyzed the expression levels of genes associated with TGF-β, BMP, and p38 signaling pathways in SSCs and In state cells. The expression levels of *Hras*, *Mapk12* (*p38γ*), *Mapk13* (*p38δ*), *Id2*, and *Id3* were found to be suppressed in In state cells (Fig. 4i). Notably, *Id2* and *Id3* are target genes inhibited by TGF-β or sustained by BMP signaling pathways [48]. Therefore, the decreased expression levels of *Id2* and *Id3* suggested an enhanced function of the TGF-β signaling pathway in In state cells, which made them sensitive to the stimulation of BMP signal, leading to robust growth inhibition and trans-differentiation [48]. Moreover, decreased expression of *Mapk12* and *Mapk13* implied the attenuated activity of p38 signaling, which might enhance cell survival [49, 50]. p38 functions as a tumor repressor, since p38 activation leads to apoptosis, senescence, and differentiation [51], and p38 inhibits the expression of Cyclin D1 activated by RAS to suppress S phase transition [52]. Thus, we proposed that the down-regulation of *Mapk12* and *Mapk13* might enhance KRAS activity in SSCs transformation. We observed that the expression of Cyclin D1 was remarkably enhanced in In state, but transcriptional levels of *Axin2*, *GSK-3β*, and *β-catenin* were not remarkably changed in In state cells (Fig. 4j), indicating that the increased Cyclin D1 was probably due to the down-regulation of p38. These observations supported the conclusions that enhanced proliferation and survival promote cell transformation [17]. Moreover, the expression changes of some representative genes (*Plzf*, *Etv5*, *Dnmt1* and *Ccnd1*) in Fig. 4j were verified using RT-PCR [Fig. 4k], confirming the consistency of transcriptomic data. Thus, we inferred that p38 played a similar role as p53, a suppressor of SSCs transformation, and the decreased expression level of p38 synergically contributed to SSCs transformation with pluripotent genes and signaling pathways activated by LIF/EGF.

Finally, the expression of *Pcgf6* was activated in GSPCs. PCGF6 is enriched in the promoters of pluripotency-associated genes, including *Oct4*, *Sox2*, *Nanog*, *Lin28* and *Myc* [53], and co-localizes with G9A (EHMT2), HDAC1 and HDAC2 on the promoters of germ cell-associated genes to inhibit their expression [54]. A recent study reported that PCGF6 and MYC interact and co-occupy a distal regulatory element of *Sox2* to activate *Sox2* expression [55]. We also observed the increased expression levels of *Sox2*, *Hdac1* and *Hdac2* in GSPCs (Fig. 4i and j), thus we proposed that activation of *Pcgf6* was another key event of SSCs transformation, which promoted the expression of pluripotent genes and inhibited germline fate. Additionally, we noticed that *Dusp9*, a negative regulator of MAPK functioning through dephosphorylating ERK [56], was activated in GSPCs. Knock-down of *Dusp9* and *Klhl13* in female mouse ESCs led to a shift towards the male pluripotency phenotype, whereas overexpression of *Dusp9* enhanced the expression levels of pluripotent genes through inhibition of MAPK [57]. This evidence further confirmed that the germline fate was suppressed accompanied by the acquirement of pluripotency.

Collectively, our results indicate that EGF and LIF modulate the expression of multiple signaling pathways during SSCs transformation, driving SSCs to acquire pluripotent characteristics while losing their germline commitment.

### Verification of the transformation mediated by RAS and p38 signaling pathways

Based on above analysis, we proposed the mechanism of transformation initiation contained three key events: EGF activates the expression of *Smad3*, *Klf4* and *Myc* through RAS-MAPK signaling; inhibition of *p38* is conducive to survival of transforming cells, and activated SMAD3 promotes the further transformation; and LIF activates pluripotent genes, such as *Stat3* and *Tbx3*, to accomplish transformation.

**Fig. 5:**
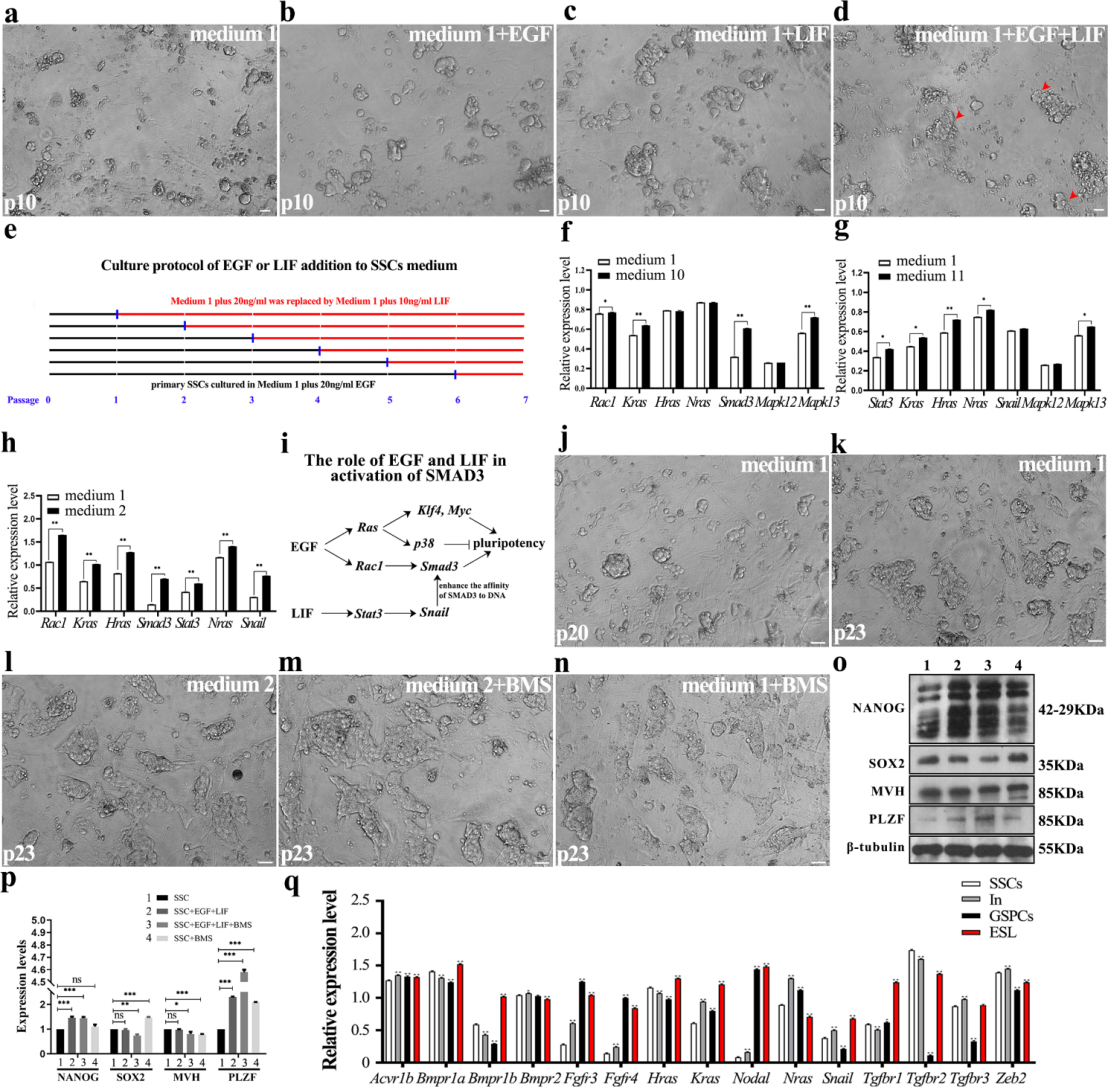
Verification of the transformation mediated by RAS and p38 signaling pathways. **a-d** The morphology of SSCs after 5 passages were cultured in medium 1 (**a**), medium 10 (**b**), medium 11 (**c**) and medium 2 (**d**) for another 5 passages was exhibited. **e** The strategy to screen the key time point of SSCs transformation through culturing SSCs in medium 10, and replacing the medium with medium 11 at different passages. **f-g** The relative expression levels of *Rac1*, *Hras*, *Kras, Snail and Gapdh* in SSCs after addition of EGF into medium 1 (**f**), and expression of *Stat3*, *Kras*, *Hras*, *Nras*, *Snail* and *Gapdh* in SSCs after addition of LIF into medium 1 (**g**) were detected using RT-PCR. **h** The relative expression levels of *Rac1*, *Hras*, *Kras, Smad3, Stat3, Nras, Snail* and *Gapdh* in SSCs after EGF supplement into medium 2 were detected using RT-PCR. **i** A regulatory pattern of EGF and LIF on *Klf4*, *Myc*, *p38*, *Snail* and *Smad3* was summarized. **j-n** The morphology of SSCs after 20 passages (**j**) were cultured in medium 1 (**k**), medium 2 (**l**), medium 2 plus BMS-582949 (**m**) and medium 1 plus BMS-582949 (**n**) for another 3 passages, respectively. **o-p**. Western blot determined the expression levels of NANOG, SOX2, MVH, PLZF and β-tubulin in SSCs of 20 passages after treatment of BMS-582949 (1. SSCs in medium 1, 2. SSCs in medium 2, 3. SSCs in medium 2 + BMS-582949, 4. SSCs in medium 1 + BMS-582949) (**o**), and the results were statistically analyzed (**p**). **q** The expression levels of *Acvr1b*, *Bmpr1a*, *Bmpr1b*, *Bmpr2*, *Fgfr3*, *Fgfr4*, *Hras*, *Kras*, *Nodal*, *Nras*, *Rac1*, *Snail*, *Tgfr1*, *Tgfr2*, *Tgfr3* and *Zeb2* in SSCs, In state cells, GSPCs and ES-like cells were detected using RT-PCR. Scale bar = 20 μm, data represent as mean ± SD *p < 0.05; **p < 0.01

To verify this hypothesis, EGF or LIF was separately added into SSCs cultured in medium 1, to test whether EGF or LIF alone could initiate transformation. Although cell growth ratio and size of colony were increased by EGF (medium 10) or LIF alone (medium 11), GSPCs colony was observed neither in EGF nor in LIF added groups within 10 passages (Fig. 5a-d; Table 3), indicating that transformation requires multiple signaling pathways that are simultaneously activated by EGF and LIF. Given the complicated process of transformation which might be composed of several steps, there is still a possibility that transformation has been activated by EGF treatment, but fails to move forward due to lack of LIF signaling. Thus, we cultured primary SSCs in medium 10 (medium 1 plus 20 ng/ml EGF), and replaced the medium with medium 11 (medium 1 plus 10ng/ml LIF) in different passages, to screen the key time point of SSCs transformation (Fig. 5e). However, none of them transformed into GSPCs state within 10 passages (data not shown). We also focused on the role of serum on SSCs transformation, and observed that removal of FBS retarded SSCs growth and affected transformation (Table 3). Thus, we concluded that 1% FBS plays a role in SSCs maintenance, rather than driving transformation. These observations further suggest that the combination of EGF and LIF is required for SSCs transformation.

**Table 3:** Cell transformation state under different culture conditions

Conditions					transformation state
LIF	EGF	MEF	FBS	siRNA	
+	+	+	+	/	Y
+	/	+	+	/	N
/	+	+	+	/	N
+	+	+	/	/	N
+	+	/	+	/	N
+	+	+	+	*Kras*	N
+	+	+	+	*Nras*	N
+	+	+	+	*Hras*	N
+ + +	+ + +	+ + +	+ + +	*Rac1* *Snail* *Smad3*	N N N

+: included; /: not included; Y: transformed colonies were observed within 10 passages; N: transformed colonies were not observed within 10 passages. n ≥ 3

We recently revealed that *p53* loss in primary SSCs increased the chromosomal accessibility of SMAD3’s target genes but could not activate *Smad3* expression, and consequently inferred that a prerequisite is needed to activate *Smad3* in SSCs transformation [14]. Here, we focused on the regulatory mechanism of EGF and LIF signals on *Smad3* activation in SSCs transformation. Published data demonstrated that EGF activated *Ras* [46] and *Rac1* [58], and RAC1 regulates the expression of *Smad3* [59]. Thus, we tested the expression changes of *Rac1* and *Ras* in SSCs after EGF supplement into medium 1, and revealed that expression levels of *Rac1*, *Kras* and *Smad3* were remarkably up-regulated, while expression levels of *Hras* and *Nras* were not changed (Fig. 5f). Thus, we confirmed that EGF activates *Smad3* expression through *Rac1*, which is a key event of SSCs transformation. Moreover, SNAIL (Snai1) and ZEB2 are SMAD-binding partners which enhance the affinity of SMAD complex to DNA [60, 61]. LIF activates *Stat3* expression, and STAT3 integrates cooperative RAS and TGF-β signals to induce *Snail* expression [62]. However, after addition of LIF to medium 1 (medium 11), we noticed that the expression of *Stat3* was strengthened, but expression levels of *Kras*, *Hras*, *Nras* and *Snail* were not changed (Fig. 5g), indicating that LIF-STAT3 signaling is insufficient to activate *Snail* in primary SSCs. On the contrary, when EGF was added with LIF (medium 2), an increased expression level of *Snail* was observed as expected (Fig. 5h), and *Zeb2* was activated in In state cells (Fig. 5q), indicating that activated transcription of *Rac1*, *Kras* and *Smad3* by EGF facilitated the enhanced expression of *Stat3* by LIF to activate *Snail*, which might cooperate with up-regulated ZEB2 to promote the binding of SMAD3 to DNA. Furthermore, we blocked the expression of *Kras*, *Hras*, *Nras*, *Rac1*, *Smad3* or *Snail* in transforming SSCs using *RNAi*, and noticed that formation of GSPCs colony was remarkably disturbed (Table 3), confirming the pivotal functions of RAS, RAC1, SNAIL and SMAD3 in promoting SSCs transformation. Combined with above observations, we concluded one of regulatory patterns as: EGF activated *Smad3* through *Rac1*, and combined with LIF to activate *Snail* through STAT3, and SNAIL and ZEB2 enhanced the binding effect of SMAD3 to regulate SSCs fate (Fig. 5i).

Moreover, p38 antagonist BMS-582949 was used to test the impact of p38 on GSPCs formation. SSCs cultured in medium 1 for 20 passages (which already expressed NANOG and usually spontaneously transformed within 5–10 more passages) were further cultured in medium 1 or medium 1 plus 2µM BMS-582949. Compared to those in medium 1, GSPCs colonies were remarkably increased within 3 passages after BMS-582949 addition. Furthermore, we demonstrated that BMS-582949 also accelerated GSPCs formation in medium 2: GSPCs colonies appeared as early as 2 passages, and GSPCs colony grew faster after BMS-582949 addition (Fig. 5j-n). The expression levels of NANOG were remarkably enhanced by BMS-582949 (Fig. 5o and p). Thus, we concluded that the blockage of p38 promoted SSCs transformation into pluripotent state.

### Analysis of the differential expression profiles of GSPCs and ES-like cells

Additionally, we compared the differential genes and signaling pathways between GSPCs and ES-like cells to explore the transformation mechanism of GSPCs into ES-like state. KEGG results showed a remarkable change of metabolic pathways in GSPCs, reflecting a close connection between metabolic pattern and pluripotency. In ES-like cells, signaling pathways including cell adhesion, Hedgehog, Wnt and TGF-β were activated [Fig. 4g], which were largely due to the impact of ESC medium (medium 3) containing a high proportion of serum and lacking of several factors such as BSA, bFGF and EGF. Interestingly, expression level of *Myc* remarkably decreased in GSPCs and ES-like cells [Fig. 4i], but targets of MYC were activated [Fig. 4h], indicating that MYC’s target genes activated in In state were constantly expressed in transformed cells. Although GSPCs and ES-like cells shared some pluripotent characteristics, e.g., both of them highly expressed *Nodal*, *Tbx3*, *Nanog*, *Esrrb*, *Lifr* and *Sox2*, we observed a remarkable expression difference of genes in pluripotency associated signaling pathways. In GSPCs, *Mapk12*, *Mapk13*, *Oct4* (*Pou5f1*), *Id1*, *Fgfr3* and *Fgfr4* were transcriptionally activated, while the expression of many genes in JAK-STAT, PI3K-AKT, Wnt and TGF-β signaling pathways were significantly down-regulated. In ES-like cells, *Nras*, *Kras*, *Klf4*, *Wnt2b*, *Wnt3*, *Wnt5b*, *Axin2*, *Bmpr1b*, *Inhbb*, *Smarcad1*, *Fzd3*, *Fzd6*, *Fzd7*, *Lefty1*, *Lefty2*, *Ctnnb1*, *Smad2*, *Pik3cb*, *Apc* and *Tcf7* (most of them belongs to Wnt, BMP, JAK-STAT signaling pathways) were activated, and expression of *Hras*, *Mapk12* and *Mapk13* was down-regulated again. Combined with role of LIF in transformation, enhanced expression of *Lifr* in GSPCs suggested that LIF signal was essential for both formation and maintenance of GSPCs. On the contrary, EGF, Wnt, JAK-STAT signaling pathways might be dispensable for GSPCs. In ES-Like cells, as previously described, genes in LIF, BMP, Wnt, TGF-β and Hedgehog signaling pathways were transcriptionally activated, implying that they were essential for ES-like cells conversion and/or maintenance.

Stemness of primed pluripotent stem cells is maintained by bFGF and Activin/Nodal signaling pathways, while that of naïve pluripotent stem cells is maintained by LIF and BMP signaling pathways [63]. We found that in GSPCs, the expression levels of *Acvr1b*, *Fgf3*, *Fgf4* and *Nodal* were enhanced, whereas the expression of *Bmpr1a*, *Bmpr1b* and *Bmpr2* was not activated, compared to in SSCs (Fig. 5q). This expression pattern is pretty close to the pattern of primed pluripotent stem cells, indicating the transformation of In state cells to GSPCs probably relied on Activin and FGF signaling. In ES-like cells, the expression levels of *Bmpr1a* and *Bmpr1b* were strengthened, while expression levels of *Fgf3* and *Fgf4* were decreased, compared to GSPCs (Fig. 5q). Additionally, *Lifr* and genes associated with Wnt signaling pathway were also highly expressed in ES-like cells (Fig. 4i), we therefore concluded that the expression profile of ES-like cells might be more likely to that of the naïve state, even though that *Nodal* was still highly expressed. Moreover, in In state cells, genes in BMP, FGF and LIF signaling pathways were partially activated (Fig. 5q), suggesting that these signaling pathways are also involved in conversion from SSCs to In state. Notably, the average expression levels of genes of TGF-β signaling pathway were decreased in GSPCs but increased in ES-like cells [Fig. 4g], similar to the expression patterns of *Smad3* and *Smad4*. This result is consistent with our previous study, which demonstrated that SMAD3 activation promoted long-term cultured SSCs to transform into pluripotent state, whereas it’s dispensable for primary SSCs [14]. Thus, we proposed that SMAD3 functions as a pivotal factor for SSCs transformation, neither a driver for transformation initiation, nor an essential factor for the maintenance of GSPCs or ES-Like cells.

### Identification of the effect of 2i on SSCs spontaneous transformation

Inhibition of p38-MAPK and activation of Wnt were observed in the initiation of SSCs transformation, which raised up a new question that whether blockage of MAPK and/or activation of Wnt could drive SSCs transformation. Thus, two inhibitors used for culture of naïve ESCs, MAPK inhibitor PD0325901 and GSK-3β inhibitor CHIR99021 (also known as “2i”), were supplied to SSCs medium to test the impact on transformation. However, primary SSCs on MEF feeder layers failed to survive in SSCs medium plus 2i (medium 4), GSPCs medium plus PD0325901 (medium 5), GSPCs medium plus CHIR99021 (medium 6) or GSPCs medium plus 2i (medium 7) for more than five passages, indicating that activation of Wnt and/or blockage of MAPK was detrimental to SSCs survival, and hampered SSCs transformation.

Given the fact that only stably cultured SSCs could transform in our transformation system (requires at least 5 passages’ culture on fresh MEF feeder), SSCs maintained in medium 1 for 5 passages were subsequently transferred to medium 1, medium 2, medium 4, or medium 7, respectively, to test the transformation effect. All the conditions contained MEF feeder layers. In medium 2, GSPCs colonies were observed after about 3 passages, while SSCs in either medium 4 or medium 7, failed to survive within 3–4 passages (data not shown), implying that addition of 2i is neither helpful for SSCs transformation, nor favorable for SSCs maintenance.

Based on these observations, we concluded that 2i could not drive primary or stably maintained SSCs to convert into pluripotent state. And it also implied that, besides p38-MAPK and Wnt signaling pathways, other signaling pathways activated by EGF/LIF were essential for the initiation of SSCs transformation.

### Detection of the role of 2i in the maintenance of GSPCs and ES-like cells

**Fig. 6:**
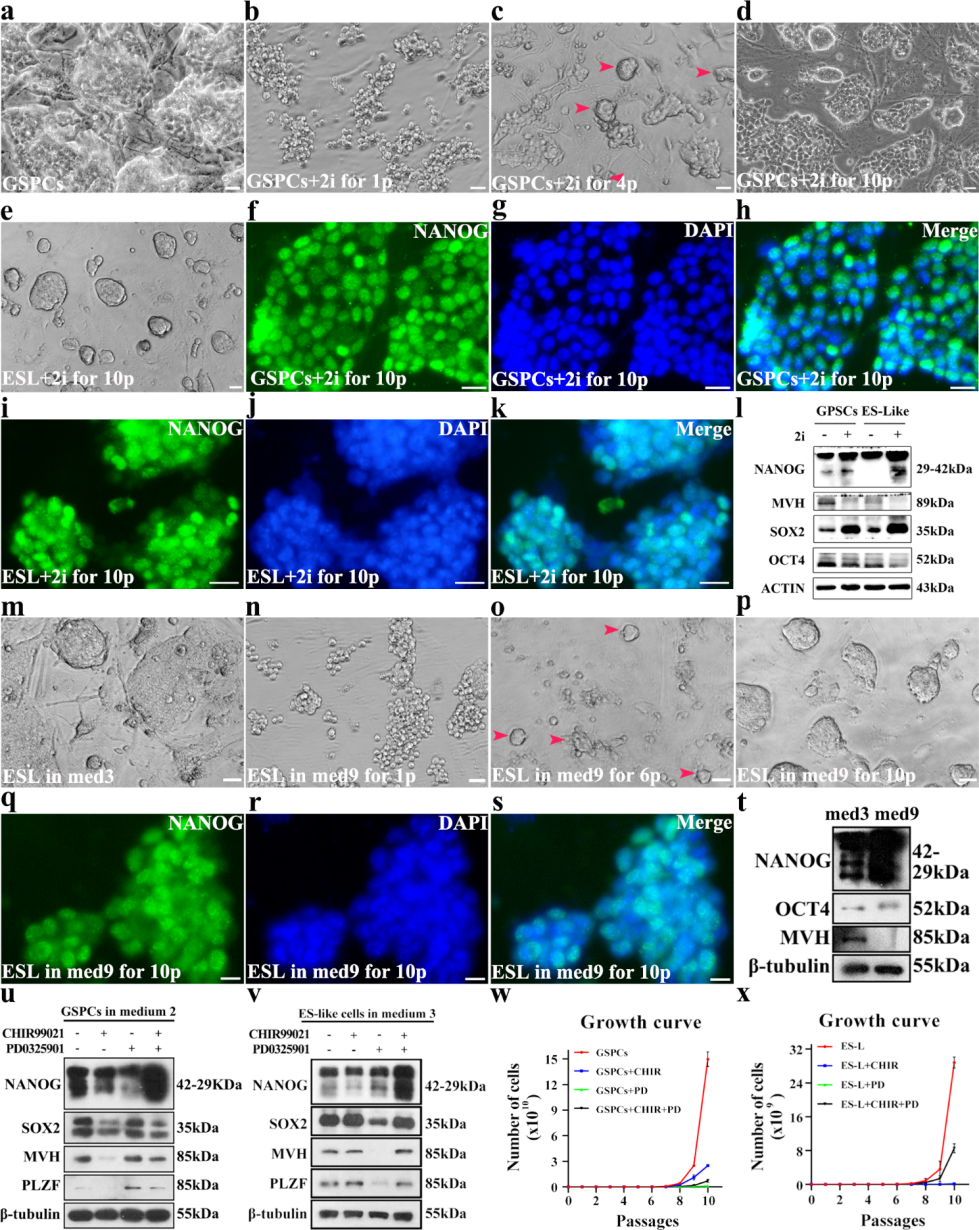
CHIR99021 + PD0325901 enhanced pluripotency in GSPCs and ES-like cells. **a-d** The morphology of GSPCs cultured in medium 2 (**a**), or in medium 7 for 1 passage (**b**), 4 passages (**c**) and 10 passages (arrowheads indicate some recovered colonies with compact structure) (**d**) was exhibited. **e** The morphology of ES-like cells cultured in medium 8 for 10 passages was exhibited. **f-k** IF staining assays were used to detect the expression of NANOG in GSPCs cultured in medium 7 (**f**, NANOG. **g**, DAPI. **h**, Merge) and ES-like cells cultured in medium 8 for 10 passages (**i**, NANOG. **j**, DAPI. **k**, Merge). **m-p** The morphologies of ES-like cells cultured in medium 3 (**m**), or medium 9 for 1 passage (**n**), 6 passages (arrowheads indicate some recovered colonies with compact structure) (**o**) and 10 passages (**p**), were exhibited. **q-s** Expression of NANOG in ES-like cells cultured in medium 9 was detected (**q**, NANOG. **r**, DAPI. **s**, Merge). **t** Expression levels of NANOG, OCT4, MVH and β-tubulin in ES-like cells cultured in medium 3 or medium 9 for 10 passages were determined using Western blot. **u-v** Expression levels of NANOG, SOX2, MVH, PLZF and β-tubulin in GSPCs (**u**) and ES-like cells (**v**) cultured with CHIR99021, PD0325901 or CHIR99021 + PD0325901 were determined using Western blot. **w-x** Growth curves of GSPCs (**w**) and ES-like cells (**x**) cultured with CHIR99021, PD0325901 or CHIR99021 + PD0325901 were exhibited. Scale bar = 20 μm, data represent as mean ± SD *p < 0.05; **p < 0.01

Primary SSCs failed to survive after the addition of 2i, indicating that MAPK signal is essential for primary SSCs growth, or hyperactive Wnt signal is detrimental to primary SSCs maintenance. Then, we asked whether addition of 2i could affect the growth of GSPCs and ES-Like cells. In medium 7, GSPCs colonies became loosened and tended to apoptosis within a few of passages (Fig. 6a and b), but GSPCs colonies recovered after 3–4 passages accompanying with the formation of some compact colonies (Fig. 6c), which could be stably maintained under this condition as well (Fig. 6d). However, ES-like cells colonies were not significantly affected after addition of 2i, just became more compact, and size of colonies was smaller than those in ESC medium or 2i naïve medium (Fig. 6e). IF staining revealed that these cells maintained in 2i-containing medium expressed pluripotent marker NANOG (Fig. 6f-k). Compared to cells in medium without 2i, the expression levels of pluripotent markers in 2i supplied groups were further enhanced (Fig. 6l), suggesting that 2i strengthened the pluripotency of GSPCs and ES-like cells.

Moreover, we tested the growth condition of ES-like cells cultured in standard 2i medium (medium 9) [15]. The compact colonies of ES-like cells (Fig. 6m) turned to a loosen structure within one passage in 2i medium, and cells seem to tend to return to grape-like colonies (Fig. 6n). Gradually, most of the cells in loosening structure failed to survive, and only a few of fraction grew and formed compact colonies (Fig. 6o). These newly appeared colonies were able to be maintained under this condition, and cell colonies gradually became compact and uniform again after 4–5 passages (Fig. 6p). The expression of NANOG confirmed their pluripotency identity (Fig. 6q-s). Furthermore, Western blot results revealed that ES-like cells cultured in standard 2i medium (medium 9) expressed stronger NANOG and lower germline marker MVH than their counterparts in ESCs medium (medium 3) (Fig. 6t), indicating that standard 2i medium further drove ES-like to pluripotent fate.

Next, CHIR99021, PD0325901 or CHIR99021 + PD0325901 were separately added to GSPCs or ES-like cells to test their independent role, but neither CHIR99021 nor PD0325901 enhanced the expression of pluripotent genes (Fig. 6u and v). It was worth noting that the growth rates of GSPCs and ES-like cells in medium supplied with CHIR99021, PD0325901 or CHIR99021 + PD0325901 were remarkably slower than control groups (Fig. 6w and x). One possibility was that cell growth was suppressed for 3–4 passages after the addition of inhibitors, and cells needed to adapt to the new environment. On the other hand, the inhibitors affected the signaling pathways even after cells grew stably, especially PD0325901, which severely affected cell proliferation [10]. However, the growth rate of cells supplied with CHIR99021 + PD0325901 was sharper after they adapted to the new medium. The doubling time of GSPCs or ES-like cells in the medium supplied with CHIR99021 + PD0325901 were slightly slower (Fig. 6x), but their expression level of NANOG was stronger than cells without CHIR99021 + PD0325901 (Fig. 6v). Interestingly, the expression levels of OCT4 and SOX2 were not obviously changed, probably because 2i directly activate *Nanog* expression [15], without affecting *Oct4* and *Sox2*. These observations implied that the pluripotency of GSPCs and ES-like cells were further enhanced by suppression of GSK-3β and MAPK.

Considering that 2i or LCDM (hLIF, CHIR99021, DiM, and MiH), both of which efficiently enhanced the pluripotency, contains CHIR99021, we finally tested in the role of CHIR99021 in GSPCs. Although addition of CHIR99021 induced similar morphological change with cells cultured in 2i medium, it took longer time for the colonies turning into the loosened state (around 3–4 passages), and the morphology change was mild compared to 2i treated group (Figure S6a and b). After 2–3 more passages, compact colonies appeared again (Figure S6c). On the contrary, the impact of CHIR99021 on ES-Like cells’ morphology was not obvious (Figure S6d). However, growth curves demonstrated that the growth rate of GSPCs and ES-like cells was suppressed by CHIR99021 or 2i (Figure S6e and f). The expression levels of NANOG and SOX2 were up-regulated, while expression levels of PLZF and MVH were down-regulated in CHIR99021 treated GSPCs or ES-like cells, compared to untreated control (Figure S6g and h). However, the expression levels of pluripotent markers in CHIR99021 treated GSPCs were not as strong as GSPCs or ES-like cells cultured in 2i medium (Figure S6g and h), suggesting that blockage of MAPK synergically functions with Wnt signaling pathway to strengthen the pluripotency of pluripotent stem cells derived from germline.

### The MEF feeder layer is essential for SSCs spontaneous transformation

Aforementioned observation revealed that as the feeder, MEFs less than three passages were more efficient for SSCs spontaneous transformation. Here, we tested whether SSCs cultured on feeder-free condition could spontaneously transform into pluripotent state in addition of EGF/LIF or not.

We first transferred primary SSCs to laminin coated dishes and cultured in medium 1 or medium 2, but found that the growth rates of these cells in either medium1 or medium 2 were remarkably inhibited, compared to those on MEF feeder. Moreover, apoptosis occurred within 2 passages, and majority of cells died within 4 passages (data not shown), indicating that primary SSCs on laminin could not transform with addition of EGF and LIF, probably due to their failure to adapt to the feeder-free condition.

**Fig. 7:**
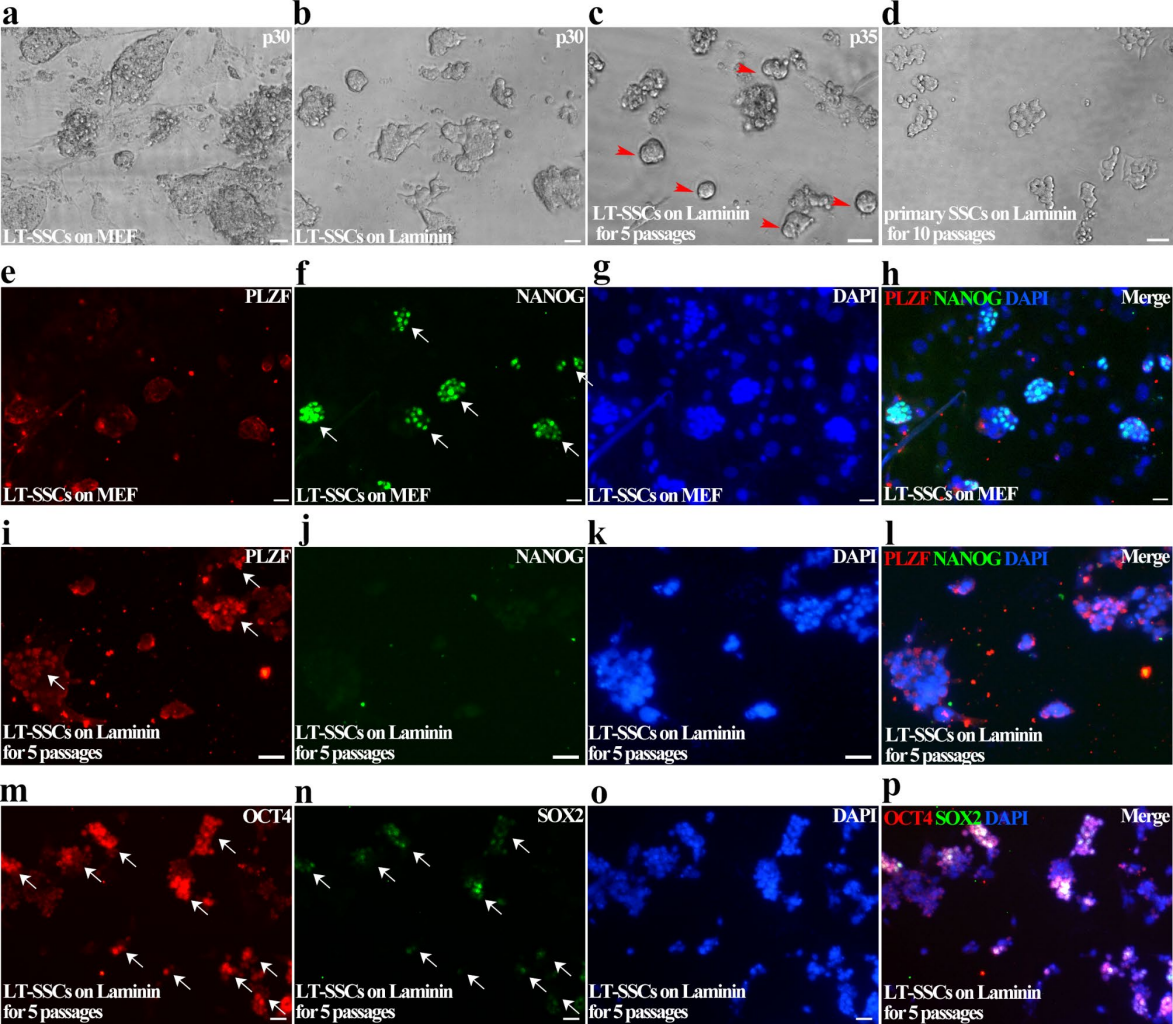
Long-term cultured SSCs failed to transform on laminin. **a-d** The morphologies of SSCs after 30 passages grown on MEF feeder (**a**) were transferred to laminin for 1 passage (**b**) and another 5 passages (arrowheads indicate some newly formed colonies) (**c**), and primary SSCs cultured on laminin for 10 passages (**d**) were exhibited. **e-h** IF staining was used to detect PLZF (**e**), NANOG (**f**), DAPI (**g**) and merge (**h**) in SSCs of 30 passages cultured on MEF feeder layer. **i-p** IF staining was used to detect PLZF (**i**), NANOG (**j**), DAPI (**i**), DAPI (**k**), merge from i to k (**l**), OCT4 (**m**), SOX2 (**n**), DAPI (**o**) and merge of m to o (**p**) in SSCs of 30 passages cultured on MEF feeder and subsequently cultured on laminin for 5 passages (arrows indicate representative cell clusters exhibited by immunofluorescence). Scale bar = 20 μm

Next, we tested whether EGF and LIF could induce long-term SSCs on laminin to transform. SSCs cultured in medium 1 for 30 passages (Fig. 7a), which already highly expressed NANOG and weakly expressed PLZF (Fig. 7e-h), were transferred to laminin coated 96-well plate (Fig. 7b). After 2–3 passages, majority of cells died, but a few of tight colonies survived and gradually became dominant colonies [Fig. 7c]. These newly formed colonies could be stably maintained on laminin, and gradually formed colonies morphologically similar to primary SSCs cultured on laminin [Fig. 7d]. IF staining showed that they highly expressed germline markers PLZF and OCT4, but weakly expressed NANOG and SOX2 (Fig. 7i-p), indicating that cells returned to germline fate after culturing on laminin. These long-term cultured SSCs expressed germline markers and pluripotent markers, suggesting that their composition was probably mixed with germline cells and pluripotent cells, therefore we proposed that germline cells in mixture were selected by laminin and finally survived. Based on these observations, we concluded that SSCs on laminin could not transform with the addition of EGF and LIF, since MEF feeder layer was indispensable for SSCs reprogramming.

In all, we reported a novel system to rapidly and stably induce spontaneous reprogramming of mouse germline stem cells into pluripotent state, and demonstrated a part of the molecular network, including EGF, LIF, RAS-MAPK, Wnt, and TGF-β signaling pathways. However, the spontaneous transformation mechanism of SSCs in EGF/LIF system is more complicated than our expectation, especially the existence of feeder layer hazes the clues of transformation mechanism. In further research, we will explore the chemical-defined spontaneous reprogramming system of germline stem cells and the underlying mechanism.

## Discussion

Until now, it’s still not clear why SSCs could occasionally transform into ES-like state after long-term culture. Undifferentiated germ cells are believed to retain some potential of pluripotency. For example, primordial germ cells (PGCs), the precursors of all germ cells formed in embryonic stage, could transform into pluripotent state during in vitro culture [64]. Shinohara’s group occasionally observed ES-like cells in testicular cells from neonatal mouse, but found that the transformation frequency of SSCs derived from neonatal testis cultured in vitro for 2 months, was too low to be observed (no ESL colony formed within 3 months), while knockout of *p53* in SSCs could induce ESL colonies within 2 months [7]. They further revealed the role of p53 is to regulate epigenetic modification in reprogramming [8], and we subsequently reported that *p53* deficiency increases the chromatin accessibility of SMAD3’s binding motif [14], which might explain why loss of *p53* accelerates SSCs transformation. In this study, we report a highly efficient system for spontaneously transforming SSCs cultured on MEF feeder layers into two pluripotent states, without using genetic editing or exogenous chemicals. The successful transformation requires that SSCs could be stably maintained on fresh MEF feeder and the existence of EGF and LIF. It’s consistent with the conclusion that stable and indefinite maintenance is a key step for cell transformation [17]. To achieve SSCs reprogramming in our system, SSCs must be cultured for at least 5 passages, and longer culture is conducive to improve transformation rate. Notably, the success rate of establishment of SSCs cell line is positively correlated to the transformation rate. Primary SSCs maintained for 5 passages in medium 1, usually could be further maintained to establish a long-term SSC line with a success rate of over 60%, and if SSCs were stably maintained in medium 1 for more than 10 passages, the rate to successfully establish long-term cell lines increased to over 90%. Consistently, SSCs cultured 5 passages in medium 1 on MEF feeder layer, could transform within 3–4 passages after replacement of medium 2 with a success rate of over 70%, and SSCs cultured in medium 1 for 8 passages could transform with a success rate more than 85%, and SSCs of more than 10 passages could almost 100% transformed into GSPCs in medium 2. This implies a connection of stable culture in vitro and SSCs transformation efficiency. Moreover, we noticed that mouse strain also affects that transformation efficiency. SSCs isolated from ICR and C57BL/6×DBA/2 strains could successfully transform in our system, whereas SSCs from C57BL/6 strain were not able to transform. Interestingly, SSCs from C57BL/6 strain are not suitable for establishment of SSCs cell line [65], confirming that stable culture in vitro is a prerequisite for transformation.

A recent study reported that 5 chemicals, SGC707 (a PRMT3 inhibitor), vitamin C (a DNA demethylation related chemical), epigallocatechin gallate (regulating DNA methyltransferase), daphnetin (inhibiting protein kinase), and tauroursodeoxycholic acid (anti-apoptosis) significantly induced SSC reprogramming [66]. These chemicals play key roles in regulating the methylation pattern, and enhance cell survival in transformation. However, it’s interesting that primary SSCs in medium 1 failed to survive after addition of 2i in our system. PD0325901 increased the laminin-binding ability of mouse SSCs cell line, rather than improving cell culture or cell activity [10], whereas hyper-activation of Wnt led to dysregulated proliferation and differentiation of SSCs, resulting in loss of germ cell pool [67–69]. Therefore, 2i might not be suitable for SSCs survival or transformation. On the contrary, in pluripotent stem cells, 2i plays different roles. We observed that supplementation of CHIR99021 inhibited the expression of pluripotent markers and germline markers in GSPCs, whereas PD0325901 inhibited the expression of SOX2, MVH and PLZF in ES-like cells. Hyperactive β-catenin seems to be detrimental to GSPCs, but the combination of CHIR99021 and PD0325901 enhanced the expression levels of NANOG in GSPCs and ES-like cells, indicating that some pluripotency associated signaling pathways could be activated only when 2i were co-existed.

Here we propose that p38 functions as a suppressor of cell survival. Under transformation stress, p38 inhibits RAS-induced transformation though down-regulation of Cyclin D1, and SSCs tend to cell cycle arrest and apoptosis. And when the expression of p38 was suppressed by EGF, it leads to the activation of RAS signaling pathway. However, evidence also suggests that p38-MAPK signaling pathway promotes the progression of cancer by enhancing cell survival [70]. This conflicting observation implies that p38 might be only an effector in regulation of cell cycle and survival, but not the factor to determine cell fate in SSCs transformation.

The role of MEF feeder cells in SSCs spontaneous reprogramming is interesting. Although fresh MEF is one of the key factors for SSCs transformation, SSCs on MEF feeder layers do not transform [13] or transform very slowly in our medium 1, while addition of EGF and LIF could drive SSCs cultured on MEF to reprogram. Therefore, we proposed that MEF provides some necessary but inadequate supports for SSCs spontaneous reprogramming. To fully reveal the mechanism of SSCs spontaneous reprogramming, it’s needed to establish a chemical defined transformation system without feeder layer. However, it is a challenging work, since MEF plays complicated roles, e.g., providing physical support for SSCs and secreting plenty of important factors. It’s typically interesting why SSCs only transform on MEFs less than three passages, it implies that fresh MEFs secrete some key factors for reprogramming. Moreover, it is worth noting that MEF feeder layer could secrete LIF, but we noticed that supplementation of EGF alone into medium 1 failed to induce transformation of SSCs cultured on MEF feeder. Thus, we infer that LIF concentration is probably pivotal for SSCs transformation.

The initiation phase of reprogramming is characterized by somatic genes being switched off by methylation, an increase in cell proliferation, a metabolic switch from oxidative phosphorylation to glycolysis, reactivation of telomerase activity and a mesenchymal-to-epithelial transition (MET) [71]. Since SSCs process the characteristics of epithelium cells and germline stem cells, SSCs might have the higher potential of reprogramming than somatic cells. Likewise, rewrite of methylation pattern, and increased activities of proliferation, glycolysis and telomerase were also observed in SSCs-derived pluripotent stem cells [7], suggesting that somatic cells and germline stem cells might share the similar signaling network of reprogramming, but germline stem cells are in a higher position in this hierarchy.

On the other hand, some differential mechanisms were also found. Inhibition of TGF-β signaling pathway enhances the initiation of somatic cell reprogramming, probably through preventing the MET for cell reprogramming, and TGF-β can also activate MAPK to induce the expression of mesodermal genes [71]. Thus, inhibitors of MAPK signaling pathway such as PD0325901 could be used in combination with TGF-β inhibitors to promote MET [72]. In SSCs, *p53* loss down-regulates *Smad3* expression, but after long-term culture when SSCs tend to transform into pluripotent state, the expression of *Smad3* is activated [14]. In this study, we demonstrated that *Smad3* activation was associated with EGF signal, and p38 was identified as the regulator during transformation, but PD0325901 failed to promote SSCs transformation. In GSPCs or ES-like cells, PD0325901 needs to synergically work with CHIR99021 to strengthen the expression of pluripotent genes. These observations imply that pluripotency-associated signaling pathways might play different roles depending on cell types, and be subjected to specific regulatory signals.

Polo et al. defined an intermediate cell population poised to become iPSCs and showed that two transcriptional waves were elicited by reprogramming factors: the first wave was driven by *c-Myc*/*Klf4* and the second by *Oct4*/*Sox2*/*Klf4* [73]. Although we also proposed an intermediate state of SSCs transformation, actually we prefer to believe that transformation is a continuous process. We subjectively defined the cells after 2 passages in medium 2 as “Intermediate state”, since GSPCs appeared after 4–5 passages in medium 2. At this stage, *Klf4* and *Myc* have been activated, but transcription of *Sox2* and *Nanog* was still inactivated, which is different from iPSCs derived from somatic cells. Interestingly, a more recent study reported that inhibition of class I Histone Deacetylase (HDAC) remarkably enhanced the efficiency of SSCs reprogramming induced by small chemicals [74], implying that acetylation of some genes associated with SSCs fate prevents reprogramming. Similarly, our previous study demonstrated that HDAC4 combines with PLZF to regulate the acetylation levels of *c-kit* and *Stra8* in SSCs [75]. Moreover, histone modification is also associated with SSCs fate, e.g., differentiation [76]. However, the connection of EGF/LIF signals and acetylation or histone modification is not known, and we will focus on these in future research.

## Conclusion

This system provides an alternative strategy to derive autogenous pluripotent cells from male patients or livestock in an efficient and safe way, and the regulatory network revealed in this study provide some insight for understanding the fate determination of germline. Nevertheless, many unknown factors in transformation are still needed to be investigated, especially the factors in serum or secreted by MEF feeder. In future research, we should explore the transformation of SSCs in feeder-free and serum-free system, which will provide important information for clinical research and animal science.

### Experimental procedures

#### Animals

In this study, ICR and C57BL/6xDBA/2 (C57BL/6 is not recommended) strains of mice were used for SSCs culture and pluripotency transformation assays. ICR, C57BL/6 and DBA/2 mice were supplied by Yangzhou University and Nanjing Medical University. Chimeric experiments were conducted using mouse embryos and conceptuses at embryonic day 2.5 (E2.5). Mouse embryonic fibroblast (MEF) feeders were prepared using E12.5-13.5 mouse embryos. All animal experiments were performed according to the Animal Protection Guidelines of Nanjing Agricultural University.

### SSCs isolation and culture

Testes from 5-day mice were harvested for SSCs isolation using the protocol of the previous study [14]. Briefly, tunica albuginea removed testes were sliced into small pieces and digested with collagenase IV and trypsin at 37 °C in a water incubator, respectively. After rinsing the cell pellets once with pre-chilled complete DMEM medium containing 10% FBS to deactivate and remove the enzyme via centrifugation, cell pellet was resuspended and filtered with a 70 μm filter. Rinse the cell sample via centrifugation again and resuspended the cell pellet to 1 × 10^5^ cell/ml and plated on mouse embryonic fibroblast (MEF) feeder layers. The protocol for preparing MEF was as previously described [14]. SSCs were cultured in medium 1 under 5% CO_2_ at 37 °C. The components of medium 1 are summarized in Table S1. SSCs are routinely passaged at a split ratio of 1:2 to 1:3 using 0.05% trypsin-EDTA every 5–7 days, while after 20 generations, they could be passaged in 3–4 days with a split ratio of 1:3 − 1:5.

For feeder-free culture, SSCs were isolated from the testes of 5-6-day mice as mentioned above. After differential plating, cells were first cultured on MEF feeder for 30 passages in medium 1, then transferred to 20ug/ml laminin-coated dishes cultured in medium 1. Passage once every 3–5 days using 0.05% trypsin-EDTA at a ratio of 1:2 to 1:4 depending on cell density.

### Conversion of SSCs into GSPCs

SSCs over five passages are feasible for conversion. Conversion was usually conducted on day 1 before the passage of SSCs (SSCs were usually passaged every 5–7 days in our laboratory), and SSC colonies usually reached 70–80% of confluence. IMEM medium was used to rinse SSCs to remove the medium 1, dead cell or debris. Then, the IMEM medium was replaced with the conversion medium (medium 2). And 24 h post addition of medium 2, cells were passaged at a split ratio of 1:1 using 0.05% trypsin-EDTA. The medium 2 was changed every 2 days. After 2–3 passages, epiblast-like cells gradually appeared. After 10 passages in medium 2, most of colonies maintained in the dishes are the epiblast-like colonies, which were passaged at a split ratio of 1:5 − 1:10 using 0.05% trypsin-EDTA every 2–3 days, and could be stably maintained on MEF feeder with medium 2 for more than 30 passages. The components of mediums are summarized in Table S1.

To verify the role of genes in SSCs transformation, siRNAs targeting *Kras*, *Nras*, *Hras*, *Rac1*, *Snail* or *Smad3* mRNA were transfected into SSCs, respectively, when EGF and LIF were suppled. The protocol of transfection was identical to previous study [75]. SSCs were maintained for 10 passages, to monitor the formation of GSPCs colony under these conditions (n ≥ 3). The sequences of siRNA are listed below:

*Kras* siRNA: CTATACATTAGTCCGAGAAAT.

*Nras* siRNA: CGATGGCACTCAAGGTTGTAT.

*Hras* siRNA: CGGGTGAAAGATTCAGATGAT.

*Rac1* siRNA: CGCAGACAGACGTGTTCTTAA.

*Snail* siRNA: UGCAGUUGAAGAUCUUCCGCGACUG.

*Smad3* siRNA: GAGAUUCGAAUGACGGUAATT.

### Conversion of GSPCs into ES-like cells

From GSPCs appearance to become dominant, it takes 5–7 passages. Conversion was usually conducted on day 1 before subculture of GSPCs, and colonies usually reached 50–60% of confluence. Medium 2 was removed and DMEM medium was used to rinse GSPCs to eliminate dead cells or debris. Then, the DMEM medium was replaced with the ES medium (medium 3), and cells could be passaged at a split ratio of 1:3 − 1:5 24 h post medium change. The medium need to be changed every day. After 1–2 passages, most GSPCs transformed into ES-like shape. And ES-Like cells were passaged at a split ratio of 1:3 − 1:5 using 0.05% trypsin-EDTA every 2–3 days. The components of mediums are summarized in Table S1.

### SSCs labelling and transplantation

SSCs cultured on MEF for more than 10 passages were infected with GFP expressing lentivirus. Lentivirus package was identical to previous study [14]. The uninfected SSCs were eliminated by puromycin, and the injection procedure followed the reported protocol [14] with minor modification: the GFP-labelled SSCs were digested into single cell suspension and filtered with 70 μm filter, and trypan blue was added to monitor the cell injection efficiency.

### Chimeric assay of multiple-cell microinjection

SSCs, GSPCs and ES-Like cells were infected with lentivirus containing GFP and zeocin resistance sequences, and screened with 50 µg /ml zeocin to obtain the GFP + cells for further culture. For chimeric experiments, SSCs, GSPCs, ES-Like cells and ESCs were harvested in the logarithmic growth phase (usually one day before passage). The cell suspensions were filtered through a 70 μm cell strainer, and were centrifuged at 300 g at room temperature for 5 min. The supernatant was removed, and the pellets were resuspended in the DMEM medium at a proper density (2–6 × 10^5^ cells/ml). The suspension was placed on ice for 20–30 min before injection. Eight to ten of the digested cells were microinjected into each E2.5 embryo of B6 diploid mice. The embryos were observed using an immunofluorescence stereomicroscope to detect GFP + cells localization on the next day after culture.

### Immunofluorescence (IF) staining, alkaline phosphatase (AP) staining and Western blot

The protocol of IF assay was identical to previous study [77]. Briefly, cells were fixed with Carnoy for 20 min at -20℃, and were rinsed with neutral PBS for three times before blocking with 10% goat serum for 30 min at room temperature. Cells were incubated with primary antibodies at 4℃ overnight, and were incubated with appropriate secondary antibodies for 1 h after rinse. Finally, 4’,6-diamidino-2-phenylindole (DAPI) was used for counterstaining.

The BCIP/NBT alkaline phosphatase staining kit (Beyotime, C3206) was used to detect alkaline phosphatase activity. Briefly, ESCs, ES-like cells from *p53*^+/+^ or *p53*^−/−^ SSCs, and primary SSCs were rinsed with PBS and incubated with BCIP/NBT solution for 30 min in dark. After removal of BCIP/NBT solution, cell samples were rinsed with Millipore H_**2**_O to terminate staining, and finally were analyzed under the microscope.

The protocol for Western blotting is identical to previously described [78] was briefly listed: protein lysates were separated with SDS-PAGE gels, and the gels were transferred to PVDF membrane for blotting. Membranes were blocked in 5% milk for 1 h prior to addition of primary antibody at 4℃ overnight, then were rinsed twice with TBST. Peroxidase-conjugated goat anti-rabbit IgG or goat anti-mouse IgG was used to detect the primary antibodies. Immuno-reactive bands were visualized using the ECL and exposed to film. The intensity of the bands was quantified using the ImageJ software.

The information of antibodies used for IF and Western blot was listed in Table S3.

#### Teratoma and immunochemistry assay

SSCs, GSPCs, ESL cells and ESCs were collected by trypsinization before injection. Approximately 10^6^ cells were injected subcutaneously into immunodeficient mice. Teratomas generally developed within 1–6 weeks, and the animals were sacrificed before the tumor size exceeded 1.5 cm in diameter. The teratomas were then embedded in paraffin and processed for hematoxylin and eosin staining [14].

### Karyotype analysis

To arrest cells in metaphase, cells were incubated with 0.02 µg/mL colchicine (SIGMA CAS:64-86-8) for 6 h, and were harvested by 0.05% trypsin digestion and centrifuged at 300 g for 5 min. Then, the pellet was resuspended in 0.4% KCl and incubated for 30 min at 37 °C, and fixed in 3 mL of freshly prepared Carnoy’s solution (methanol: acetic acid = 3:1, pre-chilled at -20 °C) on ice for 10 min. After centrifugation at 800 g for 10 min, 5ml of Carnoy’s solution was added again and incubated on ice for 50 min. Cells were harvested by centrifugation at 800 g for 10 min. The cell suspension was dropped onto pre-chilled at -20 °C slides and dried by a flame. The slides were stained with 1×Giemsa solution for 10–15 min, and dried after being rinsed carefully with water. The chromosome morphology was observed under a light microscope (with 1000× magnification).

#### Reverse transcription-polymerase chain reaction (RT-PCR)

For reverse Transcription, total RNA extracted from cells with TRNzol (Tiangen, DP424) was converted into cDNA using HiScript III All-in-one RT SuperMix (Vazyme, R333). Subsequently, PCR was performed using Premix Ex Taq (Takara, RR036). The information of primers was listed in Table S2.

#### Gene imprinting analysis

Genomic DNA was isolated from SSCs, GSPCs, ES-like cells and mouse testes using TIANamp Genomic DNA Kit (Tiangen, DP304). Purified genomic DNA (1 µg) was treated with EZ DNA Methylation™ Kit (ZYMO RESEARCH, D5001) as described previously [79]. Bisulfite genomic sequencing of DMRs of imprinted genes was carried out as described [79]. PCR amplifications of each DMR region from bisulfite-treated genomic DNAs was carried out using specific primers (Table S2), during which cytosine was converted to uracil. For combined bisulfite restriction analysis, PCR products were digested with restriction enzymes with a recognition sequence containing CpG in the original unconverted DNA. Intensity of digested DNA bands was quantified with ImageJ software.

#### RNA-seq and data analysis

Around 1 × 10^5^ SSCs (cultured in medium 1 for 5–6 passages), Intermediate state cells (SSCs cultured in medium 1 for 5 passages and further cultured in medium 2 for 2–3 passages), GSPCs (SSCs cultured in medium 1 for 5 passages and further cultured in medium 2 for 10 passages) and ES-like cells (GSPCs cultured in medium 3 for 10 passages) were collected using the identical protocols [14] for RNA-seq assay. RNA-seq library preparation and sequencing were performed according to previously described [80]. Total RNA was extracted using Trizol (Ambion Life Technologies) according to the Ambion standard RNA isolation procedure, and mRNA was purified using the NEBNext Poly (A) mRNA Magnetic Isolation Beads (NEB, USA). Then the mRNA library was constructed with a NEBNext Ultra Directional RNA Library Prep Kit for Illumina (E7420S/L, NEB) and sequenced with Illumina HiSeq 2000. DEGs analysis was performed to compare SSCs and GSPCs using the DESeq2 R package. DEGs were defined with the criteria of as q-value < 0.05 and absolute log (fold change) ≥ 1.5. Gene ontology (GO) and KEGG analysis were performed with DAVID. The heatmap was generated using the pheatmap. Gene Set Variation Analysis (GSVA) heatmap was generated by R package, GSVA. The GSVA package was performed in R 3.6.1 to calculate the enrichment score of the pathways in each sample. In all experiments, we used the gene sets database from the Molecular Signature Database (MSigDB) collection. Gene Set Enrichment Analysis is supported by the Broad Institute website (http://www.broadinstitute.org/gsea/index.jsp) and includes versions compatible with Java, R or Gene Pattern. All GSEA analyses presented here were performed using the R GSEA implementation.

The original data of RNA-seq assay has been uploaded to https://www.ebi.ac.uk/fg/annotare/login/#list:all, and E-MTAB-12,342 is the code to review the original data.

### Comparative analysis of the similarity of ESC, ESL, GSPCs and SSCs

The wild-type mouse ESCs dataset originated from the following studies: GSM5589123, GSM5589124 [19]. Raw data for ESCs were obtained and jointly analyzed with raw data for ES-like cells, GSPCs, and SSCs. Initially, data quality control was performed using FastQC (v0.11.9), followed by data cleaning with Trim-galore (0.6.10). Subsequently, HISAT2 (2.1.0) was employed to align the cleaned data to the mouse (mm39) reference genome, and featureCounts (2.0.3) quantified the reads, with expression levels represented in TPM (Transcripts Per Kilobase per Million mapped reads). Before similarity analysis, batch effects from datasets of different origins were removed using sva (3.46.0). Then, the prcomp function was applied to conduct principal component analysis on the four sample groups, and a scatter plot was generated using ggplot2 (3.4.1). Hierarchical clustering of the sample TPM matrix was performed, and a heatmap was visualized using ComplexHeatmap (2.14.0). The cor function was utilized to calculate the correlation between samples, and a correlation heatmap was constructed for visualization.

### Quantification and statistic analysis

Data was analyzed by Excel and was presented as mean ± SD (standard deviation), and statistical significance was determined by *t-test*.

### Electronic supplementary material

Below is the link to the electronic supplementary material.

Supplementary Material 1

Supplementary Material 2

Supplementary Material 3

Supplementary Material 4

Supplementary Material 5

Supplementary Material 6

Supplementary Material 7

Supplementary Material 8

Supplementary Material 9

Supplementary Material 10

Supplementary Material 11

Supplementary Material 12

Supplementary Material 13

## Data Availability

The data that supports the findings of this study are available in the method part and supplemental materials.

## Abbreviations

bFGF: Basic fibroblast growth factor
COBRA: Combined bisulfite restriction analysis
DEGs: Differential expressed genes
DMRs: Differentially methylated regions
EGF: Epidermal growth factor
EMT: Epithelial-mesenchymal transition
ESC: Embryonic stem cells
FBS: Fetal bovine serum
GDNF: Glial cell line-derived neurotrophic factor
GEO: Gene Expression Omnibus
GSPCs: Germline stem cells derived pluripotent cells
GSVA: Gene Set Variation Analysis
IMDM: Iscove’s modified Dulbecco’s medium
iPSCs: Induced pluripotent stem cells
LIF: Leukemia inhibitory factor
MEF: Mouse embryonic fibroblast
PCA: Principal component analysis
SSCs: Spermatogonial stem cells
TGF-β: Transforming Growth Factor Beta
TPM: Transcripts Per Kilobase per Million mapped reads
