# Supplementary figures and images for: A rapid and stable spontaneous reprogramming system of Spermatogonial stem cells to Pluripotent State
Source: Cell Biosci. 2023 Dec 1;13:222. doi: 10.1186/s13578-023-01150-z (PMC10693117; doi:10.1186/s13578-023-01150-z)

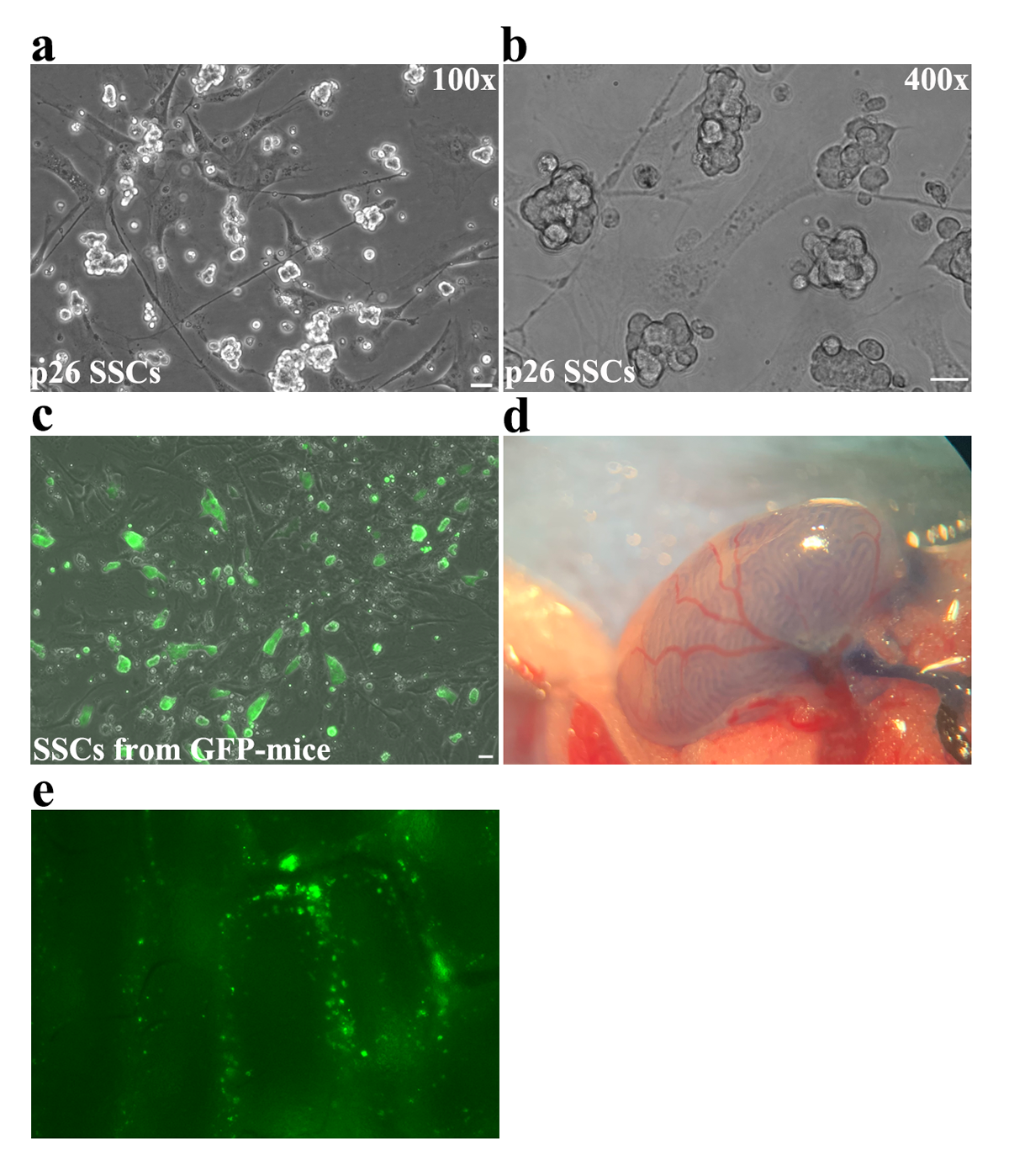

Supplement: Supplementary file 1 — Supplementary Material 1 [file 13578_2023_1150_MOESM1_ESM.tif]

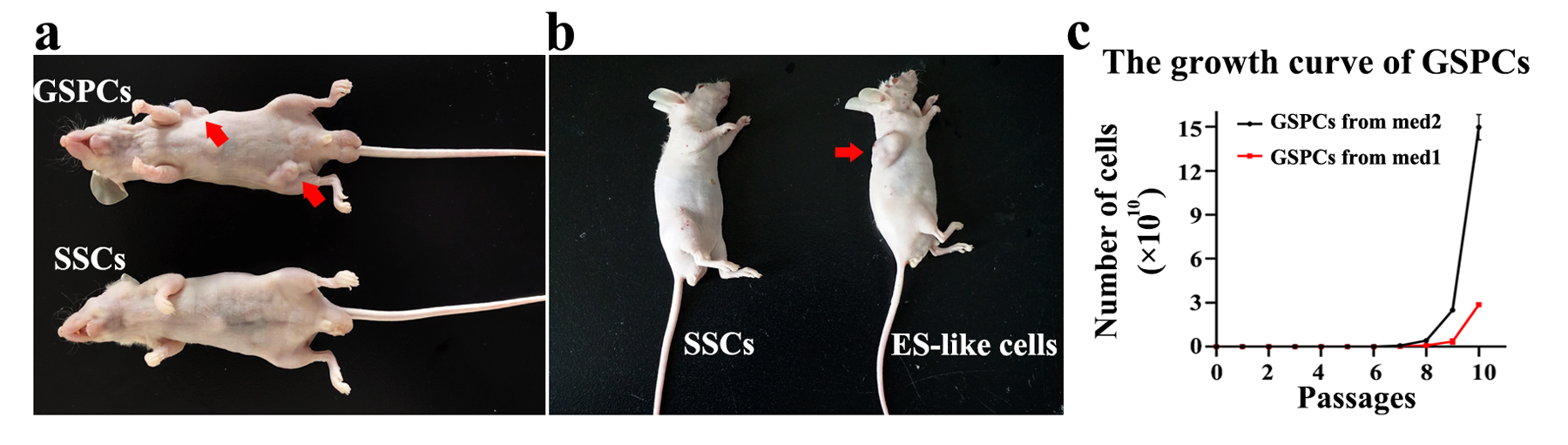

Supplement: Supplementary file 2 — Supplementary Material 2 [file 13578_2023_1150_MOESM2_ESM.tif]

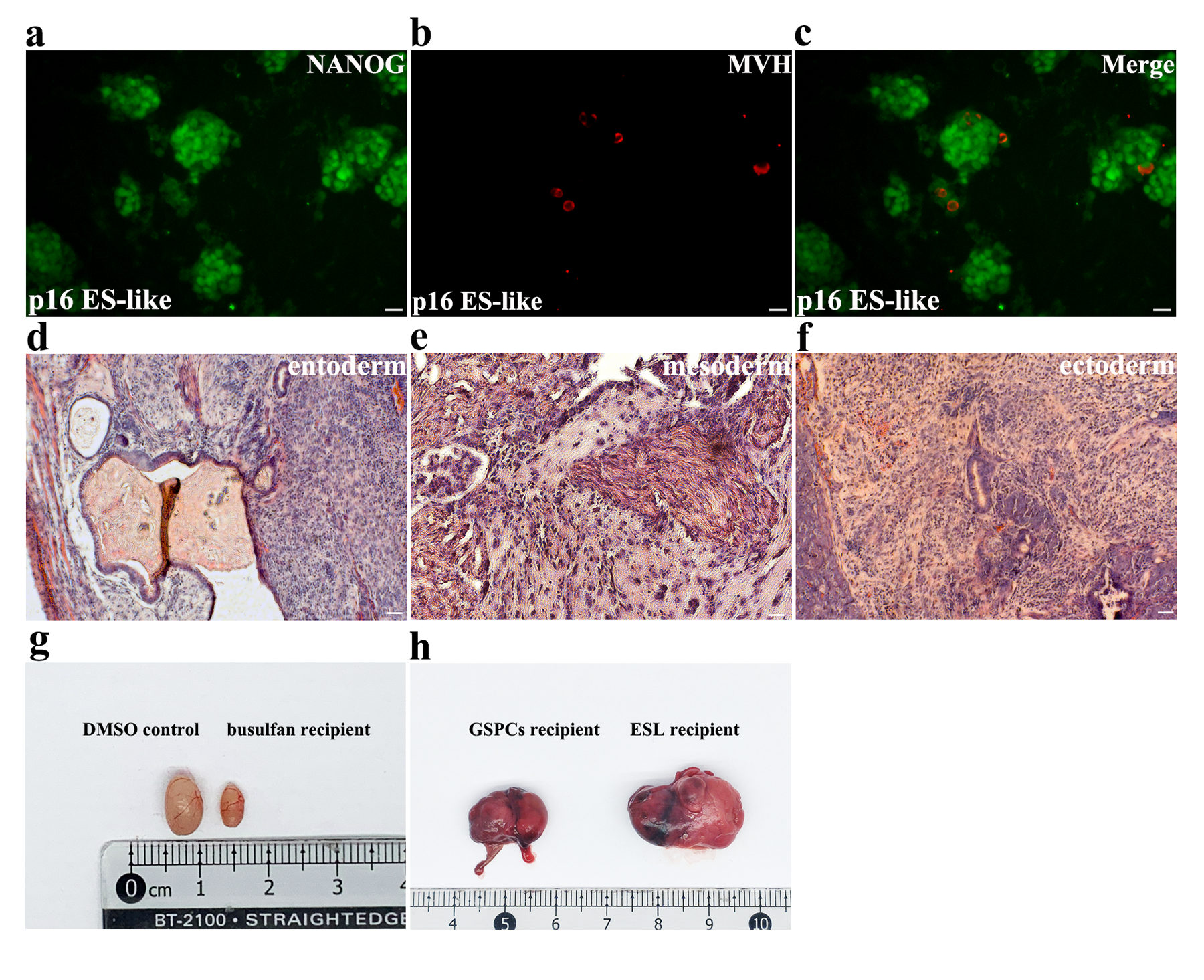

Supplement: Supplementary file 3 — Supplementary Material 3 [file 13578_2023_1150_MOESM3_ESM.tif]

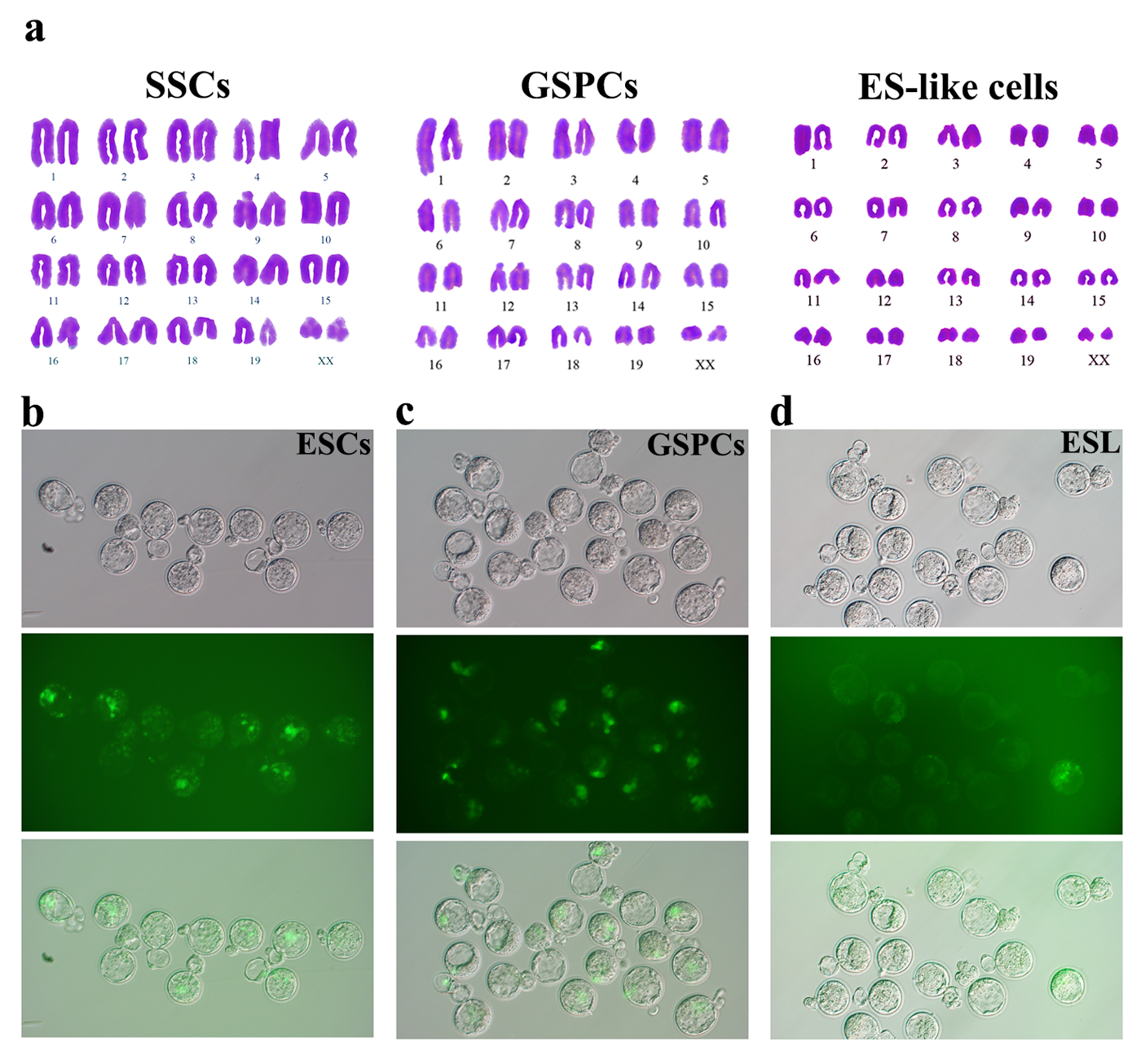

Supplement: Supplementary file 4 — Supplementary Material 4 [file 13578_2023_1150_MOESM4_ESM.tif]

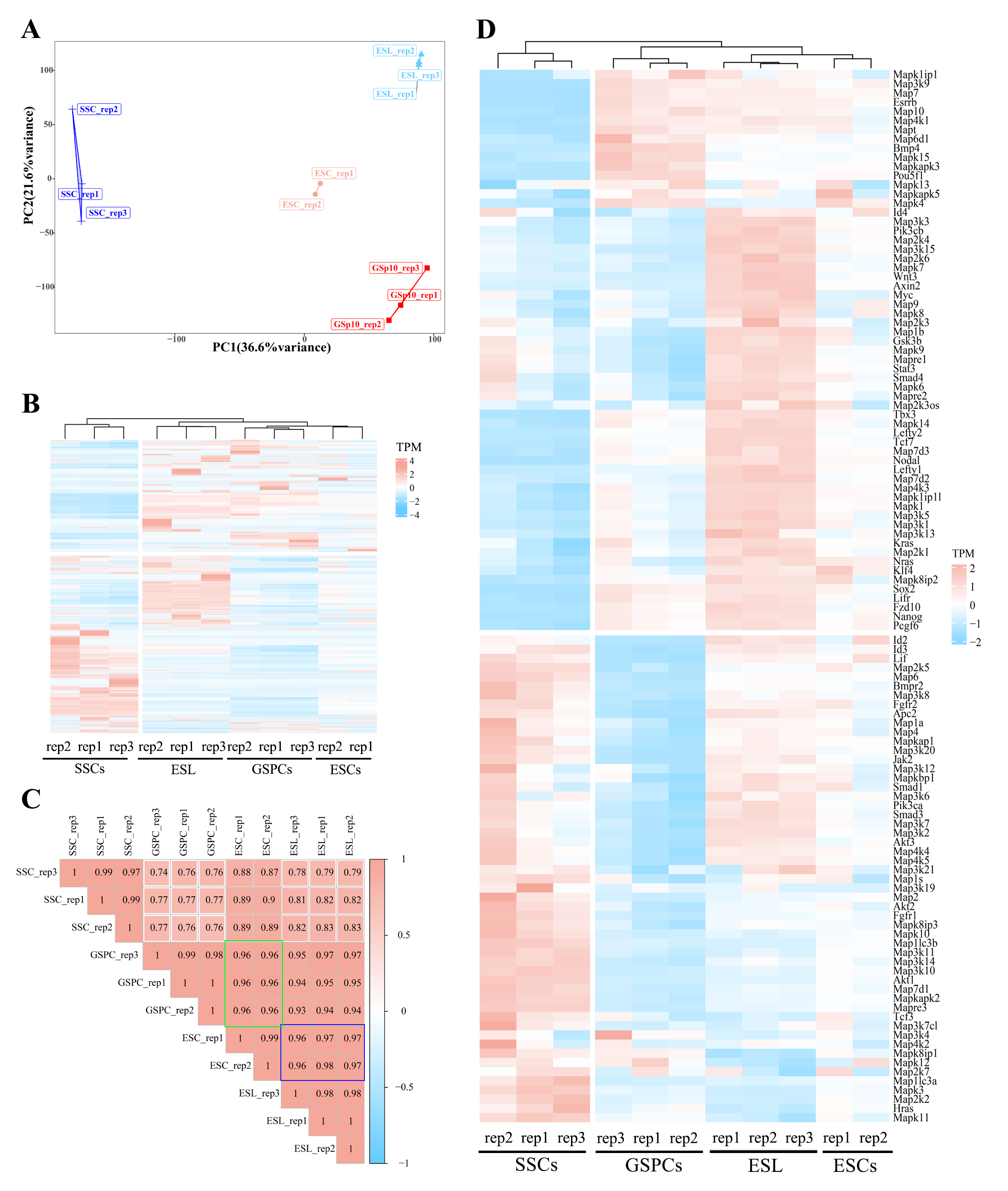

Supplement: Supplementary file 5 — Supplementary Material 5 [file 13578_2023_1150_MOESM5_ESM.tif]

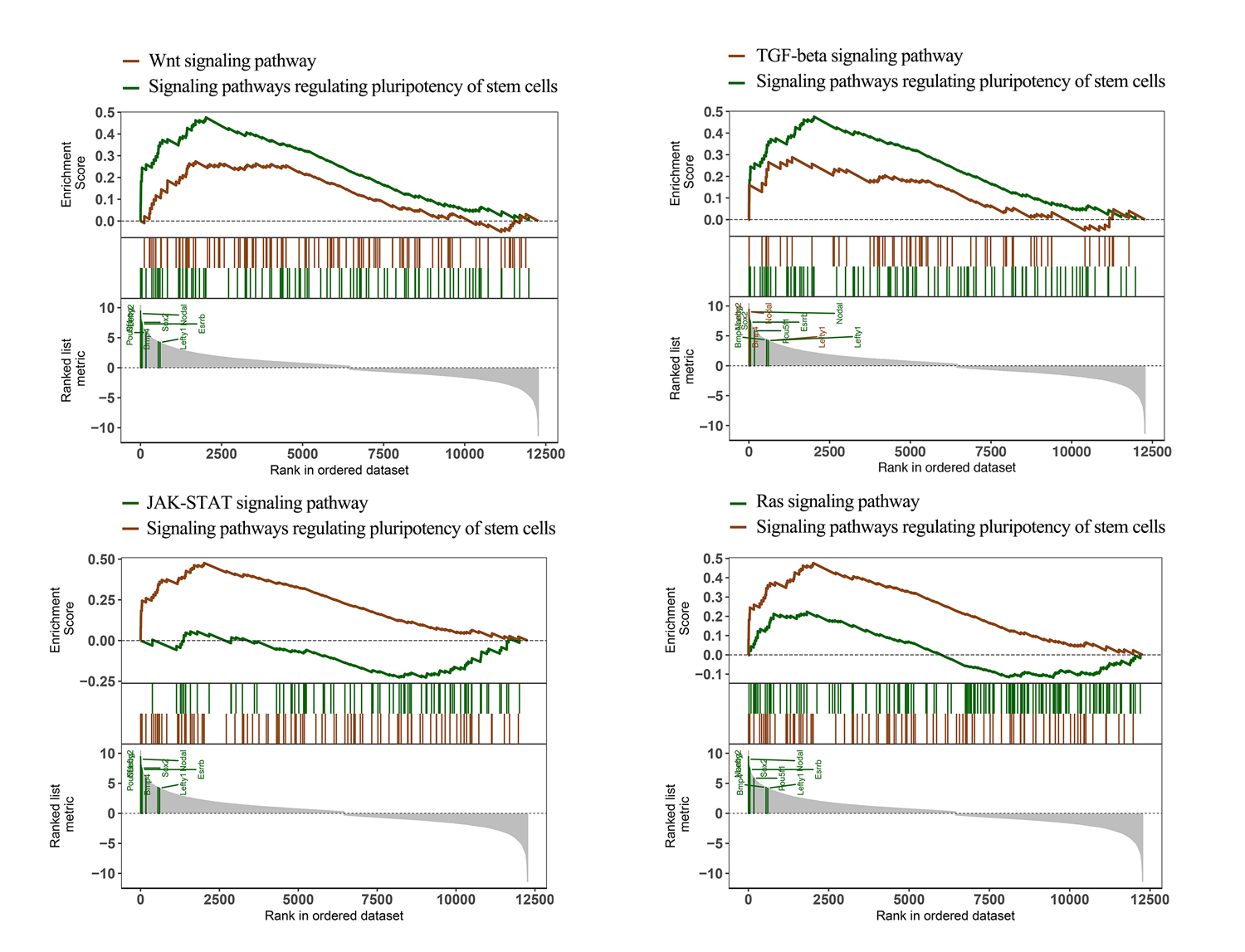

Supplement: Supplementary file 6 — Supplementary Material 6 [file 13578_2023_1150_MOESM6_ESM.tif]

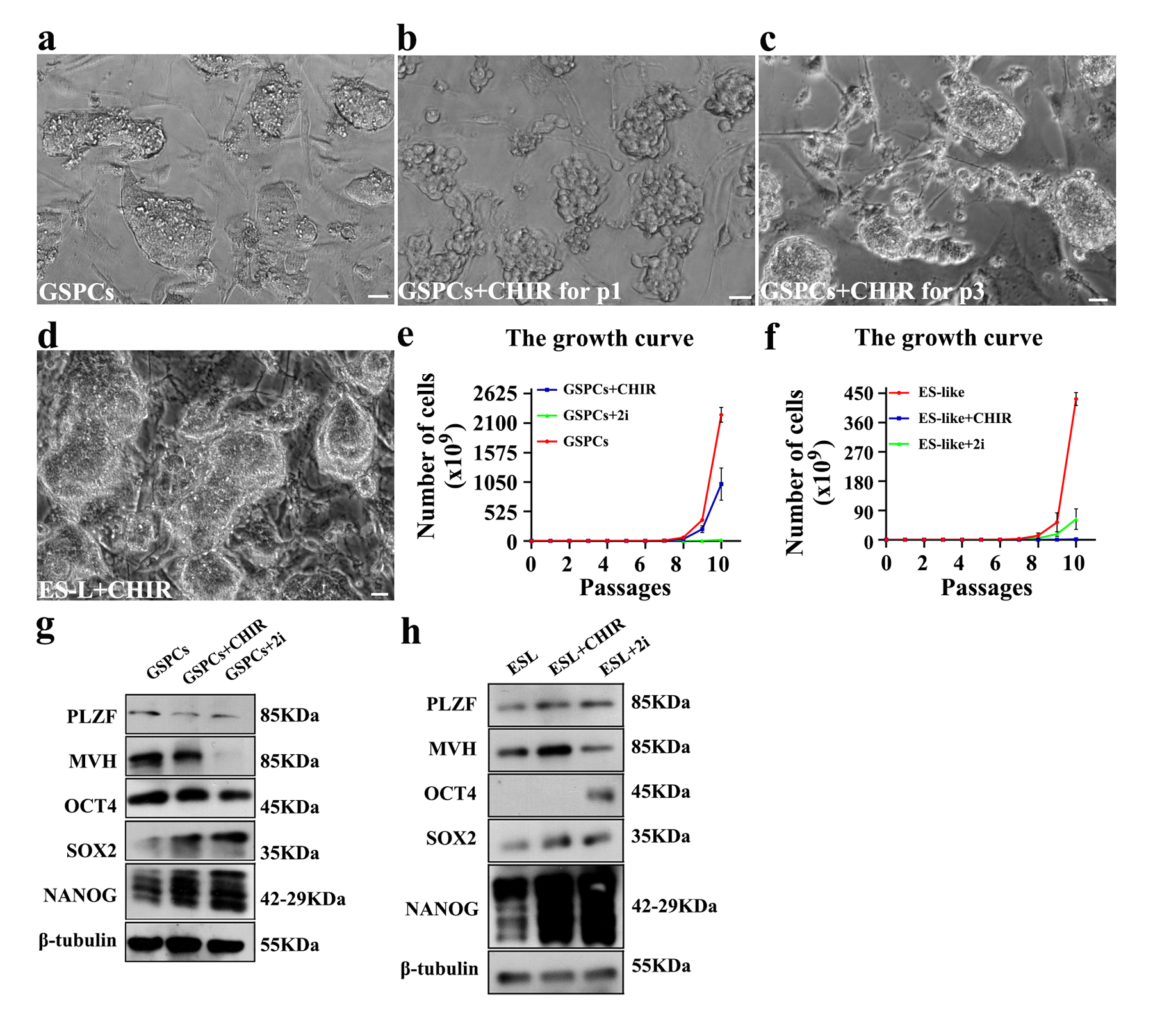

Supplement: Supplementary file 7 — Supplementary Material 7 [file 13578_2023_1150_MOESM7_ESM.tif]
