# Supplementary material for: A rapid and stable spontaneous reprogramming system of Spermatogonial stem cells to Pluripotent State
Source: Cell Biosci. 2023 Dec 1;13:222. doi: 10.1186/s13578-023-01150-z (PMC10693117; doi:10.1186/s13578-023-01150-z)
Supplement: Supplementary file 8 — Supplementary Material 8 [file 13578_2023_1150_MOESM8_ESM.docx]

**Table S1. The components of mediums used in this study.**

| **REAGENT** | **SOURCE** | **IDENTIFIER** | **Medium 1:**  **Modified SSCs medium** | **Medium 2:**  **GSPCs medium** | **Medium :3**  **ESC medium** | **Medium 4:**  **Modified SSCs medium+ CHIR99021+ PD0325901** | **Medium 5:**  **GSPCs medium+**  **PD0325901** | **Medium 6:**  **GSPCs medium+**  **CHIR99021** | **Medium 7:**  **GSPCs medium+ CHIR99021+ PD0325901** | **Medium 8:**  **ESC medium+ CHIR99021+**  **PD0325901** | **Medium 9:**  **2i ESC medium** | **Medium 10:**  **Modified SSCs medium**  **+EGF** | **Medium 11:**  **Modified SSCs medium**  **+LIF** |
| --- | --- | --- | --- | --- | --- | --- | --- | --- | --- | --- | --- | --- | --- |
| IMDM | Gibco | 12200-036 | ✓ | ✓ | - | ✓ | ✓ | ✓ | ✓ | - | - | ✓ | ✓ |
| DMEM | Gibco | 12100-046 | - | - | ✓ | - | - | - | - | ✓ | - | - | - |
| DMEM/F12 | Gibco | 12400-024 | - | - |  | - |  |  |  |  | ✓ | - | - |
| Neurobasal | Gibco | 21103-049 | - | - | - | - |  |  |  | - | ✓ | - | - |
| Pyruvic acid | Sigma | P2256 | 30 μg/ml | 30 μg/ml | 30 μg/ml | 30 μg/ml | 30 μg/ml | 30 μg/ml | 30 μg/ml | 30 μg/ml | - | 30 μg/ml | 30 μg/ml |
| D-(+)-glucose | Sigma | G7021 | 6 mg/ml | 6 mg/ml | - | 6 mg/ml | 6 mg/ml | 6 mg/ml | 6 mg/ml | - | - | 6 mg/ml | 6 mg/ml |
| DL-Lactic Acid | Sigma | L4263 | 1 μl/ml | 1 μl/ml | - | 1 μl/ml | 1 μl/ml | 1 μl/ml | 1 μl/ml | - | - | 1 μl/ml | 1 μl/ml |
| Bovine albumin (BSA) | MP Biomedicals | b810661 | 5 mg/ml | 5 mg/ml | - | 5 mg/ml | 5 mg/ml | 5 mg/ml | 5 mg/ml | - | - | 5 mg/ml | 5 mg/ml |
| L-Glutamine | Sigma | G7513 | 2 mM | 2 mM | 2 mM | 2 mM | 2 mM | 2 mM | 2 mM | 2 mM | 2 mM | 2 mM | 2 mM |
| 2-Mercaptoethanol | Sigma | M3158 | 5×10^-5^ M | 5×10^-5^ M | 5×10^-5^ M | 5×10^-5^ M | 5×10^-5^ M | 5×10^-5^ M | 5×10^-5^ M | 5×10^-5^ M | 0.1mM | 5×10^-5^ M | 5×10^-5^ M |
| MEM Vitamin Solution | Invitrogen | 11120-052 | 1× | 1× | - | 1× | 1× | 1× | 1× | - | - | 1× | 1× |
| Non-Essential Amino Acids | Invitrogen | 11140-050 | 1× | 1× | 1× | 1× | 1× | 1× | 1× | 1× | 1× | 1× | 1× |
| Ascorbic Acid | Sigma | A4544 | 10^-4^ M | 10^-4^ M | - | 10^-4^ M | 10^-4^ M | 10^-4^ M | 10^-4^ M | - | - | 10^-4^ M | 10^-4^ M |
| d-Biotin | Sigma | B4501 | 10 μg/ml | 10 μg/ml | - | 10 μg/ml | 10 μg/ml | 10 μg/ml | 10 μg/ml | - | - | 10 μg/ml | 10 μg/ml |
| β-Estradiol | Sigma | E2758 | 30 ng/ml | 30 ng/ml | - | 30 ng/ml | 30 ng/ml | 30 ng/ml | 30 ng/ml | - | - | 30 ng/ml | 30 ng/ml |
| FBS | Gibco | 1600-044 | 1% | 1% | 15% | 1% | 1% | 1% | 1% | 15% | - | 1% | 1% |
| Knockout Serum Replacement | Invitrogen | 10828-028 | 50 μl/ml | 50 μl/ml | - | 50 μl/ml | 50 μl/ml | 50 μl/ml | 50 μl/ml | - | 5% | 50 μl/ml | 50 μl/ml |
| N2 | Thermo Fisher Scientific | 17502-048 | 1× | 1× | - | 1× | 1× | 1× | 1× | - | 1× | 1× | 1× |
| B27 | Thermo Fisher Scientific | 12587-010 | - | - | - | - | - | - | - | - | 1× | - | - |
| Penicillin-streptomycin | Thermo Fisher Scientific | 15140-122 | - | - | - | - | - | - | - | - | 1% | - | - |
| Human FGF2 | Peprotech | 100-18b | 10 ng/ml | 10 ng/ml | - | 10 ng/ml | 10 ng/ml | 10 ng/ml | 10 ng/ml | - |  | 10 ng/ml | 10 ng/ml |
| Rat GDNF | Peprotech | 450-51 | 15 ng/ml | 15 ng/ml | - | 15 ng/ml | 15 ng/ml | 15 ng/ml | 15 ng/ml | - |  | 15 ng/ml | 15 ng/ml |
| Mouse EGF | Peprotech | 315-09 | - | 20 ng/ml |  | 20 ng/ml | 20 ng/ml | 20 ng/ml | 20 ng/ml |  |  | 20 ng/ml | - |
| Human LIF | Peprotech | 300-05 | - | 10 ng /ml | 10 ng /ml | 10 ng /ml | 10 ng /ml | 10 ng /ml | 10 ng /ml | 10 ng /ml | 10 ng /ml | - | 10 ng/ml |
| CHIR99021 | Tocris | 4423 | - | - | - | - | - | 3μM | 3μM | 3μM | 3μM | - | - |
| PD0325901 | Tocris | 4192 | - | - | - | - | 1μM | - | 1μM | 1μM | 1μM | - | - |
