# Supplementary material for: A rapid and stable spontaneous reprogramming system of Spermatogonial stem cells to Pluripotent State
Source: Cell Biosci. 2023 Dec 1;13:222. doi: 10.1186/s13578-023-01150-z (PMC10693117; doi:10.1186/s13578-023-01150-z)
Supplement: Supplementary file 9 — Supplementary Material 9 [file 13578_2023_1150_MOESM9_ESM.docx]

**Table S2. Information of primers**

| Gene | Accession  number | Product  Size(bp) | | | Primer Sequence (5’-3’) | Region  Amplified | |  |
| --- | --- | --- | --- | --- | --- | --- | --- | --- |
| *Plzf* | NM_001033324.3 | | 170 | F: ACCAGTGTACCATCTGCACG | | | 2132-2301 | |
|  |  | |  | R: CTGCTCTACCATGTGTTGGG | | |  | |
| *Mvh* | NM_001145885.1 | | 213 | F: GGAAACCAGCAGCAAGTGAT | | | 607-819 | |
|  |  | |  | R: TGGAGTCCTCATCCTCTGG | | |  | |
| *Integrin-β1* | NM_010578.2 | | 239 | F: GTCTGTTTGCAATATGGGGG | | | 3527-3765 | |
|  |  | |  | R: GCACTGTCAAAATGAAAAGGC | | |  | |
| *Gfra1* | NM_010279.3 | | 152 | F: CTCCTCTGGCCACTCAAAGTTA | | | 435-586 | |
|  |  | |  | R: TCCAGGTTGGGTCGGAACT | | |  | |
| *Integrin-a6* | NM_001277970.1 | | 269 | F: GAGGAATATTCCAAACTGAACTAC | | | 3087-3355 | |
|  |  | |  | R: GGAATGCTGTCATCGTACCTAGAG | | |  | |
| *Sox2* | NM_011443.4 | | 157 | F: GCGGAGTGGAAACTTTTGTCC | | | 656-812 | |
|  |  | |  | R: CGGGAAGCGTGTACTTATCCTT | | |  | |
| *Nanog* | NM_001289828.1 | | 364 | F: AGGGTCTGCTACTGAGATGCTCTG | | | 335-698 | |
|  |  | |  | R: CAACCACTGGTTTTTCTGCCACCG | | |  | |
| *Oct4* | NM_001252452.1 | | 209 | F: TTTCCCTCTGTTCCCGTCAC | | | 715-923 | |
|  |  | |  | R: TGATCAACAGCATCACTGAGC | | |  | |
| *Klf4* | NM_010637.3 | | 254 | F: CACTACCGCAAACACACAGG | | | 1952-2205 | |
|  |  | |  | R: TTCACAAGCTGACTTGCTGG | | |  | |
| *Gapdh* | NM_001289726.1 | | 275 | F: CCCACTAACATCAAATGGGG | | | 330-604 | |
|  |  | |  | R: CCTTCCACAATGCCAAAGTT | | |  | |
| *Rac1* | NM_001347530.1 | | 137 | F: GCCATCAAGTGTGTGGTGGT | | | 241-377 | |
|  |  | |  | R: CCATCTACCATAACATTGGCAGAA | | |  | |
| *Kras* | NM_001403240.1 | | 180 | F: GAGAACTGGGGAGGGCTTTC | | | 738-917 | |
|  |  | |  | R: TCCTGAGCCTGTTTCGTGTC | | |  | |
| *Hras* | NM_008284.2 | | 248 | F: TCCATCAGTACAGGGAGCAGA | | | 484-731 | |
|  |  | |  | R: ATCGGGTGGGTTCAGTTTCC | | |  | |
| *Nras* | NM_001368638.1 | | 257 | F: CGCCTTGACGATCCAGCTAA | | | 224-480 | |
|  |  | |  | R: CACGCTTAATTTGCTCCCTGT | | |  | |
| *Smad3* | NM_016769.4 | | 636 | F: CCAGCACACAATAACTTGGA | | | 957- 1592 | |
|  |  | |  | R: AGACACACTGGAACAGCGGA | | |  | |
| *Mapk12* | NM_013871.4 | | 92 | F: ATGCGCTACACGCAGACA | | | 814-905 | |
|  |  | |  | R: TGGTCATTGCCTTTGAACAG | | |  | |
| *Mapk13* | NM_011950.2 | | 78 | F: TCGGAGCTTCCATGATTTCT | | | 384-461 | |
|  |  | |  | R: CTGAATTCCATCCCCATGAT | | |  | |
| *Stat3* | NM_011486.5 | | 215 | F: GACCCGCCAACAAATTAAGA | | | 1136-1350 | |
|  |  | |  | R: TCGTGGTAAACTGGACACCA | | |  | |
| *Snail* | NM_011427.3 | | 200 | F: GAAGATGCACATCCGAAGC | | | 602-801 | |
|  |  | |  | R: ATCTCTTCACATCCGAGTGG | | |  | |
| *Acvr1b* | NM_007395.4 | | 239 | F: GGTGGGGACCAAACGATACAT | | | 1195-1433 | |
|  |  | |  | R: GCCGTAGCTTCTGGTCACAT | | |  | |
| *Bmpr1a* | NM_009758.4 | | 266 | F: GTATGCTCCATGGCACTGGT | | | 489-754 | |
|  |  | |  | R: GGCTTTCGGTGAATCCTTGC | | |  | |
| *Bmpr1b* | NM_001277216.3 | | 279 | F: CCAAGCGCTATATGCCTCCA | | | 1310-1588 | |
|  |  | |  | R: CCATCTGCCTGAGACACTCAT | | |  | |
| *Bmpr2* | NM_007561.4 | | 169 | F: TAAACCCGCAATCTCCCACC | | | 1349-1517 | |
|  |  | |  | R: GCGAATTGTGCCAACCTCAC | | |  | |
| *Fgfr3* | NM_001163215.2 | | 168 | F: CGACAGGTGTCCTTGGAATC | | | 1577-1744 | |
|  |  | |  | R: CTTACCAAGTGTCAGCCGGG | | |  | |
| *Fgfr4* | XM_006517099.3 | | 100 | F: GCCTCCGACAAGGATTTGGCA | | | 1643-1742 | |
|  |  | |  | R: GAGTGCAGACACCCAGCAGGT | | |  | |
| *Nodal* | NM_013611.5 | | 235 | F: AAAGCAGGTGTCCAGTCGAG | | | 841-1075 | |
|  |  | |  | R: GTCTGGCAAATGATGTCGGC | | |  | |
| *Tgfbr1* | NM_009370.3 | | 554 | F: CAGCTCCTCATCGTGTTGGT | | | 124-677 | |
|  |  | |  | R: AGCAGTGGTAAACCTGATCCA | | |  | |
| *Tgfbr2* | NM_009371.3 | | 259 | F: ATGTGGAAATGGAAGCCCAGA | | | 421-679 | |
|  |  | |  | R: TTGTCGTTCTTCCTCCACACG | | |  | |
| *Tgfbr3* | NM_011578.4 | | 135 | F: TGTTGGAGAGATGGCAGTGA | | | 456-590 | |
|  |  | |  | R: TGGACTGGATGAGAGGCACT | | |  | |
| *Zeb2* | NM_001289521.1 | | 130 | F: GAGCAGGTAACCGCAAGTTC | | | 1016-1145 | |
|  |  | |  | R: AAGCGTTTCTTGCAGTTTGG | | |  | |
| *H19*- outside |  | |  | F: GGTTTTTTGGTTATTGAATTTTAAAAATTAG | | |  | |
|  |  | |  | R: AAAAACCATTCCCTAAAATATCACAAATACC | | |  | |
| *H19*- inside |  | | 608 | F: TTAGTGTGGTTTATTATAGGAAGGTATAGAAGT | | |  | |
|  |  | |  | R: TAAACCTAAAATACTCAAACTTTATCACAAC | | |  | |
| *Igf2r* |  | | 251 | TTAGTGGGGTATTTTTATTTGTATGG | | |  | |
|  |  | |  | AAATATCCTAAAAATACAAACTACACAA | | |  | |
| *Meg3IG* |  | | 350 | GGTTTGGTATATATGGATGTATTGTAATATAGG | | |  | |
|  |  | |  | ATAAAACACCAAATCTATACCAAAATATACC | | |  | |
| *Peg10* |  | | 551 | GTAAAGTGATTGGTTTTGTATTTTTAAGTG | | |  | |
|  |  | |  | TTAATTACTCTCCTACAACTTTCCAAATT | | |  | |
