# Supplementary material for: A rapid and stable spontaneous reprogramming system of Spermatogonial stem cells to Pluripotent State
Source: Cell Biosci. 2023 Dec 1;13:222. doi: 10.1186/s13578-023-01150-z (PMC10693117; doi:10.1186/s13578-023-01150-z)
Supplement: Supplementary file 10 — Supplementary Material 10 [file 13578_2023_1150_MOESM10_ESM.docx]

**Table S3. Information of antibodies**

| **Antibodies** | **SOURCE** | **IDENTIFIER** |
| --- | --- | --- |
| Polyclonal rabbit anti-mouse PLZF | Santa Cruz Biotechnology | Sc-22839 |
| Rabbit IgG GFRA1 | Affinity | DF7309 |
| Monoclonal mouse anti-mouse CDH1 | Abcam | Ab76055 |
| Monoclonal rabbit anti-mouse Nanog | Cell Signaling Technology | 8822 |
| Monoclonal rabbit anti-mouse Sox2 | Cell Signaling Technology | 23064 |
| Mouse SSEA1 | Cell Signaling Technology | 4744 |
| Monoclonal mouse anti-mouse OCT4 | Santa Cruz Biotechnology | Sc-5279 |
| Monoclonal rabbit anti-mouse MVH | Cell Signaling Technology | 8761 |
| Monoclonal mouse anti-mouse Tubulin | Absin Bioscience | Abs830032ss |
| Rabbit IgG ITGB1 | Cell Signaling Technology | 8480 |
| Rabbit IgG CCND1 | Abclonal | A19038 |
| Goat anti rabbit IgG – HRP conjugated | Santa Cruz Biotechnology | Sc-2005 |
| Goat anti mouse IgG – HRP conjugated | Santa Cruz Biotechnology | Sc-2004 |
| Goat anti rabbit IgG – red | Santa Cruz Biotechnology | Sc-2091 |
| Goat anti rabbit IgG – FITC | Beyotime | A0562 |
| Goat anti mouse IgG – red | Santa Cruz Biotechnology | Sc-2092 |
| Goat anti mouse IgG – FITC | Beyotime | A0568 |
