# Supplementary material for: A rapid and stable spontaneous reprogramming system of Spermatogonial stem cells to Pluripotent State
Source: Cell Biosci. 2023 Dec 1;13:222. doi: 10.1186/s13578-023-01150-z (PMC10693117; doi:10.1186/s13578-023-01150-z)
Supplement: Supplementary file 11 — Supplementary Material 11 [file 13578_2023_1150_MOESM11_ESM.pdf]

geneID	SSC_1	SSC_2	SSC_3	GSPC p2_1	GSPC p2_2	GSPC p2_3	GSPC p10_1	GSPC p10_2	GSPC p10_3	ESL_1	ESL_2	ESL_3	Gene name	Gene description
ENSMUSG00000000001	36.61774439	48.85077935	33.93724854	48.96705543	57.05783159	64.22180868	42.11266651	39.77715524	50.76739471	61.49579421	61.46623818	62.53001446	Gnai3	guanine nucleotide binding protein (G protein), alpha inhibiting 3 [Source:MGI Symbol;Acc:MGI:95773]
ENSMUSG00000000003	0	0	0	0.100383695	0	0	0	0	0	0	0	0	Pbsn	probasin [Source:MGI Symbol;Acc:MGI:1860484]
ENSMUSG00000000028	22.20735744	19.57078162	21.31652055	26.33778379	25.98313142	24.24263898	58.51334731	54.93021611	54.47407143	35.28890216	33.57832076	36.33928224	Cdc45	cell division cycle 45 [Source:MGI Symbol;Acc:MGI:1338073]
ENSMUSG00000000031	399.4218746	357.7912815	444.2023687	249.2740805	226.9493893	178.1116656	1.542765224	1.609168548	2.090919557	85.0754084	82.62334649	76.2045517	H19	H19, imprinted maternally expressed transcript [Source:MGI Symbol;Acc:MGI:95891]
ENSMUSG00000000037	8.561414388	11.60971498	8.100173621	11.58458829	13.78737393	15.04344412	0.843413606	0.731146616	1.099119081	3.6837539	3.108634476	3.845994504	Scml2	Scm polycomb group protein like 2 [Source:MGI Symbol;Acc:MGI:1340042]
ENSMUSG00000000049	0.839895408	0.446392033	0.463011639	0.241162029	0.475162505	0.147630724	0	0	0	0	0	0	Apoh	apolipoprotein H [Source:MGI Symbol;Acc:MGI:88058]
ENSMUSG00000000056	12.53490962	15.01752826	9.575503124	13.83439152	13.67646606	16.94701459	17.36738781	17.93390007	20.3274685	28.61262262	28.19199442	27.15233718	Narf	nuclear prelamin A recognition factor [Source:MGI Symbol;Acc:MGI:1914858]
ENSMUSG00000000058	40.30071048	44.28044648	34.05265966	52.93580514	63.26699221	73.38967811	1.414043708	1.71433198	1.801802318	14.61335874	13.89216234	15.77823664	Cav2	caveolin 2 [Source:MGI Symbol;Acc:MGI:107571]
ENSMUSG00000000078	114.3430539	128.6931212	110.0445018	154.2356707	167.5315101	197.9896848	17.29231701	15.47947451	19.24160115	45.91843886	45.63763166	49.16647242	Klf6	Kruppel-like factor 6 [Source:MGI Symbol;Acc:MGI:1346318]
ENSMUSG00000000085	52.85718849	53.02216175	56.20163783	57.29883047	53.21731959	53.27639305	10.84390754	12.39219972	12.63068633	13.13735906	12.87641078	11.50668441	Scmh1	sex comb on midleg homolog 1 [Source:MGI Symbol;Acc:MGI:1352762]
ENSMUSG00000000088	51.56688766	51.48693559	71.69794387	59.50407859	57.3610921	48.60038055	112.3906728	114.2106046	81.46850862	66.10298819	57.03391022	54.27322505	Cox5a	cytochrome c oxidase subunit 5A [Source:MGI Symbol;Acc:MGI:88474]
ENSMUSG00000000093	0.795065826	0.929224231	1.008366455	0.885901331	0.935086563	0.596548638	0.063565671	0.176334174	0.088359959	0.583794305	0.83961236	0.504793002	Tbx2	T-box 2 [Source:MGI Symbol;Acc:MGI:98494]
ENSMUSG00000000094	0.099046174	0.016293823	0.173833279	0	0.029732534	0.075441679	0.063161549	0.140170496	0.276564348	0.379690559	0.27089383	0.401267001	Tbx4	T-box 4 [Source:MGI Symbol;Acc:MGI:102556]
ENSMUSG00000000103	0.091194252	0.19252726	0.046682003	0.056733985	0.23953542	0.41676607	0	0	0.014146645	0	0.063258346	0.02693953	Zfy2	zinc finger protein 2, Y-linked [Source:MGI Symbol;Acc:MGI:99213]
ENSMUSG00000000120	0.474487097	0.388228938	0.600828717	0.372870369	0.367333985	0.608687557	13.53326548	11.5408926	15.30607445	14.94396373	15.54905563	15.99014721	Ngfr	nerve growth factor receptor (TNFR superfamily, member 16) [Source:MGI Symbol;Acc:MGI:97323]
ENSMUSG00000000125	0.433642107	0.483252753	0.429638331	0.313291114	0.33803359	0.330297347	0.087420019	0.027715116	0.130198802	3.365820206	3.970596404	3.731470842	Wnt3	wingless-type MMTV integration site family, member 3 [Source:MGI Symbol;Acc:MGI:98955]
ENSMUSG00000000126	4.874574096	5.643180749	4.287097564	5.407835069	5.135141915	5.253322982	1.053254511	0.847636622	1.03773035	2.1337107	1.759008949	1.723391294	Wnt9a	wingless-type MMTV integration site family, member 9A [Source:MGI Symbol;Acc:MGI:2446084]
ENSMUSG00000000127	17.04761888	20.85280221	18.08200062	19.74675791	21.1767339	23.82920791	10.93294722	9.955864082	11.06894802	10.903814	13.49329522	11.19906673	Fer	fer (fms/fps related) protein kinase [Source:MGI Symbol;Acc:MGI:105917]
ENSMUSG00000000131	51.27031739	51.26595325	51.3456691	55.00392353	55.08828196	57.58410452	86.28175833	82.9716577	99.05177312	86.50479248	89.73377158	86.36724108	Xpo6	exportin 6 [Source:MGI Symbol;Acc:MGI:2429950]
ENSMUSG00000000134	31.50016053	34.20491322	30.5728477	39.15928603	37.82395888	39.04783538	26.71720449	25.94411351	29.4966589	31.98796614	29.18559144	28.05713719	Tfe3	transcription factor E3 [Source:MGI Symbol;Acc:MGI:98511]
ENSMUSG00000000142	2.556398394	2.856383842	2.150786333	2.716264677	2.499754073	2.911104186	2.915009515	3.4852899	4.381848343	21.00990164	22.96493781	23.3059341	Axin2	axin 2 [Source:MGI Symbol;Acc:MGI:1270862]
ENSMUSG00000000148	10.20025074	11.24561292	9.789388933	11.86352754	11.24499838	11.78333633	6.851134012	5.920256625	7.079395471	8.868754877	7.733189024	8.425831213	Brat1	BRCA1-associated ATM activator 1 [Source:MGI Symbol;Acc:MGI:1891679]
ENSMUSG00000000149	50.00854324	45.39415599	45.86210546	43.0446643	41.64679899	37.28548049	20.12671516	18.61382845	20.71156883	18.0621206	19.1599804	20.7380981	Gna12	guanine nucleotide binding protein, alpha 12 [Source:MGI Symbol;Acc:MGI:95767]
ENSMUSG00000000154	2.087072975	1.922697453	1.994281335	1.882701467	1.681150076	1.245253816	8.850633315	7.718827716	7.8026237	2.706914369	3.12709988	1.880442349	Slc22a18	solute carrier family 22 (organic cation transporter), member 18 [Source:MGI Symbol;Acc:MGI:1336884]
ENSMUSG00000000157	0	0	0	0	0	0	0	0	0	0	0	0	Itgb2l	integrin beta 2-like [Source:MGI Symbol;Acc:MGI:1277979]
ENSMUSG00000000159	0.371897043	0.128477569	0.038074546	0.104114463	0.117221223	0.254940524	0.033202121	0.147366803	0.069229303	0.033265312	0.061913354	0.197750586	Igsf5	immunoglobulin superfamily, member 5 [Source:MGI Symbol;Acc:MGI:1919308]
ENSMUSG00000000167	2.573272311	1.94311826	2.893628199	2.086406201	1.595587706	2.89182535	0.527262703	0.710430795	0.628221985	0.830132608	0.596947925	0.859859616	Pih1d2	PIH1 domain containing 2 [Source:MGI Symbol;Acc:MGI:1919864]
ENSMUSG00000000168	99.55826138	118.9019839	86.26898997	119.9482718	135.2020881	138.5092539	78.75698398	79.16228859	88.50998708	84.37242968	94.7807113	93.77581663	Dlat	dihydrolipoamide S-acetyltransferase (E2 component of pyruvate dehydrogenase complex) [Source:MGI Symbol;Acc:MGI:2385311]
ENSMUSG00000000171	122.1995073	114.4750792	121.1347416	125.1248911	115.8669735	99.8130003	125.5738203	134.2142857	118.9111688	92.45240988	94.03748298	94.6972296	Sdhd	succinate dehydrogenase complex, subunit D, integral membrane protein [Source:MGI Symbol;Acc:MGI:1914175]
ENSMUSG00000000182	0	0	0	0	0	0	0.084707506	0.234982622	0.022077812	0.148520267	0.217191637	0.105107235	Fgf23	fibroblast growth factor 23 [Source:MGI Symbol;Acc:MGI:1891427]
ENSMUSG00000000183	0	0.010639793	0	0.034488708	0	0	0	0	0	0	0	0	Fgf6	fibroblast growth factor 6 [Source:MGI Symbol;Acc:MGI:95520]
ENSMUSG00000000184	111.9423259	161.6481147	107.6444552	120.4849323	162.1872556	212.5612199	9.304879235	8.686414357	13.70809884	108.2782263	105.5494256	107.302597	Ccnd2	cyclin D2 [Source:MGI Symbol;Acc:MGI:88314]
ENSMUSG00000000194	71.60966219	77.96665505	69.17078491	68.76615476	70.50701305	77.11661942	34.36984626	33.02797471	35.9381459	46.36398068	49.43203212	44.92611751	Gpr107	G protein-coupled receptor 107 [Source:MGI Symbol;Acc:MGI:2139054]
ENSMUSG00000000197	0	0.027307531	0.036416851	0.044258449	0.049830056	0.1444983	0.074098644	0.058729468	0.044143457	0.328775684	0.315828056	0.430820913	Nalcn	sodium leak channel, non-selective [Source:MGI Symbol;Acc:MGI:2444306]
ENSMUSG00000000202	0.211516443	0.243572057	0.30935569	0.225581336	0.317474146	0.253170103	0.431627574	0.628611518	0.618736892	1.675740109	1.408528811	1.446051161	Btbd17	BTB (POZ) domain containing 17 [Source:MGI Symbol;Acc:MGI:1919264]
ENSMUSG00000000204	0.679969782	1.613021526	0.723993998	0.456866372	0.38578527	0.725088952	0.060706154	0.040416417	0.01265776	0.231122428	0.29432325	0.349511453	Slfn4	schlafen 4 [Source:MGI Symbol;Acc:MGI:1329010]
ENSMUSG00000000214	0	0	0	0	0.035965249	0.104292826	0.091682229	0	0	0	0	0.03033644	Th	tyrosine hydroxylase [Source:MGI Symbol;Acc:MGI:98735]
ENSMUSG00000000215	0	0	0	0	0	0	0	0	0	0	0	0	Ins2	insulin II [Source:MGI Symbol;Acc:MGI:96573]
ENSMUSG00000000216	0	0.198794446	0.029456536	0.134247669	0.015114784	0.010957546	0.038530445	0.08550822	0	0.012867926	0.035924649	0	Scnn1g	sodium channel, nonvoltage-gated 1 gamma [Source:MGI Symbol;Acc:MGI:104695]
ENSMUSG00000000223	0.717866582	0.663833248	0.545642128	0.456750885	0.571389261	0.546785986	0.359289586	0.430999471	0.364451328	0.69075976	0.660928188	1.050679123	Drp2	dystrophin related protein 2 [Source:MGI Symbol;Acc:MGI:107432]
ENSMUSG00000000244	0.584474932	0.416651813	0.512898126	0.571394898	0.701810893	0.678375589	1.192699221	0.661720792	0.803055432	0.323637492	0.48651642	0.517976066	Tspan32	tetraspanin 32 [Source:MGI Symbol;Acc:MGI:1350360]
ENSMUSG00000000247	0.706604381	0.642387843	0.989938207	0.38175303	0.117221223	0.339920698	1.128872116	1.252617824	1.176898143	0.266122499	0.526263512	0.164792155	Lhx2	LIM homeobox protein 2 [Source:MGI Symbol;Acc:MGI:96785]
ENSMUSG00000000248	0.131989226	0.189990474	0.067564801	0.061585089	0.034668956	0.201067611	0	0	0	0.295153116	0.082400787	0	Clec2g	C-type lectin domain family 2, member g [Source:MGI Symbol;Acc:MGI:1918059]
ENSMUSG00000000253	21.61361687	18.59537249	21.21222671	19.10304026	13.78175063	10.82375277	23.15537393	26.1366341	20.25580643	24.79940558	24.27772045	20.78375406	Gmpr	guanosine monophosphate reductase [Source:MGI Symbol;Acc:MGI:1913605]
ENSMUSG00000000263	0	0	0	0	0.021787141	0	0	0	0	0	0.069044579	0	Glra1	glycine receptor, alpha 1 subunit [Source:MGI Symbol;Acc:MGI:95747]
ENSMUSG00000000266	1.152986846	0.946356199	0.577652084	0.377726559	0.683021913	1.223887316	0.131407711	0.109359358	0.068499119	0.800918342	0.714703901	0.858751274	Mid2	midline 2 [Source:MGI Symbol;Acc:MGI:1344333]
ENSMUSG00000000275	41.03908682	45.02676432	40.22025036	51.62726714	55.63769208	62.92362017	41.17054308	39.1414183	46.08134022	67.77587786	70.78015526	71.39468523	Trim25	tripartite motif-containing 25 [Source:MGI Symbol;Acc:MGI:102749]
ENSMUSG00000000276	3.265218232	2.900374732	2.177135394	4.278533916	4.191237365	5.600187543	5.756168985	5.879169671	7.601337382	10.07054571	10.28809304	9.603397133	Dgke	diacylglycerol kinase, epsilon [Source:MGI Symbol;Acc:MGI:1889276]
ENSMUSG00000000278	143.7565979	143.757393	133.5852451	128.7323946	129.9119344	120.5933921	102.2564213	98.91148064	99.78865842	88.41036434	95.05050646	91.61353067	Scpep1	serine carboxypeptidase 1 [Source:MGI Symbol;Acc:MGI:1921867]
ENSMUSG00000000282	15.32728199	15.86279583	14.72086718	18.70878476	17.64392785	21.98617917	16.12808002	13.1977825	14.62717007	22.30990836	24.51194985	24.3202343	Mnt	max binding protein [Source:MGI Symbol;Acc:MGI:109150]
ENSMUSG00000000290	13.63326185	14.04683322	11.6558483	5.736211119	7.71481586	7.141420839	0.747370702	0.521272179	0.445237927	10.01243372	10.27321164	9.835279475	Itgb2	integrin beta 2 [Source:MGI Symbol;Acc:MGI:96611]
ENSMUSG00000000295	19.44488049	13.51723934	23.45070035	16.94703169	14.01982675	11.52254125	33.82800582	35.55688457	30.08667602	16.8185392	16.54228826	17.4222475	Hddc2	HD domain containing 2 [Source:MGI Symbol;Acc:MGI:1916942]
ENSMUSG00000000296	3.48305627	2.788077385	2.783162895	1.189145444	1.69586979	1.779438435	25.82479331	25.41560909	24.47228509	19.79489682	19.552549	17.01487949	Tpd52l1	tumor protein D52-like 1 [Source:MGI Symbol;Acc:MGI:1298386]
ENSMUSG00000000301	8.448188958	4.781816816	7.036632311	7.148788899	8.199179201	5.289650585	6.519659094	7.376186624	5.131089297	8.261144196	7.151456002	8.184932953	Pemt	phosphatidylethanolamine N-methyltransferase [Source:MGI Symbol;Acc:MGI:104535]
ENSMUSG00000000303	2.299349189	1.147388142	0.659135913	1.130076505	0.982439408	3.350959716	153.2351696	141.0737888	173.7793607	166.3469925	169.2465395	170.3550498	Cdh1	cadherin 1 [Source:MGI Symbol;Acc:MGI:88354]
ENSMUSG00000000305	1.176560214	0.806469981	0.831715661	0.36598222	0.765244972	0.490756819	3.65141191	2.830622699	3.884976287	2.079753391	2.191918161	2.358480433	Cdh4	cadherin 4 [Source:MGI Symbol;Acc:MGI:99218]
ENSMUSG00000000308	0.718738661	0.165532903	0.049055958	0.089428681	0.201373356	0.437960187	51.54777189	49.60357555	48.70119539	19.24398319	16.7916532	19.49109494	Ckmt1	creatine kinase, mitochondrial 1, ubiquitous [Source:MGI Symbol;Acc:MGI:99441]
ENSMUSG00000000317	14.60680773	17.25957906	15.61497621	11.35902747	13.06634423	13.2481215	9.976863941	9.995427136	11.13841433	15.40043372	17.04170353	16.37609404	Bcl6b	B cell CLL/lymphoma 6, member B [Source:MGI Symbol;Acc:MGI:1278332]
ENSMUSG00000000318	0.09734952	0.728669347	0.498328622	0.272534917	0.153421895	0.333672156	0.173822869	0	0	1.131999011	1.134471169	0.81959863	Clec10a	C-type lectin domain family 10, member A [Source:MGI Symbol;Acc:MGI:96975]
ENSMUSG00000000320	0.113318064	0.260983152	0.149161118	0.090639905	0.170083967	0.086312319	1.055035496	1.122576877	1.20539207	1.346644422	1.051059551	1.075984895	Alox12	arachidonate 12-lipoxygenase [Source:MGI Symbol;Acc:MGI:87998]
ENSMUSG00000000325	3.386427054	3.530010251	2.64543539	3.130203979	3.726608861	4.107449062	15.91902382	15.32686206	17.29836534	10.82429192	11.70294029	10.61064785	Arvcf	armadillo repeat gene deleted in velocardiofacial syndrome [Source:MGI Symbol;Acc:MGI:109620]
ENSMUSG00000000326	28.30216044	28.21937655	21.61387142	28.18258805	27.01620094	26.57412235	9.860497302	9.345774095	11.35082904	12.14326212	11.70752146	13.63710161	Comt	catechol-O-methyltransferase [Source:MGI Symbol;Acc:MGI:88470]
ENSMUSG00000000339	89.36685933	73.07192304	84.8126839	82.24844251	80.48074255	65.87217994	83.62618232	85.06432904	82.87093661	58.7470168	56.67725351	56.02603689	Rtca	RNA 3'-terminal phosphate cyclase [Source:MGI Symbol;Acc:MGI:1913618]
ENSMUSG00000000340	10.1285756	12.65622018	9.266416569	11.22822627	12.75493456	14.8276454	34.31042893	32.69879302	37.5419008	42.31106153	40.271562	38.896836	Dbt	dihydrolipoamide branched chain transacylase E2 [Source:MGI Symbol;Acc:MGI:105386]
ENSMUSG00000000346	143.8584293	149.4171536	120.8134101	161.4028628	169.3792527	161.0909205	66.93798253	61.53638406	69.08168019	75.78189035	72.18933848	76.69958596	Dazap2	DAZ associated protein 2 [Source:MGI Symbol;Acc:MGI:1344344]
ENSMUSG00000000355	95.65166419	89.07014129	97.7763495	100.3492576	90.33909241	90.90152894	44.21332261	42.33338905	39.63698187	46.54205196	51.42148332	50.8874783	Mcts1	malignant T cell amplified sequence 1 [Source:MGI Symbol;Acc:MGI:1916245]

ENSMUSG00000000359	24.86589723	20.26829621	24.88242853	28.90071903	28.09289756	21.76377524	0.167166467	0.370982138	0.209133951	0.87092004	1.745644062	0.896073478	Rem1	rad and gem related GTP binding protein 1 [Source:MGI Symbol;Acc:MGI:1097696]
ENSMUSG00000000365	3.592629534	4.687116343	3.401718162	3.837060141	5.912864031	5.50451698	17.89108792	17.54818923	20.08256198	27.5285551	27.05280411	27.09055777	Rnf17	ring finger protein 17 [Source:MGI Symbol;Acc:MGI:1353419]
ENSMUSG00000000374	16.99842186	22.02705323	15.58585571	25.46856706	27.8045661	34.24599773	14.11948349	11.33042698	17.60180399	17.7799588	19.48424894	19.03150009	Trappc10	trafficking protein particle complex 10 [Source:MGI Symbol;Acc:MGI:1336209]
ENSMUSG00000000378	78.16149369	78.56578158	86.78950785	75.72403019	70.88854835	59.93703686	45.96966295	43.89826106	44.19822956	55.41726501	55.28188507	54.2029611	Ccm2	cerebral cavernous malformation 2 [Source:MGI Symbol;Acc:MGI:2384924]
ENSMUSG00000000381	0	0	0	0	0	0	0	0	0	0	0	0	Wap	whey acidic protein [Source:MGI Symbol;Acc:MGI:98943]
ENSMUSG00000000384	44.12428973	40.25372364	44.76277036	48.74318617	46.58989684	42.67856338	115.7011055	125.2722281	123.1732394	127.6833384	138.9804226	129.9367397	Tbrg4	transforming growth factor beta regulated gene 4 [Source:MGI Symbol;Acc:MGI:1100868]
ENSMUSG00000000385	0.2433738	0.588540627	0.22424788	0.476936106	0.178992211	0.111224052	0.260734304	0.433973563	0.18121788	0.522461082	0.668527653	0.69018832	Tmprss2	transmembrane protease, serine 2 [Source:MGI Symbol;Acc:MGI:1354381]
ENSMUSG00000000386	5.584489011	7.491912486	5.745945678	5.810659025	5.192649155	6.550550523	0.149571138	0.11064464	0.233901599	1.898173552	1.464284981	2.078626862	Mx1	MX dynamin-like GTPase 1 [Source:MGI Symbol;Acc:MGI:97243]
ENSMUSG00000000392	2.617528075	2.802694053	1.457438374	1.778409104	1.375066246	2.413455095	0.102494274	0.045491833	0.170967563	1.72518099	1.261426778	1.953446558	Fap	fibroblast activation protein [Source:MGI Symbol;Acc:MGI:109608]
ENSMUSG00000000394	0	0	0.316164475	0	0.054076936	0	0	0	0	0	0	0	Gcg	glucagon [Source:MGI Symbol;Acc:MGI:95674]
ENSMUSG00000000399	55.99794045	51.98367168	54.5059482	55.93402866	50.55351502	45.08133397	69.50497743	75.81083321	69.49411472	68.41949059	72.64771746	66.32775845	Ndufa9	NADH:ubiquinone oxidoreductase subunit A9 [Source:MGI Symbol;Acc:MGI:1913358]
ENSMUSG00000000402	7.622280203	9.345507754	5.767195477	0.445489769	0.234066737	0.48482279	0.241513506	0.425627917	0.429521226	0.996360076	1.258359161	1.08588523	Egfl6	EGF-like-domain, multiple 6 [Source:MGI Symbol;Acc:MGI:1858599]
ENSMUSG00000000409	1.392873184	1.213811406	1.580176954	1.756495093	1.582093381	1.11827367	30.92009252	29.94624296	27.04982063	19.15980128	21.46505661	19.14985765	Lck	lymphocyte protein tyrosine kinase [Source:MGI Symbol;Acc:MGI:96756]
ENSMUSG00000000411	0	0.038710559	0.03441582	0.031369905	0	0.051209407	0.210081233	0.199808661	0.250307197	0.090206171	0	0.089373995	Tssk3	testis-specific serine kinase 3 [Source:MGI Symbol;Acc:MGI:1929914]
ENSMUSG00000000416	0.290320053	0.316722756	0.344158205	0.099813333	0.064216361	0.023277003	0.231907855	0.060548079	0.213329997	1.243751754	1.119277359	1.841645965	Cttnbp2	cortactin binding protein 2 [Source:MGI Symbol;Acc:MGI:1353467]
ENSMUSG00000000420	69.42247457	82.71572366	65.4344664	80.20576243	90.4622096	103.6412326	31.50250382	27.71539493	34.17500682	49.95287974	51.14375415	50.99122789	Galnt1	polypeptide N-acetylgalactosaminyltransferase 1 [Source:MGI Symbol;Acc:MGI:894693]
ENSMUSG00000000435	0	0	0	0	0	0	0	0	0	0.01847718	0.085974257	0	Myf5	myogenic factor 5 [Source:MGI Symbol;Acc:MGI:97252]
ENSMUSG00000000439	9.613631992	10.38107363	9.025006805	11.47020492	12.04040639	11.98462448	17.5071948	16.59228011	17.33863537	11.27710804	11.51899513	11.37943658	Mkrn2	makorin, ring finger protein, 2 [Source:MGI Symbol;Acc:MGI:1914277]
ENSMUSG00000000440	0.184197294	0.169690122	0.150863871	0.206267866	0.154823008	0.056119898	0	0.14597893	0.068577314	0.098856078	0.061330266	0.228536244	Pparg	peroxisome proliferator activated receptor gamma [Source:MGI Symbol;Acc:MGI:97747]
ENSMUSG00000000441	9.315445818	13.63250204	5.761988911	13.3499965	15.58405304	21.47627512	21.38558886	17.46780872	26.94027976	26.03889477	29.12428921	29.01737142	Raf1	v-raf-leukemia viral oncogene 1 [Source:MGI Symbol;Acc:MGI:97847]
ENSMUSG00000000486	7.761797441	5.038933792	4.853214005	3.840344732	2.353459672	5.634274734	313.1322185	344.773957	298.4292102	113.8796487	117.8141049	122.2007248	Septin1	septin 1 [Source:MGI Symbol;Acc:MGI:1858916]
ENSMUSG00000000489	4.510147487	4.774302287	4.732175314	3.895096483	3.679062366	3.051227182	1.850715626	1.998086611	1.851230311	4.38502221	3.521060024	4.865917525	Pdgfb	platelet derived growth factor, B polypeptide [Source:MGI Symbol;Acc:MGI:97528]
ENSMUSG00000000530	27.42568742	27.42568963	27.55447882	11.05944535	11.70519207	10.52059051	8.601476572	7.883247923	9.152580203	22.85381887	25.45505813	25.76615687	Acvrl1	activin A receptor, type II-like 1 [Source:MGI Symbol;Acc:MGI:1338946]
ENSMUSG00000000531	21.77880668	23.42207797	23.55376093	24.01703347	21.88144872	21.13464882	21.23870928	21.65887884	21.4330924	7.705165919	9.954025896	6.827534337	Grasp	GRP1 (general receptor for phosphoinositides 1)-associated scaffold protein [Source:MGI Symbol;Acc:MGI:1860303]
ENSMUSG00000000532	11.74566689	12.32569436	10.16053485	12.66043337	14.04957472	15.97158588	15.96416299	14.95474512	17.00593215	13.40719336	13.58738871	14.02579677	Acvr1b	activin A receptor, type 1B [Source:MGI Symbol;Acc:MGI:1338944]
ENSMUSG00000000538	16.64792684	18.07312028	16.16997279	16.66422443	17.2521162	18.96644713	3.696667505	3.368907937	4.224761685	10.00381666	10.96773369	10.8194551	Tom1l2	target of myb1-like 2 (chicken) [Source:MGI Symbol;Acc:MGI:2443306]
ENSMUSG00000000544	0.456838623	0.032879572	0.14615876	0.106578574	0.089996652	0.739427141	37.93052612	38.4679183	41.59052564	50.79798614	50.3147481	49.29190334	Gpa33	glycoprotein A33 (transmembrane) [Source:MGI Symbol;Acc:MGI:1891703]
ENSMUSG00000000552	45.9233708	39.59198955	51.21146255	36.90822078	32.21265497	27.21861653	29.05028938	28.08203509	28.02915716	22.64888191	24.73852343	22.19005201	Zfp385a	zinc finger protein 385A [Source:MGI Symbol;Acc:MGI:1352495]
ENSMUSG00000000555	88.94196012	91.58354871	90.3917595	91.56831536	85.88841184	90.18885237	40.86486112	40.11639469	46.28738813	59.73030268	64.31223103	64.0433248	Itga5	integrin alpha 5 (fibronectin receptor alpha) [Source:MGI Symbol;Acc:MGI:96604]
ENSMUSG00000000560	0.145694654	0.055924961	0.049720373	0.030213302	0	0	0	0	0	0.072400238	0	0.028692931	Gabra2	gamma-aminobutyric acid (GABA) A receptor, subunit alpha 2 [Source:MGI Symbol;Acc:MGI:95614]
ENSMUSG00000000561	15.37458983	13.06156186	17.49164123	16.30463038	17.52276987	14.03714561	49.41390126	49.02073351	41.84975228	34.55355997	33.0539078	31.25707095	Wdr77	WD repeat domain 77 [Source:MGI Symbol;Acc:MGI:1917715]
ENSMUSG00000000562	0.172015657	0.141489194	0.125791691	0	0	0.018717316	0	0	0	0.021980563	0.081820389	0.087111147	Adora3	adenosine A3 receptor [Source:MGI Symbol;Acc:MGI:104847]
ENSMUSG00000000563	222.8410703	201.4018841	214.1743147	230.0022508	219.0341022	192.9864386	342.3311309	362.3008278	331.2125135	283.0178005	277.3031962	268.2813658	Atp5pb	ATP synthase peripheral stalk-membrane subunit b [Source:MGI Symbol;Acc:MGI:1100495]
ENSMUSG00000000567	25.09908407	25.83524926	24.1728067	36.60909587	38.97645826	41.6035875	0.33444613	0.298947904	0.21307892	1.191405005	0.978792486	0.793090642	Sox9	SRY (sex determining region Y)-box 9 [Source:MGI Symbol;Acc:MGI:98371]
ENSMUSG00000000568	97.61810931	93.44808778	96.13722385	110.8877616	109.9331143	107.4024929	291.6261667	285.692054	291.801421	141.0812118	141.9059068	135.5044906	Hnrnpd	heterogeneous nuclear ribonucleoprotein D [Source:MGI Symbol;Acc:MGI:101947]
ENSMUSG00000000579	6.53286402	4.836168477	6.084842778	4.194113267	4.309240391	4.134165845	8.638956909	9.950897094	9.806555938	4.635251658	5.233516052	4.505428807	Dynlt1c	dynein light chain Tctex-type 1C [Source:MGI Symbol;Acc:MGI:3807476]
ENSMUSG00000000581	42.98588536	40.08114284	40.51934696	40.15347784	35.62952614	32.93626826	45.41320372	51.48864772	44.64885605	63.10502209	63.90297982	65.20349148	C1d	C1D nuclear receptor co-repressor [Source:MGI Symbol;Acc:MGI:1927354]
ENSMUSG00000000594	13.6164781	14.6355366	13.92977623	13.05693415	13.7804857	13.03972423	10.56478298	10.65904406	11.23744063	9.465782861	9.182033985	9.359978786	Gm2a	GM2 ganglioside activator protein [Source:MGI Symbol;Acc:MGI:95762]
ENSMUSG00000000600	17.46929459	21.35162093	12.90994966	19.97540522	24.37256027	30.56151577	11.35480071	10.1387663	14.528872	21.0408977	18.17960651	19.19877473	Krit1	KRIT1, ankyrin repeat containing [Source:MGI Symbol;Acc:MGI:1930618]
ENSMUSG00000000605	20.93567361	26.77351815	20.75369955	21.4165213	24.22517457	26.53912229	1.970483052	2.016661121	2.493099572	9.219478	9.828236249	9.381302249	Clcn4	chloride channel, voltage-sensitive 4 [Source:MGI Symbol;Acc:MGI:104571]
ENSMUSG00000000606	0.010107702	0.011639539	0.020696383	0.028297025	0	0	0	0.010013119	0.028223504	0	0	0	Vmn2r88	vomeronasal 2, receptor 88 [Source:MGI Symbol;Acc:MGI:1316662]
ENSMUSG00000000617	0	0	0	0	0	0	0.011477399	0	0.107691175	0.160989402	0.032103563	0.125324754	Grm6	glutamate receptor, metabotropic 6 [Source:MGI Symbol;Acc:MGI:1351343]
ENSMUSG00000000627	0.976472837	0.757787363	1.108369393	1.208368105	1.271274862	1.212654932	1.591932778	1.829525777	1.620143278	3.360814034	3.622328304	3.348622119	Sema4f	sema domain, immunoglobulin domain (Ig), TM domain, and short cytoplasmic domain [Source:MGI Symbol;Acc:MGI:1340055]
ENSMUSG00000000628	25.84271579	31.40313836	23.7593591	30.70861469	34.555231	38.12715714	119.607287	107.117613	131.8163907	102.7109745	112.1733961	110.1491092	Hk2	hexokinase 2 [Source:MGI Symbol;Acc:MGI:1315197]
ENSMUSG00000000631	15.84680882	17.64935083	14.7591938	14.42388357	17.3223224	17.66337592	31.51071964	27.35348516	35.56096481	21.0918709	21.84321422	23.11380668	Myo18a	myosin XVIIIA [Source:MGI Symbol;Acc:MGI:2667185]
ENSMUSG00000000632	1.796255659	1.936850136	1.738684638	1.889575428	1.818626848	1.791066023	0.218680384	0.258828677	0.243182704	0.204490144	0.19029839	0.303905502	Sez6	seizure related gene 6 [Source:MGI Symbol;Acc:MGI:104745]
ENSMUSG00000000673	0.075158931	0.173098745	0.038473581	0.280548317	0.276382732	0.143117994	0.771652091	0.744556292	0.839458198	0.672278889	0.437935595	0.53286155	Haao	3-hydroxyanthranilate 3,4-dioxygenase [Source:MGI Symbol;Acc:MGI:1349444]
ENSMUSG00000000682	26.99646024	21.96306153	30.94033847	0.171963503	0.193611642	0.350899579	1.974210916	2.099345818	1.114858608	17.30722257	21.78156881	18.61734961	Cd52	CD52 antigen [Source:MGI Symbol;Acc:MGI:1346088]
ENSMUSG00000000686	0.384182927	0.241312576	0.178783483	0.097776326	0.110085191	0.399034342	0.124723552	0.207593414	0.130029713	0.156201162	0.552370645	0.247616264	Abhd15	abhydrolase domain containing 15 [Source:MGI Symbol;Acc:MGI:1914727]
ENSMUSG00000000690	15.73957467	13.05839334	19.73369212	12.70834356	11.63914709	9.874096297	0.514380274	0.726429541	0.316883122	3.302984333	3.335351155	3.040420276	Hoxb6	homeobox B6 [Source:MGI Symbol;Acc:MGI:96187]
ENSMUSG00000000693	66.73223444	77.22927822	63.61749185	66.13775276	69.78784099	66.34563912	2.953354378	2.687691724	4.452366956	43.95887752	45.93989725	41.86604857	Loxl3	lysyl oxidase-like 3 [Source:MGI Symbol;Acc:MGI:1337004]
ENSMUSG00000000701	0	0	0	0	0	0	0	0	0	0	0	0	Tcl1b5	T cell leukemia/lymphoma 1B, 5 [Source:MGI Symbol;Acc:MGI:1351635]
ENSMUSG00000000706	0.27582364	0.404250127	0.462086541	1.07637528	1.053806955	0.744864105	0.179090229	0.09936095	0.280063997	0.426148812	0	0.133331835	Btn1a1	butyrophilin, subfamily 1, member A1 [Source:MGI Symbol;Acc:MGI:103118]
ENSMUSG00000000708	21.75872396	26.79239907	18.68883342	27.49144363	30.09606799	42.92279407	43.65324326	40.6075784	52.79206337	65.45870038	58.88077257	64.06039102	Kat2b	K(lysine) acetyltransferase 2B [Source:MGI Symbol;Acc:MGI:1343094]
ENSMUSG00000000711	51.40159897	64.63069876	45.8473107	63.92834371	74.87099043	79.91137506	24.69412241	20.20518353	26.76049335	38.63199618	38.75073181	37.32426513	Rab5b	RAB5B, member RAS oncogene family [Source:MGI Symbol;Acc:MGI:105938]
ENSMUSG00000000724	0	0	0	0.148992497	0	0.060805233	0.142541202	0.039541603	0.148605386	0	0.033225302	0	Cryba1	crystallin, beta A1 [Source:MGI Symbol;Acc:MGI:88518]
ENSMUSG00000000730	5.477003858	1.97868282	3.591614409	0.467677778	0.714607362	2.481227852	177.6287512	190.6105918	164.3281325	226.6691464	231.8859609	226.8939554	Dnmt3l	DNA (cytosine-5-)-methyltransferase 3-like [Source:MGI Symbol;Acc:MGI:1859287]
ENSMUSG00000000731	0.883282741	0.494042091	0.645927422	0.612311099	0.29166657	0.903448076	38.07689348	37.85075274	38.17001245	37.17869831	37.39239786	38.96042227	Aire	autoimmune regulator (autoimmune polyendocrinopathy candidiasis ectodermal dystrophy) [Source:MGI Symbol;Acc:MGI:1338803]
ENSMUSG00000000732	0.392595692	0.632931769	0.462227078	0.293091079	0.185618095	0.388742946	10.90056534	10.61756973	11.76626532	13.18634249	13.92152277	13.67356893	Icosl	icos ligand [Source:MGI Symbol;Acc:MGI:1354701]
ENSMUSG00000000738	83.44059141	76.30262406	88.58859106	69.65819452	69.91557512	62.25863407	79.72841142	87.93727994	84.38194856	63.92104012	68.66869094	64.83990607	Spg7	SPG7, paraplegin matrix AAA peptidase subunit [Source:MGI Symbol;Acc:MGI:2385906]
ENSMUSG00000000739	0.696253848	0.962126517	0.962306436	0.227406428	0.731526187	0.238645781	1.926958699	2.68998068	2.073742474	4.452895786	3.332476278	4.134149861	Sult5a1	sulfotransferase family 5A, member 1 [Source:MGI Symbol;Acc:MGI:1931463]
ENSMUSG00000000740	1639.824808	1223.156597	1935.626476	1589.939682	1318.355015	1042.439198	3811.615819	4295.987229	3169.025783	1921.674196	1922.486183	1957.154946	Rpl13	ribosomal protein L13 [Source:MGI Symbol;Acc:MGI:105922]
ENSMUSG00000000743	167.2065052	160.7646693	161.5785089	164.6439137	145.3183445	132.7108609	128.7037812	127.3330523	130.817484	121.0123468	129.9015329	124.3842466	Chmp1a	charged multivesicular body protein 1A [Source:MGI Symbol;Acc:MGI:1920159]
ENSMUSG00000000751	63.00600003	63.50491718	61.75679379	73.69942134	71.75696461	68.73358125	182.4879234	185.2066104	187.2098674	153.7952689	156.868847	156.5483307	Rpa1	replication protein A1 [Source:MGI Symbol;Acc:MGI:1915525]
ENSMUSG00000000753	1186.609289	1022.228051	1216.012356	872.3682521	786.7776176	679.744271	18.83626903	23.11474108	19.29904765	162.2258486	166.7993621	160.4529468	Serpinf1	serine (or cysteine) peptidase inhibitor, clade F, member 1 [Source:MGI Symbol;Acc:MGI:108080]
ENSMUSG00000000759	19.01171901	22.53814773	16.44311422	24.60794735	25.35119241	30.75409402	21.29156625	18.61180676	22.51026529	25.44148124	26.22529034	23.73376099	Tubgcp3	tubulin, gamma complex associated protein 3 [Source:MGI Symbol;Acc:MGI:2183752]
ENSMUSG00000000766	0	0	0	0	0	0	0	0	0.027207304	0	0	0.077716518	Oprm1	opioid receptor, mu 1 [Source:MGI Symbol;Acc:MGI:97441]

ENSMUSG00000000776	40.28836522	44.47130656	42.78109074	42.63060781	40.53004461	35.74161853	45.33335018	50.51481056	47.74018261	44.92177568	41.30523252	43.55878068	Polr3d	polymerase (RNA) III (DNA directed) polypeptide D [Source:MGI Symbol;Acc:MGI:1914315]
ENSMUSG00000000782	7.247920465	7.678644757	6.282578074	9.153459855	8.528913512	11.59328074	27.86748538	27.8827134	32.92080732	45.53292821	43.43877093	44.96299823	Tcf7	transcription factor 7, T cell specific [Source:MGI Symbol;Acc:MGI:98507]
ENSMUSG00000000787	129.3040209	173.9109348	105.8932186	152.0733303	188.6406782	259.3205366	239.684299	213.3186867	303.0258658	563.1461492	533.4099246	546.8863817	Ddx3x	DEAD/H (Asp-Glu-Ala-Asp/His) box polypeptide 3, X-linked [Source:MGI Symbol;Acc:MGI:103064]
ENSMUSG00000000791	0.524382384	0.637400675	0.715811784	0.761203371	0.826421527	0.887583497	0.36412184	0.606054707	0.976147227	0.547222271	0.630493407	0.490538381	Il12rb1	interleukin 12 receptor, beta 1 [Source:MGI Symbol;Acc:MGI:104579]
ENSMUSG00000000792	0.41147461	0.287685029	0.240722679	0.164563434	0.370559986	0.358186018	4.158985226	3.217319789	3.775124939	0.972714997	1.455672193	1.484682765	Slc5a5	solute carrier family 5 (sodium iodide symporter), member 5 [Source:MGI Symbol;Acc:MGI:2149330]
ENSMUSG00000000794	0.653227624	0.769924604	0.590090867	0.258175603	0.637874111	0.632183288	0.288162863	0.243619776	0.493550894	1.574164011	1.663223366	1.423428662	Kcnn3	potassium intermediate/small conductance calcium-activated channel, subfamily N, member 3 [Source:MGI Symbol;Acc:MGI:215318
ENSMUSG00000000804	32.36753427	41.70259361	24.33282019	38.25605934	49.74069765	63.03662207	24.38337481	21.66677827	30.35654078	67.93150642	65.5270423	64.94282044	Usp32	ubiquitin specific peptidase 32 [Source:MGI Symbol;Acc:MGI:2144475]
ENSMUSG00000000805	0.506963038	0.729742498	0	0.295680986	0.066580734	0.386144573	1.47096643	1.757766915	1.887441603	15.13444333	16.61607625	17.52204162	Car4	carbonic anhydrase 4 [Source:MGI Symbol;Acc:MGI:1096574]
ENSMUSG00000000811	6.183831459	5.484843193	5.322635859	7.458150667	6.344431918	7.477149924	5.535198485	5.614136039	5.364933243	3.668271467	4.220074634	4.192473198	Txnrd3	thioredoxin reductase 3 [Source:MGI Symbol;Acc:MGI:2386711]
ENSMUSG00000000817	0	0.169110975	0.112761733	0.068521293	0.03857365	0.027964181	0	0.072740354	0	0	0.15280237	0	Fasl	Fas ligand (TNF superfamily, member 6) [Source:MGI Symbol;Acc:MGI:99255]
ENSMUSG00000000823	19.91028611	25.91961723	16.9369095	25.33404886	32.22798788	34.69240139	17.183724	16.28344151	21.14733386	27.72176999	31.31493919	29.25167365	Zfp512b	zinc finger protein 512B [Source:MGI Symbol;Acc:MGI:2685478]
ENSMUSG00000000826	44.08955102	53.01451672	42.23633116	50.15859543	53.71087657	62.3393959	36.02541339	29.66708797	37.52857555	45.79742189	45.87512943	44.86967107	Dnajc5	DnaJ heat shock protein family (Hsp40) member C5 [Source:MGI Symbol;Acc:MGI:892995]
ENSMUSG00000000827	71.62597325	70.36437362	75.52674043	75.45233437	79.2998371	65.88384067	74.44189959	81.84512151	75.06480313	68.11633939	68.96705736	65.19580631	Tpd52l2	tumor protein D52-like 2 [Source:MGI Symbol;Acc:MGI:1913564]
ENSMUSG00000000838	36.47929887	52.77387749	30.33450905	42.51544712	52.57129079	65.65648472	32.68087218	30.35524522	40.59393778	47.3883086	45.4294928	46.79692495	Fmr1	fragile X mental retardation 1 [Source:MGI Symbol;Acc:MGI:95564]
ENSMUSG00000000861	0.226957584	0.230605875	0.123012799	0	0.126241038	0.162700692	2.002387677	1.639964355	2.000593669	1.050865976	0.688999777	0.875530545	Bcl11a	B cell CLL/lymphoma 11A (zinc finger protein) [Source:MGI Symbol;Acc:MGI:106190]
ENSMUSG00000000869	5.463934962	5.855057402	3.962371711	6.727655016	4.54475158	4.450792926	4.742574577	4.886561024	6.851442148	6.923761146	4.674512849	6.052842014	Il4	interleukin 4 [Source:MGI Symbol;Acc:MGI:96556]
ENSMUSG00000000876	10.14701653	10.61986633	11.38783542	11.89022388	9.446514282	9.176725778	9.930526455	11.1972376	10.3290946	6.939322892	6.329429935	7.034667193	Pxmp4	peroxisomal membrane protein 4 [Source:MGI Symbol;Acc:MGI:1891701]
ENSMUSG00000000881	12.43238597	13.60070495	11.76084345	10.59235055	10.7502011	11.28767194	10.83296665	10.81349156	12.69399573	9.55248862	9.458718344	10.73142131	Dlg3	discs large MAGUK scaffold protein 3 [Source:MGI Symbol;Acc:MGI:1888986]
ENSMUSG00000000884	4.437504079	3.277457292	4.04177829	4.112447232	3.729847033	2.657353025	8.060015612	10.45940075	7.121115136	5.748558277	6.852588552	5.668404751	Gnb1l	guanine nucleotide binding protein (G protein), beta polypeptide 1-like [Source:MGI Symbol;Acc:MGI:1338057]
ENSMUSG00000000889	0	0	0.255560552	0	0	0.09506605	0	0	0	0	0	0	Dbh	dopamine beta hydroxylase [Source:MGI Symbol;Acc:MGI:94864]
ENSMUSG00000000901	41.27059232	40.71617579	40.98454475	45.261209	39.0797998	40.44259371	48.25932921	51.17476423	50.23807215	30.87614015	31.02798962	28.57312357	Mmp11	matrix metallopeptidase 11 [Source:MGI Symbol;Acc:MGI:97008]
ENSMUSG00000000902	35.70379104	29.29473271	36.93999759	32.79711548	27.26303168	25.14342805	52.66647169	55.54375727	49.3735723	33.87528643	31.41495414	33.19891435	Smarcb1	SWI/SNF related, matrix associated, actin dependent regulator of chromatin, subfamily b, member 1 [Source:MGI Symbol;Acc:MGI:13
ENSMUSG00000000903	0.063557589	0	0	0	0.26710971	0.242053328	3.971993774	3.39999376	3.135310237	3.922702629	3.174316881	3.041620167	Vpreb3	pre-B lymphocyte gene 3 [Source:MGI Symbol;Acc:MGI:98938]
ENSMUSG00000000915	9.247831886	8.974529616	8.372175101	11.1907333	9.629833118	11.2451678	18.57044604	21.12262989	19.82024336	16.98576067	18.22823127	17.77752125	Hip1r	huntingtin interacting protein 1 related [Source:MGI Symbol;Acc:MGI:1352504]
ENSMUSG00000000916	30.60668908	24.3487665	27.78680397	30.59658673	30.39799143	27.25730766	29.72371062	32.48051019	28.8861301	25.39160858	26.44938497	23.81149705	Nsun5	NOL1/NOP2/Sun domain family, member 5 [Source:MGI Symbol;Acc:MGI:2140844]
ENSMUSG00000000934	21.45677042	17.16363453	19.96669221	19.59403905	21.29581514	16.78099899	15.05123401	13.39886397	15.29927516	10.04182916	12.27916034	13.17503742	Top1mt	DNA topoisomerase 1, mitochondrial [Source:MGI Symbol;Acc:MGI:1920210]
ENSMUSG00000000938	4.967765025	6.747417758	5.790171429	4.184139786	4.068480988	3.958496276	0.25018555	0.227135956	0.165982274	1.59511998	0.975474406	0.993397162	Hoxa10	homeobox A10 [Source:MGI Symbol;Acc:MGI:96171]
ENSMUSG00000000942	8.719072502	6.745448488	7.37889009	6.851785427	6.409711384	4.934607875	0.265096233	0.133707176	0.226124444	0	0.247167899	0.047845552	Hoxa4	homeobox A4 [Source:MGI Symbol;Acc:MGI:96176]
ENSMUSG00000000948	0	0.032989025	0.058658123	0	0.090296242	0.458225322	0.844001454	0.312173319	2.02645507	28.41754641	30.21303677	41.3064154	Gm38393	predicted gene, 38393 [Source:MGI Symbol;Acc:MGI:5613898]
ENSMUSG00000000957	531.7957475	593.2682145	524.1287092	506.584194	537.1512304	539.7788878	28.86524598	28.60734708	31.16500876	96.84331257	98.14328344	94.41922034	Mmp14	matrix metallopeptidase 14 (membrane-inserted) [Source:MGI Symbol;Acc:MGI:101900]
ENSMUSG00000000958	6.245452819	7.236082424	4.942638936	4.433687668	4.267214377	5.953606598	97.8672284	91.17300639	104.2062518	68.81908858	70.80450896	70.32346003	Slc7a7	solute carrier family 7 (cationic amino acid transporter, y+ system), member 7 [Source:MGI Symbol;Acc:MGI:1337120]
ENSMUSG00000000959	53.11996664	50.49583945	53.44153806	52.42344814	46.76246222	44.91718336	93.20517688	96.7466719	91.17377505	57.8936637	61.45508199	59.22398413	Oxa1l	oxidase assembly 1-like [Source:MGI Symbol;Acc:MGI:1916339]
ENSMUSG00000000976	18.40260083	20.35618965	18.11731852	22.16101439	24.12848579	26.65251989	14.74216893	13.1621961	18.15893206	23.54698564	23.70841979	25.7490155	Heatr6	HEAT repeat containing 6 [Source:MGI Symbol;Acc:MGI:1919790]
ENSMUSG00000000982	79.4285663	63.06890043	77.63777776	2.149207038	1.475467309	1.925366748	2.808396642	5.342142793	3.398426696	37.53330402	31.32807646	35.34512053	Ccl3	chemokine (C-C motif) ligand 3 [Source:MGI Symbol;Acc:MGI:98260]
ENSMUSG00000000983	8.121054019	6.108844392	12.94076756	16.56254565	13.28037358	13.26923819	0	0	0	0	0.109031584	0.116081902	Wfdc18	WAP four-disulfide core domain 18 [Source:MGI Symbol;Acc:MGI:107506]
ENSMUSG00000001014	0	0	0	0	0	0	0	0	0	0	0	0	Icam4	intercellular adhesion molecule 4, Landsteiner-Wiener blood group [Source:MGI Symbol;Acc:MGI:1925619]
ENSMUSG00000001016	42.23956114	39.38425325	40.07925241	41.98406255	43.0800774	37.7762002	126.209357	125.9976123	124.2555687	93.11112578	92.15124752	93.70717262	Ilf2	interleukin enhancer binding factor 2 [Source:MGI Symbol;Acc:MGI:1915031]
ENSMUSG00000001017	65.95883263	72.40365594	68.62356869	78.23173936	80.0348142	76.28628694	100.5603859	101.0259608	93.04446953	89.11718786	88.4507432	89.93118478	Chtop	chromatin target of PRMT1 [Source:MGI Symbol;Acc:MGI:1913761]
ENSMUSG00000001018	40.08095377	47.96342357	37.59388573	45.65465854	47.03170123	47.27264308	25.13443077	20.52153487	26.17809456	22.49648739	25.20022161	23.28987905	Snapin	SNAP-associated protein [Source:MGI Symbol;Acc:MGI:1333745]
ENSMUSG00000001020	0.309557466	0.118823778	0.316922663	1.444369706	0.433652934	0.62875819	0	0	0	0.092297281	0.085891788	0.457229073	S100a4	S100 calcium binding protein A4 [Source:MGI Symbol;Acc:MGI:1330282]
ENSMUSG00000001021	0.967838527	0.232190795	0.206430413	0.150528502	0.338956464	0.798617185	0	0	0.03753435	0.108213589	0	0.07147686	S100a3	S100 calcium binding protein A3 [Source:MGI Symbol;Acc:MGI:1338849]
ENSMUSG00000001023	0.302307877	0.812287141	0.412667449	0.564217487	0.105874282	0.23026244	0	0.199652682	0	0.180271505	0	0	S100a5	S100 calcium binding protein A5 [Source:MGI Symbol;Acc:MGI:1338915]
ENSMUSG00000001025	2176.245661	1705.136613	3368.665682	2367.18275	2148.683442	1401.358254	202.8224679	202.6752309	118.4182166	537.7014853	479.6615759	469.1203456	S100a6	S100 calcium binding protein A6 (calcyclin) [Source:MGI Symbol;Acc:MGI:1339467]
ENSMUSG00000001027	0.073323183	0.112580552	0	0.045615993	0.012839625	0.027924471	0.043640845	0.096849413	0	0.251412447	0.203447177	0.281583505	Scn4a	sodium channel, voltage-gated, type IV, alpha [Source:MGI Symbol;Acc:MGI:98250]
ENSMUSG00000001029	0	0.070283001	0	0.170865863	0	0	0.054489155	0.423235367	0.340843843	0.928078622	0.660453023	0.919516851	Icam2	intercellular adhesion molecule 2 [Source:MGI Symbol;Acc:MGI:96394]
ENSMUSG00000001034	28.25102736	28.21240363	27.44776036	30.71815768	28.67279308	26.22270125	25.99793154	27.96400641	27.95562783	38.32427486	39.39665889	39.67362811	Mapk7	mitogen-activated protein kinase 7 [Source:MGI Symbol;Acc:MGI:1346347]
ENSMUSG00000001036	29.74855349	33.09060939	28.01753838	36.37267957	37.9560685	39.58884533	26.75978065	27.11033644	31.23275156	38.58863237	39.84510297	39.77857058	Epn2	epsin 2 [Source:MGI Symbol;Acc:MGI:1333766]
ENSMUSG00000001039	13.36288442	10.77163383	15.45933004	15.27577961	12.98686164	10.28004995	48.25716917	46.33244315	43.53168644	42.91049667	42.76890876	36.47495231	B9d1	B9 protein domain 1 [Source:MGI Symbol;Acc:MGI:1351471]
ENSMUSG00000001052	54.26562766	71.5876323	50.27177053	74.46164324	79.66849956	88.63610409	37.48750207	34.923884	40.57649953	60.4489128	63.01359585	59.91915089	Sec24b	Sec24 related gene family, member B (S. cerevisiae) [Source:MGI Symbol;Acc:MGI:2139764]
ENSMUSG00000001053	8.513658019	5.829354779	7.471046252	11.31904761	8.806815263	8.312433042	47.04278921	49.04089124	52.5931944	44.92712958	46.32388861	52.37812271	N4bp3	NEDD4 binding protein 3 [Source:MGI Symbol;Acc:MGI:2442218]
ENSMUSG00000001054	29.0015799	27.80884709	30.23498053	30.21239654	26.84617319	23.8514018	64.50987829	62.90598669	63.37293052	56.05339948	66.2375521	58.86242204	Rmnd5b	required for meiotic nuclear division 5 homolog B [Source:MGI Symbol;Acc:MGI:1913339]
ENSMUSG00000001056	99.09451533	71.72478065	113.8095072	86.41166001	68.1512979	54.950874	330.0222581	366.5183971	260.7911558	324.5104231	323.7324013	317.4748039	Nhp2	NHP2 ribonucleoprotein [Source:MGI Symbol;Acc:MGI:1098547]
ENSMUSG00000001062	19.17206472	18.49070027	18.61467138	22.28495487	23.01007	21.11367884	10.56300701	10.35745907	11.66414682	9.222425163	9.346154376	9.00895223	Vps9d1	VPS9 domain containing 1 [Source:MGI Symbol;Acc:MGI:1914143]
ENSMUSG00000001065	8.550532838	10.1351282	6.007125027	10.85735065	11.22304407	12.49079807	6.156226614	5.464852269	6.418133257	8.253829618	8.766368124	7.13325315	Zfp276	zinc finger protein (C2H2 type) 276 [Source:MGI Symbol;Acc:MGI:1888495]
ENSMUSG00000001076	0.116766588	0.067231364	0.388520524	0.435858647	0	0.155643246	3.518321099	3.123196439	2.852890712	0.28722363	0.388785623	0.388055339	C1ql4	complement component 1, q subcomponent-like 4 [Source:MGI Symbol;Acc:MGI:3579909]
ENSMUSG00000001082	100.7349178	86.07491791	103.6462989	104.5585305	99.87024532	82.93052418	88.67727996	85.65056781	82.76512739	59.93511586	59.70873985	57.86757382	Mfsd10	major facilitator superfamily domain containing 10 [Source:MGI Symbol;Acc:MGI:1915544]
ENSMUSG00000001089	11.58587214	13.63537302	9.568679958	10.10820609	12.67370532	16.34900901	27.86338939	24.50354708	35.00302035	43.04979696	41.57563103	40.22574383	Luzp1	leucine zipper protein 1 [Source:MGI Symbol;Acc:MGI:107629]
ENSMUSG00000001095	0	0	0	0.033294758	0	0	0.143339008	0.159051671	0.166041258	0.19148242	0.17819341	0.173906282	Slc13a2	solute carrier family 13 (sodium-dependent dicarboxylate transporter), member 2 [Source:MGI Symbol;Acc:MGI:1276558]
ENSMUSG00000001098	35.68530278	62.5303019	25.35642246	45.29288256	63.27811026	80.24238136	12.13403325	9.507861239	15.34817783	39.8419592	38.84629683	38.58958618	Kctd10	potassium channel tetramerisation domain containing 10 [Source:MGI Symbol;Acc:MGI:2141207]
ENSMUSG00000001100	119.926955	109.8830581	118.6411992	117.4190867	114.0393917	99.0447872	118.8908427	112.5927233	117.6515822	108.5633721	109.3736802	114.8374436	Poldip2	polymerase (DNA-directed), delta interacting protein 2 [Source:MGI Symbol;Acc:MGI:1915061]
ENSMUSG00000001103	0.06489968	0	0	0	0	0	0.115881913	0	0	0.174153694	0	0	Sebox	SEBOX homeobox [Source:MGI Symbol;Acc:MGI:108012]
ENSMUSG00000001105	65.79990366	52.39028168	77.17643392	43.51804709	42.26753469	34.72528537	50.52802746	55.64388498	45.0387371	48.20543528	52.4024187	48.2231992	Ift20	intraflagellar transport 20 [Source:MGI Symbol;Acc:MGI:1915585]
ENSMUSG00000001119	155.9675669	168.129216	139.5893626	111.8001928	116.6097571	118.5498797	5.411404036	4.427117489	6.167537461	46.48268123	49.51365914	49.28598266	Col6a1	collagen, type VI, alpha 1 [Source:MGI Symbol;Acc:MGI:88459]
ENSMUSG00000001120	10.22916505	9.043546563	8.580729716	8.77587514	9.741976577	7.741103034	18.73607913	19.25354624	18.39681117	16.70566639	17.00202908	17.16565572	Pcbp3	poly(rC) binding protein 3 [Source:MGI Symbol;Acc:MGI:1890470]
ENSMUSG00000001123	160.8936876	158.9126626	154.8449119	113.5779121	109.4768457	100.7448368	13.98038556	14.3355816	15.19330119	43.14654232	41.98517572	39.55785482	Lgals9	lectin, galactose binding, soluble 9 [Source:MGI Symbol;Acc:MGI:109496]
ENSMUSG00000001127	67.24031761	68.7082536	61.00379552	72.12249138	72.07082746	72.6129784	46.83812349	44.12657242	48.08866619	47.92571971	47.82957677	46.61211759	Araf	Araf proto-oncogene, serine/threonine kinase [Source:MGI Symbol;Acc:MGI:88065]
ENSMUSG00000001128	44.63308306	38.3169954	46.12733166	8.511433967	9.538555883	9.295085343	8.444482151	9.370156904	8.72513409	28.51659923	30.54451893	28.0664166	Cfp	complement factor properdin [Source:MGI Symbol;Acc:MGI:97545]

ENSMUSG00000001131	664.9355712	514.4371622	825.2352184	610.5387784	518.1568795	398.8269807	75.3074412	78.29642888	57.36644198	172.0295467	156.751011	161.9537351	Timp1	tissue inhibitor of metalloproteinase 1 [Source:MGI Symbol;Acc:MGI:98752]
ENSMUSG00000001134	9.320503064	7.585756464	11.6229064	10.65963662	9.866310519	7.526281612	10.57346961	11.17713596	7.43583595	7.145974212	6.241703056	7.204262124	Uxt	ubiquitously expressed prefoldin like chaperone [Source:MGI Symbol;Acc:MGI:1277988]
ENSMUSG00000001138	9.228719418	10.62734481	8.561451645	11.5190581	10.04629297	11.71135155	12.37365799	12.31083329	14.35753554	10.71345251	11.31497112	11.08703501	Cnnm3	cyclin M3 [Source:MGI Symbol;Acc:MGI:2151055]
ENSMUSG00000001143	15.21929424	15.42167731	14.3494901	19.23457638	19.26796789	20.16340576	9.728049529	8.653201605	11.44854047	10.2049916	11.44433567	10.03185685	Lman2l	lectin, mannose-binding 2-like [Source:MGI Symbol;Acc:MGI:2443010]
ENSMUSG00000001150	41.89541376	47.95114415	46.1983111	51.67876838	55.32736261	58.01799967	88.23530339	82.8383821	100.0305997	115.4638991	118.1320251	117.5869161	Mcm3ap	minichromosome maintenance complex component 3 associated protein [Source:MGI Symbol;Acc:MGI:1930089]
ENSMUSG00000001151	6.933344183	8.047367706	5.647147497	8.582344471	8.277416817	10.63732897	20.28649597	20.42034992	26.43693991	27.28360435	28.31691688	27.7914479	Pcnt	pericentrin (kendrin) [Source:MGI Symbol;Acc:MGI:102722]
ENSMUSG00000001155	0.07292964	0.111976306	0.099553108	0.022685581	0.025541423	0.018516396	0	0	0	0.130467683	0.14164866	0.086176056	Ftcd	formiminotransferase cyclodeaminase [Source:MGI Symbol;Acc:MGI:1339962]
ENSMUSG00000001156	3.877160538	4.925033137	3.703417225	3.841902663	4.388545534	4.231852177	3.800447709	2.435197059	3.813319119	5.648953727	5.805893889	4.906091314	Mxd1	MAX dimerization protein 1 [Source:MGI Symbol;Acc:MGI:96908]
ENSMUSG00000001157	16.98005918	17.60872571	14.09198326	18.52241074	21.75346501	21.39121562	24.78377682	23.85672444	26.91475237	26.11387826	23.31948183	27.39008638	Gmcl1	germ cell-less, spermatogenesis associated 1 [Source:MGI Symbol;Acc:MGI:1345156]
ENSMUSG00000001158	78.35537861	60.47483035	93.93242455	81.50478964	68.01770335	55.30350305	111.2585851	119.3634308	85.18885668	60.10312777	53.28398985	55.83923397	Snrnp27	small nuclear ribonucleoprotein 27 (U4/U6.U5) [Source:MGI Symbol;Acc:MGI:1913868]
ENSMUSG00000001166	8.472505342	8.009723936	10.37860409	10.32320711	8.862851748	7.862383283	5.284932632	6.15747413	5.062102557	5.659021711	5.327875716	4.459221872	Oas1c	2'-5' oligoadenylate synthetase 1C [Source:MGI Symbol;Acc:MGI:2149633]
ENSMUSG00000001168	0.424730707	0.225738112	0.568631931	0.213420156	0.205960448	0.298624334	0.204179179	0.356024544	0.091228136	0.146119842	0.217566442	0.173726217	Oas1h	2'-5' oligoadenylate synthetase 1H [Source:MGI Symbol;Acc:MGI:2180853]
ENSMUSG00000001173	14.6655026	17.25965505	11.11629711	16.84729722	20.40900461	24.9215275	10.42832934	9.3658593	12.6439391	21.45916985	19.74158491	19.71701371	Ocrl	OCRL, inositol polyphosphate-5-phosphatase [Source:MGI Symbol;Acc:MGI:109589]
ENSMUSG00000001175	322.7136587	321.7712912	313.3473967	347.7416443	336.5551672	323.0541462	265.3920486	275.8860108	271.1299794	178.3820521	174.2270628	177.2816583	Calm1	calmodulin 1 [Source:MGI Symbol;Acc:MGI:88251]
ENSMUSG00000001211	60.72707926	60.96874267	62.63541386	69.26273757	68.92230097	66.31977422	19.97726379	19.32684978	22.61292273	21.23008566	22.2330204	22.48618678	Agpat3	1-acylglycerol-3-phosphate O-acyltransferase 3 [Source:MGI Symbol;Acc:MGI:1336186]
ENSMUSG00000001225	0.069965021	0	0	0.065290208	0.024503154	0.124345878	0	0	0	0	0.038825881	0	Slc26a3	solute carrier family 26, member 3 [Source:MGI Symbol;Acc:MGI:107181]
ENSMUSG00000001227	3.446274707	3.235241154	3.617755893	3.868530076	4.250578974	4.232280668	1.449196468	1.410141218	1.278411685	2.47949174	2.473713396	2.589407921	Sema6b	sema domain, transmembrane domain (TM), and cytoplasmic domain, (semaphorin) 6B [Source:MGI Symbol;Acc:MGI:1202889]
ENSMUSG00000001228	54.73055661	56.16637641	49.75910781	74.07194358	72.90693753	80.7281999	238.2777617	213.2994393	256.0196204	137.9351341	140.5184693	140.0123865	Uhrf1	ubiquitin-like, containing PHD and RING finger domains, 1 [Source:MGI Symbol;Acc:MGI:1338889]
ENSMUSG00000001229	17.55123005	18.46921507	15.32759488	17.73211614	19.73577683	19.9343068	13.56961014	12.72719748	14.73515616	13.53983117	14.14391423	14.45249024	Dpp9	dipeptidylpeptidase 9 [Source:MGI Symbol;Acc:MGI:2443967]
ENSMUSG00000001240	2.967481919	2.107278251	1.974756043	1.15383557	1.299089608	0.97945349	3.753175398	4.850527497	2.347708882	2.964015801	3.211168146	3.418812145	Ramp2	receptor (calcitonin) activity modifying protein 2 [Source:MGI Symbol;Acc:MGI:1859650]
ENSMUSG00000001247	0.650538693	1.070183923	0.451939932	0.693796593	0.854368867	1.04409677	5.662664774	5.731013001	6.855065352	7.980226289	7.445732753	8.215458408	Lsr	lipolysis stimulated lipoprotein receptor [Source:MGI Symbol;Acc:MGI:1927471]
ENSMUSG00000001248	82.62010902	73.76333552	84.91833136	75.23094998	66.93105748	61.42552295	36.55451208	38.79220228	39.39425974	34.39710705	34.80319032	33.48021066	Gramd1a	GRAM domain containing 1A [Source:MGI Symbol;Acc:MGI:105490]
ENSMUSG00000001249	3.263955598	2.566857424	4.441901269	3.231593498	2.592891524	2.637687137	8.884101312	8.793306232	7.743082949	7.512452271	8.813403217	6.843463006	Hpn	hepsin [Source:MGI Symbol;Acc:MGI:1196620]
ENSMUSG00000001260	0	0	0	0	0	0	0	0	0	0.09512009	0.188102219	0.070681936	Gabrg1	gamma-aminobutyric acid (GABA) A receptor, subunit gamma 1 [Source:MGI Symbol;Acc:MGI:103156]
ENSMUSG00000001270	25.41527024	19.24140723	26.842733	3.687245435	3.51815707	3.315659675	115.0054036	133.4835884	111.3584579	114.4455405	115.4780662	110.86734	Ckb	creatine kinase, brain [Source:MGI Symbol;Acc:MGI:88407]
ENSMUSG00000001280	25.62048466	33.96063898	21.53339408	35.49024671	41.7009619	56.98405397	75.95207226	67.05303505	90.84924139	98.29910878	99.98380518	97.59192219	Sp1	trans-acting transcription factor 1 [Source:MGI Symbol;Acc:MGI:98372]
ENSMUSG00000001281	17.40922726	14.29532002	19.84882997	30.23049193	24.42288463	23.58222634	61.15468148	67.12346132	60.67216288	34.55069165	36.47557664	40.49977464	Itgb7	integrin beta 7 [Source:MGI Symbol;Acc:MGI:96616]
ENSMUSG00000001285	45.50708736	37.10505202	59.88669958	52.68987472	40.62498653	35.45484491	65.8982923	60.40298695	57.07465488	46.42500091	45.67889534	44.15157743	Myg1	melanocyte proliferating gene 1 [Source:MGI Symbol;Acc:MGI:1929864]
ENSMUSG00000001288	96.71788006	96.21824546	94.29072873	104.5858027	109.6645433	102.2209391	173.760212	175.9174072	181.9048224	110.2311881	114.1783043	112.5909824	Rarg	retinoic acid receptor, gamma [Source:MGI Symbol;Acc:MGI:97858]
ENSMUSG00000001289	284.6057909	211.6814866	364.2584228	230.9998074	205.2865207	144.4464349	194.9601943	218.2528796	157.7896454	130.4350112	120.3662276	130.5685935	Pfdn5	prefoldin 5 [Source:MGI Symbol;Acc:MGI:1928753]
ENSMUSG00000001300	63.44905195	76.62722841	51.4422791	82.20236312	96.07998812	118.0174156	4.100932134	3.730148619	5.133387977	15.51257904	15.91861132	15.99255807	Efnb2	ephrin B2 [Source:MGI Symbol;Acc:MGI:105097]
ENSMUSG00000001305	19.5938515	15.13202244	17.82449453	15.3372822	13.64238333	11.38997455	45.37722001	50.23553687	41.5734509	35.43613397	36.74191485	35.83490907	Rrp15	ribosomal RNA processing 15 homolog (S. cerevisiae) [Source:MGI Symbol;Acc:MGI:1914473]
ENSMUSG00000001313	22.73587291	19.42122182	23.83268426	22.00841254	21.24933575	16.43870436	40.14170477	40.60885175	32.87946903	14.5088706	18.55811293	16.93125027	Rnd2	Rho family GTPase 2 [Source:MGI Symbol;Acc:MGI:1338755]
ENSMUSG00000001323	7.891687724	8.294314842	6.647379821	7.364393315	8.094065731	11.05188954	1.52839303	1.075470199	1.92375837	3.380068857	3.579949708	2.7383163	Srr	serine racemase [Source:MGI Symbol;Acc:MGI:1351636]
ENSMUSG00000001333	1.200703457	2.244008256	0.987447511	1.818478639	2.79191353	3.073501478	0.47447441	0.253493147	0.934358064	0.739476024	0.852002527	1.133869536	Sync	syncoilin [Source:MGI Symbol;Acc:MGI:1916078]
ENSMUSG00000001334	1.370433199	1.829189502	1.387095826	0.319716436	0.343602854	0.296543799	2.029883711	1.527310504	1.594428761	0.933294878	0.466669387	0.70386469	Fndc5	fibronectin type III domain containing 5 [Source:MGI Symbol;Acc:MGI:1917614]
ENSMUSG00000001348	8.190880444	9.108366843	9.105571324	3.280512895	2.326898739	2.757944547	9.760636941	10.58681185	9.03069103	11.44576778	10.53437514	8.629750489	Acp5	acid phosphatase 5, tartrate resistant [Source:MGI Symbol;Acc:MGI:87883]
ENSMUSG00000001349	3.213420767	2.797877931	3.650960627	0.987380603	0.535253314	0.92531388	2.868868883	2.36810265	2.662648507	10.65604898	9.231489764	9.620049726	Cnn1	calponin 1 [Source:MGI Symbol;Acc:MGI:104979]
ENSMUSG00000001366	37.55593258	38.86991129	32.54734214	34.65673961	35.90330525	32.9967567	21.14681773	20.11277862	21.22993857	21.59810152	22.42901426	23.064854	Fbxo9	f-box protein 9 [Source:MGI Symbol;Acc:MGI:1918788]
ENSMUSG00000001376	15.84628248	19.86720316	13.67744237	18.05226106	20.99150693	22.67664401	23.94612665	24.22656788	29.38640413	32.52072089	32.59064437	32.73745584	Vps50	VPS50 EARP/GARPII complex subunit [Source:MGI Symbol;Acc:MGI:1920538]
ENSMUSG00000001380	108.7107452	102.6157871	100.4241975	107.6997944	98.79741605	94.1211546	123.179834	119.2530337	118.9171311	107.4319824	106.6538527	110.3996065	Hars	histidyl-tRNA synthetase [Source:MGI Symbol;Acc:MGI:108087]
ENSMUSG00000001383	70.25990325	59.56449393	78.79193061	71.33961171	61.05669503	49.01985724	103.4069829	112.5390174	87.15799443	73.20110238	65.67613946	63.37794895	Zmat2	zinc finger, matrin type 2 [Source:MGI Symbol;Acc:MGI:1913742]
ENSMUSG00000001403	57.58023735	45.62697639	56.66252068	71.40656476	67.13964948	53.73777038	154.2433026	155.4399183	133.235081	93.59961423	89.37264621	86.55372413	Ube2c	ubiquitin-conjugating enzyme E2C [Source:MGI Symbol;Acc:MGI:1915862]
ENSMUSG00000001415	57.58048336	60.18201585	56.47253741	63.77153042	65.55206161	64.11417787	116.5250573	108.9327219	125.3760938	117.4952085	120.2850068	114.5840824	Smg5	Smg-5 homolog, nonsense mediated mRNA decay factor (C. elegans) [Source:MGI Symbol;Acc:MGI:2447364]
ENSMUSG00000001416	184.9659489	163.7442774	199.7614285	179.7791615	165.2237107	149.029776	404.5492607	405.8877015	368.6722969	357.9564805	341.7366399	350.6222634	Cct3	chaperonin containing Tcp1, subunit 3 (gamma) [Source:MGI Symbol;Acc:MGI:104708]
ENSMUSG00000001418	137.7387941	121.4054553	152.7740706	113.7434926	103.6788555	85.88567155	47.08536541	53.37406268	47.53681577	53.70799243	52.3816297	50.49209336	Glmp	glycosylated lysosomal membrane protein [Source:MGI Symbol;Acc:MGI:1913318]
ENSMUSG00000001419	39.57316987	49.87484073	35.64227516	43.58071913	47.21757694	53.87850362	26.50236902	24.30534447	33.25293187	34.75579994	35.3646422	34.83960837	Mef2d	myocyte enhancer factor 2D [Source:MGI Symbol;Acc:MGI:99533]
ENSMUSG00000001420	0.847388163	0.596328963	0.722958154	0.944529293	0.840854936	1.434311625	4.308011534	3.474426317	4.272201612	3.200312302	2.566745453	3.233369927	Tmem79	transmembrane protein 79 [Source:MGI Symbol;Acc:MGI:1919163]
ENSMUSG00000001424	127.131056	132.8217419	117.4726672	127.667468	122.1452587	117.962464	190.8359893	190.3642253	205.0281838	150.2863449	160.4018753	156.3309411	Snd1	staphylococcal nuclease and tudor domain containing 1 [Source:MGI Symbol;Acc:MGI:1929266]
ENSMUSG00000001435	28.56641888	30.37997321	28.26511659	23.21223912	21.62428767	22.34238461	81.06127313	72.13994415	84.27207151	104.2649065	104.760107	104.220127	Col18a1	collagen, type XVIII, alpha 1 [Source:MGI Symbol;Acc:MGI:88451]
ENSMUSG00000001436	13.85022412	11.63542939	12.45566819	13.85487414	11.15764407	11.36096243	19.39152378	19.71275225	18.87300271	16.5694999	18.25173048	17.45219999	Slc19a1	solute carrier family 19 (folate transporter), member 1 [Source:MGI Symbol;Acc:MGI:103182]
ENSMUSG00000001440	89.4794958	101.6181538	78.02612581	88.55496403	96.79288168	111.4370452	301.5069912	269.5442596	335.7954689	492.6270333	506.2235882	491.2406172	Kpnb1	karyopherin (importin) beta 1 [Source:MGI Symbol;Acc:MGI:107532]
ENSMUSG00000001441	163.0816351	175.6301964	150.6853113	182.0436141	189.6928431	195.9333008	169.5490616	161.852754	180.2677146	214.7694111	208.1595792	206.2603203	Npepps	aminopeptidase puromycin sensitive [Source:MGI Symbol;Acc:MGI:1101358]
ENSMUSG00000001444	0.034588817	0.079661601	0.017705888	0.112972004	0.018170546	0	0.432321509	0.205590905	0.338035443	0.727063673	0.734188364	0.705029588	Tbx21	T-box 21 [Source:MGI Symbol;Acc:MGI:1888984]
ENSMUSG00000001445	44.43235366	42.07263113	41.89634888	43.39588755	43.73333243	41.75613396	58.37428478	62.026519	61.44572106	70.50894981	72.39332581	69.32972639	Mrpl10	mitochondrial ribosomal protein L10 [Source:MGI Symbol;Acc:MGI:1333801]
ENSMUSG00000001467	33.7728657	35.78390508	30.38803233	54.82858572	56.42656592	59.8904895	69.52492807	66.62624071	74.50816524	26.54968955	29.4408437	29.93034461	Cyp51	cytochrome P450, family 51 [Source:MGI Symbol;Acc:MGI:106040]
ENSMUSG00000001472	195.5208737	190.7490819	187.9824307	192.0704499	184.7097105	187.66594	160.9103269	160.2978705	171.7698297	177.5032436	180.0416213	182.3415287	Tcf25	transcription factor 25 (basic helix-loop-helix) [Source:MGI Symbol;Acc:MGI:1914105]
ENSMUSG00000001473	272.1592144	257.84306	274.8068462	310.6405386	287.0304916	260.1545294	77.29011089	80.07912062	87.59814415	124.5164994	133.7163351	123.6248775	Tubb6	tubulin, beta 6 class V [Source:MGI Symbol;Acc:MGI:1915201]
ENSMUSG00000001482	30.3492469	32.0596239	30.24276738	29.07665636	31.00808795	28.76716545	56.26150414	54.59849959	58.25331131	52.91793746	55.89229032	54.6053632	Def8	differentially expressed in FDCP 8 [Source:MGI Symbol;Acc:MGI:1346331]
ENSMUSG00000001493	0.270376494	0.177915675	0.177948946	0.072088829	0.081163947	0.058840252	0.50001381	0.650484211	0.790916816	1.330149646	1.18960897	1.044033746	Meox1	mesenchyme homeobox 1 [Source:MGI Symbol;Acc:MGI:103220]
ENSMUSG00000001494	0	0.191866469	0	0.077741487	0.021882051	0.095181086	0.037187661	0	0.019384875	0.093146095	0.017336339	0.055372078	Sost	sclerostin [Source:MGI Symbol;Acc:MGI:1921749]
ENSMUSG00000001496	0	0	0	0	0	0	0	0.050624522	0	0	0	0	Nkx2-1	NK2 homeobox 1 [Source:MGI Symbol;Acc:MGI:108067]
ENSMUSG00000001497	2.182167637	2.623944649	1.839110473	2.902661048	2.875395984	3.498711645	0	0.047773435	0.011221393	0.032351891	0.010035549	0.042737915	Pax9	paired box 9 [Source:MGI Symbol;Acc:MGI:97493]
ENSMUSG00000001504	49.59934065	54.62651033	50.77944111	47.98637302	47.74711061	47.46582299	5.449989149	5.459468178	6.313166742	19.41461949	19.83160987	20.13717922	Irx2	Iroquois homeobox 2 [Source:MGI Symbol;Acc:MGI:1197526]
ENSMUSG00000001506	1991.825183	2185.019572	1992.739774	923.5074447	907.7200809	883.9600535	41.69338176	38.15025336	45.80579542	384.7716122	427.8115621	418.7125683	Col1a1	collagen, type I, alpha 1 [Source:MGI Symbol;Acc:MGI:88467]
ENSMUSG00000001507	15.67409741	17.59327185	14.97614252	20.27639709	20.26464951	21.74070393	28.02940181	24.79050222	29.92991839	15.74958771	15.2897822	14.57898898	Itga3	integrin alpha 3 [Source:MGI Symbol;Acc:MGI:96602]
ENSMUSG00000001508	0.771119854	0.577189698	0.394733425	0.107939457	0.243055475	0.14683701	0.275375298	0.725708877	0.394749701	0.379361676	0.513503591	0.61504685	Sgca	sarcoglycan, alpha (dystrophin-associated glycoprotein) [Source:MGI Symbol;Acc:MGI:894698]

ENSMUSG00000001510	1.452438408	1.596532149	1.486995787	1.494011258	1.023126499	0.678863488	0.383117072	0.245258042	0.322605363	0.797219096	0.604504227	0.628966207	Dlx3	distal-less homeobox 3 [Source:MGI Symbol;Acc:MGI:94903]
ENSMUSG00000001517	34.95343649	42.67501848	33.68875166	54.17490006	60.02525379	67.32732792	39.00704016	32.96932188	42.00866095	37.53350608	37.88138707	36.36938564	Foxm1	forkhead box M1 [Source:MGI Symbol;Acc:MGI:1347487]
ENSMUSG00000001518	10.20137539	10.27459878	10.03880012	13.07190361	13.11777107	11.34217699	13.62055335	12.60976952	12.98125364	11.65806766	15.38630107	12.03628839	Itfg2	integrin alpha FG-GAP repeat containing 2 [Source:MGI Symbol;Acc:MGI:1915450]
ENSMUSG00000001520	0.029901659	0.206599787	0.122452398	0.251133635	0.62832965	1.161553198	0.293650726	0.088865562	0.500962285	0.320955939	0.29868138	0.423993381	Nrip2	nuclear receptor interacting protein 2 [Source:MGI Symbol;Acc:MGI:1891884]
ENSMUSG00000001521	48.38482335	51.67379754	48.92166215	49.96370253	47.23989051	49.34724698	42.70080886	39.76357487	43.54517766	39.91933702	39.40596321	39.1571356	Tulp3	tubby-like protein 3 [Source:MGI Symbol;Acc:MGI:1329045]
ENSMUSG00000001524	37.40320387	31.70714417	38.64675619	31.26629753	30.84741906	25.25704343	75.02350514	83.73632696	67.28441168	50.27269919	49.61787337	51.38321446	Gtf2h4	general transcription factor II H, polypeptide 4 [Source:MGI Symbol;Acc:MGI:1338799]
ENSMUSG00000001525	1679.663426	1481.705398	1724.74061	1751.7309	1586.936075	1396.481741	1628.277615	1661.003915	1606.897603	1276.955258	1315.139484	1266.341425	Tubb5	tubulin, beta 5 class I [Source:MGI Symbol;Acc:MGI:107812]
ENSMUSG00000001542	20.11058624	27.24341695	19.62232497	17.79041989	22.22876389	24.08482299	31.30605545	28.46779627	35.74968745	50.79224506	54.27764938	54.01030629	Ell2	elongation factor RNA polymerase II 2 [Source:MGI Symbol;Acc:MGI:2183438]
ENSMUSG00000001552	53.26586597	50.85535937	51.79203061	46.73794272	44.38093331	45.5141414	97.49948687	92.83447797	107.1886515	137.215517	143.9492287	132.6556099	Jup	junction plakoglobin [Source:MGI Symbol;Acc:MGI:96650]
ENSMUSG00000001555	184.1566198	155.3723054	193.8366938	115.1974487	100.3506157	94.15266067	25.29397485	26.29196466	26.8949969	60.89229529	57.57762943	55.18539869	Fkbp10	FK506 binding protein 10 [Source:MGI Symbol;Acc:MGI:104769]
ENSMUSG00000001558	0.204202993	0.503893379	0.447988985	0.735012815	0.704943269	0.2666361	1.067802723	0.982561913	0.678799178	0.652338413	1.238414	1.26678802	Klhl10	kelch-like 10 [Source:MGI Symbol;Acc:MGI:2181067]
ENSMUSG00000001566	0.020182218	0	0.020662406	0.037667428	0	0	0.180182242	0.19993362	0.093923901	0.090262585	0.083998301	0.089429889	Mnx1	motor neuron and pancreas homeobox 1 [Source:MGI Symbol;Acc:MGI:109160]
ENSMUSG00000001569	12.42750992	14.77846066	12.18101195	14.23286353	15.13435396	14.5550266	12.54491234	11.68166821	12.80751328	14.97675327	16.29575996	15.26098401	Nom1	nucleolar protein with MIF4G domain 1 [Source:MGI Symbol;Acc:MGI:1861749]
ENSMUSG00000001576	145.9593803	166.2985377	138.750192	157.9370128	163.4590961	163.8915241	75.0219503	73.07686178	82.50648732	77.98215409	78.37575105	73.72361573	Ergic1	endoplasmic reticulum-golgi intermediate compartment (ERGIC) 1 [Source:MGI Symbol;Acc:MGI:1914708]
ENSMUSG00000001583	0.400032426	0.086373357	0.563131612	0.139988883	0.157611801	0.171392322	3.325864763	2.699719581	4.09567151	7.290573525	7.304894418	7.821566156	Tnk1	tyrosine kinase, non-receptor, 1 [Source:MGI Symbol;Acc:MGI:1930958]
ENSMUSG00000001588	1.962344338	1.294642926	1.967178869	0.991914892	1.374504691	1.338986116	16.20541112	16.03784864	14.13611382	13.40222297	14.87123476	13.56843022	Acap1	ArfGAP with coiled-coil, ankyrin repeat and PH domains 1 [Source:MGI Symbol;Acc:MGI:2388270]
ENSMUSG00000001604	4.470492242	3.592876133	4.338479186	1.390596635	1.027461781	1.667076807	84.81201548	91.57176156	81.91871902	55.69092104	55.58593013	53.15495805	Tcea3	transcription elongation factor A (SII), 3 [Source:MGI Symbol;Acc:MGI:1196908]
ENSMUSG00000001622	1.500587978	1.245770466	1.179013996	2.898345115	2.089922259	3.136524278	0	0.034570842	0	0.093644687	0.261437047	0.09278079	Csn3	casein kappa [Source:MGI Symbol;Acc:MGI:107461]
ENSMUSG00000001627	124.9452653	121.9156467	103.6211865	163.6495695	157.9184041	148.0743767	54.1900261	56.46826216	58.86950022	71.48709905	65.32579608	71.05693322	Ifrd1	interferon-related developmental regulator 1 [Source:MGI Symbol;Acc:MGI:1316717]
ENSMUSG00000001630	18.07125131	18.42699648	15.7981743	17.66286898	17.93713129	18.64260212	16.06989542	15.62240853	17.89924765	18.33444169	18.81425424	18.14949195	Stk38l	serine/threonine kinase 38 like [Source:MGI Symbol;Acc:MGI:1922250]
ENSMUSG00000001632	9.89009434	11.74700857	9.256114828	11.23806342	14.14801736	15.75849241	10.8475519	10.1292662	12.98981652	16.0562639	16.16637181	16.19429638	Brpf1	bromodomain and PHD finger containing, 1 [Source:MGI Symbol;Acc:MGI:1926033]
ENSMUSG00000001642	170.8281954	140.3520025	164.634221	119.8811973	110.6567339	92.85128258	197.6356171	210.6037504	190.5068604	187.9386632	181.557052	183.2186858	Akr1b3	aldo-keto reductase family 1, member B3 (aldose reductase) [Source:MGI Symbol;Acc:MGI:1353494]
ENSMUSG00000001655	2.150550184	2.476469086	1.897415084	1.713171545	1.267522944	1.358370624	0.437064573	0.190525934	0.244102915	0.578648824	0.815012201	1.162116153	Hoxc13	homeobox C13 [Source:MGI Symbol;Acc:MGI:99560]
ENSMUSG00000001656	1.170988041	1.029727687	1.547605029	0.357626225	0.357908507	0.356768161	0	0.042182927	0	0.114264126	0.017722353	0.150946679	Hoxc11	homeobox C11 [Source:MGI Symbol;Acc:MGI:96193]
ENSMUSG00000001657	10.7045456	9.202744837	12.01425005	4.945589645	4.8611095	4.389091718	1.051432956	1.395861093	0.959143903	7.14830265	8.297751211	7.883782348	Hoxc8	homeobox C8 [Source:MGI Symbol;Acc:MGI:96198]
ENSMUSG00000001661	43.24255991	48.11972861	40.06343948	24.5315775	26.31528979	24.65389015	2.599214007	2.375171613	2.550393729	17.15682481	18.56775336	17.5297532	Hoxc6	homeobox C6 [Source:MGI Symbol;Acc:MGI:96197]
ENSMUSG00000001663	6.281170062	5.29667236	6.126807211	1.384602684	1.351053192	1.469180235	5.47522058	8.378183858	6.352624035	4.069991846	2.964155212	3.857121394	Gstt1	glutathione S-transferase, theta 1 [Source:MGI Symbol;Acc:MGI:107379]
ENSMUSG00000001665	10.80624124	8.688134246	11.10358087	9.534159121	8.546230044	5.237857179	4.420339373	3.73706062	3.547733057	5.623812438	4.448488644	4.144123889	Gstt3	glutathione S-transferase, theta 3 [Source:MGI Symbol;Acc:MGI:2143526]
ENSMUSG00000001666	1.195235773	0.890595869	1.943481626	0.984153869	0.369349006	0.374866249	1.318156757	1.044751169	2.159513072	1.509332015	1.346059046	1.433099359	Ddt	D-dopachrome tautomerase [Source:MGI Symbol;Acc:MGI:1298381]
ENSMUSG00000001670	0.0549183	0	0.056224952	0	0	0.041830275	0	0	0	0	0.045713944	0	Tat	tyrosine aminotransferase [Source:MGI Symbol;Acc:MGI:98487]
ENSMUSG00000001672	0.357577461	0.308826621	0.152535492	0.250264059	0.093923099	0.340450353	0.452252437	0.590385702	0.748841476	3.784827718	4.241472096	4.542146542	Marveld3	MARVEL (membrane-associating) domain containing 3 [Source:MGI Symbol;Acc:MGI:1920858]
ENSMUSG00000001674	73.29618582	75.12345917	66.28465534	72.95314495	77.762071	78.57579974	203.7466298	208.915445	228.4593638	208.8602962	202.4529319	202.8464191	Ddx18	DEAD (Asp-Glu-Ala-Asp) box polypeptide 18 [Source:MGI Symbol;Acc:MGI:1914192]
ENSMUSG00000001687	8.880942265	8.914455584	8.806047016	4.43474193	5.535251372	4.602448675	32.30994473	32.50726816	35.26566966	33.27545318	35.17249391	34.56925992	Ubl3	ubiquitin-like 3 [Source:MGI Symbol;Acc:MGI:1344373]
ENSMUSG00000001700	6.646676982	6.982587568	6.566053553	7.306285166	6.353317677	5.900085016	2.453910539	1.881280154	2.155180821	6.347608856	6.184408232	6.038078985	Gramd3	GRAM domain containing 3 [Source:MGI Symbol;Acc:MGI:1914815]
ENSMUSG00000001707	26.28601405	29.28098774	25.59290361	34.42166903	35.97929864	35.89296044	67.27758787	70.79227508	67.46576177	66.60810757	67.48306163	65.64244453	Eef1e1	eukaryotic translation elongation factor 1 epsilon 1 [Source:MGI Symbol;Acc:MGI:1913393]
ENSMUSG00000001729	516.787745	517.8822949	553.6432025	495.7810243	448.7101276	404.6997201	100.8340159	102.787925	106.9499103	134.8075921	143.230386	135.2631427	Akt1	thymoma viral proto-oncogene 1 [Source:MGI Symbol;Acc:MGI:87986]
ENSMUSG00000001739	0.885359832	0.509768679	0.997067391	0.385561516	0.248056616	0.449575043	0.869472006	0.877075056	0.631776739	0.395966732	0.368486373	0.183079789	Cldn15	claudin 15 [Source:MGI Symbol;Acc:MGI:1913103]
ENSMUSG00000001741	0.851346833	0.428911718	0.599226816	0.182064409	0.204984131	0.202642337	0.015834647	0	0	0.126918273	0.324802663	0.345805401	Il16	interleukin 16 [Source:MGI Symbol;Acc:MGI:1270855]
ENSMUSG00000001750	162.8935611	136.7692783	170.6323722	148.9427095	138.7262329	123.6326917	31.78598251	32.97926265	32.71341585	34.94946876	41.76946794	43.31078142	Tcirg1	T cell, immune regulator 1, ATPase, H+ transporting, lysosomal V0 protein A3 [Source:MGI Symbol;Acc:MGI:1350931]
ENSMUSG00000001751	58.17259248	53.49409997	58.57226563	51.17325213	51.80645978	41.67701926	3.809294189	3.720831678	4.33492911	9.648868664	10.10476096	8.840868964	Naglu	alpha-N-acetylglucosaminidase (Sanfilippo disease IIIB) [Source:MGI Symbol;Acc:MGI:1351641]
ENSMUSG00000001755	29.83787813	28.65974554	29.95326524	32.92791627	29.57716458	27.25551588	18.78772747	17.53248234	19.27815841	23.1026739	22.09781521	23.55123484	Coasy	Coenzyme A synthase [Source:MGI Symbol;Acc:MGI:1918993]
ENSMUSG00000001761	25.69928149	35.39927191	24.64177128	31.00037687	32.51371893	34.16177764	12.37689663	11.68444918	13.28596494	14.63057365	15.55371479	14.5927257	Smo	smoothened, frizzled class receptor [Source:MGI Symbol;Acc:MGI:108075]
ENSMUSG00000001763	1.045712254	0.987042144	1.351403691	1.327784327	0.972609239	0.809557482	2.831373706	2.75115033	2.776315683	1.809394435	1.541124523	1.5800088	Tspan33	tetraspanin 33 [Source:MGI Symbol;Acc:MGI:1919012]
ENSMUSG00000001767	36.85718455	35.63111236	33.51231988	35.69492907	37.88773805	36.45649487	48.49462741	49.61276788	51.74890365	55.02278339	51.79598384	52.7005312	Crnkl1	crooked neck pre-mRNA splicing factor 1 [Source:MGI Symbol;Acc:MGI:1914127]
ENSMUSG00000001768	35.50059936	40.88076508	35.25038734	36.08333973	40.66986144	43.45911801	4.118551606	3.96761058	4.586525637	15.69903891	16.84370606	15.51704455	Rin2	Ras and Rab interactor 2 [Source:MGI Symbol;Acc:MGI:1921280]
ENSMUSG00000001773	0.965988502	1.88621875	0.644981701	0.293949325	0.330954014	0.46385876	0.187480986	0.062409733	0.039091412	0.544729636	0.437004337	0.595535847	Folh1	folate hydrolase 1 [Source:MGI Symbol;Acc:MGI:1858193]
ENSMUSG00000001774	56.82012088	65.18820167	52.58894954	65.56540902	75.18701514	80.09938486	61.18735546	58.240222	69.91409526	93.96980179	90.78684709	91.41919587	Chordc1	cysteine and histidine-rich domain (CHORD)-containing, zinc-binding protein 1 [Source:MGI Symbol;Acc:MGI:1914167]
ENSMUSG00000001783	89.84095993	82.03234265	91.30349791	92.53223173	90.77806229	79.93716826	103.3182984	100.189201	92.93217505	101.9508023	100.8169879	98.1649192	Rtcb	RNA 2',3'-cyclic phosphate and 5'-OH ligase [Source:MGI Symbol;Acc:MGI:106379]
ENSMUSG00000001785	39.4837266	41.42598166	35.8598252	45.13172305	42.88521553	42.74523572	98.95114939	91.99673166	100.449544	102.8752576	106.6326228	103.3569676	Pwp1	PWP1 homolog, endonuclein [Source:MGI Symbol;Acc:MGI:1914735]
ENSMUSG00000001786	9.12894179	7.435632358	8.396332293	9.176948363	8.987078378	6.430435269	13.36817043	13.06898565	11.86449638	8.812897863	8.618290866	8.567159699	Fbxo7	F-box protein 7 [Source:MGI Symbol;Acc:MGI:1917004]
ENSMUSG00000001794	473.5204329	400.7358804	478.6389341	453.5140158	406.5267741	343.7013752	442.5287665	481.3151542	422.2313819	215.999357	218.279553	227.3293664	Capns1	calpain, small subunit 1 [Source:MGI Symbol;Acc:MGI:88266]
ENSMUSG00000001802	1.764936075	1.938321348	1.388658096	0.457502596	0.44641712	0.734396968	2.261413741	2.136958563	2.235938197	2.061072259	2.190093791	2.114471737	Lrp3	low density lipoprotein receptor-related protein 3 [Source:MGI Symbol;Acc:MGI:3584516]
ENSMUSG00000001804	0	0	0	0	0	0	0	0	0	0	0	0	Dsg4	desmoglein 4 [Source:MGI Symbol;Acc:MGI:2661061]
ENSMUSG00000001815	0.030625258	0	0.125415659	0	0	0	0	0	0	0	0.025492438	0	Evx2	even-skipped homeobox 2 [Source:MGI Symbol;Acc:MGI:95462]
ENSMUSG00000001819	0.190621573	0.498887592	0.337089309	0	0	0.052797456	0.355836345	0.223171977	0.241940102	0.108504134	0.15867323	0.430012618	Hoxd13	homeobox D13 [Source:MGI Symbol;Acc:MGI:96205]
ENSMUSG00000001823	0	0.039107747	0.034768943	0.015845887	0	0	0.030319538	0.016821566	0	0	0	0.045145507	Hoxd12	homeobox D12 [Source:MGI Symbol;Acc:MGI:96204]
ENSMUSG00000001827	2.123483815	1.480050467	0.62931786	0.625768486	0.410984712	1.319473558	36.26960897	42.90263892	31.15511917	19.84379565	16.97812929	18.96740584	Folr1	folate receptor 1 (adult) [Source:MGI Symbol;Acc:MGI:95568]
ENSMUSG00000001829	30.2819535	30.48145729	31.3927165	35.17320151	34.2441747	32.37478008	59.49410124	58.39221883	62.91220535	44.15673661	45.57429671	43.51711881	Clpb	ClpB caseinolytic peptidase B [Source:MGI Symbol;Acc:MGI:1100517]
ENSMUSG00000001833	141.9763208	181.6985321	110.3569988	188.4902467	213.6360203	235.8348463	82.13988067	80.12607959	97.55034852	137.002129	123.6105528	144.2427088	Septin7	septin 7 [Source:MGI Symbol;Acc:MGI:1335094]
ENSMUSG00000001844	55.34256165	48.02825857	59.6057859	52.48129998	50.51236447	42.36904642	30.36963792	29.95961445	27.97292711	21.6916828	23.0531863	24.33481418	Zdhhc4	zinc finger, DHHC domain containing 4 [Source:MGI Symbol;Acc:MGI:1920131]
ENSMUSG00000001847	188.1828739	185.7638883	149.3211999	198.1453866	208.4754886	203.984231	97.64380069	99.21493855	108.2818398	97.49730931	91.65872829	90.87690616	Rac1	Rac family small GTPase 1 [Source:MGI Symbol;Acc:MGI:97845]
ENSMUSG00000001855	23.1023427	28.46681782	21.78070866	30.53453886	31.32470526	39.02755939	40.76896769	35.53143433	53.4918333	64.21808317	66.8448651	67.23712721	Nup214	nucleoporin 214 [Source:MGI Symbol;Acc:MGI:1095411]
ENSMUSG00000001864	55.74017181	53.56529336	51.5382535	72.85363037	65.1827126	66.27635284	11.80064145	10.02961122	10.10390359	22.74060235	26.0784309	23.57759989	Aif1l	allograft inflammatory factor 1-like [Source:MGI Symbol;Acc:MGI:1919598]
ENSMUSG00000001865	0.139702883	0	0	0	0	0	0	0	0	0	0	0	Cpa3	carboxypeptidase A3, mast cell [Source:MGI Symbol;Acc:MGI:88479]
ENSMUSG00000001870	29.778862	34.83572653	25.10172427	43.28740781	47.00011145	50.81971615	5.270330589	4.990343145	6.532407337	29.32360609	29.64720028	28.7740668	Ltbp1	latent transforming growth factor beta binding protein 1 [Source:MGI Symbol;Acc:MGI:109151]
ENSMUSG00000001891	61.46076047	62.20264453	55.94178485	61.4112014	59.88146804	59.37881386	24.24522278	27.2864423	25.99730389	42.35008064	37.92375051	39.3732137	Ugp2	UDP-glucose pyrophosphorylase 2 [Source:MGI Symbol;Acc:MGI:2183447]
ENSMUSG00000001901	0.052817293	0	0	0.08214705	0.055493026	0.174329897	2.263395657	1.866188913	2.720196053	3.716513938	3.663755675	2.933300361	Kcnh6	potassium voltage-gated channel, subfamily H (eag-related), member 6 [Source:MGI Symbol;Acc:MGI:2684139]

ENSMUSG00000001909	97.89617497	72.95604223	94.40388529	95.86939826	87.65241999	80.15736803	179.3450879	189.2520378	190.8851571	138.5794529	143.8326597	143.2428343	Trmt1	tRNA methyltransferase 1 [Source:MGI Symbol;Acc:MGI:1289155]
ENSMUSG00000001910	89.64268297	96.56827642	85.9433349	100.4646492	102.5086466	108.2115345	233.8710773	219.7465576	250.9262363	228.2636446	230.7636997	224.1845768	Nacc1	nucleus accumbens associated 1, BEN and BTB (POZ) domain containing [Source:MGI Symbol;Acc:MGI:1914080]
ENSMUSG00000001911	95.08158037	106.245596	88.34276377	94.06145228	103.2129816	111.321373	2.424014229	1.946853621	2.238323973	12.90642142	12.50577203	12.55819217	Nfix	nuclear factor I/X [Source:MGI Symbol;Acc:MGI:97311]
ENSMUSG00000001918	31.15083233	31.61362325	30.09472301	46.28803695	45.01210169	41.59341855	81.59821867	80.62649491	85.20874165	34.27834505	37.58906463	34.72345227	Slc1a5	solute carrier family 1 (neutral amino acid transporter), member 5 [Source:MGI Symbol;Acc:MGI:105305]
ENSMUSG00000001924	407.8139272	416.9253169	413.6706965	445.7602195	441.8107944	437.9526261	434.8128168	410.4702699	454.7663686	379.4946879	394.7819925	387.7810351	Uba1	ubiquitin-like modifier activating enzyme 1 [Source:MGI Symbol;Acc:MGI:98890]
ENSMUSG00000001930	0.565526502	0.427953008	0.430100828	0.090469721	0.08488231	0.184607702	4.51515183	4.866037439	4.677163396	3.902272219	3.56420278	3.451006395	Vwf	Von Willebrand factor [Source:MGI Symbol;Acc:MGI:98941]
ENSMUSG00000001942	6.605472611	9.804851148	5.690298514	8.859935039	10.45453455	12.75188632	6.455264053	7.095940096	7.107269921	8.084437381	7.537434337	6.752234007	Siae	sialic acid acetylesterase [Source:MGI Symbol;Acc:MGI:104803]
ENSMUSG00000001943	0.221510877	0	0.113390605	0.103355155	0.058183164	0.168720827	20.41870622	19.42027038	18.65867806	3.219714047	4.240865565	3.582628111	Vsig2	V-set and immunoglobulin domain containing 2 [Source:MGI Symbol;Acc:MGI:1928009]
ENSMUSG00000001946	1.367792991	0.393770985	0.758515566	1.116851701	0.059878567	0.651139483	28.49313679	35.06046546	32.25150215	2.141052434	3.320769912	2.424343609	Esam	endothelial cell-specific adhesion molecule [Source:MGI Symbol;Acc:MGI:1916774]
ENSMUSG00000001948	2.728635813	2.09477627	3.653113431	2.219867067	1.396679754	1.278986171	1.811432143	1.663447715	1.562893718	0.813566769	0.407671753	0.496037785	Spa17	sperm autoantigenic protein 17 [Source:MGI Symbol;Acc:MGI:1333778]
ENSMUSG00000001962	79.33707281	65.67437844	89.50927371	65.33558536	59.36595198	50.54113076	55.06233785	61.37703827	50.4953325	46.32402821	44.30983859	47.2062287	Fam50a	family with sequence similarity 50, member A [Source:MGI Symbol;Acc:MGI:1351626]
ENSMUSG00000001964	36.96036775	40.36282042	33.49535	38.45071694	37.04942066	35.32742067	93.8777203	97.86289294	85.07482483	56.85894835	59.58882538	56.44722874	Emd	emerin [Source:MGI Symbol;Acc:MGI:108117]
ENSMUSG00000001983	5.289112748	6.019030097	4.905312025	6.880964748	9.054739105	8.223127376	7.22188146	6.472471451	9.063907756	12.24490615	12.84538701	12.24223404	Taco1	translational activator of mitochondrially encoded cytochrome c oxidase I [Source:MGI Symbol;Acc:MGI:1917457]
ENSMUSG00000001985	0.191813163	0.270581329	0.250380559	0.138722591	0.156186099	0.273938655	3.70748508	3.282565559	4.753409826	3.73598782	3.536582155	3.761018684	Grik3	glutamate receptor, ionotropic, kainate 3 [Source:MGI Symbol;Acc:MGI:95816]
ENSMUSG00000001986	17.42910251	21.85711405	16.40911093	18.30783752	22.82106445	27.2465615	0.893628475	0.644531573	0.85012738	8.483244696	8.675620027	8.970491391	Gria3	glutamate receptor, ionotropic, AMPA3 (alpha 3) [Source:MGI Symbol;Acc:MGI:95810]
ENSMUSG00000001988	1.061292461	0.983019881	0.307066623	0.452130313	0.896894251	0.738074459	0.391357763	0.479969331	0.644222277	0.495287503	0.518528492	0.327145563	Npas1	neuronal PAS domain protein 1 [Source:MGI Symbol;Acc:MGI:109205]
ENSMUSG00000001995	4.989360484	6.654991421	4.268387815	6.29302653	7.970898346	9.179733631	9.512185009	8.726788446	12.05150077	13.97956844	13.35073085	13.00933723	Sipa1l2	signal-induced proliferation-associated 1 like 2 [Source:MGI Symbol;Acc:MGI:2676970]
ENSMUSG00000001998	3.275664957	5.425299408	2.277136993	5.283352347	7.945446928	13.10652799	6.354336764	4.486931337	8.149098971	14.46916029	16.67975733	15.7871659	Ap4e1	adaptor-related protein complex AP-4, epsilon 1 [Source:MGI Symbol;Acc:MGI:1336993]
ENSMUSG00000001999	22.67533009	19.4527728	28.29599563	23.75216308	20.28394389	17.13266264	27.27657892	27.87595119	25.21613244	12.6664101	14.02353855	14.93034363	Blvra	biliverdin reductase A [Source:MGI Symbol;Acc:MGI:88170]
ENSMUSG00000002006	4.044541905	4.996699443	4.175568739	3.964600892	3.713795504	4.340529853	20.68414338	20.6619762	20.01403096	22.92253289	23.22721546	22.85163009	Pdzd4	PDZ domain containing 4 [Source:MGI Symbol;Acc:MGI:2443483]
ENSMUSG00000002007	0	0	0.123482131	0.028138387	0.063361343	0	0.08076003	0.089612854	0.168391667	0.05394249	0.050198844	0.13361214	Srpk3	serine/arginine-rich protein specific kinase 3 [Source:MGI Symbol;Acc:MGI:1891338]
ENSMUSG00000002010	70.0628338	63.13216793	64.82249974	62.38758563	59.18720626	55.66552409	201.5937408	213.5498883	203.2757921	135.2565046	137.1016582	139.7370257	Idh3g	isocitrate dehydrogenase 3 (NAD+), gamma [Source:MGI Symbol;Acc:MGI:1099463]
ENSMUSG00000002012	0.823511729	1.461987303	1.545692989	1.088690787	0.68497452	0.810203062	6.616912663	7.750154127	5.876430526	3.836519774	3.027582776	3.375400654	Pnck	pregnancy upregulated non-ubiquitously expressed CaM kinase [Source:MGI Symbol;Acc:MGI:1347357]
ENSMUSG00000002014	91.95571671	72.95177512	107.7522176	69.4485504	59.51367354	50.37659322	65.21507621	68.08128351	55.54234441	48.10092323	44.52727055	44.00501396	Ssr4	signal sequence receptor, delta [Source:MGI Symbol;Acc:MGI:1099464]
ENSMUSG00000002015	187.7157492	163.6881866	181.7019803	144.1770078	140.2236683	113.6201897	135.6652558	141.0954024	123.170032	116.0637936	116.8529235	115.149485	Bcap31	B cell receptor associated protein 31 [Source:MGI Symbol;Acc:MGI:1350933]
ENSMUSG00000002017	49.94735431	51.06398587	51.57479392	45.51616823	45.02808585	44.83834451	45.79152705	43.7587606	48.05898183	57.05425447	58.6635203	54.60226552	Fam98a	family with sequence similarity 98, member A [Source:MGI Symbol;Acc:MGI:1919972]
ENSMUSG00000002020	79.58993094	95.26181835	76.66945866	54.95370739	59.93212845	66.75075763	4.695609524	4.198174423	4.949409443	50.08835991	54.14088949	51.9619991	Ltbp2	latent transforming growth factor beta binding protein 2 [Source:MGI Symbol;Acc:MGI:99502]
ENSMUSG00000002028	9.103082485	10.49617602	7.998581961	11.58189192	13.59454066	20.47482778	6.508950874	5.490925038	7.724856789	14.93140026	15.57467731	16.99762791	Kmt2a	lysine (K)-specific methyltransferase 2A [Source:MGI Symbol;Acc:MGI:96995]
ENSMUSG00000002031	104.7333336	86.68957145	115.6273412	100.5984191	89.65107694	76.47271482	63.30576219	70.01732381	62.10830985	66.82224465	65.88772815	63.86072006	Ift46	intraflagellar transport 46 [Source:MGI Symbol;Acc:MGI:1923818]
ENSMUSG00000002032	0.824827243	0.379932388	0.42222604	0.59011501	0.808838923	0.60731412	1.325496562	1.361848169	1.433068681	0.713195543	0.823902567	0.682250052	Tmem25	transmembrane protein 25 [Source:MGI Symbol;Acc:MGI:1918937]
ENSMUSG00000002033	0	0	0.033246981	0	0	0	0	0	0	0	0	0	Cd3g	CD3 antigen, gamma polypeptide [Source:MGI Symbol;Acc:MGI:88333]
ENSMUSG00000002043	77.80407389	63.76870312	103.9065521	80.48332527	67.58080971	49.13703826	43.87055077	43.06271583	29.43583318	24.00567156	23.65067473	23.84004437	Trappc6a	trafficking protein particle complex 6A [Source:MGI Symbol;Acc:MGI:1914341]
ENSMUSG00000002052	26.43939179	27.33270844	25.33941306	27.0795303	29.64064337	31.67141437	34.44115977	31.15346178	38.05134002	64.23590985	66.98697535	66.2017307	Supt6	SPT6, histone chaperone and transcription elongation factor [Source:MGI Symbol;Acc:MGI:107726]
ENSMUSG00000002055	28.45577348	31.14540982	28.11976389	39.59384748	41.27031152	42.25222173	70.59231372	66.09234225	79.37267382	90.33273222	95.56990677	92.74135008	Spag5	sperm associated antigen 5 [Source:MGI Symbol;Acc:MGI:1927470]
ENSMUSG00000002057	0.026775661	0.015416775	0	0	0.028132121	0.010197268	0.023904701	0	0.074765062	0.083825689	0	0.11864625	Foxn1	forkhead box N1 [Source:MGI Symbol;Acc:MGI:102949]
ENSMUSG00000002058	43.8078408	34.95390044	53.79497855	37.81852822	32.71030851	25.33816085	44.31074683	48.88359846	40.24004357	32.43409178	31.65132847	32.89826382	Unc119	unc-119 lipid binding chaperone [Source:MGI Symbol;Acc:MGI:1328357]
ENSMUSG00000002059	184.9818622	147.2447306	203.3429681	164.9958229	145.6817898	128.4136338	24.2259468	26.07646297	26.05506185	51.69422556	53.2179388	51.65748159	Rab34	RAB34, member RAS oncogene family [Source:MGI Symbol;Acc:MGI:104606]
ENSMUSG00000002064	106.0819482	81.63269507	121.7418416	90.14220583	75.10647138	62.23224579	46.43329006	43.4429566	40.04915152	50.907406	48.51870672	44.54930399	Sdf2	stromal cell derived factor 2 [Source:MGI Symbol;Acc:MGI:108019]
ENSMUSG00000002068	53.05694136	44.65714095	54.21815409	43.1039056	38.66842385	36.16054401	457.2293825	464.5776949	443.2535255	249.3101105	238.2171753	245.1275928	Ccne1	cyclin E1 [Source:MGI Symbol;Acc:MGI:88316]
ENSMUSG00000002076	3.213644188	2.203456225	1.808302175	3.182060102	2.036178535	2.578571749	62.74718182	64.9594589	58.8179101	14.81150359	15.82558637	16.65325256	Hsf2bp	heat shock transcription factor 2 binding protein [Source:MGI Symbol;Acc:MGI:1921627]
ENSMUSG00000002083	22.57074696	19.07877555	28.51596553	27.51588771	23.00780487	19.60588735	84.075367	84.2050786	77.46113793	34.10652377	34.29785621	36.13262955	Bbc3	BCL2 binding component 3 [Source:MGI Symbol;Acc:MGI:2181667]
ENSMUSG00000002100	0.041473241	0	0.08492	0.02902661	0.010893571	0.015794709	0.240671375	0.256782215	0.231609551	0.565726735	0.517834344	0.661582925	Mybpc3	myosin binding protein C, cardiac [Source:MGI Symbol;Acc:MGI:102844]
ENSMUSG00000002102	282.4316701	233.9896807	283.5277805	252.4970292	224.4063972	187.5441032	288.4383405	310.805915	281.3665922	254.0615227	260.6630268	256.5857683	Psmc3	proteasome (prosome, macropain) 26S subunit, ATPase 3 [Source:MGI Symbol;Acc:MGI:1098754]
ENSMUSG00000002103	19.55111924	21.84976778	18.17873118	18.1251453	19.83441304	21.48315692	4.034632718	4.730913451	5.086316823	11.08052067	11.24529833	10.45281857	Acp2	acid phosphatase 2, lysosomal [Source:MGI Symbol;Acc:MGI:87882]
ENSMUSG00000002104	1.952831371	0.9528753	1.728203662	1.575251823	0.938941996	1.613490247	0.590997755	0.426258477	0.462105594	0.562516432	0.358168755	0.879990108	Rapsn	receptor-associated protein of the synapse [Source:MGI Symbol;Acc:MGI:99422]
ENSMUSG00000002105	173.1487045	162.0537264	172.236353	146.5722643	141.3326185	125.2834556	13.51063505	12.55711345	13.60387011	30.11934042	27.99314917	28.84803912	Slc39a13	solute carrier family 39 (metal ion transporter), member 13 [Source:MGI Symbol;Acc:MGI:1915677]
ENSMUSG00000002107	17.10941846	20.37108131	15.2254321	21.26627579	23.79463497	29.11688009	0.885134886	0.743637238	1.199628963	4.738149909	5.211013483	4.706991198	Celf2	CUGBP, Elav-like family member 2 [Source:MGI Symbol;Acc:MGI:1338822]
ENSMUSG00000002108	2.20432827	2.132253746	3.099304488	0.987380603	1.019039963	0.514892401	0.551032743	0.320276178	0.355627712	1.209321348	1.345579338	1.276306236	Nr1h3	nuclear receptor subfamily 1, group H, member 3 [Source:MGI Symbol;Acc:MGI:1352462]
ENSMUSG00000002109	3.949252885	5.367423517	2.58577776	5.720906394	7.285438709	7.974895096	5.022219449	4.480945569	6.38991265	4.497793295	4.300318561	3.418531477	Ddb2	damage specific DNA binding protein 2 [Source:MGI Symbol;Acc:MGI:1355314]
ENSMUSG00000002111	11.30815493	8.497355686	10.15458329	0.670715109	0.251716695	0.218980093	0.385004105	1.044285801	0.579776136	8.100473991	8.136573049	8.19560201	Spi1	spleen focus forming virus (SFFV) proviral integration oncogene [Source:MGI Symbol;Acc:MGI:98282]
ENSMUSG00000002129	39.5985215	49.87866417	40.69824648	53.12077369	51.6765499	57.54294068	92.80808894	83.96660342	102.2409612	104.4111464	110.397237	106.0394825	Sf3a1	splicing factor 3a, subunit 1 [Source:MGI Symbol;Acc:MGI:1914715]
ENSMUSG00000002147	77.57640132	92.33500205	72.62572104	99.60527499	109.9168322	110.5792279	43.78300412	39.21715713	44.22228605	42.99584612	43.946554	40.5354625	Stat6	signal transducer and activator of transcription 6 [Source:MGI Symbol;Acc:MGI:103034]
ENSMUSG00000002190	0.611121196	0.495815037	0.312830699	0.336988516	0.248076634	0.560691767	7.340733896	6.136581044	7.135936617	12.78376933	13.04113478	12.44427587	Clgn	calmegin [Source:MGI Symbol;Acc:MGI:107472]
ENSMUSG00000002204	2.566775304	4.997944635	4.156774151	2.874327047	2.745841462	1.670693026	2.249893839	1.618117863	1.737490305	0.751391968	0.89347954	1.695714851	Napsa	napsin A aspartic peptidase [Source:MGI Symbol;Acc:MGI:109365]
ENSMUSG00000002205	26.24838842	24.98816606	27.11438423	22.23062555	21.52439903	22.89305122	27.97636201	31.74407295	25.37178041	20.49476674	22.32594587	21.35084327	Vrk3	vaccinia related kinase 3 [Source:MGI Symbol;Acc:MGI:2182465]
ENSMUSG00000002210	18.49748646	14.85571271	21.66037763	18.96548031	18.39194501	16.02419828	24.70032591	25.9530069	25.08613827	15.33837774	16.12420225	15.26723306	Smg9	smg-9 homolog, nonsense mediated mRNA decay factor (C. elegans) [Source:MGI Symbol;Acc:MGI:1919247]
ENSMUSG00000002221	22.9170905	27.99208252	20.44507174	29.9005898	34.48218035	41.89687131	49.17101321	45.84906249	60.63331817	46.94477433	51.39966952	48.09969676	Paxip1	PAX interacting (with transcription-activation domain) protein 1 [Source:MGI Symbol;Acc:MGI:1890430]
ENSMUSG00000002222	9.654672618	12.24819487	9.779389557	10.51345742	13.40702938	17.08444862	33.62771189	28.62041936	35.71287975	42.09170854	38.45100728	38.14588831	Rmnd5a	required for meiotic nuclear division 5 homolog A [Source:MGI Symbol;Acc:MGI:1915727]
ENSMUSG00000002227	33.9942908	34.89026059	37.80085204	29.34036285	29.2259585	29.7958005	104.8199504	101.2408101	110.9743709	55.11285143	55.89513223	53.92404115	Mov10	Mov10 RISC complex RNA helicase [Source:MGI Symbol;Acc:MGI:97054]
ENSMUSG00000002228	10.05074434	7.06522174	7.190517387	4.444756458	4.07129321	4.765453282	124.0014192	133.3157376	116.9540419	22.09628088	23.51952424	21.96398075	Ppm1j	protein phosphatase 1J [Source:MGI Symbol;Acc:MGI:1919137]
ENSMUSG00000002233	668.7677041	559.2392281	752.5690117	622.5386683	558.0888955	436.7116516	167.8229832	186.3326181	149.307911	160.5877218	165.4511635	170.8394589	Rhoc	ras homolog family member C [Source:MGI Symbol;Acc:MGI:106028]
ENSMUSG00000002240	0	0	0	0	0	0	0	0	0	0	0	0	Prr27	proline rich 27 [Source:MGI Symbol;Acc:MGI:1921029]
ENSMUSG00000002249	42.2540206	48.91998234	42.60638492	45.87146912	44.65787297	44.10352592	5.226372417	5.099757321	5.067097519	9.779896166	9.575183757	9.507992646	Tead3	TEA domain family member 3 [Source:MGI Symbol;Acc:MGI:109241]
ENSMUSG00000002250	47.46833769	48.84834323	45.299012	51.58869587	51.87228037	50.12724619	25.39095013	21.11185853	24.60566974	27.75399934	27.1160779	30.60075665	Ppard	peroxisome proliferator activator receptor delta [Source:MGI Symbol;Acc:MGI:101884]
ENSMUSG00000002257	20.3309605	13.13062164	20.3191004	10.49009608	12.03671476	10.77445928	69.1947559	74.64851579	62.92722933	28.24054533	28.20578944	27.59868977	Def6	differentially expressed in FDCP 6 [Source:MGI Symbol;Acc:MGI:1346328]
ENSMUSG00000002265	8.295333473	10.87170263	7.319860305	7.051120541	9.697252576	13.15045201	2.995720304	2.414606176	3.493713747	40.87166217	41.14612357	42.68350812	Peg3	paternally expressed 3 [Source:MGI Symbol;Acc:MGI:104748]
ENSMUSG00000002266	0.41360717	0.69138859	0.614682561	0.199211053	0.378488247	0.508124351	0.53602122	0.370084879	0.670590444	2.315245109	2.743183951	1.915513352	Zim1	zinc finger, imprinted 1 [Source:MGI Symbol;Acc:MGI:1341879]

ENSMUSG00000002274	78.59356531	62.46144152	82.18542769	89.07009253	73.15059329	66.98924414	39.72871716	43.14652174	34.50488788	10.02943749	10.85006326	10.80639319	Metrn	meteorin, glial cell differentiation regulator [Source:MGI Symbol;Acc:MGI:1917333]
ENSMUSG00000002279	18.19849684	14.21970156	17.89012995	16.60910022	13.01390918	13.61471716	5.018769686	5.81433175	5.657962505	5.48854082	5.615223171	5.201476813	Lmf1	lipase maturation factor 1 [Source:MGI Symbol;Acc:MGI:1923733]
ENSMUSG00000002280	17.34170855	15.64114005	16.59977046	20.32229018	18.22024246	16.79740137	14.96769795	13.92725717	14.76477164	12.77701854	13.66339746	13.08111873	Ciao3	cytosolic iron-sulfur assembly component 3 [Source:MGI Symbol;Acc:MGI:1914813]
ENSMUSG00000002289	214.7861523	200.0285542	218.3251967	195.2686645	185.3829003	170.1744052	19.18295898	15.99148044	19.61643154	57.36423013	56.64541517	55.6206062	Angptl4	angiopoietin-like 4 [Source:MGI Symbol;Acc:MGI:1888999]
ENSMUSG00000002297	42.28746909	39.67943415	33.07712013	48.65900787	49.82776764	53.69520947	79.12313227	74.16701744	85.84588507	83.4716056	82.16086935	76.98818932	Dbf4	DBF4 zinc finger [Source:MGI Symbol;Acc:MGI:1351328]
ENSMUSG00000002307	91.75998384	87.28981253	93.87897609	85.68999391	84.75998515	71.89454974	94.26326158	98.75481895	93.34196487	86.03417309	83.15775062	82.21105867	Daxx	Fas death domain-associated protein [Source:MGI Symbol;Acc:MGI:1197015]
ENSMUSG00000002308	17.86638794	14.82378124	21.74395168	14.49398333	12.67331908	9.064103673	39.89123505	40.89126158	36.39734494	29.38079121	31.52532823	29.59826061	Cd320	CD320 antigen [Source:MGI Symbol;Acc:MGI:1860083]
ENSMUSG00000002319	50.01059494	46.89507729	58.0066897	46.6378904	37.38110174	34.45343474	43.03775965	48.02026787	40.42228026	37.14327811	38.84796199	39.02109346	Ipo4	importin 4 [Source:MGI Symbol;Acc:MGI:1923001]
ENSMUSG00000002320	31.64795683	39.59073505	26.39875906	36.2421033	39.43882273	42.02833719	13.90229306	12.90338546	15.23447996	22.47108764	23.47308177	21.22935446	Tm9sf1	transmembrane 9 superfamily member 1 [Source:MGI Symbol;Acc:MGI:1921390]
ENSMUSG00000002324	7.088026096	7.538056156	9.305610991	12.21724035	11.03924053	8.923933914	19.65411139	22.42813498	20.25741967	21.14600478	20.40728952	18.95560088	Rec8	REC8 meiotic recombination protein [Source:MGI Symbol;Acc:MGI:1929645]
ENSMUSG00000002325	33.2253614	37.20001861	28.42515227	41.01303241	45.23136031	43.83232933	13.25527839	11.29737044	14.1049764	21.20565324	20.6102204	20.34921061	Irf9	interferon regulatory factor 9 [Source:MGI Symbol;Acc:MGI:107587]
ENSMUSG00000002326	4.393508482	3.975204252	3.177188223	3.286467797	4.76262673	5.657104048	5.13650804	5.181423951	3.764750062	5.302231996	5.311578778	4.542574546	Gmpr2	guanosine monophosphate reductase 2 [Source:MGI Symbol;Acc:MGI:1917903]
ENSMUSG00000002329	24.84879141	20.34410068	21.59938944	23.90657883	22.67153372	21.00272559	22.38481064	23.59985458	19.84551845	14.19638104	13.58479107	17.0205737	Mdp1	magnesium-dependent phosphatase 1 [Source:MGI Symbol;Acc:MGI:1915131]
ENSMUSG00000002332	98.32113402	85.65443643	99.70279815	144.4813949	120.1915195	114.9729532	49.82796289	49.31831171	47.85260939	33.93262736	31.43348975	33.74240117	Dhrs1	dehydrogenase/reductase (SDR family) member 1 [Source:MGI Symbol;Acc:MGI:1196314]
ENSMUSG00000002341	0.077744314	0.089526574	0.085716679	0.050226727	0.043983076	0.068326659	0.144155811	0.349537876	0.328408609	1.438952461	1.349044233	1.314379481	Ncan	neurocan [Source:MGI Symbol;Acc:MGI:104694]
ENSMUSG00000002342	51.4858113	46.26045295	49.44485324	50.53799797	47.23489814	42.86050802	45.23680229	44.2426902	46.43630069	34.20783908	41.3746665	37.11383568	Tmem161a	transmembrane protein 161A [Source:MGI Symbol;Acc:MGI:2384577]
ENSMUSG00000002343	24.60426099	19.25937187	24.83376403	21.73478199	20.29098669	17.35787448	34.24232504	39.64127309	33.0028017	32.47638096	32.56956451	33.30638803	Armc6	armadillo repeat containing 6 [Source:MGI Symbol;Acc:MGI:1924063]
ENSMUSG00000002345	24.62814764	19.88500298	29.90915934	20.55224063	15.04961124	16.08514434	15.46703335	18.73293833	14.80764681	12.66051525	13.1485635	13.44686639	Borcs8	BLOC-1 related complex subunit 8 [Source:MGI Symbol;Acc:MGI:1919618]
ENSMUSG00000002346	6.728605331	8.237131753	7.934877365	5.780222969	7.069477623	6.801477428	10.82128334	10.35666376	11.57430722	7.270105449	8.571448096	7.425954768	Slc25a42	solute carrier family 25, member 42 [Source:MGI Symbol;Acc:MGI:1920345]
ENSMUSG00000002365	115.2719702	125.6022605	96.76796113	114.1268212	116.1748646	130.0853569	11.86626685	13.5333834	14.19335451	50.06548912	53.39937603	49.41083656	Snx9	sorting nexin 9 [Source:MGI Symbol;Acc:MGI:1913866]
ENSMUSG00000002372	77.26109693	67.48041769	77.29831305	71.92131223	65.86427375	57.20062497	90.09450582	91.61024727	84.70063648	69.91626156	70.28814459	72.72640165	Ranbp3	RAN binding protein 3 [Source:MGI Symbol;Acc:MGI:1919060]
ENSMUSG00000002379	28.41503913	16.06322205	48.13256154	23.76149203	20.16151419	12.25512264	38.94198757	35.46061087	19.92153335	19.40902592	15.05168867	15.89416096	Ndufa11	NADH:ubiquinone oxidoreductase subunit A11 [Source:MGI Symbol;Acc:MGI:1917125]
ENSMUSG00000002384	0.080691023	0.092919861	0.103263596	0.094124421	0.211947109	0.092191338	0.414224868	0.439647749	0.582055179	0.902202681	0.789213853	0.87600203	Bmp8b	bone morphogenetic protein 8b [Source:MGI Symbol;Acc:MGI:107335]
ENSMUSG00000002393	32.49495501	33.07115477	29.60555383	31.71475617	27.30248325	25.92402325	22.09075904	22.00202531	22.2906363	19.06620967	16.54359145	17.48125076	Nr2f6	nuclear receptor subfamily 2, group F, member 6 [Source:MGI Symbol;Acc:MGI:1352453]
ENSMUSG00000002395	148.0372168	116.7425933	188.069222	150.2517235	129.6233545	98.64736236	95.17923521	110.1608446	75.87475729	63.17565696	62.83506443	62.11973183	Use1	unconventional SNARE in the ER 1 homolog (S. cerevisiae) [Source:MGI Symbol;Acc:MGI:1914273]
ENSMUSG00000002396	15.16294023	11.20687901	19.55390013	14.91512074	14.5141488	10.3277727	5.515728729	5.759273789	4.834395673	5.542527982	5.097191936	5.136071025	Ocel1	occludin/ELL domain containing 1 [Source:MGI Symbol;Acc:MGI:1924340]
ENSMUSG00000002409	9.196928696	8.132172412	8.444282057	7.645848833	7.783959817	8.700821424	2.574023301	2.621182323	3.023218393	4.292763001	4.298632638	4.544252001	Dyrk1b	dual-specificity tyrosine-(Y)-phosphorylation regulated kinase 1b [Source:MGI Symbol;Acc:MGI:1330302]
ENSMUSG00000002413	6.465420451	8.48645993	4.400152798	8.079240359	9.746986311	13.88697708	13.63428758	11.91413302	18.3645073	19.27721977	18.2074272	19.3298921	Braf	Braf transforming gene [Source:MGI Symbol;Acc:MGI:88190]
ENSMUSG00000002416	2.719400431	4.717113887	2.466926011	3.083787098	3.001832338	4.326170724	1.628789813	1.739134586	2.563145697	1.262405297	0.916912012	1.250759274	Ndufb2	NADH:ubiquinone oxidoreductase subunit B2 [Source:MGI Symbol;Acc:MGI:1915448]
ENSMUSG00000002428	8.758260819	10.55733278	7.187776871	10.22951372	11.11655628	14.01987816	18.55162519	18.72402634	21.99679268	22.55450426	22.27090025	23.56077696	Hltf	helicase-like transcription factor [Source:MGI Symbol;Acc:MGI:1196437]
ENSMUSG00000002455	57.36487668	48.6150389	58.19565338	53.64014215	51.17654717	48.84462258	79.10665504	77.92089178	77.5086857	72.65532583	71.53725287	68.23512581	Prpf6	pre-mRNA splicing factor 6 [Source:MGI Symbol;Acc:MGI:1922946]
ENSMUSG00000002458	34.35957947	33.53561131	32.77339495	29.57790288	32.25777175	27.78741729	31.23978164	30.72818192	27.0216206	38.36680803	35.21554284	37.89282344	Rgs19	regulator of G-protein signaling 19 [Source:MGI Symbol;Acc:MGI:1915153]
ENSMUSG00000002459	1.929365359	3.018244607	1.677116124	3.634874897	2.753806194	3.632315316	3.152488024	3.60624769	3.557665744	2.214197727	1.696907806	1.580805694	Rgs20	regulator of G-protein signaling 20 [Source:MGI Symbol;Acc:MGI:1929866]
ENSMUSG00000002475	3.752484404	4.609257267	2.378236099	2.80140543	3.379359345	3.83815358	3.988253122	5.062704503	5.72130736	2.237672463	2.588096484	3.610590643	Abhd3	abhydrolase domain containing 3 [Source:MGI Symbol;Acc:MGI:2147183]
ENSMUSG00000002477	152.2934586	131.6340566	180.2674158	164.1786255	142.0616503	114.6816233	368.3836509	393.5572404	292.6875177	199.9826996	187.7236237	191.431014	Snrpd1	small nuclear ribonucleoprotein D1 [Source:MGI Symbol;Acc:MGI:98344]
ENSMUSG00000002486	12.93285623	13.2971864	11.74726672	17.0368711	14.76294238	14.7020183	11.71978599	11.48730472	11.69797251	10.30694693	9.996348298	10.36267071	Tchp	trichoplein, keratin filament binding [Source:MGI Symbol;Acc:MGI:1925082]
ENSMUSG00000002489	6.673321119	8.662365991	5.284879266	6.591889021	7.564454215	11.62740511	9.156500339	7.418310511	12.45411657	22.98038403	23.22301753	22.22663782	Tiam1	T cell lymphoma invasion and metastasis 1 [Source:MGI Symbol;Acc:MGI:103306]
ENSMUSG00000002496	47.46945274	53.90497922	46.46696613	53.20087952	59.42940417	62.35965507	58.314697	50.66687181	65.48283491	61.90385505	63.69213264	62.46263943	Tsc2	TSC complex subunit 2 [Source:MGI Symbol;Acc:MGI:102548]
ENSMUSG00000002500	1.245335476	1.23626536	3.253359795	2.604766526	1.127952055	1.668138775	4.37055226	5.10489194	5.835505112	1.920557204	2.144723083	2.169237093	Rpl3l	ribosomal protein L3-like [Source:MGI Symbol;Acc:MGI:1913461]
ENSMUSG00000002504	32.17688676	29.49968026	33.93736715	27.55828403	21.21444299	21.11600953	22.09938594	21.84775255	19.87382613	16.68455757	17.12199443	16.55456088	Slc9a3r2	solute carrier family 9 (sodium/hydrogen exchanger), member 3 regulator 2 [Source:MGI Symbol;Acc:MGI:1890662]
ENSMUSG00000002524	236.3738871	207.3664764	248.7237261	226.7920396	211.5511601	175.6674251	245.7540766	257.8238837	235.8809986	184.9290356	187.9208967	184.6496264	Puf60	poly-U binding splicing factor 60 [Source:MGI Symbol;Acc:MGI:1915209]
ENSMUSG00000002546	46.45038111	50.66039732	42.48694182	47.25337366	52.60917249	51.58719577	17.61493309	17.09361405	20.02457576	26.87970917	28.10503554	29.56751874	Golga2	golgi autoantigen, golgin subfamily a, 2 [Source:MGI Symbol;Acc:MGI:2139395]
ENSMUSG00000002550	52.07190322	51.54671299	44.55956978	57.18595116	56.8791798	50.63932171	47.9632679	44.34397208	44.20011924	27.4439146	30.24841691	29.82564042	Uck1	uridine-cytidine kinase 1 [Source:MGI Symbol;Acc:MGI:98904]
ENSMUSG00000002565	0.045693969	0.122777561	0	0.085281723	0.016002945	0.023202846	0.040794535	0.045266387	0.056706763	0.095368412	0.3169635	0.12148536	Scin	scinderin [Source:MGI Symbol;Acc:MGI:1306794]
ENSMUSG00000002578	1.029712733	1.185767145	0.829544139	0.834889888	1.170556693	0.835743955	0.753527934	0.668903063	0.895567531	1.238137796	1.222466915	1.645594161	Ikzf4	IKAROS family zinc finger 4 [Source:MGI Symbol;Acc:MGI:1343139]
ENSMUSG00000002580	2.476874132	1.127632886	5.130583356	1.988860348	2.844432346	1.052980583	3.342657985	2.282508578	0.53613322	1.597224843	0.86305724	1.020961304	Mien1	migration and invasion enhancer 1 [Source:MGI Symbol;Acc:MGI:1913678]
ENSMUSG00000002588	0.076836585	0	0	0.035851319	0	0	0	0.038058793	0	0.034364256	0.031979353	0.068094473	Pon1	paraoxonase 1 [Source:MGI Symbol;Acc:MGI:103295]
ENSMUSG00000002602	344.0246984	430.2968857	306.4934007	443.3711942	505.2443229	570.4691691	17.55200534	15.87775083	20.99388817	156.1330017	157.6661396	158.0692188	Axl	AXL receptor tyrosine kinase [Source:MGI Symbol;Acc:MGI:1347244]
ENSMUSG00000002603	68.59513177	64.85098286	72.04341322	64.69351764	65.01665185	58.79187695	29.37789163	30.87531086	32.0198956	49.00448426	51.13387397	52.8065204	Tgfb1	transforming growth factor, beta 1 [Source:MGI Symbol;Acc:MGI:98725]
ENSMUSG00000002608	44.63745906	45.52626436	47.11143424	41.86943843	40.71646621	42.61322982	43.17566932	39.02815329	44.62741991	47.04084779	47.56450451	47.13650718	Ccdc97	coiled-coil domain containing 97 [Source:MGI Symbol;Acc:MGI:1196455]
ENSMUSG00000002617	2.762267225	4.241190273	2.690038243	3.415979586	3.515678201	5.011893553	2.425990262	2.335967645	2.842737269	3.595692057	3.738713516	3.861056112	Zfp40	zinc finger protein 40 [Source:MGI Symbol;Acc:MGI:99185]
ENSMUSG00000002625	53.923243	44.34431398	56.24448914	54.7547425	52.39293999	44.55942604	49.56421576	52.06955403	52.05041242	40.06895354	36.87811623	37.25982808	Akap8l	A kinase (PRKA) anchor protein 8-like [Source:MGI Symbol;Acc:MGI:1860606]
ENSMUSG00000002633	0.015113624	0.017404115	0	0.042311357	0.047637849	0	0	0.044916594	0	0.02703756	0	0.013394066	Shh	sonic hedgehog [Source:MGI Symbol;Acc:MGI:98297]
ENSMUSG00000002635	33.98501716	32.30336561	30.78350014	33.16559013	31.83343434	26.50001133	84.33783223	87.59126669	79.07964993	52.75994319	55.04387289	56.61230428	Pdcd2l	programmed cell death 2-like [Source:MGI Symbol;Acc:MGI:1915329]
ENSMUSG00000002658	108.2603771	87.84433638	128.8983722	107.2942087	92.25972481	75.83644962	82.78099014	89.81963715	74.87348654	84.86630209	81.50060134	85.77127426	Gtf2f1	general transcription factor IIF, polypeptide 1 [Source:MGI Symbol;Acc:MGI:1923848]
ENSMUSG00000002660	101.7754218	83.93366469	111.1558189	86.59332526	74.6032898	61.38772836	157.9397738	173.1487184	143.9284035	106.2897576	117.2004952	119.5619847	Clpp	caseinolytic mitochondrial matrix peptidase proteolytic subunit [Source:MGI Symbol;Acc:MGI:1858213]
ENSMUSG00000002661	19.55840356	19.17825605	25.36341678	23.17397688	17.80933753	12.91097783	29.10216218	31.17675638	22.94827415	15.37398106	15.39237624	18.27858241	Alkbh7	alkB homolog 7 [Source:MGI Symbol;Acc:MGI:1913650]
ENSMUSG00000002664	1.361661007	0.522674215	1.11524684	1.440103635	0.572257194	0.691435035	0.40521998	0	0.337967523	0.64958593	0.151126057	1.448084988	Pspn	persephin [Source:MGI Symbol;Acc:MGI:1201684]
ENSMUSG00000002668	0.873827834	0.763367888	0.709525038	0.618611003	0.316584862	0.206558954	5.353330504	4.805855378	4.907984255	3.449901748	3.837522373	3.845334927	Dennd1c	DENN/MADD domain containing 1C [Source:MGI Symbol;Acc:MGI:1918035]
ENSMUSG00000002679	56.7645051	50.18476582	59.41406052	56.0089537	53.96018511	42.90035265	58.3610283	64.97709348	47.59687623	50.03435628	45.55171856	49.81721779	Med6	mediator complex subunit 6 [Source:MGI Symbol;Acc:MGI:1917042]
ENSMUSG00000002688	6.17216743	6.822722792	5.921066205	8.046084253	7.759544376	8.783401643	0.273415331	0.210037027	0.30697406	1.085206826	0.970673603	1.19002221	Prkd1	protein kinase D1 [Source:MGI Symbol;Acc:MGI:99879]
ENSMUSG00000002699	2.465306154	2.165779183	1.09284968	0.166021468	0.267030823	0.116151284	0.158832828	0.125888505	0.094622921	3.296370422	3.617652514	2.702863888	Lcp2	lymphocyte cytosolic protein 2 [Source:MGI Symbol;Acc:MGI:1321402]
ENSMUSG00000002718	58.87053428	71.05835996	49.74208811	75.17415424	82.50066617	97.9074771	160.4370037	142.4929303	173.1285784	234.6689721	232.0466147	221.7395403	Cse1l	chromosome segregation 1-like (S. cerevisiae) [Source:MGI Symbol;Acc:MGI:1339951]
ENSMUSG00000002728	105.2978072	100.8869004	105.3053146	133.81043	128.5757198	121.6355405	101.5815471	101.67583	97.59400197	95.97192646	95.81937306	90.12338557	Naa20	N(alpha)-acetyltransferase 20, NatB catalytic subunit [Source:MGI Symbol;Acc:MGI:1915127]
ENSMUSG00000002731	31.3811213	32.67705125	31.86192924	36.48428072	35.50411898	34.04542661	25.03588765	24.28938391	25.34144588	15.49472439	15.09866561	16.83107517	Prkra	protein kinase, interferon inducible double stranded RNA dependent activator [Source:MGI Symbol;Acc:MGI:1344375]
ENSMUSG00000002732	69.46387844	62.77240689	67.11185917	41.15437098	37.85036624	31.28429243	8.167380998	10.93771752	11.27483445	12.41547302	17.3307462	14.2019909	Fkbp7	FK506 binding protein 7 [Source:MGI Symbol;Acc:MGI:1336879]
ENSMUSG00000002733	31.06012572	34.86259286	23.91637686	34.58135797	39.20989266	35.98559539	15.54831282	14.58047864	17.98921109	23.21459201	22.03947724	22.95401262	Plekha3	pleckstrin homology domain-containing, family A (phosphoinositide binding specific) member 3 [Source:MGI Symbol;Acc:MGI:19325

ENSMUSG00000002741	114.544624	115.9030386	112.9599117	99.20270995	96.06089253	91.04331783	142.4992786	136.7813816	131.9987341	94.85689671	94.68038392	98.23915182	Ykt6	YKT6 v-SNARE homolog (S. cerevisiae) [Source:MGI Symbol;Acc:MGI:1927550]
ENSMUSG00000002748	75.41785355	93.78123562	73.96024099	100.9372483	113.4295874	131.5333286	50.02017168	46.29068489	58.04930382	62.43882359	65.9633651	62.28110641	Baz1b	bromodomain adjacent to zinc finger domain, 1B [Source:MGI Symbol;Acc:MGI:1353499]
ENSMUSG00000002763	45.5937633	36.21014905	44.58571946	40.54622261	34.1325962	31.49175807	20.43305233	24.61856132	22.58943758	22.28250685	22.77132512	23.28469401	Pex6	peroxisomal biogenesis factor 6 [Source:MGI Symbol;Acc:MGI:2385054]
ENSMUSG00000002767	116.8167678	97.00348557	142.7340985	117.4475532	101.8266488	89.55847658	182.4312511	188.3562356	144.4064222	87.10987205	97.58021873	94.21376588	Mrpl2	mitochondrial ribosomal protein L2 [Source:MGI Symbol;Acc:MGI:1351622]
ENSMUSG00000002768	96.52018718	76.62575367	115.1858017	92.70406768	84.42591692	63.04073915	47.12641934	49.20606736	38.29836894	41.64271771	37.38264053	40.78970543	Mea1	male enhanced antigen 1 [Source:MGI Symbol;Acc:MGI:96957]
ENSMUSG00000002769	1.636666408	1.884705502	1.026984974	0.985361419	1.608639549	1.528113846	8.012914346	6.64231001	6.093367839	3.022367911	3.559714005	3.789896049	Gnmt	glycine N-methyltransferase [Source:MGI Symbol;Acc:MGI:1202304]
ENSMUSG00000002771	2.22046882	2.111738021	2.363779279	2.041178077	2.007968954	1.884821726	0.848184198	0.787948918	0.812293446	1.096832931	0.845995518	0.675525141	Grin2d	glutamate receptor, ionotropic, NMDA2D (epsilon 4) [Source:MGI Symbol;Acc:MGI:95823]
ENSMUSG00000002778	242.1035933	196.987175	240.4572484	194.1732696	180.9216572	148.3641149	84.00198295	94.21020689	80.64638528	69.7523848	68.13259011	67.09601998	Kdelr1	KDEL (Lys-Asp-Glu-Leu) endoplasmic reticulum protein retention receptor 1 [Source:MGI Symbol;Acc:MGI:1915387]
ENSMUSG00000002781	8.863091454	10.45796774	8.079560561	9.834429673	9.669273507	9.062793517	12.3786014	10.70457236	12.65661673	9.122429953	11.11697602	10.22185651	Tmem143	transmembrane protein 143 [Source:MGI Symbol;Acc:MGI:1917459]
ENSMUSG00000002791	0.302484976	0.08708175	0.348392137	0.317558261	0.079452229	0.230397333	0.067512925	0.337111274	0.035192576	0.372027794	0.062947057	0.201052222	Pdxk-ps	pyridoxal (pyridoxine, vitamin B6) kinase, pseudogene [Source:MGI Symbol;Acc:MGI:1933201]
ENSMUSG00000002797	15.50310794	13.13509251	19.81940068	17.06585275	17.18872589	13.21565985	14.24720163	14.37660825	11.9873316	10.51413643	11.18859458	9.895097234	Ggct	gamma-glutamyl cyclotransferase [Source:MGI Symbol;Acc:MGI:95700]
ENSMUSG00000002799	5.335684939	6.407643296	4.175013969	5.37039429	6.687147178	5.399440069	56.24424604	53.00858297	61.82964668	36.30618617	38.7989714	39.51771345	Jag2	jagged 2 [Source:MGI Symbol;Acc:MGI:1098270]
ENSMUSG00000002803	11.52752592	11.74117612	11.37334842	10.36676881	11.03226867	10.89569563	14.7070131	15.18852044	14.02251459	7.010186625	7.505393005	6.675787028	Btbd6	BTB (POZ) domain containing 6 [Source:MGI Symbol;Acc:MGI:3026623]
ENSMUSG00000002804	14.94561815	9.432169847	17.69713389	10.07559456	8.15873236	7.08955947	42.68844723	52.26803575	34.05910537	20.07654117	18.81603471	19.74992221	Nudt14	nudix (nucleoside diphosphate linked moiety X)-type motif 14 [Source:MGI Symbol;Acc:MGI:1913424]
ENSMUSG00000002808	18.03136446	20.07649199	14.99651071	19.05525821	19.11211978	18.55476469	17.73266622	16.67967953	16.93104741	13.74315913	13.3526372	13.86330392	Epdr1	ependymin related protein 1 (zebrafish) [Source:MGI Symbol;Acc:MGI:2145369]
ENSMUSG00000002812	106.0815515	116.7151706	109.6906896	118.8029664	123.7058511	122.2370597	70.51248514	63.17252795	72.47243265	82.20332631	88.92218952	87.76661219	Flii	flightless I actin binding protein [Source:MGI Symbol;Acc:MGI:1342286]
ENSMUSG00000002814	8.922610584	10.12278574	9.810847479	11.23100345	12.14936394	12.68718116	13.37193956	13.82863976	15.01184768	21.80028608	23.08233536	21.78306655	Top3a	topoisomerase (DNA) III alpha [Source:MGI Symbol;Acc:MGI:1197527]
ENSMUSG00000002820	4.129413419	5.420964281	5.263440643	4.855410947	4.468768512	4.434872274	17.27193775	17.50850558	18.75622451	11.78278433	11.85875436	11.67408502	Atg4d	autophagy related 4D, cysteine peptidase [Source:MGI Symbol;Acc:MGI:2444308]
ENSMUSG00000002825	19.68397918	15.98628032	21.28367144	17.20863336	12.26357271	10.25832107	103.4056345	103.9304377	81.57684315	57.08327597	57.60595382	58.14808901	Qtrt1	queuine tRNA-ribosyltransferase catalytic subunit 1 [Source:MGI Symbol;Acc:MGI:1931441]
ENSMUSG00000002831	0.115629124	0.183085211	0.073987656	0.242782197	0.189823305	0.192658838	0.058067465	0.021477585	0	0.045249541	0.05414039	0.096068789	Plin4	perilipin 4 [Source:MGI Symbol;Acc:MGI:1929709]
ENSMUSG00000002833	127.7507022	104.6000387	139.1331886	118.9526294	106.7654408	91.04489161	152.7500034	167.0585264	148.8847641	108.1747496	115.8420087	112.2508198	Hdgfl2	HDGF like 2 [Source:MGI Symbol;Acc:MGI:1194492]
ENSMUSG00000002835	8.202507781	10.29752452	5.794106409	11.95198083	12.39755302	12.12209102	23.38080093	21.76639032	25.73543156	21.44010506	23.43915449	23.2583674	Chaf1a	chromatin assembly factor 1, subunit A (p150) [Source:MGI Symbol;Acc:MGI:1351331]
ENSMUSG00000002844	53.91551178	48.01088777	52.69044302	46.41086282	40.55490318	36.56216218	98.8757256	99.73811058	85.37217061	54.06906374	55.01257873	57.45152978	Adprh	ADP-ribosylarginine hydrolase [Source:MGI Symbol;Acc:MGI:1098234]
ENSMUSG00000002845	41.05268992	41.45979879	36.59033843	36.13813005	36.62796405	37.99110964	83.72870234	82.51146233	87.04560484	67.78594894	67.97896775	67.31625011	Tmem39a	transmembrane protein 39a [Source:MGI Symbol;Acc:MGI:1915096]
ENSMUSG00000002846	35.76790818	30.53939951	40.17909498	34.87575606	29.87121568	26.37057078	31.45514694	34.86536921	33.54027521	30.83139047	28.72348007	27.05878406	Timmdc1	translocase of inner mitochondrial membrane domain containing 1 [Source:MGI Symbol;Acc:MGI:1922139]
ENSMUSG00000002847	5.905164857	8.375734022	7.925718655	3.864142218	3.291316901	3.949338052	2.668144676	2.924953566	2.446517206	7.923045797	6.623874043	8.520071592	Pla1a	phospholipase A1 member A [Source:MGI Symbol;Acc:MGI:1934677]
ENSMUSG00000002870	66.3987993	70.21683047	54.92486109	85.36755903	87.16082382	97.16648879	319.6242659	303.3694052	348.7551019	232.833429	232.3126072	238.6953914	Mcm2	minichromosome maintenance complex component 2 [Source:MGI Symbol;Acc:MGI:105380]
ENSMUSG00000002871	40.87227185	37.79667951	46.79938384	38.14133251	35.24033724	30.94918653	24.29803687	24.35084618	25.91153345	19.45828144	21.97672426	18.64180975	Tpra1	transmembrane protein, adipocyte asscociated 1 [Source:MGI Symbol;Acc:MGI:1345190]
ENSMUSG00000002881	29.02124866	36.84709656	24.84941275	41.80781746	48.28965846	56.11259078	6.291751277	7.371713234	7.822418202	17.8540312	17.36189335	18.11603916	Nab1	Ngfi-A binding protein 1 [Source:MGI Symbol;Acc:MGI:107564]
ENSMUSG00000002885	9.133862563	9.485830694	7.24282492	3.436558914	3.920090668	4.797998517	35.01894636	28.72924532	37.50097916	31.74823803	31.54143764	32.7221547	Adgre5	adhesion G protein-coupled receptor E5 [Source:MGI Symbol;Acc:MGI:1347095]
ENSMUSG00000002897	2.898191816	3.126047124	2.670432769	1.49651489	1.695058187	1.582041858	2.656438045	2.344708731	2.949286417	3.663874661	3.867965223	3.767058389	Il17ra	interleukin 17 receptor A [Source:MGI Symbol;Acc:MGI:107399]
ENSMUSG00000002900	257.7654077	286.4732514	245.5716485	288.7239135	309.017344	337.3705396	28.14848109	23.98869652	30.93665525	120.2959461	130.1993454	123.5629328	Lamb1	laminin B1 [Source:MGI Symbol;Acc:MGI:96743]
ENSMUSG00000002908	0.70937215	0.606824026	0.373500002	0.35935755	0.31941817	0.15437598	1.863744686	1.244843409	1.792119357	1.722259447	1.602733476	2.406881408	Kcnn1	potassium intermediate/small conductance calcium-activated channel, subfamily N, member 1 [Source:MGI Symbol;Acc:MGI:193399
ENSMUSG00000002910	3.654065691	2.790917258	1.985023406	2.540038026	1.997941279	3.038839002	20.43909913	19.76154984	24.81381572	6.136729473	6.517802297	5.782719515	Arrdc2	arrestin domain containing 2 [Source:MGI Symbol;Acc:MGI:1918057]
ENSMUSG00000002930	0	0	0	0.045706862	0	0	0	0	0	0.021905502	0.061155735	0	Ppp1r17	protein phosphatase 1, regulatory subunit 17 [Source:MGI Symbol;Acc:MGI:1333876]
ENSMUSG00000002944	12.23510168	14.62213902	10.63148691	0.639641224	0.396090404	1.057226472	0.7190355	0.899709251	1.100514319	30.14967338	30.22539193	29.40075806	Cd36	CD36 molecule [Source:MGI Symbol;Acc:MGI:107899]
ENSMUSG00000002948	29.28219576	25.23769783	31.38623251	29.1167172	28.69461074	22.32347397	29.52316995	29.18804279	24.50264545	24.12747741	22.15076205	20.96275411	Map2k7	mitogen-activated protein kinase kinase 7 [Source:MGI Symbol;Acc:MGI:1346871]
ENSMUSG00000002949	60.21670783	53.06335021	60.38400902	64.59159092	60.62946823	52.83854674	108.5547223	110.5440521	103.142743	74.17743609	75.23472946	69.62507014	Timm44	translocase of inner mitochondrial membrane 44 [Source:MGI Symbol;Acc:MGI:1343262]
ENSMUSG00000002957	86.83689078	89.11026948	79.36026506	84.46076372	84.32090854	82.12340803	77.8147044	72.94635564	83.09048103	72.96038895	72.31461994	69.85975112	Ap2a2	adaptor-related protein complex 2, alpha 2 subunit [Source:MGI Symbol;Acc:MGI:101920]
ENSMUSG00000002963	42.557373	34.58520023	42.96702141	42.60811562	40.01101168	37.02028973	33.08724657	33.25282827	26.27312039	29.14690884	29.01951225	30.16535435	Pnkp	polynucleotide kinase 3'- phosphatase [Source:MGI Symbol;Acc:MGI:1891698]
ENSMUSG00000002968	59.77822931	52.71877625	62.32151214	52.93962655	53.54107579	47.38032419	103.1441474	96.77298711	101.9132467	75.89064621	78.90058608	77.76070317	Med25	mediator complex subunit 25 [Source:MGI Symbol;Acc:MGI:1922863]
ENSMUSG00000002980	4.597233001	2.554098743	1.85787372	1.956870522	1.101608507	3.96236988	214.3599261	227.9504095	210.4551008	60.56353846	62.10048701	65.07968082	Bcam	basal cell adhesion molecule [Source:MGI Symbol;Acc:MGI:1929940]
ENSMUSG00000002981	98.35815145	100.6041089	100.3835076	100.0608405	91.27826039	90.27983648	73.8553597	68.31813946	75.59009004	78.74101518	87.27030231	83.87422097	Clptm1	cleft lip and palate associated transmembrane protein 1 [Source:MGI Symbol;Acc:MGI:1927155]
ENSMUSG00000002983	26.41689663	24.34612878	27.63336901	31.71787325	28.29151311	23.99324002	9.874307522	10.76708264	10.90809812	8.694513412	10.33962199	10.27307615	Relb	avian reticuloendotheliosis viral (v-rel) oncogene related B [Source:MGI Symbol;Acc:MGI:103289]
ENSMUSG00000002984	87.28441271	66.62878606	104.9644345	71.811727	56.134259	45.78494325	161.9390045	176.9884643	152.358143	91.21831016	95.95630427	92.12933946	Tomm40	translocase of outer mitochondrial membrane 40 [Source:MGI Symbol;Acc:MGI:1858259]
ENSMUSG00000002985	265.6476838	206.4087993	302.0798146	28.46322479	26.32383094	26.89053707	987.2042821	1098.657925	816.191569	369.2010215	376.9633408	368.8667397	Apoe	apolipoprotein E [Source:MGI Symbol;Acc:MGI:88057]
ENSMUSG00000002992	0	0.154359862	0	0	0	0	0	0	0	0	0	0	Apoc2	apolipoprotein C-II [Source:MGI Symbol;Acc:MGI:88054]
ENSMUSG00000002996	11.22085654	13.51668765	5.915143136	13.02505041	15.78297475	24.36626835	9.773390435	9.489146308	10.50051959	19.3391831	19.51576749	20.42737933	Hbp1	high mobility group box transcription factor 1 [Source:MGI Symbol;Acc:MGI:894659]
ENSMUSG00000002997	19.40469447	23.8012363	16.92201752	25.36319569	28.4399006	31.63060089	2.130239796	1.330590178	2.117913265	7.533634255	7.35278494	7.00200233	Prkar2b	protein kinase, cAMP dependent regulatory, type II beta [Source:MGI Symbol;Acc:MGI:97760]
ENSMUSG00000003031	20.63172236	27.25991966	18.1918634	29.44034724	33.36470513	36.60387	16.55241902	11.60786614	15.1582104	9.605407671	10.24750315	9.385347793	Cdkn1b	cyclin-dependent kinase inhibitor 1B [Source:MGI Symbol;Acc:MGI:104565]
ENSMUSG00000003032	53.30263696	68.76259311	44.14215275	89.15710997	99.45830037	109.6926405	90.20957568	79.01730271	103.5556632	110.1750765	111.1297749	101.8174496	Klf4	Kruppel-like factor 4 (gut) [Source:MGI Symbol;Acc:MGI:1342287]
ENSMUSG00000003033	73.87721808	76.91096509	72.51756732	84.73222295	75.31223335	71.70742798	78.3449112	71.55406221	76.98543396	66.57257292	72.25701445	72.59407958	Ap1m1	adaptor-related protein complex AP-1, mu subunit 1 [Source:MGI Symbol;Acc:MGI:102776]
ENSMUSG00000003037	60.12069047	56.16526813	66.21260653	58.3504556	52.22124308	44.08605103	70.16829577	72.35767166	63.2798782	56.18603401	55.6203335	55.20292518	Rab8a	RAB8A, member RAS oncogene family [Source:MGI Symbol;Acc:MGI:96960]
ENSMUSG00000003038	23.72083303	22.98450725	24.13118601	28.73454008	27.8205036	21.92574341	76.02380211	78.94227505	62.40759392	59.8851225	48.79944922	58.88822694	Hmgn2	high mobility group nucleosomal binding domain 2 [Source:MGI Symbol;Acc:MGI:96136]
ENSMUSG00000003039	55.52062946	52.62971065	58.82514862	54.67133474	48.42880339	42.96083821	40.45435237	43.84331983	37.14235827	43.33776052	40.99202886	41.68223755	Fam32a	family with sequence similarity 32, member A [Source:MGI Symbol;Acc:MGI:1915172]
ENSMUSG00000003051	0.398939622	0.164071244	0.058347351	0.319100486	0.059878567	0.347274391	24.9823753	26.25300554	23.8438326	15.7265399	15.06206353	15.50569767	Elf3	E74-like factor 3 [Source:MGI Symbol;Acc:MGI:1101781]
ENSMUSG00000003053	0	0	0	0	0.063943873	0	0.036223342	0.060291157	0.018882203	0	0	0	Cyp2c29	cytochrome P450, family 2, subfamily c, polypeptide 29 [Source:MGI Symbol;Acc:MGI:103238]
ENSMUSG00000003062	37.34609974	33.89035572	38.53731201	42.82015919	40.27336451	31.39801214	52.72512032	57.91909077	52.09446885	37.78284332	36.19965125	37.6380502	Stard3nl	STARD3 N-terminal like [Source:MGI Symbol;Acc:MGI:1923455]
ENSMUSG00000003068	102.7312525	97.70566352	112.3966877	100.6315556	98.91257581	85.6440457	91.18917006	90.66032491	85.28985972	73.80819889	79.27370176	76.08272959	Stk11	serine/threonine kinase 11 [Source:MGI Symbol;Acc:MGI:1341870]
ENSMUSG00000003070	11.99386287	12.09091888	10.89419796	12.2288161	10.33151151	10.55043555	5.155630345	5.900809513	6.709313512	4.027600423	4.85738011	4.580958987	Efna2	ephrin A2 [Source:MGI Symbol;Acc:MGI:102707]
ENSMUSG00000003072	302.44849	233.7110286	377.9497862	269.4985675	224.8197453	162.6890574	279.0171983	303.2024762	230.9942053	177.6841851	185.9681012	180.5873594	Atp5d	ATP synthase, H+ transporting, mitochondrial F1 complex, delta subunit [Source:MGI Symbol;Acc:MGI:1913293]
ENSMUSG00000003099	108.3252234	107.145039	97.7280644	112.5798444	114.8692668	111.8758041	186.5992561	172.9434218	185.3208986	144.6895745	158.7072531	156.6745628	Ppp5c	protein phosphatase 5, catalytic subunit [Source:MGI Symbol;Acc:MGI:102666]
ENSMUSG00000003119	6.731415493	8.804415924	6.482057532	8.851895512	10.61464898	15.25101837	10.36639887	10.4271994	14.93466368	56.43879478	57.8587614	56.72839287	Cdk12	cyclin-dependent kinase 12 [Source:MGI Symbol;Acc:MGI:1098802]
ENSMUSG00000003123	5.549308946	5.18598601	8.412756114	5.138689128	3.430988446	3.202620072	4.516031952	3.890454354	2.781191078	2.462771658	2.593880863	2.837269694	Lipe	lipase, hormone sensitive [Source:MGI Symbol;Acc:MGI:96790]
ENSMUSG00000003131	146.824411	162.0515959	129.6587686	150.3063136	171.7223249	188.5322398	48.77656184	43.40977134	55.14814198	84.46707716	85.7409341	88.36314466	Pafah1b2	platelet-activating factor acetylhydrolase, isoform 1b, subunit 2 [Source:MGI Symbol;Acc:MGI:108415]
ENSMUSG00000003134	2.802094893	3.543493127	2.798365074	3.368849519	4.063872005	5.211370441	7.750499923	6.454333311	8.928256712	8.318813347	6.453619992	7.55650049	Tbc1d8	TBC1 domain family, member 8 [Source:MGI Symbol;Acc:MGI:1927225]
ENSMUSG00000003135	20.11912229	23.92920294	21.70663352	25.43853011	25.99864042	25.48935137	26.09063468	24.65898015	27.08101756	31.70659687	35.51127074	35.49743943	Cnot11	CCR4-NOT transcription complex, subunit 11 [Source:MGI Symbol;Acc:MGI:106580]

ENSMUSG00000003153	8.685333865	2.923106185	2.03827647	2.740376162	2.483973536	9.705204492	674.3817615	615.4491972	773.3493475	490.793917	505.9314921	487.104299	Slc2a3	solute carrier family 2 (facilitated glucose transporter), member 3 [Source:MGI Symbol;Acc:MGI:95757]
ENSMUSG00000003154	7.979518552	9.118682094	7.421033088	8.401308075	9.010944541	10.7452763	4.459255427	4.477403595	4.932440802	3.726750991	4.096834002	4.394137092	Foxj2	forkhead box J2 [Source:MGI Symbol;Acc:MGI:1926805]
ENSMUSG00000003161	134.8690497	134.2049634	148.0654984	143.0569251	145.3255254	129.8753498	31.88277362	32.02978677	28.37490492	37.6153647	41.67518224	41.0353282	Sri	sorcin [Source:MGI Symbol;Acc:MGI:98419]
ENSMUSG00000003166	16.55327307	14.59941085	16.9813216	13.63908894	14.47859574	13.56254115	16.83632385	16.100524	16.58868194	13.96981699	14.86344716	14.89196511	Dgcr2	DiGeorge syndrome critical region gene 2 [Source:MGI Symbol;Acc:MGI:892866]
ENSMUSG00000003184	63.30864509	48.41262425	73.95387318	57.61985682	47.11000669	34.84197987	32.13479667	42.09608095	31.62581181	24.72649465	27.05098626	28.03461157	Irf3	interferon regulatory factor 3 [Source:MGI Symbol;Acc:MGI:1859179]
ENSMUSG00000003190	5.623041023	6.306301988	6.60783753	7.118116525	4.829070369	6.554804127	5.500308651	6.539192545	6.872070318	4.329896217	7.163375096	5.459938626	Bcl2l12	BCL2-like 12 (proline rich) [Source:MGI Symbol;Acc:MGI:1922986]
ENSMUSG00000003198	2.953982208	2.704936337	3.206449973	5.048245352	5.34722045	5.150590505	4.035307252	4.442396038	5.068254907	6.526081974	7.702553866	5.519651216	Zfp959	zinc finger protein 959 [Source:MGI Symbol;Acc:MGI:2385058]
ENSMUSG00000003199	92.96126024	81.82532236	102.4099112	88.16890692	76.51302781	59.193575	88.24042411	94.37126549	77.45896068	52.16277391	55.2134069	49.3141862	Mpnd	MPN domain containing [Source:MGI Symbol;Acc:MGI:1915297]
ENSMUSG00000003200	53.72639627	56.05004841	56.60107927	56.73060171	50.45497181	47.77790715	44.47664756	41.06159641	39.00199481	36.86370357	36.52335513	38.46525569	Sh3gl1	SH3-domain GRB2-like 1 [Source:MGI Symbol;Acc:MGI:700010]
ENSMUSG00000003206	0.820300461	1.245178469	0.64894996	0	0.039175319	0	0.366172786	0.073874953	0.416455648	1.167312672	0.713854537	0.925235122	Ebi3	Epstein-Barr virus induced gene 3 [Source:MGI Symbol;Acc:MGI:1354171]
ENSMUSG00000003208	5.601202936	5.91031804	7.3660353	6.604765962	6.599035576	4.980365881	6.423398802	6.338157093	5.97683416	4.73762549	4.974566039	4.008524295	Yju2	YJU2 splicing factor [Source:MGI Symbol;Acc:MGI:1920136]
ENSMUSG00000003226	14.85909604	21.88869309	11.12838258	19.26210684	24.45825251	33.0528572	25.71884536	23.58183895	33.35681948	65.50678744	64.40630554	70.20147964	Ranbp2	RAN binding protein 2 [Source:MGI Symbol;Acc:MGI:894323]
ENSMUSG00000003227	0	0	0	0	0	0	0.010562236	0.011720057	0	0.021164678	0.068935422	0.010484714	Edar	ectodysplasin-A receptor [Source:MGI Symbol;Acc:MGI:1343498]
ENSMUSG00000003228	10.01896941	10.98341229	9.866753032	9.380496364	10.45681997	11.0678651	3.361669959	3.746604964	4.214887574	7.344465441	7.221366953	6.865098878	Grk5	G protein-coupled receptor kinase 5 [Source:MGI Symbol;Acc:MGI:109161]
ENSMUSG00000003233	13.99633697	16.43782842	12.03605203	12.88354188	15.78214856	18.80129255	9.123364458	8.861651223	11.80080477	14.77183602	16.18401179	15.92006603	Dvl3	dishevelled segment polarity protein 3 [Source:MGI Symbol;Acc:MGI:108100]
ENSMUSG00000003234	51.89365366	54.47956793	58.26408167	49.5966073	47.8890126	47.03648389	51.61875366	52.69325897	50.67525806	52.026447	52.4474247	49.66858741	Abcf3	ATP-binding cassette, sub-family F (GCN20), member 3 [Source:MGI Symbol;Acc:MGI:1351656]
ENSMUSG00000003235	41.19928462	34.64253251	39.75938867	35.87289125	31.19344399	29.70882446	126.2991965	131.4459594	119.5712078	96.8114576	102.7421948	99.31015466	Eif2b5	eukaryotic translation initiation factor 2B, subunit 5 epsilon [Source:MGI Symbol;Acc:MGI:2446176]
ENSMUSG00000003269	51.56026387	43.98095875	57.92697467	57.16912485	48.86511146	40.25785212	34.84010029	37.8353981	29.3559994	27.412689	27.51002676	26.47739266	Cyth2	cytohesin 2 [Source:MGI Symbol;Acc:MGI:1334255]
ENSMUSG00000003271	0.313790249	0.180672801	0.281099135	0.402632868	0.247265187	0.20913231	1.785923024	2.098264153	1.861902213	2.771694935	2.187539341	1.703292917	Sult2b1	sulfotransferase family, cytosolic, 2B, member 1 [Source:MGI Symbol;Acc:MGI:1926342]
ENSMUSG00000003273	17.80303684	15.855845	17.67596539	10.2893287	12.65832914	8.930920624	6.194395219	4.635560937	6.207618486	5.147765502	5.103904756	4.766613086	Car11	carbonic anhydrase 11 [Source:MGI Symbol;Acc:MGI:1336193]
ENSMUSG00000003279	1.09337961	1.274437495	1.33781237	1.518042856	1.583061659	1.492959725	1.11899482	1.215239538	1.179011278	1.001855958	0.932326477	0.779910663	Dlgap1	DLG associated protein 1 [Source:MGI Symbol;Acc:MGI:1346065]
ENSMUSG00000003282	0.467324687	1.255679599	0.513883852	1.146780743	1.636664783	4.561468681	0.927148529	0.737293424	1.546548088	4.938721349	5.01378627	5.169263427	Plag1	pleiomorphic adenoma gene 1 [Source:MGI Symbol;Acc:MGI:1891916]
ENSMUSG00000003283	2.284152152	2.085297645	2.022484945	0.422466051	0.302686015	0.658301706	14.88093343	15.18711456	14.00092289	7.620286025	8.838597679	7.860010483	Hck	hemopoietic cell kinase [Source:MGI Symbol;Acc:MGI:96052]
ENSMUSG00000003299	77.46037502	54.21115081	78.79112826	62.86184004	52.42444527	41.40058632	139.2843018	146.5319838	123.7882865	74.61620542	78.82741517	76.70921525	Mrpl4	mitochondrial ribosomal protein L4 [Source:MGI Symbol;Acc:MGI:2137210]
ENSMUSG00000003308	10.09075157	9.90591684	10.3662777	8.108224406	8.874360159	9.412972942	6.768184265	6.841397117	5.912324461	8.499554923	7.895272632	8.850637975	Keap1	kelch-like ECH-associated protein 1 [Source:MGI Symbol;Acc:MGI:1858732]
ENSMUSG00000003309	0.937666805	1.199746141	1.093306452	1.118075049	1.012534975	1.051466764	9.464192256	9.056702508	8.581961041	12.02166504	12.05459005	9.579391916	Ap1m2	adaptor protein complex AP-1, mu 2 subunit [Source:MGI Symbol;Acc:MGI:1336974]
ENSMUSG00000003316	36.3149802	51.51414626	31.13373501	47.70034674	57.72091933	80.22209428	44.04257085	37.08360952	53.85600371	71.81315456	77.18518222	74.77348149	Glg1	golgi apparatus protein 1 [Source:MGI Symbol;Acc:MGI:104967]
ENSMUSG00000003341	0.515752423	0.322411232	0.467677999	0.330028585	0.665738935	0.527527044	0.302582755	0.423338898	0.329171108	1.00174159	1.128476899	1.057796329	Atp8b3	ATPase, class I, type 8B, member 3 [Source:MGI Symbol;Acc:MGI:1914581]
ENSMUSG00000003344	32.53907705	29.90346899	35.45608147	31.61405042	27.8205036	25.08102371	52.27722788	51.69107323	51.87361821	25.36113169	25.0799162	23.09974034	Btbd2	BTB (POZ) domain containing 2 [Source:MGI Symbol;Acc:MGI:1933831]
ENSMUSG00000003345	174.6374265	155.4662622	178.6269063	155.8739895	142.1852599	120.4014021	145.2832451	155.4080718	135.504648	107.6505526	107.4775564	109.811297	Csnk1g2	casein kinase 1, gamma 2 [Source:MGI Symbol;Acc:MGI:1920014]
ENSMUSG00000003346	126.3594872	96.63604363	130.0099548	103.9059295	83.5428573	74.87554032	112.6611071	125.8833514	106.4854633	74.38756074	79.83742127	75.85697707	Abhd17a	abhydrolase domain containing 17A [Source:MGI Symbol;Acc:MGI:106388]
ENSMUSG00000003348	36.11167963	36.79564622	41.40216414	39.59909057	36.06856957	34.03455989	39.77869504	43.55066885	44.86768849	42.16975408	43.62311757	40.76535373	Mob3a	MOB kinase activator 3A [Source:MGI Symbol;Acc:MGI:3050117]
ENSMUSG00000003352	76.97492865	80.9248476	84.00474718	76.10598354	77.60211337	70.3126343	32.71337938	35.3141147	36.20014548	25.26065433	24.44436392	24.00702941	Cacnb3	calcium channel, voltage-dependent, beta 3 subunit [Source:MGI Symbol;Acc:MGI:103307]
ENSMUSG00000003354	2.652202007	2.491540932	2.465211082	1.660849448	1.723269231	1.674584996	1.152757178	1.348262823	0.941950847	1.37345541	1.016699628	1.57727343	Ccdc65	coiled-coil domain containing 65 [Source:MGI Symbol;Acc:MGI:2146001]
ENSMUSG00000003355	26.98691161	20.0065339	32.25359907	24.99459421	19.40976252	14.0050492	19.12987349	19.27629183	16.57485073	15.22942411	15.04019536	16.0127406	Fkbp11	FK506 binding protein 11 [Source:MGI Symbol;Acc:MGI:1913370]
ENSMUSG00000003360	63.53922321	68.60268545	66.10343346	68.24720806	69.17541317	68.62410123	98.00376593	99.84825986	104.861345	93.41760666	100.15686	94.46461884	Ddx23	DEAD (Asp-Glu-Ala-Asp) box polypeptide 23 [Source:MGI Symbol;Acc:MGI:1921601]
ENSMUSG00000003363	261.2011022	223.0725175	261.8760024	166.5924976	148.5870986	132.7446751	93.2545703	97.00167756	92.66906288	114.7282136	110.2612062	114.7823449	Pld3	phospholipase D family, member 3 [Source:MGI Symbol;Acc:MGI:1333782]
ENSMUSG00000003378	45.83330778	38.41616767	49.90412936	43.00883626	35.98805392	33.95550385	11.85643644	12.06153616	12.91461528	8.039708531	7.870868932	7.87138477	Grik5	glutamate receptor, ionotropic, kainate 5 (gamma 2) [Source:MGI Symbol;Acc:MGI:95818]
ENSMUSG00000003379	1.00409649	1.088252917	1.421040329	0.744093275	1.303191057	1.349650816	3.032057802	3.013357799	2.583816227	1.056636002	1.671618073	1.256265906	Cd79a	CD79A antigen (immunoglobulin-associated alpha) [Source:MGI Symbol;Acc:MGI:101774]
ENSMUSG00000003380	260.2652339	218.3686618	303.3627672	210.4800298	172.4984064	146.3712898	134.6219665	141.7089391	100.3000874	103.4293138	102.8546557	102.7001238	Rabac1	Rab acceptor 1 (prenylated) [Source:MGI Symbol;Acc:MGI:1201692]
ENSMUSG00000003382	4.226499121	5.829970469	3.359286506	6.251185713	5.959017881	7.726194979	3.537996699	3.340555449	3.620835837	5.016281211	5.333945334	5.010280253	Etv3	ets variant 3 [Source:MGI Symbol;Acc:MGI:1350926]
ENSMUSG00000003402	191.4441918	165.1279988	222.2872098	148.2052931	131.1117298	105.7297626	113.9087681	117.7171475	106.6255313	89.51744057	90.17496019	94.87244262	Prkcsh	protein kinase C substrate 80K-H [Source:MGI Symbol;Acc:MGI:107877]
ENSMUSG00000003410	0.259760047	0.158361378	0.172079102	0.213885713	0.192649084	0.244408535	9.358164024	10.30813047	9.770513884	4.100280508	3.713965781	3.750999502	Elavl3	ELAV like RNA binding protein 3 [Source:MGI Symbol;Acc:MGI:109157]
ENSMUSG00000003411	12.50250699	11.50436819	13.57754351	14.06598409	12.24583776	10.21267853	1.721235824	1.591596486	1.903218334	2.168704147	2.796293741	2.433464386	Rab3b	RAB3B, member RAS oncogene family [Source:MGI Symbol;Acc:MGI:1917158]
ENSMUSG00000003418	0.061265051	0.082308165	0.073176495	0.066700129	0.064368749	0.015554827	0.528728414	0.475418804	0.465687808	2.575606672	2.447854803	2.452305926	St8sia6	ST8 alpha-N-acetyl-neuraminide alpha-2,8-sialyltransferase 6 [Source:MGI Symbol;Acc:MGI:2386797]
ENSMUSG00000003420	79.82096293	64.58912906	93.1694451	60.48238835	50.93033739	39.42382193	14.54939401	15.97954484	13.19493784	14.57708485	14.18832748	13.88995659	Fcgrt	Fc receptor, IgG, alpha chain transporter [Source:MGI Symbol;Acc:MGI:103017]
ENSMUSG00000003421	66.20200023	68.98069822	85.45560692	68.75955028	58.20940336	55.22315373	94.00340761	101.7448339	94.0762422	75.56722286	76.92434825	69.58632019	Nosip	nitric oxide synthase interacting protein [Source:MGI Symbol;Acc:MGI:1913644]
ENSMUSG00000003423	74.95080254	67.16224905	102.2038194	74.32539039	65.81210945	43.79537271	61.24846202	65.32191115	53.16259052	39.68007152	36.66211148	42.80109214	Pih1d1	PIH1 domain containing 1 [Source:MGI Symbol;Acc:MGI:1916095]
ENSMUSG00000003429	377.8210321	241.6592633	398.6353907	324.7194295	238.2623659	192.1833876	454.0069332	564.8529156	493.460056	191.8577832	265.4782018	239.7543308	Rps11	ribosomal protein S11 [Source:MGI Symbol;Acc:MGI:1351329]
ENSMUSG00000003435	87.04546794	93.60840925	75.78177635	94.16171424	90.35554438	91.02910417	97.59449413	84.29165379	102.3598584	106.0152767	110.7419069	107.4763895	Supt5	suppressor of Ty 5, DSIF elongation factor subunit [Source:MGI Symbol;Acc:MGI:1202400]
ENSMUSG00000003436	0.280824068	0.323383351	0.063890138	0.029117823	0.163917035	0.071299537	3.537843703	3.616551255	2.759006087	7.340344565	8.051654386	7.300280521	Dll3	delta like canonical Notch ligand 3 [Source:MGI Symbol;Acc:MGI:1096877]
ENSMUSG00000003437	106.7616756	96.16108961	119.1972517	89.41817812	82.27192256	77.73085852	127.5530844	131.1340218	124.2117356	108.3104915	103.6524857	108.137439	Paf1	Paf1, RNA polymerase II complex component [Source:MGI Symbol;Acc:MGI:1923988]
ENSMUSG00000003438	104.2354406	81.83070788	121.3160119	101.3832477	83.21417346	72.54107555	180.2164847	190.148874	167.0328766	103.7477899	107.8072667	110.330481	Timm50	translocase of inner mitochondrial membrane 50 [Source:MGI Symbol;Acc:MGI:1913775]
ENSMUSG00000003444	10.54718593	8.238912434	11.34835485	9.717078945	7.446491157	6.268255313	7.257138708	10.08246046	7.94104956	8.352579743	8.108426143	9.01972687	Med29	mediator complex subunit 29 [Source:MGI Symbol;Acc:MGI:1914474]
ENSMUSG00000003452	0.668692235	0.459881108	0.789194208	0.788682599	0.868452479	1.37236348	1.193986399	1.462877139	2.238879828	3.131872075	2.937710486	3.102979725	Bicd1	BICD cargo adaptor 1 [Source:MGI Symbol;Acc:MGI:1101760]
ENSMUSG00000003458	60.99387052	60.61709267	54.82110747	47.62389232	52.0054587	49.41804734	24.28716301	23.28399469	25.86191829	40.00750209	39.63295145	39.34969289	Ncstn	nicastrin [Source:MGI Symbol;Acc:MGI:1891700]
ENSMUSG00000003464	49.2826285	53.45915348	44.85719165	46.82349076	50.76548705	47.50055317	49.39052666	52.50343201	54.88463456	52.39350239	55.89693441	52.70195465	Pex19	peroxisomal biogenesis factor 19 [Source:MGI Symbol;Acc:MGI:1334458]
ENSMUSG00000003469	0.134632315	0.124028825	0.248104037	0.025127333	0.198033935	0.123056398	1.370241979	1.333724897	1.979901734	2.023147168	2.398251362	2.529466769	Phyhip	phytanoyl-CoA hydroxylase interacting protein [Source:MGI Symbol;Acc:MGI:1860417]
ENSMUSG00000003476	0.026792334	0	0	0	0	0	0.287035024	0.318499485	0.473806898	0.671023075	0.825170855	0.546112583	Crhr2	corticotropin releasing hormone receptor 2 [Source:MGI Symbol;Acc:MGI:894312]
ENSMUSG00000003477	0	0.248244066	0	0.080467891	0	0.0656794	0	0	0	0	0	0	Inmt	indolethylamine N-methyltransferase [Source:MGI Symbol;Acc:MGI:102963]
ENSMUSG00000003484	0.045245518	0.364717781	0.092644059	0	0	0	0	0.044822132	0	0.161884191	0.376623296	0.080195383	Cyp4f18	cytochrome P450, family 4, subfamily f, polypeptide 18 [Source:MGI Symbol;Acc:MGI:1919304]
ENSMUSG00000003500	65.8004052	61.93689453	69.77679182	70.19367301	62.97676188	57.08957202	53.7521279	55.4825244	53.32415013	58.23459091	55.70771491	60.89881794	Impdh1	inosine monophosphate dehydrogenase 1 [Source:MGI Symbol;Acc:MGI:96567]
ENSMUSG00000003505	0	0	0	0	0	0	0	0	0	0	0	0	Psg18	pregnancy specific glycoprotein 18 [Source:MGI Symbol;Acc:MGI:1347251]
ENSMUSG00000003518	13.68194491	20.39719208	10.93757316	16.91155616	21.89831719	24.39954697	4.417854505	3.538627113	4.727813013	12.37008516	11.15697035	11.90745691	Dusp3	dual specificity phosphatase 3 (vaccinia virus phosphatase VH1-related) [Source:MGI Symbol;Acc:MGI:1919599]
ENSMUSG00000003526	0.553065396	0.382129941	0.226489718	0.206444616	0.34865027	0.547637877	11.01094146	11.58787505	12.74057198	7.865792613	9.276478675	8.136326707	Prodh	proline dehydrogenase [Source:MGI Symbol;Acc:MGI:97770]
ENSMUSG00000003527	15.37695176	15.93661537	15.46662076	16.81662896	14.45532436	15.02056996	21.70017775	21.56680821	21.11682535	19.08713567	20.16523773	19.50874622	Ess2	ess-2 splicing factor [Source:MGI Symbol;Acc:MGI:107854]
ENSMUSG00000003528	140.695517	123.4340005	143.5738427	113.5701917	104.5943933	84.59188048	130.4741197	137.3611637	123.9864857	82.16839211	80.19589161	79.06372665	Slc25a1	solute carrier family 25 (mitochondrial carrier, citrate transporter), member 1 [Source:MGI Symbol;Acc:MGI:1345283]

ENSMUSG00000003531	35.7312589	25.30599044	41.56196705	29.43022937	23.00072337	18.78277206	38.37741269	39.01137523	27.16784548	21.53225409	24.12228163	23.48927462	Dgcr6	DiGeorge syndrome critical region gene 6 [Source:MGI Symbol;Acc:MGI:1202877]
ENSMUSG00000003534	66.39220183	69.73877002	65.05435369	60.4063285	58.19579577	57.77139866	13.2598641	11.92775914	13.31458634	20.10489527	20.64183087	21.9264202	Ddr1	discoidin domain receptor family, member 1 [Source:MGI Symbol;Acc:MGI:99216]
ENSMUSG00000003541	222.979635	212.0859462	210.8743436	259.7372625	245.8277258	216.3780832	35.25607297	36.55797725	36.33106053	76.60160239	77.52405939	79.5025546	Ier3	immediate early response 3 [Source:MGI Symbol;Acc:MGI:104814]
ENSMUSG00000003545	24.18543138	29.22966839	22.13389819	33.87872946	34.09287861	40.01036403	3.99420501	4.21044306	4.6172875	0.588501015	0.985785564	1.177805313	Fosb	FBJ osteosarcoma oncogene B [Source:MGI Symbol;Acc:MGI:95575]
ENSMUSG00000003546	19.02683349	17.29316572	20.77183325	15.67683237	15.40749417	12.93711341	13.9373996	15.07903154	14.21934931	13.06731214	13.5356268	13.50608919	Klc4	kinesin light chain 4 [Source:MGI Symbol;Acc:MGI:1922014]
ENSMUSG00000003549	107.1403602	89.70513109	113.5712886	131.611199	108.2826744	90.53330934	27.64433424	31.47142026	27.48366339	55.34250972	52.02771765	50.19899069	Ercc1	excision repair cross-complementing rodent repair deficiency, complementation group 1 [Source:MGI Symbol;Acc:MGI:95412]
ENSMUSG00000003555	0	0	0.068245159	0	0	0.152319228	0	0	0	0.089437567	0	0	Cyp17a1	cytochrome P450, family 17, subfamily a, polypeptide 1 [Source:MGI Symbol;Acc:MGI:88586]
ENSMUSG00000003559	12.20822932	11.22492693	12.17573191	9.420170753	10.53976923	8.121421536	14.61679565	16.62533063	17.61692882	10.46850112	11.47505468	11.35041005	As3mt	arsenic (+3 oxidation state) methyltransferase [Source:MGI Symbol;Acc:MGI:1929882]
ENSMUSG00000003573	32.37146794	40.89519793	27.81251587	43.43013959	41.61624724	45.37219464	16.25138064	15.65287034	15.42336687	18.01798855	20.0748844	19.68061867	Homer3	homer scaffolding protein 3 [Source:MGI Symbol;Acc:MGI:1347359]
ENSMUSG00000003575	5.750795375	5.872638024	5.714819793	5.996582708	6.016797763	6.336249437	5.123415673	5.004267354	5.902452704	6.524298026	6.432787106	6.688483703	Crtc1	CREB regulated transcription coactivator 1 [Source:MGI Symbol;Acc:MGI:2142523]
ENSMUSG00000003581	35.05515782	30.92227493	30.72867293	35.40690058	27.49089433	24.88591769	11.04580469	14.39019566	13.34971717	16.27233509	17.69864773	16.12221849	Rnf215	ring finger protein 215 [Source:MGI Symbol;Acc:MGI:1918923]
ENSMUSG00000003585	2.002988627	3.212686799	3.002730271	2.403200668	2.405097568	2.342947444	6.290711764	6.767694855	6.791377316	9.917929821	8.366202834	8.051336485	Sec14l2	SEC14-like lipid binding 2 [Source:MGI Symbol;Acc:MGI:1915065]
ENSMUSG00000003604	8.744431332	8.568895228	9.533535338	8.395548859	6.139673913	5.571743616	21.54384586	22.40616127	18.82134038	11.28127984	11.23323598	9.407482593	Aven	apoptosis, caspase activation inhibitor [Source:MGI Symbol;Acc:MGI:1921518]
ENSMUSG00000003617	233.067436	258.3618376	216.9345016	279.0726167	307.8003669	335.2947314	0.379218698	0.441827642	0.632563104	1.9946873	2.174469735	2.315077629	Cp	ceruloplasmin [Source:MGI Symbol;Acc:MGI:88476]
ENSMUSG00000003623	17.3338417	25.0721871	14.63185011	24.53129326	25.10679841	27.03237362	9.941060502	10.32951845	12.42966108	20.99810349	19.22230712	20.26181463	Crot	carnitine O-octanoyltransferase [Source:MGI Symbol;Acc:MGI:1921364]
ENSMUSG00000003644	11.27867939	12.08707881	13.32691812	9.815294582	9.429815898	9.666680834	120.8515788	123.0121686	127.9145627	53.28056355	53.21881195	54.96530807	Rps6ka1	ribosomal protein S6 kinase polypeptide 1 [Source:MGI Symbol;Acc:MGI:104558]
ENSMUSG00000003657	8.838654347	7.692799002	8.347480531	0.511509272	0.755871565	0.313127586	0.764626872	0.678755537	1.052246815	11.39930214	12.63286293	10.35296218	Calb2	calbindin 2 [Source:MGI Symbol;Acc:MGI:101914]
ENSMUSG00000003660	60.36476291	72.34694535	56.32972326	76.00540939	87.52237816	110.575601	109.8176238	93.78414208	128.4888912	163.4301227	175.247401	164.917082	Snrnp200	small nuclear ribonucleoprotein 200 (U5) [Source:MGI Symbol;Acc:MGI:2444401]
ENSMUSG00000003662	49.47710432	48.59413495	49.30300044	47.22696517	43.14799183	40.64438142	49.28952354	53.49711999	46.64796001	50.69887244	51.63782661	52.20297732	Ciao1	cytosolic iron-sulfur protein assembly 1 [Source:MGI Symbol;Acc:MGI:1346998]
ENSMUSG00000003665	0.076345791	0.246165088	0.312649043	0.413218899	0.256683142	0.407058454	0.068159784	0.015126277	0.014211906	0.450710749	0.533821423	0.419489015	Has1	hyaluronan synthase 1 [Source:MGI Symbol;Acc:MGI:106590]
ENSMUSG00000003680	21.27751761	15.87508753	23.32345674	20.6614983	17.73219477	14.93392267	27.10174171	25.79622498	24.67753544	19.63010841	18.77777938	19.67114889	Taf6l	TATA-box binding protein associated factor 6 like [Source:MGI Symbol;Acc:MGI:2444957]
ENSMUSG00000003721	109.3022507	117.2608588	88.44206553	114.8707412	132.1029788	135.951493	35.76555379	32.01021513	36.62221263	45.51705857	44.82827327	43.7822239	Insig2	insulin induced gene 2 [Source:MGI Symbol;Acc:MGI:1920249]
ENSMUSG00000003731	44.20735051	54.8320429	43.47503191	44.31470782	49.13947647	57.48629588	68.08969965	56.69060031	78.28547665	92.58659689	99.51502329	90.98297569	Kpna6	karyopherin (importin) alpha 6 [Source:MGI Symbol;Acc:MGI:1100836]
ENSMUSG00000003746	23.07458386	28.02318086	17.24946989	19.12275311	24.58272973	31.14644333	1.75673502	2.147282789	2.432424351	9.872940284	10.44179006	10.85813685	Man1a	mannosidase 1, alpha [Source:MGI Symbol;Acc:MGI:104677]
ENSMUSG00000003752	3.263265599	3.83834276	3.245452886	4.785354888	5.118384905	6.799810349	4.390863602	4.849094267	4.339019667	4.691111828	4.229728524	4.95769075	Itpkc	inositol 1,4,5-trisphosphate 3-kinase C [Source:MGI Symbol;Acc:MGI:2442554]
ENSMUSG00000003762	9.140371083	9.675349205	12.64221381	9.266187193	7.784390911	8.37773782	7.160165108	6.810046674	6.682757828	5.875677706	5.806996379	6.11480306	Coq8b	coenzyme Q8B [Source:MGI Symbol;Acc:MGI:1924139]
ENSMUSG00000003778	17.15752535	20.70378822	14.62893191	20.37207749	21.83300007	25.15239393	32.30695239	31.03215078	35.86247966	48.55868308	48.77597033	45.05530992	Brd8	bromodomain containing 8 [Source:MGI Symbol;Acc:MGI:1925906]
ENSMUSG00000003779	13.4248882	20.77950213	8.1798073	19.93303173	21.18604561	29.58631589	33.64332483	27.2625948	39.89738632	59.00407813	57.41256629	60.6915283	Kif20a	kinesin family member 20A [Source:MGI Symbol;Acc:MGI:1201682]
ENSMUSG00000003808	47.86263696	41.77704258	41.99787923	45.00509042	41.06362356	36.35189464	119.9471755	124.2406796	120.9964032	78.89319233	84.18009685	85.70304652	Farsa	phenylalanyl-tRNA synthetase, alpha subunit [Source:MGI Symbol;Acc:MGI:1913840]
ENSMUSG00000003809	21.73195935	20.4215115	23.22165535	21.57279766	20.87817462	20.89456881	77.85457545	83.72234116	76.08233477	56.08550021	58.5195684	52.83178883	Gcdh	glutaryl-Coenzyme A dehydrogenase [Source:MGI Symbol;Acc:MGI:104541]
ENSMUSG00000003810	73.29144562	76.73363642	80.69316573	88.82535864	96.42709839	102.9483496	24.95310381	25.50204594	30.69832378	31.79166036	32.24547591	30.7629424	Mast2	microtubule associated serine/threonine kinase 2 [Source:MGI Symbol;Acc:MGI:894676]
ENSMUSG00000003812	12.91523818	11.71310478	16.13422227	14.23793615	14.06168533	11.97806353	10.42518201	12.5955071	10.96213168	8.469746877	8.021197619	7.946826172	Dnase2a	deoxyribonuclease II alpha [Source:MGI Symbol;Acc:MGI:1329019]
ENSMUSG00000003813	53.69152417	52.96838405	47.39148782	64.73723978	66.99969435	58.95501671	96.61578458	91.05113046	89.8284176	75.96604441	75.49737893	80.26809675	Rad23a	RAD23 homolog A, nucleotide excision repair protein [Source:MGI Symbol;Acc:MGI:105126]
ENSMUSG00000003814	1405.853391	1244.046767	1541.678919	1085.031649	995.5825073	832.2817622	834.8044308	870.5314492	790.8685764	793.8470343	780.4484984	752.2817932	Calr	calreticulin [Source:MGI Symbol;Acc:MGI:88252]
ENSMUSG00000003824	22.17727587	18.32436922	22.04953225	22.231628	20.37016682	18.07621329	151.6590679	160.8548195	127.4678096	62.74233838	57.81703916	57.46276179	Syce2	synaptonemal complex central element protein 2 [Source:MGI Symbol;Acc:MGI:1919096]
ENSMUSG00000003847	22.42108899	27.47784934	19.46245989	25.61743131	31.91636699	44.22360911	14.5463359	12.79145126	18.1454265	31.15088484	30.00168113	30.79736413	Nfat5	nuclear factor of activated T cells 5 [Source:MGI Symbol;Acc:MGI:1859333]
ENSMUSG00000003848	48.02559058	38.65565049	46.43450841	44.81669202	39.23665218	39.51477665	158.6315517	166.0443039	144.7166149	114.2355598	114.2139194	116.0213909	Nob1	NIN1/RPN12 binding protein 1 homolog [Source:MGI Symbol;Acc:MGI:1914869]
ENSMUSG00000003849	13.93646451	15.05947931	15.8849067	20.19308834	17.5244093	16.14431796	44.6977729	49.87196734	46.93461415	17.17460444	14.5297048	15.76389234	Nqo1	NAD(P)H dehydrogenase, quinone 1 [Source:MGI Symbol;Acc:MGI:103187]
ENSMUSG00000003863	2.101048378	2.306273857	1.836558689	1.547892582	1.407112136	1.516102611	4.760721493	5.124352336	4.791717444	3.791647173	3.18076764	3.048890152	Ppfia3	protein tyrosine phosphatase, receptor type, f polypeptide (PTPRF), interacting protein (liprin), alpha 3 [Source:MGI Symbol;Acc:MGI:
ENSMUSG00000003865	74.26418727	70.95312539	71.81817426	77.98743513	75.64703847	73.68895654	37.08340213	42.76264439	41.20589852	43.32984147	41.05836945	39.55248651	Gys1	glycogen synthase 1, muscle [Source:MGI Symbol;Acc:MGI:101805]
ENSMUSG00000003868	91.31911757	72.44739784	93.68254506	90.34532513	76.57036603	69.94628647	478.5642697	500.8075786	431.828676	238.0505712	232.7709278	236.3909936	Ruvbl2	RuvB-like protein 2 [Source:MGI Symbol;Acc:MGI:1342299]
ENSMUSG00000003872	2.903762982	2.53320632	3.783628851	3.530878419	3.235769207	3.820284614	6.206080719	4.009793444	4.504505795	1.731564949	3.076296053	3.041274607	Lin7b	lin-7 homolog B (C. elegans) [Source:MGI Symbol;Acc:MGI:1330858]
ENSMUSG00000003873	121.8364601	90.52168078	161.5703087	103.2695319	86.64345402	61.5462675	139.2909546	138.2869464	88.64047138	72.03852747	66.0979124	69.19324591	Bax	BCL2-associated X protein [Source:MGI Symbol;Acc:MGI:99702]
ENSMUSG00000003882	1.171235055	1.193964232	0.688009262	0	0.060519837	0.043874191	0.017141836	0.095104524	0.089355537	1.631573764	1.82200973	1.956842499	Il7r	interleukin 7 receptor [Source:MGI Symbol;Acc:MGI:96562]
ENSMUSG00000003923	35.12593113	34.68107818	32.33046562	42.15679669	42.98549431	40.52319254	85.6626247	88.91942922	84.99223407	82.40265251	84.63452572	84.23429055	Tfam	transcription factor A, mitochondrial [Source:MGI Symbol;Acc:MGI:107810]
ENSMUSG00000003929	4.713470105	5.444452826	4.588776062	6.800185762	7.519528758	10.23086867	5.24073614	4.955827723	5.28874884	8.522704919	7.714589107	7.265240914	Zfp81	zinc finger protein 81 [Source:MGI Symbol;Acc:MGI:1890752]
ENSMUSG00000003934	1.365340016	1.868030749	1.38398524	1.740867088	1.661757225	1.493004396	1.786176061	1.432914139	1.950241435	1.596107936	2.182995238	1.832967143	Efnb3	ephrin B3 [Source:MGI Symbol;Acc:MGI:109196]
ENSMUSG00000003948	86.91118848	85.99105689	81.37260067	102.5341117	107.5106407	102.9219245	5.799472429	5.903857022	5.953751981	20.23905526	17.08164006	16.28482772	Mmd	monocyte to macrophage differentiation-associated [Source:MGI Symbol;Acc:MGI:1914718]
ENSMUSG00000003949	3.548106956	4.463707556	2.834630017	2.373227479	1.637670255	3.046203062	0.366204519	0.264125844	0.114535228	1.137394621	0.90481144	0.890615982	Hlf	hepatic leukemia factor [Source:MGI Symbol;Acc:MGI:96108]
ENSMUSG00000003955	487.8706676	370.6489	584.2069442	444.5402789	377.975342	290.310437	258.9951003	288.0232513	221.9546435	247.9056675	246.5673876	261.5133221	Fam162a	family with sequence similarity 162, member A [Source:MGI Symbol;Acc:MGI:1917436]
ENSMUSG00000003970	1741.839187	1285.969946	1993.241682	1607.391115	1290.65218	1051.870878	3815.048459	4321.681853	3328.806982	1867.052106	1885.411493	1913.227195	Rpl8	ribosomal protein L8 [Source:MGI Symbol;Acc:MGI:1350927]
ENSMUSG00000003974	0.028224656	0	0	0.013169393	0.014827261	0	0	0	0	0	0.011747089	0	Grm3	glutamate receptor, metabotropic 3 [Source:MGI Symbol;Acc:MGI:1351340]
ENSMUSG00000003992	16.98492941	18.37013776	16.57073808	14.79338657	17.00560582	17.8075561	3.330080229	2.936300654	3.130777325	7.328204123	6.015682646	6.198072744	Ssbp2	single-stranded DNA binding protein 2 [Source:MGI Symbol;Acc:MGI:1914220]
ENSMUSG00000004018	7.06870693	6.637213972	6.123522563	10.01299869	9.673894575	10.02272072	29.45030936	32.60679841	31.61419964	24.5778252	24.01873031	23.58007754	Fancl	Fanconi anemia, complementation group L [Source:MGI Symbol;Acc:MGI:1914280]
ENSMUSG00000004031	0.051531123	0.089011106	0.065946482	0.012021999	0.027070849	0	0.149518893	0.114860069	0.107916875	0.334176954	0.343155693	0.433847419	Brinp2	bone morphogenic protein/retinoic acid inducible neural-specific 2 [Source:MGI Symbol;Acc:MGI:2443333]
ENSMUSG00000004032	43.28863588	35.76911829	53.74694129	33.25892662	24.59944771	20.60760669	10.91595007	9.896206479	8.135740575	13.12407076	13.55584284	12.95688781	Gstm5	glutathione S-transferase, mu 5 [Source:MGI Symbol;Acc:MGI:1309466]
ENSMUSG00000004035	3.84182927	3.476050203	3.1465893	1.43405278	0.4036457	1.045089944	6.56580414	7.93797674	6.640803186	4.614628621	3.472044052	3.015612361	Gstm7	glutathione S-transferase, mu 7 [Source:MGI Symbol;Acc:MGI:1915562]
ENSMUSG00000004038	0.262568957	0.075590413	0.369622237	0.214396907	0.068967686	0.324990289	1.846022736	2.308493659	2.138398632	2.818340787	3.278432538	2.646906428	Gstm3	glutathione S-transferase, mu 3 [Source:MGI Symbol;Acc:MGI:106026]
ENSMUSG00000004040	96.61642673	115.0770612	85.93714013	91.49375744	103.1457127	114.3885202	70.06668997	61.09620613	81.04789763	106.0187195	112.5673229	107.7079377	Stat3	signal transducer and activator of transcription 3 [Source:MGI Symbol;Acc:MGI:103038]
ENSMUSG00000004043	6.666135733	8.431788196	5.644931937	7.2961202	7.395015404	7.656724228	3.133968317	3.354568154	3.993364099	4.250010296	4.353513913	5.090672072	Stat5a	signal transducer and activator of transcription 5A [Source:MGI Symbol;Acc:MGI:103036]
ENSMUSG00000004044	330.1892105	333.641365	319.7662614	401.2638633	395.0008731	368.9801909	89.28908009	88.64122064	89.43988827	85.30417221	86.53011893	88.79913034	Cavin1	caveolae associated 1 [Source:MGI Symbol;Acc:MGI:1277968]
ENSMUSG00000004054	32.2127262	33.31312074	36.55456255	34.14626462	29.62774104	29.91547492	16.26407208	14.71637383	15.84015502	13.47428502	15.61971553	13.71953082	Map3k11	mitogen-activated protein kinase kinase kinase 11 [Source:MGI Symbol;Acc:MGI:1346880]
ENSMUSG00000004056	75.74366423	84.20497252	76.62264343	83.56761792	83.34080953	76.40832578	48.52799837	47.87382371	55.5757381	51.39290336	52.21241966	54.48984809	Akt2	thymoma viral proto-oncogene 2 [Source:MGI Symbol;Acc:MGI:104874]
ENSMUSG00000004069	28.51800421	30.29152153	28.03793131	33.18352873	32.55210298	34.72781965	60.10319366	54.88295879	59.45344528	43.7291681	49.17232057	46.22305338	Dnaja3	DnaJ heat shock protein family (Hsp40) member A3 [Source:MGI Symbol;Acc:MGI:1933786]
ENSMUSG00000004070	45.89982792	41.06763529	37.74689911	37.78465564	39.30587833	35.56329899	38.04333711	41.34789675	38.15127103	39.86519534	35.85151462	37.98538862	Hmox2	heme oxygenase 2 [Source:MGI Symbol;Acc:MGI:109373]
ENSMUSG00000004071	13.38554033	15.94631515	13.88989247	16.80377611	17.23708034	17.95216781	17.81494383	17.44602205	17.32851665	19.98953067	19.75629725	18.21076576	Cdip1	cell death inducing Trp53 target 1 [Source:MGI Symbol;Acc:MGI:1913876]
ENSMUSG00000004085	24.17721498	27.84130589	24.11537474	25.68729505	28.72872293	31.33815329	8.382313968	8.702852784	9.556603149	18.49910761	19.01295644	18.15002992	Map3k20	mitogen-activated protein kinase kinase kinase 20 [Source:MGI Symbol;Acc:MGI:2443258]

ENSMUSG00000004096	166.3988009	136.6250373	179.4721304	160.8703192	142.0337971	120.213266	138.3032869	138.2754191	119.9940095	117.9216237	107.313397	110.746137	Cwc15	CWC15 spliceosome-associated protein [Source:MGI Symbol;Acc:MGI:1913320]
ENSMUSG00000004098	18.58885498	18.24937366	18.46984649	21.69550843	20.50259789	20.36386988	14.60728076	13.45052132	13.74571715	5.922993829	6.738392858	6.468093726	Col5a3	collagen, type V, alpha 3 [Source:MGI Symbol;Acc:MGI:1858212]
ENSMUSG00000004099	38.06507639	44.42662352	30.32591533	50.78940332	56.26896929	61.10473913	107.9904898	100.5125016	128.6112741	87.07203557	88.24151891	86.18188513	Dnmt1	DNA methyltransferase (cytosine-5) 1 [Source:MGI Symbol;Acc:MGI:94912]
ENSMUSG00000004100	51.05171615	39.48836647	55.56956047	42.60478504	36.17865423	31.7301235	107.469824	120.975946	102.0495964	72.30221154	72.06155704	77.50569812	Ppan	peter pan homolog [Source:MGI Symbol;Acc:MGI:2178445]
ENSMUSG00000004105	88.17369965	100.8172806	80.25325258	105.4352612	114.1145455	118.0261203	2.261519635	2.801618216	2.745304741	19.69404408	20.94131832	18.83580978	Angptl2	angiopoietin-like 2 [Source:MGI Symbol;Acc:MGI:1347002]
ENSMUSG00000004110	0	0.116355438	0.063659321	0.021759471	0.03266497	0.047361301	0	0	0	0.055618449	0.038818868	0.061993523	Cacna1e	calcium channel, voltage-dependent, R type, alpha 1E subunit [Source:MGI Symbol;Acc:MGI:106217]
ENSMUSG00000004113	0.277987462	0.182923916	0.294765864	0.324266665	0.396381195	0.393227751	1.48022165	1.937538532	2.014470473	1.456397435	1.652832279	1.425364688	Cacna1b	calcium channel, voltage-dependent, N type, alpha 1B subunit [Source:MGI Symbol;Acc:MGI:88296]
ENSMUSG00000004127	7.07342776	8.801280316	7.20410411	9.532593372	9.728860994	9.362006749	11.08814746	10.629161	12.87660593	12.02954372	13.1981057	12.66754904	Trmt10a	tRNA methyltransferase 10A [Source:MGI Symbol;Acc:MGI:1920421]
ENSMUSG00000004151	9.595513008	10.59871724	8.420413567	18.39052543	21.26680098	26.73991258	10.68050556	10.44042277	14.05117108	8.471492805	8.402221099	9.134858012	Etv1	ets variant 1 [Source:MGI Symbol;Acc:MGI:99254]
ENSMUSG00000004187	6.429544158	5.47629483	5.316650886	6.763251572	6.155686141	5.33193616	9.5103087	10.40206363	8.268326154	6.329585982	6.476172445	6.760077268	Kifc2	kinesin family member C2 [Source:MGI Symbol;Acc:MGI:109187]
ENSMUSG00000004207	589.0299633	592.4325053	593.2180293	443.1962965	430.25079	393.773144	131.6393691	130.6588958	134.5657568	253.1357637	256.0444619	256.6448642	Psap	prosaposin [Source:MGI Symbol;Acc:MGI:97783]
ENSMUSG00000004221	12.00657461	15.27351049	11.52019326	12.30821382	15.2558655	16.29690992	6.877710987	5.141468228	6.550995232	11.10992844	12.14819797	11.42676732	Ikbkg	inhibitor of kappaB kinase gamma [Source:MGI Symbol;Acc:MGI:1338074]
ENSMUSG00000004231	0.041788755	0.160406331	0	0	0.04390578	0.095489215	0.198976259	0.151791623	0.155581035	0.02491937	0.092759794	0.012344741	Pax2	paired box 2 [Source:MGI Symbol;Acc:MGI:97486]
ENSMUSG00000004233	3.114823249	3.977756939	3.352468229	4.17372577	5.244584495	5.246884974	1.978676938	1.661517961	2.19294658	2.696836623	2.825461175	3.60979701	Wars2	tryptophanyl tRNA synthetase 2 (mitochondrial) [Source:MGI Symbol;Acc:MGI:1917810]
ENSMUSG00000004263	3.958248339	4.102313794	7.914148845	4.302158339	4.648041389	2.607024369	0.997788417	1.037967071	0.520118851	0.958033795	0.717112767	0.763483481	Atn1	atrophin 1 [Source:MGI Symbol;Acc:MGI:104725]
ENSMUSG00000004264	146.9830888	137.1688638	161.3508604	164.0754181	137.8658846	133.2857262	363.285301	367.6020034	344.3018651	217.9464044	235.2026284	223.7842661	Phb2	prohibitin 2 [Source:MGI Symbol;Acc:MGI:102520]
ENSMUSG00000004266	14.79244775	12.73686609	17.26185353	11.32856741	11.7626659	9.109602392	39.81978604	40.53241526	36.78495735	25.33690576	25.93635811	27.89240677	Ptpn6	protein tyrosine phosphatase, non-receptor type 6 [Source:MGI Symbol;Acc:MGI:96055]
ENSMUSG00000004267	203.6131031	187.8580133	222.7617126	215.4478761	195.1354042	177.5213856	14.55788293	17.14996544	16.28041468	30.84177606	33.24571413	35.52280604	Eno2	enolase 2, gamma neuronal [Source:MGI Symbol;Acc:MGI:95394]
ENSMUSG00000004268	67.38720081	55.73868083	69.5053765	63.79387457	59.96954428	49.00542019	137.2440111	140.6746384	121.1128468	100.6197455	98.71225068	99.13441036	Emg1	EMG1 N1-specific pseudouridine methyltransferase [Source:MGI Symbol;Acc:MGI:1315195]
ENSMUSG00000004270	63.25483294	70.21540781	56.50273897	73.68695147	71.65008256	75.10043002	58.16891053	56.97390046	61.31175307	49.47913243	49.87649294	50.33244044	Lpcat3	lysophosphatidylcholine acyltransferase 3 [Source:MGI Symbol;Acc:MGI:1315211]
ENSMUSG00000004285	238.8238113	185.0330817	299.9411605	208.856703	172.5835803	128.7995088	165.6044731	181.2065234	126.695637	105.1817125	111.9473437	112.5616544	Atp6v1f	ATPase, H+ transporting, lysosomal V1 subunit F [Source:MGI Symbol;Acc:MGI:1913394]
ENSMUSG00000004296	0	0.130187902	0.046297688	0	0	0.017222291	0	0.044798579	0	0.040449781	0.094106347	0.02003831	Il12b	interleukin 12b [Source:MGI Symbol;Acc:MGI:96540]
ENSMUSG00000004317	11.47833809	13.89357157	11.28838911	13.1411382	15.47614307	21.08896498	3.219426691	2.591457786	3.822873554	11.11996074	12.48503017	11.75403454	Clcn5	chloride channel, voltage-sensitive 5 [Source:MGI Symbol;Acc:MGI:99486]
ENSMUSG00000004319	6.768962025	7.683134268	5.023763489	8.696752806	10.49460624	13.76109994	27.92154708	25.17970404	35.81587407	44.36915664	43.67931332	44.36377147	Clcn3	chloride channel, voltage-sensitive 3 [Source:MGI Symbol;Acc:MGI:103555]
ENSMUSG00000004328	0.601669533	0.314933362	0.149329662	0.323269525	0.229872805	0.555491875	7.699248295	7.333090748	8.586809606	6.131981083	6.374189704	4.992825233	Hif3a	hypoxia inducible factor 3, alpha subunit [Source:MGI Symbol;Acc:MGI:1859778]
ENSMUSG00000004341	0	0.272849756	0.097031388	0.132665676	0.248944484	0	0.42307108	0.704171493	0.838032906	1.314016473	1.104485147	0.881928412	Gpx6	glutathione peroxidase 6 [Source:MGI Symbol;Acc:MGI:1922762]
ENSMUSG00000004344	0	0	0	0	0	0	0	0	0	0	0	0	Gpx5	glutathione peroxidase 5 [Source:MGI Symbol;Acc:MGI:104886]
ENSMUSG00000004347	0.247514765	0.42003828	0.466796475	0.425483416	0.492733715	0.565582554	0.081412043	0.025810383	0.024250167	0.326267956	0.412062146	0.311713117	Pde1c	phosphodiesterase 1C [Source:MGI Symbol;Acc:MGI:108413]
ENSMUSG00000004356	34.56575737	38.39689153	31.30401059	42.47747334	47.2191921	56.90665963	69.00674751	65.28653415	81.14996887	123.1779931	130.695126	129.623266	Utp20	UTP20 small subunit processome component [Source:MGI Symbol;Acc:MGI:1917933]
ENSMUSG00000004359	0.142102007	0.09818266	0.349159183	0.053042903	0	0.043294611	0.355223221	0.281544569	0.238072895	3.838628086	3.075425239	2.795741361	Spic	Spi-C transcription factor (Spi-1/PU.1 related) [Source:MGI Symbol;Acc:MGI:1341168]
ENSMUSG00000004360	0.075412973	0.161277843	0.066177642	0.100534496	0.12450964	0.196939533	1.57737508	1.280696476	1.69461858	0.183092479	0.197288748	0.07638038	9330159F19	RIKEN cDNA 9330159F19 gene [Source:MGI Symbol;Acc:MGI:3036239]
ENSMUSG00000004364	59.53405287	67.08723062	55.47526271	60.69888511	70.73604385	75.49397435	72.66102997	71.24029688	88.79035864	93.34242444	89.21452643	87.35683087	Cul3	cullin 3 [Source:MGI Symbol;Acc:MGI:1347360]
ENSMUSG00000004366	0	0	0	0	0.062702245	0	0	0	0.055546673	0	0	0	Sst	somatostatin [Source:MGI Symbol;Acc:MGI:98326]
ENSMUSG00000004371	3.292502444	2.081600056	3.040365355	3.614716684	3.391471744	2.434079552	0.374638487	0.671524983	0.120177499	2.165489481	2.17641929	1.458948341	Il11	interleukin 11 [Source:MGI Symbol;Acc:MGI:107613]
ENSMUSG00000004383	13.27153746	14.54027362	11.43633471	11.42389253	13.01501261	15.08381322	4.41998321	4.512961395	4.927488065	7.070547363	7.13393894	6.73801144	Large1	LARGE xylosyl- and glucuronyltransferase 1 [Source:MGI Symbol;Acc:MGI:1342270]
ENSMUSG00000004393	65.45491789	51.59913364	57.76448113	57.96717172	50.49119319	48.1950955	86.20499828	85.64841994	88.67138926	116.9365514	120.2661786	118.8547826	Ddx56	DEAD (Asp-Glu-Ala-Asp) box polypeptide 56 [Source:MGI Symbol;Acc:MGI:1277172]
ENSMUSG00000004394	74.09625963	80.69121294	77.16845227	76.55134192	72.11991345	69.07182146	33.07572663	32.26746863	34.02777559	43.23167551	43.36221376	44.66616458	Tmed4	transmembrane p24 trafficking protein 4 [Source:MGI Symbol;Acc:MGI:1915070]
ENSMUSG00000004415	0.92463366	0.722517879	0.845208177	0.986117644	0.797997945	0.377291105	0.619118907	1.079549481	0.368833322	1.388284282	1.539328493	1.199884184	Col26a1	collagen, type XXVI, alpha 1 [Source:MGI Symbol;Acc:MGI:2155345]
ENSMUSG00000004446	35.0304978	39.0121959	34.43369882	40.05570151	36.62167347	39.29158156	63.45310717	64.80374411	73.95439912	49.23460633	47.56201971	50.94704636	Bid	BH3 interacting domain death agonist [Source:MGI Symbol;Acc:MGI:108093]
ENSMUSG00000004451	52.70704424	48.20891865	53.23661854	47.31080863	43.74534359	38.59858133	43.32624921	46.37963588	44.63833812	32.81175701	32.79571728	30.36471615	Ralb	v-ral simian leukemia viral oncogene B [Source:MGI Symbol;Acc:MGI:1927244]
ENSMUSG00000004455	149.4437697	161.7213541	128.553439	166.0235218	184.3861324	191.9921578	210.4345374	194.6363888	215.0193019	192.3472385	186.3798757	184.059305	Ppp1cc	protein phosphatase 1 catalytic subunit gamma [Source:MGI Symbol;Acc:MGI:104872]
ENSMUSG00000004460	75.80470058	71.16407569	80.44439191	59.50542473	55.89837197	48.23420847	50.34148747	54.11584756	48.43489444	60.9508589	62.86271105	59.5396436	Dnajb11	DnaJ heat shock protein family (Hsp40) member B11 [Source:MGI Symbol;Acc:MGI:1915088]
ENSMUSG00000004462	5.561689789	7.279342459	5.888447063	7.291425988	7.639236644	7.893868779	16.90641119	14.83573009	16.1358183	18.10341924	17.95360404	17.74406284	Tbccd1	TBCC domain containing 1 [Source:MGI Symbol;Acc:MGI:1917823]
ENSMUSG00000004473	0.621297843	0.732094925	1.005894229	0.229217301	0.425061422	0.418204427	0.361187345	0.357839554	0.336208458	0.103392791	0.445004833	0.115243836	Clec11a	C-type lectin domain family 11, member a [Source:MGI Symbol;Acc:MGI:1298219]
ENSMUSG00000004500	2.329607916	3.813732107	2.475280081	2.984777106	2.870999439	3.280279502	1.056773861	0.973021984	0.843880278	2.275261694	2.400370057	2.120354665	Zfp324	zinc finger protein 324 [Source:MGI Symbol;Acc:MGI:2444641]
ENSMUSG00000004508	3.877476071	5.151127687	3.103024668	4.58395302	5.567310518	6.941692008	0.354569943	0.393437506	0.262649281	2.570850316	2.740421612	2.435985896	Gab2	growth factor receptor bound protein 2-associated protein 2 [Source:MGI Symbol;Acc:MGI:1333854]
ENSMUSG00000004530	82.32166278	109.6024615	66.01908559	102.0590079	109.856822	126.5306571	114.8050921	105.1590563	147.3140392	158.2254284	150.4260564	153.8403524	Coro1c	coronin, actin binding protein 1C [Source:MGI Symbol;Acc:MGI:1345964]
ENSMUSG00000004535	86.08070896	99.31767224	78.6525835	91.69078778	100.415829	108.6803155	74.37183552	73.56661746	82.88164176	108.0337898	101.3263951	102.5781446	Tax1bp1	Tax1 (human T cell leukemia virus type I) binding protein 1 [Source:MGI Symbol;Acc:MGI:1289308]
ENSMUSG00000004540	0.081959024	0.141570045	0.062931786	0.076482815	0.086111083	0.156066765	0.128049514	0.040596045	0.038142049	0.6048109	0.47755834	0.617389885	Psg17	pregnancy specific glycoprotein 17 [Source:MGI Symbol;Acc:MGI:1347250]
ENSMUSG00000004542	0	0	0	0	0.030985825	0	0	0	0	0	0.049097842	0	Psg19	pregnancy specific glycoprotein 19 [Source:MGI Symbol;Acc:MGI:1347252]
ENSMUSG00000004552	0	0.026754598	0	0	0	0	0	0	0	0.020781839	0.019339566	0	Ctse	cathepsin E [Source:MGI Symbol;Acc:MGI:107361]
ENSMUSG00000004558	50.94915474	48.68671311	49.25742901	36.557644	37.61736937	32.28485615	86.76787935	90.55341775	86.97241114	44.51966259	44.63744929	40.03006413	Ndrg2	N-myc downstream regulated gene 2 [Source:MGI Symbol;Acc:MGI:1352498]
ENSMUSG00000004561	28.38839784	22.2074254	28.37908346	28.7801266	24.5561591	23.57578514	34.99798881	36.80988575	33.96223385	23.96357837	23.47582771	24.26938753	Mettl17	methyltransferase like 17 [Source:MGI Symbol;Acc:MGI:1098577]
ENSMUSG00000004562	72.80266467	69.23659996	69.530883	75.62636674	78.30726571	82.79667019	7.66010483	7.675189839	8.860080148	26.68957192	28.0707655	30.01831336	Arhgef40	Rho guanine nucleotide exchange factor (GEF) 40 [Source:MGI Symbol;Acc:MGI:2685515]
ENSMUSG00000004565	22.69380913	22.97349413	23.65380061	21.63214777	19.8830387	26.7778202	116.6410498	99.22321878	119.5269075	94.9354546	104.2702245	100.4454343	Pnpla6	patatin-like phospholipase domain containing 6 [Source:MGI Symbol;Acc:MGI:1354723]
ENSMUSG00000004567	78.270172	71.27215799	77.17564988	73.44981969	68.59987814	66.71754753	65.17796396	62.50825245	62.2684344	45.68197878	44.61133343	45.51973754	Mcoln1	mucolipin 1 [Source:MGI Symbol;Acc:MGI:1890498]
ENSMUSG00000004568	16.95197182	19.4008845	16.62262663	18.42106883	18.37469072	21.69036016	24.50510961	22.02187766	25.02031192	34.30043295	36.43740347	35.47707869	Arhgef18	rho/rac guanine nucleotide exchange factor (GEF) 18 [Source:MGI Symbol;Acc:MGI:2142567]
ENSMUSG00000004591	24.00380236	31.55679428	20.27620842	34.67485133	40.56255852	51.70823463	34.6496676	31.67490669	43.36928844	55.55495319	57.69981106	59.53738487	Pkn2	protein kinase N2 [Source:MGI Symbol;Acc:MGI:109211]
ENSMUSG00000004609	2.778556285	3.04192115	1.863055602	2.592903071	2.549263972	3.129851903	0.157223141	0.251994552	0.418886078	5.828327786	5.081794065	5.601149711	Cd33	CD33 antigen [Source:MGI Symbol;Acc:MGI:99440]
ENSMUSG00000004610	2.334276012	2.86724141	4.062684919	2.105697753	2.043775694	0.829722041	1.667190773	3.314487253	1.66569708	1.113575881	1.360134512	1.792867128	Etfb	electron transferring flavoprotein, beta polypeptide [Source:MGI Symbol;Acc:MGI:106098]
ENSMUSG00000004612	0	0	0	0	0	0	0	0.183205649	0	0.220561412	0	0.218526674	Nkg7	natural killer cell group 7 sequence [Source:MGI Symbol;Acc:MGI:1931250]
ENSMUSG00000004626	28.2021924	23.44477706	34.3728544	15.74056434	15.06943945	16.36655213	148.2880509	150.3878222	147.4468762	92.20774965	92.96290152	96.49862398	Stxbp2	syntaxin binding protein 2 [Source:MGI Symbol;Acc:MGI:107370]
ENSMUSG00000004630	0	0	0	0	0	0.164486929	0.048199315	0	0	0.144873148	0	0.191382207	Pcp2	Purkinje cell protein 2 (L7) [Source:MGI Symbol;Acc:MGI:97508]
ENSMUSG00000004631	7.712653479	7.401264126	7.711452603	2.883137559	2.677431852	3.177774538	5.154194257	5.607488351	5.079609365	7.68549014	7.527550874	8.274120829	Sgce	sarcoglycan, epsilon [Source:MGI Symbol;Acc:MGI:1329042]
ENSMUSG00000004633	1.472760453	1.237591972	1.996818002	1.04005308	1.672833241	0.970183775	0.995019347	1.301251595	0.666868388	1.174933293	1.789187119	2.010708203	Chn2	chimerin 2 [Source:MGI Symbol;Acc:MGI:1917243]
ENSMUSG00000004637	7.597967182	7.33725789	7.587686217	10.0010521	7.786810719	9.015901588	15.56587024	15.74039977	16.02958605	13.49701919	13.40358909	12.73459456	Wwox	WW domain-containing oxidoreductase [Source:MGI Symbol;Acc:MGI:1931237]
ENSMUSG00000004642	58.21501294	62.5121598	49.67811806	72.81702676	72.53625692	76.17676778	133.441072	130.2859695	137.8471957	81.80182326	77.70487768	76.6494996	Slbp	stem-loop binding protein [Source:MGI Symbol;Acc:MGI:108402]

ENSMUSG00000004651	0	0	0.104037028	0	0	0	0	0	0	0	0.012083966	0	Tyr	tyrosinase [Source:MGI Symbol;Acc:MGI:98880]
ENSMUSG00000004654	0	0	0	0	0	0	0	0	0	0	0	0	Ghrhr	growth hormone releasing hormone receptor [Source:MGI Symbol;Acc:MGI:95710]
ENSMUSG00000004655	0.458621111	2.225672952	0.855220693	1.314502891	0.96370987	0.798453491	0.277838686	0.405651387	0.10671643	0.293018396	0.218146177	0.159673373	Aqp1	aquaporin 1 [Source:MGI Symbol;Acc:MGI:103201]
ENSMUSG00000004661	4.252815657	4.129126302	4.140570028	4.416104394	4.227328218	4.09675258	39.12210429	36.76066323	43.54181009	32.0173635	32.58919193	33.18153916	Arid3b	AT rich interactive domain 3B (BRIGHT-like) [Source:MGI Symbol;Acc:MGI:1930768]
ENSMUSG00000004665	223.2245768	212.5390328	237.4346031	187.2247809	165.1585641	139.458975	21.8054783	19.26094122	21.12456722	69.9739873	72.90310272	69.55369739	Cnn2	calponin 2 [Source:MGI Symbol;Acc:MGI:105093]
ENSMUSG00000004667	88.74625615	65.91309023	102.1427561	80.09007732	63.03351177	57.35453611	244.8650536	273.3514045	210.134437	152.1700152	146.7496981	149.5304179	Polr2e	polymerase (RNA) II (DNA directed) polypeptide E [Source:MGI Symbol;Acc:MGI:1913670]
ENSMUSG00000004668	0.031550438	0.196192539	0.012920443	0.01177694	0.013259516	0.014418839	0.016900508	0.062510409	0.064604879	0.197547855	0.131312786	0.178948959	Abca13	ATP-binding cassette, sub-family A (ABC1), member 13 [Source:MGI Symbol;Acc:MGI:2388707]
ENSMUSG00000004677	31.2512413	38.70733503	30.06860008	37.3564913	41.33185266	47.48293658	19.68370433	17.50862947	22.552759	28.79858623	30.60332688	28.87937756	Myo9b	myosin IXb [Source:MGI Symbol;Acc:MGI:106624]
ENSMUSG00000004698	1.421124369	1.424359165	1.050787619	1.473522122	2.115251592	2.104753619	0.176214927	0.091247992	0.171464257	0.965141664	1.00769191	1.189466752	Hdac9	histone deacetylase 9 [Source:MGI Symbol;Acc:MGI:1931221]
ENSMUSG00000004707	1.911492287	2.590974209	2.364674236	0.018580971	0.167360734	0.136495227	0.213317098	0.177525511	0.33358849	5.966436249	6.331349576	6.56429456	Ly9	lymphocyte antigen 9 [Source:MGI Symbol;Acc:MGI:96885]
ENSMUSG00000004709	0.053297053	0.040916198	0.200072152	0	0.018665697	0	0	0	0.01653557	0.381383647	0.443644205	0.314887735	Cd244a	CD244 molecule A [Source:MGI Symbol;Acc:MGI:109294]
ENSMUSG00000004730	31.20570706	38.43560558	25.1962403	0.775550039	1.042185532	0.939317725	2.249841921	2.310559969	3.618147645	47.8401825	46.57309603	50.2023848	Adgre1	adhesion G protein-coupled receptor E1 [Source:MGI Symbol;Acc:MGI:106912]
ENSMUSG00000004748	1.842053815	2.610731888	2.707931955	1.410440495	1.488748134	1.798793668	19.94536331	19.04360045	17.36489007	14.11084607	16.27682376	12.64120441	Mtfp1	mitochondrial fission process 1 [Source:MGI Symbol;Acc:MGI:1916686]
ENSMUSG00000004768	22.66216399	25.35041914	20.09267866	25.74383906	26.3780033	31.05352558	26.46393346	23.89908759	28.57207109	28.25321456	29.4826518	30.20769257	Rab23	RAB23, member RAS oncogene family [Source:MGI Symbol;Acc:MGI:99833]
ENSMUSG00000004771	88.44113989	89.48982439	88.51131537	83.53830415	89.70093773	77.13568973	64.26125985	61.91432496	64.14204479	60.88004179	57.97797653	55.18706655	Rab11a	RAB11A, member RAS oncogene family [Source:MGI Symbol;Acc:MGI:1858202]
ENSMUSG00000004788	115.281965	84.83389455	119.681319	110.0327971	96.52586891	79.95166491	100.438367	105.7381486	92.52248166	89.18715592	91.16257613	90.64966649	Eif2b2	eukaryotic translation initiation factor 2B, subunit 2 beta [Source:MGI Symbol;Acc:MGI:2145118]
ENSMUSG00000004789	120.2471795	133.7262989	122.2577507	145.6067428	153.3591834	151.8993605	119.1305436	108.7535141	122.1576436	118.1981794	113.0928215	117.57891	Dlst	dihydrolipoamide S-succinyltransferase (E2 component of 2-oxo-glutarate complex) [Source:MGI Symbol;Acc:MGI:1926170]
ENSMUSG00000004791	12.77890528	11.62316471	14.22059727	10.05277171	8.561690132	7.688023492	3.196644959	3.180120632	2.987885049	4.389947384	5.087332385	4.70506428	Pgf	placental growth factor [Source:MGI Symbol;Acc:MGI:105095]
ENSMUSG00000004798	11.67418836	14.58426938	11.17068572	12.30590407	14.86782592	16.81529559	1.207187387	1.379305588	1.682213894	5.748047249	5.505145142	6.157740365	Ulk2	unc-51 like kinase 2 [Source:MGI Symbol;Acc:MGI:1352758]
ENSMUSG00000004814	0.046366905	0	0	0	0	0	0	0	0	0	0	0	Ccl24	chemokine (C-C motif) ligand 24 [Source:MGI Symbol;Acc:MGI:1928953]
ENSMUSG00000004815	1.309562673	2.685727369	0.485213191	1.629416492	3.393899841	4.607362321	2.226948062	1.124333954	3.320016619	3.938044095	3.789321609	4.785955864	Dgkq	diacylglycerol kinase, theta [Source:MGI Symbol;Acc:MGI:102918]
ENSMUSG00000004821	0	0	0	0	0	0	0	0	0	0	0	0	Tmed11	transmembrane protein p24 trafficking protein 11 [Source:MGI Symbol;Acc:MGI:1914616]
ENSMUSG00000004837	8.4745988	5.715178081	7.765467391	6.990812246	5.558803018	4.636150906	0.125402135	0.231914297	0.043579055	1.047006702	1.909713709	1.90871993	Grap	GRB2-related adaptor protein [Source:MGI Symbol;Acc:MGI:1918770]
ENSMUSG00000004842	0.057256803	0.131868305	0.146547739	0	0.060157443	0.043611471	0	0	0.026646142	0.025607429	0	0.025371194	Pou1f1	POU domain, class 1, transcription factor 1 [Source:MGI Symbol;Acc:MGI:97588]
ENSMUSG00000004843	23.29811834	23.93376715	20.81266579	19.35053523	18.94371937	16.77913132	17.08039421	17.86158226	16.78186483	24.73767455	22.32326129	21.85692367	Chmp2b	charged multivesicular body protein 2B [Source:MGI Symbol;Acc:MGI:1916192]
ENSMUSG00000004846	151.3128868	137.8900056	166.1572995	116.2676118	106.0522208	94.030657	30.81677796	32.75831721	31.86230839	39.76381056	38.44872324	38.59639856	Plod3	procollagen-lysine, 2-oxoglutarate 5-dioxygenase 3 [Source:MGI Symbol;Acc:MGI:1347008]
ENSMUSG00000004849	343.9499408	272.8424232	387.6484187	309.4515443	256.8813748	206.9759856	131.5733568	138.891974	114.1969992	91.34676256	90.97671472	88.82897565	Ap1s1	adaptor protein complex AP-1, sigma 1 [Source:MGI Symbol;Acc:MGI:1098244]
ENSMUSG00000004864	25.67829112	19.10434473	28.89448669	9.807070623	8.124997307	6.217498539	29.15044226	33.00065628	27.89906331	21.16542544	24.97828182	22.84459728	Mapk13	mitogen-activated protein kinase 13 [Source:MGI Symbol;Acc:MGI:1346864]
ENSMUSG00000004865	70.19049547	65.31527059	66.68583738	73.92915608	77.04808903	78.95559517	95.40931325	96.39798448	109.2658669	108.4071167	109.5542734	103.4924696	Srpk1	serine/arginine-rich protein specific kinase 1 [Source:MGI Symbol;Acc:MGI:106908]
ENSMUSG00000004872	1.074448139	1.673970122	1.164718719	2.18225423	2.25775973	2.339626033	0	0	0.011765321	0.011306688	0.021043993	0.011202381	Pax3	paired box 3 [Source:MGI Symbol;Acc:MGI:97487]
ENSMUSG00000004880	9.992048159	18.29977826	6.605950115	17.75423699	26.92354863	39.20525519	114.0266105	88.06419778	158.7895665	125.5888397	126.6941405	129.6584111	Lbr	lamin B receptor [Source:MGI Symbol;Acc:MGI:2138281]
ENSMUSG00000004885	12.88148441	11.28108469	15.57066957	11.06114672	11.31629604	7.667884074	5.750147968	5.790681197	5.591768326	4.792841095	6.172216278	5.755910142	Crabp2	cellular retinoic acid binding protein II [Source:MGI Symbol;Acc:MGI:88491]
ENSMUSG00000004891	4.307667202	5.027084116	5.781549522	3.426310203	3.817141325	4.015092786	7.029100958	5.9189307	6.171067469	13.86945977	14.27862001	13.74151033	Nes	nestin [Source:MGI Symbol;Acc:MGI:101784]
ENSMUSG00000004892	0.392816251	0.730716171	0.835260362	0.169186002	0	0.023015464	2.8865094	3.232859236	3.009311245	1.405459446	1.509138012	1.33893626	Bcan	brevican [Source:MGI Symbol;Acc:MGI:1096385]
ENSMUSG00000004894	0.197529401	0.037910877	0.067409717	0.06144373	0.172946895	0.175530332	0	0	0	0	0	0.029175903	Hapln2	hyaluronan and proteoglycan link protein 2 [Source:MGI Symbol;Acc:MGI:2137300]
ENSMUSG00000004895	40.6915428	37.34508514	46.25591207	40.28738653	32.37165435	29.46382894	57.85104989	58.16123288	55.10335855	44.04387576	43.58089565	43.38321886	Prcc	papillary renal cell carcinoma (translocation-associated) [Source:MGI Symbol;Acc:MGI:2137738]
ENSMUSG00000004896	10.09205946	9.871002622	9.229789418	10.89542357	12.95468985	12.60786662	13.9107764	11.60811849	13.12700354	12.02985014	11.88037675	12.23695764	Rrnad1	ribosomal RNA adenine dimethylase domain containing 1 [Source:MGI Symbol;Acc:MGI:2387197]
ENSMUSG00000004897	400.6844151	324.7242744	403.4945286	358.5915942	319.1357143	282.9838843	777.0913703	854.9443941	743.4180769	524.0149772	500.7835574	511.6671414	Hdgf	heparin binding growth factor [Source:MGI Symbol;Acc:MGI:1194494]
ENSMUSG00000004902	0.045032431	0.129642898	0.230519363	0.126070574	0.141941345	0.120051358	0.221121609	0.178444156	0.230528868	0.281963124	0.243652214	0.359179636	Slc25a18	solute carrier family 25 (mitochondrial carrier), member 18 [Source:MGI Symbol;Acc:MGI:1919053]
ENSMUSG00000004929	51.48331853	43.649902	51.87160505	45.52096245	40.65116135	37.9690261	172.5441473	186.3207251	175.3503391	137.2521927	147.6890154	142.2532829	Thop1	thimet oligopeptidase 1 [Source:MGI Symbol;Acc:MGI:1354165]
ENSMUSG00000004931	53.05357957	50.00671992	57.66932408	52.10226363	49.95336676	43.3206778	37.66162164	34.75948596	39.91057314	36.62349741	37.51063776	37.20927032	Apba3	amyloid beta (A4) precursor protein-binding, family A, member 3 [Source:MGI Symbol;Acc:MGI:1888527]
ENSMUSG00000004933	0.977920178	0.929053418	1.351603131	1.528569895	1.284327226	0.595891284	2.531888109	2.300827007	2.116233575	1.224617112	1.261730841	1.104987579	Matk	megakaryocyte-associated tyrosine kinase [Source:MGI Symbol;Acc:MGI:99259]
ENSMUSG00000004934	54.81658169	50.31112662	66.4368475	59.32258841	52.64766187	49.1017651	79.81675828	78.3914776	71.04560153	50.70250181	55.47405759	53.85497028	Pias4	protein inhibitor of activated STAT 4 [Source:MGI Symbol;Acc:MGI:2136940]
ENSMUSG00000004936	111.3611959	130.2357558	93.9343775	114.0907758	119.8555329	118.1703075	166.0491326	158.8102354	175.1141253	172.2344928	189.6630689	185.0832832	Map2k1	mitogen-activated protein kinase kinase 1 [Source:MGI Symbol;Acc:MGI:1346866]
ENSMUSG00000004937	166.1366729	153.8906759	169.3600309	159.0993827	149.8071414	129.3774816	141.0782865	139.6025923	142.1431707	122.014289	113.9418173	123.2390931	Sgta	small glutamine-rich tetratricopeptide repeat (TPR)-containing, alpha [Source:MGI Symbol;Acc:MGI:1098703]
ENSMUSG00000004939	0	0	0	0	0	0	0.020719986	0.045982576	0	0.020759421	0	0	Nmrk2	nicotinamide riboside kinase 2 [Source:MGI Symbol;Acc:MGI:1916814]
ENSMUSG00000004945	85.24702765	77.41383147	92.095116	81.8658554	76.85414119	62.00073551	60.96663311	68.30165243	49.42536464	37.763487	38.93505052	40.19656777	Tmem242	transmembrane protein 242 [Source:MGI Symbol;Acc:MGI:1917794]
ENSMUSG00000004947	23.25970217	21.6129838	24.7367239	26.48226182	25.69497401	22.13814885	17.49154021	17.23296577	17.37775399	14.40228824	16.27594569	15.27749463	Dtx2	deltex 2, E3 ubiquitin ligase [Source:MGI Symbol;Acc:MGI:1921448]
ENSMUSG00000004948	0.878132405	1.011214605	1.070268469	1.326742708	1.142289871	0.700707924	3.397231993	3.355387428	2.685511618	7.779880472	6.021690435	7.374585066	Zp3	zona pellucida glycoprotein 3 [Source:MGI Symbol;Acc:MGI:99215]
ENSMUSG00000004951	65.55157851	44.41625065	67.06318569	23.16378383	16.56488001	22.90964801	760.5051917	847.7290998	701.1440333	398.9821247	402.0317332	401.9549146	Hspb1	heat shock protein 1 [Source:MGI Symbol;Acc:MGI:96240]
ENSMUSG00000004952	37.16389613	34.83061308	42.55671699	45.00477171	43.98952195	40.93889148	9.45507891	10.47025315	10.19723778	14.5266613	12.91052626	14.392649	Rasa4	RAS p21 protein activator 4 [Source:MGI Symbol;Acc:MGI:1858600]
ENSMUSG00000004961	0.796823849	1.146979528	0.866768812	1.068900452	0.523244425	0.568993414	1.289387463	1.924083403	1.90048057	4.989180826	6.176752831	5.649319213	Syt5	synaptotagmin V [Source:MGI Symbol;Acc:MGI:1926368]
ENSMUSG00000004980	500.9391139	527.7779254	465.4306895	595.7142749	632.8551575	617.107034	1155.466723	1109.427995	1105.519494	770.855256	762.1588276	775.7137662	Hnrnpa2b1	heterogeneous nuclear ribonucleoprotein A2/B1 [Source:MGI Symbol;Acc:MGI:104819]
ENSMUSG00000004988	0.781243494	0.346016171	0.307627446	0.392561934	0.631401094	1.144344296	0.375564216	1.369266057	0.503411122	1.612624289	1.000471382	0.905390195	Fxyd4	FXYD domain-containing ion transport regulator 4 [Source:MGI Symbol;Acc:MGI:1889005]
ENSMUSG00000004994	15.54883294	13.73895478	17.50624958	18.44889522	17.10585019	14.53342457	18.99593385	24.44905979	18.48113999	17.14428932	15.99031678	14.12329803	Ccdc130	coiled-coil domain containing 130 [Source:MGI Symbol;Acc:MGI:1914986]
ENSMUSG00000004996	27.27926109	21.06796818	28.48687354	23.79339642	21.97361205	21.19368198	45.52631057	54.51640538	48.78345507	37.44687176	35.6049413	34.71609328	Mri1	methylthioribose-1-phosphate isomerase 1 [Source:MGI Symbol;Acc:MGI:1915123]
ENSMUSG00000005034	26.57595589	33.48626248	21.38943872	32.29321049	38.14503553	46.9605067	16.37515733	13.95906831	18.46393374	31.0241456	30.43058356	29.48219086	Prkacb	protein kinase, cAMP dependent, catalytic, beta [Source:MGI Symbol;Acc:MGI:97594]
ENSMUSG00000005043	5.397656461	5.961578803	5.248039274	5.2904385	7.240464398	6.94266236	0.848612161	0.622152412	0.837319539	3.142804355	3.150754727	4.001322543	Sgsh	N-sulfoglucosamine sulfohydrolase (sulfamidase) [Source:MGI Symbol;Acc:MGI:1350341]
ENSMUSG00000005045	0.779275714	0.969166127	0.740373953	1.099537426	0.792553828	1.087402307	0.923915644	0.759631885	1.224336565	1.533496197	1.484153345	1.281778309	Chd5	chromodomain helicase DNA binding protein 5 [Source:MGI Symbol;Acc:MGI:3036258]
ENSMUSG00000005054	413.8013282	373.3058864	486.1089101	434.6963571	396.5286828	313.6087879	155.9528404	160.3240839	120.7452031	122.6764036	116.3008992	115.5370097	Cstb	cystatin B [Source:MGI Symbol;Acc:MGI:109514]
ENSMUSG00000005057	4.188422799	4.773460612	3.050281248	4.271217913	5.37600173	4.93336989	2.447910922	1.860736954	2.270724597	3.611264281	3.773980858	4.477220119	Sh2b2	SH2B adaptor protein 2 [Source:MGI Symbol;Acc:MGI:1345171]
ENSMUSG00000005069	33.99424274	33.61097695	30.29703883	28.91266574	29.93606006	28.92171314	37.08782681	36.35340353	37.4079558	27.54073861	30.00004779	29.97256573	Pex5	peroxisomal biogenesis factor 5 [Source:MGI Symbol;Acc:MGI:1098808]
ENSMUSG00000005078	41.93009136	46.9098078	39.7985923	50.04700955	49.72413261	46.48745657	30.82568201	26.58793164	26.98094892	28.6630693	26.59432819	27.9754162	Jkamp	JNK1/MAPK8-associated membrane protein [Source:MGI Symbol;Acc:MGI:1915057]
ENSMUSG00000005087	107.44027	117.6024845	99.90223872	137.3527845	148.2502641	159.6457396	9.806584659	9.691161221	12.20258788	118.4955327	111.6696569	113.4976143	Cd44	CD44 antigen [Source:MGI Symbol;Acc:MGI:88338]
ENSMUSG00000005089	0.525347817	0.604965016	0.281729507	0.210105407	0.157703436	0.152437305	0	0.173477287	0	0.268520696	0.083295059	0.288213815	Slc1a2	solute carrier family 1 (glial high affinity glutamate transporter), member 2 [Source:MGI Symbol;Acc:MGI:101931]
ENSMUSG00000005102	7.795015911	9.135941287	7.259281165	9.267845126	9.900694518	11.99778963	10.19653534	9.449530381	11.26449565	9.75111118	10.814274	9.343892104	Eif2ak4	eukaryotic translation initiation factor 2 alpha kinase 4 [Source:MGI Symbol;Acc:MGI:1353427]
ENSMUSG00000005103	240.6082789	256.4873615	216.5688248	213.5567987	222.4499688	221.7979458	75.62574406	80.31181946	86.49098831	140.8794221	138.4118614	134.1878016	Wdr1	WD repeat domain 1 [Source:MGI Symbol;Acc:MGI:1337100]

ENSMUSG00000005107	2.324135368	2.848237766	2.313943767	2.129049618	2.621096753	3.134482648	0.228433225	0.337965096	0.218305583	2.117028874	2.094346534	1.795156564	Slc2a9	solute carrier family 2 (facilitated glucose transporter), member 9 [Source:MGI Symbol;Acc:MGI:2152844]
ENSMUSG00000005124	76.54541519	93.88329276	68.34539168	40.69355385	44.25656428	54.64253382	2.111693733	1.196515023	1.943906185	37.31757207	31.48114845	31.77742057	Ccn4	cellular communication network factor 4 [Source:MGI Symbol;Acc:MGI:1197008]
ENSMUSG00000005125	76.92078988	90.80845672	73.79398468	97.51558903	101.9177744	92.80627762	36.15957888	30.19678448	31.62393863	27.2654478	26.49467501	29.81231981	Ndrg1	N-myc downstream regulated gene 1 [Source:MGI Symbol;Acc:MGI:1341799]
ENSMUSG00000005131	0.801390174	0.638890603	0.441784744	0.46021178	0.550531097	0.375633474	0.880569721	0.977096795	0.918032127	0.661684236	0.564449327	0.819475029	4930550C14	RIKEN cDNA 4930550C14 gene [Source:MGI Symbol;Acc:MGI:1922561]
ENSMUSG00000005142	63.09928407	62.28177363	59.8153557	48.49205949	52.03805517	46.47598454	55.82514622	51.06930635	63.26340183	57.98666225	64.04861621	60.39305307	Man2b1	mannosidase 2, alpha B1 [Source:MGI Symbol;Acc:MGI:107286]
ENSMUSG00000005148	8.974671704	8.034624874	7.768078936	12.16772056	13.74321219	15.01350204	156.4334096	146.8544276	176.5932606	56.91305666	56.6119086	57.21165004	Klf5	Kruppel-like factor 5 [Source:MGI Symbol;Acc:MGI:1338056]
ENSMUSG00000005150	17.3739128	15.81841551	19.88689712	17.33243648	14.4325117	12.76182638	27.36021981	29.51609958	27.26367599	24.47031675	27.41021676	25.37621355	Wdr83	WD repeat domain containing 83 [Source:MGI Symbol;Acc:MGI:1915086]
ENSMUSG00000005161	281.7440406	227.3448364	347.3514127	223.4704969	205.7196787	153.0509357	240.809533	255.6053905	198.7689671	148.8156312	146.3806456	147.844519	Prdx2	peroxiredoxin 2 [Source:MGI Symbol;Acc:MGI:109486]
ENSMUSG00000005198	42.81670894	50.29548401	44.60067138	53.58059622	58.80746217	70.81427794	86.35000782	72.33065819	96.0182466	189.3197883	203.2353626	199.7947817	Polr2a	polymerase (RNA) II (DNA directed) polypeptide A [Source:MGI Symbol;Acc:MGI:98086]
ENSMUSG00000005202	0.043071559	0	0.044096347	0	0	0	0.038453307	0.042668516	0.080178482	0.231158957	0.215116369	0.038171076	Shbg	sex hormone binding globulin [Source:MGI Symbol;Acc:MGI:98295]
ENSMUSG00000005204	151.6407192	142.9043994	142.2372554	139.6466719	136.4452673	141.2058107	329.817855	322.9595477	338.0358324	386.9359764	381.6735793	388.6711687	Senp3	SUMO/sentrin specific peptidase 3 [Source:MGI Symbol;Acc:MGI:2158736]
ENSMUSG00000005220	0.200563048	0.106596305	0.110564988	0.143970878	0.048628525	0.117511727	0.151510719	0.122268477	0.244114584	0.11039933	0.064210964	0.08203565	Corin	corin, serine peptidase [Source:MGI Symbol;Acc:MGI:1349451]
ENSMUSG00000005225	4.524562371	4.886790653	3.440779626	4.334590869	4.869723263	6.288882953	8.920772827	8.447649929	9.776512391	9.126197668	9.194306342	9.85996533	Plekha8	pleckstrin homology domain containing, family A (phosphoinositide binding specific) member 8 [Source:MGI Symbol;Acc:MGI:268116
ENSMUSG00000005232	0	0	0	0	0	0	0	0	0	0.032952026	0.122660532	0.06529607	G6pc2	glucose-6-phosphatase, catalytic, 2 [Source:MGI Symbol;Acc:MGI:1277193]
ENSMUSG00000005233	37.59243008	35.24805193	35.10480579	49.24556757	46.30667399	44.01719778	83.77350101	86.08018612	83.36762154	44.99613561	48.0691011	46.43394517	Spc25	SPC25, NDC80 kinetochore complex component, homolog (S. cerevisiae) [Source:MGI Symbol;Acc:MGI:1913692]
ENSMUSG00000005237	0.331860045	0.494003824	0.40604971	0.385219595	0.442218313	0.517873915	1.40189648	0.898061502	1.454004184	1.274244184	1.327299562	1.068811663	Dnah2	dynein, axonemal, heavy chain 2 [Source:MGI Symbol;Acc:MGI:107731]
ENSMUSG00000005251	1.174266724	1.214245997	1.067264207	1.386530563	1.460363389	1.651934763	3.744137824	3.786590202	4.583737476	4.14782595	4.12926384	4.470607874	Ripk4	receptor-interacting serine-threonine kinase 4 [Source:MGI Symbol;Acc:MGI:1919638]
ENSMUSG00000005262	75.87571676	72.67969636	69.04977975	64.61528607	61.47169626	63.58300363	55.19564686	58.09711362	56.25748051	60.698902	65.95827176	68.42692533	Ufd1	ubiquitin recognition factor in ER-associated degradation 1 [Source:MGI Symbol;Acc:MGI:109353]
ENSMUSG00000005267	1.319004113	1.852318341	1.482131782	1.441022008	1.554828145	1.776535698	0.560067031	0.398372408	0.568922526	2.201367796	2.182486244	2.494676008	Zfp287	zinc finger protein 287 [Source:MGI Symbol;Acc:MGI:2176561]
ENSMUSG00000005268	0.856117943	1.025298373	0.315535417	0.191739647	0.37778539	0.378211937	0.030572904	0.101772816	0.031873579	0.398204188	0.313558003	0.318659371	Prlr	prolactin receptor [Source:MGI Symbol;Acc:MGI:97763]
ENSMUSG00000005299	30.57180166	33.0251975	32.25774991	33.82845034	33.14706407	29.62684647	34.67149554	32.88682952	37.18849784	26.38082463	28.41291399	27.833052	Letm1	leucine zipper-EF-hand containing transmembrane protein 1 [Source:MGI Symbol;Acc:MGI:1932557]
ENSMUSG00000005312	61.28244742	69.75417248	53.55444515	70.89995685	76.23775087	86.28601381	102.1098928	93.88842267	115.8196442	114.5053554	115.7963062	112.2417663	Ubqln1	ubiquilin 1 [Source:MGI Symbol;Acc:MGI:1860276]
ENSMUSG00000005320	0.266889311	0.648822052	0.121439697	0.110691875	0.155783316	0.135523173	6.168615437	5.934129039	6.348245934	2.122010551	1.999425854	1.550545401	Fgfr4	fibroblast growth factor receptor 4 [Source:MGI Symbol;Acc:MGI:95525]
ENSMUSG00000005338	4.338557071	5.551190547	5.447124713	2.18261643	1.275587406	2.230265305	0.446313657	0.247619032	0.681334113	2.331636137	2.214404335	1.914556625	Cadm3	cell adhesion molecule 3 [Source:MGI Symbol;Acc:MGI:2137858]
ENSMUSG00000005339	0	0	0	0	0	0	0	0	0	0	0	0	Fcer1a	Fc receptor, IgE, high affinity I, alpha polypeptide [Source:MGI Symbol;Acc:MGI:95494]
ENSMUSG00000005354	162.1972295	130.9868543	171.7779639	154.0654438	129.9297665	114.3890134	149.7579077	168.1722898	138.9987153	95.50578374	100.210301	94.58747843	Txn2	thioredoxin 2 [Source:MGI Symbol;Acc:MGI:1929468]
ENSMUSG00000005355	0.151775971	0.065541687	0.077693563	0.035408711	0.099665605	0.101154385	0.084688832	0.037588931	0.08829178	0.305460051	0.584314106	0.638911101	Casp14	caspase 14 [Source:MGI Symbol;Acc:MGI:1335092]
ENSMUSG00000005357	1.354720007	0.520009909	1.452997758	0.120400233	0.271114353	0.294818776	0.575934844	1.086415908	0.780568171	1.702241391	1.933144704	1.486440063	Slc1a6	solute carrier family 1 (high affinity aspartate/glutamate transporter), member 6 [Source:MGI Symbol;Acc:MGI:1096331]
ENSMUSG00000005360	4.96962802	5.751539829	4.423122045	1.887656242	1.495574084	2.682029547	14.80409931	14.17563014	17.96749514	10.25303341	8.418940551	8.321516055	Slc1a3	solute carrier family 1 (glial high affinity glutamate transporter), member 3 [Source:MGI Symbol;Acc:MGI:99917]
ENSMUSG00000005362	6.64306546	9.928504538	6.180958615	8.648731265	12.21961115	14.04161466	9.806513663	8.261133318	10.05457347	16.0923407	16.94200398	16.78492004	Crbn	cereblon [Source:MGI Symbol;Acc:MGI:1913277]
ENSMUSG00000005364	0	0	0	0.015952911	0	0	0	0	0	0	0	0	Il5ra	interleukin 5 receptor, alpha [Source:MGI Symbol;Acc:MGI:96558]
ENSMUSG00000005370	37.14217123	44.39569376	30.60498332	47.64395137	51.2906133	61.527712	318.3503452	302.8868884	380.9989661	342.6495095	350.0092292	347.313375	Msh6	mutS homolog 6 [Source:MGI Symbol;Acc:MGI:1343961]
ENSMUSG00000005371	30.2046803	36.84228755	22.94187209	35.05741281	41.37006287	47.01673508	19.1485859	17.59132358	20.47151378	29.54393567	30.84796978	28.58716225	Fbxo11	F-box protein 11 [Source:MGI Symbol;Acc:MGI:2147134]
ENSMUSG00000005373	2.499494492	2.545270366	2.093697921	2.332487191	2.843153962	2.643319955	4.094141913	4.297373028	4.364453863	3.067211914	3.30141147	3.350130369	Mlxipl	MLX interacting protein-like [Source:MGI Symbol;Acc:MGI:1927999]
ENSMUSG00000005374	23.81650618	24.56906333	22.45283047	25.94788858	25.69033589	31.47394417	41.63971728	38.81154214	44.51958264	35.46999853	36.42814134	34.79099884	Tbl2	transducin (beta)-like 2 [Source:MGI Symbol;Acc:MGI:1351652]
ENSMUSG00000005378	88.87568676	67.30255063	89.81240849	79.12597103	69.62564537	56.25914772	135.2736974	148.1077248	122.4497454	93.85300891	98.06545586	94.92442317	Bud23	BUD23, rRNA methyltransferase and ribosome maturation factor [Source:MGI Symbol;Acc:MGI:1913388]
ENSMUSG00000005397	170.2032072	245.5331534	141.7998421	245.7671735	322.1848728	434.803873	27.33764167	22.71437145	33.77900255	57.66073641	58.84856398	55.51143597	Nid1	nidogen 1 [Source:MGI Symbol;Acc:MGI:97342]
ENSMUSG00000005410	40.42396205	33.01648565	35.08313272	46.36029357	47.06278664	48.05435442	360.530804	347.4038573	353.3300832	257.5170336	269.6568164	274.0456772	Mcm5	minichromosome maintenance complex component 5 [Source:MGI Symbol;Acc:MGI:103197]
ENSMUSG00000005413	457.3061812	429.4573206	433.9447342	201.1785169	191.8653374	176.9293759	147.2350892	152.3910425	158.8297409	339.5282289	334.5760689	344.4976065	Hmox1	heme oxygenase 1 [Source:MGI Symbol;Acc:MGI:96163]
ENSMUSG00000005417	52.60005203	62.94549353	48.83795349	56.44607208	58.42712488	60.85971983	25.67038494	21.02530005	26.33011019	44.45247578	47.57860247	47.85911115	Mprip	myosin phosphatase Rho interacting protein [Source:MGI Symbol;Acc:MGI:1349438]
ENSMUSG00000005442	59.94914993	68.05246538	53.79533797	64.11895447	69.78779594	80.94592574	45.83826506	39.80332535	55.81624991	54.47260826	60.13040311	57.233711	Cic	capicua transcriptional repressor [Source:MGI Symbol;Acc:MGI:1918972]
ENSMUSG00000005447	91.65733296	67.10894449	106.7245237	88.74125578	79.75539667	62.25374539	61.58257534	65.91625933	51.30007296	40.47188216	49.52513839	49.43518314	Pafah1b3	platelet-activating factor acetylhydrolase, isoform 1b, subunit 3 [Source:MGI Symbol;Acc:MGI:108414]
ENSMUSG00000005465	0.801118343	0.485541554	0.690677122	0.472162405	0.465151732	0.979527319	3.576101065	3.654837505	4.140309148	2.055457293	1.438992547	1.718876644	Il27ra	interleukin 27 receptor, alpha [Source:MGI Symbol;Acc:MGI:1355318]
ENSMUSG00000005469	72.73640461	67.36900955	67.03707497	68.00749538	65.96774201	60.41261258	84.20753376	84.04574106	83.14883689	49.35589594	49.7499908	48.83833386	Prkaca	protein kinase, cAMP dependent, catalytic, alpha [Source:MGI Symbol;Acc:MGI:97592]
ENSMUSG00000005470	22.55735877	23.32330735	23.18305288	33.70458594	33.33542666	32.27744457	89.13022202	85.94059799	89.321744	63.71896655	59.04357655	57.77712832	Asf1b	anti-silencing function 1B histone chaperone [Source:MGI Symbol;Acc:MGI:1914179]
ENSMUSG00000005474	0.134885542	0	0	0	0.070859433	0.051369938	0.06021137	0	0	0	0	0	Myl10	myosin, light chain 10, regulatory [Source:MGI Symbol;Acc:MGI:1891705]
ENSMUSG00000005481	42.12592949	34.00456955	40.83822374	40.97742061	36.1996384	32.22106178	194.9996149	198.5901897	185.3254855	135.401122	142.2548953	142.2376472	Ddx39	DEAD (Asp-Glu-Ala-Asp) box polypeptide 39 [Source:MGI Symbol;Acc:MGI:1915528]
ENSMUSG00000005483	31.74546213	28.8657691	32.68149844	32.9052603	29.84521803	30.24173733	33.60807233	33.18258298	33.87405158	34.18520911	33.47806412	31.30157407	Dnajb1	DnaJ heat shock protein family (Hsp40) member B1 [Source:MGI Symbol;Acc:MGI:1931874]
ENSMUSG00000005493	0.382012402	0.799830761	0.604429583	0.891219242	0.820974304	1.256469718	0.465070873	0.82568228	0.533342212	0.170850537	0.101177614	0.230828721	Msh4	mutS homolog 4 [Source:MGI Symbol;Acc:MGI:1860077]
ENSMUSG00000005501	9.019007226	10.99778271	7.828152865	9.17400214	10.03423052	12.53620343	5.482188437	5.030288717	6.058893935	9.7309198	10.12456446	9.641149487	Usp40	ubiquitin specific peptidase 40 [Source:MGI Symbol;Acc:MGI:2443184]
ENSMUSG00000005503	0	0	0.0225216	0	0	0	0.039278992	0.130754134	0.040950053	3.42377615	3.845370067	3.528658358	Evx1	even-skipped homeobox 1 [Source:MGI Symbol;Acc:MGI:95461]
ENSMUSG00000005505	0.276710533	1.473739613	0.495764874	1.258830897	1.417302585	1.844197303	1.235204311	0.959424225	1.223366365	1.175677429	1.900252236	1.532673018	Kbtbd4	kelch repeat and BTB (POZ) domain containing 4 [Source:MGI Symbol;Acc:MGI:1914386]
ENSMUSG00000005506	3.40718704	5.321462915	1.942560108	4.474190276	6.677283625	11.02568404	10.50989606	7.498430297	14.16627171	24.60930424	24.70158624	24.56308725	Celf1	CUGBP, Elav-like family member 1 [Source:MGI Symbol;Acc:MGI:1342295]
ENSMUSG00000005510	161.3260067	121.9571173	181.8259783	129.2044573	114.0570897	80.76119698	148.8713184	157.8011325	122.3851579	91.69840699	81.38333024	83.5318213	Ndufs3	NADH:ubiquinone oxidoreductase core subunit S3 [Source:MGI Symbol;Acc:MGI:1915599]
ENSMUSG00000005514	227.2043333	211.5092082	229.3250114	192.5779557	184.4889326	167.49203	103.5095664	97.35391272	103.0195156	110.3805828	104.9727294	102.6470642	Por	P450 (cytochrome) oxidoreductase [Source:MGI Symbol;Acc:MGI:97744]
ENSMUSG00000005533	35.25287243	40.21196177	31.58017817	37.42796639	41.15720081	53.58586524	8.760279315	8.04548599	9.431044613	21.44967938	20.70746418	20.66147262	Igf1r	insulin-like growth factor I receptor [Source:MGI Symbol;Acc:MGI:96433]
ENSMUSG00000005534	7.920732701	9.151585399	7.202150309	8.748867789	10.62826169	13.76831168	5.064527473	6.182974835	5.932295954	12.71498582	13.06534456	12.03518535	Insr	insulin receptor [Source:MGI Symbol;Acc:MGI:96575]
ENSMUSG00000005540	0	0	0	0.035191479	0	0.057447889	0	0	0.105300135	0	0.094172328	0.1336824	Fcer2a	Fc receptor, IgE, low affinity II, alpha polypeptide [Source:MGI Symbol;Acc:MGI:95497]
ENSMUSG00000005547	0	0.155165498	0	0	0	0	0	0	0	0	0.07477427	0	Cyp2a5	cytochrome P450, family 2, subfamily a, polypeptide 5 [Source:MGI Symbol;Acc:MGI:88597]
ENSMUSG00000005553	0.079424989	0.022865489	0	0	0.125173009	0.060496577	0.744542626	1.278578727	1.219771112	0.497306441	0.479321361	0.580704132	Atp4a	ATPase, H+/K+ exchanging, gastric, alpha polypeptide [Source:MGI Symbol;Acc:MGI:88113]
ENSMUSG00000005566	415.6722334	344.442722	424.907942	395.9689497	341.0766196	319.3357793	1795.11472	1842.744939	1808.001102	1401.724114	1419.518523	1398.911272	Trim28	tripartite motif-containing 28 [Source:MGI Symbol;Acc:MGI:109274]
ENSMUSG00000005575	248.1097823	193.1362505	267.6636723	207.2393459	172.6229091	137.903853	118.2096134	126.6195726	95.78156897	73.91786504	69.35708237	66.35385118	Ube2m	ubiquitin-conjugating enzyme E2M [Source:MGI Symbol;Acc:MGI:108278]
ENSMUSG00000005580	0.287103556	0.522584216	0.322379791	0.553125825	0.6422189	1.312089505	0.463028805	0.412863355	0.74133169	1.027229408	1.225760482	1.296814236	Adcy9	adenylate cyclase 9 [Source:MGI Symbol;Acc:MGI:108450]
ENSMUSG00000005583	1.930140596	2.177753972	1.536938498	3.038346533	3.70761469	4.766860274	0.052217745	0.096569659	0.127024948	0.837074031	0.535549112	1.157636889	Mef2c	myocyte enhancer factor 2C [Source:MGI Symbol;Acc:MGI:99458]
ENSMUSG00000005609	73.20141319	72.54203227	63.63737245	73.15063266	76.82191232	79.84286687	51.11192185	53.04058819	55.23304889	81.56555414	83.63460943	83.25972514	Ctr9	CTR9 homolog, Paf1/RNA polymerase II complex component [Source:MGI Symbol;Acc:MGI:109345]
ENSMUSG00000005610	646.9640491	791.738005	528.4596306	778.0499125	899.0821488	1046.55218	603.0781802	539.2687981	713.7026607	985.3913761	986.9780945	969.2596215	Eif4g2	eukaryotic translation initiation factor 4, gamma 2 [Source:MGI Symbol;Acc:MGI:109207]
ENSMUSG00000005611	0.273100416	0.7687513	0.497063472	0.368120742	0.828925442	0.739611185	0.013545435	0.045090812	0	0.651418331	0.378880911	0.726084927	Mrvi1	MRV integration site 1 [Source:MGI Symbol;Acc:MGI:1338023]

ENSMUSG00000005615	17.36653039	24.48942009	12.59581115	20.93128214	25.51315713	30.45998112	9.175706255	7.684178854	11.17044437	18.48270393	21.27131413	19.72523312	Pcyt1a	phosphate cytidylyltransferase 1, choline, alpha isoform [Source:MGI Symbol;Acc:MGI:88557]
ENSMUSG00000005621	11.89904659	15.20520372	10.68885999	14.47101253	15.56682505	18.05348594	14.73540344	12.92844622	14.3798247	13.20126946	15.14469133	14.52520098	Zfp592	zinc finger protein 592 [Source:MGI Symbol;Acc:MGI:2443541]
ENSMUSG00000005625	215.8979274	169.8445089	239.5978333	187.2119852	160.8088387	132.0900273	239.8705652	268.4441853	219.8708007	180.9518036	180.4278675	179.252042	Psmd4	proteasome (prosome, macropain) 26S subunit, non-ATPase, 4 [Source:MGI Symbol;Acc:MGI:1201670]
ENSMUSG00000005628	2.228172557	1.482494138	1.723563299	1.709641749	2.184982015	2.225163657	1.945055902	1.471548252	1.751286258	1.727308042	0.824323947	1.711373168	Tmod4	tropomodulin 4 [Source:MGI Symbol;Acc:MGI:1355285]
ENSMUSG00000005640	0	0.093021619	0.027567115	0	0	0.082037599	0.024039333	0.013337249	0.037593071	0.048170171	0.044827128	0.047725788	Insrr	insulin receptor-related receptor [Source:MGI Symbol;Acc:MGI:1346037]
ENSMUSG00000005649	0	0	0	0.137629744	0.154955676	0.056167987	0	0	0	0	0	0	Cabp5	calcium binding protein 5 [Source:MGI Symbol;Acc:MGI:1352746]
ENSMUSG00000005656	199.3022815	217.4597561	181.3524641	222.0024015	224.8617043	234.1451667	110.1260995	110.5461545	122.5680077	122.7596949	134.5100791	124.8224064	Snx6	sorting nexin 6 [Source:MGI Symbol;Acc:MGI:1919433]
ENSMUSG00000005667	58.2355432	51.4442248	48.85052542	95.0097355	94.71221272	88.18224324	112.2621807	117.9990152	105.0188064	79.20582651	75.03697667	69.41690993	Mthfd2	methylenetetrahydrofolate dehydrogenase (NAD+ dependent), methenyltetrahydrofolate cyclohydrolase [Source:MGI Symbol;Acc:M
ENSMUSG00000005672	0.725912911	0.626944525	0.334432941	1.202402753	1.449106788	1.907555819	19.16692213	18.31958617	24.76257112	29.15410696	30.60522553	29.35156097	Kit	KIT proto-oncogene receptor tyrosine kinase [Source:MGI Symbol;Acc:MGI:96677]
ENSMUSG00000005674	1.752398514	3.954144065	1.454669792	2.939137343	2.712001486	2.885989709	4.038105189	4.598055118	3.416410834	3.240868935	2.976526251	3.651692579	Tomm40l	translocase of outer mitochondrial membrane 40-like [Source:MGI Symbol;Acc:MGI:3589112]
ENSMUSG00000005677	0.024119108	0.111097569	0	0	0	0.018371088	0	0	0.022449076	0	0	0	Nr1i3	nuclear receptor subfamily 1, group I, member 3 [Source:MGI Symbol;Acc:MGI:1346307]
ENSMUSG00000005681	0.354874126	0.204327899	0.181658764	0.082790676	0	0	0.396029423	0.790995112	0	0.47613979	0.590793822	0.235873637	Apoa2	apolipoprotein A-II [Source:MGI Symbol;Acc:MGI:88050]
ENSMUSG00000005682	10.3423506	10.33418791	8.52588618	11.14927564	12.29249718	13.51497546	19.64024337	17.17200525	21.44845646	14.94612815	15.37955469	15.62933525	Pan2	PAN2 poly(A) specific ribonuclease subunit [Source:MGI Symbol;Acc:MGI:1918984]
ENSMUSG00000005683	104.0230019	109.4100097	92.44286703	119.5530868	129.007616	128.5537471	91.8199227	85.49609318	99.37322353	92.50092694	93.8245099	92.89509969	Cs	citrate synthase [Source:MGI Symbol;Acc:MGI:88529]
ENSMUSG00000005686	33.14168318	39.82134751	28.34645873	28.8048142	32.18715222	36.89287005	3.874637385	3.908518937	5.184356184	11.27236556	14.83676395	12.4435854	Ampd3	adenosine monophosphate deaminase 3 [Source:MGI Symbol;Acc:MGI:1096344]
ENSMUSG00000005687	52.54763933	47.63950636	54.7822955	50.29279179	50.94144028	43.68648784	146.5129658	156.2868371	130.5289106	73.98720239	76.85621157	72.36730571	Bcas2	breast carcinoma amplified sequence 2 [Source:MGI Symbol;Acc:MGI:1915433]
ENSMUSG00000005696	0	0	0	0	0	0	0	0.167160187	0	0.120746561	0.084275001	0	Sh2d1a	SH2 domain containing 1A [Source:MGI Symbol;Acc:MGI:1328352]
ENSMUSG00000005698	38.16780194	46.3564081	33.19782919	49.95591897	55.28507771	65.54804127	115.7105498	101.4424078	125.3395735	146.190077	146.8247504	145.1305395	Ctcf	CCCTC-binding factor [Source:MGI Symbol;Acc:MGI:109447]
ENSMUSG00000005699	9.575824794	7.608186072	10.488631	9.989592153	8.962582063	7.452984285	13.62626019	14.87140848	11.28693289	6.695185598	7.588026098	10.33924238	Pard6a	par-6 family cell polarity regulator alpha [Source:MGI Symbol;Acc:MGI:1927223]
ENSMUSG00000005705	0.124720255	0.861730707	0.191531523	0.581934461	0.982789529	0.997470193	0.946452926	0.6795425	0.928675977	0.278898307	1.141987334	0.994771427	Agrp	agouti related neuropeptide [Source:MGI Symbol;Acc:MGI:892013]
ENSMUSG00000005716	0	0.256733242	0	0	0	0	0	0	0	0	0	0	Pvalb	parvalbumin [Source:MGI Symbol;Acc:MGI:97821]
ENSMUSG00000005718	7.456927633	7.890789373	8.081404213	9.184206874	8.258193984	7.803338247	23.63068307	23.49246563	20.54043134	15.71366621	18.31385905	17.47386031	Tfap4	transcription factor AP4 [Source:MGI Symbol;Acc:MGI:103239]
ENSMUSG00000005732	64.86225022	37.71404964	75.83747528	46.86228911	38.49421166	31.3543539	134.7372481	141.3300074	97.00023457	64.35302298	57.53750541	59.55906648	Ranbp1	RAN binding protein 1 [Source:MGI Symbol;Acc:MGI:96269]
ENSMUSG00000005763	0.04906327	0	0.050230616	0.091570075	0.051548824	0.018685303	0	0.02430208	0	0.043885937	0.061260334	0	Cd247	CD247 antigen [Source:MGI Symbol;Acc:MGI:88334]
ENSMUSG00000005774	1.918336226	3.634264569	1.119256597	2.598619131	4.854584521	6.394547651	2.54134701	1.879951097	4.146987885	4.29899145	4.447061721	4.423855673	Rfx5	regulatory factor X, 5 (influences HLA class II expression) [Source:MGI Symbol;Acc:MGI:1858421]
ENSMUSG00000005779	465.5582317	367.7323064	504.4238765	420.2691772	340.3765829	283.9144811	561.1472143	626.6247437	482.3361476	363.118299	361.9170325	380.275958	Psmb4	proteasome (prosome, macropain) subunit, beta type 4 [Source:MGI Symbol;Acc:MGI:1098257]
ENSMUSG00000005800	12.01248918	15.26329409	12.08030779	0.778232354	0.54063556	1.108234925	0.57028237	0.984349473	1.27166378	12.60183311	12.68729733	11.82513168	Mmp8	matrix metallopeptidase 8 [Source:MGI Symbol;Acc:MGI:1202395]
ENSMUSG00000005802	0.209457173	0.333086684	0.102114627	0.363000843	1.089862102	3.122420603	0.062332865	0.029642453	0.092835307	0.74049634	0.855154885	0.813219108	Slc30a4	solute carrier family 30 (zinc transporter), member 4 [Source:MGI Symbol;Acc:MGI:1345282]
ENSMUSG00000005803	40.2819886	37.56945865	38.89719964	40.46414304	41.53568901	34.99276478	2.563467051	2.555901893	2.130274017	8.337818634	8.763703575	9.367270591	Sqor	sulfide quinone oxidoreductase [Source:MGI Symbol;Acc:MGI:1929899]
ENSMUSG00000005804	38.67692755	40.20055041	31.51813825	47.33564708	50.90627243	53.30416187	25.22333814	23.25923614	30.80006445	34.27607571	35.2762434	29.81561453	Bloc1s6	biogenesis of lysosomal organelles complex-1, subunit 6, pallidin [Source:MGI Symbol;Acc:MGI:1927580]
ENSMUSG00000005813	58.36653383	55.81904617	45.25095088	61.24088252	62.89184423	67.42084245	70.58402779	66.43252863	76.33858678	72.78676634	76.7991779	75.59134984	Metap1	methionyl aminopeptidase 1 [Source:MGI Symbol;Acc:MGI:1922874]
ENSMUSG00000005823	47.45307118	39.57491237	49.66064331	41.11471452	41.54344379	34.20052861	10.76368327	10.83039765	11.15623807	13.96706941	11.94545412	11.78427087	Gpr108	G protein-coupled receptor 108 [Source:MGI Symbol;Acc:MGI:1925558]
ENSMUSG00000005824	0.1164821	0.348751328	0.453163375	0.065219509	0	0.017744462	0.020798513	0	0.043366704	0.145866684	0.252094955	0.144521025	Tnfsf14	tumor necrosis factor (ligand) superfamily, member 14 [Source:MGI Symbol;Acc:MGI:1355317]
ENSMUSG00000005836	1.681446353	2.094982723	1.213482869	0.900302194	0.897794919	0.881812214	1.132020043	0.81920271	1.05189956	5.596904783	5.34614477	6.449126738	Gata6	GATA binding protein 6 [Source:MGI Symbol;Acc:MGI:107516]
ENSMUSG00000005846	129.0387948	112.3194009	116.6285713	122.4637524	113.6582234	111.0477662	376.5239005	393.2951045	389.8493871	321.6375064	318.4283387	322.8062051	Rsl1d1	ribosomal L1 domain containing 1 [Source:MGI Symbol;Acc:MGI:1913659]
ENSMUSG00000005864	0.025074876	0	0.025671474	0.023399463	0.026345174	0.038198159	0	0.024840238	0	0	0	0.066665917	Cnga2	cyclic nucleotide gated channel alpha 2 [Source:MGI Symbol;Acc:MGI:108040]
ENSMUSG00000005871	11.75164833	15.56602708	9.226037306	14.69768811	18.89366512	25.78635209	17.06018165	15.03968589	22.02703334	34.67547914	35.99427038	35.65066844	Apc	APC, WNT signaling pathway regulator [Source:MGI Symbol;Acc:MGI:88039]
ENSMUSG00000005873	305.811308	286.7301805	320.2428222	298.3780535	299.37398	274.7947719	63.03431856	62.21960753	59.12228335	75.99778553	72.78147404	72.68426584	Reep5	receptor accessory protein 5 [Source:MGI Symbol;Acc:MGI:1270152]
ENSMUSG00000005881	321.8246404	267.9495845	335.428153	272.6675561	231.7704696	188.0669008	146.7807234	156.9993344	136.2553778	113.5023912	116.9721925	121.2794448	Ergic3	ERGIC and golgi 3 [Source:MGI Symbol;Acc:MGI:1913616]
ENSMUSG00000005882	33.64353418	34.9577685	30.83657514	35.25285652	35.24113005	35.81916586	30.21766868	33.63078693	30.93560444	26.09302287	23.94372227	27.38362919	Uqcc1	ubiquinol-cytochrome c reductase complex assembly factor 1 [Source:MGI Symbol;Acc:MGI:1929472]
ENSMUSG00000005883	2.501656269	2.277192579	1.951373209	2.934805689	4.50581246	5.353452983	0.042541367	0.023602352	0	0.23442283	0.118992942	0.274489347	Spo11	SPO11 meiotic protein covalently bound to DSB [Source:MGI Symbol;Acc:MGI:1349669]
ENSMUSG00000005886	7.254580017	11.66910983	6.405456541	10.86519918	13.99401112	19.36425186	7.322010544	6.527597849	9.669911102	12.19987278	13.8240572	13.00578105	Ncoa2	nuclear receptor coactivator 2 [Source:MGI Symbol;Acc:MGI:1276533]
ENSMUSG00000005891	0.049119278	0	0	0	0	0	0	0	0	0	0	0	Prl4a1	prolactin family 4, subfamily a, member 1 [Source:MGI Symbol;Acc:MGI:1206587]
ENSMUSG00000005892	1.461227549	0.250611712	0.031829661	0.348151542	0.16332485	2.415426376	97.81354462	97.54050554	97.75002445	553.8207011	546.2849888	541.4376518	Trh	thyrotropin releasing hormone [Source:MGI Symbol;Acc:MGI:98823]
ENSMUSG00000005893	0.835345595	1.905025153	0.670761328	1.360357643	2.75345677	8.084341596	1.14060092	0.843754884	2.103838184	7.530572788	8.316823008	8.651393679	Nr2c2	nuclear receptor subfamily 2, group C, member 2 [Source:MGI Symbol;Acc:MGI:1352466]
ENSMUSG00000005897	5.969445142	7.153275143	5.289372295	6.927888781	7.704516226	8.331986937	1.650215562	1.696028447	2.256290227	4.634818486	5.095076653	4.323519475	Nr2c1	nuclear receptor subfamily 2, group C, member 1 [Source:MGI Symbol;Acc:MGI:1352465]
ENSMUSG00000005899	17.81918116	18.13369111	18.85596584	19.55038247	19.76200968	20.07825234	67.84774387	60.71161401	70.43422207	52.69700693	57.10769873	54.57759232	Smpd4	sphingomyelin phosphodiesterase 4 [Source:MGI Symbol;Acc:MGI:1924876]
ENSMUSG00000005907	8.364068908	9.595982507	7.231038909	7.776303483	10.12223687	12.7415199	8.71179196	8.960941345	13.638048	17.95550915	18.02447513	17.8996788	Pex1	peroxisomal biogenesis factor 1 [Source:MGI Symbol;Acc:MGI:1918632]
ENSMUSG00000005917	1.72464727	1.514761008	1.556193621	1.377547983	1.888798627	1.569679657	0.091339667	0.086873331	0.095225564	0.052293433	0.060830291	0.103622024	Otx1	orthodenticle homeobox 1 [Source:MGI Symbol;Acc:MGI:97450]
ENSMUSG00000005936	47.65256688	53.76699698	47.04771942	51.36131972	51.24899236	54.09349768	51.58513928	47.51069282	56.04595955	54.20891887	57.58290822	52.58460466	Kctd20	potassium channel tetramerisation domain containing 20 [Source:MGI Symbol;Acc:MGI:1914239]
ENSMUSG00000005947	0.482457621	0.478052693	0.425015191	0.492102597	0.589417449	0.529853789	1.82307782	2.289680476	1.524687385	1.525468289	1.746481282	1.640659524	Itgae	integrin alpha E, epithelial-associated [Source:MGI Symbol;Acc:MGI:1298377]
ENSMUSG00000005949	27.41117611	30.27837797	26.4975941	31.42565422	31.24101835	35.46225199	9.216414066	9.731396536	12.15435631	18.36267979	17.2351882	19.13161452	Ctns	cystinosis, nephropathic [Source:MGI Symbol;Acc:MGI:1932872]
ENSMUSG00000005950	2.220639117	1.461245769	2.096320217	0.915025634	0.727211555	0.658995053	3.012424884	2.771251493	3.784805874	3.353509134	3.984987486	2.939198418	P2rx5	purinergic receptor P2X, ligand-gated ion channel, 5 [Source:MGI Symbol;Acc:MGI:2137026]
ENSMUSG00000005951	3.509738944	3.656726259	2.955482888	3.91326267	4.342041875	3.807433895	1.125859071	1.174016992	1.230323511	0.883374934	1.277367381	1.333035863	Shpk	sedoheptulokinase [Source:MGI Symbol;Acc:MGI:1921887]
ENSMUSG00000005952	0.081377755	0.187421336	0.124970923	0.265791343	0.064125274	0.201447881	0	0	0	0.10918572	0.033869386	0.054089227	Trpv1	transient receptor potential cation channel, subfamily V, member 1 [Source:MGI Symbol;Acc:MGI:1341787]
ENSMUSG00000005958	9.825961359	11.0087341	10.00527199	6.306049075	5.497301695	5.782061349	22.61187618	23.84306485	25.42279198	13.69130873	15.57576407	14.5552637	Ephb3	Eph receptor B3 [Source:MGI Symbol;Acc:MGI:104770]
ENSMUSG00000005968	10.20172707	9.52616367	8.379504114	11.26588746	13.35979516	10.13055665	8.246667042	8.687333091	9.685800232	8.183922197	8.735229821	8.626533466	Tuft1	tuftelin 1 [Source:MGI Symbol;Acc:MGI:109572]
ENSMUSG00000005973	108.7586941	120.5155224	93.71830176	89.2860816	94.06779552	93.25432577	57.15456779	55.75613114	57.73319245	59.41892211	57.91170126	55.65175538	Rcn1	reticulocalbin 1 [Source:MGI Symbol;Acc:MGI:104559]
ENSMUSG00000005980	0.970567395	1.583349183	0.53823238	1.245361625	1.104714546	1.601737851	1.083125585	0.761175852	1.27976612	2.893832058	2.894973018	3.261366848	Dnase1	deoxyribonuclease I [Source:MGI Symbol;Acc:MGI:103157]
ENSMUSG00000005981	101.4210745	85.97230093	96.36141431	95.73766391	90.08526682	86.24200404	283.1436207	274.9032419	280.6376146	253.416055	257.7689897	249.6008243	Trap1	TNF receptor-associated protein 1 [Source:MGI Symbol;Acc:MGI:1915265]
ENSMUSG00000005982	19.20843026	20.68796179	17.3253025	18.78569047	20.98205269	20.06759698	23.37828491	17.99581455	23.02919524	18.3651806	20.76243853	15.60144004	Naa60	N(alpha)-acetyltransferase 60, NatF catalytic subunit [Source:MGI Symbol;Acc:MGI:1922013]
ENSMUSG00000005983	1.738975156	1.180972911	2.465100011	1.04024554	1.827071933	1.324545912	0.875779057	1.766874516	1.4525599	1.116749283	0.70520273	1.066931012	1700037C18	RIKEN cDNA 1700037C18 gene [Source:MGI Symbol;Acc:MGI:1920511]
ENSMUSG00000005986	17.07306328	13.54018067	18.59735436	16.06818529	15.18418137	13.00424338	16.54243716	18.39186322	17.78832872	10.35463055	10.09054106	9.775186227	Ankrd13d	ankyrin repeat domain 13 family, member D [Source:MGI Symbol;Acc:MGI:1915673]
ENSMUSG00000005994	0	0	0	0	0	0.018933576	0.04438458	0	0	0.022234527	0.020691436	0	Tyrp1	tyrosinase-related protein 1 [Source:MGI Symbol;Acc:MGI:98881]
ENSMUSG00000006005	62.33446077	83.81622841	49.9616984	78.51147495	102.6146128	125.2673695	110.7550213	96.62292002	139.3580232	217.0424039	213.117689	217.6172015	Tpr	translocated promoter region, nuclear basket protein [Source:MGI Symbol;Acc:MGI:1922066]
ENSMUSG00000006007	0.319123519	0.520606777	0.653432636	0.148900412	0.027940864	0.222814726	0.047484368	0	0.173265798	0.356810566	0.664095343	0.518494378	Pdc	phosducin [Source:MGI Symbol;Acc:MGI:98090]
ENSMUSG00000006010	17.36152266	22.58587938	17.97292144	19.07120726	26.40756018	26.7614541	10.74405316	10.81837778	11.85472915	16.02762809	14.69358594	14.14157805	Odr4	odr4 GPCR localization factor homolog [Source:MGI Symbol;Acc:MGI:2385108]

ENSMUSG00000006014	0.636150128	0.508721926	0.7417422	0.791526462	0.90973618	0.794113847	0.61526871	0.43763704	0.773022637	0.52160283	0.279474593	0.375847931	Prg4	proteoglycan 4 (megakaryocyte stimulating factor, articular superficial zone protein) [Source:MGI Symbol;Acc:MGI:1891344]
ENSMUSG00000006019	6.008664186	5.758331713	6.303008619	6.246932822	6.93448432	5.856526024	7.812580444	6.857954254	6.706137212	4.448780868	5.158256668	5.074857044	Dhx34	DEAH (Asp-Glu-Ala-His) box polypeptide 34 [Source:MGI Symbol;Acc:MGI:1918973]
ENSMUSG00000006021	19.38646155	18.21050574	23.34392746	22.35651513	21.25177358	15.96683057	16.51041347	19.15300605	17.55512258	10.38564172	8.659024841	13.03689049	Kptn	kaptin [Source:MGI Symbol;Acc:MGI:1890380]
ENSMUSG00000006024	271.7974288	255.4527809	278.5142266	223.6471234	210.6984735	194.0400586	118.0351986	131.1676199	120.5565832	147.5299233	148.5932802	137.6853233	Napa	N-ethylmaleimide sensitive fusion protein attachment protein alpha [Source:MGI Symbol;Acc:MGI:104563]
ENSMUSG00000006050	111.1351953	88.4884279	112.62247	100.8298443	82.5622622	71.1759034	70.582444	82.88359275	62.33913383	50.03435628	49.20298046	47.80094416	Sra1	steroid receptor RNA activator 1 [Source:MGI Symbol;Acc:MGI:1344414]
ENSMUSG00000006056	0.365492947	0.038262174	0.034017182	0	0.104729695	0.329005613	9.581465533	10.76343799	9.370573677	27.99665199	32.41496375	28.97511831	Calcoco2	calcium binding and coiled-coil domain 2 [Source:MGI Symbol;Acc:MGI:1343177]
ENSMUSG00000006057	61.57302365	50.57172994	77.4962691	50.5457883	46.03922893	33.3518072	100.028476	110.8335995	73.62374029	94.41534136	93.8681579	97.79244912	Atp5g1	ATP synthase, H+ transporting, mitochondrial F0 complex, subunit C1 (subunit 9) [Source:MGI Symbol;Acc:MGI:107653]
ENSMUSG00000006058	124.2044786	80.76352767	149.379822	101.0912449	83.14745776	68.86875702	80.89119051	93.32619519	64.70496177	76.63683172	83.94087117	79.87751621	Snf8	SNF8, ESCRT-II complex subunit, homolog (S. cerevisiae) [Source:MGI Symbol;Acc:MGI:1343161]
ENSMUSG00000006095	102.4647259	74.35640671	122.5006802	92.54569405	78.92399027	62.5299408	109.8634932	116.8755409	95.62968606	78.92279486	80.22794287	79.7279408	Tbcb	tubulin folding cofactor B [Source:MGI Symbol;Acc:MGI:1913661]
ENSMUSG00000006127	15.86631087	17.01645446	14.57094242	14.38630384	16.29688966	17.67668697	13.19255308	14.82638181	15.27466263	16.92453777	17.03124954	19.34977332	Inpp5k	inositol polyphosphate 5-phosphatase K [Source:MGI Symbol;Acc:MGI:1194899]
ENSMUSG00000006134	19.10359961	22.81182576	15.7580248	22.21336327	26.34502565	29.96218483	31.01283578	29.27654331	38.00257041	50.70379067	52.00415535	50.27178996	Crkl	v-crk avian sarcoma virus CT10 oncogene homolog-like [Source:MGI Symbol;Acc:MGI:104686]
ENSMUSG00000006143	0.590780153	0.850392145	0.554433355	0.64319072	0.724160706	0.487485433	1.274634746	1.609442762	0.824810901	1.497243543	1.147451388	1.352540098	Upk3bl	uroplakin 3B-like [Source:MGI Symbol;Acc:MGI:1916915]
ENSMUSG00000006154	0.241733078	0.357901798	0.106064808	0.708969914	0.435393114	0.341944036	0.369966492	0.273681205	0.064284352	0.278002967	0.488673261	0.244834059	Eps8l1	EPS8-like 1 [Source:MGI Symbol;Acc:MGI:1914675]
ENSMUSG00000006169	20.50650646	26.51545003	17.25688016	22.38339032	26.34987711	32.18023591	21.90648218	19.24096484	24.77631999	42.24662327	43.52470507	42.9269	Clint1	clathrin interactor 1 [Source:MGI Symbol;Acc:MGI:2144243]
ENSMUSG00000006179	0.437948986	0.220640337	0.308253659	0	0.115033889	0.125091682	3.958782671	3.850426446	4.636733828	1.983158573	2.734112632	2.328727004	Prss16	protease, serine 16 (thymus) [Source:MGI Symbol;Acc:MGI:1859181]
ENSMUSG00000006191	8.274709198	7.757210192	6.061360536	9.962238813	8.914316294	10.74121478	12.92287344	13.46200932	14.08011882	11.69650549	10.84595525	10.49377876	Cdkal1	CDK5 regulatory subunit associated protein 1-like 1 [Source:MGI Symbol;Acc:MGI:1921765]
ENSMUSG00000006200	0	0.098117853	0	0	0.089521422	0.097348576	1.217104287	0.759668573	0.713747255	1.981558665	2.375970948	3.246960201	Rhox6	reproductive homeobox 6 [Source:MGI Symbol;Acc:MGI:1202888]
ENSMUSG00000006204	0.020558284	0.01183696	0.03157113	0.038369305	0.010799885	0.046976618	0.009176984	0	0	0	0.008556349	0.009109628	Cdcp3	CUB domain containing protein 3 [Source:MGI Symbol;Acc:MGI:1918645]
ENSMUSG00000006205	74.04984444	62.84922195	73.973173	41.53354827	37.88323894	31.62076132	21.50225874	23.85931246	21.10609949	20.6927937	21.71874364	19.28488961	Htra1	HtrA serine peptidase 1 [Source:MGI Symbol;Acc:MGI:1929076]
ENSMUSG00000006215	23.09153452	21.09850343	24.80361301	24.32759097	21.60984132	21.12530714	24.98655318	25.40050514	23.79459853	23.36366287	25.04030239	22.41007067	Zbtb17	zinc finger and BTB domain containing 17 [Source:MGI Symbol;Acc:MGI:107410]
ENSMUSG00000006216	0.361583931	0.312286863	0.370186976	0.337424183	0.221609404	0.022950995	0.995342858	0.895501002	0.701140608	1.644093802	1.254092279	2.136297181	Clcnkb	chloride channel, voltage-sensitive Kb [Source:MGI Symbol;Acc:MGI:1930643]
ENSMUSG00000006218	0.480429722	0.138309883	0.430377881	0.140102854	0.15774012	0.434547378	13.13557467	12.58250773	12.12933131	4.404763772	4.374007822	4.869728931	Fam131c	family with sequence similarity 131, member C [Source:MGI Symbol;Acc:MGI:2685539]
ENSMUSG00000006219	113.3627505	121.1923398	94.54321967	122.4662502	130.5228137	131.9753116	174.3416041	152.6013234	190.0945388	151.7444151	151.0869123	151.1205955	Fblim1	filamin binding LIM protein 1 [Source:MGI Symbol;Acc:MGI:1921452]
ENSMUSG00000006221	0.57495632	0.518936783	0.699999642	0.058004302	0.163265866	0.082852345	0	0.04618185	0.057853596	0.111196725	0.142284446	0.192799085	Hspb7	heat shock protein family, member 7 (cardiovascular) [Source:MGI Symbol;Acc:MGI:1352494]
ENSMUSG00000006235	0.746775409	0.409500129	0.291254547	0.265477539	0.186811233	0.270859675	8.095692387	6.129667355	6.288709743	4.612194317	3.877694783	3.844805266	Epor	erythropoietin receptor [Source:MGI Symbol;Acc:MGI:95408]
ENSMUSG00000006241	3.302338352	2.673264141	3.113114946	3.35629374	3.675752328	4.831428574	1.86819959	2.396894172	2.647626278	1.930247554	1.388040007	2.057322365	Ccdc159	coiled-coil domain containing 159 [Source:MGI Symbol;Acc:MGI:1914369]
ENSMUSG00000006262	14.32149032	16.30236986	10.33659601	17.04282577	22.753092	28.6224125	5.356034914	4.475487516	6.477662977	14.42173958	14.02986217	13.98881902	Mob1b	MOB kinase activator 1B [Source:MGI Symbol;Acc:MGI:1915723]
ENSMUSG00000006269	0.718471967	0.643500203	0.776431126	1.303684345	0.670995449	0.608052331	0.178176503	0.197708013	0	0.107109368	0.066450604	0.106121256	Atp6v1b1	ATPase, H+ transporting, lysosomal V1 subunit B1 [Source:MGI Symbol;Acc:MGI:103285]
ENSMUSG00000006270	0	0	0	0	0	0	0	0.263529198	0	0.237947242	0.719659013	0.235752116	Vax1	ventral anterior homeobox 1 [Source:MGI Symbol;Acc:MGI:1277163]
ENSMUSG00000006273	114.5346084	124.1007334	108.2811035	115.6481465	119.6122377	117.9114193	209.2289953	195.5908172	230.6953415	181.128483	181.7364666	180.7674337	Atp6v1b2	ATPase, H+ transporting, lysosomal V1 subunit B2 [Source:MGI Symbol;Acc:MGI:109618]
ENSMUSG00000006276	26.14862951	26.82734752	25.40212605	28.77610466	27.24859287	29.1897864	48.7107988	44.07378453	52.80714552	43.49427186	46.33641347	44.49891985	Eps15l1	epidermal growth factor receptor pathway substrate 15-like 1 [Source:MGI Symbol;Acc:MGI:104582]
ENSMUSG00000006281	8.502060708	9.635855848	7.839807239	9.5816729	10.68706901	12.60089464	23.01121722	20.79958849	25.25847469	15.63895511	17.95636398	16.6002297	Tep1	telomerase associated protein 1 [Source:MGI Symbol;Acc:MGI:109573]
ENSMUSG00000006288	26.50375164	25.60612552	21.81259816	26.85881403	25.85740505	23.85342591	56.17563671	50.25463476	56.11659635	50.025746	48.15647016	50.16140483	Ttc5	tetratricopeptide repeat domain 5 [Source:MGI Symbol;Acc:MGI:2683584]
ENSMUSG00000006289	19.19464888	15.8598517	19.29512307	17.28980616	17.27298956	14.00183901	58.16588753	59.28554559	55.91768326	29.0215865	26.59716764	27.57183742	Osgep	O-sialoglycoprotein endopeptidase [Source:MGI Symbol;Acc:MGI:1913496]
ENSMUSG00000006299	139.7802253	111.3427884	162.0037462	129.7131914	116.0346838	93.97594178	115.4394202	118.5183896	95.27635105	90.86506843	88.34829285	89.55706094	Aamp	angio-associated migratory protein [Source:MGI Symbol;Acc:MGI:107809]
ENSMUSG00000006301	45.4091294	46.21534261	46.93417311	49.35500378	50.14320768	45.24652643	9.69346785	9.290044564	10.19569554	21.94173784	22.36044189	21.08357609	Tmbim1	transmembrane BAX inhibitor motif containing 1 [Source:MGI Symbol;Acc:MGI:1916910]
ENSMUSG00000006304	516.0722036	477.0416213	506.7783483	541.7663961	490.2492034	432.5759949	211.0237642	220.1890253	201.7516344	221.9690952	203.5180919	205.3100168	Arpc2	actin related protein 2/3 complex, subunit 2 [Source:MGI Symbol;Acc:MGI:1923959]
ENSMUSG00000006307	19.21446777	21.82804618	19.77774919	22.11403522	21.52193656	27.46464903	23.73600739	21.64022259	28.49627975	36.85387927	39.03079565	35.434551	Kmt2b	lysine (K)-specific methyltransferase 2B [Source:MGI Symbol;Acc:MGI:109565]
ENSMUSG00000006310	5.951603924	4.260332184	4.775757965	3.602555021	2.619547418	3.614331248	24.30547309	23.9023445	23.05633396	22.2654684	24.00065394	26.79994797	Zbtb32	zinc finger and BTB domain containing 32 [Source:MGI Symbol;Acc:MGI:1891838]
ENSMUSG00000006311	0.16423087	0	0.04203459	0	0	0.03127292	0.916384878	1.993002421	1.146445177	1.983158573	1.401232724	2.219567926	Etv2	ets variant 2 [Source:MGI Symbol;Acc:MGI:99253]
ENSMUSG00000006313	0.575328127	0.871736728	0.682019356	1.328088289	0.922618742	0.853369995	1.757188962	1.439859739	1.409189005	2.898108657	2.41971854	3.38124278	Upk1a	uroplakin 1A [Source:MGI Symbol;Acc:MGI:98911]
ENSMUSG00000006315	97.53508434	66.61952302	105.1703185	79.93018041	68.29728523	47.09617979	65.03748082	69.93838818	51.96726364	46.2268336	49.64430194	50.65644577	Tmem147	transmembrane protein 147 [Source:MGI Symbol;Acc:MGI:1915011]
ENSMUSG00000006333	1341.788619	978.8305242	1617.966307	1208.714534	1020.153588	754.5336536	2206.988821	2459.1534	1796.167655	1187.015351	1170.534974	1174.063093	Rps9	ribosomal protein S9 [Source:MGI Symbol;Acc:MGI:1924096]
ENSMUSG00000006335	48.92363376	33.66287714	54.50532652	40.32361969	33.39541031	23.93244162	44.00345572	46.35861967	29.24493127	25.06066737	25.74970285	27.09159754	Tfpt	TCF3 (E2A) fusion partner [Source:MGI Symbol;Acc:MGI:1916964]
ENSMUSG00000006342	1.071482659	1.298807749	1.140277774	0.578883691	0.607320135	0.740959182	7.828977468	7.905055635	7.97833687	4.136321419	3.121654285	3.860769184	Susd2	sushi domain containing 2 [Source:MGI Symbol;Acc:MGI:1918983]
ENSMUSG00000006344	0.845718121	0.546327353	1.013666352	0.365731581	0.303411533	0.329939805	0.073662232	0.102171255	0.057597054	0.258308499	0.463593307	0.54841186	Ggt5	gamma-glutamyltransferase 5 [Source:MGI Symbol;Acc:MGI:1346063]
ENSMUSG00000006345	1.088076704	1.879464386	0.101269541	1.292295838	0.103927169	0.301370305	15.71918167	16.51139158	14.45452135	14.24497146	12.80350376	12.88629193	Ggt1	gamma-glutamyltransferase 1 [Source:MGI Symbol;Acc:MGI:95706]
ENSMUSG00000006356	43.77035831	28.25768132	38.96929825	18.15959674	16.18865511	14.90913671	175.0576109	187.8590078	155.3099722	89.68614975	89.68469616	94.77594614	Crip2	cysteine rich protein 2 [Source:MGI Symbol;Acc:MGI:1915587]
ENSMUSG00000006360	10.64821971	6.57687609	15.46040727	4.019864485	3.458004103	2.986159361	52.02641654	47.66039664	25.90355695	15.06615557	10.43483774	12.56798771	Crip1	cysteine-rich protein 1 (intestinal) [Source:MGI Symbol;Acc:MGI:88501]
ENSMUSG00000006362	0.362224754	0.197583359	0.136626385	0.088953207	0.100151348	0.101647383	0.306365695	0.453265549	0.248421853	0.204632519	0.396731009	0.422384855	Cbfa2t3	CBFA2/RUNX1 translocation partner 3 [Source:MGI Symbol;Acc:MGI:1338013]
ENSMUSG00000006369	65.51092368	61.4029052	62.26997375	40.02033035	36.1495222	32.6187671	14.35780192	14.47784595	15.10143279	30.28352067	32.01631645	30.81702293	Fbln1	fibulin 1 [Source:MGI Symbol;Acc:MGI:95487]
ENSMUSG00000006373	120.4195214	127.00899	108.4107611	121.9091589	126.7147534	121.6539172	66.38301923	60.82848487	64.09589571	59.794175	60.60130466	61.02907525	Pgrmc1	progesterone receptor membrane component 1 [Source:MGI Symbol;Acc:MGI:1858305]
ENSMUSG00000006378	0.733600448	0.552355256	1.01103525	0.552933137	0.859699169	0.236402772	2.191535937	2.934894432	1.050469548	1.03475844	0.962945505	0.900186602	Gcat	glycine C-acetyltransferase (2-amino-3-ketobutyrate-coenzyme A ligase) [Source:MGI Symbol;Acc:MGI:1349389]
ENSMUSG00000006386	1.05222503	1.168416526	0.564279187	1.122193267	0.881792527	1.259438348	0.480884636	0.210957616	0.408069957	5.333412516	5.286508258	4.78465267	Tek	TEK receptor tyrosine kinase [Source:MGI Symbol;Acc:MGI:98664]
ENSMUSG00000006389	0.023283814	0.026812508	0	0	0.024463376	0	0.041574517	0.023065935	0.043343238	0.020826822	0.019381426	0.061904066	Mpl	myeloproliferative leukemia virus oncogene [Source:MGI Symbol;Acc:MGI:97076]
ENSMUSG00000006390	133.889615	123.8737891	145.5435024	135.8980813	125.9688094	106.4360185	33.64096929	33.39714475	33.011388	26.9042095	28.44485839	28.61818964	Elovl1	elongation of very long chain fatty acids (FEN1/Elo2, SUR4/Elo3, yeast)-like 1 [Source:MGI Symbol;Acc:MGI:1858959]
ENSMUSG00000006392	62.53026349	52.47393024	64.06708102	64.83579383	61.41887348	52.7742025	57.15172053	65.60013532	59.71694197	45.55980875	47.14426149	45.52168578	Med8	mediator complex subunit 8 [Source:MGI Symbol;Acc:MGI:1915269]
ENSMUSG00000006395	32.1006179	23.22797399	48.01462191	30.46565461	31.23918718	24.58918585	4.430919676	8.750703126	4.449905725	5.987016991	6.253740163	5.770376053	Hyi	hydroxypyruvate isomerase (putative) [Source:MGI Symbol;Acc:MGI:1915430]
ENSMUSG00000006398	110.9550133	105.6895979	104.6989403	114.5348717	103.3646502	96.63563172	349.9481318	348.4559589	330.4249414	196.737032	202.6609226	203.2891065	Cdc20	cell division cycle 20 [Source:MGI Symbol;Acc:MGI:1859866]
ENSMUSG00000006403	7.464860894	10.24426454	6.598877393	7.66749828	9.422456397	11.66187334	1.038736183	0.785400879	1.437514412	2.3945612	2.442644056	2.171723215	Adamts4	a disintegrin-like and metallopeptidase (reprolysin type) with thrombospondin type 1 motif, 4 [Source:MGI Symbol;Acc:MGI:1339949
ENSMUSG00000006411	0.204836598	0.235879872	0.24903087	0.179203263	0.201762801	0.097512706	1.943030405	1.268248965	1.608638934	1.946728439	1.66243159	1.667818206	Nectin4	nectin cell adhesion molecule 4 [Source:MGI Symbol;Acc:MGI:1918990]
ENSMUSG00000006412	52.79441038	45.56056335	72.63707598	49.38371474	49.81246328	39.73571852	143.9346084	151.9398548	104.5317815	70.70437275	66.2838006	68.57265305	Pfdn2	prefoldin 2 [Source:MGI Symbol;Acc:MGI:1276111]
ENSMUSG00000006418	40.0633837	37.95619947	38.91635084	43.37090394	38.88712319	38.19696556	29.50371605	25.92389295	28.75323699	32.10414503	32.65993934	34.49683445	Rnf114	ring finger protein 114 [Source:MGI Symbol;Acc:MGI:1933159]
ENSMUSG00000006423	24.37623049	31.57076936	18.63127279	34.46199864	39.48469639	47.40205829	11.68304966	9.952548844	14.42136878	24.59345741	22.8209358	23.99924093	C330007P06	RIKEN cDNA C330007P06 gene [Source:MGI Symbol;Acc:MGI:1924894]
ENSMUSG00000006435	3.386067627	3.668051234	3.795481588	3.309695685	3.079509088	3.333469595	8.328196979	8.922922396	8.931645923	6.668055138	7.575575529	7.14028262	Neurl1a	neuralized E3 ubiquitin protein ligase 1A [Source:MGI Symbol;Acc:MGI:1334263]
ENSMUSG00000006442	98.60483456	71.42852252	103.8634518	62.9561022	55.56299811	48.7160472	287.4084182	322.0843274	262.9437209	191.4401444	198.7900019	201.3795545	Srm	spermidine synthase [Source:MGI Symbol;Acc:MGI:102690]
ENSMUSG00000006445	42.01092224	51.65871798	37.77182804	67.25707537	72.39440168	79.58772309	129.1050569	117.9987149	148.6689449	144.8499815	151.9071131	151.0629866	Epha2	Eph receptor A2 [Source:MGI Symbol;Acc:MGI:95278]

ENSMUSG00000006456	8.704655757	8.525555499	9.999935841	10.82502192	9.838777971	11.1247421	23.24851262	22.40217038	25.61917614	21.99780701	23.14063037	24.11727575	Rbm14	RNA binding motif protein 14 [Source:MGI Symbol;Acc:MGI:1929092]
ENSMUSG00000006457	1.956851126	1.920523861	2.253836057	1.743096712	1.051356241	1.270310877	11.53438254	11.74138337	11.79742448	6.305263742	7.163375096	8.572513457	Actn3	actinin alpha 3 [Source:MGI Symbol;Acc:MGI:99678]
ENSMUSG00000006462	0.726159126	0.393510382	0.641396098	0.637778602	0.523590715	0.73746969	0.025423464	0.014105178	0.013252532	0.101887403	0.023704087	0.138802764	A530013C2	RIKEN cDNA A530013C23 gene [Source:MGI Symbol;Acc:MGI:3041178]
ENSMUSG00000006463	11.74731728	11.73950344	10.73658399	14.80554491	13.0281294	13.62727323	9.42289046	8.248725758	9.907556836	8.112264879	7.287010754	7.877875184	Zdhhc24	zinc finger, DHHC domain containing 24 [Source:MGI Symbol;Acc:MGI:1917855]
ENSMUSG00000006464	3.628108999	4.203429346	3.125558112	3.385691704	4.090829835	5.628032336	2.370068118	2.849028381	3.726939036	3.166105203	3.719798893	3.097685831	Bbs1	Bardet-Biedl syndrome 1 (human) [Source:MGI Symbol;Acc:MGI:1277215]
ENSMUSG00000006469	0.052925569	0.426625596	0	0.246946358	0.111213575	0.201562243	2.409787894	2.883667434	3.300483582	1.704264194	2.86358783	2.392101032	Slc34a3	solute carrier family 34 (sodium phosphate), member 3 [Source:MGI Symbol;Acc:MGI:2159410]
ENSMUSG00000006471	2.416625502	3.469550328	3.887908296	3.39737668	3.858040297	4.972280301	5.968172077	5.56530032	5.491787371	7.523541202	8.41213268	8.066041109	Ndor1	NADPH dependent diflavin oxidoreductase 1 [Source:MGI Symbol;Acc:MGI:1926047]
ENSMUSG00000006476	31.2453794	33.80616455	28.63782912	34.39712788	32.88118181	35.59308502	5.417191241	5.562062046	5.061800309	7.769659683	8.990895015	9.103701061	Nsmf	NMDA receptor synaptonuclear signaling and neuronal migration factor [Source:MGI Symbol;Acc:MGI:1861755]
ENSMUSG00000006488	0.73763122	0.802230253	0.922999528	1.109000816	0.99027745	1.279747472	0	0	0	0	0	0	Prl7a1	prolactin family 7, subfamily a, member 1 [Source:MGI Symbol;Acc:MGI:1206572]
ENSMUSG00000006490	14.79960728	14.78069415	13.65526227	9.931805027	7.870662642	7.236726425	0.244680599	0.135751107	0.127545069	0.490292565	0.532310251	0.485769486	Prl8a9	prolactin family8, subfamily a, member 9 [Source:MGI Symbol;Acc:MGI:1914560]
ENSMUSG00000006494	39.34511533	42.82906992	30.75576779	45.37633154	50.28451641	64.19991591	193.6544256	183.2745592	220.570869	147.1913333	143.4267765	140.1527325	Pdk1	pyruvate dehydrogenase kinase, isoenzyme 1 [Source:MGI Symbol;Acc:MGI:1926119]
ENSMUSG00000006498	114.5224833	146.442966	86.75216731	150.2596761	169.6266066	188.1173449	257.2586467	207.3389276	292.9766019	312.3639635	296.9254712	301.1709303	Ptbp1	polypyrimidine tract binding protein 1 [Source:MGI Symbol;Acc:MGI:97791]
ENSMUSG00000006517	12.10550772	10.51719524	12.39353	11.40850902	10.47724815	8.234586801	51.66209344	53.09827045	44.57838704	20.81995284	20.15322799	20.88281076	Mvd	mevalonate (diphospho) decarboxylase [Source:MGI Symbol;Acc:MGI:2179327]
ENSMUSG00000006519	267.864845	196.68565	327.3184637	199.2499612	161.9088611	115.8047154	44.50092834	48.5736404	38.59387712	57.50818231	64.91808681	59.19468344	Cyba	cytochrome b-245, alpha polypeptide [Source:MGI Symbol;Acc:MGI:1316658]
ENSMUSG00000006522	0.053988065	0	0.082908877	0.125951938	0.170169329	0.020560866	0	0	0.025124938	0.048291049	0.112349045	0.119613879	Itih3	inter-alpha trypsin inhibitor, heavy chain 3 [Source:MGI Symbol;Acc:MGI:96620]
ENSMUSG00000006526	21.08251996	18.0079622	22.46812092	18.73381324	17.43821421	15.36396397	21.92687323	19.60218749	22.43556147	14.18090623	13.97536947	14.22013001	Stimate	STIM activating enhancer [Source:MGI Symbol;Acc:MGI:1921500]
ENSMUSG00000006527	7.060885129	6.708426413	6.177166543	7.620545204	6.735479283	8.805108597	5.885866127	6.337841363	6.632497744	7.87438901	8.810799831	8.838905378	Sfmbt1	Scm-like with four mbt domains 1 [Source:MGI Symbol;Acc:MGI:1859609]
ENSMUSG00000006529	0	0	0.060929807	0.111074627	0	0.030220397	0	0	0	0	0	0	Itih1	inter-alpha trypsin inhibitor, heavy chain 1 [Source:MGI Symbol;Acc:MGI:96618]
ENSMUSG00000006538	0.200963358	0.077139879	0.29718695	0.0208373	0.211144196	0.170077948	0.159480453	0.132721892	0.145482128	0.239675971	0.223042297	0.178098673	Ihh	Indian hedgehog [Source:MGI Symbol;Acc:MGI:96533]
ENSMUSG00000006542	0.661976736	0.482790152	0.768090516	0.63833734	1.321473921	0.722709173	0.137899476	0.174875273	0.164304211	0.078949675	0.348984941	0.312885372	Prkag3	protein kinase, AMP-activated, gamma 3 non-catalytic subunit [Source:MGI Symbol;Acc:MGI:1891343]
ENSMUSG00000006546	0	0	0.053591545	0	0.054997954	0	0.046733399	0	0	0	0.043572841	0.139171185	Cryba2	crystallin, beta A2 [Source:MGI Symbol;Acc:MGI:104336]
ENSMUSG00000006567	0.212083542	0.366337651	0.267236399	0.441497955	0.334241595	0.490833674	1.660395588	1.446450567	2.057501434	2.072148332	2.077718274	2.197611948	Atp7b	ATPase, Cu++ transporting, beta polypeptide [Source:MGI Symbol;Acc:MGI:103297]
ENSMUSG00000006570	0	0	0	0	0	0	0	0	0	0	0	0	Defb2	defensin beta 2 [Source:MGI Symbol;Acc:MGI:1338754]
ENSMUSG00000006574	0	0.165995027	0	0.168146892	0	0.027448929	0.225212712	0	0.268336024	0.741392987	0.539952897	0.638742122	Slc4a1	solute carrier family 4 (anion exchanger), member 1 [Source:MGI Symbol;Acc:MGI:109393]
ENSMUSG00000006575	8.680001077	7.584109534	11.16866312	7.879410878	5.677653796	5.736739876	16.82534425	19.27196882	19.83596123	12.8998129	13.88817621	12.87060362	Rundc3a	RUN domain containing 3A [Source:MGI Symbol;Acc:MGI:1858752]
ENSMUSG00000006576	26.44459148	22.98055412	24.38606707	27.54576381	27.40305805	28.02698987	6.040430923	6.385419646	7.409887658	6.605573594	7.301952293	7.906524881	Slc4a3	solute carrier family 4 (anion exchanger), member 3 [Source:MGI Symbol;Acc:MGI:109350]
ENSMUSG00000006585	35.44263152	35.70319889	37.86495536	48.52490518	47.68865769	44.54581591	242.0107705	252.7927925	264.406432	117.1250476	119.7651337	121.5399164	Cdt1	chromatin licensing and DNA replication factor 1 [Source:MGI Symbol;Acc:MGI:1914427]
ENSMUSG00000006586	1.756264809	2.171137239	1.454305979	1.482256235	1.614582798	2.13444252	0.080703475	0.140721586	0.240391066	0.993386887	1.139432413	1.201667165	Runx1t1	RUNX1 translocation partner 1 [Source:MGI Symbol;Acc:MGI:104793]
ENSMUSG00000006587	0.264302751	0.182614922	0.16235473	0.246642984	0.444307797	0.362366322	2.689983639	2.382648735	2.902825479	3.782601118	2.486060767	2.248623372	Snai3	snail family zinc finger 3 [Source:MGI Symbol;Acc:MGI:1353563]
ENSMUSG00000006589	305.8934316	215.3902429	360.7612308	286.9148507	224.0551656	189.8906893	560.9150738	635.9647308	462.6283806	235.1848006	240.1901377	243.4728187	Aprt	adenine phosphoribosyl transferase [Source:MGI Symbol;Acc:MGI:88061]
ENSMUSG00000006599	46.98036564	52.8273577	44.61811083	49.69540898	48.11187123	52.03428325	64.96230621	64.93802874	70.70977363	72.6253341	70.09249813	71.61246841	Gtf2h1	general transcription factor II H, polypeptide 1 [Source:MGI Symbol;Acc:MGI:1277216]
ENSMUSG00000006611	6.240416214	7.213074518	7.298172901	3.118928479	2.357413667	2.385507206	1.043314885	1.296603732	1.044192978	1.484326786	1.264582784	1.512059773	Hfe	homeostatic iron regulator [Source:MGI Symbol;Acc:MGI:109191]
ENSMUSG00000006638	0.096983819	0.223363707	0.165485538	0	0.033965679	0.147741641	0.028861648	0.096076291	0.03008952	0.23133263	0.026909749	0.315147973	Abhd1	abhydrolase domain containing 1 [Source:MGI Symbol;Acc:MGI:1931013]
ENSMUSG00000006641	5.188550581	4.714555891	3.963250018	5.068832812	5.770821607	5.542097696	8.05210083	7.288357379	9.564258041	10.24291145	12.29887057	11.69313281	Slc5a6	solute carrier family 5 (sodium-dependent vitamin transporter), member 6 [Source:MGI Symbol;Acc:MGI:2660847]
ENSMUSG00000006642	0	0.012038269	0	0	0	0	0.074664439	0.062136804	0.019460229	0.074806543	0.156633566	0.046322771	Tcf23	transcription factor 23 [Source:MGI Symbol;Acc:MGI:1934960]
ENSMUSG00000006649	0.524737656	0.52091585	0.537222563	0.641645146	0.58934309	0.895841607	23.97293669	20.86479677	26.50856371	17.91681193	19.64054063	18.55330981	Nphs1	nephrosis 1, nephrin [Source:MGI Symbol;Acc:MGI:1859637]
ENSMUSG00000006651	62.39130735	58.37083091	55.43241483	25.79556758	21.98768211	22.24660795	117.9219611	121.1607429	113.7456584	53.30000191	53.15549929	56.30381154	Aplp1	amyloid beta (A4) precursor-like protein 1 [Source:MGI Symbol;Acc:MGI:88046]
ENSMUSG00000006673	76.88386946	79.31552501	67.48223822	83.80751528	99.19334319	103.0520854	76.68899439	66.51505905	80.86114403	103.9598037	108.6051631	105.2077063	Qrich1	glutamine-rich 1 [Source:MGI Symbol;Acc:MGI:1916482]
ENSMUSG00000006675	1.045696824	0.713584383	0.991274757	0.68669314	0.610373335	0.855487487	8.782513892	7.941989626	8.291003466	3.325692864	3.933086236	3.706889025	P4htm	prolyl 4-hydroxylase, transmembrane (endoplasmic reticulum) [Source:MGI Symbol;Acc:MGI:1921693]
ENSMUSG00000006676	105.1896428	117.513014	93.39077042	111.1037394	114.9552914	121.4772886	58.56925193	57.03933202	69.23846335	87.14391642	91.97440443	89.9581826	Usp19	ubiquitin specific peptidase 19 [Source:MGI Symbol;Acc:MGI:1918722]
ENSMUSG00000006678	6.424916506	8.074735879	5.032089399	11.42769545	13.11305608	15.83119359	26.98774543	23.4483708	31.66459232	34.45160655	33.72232136	33.18231706	Pola1	polymerase (DNA directed), alpha 1 [Source:MGI Symbol;Acc:MGI:99660]
ENSMUSG00000006699	246.6272888	242.7313167	229.1559404	219.8730372	219.8375256	219.040319	235.1559458	228.7123662	237.50183	213.7619055	212.3227602	215.285769	Cdc42	cell division cycle 42 [Source:MGI Symbol;Acc:MGI:106211]
ENSMUSG00000006705	17.98249389	16.81023239	17.53653188	18.18167054	19.48101269	21.43089174	15.55512559	15.94824808	18.35357833	21.61328344	23.78808898	22.27462422	Pknox1	Pbx/knotted 1 homeobox [Source:MGI Symbol;Acc:MGI:1201409]
ENSMUSG00000006711	0.080457157	0.222361327	0.115320027	0.135146335	0.321225897	0.625084163	0.114928509	0	0.104840711	0.345441733	0.187522908	0.256691206	D130043K2	RIKEN cDNA D130043K22 gene [Source:MGI Symbol;Acc:MGI:3036268]
ENSMUSG00000006715	50.87165523	40.58042609	48.73628693	58.8578827	53.9066277	44.88796244	210.2119166	220.251532	171.2988392	97.48660849	97.43096887	101.2694102	Gmnn	geminin [Source:MGI Symbol;Acc:MGI:1927344]
ENSMUSG00000006717	183.4892987	137.1439467	219.0460165	175.0137793	140.509532	115.3494841	98.1124204	99.4603113	71.53607065	49.72468299	44.95635405	49.96725442	Acot13	acyl-CoA thioesterase 13 [Source:MGI Symbol;Acc:MGI:1914084]
ENSMUSG00000006720	2.570424479	2.141771195	2.096707392	2.65219669	2.942163506	2.865140221	6.194138685	7.142261999	5.776881011	5.476917993	6.210113913	5.222670773	Zfp184	zinc finger protein 184 (Kruppel-like) [Source:MGI Symbol;Acc:MGI:1922244]
ENSMUSG00000006724	0.242141181	0.641327439	0.718916857	0.813463817	0.636020236	0.608634035	0.799859089	0.935513821	0.608512713	0.931335146	0.846544046	0.922743332	Cyp27b1	cytochrome P450, family 27, subfamily b, polypeptide 1 [Source:MGI Symbol;Acc:MGI:1098274]
ENSMUSG00000006728	532.1416901	430.3834908	562.7438895	521.0596695	454.8639375	375.1754851	494.8105295	531.6909092	471.8558933	303.3841748	292.8772278	283.9533164	Cdk4	cyclin-dependent kinase 4 [Source:MGI Symbol;Acc:MGI:88357]
ENSMUSG00000006731	14.04087498	15.26289789	12.17843371	13.99254158	12.77354147	12.219898	7.938360426	8.784939249	8.58671559	6.929966244	7.520551693	7.732212253	B4galnt1	beta-1,4-N-acetyl-galactosaminyl transferase 1 [Source:MGI Symbol;Acc:MGI:1342057]
ENSMUSG00000006732	19.20772608	14.92896681	17.79189772	19.59430342	20.0972284	15.62975476	37.56276858	35.53467156	33.27558893	20.66682852	21.88073447	21.63918991	Mettl1	methyltransferase like 1 [Source:MGI Symbol;Acc:MGI:1339986]
ENSMUSG00000006736	134.1695211	158.5013324	123.524033	149.7944584	165.3946978	150.5054809	72.7889526	64.12303314	71.21131553	82.07808391	78.96227591	82.63960901	Tspan31	tetraspanin 31 [Source:MGI Symbol;Acc:MGI:1914375]
ENSMUSG00000006740	79.06208815	102.6056124	67.61455633	105.2638865	118.7471829	152.1891061	66.073549	66.34330655	84.76043504	113.7664811	111.4364018	122.4359571	Kif5b	kinesin family member 5B [Source:MGI Symbol;Acc:MGI:1098268]
ENSMUSG00000006763	9.180126536	8.577661002	9.192437736	10.157352	9.052944598	8.597302357	17.30226116	17.29763813	16.83913171	11.60881581	12.54596106	11.43035602	Saal1	serum amyloid A-like 1 [Source:MGI Symbol;Acc:MGI:1926185]
ENSMUSG00000006764	0.117886268	0.237566171	0.331900515	0.440038113	0.154823008	0.089791837	0.052623088	0.029195786	0.082292777	0.421785933	0.539706343	0.182828995	Tph2	tryptophan hydroxylase 2 [Source:MGI Symbol;Acc:MGI:2651811]
ENSMUSG00000006777	0.219953931	0.063322065	0.365929235	0.102628697	0.115548418	0.062825599	0	0	0	0.221336548	0.526382196	0.341125026	Krt23	keratin 23 [Source:MGI Symbol;Acc:MGI:2148866]
ENSMUSG00000006782	12.64559448	10.04754067	12.45699742	9.569912217	8.263075965	6.882544334	26.52759089	27.06741329	27.47816785	25.27376458	28.71319518	27.54043116	Cnp	2',3'-cyclic nucleotide 3' phosphodiesterase [Source:MGI Symbol;Acc:MGI:88437]
ENSMUSG00000006784	0.393426982	0.453051394	0.423987009	0.541047825	0.609159246	0.583560521	1.737726796	1.435904398	1.406923995	1.092776716	1.275480647	1.027643245	Ttc25	tetratricopeptide repeat domain 25 [Source:MGI Symbol;Acc:MGI:1921657]
ENSMUSG00000006800	37.27086636	42.61258344	34.92714123	15.64073565	17.35138314	20.10139932	66.78696772	60.71138594	75.13983667	75.60136252	78.40484599	72.83384796	Sulf2	sulfatase 2 [Source:MGI Symbol;Acc:MGI:1919293]
ENSMUSG00000006818	124.767726	120.8162353	125.8817184	72.3874116	67.4214021	60.74723318	98.67344869	100.4436065	97.58227519	90.20478393	87.31137984	89.90041198	Sod2	superoxide dismutase 2, mitochondrial [Source:MGI Symbol;Acc:MGI:98352]
ENSMUSG00000006850	14.47393375	14.08583122	14.09349869	15.34200524	14.62865146	13.2713813	31.56759946	32.10575732	30.71411163	14.42419215	13.8814947	12.96658185	Tmco6	transmembrane and coiled-coil domains 6 [Source:MGI Symbol;Acc:MGI:1919233]
ENSMUSG00000006906	11.30092591	10.88054135	9.882179349	12.06824512	11.30207959	11.45537451	3.413642246	2.845090151	3.764493706	3.268133005	3.861772119	3.328345946	Stambp	STAM binding protein [Source:MGI Symbol;Acc:MGI:1917777]
ENSMUSG00000006920	17.91057813	19.57715976	14.2673398	18.09923041	19.37139963	20.29910126	18.36302707	17.62437665	21.90779964	36.08916157	35.3185884	34.05860435	Ezh1	enhancer of zeste 1 polycomb repressive complex 2 subunit [Source:MGI Symbol;Acc:MGI:1097695]
ENSMUSG00000006930	17.52484769	18.90100728	17.88346316	21.08722379	19.58029152	20.07680072	115.2954428	105.1249383	120.4392034	92.21818698	93.3610741	90.78661745	Hap1	huntingtin-associated protein 1 [Source:MGI Symbol;Acc:MGI:1261831]
ENSMUSG00000006931	152.8133917	134.7077668	163.3545259	120.1376564	107.7149965	92.44697622	17.50902111	19.08568904	14.39065171	35.92003935	37.25646051	36.87611281	P3h4	prolyl 3-hydroxylase family member 4 (non-enzymatic) [Source:MGI Symbol;Acc:MGI:1913430]
ENSMUSG00000006932	178.8858442	288.4982591	122.6775408	248.6104258	318.5990123	408.0091692	267.6466678	224.1122215	324.2419468	422.831586	430.2265217	416.3019128	Ctnnb1	catenin (cadherin associated protein), beta 1 [Source:MGI Symbol;Acc:MGI:88276]
ENSMUSG00000006941	95.61886184	71.37252853	104.3218616	90.08999846	80.43086357	66.51327347	30.13534737	35.49300839	29.96984699	24.67969999	28.57678418	25.31984033	Eif1b	eukaryotic translation initiation factor 1B [Source:MGI Symbol;Acc:MGI:1916219]

ENSMUSG00000006948	0	0	0	0	0	0	0	0	0	0	0	0.061061497	Klk4	kallikrein related-peptidase 4 (prostase, enamel matrix, prostate) [Source:MGI Symbol;Acc:MGI:1861379]
ENSMUSG00000006958	3.986766499	2.524117075	2.992105587	2.756938678	2.686799282	2.770487533	2.765188831	2.800812437	3.163720349	1.363914644	1.335365236	1.252797533	Chrd	chordin [Source:MGI Symbol;Acc:MGI:1313268]
ENSMUSG00000006998	360.0000433	366.340093	351.0819281	344.958041	340.6294004	331.8298589	448.7613932	453.5922095	486.3160834	486.9027912	500.7224022	484.0301455	Psmd2	proteasome (prosome, macropain) 26S subunit, non-ATPase, 2 [Source:MGI Symbol;Acc:MGI:1096584]
ENSMUSG00000007021	3.010409722	2.26765274	2.108761056	3.126099274	3.281824267	2.499859544	7.840590941	7.130467948	7.584268568	5.324742323	3.674008795	3.590631215	Syngr3	synaptogyrin 3 [Source:MGI Symbol;Acc:MGI:1341881]
ENSMUSG00000007029	133.8595176	119.3043813	132.9501989	148.5505106	137.2209259	136.3380369	243.2756248	241.8433362	249.2684582	147.8791964	161.4855956	149.7293448	Vars	valyl-tRNA synthetase [Source:MGI Symbol;Acc:MGI:90675]
ENSMUSG00000007030	0.10388336	0.071776218	0.09571952	0.08724802	0.043658444	0.094951291	0.02782341	0.072037888	0.125697482	0.176550304	0.302653463	0.174921581	Vwa7	von Willebrand factor A domain containing 7 [Source:MGI Symbol;Acc:MGI:1306798]
ENSMUSG00000007033	0.034812692	0.360797444	0.071281958	0.113703214	0.420627557	0.411001068	0.077699948	0.120704261	0	0.077847828	0.1593793	0.138833391	Hspa1l	heat shock protein 1-like [Source:MGI Symbol;Acc:MGI:96231]
ENSMUSG00000007034	0.204816285	0.314475307	0.314534115	0.557465975	0.286923084	0.286008907	5.333280018	5.495200517	6.211510609	2.290041633	2.699407513	2.132780469	Slc44a4	solute carrier family 44, member 4 [Source:MGI Symbol;Acc:MGI:1917379]
ENSMUSG00000007035	2.423381667	2.170504439	3.721560561	2.764006609	2.34811666	2.91233475	2.283736631	2.800822283	2.83201134	1.830466486	2.017220772	2.099934676	Msh5	mutS homolog 5 [Source:MGI Symbol;Acc:MGI:1329021]
ENSMUSG00000007036	80.61659214	65.28938266	81.01563255	68.18119277	70.19807844	54.4521346	27.73737112	29.263513	25.65321827	28.15211751	31.55028843	30.56210856	Abhd16a	abhydrolase domain containing 16A [Source:MGI Symbol;Acc:MGI:99476]
ENSMUSG00000007038	30.61338744	24.52473732	29.7049577	22.32386015	22.18940396	19.18332584	18.11489449	19.00565319	20.41037034	10.13398748	8.987078389	8.658618267	Neu1	neuraminidase 1 [Source:MGI Symbol;Acc:MGI:97305]
ENSMUSG00000007039	222.431418	179.6237632	231.7196505	207.3938101	166.0602819	139.5492726	127.9655467	143.6234847	118.7905981	91.57191562	102.048737	99.85403239	Ddah2	dimethylarginine dimethylaminohydrolase 2 [Source:MGI Symbol;Acc:MGI:1859016]
ENSMUSG00000007041	457.5921351	367.1812158	470.386495	433.900915	392.3142639	313.9212037	108.0937452	119.610712	97.88636775	187.1081655	189.1006984	183.731268	Clic1	chloride intracellular channel 1 [Source:MGI Symbol;Acc:MGI:2148924]
ENSMUSG00000007050	66.3151346	55.32715243	72.24792364	62.0877604	54.26144167	47.14640485	152.9290959	158.9960327	125.5787083	78.68325698	76.94499865	69.25786111	Lsm2	LSM2 homolog, U6 small nuclear RNA and mRNA degradation associated [Source:MGI Symbol;Acc:MGI:90676]
ENSMUSG00000007080	11.11556421	13.75882286	11.58522094	20.15586616	24.6369947	29.28642331	34.7261472	30.3008534	41.34059675	46.94125315	48.23920993	45.68843901	Pole	polymerase (DNA directed), epsilon [Source:MGI Symbol;Acc:MGI:1196391]
ENSMUSG00000007097	0.055041238	0	0.014087704	0	0	0	0.479110748	0.831524372	0.653184115	0.566180509	0.618519756	0.634125694	Atp1a2	ATPase, Na+/K+ transporting, alpha 2 polypeptide [Source:MGI Symbol;Acc:MGI:88106]
ENSMUSG00000007107	0.06808305	0	0.069702928	0	0	0.017285876	0.263392987	0.314747821	0.232352672	0.223295164	0.113344543	0.120673749	Atp1a4	ATPase, Na+/K+ transporting, alpha 4 polypeptide [Source:MGI Symbol;Acc:MGI:1351335]
ENSMUSG00000007122	0.465172841	0.044639203	0.396867119	0	0.366553933	0.029526145	2.145694552	2.150493217	1.443212667	0.554781497	1.032558572	0.7557873	Casq1	calsequestrin 1 [Source:MGI Symbol;Acc:MGI:1309468]
ENSMUSG00000007207	27.84012058	26.21724735	28.14983673	32.23383264	29.6124356	29.60157177	11.21132204	11.50959934	10.58067104	6.442672682	8.054886844	8.825519426	Stx1a	syntaxin 1A (brain) [Source:MGI Symbol;Acc:MGI:109355]
ENSMUSG00000007209	0	0	0	0	0	0	0.165225179	0.366673959	0.043063604	0.496618921	0.616204309	0.287021863	Ceacam9	carcinoembryonic antigen-related cell adhesion molecule 9 [Source:MGI Symbol;Acc:MGI:1347247]
ENSMUSG00000007216	3.094118412	2.181111071	3.15293323	3.049289647	2.263454111	2.367746787	2.788174892	2.621145877	2.987537513	2.172707779	2.190413756	2.075783118	Zfp775	zinc finger protein 775 [Source:MGI Symbol;Acc:MGI:2683557]
ENSMUSG00000007279	0.130636775	0.206848073	0.016718122	0.152385115	0.188725428	0.236321211	0.160365615	0.323535846	0.501564326	0.423586727	0.70682259	0.607811005	Scube2	signal peptide, CUB domain, EGF-like 2 [Source:MGI Symbol;Acc:MGI:1928765]
ENSMUSG00000007338	63.10215119	58.90463822	67.89172259	66.15847446	59.69880019	55.04308625	103.1002395	105.41826	83.7031663	67.17438697	64.63550306	65.7593779	Mrpl49	mitochondrial ribosomal protein L49 [Source:MGI Symbol;Acc:MGI:108180]
ENSMUSG00000007379	3.621209372	4.45859066	3.86130673	4.875946493	6.174345168	6.967103979	15.06837753	13.86519274	19.17321057	17.86541013	19.37907823	16.92328094	Dennd2c	DENN/MADD domain containing 2C [Source:MGI Symbol;Acc:MGI:3036254]
ENSMUSG00000007411	53.40753087	56.89725314	45.51570964	55.15545126	57.17970742	60.40392441	74.64725615	69.01816361	80.79246263	54.53542852	52.97517644	51.35182206	Mark3	MAP/microtubule affinity regulating kinase 3 [Source:MGI Symbol;Acc:MGI:1341865]
ENSMUSG00000007415	88.87026288	89.48240124	80.97973866	105.1507111	103.5155369	97.21681276	65.61986773	66.38836431	61.05221653	49.29532376	48.43782541	50.67765929	Gatad1	GATA zinc finger domain containing 1 [Source:MGI Symbol;Acc:MGI:1914460]
ENSMUSG00000007440	0	0	0	0.007492718	0	0	0	0.007954067	0	0	0.026733999	0.056925404	Pcdha11	protocadherin alpha 11 [Source:MGI Symbol;Acc:MGI:1298372]
ENSMUSG00000007457	0	0	0	0	0	0	0	0	0	0	0.111718264	0.237884621	2310003L06	RIKEN cDNA 2310003L06 gene [Source:MGI Symbol;Acc:MGI:1921498]
ENSMUSG00000007458	76.9433587	81.75577531	71.81323118	80.72693975	79.3889819	79.70073752	85.33923689	84.78992865	88.32655607	77.70917379	80.3170353	77.8612092	M6pr	mannose-6-phosphate receptor, cation dependent [Source:MGI Symbol;Acc:MGI:96904]
ENSMUSG00000007476	5.429206562	3.545916372	5.745043929	3.705311514	3.192677849	2.754313881	1.817651993	2.167415985	1.79127561	1.458701174	1.399623665	1.723521116	Lrrc8a	leucine rich repeat containing 8A VRAC subunit A [Source:MGI Symbol;Acc:MGI:2652847]
ENSMUSG00000007480	0.050961494	0.019561593	0	0.015852143	0.035695474	0.077632895	0.227486305	0.336564135	0.31621912	0.82050935	1.498850691	1.339845447	Mc5r	melanocortin 5 receptor [Source:MGI Symbol;Acc:MGI:99420]
ENSMUSG00000007564	485.3798275	445.1769727	481.5783581	459.7046138	436.7619873	369.2357146	413.3034896	415.881779	425.8209309	315.5278389	313.1198446	317.8500468	Ppp2r1a	protein phosphatase 2, regulatory subunit A, alpha [Source:MGI Symbol;Acc:MGI:1926334]
ENSMUSG00000007570	43.45627727	35.71506097	42.6288552	45.94196594	43.33556282	38.6662845	46.43518814	45.3451437	44.36264754	22.35299455	23.2349739	25.36928125	Fance	Fanconi anemia, complementation group E [Source:MGI Symbol;Acc:MGI:1920025]
ENSMUSG00000007589	19.33750865	18.71222884	19.92716494	22.81662329	26.32719716	24.2142176	48.04116456	47.61557928	45.82094101	26.94157646	26.29380035	27.18651793	Tinf2	Terf1 (TRF1)-interacting nuclear factor 2 [Source:MGI Symbol;Acc:MGI:107246]
ENSMUSG00000007591	1.078090336	1.131934403	1.623148497	1.568264057	1.499170468	1.400805598	0.452938589	0.282706433	0.059026016	0.170175238	0.290335763	0.168605327	Tssk4	testis-specific serine kinase 4 [Source:MGI Symbol;Acc:MGI:1918349]
ENSMUSG00000007594	13.83379358	14.20626335	13.51916655	11.54028277	11.5458962	11.74573461	2.218812031	2.19506801	2.285337804	5.142442271	4.212283894	5.22768422	Hapln4	hyaluronan and proteoglycan link protein 4 [Source:MGI Symbol;Acc:MGI:2679531]
ENSMUSG00000007603	21.76044565	16.35657426	20.30048678	22.25054075	19.67914914	18.06033179	36.35208462	33.62038692	35.71284374	28.15448948	28.62827723	29.20389708	Dus3l	dihydrouridine synthase 3-like (S. cerevisiae) [Source:MGI Symbol;Acc:MGI:2147092]
ENSMUSG00000007610	22.56088281	21.32942294	21.38752983	25.71006468	22.28203602	21.21050869	36.33837547	38.66698519	34.28286091	24.85168934	23.37337132	25.91538437	Gtpbp3	GTP binding protein 3 [Source:MGI Symbol;Acc:MGI:1917609]
ENSMUSG00000007613	11.52119526	14.23609387	9.00030012	13.69961035	17.37255011	21.25210835	9.963948582	8.606815218	11.49055864	21.7934654	20.48107844	19.49251832	Tgfbr1	transforming growth factor, beta receptor I [Source:MGI Symbol;Acc:MGI:98728]
ENSMUSG00000007617	6.777380867	8.598565211	6.414201265	10.6266988	12.46126724	14.3911565	12.59388073	10.61742727	15.40351982	16.28337039	17.10485399	17.61726229	Homer1	homer scaffolding protein 1 [Source:MGI Symbol;Acc:MGI:1347345]
ENSMUSG00000007646	4.415236624	4.968818031	3.53404246	4.775716813	5.229320406	5.793541894	11.66421175	12.26686009	15.13050967	11.68641143	11.27630175	10.88495227	Rad51c	RAD51 paralog C [Source:MGI Symbol;Acc:MGI:2150020]
ENSMUSG00000007653	0	0	0.023645867	0	0.048532816	0.052776201	0.020619889	0	0.021497129	0.619774016	1.115071808	0.798273367	Gabrb2	gamma-aminobutyric acid (GABA) A receptor, subunit beta 2 [Source:MGI Symbol;Acc:MGI:95620]
ENSMUSG00000007655	360.8345731	423.1796469	308.9848641	361.8153922	399.7392819	421.8459348	15.57358949	15.06881319	16.84229183	110.3876484	106.4051609	107.6790779	Cav1	caveolin 1, caveolae protein [Source:MGI Symbol;Acc:MGI:102709]
ENSMUSG00000007656	52.97226642	52.41976196	59.55112115	57.04731912	60.09398415	58.05398168	77.46214781	76.67454312	61.56336036	67.35394115	62.37377827	66.54656904	Arpp19	cAMP-regulated phosphoprotein 19 [Source:MGI Symbol;Acc:MGI:1891691]
ENSMUSG00000007659	85.32668903	84.66811326	87.05777347	97.69861817	91.67498093	78.98694722	34.71158912	34.0601648	34.44825185	43.26956708	44.61912186	46.98657299	Bcl2l1	BCL2-like 1 [Source:MGI Symbol;Acc:MGI:88139]
ENSMUSG00000007670	84.78999866	84.28468003	85.38553365	97.56431109	94.17973001	94.51760312	122.4228758	125.9224504	130.2164372	100.8838031	101.6907212	102.84656	Khsrp	KH-type splicing regulatory protein [Source:MGI Symbol;Acc:MGI:1336214]
ENSMUSG00000007682	0.162790496	0.187461624	0.159088092	0.048336087	0.124390902	0.084541756	0.006606166	0.007330326	0.027548857	0.086043605	0.067753333	0.144268943	Dio2	deiodinase, iodothyronine, type II [Source:MGI Symbol;Acc:MGI:1338833]
ENSMUSG00000007721	103.0368274	71.54683422	131.0208742	82.46768094	61.52791309	49.27125748	273.4259064	324.1380325	223.7723892	118.2685155	108.4143092	113.4154665	Ccdc124	coiled-coil domain containing 124 [Source:MGI Symbol;Acc:MGI:1916403]
ENSMUSG00000007739	267.4307595	257.1389967	258.6167146	283.6508266	276.6945447	273.9364588	676.109016	668.1696304	662.7679922	729.2196827	716.6910819	723.2453088	Cct4	chaperonin containing Tcp1, subunit 4 (delta) [Source:MGI Symbol;Acc:MGI:104689]
ENSMUSG00000007777	26.88649943	25.32659884	23.39468856	23.67784215	21.09368195	18.596279	37.1088391	36.27944365	33.05346151	34.24661955	32.83563496	35.49534153	0610009B22	RIKEN cDNA 0610009B22 gene [Source:MGI Symbol;Acc:MGI:1913300]
ENSMUSG00000007783	53.13676751	47.21623155	60.71751945	46.37832916	42.86418332	37.16123081	15.56919482	18.24566708	18.41413369	21.95239563	21.49012529	19.99455479	Cpt1c	carnitine palmitoyltransferase 1c [Source:MGI Symbol;Acc:MGI:2446526]
ENSMUSG00000007805	24.47287113	18.66785072	33.12491465	9.85604308	8.398846896	6.684063368	1.196103396	1.282978291	1.205423346	8.049118032	7.713546094	7.678031665	Twist2	twist basic helix-loop-helix transcription factor 2 [Source:MGI Symbol;Acc:MGI:104685]
ENSMUSG00000007812	24.39393903	31.23878289	21.70566741	35.73309012	40.69187141	48.76367377	14.7921407	15.60742726	18.6067348	30.08855567	31.1964638	29.27467901	Zfp655	zinc finger protein 655 [Source:MGI Symbol;Acc:MGI:1919861]
ENSMUSG00000007815	183.8872183	208.6352026	151.8869929	204.3424002	222.9000799	220.6285863	153.0546222	142.5258393	163.2187733	178.5030927	168.2148696	165.9257498	Rhoa	ras homolog family member A [Source:MGI Symbol;Acc:MGI:1096342]
ENSMUSG00000007817	30.54718604	39.67278064	26.09714934	36.6891391	44.70394886	56.04125953	11.27159534	11.34998399	14.52135468	23.3017513	25.28093331	21.15816058	Zmiz1	zinc finger, MIZ-type containing 1 [Source:MGI Symbol;Acc:MGI:3040693]
ENSMUSG00000007827	3.18491237	4.908643604	2.618432815	4.112916982	4.39407954	8.172064211	6.605918831	5.115101724	8.878185739	13.03555048	13.10830695	12.38784829	Ankrd26	ankyrin repeat domain 26 [Source:MGI Symbol;Acc:MGI:1917887]
ENSMUSG00000007833	27.65164063	24.42906352	31.04146933	27.48489994	23.72560583	22.76397092	36.89612728	39.02412879	35.54750271	29.20908456	28.90338556	26.54523133	Aldh16a1	aldehyde dehydrogenase 16 family, member A1 [Source:MGI Symbol;Acc:MGI:1916998]
ENSMUSG00000007836	158.7780122	144.2634702	162.6873616	165.2769926	149.4170372	134.2657881	239.856152	243.5785464	192.7683208	131.7760133	135.9650322	139.3032885	Hnrnpa0	heterogeneous nuclear ribonucleoprotein A0 [Source:MGI Symbol;Acc:MGI:1924384]
ENSMUSG00000007837	32.85899015	23.0899638	31.87280979	29.8129501	21.36937233	16.69757796	11.09191439	15.06398408	10.71549205	10.25487621	9.583110496	13.05106103	Prrg2	proline-rich Gla (G-carboxyglutamic acid) polypeptide 2 [Source:MGI Symbol;Acc:MGI:1929596]
ENSMUSG00000007850	204.8303336	242.0221705	159.9329198	265.3509074	311.0087236	358.5038377	301.4278243	280.4124061	333.3906187	507.0968009	515.3338175	515.9093696	Hnrnph1	heterogeneous nuclear ribonucleoprotein H1 [Source:MGI Symbol;Acc:MGI:1891925]
ENSMUSG00000007867	59.81359453	47.01834842	72.55664738	55.8792207	52.90632921	40.4855549	16.88826402	21.31292385	14.5069682	15.66924947	11.36603634	14.521199	Ift43	intraflagellar transport 43 [Source:MGI Symbol;Acc:MGI:1923661]
ENSMUSG00000007872	118.2157015	89.63014475	135.6740114	52.1364529	41.3620609	33.28879583	32.12970125	38.21820114	32.44819999	97.52971583	103.1844803	99.27532386	Id3	inhibitor of DNA binding 3 [Source:MGI Symbol;Acc:MGI:96398]
ENSMUSG00000007877	0.181554801	0.104534843	0.325280328	0.550627861	0.667633393	0.622291531	0.040521998	0.089927949	0.253475643	0	0.151126057	0.080449166	Tcap	titin-cap [Source:MGI Symbol;Acc:MGI:1330233]
ENSMUSG00000007880	12.67561832	14.18076779	11.08238523	12.88180185	15.56585195	19.85283206	36.29502473	32.84140007	46.92288336	58.32953863	62.66074439	59.57566268	Arid1a	AT rich interactive domain 1A (SWI-like) [Source:MGI Symbol;Acc:MGI:1935147]
ENSMUSG00000007888	120.0083582	98.40603103	130.4110903	97.71615585	89.94242357	78.49721726	10.11859793	8.992166007	8.215210569	14.71334845	16.78133327	16.08870804	Crlf1	cytokine receptor-like factor 1 [Source:MGI Symbol;Acc:MGI:1340030]
ENSMUSG00000007891	651.3517311	527.7167546	896.7637125	424.8430027	386.0387107	298.4406133	74.17270143	76.35179313	58.119163	150.1204842	139.3084291	140.0096307	Ctsd	cathepsin D [Source:MGI Symbol;Acc:MGI:88562]
ENSMUSG00000007892	2505.309991	1782.988181	3613.079046	2320.935393	1883.528935	1264.788207	4083.599067	4612.048383	2684.416776	1824.437588	1834.053712	1786.362285	Rplp1	ribosomal protein, large, P1 [Source:MGI Symbol;Acc:MGI:1927099]

ENSMUSG00000007907	0	0	0	0	0	0	0	0	0	0.076720199	0.237985884	0.101349913	Cabs1	calcium binding protein, spermatid specific 1 [Source:MGI Symbol;Acc:MGI:1918227]
ENSMUSG00000007908	0.615395993	0.850392145	0.604836387	0.298624263	0.439669	0.618731511	0.725223218	0.682793899	1.420507663	2.950450511	3.114510911	3.075937964	Hmgcll1	3-hydroxymethyl-3-methylglutaryl-Coenzyme A lyase-like 1 [Source:MGI Symbol;Acc:MGI:2446108]
ENSMUSG00000007944	0.188721438	0.1931755	0.23614754	0.019567972	0.176250754	0.03194349	0.262089648	0.540093587	0.390342607	0.375126378	0.279273883	0.334499164	Ttc9b	tetratricopeptide repeat domain 9B [Source:MGI Symbol;Acc:MGI:1920282]
ENSMUSG00000007946	0.4643362	0.222794585	0.554614661	0.649966368	0.609824438	0.324203442	5.181859812	7.129863015	5.402313605	2.457415119	3.317570301	2.606205716	Phox2a	paired-like homeobox 2a [Source:MGI Symbol;Acc:MGI:106633]
ENSMUSG00000007950	70.55142951	65.56163006	75.23860167	61.55677529	56.95595465	51.49943276	21.92696834	23.78621707	19.8133226	18.59218809	21.10033586	18.92882665	Abhd8	abhydrolase domain containing 8 [Source:MGI Symbol;Acc:MGI:1918946]
ENSMUSG00000007987	39.57012339	37.23159866	39.89404714	41.46690935	32.57533967	26.83181503	7.575265616	9.122728153	7.897542964	11.18668929	12.58611699	14.32656793	Ift22	intraflagellar transport 22 [Source:MGI Symbol;Acc:MGI:1914536]
ENSMUSG00000007989	1.131296109	1.250033373	1.031010111	1.305903382	1.380983585	1.89770545	1.266872847	1.269706102	1.679873225	2.158367719	2.068451775	1.970393251	Fzd3	frizzled class receptor 3 [Source:MGI Symbol;Acc:MGI:108476]
ENSMUSG00000008028	0	0.184886252	0.123280551	0.112369807	0.084343878	0.061145561	0.680860287	0.715732517	0.485670681	0.68215612	0.968926669	0.747006528	1700008O03	RIKEN cDNA 1700008O03 gene [Source:MGI Symbol;Acc:MGI:1916599]
ENSMUSG00000008035	33.48087312	25.98268133	34.71580314	37.31676448	33.69256761	29.70858985	21.40529182	25.15137083	16.69710894	13.74843774	11.15490749	12.74891142	Mid1ip1	Mid1 interacting protein 1 (gastrulation specific G12-like (zebrafish)) [Source:MGI Symbol;Acc:MGI:1915291]
ENSMUSG00000008036	287.0164578	193.1484303	362.7295952	201.0231438	163.3402458	119.8756774	185.6514046	209.2603319	161.7909194	118.6508008	110.3593217	117.4954945	Ap2s1	adaptor-related protein complex 2, sigma 1 subunit [Source:MGI Symbol;Acc:MGI:2141861]
ENSMUSG00000008090	86.27051654	86.68091443	85.3352936	89.20780932	93.7072339	82.95161551	10.19947738	8.637992297	10.83215265	11.55594028	14.48672631	11.0708433	Fgfrl1	fibroblast growth factor receptor-like 1 [Source:MGI Symbol;Acc:MGI:2150920]
ENSMUSG00000008129	3.548470285	2.875506405	3.444525367	3.45854635	3.396837603	2.502597788	3.379193028	3.385070373	2.813471243	3.174022883	3.260057695	3.21462483	4930432K21	RIKEN cDNA 4930432K21 gene [Source:MGI Symbol;Acc:MGI:1921916]
ENSMUSG00000008136	107.4742212	92.26307463	96.44622496	123.9000912	117.2399672	95.60320718	3.082887297	3.613011437	3.141818018	22.28068026	20.15304806	22.28144476	Fhl2	four and a half LIM domains 2 [Source:MGI Symbol;Acc:MGI:1338762]
ENSMUSG00000008140	115.4111559	95.3198309	116.9997899	103.1059643	74.0573554	69.51980818	90.76323148	103.5663605	92.14698846	60.9610247	59.31031753	58.94442216	Emc10	ER membrane protein complex subunit 10 [Source:MGI Symbol;Acc:MGI:1916933]
ENSMUSG00000008153	2.379323444	2.310770216	2.318539484	2.247096694	2.258909977	2.947696726	16.78890358	19.82201312	18.49038108	23.74407519	25.74710769	23.9823198	Clstn3	calsyntenin 3 [Source:MGI Symbol;Acc:MGI:2178323]
ENSMUSG00000008167	32.9197859	24.27696515	33.32855714	30.3788677	23.47758109	19.75559204	35.7727196	35.51055937	32.77592849	30.36801674	29.9765658	29.88158064	Fbxw9	F-box and WD-40 domain protein 9 [Source:MGI Symbol;Acc:MGI:1915878]
ENSMUSG00000008193	0.203375702	0.10037039	0.237959488	0.2982365	0.122102143	0.287685529	0.674399869	0.431727019	0.351545557	0.649695584	0.532053518	0.592205812	Spib	Spi-B transcription factor (Spi-1/PU.1 related) [Source:MGI Symbol;Acc:MGI:892986]
ENSMUSG00000008200	39.03003722	44.35097532	32.32611166	43.11443573	54.80288951	62.81687289	72.79937273	69.76532627	89.46231329	87.86701196	87.80288	82.78132367	Fnbp4	formin binding protein 4 [Source:MGI Symbol;Acc:MGI:1860513]
ENSMUSG00000008206	12.56487827	9.222262575	11.43097783	11.44961836	12.09040679	12.06965198	33.44466334	32.58173758	36.35831898	18.62500433	16.29687251	16.27573561	Cers4	ceramide synthase 4 [Source:MGI Symbol;Acc:MGI:1914510]
ENSMUSG00000008226	13.66638044	15.84708164	13.56952143	15.09084281	15.69131757	15.16734338	20.46716249	20.32345134	21.92816476	19.0029016	18.07999978	19.0524019	Scrn3	secernin 3 [Source:MGI Symbol;Acc:MGI:1921866]
ENSMUSG00000008301	55.90539558	46.92630597	58.88749328	49.21753496	40.88693498	36.72618195	48.27278354	54.97480652	50.5915017	43.04426239	45.77185999	49.09611479	Phax	phosphorylated adaptor for RNA export [Source:MGI Symbol;Acc:MGI:1891839]
ENSMUSG00000008305	33.5594907	34.38209847	30.39290854	37.20804033	40.20708091	40.20730367	19.08557183	19.06501039	19.61169791	19.2134614	19.59844891	20.36678137	Tle1	transducin-like enhancer of split 1 [Source:MGI Symbol;Acc:MGI:104636]
ENSMUSG00000008307	0.837897243	1.759490246	0.555068445	1.149870499	1.501765443	2.890721179	1.452107886	1.415978904	1.192758235	3.879657549	5.251500643	3.756506075	1700109H08	RIKEN cDNA 1700109H08 gene [Source:MGI Symbol;Acc:MGI:1924286]
ENSMUSG00000008318	11.4060891	12.27414875	12.36201171	10.82749174	10.70288341	8.867559514	13.27313971	13.52026431	15.04588782	10.51910598	13.09574972	13.41971509	Relt	RELT tumor necrosis factor receptor [Source:MGI Symbol;Acc:MGI:2443373]
ENSMUSG00000008333	49.88487908	46.43399914	50.42673601	47.65847757	46.22073547	39.77484503	107.3035071	119.5800051	106.8667282	79.59483829	76.69315456	75.41184252	Snrpb2	U2 small nuclear ribonucleoprotein B [Source:MGI Symbol;Acc:MGI:104805]
ENSMUSG00000008348	56.48036742	44.78125776	71.70827082	39.9081409	47.34903811	36.51199448	14.23931454	14.19414684	15.00823601	14.81512351	13.25807967	12.87276772	Ubc	ubiquitin C [Source:MGI Symbol;Acc:MGI:98889]
ENSMUSG00000008373	26.9348249	23.58924694	28.69419031	26.27229507	23.11186978	21.13988343	52.53906555	52.59546735	44.76196672	41.23173741	38.89488822	37.49983889	Prpf31	pre-mRNA processing factor 31 [Source:MGI Symbol;Acc:MGI:1916238]
ENSMUSG00000008384	44.91098417	29.66777248	47.35616896	43.01441313	32.66301001	25.64567115	26.50624931	35.37945359	28.28471326	20.44673213	18.66954634	20.73476692	Sertad1	SERTA domain containing 1 [Source:MGI Symbol;Acc:MGI:1913438]
ENSMUSG00000008393	77.20436864	75.88461508	70.50209671	80.37720318	83.94700681	78.28839709	38.6433539	39.74733524	37.82108466	51.49684514	49.06335462	50.08779713	Carhsp1	calcium regulated heat stable protein 1 [Source:MGI Symbol;Acc:MGI:1196368]
ENSMUSG00000008398	63.4950632	68.34079774	66.97762192	65.2765589	73.22724659	66.72487527	10.07138083	9.791574459	8.37230172	25.82271821	24.34772352	25.77206643	Elk3	ELK3, member of ETS oncogene family [Source:MGI Symbol;Acc:MGI:101762]
ENSMUSG00000008429	16.32731812	17.63950987	13.27584143	17.20352391	18.14775725	21.36928424	8.292042432	6.87227889	9.667446612	10.25050648	8.932949941	9.295461624	Herpud2	HERPUD family member 2 [Source:MGI Symbol;Acc:MGI:1915393]
ENSMUSG00000008435	16.25875452	19.73400622	13.6488419	18.42540541	18.98357957	20.26841103	18.31654452	17.84644335	18.87288402	18.06578013	16.85630318	18.96033109	Rdh13	retinol dehydrogenase 13 (all-trans and 9-cis) [Source:MGI Symbol;Acc:MGI:1918732]
ENSMUSG00000008438	0.029562685	0	0.10593121	0.013793706	0.124241342	0.123845492	0.052785784	0.073215128	0.013757867	0.039664685	0.024607953	0.026199179	Adam21	a disintegrin and metallopeptidase domain 21 [Source:MGI Symbol;Acc:MGI:1861229]
ENSMUSG00000008450	118.7555197	105.8964377	101.1452387	114.5896182	116.7745552	102.0490715	429.7055066	452.8803963	417.7690373	303.7353592	282.849874	280.4902285	Nutf2	nuclear transport factor 2 [Source:MGI Symbol;Acc:MGI:1915301]
ENSMUSG00000008461	0.191237724	0	0.065262593	0.089229951	0.033487643	0.048554105	0	0.063149404	0	0.171057628	0.106124075	0.084739788	Fut1	fucosyltransferase 1 [Source:MGI Symbol;Acc:MGI:109375]
ENSMUSG00000008475	138.4190645	127.1523041	138.2267059	126.4588043	122.6422115	111.489796	59.92822051	61.17652358	59.69126608	89.26267781	88.34503529	84.53747629	Arpc5	actin related protein 2/3 complex, subunit 5 [Source:MGI Symbol;Acc:MGI:1915021]
ENSMUSG00000008482	0.542946219	0.625230481	0.880118563	0.633335613	0.665527294	0.344626926	0.161576673	0.044822132	0.126338017	0.121413143	0.301298637	0.240586149	Rnf151	ring finger protein 151 [Source:MGI Symbol;Acc:MGI:1914754]
ENSMUSG00000008489	2.623688282	2.672699013	1.673654909	2.37304794	2.989855951	4.627101413	9.91002333	8.737099177	11.75927245	18.10667461	17.60604386	17.29574054	Elavl2	ELAV like RNA binding protein 1 [Source:MGI Symbol;Acc:MGI:1100887]
ENSMUSG00000008496	1.788112452	1.703440091	1.764087088	0.758471436	0.49529325	0.445736586	0.565991428	0.789069338	0.453900471	2.311895222	2.679161823	2.391409965	Pou2f2	POU domain, class 2, transcription factor 2 [Source:MGI Symbol;Acc:MGI:101897]
ENSMUSG00000008540	181.6636412	166.5976061	178.4565646	130.4475868	117.3455712	99.36715185	5.732828988	6.035037111	5.210476346	26.36229329	28.96415696	27.67553924	Mgst1	microsomal glutathione S-transferase 1 [Source:MGI Symbol;Acc:MGI:1913850]
ENSMUSG00000008575	26.53160727	25.83350815	26.00104731	28.01428234	30.72399267	34.53477388	3.555367446	3.226865149	4.097825272	5.676857555	5.326612251	5.76416801	Nfib	nuclear factor I/B [Source:MGI Symbol;Acc:MGI:103188]
ENSMUSG00000008590	0.053540217	0.020551437	0.091356803	0	0.075003432	0.027187076	0	0	0	0	0	0	Htr3b	5-hydroxytryptamine (serotonin) receptor 3B [Source:MGI Symbol;Acc:MGI:1861899]
ENSMUSG00000008601	1.478077826	1.449922213	1.401152996	1.123888693	1.437923612	1.709586318	3.812161091	3.850426446	3.974343281	3.182847092	2.460701369	2.668333025	Rab25	RAB25, member RAS oncogene family [Source:MGI Symbol;Acc:MGI:1858203]
ENSMUSG00000008604	48.95890728	53.00204696	46.7507035	60.13798059	61.72008546	61.65832213	183.6030009	169.3284057	193.4073497	145.8181146	153.0033295	152.6484117	Ubqln4	ubiquilin 4 [Source:MGI Symbol;Acc:MGI:2150152]
ENSMUSG00000008658	0.062045404	0.035724236	0	0	0	0.094517724	0	0.061464813	0	0.249741671	0.49064213	0.27493082	Rbfox1	RNA binding protein, fox-1 homolog (C. elegans) 1 [Source:MGI Symbol;Acc:MGI:1926224]
ENSMUSG00000008668	1295.776194	1035.11529	1539.093486	1368.958031	1165.887073	911.8889271	2515.419709	2488.179384	1777.475578	1232.055146	1191.203731	1193.024062	Rps18	ribosomal protein S18 [Source:MGI Symbol;Acc:MGI:98146]
ENSMUSG00000008682	1235.355911	987.0803794	1374.973749	1106.84185	943.3425855	761.5610305	2033.562225	2166.212556	1767.161629	1221.564981	1237.455062	1167.731302	Rpl10	ribosomal protein L10 [Source:MGI Symbol;Acc:MGI:105943]
ENSMUSG00000008683	361.1848282	303.6905796	465.5801798	373.4489318	352.0901611	253.7872146	442.1838848	437.3732814	285.9423292	277.7962089	240.7433211	243.9685838	Rps15a	ribosomal protein S15A [Source:MGI Symbol;Acc:MGI:2389091]
ENSMUSG00000008690	101.6876121	85.1069816	100.1818822	109.4794618	101.5715336	90.77437793	169.4834926	184.8191313	166.1806466	82.70032527	81.99339554	86.06693293	Ncaph2	non-SMC condensin II complex, subunit H2 [Source:MGI Symbol;Acc:MGI:1289164]
ENSMUSG00000008730	16.94276164	19.22553769	13.60650006	21.5500593	22.52988483	28.06253081	13.10649183	12.34889016	14.94715047	21.71007961	22.517948	20.66402475	Hipk1	homeodomain interacting protein kinase 1 [Source:MGI Symbol;Acc:MGI:1314873]
ENSMUSG00000008734	39.00339405	58.38602662	33.83444162	75.14499326	85.54462427	104.6688909	10.69647742	9.490547054	12.44415649	11.51691335	11.69730561	10.57624873	Gprc5b	G protein-coupled receptor, family C, group 5, member B [Source:MGI Symbol;Acc:MGI:1927596]
ENSMUSG00000008763	34.86301572	36.49439022	31.69435823	33.12993221	38.89208465	52.83864383	17.5381347	16.01400109	21.96314855	43.50537453	42.0818735	42.62159644	Man1a2	mannosidase, alpha, class 1A, member 2 [Source:MGI Symbol;Acc:MGI:104676]
ENSMUSG00000008789	0	0	0	0	0	0	0	0	0	0.035277696	0	0	Ceacam5	carcinoembryonic antigen-related cell adhesion molecule 5 [Source:MGI Symbol;Acc:MGI:1920500]
ENSMUSG00000008813	0	0	0	0	0	0	0	0	0	0	0	0	Tppp2	tubulin polymerization-promoting protein family member 2 [Source:MGI Symbol;Acc:MGI:2684923]
ENSMUSG00000008822	25.84503327	20.10938134	28.17627691	29.00697668	24.07196172	17.87673853	26.50375484	27.12553945	20.02457576	12.43361251	13.14060691	12.31890914	Acyp1	acylphosphatase 1, erythrocyte (common) type [Source:MGI Symbol;Acc:MGI:1913454]
ENSMUSG00000008843	0.040326605	0.139314477	0.082572165	0	0	0	0	0	0	0	0	0	Cldn13	claudin 13 [Source:MGI Symbol;Acc:MGI:1913102]
ENSMUSG00000008845	0.022208252	0	0.034104968	0.020724376	0.011666663	0.008457812	0.019827021	0.044000875	0.010335265	0.019864757	0.018486129	0	Cd163	CD163 antigen [Source:MGI Symbol;Acc:MGI:2135946]
ENSMUSG00000008855	40.78691315	42.8437771	43.87763765	40.38084747	41.88041269	36.54136276	42.39555526	41.70864373	42.9065655	40.03533082	40.03539762	43.44473626	Hdac5	histone deacetylase 5 [Source:MGI Symbol;Acc:MGI:1333784]
ENSMUSG00000008859	75.90389758	72.81470038	77.28880456	82.17790625	80.80244235	79.31156368	42.33895207	52.86216238	44.40336035	46.00622504	42.40683823	44.78452247	Rala	v-ral simian leukemia viral oncogene A (ras related) [Source:MGI Symbol;Acc:MGI:1927243]
ENSMUSG00000008874	0	0	0	0	0	0	0	0	0	0	0	0	Clec3a	C-type lectin domain family 3, member a [Source:MGI Symbol;Acc:MGI:2685642]
ENSMUSG00000008892	101.7288405	91.53523267	89.87414087	96.99634978	92.47486222	86.13932473	153.1244478	157.0786461	153.4609695	97.59080493	99.52755757	97.73216333	Vdac3	voltage-dependent anion channel 3 [Source:MGI Symbol;Acc:MGI:106922]
ENSMUSG00000008932	0.032684001	0.056455962	0.100384923	0.122000692	0.120189225	0.112026656	0.043769298	0.01618908	0	0	0.040809201	0.057930733	Slc1a7	solute carrier family 1 (glutamate transporter), member 7 [Source:MGI Symbol;Acc:MGI:2444087]
ENSMUSG00000008958	63.52004761	51.73039219	63.13121771	57.19042882	57.94305591	42.38119394	88.86816593	92.19340475	76.64335864	49.80397057	45.96919406	46.22271986	Vps72	vacuolar protein sorting 72 [Source:MGI Symbol;Acc:MGI:1202305]
ENSMUSG00000008976	24.2020178	27.62003812	17.47282529	28.86101516	35.55881174	46.78330789	63.91715215	60.51198772	78.93160507	81.00800541	81.04445942	82.03381177	Gabpa	GA repeat binding protein, alpha [Source:MGI Symbol;Acc:MGI:95610]
ENSMUSG00000008999	0.832809442	0.916399644	0.833676993	0.967137531	0.933333024	0.76120306	3.27145854	2.713387294	3.06612859	2.747958029	3.373718594	2.739008638	Bmp7	bone morphogenetic protein 7 [Source:MGI Symbol;Acc:MGI:103302]
ENSMUSG00000009013	265.5066728	219.9071995	321.004023	247.8400731	228.8179046	166.9342188	280.9460317	302.5614708	238.239224	204.2225823	201.0144166	199.3676337	Dynll1	dynein light chain LC8-type 1 [Source:MGI Symbol;Acc:MGI:1861457]
ENSMUSG00000009030	31.96201836	39.7901699	22.56393065	45.44772369	53.25407441	59.03947288	27.65952868	24.72437938	32.11755071	41.03573192	41.47217161	44.87806309	Pdcl	phosducin-like [Source:MGI Symbol;Acc:MGI:1914716]

ENSMUSG00000009035	74.32894789	88.61455514	70.17289108	64.92238945	68.33941249	75.89154569	51.15857434	45.70876914	56.2315941	63.28774387	61.72148954	67.67279977	Tmem184b	transmembrane protein 184b [Source:MGI Symbol;Acc:MGI:2445179]
ENSMUSG00000009047	0	0	0	0	0	0	0	0	0	0	0	0	Gm5965	predicted gene 5965 [Source:MGI Symbol;Acc:MGI:3645197]
ENSMUSG00000009070	0.156183259	0.134889788	0.199874093	0.036436913	0.123071647	0.178442944	0	0	0	0	0	0	Rsph14	radial spoke head homolog 14 (Chlamydomonas) [Source:MGI Symbol;Acc:MGI:1918486]
ENSMUSG00000009073	55.08950336	68.11352148	53.73039607	67.11604043	67.6879367	75.88585219	120.8797991	105.2435703	129.5529371	147.4046701	149.8289956	150.4831569	Nf2	neurofibromin 2 [Source:MGI Symbol;Acc:MGI:97307]
ENSMUSG00000009075	0.049119278	0.09427229	0.067050609	0.030558202	0.068810226	0.074826531	0.877051463	0.600135603	0.731491352	1.025174142	1.158460584	0.928655213	Cabp7	calcium binding protein 7 [Source:MGI Symbol;Acc:MGI:2183437]
ENSMUSG00000009076	16.58929651	14.47681631	16.58593757	15.1784984	16.27227121	10.26656068	18.57103638	19.83642611	13.38992717	25.09832747	24.81289566	21.70818972	Zmat5	zinc finger, matrin type 5 [Source:MGI Symbol;Acc:MGI:1914428]
ENSMUSG00000009079	298.7567495	284.8772588	314.5215243	334.9746902	318.3929076	308.934502	440.378493	449.5630889	435.4689401	370.5937373	367.9910246	363.0785388	Ewsr1	Ewing sarcoma breakpoint region 1 [Source:MGI Symbol;Acc:MGI:99960]
ENSMUSG00000009090	61.72345109	64.18061896	57.80414659	58.88553127	58.87350019	61.48230662	64.05458777	58.1171284	70.86157386	83.61101605	90.26186383	85.54918396	Ap1b1	adaptor protein complex AP-1, beta 1 subunit [Source:MGI Symbol;Acc:MGI:1096368]
ENSMUSG00000009092	1.283599507	0.578399015	1.142730226	1.093674494	0.879539267	0.892677605	7.274408164	7.408383388	9.349996464	7.487931201	5.435245695	6.726426767	Derl3	Der1-like domain family, member 3 [Source:MGI Symbol;Acc:MGI:1917627]
ENSMUSG00000009093	0.31638594	0.468430295	0.138820116	0.126534069	0.047487729	0	0.363165637	0.13432515	0.126205309	0.525570969	0.489095989	0.040055572	Gstt4	glutathione S-transferase, theta 4 [Source:MGI Symbol;Acc:MGI:1923136]
ENSMUSG00000009097	0.430524058	0.550856205	0.685638135	0.267838651	0.07538908	0.109307462	0.277594832	0.04738838	0.089047585	0.363699515	0.219002574	0.169573784	Tbx1	T-box 1 [Source:MGI Symbol;Acc:MGI:98493]
ENSMUSG00000009108	2.885859395	2.83780195	2.25738735	3.752095894	3.100193647	3.136624432	1.389535038	1.284878604	1.237389007	1.421183385	1.646442656	1.379336417	Gnat2	guanine nucleotide binding protein, alpha transducing 2 [Source:MGI Symbol;Acc:MGI:95779]
ENSMUSG00000009112	16.37997477	20.97939461	12.8188996	19.47397308	21.45567996	24.82650986	26.18656911	24.29903019	31.33946307	25.48087073	25.53227613	26.52277089	Bcl2l13	BCL2-like 13 (apoptosis facilitator) [Source:MGI Symbol;Acc:MGI:2136959]
ENSMUSG00000009114	0	0	0	0	0	0.021117281	0.049503678	0.329581297	0.154829194	0.024798947	0.184623069	0.073710512	2610028H24	RIKEN cDNA 2610028H24 gene [Source:MGI Symbol;Acc:MGI:1924214]
ENSMUSG00000009115	0.10793099	0	0	0.033573142	0.075599195	0	0.032119443	0	0.033485913	0	0	0	Spatc1l	spermatogenesis and centriole associated 1 like [Source:MGI Symbol;Acc:MGI:1923823]
ENSMUSG00000009145	1.303893571	1.062600335	1.581362828	2.22762884	2.149766187	1.971025017	0.590997755	0.496804752	0.84019199	0.628008775	0.651215918	0.906656475	Dqx1	DEAQ RNA-dependent ATPase [Source:MGI Symbol;Acc:MGI:2136388]
ENSMUSG00000009185	469.0189378	344.325499	492.2472576	64.98419994	55.29393144	41.8173414	3.909143663	5.255241497	3.918703671	11.59911315	8.971741803	9.99962667	Ccl8	chemokine (C-C motif) ligand 8 [Source:MGI Symbol;Acc:MGI:101878]
ENSMUSG00000009207	10.25589405	12.9645686	8.552557535	13.45645225	17.17592023	19.38248532	13.67697313	12.2988557	17.99101669	21.42818677	18.67869313	21.54941926	Lnpk	lunapark, ER junction formation factor [Source:MGI Symbol;Acc:MGI:1918115]
ENSMUSG00000009210	0	0	0	0	0	0	0	0	0	0.127725552	0.079240875	0.210912083	Prr29	proline rich 29 [Source:MGI Symbol;Acc:MGI:1922823]
ENSMUSG00000009214	0.033200994	0.038232651	0	0	0.069765923	0	0	0	0	0	0	0	Mymk	myomaker, myoblast fusion factor [Source:MGI Symbol;Acc:MGI:1913389]
ENSMUSG00000009216	0.043624692	0.083726792	0.029775093	0.067849743	0.045834726	0.132912554	0.934731157	0.749085529	0.771477403	0.273148798	0.254192087	0.28351602	Fam163b	family with sequence similarity 163, member B [Source:MGI Symbol;Acc:MGI:1926106]
ENSMUSG00000009246	0.958951029	1.022482782	0.490883514	1.408603226	1.100821619	0.608679706	0.221959537	0.175921782	0.132229968	0.301804111	0.118256295	0.330495666	Trpm5	transient receptor potential cation channel, subfamily M, member 5 [Source:MGI Symbol;Acc:MGI:1861718]
ENSMUSG00000009248	2.240326226	1.678950529	2.075188646	1.161464235	0.747244931	1.110524666	4.317702606	3.804621117	4.468293765	4.834851974	4.528910701	4.443586414	Ascl2	achaete-scute family bHLH transcription factor 2 [Source:MGI Symbol;Acc:MGI:96920]
ENSMUSG00000009281	16.7244308	17.92607708	20.71845162	6.779158596	6.206707899	5.411665745	0.677070712	0.43495763	0.260059425	5.284062173	4.518641062	5.235315301	Rarres2	retinoic acid receptor responder (tazarotene induced) 2 [Source:MGI Symbol;Acc:MGI:1918910]
ENSMUSG00000009291	364.6127176	395.6348008	322.8054134	460.0940757	478.710362	476.0416006	104.9153441	99.6058541	114.506729	154.8881925	157.1173052	153.3128306	Pttg1ip	pituitary tumor-transforming 1 interacting protein [Source:MGI Symbol;Acc:MGI:2652132]
ENSMUSG00000009292	0.014873311	0.154146436	0.16749905	0.069397646	0.03125359	0.113287316	0.013278553	0.073670667	0.027686935	0.319291805	0.371415923	0.382251723	Trpm2	transient receptor potential cation channel, subfamily M, member 2 [Source:MGI Symbol;Acc:MGI:1351901]
ENSMUSG00000009293	120.3650233	118.0599792	141.9657183	115.5349402	112.1769073	86.96040142	52.96127881	49.84382458	46.67061124	48.80274136	45.60682144	47.1322702	Ube2g2	ubiquitin-conjugating enzyme E2G 2 [Source:MGI Symbol;Acc:MGI:1343188]
ENSMUSG00000009350	0	0.038144354	0	0	0.174012003	0	0	0	0	0.059257839	0.055145305	0.029355585	Mpo	myeloperoxidase [Source:MGI Symbol;Acc:MGI:97137]
ENSMUSG00000009356	0.722300755	0.356471335	0.176068146	0.096291314	0.180688722	0.366775611	0	0	0	0.03076576	0.114522384	0.121927753	Lpo	lactoperoxidase [Source:MGI Symbol;Acc:MGI:1923363]
ENSMUSG00000009376	16.75936146	20.78886186	13.27243217	20.3354085	23.43305302	30.28375808	0.74875335	0.661848312	1.270141575	6.039571862	6.602516193	5.505146795	Met	met proto-oncogene [Source:MGI Symbol;Acc:MGI:96969]
ENSMUSG00000009378	0.117031245	0.651376225	0.019969288	0.072807757	0.122960068	0	0.052241415	0.096613435	0.054463941	0.348938952	0.194833411	0.224717933	Slc16a12	solute carrier family 16 (monocarboxylic acid transporters), member 12 [Source:MGI Symbol;Acc:MGI:2147716]
ENSMUSG00000009394	0.205348877	0.127329886	0.226406572	0.221109459	0.099577794	0.252663155	0.028204739	0	0	0.028258419	0.026297265	0.097992046	Syn2	synapsin II [Source:MGI Symbol;Acc:MGI:103020]
ENSMUSG00000009406	8.065056573	10.41617363	7.511843838	10.49229642	10.86123216	12.79510436	5.87427696	5.105684899	6.815406179	9.180250177	9.964929802	9.450958083	Elk1	ELK1, member of ETS oncogene family [Source:MGI Symbol;Acc:MGI:101833]
ENSMUSG00000009418	14.47476069	17.0870021	15.29052199	16.87697332	21.08929437	28.29504804	11.97805005	10.74648272	14.43483592	17.83148132	19.72046101	17.86741695	Nav1	neuron navigator 1 [Source:MGI Symbol;Acc:MGI:2183683]
ENSMUSG00000009470	32.69555462	41.65201313	28.92781766	37.56507183	46.17973676	60.13084778	50.61508518	47.01424666	63.60580712	93.78885784	90.90805066	97.19163476	Tnpo1	transportin 1 [Source:MGI Symbol;Acc:MGI:2681523]
ENSMUSG00000009471	0	0.05325042	0	0.043152582	0	0	0.061926148	0.068714424	0.172161855	0.165450688	0.096230187	0	Myod1	myogenic differentiation 1 [Source:MGI Symbol;Acc:MGI:97275]
ENSMUSG00000009487	0.016335796	0	0.025086703	0.007622148	0	0	0.007292114	0.040457335	0.053216412	0.270321708	0.353545468	0.260589326	Otog	otogelin [Source:MGI Symbol;Acc:MGI:1202064]
ENSMUSG00000009535	28.58610495	31.32158276	27.03851549	34.63909342	34.71873333	36.17003963	57.04139214	54.2068911	60.63961763	63.48510864	63.7494214	59.3640658	Rnmt	RNA (guanine-7-) methyltransferase [Source:MGI Symbol;Acc:MGI:1915147]
ENSMUSG00000009545	0.250748764	0.404250127	0.205371796	0.233994626	0.052690348	0.190990796	0.716360915	0.794887603	0.933546656	0	0	0.044443945	Kcnq1	potassium voltage-gated channel, subfamily Q, member 1 [Source:MGI Symbol;Acc:MGI:108083]
ENSMUSG00000009549	245.7035345	202.9836769	245.920214	204.048877	190.7078987	163.6266112	184.2543075	188.8711409	149.9016533	140.684097	130.2818888	131.3970371	Srp14	signal recognition particle 14 [Source:MGI Symbol;Acc:MGI:107169]
ENSMUSG00000009551	0	0	0	0	0	0	0.041484724	0	0	0.083127357	0.077358262	0	6330409D2	RIKEN cDNA 6330409D20 gene [Source:MGI Symbol;Acc:MGI:1917980]
ENSMUSG00000009555	108.9964534	92.45595739	116.7315147	101.2070309	92.9480173	76.04193116	113.2147553	122.8686698	110.2615047	91.72762963	90.27660413	91.18215064	Cdk9	cyclin-dependent kinase 9 (CDC2-related kinase) [Source:MGI Symbol;Acc:MGI:1328368]
ENSMUSG00000009563	31.62914963	24.9134225	34.60074129	24.64644442	20.66424999	18.86340553	37.80556329	45.3297261	35.86491181	19.13627432	20.58133889	20.27589149	Tor2a	torsin family 2, member A [Source:MGI Symbol;Acc:MGI:1353596]
ENSMUSG00000009566	32.18319274	29.49617682	28.84158594	29.12772777	25.89287905	27.30049055	106.9790711	108.3552542	103.4055961	78.00122693	77.28518418	81.07149306	Fpgs	folylpolyglutamyl synthetase [Source:MGI Symbol;Acc:MGI:95576]
ENSMUSG00000009569	5.365984452	6.6812675	4.955735083	6.530547447	7.939938478	9.868820452	4.610980146	3.26698495	5.410641411	9.379504405	9.389251067	9.629821605	Mrtfb	myocardin related transcription factor B [Source:MGI Symbol;Acc:MGI:3050795]
ENSMUSG00000009575	34.59997363	40.02784719	31.15276372	43.58667482	48.80223099	59.77486521	73.98457025	60.77565817	81.3351656	78.47274814	82.45972676	79.35177221	Cbx5	chromobox 5 [Source:MGI Symbol;Acc:MGI:109372]
ENSMUSG00000009580	0	0	0	0	0	0	0	0	0	0	0	0	Odam	odontogenic, ameloblast asssociated [Source:MGI Symbol;Acc:MGI:1916842]
ENSMUSG00000009585	38.03462824	36.1758272	39.53407069	42.15844232	40.75459738	38.19212796	88.41637465	93.13258762	93.79932857	59.5498697	57.10882372	61.54784616	Apobec3	apolipoprotein B mRNA editing enzyme, catalytic polypeptide 3 [Source:MGI Symbol;Acc:MGI:1933111]
ENSMUSG00000009588	0	0	0.018416493	0.100719426	0.037799597	0.082209082	0	0	0	0	0	0	St6galnac1	ST6 (alpha-N-acetyl-neuraminyl-2,3-beta-galactosyl-1,3)-N-acetylgalactosaminide alpha-2,6-sialyltransferase 1 [Source:MGI Sym
ENSMUSG00000009596	28.07703569	27.13520311	28.1454729	41.34297237	40.35810645	36.68381173	3.137161822	2.218318735	2.917912561	1.602381306	2.294115323	1.434945172	Taf7l	TATA-box binding protein associated factor 7 like [Source:MGI Symbol;Acc:MGI:1921719]
ENSMUSG00000009614	22.82715546	23.62572511	26.45285651	24.79779469	24.60892187	24.80052009	0.468855013	0.277466869	0.032586779	1.096077155	0.699434509	1.085965553	Sardh	sarcosine dehydrogenase [Source:MGI Symbol;Acc:MGI:2183102]
ENSMUSG00000009621	15.06988514	17.63282814	16.08256753	15.1937635	15.18518015	17.9896314	19.57131582	17.83170383	21.30623064	17.04664153	17.47746151	17.67039362	Vav2	vav 2 oncogene [Source:MGI Symbol;Acc:MGI:102718]
ENSMUSG00000009628	3.179606389	3.623732986	2.393900849	4.190726102	5.051208754	6.408328057	1.638825669	1.818471918	2.827238223	6.069331416	5.584449351	5.451707806	Tex15	testis expressed gene 15 [Source:MGI Symbol;Acc:MGI:1934816]
ENSMUSG00000009630	59.78294327	58.52172199	53.16857699	61.5559102	61.08816332	54.28127448	71.15508368	68.71205402	66.60318391	67.70109265	62.68562754	64.14323178	Ppp2cb	protein phosphatase 2 (formerly 2A), catalytic subunit, beta isoform [Source:MGI Symbol;Acc:MGI:1321161]
ENSMUSG00000009633	0.93862373	1.592866174	1.314992543	1.383013014	0.622847096	0.714932712	1.984693953	2.055436973	1.149516404	1.944283031	2.220564037	2.626836144	G0s2	G0/G1 switch gene 2 [Source:MGI Symbol;Acc:MGI:1316737]
ENSMUSG00000009640	51.69581975	42.80687787	60.22183174	51.11749819	47.75811607	34.47580506	47.15019242	54.53208269	44.38751082	31.52774327	33.98915668	34.61662073	Dmap1	DNA methyltransferase 1-associated protein 1 [Source:MGI Symbol;Acc:MGI:1913483]
ENSMUSG00000009646	0	0.104534843	0.092937237	0.042355989	0	0	0.607829969	0.539567692	0.549197226	0.365392086	0.680067256	0.321796664	Pla2g12b	phospholipase A2, group XIIB [Source:MGI Symbol;Acc:MGI:1917086]
ENSMUSG00000009647	9.287087109	10.54925171	8.603753264	10.10313313	11.1098448	10.34159713	20.54800405	20.4004057	20.76448677	13.65702033	11.86893528	13.70995342	Mcu	mitochondrial calcium uniporter [Source:MGI Symbol;Acc:MGI:3026965]
ENSMUSG00000009654	1.507367408	0.852125428	1.038174049	0.971867616	1.123009182	1.064633791	0.09787224	0.271502215	0.153054082	0.907041246	0.958158452	0.801519653	Oit3	oncoprotein induced transcript 3 [Source:MGI Symbol;Acc:MGI:1201782]
ENSMUSG00000009670	3.368677665	3.928103048	3.564754457	5.151268538	6.275628402	6.296061859	3.336026801	3.084765294	3.662389514	4.064025396	3.970489157	3.687853282	Tex11	testis expressed gene 11 [Source:MGI Symbol;Acc:MGI:1933237]
ENSMUSG00000009681	36.14475171	42.61636945	37.3834043	43.31743383	44.06407724	51.1174553	19.07901062	17.46560522	20.0628271	27.88263848	24.69893419	26.49635928	Bcr	BCR activator of RhoGEF and GTPase [Source:MGI Symbol;Acc:MGI:88141]
ENSMUSG00000009687	111.2283888	90.21148624	125.494687	101.0662566	94.33857756	67.23861209	7.655949815	6.946064279	6.197524034	33.11860178	31.07208424	33.30482297	Fxyd5	FXYD domain-containing ion transport regulator 5 [Source:MGI Symbol;Acc:MGI:1201785]
ENSMUSG00000009731	8.551537826	8.2062795	8.91551006	8.937773139	8.400750776	8.53275264	3.957277117	3.572156167	3.515410783	3.824574333	3.571010109	3.751398706	Kcnd1	potassium voltage-gated channel, Shal-related family, member 1 [Source:MGI Symbol;Acc:MGI:96671]
ENSMUSG00000009733	14.79896166	16.39978079	13.59445177	15.93842406	18.37087141	20.34382671	12.46559752	10.31756384	13.53840576	11.58265655	12.19208129	13.22749167	Tfcp2	transcription factor CP2 [Source:MGI Symbol;Acc:MGI:98509]
ENSMUSG00000009734	0.053253079	0	0	0	0.055950889	0	0.095086272	0	0.074348672	0.28580173	0.088655632	0.094388378	Pou6f2	POU domain, class 6, transcription factor 2 [Source:MGI Symbol;Acc:MGI:2443631]
ENSMUSG00000009739	9.675940751	12.82822814	10.57805304	10.96094154	10.83797386	13.08653698	1.457270569	1.266939548	1.284329458	5.403668333	6.149240647	5.592428337	Pou6f1	POU domain, class 6, transcription factor 1 [Source:MGI Symbol;Acc:MGI:102935]
ENSMUSG00000009741	20.32196142	28.84121118	14.69444985	27.07135286	32.68148111	39.47227618	36.61140346	30.16760488	42.70684319	40.30843495	39.36948473	40.90571738	Ubp1	upstream binding protein 1 [Source:MGI Symbol;Acc:MGI:104889]

ENSMUSG00000009772	4.763046514	5.749863578	4.829257375	4.680993673	4.762587548	4.959918631	4.786449732	4.285379341	5.932414423	7.141884669	7.431522827	7.096390819	Nuak2	NUAK family, SNF1-like kinase, 2 [Source:MGI Symbol;Acc:MGI:1921387]
ENSMUSG00000009828	9.363369516	10.49664864	10.26361539	10.43589043	10.75891928	11.90751615	13.08556736	12.17647245	14.59692258	9.085586833	8.978317114	8.459317957	Cilk1	ciliogenesis associated kinase 1 [Source:MGI Symbol;Acc:MGI:1934157]
ENSMUSG00000009863	113.3158606	91.14343712	118.4336674	102.6961985	98.85867869	79.13238665	130.7646996	138.701384	117.8409589	83.86077839	90.94861051	88.84367846	Sdhb	succinate dehydrogenase complex, subunit B, iron sulfur (Ip) [Source:MGI Symbol;Acc:MGI:1914930]
ENSMUSG00000009876	2.947156701	2.172033562	4.103497279	2.530219151	1.9198053	2.020316341	12.84003342	12.84614588	10.42375177	6.010449543	4.121393891	5.746054131	Cox4i2	cytochrome c oxidase subunit 4I2 [Source:MGI Symbol;Acc:MGI:2135755]
ENSMUSG00000009894	154.881149	161.220812	155.771825	145.131849	143.7781965	132.2782301	72.48175824	76.92799131	79.50055452	134.4206587	138.2335381	141.1486621	Snap47	synaptosomal-associated protein, 47 [Source:MGI Symbol;Acc:MGI:1915076]
ENSMUSG00000009900	0	0	0	0	0	0	0.19478738	0.169824094	0.275600825	0.41819594	0.233503716	0.428149238	Wnt3a	wingless-type MMTV integration site family, member 3A [Source:MGI Symbol;Acc:MGI:98956]
ENSMUSG00000009905	14.69733793	16.70361289	13.89770612	15.42455946	17.75431383	18.06902751	9.719103141	9.394368082	9.720133926	16.3570554	16.50059969	15.66944281	Kdsr	3-ketodihydrosphingosine reductase [Source:MGI Symbol;Acc:MGI:1918000]
ENSMUSG00000009907	36.67894931	41.30801404	28.6384304	39.52226191	42.3197544	49.2275097	47.88220655	44.11732006	52.55943415	65.28511537	63.76929503	66.28782823	Vps4b	vacuolar protein sorting 4B [Source:MGI Symbol;Acc:MGI:1100499]
ENSMUSG00000009927	741.3259153	581.5479054	952.5127503	769.3769443	707.4620882	519.0672683	1329.399152	1396.846762	1003.785117	701.8935782	700.3342248	702.2133832	Rps25	ribosomal protein S25 [Source:MGI Symbol;Acc:MGI:1922867]
ENSMUSG00000009941	11.54517179	12.20365598	12.37422758	17.10808853	15.34035072	15.37801238	0.224446338	0.153261475	0.089998093	0.276767399	0.45072922	0.102830305	Nxf2	nuclear RNA export factor 2 [Source:MGI Symbol;Acc:MGI:1933192]
ENSMUSG00000009995	37.77216687	38.24134018	32.95575536	44.10798702	41.09847123	37.05698924	27.31965474	29.10344336	28.82325299	22.77933616	24.65801939	23.03862992	Taz	tafazzin [Source:MGI Symbol;Acc:MGI:109626]
ENSMUSG00000010021	1.198129806	1.319720755	1.759956727	0.972239173	1.094632406	0.773720826	2.302100819	2.657664838	2.497011263	1.258081225	1.170769537	1.177226476	Kif19a	kinesin family member 19A [Source:MGI Symbol;Acc:MGI:2447024]
ENSMUSG00000010025	42.50310947	51.63676628	42.38482681	64.15241239	66.40489252	73.73154215	24.13240833	23.39477233	24.35223133	34.49348879	35.90434038	38.71490873	Aldh3a2	aldehyde dehydrogenase family 3, subfamily A2 [Source:MGI Symbol;Acc:MGI:1353452]
ENSMUSG00000010044	0.823949767	0.263561253	0.984146015	0.170865863	0.865691202	0.59272165	0.653869857	0.680199697	0.553871244	0.08188929	0.152412236	0.405669199	Zmynd10	zinc finger, MYND domain containing 10 [Source:MGI Symbol;Acc:MGI:2387863]
ENSMUSG00000010045	24.92671287	22.0646658	24.3150752	20.7355927	20.64664311	18.28413913	8.842069875	9.092477996	8.688876994	12.59542382	12.40693955	12.07947528	Tmem115	transmembrane protein 115 [Source:MGI Symbol;Acc:MGI:1930765]
ENSMUSG00000010047	29.3181304	30.09622073	32.67887408	24.33197946	22.34692834	21.421995	33.93403183	32.25739826	29.68080789	31.94555957	31.25697455	34.71558891	Hyal2	hyaluronoglucosaminidase 2 [Source:MGI Symbol;Acc:MGI:1196334]
ENSMUSG00000010048	15.26277751	14.34961677	14.38440823	17.28667705	16.03599242	16.97624198	64.65940452	66.15498423	65.58097212	62.2016405	59.27710296	61.88722141	Ifrd2	interferon-related developmental regulator 2 [Source:MGI Symbol;Acc:MGI:1316708]
ENSMUSG00000010051	3.34306547	4.396122439	2.473108783	2.395119731	3.58040817	4.402725579	0.80873377	0.940118947	1.846878165	3.723397248	3.895870666	3.344991639	Hyal1	hyaluronoglucosaminidase 1 [Source:MGI Symbol;Acc:MGI:96298]
ENSMUSG00000010054	33.57525944	31.84432254	32.81670154	26.46479221	28.59768623	23.75288869	31.47883875	31.86174475	24.82845126	18.36925668	16.28039795	18.63312388	Tusc2	tumor suppressor 2, mitochondrial calcium regulator [Source:MGI Symbol;Acc:MGI:1931086]
ENSMUSG00000010057	19.61278641	16.58211198	20.86685536	13.95602507	15.03951008	11.39116904	30.59455111	35.51448483	35.63459758	20.4097061	20.0247278	19.61553588	Nprl2	NPR2 like, GATOR1 complex subunit [Source:MGI Symbol;Acc:MGI:1914482]
ENSMUSG00000010064	0.635441803	0.801433797	0.588602499	0.790645133	0.985554057	0.576195862	6.375461013	4.676253334	5.125840772	4.032845984	5.566476428	5.390094123	Slc38a3	solute carrier family 38, member 3 [Source:MGI Symbol;Acc:MGI:1923507]
ENSMUSG00000010066	0.122588284	0.268216751	0.163156482	0.274553695	0.180318079	0.196083884	3.195765638	2.635273205	3.502874506	1.425480236	1.479614514	1.227640521	Cacna2d2	calcium channel, voltage-dependent, alpha 2/delta subunit 2 [Source:MGI Symbol;Acc:MGI:1929813]
ENSMUSG00000010067	32.27136587	37.86142424	24.51294567	40.23443243	42.44356232	43.41236201	8.829220492	7.849114429	8.208999472	13.63115476	15.62174206	12.4034448	Rassf1	Ras association (RalGDS/AF-6) domain family member 1 [Source:MGI Symbol;Acc:MGI:1928386]
ENSMUSG00000010080	0.144390899	0.166273542	0.246377238	0.022457202	0.126421472	0.091649946	0.042969635	0.071519879	0	0.172205666	0.300477143	0.447869686	Epn3	epsin 3 [Source:MGI Symbol;Acc:MGI:1919139]
ENSMUSG00000010086	0.072683256	0	0.074412585	0	0	0.01845384	0.043259971	0.02400104	0	0.39008074	0.52434615	0.300597222	Rnf112	ring finger protein 112 [Source:MGI Symbol;Acc:MGI:106611]
ENSMUSG00000010095	263.7134362	231.1823517	262.1043913	322.6097025	282.363066	250.5262895	322.9945105	342.3899212	314.053081	191.1152706	201.3774769	200.3383577	Slc3a2	solute carrier family 3 (activators of dibasic and neutral amino acid transport), member 2 [Source:MGI Symbol;Acc:MGI:96955]
ENSMUSG00000010097	132.0295179	135.9569166	128.4470874	157.1268988	152.7168719	155.7340963	146.886271	148.3304718	157.1753543	170.157325	171.5439826	168.2196295	Nxf1	nuclear RNA export factor 1 [Source:MGI Symbol;Acc:MGI:1858330]
ENSMUSG00000010110	80.86029838	79.23135107	88.175438	77.55893183	74.03681121	67.7646417	24.31084968	24.98519897	26.40922311	40.65717543	42.70917277	42.49270539	Stx5a	syntaxin 5A [Source:MGI Symbol;Acc:MGI:1928483]
ENSMUSG00000010122	0.367764853	0.931700294	0.577322937	0.617745813	0.437915334	0.392167768	2.757989523	3.351776058	3.263264198	4.824702337	4.632724065	4.75846503	Slc47a1	solute carrier family 47, member 1 [Source:MGI Symbol;Acc:MGI:1914723]
ENSMUSG00000010136	0	0	0.047470097	0	0.097431721	0	0.745115704	0.367464204	0.302094893	0.622111525	1.080681588	1.314927748	Pifo	primary cilia formation [Source:MGI Symbol;Acc:MGI:1923670]
ENSMUSG00000010142	0.249007453	0.573489764	0.101972801	0.046473933	0	0.113798683	1.156002553	0.394683775	0.185412739	3.073691759	1.824007548	1.986088786	Tnfrsf13b	tumor necrosis factor receptor superfamily, member 13b [Source:MGI Symbol;Acc:MGI:1889411]
ENSMUSG00000010154	4.640327119	3.334214287	4.515159334	5.36811201	4.452334383	4.907339988	26.5000865	24.8840733	23.52262999	8.28417458	8.603073032	9.601199264	Spire2	spire type actin nucleation factor 2 [Source:MGI Symbol;Acc:MGI:2446256]
ENSMUSG00000010175	0.071999143	0.051819196	0.018428049	0.033594209	0.037823316	0.020565167	0.056244296	0.098072438	0.016753462	0.354208433	0.12735555	0.247253722	Prox1	prospero homeobox 1 [Source:MGI Symbol;Acc:MGI:97772]
ENSMUSG00000010205	0	0	0	0	0	0	0	0	0	0	0.020643732	0	Raver1	ribonucleoprotein, PTB-binding 1 [Source:MGI Symbol;Acc:MGI:1919016]
ENSMUSG00000010277	115.15345	128.439996	110.1854851	118.8602949	132.3877467	162.298746	173.4409134	159.1701175	194.2608284	343.4919191	360.6442674	352.361336	2610507B11	RIKEN cDNA 2610507B11 gene [Source:MGI Symbol;Acc:MGI:1919753]
ENSMUSG00000010290	10.32783253	10.14489794	8.051192055	11.55370458	13.70107508	15.7709573	9.198235735	7.667228823	8.767266542	12.19861514	11.94241105	11.39135065	AI597479	expressed sequence AI597479 [Source:MGI Symbol;Acc:MGI:2138299]
ENSMUSG00000010307	64.29173223	56.69290413	70.63044115	47.28072015	36.76943226	33.92111136	7.458884166	6.713571013	5.406635456	11.61053652	11.22262098	11.18565204	Tmem86a	transmembrane protein 86A [Source:MGI Symbol;Acc:MGI:1915143]
ENSMUSG00000010311	2.993028423	3.173626001	3.397969712	2.157005008	1.556760274	1.354298373	0.132282555	0.088069933	0.137910301	1.060274556	1.356699829	0.682820607	Optc	opticin [Source:MGI Symbol;Acc:MGI:2151113]
ENSMUSG00000010342	15.73151361	17.7218282	11.26769983	18.68413275	20.48093613	21.09945982	20.56749051	18.35621765	22.83148884	26.55780917	27.43200001	27.63533483	Tex14	testis expressed gene 14 [Source:MGI Symbol;Acc:MGI:1933227]
ENSMUSG00000010358	29.22689739	21.50261999	43.06995302	26.55088457	20.00642562	15.85295681	4.348851404	4.240650372	2.782145174	9.968581342	9.338190519	9.255241876	Ifi35	interferon-induced protein 35 [Source:MGI Symbol;Acc:MGI:1917360]
ENSMUSG00000010362	24.40168191	22.10169184	28.22821822	22.92974918	18.85662752	16.12826805	65.96234811	67.24788341	56.17420464	49.27472537	42.69748577	42.24724441	Rdm1	RAD52 motif 1 [Source:MGI Symbol;Acc:MGI:1913849]
ENSMUSG00000010376	219.0861303	170.6255006	261.7900499	191.501202	171.2144823	123.586487	185.9128044	213.1990026	142.9577733	142.1724061	118.764304	127.1304858	Nedd8	neural precursor cell expressed, developmentally down-regulated gene 8 [Source:MGI Symbol;Acc:MGI:97301]
ENSMUSG00000010392	25.98188265	29.16041707	23.56326745	29.49144798	31.7997235	37.43211548	13.76396048	11.89896004	14.2983992	27.22000005	26.55021153	26.01150903	Gosr1	golgi SNAP receptor complex member 1 [Source:MGI Symbol;Acc:MGI:1858260]
ENSMUSG00000010406	144.8625757	112.1942604	219.2123879	142.9272604	126.9062079	93.56202558	179.9095666	172.1272326	105.9395413	89.07215075	79.9910219	78.08166198	Mrpl52	mitochondrial ribosomal protein L52 [Source:MGI Symbol;Acc:MGI:1916086]
ENSMUSG00000010435	0	0	0	0	0	0.039871071	0	0.051856262	0	0	0	0	Spaca7	sperm acrosome associated 7 [Source:MGI Symbol;Acc:MGI:1925884]
ENSMUSG00000010453	37.98193679	43.78520825	38.04892835	54.20528845	51.97024631	55.26141846	32.92441197	31.19013832	35.65629587	32.05502992	29.93243155	31.90416812	Kansl3	KAT8 regulatory NSL complex subunit 3 [Source:MGI Symbol;Acc:MGI:1918055]
ENSMUSG00000010461	1.796452322	2.287512765	1.962986661	1.628061526	1.742271602	2.289313364	0.092528753	0.08555971	0.064310159	0.695286426	0.632654565	1.010345929	Eya4	EYA transcriptional coactivator and phosphatase 4 [Source:MGI Symbol;Acc:MGI:1337104]
ENSMUSG00000010476	6.424836672	7.33957727	7.154232186	7.452638361	7.073044453	7.486748319	0.11426191	0.063393587	0.083386098	0.206062878	0.170455089	0.26087353	Ebf3	early B cell factor 3 [Source:MGI Symbol;Acc:MGI:894289]
ENSMUSG00000010492	0.759645435	0.49986901	1.166579135	1.215237665	0.912147659	0.785253962	0.290654634	0.752537034	0.505033437	0.339742452	0.316164096	0.625129585	Uckl1os	uridine-cytidine kinase 1-like 1, opposite strand [Source:MGI Symbol;Acc:MGI:3801877]
ENSMUSG00000010505	0	0	0	0	0.035250151	0.025554792	0	0	0	0	0.041891082	0.118933036	Myt1	myelin transcription factor 1 [Source:MGI Symbol;Acc:MGI:1100535]
ENSMUSG00000010517	73.009916	64.70250872	77.90340819	84.10131339	80.04172667	74.49878627	79.38272025	80.95812611	84.81853015	62.08832374	59.08110768	55.912421	Faf1	Fas-associated factor 1 [Source:MGI Symbol;Acc:MGI:109419]
ENSMUSG00000010529	1.062431798	0.367033449	1.051452885	0.462673819	0.706961357	0.269745026	0.727194768	0.771826049	0.560358499	0.760256126	0.530620377	0.251080854	Gm266	predicted gene 266 [Source:MGI Symbol;Acc:MGI:2685112]
ENSMUSG00000010538	1.00787449	0.892784065	1.26997478	0.217045826	0.977477153	0.708627473	2.145694552	1.689673242	1.298891401	1.45630143	0.83895384	1.236742855	Tsacc	TSSK6 activating co-chaperone [Source:MGI Symbol;Acc:MGI:1924177]
ENSMUSG00000010554	15.84382304	15.15613414	14.99711618	17.3531503	15.39068719	17.55146196	32.21090477	30.77156641	32.64988968	47.19002595	44.99090076	46.69310132	Mettl16	methyltransferase like 16 [Source:MGI Symbol;Acc:MGI:1914743]
ENSMUSG00000010592	70.99255676	80.52223145	62.60216396	97.63023419	108.1759347	116.5047543	5.506746828	6.551043444	7.893905814	8.488042205	6.640068576	8.777663795	Dazl	deleted in azoospermia-like [Source:MGI Symbol;Acc:MGI:1342328]
ENSMUSG00000010601	3.919779429	2.598870385	3.131388385	3.657873757	3.619161441	3.121335299	0.159067719	0.176504545	0.082917498	0.292179179	0.370775108	0.315800453	Apol7a	apolipoprotein L 7a [Source:MGI Symbol;Acc:MGI:1923011]
ENSMUSG00000010607	49.61448086	33.37008195	66.65632027	40.3876089	34.20279479	26.8020781	70.61389356	82.14485519	51.72528899	24.06900521	24.95694822	24.95870875	Pigyl	phosphatidylinositol glycan anchor biosynthesis, class Y-like [Source:MGI Symbol;Acc:MGI:1913518]
ENSMUSG00000010608	94.2467654	111.9298724	81.19176662	111.9314132	128.9623926	159.8809026	157.555911	154.0227528	186.6730141	206.3101968	192.0989819	192.2571865	Rbm25	RNA binding motif protein 25 [Source:MGI Symbol;Acc:MGI:1914289]
ENSMUSG00000010609	22.34298983	19.92669272	24.84302554	13.02358565	11.14534813	9.165615941	10.15122877	9.950409748	11.35445898	12.18686763	12.41725267	13.01454297	Psen2	presenilin 2 [Source:MGI Symbol;Acc:MGI:109284]
ENSMUSG00000010651	2.388365404	2.008174201	2.212315649	2.264160865	1.43392023	1.414913666	6.904520027	7.436050964	5.998551329	4.442223431	4.481054025	5.039590928	Acaa1b	acetyl-Coenzyme A acyltransferase 1B [Source:MGI Symbol;Acc:MGI:3605455]
ENSMUSG00000010660	41.06192074	38.31708429	39.09939209	41.87853584	38.01796424	35.72488081	22.1745707	21.2544789	23.30099172	11.59212515	12.32637442	11.65946674	Plcd1	phospholipase C, delta 1 [Source:MGI Symbol;Acc:MGI:97614]
ENSMUSG00000010663	89.8950568	96.16404252	93.50742446	120.8521748	120.2509764	119.4024947	65.18478722	61.57726988	71.39075233	41.38462168	44.12838735	43.84619703	Fads1	fatty acid desaturase 1 [Source:MGI Symbol;Acc:MGI:1923517]
ENSMUSG00000010721	12.48688996	15.05420395	11.00056191	18.59548122	22.06535402	24.25500733	4.636526087	4.273874249	4.879238286	4.601663781	5.258020312	5.540308513	Lmbr1	limb region 1 [Source:MGI Symbol;Acc:MGI:1861746]
ENSMUSG00000010751	16.73872631	17.25123853	17.27696059	21.30704294	21.73200596	23.49077557	9.712698011	9.190775151	9.980473984	12.56143796	11.2344353	12.03012654	Tnfrsf22	tumor necrosis factor receptor superfamily, member 22 [Source:MGI Symbol;Acc:MGI:1930270]
ENSMUSG00000010755	108.1881155	102.251375	122.0515749	137.0831098	125.0925511	114.474133	95.69291606	103.3562221	96.64190417	83.97688003	86.58954305	90.78774889	Cars	cysteinyl-tRNA synthetase [Source:MGI Symbol;Acc:MGI:1351477]
ENSMUSG00000010760	3.276718512	0.496488133	2.736712938	0.482807349	0.452989162	1.576305602	121.711191	132.2340927	102.6510317	44.27273086	46.51169401	43.86430319	Phlda2	pleckstrin homology like domain, family A, member 2 [Source:MGI Symbol;Acc:MGI:1202307]
ENSMUSG00000010796	6.896552056	8.977612587	6.101313398	8.499385988	7.63973324	8.707383532	0.066924833	0.519827411	0.418632246	1.374570228	0.811183591	1.096154926	Asz1	ankyrin repeat, SAM and basic leucine zipper domain containing 1 [Source:MGI Symbol;Acc:MGI:1921318]

ENSMUSG00000010797	0.114488216	0.087892711	0.058606098	0.053419261	0	0.043601801	0.08517706	0.018902815	0.124321091	0.921663053	0.555916027	0.422759475	Wnt2	wingless-type MMTV integration site family, member 2 [Source:MGI Symbol;Acc:MGI:98954]
ENSMUSG00000010803	0	0	0	0	0	0	0.02159351	0.047921133	0.247633877	0.23798068	0.22146466	0.235785245	Gabra1	gamma-aminobutyric acid (GABA) A receptor, subunit alpha 1 [Source:MGI Symbol;Acc:MGI:95613]
ENSMUSG00000010825	0	0.033856861	0	0.013718305	0.061781098	0.044788549	0.026248619	0.087377891	0.136826619	0.184090034	0.26920781	0.091195877	Grid2ip	glutamate receptor, ionotropic, delta 2 (Grid2) interacting protein 1 [Source:MGI Symbol;Acc:MGI:2176213]
ENSMUSG00000010830	77.48787213	68.40712717	79.01930918	51.50800488	42.70920507	38.44153377	58.69767922	62.29234441	57.56178546	25.83139912	25.38403648	26.13360654	Kdelr3	KDEL (Lys-Asp-Glu-Leu) endoplasmic reticulum protein retention receptor 3 [Source:MGI Symbol;Acc:MGI:2145953]
ENSMUSG00000010841	0	0	0.083749525	0.534361872	0	0.186924072	0	0	0.228417214	0	0.204278757	0	Cfap97d1	CFAP97 domain containing 1 [Source:MGI Symbol;Acc:MGI:1922687]
ENSMUSG00000010911	47.3041893	38.29536696	53.50194425	46.93016889	42.85520399	38.25358876	42.59565049	44.50837725	39.35429243	29.80692664	29.82856507	26.70506259	Apip	APAF1 interacting protein [Source:MGI Symbol;Acc:MGI:1926788]
ENSMUSG00000010914	13.15991552	17.33949258	14.58316186	16.88009893	17.24441009	19.07125141	24.66383242	23.19275791	23.969853	26.05542741	24.32921782	24.41728949	Pdhx	pyruvate dehydrogenase complex, component X [Source:MGI Symbol;Acc:MGI:1351627]
ENSMUSG00000010936	14.21111868	11.57933582	14.66083438	13.26158245	13.87171009	14.46706294	39.38799794	34.52720659	37.86788045	30.11518838	29.25007025	28.69024115	Vac14	Vac14 homolog (S. cerevisiae) [Source:MGI Symbol;Acc:MGI:2157980]
ENSMUSG00000011008	5.753809419	6.877876079	5.730634292	1.138070956	1.412759948	1.405281408	0	0.154890435	0.203738416	1.874050688	1.249425889	1.136227538	Mcoln2	mucolipin 2 [Source:MGI Symbol;Acc:MGI:1915529]
ENSMUSG00000011034	0.094360719	0.072440812	0.053669895	0.009783986	0.022031344	0.055901107	0.187206891	0.114250566	0.234205564	0.67522748	0.855276266	0.715456545	Slc5a1	solute carrier family 5 (sodium/glucose cotransporter), member 1 [Source:MGI Symbol;Acc:MGI:107678]
ENSMUSG00000011096	191.5616096	154.6105209	198.2128548	142.7092766	124.3800144	104.9829094	59.70033439	61.46052459	59.64853407	70.43803755	75.94336489	72.02453585	Akt1s1	AKT1 substrate 1 (proline-rich) [Source:MGI Symbol;Acc:MGI:1914855]
ENSMUSG00000011114	101.5783241	99.07015624	92.75103728	109.1818925	101.8767873	93.48999477	118.5129589	121.1209985	114.0794013	72.80416016	77.24840102	76.31025332	Tbrg1	transforming growth factor beta regulated gene 1 [Source:MGI Symbol;Acc:MGI:1100877]
ENSMUSG00000011118	0	0	0	0	0	0	0	0	0	0.027413081	0.05102119	0.054320377	Panx3	pannexin 3 [Source:MGI Symbol;Acc:MGI:1918881]
ENSMUSG00000011148	14.05990897	14.44840078	16.20790337	11.74299823	11.32146566	11.15890754	13.50779603	11.9908045	13.25812392	9.935572794	10.44402887	10.72692345	Adssl1	adenylosuccinate synthetase like 1 [Source:MGI Symbol;Acc:MGI:87947]
ENSMUSG00000011154	2.256738872	3.014550951	2.495267319	1.375888401	1.865238309	1.283458151	0.053727069	0.05961657	0	0.134573309	0.150280596	0.079999101	Cfap161	cilia and flagella associated protein 161 [Source:MGI Symbol;Acc:MGI:1922806]
ENSMUSG00000011158	42.48151553	42.11708828	45.35888474	43.99868376	42.25828402	42.46735386	34.18270913	34.67865576	37.87699415	25.44119811	27.40901573	25.43270847	Brf1	BRF1, RNA polymerase III transcription initiation factor 90 kDa subunit [Source:MGI Symbol;Acc:MGI:1919558]
ENSMUSG00000011171	0.977920178	0.925031542	1.322997774	0.130368435	0.330255572	0.266022895	0	0.034598902	0.032507428	0.281162092	0.232577114	0.30952033	Vipr2	vasoactive intestinal peptide receptor 2 [Source:MGI Symbol;Acc:MGI:107166]
ENSMUSG00000011179	245.1156048	252.1065122	191.7498754	264.9624915	265.8525076	275.8431037	744.5033208	726.0169069	815.0757301	715.5168614	740.6441145	732.8432284	Odc1	ornithine decarboxylase, structural 1 [Source:MGI Symbol;Acc:MGI:97402]
ENSMUSG00000011254	15.16551231	14.24782298	14.69516573	15.60682346	13.81839165	12.34281985	29.80412829	34.22935996	27.17297149	36.10606958	33.00558865	32.348215	Thg1l	tRNA-histidine guanylyltransferase 1-like (S. cerevisiae) [Source:MGI Symbol;Acc:MGI:1913878]
ENSMUSG00000011256	39.05521895	52.12187346	33.72340345	40.58064249	46.88198801	59.35584333	14.13993565	11.30038431	17.3319094	46.75878468	49.35108204	43.50396488	Adam19	a disintegrin and metallopeptidase domain 19 (meltrin beta) [Source:MGI Symbol;Acc:MGI:105377]
ENSMUSG00000011257	104.6671483	102.6630786	101.0053696	123.7612332	117.4444963	116.0025822	205.6746718	200.6062781	211.9181866	148.5588763	156.0986759	153.8852389	Pabpc4	poly(A) binding protein, cytoplasmic 4 [Source:MGI Symbol;Acc:MGI:2385206]
ENSMUSG00000011263	0.290733026	0.143483153	0.113390605	0.633050326	0.509102684	0.463982274	0.1483199	0.246867844	0.335031512	1.064982338	1.42898731	1.288273807	Exoc3l2	exocyst complex component 3-like 2 [Source:MGI Symbol;Acc:MGI:1921713]
ENSMUSG00000011267	2.538085918	2.055049035	1.380439177	1.036218783	0.666666446	1.268671767	33.38728792	38.77577113	31.89167458	24.19243601	23.86691334	22.31741426	Zfp296	zinc finger protein 296 [Source:MGI Symbol;Acc:MGI:1926956]
ENSMUSG00000011305	0.064997716	0.149696422	0.288358133	0.141527867	0.045527007	0.280542976	1.740854414	2.339485095	1.87541344	1.16277844	1.244392955	1.305658368	Plin5	perilipin 5 [Source:MGI Symbol;Acc:MGI:1914218]
ENSMUSG00000011306	24.4941468	23.341819	24.89253864	24.64519016	25.15013753	21.93664471	36.95310163	35.19714213	36.95555268	35.91038567	35.84413338	35.18732643	Sugp1	SURP and G patch domain containing 1 [Source:MGI Symbol;Acc:MGI:1917866]
ENSMUSG00000011349	0.770738818	0.734520306	0.952334007	0.57043236	0.949402609	0.890708405	1.518561248	1.500724718	1.92948352	1.331269992	0.840667862	0.635940968	Dmrtc2	doublesex and mab-3 related transcription factor like family C2 [Source:MGI Symbol;Acc:MGI:1918491]
ENSMUSG00000011350	0	0	0	0	0	0	0	0	0	0	0.02890789	0	Gm5893	predicted gene 5893 [Source:MGI Symbol;Acc:MGI:3645761]
ENSMUSG00000011382	1.105833788	1.653551154	1.233530596	0.539076227	0.606939448	0.942865956	7.957046873	8.240670211	9.171209612	4.428994979	5.055853539	4.666051629	Dhdh	dihydrodiol dehydrogenase (dimeric) [Source:MGI Symbol;Acc:MGI:1919005]
ENSMUSG00000011427	3.994205621	5.393997903	2.806704548	4.083117366	5.074013789	6.181429209	1.78296791	1.546760718	2.16299215	5.066770257	5.062722905	5.341824623	Zfp790	zinc finger protein 790 [Source:MGI Symbol;Acc:MGI:1923431]
ENSMUSG00000011463	0.067814796	0.039046112	0	0.063283653	0	0.258266514	0.030271753	0.201540651	0	0.212305567	0.197571417	0.210346992	Cpb1	carboxypeptidase B1 (tissue) [Source:MGI Symbol;Acc:MGI:1923953]
ENSMUSG00000011486	0.071773958	0	0.110222478	0.100467417	0.037705019	0.082003388	1.762149269	1.599802457	1.569900018	1.444502491	2.120932576	1.208549106	Slc25a41	solute carrier family 25, member 41 [Source:MGI Symbol;Acc:MGI:2144215]
ENSMUSG00000011589	15.01305494	12.5031488	16.87983419	12.75904903	8.379735021	11.07782317	38.83370762	33.0649099	36.80529501	44.54292681	44.24109385	45.79512386	Fsd1	fibronectin type 3 and SPRY domain-containing protein [Source:MGI Symbol;Acc:MGI:1934858]
ENSMUSG00000011632	0	0	0	0	0	0	0.273415331	0.606773633	0.095015781	0.593527358	0.339899174	0.271408574	Pinlyp	phospholipase A2 inhibitor and LY6/PLAUR domain containing [Source:MGI Symbol;Acc:MGI:3615324]
ENSMUSG00000011658	6.899891144	6.363820492	7.194873826	7.154294492	6.846694093	7.956276725	4.477455152	5.158138957	5.381511823	3.200187256	2.579240476	3.312212211	Fuz	fuzzy planar cell polarity protein [Source:MGI Symbol;Acc:MGI:1917550]
ENSMUSG00000011751	0.507085243	0.53084975	0.792883869	0.507618426	0.494027049	0.568822728	8.856735158	7.736038783	9.8685503	5.442909322	6.024479805	5.907454487	Sptbn4	spectrin beta, non-erythrocytic 4 [Source:MGI Symbol;Acc:MGI:1890574]
ENSMUSG00000011752	365.6772795	385.4404532	295.9248646	387.6525709	402.6947995	378.8610908	513.523592	480.7081779	543.0584446	435.8617784	480.1261157	470.6251956	Pgam1	phosphoglycerate mutase 1 [Source:MGI Symbol;Acc:MGI:97552]
ENSMUSG00000011831	45.66590891	60.264769	37.16529373	54.36758776	67.34544944	87.5328182	28.83759706	27.27232267	34.91888831	51.71489145	48.07650848	50.16105231	Evi5	ecotropic viral integration site 5 [Source:MGI Symbol;Acc:MGI:104736]
ENSMUSG00000011832	10.02004011	10.94813031	11.28647522	13.21834946	12.07650212	13.52294496	6.29439019	5.143039221	6.005384191	5.331749065	6.01097513	6.32392759	Evi5l	ecotropic viral integration site 5 like [Source:MGI Symbol;Acc:MGI:2442167]
ENSMUSG00000011837	46.08401274	37.78873565	53.09410311	45.15313266	39.623244	33.15659675	49.40120339	47.89188141	43.51644188	30.17636274	31.98433044	32.71714196	Snapc2	small nuclear RNA activating complex, polypeptide 2 [Source:MGI Symbol;Acc:MGI:1914861]
ENSMUSG00000011877	102.2077246	94.46330626	98.0501905	105.9286987	97.60944477	88.60920089	86.47108328	83.79501377	88.80366483	89.52495035	90.01372739	90.7285413	Git1	GIT ArfGAP 1 [Source:MGI Symbol;Acc:MGI:1927140]
ENSMUSG00000011884	68.63191166	64.22771118	64.26800885	64.69935384	60.6519134	57.41065601	61.99657528	59.44991944	60.99952103	42.73877773	40.04866385	39.63471429	Gltp	glycolipid transfer protein [Source:MGI Symbol;Acc:MGI:1929253]
ENSMUSG00000011958	165.2533175	169.0679711	163.9622591	171.0873042	174.2534854	174.1337581	28.55200519	31.54243023	34.22024803	53.97126928	47.85345424	53.25511137	Bnip2	BCL2/adenovirus E1B interacting protein 2 [Source:MGI Symbol;Acc:MGI:109327]
ENSMUSG00000011960	27.61051824	32.05835221	24.5671507	29.41599812	32.35002472	42.18283039	29.93984754	26.7176366	34.06626392	47.36823081	50.87862375	46.38388407	Ccnt1	cyclin T1 [Source:MGI Symbol;Acc:MGI:1328363]
ENSMUSG00000012017	50.80200025	47.02375321	51.2346823	36.96287165	35.12625335	30.50145924	1.45423548	1.661577272	1.44104893	7.256164575	7.584908038	8.58990844	Scarf2	scavenger receptor class F, member 2 [Source:MGI Symbol;Acc:MGI:1858430]
ENSMUSG00000012042	0.075356371	0	0	0	0	0.057397584	0.022425484	0	0.070138619	0.04493633	0	0	4930579F01	RIKEN cDNA 4930579F01 gene [Source:MGI Symbol;Acc:MGI:1914991]
ENSMUSG00000012076	44.55366516	44.91921717	32.03369859	43.99199505	52.5407094	54.74071116	22.77658372	22.66358175	29.59593582	42.62201217	42.35701386	39.97606335	Brms1l	breast cancer metastasis-suppressor 1-like [Source:MGI Symbol;Acc:MGI:1196337]
ENSMUSG00000012114	40.96510126	50.72172283	43.68608699	45.15982578	45.67080754	49.03529942	47.79751024	39.84590111	53.49276057	44.935439	50.54209986	49.9092429	Med15	mediator complex subunit 15 [Source:MGI Symbol;Acc:MGI:2137379]
ENSMUSG00000012117	15.85838091	14.29069746	17.62989492	13.11803258	12.92325588	10.90794357	12.13823107	12.59845116	12.8386254	12.12203943	12.35947311	12.59417526	Dhdds	dehydrodolichyl diphosphate synthase [Source:MGI Symbol;Acc:MGI:1914672]
ENSMUSG00000012123	0.748061332	0.758059349	0.949666033	1.005233103	0.848834906	1.071195389	20.81027213	19.38616439	20.69298997	5.915049074	6.027596569	6.364323453	Crybg2	crystallin beta-gamma domain containing 2 [Source:MGI Symbol;Acc:MGI:1334463]
ENSMUSG00000012126	2.113989167	1.775059413	3.020983381	2.096036202	1.943448691	2.34818981	4.757622664	4.581078821	5.451931579	4.490919311	5.02242778	4.722703651	Ubxn11	UBX domain protein 11 [Source:MGI Symbol;Acc:MGI:1914836]
ENSMUSG00000012187	0.215411704	0.062014413	0.055134231	0	0	0.082037599	0	0	0	0.096340341	0	0.23862894	Mogat1	monoacylglycerol O-acyltransferase 1 [Source:MGI Symbol;Acc:MGI:1915643]
ENSMUSG00000012211	0.099029891	0.057019005	0.152079115	0	0.052023381	0	0	0	0	0	0	0	Tex22	testis expressed gene 22 [Source:MGI Symbol;Acc:MGI:1922921]
ENSMUSG00000012282	0.246440366	0.05675775	0.126151919	0.20697669	0.051785015	0.075083667	0.132009808	0.073240288	0.13762595	1.168305967	1.005170045	1.004646973	Wnt8a	wingless-type MMTV integration site family, member 8A [Source:MGI Symbol;Acc:MGI:107924]
ENSMUSG00000012296	84.07726119	87.74684175	92.40631638	99.45997608	93.26072809	88.75143241	88.19184808	85.93210799	96.83245556	65.88127953	65.64745628	67.17120112	Tjap1	tight junction associated protein 1 [Source:MGI Symbol;Acc:MGI:1921344]
ENSMUSG00000012350	0.504343781	0.73361405	0.516343463	0.371562102	0.306780692	0.303275947	0.142189466	0.131480098	0.024706448	0.712300426	0.331433148	0.611632031	Ehf	ets homologous factor [Source:MGI Symbol;Acc:MGI:1270840]
ENSMUSG00000012396	1.329065261	0.263052448	0.297650339	0.11627455	0.043637373	1.834838993	145.4832818	140.3237969	157.4132674	171.7841279	161.4870901	163.9052361	Nanog	Nanog homeobox [Source:MGI Symbol;Acc:MGI:1919200]
ENSMUSG00000012405	506.963246	465.2118816	554.7302525	516.9375449	500.5100568	404.2727496	982.7133973	1015.456941	852.1301396	806.6875405	740.4246526	732.2937765	Rpl15	ribosomal protein L15 [Source:MGI Symbol;Acc:MGI:1913730]
ENSMUSG00000012422	29.26027308	29.55585142	29.33356173	31.87885436	32.4945886	30.44834386	16.23502866	14.77785346	14.80738464	18.84590131	19.16985951	17.68352297	Tmem167	transmembrane protein 167 [Source:MGI Symbol;Acc:MGI:1913324]
ENSMUSG00000012428	49.35660775	66.47958784	41.23178917	68.44545287	80.19998662	94.83170662	0.90101401	0.486380513	0.698162705	4.159872118	4.711253034	4.29348356	Steap4	STEAP family member 4 [Source:MGI Symbol;Acc:MGI:1923560]
ENSMUSG00000012429	7.828012649	6.755859443	6.826963863	7.989316636	7.400331145	7.339406128	7.140930067	7.281704462	5.682757132	7.596911317	5.749438989	6.786236308	Mplkip	M-phase specific PLK1 intereacting protein [Source:MGI Symbol;Acc:MGI:1913558]
ENSMUSG00000012443	2.924161015	5.100868837	2.095614218	5.669796392	8.317966452	12.32428564	15.34466987	12.64935173	21.55174577	30.78649814	32.77879669	31.24153153	Kif11	kinesin family member 11 [Source:MGI Symbol;Acc:MGI:1098231]
ENSMUSG00000012483	45.5745522	41.70661892	44.3129144	47.81590186	52.76533014	36.5556384	94.73012091	99.25012467	78.20248522	57.6750147	53.88426385	57.65063401	Rpa3	replication protein A3 [Source:MGI Symbol;Acc:MGI:1915490]
ENSMUSG00000012519	11.53543619	11.09486488	10.50141227	3.149892016	3.787444797	4.44308886	2.750187573	3.92820074	3.751748391	9.174968373	11.42975691	9.584050683	Mlkl	mixed lineage kinase domain-like [Source:MGI Symbol;Acc:MGI:1921818]
ENSMUSG00000012520	0.061615973	0	0	0	0	0.046931772	0	0	0	0	0	0.127413047	Phox2b	paired-like homeobox 2b [Source:MGI Symbol;Acc:MGI:1100882]
ENSMUSG00000012535	14.41837902	23.71068799	10.22769018	20.97156926	26.57912071	35.13778712	48.28317558	38.53252278	62.10214865	70.94074515	72.86731434	73.74730716	Tnpo3	transportin 3 [Source:MGI Symbol;Acc:MGI:1196412]
ENSMUSG00000012609	9.984690675	11.08590826	9.256633073	9.302374117	10.61011553	11.42012359	5.981451201	5.670558727	6.614315652	8.669247149	8.365392686	8.646917163	Ttll5	tubulin tyrosine ligase-like family, member 5 [Source:MGI Symbol;Acc:MGI:2443657]
ENSMUSG00000012640	6.108266915	7.902101548	5.600533156	7.594166293	9.84997053	13.54541733	4.883829156	3.848964685	5.505408969	16.51213292	16.49249657	15.96579837	Zfp715	zinc finger protein 715 [Source:MGI Symbol;Acc:MGI:1917180]

ENSMUSG00000012705	0.085458764	0.04920508	0.087492056	0.518366645	0.359152479	0.423100564	0.267034735	0.084659082	0.357936806	0.038220423	0.248975301	0.189339149	Retn	resistin [Source:MGI Symbol;Acc:MGI:1888506]
ENSMUSG00000012777	0.060518267	0	0.154895395	0	0	0.046095669	0.135073327	0	0.112655841	0.02706608	0.050375352	0.214531109	Acp4	acid phosphatase 4 [Source:MGI Symbol;Acc:MGI:3644563]
ENSMUSG00000012819	0.266521948	0.221659912	0.136431608	0.255622622	0.186682664	0.219922024	0.218115999	0.212689192	0.254958466	0.430440092	0.332778953	0.406785976	Cdh23	cadherin 23 (otocadherin) [Source:MGI Symbol;Acc:MGI:1890219]
ENSMUSG00000012848	1223.82949	870.9915496	1369.912027	1181.490346	992.3805471	762.9203753	2245.840719	2457.106877	1857.202384	1175.560655	1197.79897	1220.888498	Rps5	ribosomal protein S5 [Source:MGI Symbol;Acc:MGI:1097682]
ENSMUSG00000012889	55.10547618	48.8891144	52.46206825	58.60109682	52.58058822	47.40734463	0.391527325	0.267351456	0.15699393	0.30174807	0.631814738	0.627825165	Podnl1	podocan-like 1 [Source:MGI Symbol;Acc:MGI:2685352]
ENSMUSG00000013033	19.1213275	19.00390525	19.28341916	16.27288734	17.08460612	16.48337985	49.19969421	46.15711199	49.09118701	42.32593796	46.35818903	46.2352938	Adgrl1	adhesion G protein-coupled receptor L1 [Source:MGI Symbol;Acc:MGI:1929461]
ENSMUSG00000013076	39.85400848	42.87938852	39.7240396	45.36219821	49.60269335	69.67344247	6.406952934	5.931848736	7.435699111	25.07100747	25.00919592	24.9863858	Amotl1	angiomotin-like 1 [Source:MGI Symbol;Acc:MGI:1922973]
ENSMUSG00000013083	0	0	0	0	0	0	0.217033074	0	0.075422131	0.072482046	0	0.215440139	2200002J24I	RIKEN cDNA 2200002J24 gene [Source:MGI Symbol;Acc:MGI:1916397]
ENSMUSG00000013089	22.97081315	24.47229129	21.09716533	28.61102416	30.58209771	35.39303227	164.5528627	141.7628775	175.4206176	224.5416151	226.1114181	221.5178947	Etv5	ets variant 5 [Source:MGI Symbol;Acc:MGI:1096867]
ENSMUSG00000013091	0.172458869	0.198595253	0	0.321871566	0.181195665	0.0656794	0.153967351	0.341690162	0	0.308520773	0	0.45851188	Tmem190	transmembrane protein 190 [Source:MGI Symbol;Acc:MGI:1925302]
ENSMUSG00000013150	8.958751825	8.18202258	8.99117722	9.801566897	7.697005996	6.655647268	8.727066848	8.700044813	8.317900701	8.941050649	8.559314858	8.056798324	Gfod2	glucose-fructose oxidoreductase domain containing 2 [Source:MGI Symbol;Acc:MGI:1917825]
ENSMUSG00000013155	14.18978079	10.04586216	15.06461605	12.73159053	9.004908939	10.09200026	14.87404411	15.96303341	12.84096271	9.309081297	8.226233596	8.138119903	Enkd1	enkurin domain containing 1 [Source:MGI Symbol;Acc:MGI:2142593]
ENSMUSG00000013158	0.447281578	0.360547411	0.274754159	0.375656238	0.187976376	0.204411771	0.998307018	1.019121099	0.874253827	0.360074529	0.335085114	0.792783881	4933405L10	RIKEN cDNA 4933405L10 gene [Source:MGI Symbol;Acc:MGI:1918296]
ENSMUSG00000013160	155.6547459	134.305266	161.718129	108.459676	99.59426523	83.32117089	170.3756545	178.2753969	166.9718385	125.70083	125.3927267	128.447679	Atp6v0d1	ATPase, H+ transporting, lysosomal V0 subunit D1 [Source:MGI Symbol;Acc:MGI:1201778]
ENSMUSG00000013236	179.7113906	212.2071115	176.4270812	238.5796851	254.7381811	302.7720993	43.00012543	37.13935245	46.96633171	78.11913967	78.25337151	77.94382499	Ptprs	protein tyrosine phosphatase, receptor type, S [Source:MGI Symbol;Acc:MGI:97815]
ENSMUSG00000013275	19.24112743	23.29408926	18.05343018	24.12636929	26.02976281	27.70226718	23.55318427	21.22194018	28.12416404	25.79556337	27.71316406	27.75521985	Slc41a1	solute carrier family 41, member 1 [Source:MGI Symbol;Acc:MGI:2444823]
ENSMUSG00000013338	0.176185801	0.157801005	0	0.164413695	0.020567934	0.089465024	0.087385928	0.193930153	0.127545069	0.332698526	0.342199447	0.346978205	Fer1l4	fer-1-like 4 (C. elegans) [Source:MGI Symbol;Acc:MGI:1921812]
ENSMUSG00000013353	0.065293608	0.175440875	0.200541351	0.142172152	0.137202787	0.2486648	0.019430882	0.064682622	0.101287687	0.136275045	0.217401369	0.019288267	4931406B18	RIKEN cDNA 4931406B18 gene [Source:MGI Symbol;Acc:MGI:1921304]
ENSMUSG00000013367	51.52229098	46.708911	50.19665389	47.36781428	47.88020478	43.38877437	3.541795056	3.82927237	3.739814386	10.78208987	12.42583091	10.70515941	Iglon5	IgLON family member 5 [Source:MGI Symbol;Acc:MGI:2686277]
ENSMUSG00000013415	18.29184615	21.12948508	18.74646418	16.82200767	18.4418074	21.85897235	106.6139122	102.4710356	120.0768834	169.8557036	171.7947754	169.1286715	Igf2bp1	insulin-like growth factor 2 mRNA binding protein 1 [Source:MGI Symbol;Acc:MGI:1890357]
ENSMUSG00000013418	0.024987507	0.172646396	0.102328107	0.116589657	0.341293925	0.114195194	1.182338714	1.237684312	1.302411432	1.698655891	1.705565499	1.306528093	B4galnt2	beta-1,4-N-acetyl-galactosaminyl transferase 2 [Source:MGI Symbol;Acc:MGI:1342058]
ENSMUSG00000013419	20.64183431	20.48716395	21.00536333	18.7683345	18.6103758	17.39562428	1.168301138	1.327234939	1.029503895	4.22224975	4.66838348	4.459423684	Zfp651	zinc finger protein 651 [Source:MGI Symbol;Acc:MGI:2670992]
ENSMUSG00000013465	66.52502471	66.8059826	67.73545487	69.64914329	61.18217893	57.68005999	78.29096633	75.91940922	70.61921393	48.96398089	53.22575409	52.47706579	Nelfb	negative elongation factor complex member B [Source:MGI Symbol;Acc:MGI:1931035]
ENSMUSG00000013483	0.093999973	0.284145229	0.252620768	0.219298077	0.135797789	0.080547839	0.356664402	0.384121511	0.240601129	0.168161513	0.156490991	0.239502132	Card14	caspase recruitment domain family, member 14 [Source:MGI Symbol;Acc:MGI:2386258]
ENSMUSG00000013495	17.72163236	18.30892637	15.76459562	19.64098122	18.06896789	19.36252357	7.280316441	7.423983003	9.074132796	7.64054388	6.882755849	7.529684197	Tmem175	transmembrane protein 175 [Source:MGI Symbol;Acc:MGI:1919642]
ENSMUSG00000013523	1.358502515	1.085491966	1.447593273	1.009011363	0.349548853	0.844691254	3.836535039	2.938766562	3.044974149	6.918906326	8.354193894	8.402998327	Bcas1	breast carcinoma amplified sequence 1 [Source:MGI Symbol;Acc:MGI:1924210]
ENSMUSG00000013539	9.750738406	14.57641676	10.76120457	10.93577047	10.99661798	10.18651993	12.0196165	10.01397254	10.07473458	10.52217792	11.46735722	10.62330401	Tango2	transport and golgi organization 2 [Source:MGI Symbol;Acc:MGI:101825]
ENSMUSG00000013562	0	0.051453287	0.091489616	0.041696239	0.046945294	0	0.039890814	0.177054404	0.374291136	1.039135125	1.264562582	1.979901593	4833413E03	RIKEN cDNA 4833413E03 gene [Source:MGI Symbol;Acc:MGI:1925354]
ENSMUSG00000013584	0.665193054	1.116177251	0.933969969	0.780367944	0.419335108	0.448760883	0.373289218	0.60248548	0.512997082	0.917999198	0.933390306	0.943216724	Aldh1a2	aldehyde dehydrogenase family 1, subfamily A2 [Source:MGI Symbol;Acc:MGI:107928]
ENSMUSG00000013593	212.2322999	198.8204236	211.8764266	210.8154575	190.0496552	162.0651571	226.8517759	230.8797123	218.8966668	189.502103	189.2584464	191.4750779	Ndufs2	NADH:ubiquinone oxidoreductase core subunit S2 [Source:MGI Symbol;Acc:MGI:2385112]
ENSMUSG00000013611	0.710725195	0.511522873	0.545726431	0.552697561	0.808958209	0.744351467	0.660957572	0.410710164	0.661513859	0.821147252	1.429716984	1.338457081	Snx31	sorting nexin 31 [Source:MGI Symbol;Acc:MGI:1913946]
ENSMUSG00000013622	93.76195078	73.16832635	113.5470358	78.78790859	65.56061299	52.03340729	21.6537702	24.29619566	18.08019703	18.0548846	16.6663645	19.37901714	Atraid	all-trans retinoic acid induced differentiation factor [Source:MGI Symbol;Acc:MGI:1918918]
ENSMUSG00000013629	36.3685142	40.38518515	31.52014454	44.51182947	46.51921786	51.38278079	134.6326234	114.3147734	150.2941709	117.0474557	128.432691	119.9572723	Cad	carbamoyl-phosphate synthetase 2, aspartate transcarbamylase, and dihydroorotase [Source:MGI Symbol;Acc:MGI:1916969]
ENSMUSG00000013643	0	0	0	0	0	0	0	0	0	0	0	0	Lypd8	LY6/PLAUR domain containing 8 [Source:MGI Symbol;Acc:MGI:1917413]
ENSMUSG00000013646	21.62177378	20.70754459	20.37480084	21.92154499	19.71020108	18.24559088	15.81788109	16.53574792	16.55241749	18.11198417	21.22022496	19.88013024	Sh3bp5l	SH3 binding domain protein 5 like [Source:MGI Symbol;Acc:MGI:1933124]
ENSMUSG00000013653	0	0	0.047572624	0.043362287	0	0	0	0	0	0	0	0	1810065E05	RIKEN cDNA 1810065E05 gene [Source:MGI Symbol;Acc:MGI:1917114]
ENSMUSG00000013662	42.97182169	52.87746817	39.64315755	53.58497481	61.3626516	68.90025673	38.21249312	37.62975405	44.58500802	44.3169165	43.66262652	44.30983262	Atad1	ATPase family, AAA domain containing 1 [Source:MGI Symbol;Acc:MGI:1915229]
ENSMUSG00000013663	18.00537896	23.64024939	13.27629286	19.44721789	24.53670515	33.89778584	14.10768834	13.34641927	19.45089628	27.63528016	24.82861682	25.13520133	Pten	phosphatase and tensin homolog [Source:MGI Symbol;Acc:MGI:109583]
ENSMUSG00000013668	0	0.054271101	0	0	0	0	0	0	0	0	0	0	4933402N03	RIKEN cDNA 4933402N03 gene [Source:MGI Symbol;Acc:MGI:1914681]
ENSMUSG00000013698	111.7997828	121.5377893	107.0300023	111.9551484	110.4992164	104.0020217	37.11395264	34.81393936	36.56546039	55.99411309	57.69703845	53.22401438	Pea15a	phosphoprotein enriched in astrocytes 15A [Source:MGI Symbol;Acc:MGI:104799]
ENSMUSG00000013701	114.5633488	125.3189833	99.72939975	121.0738894	119.3625183	113.0248517	222.8595076	217.5102284	219.2691046	222.4818278	239.6745917	221.2238096	Timm23	translocase of inner mitochondrial membrane 23 [Source:MGI Symbol;Acc:MGI:1858317]
ENSMUSG00000013707	2.032539978	1.104044466	1.413441183	0	0.080585238	0.058420714	2.328172974	2.697357564	1.963193702	1.475029014	2.106875028	1.971219672	Tnfaip8l2	tumor necrosis factor, alpha-induced protein 8-like 2 [Source:MGI Symbol;Acc:MGI:1917019]
ENSMUSG00000013736	23.83001322	29.6780953	22.48500271	31.8230254	34.84807873	35.10689363	41.01585982	39.25870174	48.70560875	55.86100675	53.66313114	49.95013296	Trnt1	tRNA nucleotidyl transferase, CCA-adding, 1 [Source:MGI Symbol;Acc:MGI:1917297]
ENSMUSG00000013766	0.361583931	0.277588323	0.308489147	0.168712092	0	0.091803979	4.357987647	5.373006013	3.253292421	8.463040225	9.17995548	8.545188726	Ly6g6e	lymphocyte antigen 6 complex, locus G6E [Source:MGI Symbol;Acc:MGI:1917524]
ENSMUSG00000013787	218.3738276	209.9209575	227.4499016	231.8517585	218.8685045	205.6380109	174.4177536	169.7813268	182.1998949	91.42793593	94.04205024	92.93146055	Ehmt2	euchromatic histone lysine N-methyltransferase 2 [Source:MGI Symbol;Acc:MGI:2148922]
ENSMUSG00000013822	92.43919155	75.27258952	93.21582608	88.18395371	72.35494013	60.09877229	71.03990956	76.32882058	58.06168857	43.69028719	48.54129177	45.20300065	Elof1	ELF1 homolog, elongation factor 1 [Source:MGI Symbol;Acc:MGI:1913376]
ENSMUSG00000013833	33.52543449	30.5833474	31.72675014	33.72996163	30.80255887	30.26927036	32.29435271	33.37736391	32.81229059	20.09151612	21.73601834	23.53669571	Med16	mediator complex subunit 16 [Source:MGI Symbol;Acc:MGI:2158394]
ENSMUSG00000013846	37.04261585	36.0570867	36.53616135	45.52647908	47.5773132	52.45274482	1.377950162	1.502408231	1.386605059	10.62440744	10.73612518	10.76427908	St3gal1	ST3 beta-galactoside alpha-2,3-sialyltransferase 1 [Source:MGI Symbol;Acc:MGI:98304]
ENSMUSG00000013858	98.14140364	80.78031069	98.85389846	75.87010729	65.85594809	61.44374403	52.5462852	57.93347256	51.55498511	47.59607628	50.93845689	48.43862064	Tmem259	transmembrane protein 259 [Source:MGI Symbol;Acc:MGI:2177957]
ENSMUSG00000013878	13.90968961	16.45982537	12.60795356	13.47635598	14.89361208	16.78065847	8.278836087	7.840581458	9.043356795	12.70604792	11.87340484	11.67280404	Rnf170	ring finger protein 170 [Source:MGI Symbol;Acc:MGI:1924983]
ENSMUSG00000013921	75.78420579	80.3687955	78.32536213	77.70013303	76.49910204	77.0790056	13.11334286	10.9354269	12.41314016	45.70194122	49.65606611	46.99730766	Clip3	CAP-GLY domain containing linker protein 3 [Source:MGI Symbol;Acc:MGI:1923936]
ENSMUSG00000013928	0.104522642	0.080242131	0.071339676	0.032512938	0	0.159226011	0	0	0.032428463	0.218150417	0.116006074	0.15438423	Thap8	THAP domain containing 8 [Source:MGI Symbol;Acc:MGI:1922879]
ENSMUSG00000013936	0	0.397190506	0.264843188	0.080467891	0.362391329	0	0.923804106	2.050140972	1.605176414	1.311213283	1.507323417	1.451954287	Myl2	myosin, light polypeptide 2, regulatory, cardiac, slow [Source:MGI Symbol;Acc:MGI:97272]
ENSMUSG00000013973	0.752772705	0.832182208	0.554891885	1.23635621	1.202180614	2.110013925	1.586057656	0.865045195	1.008935938	1.939211742	2.305915006	2.054747139	Dedd	death effector domain-containing [Source:MGI Symbol;Acc:MGI:1333874]
ENSMUSG00000013974	0.86402586	0.563816443	0.678180559	0.645035789	0.42363886	0.4826161	0.308553045	0.114125429	0.026806661	0.077285073	0.143842873	0.10209613	Mcemp1	mast cell expressed membrane protein 1 [Source:MGI Symbol;Acc:MGI:1916439]
ENSMUSG00000013997	13.0179182	11.58100892	13.84655353	13.81598276	12.10786951	11.58164399	15.71841518	15.56517315	16.6602484	13.3860813	12.39046281	12.55336168	Nit1	nitrilase 1 [Source:MGI Symbol;Acc:MGI:1350916]
ENSMUSG00000014030	0.100690689	0	0.137448519	0.125283862	0.141055595	0.051129517	0.209753493	0.199496947	0.187437527	0.090065444	0.474950767	0.178469133	Pax5	paired box 5 [Source:MGI Symbol;Acc:MGI:97489]
ENSMUSG00000014039	3.818901751	4.909458511	2.696388688	3.88631595	5.240290918	6.156099515	7.230339787	4.989865875	6.955743991	7.141034312	7.782702858	7.950433457	Prdm15	PR domain containing 15 [Source:MGI Symbol;Acc:MGI:1930121]
ENSMUSG00000014074	5.033824338	7.153383712	5.00282244	7.408529047	9.142939305	10.69693455	11.15154289	10.59678043	13.40786778	15.13652442	13.9077351	14.94942748	Rnf168	ring finger protein 168 [Source:MGI Symbol;Acc:MGI:1917488]
ENSMUSG00000014075	12.12021166	10.35780835	13.04856806	12.31502952	11.82202998	11.63887739	16.77355614	16.99529359	14.06084012	9.443375123	9.163801071	10.0025779	Tctex1d2	Tctex1 domain containing 2 [Source:MGI Symbol;Acc:MGI:1913311]
ENSMUSG00000014077	61.57674654	78.17294126	51.69547854	70.04581137	77.61724058	80.46936741	39.55154108	33.10884606	43.65815374	55.0271799	53.80961656	55.80174889	Chp1	calcineurin-like EF hand protein 1 [Source:MGI Symbol;Acc:MGI:1927185]
ENSMUSG00000014104	0	0	0	0	0	0	0	0	0	0	0	0	Sval2	seminal vesicle antigen-like 2 [Source:MGI Symbol;Acc:MGI:1934254]
ENSMUSG00000014158	3.630938077	3.534632693	2.912545468	3.318469243	2.870993684	3.549687316	0.517990638	0.463525965	0.627128949	1.07143534	1.573512146	2.040168454	Trpv4	transient receptor potential cation channel, subfamily V, member 4 [Source:MGI Symbol;Acc:MGI:1926945]
ENSMUSG00000014164	0.491052643	0.586677574	0.609567513	0.57853085	0.960918607	0.841558305	0.482240679	0.267551683	0.394207055	1.17495361	1.149614406	1.038999258	Klhl3	kelch-like 3 [Source:MGI Symbol;Acc:MGI:2445185]
ENSMUSG00000014177	46.88688799	51.46175573	37.90193604	37.28211676	42.59126473	45.64554293	46.00189676	39.57768833	44.73140193	57.66641954	56.38820237	54.018008	Tvp23b	trans-golgi network vesicle protein 23B [Source:MGI Symbol;Acc:MGI:1914760]
ENSMUSG00000014195	74.88335632	66.55115183	81.54665541	75.96459024	70.07591135	62.24438499	100.5504959	106.023971	94.21442588	119.3178343	111.5392017	111.7513028	Dnajc7	DnaJ heat shock protein family (Hsp40) member C7 [Source:MGI Symbol;Acc:MGI:1928373]
ENSMUSG00000014198	0.142951787	0.329232662	0.329294229	0.366850711	0.26283906	0.517198	3.764910947	3.186317519	3.925082957	2.045871967	1.249425889	1.393561301	Zfp385c	zinc finger protein 385C [Source:MGI Symbol;Acc:MGI:3608347]

ENSMUSG00000014226	136.7962324	127.3473159	118.9860685	131.7943771	124.207441	116.3371225	352.8673643	368.5532904	344.0434075	281.8519877	272.0685778	279.7829274	Cacybp	calcyclin binding protein [Source:MGI Symbol;Acc:MGI:1270839]
ENSMUSG00000014232	5.266976276	3.95779326	8.225523896	4.70678734	4.361383491	3.263802885	9.005972013	8.799319183	8.765944991	3.154096829	3.97552456	4.074366372	Cluap1	clusterin associated protein 1 [Source:MGI Symbol;Acc:MGI:1924029]
ENSMUSG00000014243	26.68706759	20.64563151	27.80346939	25.06849641	23.22175915	18.67044618	39.7793161	42.04352738	31.78764622	34.54452719	31.21744065	33.39233867	Zswim7	zinc finger SWIM-type containing 7 [Source:MGI Symbol;Acc:MGI:1916997]
ENSMUSG00000014245	3.570049208	4.796277713	2.981702524	3.769868006	5.363141114	5.128394787	15.06958249	16.78353974	18.62547876	24.79028402	23.74757901	20.50961904	Pigl	phosphatidylinositol glycan anchor biosynthesis, class L [Source:MGI Symbol;Acc:MGI:2681271]
ENSMUSG00000014294	140.2939332	104.643033	259.3075637	129.1650336	126.2987635	72.93938509	98.18621792	93.00184953	51.11167939	54.63676345	46.39913414	47.93279453	Ndufa2	NADH:ubiquinone oxidoreductase subunit A2 [Source:MGI Symbol;Acc:MGI:1343103]
ENSMUSG00000014301	18.91769723	16.80127254	23.54516821	12.51911593	14.28998568	11.91354486	19.26262079	21.25167982	13.46480094	15.04127421	13.48278933	13.3684321	Pam16	presequence translocase-asssociated motor 16 homolog (S. cerevisiae) [Source:MGI Symbol;Acc:MGI:1913699]
ENSMUSG00000014303	25.70125605	30.40222664	25.6209666	29.63655459	28.34711156	30.56825368	21.65279212	21.7791067	25.47152991	15.73624649	15.88959535	15.29165836	Glis2	GLIS family zinc finger 2 [Source:MGI Symbol;Acc:MGI:1932535]
ENSMUSG00000014313	153.0495695	132.9527376	205.7595786	161.8609138	142.3640832	107.0075167	253.6334775	252.8427612	174.914462	122.8347829	117.2761668	108.1221801	Cox6c	cytochrome c oxidase subunit 6C [Source:MGI Symbol;Acc:MGI:104614]
ENSMUSG00000014329	8.231536802	13.66651042	6.978929855	8.783573276	13.75905341	16.32466843	0.939493714	0.949815085	1.153589691	6.118331611	5.246004316	5.284723204	Bicc1	BicC family RNA binding protein 1 [Source:MGI Symbol;Acc:MGI:1933388]
ENSMUSG00000014349	56.11896599	56.51644777	57.85095354	58.71830822	58.75837223	56.44642433	46.55530476	43.92691185	48.0249145	67.26005632	68.13002358	59.0167997	Ube2z	ubiquitin-conjugating enzyme E2Z [Source:MGI Symbol;Acc:MGI:1343160]
ENSMUSG00000014351	0	0.235950074	0.20977262	0.063735679	0.215277706	0.052022255	0	0.270640303	0.127140164	0.427644071	0.17055655	0.18158526	Gip	gastric inhibitory polypeptide [Source:MGI Symbol;Acc:MGI:107504]
ENSMUSG00000014353	14.05848802	18.60704266	12.39441885	17.46668632	21.88889222	27.21380745	10.25177196	9.799232239	12.35217826	14.4238166	14.47422341	14.58151102	Tmem87b	transmembrane protein 87B [Source:MGI Symbol;Acc:MGI:1919727]
ENSMUSG00000014355	59.83081604	69.18441919	55.52031711	69.01546235	79.04928522	96.42929306	58.12571878	51.10935715	69.38769033	83.35354778	87.86352128	84.02452566	Anapc1	anaphase promoting complex subunit 1 [Source:MGI Symbol;Acc:MGI:103097]
ENSMUSG00000014361	1.770970972	3.43175754	1.875628024	2.56444295	3.195251567	3.823480693	2.213515577	1.863292803	2.739666624	2.447148586	2.612812091	2.197269274	Mertk	MER proto-oncogene tyrosine kinase [Source:MGI Symbol;Acc:MGI:96965]
ENSMUSG00000014402	169.3762444	145.1985206	168.540842	159.9872664	148.4873457	130.5059141	116.8125154	129.4126877	113.8011304	102.8039918	94.33640432	88.76314789	Tsg101	tumor susceptibility gene 101 [Source:MGI Symbol;Acc:MGI:106581]
ENSMUSG00000014418	15.44659034	18.52083375	13.26224192	15.14456754	16.87904655	20.99477432	11.53257776	9.858916995	13.22553376	15.5253784	14.72050865	15.25316188	Hps5	HPS5, biogenesis of lysosomal organelles complex 2 subunit 2 [Source:MGI Symbol;Acc:MGI:2180307]
ENSMUSG00000014426	29.67157107	29.15698292	26.38506054	31.66217758	33.70830392	37.33712859	31.43492218	33.25711935	37.55923054	23.97914893	23.17747021	24.34228329	Map3k4	mitogen-activated protein kinase kinase kinase 4 [Source:MGI Symbol;Acc:MGI:1346875]
ENSMUSG00000014444	13.57166508	32.52488331	10.01878376	24.0943957	33.41880002	50.78182784	28.99338469	18.45731071	38.3564473	61.39483537	62.65378678	61.57400596	Piezo1	piezo-type mechanosensitive ion channel component 1 [Source:MGI Symbol;Acc:MGI:3603204]
ENSMUSG00000014453	0	0.100847047	0.029886194	0	0.030670501	0.044469499	0	0	0	0.052222478	0	0.051740712	Blk	B lymphoid kinase [Source:MGI Symbol;Acc:MGI:88169]
ENSMUSG00000014470	62.61509094	57.40803818	67.18988933	55.08180482	50.60910612	41.31349254	19.1480838	23.88101045	20.29266998	21.5231367	21.43110675	24.11287691	Rnf166	ring finger protein 166 [Source:MGI Symbol;Acc:MGI:1915968]
ENSMUSG00000014496	18.69396085	22.7829654	15.21329195	25.58690924	31.2966893	40.48402787	27.35441886	25.11970748	34.05367458	69.6746512	66.95273357	69.07719051	Ankrd28	ankyrin repeat domain 28 [Source:MGI Symbol;Acc:MGI:2145661]
ENSMUSG00000014498	8.695694968	17.49218344	6.723633439	13.1764239	18.50244346	28.2349392	6.0803613	4.120418291	8.591663653	16.42646388	18.10078388	16.23181302	Ankrd52	ankyrin repeat domain 52 [Source:MGI Symbol;Acc:MGI:2444029]
ENSMUSG00000014503	8.246338766	12.13146383	5.36372453	12.42551578	12.36025231	15.91881002	4.069441684	3.915956998	5.38683093	6.581019305	5.274133312	6.905827135	Pkd2l2	polycystic kidney disease 2-like 2 [Source:MGI Symbol;Acc:MGI:1858231]
ENSMUSG00000014504	70.14817991	62.04960021	81.36366807	70.11082854	61.0256208	51.72277202	78.25954142	87.89597876	69.53115719	58.8302414	61.44267969	60.55845906	Srp19	signal recognition particle 19 [Source:MGI Symbol;Acc:MGI:1913634]
ENSMUSG00000014529	0	0	0.162704525	0	0.0417436	0	0	0	0	0	0	0	Tmbim7	transmembrane BAX inhibitor motif containing 7 [Source:MGI Symbol;Acc:MGI:1922305]
ENSMUSG00000014542	0	0	0	0	0	0	0	0	0	0.099968744	0	0.074284879	Clec4f	C-type lectin domain family 4, member f [Source:MGI Symbol;Acc:MGI:1859834]
ENSMUSG00000014543	0.145982995	0.462294039	0.14945632	0.204343399	0.076689259	0.250183364	0.032582573	0	0	0	0.030379029	0	Klra17	killer cell lectin-like receptor, subfamily A, member 17 [Source:MGI Symbol;Acc:MGI:2180674]
ENSMUSG00000014547	5.211552402	5.654941635	4.810146999	4.929390555	5.419792226	6.564239379	4.091896856	3.409717202	4.521279907	7.44749668	8.050142895	7.582668895	Wdfy2	WD repeat and FYVE domain containing 2 [Source:MGI Symbol;Acc:MGI:2442811]
ENSMUSG00000014550	12.98411527	16.50771516	10.6672559	16.77513576	22.03875445	26.4599198	17.3776615	14.8816158	19.22529786	27.11978046	26.01917777	28.24960262	Rbsn	rabenosyn, RAB effector [Source:MGI Symbol;Acc:MGI:1925537]
ENSMUSG00000014551	12.20766995	10.84981146	15.42282904	12.89878731	10.10429218	8.514487836	26.67654196	29.53048418	20.85858902	22.91819523	21.04702992	20.99275372	Mrps25	mitochondrial ribosomal protein S25 [Source:MGI Symbol;Acc:MGI:1928140]
ENSMUSG00000014554	31.48812842	29.09036079	36.62223933	29.60625094	24.33332527	24.64848004	26.09377645	29.66130416	25.24757571	19.7583385	18.25505266	18.13509569	Dguok	deoxyguanosine kinase [Source:MGI Symbol;Acc:MGI:1351602]
ENSMUSG00000014592	4.697227536	4.649927184	5.098249476	3.910981699	3.6488246	4.205476246	17.58359805	18.9279756	20.27111637	10.9093114	10.95575015	12.46752971	Camta1	calmodulin binding transcription activator 1 [Source:MGI Symbol;Acc:MGI:2140230]
ENSMUSG00000014599	301.696979	329.6325745	275.963317	227.5161866	234.8605096	244.9730341	19.00463369	18.36618978	22.28979926	155.1128901	157.7995374	151.4004768	Csf1	colony stimulating factor 1 (macrophage) [Source:MGI Symbol;Acc:MGI:1339753]
ENSMUSG00000014601	50.16955798	44.91371233	48.58795438	45.87350782	48.24771164	46.54927896	40.43763905	40.82221522	42.1579926	37.52893383	39.80775441	38.56317724	Strip1	striatin interacting protein 1 [Source:MGI Symbol;Acc:MGI:2443884]
ENSMUSG00000014602	14.2501454	16.7779943	11.68558882	4.255360494	5.609044991	5.718245927	2.358262447	2.21737043	3.610246615	21.17770815	22.3580625	20.25541044	Kif1a	kinesin family member 1A [Source:MGI Symbol;Acc:MGI:108391]
ENSMUSG00000014603	2.576767524	1.625906993	1.662349064	0.922311222	0.630468755	0.914123627	0.031513416	0.069935763	0.164270515	0.663041258	0.763936639	0.469231805	Alx3	aristaless-like homeobox 3 [Source:MGI Symbol;Acc:MGI:1277097]
ENSMUSG00000014606	122.8799247	121.541005	132.0517393	130.1278978	116.55017	107.0331544	89.74149461	94.19347036	90.93400912	80.92106398	79.48870339	83.21972406	Slc25a11	solute carrier family 25 (mitochondrial carrier oxoglutarate carrier), member 11 [Source:MGI Symbol;Acc:MGI:1915113]
ENSMUSG00000014609	0.039403377	0.136125043	0.242045331	0.073541168	0.165598236	0	0.140713751	0.312277273	0.25672533	0.528680857	0.262394692	0.314282182	Chrne	cholinergic receptor, nicotinic, epsilon polypeptide [Source:MGI Symbol;Acc:MGI:87894]
ENSMUSG00000014633	32.15970025	25.20338684	41.00365157	32.37287313	27.2709925	20.6774963	49.27026266	52.80295321	35.75322177	23.61660682	24.37365229	22.0352771	Cmc2	COX assembly mitochondrial protein 2 [Source:MGI Symbol;Acc:MGI:1913781]
ENSMUSG00000014668	18.72339782	20.39476505	18.03562296	19.34048193	19.82039573	22.56689544	32.5695725	30.51702725	31.32470582	32.16810326	30.68057478	30.26421007	Chfr	checkpoint with forkhead and ring finger domains [Source:MGI Symbol;Acc:MGI:2444898]
ENSMUSG00000014686	1.053428139	1.213076654	0.78812877	1.512372047	1.787899596	1.543032987	3.508701361	3.371535976	4.223639332	0.61609739	1.180405501	1.436267597	Ceacam16	carcinoembryonic antigen-related cell adhesion molecule 16 [Source:MGI Symbol;Acc:MGI:2685615]
ENSMUSG00000014704	10.20563007	9.267853063	10.49808554	11.49181225	10.59530165	10.1368661	0.043284343	0.096058248	0.045125805	0.173466891	0.020178521	0.214833266	Hoxa2	homeobox A2 [Source:MGI Symbol;Acc:MGI:96174]
ENSMUSG00000014725	0.099602981	0	0.118968268	0.077456554	0	0.050577192	0	0.016445157	0	0.029697505	0	0	Adam28	a disintegrin and metallopeptidase domain 28 [Source:MGI Symbol;Acc:MGI:105988]
ENSMUSG00000014747	0.086576434	0.232626834	0.029545439	0.080791706	0.24256643	0.571512097	0.180351428	0.114355058	0.107442392	0.232321729	0.408374838	0.179027722	Ankrd53	ankyrin repeat domain 53 [Source:MGI Symbol;Acc:MGI:1922555]
ENSMUSG00000014748	78.77350766	79.06631692	80.50434004	74.60808968	75.17060225	71.3944486	37.41446725	36.49283185	37.15497456	48.78650425	48.52533716	46.79721702	Tex261	testis expressed gene 261 [Source:MGI Symbol;Acc:MGI:1096575]
ENSMUSG00000014763	8.21830282	10.29227881	6.780174843	12.60982744	13.37352183	14.26881799	2.017500375	1.717826231	2.360882458	5.593749453	6.219007318	5.795547572	Fam120b	family with sequence similarity 120, member B [Source:MGI Symbol;Acc:MGI:1914794]
ENSMUSG00000014767	17.55784717	20.26801757	15.15117154	22.31192257	23.32317815	26.61667481	26.40543896	24.42723777	29.00179611	28.59816587	25.68774916	28.31538748	Tbp	TATA box binding protein [Source:MGI Symbol;Acc:MGI:101838]
ENSMUSG00000014769	229.932705	181.9391804	247.135414	212.9456537	176.367287	149.6888545	148.0447669	168.3856422	142.7286053	126.1364721	124.3141467	128.8279761	Psmb1	proteasome (prosome, macropain) subunit, beta type 1 [Source:MGI Symbol;Acc:MGI:104884]
ENSMUSG00000014771	0	0	0	0	0	0	0	0	0	0	0	0	Pdcd2	programmed cell death 2 [Source:MGI Symbol;Acc:MGI:104643]
ENSMUSG00000014773	4.029423609	3.72864248	3.204469705	1.443645106	1.738781475	1.260539255	3.822213741	3.813516475	4.721513683	3.732946571	4.252505283	3.539090653	Dll1	delta like canonical Notch ligand 1 [Source:MGI Symbol;Acc:MGI:104659]
ENSMUSG00000014776	0.867072803	0.561644384	1.026406333	0.240212871	0.227749715	0.165108416	0.882961067	1.677654585	0.78181593	0.739221017	0.766860054	0.948519984	Nol3	nucleolar protein 3 (apoptosis repressor with CARD domain) [Source:MGI Symbol;Acc:MGI:1925938]
ENSMUSG00000014778	22.24799286	20.82122804	22.40636557	18.15782535	20.04394072	20.35439176	14.83217734	14.48381436	15.48005099	17.29122531	18.89622946	17.4046605	Fhod1	formin homology 2 domain containing 1 [Source:MGI Symbol;Acc:MGI:2679008]
ENSMUSG00000014782	2.013365072	2.434418047	1.987651663	2.429070685	2.644203112	3.023271957	0.9501	0.755070163	0.762968461	0.939044634	1.256942488	1.299985387	Plekhg4	pleckstrin homology domain containing, family G (with RhoGef domain) member 4 [Source:MGI Symbol;Acc:MGI:2142544]
ENSMUSG00000014786	10.90684635	10.01335233	10.38812431	13.30567601	11.58039632	11.63232103	11.59848913	11.59203995	13.00311049	8.056100027	8.820001456	8.573779427	Slc9a5	solute carrier family 9 (sodium/hydrogen exchanger), member 5 [Source:MGI Symbol;Acc:MGI:2685542]
ENSMUSG00000014791	1.84407805	2.12355067	2.686703171	2.029736242	3.15464639	1.962839697	3.208273527	2.41234193	2.970678822	3.129790672	2.223795016	2.43044887	Elmo3	engulfment and cell motility 3 [Source:MGI Symbol;Acc:MGI:2679007]
ENSMUSG00000014813	4.746769088	6.09186325	4.271924191	5.778580084	6.883039177	8.11346377	1.125810591	1.025782849	1.087825417	3.970762316	4.232825885	4.02498851	Stc1	stanniocalcin 1 [Source:MGI Symbol;Acc:MGI:109131]
ENSMUSG00000014837	1.403461806	2.300649037	0.862112341	2.326621317	2.376646053	3.18182165	3.243003797	3.418573357	4.979249844	4.046700992	4.288129374	4.243492847	4931428F04	RIKEN cDNA 4931428F04 gene [Source:MGI Symbol;Acc:MGI:1921606]
ENSMUSG00000014846	10.38893184	7.471388572	9.495073354	7.206082054	3.763874793	4.062644556	28.85556106	28.27264829	21.7473191	15.27413282	14.31349695	15.30960281	Tppp3	tubulin polymerization-promoting protein family member 3 [Source:MGI Symbol;Acc:MGI:1915221]
ENSMUSG00000014850	7.817645377	9.798881317	8.089477995	9.974804528	12.70589366	12.93081184	17.06495222	16.1118645	20.13186769	24.05263558	23.79650178	23.83074365	Msh3	mutS homolog 3 [Source:MGI Symbol;Acc:MGI:109519]
ENSMUSG00000014852	0	0	0.076987505	0	0.015801579	0.034366327	0	0.014898933	0	0.040357882	0	0.013328523	Adamts13	a disintegrin-like and metallopeptidase (reprolysin type) with thrombospondin type 1 motif, 13 [Source:MGI Symbol;Acc:MGI:268555
ENSMUSG00000014856	47.08073699	38.5589787	58.79092127	41.94471105	36.46916387	28.5909672	42.79424264	45.31967451	40.14840593	33.76639429	33.99718292	34.4944491	Tmem208	transmembrane protein 208 [Source:MGI Symbol;Acc:MGI:1913570]
ENSMUSG00000014859	118.5403499	103.5572139	106.6011607	118.3615799	115.8293675	107.3651079	332.6653944	352.9142592	356.0839728	195.9357397	185.5909563	190.6971995	E2f4	E2F transcription factor 4 [Source:MGI Symbol;Acc:MGI:103012]
ENSMUSG00000014867	335.5222072	330.3865515	328.3292797	254.3694003	239.792584	225.520209	102.4571159	98.15890697	103.3120286	141.4557725	145.6422367	144.269055	Surf4	surfeit gene 4 [Source:MGI Symbol;Acc:MGI:98445]
ENSMUSG00000014873	19.26476533	18.19915185	17.62680521	18.40816276	17.99848599	16.05751279	19.6451298	19.77016529	21.09827956	14.03314592	13.53935507	12.54909933	Surf2	surfeit gene 2 [Source:MGI Symbol;Acc:MGI:98444]
ENSMUSG00000014905	28.62514028	35.43731181	25.27892838	35.77669228	40.59846873	40.72552353	10.21154349	9.802146412	11.6317156	22.60017716	19.2181969	20.836334	Dnajb9	DnaJ heat shock protein family (Hsp40) member B9 [Source:MGI Symbol;Acc:MGI:1351618]
ENSMUSG00000014907	11.59105389	13.87275898	11.05605833	13.99661107	15.73340248	17.91329628	48.57025888	45.50243865	53.36164138	48.98468447	45.22554717	47.08658727	Naf1	nuclear assembly factor 1 ribonucleoprotein [Source:MGI Symbol;Acc:MGI:2682306]
ENSMUSG00000014932	11.61186215	14.02481713	10.5493122	14.59992804	16.55375992	19.65721194	14.05215643	14.35949465	15.23656845	37.13503896	33.41684318	38.27253451	Yes1	YES proto-oncogene 1, Src family tyrosine kinase [Source:MGI Symbol;Acc:MGI:99147]

ENSMUSG00000014956	57.61996658	74.35570019	50.64611767	74.87392696	85.79066924	93.74894275	97.01984075	91.12739365	116.2187429	111.8022533	109.2831177	111.7060312	Ppp1cb	protein phosphatase 1 catalytic subunit beta [Source:MGI Symbol;Acc:MGI:104871]
ENSMUSG00000014959	91.71793543	84.5062952	85.49911686	74.87884521	74.23767907	64.95466733	63.93975107	62.02228051	62.09189515	70.10059294	68.82373659	67.81995783	Gorasp2	golgi reassembly stacking protein 2 [Source:MGI Symbol;Acc:MGI:2135962]
ENSMUSG00000014980	11.59365609	10.74567809	11.38700005	10.20329338	9.507116235	7.394795456	26.21298367	24.27759124	25.79288181	20.48757027	20.98798289	20.21503381	Tsen15	tRNA splicing endonuclease subunit 15 [Source:MGI Symbol;Acc:MGI:1913887]
ENSMUSG00000015001	0	0.036730553	0	0.148826827	0	0	0	0	0.059376059	0.228245909	0.106202744	0.62188577	Oc90	otoconin 90 [Source:MGI Symbol;Acc:MGI:1313269]
ENSMUSG00000015002	55.12003757	61.12152275	48.84781161	61.55172362	72.41914794	91.10348015	46.38958327	42.84167478	52.29202506	78.29134425	73.37925691	78.24485886	Efr3a	EFR3 homolog A [Source:MGI Symbol;Acc:MGI:1923990]
ENSMUSG00000015013	41.3655511	34.52907067	53.20320072	39.2320816	32.37745505	27.66792322	38.27566982	40.10330357	30.03686364	25.00788153	19.89826416	22.19814017	Trappc2l	trafficking protein particle complex 2-like [Source:MGI Symbol;Acc:MGI:1916295]
ENSMUSG00000015016	6.488578347	6.591552876	8.870658126	6.933106196	6.220005531	4.987026375	8.400514786	9.399047967	8.028075932	9.205550043	8.925663274	8.65156562	Acsf3	acyl-CoA synthetase family member 3 [Source:MGI Symbol;Acc:MGI:2182591]
ENSMUSG00000015023	32.91167075	38.30501989	28.61429645	38.24140746	37.33722721	46.69810232	94.20785641	85.02968578	107.3043115	87.22793569	89.40894139	86.59347106	Ddx19a	DEAD (Asp-Glu-Ala-Asp) box polypeptide 19a [Source:MGI Symbol;Acc:MGI:99526]
ENSMUSG00000015027	21.4758941	21.40374204	19.99757525	17.84599015	16.00960056	13.86518282	9.381167572	8.737918869	9.16157061	10.93122277	8.959539261	10.08267533	Galns	galactosamine (N-acetyl)-6-sulfate sulfatase [Source:MGI Symbol;Acc:MGI:1355303]
ENSMUSG00000015053	29.20345022	31.35495393	26.35410533	32.57422415	32.75796146	32.77402064	0.970772486	0.769419634	0.702254333	1.230662153	0.923591425	1.1209776	Gata2	GATA binding protein 2 [Source:MGI Symbol;Acc:MGI:95662]
ENSMUSG00000015083	2.9112644	4.626409443	3.815079855	3.640437097	5.138699244	5.100152061	6.445794188	6.171807448	5.636145816	3.124868718	3.877334287	2.838037561	C8g	complement component 8, gamma polypeptide [Source:MGI Symbol;Acc:MGI:88237]
ENSMUSG00000015085	0.367503099	0.211599355	0.250831318	0.228631902	0.225237173	0.163286936	0.683538329	0.728128359	0.598599418	0.273935704	0.560833637	0.379972004	Entpd2	ectonucleoside triphosphate diphosphohydrolase 2 [Source:MGI Symbol;Acc:MGI:1096863]
ENSMUSG00000015087	77.41052039	79.42002876	85.54124362	81.14914634	79.86395075	72.84347618	75.92001945	77.69555731	76.76538989	47.91465367	47.35194498	47.85963342	Rabl6	RAB, member RAS oncogene family-like 6 [Source:MGI Symbol;Acc:MGI:2442633]
ENSMUSG00000015090	0.576274391	0.663609584	0.44248912	0.448141494	0	0.475515924	0.557358374	1.094190287	0.581070279	0.902061712	1.279174124	0.680944727	Ptgds	prostaglandin D2 synthase (brain) [Source:MGI Symbol;Acc:MGI:99261]
ENSMUSG00000015092	241.2957892	168.5113773	283.8851956	196.7578523	162.1872955	120.4243682	202.1922203	221.5803834	166.5140555	117.2462651	122.7933112	126.2635108	Edf1	endothelial differentiation-related factor 1 [Source:MGI Symbol;Acc:MGI:1891227]
ENSMUSG00000015093	0.357082887	0.479732516	0.243719227	0.166611941	0.687816738	0.407975361	0.531325782	0.23582765	0.720109225	0.745271825	0.247696234	0.052742607	Clic3	chloride intracellular channel 3 [Source:MGI Symbol;Acc:MGI:1916704]
ENSMUSG00000015094	226.3890386	172.2959877	243.7557844	174.5082894	145.4320506	120.587587	46.46368064	50.47441648	45.21951817	41.13528681	38.92479483	39.18206397	Npdc1	neural proliferation, differentiation and control 1 [Source:MGI Symbol;Acc:MGI:1099802]
ENSMUSG00000015095	69.51111245	70.51891321	76.93765717	75.73523854	71.62434969	63.3302504	38.0022348	38.13891636	39.10276985	35.47011294	33.93177677	36.94509133	Fbxw5	F-box and WD-40 domain protein 5 [Source:MGI Symbol;Acc:MGI:1354731]
ENSMUSG00000015112	3.594785059	3.167405746	2.425661878	2.414291389	2.804060252	3.630033931	74.59289386	74.83803894	85.19316346	66.50135962	69.25351556	68.0358597	Slc25a13	solute carrier family 25 (mitochondrial carrier, adenine nucleotide translocator), member 13 [Source:MGI Symbol;Acc:MGI:1354721]
ENSMUSG00000015120	256.1717153	267.0023001	225.7503381	304.776345	307.1969679	332.0316304	256.8562365	253.5945364	265.0771719	268.0339369	275.4155092	269.5476916	Ube2i	ubiquitin-conjugating enzyme E2I [Source:MGI Symbol;Acc:MGI:107365]
ENSMUSG00000015126	8.374672128	6.650941694	6.467402504	37.9302148	6.257862859	6.29635128	5.027447345	6.508308961	6.551664888	4.294378798	3.695868862	23.12927021	Tsr3	TSR3 20S rRNA accumulation [Source:MGI Symbol;Acc:MGI:1915577]
ENSMUSG00000015127	0.724449507	1.800203935	0.31228888	1.458832602	2.363571806	4.762905968	1.804153536	1.265366325	3.016551067	5.763815965	6.125526777	5.440316929	Unkl	unkempt family like zinc finger [Source:MGI Symbol;Acc:MGI:1921404]
ENSMUSG00000015133	28.46920601	31.642423	26.94220227	28.12975969	30.48599069	35.54640602	0.793317082	0.406282901	0.763446892	7.107587118	7.567346209	7.556917967	Lrrk1	leucine-rich repeat kinase 1 [Source:MGI Symbol;Acc:MGI:2142227]
ENSMUSG00000015134	0.583832942	0.705929327	0.687382466	0.544823308	0.644080519	0.289051742	0.364862929	0.462695769	0.271703877	1.38389567	1.482245186	1.681573138	Aldh1a3	aldehyde dehydrogenase family 1, subfamily A3 [Source:MGI Symbol;Acc:MGI:1861722]
ENSMUSG00000015143	222.2390541	274.4603874	224.4826857	227.2048159	244.7555958	255.5262626	156.33894	143.2439667	168.7338111	233.6626016	232.412763	237.8210555	Actn1	actinin, alpha 1 [Source:MGI Symbol;Acc:MGI:2137706]
ENSMUSG00000015149	55.05466059	60.53436469	51.55124766	59.5784778	55.55289792	53.04045778	26.30946063	23.75977576	25.81156717	34.37405073	32.78250836	29.46770782	Sirt2	sirtuin 2 [Source:MGI Symbol;Acc:MGI:1927664]
ENSMUSG00000015165	235.3173858	215.1630291	230.7346005	247.8878943	234.9036197	220.2587824	497.5105207	510.3810109	499.5004441	302.627566	300.2924792	304.5329123	Hnrnpl	heterogeneous nuclear ribonucleoprotein L [Source:MGI Symbol;Acc:MGI:104816]
ENSMUSG00000015176	72.0178444	64.97710783	72.73497133	72.4037928	66.96400572	64.01816639	216.6982288	232.4113833	232.450826	189.0133286	190.7533017	197.6561677	Nolc1	nucleolar and coiled-body phosphoprotein 1 [Source:MGI Symbol;Acc:MGI:1918019]
ENSMUSG00000015189	21.37998966	30.72688371	17.21542695	24.1797854	29.23377216	37.09446486	6.292604801	5.918409431	8.653365813	13.35970752	13.74563396	13.20671582	Casd1	CAS1 domain containing 1 [Source:MGI Symbol;Acc:MGI:2384865]
ENSMUSG00000015202	4.028992029	5.481652512	3.816589487	5.60623366	5.784736503	6.803803684	5.325084736	5.269639952	6.886158676	6.630549355	6.301669528	6.874342365	Cnksr3	Cnksr family member 3 [Source:MGI Symbol;Acc:MGI:2674130]
ENSMUSG00000015214	7.839718511	10.18221826	5.394690138	8.227385243	8.777003264	12.98039169	8.880853361	8.173773663	9.23475059	11.21990895	10.12029994	11.04806378	Mtmr1	myotubularin related protein 1 [Source:MGI Symbol;Acc:MGI:1858271]
ENSMUSG00000015217	13.72432809	16.32002634	11.49616343	16.65846887	19.9992189	17.52008984	18.022136	17.46233612	18.49109524	19.88783392	17.81523397	17.69139965	Hmgb3	high mobility group box 3 [Source:MGI Symbol;Acc:MGI:1098219]
ENSMUSG00000015222	9.344570589	14.53375458	8.916558801	16.5200206	20.54923561	24.09030577	1.850599031	1.805057265	2.240511973	3.080233517	3.68744056	3.496256996	Map2	microtubule-associated protein 2 [Source:MGI Symbol;Acc:MGI:97175]
ENSMUSG00000015224	2.314168781	2.318232694	2.561849268	2.774048753	2.767452807	3.711618428	0.352738057	0.149106594	0.227651495	1.043399176	0.720409389	1.017099775	Cyp2j9	cytochrome P450, family 2, subfamily j, polypeptide 9 [Source:MGI Symbol;Acc:MGI:1921769]
ENSMUSG00000015242	0	0	0	0	0	0	0	0	0	0	0.03525283	0	Nipsnap3a	nipsnap homolog 3A [Source:MGI Symbol;Acc:MGI:1920648]
ENSMUSG00000015243	27.89562048	37.76141742	22.85225445	30.52071743	41.04708883	58.66543756	2.227351568	1.954038679	3.134849331	15.3840286	15.66623254	15.15919422	Abca1	ATP-binding cassette, sub-family A (ABC1), member 1 [Source:MGI Symbol;Acc:MGI:99607]
ENSMUSG00000015247	53.15016798	50.49032921	43.91284434	60.65377665	64.61737485	59.67084348	34.32213228	35.47657578	36.162525	36.90460067	34.23005188	32.78303515	Nipsnap3b	nipsnap homolog 3B [Source:MGI Symbol;Acc:MGI:1913786]
ENSMUSG00000015289	20.22486286	16.92658967	25.53371009	16.36391734	17.72723442	13.24429601	58.37873799	55.21653045	46.08395518	31.9305132	30.97178445	28.50173441	Lage3	L antigen family, member 3 [Source:MGI Symbol;Acc:MGI:1913442]
ENSMUSG00000015290	29.75767414	26.81803681	30.23861504	26.9414658	25.32287248	22.04644172	32.47441272	27.06228647	27.662816	20.59965898	23.63175289	23.03043986	Ubl4a	ubiquitin-like 4A [Source:MGI Symbol;Acc:MGI:95049]
ENSMUSG00000015291	136.8648075	129.3194035	127.7283924	130.2804138	135.150149	124.3593371	88.70286634	81.76616097	90.31339046	83.67451354	81.19924809	82.50209837	Gdi1	guanosine diphosphate (GDP) dissociation inhibitor 1 [Source:MGI Symbol;Acc:MGI:99846]
ENSMUSG00000015305	10.67255339	15.83480983	8.502222817	6.329223511	8.796429867	10.61548467	6.915881747	5.629828942	9.854861971	33.93416217	34.62016384	34.68535434	Sash1	SAM and SH3 domain containing 1 [Source:MGI Symbol;Acc:MGI:1917347]
ENSMUSG00000015312	52.41389625	42.7877249	57.01169293	51.74139426	45.81514141	31.8208612	44.5165423	45.91576212	35.70377378	11.66693388	13.70175773	11.43134056	Gadd45b	growth arrest and DNA-damage-inducible 45 beta [Source:MGI Symbol;Acc:MGI:107776]
ENSMUSG00000015314	0	0.099747389	0.110851158	0	0	0.016494223	0.077332369	0.107261821	0	0	0	0.115147171	Slamf6	SLAM family member 6 [Source:MGI Symbol;Acc:MGI:1353620]
ENSMUSG00000015316	0	0.041790426	0	0	0	0	0	0	0	0.032461034	0.040277622	0	Slamf1	signaling lymphocytic activation molecule family member 1 [Source:MGI Symbol;Acc:MGI:1351314]
ENSMUSG00000015335	13.38664066	11.56155365	15.06059836	7.721822661	7.457440548	6.078600409	36.11652947	34.97505452	32.24470148	26.57972378	25.99019426	25.2263831	Zdhhc12	zinc finger, DHHC domain containing 12 [Source:MGI Symbol;Acc:MGI:1913470]
ENSMUSG00000015337	6.921798136	7.131774121	7.210810475	4.948358981	4.550602129	3.699795368	11.78103709	12.71156512	12.13153978	10.2103542	10.68104377	9.506321489	Endog	endonuclease G [Source:MGI Symbol;Acc:MGI:1261433]
ENSMUSG00000015340	18.15535792	19.73978011	17.52010758	0.64850839	0.51718803	1.257146148	2.455862138	1.749782443	2.69509768	23.88015141	22.51208728	21.24767502	Cybb	cytochrome b-245, beta polypeptide [Source:MGI Symbol;Acc:MGI:88574]
ENSMUSG00000015341	42.30857748	44.95805472	37.24881387	44.30490991	45.7707556	46.07484583	59.26204	61.16836575	61.4911683	50.14498236	49.98382494	46.47601125	Golga7	golgi autoantigen, golgin subfamily a, 7 [Source:MGI Symbol;Acc:MGI:1931029]
ENSMUSG00000015342	0.135629591	0.263561253	0.130178044	0.189850959	0.285001219	0.368029783	0.771929692	0.646609589	0.954678356	1.281415744	1.531178483	1.292131522	Xk	X-linked Kx blood group [Source:MGI Symbol;Acc:MGI:103569]
ENSMUSG00000015354	0.799027629	0.861390236	0.643987854	0.2062407	0.303651289	0.349624084	13.43229785	14.66895035	15.58609808	18.30877578	17.98626975	18.48639828	Pcolce2	procollagen C-endopeptidase enhancer 2 [Source:MGI Symbol;Acc:MGI:1923727]
ENSMUSG00000015355	1.714607674	2.115491654	2.507718228	0.533347903	0.428921426	0.715182614	0.036446731	0.161767923	0.037997297	2.629158993	2.344748015	2.315470493	Cd48	CD48 antigen [Source:MGI Symbol;Acc:MGI:88339]
ENSMUSG00000015357	17.09288447	20.49691298	14.90963341	19.07715552	19.92230978	20.55319938	25.55564445	22.89333134	25.30648187	27.25551432	26.1038245	25.10550875	Clpx	caseinolytic mitochondrial matrix peptidase chaperone subunit [Source:MGI Symbol;Acc:MGI:1346017]
ENSMUSG00000015363	29.74338932	30.49950519	30.90588055	28.34827639	25.53803234	25.12030392	19.37740427	19.97169757	19.29605137	19.07368245	18.77128282	18.26029949	Trabd	TraB domain containing [Source:MGI Symbol;Acc:MGI:1915226]
ENSMUSG00000015365	11.25140942	10.820619	9.954013901	12.19250559	12.57095352	13.49153194	9.699250223	9.207667717	11.80036582	4.667417997	4.258662125	4.696615452	Mov10l1	Mov10 like RISC complex RNA helicase 1 [Source:MGI Symbol;Acc:MGI:1891384]
ENSMUSG00000015377	4.483593652	3.565758044	4.8341284	5.020819111	4.975712016	4.546314813	4.115430475	4.330597357	4.942568684	4.762431528	5.248321057	4.17214457	Dennd6b	DENN/MADD domain containing 6B [Source:MGI Symbol;Acc:MGI:1916690]
ENSMUSG00000015396	0.148502115	0.277887706	0.228053064	0.0519674	0.019503157	0.028277844	0.132579307	0.091945314	0.01727746	0.547930496	0.602613522	0.690932699	Cd83	CD83 antigen [Source:MGI Symbol;Acc:MGI:1328316]
ENSMUSG00000015401	0.298808943	2.556125806	0.174810517	0.995869986	0.179398089	0	0.457319691	0.888038494	0.119193903	0.305460051	0.142130458	0.189151313	Cltrn	collectrin, amino acid transport regulator [Source:MGI Symbol;Acc:MGI:1926234]
ENSMUSG00000015405	0.222178078	0.307019374	0.045492857	0.400843669	0.031124488	0.033845804	0.039671106	0.220099041	0	0.026497739	0.012329389	0.013126645	Ace2	angiotensin I converting enzyme (peptidyl-dipeptidase A) 2 [Source:MGI Symbol;Acc:MGI:1917258]
ENSMUSG00000015437	0	0	0	0.13981016	0	0.068469402	0	0	0	0	0	0	Gzmb	granzyme B [Source:MGI Symbol;Acc:MGI:109267]
ENSMUSG00000015441	0.046820988	0	0	0	0	0	0	0	0.043579055	0	0	0	Gzmf	granzyme F [Source:MGI Symbol;Acc:MGI:109254]
ENSMUSG00000015443	0	0	0	0	0	0	0	0	0	0.039555978	0	0	Gzmn	granzyme N [Source:MGI Symbol;Acc:MGI:2675494]
ENSMUSG00000015451	2.013648445	2.542565457	2.296648512	3.626340363	2.542503947	3.49804818	0.820021515	0.769925	0.789145843	1.042777414	0.573422883	0.673117776	C4a	complement component 4A (Rodgers blood group) [Source:MGI Symbol;Acc:MGI:98320]
ENSMUSG00000015452	2.729202332	2.072921706	2.496888675	3.00744672	2.165850609	1.724948455	0.6221029	1.179257595	0.78368785	0.467465179	0.628366236	0.643267623	Ager	advanced glycosylation end product-specific receptor [Source:MGI Symbol;Acc:MGI:893592]
ENSMUSG00000015461	57.32378494	64.36921334	61.57095782	54.02588898	50.90149514	44.9658477	42.29771422	42.76710983	41.07775442	43.43049726	47.47887387	44.13559888	Atf6b	activating transcription factor 6 beta [Source:MGI Symbol;Acc:MGI:105121]
ENSMUSG00000015467	1.482237676	1.00404287	1.829931366	1.301835148	1.19089795	0.796936672	0.194604124	0.388685541	0.243459888	0.2339694	0.508040787	0.231810971	Egfl8	EGF-like domain 8 [Source:MGI Symbol;Acc:MGI:1932094]
ENSMUSG00000015468	2.976113896	2.081912421	2.02179041	1.37565244	1.607276994	2.563449578	39.90476438	34.77299276	41.93899513	20.2267076	19.19339998	19.17737547	Notch4	notch 4 [Source:MGI Symbol;Acc:MGI:107471]
ENSMUSG00000015474	26.7668854	20.46610428	28.17723361	24.34052795	19.05099703	13.18085613	6.809321959	6.379621019	4.721513683	3.282418537	3.473877555	4.272415743	Ppt2	palmitoyl-protein thioesterase 2 [Source:MGI Symbol;Acc:MGI:1860075]

ENSMUSG00000015476	1.441439008	1.130138631	0.722168036	0.429295914	0.418893898	0.420479238	0.301185599	0.303819299	0.371089786	0.329191444	0.587161893	0.407693208	Prrt1	proline-rich transmembrane protein 1 [Source:MGI Symbol;Acc:MGI:1932118]
ENSMUSG00000015478	27.6288185	26.91321059	27.45150749	29.33317223	31.74099825	24.01128249	45.41040118	45.6320917	31.99716422	25.28051686	25.63734245	27.17471208	Rnf5	ring finger protein 5 [Source:MGI Symbol;Acc:MGI:1860076]
ENSMUSG00000015484	0.033252309	0	0	0.041374011	0.011645626	0.109753289	0.019791269	0.010980383	0.010316628	0.049572342	0.064584783	0.186637091	Fam163a	family with sequence similarity 163, member A [Source:MGI Symbol;Acc:MGI:3618859]
ENSMUSG00000015488	30.21295743	30.34858243	32.02517468	31.50553105	27.11051276	27.39477786	10.82628393	8.293099272	11.38306548	16.67092859	15.82939573	14.96006669	Cacfd1	calcium channel flower domain containing 1 [Source:MGI Symbol;Acc:MGI:1924317]
ENSMUSG00000015501	4.501580777	5.374510092	3.999845671	5.177241631	6.437993551	9.592536131	0.228499832	0.178974868	0.294272982	3.023284972	3.465138583	3.062106937	Hivep2	human immunodeficiency virus type I enhancer binding protein 2 [Source:MGI Symbol;Acc:MGI:1338076]
ENSMUSG00000015519	0	0	0	0	0	0	0	0	0	0	0	0	2310057J18I	RIKEN cDNA 2310057J18 gene [Source:MGI Symbol;Acc:MGI:1914969]
ENSMUSG00000015522	26.37170658	34.28073616	20.88862568	33.02422185	36.95003399	45.11148807	48.10464441	41.8253623	56.11667283	45.76503385	47.31251797	45.04988484	Arnt	aryl hydrocarbon receptor nuclear translocator [Source:MGI Symbol;Acc:MGI:88071]
ENSMUSG00000015533	0.669130412	0.822777935	0.301886796	0.867840059	0.679197192	1.641292546	0.91126433	0.786454755	1.498940305	12.11244909	11.8477013	11.46801377	Itga2	integrin alpha 2 [Source:MGI Symbol;Acc:MGI:96600]
ENSMUSG00000015536	53.99351407	45.78985302	49.63367345	48.80501458	43.88151731	38.38102918	17.901267	20.32127027	18.11815588	17.54963105	18.15200275	19.38035958	Mocs2	molybdenum cofactor synthesis 2 [Source:MGI Symbol;Acc:MGI:1336894]
ENSMUSG00000015542	27.30654035	23.64321839	35.63361104	29.68075585	23.47354991	21.07464067	23.9169404	26.79485378	19.59135177	22.15833641	20.44833636	21.17476198	Nat9	N-acetyltransferase 9 (GCN5-related, putative) [Source:MGI Symbol;Acc:MGI:1913426]
ENSMUSG00000015568	34.25710866	35.74523381	35.87040531	17.12851263	19.5919779	20.63686917	7.656867856	9.349043984	9.44912031	11.7541581	11.15474604	10.68124397	Lpl	lipoprotein lipase [Source:MGI Symbol;Acc:MGI:96820]
ENSMUSG00000015575	180.8484526	165.5667632	194.464517	155.8811347	142.0107886	114.1112299	68.34897329	65.7131871	59.53049745	73.37556171	72.10282136	77.19327011	Atp6v0e	ATPase, H+ transporting, lysosomal V0 subunit E [Source:MGI Symbol;Acc:MGI:1328318]
ENSMUSG00000015579	0.029860158	0	0	0	0.125491515	0.090975769	0	0.118322964	0.02779261	0.427347302	0	0.344016489	Nkx2-5	NK2 homeobox 5 [Source:MGI Symbol;Acc:MGI:97350]
ENSMUSG00000015597	7.065378157	8.328945817	6.479280379	8.655652421	9.886021367	13.77274019	7.374057591	6.147852483	8.622774517	10.80258875	11.30716925	11.04914515	Zfp318	zinc finger protein 318 [Source:MGI Symbol;Acc:MGI:1889348]
ENSMUSG00000015599	0.150338593	0.091117167	0.056705728	0.059070949	0.033253636	0.054241667	0.063577361	0	0.051552788	0.268948643	0.38859795	0.378664372	Ttbk1	tau tubulin kinase 1 [Source:MGI Symbol;Acc:MGI:2147036]
ENSMUSG00000015605	42.05056765	39.36746554	33.33120067	36.84537881	33.77780604	38.54671745	53.25695678	48.64029023	56.50874599	45.52222538	45.9242892	45.71615278	Srf	serum response factor [Source:MGI Symbol;Acc:MGI:106658]
ENSMUSG00000015619	2.539386167	2.545166376	3.081250287	2.896316434	3.112703886	1.970023098	0.755702047	0.093171252	0.262617387	1.346027239	2.27035932	1.750362838	Gata3	GATA binding protein 3 [Source:MGI Symbol;Acc:MGI:95663]
ENSMUSG00000015627	0	0	0	0	0	0	0	0.013007582	0.02444257	0	0	0	Gata5	GATA binding protein 5 [Source:MGI Symbol;Acc:MGI:109497]
ENSMUSG00000015647	4.206585234	5.041507566	4.50917915	5.008417248	5.015449354	6.679351432	45.97303253	41.28031834	56.05408367	45.1051129	54.049125	53.17337307	Lama5	laminin, alpha 5 [Source:MGI Symbol;Acc:MGI:105382]
ENSMUSG00000015652	39.97399641	33.33887162	43.10050405	36.80735468	31.90628229	27.00822071	41.06279154	38.6351562	37.49457601	28.78678143	27.85756982	29.14891788	Steap1	six transmembrane epithelial antigen of the prostate 1 [Source:MGI Symbol;Acc:MGI:1917608]
ENSMUSG00000015653	27.88150249	36.88910012	25.36798743	33.96676608	37.59459129	49.69756264	10.99916001	10.14336457	13.11260471	25.120543	24.73380209	25.58242161	Steap2	six transmembrane epithelial antigen of prostate 2 [Source:MGI Symbol;Acc:MGI:1921301]
ENSMUSG00000015656	1441.27943	1216.702456	1536.707235	1143.557991	1048.134646	884.5788107	2771.834727	2848.203695	2539.669731	2428.183895	2336.017386	2378.253158	Hspa8	heat shock protein 8 [Source:MGI Symbol;Acc:MGI:105384]
ENSMUSG00000015659	3.258519873	5.536258729	3.035257462	5.209234555	7.240065881	10.31435659	6.366707658	5.106634765	9.769904748	9.795199387	10.27150619	9.941539397	Serac1	serine active site containing 1 [Source:MGI Symbol;Acc:MGI:2447813]
ENSMUSG00000015665	0	0	0	0	0	0	0	0	0	0	0	0	Awat1	acyl-CoA wax alcohol acyltransferase 1 [Source:MGI Symbol;Acc:MGI:3588200]
ENSMUSG00000015668	30.09227043	25.17306582	31.02071638	29.27588778	25.29339998	20.44424438	15.84149529	16.78992393	14.03651934	11.2926911	10.6529292	10.48348344	Pdzd11	PDZ domain containing 11 [Source:MGI Symbol;Acc:MGI:1919871]
ENSMUSG00000015671	107.8335884	89.26814705	112.341503	90.06281942	88.995925	71.45105437	110.9201884	118.6206165	97.68085737	95.15481742	98.58040587	97.80052666	Psma2	proteasome subunit alpha 2 [Source:MGI Symbol;Acc:MGI:104885]
ENSMUSG00000015672	16.59384636	15.22042426	19.19468207	18.13850961	16.17416862	13.31603213	18.82070032	19.95954768	16.41663723	16.23369568	16.4938838	15.0602334	Mrpl32	mitochondrial ribosomal protein L32 [Source:MGI Symbol;Acc:MGI:2137226]
ENSMUSG00000015697	19.64567612	20.75420615	17.30498753	22.31104266	25.14374997	27.37697076	29.91563138	24.86224742	32.75201528	36.60701396	35.88346728	35.71225018	Setdb1	SET domain, bifurcated 1 [Source:MGI Symbol;Acc:MGI:1934229]
ENSMUSG00000015702	3.246860506	3.878699742	3.727976039	3.964377219	5.037316149	4.599457057	0.595999146	1.08217949	0.649598367	0.162854583	0.530433276	0.645408824	Anxa9	annexin A9 [Source:MGI Symbol;Acc:MGI:1923711]
ENSMUSG00000015709	5.252411361	6.708729727	4.461581843	4.109524384	4.168997374	5.118756954	6.265962338	6.20301515	7.108937877	7.570394238	8.95421887	8.8624448	Arnt2	aryl hydrocarbon receptor nuclear translocator 2 [Source:MGI Symbol;Acc:MGI:107188]
ENSMUSG00000015711	23.04233789	21.69848487	19.49373974	23.53400055	25.7112204	24.45072567	43.4596254	42.6258477	44.77646578	32.64692089	31.8819607	32.71596677	Prune1	prune exopolyphosphatase [Source:MGI Symbol;Acc:MGI:1925152]
ENSMUSG00000015714	184.1054971	197.9442228	178.7365889	217.3060143	215.9438321	212.9303943	54.27672517	53.19496158	60.40199902	74.25495971	77.21178894	76.93000956	Cers2	ceramide synthase 2 [Source:MGI Symbol;Acc:MGI:1924143]
ENSMUSG00000015721	0.040101107	0.015392829	0.013685073	0.024947796	0.028088424	0	0.035801355	0	0.024882977	0	0	0	Nlrp5	NLR family, pyrin domain containing 5 [Source:MGI Symbol;Acc:MGI:1345193]
ENSMUSG00000015733	160.0352172	179.6031586	138.7498275	181.1241763	190.4943213	198.0107248	110.0100735	99.05828047	114.1833218	125.2076881	129.3994638	126.4376951	Capza2	capping protein (actin filament) muscle Z-line, alpha 2 [Source:MGI Symbol;Acc:MGI:106222]
ENSMUSG00000015745	101.7491832	79.03828655	114.2750761	74.10990729	65.6141872	48.05239601	10.15191704	13.06712033	13.42027593	13.87356927	13.2514867	11.77041033	Plekho1	pleckstrin homology domain containing, family O member 1 [Source:MGI Symbol;Acc:MGI:1914470]
ENSMUSG00000015747	15.25459837	14.78005112	17.79861698	14.16170948	14.45229248	12.14001009	14.22946306	15.60689656	12.41132018	11.97455677	12.98252271	12.6099642	Vps45	vacuolar protein sorting 45 [Source:MGI Symbol;Acc:MGI:891965]
ENSMUSG00000015748	37.37382002	33.71130632	34.82570088	36.33326331	35.95623951	33.94051555	74.30552882	78.86219462	76.72660856	54.66292281	63.00436001	59.87466349	Prpf3	pre-mRNA processing factor 3 [Source:MGI Symbol;Acc:MGI:1918017]
ENSMUSG00000015749	75.91378374	75.61526578	68.97511135	88.31108153	99.9144968	97.75278612	253.6524235	255.1557153	249.0070074	185.5317353	193.334102	187.5419779	Anp32e	acidic (leucine-rich) nuclear phosphoprotein 32 family, member E [Source:MGI Symbol;Acc:MGI:1913721]
ENSMUSG00000015750	49.56270084	51.12378672	50.14887287	44.80237268	45.52480092	41.82791002	32.02272165	30.62082974	27.41113171	23.2751676	25.34376649	23.38900293	Aph1a	aph1 homolog A, gamma secretase subunit [Source:MGI Symbol;Acc:MGI:2385110]
ENSMUSG00000015755	38.91515391	43.17602938	32.08009211	40.25396201	47.13159516	53.90161752	41.01045232	37.24961466	46.40329618	55.42035999	55.39246508	55.71068508	Tab2	TGF-beta activated kinase 1/MAP3K7 binding protein 2 [Source:MGI Symbol;Acc:MGI:1915902]
ENSMUSG00000015757	17.0964254	18.87834094	15.97738713	19.4310249	25.25491906	26.31976026	24.65317453	24.90901837	31.15154191	38.41179029	37.71316725	36.45362289	Ppil4	peptidylprolyl isomerase (cyclophilin)-like 4 [Source:MGI Symbol;Acc:MGI:1914668]
ENSMUSG00000015759	119.3666738	94.98584752	121.2712525	105.8485984	90.76573568	80.03694229	61.14487873	69.74016588	61.7746565	54.23979817	59.18773245	56.9785101	Cnih1	cornichon family AMPA receptor auxiliary protein 1 [Source:MGI Symbol;Acc:MGI:1277202]
ENSMUSG00000015766	100.5707298	130.9339532	83.63080038	186.2871713	222.5514159	248.7429297	16.24172069	15.43806608	20.43959288	29.77333357	28.43080478	31.33615898	Eps8	epidermal growth factor receptor pathway substrate 8 [Source:MGI Symbol;Acc:MGI:104684]
ENSMUSG00000015776	36.53409485	40.80038787	35.16992003	38.14112405	42.9426334	41.48320095	32.23624118	31.54710174	31.78726362	28.12582158	28.63680254	26.77747678	Med22	mediator complex subunit 22 [Source:MGI Symbol;Acc:MGI:98446]
ENSMUSG00000015787	0	0	0	0	0	0	0	0	0.066934515	0.085767056	0.099768455	0.10621979	Abo	ABO blood group (transferase A, alpha 1-3-N-acetylgalactosaminyltransferase, transferase B, alpha 1-3-galactosyltransferase) [Sou
ENSMUSG00000015790	56.72455822	43.56940167	48.78465373	42.70966741	39.93201594	35.16469853	39.68855613	40.87332611	35.95925143	32.77092097	29.97406953	30.6985055	Surf1	surfeit gene 1 [Source:MGI Symbol;Acc:MGI:98443]
ENSMUSG00000015804	75.12106497	61.93689453	85.41987573	70.74130721	63.99293816	50.9086761	47.69933057	54.28596532	40.32650022	30.06539201	28.94618676	31.47813185	Med28	mediator complex subunit 28 [Source:MGI Symbol;Acc:MGI:1914249]
ENSMUSG00000015806	23.12563465	20.71249995	22.24906159	23.16546798	24.11415361	22.12578114	56.72800819	60.87557804	51.35857791	30.19045945	29.0377705	27.9821416	Qdpr	quinoid dihydropteridine reductase [Source:MGI Symbol;Acc:MGI:97836]
ENSMUSG00000015812	6.911815918	4.372016084	5.780612017	5.269008404	5.727750741	7.859833003	1.91205156	0.096438569	1.540351982	1.393229552	0.48620193	1.03528248	Gnrh1	gonadotropin releasing hormone 1 [Source:MGI Symbol;Acc:MGI:95789]
ENSMUSG00000015829	0.057632585	0.066366884	0.082605345	0.043025425	0.036331357	0.035118158	0.144068566	0.182698517	0.150197729	0.051550986	0.086351963	0.071505581	Tnr	tenascin R [Source:MGI Symbol;Acc:MGI:99516]
ENSMUSG00000015837	599.159369	587.2550203	590.7399542	622.9703652	592.5175341	531.0625062	181.1345247	182.8516315	176.77997	297.5100903	310.1002803	305.593728	Sqstm1	sequestosome 1 [Source:MGI Symbol;Acc:MGI:107931]
ENSMUSG00000015839	89.94073004	100.6403471	79.7702909	107.7038722	116.5364861	117.4249204	42.62657953	42.64758449	46.84995084	45.08253889	43.46974178	45.01666209	Nfe2l2	nuclear factor, erythroid derived 2, like 2 [Source:MGI Symbol;Acc:MGI:108420]
ENSMUSG00000015843	0.696629592	0.855685105	1.236220729	0.151686101	0.292768386	0.106122031	0.621935036	0.345055432	0.302583983	0.87236621	0.869810791	0.761423353	Rxrg	retinoid X receptor gamma [Source:MGI Symbol;Acc:MGI:98216]
ENSMUSG00000015846	31.78923944	32.66686138	34.66859016	26.97539668	28.84617518	28.11458156	12.25572694	12.36663148	13.37545157	15.39518655	18.26229121	17.36655177	Rxra	retinoid X receptor alpha [Source:MGI Symbol;Acc:MGI:98214]
ENSMUSG00000015850	12.69185612	13.18943941	12.9206932	12.48824269	13.53497522	11.4446082	3.932898729	3.963000782	3.21368399	3.429198996	3.587633902	3.566386769	Adamtsl4	ADAMTS-like 4 [Source:MGI Symbol;Acc:MGI:2389008]
ENSMUSG00000015852	0.900056088	0.534947537	0.921470821	0.35222349	0.152524691	0.132688343	0.077762862	0.201336663	0.135118595	4.726592361	4.205220198	4.142643492	Fcrls	Fc receptor-like S, scavenger receptor [Source:MGI Symbol;Acc:MGI:1933397]
ENSMUSG00000015854	0	0	0.021636665	0	0	0.01609726	0	0	0	0.018903716	0.017591786	0.018729325	Cd5l	CD5 antigen-like [Source:MGI Symbol;Acc:MGI:1334419]
ENSMUSG00000015869	98.54911728	83.71470583	110.3082555	87.26905062	78.06638037	68.02591582	111.7593436	114.2132089	111.8711682	123.1847714	116.6936911	117.5123887	Prpsap1	phosphoribosyl pyrophosphate synthetase-associated protein 1 [Source:MGI Symbol;Acc:MGI:1915013]
ENSMUSG00000015879	0.025440572	0.117184507	0.069455657	0.158271493	0.098007785	0.09042891	0.537535404	0.420041858	0.378864658	0.637167752	0.46588762	0.909357798	Fam184b	family with sequence similarity 184, member B [Source:MGI Symbol;Acc:MGI:2442958]
ENSMUSG00000015880	13.3487718	18.49930348	10.50420817	23.88668112	27.52783741	33.12555716	39.03291484	34.85331276	48.04576429	52.19057156	46.02704914	53.28223935	Ncapg	non-SMC condensin I complex, subunit G [Source:MGI Symbol;Acc:MGI:1930197]
ENSMUSG00000015882	2.879315299	4.164493551	2.417213943	4.22385353	5.00970123	6.842541782	3.084704556	2.498677903	3.891285333	3.482048349	3.365022295	3.980683305	Lcorl	ligand dependent nuclear receptor corepressor-like [Source:MGI Symbol;Acc:MGI:2651932]
ENSMUSG00000015889	94.94902743	93.58870858	92.15483864	90.04645949	90.73333245	79.6965458	125.8406034	124.0330052	127.0210392	101.7807867	103.6870318	105.1827056	Lta4h	leukotriene A4 hydrolase [Source:MGI Symbol;Acc:MGI:96836]
ENSMUSG00000015890	0.053319068	0.081866197	0.072783561	0.016585493	0	0.01353739	0.507755196	0.651448313	0.314305609	0.17487304	0.355061963	0.34651958	Amdhd1	amidohydrolase domain containing 1 [Source:MGI Symbol;Acc:MGI:1919011]
ENSMUSG00000015932	194.1573263	201.2576349	169.4317315	187.1727789	191.526188	189.2695404	165.4190655	163.1202639	183.4171502	242.9917919	234.2247477	234.7238921	Dstn	destrin [Source:MGI Symbol;Acc:MGI:1929270]
ENSMUSG00000015937	63.93980351	67.49978444	66.54509428	79.97760384	74.31716037	69.24435939	74.36149469	71.29559079	72.32389496	47.82715165	50.11424751	49.96635563	Macroh2a1	macroH2A.1 histone [Source:MGI Symbol;Acc:MGI:1349392]
ENSMUSG00000015942	10.17878207	9.997656853	8.860604404	10.97172829	12.00979994	10.71737424	1.482167045	1.536795268	1.139918924	2.653503058	2.763857564	2.96669657	Gtf2ird2	GTF2I repeat domain containing 2 [Source:MGI Symbol;Acc:MGI:2149780]
ENSMUSG00000015943	22.87725812	17.60392729	27.58053925	20.75012022	20.72281922	15.10454864	38.98749786	42.73175043	30.89505975	25.19646991	27.76364012	29.22733192	Bola1	bolA-like 1 (E. coli) [Source:MGI Symbol;Acc:MGI:1916418]

ENSMUSG00000015944	33.3181873	35.30856659	36.03157541	33.22080552	34.37535194	33.43704582	5.882148914	6.036307697	6.160333387	9.853563766	9.382097483	9.044428039	Castor2	cytosolic arginine sensor for mTORC1 subunit 2 [Source:MGI Symbol;Acc:MGI:1933384]
ENSMUSG00000015947	5.881009622	6.150974353	6.518075891	0	0.141718866	0.061643926	0	0.320696033	0.200873487	2.943907146	3.61537113	2.821117714	Fcgr1	Fc receptor, IgG, high affinity I [Source:MGI Symbol;Acc:MGI:95498]
ENSMUSG00000015950	3.225895858	3.257580612	3.023193641	0.115782808	0.234645251	0.396917205	0.708924643	0.319570946	0.230963965	3.773330036	5.081286833	4.398258903	Ncf1	neutrophil cytosolic factor 1 [Source:MGI Symbol;Acc:MGI:97283]
ENSMUSG00000015957	2.381747197	1.53712001	1.795316766	1.782970731	1.209954992	1.893875888	1.144968278	1.218622167	0.828267124	2.341117176	2.200428481	2.365910145	Wnt11	wingless-type MMTV integration site family, member 11 [Source:MGI Symbol;Acc:MGI:101948]
ENSMUSG00000015961	28.88859224	31.20873189	23.36317423	32.45632553	33.19953639	38.27873976	83.33445979	79.27901843	92.07100695	102.8160964	99.1298644	102.2330818	Adss	adenylosuccinate synthetase, non muscle [Source:MGI Symbol;Acc:MGI:87948]
ENSMUSG00000015962	0	0.258070394	0	0	0.117729996	0	0.100038682	0	0.052147333	0.200458158	0.13990967	0	1700016C15	RIKEN cDNA 1700016C15 gene [Source:MGI Symbol;Acc:MGI:1916678]
ENSMUSG00000015966	0	0	0.096183952	0.131507023	0.049354059	0.071558997	0.06290642	0.069802152	0.043721781	0.126052292	0.117304177	0.14570433	Il17rb	interleukin 17 receptor B [Source:MGI Symbol;Acc:MGI:1355292]
ENSMUSG00000015968	0.143907986	0.272250086	0.11576081	0.095923263	0.118798734	0.109612109	0.036707935	0.091646591	0.143511054	0.367777987	0.359366644	0.346165881	Cacna1d	calcium channel, voltage-dependent, L type, alpha 1D subunit [Source:MGI Symbol;Acc:MGI:88293]
ENSMUSG00000015970	0.02723322	0.250883623	0	0.076240781	0.01430643	0.041486102	0.024313199	0.067445962	0.050695129	0.133977098	0.045337817	0.096538999	Chdh	choline dehydrogenase [Source:MGI Symbol;Acc:MGI:1860776]
ENSMUSG00000015971	24.51025931	21.91613191	19.6891357	22.63292849	24.8226045	24.23278051	32.81056502	31.65845453	32.98477864	29.27433516	30.33033819	27.8916405	Actr8	ARP8 actin-related protein 8 [Source:MGI Symbol;Acc:MGI:1860775]
ENSMUSG00000015980	7.548014066	8.747288792	9.745637474	9.331757977	7.324252693	6.444947075	2.832827228	4.762664548	4.564260844	4.128318181	4.602168358	5.240611482	Lrrc27	leucine rich repeat containing 27 [Source:MGI Symbol;Acc:MGI:1923862]
ENSMUSG00000015981	0.441857332	0.169607144	0.40928741	0.431968955	0.176854057	0.16026416	0.563543092	0.729537736	0.607102052	0.489333558	0.455373478	0.242409663	Stk32c	serine/threonine kinase 32C [Source:MGI Symbol;Acc:MGI:2385336]
ENSMUSG00000015994	80.92775194	76.83221959	78.39383408	76.43216405	68.60373002	63.2845564	133.8886683	133.6846595	131.9310744	139.314058	143.5724706	142.358034	Fnta	farnesyltransferase, CAAX box, alpha [Source:MGI Symbol;Acc:MGI:104683]
ENSMUSG00000016018	35.09832284	40.34987859	30.16000474	39.24899676	47.09071249	49.0603599	73.76176417	69.80164412	77.06386221	86.06592344	89.13879044	81.0291688	Mtrex	Mtr4 exosome RNA helicase [Source:MGI Symbol;Acc:MGI:1919448]
ENSMUSG00000016024	5.900888423	5.722253773	6.590494124	2.713784919	2.402804322	2.688157861	0.050413194	0.279697163	0.157673825	0.580855136	0.470037736	0.450388638	Lbp	lipopolysaccharide binding protein [Source:MGI Symbol;Acc:MGI:1098776]
ENSMUSG00000016028	0.209534315	0.340644049	0.189282083	0.241541975	0.258999246	0.337973286	6.173226335	4.774192624	7.743677249	4.145366759	3.642220222	4.292819119	Celsr1	cadherin, EGF LAG seven-pass G-type receptor 1 [Source:MGI Symbol;Acc:MGI:1100883]
ENSMUSG00000016087	2.99435616	3.400920043	3.884492987	3.598119082	3.555468316	3.702308337	0.018310226	0.040634745	0.076356819	1.302500292	0.870667612	1.381363564	Fli1	Friend leukemia integration 1 [Source:MGI Symbol;Acc:MGI:95554]
ENSMUSG00000016128	14.2106373	19.15508669	12.27057046	23.45884189	23.58795514	31.04553926	0.668768581	0.218258309	0.697220252	3.764056107	3.044342719	3.397401647	Stard13	StAR-related lipid transfer (START) domain containing 13 [Source:MGI Symbol;Acc:MGI:2385331]
ENSMUSG00000016150	0	0.006879011	0	0	0	0	0.021332697	0.023671164	0	0.037403271	0.144202331	0.17470302	Tenm1	teneurin transmembrane protein 1 [Source:MGI Symbol;Acc:MGI:1345185]
ENSMUSG00000016179	0.691220688	0.53065077	0.530750002	0.564406315	0.332859104	0.372930623	0	0	0	0.103046764	0.095895249	0.229716293	Camk1g	calcium/calmodulin-dependent protein kinase I gamma [Source:MGI Symbol;Acc:MGI:2388073]
ENSMUSG00000016181	11.71593747	11.19250353	10.75757026	12.9432456	11.1226815	11.54493889	28.14275024	28.23507006	29.41338784	25.04772415	26.93918839	26.72562551	Utp25	UTP25 small subunit processome component [Source:MGI Symbol;Acc:MGI:2138080]
ENSMUSG00000016194	3.099822451	2.932175453	2.266839633	1.687410304	1.667202135	0.983782781	0.362404284	0.255901316	0.034347471	0.231059832	0.829378763	0.555968593	Hsd11b1	hydroxysteroid 11-beta dehydrogenase 1 [Source:MGI Symbol;Acc:MGI:103562]
ENSMUSG00000016200	0.420988059	0.464589833	0.215502243	0.270090658	0.433100482	0.58787481	1.44858296	1.607375015	1.86123248	3.553821599	3.16117144	2.969174397	Syt14	synaptotagmin XIV [Source:MGI Symbol;Acc:MGI:2444490]
ENSMUSG00000016206	20.13781106	18.40016783	17.30842932	19.32281409	19.71183283	19.87409318	1.816557772	2.163899084	1.893840267	4.710627317	4.358799172	4.057256201	H2-M3	histocompatibility 2, M region locus 3 [Source:MGI Symbol;Acc:MGI:95915]
ENSMUSG00000016239	3.410794767	3.348620721	2.686112817	4.61112093	5.053775425	6.095168472	0.566072545	0.259913705	0.427353751	2.170815176	2.65714625	2.11203588	Lonrf3	LON peptidase N-terminal domain and ring finger 3 [Source:MGI Symbol;Acc:MGI:1921615]
ENSMUSG00000016252	96.76287322	76.80174922	171.4891168	107.7451655	95.26038509	56.02796861	50.89968164	45.82278624	21.35954748	23.50515089	18.37746825	18.24931867	Atp5e	ATP synthase, H+ transporting, mitochondrial F1 complex, epsilon subunit [Source:MGI Symbol;Acc:MGI:1855697]
ENSMUSG00000016253	62.37686826	56.07575216	62.64141282	61.37553407	59.05146952	51.74555943	110.7649777	105.8136605	109.8327822	61.83259698	68.32850238	66.89253666	Nelfcd	negative elongation factor complex member C/D, Th1l [Source:MGI Symbol;Acc:MGI:1926424]
ENSMUSG00000016255	0.061206953	0	0	0	0	0.046620229	0	0	0	0.246366783	0.152845841	0.081364661	Tubb1	tubulin, beta 1 class VI [Source:MGI Symbol;Acc:MGI:107814]
ENSMUSG00000016256	332.6720214	287.1877547	373.1754463	226.1802773	205.0531358	165.917058	83.62761778	85.04463021	65.89627915	101.9490407	96.73787603	93.49309665	Ctsz	cathepsin Z [Source:MGI Symbol;Acc:MGI:1891190]
ENSMUSG00000016257	116.0157841	112.0190159	117.9207252	127.0877065	123.1472084	112.2317017	130.5891919	134.4399136	128.8127356	112.7948307	109.139633	105.4604881	Prelid3b	PRELI domain containing 3B [Source:MGI Symbol;Acc:MGI:1913640]
ENSMUSG00000016262	22.3119241	29.3143561	19.36310621	22.54722425	26.23588598	30.04431983	1.424301414	0.984906744	1.506416231	4.136267208	3.579762944	3.503883236	Sertad4	SERTA domain containing 4 [Source:MGI Symbol;Acc:MGI:2443496]
ENSMUSG00000016283	0.634549329	0.661124155	0.804324793	0.169186002	0.158737073	0.253170103	0.24279051	0.179603291	0.365617254	0.351364862	0.377284503	0.133893626	H2-M2	histocompatibility 2, M region locus 2 [Source:MGI Symbol;Acc:MGI:95914]
ENSMUSG00000016308	63.87953063	67.756366	63.76984184	74.51416047	69.12245962	63.93156677	65.65103298	70.16708592	57.92559154	62.26413302	60.15115216	63.28839668	Ube2a	ubiquitin-conjugating enzyme E2A [Source:MGI Symbol;Acc:MGI:102959]
ENSMUSG00000016319	281.9271362	280.7640389	280.5130762	321.1473548	313.6043967	289.678994	708.9426676	687.7368734	654.831718	526.2593077	522.7900051	503.9427655	Slc25a5	solute carrier family 25 (mitochondrial carrier, adenine nucleotide translocator), member 5 [Source:MGI Symbol;Acc:MGI:1353496]
ENSMUSG00000016327	0	0.011811565	0	0.02871524	0	0.007812639	0	0	0	0	0	0	Atp1b4	ATPase, (Na+)/K+ transporting, beta 4 polypeptide [Source:MGI Symbol;Acc:MGI:1915071]
ENSMUSG00000016344	72.87661645	49.90386258	75.66065856	60.39772232	47.90249703	39.18038462	94.45750173	117.9196573	81.81674101	40.9651418	40.9823605	38.9510123	Ppdpf	pancreatic progenitor cell differentiation and proliferation factor [Source:MGI Symbol;Acc:MGI:1913746]
ENSMUSG00000016346	0.125204135	0.064079555	0.042727692	0.025964098	0.131546998	0.116558108	2.670285686	2.990562221	2.49902562	2.053189283	2.095976224	1.972604187	Kcnq2	potassium voltage-gated channel, subfamily Q, member 2 [Source:MGI Symbol;Acc:MGI:1309503]
ENSMUSG00000016349	0.309706006	0.332866228	0.549596212	0.019267504	0.021693051	0.031452995	0.847928641	0.61361585	0.749480283	0.627922676	0.962449629	0.731917556	Eef1a2	eukaryotic translation elongation factor 1 alpha 2 [Source:MGI Symbol;Acc:MGI:1096317]
ENSMUSG00000016356	0.834347409	1.080893173	0.732170363	1.640619939	0.95488484	1.395846447	0.598569402	0.472308562	0.845913519	0.812938348	0.781323807	0.699807461	Col20a1	collagen, type XX, alpha 1 [Source:MGI Symbol;Acc:MGI:1920618]
ENSMUSG00000016382	40.5213161	51.90994531	35.08570951	32.84997124	38.07109959	43.45783524	9.860820809	7.887460008	10.61144842	45.15517314	41.80985661	41.07544379	Pls3	plastin 3 (T-isoform) [Source:MGI Symbol;Acc:MGI:104807]
ENSMUSG00000016386	2.182872555	3.067553848	2.386321378	3.279948748	2.487818035	2.733516769	4.393100251	5.167880074	3.891276115	3.507931587	3.664839365	3.3772048	Mpped2	metallophosphoesterase domain containing 2 [Source:MGI Symbol;Acc:MGI:1924265]
ENSMUSG00000016409	9.737167283	12.15247286	10.13591597	12.48768084	15.29805294	17.83028261	9.308276903	8.747908904	10.90255031	13.2672383	13.22190599	13.61085979	Nkap	NFKB activating protein [Source:MGI Symbol;Acc:MGI:1914300]
ENSMUSG00000016427	91.39702665	78.04935635	136.7827625	89.79429292	93.65350913	58.19539703	80.95540846	73.14554772	44.25479035	55.11403299	44.10861038	42.22832405	Ndufa1	NADH:ubiquinone oxidoreductase subunit A1 [Source:MGI Symbol;Acc:MGI:1929511]
ENSMUSG00000016458	0.332351863	0.535808356	0.181471679	0.268792591	0.279351092	0.405034241	0.178029705	0.131696749	0.226848953	0.614380514	0.645515264	0.687256264	Wt1	Wilms tumor 1 homolog [Source:MGI Symbol;Acc:MGI:98968]
ENSMUSG00000016477	8.814619301	11.56966322	7.041630828	9.200469553	11.87550752	14.74205129	11.42176512	10.19718179	11.87491209	19.39096396	21.96299402	19.18087186	E2f3	E2F transcription factor 3 [Source:MGI Symbol;Acc:MGI:1096340]
ENSMUSG00000016481	17.19615482	18.04725569	14.32868351	16.18938923	15.55870696	16.87068831	7.324674064	7.599177099	8.70016987	16.24492087	16.57640518	15.41974009	Cr1l	complement component (3b/4b) receptor 1-like [Source:MGI Symbol;Acc:MGI:88513]
ENSMUSG00000016487	83.94566615	115.8262475	71.26607895	107.0431036	126.2638607	160.6216478	30.93168574	26.83462345	37.33728552	62.62320731	62.72346839	63.18685924	Ppfibp1	PTPRF interacting protein, binding protein 1 (liprin beta 1) [Source:MGI Symbol;Acc:MGI:1914783]
ENSMUSG00000016493	0.77879616	0.63185314	0.489268102	0.908449725	1.004216039	1.590840985	0.110614553	0	0.082371764	0.07916077	0.191534093	0.109802687	Cd46	CD46 antigen, complement regulatory protein [Source:MGI Symbol;Acc:MGI:1203290]
ENSMUSG00000016494	138.5230844	145.9372191	139.4485731	248.3912815	246.5121162	231.8989869	3.188100064	3.576450514	2.848913651	12.8468725	10.95901662	12.2240912	Cd34	CD34 antigen [Source:MGI Symbol;Acc:MGI:88329]
ENSMUSG00000016495	53.24916538	47.59874729	59.95037199	48.05770576	48.64937145	39.65570476	23.9462673	26.0118362	19.76178605	25.75966253	23.17286038	21.56861144	Plgrkt	plasminogen receptor, C-terminal lysine transmembrane protein [Source:MGI Symbol;Acc:MGI:1915009]
ENSMUSG00000016496	3.647086075	3.160115436	2.797354375	1.341405527	1.135824674	1.30299806	0.265149462	0.470743763	0.486516473	4.335474953	4.449915507	3.726959763	Cd274	CD274 antigen [Source:MGI Symbol;Acc:MGI:1926446]
ENSMUSG00000016498	0.076427154	0.029336599	0.052163707	0.023773522	0	0	0	0	0	0.091149713	0	0	Pdcd1lg2	programmed cell death 1 ligand 2 [Source:MGI Symbol;Acc:MGI:1930125]
ENSMUSG00000016503	8.130304354	8.802581872	6.83044934	9.7547997	9.819258097	10.73950959	13.98657587	12.79043013	13.80224996	12.15282303	10.38756841	11.92102105	Gtf3a	general transcription factor III A [Source:MGI Symbol;Acc:MGI:1913846]
ENSMUSG00000016510	6.244439773	6.317417679	5.280058191	6.440598971	6.799841068	7.62544781	5.191196496	3.731640016	5.247333014	6.964919884	5.534570065	5.758023799	Mtif3	mitochondrial translational initiation factor 3 [Source:MGI Symbol;Acc:MGI:1923616]
ENSMUSG00000016520	6.821589534	8.459673399	5.741566146	7.727708196	9.079566985	11.066228	7.217424946	7.066403868	9.203368281	9.445979552	10.01889733	9.707614456	Lnx2	ligand of numb-protein X 2 [Source:MGI Symbol;Acc:MGI:2155959]
ENSMUSG00000016524	0.32076615	0.158305162	0	0.213809786	0	0.279224883	0	0	0	0.04098825	0	0	Il19	interleukin 19 [Source:MGI Symbol;Acc:MGI:1890472]
ENSMUSG00000016526	5.215574282	4.996372265	4.396018698	6.713225134	7.038703693	6.934941671	33.91907178	31.93597994	35.55042238	30.64559078	29.60412592	28.60964009	Dyrk3	dual-specificity tyrosine-(Y)-phosphorylation regulated kinase 3 [Source:MGI Symbol;Acc:MGI:1330300]
ENSMUSG00000016528	66.89528079	68.41482695	72.00608602	60.58283936	54.42927847	50.67439225	26.68363235	23.88496509	25.70860548	30.22341155	31.98152734	30.83039943	Mapkapk2	MAP kinase-activated protein kinase 2 [Source:MGI Symbol;Acc:MGI:109298]
ENSMUSG00000016529	1.478077826	0.794305212	1.916777299	0.214560569	0.207061	0.150110018	0.029324316	0.032538815	0	0.411321778	0.30075239	0.465745401	Il10	interleukin 10 [Source:MGI Symbol;Acc:MGI:96537]
ENSMUSG00000016534	209.4097003	244.8641454	180.5939874	219.5067037	236.7378937	262.606232	21.06051724	17.86956479	23.90376234	67.92860834	68.27420483	67.28734981	Lamp2	lysosomal-associated membrane protein 2 [Source:MGI Symbol;Acc:MGI:96748]
ENSMUSG00000016541	101.1180571	89.63422961	87.42222497	88.10133237	86.42560808	78.47491364	127.7388101	131.1124422	130.6094425	122.0408309	124.9430189	121.8205546	Atxn10	ataxin 10 [Source:MGI Symbol;Acc:MGI:1859293]
ENSMUSG00000016552	1.10329456	0.865615658	0.69510454	0.678841553	0.904421133	1.089696939	4.297181478	3.591188634	5.360199204	6.864717586	7.922301282	7.134474137	Foxred2	FAD-dependent oxidoreductase domain containing 2 [Source:MGI Symbol;Acc:MGI:106315]
ENSMUSG00000016554	86.23350279	69.06440178	114.0388508	79.52447764	73.399762	59.83359665	157.2842025	159.879718	119.4902377	93.59483925	82.7645358	86.5128053	Eif3d	eukaryotic translation initiation factor 3, subunit D [Source:MGI Symbol;Acc:MGI:1933181]
ENSMUSG00000016559	579.1651237	529.7838074	536.3840913	689.7057743	673.8066318	622.3017746	575.7235927	592.0533596	570.7329377	594.7503679	593.5035468	588.5203068	H3f3b	H3.3 histone B [Source:MGI Symbol;Acc:MGI:1101768]
ENSMUSG00000016619	26.10369109	24.54432952	25.94496464	25.90158336	27.6024386	27.39526597	40.85828313	38.57172417	43.2093456	45.65899161	47.13255801	48.87601825	Nup50	nucleoporin 50 [Source:MGI Symbol;Acc:MGI:1351502]
ENSMUSG00000016624	2.108775009	2.046373167	2.231721926	2.542758784	3.460328332	3.013910488	6.726830173	6.971297779	7.299707684	3.793692721	4.062927505	4.724616495	Phf21b	PHD finger protein 21B [Source:MGI Symbol;Acc:MGI:2443812]
ENSMUSG00000016626	0.365422402	0.441842814	0.280587581	0.409207417	0.537508668	0.473170574	0.521985278	0.488703986	0.578204311	2.288031971	1.885899176	1.991654895	Nlrp14	NLR family, pyrin domain containing 14 [Source:MGI Symbol;Acc:MGI:1924108]

ENSMUSG00000016637	43.33583417	31.73528501	46.7268511	39.723262	35.78991869	27.77988416	26.34161422	27.14860538	21.88400065	22.26803769	17.22621154	18.29471499	Ift27	intraflagellar transport 27 [Source:MGI Symbol;Acc:MGI:1914292]
ENSMUSG00000016664	40.8279014	54.10548306	34.24954599	58.54805256	61.49118383	63.26562852	49.07403556	44.48116207	53.68939573	56.24520004	63.17644739	58.11222309	Pacsin2	protein kinase C and casein kinase substrate in neurons 2 [Source:MGI Symbol;Acc:MGI:1345153]
ENSMUSG00000016756	0.369842111	0.571912386	0.42191497	0.108469611	0.055511196	0.032194519	0.009433903	0	0.009835253	0.141777872	0.237489106	0.271575205	Cmah	cytidine monophospho-N-acetylneuraminic acid hydroxylase [Source:MGI Symbol;Acc:MGI:103227]
ENSMUSG00000016757	33.36870445	31.89542536	31.48932255	32.24536548	31.62562553	27.02697532	27.67414096	28.53360915	28.97077491	32.59870569	32.61655898	29.245638	Ttll12	tubulin tyrosine ligase-like family, member 12 [Source:MGI Symbol;Acc:MGI:3039573]
ENSMUSG00000016758	3.911680712	3.193064298	3.244354445	2.58756589	3.225449641	2.187449018	3.757494357	5.739038183	3.917350839	1.284408544	2.637836629	1.184796809	Bik	BCL2-interacting killer [Source:MGI Symbol;Acc:MGI:1206591]
ENSMUSG00000016763	0.109431556	0.302438549	0.246477493	0.183815514	0.14946799	0.341743348	0.224705403	0.151770567	0.183337927	0.215344675	0.273272193	0.261848532	Scube1	signal peptide, CUB domain, EGF-like 1 [Source:MGI Symbol;Acc:MGI:1890616]
ENSMUSG00000016831	19.9874266	26.87723106	16.98689747	24.88917807	27.03524745	31.03366321	36.75624747	36.46995605	41.53981473	44.60019924	43.42462868	42.82618527	Tox4	TOX high mobility group box family member 4 [Source:MGI Symbol;Acc:MGI:1915389]
ENSMUSG00000016833	10.89102804	7.709986871	20.38102032	11.66283589	10.97380342	7.683535958	15.3818814	14.32652972	7.394967812	9.422365211	6.762107196	5.933543261	Mrps18c	mitochondrial ribosomal protein S18C [Source:MGI Symbol;Acc:MGI:1915985]
ENSMUSG00000016918	18.04089527	23.08710401	16.08447282	5.169623509	5.389275008	6.418617802	12.66538785	10.01765494	14.52736593	23.16356832	22.70272659	22.50828468	Sulf1	sulfatase 1 [Source:MGI Symbol;Acc:MGI:2138563]
ENSMUSG00000016921	96.91911833	98.82260438	90.5006479	110.4733993	111.497038	115.1737899	173.69729	176.6069524	196.1309912	203.3188107	209.173217	205.2546585	Srsf6	serine and arginine-rich splicing factor 6 [Source:MGI Symbol;Acc:MGI:1915246]
ENSMUSG00000016933	45.53408716	52.97719665	43.62792905	50.01604398	52.35376621	53.91068106	53.83798068	51.99457605	60.79264813	43.50960121	44.17519187	41.53882087	Plcg1	phospholipase C, gamma 1 [Source:MGI Symbol;Acc:MGI:97615]
ENSMUSG00000016940	50.93642613	40.90226425	54.05330159	54.01340416	42.83444526	37.70910483	23.76084796	25.27926721	20.96781833	18.78331641	22.48580677	19.81733193	Kctd2	potassium channel tetramerisation domain containing 2 [Source:MGI Symbol;Acc:MGI:1917632]
ENSMUSG00000016942	0.72384372	0.740927336	0.548937697	0.800567782	0.535176354	0.347139159	0.287213862	0.212465284	0.42419662	0.35970062	0.133894862	0.095035276	Tmprss6	transmembrane serine protease 6 [Source:MGI Symbol;Acc:MGI:1919003]
ENSMUSG00000016946	49.72379195	52.94190985	41.98063677	46.62357489	49.58533396	53.26344986	64.26385339	62.37641337	69.24127165	63.16178714	66.6055174	63.84492549	Kctd5	potassium channel tetramerisation domain containing 5 [Source:MGI Symbol;Acc:MGI:1916509]
ENSMUSG00000016982	0	0	0	0	0	0	0.078333715	0.052152342	0.097999555	0.031393121	0.116857669	0.031103511	Pom121l2	POM121 membrane glycoprotein-like 2 (rat) [Source:MGI Symbol;Acc:MGI:2684870]
ENSMUSG00000016984	4.307989582	5.635739411	4.094701392	5.248256243	5.660491951	6.907212951	11.40053734	10.54187631	12.90951145	21.51095659	21.66985154	20.5653443	Etaa1	Ewing tumor-associated antigen 1 [Source:MGI Symbol;Acc:MGI:1915395]
ENSMUSG00000016995	1.70178587	1.671503629	3.006279967	1.043149676	0.876469918	0.622693684	0.059581007	0.165280526	0.07764473	0.58202043	0.49996414	0.473149651	Matn4	matrilin 4 [Source:MGI Symbol;Acc:MGI:1328314]
ENSMUSG00000016998	0	0	0	0	0	0	0	0	0	0	0	0	Svs4	seminal vesicle secretory protein 4 [Source:MGI Symbol;Acc:MGI:98450]
ENSMUSG00000017000	0	0	0	0	0	0	0	0	0	0	0	0	Svs6	seminal vesicle secretory protein 6 [Source:MGI Symbol;Acc:MGI:106178]
ENSMUSG00000017002	435.2118044	339.3574459	507.9123565	412.7532309	361.5975265	266.4953189	1.114324011	1.366630232	1.222874856	0.822643556	1.257691811	1.397236202	Slpi	secretory leukocyte peptidase inhibitor [Source:MGI Symbol;Acc:MGI:109297]
ENSMUSG00000017003	0	0	0	0	0	0	0	0	0	0	0	0	Svs3a	seminal vesicle secretory protein 3A [Source:MGI Symbol;Acc:MGI:1927635]
ENSMUSG00000017004	0	0	0.087405258	0.079669599	0	0.390166912	0	0	0	0	0	0	Svs5	seminal vesicle secretory protein 5 [Source:MGI Symbol;Acc:MGI:98453]
ENSMUSG00000017007	0.10527767	0.138551599	0.030795002	0.084208622	0.205420531	0.160376193	0	0	0	0.053810509	0	0.039985569	Rbpjl	recombination signal binding protein for immunoglobulin kappa J region-like [Source:MGI Symbol;Acc:MGI:1196616]
ENSMUSG00000017009	153.8506312	146.6093469	160.3794146	165.3494566	155.8131906	143.4165375	45.21060221	45.11322825	43.24717959	56.83358179	57.70741055	56.56693171	Sdc4	syndecan 4 [Source:MGI Symbol;Acc:MGI:1349164]
ENSMUSG00000017049	0	0	0	0	0	0	0	0	0	0	0	0	Ccdc70	coiled-coil domain containing 70 [Source:MGI Symbol;Acc:MGI:1915179]
ENSMUSG00000017057	15.77633942	17.7134179	13.76632819	20.17409379	21.64202093	24.5010526	0.786511021	0.643062358	0.528666063	4.655467915	4.650790404	4.345425292	Il13ra1	interleukin 13 receptor, alpha 1 [Source:MGI Symbol;Acc:MGI:105052]
ENSMUSG00000017064	0	0	0	0	0	0	0	0	0	0	0	0	Prl5a1	prolactin family 5, subfamily a, member 1 [Source:MGI Symbol;Acc:MGI:106332]
ENSMUSG00000017119	67.6895913	85.51605458	60.66498933	75.75223281	87.85258282	102.6919313	64.77011963	57.13136005	76.15316275	118.3552837	118.2491471	115.5180381	Nbr1	NBR1, autophagy cargo receptor [Source:MGI Symbol;Acc:MGI:108498]
ENSMUSG00000017132	16.3777948	17.05293871	17.06867814	20.27121445	19.34555389	18.54393304	5.734867104	5.756311057	4.63246596	9.013421184	9.163405749	9.473473757	Cyth1	cytohesin 1 [Source:MGI Symbol;Acc:MGI:1334257]
ENSMUSG00000017144	43.79147548	50.15067634	35.8913807	35.00079169	37.00204871	39.99256368	13.0857879	12.05378614	14.31528235	33.29756867	29.41563268	32.24303467	Rnd3	Rho family GTPase 3 [Source:MGI Symbol;Acc:MGI:1921444]
ENSMUSG00000017146	12.79414614	15.00196946	9.489280157	18.08322659	21.95412096	28.16823043	49.54536523	43.80540181	60.06286208	99.19578962	100.5904219	99.52212238	Brca1	breast cancer 1, early onset [Source:MGI Symbol;Acc:MGI:104537]
ENSMUSG00000017165	0.093540191	0	0	0	0.196557906	0.213743613	0.417552762	0	0.087063373	0.083669492	0.233588318	0.165795238	Gast	gastrin [Source:MGI Symbol;Acc:MGI:104768]
ENSMUSG00000017167	1.621322444	1.412453427	1.428955691	1.026202907	0.888761173	1.535611059	0.163628146	0.209497941	0.14434491	0.769254898	0.739339173	0.437303953	Cntnap1	contactin associated protein-like 1 [Source:MGI Symbol;Acc:MGI:1858201]
ENSMUSG00000017176	50.05259773	52.38856895	52.62457997	58.25509974	58.45313378	46.45053087	53.90538442	49.9528853	47.36623975	51.05376728	56.95851225	55.03505704	Nt5c3b	5'-nucleotidase, cytosolic IIIB [Source:MGI Symbol;Acc:MGI:1915356]
ENSMUSG00000017188	101.2520953	76.38533139	111.3646868	86.30611704	78.21269337	61.20784839	75.91662163	76.20546287	67.51919773	70.83354083	84.22134557	83.90533867	Coa3	cytochrome C oxidase assembly factor 3 [Source:MGI Symbol;Acc:MGI:1098757]
ENSMUSG00000017195	0.138578061	0.279264581	0.390156805	0.387956308	0.363996122	0.105552401	0.123719337	0.102960985	0.128982775	0.154943504	0.086514192	0.460542327	Zpbp2	zona pellucida binding protein 2 [Source:MGI Symbol;Acc:MGI:1916626]
ENSMUSG00000017204	0.078922391	0.018176638	0.064640132	0	0.066336491	0.108204764	0.394576638	0.250188394	0.293830899	0.94596249	0.880312053	1.077121666	Gsdma	gasdermin A [Source:MGI Symbol;Acc:MGI:1889509]
ENSMUSG00000017210	36.23321079	40.01087688	35.19101947	42.65294504	39.68430481	37.5720617	62.19442368	55.76984108	62.27816185	71.96251465	71.61310723	74.0239902	Med24	mediator complex subunit 24 [Source:MGI Symbol;Acc:MGI:1344385]
ENSMUSG00000017211	0	0	0	0	0	0	0	0	0	0.087771873	0.081680446	0	Gsdma2	gasdermin A2 [Source:MGI Symbol;Acc:MGI:1921490]
ENSMUSG00000017221	133.085425	113.6630211	149.2947566	112.4556295	101.2267934	85.32516388	226.4019986	240.1111023	212.5553302	173.0821013	174.6385428	177.345669	Psmd3	proteasome (prosome, macropain) 26S subunit, non-ATPase, 3 [Source:MGI Symbol;Acc:MGI:98858]
ENSMUSG00000017264	41.61771774	36.04874314	37.57764022	42.53681032	39.20269098	41.96334468	72.8774733	70.8663754	72.8763249	59.48338047	59.20211639	62.55607089	Exosc10	exosome component 10 [Source:MGI Symbol;Acc:MGI:1355322]
ENSMUSG00000017286	37.45693636	36.13790726	32.36912612	39.55837949	41.01191438	34.15451761	60.37904131	59.45009024	55.57522664	58.06218952	62.05092078	61.92878905	Glod4	glyoxalase domain containing 4 [Source:MGI Symbol;Acc:MGI:1914451]
ENSMUSG00000017288	42.62287757	46.24102479	51.00786865	45.51568619	44.21394842	37.69141101	30.14645657	29.36596106	29.57282202	39.24381803	35.50738166	36.99460903	Vps53	VPS53 GARP complex subunit [Source:MGI Symbol;Acc:MGI:1915549]
ENSMUSG00000017291	6.736191307	8.759434642	5.776151706	8.795902625	10.92226402	13.26548172	4.8688631	4.649388557	6.157337968	13.47444285	13.40582087	14.31361446	Taok1	TAO kinase 1 [Source:MGI Symbol;Acc:MGI:1914490]
ENSMUSG00000017299	23.54001897	25.2176915	30.66330645	29.28954521	27.04955401	24.18011773	21.51964412	18.13757763	15.5136761	16.33100838	17.24674338	16.5894043	Dnttip1	deoxynucleotidyltransferase, terminal, interacting protein 1 [Source:MGI Symbol;Acc:MGI:1923483]
ENSMUSG00000017300	0.086402586	0	0.088458334	0.080629474	0	0	0	0	0	0	0	0	Tnnc2	troponin C2, fast [Source:MGI Symbol;Acc:MGI:98780]
ENSMUSG00000017307	10.2567907	10.65890743	10.28739564	11.0888965	9.987884286	11.05170463	22.70645444	22.55204733	22.97393188	14.50757647	15.47899851	15.70395784	Acot8	acyl-CoA thioesterase 8 [Source:MGI Symbol;Acc:MGI:2158201]
ENSMUSG00000017309	0.020172756	0.069689895	0	0.018824884	0.063584133	0.061460892	0.054029331	0.039967977	0	0.108264322	0.134334273	0.268163887	Cd300lg	CD300 molecule like family member G [Source:MGI Symbol;Acc:MGI:1289168]
ENSMUSG00000017310	0	0	0.058658123	0.267333408	0	0	0	0	0	0	0	0	Spint4	serine protease inhibitor, Kunitz type 4 [Source:MGI Symbol;Acc:MGI:1925489]
ENSMUSG00000017311	0	0	0	0	0	0	0.458228876	0.338972944	0.159241159	0.382584159	0.142413024	0.530676606	Pyy	peptide YY [Source:MGI Symbol;Acc:MGI:99924]
ENSMUSG00000017314	13.65983741	14.76200465	12.93597823	12.99227305	15.92503033	15.94682201	9.94377175	7.858978025	10.63576368	8.59987527	10.73631615	8.590379628	Mpp2	membrane protein, palmitoylated 2 (MAGUK p55 subfamily member 2) [Source:MGI Symbol;Acc:MGI:1858257]
ENSMUSG00000017316	0	0	0	0	0	0	0.155525725	0	0	0	0	0.077192115	Ppy	pancreatic polypeptide [Source:MGI Symbol;Acc:MGI:97753]
ENSMUSG00000017344	0.076495089	0.132132042	0.078315111	0.178459901	0.040185172	0.116529851	0.068293074	0.075779285	0	0	0	0	Vtn	vitronectin [Source:MGI Symbol;Acc:MGI:98940]
ENSMUSG00000017376	20.86505815	21.5090539	19.84566698	22.14924505	23.26230039	25.26150029	23.54918	21.50472888	25.16587764	36.51161235	36.40465904	39.38448759	Nlk	nemo like kinase [Source:MGI Symbol;Acc:MGI:1201387]
ENSMUSG00000017386	59.03715896	56.88045601	59.38031027	58.6918064	55.70972049	50.98531981	56.42818082	60.99091172	60.23778405	52.97428046	54.68330676	53.0185205	Traf4	TNF receptor associated factor 4 [Source:MGI Symbol;Acc:MGI:1202880]
ENSMUSG00000017390	5.155999825	5.424817659	5.012842264	7.234549024	4.793640652	5.198637756	49.37633398	52.76785974	48.78400957	20.83658869	20.90346957	21.893547	Aldoc	aldolase C, fructose-bisphosphate [Source:MGI Symbol;Acc:MGI:101863]
ENSMUSG00000017400	4.015992197	4.59709395	3.352865709	2.743820985	2.034374326	2.221350288	19.86901619	21.05243256	20.58081398	11.84574076	13.07315955	11.96949508	Stac2	SH3 and cysteine rich domain 2 [Source:MGI Symbol;Acc:MGI:2144518]
ENSMUSG00000017404	428.8878495	305.5017213	531.0584577	381.1973026	390.5822569	248.5185883	483.49085	477.0496657	383.7826971	418.9939975	356.0755608	370.5577394	Rpl19	ribosomal protein L19 [Source:MGI Symbol;Acc:MGI:98020]
ENSMUSG00000017405	12.03213181	12.80076669	11.78613138	15.13263981	12.56364658	12.84183432	12.86706918	13.14583105	12.76595509	12.97695529	11.27262997	12.98888353	Nek8	NIMA (never in mitosis gene a)-related expressed kinase 8 [Source:MGI Symbol;Acc:MGI:1890646]
ENSMUSG00000017412	0.142478437	0.236991797	0.097245585	0.125571951	0.124747015	0.156755801	0	0.015682799	0	0.141603997	0.092243609	0.126267896	Cacnb4	calcium channel, voltage-dependent, beta 4 subunit [Source:MGI Symbol;Acc:MGI:103301]
ENSMUSG00000017417	5.74777574	6.68780568	5.904963037	8.269083562	8.806815263	8.208706467	6.27181291	8.540986181	8.526234021	5.641093401	6.728123641	6.066598473	Plxdc1	plexin domain containing 1 [Source:MGI Symbol;Acc:MGI:1919574]
ENSMUSG00000017418	3.828621768	5.444267642	3.088260209	5.183276714	6.384322679	8.836945234	12.14463536	10.08538763	14.32158738	18.48512035	18.60256323	17.90331521	Arl5b	ADP-ribosylation factor-like 5B [Source:MGI Symbol;Acc:MGI:1923119]
ENSMUSG00000017421	68.23959916	82.20216365	54.15812513	75.75456846	84.1098961	97.59633072	90.33785435	86.76191144	106.9337177	138.6964492	135.0667381	137.2818345	Zfp207	zinc finger protein 207 [Source:MGI Symbol;Acc:MGI:1340045]
ENSMUSG00000017428	104.2203285	110.938296	96.36917034	116.1608133	115.3412228	112.0407449	159.812592	149.4167989	170.5455904	227.9986256	220.7333813	224.6804243	Psmd11	proteasome (prosome, macropain) 26S subunit, non-ATPase, 11 [Source:MGI Symbol;Acc:MGI:1916327]
ENSMUSG00000017446	6.797003432	9.087394432	6.63437503	6.772875779	8.139918096	11.10017031	0.231414784	0.028531357	0.026806661	0.566757202	1.1747168	0.918865173	C1qtnf1	C1q and tumor necrosis factor related protein 1 [Source:MGI Symbol;Acc:MGI:1919254]
ENSMUSG00000017453	0.51443111	0.930904649	0.075238685	0.411478851	0.30885273	0.615737365	70.56392067	69.78116998	72.43732103	41.08450723	38.26380122	40.47754101	Pipox	pipecolic acid oxidase [Source:MGI Symbol;Acc:MGI:1197006]
ENSMUSG00000017466	727.9471726	696.0878882	674.512774	542.7792614	542.2956272	548.2765024	31.28364132	30.14998984	32.57837484	137.3850037	130.0828797	117.3267149	Timp2	tissue inhibitor of metalloproteinase 2 [Source:MGI Symbol;Acc:MGI:98753]

ENSMUSG00000017478	42.37141235	46.0413824	37.5086258	45.69060712	44.3087544	48.88045743	107.4584881	90.99168692	114.3837868	98.02765706	102.5778077	98.44677245	Zc3h18	zinc finger CCCH-type containing 18 [Source:MGI Symbol;Acc:MGI:1923264]
ENSMUSG00000017485	49.24538843	59.87594708	43.30004452	55.07917953	64.21006336	79.68219641	33.28502461	31.11089672	39.80366636	39.73494952	34.97006669	36.54681627	Top2b	topoisomerase (DNA) II beta [Source:MGI Symbol;Acc:MGI:98791]
ENSMUSG00000017491	0.485713342	0.361915453	0.234009297	0.159974015	0.420263252	0.41348366	0.076523614	0	0	0.408902697	0.237827858	0.3291685	Rarb	retinoic acid receptor, beta [Source:MGI Symbol;Acc:MGI:97857]
ENSMUSG00000017493	651.5378719	601.4632666	675.9395144	774.320617	730.1735745	630.7960067	18.1196015	16.35852481	16.18215928	60.02561112	60.0682003	61.63154164	Igfbp4	insulin-like growth factor binding protein 4 [Source:MGI Symbol;Acc:MGI:96439]
ENSMUSG00000017499	5.230807646	6.195645149	4.798871588	7.302937554	8.193740048	8.94119161	32.72601083	30.59316445	34.5735334	33.25010293	36.563296	34.7735487	Cdc6	cell division cycle 6 [Source:MGI Symbol;Acc:MGI:1345150]
ENSMUSG00000017548	32.11853244	42.3472363	24.21670147	46.67018981	55.01580599	66.51514352	107.6245666	100.5084879	133.3877478	216.8155439	215.7624237	219.3579714	Suz12	SUZ12 polycomb repressive complex 2 subunit [Source:MGI Symbol;Acc:MGI:1261758]
ENSMUSG00000017550	2.057313253	2.873166216	1.496790597	3.610953653	3.844776616	5.934664978	12.61476591	10.38977936	16.47596834	20.30504513	20.84155928	22.35992499	Atad5	ATPase family, AAA domain containing 5 [Source:MGI Symbol;Acc:MGI:2442925]
ENSMUSG00000017561	26.39881509	31.41898015	22.81913034	32.98426761	34.57888273	39.08320022	66.41451259	64.76873846	74.15419679	87.75030099	86.66052238	87.27700081	Crlf3	cytokine receptor-like factor 3 [Source:MGI Symbol;Acc:MGI:1860086]
ENSMUSG00000017588	0.083280944	0.319674294	0.312628872	0.051810939	0	0.063433588	0	0.082501641	0	0.049661892	0.092430646	0.172213118	Krt27	keratin 27 [Source:MGI Symbol;Acc:MGI:1339999]
ENSMUSG00000017607	0.300548227	0.317255339	0.102566357	0.186977778	0.394717564	0.34338322	0.134161306	0.04962264	0.466229936	2.733138473	2.877024922	2.929885576	Tns4	tensin 4 [Source:MGI Symbol;Acc:MGI:2144377]
ENSMUSG00000017615	32.77704254	32.74185407	29.90940705	34.2548633	34.75147976	38.15757371	14.10801932	13.77598176	16.05603003	25.61752463	28.39329231	26.40988832	Tnfaip1	tumor necrosis factor, alpha-induced protein 1 (endothelial) [Source:MGI Symbol;Acc:MGI:104961]
ENSMUSG00000017631	25.90064316	31.28629263	21.58315864	24.67127695	29.2505971	35.0468376	30.11649967	25.0522926	35.13327093	46.16153803	49.46553919	49.10735368	Abr	active BCR-related gene [Source:MGI Symbol;Acc:MGI:107771]
ENSMUSG00000017639	2.416713639	2.44142367	2.001863808	2.55251876	2.746892971	3.639698488	7.178880056	6.834064936	7.463844935	12.66555748	14.53888997	13.45409096	Rab11fip4	RAB11 family interacting protein 4 (class II) [Source:MGI Symbol;Acc:MGI:2442920]
ENSMUSG00000017652	18.12351976	14.7506853	18.71713119	4.959047032	4.458331856	3.83622177	3.292701778	2.710768195	2.657639548	13.26681974	14.12868452	14.05821371	Cd40	CD40 antigen [Source:MGI Symbol;Acc:MGI:88336]
ENSMUSG00000017664	57.97936722	50.00258684	60.01798676	48.36441345	42.38984464	39.0127993	32.63506306	29.79911714	29.29613744	25.17874442	28.55227231	23.77363135	Slc35c2	solute carrier family 35, member C2 [Source:MGI Symbol;Acc:MGI:2385166]
ENSMUSG00000017667	2.936740181	3.550897646	2.65226223	3.083082393	3.228772036	4.425790965	0.753783243	0.690949146	0.888354123	3.358608644	2.854873439	2.955822959	Zfp334	zinc finger protein 334 [Source:MGI Symbol;Acc:MGI:2388656]
ENSMUSG00000017670	37.77577314	44.40806029	30.87690782	39.11185659	41.89702748	45.22681485	19.85223384	18.90627629	23.7931887	38.37050564	39.44228658	37.72558475	Elmo2	engulfment and cell motility 2 [Source:MGI Symbol;Acc:MGI:2153045]
ENSMUSG00000017677	158.4370391	185.3859735	134.3593631	200.5953028	233.7592975	272.0711913	73.67420227	73.71226949	91.45509816	165.6357124	161.1247727	170.8626508	Wsb1	WD repeat and SOCS box-containing 1 [Source:MGI Symbol;Acc:MGI:1926139]
ENSMUSG00000017679	18.94793251	21.52399436	18.67622735	21.01471777	23.77256502	25.47642558	4.887774673	4.618506361	5.653061149	12.35746833	13.28001447	11.73174354	Ttpal	tocopherol (alpha) transfer protein-like [Source:MGI Symbol;Acc:MGI:1923330]
ENSMUSG00000017686	22.76794507	27.84130589	18.62523892	25.8239296	28.67103473	32.96918707	34.23186698	31.96485304	37.42577027	57.72376411	57.55685051	56.49379287	Rhot1	ras homolog family member T1 [Source:MGI Symbol;Acc:MGI:1926078]
ENSMUSG00000017688	0	0	0	0	0.019863057	0.014399833	0.033756462	0	0	0	0	0	Hnf4g	hepatocyte nuclear factor 4, gamma [Source:MGI Symbol;Acc:MGI:1353604]
ENSMUSG00000017692	1.845465618	1.705711317	1.76507323	1.79013812	1.607293485	2.108486655	0.28182455	0.481104376	0.406819824	0.456121498	0.60638051	1.054465197	Rhbdl3	rhomboid like 3 [Source:MGI Symbol;Acc:MGI:2179276]
ENSMUSG00000017697	8.344219713	7.394789195	6.731845204	4.449536418	5.37328538	4.481166361	22.24559914	22.85568241	20.07826095	26.62170328	27.68686085	24.36552891	Ada	adenosine deaminase [Source:MGI Symbol;Acc:MGI:87916]
ENSMUSG00000017707	157.0179902	188.9420656	137.3357558	153.7475174	160.3928953	168.8501704	46.42984918	42.1504665	46.67438291	107.2905717	100.6767099	109.5080627	Serinc3	serine incorporator 3 [Source:MGI Symbol;Acc:MGI:1349457]
ENSMUSG00000017713	1.461635165	0.915396296	1.968962315	1.43576202	1.023787901	0.957048278	1.923032027	2.057624353	1.885508326	1.651450285	1.13128391	1.908917756	Tha1	threonine aldolase 1 [Source:MGI Symbol;Acc:MGI:1919026]
ENSMUSG00000017715	55.24708291	52.56319074	57.81422883	63.17987982	52.90373115	48.92804029	36.01771863	34.9659896	36.30047502	36.32947853	36.53830544	38.02748197	Pgs1	phosphatidylglycerophosphate synthase 1 [Source:MGI Symbol;Acc:MGI:1921701]
ENSMUSG00000017716	32.91950633	26.6840689	34.29944002	39.365787	35.79079851	27.18667008	89.73035274	97.67510921	85.46687217	74.47718107	79.94808403	77.60161874	Birc5	baculoviral IAP repeat-containing 5 [Source:MGI Symbol;Acc:MGI:1203517]
ENSMUSG00000017718	3.576583382	3.698352311	2.877034152	4.359325838	4.371287779	4.586695	1.629128671	1.663094991	1.800343537	1.240494251	1.670846608	1.261393794	Afmid	arylformamidase [Source:MGI Symbol;Acc:MGI:2448704]
ENSMUSG00000017720	0.09213809	0	0	0	0.145208731	0.105269874	0.164517576	0	0.128637532	0.453284401	0.230086995	0.081655042	Trp53tg5	transformation related protein 53 target 5 [Source:MGI Symbol;Acc:MGI:1920853]
ENSMUSG00000017721	56.76191009	50.45178298	53.65595269	54.43162397	50.78789836	50.13082368	44.12753343	42.60428683	47.09877455	39.71444178	38.04523951	38.80799405	Pigt	phosphatidylinositol glycan anchor biosynthesis, class T [Source:MGI Symbol;Acc:MGI:1926178]
ENSMUSG00000017723	3.026718726	1.413009001	1.591240977	1.221398565	0.429736866	1.993856769	113.3457291	132.9829203	90.98619025	24.43912694	21.58545537	23.85118943	Wfdc2	WAP four-disulfide core domain 2 [Source:MGI Symbol;Acc:MGI:1914951]
ENSMUSG00000017724	52.1245836	49.32180645	55.67265076	77.98189216	68.64351279	67.50604802	268.1272076	270.7603249	253.9881852	112.3509018	118.1166813	115.625416	Etv4	ets variant 4 [Source:MGI Symbol;Acc:MGI:99423]
ENSMUSG00000017733	0.05530654	0.063688323	0	0.103222308	0	0	0.049376419	0	0	0.445233544	0.046037115	0.245070082	Eppin	epididymal peptidase inhibitor [Source:MGI Symbol;Acc:MGI:1922776]
ENSMUSG00000017734	2.292275025	1.812787157	2.74266256	2.834969552	2.727587885	2.608458639	0.320534726	0.164156025	0.102821955	0.321144777	0.183912069	0.34265768	Dbndd2	dysbindin (dystrobrevin binding protein 1) domain containing 2 [Source:MGI Symbol;Acc:MGI:106562]
ENSMUSG00000017737	38.21062626	40.09875859	33.47290671	31.78171026	35.60809631	35.20360055	16.55250083	14.15375638	16.51447638	151.2473057	156.9581223	158.597548	Mmp9	matrix metallopeptidase 9 [Source:MGI Symbol;Acc:MGI:97011]
ENSMUSG00000017740	4.697103669	5.517569912	3.572847999	4.207225429	5.787298973	6.393879519	1.010474022	0.896992849	1.106136145	2.193527237	2.386744881	2.925584533	Slc12a5	solute carrier family 12, member 5 [Source:MGI Symbol;Acc:MGI:1862037]
ENSMUSG00000017747	27.62739642	25.43593659	26.65956429	24.74135016	22.67512985	19.80847401	19.1169682	18.63626151	16.97530755	18.57935582	17.14936738	16.34267344	Ghdc	GH3 domain containing [Source:MGI Symbol;Acc:MGI:1931556]
ENSMUSG00000017754	41.74560785	42.16300443	41.60293089	38.63275789	41.56542404	34.99240721	38.50771834	35.96233887	41.17866023	27.04392158	29.5251117	28.57663285	Pltp	phospholipid transfer protein [Source:MGI Symbol;Acc:MGI:103151]
ENSMUSG00000017756	26.65305407	29.98391631	24.21206518	29.48190593	32.47090317	35.03429792	40.11105242	37.53512409	46.06157671	34.70067279	36.64527251	35.24647793	Slc12a7	solute carrier family 12, member 7 [Source:MGI Symbol;Acc:MGI:1342283]
ENSMUSG00000017760	490.2738392	439.0964635	501.7960422	347.3319195	319.7292407	283.0387074	79.69215407	80.23418201	81.56879025	151.0839717	152.8986056	153.2728631	Ctsa	cathepsin A [Source:MGI Symbol;Acc:MGI:97748]
ENSMUSG00000017764	5.178906859	6.911293518	4.980035034	6.54921742	6.560037196	6.064484134	21.71368298	20.09024768	21.78151267	18.89763478	20.02360755	20.28893579	Zswim1	zinc finger SWIM-type containing 1 [Source:MGI Symbol;Acc:MGI:1919221]
ENSMUSG00000017765	58.74283562	62.71988931	59.59310345	64.94841309	65.6509777	66.27399203	50.45192342	47.25458643	50.47794192	41.00392744	41.71862968	40.31663667	Slc12a4	solute carrier family 12, member 4 [Source:MGI Symbol;Acc:MGI:1309465]
ENSMUSG00000017767	0.068190947	0.078525381	0.209440175	0.38180803	0.214936537	0.415518487	0	0.202658547	0.063469337	0.365971154	0.056762085	0	Spata25	spermatogenesis associated 25 [Source:MGI Symbol;Acc:MGI:1922892]
ENSMUSG00000017774	95.09701906	120.5698103	91.64747989	110.1790342	118.7622709	133.2209472	51.38794595	46.316473	61.33091239	80.71891511	82.52431507	77.23595303	Myo1c	myosin IC [Source:MGI Symbol;Acc:MGI:106612]
ENSMUSG00000017776	85.68532845	110.7255731	69.43936751	104.0252528	119.7456417	148.8984776	52.29507726	47.84158242	61.78407972	116.7936177	112.2972478	115.4599945	Crk	v-crk avian sarcoma virus CT10 oncogene homolog [Source:MGI Symbol;Acc:MGI:88508]
ENSMUSG00000017778	189.7909854	172.8771677	297.8603049	206.1587379	227.9441462	149.8147116	611.9432365	551.744189	309.5582714	283.6077201	222.43787	238.5025963	Cox7c	cytochrome c oxidase subunit 7C [Source:MGI Symbol;Acc:MGI:103226]
ENSMUSG00000017781	116.8985975	115.746205	122.2124663	126.0726021	125.8965827	113.4472033	64.34893277	67.91808328	61.91142569	83.16729864	86.8974827	82.62129349	Pitpna	phosphatidylinositol transfer protein, alpha [Source:MGI Symbol;Acc:MGI:99887]
ENSMUSG00000017801	20.48430671	19.63531337	16.87107454	16.37464522	17.19358916	18.82762888	29.21980921	26.26326609	28.51016726	40.19399325	40.23047982	38.77521425	Mlx	MAX-like protein X [Source:MGI Symbol;Acc:MGI:108398]
ENSMUSG00000017802	33.58622327	34.36760907	31.78339134	34.09257552	32.35788167	34.68292586	19.68012828	21.0331808	22.42292528	33.79931747	33.99518042	34.23828071	Retreg3	reticulophagy regulator family member 3 [Source:MGI Symbol;Acc:MGI:1915248]
ENSMUSG00000017817	2.53048243	2.700762506	2.727595697	1.247896753	1.10237831	1.065567011	0.505096097	0.458561594	0.545733119	2.85232359	2.936932414	2.771313178	Jph2	junctophilin 2 [Source:MGI Symbol;Acc:MGI:1891496]
ENSMUSG00000017830	17.78978214	20.63722188	18.25790818	15.0882213	16.52727728	15.71951499	3.755423616	4.340717688	4.323024502	7.642722447	7.805306876	7.533384429	Dhx58	DEXH (Asp-Glu-X-His) box polypeptide 58 [Source:MGI Symbol;Acc:MGI:1931560]
ENSMUSG00000017831	24.62288288	36.55960327	15.94451974	36.06058042	39.29049127	52.9940441	25.8984263	22.79171757	31.88462492	38.85963128	36.61109804	39.37399708	Rab5a	RAB5A, member RAS oncogene family [Source:MGI Symbol;Acc:MGI:105926]
ENSMUSG00000017832	0.811858261	2.15745351	2.173841084	1.923170927	1.377902301	2.092970839	0.167263516	0.185598756	1.046276818	0.614466807	0.363886979	0.664143478	Hspb9	heat shock protein, alpha-crystallin-related, B9 [Source:MGI Symbol;Acc:MGI:1922732]
ENSMUSG00000017837	31.30668319	33.95525182	29.51724379	31.49092551	27.72929848	28.64260856	32.82487531	28.56148171	31.17192843	33.27808937	33.27151377	32.61621543	Nkiras2	NFKB inhibitor interacting Ras-like protein 2 [Source:MGI Symbol;Acc:MGI:1919216]
ENSMUSG00000017843	64.19097433	70.87573225	58.82717636	66.87514713	71.73798992	76.61271285	138.2119937	143.8692995	149.8259464	73.52341079	75.27399902	74.7661061	Ppp2r5c	protein phosphatase 2, regulatory subunit B', gamma [Source:MGI Symbol;Acc:MGI:1349475]
ENSMUSG00000017858	37.0102312	37.48036624	34.87295163	38.5106619	38.36900092	36.07948799	54.02203075	56.00979607	54.7198683	46.69092019	43.96172157	43.21245431	Ift52	intraflagellar transport 52 [Source:MGI Symbol;Acc:MGI:2387217]
ENSMUSG00000017861	21.57973629	17.1952657	16.82970289	25.69944814	22.98261542	32.07563968	530.9084855	508.7203528	575.9275245	458.9265275	489.0611719	474.9200801	Mybl2	myeloblastosis oncogene-like 2 [Source:MGI Symbol;Acc:MGI:101785]
ENSMUSG00000017868	0.794500747	0.625989993	0.299675171	0.995057031	0.922618742	1.369565488	1.30662769	1.801967323	1.32329558	3.160576442	2.871615381	2.705249798	Sgk2	serum/glucocorticoid regulated kinase 2 [Source:MGI Symbol;Acc:MGI:1351318]
ENSMUSG00000017897	0.173922748	0.320449576	0.1068365	0.357064071	0.402014149	0.13247381	0.279493684	0.379049575	0.356136351	0.497823332	0.173727771	0.431576933	Eya2	EYA transcriptional coactivator and phosphatase 2 [Source:MGI Symbol;Acc:MGI:109341]
ENSMUSG00000017922	0	0	0	0	0	0	0	0	0	0	0	0	Prl3c1	prolactin family 3, subfamily c, member 1 [Source:MGI Symbol;Acc:MGI:1351649]
ENSMUSG00000017929	232.3666882	263.1416024	220.6560434	338.620666	355.4204386	361.4688101	54.73901536	51.92140224	60.14850165	60.07457036	61.62963488	59.89058892	B4galt5	UDP-Gal:betaGlcNAc beta 1,4-galactosyltransferase, polypeptide 5 [Source:MGI Symbol;Acc:MGI:1927169]
ENSMUSG00000017943	0.230647046	0.177067953	0.393557923	0.573962756	0.619292032	0.29279947	0.960943342	2.310275248	1.526590648	0.641848159	0.575971196	0.794908674	Gdap1l1	ganglioside-induced differentiation-associated protein 1-like 1 [Source:MGI Symbol;Acc:MGI:2385163]
ENSMUSG00000017950	0.081959024	1.462890461	0.020977262	0.267689852	0.064583312	0.140460088	2.012206642	2.496656794	2.345736018	1.832760303	1.995511634	1.852169656	Hnf4a	hepatic nuclear factor 4, alpha [Source:MGI Symbol;Acc:MGI:109128]
ENSMUSG00000017969	32.86289804	27.81904238	35.22983756	25.73311818	21.91360441	18.04462372	14.29147321	13.86135849	11.52806534	17.92447042	20.4980489	17.39668157	Ptgis	prostaglandin I2 (prostacyclin) synthase [Source:MGI Symbol;Acc:MGI:1097156]
ENSMUSG00000017978	0.0967204	0.111378512	0	0.101540124	0.076215204	0.128922812	0.032381164	0.263492175	0.112529226	0.140585435	0.10063747	0.107144998	Cadps2	Ca2+-dependent activator protein for secretion 2 [Source:MGI Symbol;Acc:MGI:2443963]
ENSMUSG00000017999	70.75410216	63.41538814	67.35731159	74.44584318	75.61455827	65.83877855	172.2162692	174.8003513	162.6512315	141.8157406	139.7435835	140.6612146	Ddx27	DEAD (Asp-Glu-Ala-Asp) box polypeptide 27 [Source:MGI Symbol;Acc:MGI:2385884]
ENSMUSG00000018001	42.1856395	53.50650858	36.28613276	58.28405371	69.79928207	77.23541177	10.84336916	10.8116791	13.17538636	27.16001645	27.21794706	31.02727554	Cyth3	cytohesin 3 [Source:MGI Symbol;Acc:MGI:1335107]

ENSMUSG00000018008	7.640572605	7.1566845	8.346348168	0.375266148	0.537737007	0.612598517	2.023552211	2.679959838	2.143667414	10.20241759	8.977041858	8.90955717	Cyth4	cytohesin 4 [Source:MGI Symbol;Acc:MGI:2441702]
ENSMUSG00000018012	17.5040322	12.54339123	18.30886973	13.24963626	9.907112585	10.03033629	15.67558277	13.52860657	12.05509725	19.38940371	16.82581178	18.53816273	Rac3	Rac family small GTPase 3 [Source:MGI Symbol;Acc:MGI:2180784]
ENSMUSG00000018040	42.0917283	40.45225332	36.82742542	39.77467144	38.54845723	33.05942575	46.35987106	47.0183516	46.58838163	54.07307902	49.0987502	49.20196265	Rrp7a	ribosomal RNA processing 7 homolog A (S. cerevisiae) [Source:MGI Symbol;Acc:MGI:1922028]
ENSMUSG00000018042	250.0183563	226.1271378	257.4309312	229.8123736	219.0174249	188.3127047	140.0641723	137.6647468	130.5529743	101.9756692	101.4373487	97.24960458	Cyb5r3	cytochrome b5 reductase 3 [Source:MGI Symbol;Acc:MGI:94893]
ENSMUSG00000018068	10.09505816	13.47559157	7.670328677	12.56776397	17.66953211	26.01577966	18.61129963	15.8081129	25.49156935	74.364177	74.36570622	69.08454695	Ints2	integrator complex subunit 2 [Source:MGI Symbol;Acc:MGI:1917672]
ENSMUSG00000018076	9.360285825	13.07326136	7.378209393	12.08164742	15.51781095	22.88046406	10.99081145	8.903132722	14.08625329	17.48832521	17.6861261	17.89053124	Med13l	mediator complex subunit 13-like [Source:MGI Symbol;Acc:MGI:2670178]
ENSMUSG00000018102	125.9216041	105.399878	130.9373111	73.95047951	58.93506553	50.83375346	26.55144326	28.65076441	26.08737984	41.95055158	42.26143439	42.32224629	H2bc4	H2B clustered histone 4 [Source:MGI Symbol;Acc:MGI:1915274]
ENSMUSG00000018126	0.520086477	0.239562525	0.81643977	0.420624667	0.072857886	0.316912367	2.012059238	2.026531035	1.419953801	1.581697248	1.125590768	1.720743483	Baiap2l2	BAI1-associated protein 2-like 2 [Source:MGI Symbol;Acc:MGI:2652819]
ENSMUSG00000018143	16.08095197	20.67236961	14.79275377	18.44236979	19.65579059	19.2672434	5.596983826	4.534664649	6.112960223	10.43198348	10.2049932	10.91779279	Mafk	v-maf musculoaponeurotic fibrosarcoma oncogene family, protein K (avian) [Source:MGI Symbol;Acc:MGI:99951]
ENSMUSG00000018160	18.67711581	20.1901368	18.5199902	22.50787834	27.53435128	32.03210066	30.04999544	26.84984226	34.65266147	56.88142497	57.73428078	56.34297667	Med1	mediator complex subunit 1 [Source:MGI Symbol;Acc:MGI:1100846]
ENSMUSG00000018166	1.380401953	1.515477207	1.105699764	1.568495228	1.548024199	2.102854383	4.712460489	4.810995776	5.179228704	5.054104627	5.774994883	5.24834526	Erbb3	erb-b2 receptor tyrosine kinase 3 [Source:MGI Symbol;Acc:MGI:95411]
ENSMUSG00000018167	51.21247762	52.30683917	52.10166469	52.2595513	48.21219332	45.6223078	20.93035238	18.48064161	21.48816602	23.91112861	27.45762132	27.14277352	Stard3	START domain containing 3 [Source:MGI Symbol;Acc:MGI:1929618]
ENSMUSG00000018168	0.035125296	0.020224292	0.01798051	0	0	0.013377151	0	0.191381357	0.081732962	0.125674992	0.146191328	0.233466763	Ikzf3	IKAROS family zinc finger 3 [Source:MGI Symbol;Acc:MGI:1342542]
ENSMUSG00000018169	1.14488216	0.494396497	0.830253053	0.53419261	0.551320955	0.399683178	18.05753673	19.28087124	18.60376329	9.472648043	12.42869404	11.32995394	Mfng	MFNG O-fucosylpeptide 3-beta-N-acetylglucosaminyltransferase [Source:MGI Symbol;Acc:MGI:1095404]
ENSMUSG00000018171	421.8508906	405.3522581	382.6134407	433.4407695	419.2912343	376.5165657	63.75422964	67.54095586	74.22254725	155.5781176	149.6994056	157.4548811	Vmp1	vacuole membrane protein 1 [Source:MGI Symbol;Acc:MGI:1923159]
ENSMUSG00000018189	32.21283964	26.20355233	28.32957548	38.84270054	38.61646344	31.02654837	115.7879715	130.0575375	124.0259905	82.33660706	76.85623335	77.09568761	Uchl5	ubiquitin carboxyl-terminal esterase L5 [Source:MGI Symbol;Acc:MGI:1914848]
ENSMUSG00000018196	15.3248268	13.8308062	16.76247572	12.79412987	11.78571038	9.687646653	15.80598011	14.84468297	14.4482785	13.75837113	14.9492423	13.68165441	Glrx2	glutaredoxin 2 (thioltransferase) [Source:MGI Symbol;Acc:MGI:1916617]
ENSMUSG00000018199	5.608118652	6.996206658	5.060920896	7.217429472	7.524346086	10.56369305	4.824628299	4.121085474	5.262692544	8.107377955	7.478971245	7.933417527	Ro60	Ro60, Y RNA binding protein [Source:MGI Symbol;Acc:MGI:106652]
ENSMUSG00000018209	28.35135684	33.09223589	25.6296821	31.60444064	35.07646753	39.00270049	33.21481556	30.87555799	39.65422488	54.39379559	56.36622582	54.45604408	Stk4	serine/threonine kinase 4 [Source:MGI Symbol;Acc:MGI:1929004]
ENSMUSG00000018211	0	0.7536048	0	0.152674821	0.25784212	0	0	0	0	0.073171039	0	0	Wfdc15b	WAP four-disulfide core domain 15B [Source:MGI Symbol;Acc:MGI:2445041]
ENSMUSG00000018217	82.47454895	90.21366523	78.47184916	40.3004256	40.20355484	38.83106745	2.600822691	2.768925148	3.774073748	27.00848143	25.92053843	26.93376251	Pmp22	peripheral myelin protein 22 [Source:MGI Symbol;Acc:MGI:97631]
ENSMUSG00000018238	0.247859953	1.427117385	0.507514403	1.063974643	1.45833285	0.679645589	0.840878142	0.540189314	0.738233208	0.26604585	1.15538308	0.439319178	Gdf9	growth differentiation factor 9 [Source:MGI Symbol;Acc:MGI:95692]
ENSMUSG00000018239	18.39585777	16.95909122	16.36533046	23.18239768	18.88727551	18.36303227	66.67620835	74.66013276	77.07388478	92.25860879	80.14080421	90.4785607	Zcchc10	zinc finger, CCHC domain containing 10 [Source:MGI Symbol;Acc:MGI:1196228]
ENSMUSG00000018259	0.055808674	0.128533114	0.171409534	0	0	0	0	0	0	0	0	0	Prl8a2	prolactin family 8, subfamily a, member 2 [Source:MGI Symbol;Acc:MGI:894281]
ENSMUSG00000018263	0.402762757	0.566869134	0.526885988	0.146164506	0.11754633	0.323820277	0.099882616	0.044332655	0	0.440319948	0.540140088	0.376768169	Tbx5	T-box 5 [Source:MGI Symbol;Acc:MGI:102541]
ENSMUSG00000018286	255.014397	201.1952661	290.6768169	232.6524546	197.2409256	145.7326143	402.3424484	462.8497774	346.9380166	399.7398824	397.9038213	413.9653167	Psmb6	proteasome (prosome, macropain) subunit, beta type 6 [Source:MGI Symbol;Acc:MGI:104880]
ENSMUSG00000018287	105.3639486	71.78248741	105.5838234	96.73563449	85.89021669	67.53311793	130.676982	146.7404028	117.4247063	96.19612856	95.27554516	91.86777656	Spag7	sperm associated antigen 7 [Source:MGI Symbol;Acc:MGI:107380]
ENSMUSG00000018293	1139.433773	869.9060477	1397.229768	990.886683	809.2634086	626.9282364	715.6232787	757.34092	544.9436076	605.4876923	618.7980414	621.0423829	Pfn1	profilin 1 [Source:MGI Symbol;Acc:MGI:97549]
ENSMUSG00000018322	35.03602287	28.43506645	33.75595229	30.5395335	28.4994957	28.57022702	37.99511932	42.05885305	40.37258885	39.01820439	38.21601061	41.71975273	Tomm34	translocase of outer mitochondrial membrane 34 [Source:MGI Symbol;Acc:MGI:1914395]
ENSMUSG00000018326	176.1471757	189.3966726	168.0875088	185.5668753	188.1143645	187.0037076	122.1278038	117.9440209	132.6744807	131.0703609	137.0167059	135.5090585	Ywhab	tyrosine 3-monooxygenase/tryptophan 5-monooxygenase activation protein, beta polypeptide [Source:MGI Symbol;Acc:MGI:18919
ENSMUSG00000018334	10.02409831	11.50105878	9.924889418	10.41459455	10.89774625	12.42286131	4.842758435	4.266215592	4.758821667	11.35952329	12.31014417	11.43337517	Ksr1	kinase suppressor of ras 1 [Source:MGI Symbol;Acc:MGI:105051]
ENSMUSG00000018339	85.56001526	113.9781928	82.25347968	39.99889274	25.93277934	21.76523026	42.55582005	47.79510353	35.15382694	81.19886649	80.18291854	85.33109086	Gpx3	glutathione peroxidase 3 [Source:MGI Symbol;Acc:MGI:105102]
ENSMUSG00000018340	210.9530158	206.1824445	220.7856825	185.8976276	174.2980573	160.9856549	66.19702532	64.10188066	67.51483239	101.1781871	105.9887132	101.3472735	Anxa6	annexin A6 [Source:MGI Symbol;Acc:MGI:88255]
ENSMUSG00000018341	0	0.053107734	0.078692837	0.100419559	0.048454789	0.105382703	0.096071446	0.182747471	0.028616757	0.082503679	0.038388934	0.177108882	Il12rb2	interleukin 12 receptor, beta 2 [Source:MGI Symbol;Acc:MGI:1270861]
ENSMUSG00000018347	7.641979903	8.063175771	9.076382259	9.818807572	8.523528088	7.111073594	4.453165498	4.642972912	4.449905725	6.465170204	6.314173586	6.121949265	Zkscan6	zinc finger with KRAB and SCAN domains 6 [Source:MGI Symbol;Acc:MGI:1289293]
ENSMUSG00000018362	173.1570662	188.8387095	170.9138313	196.4275688	217.045593	203.5299787	351.3213018	325.3773041	336.3319654	500.6545767	462.0738723	476.1990916	Kpna2	karyopherin (importin) alpha 2 [Source:MGI Symbol;Acc:MGI:103561]
ENSMUSG00000018363	19.95204151	27.06256102	15.76771544	22.9131527	26.56479322	36.25318857	16.08451467	13.99554273	19.8665286	36.25523904	35.40697438	35.78533834	Smurf2	SMAD specific E3 ubiquitin protein ligase 2 [Source:MGI Symbol;Acc:MGI:1913563]
ENSMUSG00000018372	15.62407457	15.40994034	14.53833567	16.70570666	18.13567775	19.49091881	13.85349576	15.37210058	13.81347906	21.71116052	20.97464668	20.21769388	Cep95	centrosomal protein 95 [Source:MGI Symbol;Acc:MGI:2443502]
ENSMUSG00000018377	1.053180215	1.609280573	0.912355382	1.578166815	2.425864105	4.412035727	0.714232401	0.822621678	1.272448919	2.889541368	2.958748716	2.360309234	Vezf1	vascular endothelial zinc finger 1 [Source:MGI Symbol;Acc:MGI:1313291]
ENSMUSG00000018378	20.89415919	23.16036257	18.43790923	21.58626816	23.00072337	24.08767306	2.719848691	2.457129065	1.95728936	5.642972513	7.697486397	7.287303214	Cuedc1	CUE domain containing 1 [Source:MGI Symbol;Acc:MGI:2144281]
ENSMUSG00000018379	12.79025497	15.09756146	10.34445738	14.46537088	16.72655994	20.76063392	21.07756598	15.8687291	27.15818752	40.84662181	45.19162467	41.21236606	Srsf1	serine and arginine-rich splicing factor 1 [Source:MGI Symbol;Acc:MGI:98283]
ENSMUSG00000018381	0.150186694	0.230596931	0.128133363	0.046717252	0.131495981	0.133460235	0.268166521	0.024796886	0.093191743	0.58213329	0.229194666	0.399297431	Abi3	ABI gene family, member 3 [Source:MGI Symbol;Acc:MGI:1913860]
ENSMUSG00000018387	3.216207726	2.66085933	2.813206108	2.010587787	2.772208201	1.950268282	0.362404284	0.386664076	0.494075164	1.18703815	1.58550755	1.106905796	Shroom1	shroom family member 1 [Source:MGI Symbol;Acc:MGI:1919024]
ENSMUSG00000018395	16.34436302	16.92993462	16.48513493	19.39830093	22.2080913	23.93446939	21.68347833	22.27320578	21.25261608	34.58618255	32.49966799	32.69216485	Kif3a	kinesin family member 3A [Source:MGI Symbol;Acc:MGI:107689]
ENSMUSG00000018398	55.21595736	64.00232917	48.73826415	59.7302051	60.07420269	70.23794967	17.07507021	14.59247045	18.62987124	32.31429511	33.03498224	33.91558893	Septin8	septin 8 [Source:MGI Symbol;Acc:MGI:894310]
ENSMUSG00000018401	15.07171112	20.21187609	13.08646314	17.54318046	20.2142242	25.64242952	11.84414327	9.726020502	12.85342823	26.54251978	28.57287577	27.18205007	Mtmr4	myotubularin related protein 4 [Source:MGI Symbol;Acc:MGI:2180699]
ENSMUSG00000018405	2.623688282	3.021311928	2.810087255	26.48020161	2.88383269	3.597148606	1.909931769	1.359548613	1.953617147	2.094091979	2.923140869	17.52825825	Mrm1	mitochondrial rRNA methyltransferase 1 [Source:MGI Symbol;Acc:MGI:2443470]
ENSMUSG00000018411	4.24093729	4.990598696	4.753839949	4.679757848	6.114506367	6.224706078	29.79223934	25.51417647	29.93602045	20.48999649	21.41282268	19.72486221	Mapt	microtubule-associated protein tau [Source:MGI Symbol;Acc:MGI:97180]
ENSMUSG00000018412	15.86090709	20.5233912	12.47589271	18.73548442	20.77427047	24.1619319	18.16578769	15.36629983	22.15976107	38.68963243	40.36788009	38.64240905	Kansl1	KAT8 regulatory NSL complex subunit 1 [Source:MGI Symbol;Acc:MGI:1923969]
ENSMUSG00000018415	13.20974576	16.03752656	14.00861556	15.74016331	17.35999425	18.4189996	18.46473986	15.27426209	19.39713905	29.28934241	28.90421851	28.87931982	Gid4	GID complex subunit 4, VID24 homolog [Source:MGI Symbol;Acc:MGI:1914021]
ENSMUSG00000018417	58.49322553	72.47503282	48.87187266	80.04008695	96.40280973	105.6610297	31.05004309	26.4995045	34.99182002	55.56749337	57.35094785	56.82894538	Myo1b	myosin IB [Source:MGI Symbol;Acc:MGI:107752]
ENSMUSG00000018425	45.35512836	54.57723684	36.55336916	48.14128584	53.2963219	60.45034648	38.49178636	33.09336507	39.9422112	80.57293895	76.29616132	80.82241147	Dhx40	DEAH (Asp-Glu-Ala-His) box polypeptide 40 [Source:MGI Symbol;Acc:MGI:1914737]
ENSMUSG00000018427	3.438386513	4.314337772	3.453776118	3.314591649	3.101362209	3.211928144	4.06881794	3.984625039	4.815559493	6.383228497	6.412746269	6.295594517	Ypel2	yippee like 2 [Source:MGI Symbol;Acc:MGI:1925114]
ENSMUSG00000018428	14.59746847	16.84798273	13.32958864	17.15549979	18.16358912	19.8718589	45.96142324	40.89843975	51.62646317	80.88266187	85.33291097	85.05212512	Akap1	A kinase (PRKA) anchor protein 1 [Source:MGI Symbol;Acc:MGI:104729]
ENSMUSG00000018433	20.25754569	22.83285218	16.67079663	23.795396	25.70757594	33.21580724	54.10299717	53.86221694	66.78191018	91.07986051	88.42458774	92.77166478	Nol11	nucleolar protein 11 [Source:MGI Symbol;Acc:MGI:1916229]
ENSMUSG00000018442	39.78898673	38.35815951	38.73559931	39.96886414	39.93401454	40.72266242	19.29573705	21.36191908	22.41831815	33.15716183	35.77570666	33.88130497	Derl2	Der1-like domain family, member 2 [Source:MGI Symbol;Acc:MGI:2151483]
ENSMUSG00000018446	117.4957469	105.3118962	118.0068405	113.4056042	106.0226042	89.95098853	233.3434789	241.9643708	216.5196001	269.9221852	260.1713439	256.9543872	C1qbp	complement component 1, q subcomponent binding protein [Source:MGI Symbol;Acc:MGI:1194505]
ENSMUSG00000018449	50.22437828	37.54263641	54.4263296	49.47237372	40.42518569	35.73822387	43.85586583	38.62512821	38.68228628	30.7378298	33.00531187	38.00275826	Rpain	RPA interacting protein [Source:MGI Symbol;Acc:MGI:1916973]
ENSMUSG00000018451	1.963490318	1.22743259	1.924055256	2.277283293	2.387140669	2.051046924	0.751268333	1.000345839	0.417722571	4.039480173	4.086019041	3.181885137	6330403K07	RIKEN cDNA 6330403K07 gene [Source:MGI Symbol;Acc:MGI:1918001]
ENSMUSG00000018459	0.613070803	1.464260092	0.41843826	0.317837555	0.166996427	0.190244975	0.283803671	0.337407765	0.105670584	11.63778614	11.03802389	12.37558648	Slc13a3	solute carrier family 13 (sodium-dependent dicarboxylate transporter), member 3 [Source:MGI Symbol;Acc:MGI:2149635]
ENSMUSG00000018470	3.265874145	3.898078237	4.19777675	4.582612983	4.157662518	3.250166035	6.703977303	6.376165584	6.501053405	2.601402713	2.599451905	3.084434396	Kcnab3	potassium voltage-gated channel, shaker-related subfamily, beta member 3 [Source:MGI Symbol;Acc:MGI:1336208]
ENSMUSG00000018474	72.21195793	87.32368699	70.62030799	72.63369	78.71305408	93.34372966	10.58800592	8.737515535	13.03355401	29.01134582	31.83884766	29.94266056	Chd3	chromodomain helicase DNA binding protein 3 [Source:MGI Symbol;Acc:MGI:1344395]
ENSMUSG00000018476	15.78525493	17.2398558	16.02982665	16.64106016	21.70585926	21.67214233	14.34196093	13.05625451	15.5042944	15.36813364	15.34732296	16.05107667	Kdm6b	KDM1 lysine (K)-specific demethylase 6B [Source:MGI Symbol;Acc:MGI:2448492]
ENSMUSG00000018479	0.05202961	0	0.532675335	0	0.273327197	0.356670116	0.092901703	0.206170968	0.242135136	0	0.173237608	0.414989289	1700125H20	RIKEN cDNA 1700125H20 gene [Source:MGI Symbol;Acc:MGI:1920884]
ENSMUSG00000018481	11.21816525	13.16362654	8.806360398	13.25195238	14.25569438	16.46054015	14.84764126	12.70267409	17.93939249	30.78799105	29.38280579	30.33285827	Appbp2	amyloid beta precursor protein (cytoplasmic tail) binding protein 2 [Source:MGI Symbol;Acc:MGI:1914134]
ENSMUSG00000018486	0.028571373	0.04386854	0.009750387	0.017774891	0.010006268	0	0	0.132085407	0	0.212970155	0.063420762	0.211005449	Wnt9b	wingless-type MMTV integration site family, member 9B [Source:MGI Symbol;Acc:MGI:1197020]
ENSMUSG00000018500	11.47426342	10.20532029	10.74445127	11.6651838	11.58999164	10.35623525	2.790021922	1.732758037	1.823376004	10.24259701	10.85183382	11.69822442	Adora2b	adenosine A2b receptor [Source:MGI Symbol;Acc:MGI:99403]

ENSMUSG00000018501	30.84221761	39.95102155	26.54700042	39.35488042	47.83378155	64.14008812	53.80218822	44.3044312	68.15046548	115.662922	124.3332825	118.8430025	Ncor1	nuclear receptor co-repressor 1 [Source:MGI Symbol;Acc:MGI:1349717]
ENSMUSG00000018507	3.728463816	3.133107545	2.372837833	0.752289983	1.1646171	0.828944784	3.076786907	3.034720773	2.438590022	6.219366931	7.230479312	5.894078945	Trpv2	transient receptor potential cation channel, subfamily V, member 2 [Source:MGI Symbol;Acc:MGI:1341836]
ENSMUSG00000018509	15.11621713	10.03944434	15.61983381	13.31888235	10.26790237	11.40664444	112.6710181	127.3899926	88.08195743	36.47552369	34.35376782	35.39132331	Cenpv	centromere protein V [Source:MGI Symbol;Acc:MGI:1920389]
ENSMUSG00000018537	35.4999903	33.23861007	39.63483922	32.54486585	29.63864854	27.56100214	67.47837437	67.2698371	63.36961181	56.50757442	56.12796669	58.28058047	Pcgf2	polycomb group ring finger 2 [Source:MGI Symbol;Acc:MGI:99161]
ENSMUSG00000018541	15.37948921	19.35374645	13.12844288	17.83052951	20.25422183	21.31933818	13.42618167	12.59783717	14.86973448	27.0749506	27.3236659	26.33435745	Cwc25	CWC25 spliceosome-associated protein [Source:MGI Symbol;Acc:MGI:1914730]
ENSMUSG00000018543	1.31016229	0.6352502	0.070596555	0.321742611	0.796941509	0.157567407	0.554060395	0.341553267	0.834357323	0.5551149	1.0331791	1.344429332	1700001P01	RIKEN cDNA 1700001P01 gene [Source:MGI Symbol;Acc:MGI:1919465]
ENSMUSG00000018547	29.42445438	36.22483238	31.86159197	34.06270162	38.75228141	41.27314499	39.9841998	35.22121554	45.7541994	53.3499271	57.72687888	52.61392604	Pip4k2b	phosphatidylinositol-5-phosphate 4-kinase, type II, beta [Source:MGI Symbol;Acc:MGI:1934234]
ENSMUSG00000018548	13.34647765	19.21144386	13.2014417	18.53611679	21.80078033	25.78505339	41.23856305	37.97763329	48.73509432	61.88230781	61.44995438	64.22221832	Trim37	tripartite motif-containing 37 [Source:MGI Symbol;Acc:MGI:2153072]
ENSMUSG00000018554	1.784591661	2.23772006	1.745849547	2.590546957	2.583332477	1.751975297	21.95134518	22.27544299	22.29464288	13.30229252	13.40244373	13.28501196	Ybx2	Y box protein 2 [Source:MGI Symbol;Acc:MGI:1096372]
ENSMUSG00000018559	180.8533097	136.3351666	197.208819	158.0713375	131.3579812	108.1111959	59.71054378	61.01047257	47.17857037	43.86800468	47.37070628	45.69570299	Ctdnep1	CTD nuclear envelope phosphatase 1 [Source:MGI Symbol;Acc:MGI:1914431]
ENSMUSG00000018565	106.0021956	81.52089653	99.92074056	90.13438288	78.62559199	64.77831767	87.75111288	93.41857599	80.49433915	103.2371904	110.7711032	103.4631963	Elp5	elongator acetyltransferase complex subunit 5 [Source:MGI Symbol;Acc:MGI:1859017]
ENSMUSG00000018566	0.80217166	0.84984255	0.952658655	0.389258174	0.53939828	0.684319596	5.156356251	3.782606917	3.94219836	1.922963271	2.457235305	2.360202155	Slc2a4	solute carrier family 2 (facilitated glucose transporter), member 4 [Source:MGI Symbol;Acc:MGI:95758]
ENSMUSG00000018567	602.4975188	507.1438382	695.5910929	503.4840902	437.4112178	341.6841991	108.548395	113.8481643	92.95812234	148.8760442	145.6908914	145.3598339	Gabarap	gamma-aminobutyric acid receptor associated protein [Source:MGI Symbol;Acc:MGI:1861742]
ENSMUSG00000018569	0.678847502	0.360797444	0.534614687	0.341109642	0.54864464	0.676163047	18.69460738	16.91584005	14.24077839	8.500982864	8.693416378	10.04228194	Cldn7	claudin 7 [Source:MGI Symbol;Acc:MGI:1859285]
ENSMUSG00000018570	4.637079864	3.987539746	4.069207156	4.074355694	3.037087867	3.394370227	7.930288279	9.545326287	8.043461502	3.124285584	3.809772619	3.575793722	2810408A11	RIKEN cDNA 2810408A11 gene [Source:MGI Symbol;Acc:MGI:1917669]
ENSMUSG00000018572	86.22624587	82.10171511	98.15576126	86.49899474	80.88411953	63.65566243	75.74163989	72.07155524	65.8873138	82.39634658	80.59501485	79.02601963	Phf23	PHD finger protein 23 [Source:MGI Symbol;Acc:MGI:1925496]
ENSMUSG00000018574	43.5306156	53.06335021	34.32437448	49.48898121	48.9418599	45.80713656	21.37990083	19.43799877	19.23365894	42.53023932	40.57531136	41.38481308	Acadvl	acyl-Coenzyme A dehydrogenase, very long chain [Source:MGI Symbol;Acc:MGI:895149]
ENSMUSG00000018581	0.065282601	0.016705838	0.014852411	0.081227534	0.129558566	0.03867467	0.064758689	0.035928732	0.074265116	0.142740266	0.169061449	0.173565143	Dnah11	dynein, axonemal, heavy chain 11 [Source:MGI Symbol;Acc:MGI:1100864]
ENSMUSG00000018583	75.19369789	79.7165864	69.74939615	83.34488327	87.33467467	88.10713278	165.0217045	160.3260023	172.159898	226.1273529	224.0321997	228.0029926	G3bp1	GTPase activating protein (SH3 domain) binding protein 1 [Source:MGI Symbol;Acc:MGI:1351465]
ENSMUSG00000018585	229.3200284	171.0257475	316.4859818	208.2614714	187.8124377	133.7391487	327.0573405	334.0334286	205.5056692	212.9253623	198.0656709	212.7183462	Atox1	antioxidant 1 copper chaperone [Source:MGI Symbol;Acc:MGI:1333855]
ENSMUSG00000018589	0.013659837	0	0.013984841	0	0.043055541	0	0	0	0.012714016	0.012218402	0.022740873	0	Glra2	glycine receptor, alpha 2 subunit [Source:MGI Symbol;Acc:MGI:95748]
ENSMUSG00000018593	1657.317836	1632.507853	1732.218256	755.1545337	718.1533408	650.3714462	147.0124189	144.3232131	148.1163086	436.2969469	439.5453659	433.4014156	Sparc	secreted acidic cysteine rich glycoprotein [Source:MGI Symbol;Acc:MGI:98373]
ENSMUSG00000018595	0	0.088009797	0	0	0	0.019404394	0	0	0	0.227874283	0.042411931	0.045154416	Glra4	glycine receptor, alpha 4 subunit [Source:MGI Symbol;Acc:MGI:95750]
ENSMUSG00000018599	16.05486983	15.46871356	17.4664598	16.59096121	14.79270517	13.02206496	14.07689561	12.95142618	13.07113376	16.25618613	16.41357649	16.26537086	Mief2	mitochondrial elongation factor 2 [Source:MGI Symbol;Acc:MGI:2144199]
ENSMUSG00000018604	6.871938923	7.01306501	6.024359367	7.411177452	8.704438726	9.914741978	34.01863711	31.59231619	34.59776877	39.98478878	38.8196929	41.22049085	Tbx3	T-box 3 [Source:MGI Symbol;Acc:MGI:98495]
ENSMUSG00000018620	0	0	0	0	0	0	0	0	0	0.011709147	0.021793049	0.011601127	Mmp20	matrix metallopeptidase 20 (enamelysin) [Source:MGI Symbol;Acc:MGI:1353466]
ENSMUSG00000018623	0	0	0	0	0.042329886	0	0	0	0	0	0	0	Mmp7	matrix metallopeptidase 7 [Source:MGI Symbol;Acc:MGI:103189]
ENSMUSG00000018634	0	0	0.017907419	0.032645104	0	0	0	0.01732758	0	0.062582059	0.043679116	0.046503542	Crhr1	corticotropin releasing hormone receptor 1 [Source:MGI Symbol;Acc:MGI:88498]
ENSMUSG00000018648	28.77723288	27.28584659	31.00884651	40.92257877	39.79630897	36.27898374	4.721378713	6.803806496	4.474765533	7.863463201	8.06094085	8.33871978	Dusp14	dual specificity phosphatase 14 [Source:MGI Symbol;Acc:MGI:1927168]
ENSMUSG00000018651	38.27270759	35.10192002	32.08858646	45.43683018	42.35305614	41.39876291	104.1244729	102.660989	109.1444771	103.9985365	103.2280053	97.8610754	Tada2a	transcriptional adaptor 2A [Source:MGI Symbol;Acc:MGI:2144471]
ENSMUSG00000018654	0.536118676	0.548771747	0.528545688	0.018529524	0.041724336	0.10586901	0.248180875	0.137693094	0.166332424	1.172222326	1.520605696	1.460558878	Ikzf1	IKAROS family zinc finger 1 [Source:MGI Symbol;Acc:MGI:1342540]
ENSMUSG00000018656	0	0	0.058893383	0.05368112	0	0.065723303	0	0	0	0.02572725	0	0	Tcaf3	TRPM8 channel-associated factor 3 [Source:MGI Symbol;Acc:MGI:3042585]
ENSMUSG00000018659	6.052034309	4.872793989	6.800518898	6.313439911	6.953137585	4.947021983	16.18739134	19.93593106	15.38766714	13.00536771	13.65915148	13.03800858	Pnpo	pyridoxine 5'-phosphate oxidase [Source:MGI Symbol;Acc:MGI:2144151]
ENSMUSG00000018661	30.65519084	37.98262547	30.67289149	40.28321444	41.08187249	41.32491617	21.65877879	18.76501373	21.48032036	36.74699213	37.17664377	34.89869226	Cog1	component of oligomeric golgi complex 1 [Source:MGI Symbol;Acc:MGI:1333873]
ENSMUSG00000018666	57.89069532	59.83207527	51.9976073	64.22878732	67.52913848	66.80331365	240.7616202	235.3029908	228.3024752	246.3390661	244.732021	250.8380346	Cbx1	chromobox 1 [Source:MGI Symbol;Acc:MGI:105369]
ENSMUSG00000018669	92.98745344	72.56164245	105.4293972	87.96468415	72.68175292	59.55674303	127.5054297	139.6291355	108.7896418	93.5957364	97.11611296	97.96991323	Cdk5rap3	CDK5 regulatory subunit associated protein 3 [Source:MGI Symbol;Acc:MGI:1933126]
ENSMUSG00000018672	149.3796742	112.2574088	176.2090008	95.91294083	81.22490392	58.97510491	11.84856495	11.55555484	7.921201477	18.0462249	15.60486276	19.14556625	Copz2	coatomer protein complex, subunit zeta 2 [Source:MGI Symbol;Acc:MGI:1929008]
ENSMUSG00000018677	252.59316	210.3965271	289.5547687	232.6977147	198.0203138	153.7308637	290.1200254	314.8183523	265.2056559	171.4349613	181.2692848	181.1105277	Slc25a39	solute carrier family 25, member 39 [Source:MGI Symbol;Acc:MGI:1196386]
ENSMUSG00000018678	5.983067832	6.657769931	5.823910599	8.461737944	8.875552414	9.858179839	17.74057742	16.29179554	20.39967588	18.35665255	18.06326573	18.88787606	Sp2	Sp2 transcription factor [Source:MGI Symbol;Acc:MGI:1926162]
ENSMUSG00000018697	45.68222717	35.36132833	44.63443853	42.54146	37.87275421	34.69907324	45.60706243	53.54832997	42.25450128	52.36284999	51.54264019	48.38007291	Aatf	apoptosis antagonizing transcription factor [Source:MGI Symbol;Acc:MGI:1929608]
ENSMUSG00000018698	5.083417219	4.990138436	3.839285847	5.002983358	4.115153575	4.760590473	0.024799777	0.137591503	0.1809839	17.24380159	17.27256681	18.66025936	Lhx1	LIM homeobox protein 1 [Source:MGI Symbol;Acc:MGI:99783]
ENSMUSG00000018707	174.8750386	195.7227206	173.1166163	191.481249	215.5280293	286.4127592	149.2422044	134.7248477	176.4094409	178.2679063	198.0879909	184.1283925	Dync1h1	dynein cytoplasmic 1 heavy chain 1 [Source:MGI Symbol;Acc:MGI:103147]
ENSMUSG00000018727	1.664684979	1.916970327	1.444943446	1.78985225	1.48286326	1.846811928	7.560198362	8.783290737	6.770293908	14.53414373	16.71855836	12.37956368	Cpsf4l	cleavage and polyadenylation specific factor 4-like [Source:MGI Symbol;Acc:MGI:1277182]
ENSMUSG00000018733	2.637061451	2.104651756	1.710766998	1.43753349	1.892824009	1.78984336	0.675989544	0.750090767	1.312290159	2.148600064	2.173354095	2.105639761	Pex12	peroxisomal biogenesis factor 12 [Source:MGI Symbol;Acc:MGI:2144177]
ENSMUSG00000018736	68.22448479	82.17698685	59.29354212	73.46532887	71.37801454	79.52089382	28.46815079	27.46576199	30.27525145	53.60823887	52.52608748	56.03607646	Ndel1	nudE neurodevelopment protein 1 like 1 [Source:MGI Symbol;Acc:MGI:1932915]
ENSMUSG00000018740	0.968825472	0.848865906	0.603750859	0.845129489	0.442567972	0.994610514	2.632442276	2.858414652	2.626816595	2.618613473	3.103077318	2.59445607	Slc25a35	solute carrier family 25, member 35 [Source:MGI Symbol;Acc:MGI:1919248]
ENSMUSG00000018750	30.477763	34.78612045	26.71116383	32.11607045	38.82285592	43.79912996	1.605301048	1.977212325	2.376506275	15.52063834	15.01225495	15.64835454	Zbtb4	zinc finger and BTB domain containing 4 [Source:MGI Symbol;Acc:MGI:1922830]
ENSMUSG00000018752	0.325767731	0.15630762	0.333518613	0.152000547	0.228180787	0.103388078	0.266601511	0.295826072	0.101070414	0.145695772	0.112986989	0.336820609	Tnfsfm13	tumor necrosis factor (ligand) superfamily, membrane-bound member 13 [Source:MGI Symbol;Acc:MGI:3845075]
ENSMUSG00000018761	68.86834692	55.25164242	66.66107702	61.3987042	55.19481195	49.29108659	157.6228536	152.4607039	143.2975594	152.8318453	172.356869	167.4619624	Mpdu1	mannose-P-dolichol utilization defect 1 [Source:MGI Symbol;Acc:MGI:1346040]
ENSMUSG00000018765	188.5302548	174.2850354	184.7321535	177.5482042	156.8532089	146.9487338	266.8162147	269.0061649	248.087081	241.9721712	248.8883532	246.2473769	Fxr2	fragile X mental retardation, autosomal homolog 2 [Source:MGI Symbol;Acc:MGI:1346074]
ENSMUSG00000018770	338.3755824	291.3922292	415.5291269	326.7931363	297.0163092	222.4133343	501.7815913	509.0807199	373.8785428	296.3373991	299.7111601	302.4453049	Atp5g3	ATP synthase, H+ transporting, mitochondrial F0 complex, subunit C3 (subunit 9) [Source:MGI Symbol;Acc:MGI:2442035]
ENSMUSG00000018774	36.50627494	30.14696845	39.32242965	9.510034224	7.947146489	7.865764953	23.77677284	20.55014552	20.90987279	31.68167337	33.25314123	30.82744295	Cd68	CD68 antigen [Source:MGI Symbol;Acc:MGI:88342]
ENSMUSG00000018776	0	0	0.084933644	0	0	0.063188986	0.074064629	0.164367022	0.386077939	0.074205591	0.230185575	0.098028033	Slc35g3	solute carrier family 35, member G3 [Source:MGI Symbol;Acc:MGI:1927128]
ENSMUSG00000018796	13.02177921	19.36059449	12.47475049	17.38912055	18.466098	20.71813089	34.12944415	29.0675117	35.97091985	30.60541948	29.99765772	29.14505759	Acsl1	acyl-CoA synthetase long-chain family member 1 [Source:MGI Symbol;Acc:MGI:102797]
ENSMUSG00000018800	6.351445655	7.209343308	4.21096239	7.46449654	9.299519407	12.78247385	1.470861854	1.643351931	1.924728169	4.319351917	4.993744455	4.772906475	Abca5	ATP-binding cassette, sub-family A (ABC1), member 5 [Source:MGI Symbol;Acc:MGI:2386607]
ENSMUSG00000018809	9.158191757	9.405072051	10.85167084	10.05938489	12.14598923	12.46464846	14.47593943	14.01031003	16.71276526	18.47852119	20.87026589	20.43689519	Smyd4	SET and MYND domain containing 4 [Source:MGI Symbol;Acc:MGI:2442796]
ENSMUSG00000018819	170.13563	142.2317897	205.6446806	136.6756694	122.3222775	97.79466388	1.089156782	1.273875908	0.644469105	8.759330278	9.139478571	8.21099327	Lsp1	lymphocyte specific 1 [Source:MGI Symbol;Acc:MGI:96832]
ENSMUSG00000018820	21.22605078	24.21202307	20.49956197	26.12197714	28.0412691	26.85675424	23.22342372	22.11976139	25.44765539	24.61257255	26.42980852	27.05059867	Zfyve27	zinc finger, FYVE domain containing 27 [Source:MGI Symbol;Acc:MGI:1919602]
ENSMUSG00000018821	43.51135116	33.79499382	51.41260123	46.91247972	37.09111406	27.95190787	68.25581927	81.90328093	59.92391749	34.88871765	35.23630268	36.46875049	Avpi1	arginine vasopressin-induced 1 [Source:MGI Symbol;Acc:MGI:1916784]
ENSMUSG00000018822	0.09058629	0.182550847	0.278224738	0.105667047	0.237938518	0.086247423	0.849170458	0.515997104	0.695590526	2.876469066	1.357271071	1.384826539	Sfrp5	secreted frizzled-related sequence protein 5 [Source:MGI Symbol;Acc:MGI:1860298]
ENSMUSG00000018830	0.378019565	0.558331058	0.294466913	0.199387018	0.172682652	0.212818316	0.110050193	0.113972855	0.09178568	0.962934224	0.950829964	1.325475297	Myh11	myosin, heavy polypeptide 11, smooth muscle [Source:MGI Symbol;Acc:MGI:102643]
ENSMUSG00000018841	9.405133951	8.615166651	9.344416709	9.714229362	10.2858469	10.28970742	14.7896234	13.31925395	14.16542269	18.10636073	19.03827956	18.39396446	Rad51d	RAD51 paralog D [Source:MGI Symbol;Acc:MGI:1261809]
ENSMUSG00000018844	0.019865415	0.11438023	0.081352263	0.037076157	0.0417436	0.04539338	0.035470779	0.177115711	0.018489013	0.017769144	0.082679768	0.123236534	Fndc8	fibronectin type III domain containing 8 [Source:MGI Symbol;Acc:MGI:1926169]
ENSMUSG00000018845	0.881731308	0.426450749	0.722168036	0.427864928	0.296447989	0.40295927	0.330619646	0.174696097	0.361098907	1.766660751	1.70276949	1.390913327	Unc45b	unc-45 myosin chaperone B [Source:MGI Symbol;Acc:MGI:2443377]
ENSMUSG00000018846	5.991086903	7.846235663	4.718177556	7.017059779	9.009792296	13.56549417	5.952844117	5.657633527	8.008744373	13.11283345	13.26622882	13.43647225	Pank3	pantothenate kinase 3 [Source:MGI Symbol;Acc:MGI:2387464]
ENSMUSG00000018848	215.3222865	207.362393	197.4756043	219.1868006	218.4360126	210.5482538	242.290553	244.3525606	241.5503684	329.0178191	323.6992226	325.0660778	Rars	arginyl-tRNA synthetase [Source:MGI Symbol;Acc:MGI:1914297]
ENSMUSG00000018849	10.47570232	9.073936924	11.35997696	13.18440409	13.20285645	12.4761755	26.0474451	27.76483406	29.42212093	29.15879567	31.74373374	31.59758039	Wwc1	WW, C2 and coiled-coil domain containing 1 [Source:MGI Symbol;Acc:MGI:2388637]

ENSMUSG00000018858	32.45215784	24.35837533	35.7693166	24.4632533	26.30820206	19.41654164	31.75681739	33.35741233	25.87208843	25.59126335	29.721987	25.11484374	Mrpl58	mitochondrial ribosomal protein L58 [Source:MGI Symbol;Acc:MGI:1915822]
ENSMUSG00000018861	14.40195058	12.66310787	17.75890383	13.67138198	10.17466687	10.83461028	26.76055374	31.41591743	26.87552295	20.24346459	21.43697576	18.39055997	Fdxr	ferredoxin reductase [Source:MGI Symbol;Acc:MGI:104724]
ENSMUSG00000018862	0	0	0.036771494	0	0	0	0	0	0	0	0	0.047745707	Otop3	otopetrin 3 [Source:MGI Symbol;Acc:MGI:1916852]
ENSMUSG00000018865	8.275603278	6.799339259	8.710493556	4.794121341	3.785677658	3.824521047	54.00078352	53.95589474	53.16086726	29.31822398	30.12797143	28.20310455	Sult4a1	sulfotransferase family 4A, member 1 [Source:MGI Symbol;Acc:MGI:1888971]
ENSMUSG00000018868	0.022259952	0	0	0	0	0	0	0	0	0.11946601	0.092645824	0.177545857	Pnpla5	patatin-like phospholipase domain containing 5 [Source:MGI Symbol;Acc:MGI:1923022]
ENSMUSG00000018882	50.94549414	46.53316344	54.86945801	51.27670528	46.59981926	41.29257593	95.93203298	103.2116969	97.08221135	109.1720907	101.0075086	101.7225228	Mrpl45	mitochondrial ribosomal protein L45 [Source:MGI Symbol;Acc:MGI:1914286]
ENSMUSG00000018893	0	0	0.11684947	0.159761848	0	0.086933742	0.152844247	0.791461363	0.106231171	0.561495531	0	0	Mb	myoglobin [Source:MGI Symbol;Acc:MGI:96922]
ENSMUSG00000018899	82.42345891	77.82577254	81.48687348	87.01704797	76.19108586	70.20923532	24.8586789	21.86232366	21.82233767	42.79861861	43.7125067	38.47186384	Irf1	interferon regulatory factor 1 [Source:MGI Symbol;Acc:MGI:96590]
ENSMUSG00000018900	7.64704613	8.742155364	5.956840014	7.161953226	7.714233331	9.07458395	8.830716615	7.712725823	8.045933854	11.35060438	10.33223452	10.21460937	Slc22a5	solute carrier family 22 (organic cation transporter), member 5 [Source:MGI Symbol;Acc:MGI:1329012]
ENSMUSG00000018906	290.9475377	274.1514191	294.4465946	184.6854241	181.9142579	168.1522767	23.39008621	27.26452598	28.31827281	72.50593836	72.91838697	71.02294204	P4ha2	procollagen-proline, 2-oxoglutarate 4-dioxygenase (proline 4-hydroxylase), alpha II polypeptide [Source:MGI Symbol;Acc:MGI:8942
ENSMUSG00000018907	0.335577461	0.042937189	0.076347054	0.034795041	0.117525958	0	0	0.110812429	0	0.333517906	0.279334384	0.528705784	Alox12e	arachidonate lipoxygenase, epidermal [Source:MGI Symbol;Acc:MGI:1274790]
ENSMUSG00000018909	9.421270211	10.9097994	8.85363852	9.070618155	9.787748662	12.54463127	5.633550903	5.467531118	5.546022208	17.07431841	17.82065128	17.15046081	Arrb1	arrestin, beta 1 [Source:MGI Symbol;Acc:MGI:99473]
ENSMUSG00000018914	0	0	0	0	0	0	0	0	0	0	0.113885137	0	Il3	interleukin 3 [Source:MGI Symbol;Acc:MGI:96552]
ENSMUSG00000018916	1.290854635	0.805179629	1.046240942	2.459400517	2.203905519	2.376116496	0.288111406	0.319693858	0.550675833	1.058419075	0.984964076	2.097309758	Csf2	colony stimulating factor 2 (granulocyte-macrophage) [Source:MGI Symbol;Acc:MGI:1339752]
ENSMUSG00000018919	1.601060012	1.305956226	0.614682561	0.93380181	0.841084993	1.117873572	9.350592386	8.326909783	9.934673246	11.09885542	12.66084901	10.93734476	Tm4sf5	transmembrane 4 superfamily member 5 [Source:MGI Symbol;Acc:MGI:1922854]
ENSMUSG00000018920	21.5601165	18.80557309	27.96707355	17.31565222	15.92894373	11.68753721	3.112504168	3.067425074	2.070771694	8.350001233	8.037795617	7.785129262	Cxcl16	chemokine (C-X-C motif) ligand 16 [Source:MGI Symbol;Acc:MGI:1932682]
ENSMUSG00000018921	29.74755887	25.60512738	33.2540498	28.10140845	26.16609521	22.7729438	97.16133176	97.23555053	98.36985191	114.82853	114.0442283	116.8404408	Pelp1	proline, glutamic acid and leucine rich protein 1 [Source:MGI Symbol;Acc:MGI:1922523]
ENSMUSG00000018923	23.05721405	17.566946	26.10945276	19.6692273	18.04662234	13.70388684	30.51355797	38.87661888	28.8310536	35.9880714	34.02360837	37.51369649	Med11	mediator complex subunit 11 [Source:MGI Symbol;Acc:MGI:1913422]
ENSMUSG00000018924	0.121121711	0.278955753	0.093002639	0.169543186	0.063628879	0.046128108	0.486606174	0.539947402	0.535491822	2.464746615	2.369307738	2.173656073	Alox15	arachidonate 15-lipoxygenase [Source:MGI Symbol;Acc:MGI:87997]
ENSMUSG00000018925	0	0.052852817	0.187956268	0.321227823	0.120555516	0.139835821	0.061463767	0.022733785	0	0.123161492	0.076409334	0	Heatr9	HEAT repeat containing 9 [Source:MGI Symbol;Acc:MGI:3650286]
ENSMUSG00000018927	226.1181031	223.0287453	222.79699	5.710314004	5.969947231	4.069009334	3.338536979	2.646074067	4.249007046	90.26861639	83.23583139	87.11173712	Ccl6	chemokine (C-C motif) ligand 6 [Source:MGI Symbol;Acc:MGI:98263]
ENSMUSG00000018930	27.44695966	22.89788222	31.17030431	5.718828661	4.383836931	3.922950955	0.291021622	1.097938501	0.485444261	13.93730903	11.8846905	12.47985972	Ccl4	chemokine (C-C motif) ligand 4 [Source:MGI Symbol;Acc:MGI:98261]
ENSMUSG00000018931	12.88766795	13.98839435	10.90132879	13.03615337	12.54346371	13.9654627	14.79319259	14.94819097	16.80050423	27.35073393	27.3800817	24.07066017	Natd1	N-acetyltransferase domain containing 1 [Source:MGI Symbol;Acc:MGI:1344388]
ENSMUSG00000018932	72.82012753	71.38830376	70.36917889	62.80499333	60.95050063	54.3484774	70.56234965	76.67116762	72.81663913	68.25990789	78.17498228	70.4777792	Map2k3	mitogen-activated protein kinase kinase 3 [Source:MGI Symbol;Acc:MGI:1346868]
ENSMUSG00000018965	258.7842362	268.6379215	234.7690813	302.8228902	309.8787733	291.745333	230.6043978	228.3184941	232.2730611	231.4475157	231.5602647	225.3073948	Ywhah	tyrosine 3-monooxygenase/tryptophan 5-monooxygenase activation protein, eta polypeptide [Source:MGI Symbol;Acc:MGI:109194]
ENSMUSG00000018973	1.020201246	0.621960447	0.645116635	0.336012367	0.914254695	0.274259587	0	0	0	0.053679172	0.249769006	0.398879756	Hoxb1	homeobox B1 [Source:MGI Symbol;Acc:MGI:96182]
ENSMUSG00000018974	67.1424281	60.22968555	59.96400354	71.80942019	71.16397598	68.28812922	107.2906048	109.7246674	114.2617203	82.43111805	83.65848904	80.69928883	Sart3	squamous cell carcinoma antigen recognized by T cells 3 [Source:MGI Symbol;Acc:MGI:1858230]
ENSMUSG00000018983	1.965683919	1.438719966	1.15971855	1.305804157	1.260162184	1.50991423	22.99239814	24.44013954	24.59076822	7.569449058	7.709710711	6.923860743	E2f2	E2F transcription factor 2 [Source:MGI Symbol;Acc:MGI:1096341]
ENSMUSG00000018986	0.136598374	0.337071535	0.279696827	0.327783493	0.881613464	1.516077145	0.034843405	0.057994351	0.054488642	0.366552061	0.942121895	0.778222545	Slfn3	schlafen 3 [Source:MGI Symbol;Acc:MGI:1329005]
ENSMUSG00000018995	5.578208394	5.352994763	4.978758471	5.895108934	6.587140014	5.864824746	8.002207829	8.052860978	8.409212425	8.699773008	8.175375456	8.999788032	Nars2	asparaginyl-tRNA synthetase 2 (mitochondrial)(putative) [Source:MGI Symbol;Acc:MGI:2142075]
ENSMUSG00000018999	15.09837779	17.45906257	13.98791088	16.02848808	15.664808	15.45184292	5.351322955	5.601113731	5.028119989	8.639236233	8.983044274	9.03940673	Slc35b4	solute carrier family 35, member B4 [Source:MGI Symbol;Acc:MGI:1931249]
ENSMUSG00000019027	0.13096813	0.213656757	0.117323685	0.2087879	0.177737206	0.249390138	0.316672862	0.259485186	0.248878684	0.239176963	0.32705327	0.23213436	Dnah1	dynein, axonemal, heavy chain 1 [Source:MGI Symbol;Acc:MGI:107721]
ENSMUSG00000019039	41.3655511	39.67551795	36.7102085	43.74207368	45.04452009	38.98841827	61.28746646	60.54105905	60.46067561	38.95279688	46.11206432	43.47519568	Dalrd3	DALR anticodon binding domain containing 3 [Source:MGI Symbol;Acc:MGI:1915039]
ENSMUSG00000019054	257.2891957	179.9892924	287.7237817	220.6247102	181.4508734	140.4984655	109.4718382	124.2671571	100.6152864	82.96996029	79.75615715	82.51710523	Fis1	fission, mitochondrial 1 [Source:MGI Symbol;Acc:MGI:1913687]
ENSMUSG00000019055	143.4368352	150.6848283	133.8359311	142.3666787	137.7520885	132.2282405	49.90041497	47.60182516	53.57379054	51.60000733	47.12297673	48.19417734	Plod1	procollagen-lysine, 2-oxoglutarate 5-dioxygenase 1 [Source:MGI Symbol;Acc:MGI:99907]
ENSMUSG00000019066	15.30512773	13.58785972	14.16802935	13.8976361	13.3121406	13.10611794	12.60039942	10.64511319	12.70995437	6.230213939	6.999357455	5.441756367	Rab3d	RAB3D, member RAS oncogene family [Source:MGI Symbol;Acc:MGI:97844]
ENSMUSG00000019080	16.14833837	15.855845	18.29464038	12.94359168	12.87107417	8.675476051	5.694437192	5.365842005	5.135714725	7.516473433	7.795437604	8.344376791	Mfsd3	major facilitator superfamily domain containing 3 [Source:MGI Symbol;Acc:MGI:1916822]
ENSMUSG00000019082	39.53051289	38.59934643	39.84520815	35.24125284	35.07480421	33.03269483	23.07316807	22.03625118	23.67546526	26.671219	27.70366059	25.4620658	Slc25a22	solute carrier family 25 (mitochondrial carrier, glutamate), member 22 [Source:MGI Symbol;Acc:MGI:1915517]
ENSMUSG00000019087	220.8342628	227.7908137	185.6021756	172.0693458	174.5407039	166.5554376	59.1960387	63.20239215	64.04701428	120.9730355	115.9010048	118.2759986	Atp6ap1	ATPase, H+ transporting, lysosomal accessory protein 1 [Source:MGI Symbol;Acc:MGI:109629]
ENSMUSG00000019088	18.89442823	15.54136099	16.49927452	16.33550159	16.59862611	14.57295014	3.720996012	3.263787232	2.992602651	4.864254053	5.980493046	4.467600457	Dnase1l1	deoxyribonuclease 1-like 1 [Source:MGI Symbol;Acc:MGI:109628]
ENSMUSG00000019102	0.914778875	0.702276599	1.144664769	0.537487499	0.747539122	0.954833672	0.393222916	0.201381958	0.409951945	2.2729114	1.748540378	3.39292774	Aldh3a1	aldehyde dehydrogenase family 3, subfamily A1 [Source:MGI Symbol;Acc:MGI:1353451]
ENSMUSG00000019122	26.26709686	29.11252387	28.31560618	9.672694426	8.473239455	8.049083799	1.602499788	1.42754014	1.43536912	24.03801895	23.77971169	22.51678567	Ccl9	chemokine (C-C motif) ligand 9 [Source:MGI Symbol;Acc:MGI:104533]
ENSMUSG00000019124	39.60121874	41.88662542	40.51782547	51.80265542	48.28353535	52.17166773	6.454588853	5.873445294	6.566198098	4.721488898	4.935232261	4.722272499	Scrn1	secernin 1 [Source:MGI Symbol;Acc:MGI:1917188]
ENSMUSG00000019132	70.99366937	75.92983138	63.92698633	78.05338064	84.571096	87.06202629	41.28554549	42.22932819	47.30146299	57.20087529	54.74151294	54.55665806	BC005537	cDNA sequence BC005537 [Source:MGI Symbol;Acc:MGI:2441726]
ENSMUSG00000019139	89.36685933	69.11216035	88.6352504	68.84516589	62.47043805	55.22073171	289.2684795	315.6841047	282.3112572	193.4029228	199.0342775	202.9985213	Isyna1	myo-inositol 1-phosphate synthase A1 [Source:MGI Symbol;Acc:MGI:1919030]
ENSMUSG00000019143	31.81966713	30.90101985	27.16441245	32.50370951	36.24399395	35.62836769	20.55164821	21.08090135	24.41565692	29.1502949	27.15509611	26.77605982	Hars2	histidyl-tRNA synthetase 2 [Source:MGI Symbol;Acc:MGI:1918041]
ENSMUSG00000019146	0.039045815	0	0.039974819	0	0.032819106	0	0	0	0.014536897	0	0	0.006920672	Cacng2	calcium channel, voltage-dependent, gamma subunit 2 [Source:MGI Symbol;Acc:MGI:1316660]
ENSMUSG00000019158	75.3709047	46.2767603	99.39398063	55.3767911	49.20958298	33.55332364	66.52823473	77.42475639	47.561674	45.25005293	42.63012524	40.80271175	Tmem160	transmembrane protein 160 [Source:MGI Symbol;Acc:MGI:1916344]
ENSMUSG00000019173	291.4598584	294.367646	283.5863606	292.6049044	276.2136613	257.4310743	179.4531739	183.7159635	169.1941351	202.3218773	209.2746153	213.4991767	Rab5c	RAB5C, member RAS oncogene family [Source:MGI Symbol;Acc:MGI:105306]
ENSMUSG00000019178	0.937848471	0.518390748	1.113788366	0.665140435	0.985360034	0.457179015	0.03349159	0.185814506	0.034916436	0.234887327	0.499625116	0.132983103	Styxl1	serine/threonine/tyrosine interacting-like 1 [Source:MGI Symbol;Acc:MGI:1923821]
ENSMUSG00000019179	608.074928	474.3575437	694.2447175	560.9526287	469.3489136	366.4763095	481.2482144	524.7513932	440.2850339	360.1081534	368.4565807	366.2381527	Mdh2	malate dehydrogenase 2, NAD (mitochondrial) [Source:MGI Symbol;Acc:MGI:97050]
ENSMUSG00000019188	136.3825732	120.9045737	153.3377236	124.0554157	101.9596417	92.39137698	26.31353813	25.26050946	26.52236562	47.29407142	42.04368424	46.08102315	H13	histocompatibility 13 [Source:MGI Symbol;Acc:MGI:95886]
ENSMUSG00000019189	91.90722915	108.7231279	81.51870196	106.9892844	117.5043754	131.7712389	66.79074687	60.89048403	72.22519145	111.4316107	111.7933435	106.6554226	Rnf145	ring finger protein 145 [Source:MGI Symbol;Acc:MGI:1921565]
ENSMUSG00000019194	86.52981618	58.92269715	88.48485734	60.89208727	52.30538559	41.82932304	9.769554725	12.47993814	8.330475003	19.00230051	19.28600987	19.48549525	Scn1b	sodium channel, voltage-gated, type I, beta [Source:MGI Symbol;Acc:MGI:98247]
ENSMUSG00000019210	191.8450327	161.7278334	197.2826448	158.1540586	140.2272433	109.0105263	93.82222467	108.3694975	92.69033357	95.90785134	95.96341232	93.75197919	Atp6v1e1	ATPase, H+ transporting, lysosomal V1 subunit E1 [Source:MGI Symbol;Acc:MGI:894326]
ENSMUSG00000019214	12.27911283	11.33578847	11.3880877	16.67765554	15.71991885	15.31026192	46.96377124	46.69421398	52.36162448	29.49821116	32.31249054	28.64082913	Chtf18	CTF18, chromosome transmission fidelity factor 18 [Source:MGI Symbol;Acc:MGI:2384887]
ENSMUSG00000019230	1.081648069	1.564561652	1.053364658	0.443140773	0.609799512	0.612880214	0.141317673	0.03920219	0.061387418	0.920313114	0.999183222	0.935203058	Lhx9	LIM homeobox protein 9 [Source:MGI Symbol;Acc:MGI:1316721]
ENSMUSG00000019232	0.019132276	0.022031799	0.1175249	0.071415701	0.04020304	0.058290833	0.05124258	0.075812979	0.017807537	0	0.047777068	0	Etnppl	ethanolamine phosphate phospholyase [Source:MGI Symbol;Acc:MGI:1919010]
ENSMUSG00000019235	2.271177381	1.64055498	2.705704427	1.849680361	1.583592725	1.006495835	2.912450549	2.986263802	2.517485053	6.500846527	6.599654599	6.514066252	Rps6kl1	ribosomal protein S6 kinase-like 1 [Source:MGI Symbol;Acc:MGI:2443413]
ENSMUSG00000019254	117.1092661	94.47201392	111.5085064	91.22811918	82.95705924	74.01639656	79.4069513	93.05806098	81.6596027	70.41588045	73.58208507	72.25545857	Ppp1r12c	protein phosphatase 1, regulatory subunit 12C [Source:MGI Symbol;Acc:MGI:1924258]
ENSMUSG00000019256	4.462467824	4.624884984	4.217207837	6.69491573	8.042644589	9.922409918	1.271812081	0.935150229	2.092715935	4.590307916	4.614619378	5.110752284	Ahr	aryl-hydrocarbon receptor [Source:MGI Symbol;Acc:MGI:105043]
ENSMUSG00000019261	73.2583988	63.18304195	75.09722913	75.87457068	69.34892119	62.48271689	61.81052376	60.86757806	64.57081798	53.32644824	57.49397304	58.20503723	Map1s	microtubule-associated protein 1S [Source:MGI Symbol;Acc:MGI:2443304]
ENSMUSG00000019278	11.64414101	11.99737147	11.49189136	2.73910531	2.101136235	1.768912658	0.287967422	0.223673863	0.570415202	2.30812392	2.147938559	1.772293921	Dpep1	dipeptidase 1 [Source:MGI Symbol;Acc:MGI:94917]
ENSMUSG00000019295	24.63187686	26.49563919	22.28991103	24.0680427	27.17913271	27.53410237	6.07276735	5.411185457	6.427049466	8.665554105	8.04271156	8.996625418	Tmem129	transmembrane protein 129 [Source:MGI Symbol;Acc:MGI:1915616]
ENSMUSG00000019297	19.96287667	19.77486338	18.81960076	22.50779982	22.42988675	22.84520169	45.24416146	49.37252382	47.44144389	39.36070937	42.83405925	42.85412146	Nop9	NOP9 nucleolar protein [Source:MGI Symbol;Acc:MGI:1915092]
ENSMUSG00000019301	0.257078344	0.333043794	0.789584768	0.299876608	0.202576483	0.244764904	0.086067634	0.127336364	0.089729242	0.373669576	0.61522639	0.655008818	Hsd17b1	hydroxysteroid (17-beta) dehydrogenase 1 [Source:MGI Symbol;Acc:MGI:105077]
ENSMUSG00000019302	31.11597129	28.67448821	30.02079273	26.93629138	25.42967907	26.67441124	19.58090478	18.49093486	18.41148494	28.93724879	29.18412076	29.12930219	Atp6v0a1	ATPase, H+ transporting, lysosomal V0 subunit A1 [Source:MGI Symbol;Acc:MGI:103286]

ENSMUSG00000019303	12.4257877	9.426493216	14.56947592	15.82634639	14.55487322	10.99928107	30.3945821	33.64323004	29.96848704	24.89514313	25.0193979	23.92142216	Psmc3ip	proteasome (prosome, macropain) 26S subunit, ATPase 3, interacting protein [Source:MGI Symbol;Acc:MGI:1098610]
ENSMUSG00000019312	3.493292182	2.139057698	3.71832782	4.346510487	2.38858383	3.63217239	39.50393499	43.77938224	37.46866496	35.63608742	36.83230236	37.56779077	Grb7	growth factor receptor bound protein 7 [Source:MGI Symbol;Acc:MGI:102683]
ENSMUSG00000019320	1.35971776	1.060692995	1.436974522	2.128420843	1.751188682	2.739520583	0.822336326	1.042834195	0.694021994	0.549267614	1.642975939	1.360501187	Noxo1	NADPH oxidase organizer 1 [Source:MGI Symbol;Acc:MGI:1919143]
ENSMUSG00000019326	0.230021986	0.297992409	0.132465838	0.187821146	0.151046837	0.153303137	0.526230878	1.039654822	0.869761059	1.568837921	1.603562083	1.516142886	Aoc3	amine oxidase, copper containing 3 [Source:MGI Symbol;Acc:MGI:1306797]
ENSMUSG00000019338	11.84431382	15.35008535	10.1074058	14.22890034	15.77892342	16.60303494	14.21566634	13.08477057	14.92101157	15.19706896	16.31207982	15.72713018	Zfp687	zinc finger protein 687 [Source:MGI Symbol;Acc:MGI:1925516]
ENSMUSG00000019359	0.967185037	1.139664761	0.483584555	0.356826515	1.039814954	1.13072941	0.140566638	0.044564399	0.041870519	0.140834169	0.056168649	0.119601374	Gdpd2	glycerophosphodiester phosphodiesterase domain containing 2 [Source:MGI Symbol;Acc:MGI:1918834]
ENSMUSG00000019362	175.5366719	127.6543528	244.8618582	152.9873579	127.7722114	93.42724065	245.7053978	264.6124052	187.5678606	168.8222161	153.1520292	160.4553392	D8Ertd738e	DNA segment, Chr 8, ERATO Doi 738, expressed [Source:MGI Symbol;Acc:MGI:1289231]
ENSMUSG00000019368	0.06138158	0	0.031421006	0	0	0.187012957	2.055002893	2.249866426	2.342389747	4.557063065	4.93056845	4.787012286	Sec14l4	SEC14-like lipid binding 4 [Source:MGI Symbol;Acc:MGI:2144095]
ENSMUSG00000019370	620.9653141	596.3674319	637.000943	620.2850134	558.4013146	511.6165369	377.0390432	390.7369373	346.0688952	341.0395875	345.9368157	333.9553463	Calm3	calmodulin 3 [Source:MGI Symbol;Acc:MGI:103249]
ENSMUSG00000019373	46.50420412	53.09600447	35.62917168	52.59366575	60.83246109	59.11387771	82.44660398	74.74777997	84.11140821	126.1287651	113.2736154	122.8207053	Cops3	COP9 signalosome subunit 3 [Source:MGI Symbol;Acc:MGI:1349409]
ENSMUSG00000019428	265.393023	195.1340465	267.4656498	221.8373237	184.0681442	157.0084045	287.2969563	311.0707843	267.3599276	172.0398497	170.3200006	176.2467821	Fkbp8	FK506 binding protein 8 [Source:MGI Symbol;Acc:MGI:1341070]
ENSMUSG00000019429	0	0	0	0	0	0	0	0.029808285	0	0.053829324	0.025046766	0.133331835	Ffar3	free fatty acid receptor 3 [Source:MGI Symbol;Acc:MGI:2685324]
ENSMUSG00000019432	118.6453225	104.7218916	126.1081971	134.2309366	130.0412317	120.3905874	198.9865356	200.4884699	196.2031787	152.0214346	153.4623994	147.9174177	Ddx39b	DEAD (Asp-Glu-Ala-Asp) box polypeptide 39B [Source:MGI Symbol;Acc:MGI:99240]
ENSMUSG00000019433	176.1495763	165.2906529	178.8331526	168.8074601	150.808991	130.8468505	200.7894274	215.8304963	195.3220977	133.9030009	136.5490006	136.3408417	Gipc1	GIPC PDZ domain containing family, member 1 [Source:MGI Symbol;Acc:MGI:1926252]
ENSMUSG00000019437	8.340796725	10.18197201	8.502597376	8.685444457	8.199179201	6.925624993	7.574309829	9.078383537	8.429646702	7.65276536	8.581747203	8.724250241	Tlcd1	TLC domain containing 1 [Source:MGI Symbol;Acc:MGI:1915572]
ENSMUSG00000019461	78.02376012	75.89263257	72.86109837	81.94411464	75.03998765	65.54804127	10.42806661	9.94755566	10.95765412	20.75129088	22.46240742	23.64894733	Plscr3	phospholipid scramblase 3 [Source:MGI Symbol;Acc:MGI:1917560]
ENSMUSG00000019464	0.042786034	0.098540634	0.043804027	0.159708894	0.074922636	0.108631159	0.241923178	0.183671203	0.10619596	0.089299226	0.10684517	0.088475417	Ptger1	prostaglandin E receptor 1 (subtype EP1) [Source:MGI Symbol;Acc:MGI:97793]
ENSMUSG00000019467	213.6906524	196.0282704	229.0527266	215.4105814	188.3301302	165.5405665	58.46338164	59.70066681	55.43055573	42.3561037	43.14480765	42.56759999	Arhgef25	Rho guanine nucleotide exchange factor (GEF) 25 [Source:MGI Symbol;Acc:MGI:1277173]
ENSMUSG00000019470	29.66094656	25.03798303	28.4570422	30.32415219	28.74273024	26.18578784	78.0214992	81.84597531	79.9791598	50.11777686	55.71175701	53.0262916	Xab2	XPA binding protein 2 [Source:MGI Symbol;Acc:MGI:1914689]
ENSMUSG00000019471	78.60415664	64.70905474	88.68520143	54.4693867	52.05470378	43.49440078	169.3927839	192.2867409	159.4967895	97.67117892	97.55761454	96.84027665	Cdc37	cell division cycle 37 [Source:MGI Symbol;Acc:MGI:109531]
ENSMUSG00000019478	38.91486566	31.5013651	37.86135079	31.36270688	27.74736174	22.68488618	57.87935948	63.53125867	53.7562225	31.86479816	33.79661195	34.37794371	Rab4a	RAB4A, member RAS oncogene family [Source:MGI Symbol;Acc:MGI:105069]
ENSMUSG00000019487	110.3833176	96.87515535	115.6805153	119.6383938	109.7458626	97.78012929	66.75715192	70.41655785	64.7408725	55.45903816	60.74767699	61.61316569	Trip10	thyroid hormone receptor interactor 10 [Source:MGI Symbol;Acc:MGI:2146901]
ENSMUSG00000019489	0	0	0	0	0.053691589	0	0	0	0	0	0	0.090576971	Cd70	CD70 antigen [Source:MGI Symbol;Acc:MGI:1195273]
ENSMUSG00000019494	192.5361016	164.9554518	206.8070527	200.4276812	170.3917582	149.8676663	161.0043884	180.3123845	136.0383616	109.4399788	108.2687784	113.2373058	Cops6	COP9 signalosome subunit 6 [Source:MGI Symbol;Acc:MGI:1349439]
ENSMUSG00000019505	626.7756382	542.6812222	690.5328458	580.6965723	561.3019409	407.0560789	584.0022208	616.5944798	501.4019715	526.850338	478.4175451	487.5906139	Ubb	ubiquitin B [Source:MGI Symbol;Acc:MGI:98888]
ENSMUSG00000019518	8.376694409	8.223554064	6.539969917	11.44430356	8.959351606	12.43943921	12.32073051	13.2235648	14.13499466	9.891515167	12.13961326	11.42918992	Ap4m1	adaptor-related protein complex AP-4, mu 1 [Source:MGI Symbol;Acc:MGI:1337063]
ENSMUSG00000019528	16.14641859	15.09892093	17.02884722	7.052906286	6.015744291	5.822131411	7.003107129	7.827500975	6.981289221	14.39137532	11.91512824	12.53336987	Gyg	glycogenin [Source:MGI Symbol;Acc:MGI:1351614]
ENSMUSG00000019539	461.8391027	336.8766462	575.9364493	243.3057402	194.5875618	145.238569	85.25769431	87.8867478	63.54465375	78.56210348	76.19226238	77.14402173	Rcn3	reticulocalbin 3, EF-hand calcium binding domain [Source:MGI Symbol;Acc:MGI:1277122]
ENSMUSG00000019558	109.8144109	117.3865598	109.3216091	98.2190225	104.3165877	109.4861981	110.0330684	106.9434034	117.9409551	88.01158187	93.38274229	89.45871005	Slc6a8	solute carrier family 6 (neurotransmitter transporter, creatine), member 8 [Source:MGI Symbol;Acc:MGI:2147834]
ENSMUSG00000019564	5.240571396	6.579594547	5.514293512	5.954533223	6.143335056	7.502820625	5.360967794	4.698817719	6.255210021	7.009649359	7.058358038	7.170749204	Arid3a	AT rich interactive domain 3A (BRIGHT-like) [Source:MGI Symbol;Acc:MGI:1328360]
ENSMUSG00000019577	2.108107122	3.391980264	2.51304783	1.751661785	1.699104582	2.133610739	6.032936814	5.893238004	7.3647433	6.819344377	6.370115438	5.297658571	Pdk4	pyruvate dehydrogenase kinase, isoenzyme 4 [Source:MGI Symbol;Acc:MGI:1351481]
ENSMUSG00000019578	4.981486007	5.902710754	4.015948983	5.232528152	5.183280348	5.7556166	4.125085001	3.194554582	4.45737201	5.424478482	4.647379816	4.030827173	Ubxn6	UBX domain protein 6 [Source:MGI Symbol;Acc:MGI:1913780]
ENSMUSG00000019579	111.9825021	87.52045028	127.4134384	92.72555706	80.68105382	57.62671441	70.53724064	80.56431696	63.03268521	44.26336568	47.50966988	42.85037743	Mydgf	myeloid derived growth factor [Source:MGI Symbol;Acc:MGI:2156020]
ENSMUSG00000019590	3.698061622	2.88336483	4.495932428	4.996717475	4.209189891	2.376629966	17.33311231	15.30256484	14.41339204	19.64023351	17.47555697	18.26419338	Cyb561	cytochrome b-561 [Source:MGI Symbol;Acc:MGI:103253]
ENSMUSG00000019647	5.149786751	6.497105745	4.910488435	4.94704039	5.649382111	5.854907177	0.180298523	0.025007831	0.140976773	1.908027667	1.838648639	2.01347106	Sema6a	sema domain, transmembrane domain (TM), and cytoplasmic domain, (semaphorin) 6A [Source:MGI Symbol;Acc:MGI:1203727]
ENSMUSG00000019659	68.36434086	49.95699519	93.68348603	63.73043768	54.50279169	38.81134023	86.37023933	100.0445472	64.94813059	41.65309519	37.17179018	44.5302012	Ccdc12	coiled-coil domain containing 12 [Source:MGI Symbol;Acc:MGI:1919904]
ENSMUSG00000019689	11.67916098	12.23569672	21.30690469	12.12798922	14.39285237	9.497777409	32.69182479	27.14135609	14.13980248	11.46784304	11.11054096	10.58382661	Fmc1	formation of mitochondrial complex V assembly factor 1 [Source:MGI Symbol;Acc:MGI:1913367]
ENSMUSG00000019699	10.20605536	13.73477802	8.470397582	14.3425405	15.76739243	23.32130311	3.841485407	3.439980692	5.438253207	11.18176707	10.55655277	10.10187475	Akt3	thymoma viral proto-oncogene 3 [Source:MGI Symbol;Acc:MGI:1345147]
ENSMUSG00000019710	48.47513186	46.07574238	47.74114359	46.52642515	42.5671313	41.80522593	118.5736002	122.5648766	107.2006987	76.90098052	75.12127535	76.11728784	Mrpl24	mitochondrial ribosomal protein L24 [Source:MGI Symbol;Acc:MGI:1914957]
ENSMUSG00000019715	40.38344658	42.90001353	39.17026922	42.06554821	43.01639988	44.57834322	63.55912219	63.32983087	71.08290963	66.77912087	67.1372601	65.70084877	Gle1	GLE1 RNA export mediator (yeast) [Source:MGI Symbol;Acc:MGI:1921662]
ENSMUSG00000019718	10.34148987	10.57835108	9.635528203	10.43937832	9.947606395	11.56856393	3.52199055	3.824322944	3.094826378	3.579103618	4.222028544	4.619900044	L3hypdh	L-3-hydroxyproline dehydratase (trans-) [Source:MGI Symbol;Acc:MGI:1914467]
ENSMUSG00000019726	5.730370212	7.868941933	4.581745527	6.057330775	7.272758344	10.01374761	3.123895028	2.411362038	3.575395672	6.543181083	7.408204651	6.224404596	Lyst	lysosomal trafficking regulator [Source:MGI Symbol;Acc:MGI:107448]
ENSMUSG00000019731	21.83459708	23.82337605	20.3172016	19.21136224	19.76978185	21.8964362	31.88192042	28.64550502	33.64630912	39.54367078	41.23431824	40.68804838	Slc35e1	solute carrier family 35, member E1 [Source:MGI Symbol;Acc:MGI:2142403]
ENSMUSG00000019732	2.439194533	2.966658897	2.04828934	2.148338942	2.274813471	2.525895858	2.715954651	1.601863066	2.091739124	2.328889685	1.916316905	1.845924049	Calr3	calreticulin 3 [Source:MGI Symbol;Acc:MGI:1920566]
ENSMUSG00000019734	0.679397176	1.401729718	0.289817436	1.637839227	1.130207959	1.574015465	1.61746754	1.62651261	2.134198206	1.823114197	1.578770169	1.58050855	Tmc4	transmembrane channel-like gene family 4 [Source:MGI Symbol;Acc:MGI:2669035]
ENSMUSG00000019737	3.791056196	2.328317915	4.010630708	5.247664504	3.053719007	3.080084171	43.3224786	46.06846389	43.16604584	18.8201069	13.46419989	12.65497277	Syne4	spectrin repeat containing, nuclear envelope family member 4 [Source:MGI Symbol;Acc:MGI:2141950]
ENSMUSG00000019738	48.97484315	33.66704133	63.49950076	47.32098923	44.2538241	30.00615709	89.51177129	94.49684087	59.72781331	49.42120826	46.5413142	48.23336697	Polr2i	polymerase (RNA) II (DNA directed) polypeptide I [Source:MGI Symbol;Acc:MGI:1917170]
ENSMUSG00000019756	0.033800855	0	0	0	0	0	0	0	0	0	0	0	Prl8a1	prolactin family 8, subfamily a, member 1 [Source:MGI Symbol;Acc:MGI:1920494]
ENSMUSG00000019761	2.693336719	1.457338695	3.75407562	2.876757462	2.863875371	2.026749391	2.839106862	2.700279945	2.265223502	4.121637424	2.485032085	2.501932661	Krt10	keratin 10 [Source:MGI Symbol;Acc:MGI:96685]
ENSMUSG00000019762	0	0	0	0.034825219	0	0	0	0.110908537	0	0.2002843	0.093192217	0.066145542	Iyd	iodotyrosine deiodinase [Source:MGI Symbol;Acc:MGI:1917587]
ENSMUSG00000019763	13.82760143	14.67242938	15.86736391	18.63433243	15.14259207	14.82785788	29.57570804	27.56195589	25.5459151	25.82056773	21.66399361	23.064854	Rmnd1	required for meiotic nuclear division 1 homolog [Source:MGI Symbol;Acc:MGI:1913334]
ENSMUSG00000019767	0.077997259	0.029939284	0.106470695	0.02426192	0.054632409	0.099015168	0.20890253	0.231802193	0.241988831	0.58138922	0.497757182	0.622107811	Ccdc170	coiled-coil domain containing 170 [Source:MGI Symbol;Acc:MGI:2685067]
ENSMUSG00000019768	0.063982882	0.147359154	0.081881506	0.179123321	0	0.097469206	0.014280615	0.01584604	0.044664481	0.357694855	0.226352001	0.170109612	Esr1	estrogen receptor 1 (alpha) [Source:MGI Symbol;Acc:MGI:1352467]
ENSMUSG00000019772	0.105929315	0.073189831	0.065069793	0	0	0	0.018914256	0	0	0.037900508	0.052905281	0.018775433	Vip	vasoactive intestinal polypeptide [Source:MGI Symbol;Acc:MGI:98933]
ENSMUSG00000019773	14.58842567	14.16912519	13.1628727	20.59240858	21.59780962	19.86644402	96.36602948	91.9667262	99.53997164	66.13471619	66.81932506	70.22592293	Fbxo5	F-box protein 5 [Source:MGI Symbol;Acc:MGI:1914391]
ENSMUSG00000019774	7.617159529	7.524041318	8.110327731	7.929601052	7.540647907	8.096807616	4.745186538	3.689097755	4.915552823	5.359850549	6.340516902	5.940452501	Mtrf1l	mitochondrial translational release factor 1-like [Source:MGI Symbol;Acc:MGI:1918830]
ENSMUSG00000019775	1.237240864	1.298102326	1.313592125	1.325620674	1.194000314	1.675349298	0.360011412	0.481186353	0.511810243	1.721506483	1.685948771	1.437598324	Rgs17	regulator of G-protein signaling 17 [Source:MGI Symbol;Acc:MGI:1927469]
ENSMUSG00000019777	85.82577781	81.79367803	79.2151508	84.98153966	83.31825597	80.06644325	172.7865735	172.5392522	168.1586165	119.5999194	111.9411004	108.3657471	Hdac2	histone deacetylase 2 [Source:MGI Symbol;Acc:MGI:1097691]
ENSMUSG00000019779	5.479037337	6.838324306	4.887230197	5.709968446	7.60078856	9.396135539	0.190390051	0.227511196	0.320637507	1.966217115	1.63859133	1.991691934	Frk	fyn-related kinase [Source:MGI Symbol;Acc:MGI:103265]
ENSMUSG00000019782	65.30526191	55.50800168	93.01158649	65.05879953	54.87946493	40.90529664	94.48109046	113.5789993	84.20460845	59.14479896	52.41051652	53.57914456	Rwdd1	RWD domain containing 1 [Source:MGI Symbol;Acc:MGI:1913771]
ENSMUSG00000019785	0.016182207	0	0	0	0	0	0.245600797	0.288554065	0.421728561	0.535560269	0.336751368	0.200774978	Clvs2	clavesin 2 [Source:MGI Symbol;Acc:MGI:2443223]
ENSMUSG00000019787	0	0	0	0.044027936	0	0	0	0	0	0.042201718	0.03927289	0	Trdn	triadin [Source:MGI Symbol;Acc:MGI:1924007]
ENSMUSG00000019789	0.036188804	0.062509902	0.111149496	0.050656196	0.038022135	0.041346536	0.25846832	0.34057658	0.202098326	0.307515291	0.436791164	0.52917824	Hey2	hairy/enhancer-of-split related with YRPW motif 2 [Source:MGI Symbol;Acc:MGI:1341884]
ENSMUSG00000019790	7.250702104	10.16303447	7.826286158	9.276785196	9.42773547	11.2703647	2.504361436	2.242610363	3.175838169	9.06807595	9.981752183	9.856694388	Stxbp5	syntaxin binding protein 5 (tomosyn) [Source:MGI Symbol;Acc:MGI:1926058]
ENSMUSG00000019791	3.078518455	1.548422363	3.550263585	2.641676173	2.379385176	3.180373713	6.223459089	5.152960207	5.434300988	4.810995796	4.653837439	4.766613086	Hint3	histidine triad nucleotide binding protein 3 [Source:MGI Symbol;Acc:MGI:1914097]
ENSMUSG00000019792	7.317770039	7.247037999	6.173308531	8.549686097	7.811505347	7.647271482	14.68649523	14.26661025	16.51005838	13.61479083	13.15721956	14.88999135	Trmt11	tRNA methyltransferase 11 [Source:MGI Symbol;Acc:MGI:1920931]
ENSMUSG00000019794	23.55605527	20.2950064	22.75226109	20.05668224	22.89771968	16.92724143	51.96175066	52.8032479	45.73044974	28.45264247	29.05424864	28.5330126	Katna1	katanin p60 (ATPase-containing) subunit A1 [Source:MGI Symbol;Acc:MGI:1344353]

ENSMUSG00000019795	19.91059916	22.05044865	17.94600894	18.52097686	16.14821113	17.65685245	33.62362873	32.96818331	32.42355662	30.10866742	30.16018813	31.06917105	Pcmt1	protein-L-isoaspartate (D-aspartate) O-methyltransferase 1 [Source:MGI Symbol;Acc:MGI:97502]
ENSMUSG00000019796	0.796414171	1.426618445	0.294436634	0.536756002	1.092437513	1.617638037	8.176735005	5.632309953	11.73676934	8.152720897	7.771065014	7.567764118	Lrp11	low density lipoprotein receptor-related protein 11 [Source:MGI Symbol;Acc:MGI:2442989]
ENSMUSG00000019797	30.50267466	20.74579899	36.80915785	22.62015328	20.30921759	15.01774531	40.34767692	46.57055777	33.42826753	26.83437727	21.52874186	22.74954961	Mtres1	mitochondrial transcription rescue factor 1 [Source:MGI Symbol;Acc:MGI:1915101]
ENSMUSG00000019802	41.51961366	45.30872498	41.6565409	39.80743003	42.15860415	44.85122289	17.05551025	16.72098632	18.59042338	39.86049415	37.2112323	37.46430398	Sec63	SEC63-like (S. cerevisiae) [Source:MGI Symbol;Acc:MGI:2155302]
ENSMUSG00000019803	0	0	0	0	0	0.050577192	0.059282182	0	0	0.029697505	0.082909434	0	Nr2e1	nuclear receptor subfamily 2, group E, member 1 [Source:MGI Symbol;Acc:MGI:1100526]
ENSMUSG00000019804	177.5025657	150.732405	179.4614566	142.8262959	119.6964167	104.6011967	242.4803032	250.2177097	232.0979333	144.7435296	137.0079171	145.5108877	Snx3	sorting nexin 3 [Source:MGI Symbol;Acc:MGI:1860188]
ENSMUSG00000019806	15.63115618	14.11273867	12.84752948	15.11176937	15.98611371	15.65103885	2.48964944	1.84170803	2.299581753	7.548805229	6.699119973	7.219020615	Aig1	androgen-induced 1 [Source:MGI Symbol;Acc:MGI:1913503]
ENSMUSG00000019808	3.756921175	2.805327283	3.15517481	2.711592296	2.92959771	3.599329688	16.53463364	16.48625897	10.87282558	10.73777507	11.53176346	10.19652022	Adat2	adenosine deaminase, tRNA-specific 2 [Source:MGI Symbol;Acc:MGI:1914007]
ENSMUSG00000019809	8.432014867	9.871192566	7.944318558	9.463254542	8.947479673	10.60461475	8.953462082	9.019140952	10.40339288	13.02978028	12.42864234	11.79240217	Pex3	peroxisomal biogenesis factor 3 [Source:MGI Symbol;Acc:MGI:1929646]
ENSMUSG00000019810	18.85320558	18.90629126	18.06810495	12.08821153	12.106545	11.24336297	5.491038067	5.421490548	5.350790956	13.60776407	14.20972889	13.32649353	Fuca2	fucosidase, alpha-L- 2, plasma [Source:MGI Symbol;Acc:MGI:1914098]
ENSMUSG00000019813	4.311358042	5.32404156	5.227030346	6.802533819	6.437044669	8.490566981	15.82681859	16.91546494	17.29215178	15.83156956	16.26751958	15.03195584	Cep57l1	centrosomal protein 57-like 1 [Source:MGI Symbol;Acc:MGI:1915511]
ENSMUSG00000019814	23.09766902	22.91790561	21.29737417	24.34694335	27.25930337	23.8778989	73.84678565	72.67404824	75.82567243	59.30421287	54.51130137	52.70631728	Ltv1	LTV1 ribosome biogenesis factor [Source:MGI Symbol;Acc:MGI:2447810]
ENSMUSG00000019815	0	0	0	0	0	0	0	0	0	0	0	0	Zc2hc1b	zinc finger, C2HC-type containing 1B [Source:MGI Symbol;Acc:MGI:1922372]
ENSMUSG00000019817	2.810023695	4.601026451	2.652125267	2.86810556	4.428569961	4.113474019	2.27353218	2.131292385	3.351051454	29.41580287	25.254552	29.84483459	Plagl1	pleiomorphic adenoma gene-like 1 [Source:MGI Symbol;Acc:MGI:1100874]
ENSMUSG00000019818	158.3712404	189.7324897	131.3273149	149.1334888	162.6742836	184.2110225	132.3624784	123.2998719	151.7064094	190.6415796	188.475692	187.6064453	Cd164	CD164 antigen [Source:MGI Symbol;Acc:MGI:1859568]
ENSMUSG00000019820	9.844782225	13.41704269	8.241953827	11.77790094	15.13313108	24.16726177	18.6824565	15.03735174	22.60311369	45.97083388	49.41093895	47.2229124	Utrn	utrophin [Source:MGI Symbol;Acc:MGI:104631]
ENSMUSG00000019822	12.85607258	12.14567395	13.67231424	10.7728843	9.400022291	8.433315066	19.20742704	18.68779542	19.21870869	10.84234175	10.74506264	11.69564089	Smpd2	sphingomyelin phosphodiesterase 2, neutral [Source:MGI Symbol;Acc:MGI:1278330]
ENSMUSG00000019823	7.897786238	9.371984488	7.395453307	5.594972569	6.881176604	7.005974193	15.67119676	12.71380908	16.49478205	18.6301572	18.21910454	17.2419625	Mical1	microtubule associated monooxygenase, calponin and LIM domain containing 1 [Source:MGI Symbol;Acc:MGI:2385847]
ENSMUSG00000019826	8.312581736	8.438448643	7.455356872	7.885382289	8.998356074	8.912998694	11.55103382	10.07231313	12.25559453	10.01629354	10.97952117	10.20801014	Zbtb24	zinc finger and BTB domain containing 24 [Source:MGI Symbol;Acc:MGI:3039618]
ENSMUSG00000019828	0.104692184	0.017222633	0.06124748	0.153523899	0.078568506	0.250618163	0	0	0	0	0.049797533	0	Grm1	glutamate receptor, metabotropic 1 [Source:MGI Symbol;Acc:MGI:1351338]
ENSMUSG00000019831	4.247932545	4.947511695	3.952135059	5.3960004	5.651039794	5.683782858	17.65006809	14.14461313	19.48337101	16.25336418	16.01272474	17.23424071	Wasf1	WAS protein family, member 1 [Source:MGI Symbol;Acc:MGI:1890563]
ENSMUSG00000019832	20.89802024	24.86731606	18.26824323	19.02664797	18.77580843	22.38673763	1.602612218	1.645580671	1.421420695	8.747265084	9.946654087	9.640074286	Rab32	RAB32, member RAS oncogene family [Source:MGI Symbol;Acc:MGI:1915094]
ENSMUSG00000019834	0.172267078	0.044083199	0.05878859	0.053585602	0.100552309	0.116633525	0	0	0	0.051362944	0	0.016963036	Slc22a16	solute carrier family 22 (organic cation transporter), member 16 [Source:MGI Symbol;Acc:MGI:1918090]
ENSMUSG00000019836	0.300298519	0.247006558	0.175681955	0.480400532	0.180292396	0.718871838	0.651099222	0.424983526	0.359364271	0.920948348	0.464226701	1.216603141	Amd-ps4	S-adenosylmethionine decarboxylase, pseudogene 4 [Source:MGI Symbol;Acc:MGI:3650439]
ENSMUSG00000019837	28.09337654	31.18965046	33.23584948	31.36990456	27.14517329	24.90971881	42.1448678	44.29091988	37.63547498	45.57559411	44.17148148	43.53790346	Gtf3c6	general transcription factor IIIC, polypeptide 6, alpha [Source:MGI Symbol;Acc:MGI:1914621]
ENSMUSG00000019838	4.024376315	4.510143802	3.347603147	3.571060868	4.266004153	4.830575902	1.84455458	1.95776336	2.725683381	4.37107593	4.621049905	3.805329528	Slc16a10	solute carrier family 16 (monocarboxylic acid transporters), member 10 [Source:MGI Symbol;Acc:MGI:1919722]
ENSMUSG00000019841	3.082900868	4.463006525	2.930804773	4.753755308	5.275075017	7.128433352	6.38936928	5.613639445	7.672329778	10.97780276	10.88192686	11.01964185	Rev3l	REV3 like, DNA directed polymerase zeta catalytic subunit [Source:MGI Symbol;Acc:MGI:1337131]
ENSMUSG00000019842	6.610051457	10.03595864	7.58629749	7.347064927	11.32419716	10.16571873	9.05868868	8.883860626	11.48180176	11.1095402	12.89687884	13.17115005	Traf3ip2	TRAF3 interacting protein 2 [Source:MGI Symbol;Acc:MGI:2143599]
ENSMUSG00000019843	28.54298119	31.15652789	28.04583049	27.07731908	32.35590114	29.87412038	18.44315245	19.41596204	20.08748019	22.89127215	24.17170288	22.8198482	Fyn	Fyn proto-oncogene [Source:MGI Symbol;Acc:MGI:95602]
ENSMUSG00000019845	3.260006822	3.09036326	2.581546684	4.353181565	4.390259463	5.322863126	10.77352542	9.420865461	12.32152632	7.394716017	7.391259779	8.427866754	Tube1	tubulin, epsilon 1 [Source:MGI Symbol;Acc:MGI:1919174]
ENSMUSG00000019846	61.49726192	69.1624738	57.41002929	53.56336797	57.87007886	67.01572038	2.697058066	2.636817144	2.766203255	22.68379954	22.46880272	21.5194039	Lama4	laminin, alpha 4 [Source:MGI Symbol;Acc:MGI:109321]
ENSMUSG00000019848	0.064462154	0.37115742	0.03299794	0.120310046	0	0.098199313	0.489177917	0.510871583	0.209995551	0.691918497	0.295120322	0.39989563	Popdc3	popeye domain containing 3 [Source:MGI Symbol;Acc:MGI:1930153]
ENSMUSG00000019849	6.5253117	8.135130389	5.810239164	6.70265225	7.128797296	7.515272088	6.169988452	5.852135498	7.227382782	10.76170431	11.16472255	11.32512042	Prep	prolyl endopeptidase [Source:MGI Symbol;Acc:MGI:1270863]
ENSMUSG00000019850	8.720316288	10.70183923	7.833625485	9.568611349	11.58686921	12.62354287	2.336972764	1.426999221	2.248980431	8.548263229	8.560981036	7.686978627	Tnfaip3	tumor necrosis factor, alpha-induced protein 3 [Source:MGI Symbol;Acc:MGI:1196377]
ENSMUSG00000019851	4.122093865	3.502988297	3.881653194	2.777007945	3.080279887	3.425153796	18.79415248	19.69698495	21.07094191	32.43478723	29.61497798	30.18203778	Perp	PERP, TP53 apoptosis effector [Source:MGI Symbol;Acc:MGI:1929938]
ENSMUSG00000019852	0.072474417	0.068730132	0.082928045	0.075588634	0.080625159	0.139629732	0.194110528	0.177378936	0.186496594	0.831306516	0.848135662	0.702736577	Arfgef3	ARFGEF family member 3 [Source:MGI Symbol;Acc:MGI:106387]
ENSMUSG00000019853	7.949409137	7.451054981	9.108547973	4.075728953	4.370299303	3.397092365	4.703859682	4.830318939	4.538330275	7.875357244	10.2911001	8.990518345	Hebp2	heme binding protein 2 [Source:MGI Symbol;Acc:MGI:1860084]
ENSMUSG00000019854	20.99811938	19.86360001	19.80126398	18.73321977	21.6337786	21.53898705	43.77450227	41.60325792	44.54962436	35.54791338	34.27928234	34.52183417	Reps1	RalBP1 associated Eps domain containing protein [Source:MGI Symbol;Acc:MGI:1196373]
ENSMUSG00000019856	0.671916326	0.565429908	0.661445198	0.434091652	0.597347149	1.102309403	1.82268677	1.689673242	1.635641023	4.091513542	5.829653607	3.710228564	Fam184a	family with sequence similarity 184, member A [Source:MGI Symbol;Acc:MGI:1923156]
ENSMUSG00000019857	8.754356449	8.369208013	11.66835034	7.437256451	8.980923127	7.894701708	22.41480929	23.64466408	18.83309429	19.98271575	18.59590176	16.32176151	Asf1a	anti-silencing function 1A histone chaperone [Source:MGI Symbol;Acc:MGI:1913653]
ENSMUSG00000019861	9.355070106	9.687208673	6.96669013	9.508253319	10.29886129	12.93469073	7.165017726	6.872819264	8.549818864	12.28155331	12.93834322	13.97849456	Gopc	golgi associated PDZ and coiled-coil motif containing [Source:MGI Symbol;Acc:MGI:2149946]
ENSMUSG00000019863	6.184124574	5.846992766	5.598167953	5.892416566	5.403112178	6.495305149	19.64329906	21.47414113	18.69162367	16.15795865	15.44297959	14.48011963	Qrsl1	glutaminyl-tRNA synthase (glutamine-hydrolyzing)-like 1 [Source:MGI Symbol;Acc:MGI:1923813]
ENSMUSG00000019864	4.247447296	3.250642656	4.402524085	4.283694141	3.68651523	3.797847885	17.52339143	16.77853723	21.46104134	11.53931058	10.67259441	10.70807501	Rtn4ip1	reticulon 4 interacting protein 1 [Source:MGI Symbol;Acc:MGI:2178759]
ENSMUSG00000019865	0.334294578	0.714920869	0	0.668481873	0.25087857	0.181875808	0.085271596	0.189237948	0.044449669	0.128150831	0.198761795	0.211614343	Nmbr	neuromedin B receptor [Source:MGI Symbol;Acc:MGI:1100525]
ENSMUSG00000019866	1.911997872	2.644082464	2.333257449	2.795848189	3.407887517	4.39500852	0.10668676	0.160660803	0.174783046	0.893293407	1.016031183	0.839665229	Crybg1	crystallin beta-gamma domain containing 1 [Source:MGI Symbol;Acc:MGI:109544]
ENSMUSG00000019867	0	0	0	0.027130728	0	0	0.051911965	0	0	0	0	0	Gje1	gap junction protein, epsilon 1 [Source:MGI Symbol;Acc:MGI:1923993]
ENSMUSG00000019868	23.52741443	22.00959319	26.62007453	27.70454164	31.14124297	25.71670616	33.43434875	26.52702134	26.05226655	26.07809221	25.72192749	26.0094784	Vta1	vesicle (multivesicular body) trafficking 1 [Source:MGI Symbol;Acc:MGI:1913451]
ENSMUSG00000019872	26.78138461	33.40626477	22.27500787	8.166809051	7.832702991	6.542459863	2.749085602	1.806179987	1.99868647	21.88957786	19.62845718	18.20469179	Smpdl3a	sphingomyelin phosphodiesterase, acid-like 3A [Source:MGI Symbol;Acc:MGI:1931437]
ENSMUSG00000019873	16.36423369	19.03726078	14.67888365	16.15239052	19.56252302	25.59019671	5.69965434	5.101818882	7.204309127	17.48585724	16.80501417	16.3658217	Reep3	receptor accessory protein 3 [Source:MGI Symbol;Acc:MGI:88930]
ENSMUSG00000019874	0.71543804	1.23579552	1.098690326	0.143064648	0.268457947	0.07784803	0.045623342	0	0	1.279884867	1.318673563	1.630385474	Fabp7	fatty acid binding protein 7, brain [Source:MGI Symbol;Acc:MGI:101916]
ENSMUSG00000019876	0.344227903	0.6342338	0.211450801	0	0	0	0.040975844	0.090935142	0.213595475	0.718442039	0.496660673	0.97620236	Pkib	protein kinase inhibitor beta, cAMP dependent, testis specific [Source:MGI Symbol;Acc:MGI:101937]
ENSMUSG00000019877	150.0158745	192.012777	120.0827282	153.2860378	180.4038864	205.2489718	41.85499026	44.85257109	53.49849345	144.9957105	140.0600205	145.3396598	Serinc1	serine incorporator 1 [Source:MGI Symbol;Acc:MGI:1926228]
ENSMUSG00000019878	10.24168526	13.64686698	8.889390574	12.65452418	14.51174691	14.49104535	17.58373726	18.23263762	21.12448429	21.78535845	22.91052183	21.03474602	Hsf2	heat shock factor 2 [Source:MGI Symbol;Acc:MGI:96239]
ENSMUSG00000019880	2.020014825	1.950965279	2.045838979	0.425655242	0.296672458	0.446692861	0.096958238	0.064552016	0.060649901	1.748569901	2.314265553	1.097211291	Rspo3	R-spondin 3 [Source:MGI Symbol;Acc:MGI:1920030]
ENSMUSG00000019883	0.149318061	0.630473883	0.229306103	0.185788214	0.392206348	0.568664326	0.533231057	0.345148564	0.486426942	1.135272577	0.766468706	1.146854276	Echdc1	enoyl Coenzyme A hydratase domain containing 1 [Source:MGI Symbol;Acc:MGI:1277169]
ENSMUSG00000019888	0	0	0	0	0	0.039086489	0.091627559	0.254179175	0.238814261	0.344257302	0.25629249	0.500252775	Mgat4c	MGAT4 family, member C [Source:MGI Symbol;Acc:MGI:1914819]
ENSMUSG00000019889	8.555800956	10.71432227	8.158380579	11.10657248	12.55101335	15.20213781	20.12456202	18.17268405	23.80820979	17.78690124	18.76354732	18.45518136	Ptprk	protein tyrosine phosphatase, receptor type, K [Source:MGI Symbol;Acc:MGI:103310]
ENSMUSG00000019890	0.034477955	0.039703138	0.035298277	0.064348522	0.072449228	0	0	0	0	0.246717733	0	0	Nts	neurotensin [Source:MGI Symbol;Acc:MGI:1328351]
ENSMUSG00000019891	2.309199543	2.506016525	2.339386144	2.83184123	2.540506791	2.909972049	13.43818301	14.38427735	16.37300238	11.90650491	12.2978988	12.88954323	Dcbld1	discoidin, CUB and LCCL domain containing 1 [Source:MGI Symbol;Acc:MGI:1913936]
ENSMUSG00000019892	0.057218734	0.021963438	0	0	0	0.029054983	0.085139304	0.037788872	0.035504567	0.221783494	0.349278041	0.185931716	Lrriq1	leucine-rich repeats and IQ motif containing 1 [Source:MGI Symbol;Acc:MGI:1922228]
ENSMUSG00000019893	0	0	0	0	0	0	0	0.024219232	0	0.005467041	0.010175249	0.005416606	Ros1	Ros1 proto-oncogene [Source:MGI Symbol;Acc:MGI:97999]
ENSMUSG00000019894	0.134045133	0.21224481	0.257314546	0.218905253	0.246462795	0.472211357	2.946933899	2.805205709	3.259451974	8.497927154	7.991849552	8.404682264	Slc6a15	solute carrier family 6 (neurotransmitter transporter), member 15 [Source:MGI Symbol;Acc:MGI:2143484]
ENSMUSG00000019897	25.58765928	21.04678928	23.29016391	27.94232077	23.57451395	22.46642083	28.36415344	29.93247507	25.8247353	25.56588819	23.38697924	23.55521044	Ccdc59	coiled-coil domain containing 59 [Source:MGI Symbol;Acc:MGI:1289302]
ENSMUSG00000019899	1.543270992	2.334399672	1.205076753	1.061809292	1.016843635	1.404600884	0.338611176	0.207298955	0.474746781	2.234407774	2.950265427	2.596282872	Lama2	laminin, alpha 2 [Source:MGI Symbol;Acc:MGI:99912]
ENSMUSG00000019900	0	0	0	0	0	0.024772502	0	0	0	0.058182867	0.040608702	0.129703757	Rfx6	regulatory factor X, 6 [Source:MGI Symbol;Acc:MGI:2445208]
ENSMUSG00000019905	0.050149753	0	0	0	0	0	0	0	0	0	0	0	Gprc6a	G protein-coupled receptor, family C, group 6, member A [Source:MGI Symbol;Acc:MGI:2429498]
ENSMUSG00000019906	0.044774701	0	0	0	0	0.01705204	0	0.044355721	0	0.40049913	0.335433797	0.41664464	Lin7a	lin-7 homolog A (C. elegans) [Source:MGI Symbol;Acc:MGI:2135609]

ENSMUSG00000019907	26.23502944	32.98925808	21.31623233	30.97047496	39.65957111	51.42554938	34.70057017	32.08700677	44.32813372	54.01909276	56.02613237	56.06294597	Ppp1r12a	protein phosphatase 1, regulatory subunit 12A [Source:MGI Symbol;Acc:MGI:1309528]
ENSMUSG00000019909	0	0	0	0.124314173	0	0	0	0	0	0	0.110888159	0.059029264	Fam162b	family with sequence similarity 162, member B [Source:MGI Symbol;Acc:MGI:1924546]
ENSMUSG00000019913	0.123727127	0.04383943	0.048719587	0.008881548	0.039998512	0.072492857	0.008496982	0.009428411	0.017716944	0.034052613	0.047534009	0.050607704	Sim1	single-minded family bHLH transcription factor 1 [Source:MGI Symbol;Acc:MGI:98306]
ENSMUSG00000019916	95.19845799	99.08312961	94.28255987	87.13665505	87.22477853	88.59293191	38.09431935	36.31952042	39.09804844	45.70137214	46.21940189	44.00703635	P4ha1	procollagen-proline, 2-oxoglutarate 4-dioxygenase (proline 4-hydroxylase), alpha 1 polypeptide [Source:MGI Symbol;Acc:MGI:9746
ENSMUSG00000019917	9.245988223	11.19747521	8.022841791	13.93443845	15.4124972	14.84930641	18.83415666	16.5438465	21.54782222	21.19936848	21.19977194	20.53798864	Septin10	septin 10 [Source:MGI Symbol;Acc:MGI:1918110]
ENSMUSG00000019920	32.26618172	40.18883605	24.81787011	34.32880466	45.75299688	52.84909545	24.43740114	21.34373772	28.17916204	45.42507453	46.03547321	47.67072519	Lims1	LIM and senescent cell antigen-like domains 1 [Source:MGI Symbol;Acc:MGI:1195263]
ENSMUSG00000019923	240.5130098	225.8554179	226.4257954	230.945632	222.1676085	208.496364	105.7012494	106.9667499	104.0270414	154.1636703	147.4348742	148.8742175	Zwint	ZW10 interactor [Source:MGI Symbol;Acc:MGI:1289227]
ENSMUSG00000019927	29.10077883	28.85754424	25.65594725	27.65443305	28.62570423	30.68329752	20.28522123	20.94784719	22.1181079	24.87054649	25.38703519	25.00149097	Ube2d1	ubiquitin-conjugating enzyme E2D 1 [Source:MGI Symbol;Acc:MGI:2384911]
ENSMUSG00000019929	1295.28439	1415.401077	1158.718815	1139.670553	1161.529248	1117.344241	34.54557829	31.28741965	36.28270377	257.4645764	242.6060147	250.5906909	Dcn	decorin [Source:MGI Symbol;Acc:MGI:94872]
ENSMUSG00000019932	0.075488575	0.195590193	0.057963487	0	0	0.057498282	0	0	0	0.050642061	0.015709156	0	Kera	keratocan [Source:MGI Symbol;Acc:MGI:1202398]
ENSMUSG00000019933	2.123483815	0.514800162	1.373057149	1.460126467	1.056817832	1.106655242	0.698451892	0.110716488	0.312071311	0.099968744	0.744246763	0.297139517	Mrln	myoregulin [Source:MGI Symbol;Acc:MGI:1916813]
ENSMUSG00000019935	0.06808305	0.172482491	0.153346441	0.381203903	0.257515737	0.311145766	0	0.013489192	0	0.024359472	0	0.012067375	Slc17a8	solute carrier family 17 (sodium-dependent inorganic phosphate cotransporter), member 8 [Source:MGI Symbol;Acc:MGI:3039629]
ENSMUSG00000019936	0.060575065	0.069755301	0.041344205	0.075370207	0.021214603	0.061518575	0	0	0	0	0	0	Epyc	epiphycan [Source:MGI Symbol;Acc:MGI:107942]
ENSMUSG00000019942	95.38040106	88.20056869	100.8194998	119.7052986	108.5295361	94.46314206	167.4485585	168.9001292	150.7983179	111.7189539	115.006435	114.3675343	Cdk1	cyclin-dependent kinase 1 [Source:MGI Symbol;Acc:MGI:88351]
ENSMUSG00000019943	40.17199167	53.41803788	32.97225459	56.69167979	63.40577609	84.30231956	22.018051	21.70151853	28.79644462	52.91870458	50.65266401	54.26674234	Atp2b1	ATPase, Ca++ transporting, plasma membrane 1 [Source:MGI Symbol;Acc:MGI:104653]
ENSMUSG00000019944	7.124929334	7.883541083	5.6849987	8.376152713	10.09661323	10.58347867	3.23707963	3.014202548	3.162396172	2.925720484	2.870415477	2.426843669	Rhobtb1	Rho-related BTB domain containing 1 [Source:MGI Symbol;Acc:MGI:1916538]
ENSMUSG00000019945	0.151109703	0.17401059	0.232057508	0.387786002	0.317529892	0.115097526	0.101180476	0.074847845	0	0.270328122	0.094337688	0.167396421	Cabcoco1	ciliary associated calcium binding coiled-coil 1 [Source:MGI Symbol;Acc:MGI:1920537]
ENSMUSG00000019947	9.547884774	10.40327744	7.651336991	7.933002942	9.471443132	12.21919445	20.35407452	17.5151039	24.24241274	38.18450189	38.35044135	36.96460664	Arid5b	AT rich interactive domain 5B (MRF1-like) [Source:MGI Symbol;Acc:MGI:2175912]
ENSMUSG00000019948	21.18139345	18.81627176	16.54282813	20.33087485	20.66484313	20.051616	34.5787716	40.94719266	37.12009965	33.61607189	32.49210223	34.37861028	Actr6	ARP6 actin-related protein 6 [Source:MGI Symbol;Acc:MGI:1914269]
ENSMUSG00000019951	28.46323984	37.54083816	24.35853267	36.23953671	40.48117474	53.21966577	17.92239315	16.95968597	23.26293205	38.28333385	38.14965381	38.0991139	Uhrf1bp1l	UHRF1 (ICBP90) binding protein 1-like [Source:MGI Symbol;Acc:MGI:2442888]
ENSMUSG00000019952	10.20178296	10.29518298	9.349521552	11.59306913	10.57453973	10.73667725	11.60525228	12.44272673	12.83921174	5.494776588	7.099457154	7.485618001	Poc1b	POC1 centriolar protein B [Source:MGI Symbol;Acc:MGI:1918511]
ENSMUSG00000019960	42.16639673	50.92183574	37.61444345	71.93080296	79.41425869	81.01768802	89.75538974	84.32231628	103.4622748	70.82965173	71.46490299	67.14267484	Dusp6	dual specificity phosphatase 6 [Source:MGI Symbol;Acc:MGI:1914853]
ENSMUSG00000019961	61.29020165	68.93629245	45.97718529	85.79251367	97.0468873	114.174087	128.1524162	114.4967521	142.9177406	139.1636715	138.7161862	139.2588398	Tmpo	thymopoietin [Source:MGI Symbol;Acc:MGI:106920]
ENSMUSG00000019966	48.8558042	70.69942979	44.50502423	68.94551212	88.6126772	107.793635	0.975467878	0.876226615	1.13803514	10.26190639	9.289866735	9.291149087	Kitl	kit ligand [Source:MGI Symbol;Acc:MGI:96974]
ENSMUSG00000019969	37.27524058	40.53039031	35.06856097	31.78441026	33.65225944	34.04447425	25.74658478	25.74313836	28.21440225	36.47707824	35.35407316	38.30428853	Psen1	presenilin 1 [Source:MGI Symbol;Acc:MGI:1202717]
ENSMUSG00000019970	91.54192575	96.84843054	71.90359959	107.5171879	110.7852197	114.2648822	96.1356348	91.27864676	102.5434319	197.4869718	199.1050924	192.4245037	Sgk1	serum/glucocorticoid regulated kinase 1 [Source:MGI Symbol;Acc:MGI:1340062]
ENSMUSG00000019971	2.438156581	3.595527153	2.126836017	4.237068346	5.672278162	8.41379888	2.532115273	2.08261586	3.658725609	5.797117803	5.363729835	5.523152717	Cep290	centrosomal protein 290 [Source:MGI Symbol;Acc:MGI:2384917]
ENSMUSG00000019975	98.93436703	115.0043062	92.95981679	94.61748719	103.9463989	116.2515342	14.83437647	15.87752815	17.49532524	41.08892307	40.48488339	39.51342014	Ikbip	IKBKB interacting protein [Source:MGI Symbol;Acc:MGI:1914704]
ENSMUSG00000019977	39.9018503	37.92509639	40.69879171	35.84635738	36.82366138	35.26892878	73.00151507	75.69406588	75.74694195	60.91486442	65.78407441	61.59321768	Hbs1l	Hbs1-like (S. cerevisiae) [Source:MGI Symbol;Acc:MGI:1891704]
ENSMUSG00000019978	45.39269046	55.85016047	37.7590716	51.22667659	59.40245284	70.16144345	70.89962863	63.01309249	81.02041852	99.04961728	101.4530335	95.02296815	Epb41l2	erythrocyte membrane protein band 4.1 like 2 [Source:MGI Symbol;Acc:MGI:103009]
ENSMUSG00000019979	18.23385425	23.84263821	15.29470865	24.31737024	27.37863504	38.8637149	16.60840226	16.64236892	19.69413722	26.02701092	26.69125572	25.51003228	Apaf1	apoptotic peptidase activating factor 1 [Source:MGI Symbol;Acc:MGI:1306796]
ENSMUSG00000019982	0.070050448	0.100833365	0	0.032684964	0.018399804	0	2.001262239	1.422596464	1.825602349	2.052064955	2.11373502	1.784812389	Myb	myeloblastosis oncogene [Source:MGI Symbol;Acc:MGI:97249]
ENSMUSG00000019984	16.31218148	19.42614861	12.36354172	19.10584308	22.07711919	24.82108471	22.59111885	19.91414819	27.11445146	36.19464195	35.39932123	35.69608695	Med23	mediator complex subunit 23 [Source:MGI Symbol;Acc:MGI:1917458]
ENSMUSG00000019986	5.74223592	7.185561429	5.722089438	7.93066911	10.41721926	10.55533405	7.296771659	7.944942254	8.961175808	10.4437987	10.5793618	11.73842084	Ahi1	Abelson helper integration site 1 [Source:MGI Symbol;Acc:MGI:87971]
ENSMUSG00000019987	42.97925615	46.43141109	45.36272904	4.180739683	4.034609647	3.862384583	1.846022736	2.341007654	1.557976146	31.31000468	33.23510986	33.50809016	Arg1	arginase, liver [Source:MGI Symbol;Acc:MGI:88070]
ENSMUSG00000019988	13.1632321	16.97958479	11.7747136	18.9384316	22.4492397	29.34346894	14.33675862	13.04005684	16.39395174	18.7284123	19.3478075	18.15173781	Nedd1	neural precursor cell expressed, developmentally down-regulated gene 1 [Source:MGI Symbol;Acc:MGI:97293]
ENSMUSG00000019989	2.285791887	2.347643956	1.739320719	2.248364157	2.174413015	2.42334826	36.40172819	37.69924219	41.48666593	34.95138368	32.29432563	34.6289479	Enpp3	ectonucleotide pyrophosphatase/phosphodiesterase 3 [Source:MGI Symbol;Acc:MGI:2143702]
ENSMUSG00000019990	0.811545767	1.640207215	0.84781082	0.525488163	0.887461215	1.147973779	0.103504226	0.180478955	0.231230667	2.69623167	2.68833361	2.686036012	Pde7b	phosphodiesterase 7B [Source:MGI Symbol;Acc:MGI:1352752]
ENSMUSG00000019992	3.547480162	4.507702914	4.633783248	5.707672756	5.205222155	5.660331922	19.00265702	17.75319599	22.68597993	17.26077241	15.4519143	16.42398027	Mtfr2	mitochondrial fission regulator 2 [Source:MGI Symbol;Acc:MGI:1919054]
ENSMUSG00000019996	1.327015816	1.337111062	0.984977067	0.990679484	1.045681997	1.566684101	29.73671048	26.71920908	31.3105934	22.73074961	22.00929048	22.78566009	Map7	microtubule-associated protein 7 [Source:MGI Symbol;Acc:MGI:1328328]
ENSMUSG00000019997	100.7863376	94.24067851	95.68229573	72.11048886	70.71576402	62.14660909	13.26017325	13.34501883	13.043531	81.1248649	74.26251246	77.75264282	Ccn2	cellular communication network factor 2 [Source:MGI Symbol;Acc:MGI:95537]
ENSMUSG00000019998	48.43303219	56.55962653	42.47135509	56.8078879	61.27614716	62.04219366	28.71172323	28.88791059	32.72477253	39.55043611	41.1313187	39.79085707	Stx7	syntaxin 7 [Source:MGI Symbol;Acc:MGI:1858210]
ENSMUSG00000020000	12.70574314	14.29367061	9.881112339	3.146609847	2.695555609	4.00137106	1.265225177	1.065041056	1.341794401	4.655273616	5.85761507	5.543454628	Moxd1	monooxygenase, DBH-like 1 [Source:MGI Symbol;Acc:MGI:1921582]
ENSMUSG00000020003	5.940963946	6.140017967	5.369742803	6.703844653	6.881797738	5.661139324	8.976781324	8.323244669	9.18516069	8.860459041	8.473142986	7.611160352	Pex7	peroxisomal biogenesis factor 7 [Source:MGI Symbol;Acc:MGI:1321392]
ENSMUSG00000020007	0	0	0.082597969	0	0	0	0.024009284	0	0	0.120274895	0.246241019	0.333662916	Il20ra	interleukin 20 receptor, alpha [Source:MGI Symbol;Acc:MGI:3605069]
ENSMUSG00000020009	50.88550616	50.44955463	50.49583938	43.87707951	44.81933664	40.35653784	8.667213729	9.006034744	10.79718999	13.72467634	15.92241596	14.5459168	Ifngr1	interferon gamma receptor 1 [Source:MGI Symbol;Acc:MGI:107655]
ENSMUSG00000020010	0.926621218	0.793448897	0.900018364	0.022171992	0.02496318	0.036194391	0.063635871	0.070611564	0.110571926	0.425046564	0.059332207	0.273731508	Vnn3	vanin 3 [Source:MGI Symbol;Acc:MGI:1347055]
ENSMUSG00000020014	0.223640789	0.291871714	0.198433559	0.375656238	0.250635168	0.363398704	0.386012047	0.354476904	0.56895884	0.360074529	0.322674554	0.237835164	Cfap54	cilia and flagella associated protein 54 [Source:MGI Symbol;Acc:MGI:1922208]
ENSMUSG00000020015	21.29637815	26.15461774	17.70454359	28.89525589	33.39120729	40.86381054	23.58380282	19.4783937	30.11290633	33.15324192	33.34596445	28.72035227	Cdk17	cyclin-dependent kinase 17 [Source:MGI Symbol;Acc:MGI:97517]
ENSMUSG00000020017	1.418521577	1.132560357	1.645908249	0.882494018	0.953845838	0.921994427	2.44841927	1.667560635	2.376543057	2.114723427	2.629194815	1.910835664	Hal	histidine ammonia lyase [Source:MGI Symbol;Acc:MGI:96010]
ENSMUSG00000020018	0	0.057548799	0	0.093271726	0.105013515	0.228390388	0.133849666	0.148522117	0.139544082	0.402313237	0.332793268	0.354312703	Snrpf	small nuclear ribonucleoprotein polypeptide F [Source:MGI Symbol;Acc:MGI:1917128]
ENSMUSG00000020019	6.615070267	7.500399716	6.27234026	3.020058172	2.715920733	2.992140957	0.436119441	0.725889554	0.378894527	3.677658092	3.236056394	3.896266515	Ntn4	netrin 4 [Source:MGI Symbol;Acc:MGI:1888978]
ENSMUSG00000020020	0.893104269	0.936629094	0.718420685	0.818547547	0.821055448	0.959654422	5.780737862	5.055697281	7.273567178	11.08419195	11.33714294	9.794700871	Usp44	ubiquitin specific peptidase 44 [Source:MGI Symbol;Acc:MGI:3045318]
ENSMUSG00000020021	4.500465371	4.750640787	3.898690597	5.761209693	6.607719343	9.009284922	5.292827698	4.429772976	5.262912302	6.618949631	7.936625777	7.184275037	Fgd6	FYVE, RhoGEF and PH domain containing 6 [Source:MGI Symbol;Acc:MGI:1261419]
ENSMUSG00000020022	135.0000323	103.7531667	151.7859892	123.3121123	111.3377829	83.30532851	149.6111155	170.796352	107.2106152	94.30213066	87.42928024	80.24071697	Ndufa12	NADH:ubiquinone oxidoreductase subunit A12 [Source:MGI Symbol;Acc:MGI:1913664]
ENSMUSG00000020023	9.934779989	12.10584757	8.032254297	16.09457583	18.23744661	25.07144864	2.400928379	1.849468599	2.130714156	16.81860514	16.55790473	16.23012059	Tmcc3	transmembrane and coiled coil domains 3 [Source:MGI Symbol;Acc:MGI:2442900]
ENSMUSG00000020024	11.1492076	12.98193733	9.427276928	12.60682992	13.32919383	15.03276082	20.55908646	20.47455911	24.26651945	18.29254843	17.51420652	17.1742566	Cep83	centrosomal protein 83 [Source:MGI Symbol;Acc:MGI:1924298]
ENSMUSG00000020027	4.882020113	5.866327431	4.284151182	5.687701934	7.391352965	7.621865264	50.70544163	45.05903562	54.78182037	36.04686914	37.12940369	36.3861543	Socs2	suppressor of cytokine signaling 2 [Source:MGI Symbol;Acc:MGI:1201787]
ENSMUSG00000020029	56.0576748	56.943353	52.45099696	61.7938257	65.60135416	70.83680847	71.0854309	75.41189691	71.14026853	58.41538074	56.54826322	57.00928648	Nudt4	nudix (nucleoside diphosphate linked moiety X)-type motif 4 [Source:MGI Symbol;Acc:MGI:1918457]
ENSMUSG00000020032	14.48540213	17.37024106	13.92506517	6.931730269	7.894229215	8.448703796	6.058804022	4.675001852	6.515617428	18.30027559	18.14579278	17.3354358	Nuak1	NUAK family, SNF1-like kinase, 1 [Source:MGI Symbol;Acc:MGI:1925226]
ENSMUSG00000020033	0	0	0.068192338	0	0.034990958	0	0	0	0	0.059578895	0	0	4930463O1	RIKEN cDNA 4930463O16 gene [Source:MGI Symbol;Acc:MGI:1923054]
ENSMUSG00000020034	9.597040177	10.65678921	9.393494527	8.562138656	9.390698481	10.60335962	4.236932434	4.41407369	5.177923389	6.957077255	7.044863375	7.920989393	Tcp11l2	t-complex 11 (mouse) like 2 [Source:MGI Symbol;Acc:MGI:2444679]
ENSMUSG00000020037	0	0	0	0	0.022171809	0.016073576	0.188400461	0.543537048	0.294623479	0.453021674	0.544542982	0.579754804	Rfx4	regulatory factor X, 4 (influences HLA class II expression) [Source:MGI Symbol;Acc:MGI:1918387]
ENSMUSG00000020038	13.54832853	14.06069831	9.503984008	13.8137329	14.23468916	16.37108514	62.23281024	65.07794629	71.30227948	43.91144074	46.99006987	45.87807192	Cry1	cryptochrome 1 (photolyase-like) [Source:MGI Symbol;Acc:MGI:1270841]
ENSMUSG00000020042	3.201081274	2.863241906	2.972383664	4.543317388	4.864978203	5.148583173	4.359886552	3.908598492	5.432272456	4.341549148	4.994533155	4.776509169	Btbd11	BTB (POZ) domain containing 11 [Source:MGI Symbol;Acc:MGI:1921257]
ENSMUSG00000020044	25.90394265	64.899212	16.19823855	33.38781944	56.45584643	98.62406035	1.668745306	1.346670046	1.581581236	26.4467553	27.31449302	25.76773726	Timp3	tissue inhibitor of metalloproteinase 3 [Source:MGI Symbol;Acc:MGI:98754]
ENSMUSG00000020048	261.2026004	342.0999005	236.6515196	312.4178816	364.9635688	380.5675524	193.5462858	173.3222818	205.9140244	299.9825364	274.2713765	274.872724	Hsp90b1	heat shock protein 90, beta (Grp94), member 1 [Source:MGI Symbol;Acc:MGI:98817]

ENSMUSG00000020051	0.125937038	2.513731524	0.128933415	0.313392998	0.088211353	0.127898617	0.187389532	0.706965279	0.078144686	0.150196942	0.209659759	0.037202834	Pah	phenylalanine hydroxylase [Source:MGI Symbol;Acc:MGI:97473]
ENSMUSG00000020052	0.05202961	0	0	0	0.054665439	0.039630013	0	0	0	0.046539258	0	0.076849868	Ascl1	achaete-scute family bHLH transcription factor 1 [Source:MGI Symbol;Acc:MGI:96919]
ENSMUSG00000020053	14.10913756	16.42399932	13.21118909	8.443679402	10.12813649	10.01242993	0.156475984	0.368961004	0.4078325	4.232892432	4.030310326	4.562745724	Igf1	insulin-like growth factor 1 [Source:MGI Symbol;Acc:MGI:96432]
ENSMUSG00000020056	40.87482003	30.7424002	43.68476532	33.7054741	29.41604864	24.16186923	32.24604539	32.49165242	28.98238911	25.00313144	25.73287508	23.53981186	Washc3	WASH complex subunit 3 [Source:MGI Symbol;Acc:MGI:1914532]
ENSMUSG00000020057	31.01093542	32.23221016	25.63040012	18.2313013	20.1382509	19.89614788	1.028276397	0.990297472	0.970888522	5.403865986	4.196724804	5.180682506	Dram1	DNA-damage regulated autophagy modulator 1 [Source:MGI Symbol;Acc:MGI:1918962]
ENSMUSG00000020059	3.831850493	4.41257282	4.045614814	6.630166099	6.542205624	6.262939009	2.31629454	2.174788148	1.560356553	2.713437332	1.794166304	2.157798874	Sycp3	synaptonemal complex protein 3 [Source:MGI Symbol;Acc:MGI:109542]
ENSMUSG00000020061	0	0	0.038557768	0.035145276	0.019784822	0.043029349	0.235364533	0.149237104	0.087634905	0.656906209	0.391869535	0.750976241	Mybpc1	myosin binding protein C, slow-type [Source:MGI Symbol;Acc:MGI:1336213]
ENSMUSG00000020062	0.008071373	0.130124408	0.033053649	0.045192434	0.059361889	0.006147819	0.057647502	0.071962602	0.015025005	0.064976871	0.107497657	0.085836589	Slc5a8	solute carrier family 5 (iodide transporter), member 8 [Source:MGI Symbol;Acc:MGI:2384916]
ENSMUSG00000020063	3.763338874	3.571898803	2.528451668	5.130645955	4.849816181	6.471945312	24.97556109	23.51921559	29.54086715	37.4623219	36.09832001	36.90827434	Sirt1	sirtuin 1 [Source:MGI Symbol;Acc:MGI:2135607]
ENSMUSG00000020064	24.93337803	27.31886497	23.07627043	28.66703063	31.94426407	34.19636505	90.09645176	85.09536589	102.252632	85.86862913	90.1332985	85.14639415	Herc4	hect domain and RLD 4 [Source:MGI Symbol;Acc:MGI:1914595]
ENSMUSG00000020067	0.230943861	0.279940766	0.049776554	0.170141855	0.17878996	0.249971344	1.171978599	0.710430795	0.905065571	1.01112454	1.264720179	1.120288725	Mypn	myopalladin [Source:MGI Symbol;Acc:MGI:1916052]
ENSMUSG00000020069	15.36731708	17.00300021	12.35928374	17.23820146	17.17782936	17.88332054	67.26842635	59.51296557	61.51879976	28.17160425	30.27818339	29.03492154	Hnrnph3	heterogeneous nuclear ribonucleoprotein H3 [Source:MGI Symbol;Acc:MGI:1926462]
ENSMUSG00000020070	6.09407995	7.309114069	5.003219366	8.503089424	9.225769944	13.24307712	11.10728134	9.142376385	11.32611751	17.06706201	15.46122136	16.5990291	Rufy2	RUN and FYVE domain-containing 2 [Source:MGI Symbol;Acc:MGI:1917682]
ENSMUSG00000020072	1.106956948	1.439197549	1.023620751	1.499507471	1.463173789	1.332719511	0.573831845	1.131972719	0.897366881	1.980293705	2.169819012	1.297468126	Pbld2	phenazine biosynthesis-like protein domain containing 2 [Source:MGI Symbol;Acc:MGI:1914557]
ENSMUSG00000020074	38.10381385	44.08757007	31.55219186	45.46915449	50.43016525	60.06841863	57.13601713	57.93608097	71.64911496	92.44419771	87.74756677	89.86171843	Ccar1	cell division cycle and apoptosis regulator 1 [Source:MGI Symbol;Acc:MGI:1914750]
ENSMUSG00000020075	51.58936405	55.09802909	45.65832182	58.72446133	63.15036969	73.34894098	205.4794538	193.8588032	245.1550405	264.143715	270.7718477	266.0763146	Ddx21	DEAD (Asp-Glu-Ala-Asp) box polypeptide 21 [Source:MGI Symbol;Acc:MGI:1860494]
ENSMUSG00000020076	43.75739816	47.79206854	41.79017065	45.06372314	44.53821093	45.2486214	44.84105518	43.23511074	48.50844822	44.60040148	44.59570732	45.54146796	Ddx50	DEAD (Asp-Glu-Ala-Asp) box polypeptide 50 [Source:MGI Symbol;Acc:MGI:2182303]
ENSMUSG00000020077	19.84185868	19.06252775	21.88102546	1.798706517	1.131697034	1.165874252	6.529006818	4.155879749	4.590614206	17.34240382	15.33660676	18.3881991	Srgn	serglycin [Source:MGI Symbol;Acc:MGI:97756]
ENSMUSG00000020078	41.66046834	49.08183515	32.80393594	43.3450111	46.15156195	50.73216508	53.91056272	52.47235499	59.02922631	53.5925334	51.93713836	50.29451662	Vps26a	VPS26 retromer complex component A [Source:MGI Symbol;Acc:MGI:1353654]
ENSMUSG00000020079	15.96834993	16.1861751	17.22932452	16.47779757	16.37545754	13.93502803	52.13037975	51.71936188	54.73384041	37.17652455	39.71693525	35.02577912	Supv3l1	suppressor of var1, 3-like 1 (S. cerevisiae) [Source:MGI Symbol;Acc:MGI:2441711]
ENSMUSG00000020080	0	0.124967253	0.027775694	0.025317451	0	0.020664578	0.072663658	0.134381613	0.202013375	0.072801954	0.270997797	0.168304119	Hkdc1	hexokinase domain containing 1 [Source:MGI Symbol;Acc:MGI:2384910]
ENSMUSG00000020081	0.038112035	0.175551871	0.117056466	0.088913813	0.020021399	0.101602367	0.187140565	0.094388502	0.088682798	0.221587052	0.142759911	0.084439559	Tacr2	tachykinin receptor 2 [Source:MGI Symbol;Acc:MGI:98477]
ENSMUSG00000020083	3.076743527	2.221002835	3.761131287	2.528340653	2.364148985	1.923768554	29.88733044	25.52091842	23.03787905	9.20096528	8.829987445	8.302147873	Fam241b	family with sequence similarity 241, member B [Source:MGI Symbol;Acc:MGI:1917144]
ENSMUSG00000020085	27.871881	22.99815973	28.88216877	27.90809098	22.83572266	23.65721269	30.33229612	25.89797366	29.79227663	16.3171993	15.94683581	17.87949432	Aifm2	apoptosis-inducing factor, mitochondrion-associated 2 [Source:MGI Symbol;Acc:MGI:1918611]
ENSMUSG00000020086	8.012201182	8.588582708	7.93953199	8.270693366	8.210246321	7.609600194	17.97991791	18.06943981	19.83123503	8.865503978	9.435894092	8.573328916	Macroh2a2	macroH2A.2 histone [Source:MGI Symbol;Acc:MGI:3037658]
ENSMUSG00000020087	31.72478778	29.67614233	33.09871772	27.26095576	20.86913723	18.07000057	37.45967392	38.24805794	31.08680086	27.00398938	27.60803483	29.88795232	Tysnd1	trypsin domain containing 1 [Source:MGI Symbol;Acc:MGI:1919017]
ENSMUSG00000020088	372.9135612	352.759926	358.1586926	318.4738119	311.869181	297.3489335	234.1250983	232.4248897	240.0751607	253.6298909	255.4347539	258.0901944	Sar1a	secretion associated Ras related GTPase 1A [Source:MGI Symbol;Acc:MGI:98230]
ENSMUSG00000020089	90.1642404	69.70715948	93.46085354	71.40931001	69.66190873	62.15890959	301.1637253	313.4937344	287.1706206	256.3823396	256.9860816	246.1738995	Ppa1	pyrophosphatase (inorganic) 1 [Source:MGI Symbol;Acc:MGI:97831]
ENSMUSG00000020090	0.185520931	0.028484918	0.088636318	0.092333378	0.168930192	0.141307936	0.011041924	0	0	0.033188818	0	0.021921762	Npffr1	neuropeptide FF receptor 1 [Source:MGI Symbol;Acc:MGI:2685082]
ENSMUSG00000020091	24.86408872	27.44957901	21.6776685	25.86628422	26.57541445	28.8262202	90.02915009	79.0287712	97.2717527	67.3619528	69.6555222	65.40698057	Eif4ebp2	eukaryotic translation initiation factor 4E binding protein 2 [Source:MGI Symbol;Acc:MGI:109198]
ENSMUSG00000020092	13.83915132	15.38410268	14.07020031	15.80175884	15.37183623	15.67453161	72.86831015	68.33441359	76.14696535	57.41014382	60.53331463	57.85828682	Pald1	phosphatase domain containing, paladin 1 [Source:MGI Symbol;Acc:MGI:1351623]
ENSMUSG00000020096	0.157325367	0.181168247	0.161068556	0.293626895	0.206619371	0.179747828	0	0.077926595	0	0.070361913	0.065478749	0	Tbata	thymus, brain and testes associated [Source:MGI Symbol;Acc:MGI:1923820]
ENSMUSG00000020097	96.87805196	133.3300284	81.66166238	120.1023466	140.5973325	166.9682419	48.87502242	43.41146083	59.38322467	103.1303594	110.3679344	103.6752516	Sgpl1	sphingosine phosphate lyase 1 [Source:MGI Symbol;Acc:MGI:1261415]
ENSMUSG00000020098	2.244964582	3.533095897	2.834666534	2.164796196	0.786231623	0.740977858	14.02977279	16.82794335	13.16398198	6.89436615	7.786277278	7.029718082	Pcbd1	pterin 4 alpha carbinolamine dehydratase/dimerization cofactor of hepatocyte nuclear factor 1 alpha (TCF1) 1 [Source:MGI Symbol;A
ENSMUSG00000020099	10.02416327	10.84078611	9.022122924	7.013811508	7.683099396	9.694088026	5.862842045	5.347117957	6.372531396	23.8067551	25.57542152	25.51705595	Unc5b	unc-5 netrin receptor B [Source:MGI Symbol;Acc:MGI:894703]
ENSMUSG00000020100	9.736121595	11.27117536	8.715182859	8.58708737	11.1527129	12.98097974	8.261424364	6.896612924	9.366721862	15.05916826	13.88495613	15.11877278	Slc29a3	solute carrier family 29 (nucleoside transporters), member 3 [Source:MGI Symbol;Acc:MGI:1918529]
ENSMUSG00000020101	7.346897867	8.271724468	6.587476239	2.510957015	3.392467609	2.887107854	0.898226604	0.950331569	1.263105377	8.559857665	9.270708389	9.579881439	Vsir	V-set immunoregulatory receptor [Source:MGI Symbol;Acc:MGI:1921298]
ENSMUSG00000020102	0.22851743	0.650134226	0.151382303	0.100352334	0.098862302	0.174057592	0.180013374	0.146480576	0.08758015	0.252498374	0.179028431	0.1191281	Slc16a7	solute carrier family 16 (monocarboxylic acid transporters), member 7 [Source:MGI Symbol;Acc:MGI:1330284]
ENSMUSG00000020105	45.33285708	54.95064766	41.69305386	52.65624321	52.7073285	57.84381652	8.538657137	7.373665597	10.34444909	18.87916834	18.8590278	17.98210434	Lrig3	leucine-rich repeats and immunoglobulin-like domains 3 [Source:MGI Symbol;Acc:MGI:2443955]
ENSMUSG00000020107	44.86799567	33.84655575	46.59057688	33.80952688	31.38532882	22.84434524	27.66869136	28.72094757	26.94757036	25.96864646	24.19969189	27.50105394	Anapc16	anaphase promoting complex subunit 16 [Source:MGI Symbol;Acc:MGI:1289325]
ENSMUSG00000020108	29.47022357	27.89454213	25.10099726	23.33706404	20.83961797	19.92585756	96.8798542	90.30364772	83.52130745	58.22538502	58.4702839	57.88379378	Ddit4	DNA-damage-inducible transcript 4 [Source:MGI Symbol;Acc:MGI:1921997]
ENSMUSG00000020109	13.65002943	14.92091593	14.12815857	14.64256502	13.98743009	15.06064656	10.86582034	10.49879452	11.2758055	15.04011915	14.68211345	16.69351702	Dnajb12	DnaJ heat shock protein family (Hsp40) member B12 [Source:MGI Symbol;Acc:MGI:1931881]
ENSMUSG00000020111	45.14825338	39.91224579	41.99478746	37.84137738	37.51451625	34.05310909	16.63906347	15.68129394	14.83586611	23.8856861	22.77797455	22.68944425	Micu1	mitochondrial calcium uptake 1 [Source:MGI Symbol;Acc:MGI:2384909]
ENSMUSG00000020114	61.59978698	68.16346188	52.59648002	60.18922709	69.72307816	78.91727223	81.56686042	75.95720676	90.17191568	117.2240674	121.7832267	121.234298	Cand1	cullin associated and neddylation disassociated 1 [Source:MGI Symbol;Acc:MGI:1261820]
ENSMUSG00000020115	40.27245978	38.83825124	32.0604447	39.0925773	41.95071334	47.99334167	30.0798457	32.01204575	36.65026436	38.8885617	38.97128405	42.28508646	Tbk1	TANK-binding kinase 1 [Source:MGI Symbol;Acc:MGI:1929658]
ENSMUSG00000020116	38.82648706	35.27499696	34.97507755	32.82076264	34.43602374	29.60543856	89.8472417	91.53734286	83.540027	134.2192655	125.5689287	122.5913303	Pno1	partner of NOB1 homolog [Source:MGI Symbol;Acc:MGI:1913499]
ENSMUSG00000020120	2.721169191	3.596479395	2.152750998	0.173137526	0.406111376	0.423954273	0.33128153	0.214431142	0.359766004	6.818026382	6.859645933	7.741678458	Plek	pleckstrin [Source:MGI Symbol;Acc:MGI:1860485]
ENSMUSG00000020121	4.08731548	4.462414202	4.058798033	4.481182317	5.62023994	6.992017899	1.734800054	1.427130639	1.985307018	2.187610858	2.147339277	2.494028544	Srgap1	SLIT-ROBO Rho GTPase activating protein 1 [Source:MGI Symbol;Acc:MGI:2152936]
ENSMUSG00000020122	20.82258463	25.70538323	17.42709999	13.95520194	17.7314521	22.06007683	1.48199437	1.644448947	2.222694027	18.31271767	17.27209731	18.18249161	Egfr	epidermal growth factor receptor [Source:MGI Symbol;Acc:MGI:95294]
ENSMUSG00000020123	2.965259086	3.878595043	3.01931153	0.406046405	0.60955036	0.527821306	0.043162757	0.031929474	0	0.619843653	0.590240645	0.656971392	Avpr1a	arginine vasopressin receptor 1A [Source:MGI Symbol;Acc:MGI:1859216]
ENSMUSG00000020124	28.95777156	31.05604342	23.8875948	32.96491109	36.7873139	39.22933826	42.60219242	41.12607658	48.31041148	52.22892711	52.99019625	55.16709288	Usp15	ubiquitin specific peptidase 15 [Source:MGI Symbol;Acc:MGI:101857]
ENSMUSG00000020125	0.393553242	0.100710398	0	0.285644659	0.275660478	0.266455452	0	0	0	0	0.036399264	0	Elane	elastase, neutrophil expressed [Source:MGI Symbol;Acc:MGI:2679229]
ENSMUSG00000020128	12.73328484	14.80719108	10.63772127	12.41653679	15.61434437	17.66745834	18.55280982	17.04325249	24.6853249	37.86409659	41.8905601	42.01593114	Vps54	VPS54 GARP complex subunit [Source:MGI Symbol;Acc:MGI:2178798]
ENSMUSG00000020130	34.01148854	44.50955264	31.87849723	45.64013565	53.51867099	60.30660783	47.71256691	42.65196648	52.07371295	66.00969232	64.96198004	68.11116248	Tbc1d15	TBC1 domain family, member 15 [Source:MGI Symbol;Acc:MGI:1913937]
ENSMUSG00000020131	4.092683932	3.133465512	4.031516985	3.757292013	4.439480105	2.527559432	4.147619206	4.120133351	3.829893153	1.939239437	1.93355883	2.156616718	Pcsk4	proprotein convertase subtilisin/kexin type 4 [Source:MGI Symbol;Acc:MGI:97514]
ENSMUSG00000020132	63.61832244	66.6179863	57.39896574	74.27582367	82.04903022	81.74849363	16.44940134	16.07666015	16.56090511	27.56207654	28.74031323	28.51215349	Rab21	RAB21, member RAS oncogene family [Source:MGI Symbol;Acc:MGI:894308]
ENSMUSG00000020133	32.59101352	27.85470041	34.32816343	30.12119593	26.04481651	21.62409609	13.76784643	14.60497511	11.26913663	15.40452262	14.50920122	14.61491556	2310011J03I	RIKEN cDNA 2310011J03 gene [Source:MGI Symbol;Acc:MGI:1913624]
ENSMUSG00000020134	6.329426685	8.291686825	4.741115996	6.245193415	7.855021575	8.757430616	9.577792564	7.765842698	10.89055875	17.27024132	17.75134618	17.70272498	Peli1	pellino 1 [Source:MGI Symbol;Acc:MGI:1914495]
ENSMUSG00000020135	2.267104871	2.222431665	1.963961438	1.898637833	2.284235485	2.576935965	0.394424333	0.40310853	0.5843432	2.027871776	1.974234693	1.658848326	Apc2	APC regulator of WNT signaling pathway 2 [Source:MGI Symbol;Acc:MGI:1346052]
ENSMUSG00000020137	5.795271979	6.592563864	6.249976001	8.453363755	8.70470726	9.535429345	1.155333957	1.268045813	1.24376247	2.667358245	3.055967473	2.829738269	Thap2	THAP domain containing, apoptosis associated protein 2 [Source:MGI Symbol;Acc:MGI:1914066]
ENSMUSG00000020140	0.393444195	0.432477069	0.40280528	0.016688894	0.056369474	0.05448715	0	0	0.016645533	0.111976627	0.163751301	0.253585401	Lgr5	leucine rich repeat containing G protein coupled receptor 5 [Source:MGI Symbol;Acc:MGI:1341817]
ENSMUSG00000020142	8.287867237	9.188073813	8.360775877	11.21496213	11.60644439	12.58340828	1.389200929	1.924121363	1.93106963	4.955362688	4.5104093	4.626023062	Slc1a4	solute carrier family 1 (glutamate/neutral amino acid transporter), member 4 [Source:MGI Symbol;Acc:MGI:2135601]
ENSMUSG00000020143	0.756515671	0.855883058	0.720163251	0.049541614	0.097612038	0.202183964	1.729971837	1.577761173	1.815923897	1.804494413	2.176411002	2.011328409	Dock2	dedicator of cyto-kinesis 2 [Source:MGI Symbol;Acc:MGI:2149010]
ENSMUSG00000020149	138.3545855	152.5225659	121.3905637	129.8786085	137.4540505	138.2816387	180.6905356	173.2068344	187.4361602	244.3743868	240.9071636	233.8011991	Rab1a	RAB1A, member RAS oncogene family [Source:MGI Symbol;Acc:MGI:97842]
ENSMUSG00000020150	38.05560906	32.18910139	44.37379947	42.79129755	34.20872751	30.70072493	2.990937543	3.318800794	2.338636585	4.214010917	4.444429804	3.850402785	Gamt	guanidinoacetate methyltransferase [Source:MGI Symbol;Acc:MGI:1098221]
ENSMUSG00000020151	0.214963303	0.123770647	0.073359284	0.234033593	0.056463345	0.218311545	0.015992862	0.141967852	0	0.0160233	0.029822544	0.015875481	Ptprr	protein tyrosine phosphatase, receptor type, R [Source:MGI Symbol;Acc:MGI:109559]
ENSMUSG00000020152	120.5508874	139.83225	104.8516368	133.519586	145.3391392	162.6592011	186.7891932	175.2902507	216.3082515	269.3409217	267.1378238	275.42079	Actr2	ARP2 actin-related protein 2 [Source:MGI Symbol;Acc:MGI:1913963]

ENSMUSG00000020153	119.6710073	80.8362876	142.1833274	111.6160551	86.49991014	68.57999913	118.4034929	139.3946591	99.15252492	67.02896933	68.26770099	74.58594574	Ndufs7	NADH:ubiquinone oxidoreductase core subunit S7 [Source:MGI Symbol;Acc:MGI:1922656]
ENSMUSG00000020154	0.472925722	0.660660208	0.484177885	0.296629296	0.252514931	0.354313737	0.089980739	0.076803329	0.115457013	0.436890429	0.548546742	0.487826348	Ptprb	protein tyrosine phosphatase, receptor type, B [Source:MGI Symbol;Acc:MGI:97809]
ENSMUSG00000020155	0.030800636	0	0.084089239	0.009580882	0.032361001	0.015640191	0.064162247	0.061024836	0.057335936	0.247953971	0.307661064	0.391246696	Kcnmb1	potassium large conductance calcium-activated channel, subfamily M, beta member 1 [Source:MGI Symbol;Acc:MGI:1334203]
ENSMUSG00000020156	37.3919852	36.23803385	37.8121668	43.70167592	43.7428622	38.63899858	42.66232239	43.1558316	43.94380702	37.85561524	40.85859374	40.3680483	Pwwp3a	PWWP domain containing 3A, DNA repair factor [Source:MGI Symbol;Acc:MGI:1915364]
ENSMUSG00000020159	0	0	0	0	0	0.052466415	2.172878619	2.388321243	3.120158016	2.341317058	3.115341891	3.031911846	Gabrp	gamma-aminobutyric acid (GABA) A receptor, pi [Source:MGI Symbol;Acc:MGI:2387597]
ENSMUSG00000020160	11.17878883	11.51193324	11.39434454	12.16667612	11.69336761	13.23151448	0.703447972	0.780559157	0.618785174	2.345618551	2.049606608	1.669337458	Meis1	Meis homeobox 1 [Source:MGI Symbol;Acc:MGI:104717]
ENSMUSG00000020163	126.94638	100.8991093	200.8883685	133.1283567	115.0521448	68.93197457	162.5469829	166.9172774	87.14327807	90.07508765	78.96148648	89.02538552	Uqcr11	ubiquinol-cytochrome c reductase, complex III subunit XI [Source:MGI Symbol;Acc:MGI:1913844]
ENSMUSG00000020164	0	0	0	0.194919795	0	0	0	0	0	0	0	0.092555594	Kcnmb4os1	potassium large conductance calcium-activated channel, subfamily M, beta member 4, opposite strand 1 [Source:MGI Symbol;Acc:M
ENSMUSG00000020166	54.50060893	57.72759102	47.83567721	60.36516123	64.25050771	69.21951483	63.43184642	59.7525886	66.69873629	76.34356884	76.90329873	73.73121004	Cnot2	CCR4-NOT transcription complex, subunit 2 [Source:MGI Symbol;Acc:MGI:1919318]
ENSMUSG00000020167	86.70689506	89.06145723	90.68775096	100.3569375	91.68190727	96.72048827	79.56021754	77.43096013	79.60013501	63.74195408	66.0965352	64.40750939	Tcf3	transcription factor 3 [Source:MGI Symbol;Acc:MGI:98510]
ENSMUSG00000020168	0	0	0	0	0	0.011978809	0	0	0	0	0	0	Olfr299	olfactory receptor 299 [Source:MGI Symbol;Acc:MGI:3030133]
ENSMUSG00000020169	0.189901381	0.072893734	0.14581473	0.118141899	0.049880454	0.048214815	0	0.031354062	0.044188104	0.056620767	0.065864059	0.070123032	Best3	bestrophin 3 [Source:MGI Symbol;Acc:MGI:3580298]
ENSMUSG00000020170	21.86780585	29.90478162	17.88152144	26.84047183	34.54703046	45.60624811	14.17277689	11.38323788	16.55277175	28.06743645	30.76955079	29.28118277	Frs2	fibroblast growth factor receptor substrate 2 [Source:MGI Symbol;Acc:MGI:1100860]
ENSMUSG00000020171	51.321688	45.81254098	48.07191549	54.09877321	47.06042823	42.44754442	91.45882855	94.73733562	91.39708602	62.67733071	60.86200908	64.12292911	Yeats4	YEATS domain containing 4 [Source:MGI Symbol;Acc:MGI:1927224]
ENSMUSG00000020173	0.727383356	0.303275963	0.256789567	0.5383445	0.408469213	1.088964836	36.5674478	32.26444957	45.33690018	43.23305812	45.07644078	42.94536395	Cobl	cordon-bleu WH2 repeat [Source:MGI Symbol;Acc:MGI:105056]
ENSMUSG00000020175	3.732714927	3.827354148	3.900050671	4.676222018	4.593358549	3.894714277	0.091300901	0.278600312	0.333148022	1.006221343	1.340738654	1.223515658	Rab36	RAB36, member RAS oncogene family [Source:MGI Symbol;Acc:MGI:1924127]
ENSMUSG00000020176	72.60257733	80.4627169	59.87875237	55.21600644	62.90632206	72.69401208	25.41319633	21.52415203	29.35217795	52.33447955	49.78510194	50.28328017	Grb10	growth factor receptor bound protein 10 [Source:MGI Symbol;Acc:MGI:103232]
ENSMUSG00000020177	0	0.13119907	0	0.035439963	0.079802857	0.057853523	0	0	0.035347883	0	0	0.03365658	9530003J23I	RIKEN cDNA 9530003J23 gene [Source:MGI Symbol;Acc:MGI:1924647]
ENSMUSG00000020178	0.743294415	1.09370332	0.676426107	0.732165143	0.564019327	0.503247918	2.101388371	2.127201613	2.113918746	0.55404942	0.99682283	0.585534045	Adora2a	adenosine A2a receptor [Source:MGI Symbol;Acc:MGI:99402]
ENSMUSG00000020180	77.51982456	68.85265784	93.24020806	83.06914278	76.43839489	60.18458822	223.3402341	228.6548007	157.4125956	127.8380172	116.9430062	120.9981089	Snrpd3	small nuclear ribonucleoprotein D3 [Source:MGI Symbol;Acc:MGI:1914582]
ENSMUSG00000020181	2.08957303	2.069375653	2.036603642	3.057529781	3.688324341	5.322276856	0.097007207	0.082800792	0.054456888	0.575674711	0.549637367	0.711103118	Nav3	neuron navigator 3 [Source:MGI Symbol;Acc:MGI:2183703]
ENSMUSG00000020182	2.024870015	2.498294905	1.295654418	0.944787714	1.177695688	1.432142078	12.39605375	13.25341483	13.05804268	24.67757844	23.23582174	23.26427631	Ddc	dopa decarboxylase [Source:MGI Symbol;Acc:MGI:94876]
ENSMUSG00000020183	0.702751784	0.531796015	0.61669039	0.37474081	0.443011986	0.611740936	0.645326527	0.716066504	0.934417908	1.957624048	2.139318741	2.366624509	Cpm	carboxypeptidase M [Source:MGI Symbol;Acc:MGI:1917824]
ENSMUSG00000020184	134.3459196	159.9699496	119.5293103	172.7433595	194.7311422	211.154165	126.5752343	117.1515138	146.8627343	243.9474664	249.1451617	237.6234417	Mdm2	transformed mouse 3T3 cell double minute 2 [Source:MGI Symbol;Acc:MGI:96952]
ENSMUSG00000020185	3.911680712	6.209595178	3.597487304	10.06414853	9.07881483	10.92467355	13.45605058	13.28910609	17.83390569	14.96424784	14.05449043	15.70754472	E2f7	E2F transcription factor 7 [Source:MGI Symbol;Acc:MGI:1289147]
ENSMUSG00000020186	123.6522582	90.10964162	137.5913533	131.8246253	114.2346622	90.37828623	483.7818415	534.3388194	412.2911927	164.2302194	164.7888175	165.0949995	Csrp2	cysteine and glycine-rich protein 2 [Source:MGI Symbol;Acc:MGI:1202907]
ENSMUSG00000020189	10.18441239	14.2603717	8.791687292	12.61896641	15.63792454	20.88882666	5.037055706	5.47250007	6.067684046	13.39526423	12.68355432	13.68932995	Osbpl8	oxysterol binding protein-like 8 [Source:MGI Symbol;Acc:MGI:2443807]
ENSMUSG00000020190	70.89639378	77.76829806	76.42120021	73.464659	70.08592595	68.88633037	57.48693088	57.01650865	59.3681849	60.18521839	59.0829304	57.50471341	Mknk2	MAP kinase-interacting serine/threonine kinase 2 [Source:MGI Symbol;Acc:MGI:894279]
ENSMUSG00000020191	0.61615973	0.295641501	0.578251494	0.239579223	0	0.07821962	0	0	0	0.183713443	0.299186311	0.091009319	Spata48	spermatogenesis associated 48 [Source:MGI Symbol;Acc:MGI:1921112]
ENSMUSG00000020193	0.111420284	0.24494817	0.145181616	0.141784879	0.085138076	0.115727474	0.036172179	0.050171666	0.065994365	0.099662813	0.092746146	0.089766725	Zpbp	zona pellucida binding protein [Source:MGI Symbol;Acc:MGI:1855701]
ENSMUSG00000020196	46.16598173	50.96030044	45.39829118	58.13888978	53.49651005	63.45414551	54.42104327	50.94380826	64.35676154	80.18326327	85.96050115	79.92021274	Cabin1	calcineurin binding protein 1 [Source:MGI Symbol;Acc:MGI:1298375]
ENSMUSG00000020198	103.7289433	112.2430563	99.17522296	109.2950103	125.1578749	127.8930369	70.10319792	67.12517145	86.85470441	84.76756293	89.55260664	86.93592407	Ap3d1	adaptor-related protein complex 3, delta 1 subunit [Source:MGI Symbol;Acc:MGI:107734]
ENSMUSG00000020205	34.20690339	22.37443526	35.24942165	30.67086586	26.98420386	19.90256271	6.21416757	7.07972557	4.330574343	7.424581748	4.77145227	5.77271481	Phlda1	pleckstrin homology like domain, family A, member 1 [Source:MGI Symbol;Acc:MGI:1096880]
ENSMUSG00000020211	59.54235083	52.83991315	63.88436674	65.2101891	58.05560413	50.60129553	111.4555971	106.2732316	102.6650613	73.63078723	73.83732826	73.69630701	Sf3a2	splicing factor 3a, subunit 2 [Source:MGI Symbol;Acc:MGI:104912]
ENSMUSG00000020212	7.589867404	10.40174272	7.534087056	11.90331134	13.28051203	15.21101427	19.86442152	18.41116426	21.59592074	18.09528131	18.78524254	19.30941854	Mdm1	transformed mouse 3T3 cell double minute 1 [Source:MGI Symbol;Acc:MGI:96951]
ENSMUSG00000020213	0	0	0	0	0.050455712	0	0	0	0	0	0	0	Glipr1l1	GLI pathogenesis-related 1 like 1 [Source:MGI Symbol;Acc:MGI:1916536]
ENSMUSG00000020214	0.028723957	0.09923134	0.08822213	0.134023624	0.181074706	0.175028148	0	0	0	0	0	0	Glipr1l2	GLI pathogenesis-related 1 like 2 [Source:MGI Symbol;Acc:MGI:1914787]
ENSMUSG00000020216	1.607254081	0.581691219	0.470141411	0.728504934	0.6272232	0.594619371	3.81278701	5.049593484	2.69273911	0.944741971	0.993851401	1.058116886	Jsrp1	junctional sarcoplasmic reticulum protein 1 [Source:MGI Symbol;Acc:MGI:1916700]
ENSMUSG00000020218	0.324335335	0.67227986	0.420599374	0.564973557	0.772403429	0.312917785	0.038607894	0.192780216	0.181126816	0.734946092	0.557951327	0.632354701	Wif1	Wnt inhibitory factor 1 [Source:MGI Symbol;Acc:MGI:1344332]
ENSMUSG00000020219	167.7982197	120.0908171	226.3942738	158.9909414	135.1512152	93.66108592	163.3781443	171.2691246	106.6578451	93.80001385	82.83992906	84.54316144	Timm13	translocase of inner mitochondrial membrane 13 [Source:MGI Symbol;Acc:MGI:1353432]
ENSMUSG00000020220	5.654109345	6.288469797	4.689527632	5.196175275	6.444366072	8.564202665	6.696401708	5.757183688	7.500888991	10.42862562	11.30149913	11.02378382	Vps13d	vacuolar protein sorting 13D [Source:MGI Symbol;Acc:MGI:2448530]
ENSMUSG00000020224	38.32121104	30.88255635	44.49345147	35.94633378	29.53517254	23.81040174	51.76238811	53.69280508	43.19631135	44.41831558	41.83234455	40.48316076	Llph	LLP homolog, long-term synaptic facilitation (Aplysia) [Source:MGI Symbol;Acc:MGI:1913475]
ENSMUSG00000020225	153.6217751	132.4910961	167.5900823	143.4916089	115.0619741	111.9139634	69.24951955	66.43359541	52.50591103	69.6387619	65.82399022	65.93549072	Tmbim4	transmembrane BAX inhibitor motif containing 4 [Source:MGI Symbol;Acc:MGI:1915462]
ENSMUSG00000020226	0.053752015	0.092847312	0.01375773	0.025080248	0.014118775	0.061412906	0.647845759	0.985107036	0.863020442	0.985638114	1.017906205	1.178999864	Slc5a4b	solute carrier family 5 (neutral amino acid transporters, system A), member 4b [Source:MGI Symbol;Acc:MGI:1890478]
ENSMUSG00000020227	6.404923762	7.448145833	6.729309083	2.665140549	3.177158537	2.783152558	5.080734726	4.867959181	5.883257496	7.945539178	6.76482714	7.872239475	Irak3	interleukin-1 receptor-associated kinase 3 [Source:MGI Symbol;Acc:MGI:1921164]
ENSMUSG00000020228	6.795484054	8.35475973	5.133300607	7.76255882	8.739771728	11.53382897	30.7062306	26.0736404	35.56953023	33.57898368	32.54013963	35.0135185	Helb	helicase (DNA) B [Source:MGI Symbol;Acc:MGI:2152895]
ENSMUSG00000020229	0.439707175	0.33756361	0.45016898	0.312630484	0.439983634	0.255174857	0	0	0.019488638	0.037457875	0.087145682	0	Slc5a4a	solute carrier family 5, member 4a [Source:MGI Symbol;Acc:MGI:1927848]
ENSMUSG00000020230	37.77604483	41.04066777	34.39113602	39.68271292	38.09970002	37.02334193	10.46473611	10.4359834	12.31856661	16.16494588	17.88373645	17.96191304	Prmt2	protein arginine N-methyltransferase 2 [Source:MGI Symbol;Acc:MGI:1316652]
ENSMUSG00000020231	14.92402071	20.86443191	12.3331321	20.76511676	24.51933163	31.42739752	6.346234039	5.205933794	8.10288869	10.33907399	10.68721164	9.984359289	Dip2a	disco interacting protein 2 homolog A [Source:MGI Symbol;Acc:MGI:2385920]
ENSMUSG00000020232	145.825423	126.8872461	168.7237995	153.3810658	126.642058	110.7682777	46.72558852	51.3330775	45.7385853	37.34440563	39.30877142	42.84198206	Hmg20b	high mobility group 20B [Source:MGI Symbol;Acc:MGI:1341190]
ENSMUSG00000020234	0.201302867	0.521573849	0.154569299	0.610520716	0.211500905	0.689979382	0.224648269	0.199419171	0.140523339	0.135045496	0.125673247	0	4930404N11	RIKEN cDNA 4930404N11 gene [Source:MGI Symbol;Acc:MGI:1921072]
ENSMUSG00000020235	51.09003121	47.25770547	47.89521582	54.54102376	48.62276394	47.54919387	85.10644485	82.63125394	78.71487217	57.14606717	61.16968722	62.46830639	Fzr1	fizzy and cell division cycle 20 related 1 [Source:MGI Symbol;Acc:MGI:1926790]
ENSMUSG00000020238	135.7315229	132.8609254	145.8040055	134.8844984	121.6594551	116.1120202	95.3081684	98.87089828	101.3070329	100.0755066	98.06173675	100.7004862	Ncln	nicalin [Source:MGI Symbol;Acc:MGI:1926081]
ENSMUSG00000020241	43.92630755	45.35897945	48.85350544	23.39686555	24.21479959	23.28010345	1.372872698	1.447670299	1.66241761	13.47292408	13.86562283	13.25552247	Col6a2	collagen, type VI, alpha 2 [Source:MGI Symbol;Acc:MGI:88460]
ENSMUSG00000020246	15.31483532	19.56597735	13.328509	22.73363927	24.26871305	31.06011774	5.473194257	4.981355734	6.176203196	8.831283639	8.466849433	9.13643234	Hcfc2	host cell factor C2 [Source:MGI Symbol;Acc:MGI:1915183]
ENSMUSG00000020248	27.47482876	26.27683446	26.74238673	31.61739698	33.09956693	30.0072192	94.04711012	90.71487005	88.95030277	57.71718058	59.75893115	54.96054874	Nfyb	nuclear transcription factor-Y beta [Source:MGI Symbol;Acc:MGI:97317]
ENSMUSG00000020250	118.976703	144.5721019	109.3263279	148.2657895	158.1197232	180.3219983	161.5012519	151.88154	184.6877282	112.1112298	120.9390758	116.7306427	Txnrd1	thioredoxin reductase 1 [Source:MGI Symbol;Acc:MGI:1354175]
ENSMUSG00000020251	4.800553195	6.625570998	3.794492429	4.842133915	5.748391587	6.829041216	0.535728072	0.349678816	0.32854103	1.231362337	1.410344564	0.90718149	Glt8d2	glycosyltransferase 8 domain containing 2 [Source:MGI Symbol;Acc:MGI:1922032]
ENSMUSG00000020253	50.03531262	43.37613335	55.07734888	40.76784799	38.04415861	31.48455097	60.70806447	63.63596498	54.18672086	31.88402023	27.98805024	31.13145334	Ppm1m	protein phosphatase 1M [Source:MGI Symbol;Acc:MGI:1915155]
ENSMUSG00000020255	0.436995253	0.447308887	0.409841602	0.60533864	0.623188405	0.7742586	0.553866981	0.55436891	0.618071563	0.938304119	0.93453785	0.942959658	D10Wsu102	DNA segment, Chr 10, Wayne State University 102, expressed [Source:MGI Symbol;Acc:MGI:106381]
ENSMUSG00000020256	4.961356039	9.743699299	3.250815948	11.21266928	18.93631448	26.23295824	0.515419426	0.393194402	0.97394163	2.727239546	2.522949719	2.909932349	Aldh1l2	aldehyde dehydrogenase 1 family, member L2 [Source:MGI Symbol;Acc:MGI:2444680]
ENSMUSG00000020257	51.03577596	52.60124076	42.91447685	56.29464409	58.89057099	66.5247505	115.3463234	100.2977728	122.0836056	126.1181838	124.4102728	123.0354382	Wdr82	WD repeat domain containing 82 [Source:MGI Symbol;Acc:MGI:1924555]
ENSMUSG00000020258	0.258300077	0.297445817	0.176297149	0.562429905	0.339232004	0.344299366	0.980068813	0.469118884	0.540934013	0.40432581	0.573356686	0.57227971	Glyctk	glycerate kinase [Source:MGI Symbol;Acc:MGI:2444085]
ENSMUSG00000020260	78.12218705	71.68708033	72.80399371	60.01951634	53.43212039	52.36309044	25.62910054	25.77574998	27.21981313	29.67862674	30.03534086	27.46102731	Pofut2	protein O-fucosyltransferase 2 [Source:MGI Symbol;Acc:MGI:1916863]
ENSMUSG00000020261	12.66180351	16.37526749	10.85794899	11.35123468	12.93940299	15.57919995	6.608591596	7.032836039	7.494106824	15.68171668	16.05273443	16.74548581	Slc36a1	solute carrier family 36 (proton/amino acid symporter), member 1 [Source:MGI Symbol;Acc:MGI:2445299]
ENSMUSG00000020262	6.014854628	7.888416285	6.488669522	6.382147396	8.648446094	9.188264134	5.449479687	4.88823467	5.878298968	5.679042411	5.683599448	5.212056349	Adarb1	adenosine deaminase, RNA-specific, B1 [Source:MGI Symbol;Acc:MGI:891999]
ENSMUSG00000020263	67.48381752	83.09286749	55.18905502	97.56392789	105.9940348	114.8011341	33.81082662	27.77864232	35.28675242	36.68609529	39.64002511	35.16939243	Appl2	adaptor protein, phosphotyrosine interaction, PH domain and leucine zipper containing 2 [Source:MGI Symbol;Acc:MGI:2384914]

ENSMUSG00000020264	0	0	0.062901833	0.019111603	0	0.046797745	0.20112489	0.223171977	0.095309737	0.201507677	0.153427834	0.072599533	Slc36a2	solute carrier family 36 (proton/amino acid symporter), member 2 [Source:MGI Symbol;Acc:MGI:1891430]
ENSMUSG00000020265	69.67100831	61.21929708	73.29491201	53.61423536	51.43358536	46.18864827	112.9097563	114.3570938	104.7819681	92.54656301	93.43680621	88.61464176	Sumo3	small ubiquitin-like modifier 3 [Source:MGI Symbol;Acc:MGI:1336201]
ENSMUSG00000020267	333.6123217	263.3947936	402.0492374	363.8908929	315.0454685	244.134111	310.6043135	327.1954461	248.1993469	291.2551534	292.3081186	278.470554	Hint1	histidine triad nucleotide binding protein 1 [Source:MGI Symbol;Acc:MGI:1321133]
ENSMUSG00000020268	2.49259886	1.973369558	2.392410402	1.889917494	2.455194697	2.159620476	3.032019619	3.302654384	3.654013825	3.260747332	3.267868436	2.01571473	Lyrm7	LYR motif containing 7 [Source:MGI Symbol;Acc:MGI:1922780]
ENSMUSG00000020270	0	0	0	0	0	0	0	0	0	0	0	0	Smim23	small integral membrane protein 23 [Source:MGI Symbol;Acc:MGI:1916601]
ENSMUSG00000020271	36.76302522	46.99501482	35.21059431	44.53069679	49.31816547	54.68785813	28.72421202	23.14981103	31.15984752	58.19392633	61.55540937	58.22733971	Fbxw11	F-box and WD-40 domain protein 11 [Source:MGI Symbol;Acc:MGI:2144023]
ENSMUSG00000020272	9.361910005	12.52235562	8.53173633	12.50473862	13.23674137	16.12781332	12.00379799	10.29313089	13.11979234	21.8075929	21.17539	21.16409719	Stk10	serine/threonine kinase 10 [Source:MGI Symbol;Acc:MGI:1099439]
ENSMUSG00000020273	4.56766682	6.18089786	3.821136944	6.31907272	7.357322358	9.151275486	8.26688929	7.852593174	12.00978274	19.80834618	19.95744115	21.48421396	Papolg	poly(A) polymerase gamma [Source:MGI Symbol;Acc:MGI:2442119]
ENSMUSG00000020275	0.881778548	1.161420471	0.731647338	1.043366555	1.422978143	1.65055341	0.684325689	0.582351522	0.836815917	1.484668405	1.568727335	1.501617195	Rel	reticuloendotheliosis oncogene [Source:MGI Symbol;Acc:MGI:97897]
ENSMUSG00000020277	216.7387145	200.7715328	237.7659374	226.4881248	196.7474959	180.5678318	463.9551625	477.0370013	422.9097954	344.6148415	371.8104666	350.8352979	Pfkl	phosphofructokinase, liver, B-type [Source:MGI Symbol;Acc:MGI:97547]
ENSMUSG00000020279	0	0.044299969	0	0	0.040418702	0.073254405	0	0.076219665	0.12532144	0.154846534	0.11207784	0.068185793	Il9r	interleukin 9 receptor [Source:MGI Symbol;Acc:MGI:96564]
ENSMUSG00000020280	11.18845249	13.66675583	11.13349701	12.95352171	13.95250651	18.27858502	20.81777025	21.05395758	23.79591142	46.11073492	46.10933119	42.46513627	Pus10	pseudouridylate synthase 10 [Source:MGI Symbol;Acc:MGI:1921717]
ENSMUSG00000020282	55.89099992	58.77963307	60.54758617	51.32266424	48.45170934	45.39686559	12.71630789	12.95675547	13.66056647	19.78896698	21.25570395	21.62224489	Rhbdf1	rhomboid 5 homolog 1 [Source:MGI Symbol;Acc:MGI:104328]
ENSMUSG00000020283	26.13909605	26.28341878	23.62596837	22.3009411	23.54945417	25.12755067	30.97426605	29.74114539	29.47123916	47.07222299	42.72798205	41.07050923	Pex13	peroxisomal biogenesis factor 13 [Source:MGI Symbol;Acc:MGI:1919379]
ENSMUSG00000020284	15.44249116	11.18709666	14.91891822	13.50410943	11.41903431	9.588636329	5.854380395	5.453542133	4.860151031	5.141384031	4.53186242	4.35855872	Cfap410	cilia and flagella associated protein 410 [Source:MGI Symbol;Acc:MGI:1915134]
ENSMUSG00000020286	0.179285366	0.051614079	0	0.083653079	0.470919983	0.170698024	0.040015473	0	0.083435732	0.160366527	0.410401698	0.317774206	1700093K21	RIKEN cDNA 1700093K21 gene [Source:MGI Symbol;Acc:MGI:1914608]
ENSMUSG00000020287	1.912377237	2.461283129	1.900293146	2.047040047	2.304737798	2.763299779	3.841485407	3.482503897	4.161970648	5.257506517	4.354208392	4.06252514	Mpg	N-methylpurine-DNA glycosylase [Source:MGI Symbol;Acc:MGI:97073]
ENSMUSG00000020288	8.56220699	11.14316334	7.9867314	10.83103919	9.452908006	10.78664861	9.245773469	8.535940443	10.11981087	12.9589931	12.17275995	11.70725943	Ahsa2	AHA1, activator of heat shock protein ATPase 2 [Source:MGI Symbol;Acc:MGI:1916133]
ENSMUSG00000020289	21.55701577	19.99712062	21.31089638	21.739822	20.07261318	19.78603897	18.35461218	18.72825515	18.23311272	24.35785811	25.49193126	25.09342237	Nprl3	nitrogen permease regulator-like 3 [Source:MGI Symbol;Acc:MGI:109258]
ENSMUSG00000020290	41.04943275	48.35856287	35.01957812	54.92805128	62.23186612	72.55020592	108.6445027	104.7052722	133.849731	224.3494396	220.5448021	225.0859763	Xpo1	exportin 1 [Source:MGI Symbol;Acc:MGI:2144013]
ENSMUSG00000020295	0	0	0	0	0	0	0.063600752	0.141145191	0.066306542	0.127443597	0.474395702	0.063133948	Hbq1a	hemoglobin, theta 1A [Source:MGI Symbol;Acc:MGI:2685722]
ENSMUSG00000020297	0.186155386	0.091871784	0.05445272	0.148900412	0.111763457	0.182302958	2.065570027	1.659720893	2.351464397	0.832557987	1.39460022	1.225532166	Nsg2	neuron specific gene family member 2 [Source:MGI Symbol;Acc:MGI:1202070]
ENSMUSG00000020299	0.236656683	0.148648547	0.198235126	0.160613911	0.203437433	0.163870103	0.672259946	0.596761868	0.360442364	0.327147714	0.232809691	0.32412969	4930524B15	RIKEN cDNA 4930524B15 gene [Source:MGI Symbol;Acc:MGI:1914842]
ENSMUSG00000020300	6.883443325	8.985663455	5.135625023	8.148590062	9.32079966	12.06383963	2.288967989	1.960126416	2.775426951	9.597064154	9.596015214	10.43880033	Cpeb4	cytoplasmic polyadenylation element binding protein 4 [Source:MGI Symbol;Acc:MGI:1914829]
ENSMUSG00000020303	5.149686043	5.007663813	5.717419707	3.951273085	3.871563433	3.259969074	6.620432297	6.257837216	6.433427532	9.683408561	11.35471159	10.89221799	Stc2	stanniocalcin 2 [Source:MGI Symbol;Acc:MGI:1316731]
ENSMUSG00000020305	6.745223876	7.025504555	7.915799755	8.060759004	7.594659002	8.603755535	5.15913108	5.425470554	5.209950455	8.951108698	8.648342418	8.333208465	Asb3	ankyrin repeat and SOCS box-containing 3 [Source:MGI Symbol;Acc:MGI:1929749]
ENSMUSG00000020307	277.3576065	188.4909936	326.1412208	247.0847781	202.24774	161.4743414	497.4835926	597.8836006	438.9012278	291.3043888	300.359061	293.0212676	Cdc34	cell division cycle 34 [Source:MGI Symbol;Acc:MGI:102657]
ENSMUSG00000020308	83.97399215	62.84495965	108.698775	67.20014963	56.02579978	43.78935537	37.88352829	50.76190162	34.45445677	32.15315586	33.29037389	35.81222999	Tpgs1	tubulin polyglutamylase complex subunit 1 [Source:MGI Symbol;Acc:MGI:106618]
ENSMUSG00000020309	7.779786574	8.279482045	6.87018812	8.636266441	7.747783779	6.768242479	4.67430101	3.981334532	5.490886241	8.014203795	8.255989292	8.397734794	Chac2	ChaC, cation transport regulator 2 [Source:MGI Symbol;Acc:MGI:1915294]
ENSMUSG00000020310	0.108475179	0.333105987	0.037018698	0.033742418	0.265931284	0.082623581	1.226692819	1.683541884	1.716392208	0.970284867	0.872848226	1.153600478	Madcam1	mucosal vascular addressin cell adhesion molecule 1 [Source:MGI Symbol;Acc:MGI:103579]
ENSMUSG00000020311	58.77472506	50.51917346	42.54361149	44.82888279	42.15609333	46.6276184	15.37345921	12.84614588	14.38164247	28.54586938	28.21192247	28.0213419	Erlec1	endoplasmic reticulum lectin 1 [Source:MGI Symbol;Acc:MGI:1914003]
ENSMUSG00000020312	16.58321827	17.22239216	14.67852966	13.88058954	13.28550051	14.93672272	2.716935594	2.611719867	4.044297828	5.735347485	5.852076478	6.56220561	Shc2	SHC (Src homology 2 domain containing) transforming protein 2 [Source:MGI Symbol;Acc:MGI:106180]
ENSMUSG00000020315	63.77653764	89.29556575	51.75537592	73.36124385	94.40335103	125.7171167	34.96207748	30.41720091	44.87889657	105.3324333	108.5709583	107.745036	Sptbn1	spectrin beta, non-erythrocytic 1 [Source:MGI Symbol;Acc:MGI:98388]
ENSMUSG00000020317	0	0.046569094	0	0	0	0.030802651	0.036104186	0	0	0	0.168312385	0	Theg	testicular haploid expressed gene [Source:MGI Symbol;Acc:MGI:1338756]
ENSMUSG00000020319	7.196719559	6.434093772	5.658087181	6.064110273	6.699891922	6.42990666	1.111509539	0.992697935	1.158796904	3.042097554	3.437611196	3.417698585	Wdpcp	WD repeat containing planar cell polarity effector [Source:MGI Symbol;Acc:MGI:2144467]
ENSMUSG00000020321	135.0894385	128.3157749	147.4907672	124.677603	112.7325208	94.97827586	143.8899973	150.1018223	128.0491251	185.9144902	180.9921029	179.3934323	Mdh1	malate dehydrogenase 1, NAD (soluble) [Source:MGI Symbol;Acc:MGI:97051]
ENSMUSG00000020323	0.230098865	0.058882372	0	0.023858276	0.080585238	0.097367857	0	0.075981903	0.071388862	0	0.063844698	0	Prss57	protease, serine 57 [Source:MGI Symbol;Acc:MGI:1920356]
ENSMUSG00000020325	4.146948465	5.852587715	3.70294277	1.338449261	1.637982549	1.614951742	1.837232151	1.173755227	1.886373079	4.713381389	4.697720624	4.283043643	Fstl3	follistatin-like 3 [Source:MGI Symbol;Acc:MGI:1890391]
ENSMUSG00000020326	250.1753646	300.9937907	217.5054337	267.6402341	297.6877682	349.8376114	88.88107803	89.67625049	108.4741847	305.3533236	297.3232193	312.3611474	Ccng1	cyclin G1 [Source:MGI Symbol;Acc:MGI:102890]
ENSMUSG00000020327	0.132395347	0.38115012	0.440522502	0.432422069	0.730288219	0.655480413	0.768297081	1.245986317	0.770175991	0.562516432	0.275514427	0.205331025	Fgf22	fibroblast growth factor 22 [Source:MGI Symbol;Acc:MGI:1914362]
ENSMUSG00000020328	81.88956763	78.52338493	80.47802125	79.18888062	80.40075019	68.0874327	170.6810102	174.4769868	165.0451717	184.1924105	183.7240937	195.1426006	Nudcd2	NudC domain containing 2 [Source:MGI Symbol;Acc:MGI:1277103]
ENSMUSG00000020329	23.37821285	23.81790391	21.84219086	23.19932148	22.53337371	21.77522352	27.66831869	27.85363326	29.7651314	30.95509195	33.27455029	33.6946804	Polrmt	polymerase (RNA) mitochondrial (DNA directed) [Source:MGI Symbol;Acc:MGI:1915843]
ENSMUSG00000020330	14.90303168	19.01153155	12.19597047	22.16398173	26.79780846	31.0986112	74.49083707	64.5141348	86.19423938	126.4509866	125.9865281	127.8032733	Hmmr	hyaluronan mediated motility receptor (RHAMM) [Source:MGI Symbol;Acc:MGI:104667]
ENSMUSG00000020331	0.6299524	0.646572292	0.532777055	0.293930614	0.100718723	0.292066384	7.64140159	8.302679437	8.935218083	3.307368211	3.408416858	2.949171177	Hcn2	hyperpolarization-activated, cyclic nucleotide-gated K+ 2 [Source:MGI Symbol;Acc:MGI:1298210]
ENSMUSG00000020332	0	0.169573975	0.060304244	0.164901346	0	0.157028162	0.026293535	0	0	0	0.04903063	0	Meikin	meiotic kinetochore factor [Source:MGI Symbol;Acc:MGI:1922097]
ENSMUSG00000020333	0.809630504	0.738095489	0.828893771	0.440728099	0.513932274	0.411120604	3.418334721	2.924156545	3.657958567	3.681326458	3.847049738	3.572624155	Acsl6	acyl-CoA synthetase long-chain family member 6 [Source:MGI Symbol;Acc:MGI:894291]
ENSMUSG00000020334	10.56388025	8.350089459	9.119456414	10.85414799	10.63497651	8.07435239	0.131445181	0.145854055	0.30833393	1.284029674	1.623861763	1.17432384	Slc22a4	solute carrier family 22 (organic cation transporter), member 4 [Source:MGI Symbol;Acc:MGI:1353479]
ENSMUSG00000020335	0.498804923	0.634862616	0.215020135	0.342982727	0.524074465	0.559897851	0.562511591	0.442122886	0.635312959	0.892338452	0.786703793	0.674712774	Zfp354b	zinc finger protein 354B [Source:MGI Symbol;Acc:MGI:1351476]
ENSMUSG00000020340	1.263067157	1.45448676	0.660927433	0.82943692	0.865042839	1.104582126	8.970983534	7.451876832	10.28442008	8.243236981	9.493535814	9.203636487	Cyfip2	cytoplasmic FMR1 interacting protein 2 [Source:MGI Symbol;Acc:MGI:1924134]
ENSMUSG00000020346	107.9832326	107.6392059	105.674049	80.2735223	80.70494133	74.93543769	25.78049324	29.95521687	24.60972009	48.03785312	49.77382612	49.78550754	Mgat1	mannoside acetylglucosaminyltransferase 1 [Source:MGI Symbol;Acc:MGI:96973]
ENSMUSG00000020349	64.58762323	61.50395014	57.15072835	61.22515165	59.25190394	56.23044021	64.77622107	67.40754163	68.14832147	92.09405412	92.06450387	91.82132918	Ppp2ca	protein phosphatase 2 (formerly 2A), catalytic subunit, alpha isoform [Source:MGI Symbol;Acc:MGI:1321159]
ENSMUSG00000020354	2.944150676	1.979453663	3.201417248	2.901016248	2.901171301	2.716079058	0.146933143	0.307964051	0.391470669	1.063203491	0.715424202	0.842716126	Sgcd	sarcoglycan, delta (dystrophin-associated glycoprotein) [Source:MGI Symbol;Acc:MGI:1346525]
ENSMUSG00000020357	1.503942269	1.913108711	1.486013723	0.775163665	0.937055166	0.919084505	4.535466412	4.305028715	4.874911961	6.030120315	6.361298348	6.346443597	Flt4	FMS-like tyrosine kinase 4 [Source:MGI Symbol;Acc:MGI:95561]
ENSMUSG00000020358	114.6021632	142.212218	96.14290069	138.6329869	139.7729348	149.9495913	322.4273551	288.0101091	306.6237359	384.4432022	418.0050916	415.9828154	Hnrnpab	heterogeneous nuclear ribonucleoprotein A/B [Source:MGI Symbol;Acc:MGI:1330294]
ENSMUSG00000020359	5.760406078	6.357011893	5.942139225	5.273707282	6.510731446	7.21294369	9.525792921	9.121758484	11.3120575	12.80329916	13.3677588	12.68518533	Phykpl	5-phosphohydroxy-L-lysine phospholyase [Source:MGI Symbol;Acc:MGI:1920197]
ENSMUSG00000020361	132.0636429	136.4844444	115.101193	127.6110528	139.0310612	151.1456547	327.5787214	318.9870124	358.8975695	495.3209803	501.8106941	490.6690339	Hspa4	heat shock protein 4 [Source:MGI Symbol;Acc:MGI:1342292]
ENSMUSG00000020362	23.34851166	31.17394977	21.88736329	29.44475947	35.20210929	43.6482232	27.9762104	25.94071958	35.01987966	61.26391959	61.73559721	61.88389068	Cnot6	CCR4-NOT transcription complex, subunit 6 [Source:MGI Symbol;Acc:MGI:2144529]
ENSMUSG00000020363	12.83747383	13.31664546	10.99032395	9.368223666	9.28746251	11.36827694	33.92034258	31.93271522	36.78292685	29.09775021	31.03471867	28.75058299	Gfpt2	glutamine fructose-6-phosphate transaminase 2 [Source:MGI Symbol;Acc:MGI:1338883]
ENSMUSG00000020364	2.898596358	2.886817478	2.265773451	3.125282071	3.168903516	4.132181937	2.273068288	1.319325282	2.898413788	4.502233663	4.206078231	4.929333151	Zfp354a	zinc finger protein 354A [Source:MGI Symbol;Acc:MGI:103172]
ENSMUSG00000020366	33.20971515	33.24665675	26.50440526	32.33586872	35.43192579	39.91582215	23.48211478	21.50378585	25.51722059	34.607256	33.99930269	34.70536997	Mapk9	mitogen-activated protein kinase 9 [Source:MGI Symbol;Acc:MGI:1346862]
ENSMUSG00000020368	269.7572437	329.6849282	238.1345338	299.7498947	345.0901634	372.1558323	247.075538	224.9401505	280.5943679	520.6900659	504.1225224	521.7256632	Canx	calnexin [Source:MGI Symbol;Acc:MGI:88261]
ENSMUSG00000020372	920.7476875	733.9035586	988.0908187	820.2293872	731.7356868	600.1206305	1609.325951	1777.146353	1506.377543	1417.581322	1427.986946	1456.337556	Rack1	receptor for activated C kinase 1 [Source:MGI Symbol;Acc:MGI:101849]
ENSMUSG00000020374	0.020597649	0	0.210877215	0.057664162	0.129846774	0.172577418	0.110334669	0.306073583	0.172543018	0.460602757	0.445782079	0.200795573	Rasgef1c	RasGEF domain family, member 1C [Source:MGI Symbol;Acc:MGI:1921813]
ENSMUSG00000020375	39.64812939	40.01796416	37.09833405	35.84921369	34.65307221	28.82766279	12.57931345	14.45898799	13.55554982	24.27398173	24.64053768	24.07804555	Rufy1	RUN and FYVE domain containing 1 [Source:MGI Symbol;Acc:MGI:2429762]
ENSMUSG00000020376	26.53852842	25.49906387	29.38885589	22.64378937	21.5770593	20.97583498	3.896963005	4.699503463	3.61133596	12.58077943	12.75479433	12.49158202	Rnf130	ring finger protein 130 [Source:MGI Symbol;Acc:MGI:1891717]
ENSMUSG00000020377	0.141541078	0	0.362271794	0.396251426	0.594846294	0.592951031	0.252729303	0.070108302	0.197610945	0.443118034	0.471274677	0.313592966	Ltc4s	leukotriene C4 synthase [Source:MGI Symbol;Acc:MGI:107498]
ENSMUSG00000020380	19.21280682	23.77690052	16.57921413	25.38937966	29.68203145	33.95212532	29.21458802	27.11882645	35.6088818	59.723856	59.44143215	61.48497365	Rad50	RAD50 double strand break repair protein [Source:MGI Symbol;Acc:MGI:109292]

ENSMUSG00000020381	2.738655284	3.517591072	3.666527556	3.243732604	4.703435814	3.770817552	10.39128856	9.860814217	9.019637552	9.987085521	8.899419489	8.494722954	Mrnip	MRN complex interacting protein [Source:MGI Symbol;Acc:MGI:1915317]
ENSMUSG00000020383	0.071416577	0.08223986	0.073115768	0	0.037517277	0	0	0	0	0.095820663	0.029723548	0.063291128	Il13	interleukin 13 [Source:MGI Symbol;Acc:MGI:96541]
ENSMUSG00000020385	40.07078575	40.31147034	37.2494545	40.49376155	38.56754842	45.67377641	33.82098734	35.56167708	39.29493021	49.07034507	46.91268719	45.49377612	Clk4	CDC like kinase 4 [Source:MGI Symbol;Acc:MGI:1098551]
ENSMUSG00000020386	88.72921381	83.90133444	79.67371487	79.64536478	75.23260075	71.69576631	63.0023082	58.72534404	55.83531898	86.68329055	81.62071244	85.06199272	Sar1b	secretion associated Ras related GTPase 1B [Source:MGI Symbol;Acc:MGI:1913647]
ENSMUSG00000020387	4.756992954	5.572073704	4.870174491	6.207870558	6.403665753	7.071126581	2.826866097	3.166197013	3.279201558	7.858486138	8.185476984	8.036300522	Jade2	jade family PHD finger 2 [Source:MGI Symbol;Acc:MGI:1924151]
ENSMUSG00000020388	26.26540763	20.81865266	33.82494409	9.685946522	7.935775022	6.235600755	2.218751481	2.510242447	2.313144652	11.07128364	10.91136977	12.30790242	Pdlim4	PDZ and LIM domain 4 [Source:MGI Symbol;Acc:MGI:1353470]
ENSMUSG00000020389	3.077066539	4.618038092	3.563429383	3.836469453	3.524446858	5.398299999	2.251749946	3.173202027	3.497845004	4.602312509	4.387882166	3.866935821	Cdkl3	cyclin-dependent kinase-like 3 [Source:MGI Symbol;Acc:MGI:2388268]
ENSMUSG00000020390	207.058299	202.7025639	197.7278576	202.9575789	189.929735	185.4016416	114.4529834	123.1241152	118.1571331	138.5100269	138.7958193	150.1743119	Ube2b	ubiquitin-conjugating enzyme E2B [Source:MGI Symbol;Acc:MGI:102944]
ENSMUSG00000020392	48.81207405	44.21341394	45.58605324	70.06538035	65.92727234	61.93891835	82.70858055	80.69573435	78.28230913	118.9543058	125.2575589	120.5432064	Cdkn2aipnl	CDKN2A interacting protein N-terminal like [Source:MGI Symbol;Acc:MGI:1261797]
ENSMUSG00000020393	15.78781581	18.00792098	14.94708191	12.55545314	14.29347578	15.05811346	6.842048636	6.552928826	8.318667045	14.33245912	13.43578314	15.27213347	Kremen1	kringle containing transmembrane protein 1 [Source:MGI Symbol;Acc:MGI:1933988]
ENSMUSG00000020395	0	0.03628672	0.032260894	0.058811392	0.03310752	0	0	0	0	0	0.026229861	0	Itk	IL2 inducible T cell kinase [Source:MGI Symbol;Acc:MGI:96621]
ENSMUSG00000020396	0.409384711	0.359773649	0.25368096	0.422244885	0.271657396	0.434908136	4.732125239	5.133458374	5.003637108	21.11345376	20.33867641	19.20011801	Nefh	neurofilament, heavy polypeptide [Source:MGI Symbol;Acc:MGI:97309]
ENSMUSG00000020397	40.17622064	42.37046649	39.96406789	40.26755022	40.07100945	35.75347706	46.99999191	48.23663637	47.71006876	54.16014965	47.62016399	49.14666977	Med7	mediator complex subunit 7 [Source:MGI Symbol;Acc:MGI:1913463]
ENSMUSG00000020399	7.34073212	9.038163741	6.676616747	6.712633956	9.899003446	9.198192387	0.424231062	0.76291502	0.442279282	3.81068974	3.559864623	2.918779072	Havcr2	hepatitis A virus cellular receptor 2 [Source:MGI Symbol;Acc:MGI:2159682]
ENSMUSG00000020400	47.90009019	48.05722681	49.16260605	36.90626407	36.08092781	32.02458681	48.1631857	48.06997437	53.49565973	62.83004884	62.44481469	63.78169613	Tnip1	TNFAIP3 interacting protein 1 [Source:MGI Symbol;Acc:MGI:1926194]
ENSMUSG00000020401	0	0	0	0	0.069178758	0.025075762	0.05878325	0	0	0	0	0	Fam71b	family with sequence similarity 71, member B [Source:MGI Symbol;Acc:MGI:3650836]
ENSMUSG00000020402	302.7602521	306.9155291	265.0045169	302.4108007	305.5107239	286.9654689	730.5601365	699.0639023	726.7350446	562.4148419	581.7081953	567.8438131	Vdac1	voltage-dependent anion channel 1 [Source:MGI Symbol;Acc:MGI:106919]
ENSMUSG00000020405	0	0	0.275901358	0	0	0.068421755	0.080198025	0.355956975	0	0.482103963	0.972065514	0.796094044	Fabp6	fatty acid binding protein 6 [Source:MGI Symbol;Acc:MGI:96565]
ENSMUSG00000020407	29.88242865	20.79934738	28.10522253	27.88579141	27.1017775	28.69304502	434.6944013	457.557559	383.1146048	513.4363884	506.0340584	507.2802199	Upp1	uridine phosphorylase 1 [Source:MGI Symbol;Acc:MGI:1097668]
ENSMUSG00000020409	32.86486225	39.44027537	33.24172673	37.06119851	39.4143787	39.76204458	25.30081768	24.76708737	26.33116629	54.83429232	48.80563894	51.50133679	Slu7	SLU7 splicing factor homolog (S. cerevisiae) [Source:MGI Symbol;Acc:MGI:2385598]
ENSMUSG00000020411	0.262368828	0.215807995	0.217447228	0.08161276	0.328167235	0.076130129	0.245391054	0.086637902	0.290716837	0.994607726	1.435170969	1.140444014	Nipal4	NIPA-like domain containing 4 [Source:MGI Symbol;Acc:MGI:2444671]
ENSMUSG00000020412	18.20126854	18.68520565	17.62325141	22.0645879	21.88627955	21.59569174	15.33723557	15.23245532	15.44462876	22.91610701	22.96027086	24.72990616	Ascc2	activating signal cointegrator 1 complex subunit 2 [Source:MGI Symbol;Acc:MGI:1922702]
ENSMUSG00000020413	2.981829132	3.972745209	2.99952872	4.173890927	5.482555932	8.002037271	17.10250352	15.50811461	21.08954111	33.10709436	33.01732921	33.2472223	Hus1	HUS1 checkpoint clamp component [Source:MGI Symbol;Acc:MGI:1277962]
ENSMUSG00000020415	43.52817349	28.51494706	47.00801408	37.87379651	35.23332165	29.56005216	111.6261308	125.0724873	86.97125421	47.17879749	39.60653198	41.18353706	Pttg1	pituitary tumor-transforming gene 1 [Source:MGI Symbol;Acc:MGI:1353578]
ENSMUSG00000020419	3.731252635	3.153982083	2.641509468	3.407859742	4.00368871	2.781558947	11.80087307	10.18458907	10.75120212	6.462001734	7.533438754	7.879862224	Hormad2	HORMA domain containing 2 [Source:MGI Symbol;Acc:MGI:1923078]
ENSMUSG00000020420	1.021607334	0.820407475	0.908293819	0.790274634	1.016869391	0.962436095	2.040151575	1.984145988	1.914251853	3.282478856	3.479865128	3.538104559	Zfp607a	zinc finger protein 607A [Source:MGI Symbol;Acc:MGI:3584526]
ENSMUSG00000020422	18.78330504	27.1093843	15.91302239	17.13952238	21.79686778	26.09652547	35.58002297	28.83297443	40.67815068	88.56041316	91.59194964	89.87649976	Tns3	tensin 3 [Source:MGI Symbol;Acc:MGI:2443012]
ENSMUSG00000020423	22.79270609	22.77868728	20.29968802	16.36587248	16.06930747	16.58415627	23.93400861	21.70919039	23.69659968	22.52030114	22.08032681	23.14665924	Btg2	BTG anti-proliferation factor 2 [Source:MGI Symbol;Acc:MGI:108384]
ENSMUSG00000020424	5.740776648	5.036799555	5.261641066	3.112277189	1.809481611	1.561659179	37.41421302	38.18452685	38.87871889	29.88074645	30.31008776	32.22157766	Castor1	cytosolic arginine sensor for mTORC1 subunit 1 [Source:MGI Symbol;Acc:MGI:1919212]
ENSMUSG00000020427	28.10916711	30.88146567	27.51932337	4.705637596	5.976692934	5.364464395	6.715623399	5.986196529	6.419500801	18.89917574	16.70297873	18.2077695	Igfbp3	insulin-like growth factor binding protein 3 [Source:MGI Symbol;Acc:MGI:96438]
ENSMUSG00000020428	0	0	0	0	0	0	0	0	0	0	0	0	Gabra6	gamma-aminobutyric acid (GABA) A receptor, subunit alpha 6 [Source:MGI Symbol;Acc:MGI:95618]
ENSMUSG00000020429	0.028215402	0	0	0.052660299	0	0	0	0.027951376	0	0	0	0	Igfbp1	insulin-like growth factor binding protein 1 [Source:MGI Symbol;Acc:MGI:96436]
ENSMUSG00000020430	52.38354453	49.41210709	60.73981919	57.30166304	54.93700804	48.0794701	110.962041	115.5838987	105.6648995	153.4181998	141.241256	144.473584	Pes1	pescadillo ribosomal biogenesis factor 1 [Source:MGI Symbol;Acc:MGI:1890613]
ENSMUSG00000020431	0.119293059	0.131399391	0.053100591	0.038720808	0.005449412	0.031604649	2.102259372	2.075800684	2.471693054	1.266539877	1.631964673	1.406541566	Adcy1	adenylate cyclase 1 [Source:MGI Symbol;Acc:MGI:99677]
ENSMUSG00000020432	80.33310388	84.16037399	83.37572778	64.65390499	56.21434963	52.40140023	13.63362309	15.93817007	14.85130324	41.63105145	43.81910498	40.22854968	Tcn2	transcobalamin 2 [Source:MGI Symbol;Acc:MGI:98534]
ENSMUSG00000020434	0.86389242	0.650456962	0.527266315	1.255765137	1.064751899	1.417254946	0.31147179	0.148120706	0.402037815	0.534967872	0.566985288	0.530032652	4921536K21	RIKEN cDNA 4921536K21 gene [Source:MGI Symbol;Acc:MGI:1914680]
ENSMUSG00000020435	0.446469394	0.192799672	0.247591549	0.381918077	0.508178243	0.53844046	0.398597708	0.313289845	0.294351741	0.765432967	0.727796432	0.659453605	Osbp2	oxysterol binding protein 2 [Source:MGI Symbol;Acc:MGI:1921559]
ENSMUSG00000020436	0.033213808	0	0	0	0	0	0	0	0	0.074272417	0.069117861	0.029434894	Gabrg2	gamma-aminobutyric acid (GABA) A receptor, subunit gamma 2 [Source:MGI Symbol;Acc:MGI:95623]
ENSMUSG00000020437	1.134155946	1.146115746	1.540288468	0.086398016	0.267504842	0.370228313	10.35279284	11.25835999	11.74114448	13.27099863	14.19958969	14.62547662	Myo1g	myosin IG [Source:MGI Symbol;Acc:MGI:1927091]
ENSMUSG00000020439	52.87381062	47.36457863	62.50627956	54.27900311	47.93277849	42.55323601	17.88050532	18.0235701	17.03217223	15.04821027	14.88128878	14.34888329	Smtn	smoothelin [Source:MGI Symbol;Acc:MGI:1354727]
ENSMUSG00000020440	204.0858684	142.1763403	232.9015112	153.7353712	132.0915426	92.35676908	132.0664844	154.1193658	112.1290484	70.58874346	73.09096859	74.02029572	Arf5	ADP-ribosylation factor 5 [Source:MGI Symbol;Acc:MGI:99434]
ENSMUSG00000020441	7.668111839	7.711986868	8.261939531	6.562046961	7.986216549	6.529299053	10.88171742	11.07940322	8.664330057	6.469572557	6.717406218	7.982685884	2310033P09	RIKEN cDNA 2310033P09 gene [Source:MGI Symbol;Acc:MGI:1915112]
ENSMUSG00000020444	108.8774415	76.44782411	121.5370117	103.8254213	89.93226186	66.30886675	164.2235012	178.5718548	145.1781743	139.9312492	130.411802	138.6403463	Guk1	guanylate kinase 1 [Source:MGI Symbol;Acc:MGI:95871]
ENSMUSG00000020447	0.183017969	0.177477558	0.108478789	0.089889138	0.1012051	0.161412235	0.103196384	0.162220598	0.098621148	0.301562306	0.272615573	0.196341349	Npc1l1	NPC1 like intracellular cholesterol transporter 1 [Source:MGI Symbol;Acc:MGI:2685089]
ENSMUSG00000020448	24.59061781	25.99472695	24.32051313	23.02360874	24.18816275	26.27197772	20.40789123	17.96259681	21.65536508	25.73153812	28.4906964	28.00385188	Rnf185	ring finger protein 185 [Source:MGI Symbol;Acc:MGI:1922078]
ENSMUSG00000020451	43.90815902	42.80591916	42.09665965	33.46904957	32.84996964	35.68386359	63.41143552	64.71061809	64.49504132	76.27901279	76.80125577	75.8871532	Limk2	LIM motif-containing protein kinase 2 [Source:MGI Symbol;Acc:MGI:1197517]
ENSMUSG00000020453	12.26250956	13.41907562	10.532606	12.28491673	12.44829616	13.66667376	24.18068151	23.59515762	25.84474991	41.34458031	41.49887272	40.94156056	Patz1	POZ (BTB) and AT hook containing zinc finger 1 [Source:MGI Symbol;Acc:MGI:1891832]
ENSMUSG00000020454	34.8158727	39.66116748	26.46967725	43.45047357	48.18287328	52.00358468	51.55402928	48.14240657	58.47306788	78.44094506	78.91787898	77.01229365	Eif4enif1	eukaryotic translation initiation factor 4E nuclear import factor 1 [Source:MGI Symbol;Acc:MGI:1921453]
ENSMUSG00000020455	26.01536592	27.26972599	22.9674125	27.00772757	27.14851076	27.17162395	15.43160805	15.76095075	16.60901267	23.4846677	24.74305891	24.74175087	Trim11	tripartite motif-containing 11 [Source:MGI Symbol;Acc:MGI:2137355]
ENSMUSG00000020456	65.42086415	69.13711704	63.83081151	60.23021675	65.79638472	65.22599874	62.80523761	60.82962998	73.28722285	77.04866133	78.631984	78.78782688	Ogdh	oxoglutarate (alpha-ketoglutarate) dehydrogenase (lipoamide) [Source:MGI Symbol;Acc:MGI:1098267]
ENSMUSG00000020457	114.6242382	101.9037208	116.2085893	116.2935632	109.5022239	101.4525993	197.1932309	209.9121934	200.8124909	248.1293775	252.4076036	248.0437527	Drg1	developmentally regulated GTP binding protein 1 [Source:MGI Symbol;Acc:MGI:1343297]
ENSMUSG00000020458	401.4666741	457.8269204	359.6386282	466.5888808	488.8919018	503.1672964	142.0191176	124.0213137	134.6204699	341.2999041	333.9366177	321.4774499	Rtn4	reticulon 4 [Source:MGI Symbol;Acc:MGI:1915835]
ENSMUSG00000020459	17.9030337	19.20660456	17.45170936	18.7630405	22.02538981	23.58983562	34.69905026	31.52978777	35.00740201	46.15271074	44.0450766	43.82843099	Mtif2	mitochondrial translational initiation factor 2 [Source:MGI Symbol;Acc:MGI:1924034]
ENSMUSG00000020460	864.69332	674.630419	1196.875164	887.7822412	738.402533	524.8395249	1559.002828	1657.55345	1090.505022	1143.199512	1059.88104	1133.500592	Rps27a	ribosomal protein S27A [Source:MGI Symbol;Acc:MGI:1925544]
ENSMUSG00000020461	0.180918659	0.451397129	0.401316925	0.422075801	0.380168059	0.711979425	0.323040118	0.388322369	0.308718057	0.431539917	0.100397689	0.213779424	Clhc1	clathrin heavy chain linker domain containing 1 [Source:MGI Symbol;Acc:MGI:1920574]
ENSMUSG00000020462	70.98709052	67.41702529	73.18357694	64.43985556	71.19789307	59.24244053	40.1408671	40.51419856	37.41919572	57.19985786	62.34942264	61.76827649	Cfap36	cilia and flagella associated protein 36 [Source:MGI Symbol;Acc:MGI:1913994]
ENSMUSG00000020463	24.99326136	33.30009491	22.22558548	32.41367422	36.69886127	46.38393089	29.61869732	27.73712987	38.08037046	78.86796731	79.23267409	76.8331165	Ppp4r3b	protein phosphatase 4 regulatory subunit 3B [Source:MGI Symbol;Acc:MGI:2144474]
ENSMUSG00000020464	35.63145565	43.00786609	28.83195027	42.11603546	47.08776529	52.86191756	53.52181916	49.16340749	62.93116675	85.06273017	86.06111035	84.96353932	Pnpt1	polyribonucleotide nucleotidyltransferase 1 [Source:MGI Symbol;Acc:MGI:1918951]
ENSMUSG00000020467	149.0689832	166.7792531	124.992663	191.3490597	210.2770022	221.1157342	2.192560084	2.299596162	2.12927467	9.208223385	7.365002542	5.992739517	Efemp1	epidermal growth factor-containing fibulin-like extracellular matrix protein 1 [Source:MGI Symbol;Acc:MGI:1339998]
ENSMUSG00000020469	0.145859281	0.25194669	0.522653816	0.680567421	0.689618415	0.277745938	14.84506225	17.55606948	10.6571471	6.653851813	6.677722547	7.36805277	Myl7	myosin, light polypeptide 7, regulatory [Source:MGI Symbol;Acc:MGI:107495]
ENSMUSG00000020471	40.20338509	29.59207183	38.87944809	53.13237976	43.95253173	44.9815157	332.2011812	347.7343459	295.9692978	172.2239303	180.5767472	182.3349874	Pold2	polymerase (DNA directed), delta 2, regulatory subunit [Source:MGI Symbol;Acc:MGI:1097163]
ENSMUSG00000020472	24.02619133	27.27771278	25.58388396	31.62724025	29.45514753	28.39225502	30.00216371	24.72969628	31.25432877	39.3261798	37.50696398	37.37163073	Zkscan17	zinc finger with KRAB and SCAN domains 17 [Source:MGI Symbol;Acc:MGI:2679270]
ENSMUSG00000020473	631.0234028	695.1071104	665.1364455	555.8041666	569.5516461	550.4165059	10.29086167	9.294903757	12.2637734	71.58302774	71.14561637	70.77649005	Aebp1	AE binding protein 1 [Source:MGI Symbol;Acc:MGI:1197012]
ENSMUSG00000020474	44.74835337	45.47845994	42.35667876	46.9893771	45.64333548	44.83078471	14.84274895	15.80721665	18.91292278	19.68555497	19.00866004	18.99588812	Polm	polymerase (DNA directed), mu [Source:MGI Symbol;Acc:MGI:1860191]
ENSMUSG00000020475	2.458770733	3.598238635	2.150169355	2.294484448	1.560763372	2.926251843	6.173815833	8.778894824	4.815433695	5.223366865	5.457809597	6.21929517	Pgam2	phosphoglycerate mutase 2 [Source:MGI Symbol;Acc:MGI:1933118]
ENSMUSG00000020476	92.80057082	86.54882532	97.10202774	94.07038235	85.66267804	73.5509998	60.14701874	59.04692755	52.40000209	60.82577496	61.74698958	60.38031218	Dbnl	drebrin-like [Source:MGI Symbol;Acc:MGI:700006]
ENSMUSG00000020477	73.71697389	58.55175495	88.56751203	64.26968074	52.94667915	43.10817129	125.6269552	142.9181951	103.1897057	118.815704	117.8299913	115.7728728	Mrps24	mitochondrial ribosomal protein S24 [Source:MGI Symbol;Acc:MGI:1928142]

ENSMUSG00000020481	0	0.082103588	0	0.066534346	0.074910221	0.013576645	0.12730689	0.105946597	0.066361477	0.382647553	0.31158011	0.394914092	Ankrd36	ankyrin repeat domain 36 [Source:MGI Symbol;Acc:MGI:1923639]
ENSMUSG00000020482	6.992767298	7.303116627	5.695961176	7.454352647	8.030777785	8.368455447	21.49728044	20.55445681	23.80145304	28.58062509	30.66527862	28.984174	Ccdc117	coiled-coil domain containing 117 [Source:MGI Symbol;Acc:MGI:2144383]
ENSMUSG00000020483	84.94077378	97.20879032	89.00078219	89.00755667	97.9130313	91.50386535	93.79008115	81.06716406	76.92044565	92.85270829	97.04806494	95.6813931	Dynll2	dynein light chain LC8-type 2 [Source:MGI Symbol;Acc:MGI:1915347]
ENSMUSG00000020484	89.71750512	87.5268731	77.99298871	83.6262239	81.96275373	73.64948458	74.73323136	72.04657725	73.89486304	92.60587735	90.3470623	95.57709578	Xbp1	X-box binding protein 1 [Source:MGI Symbol;Acc:MGI:98970]
ENSMUSG00000020485	81.40376768	61.46409381	91.07494475	70.08978574	59.29996974	46.53410564	79.46889659	88.45134555	63.74235185	93.03706884	95.73951066	98.48574621	Supt4a	SPT4A, DSIF elongation factor subunit [Source:MGI Symbol;Acc:MGI:107416]
ENSMUSG00000020486	4.919366834	4.414210746	4.251516352	3.010765599	2.785664753	2.773747849	1.112234082	0.569610437	0.505445862	1.885824633	1.276325184	1.30223728	Septin4	septin 4 [Source:MGI Symbol;Acc:MGI:1270156]
ENSMUSG00000020490	0.036729396	0	0.018801643	0	0.019295057	0	0.065582337	0	0	0	0.045860276	0.016275247	Btnl10	butyrophilin-like 10 [Source:MGI Symbol;Acc:MGI:2182073]
ENSMUSG00000020491	2.808386152	2.956800932	2.341238472	2.040433139	2.529136691	3.300320959	4.530982788	4.014182394	5.806660197	8.540919344	9.083627161	8.284351322	2810021J22I	RIKEN cDNA 2810021J22 gene [Source:MGI Symbol;Acc:MGI:1917194]
ENSMUSG00000020492	9.401893994	9.88389288	9.799024158	13.41084004	13.31924865	12.49455774	15.67850343	13.78907015	13.82273865	18.03176377	14.85319246	13.56170232	Ska2	spindle and kinetochore associated complex subunit 2 [Source:MGI Symbol;Acc:MGI:1913390]
ENSMUSG00000020493	4.860958876	6.491712167	4.596456022	8.295305306	9.327762337	11.72459734	6.529721534	5.651491837	7.582527642	9.984326004	8.804357466	10.56034154	Prr11	proline rich 11 [Source:MGI Symbol;Acc:MGI:2444496]
ENSMUSG00000020495	15.82882756	16.78034714	12.46572119	17.35524578	17.51796544	18.68313636	15.09593307	13.67684661	18.16926363	34.3422977	36.40537731	36.13140581	Smg8	smg-8 homolog, nonsense mediated mRNA decay factor (C. elegans) [Source:MGI Symbol;Acc:MGI:1921383]
ENSMUSG00000020496	87.09460466	81.43001464	88.4887427	85.51928501	79.83942112	73.16560482	97.46611256	103.3168315	94.68778359	90.3845162	96.46114899	89.25721457	Rnf187	ring finger protein 187 [Source:MGI Symbol;Acc:MGI:1914224]
ENSMUSG00000020513	4.1712556	5.032149307	4.245081237	4.494965205	4.747786462	4.425343814	4.167335583	4.058433278	4.483286437	6.99579308	7.750333701	6.38115543	Tubd1	tubulin, delta 1 [Source:MGI Symbol;Acc:MGI:1891826]
ENSMUSG00000020514	66.29519182	58.30870209	73.75125423	68.33746141	62.21040688	47.25820653	81.49008905	94.11668972	76.76665786	105.5917691	93.79427877	92.92004158	Mrpl22	mitochondrial ribosomal protein L22 [Source:MGI Symbol;Acc:MGI:1333794]
ENSMUSG00000020515	78.75659891	73.53008652	73.43189791	70.51189306	69.31470859	62.54994869	80.91447761	80.35255575	77.74983834	98.85298634	98.54541424	100.1175089	Cnot8	CCR4-NOT transcription complex, subunit 8 [Source:MGI Symbol;Acc:MGI:1916375]
ENSMUSG00000020516	26.24061003	32.53022487	23.91471726	27.46163781	31.76738758	35.33475081	24.14815028	24.7407254	29.38156931	56.60913978	57.80491167	58.38919653	Rps6kb1	ribosomal protein S6 kinase, polypeptide 1 [Source:MGI Symbol;Acc:MGI:1270849]
ENSMUSG00000020519	41.15930938	29.1872907	43.66951996	38.19987683	30.59711555	24.288653	41.28512119	42.6258477	31.66353422	29.15856358	25.95245365	25.31150233	Sap30l	SAP30-like [Source:MGI Symbol;Acc:MGI:1354709]
ENSMUSG00000020520	14.067518	13.89840903	13.65210602	11.68553225	11.15511756	11.69920922	5.173517499	5.331530177	5.889581106	11.03401141	11.95374358	11.64057091	Galnt10	polypeptide N-acetylgalactosaminyltransferase 10 [Source:MGI Symbol;Acc:MGI:1890480]
ENSMUSG00000020521	25.86736496	30.76122345	21.52313871	30.8561245	35.58084323	35.69809683	15.99428868	15.82340284	18.26989971	31.23187075	30.66213663	32.13856598	Rnft1	ring finger protein, transmembrane 1 [Source:MGI Symbol;Acc:MGI:1924142]
ENSMUSG00000020522	11.82985434	13.05650325	10.70799156	12.76346699	15.1998882	18.83825286	7.808005312	8.103043763	10.55311784	25.85410483	25.41162668	25.50996256	Mfap3	microfibrillar-associated protein 3 [Source:MGI Symbol;Acc:MGI:1924068]
ENSMUSG00000020523	49.02548962	56.93878137	57.92794155	50.3947133	56.17172935	51.27364204	55.52084135	53.8838242	52.80348061	65.87208739	61.89954787	65.8490159	Fam114a2	family with sequence similarity 114, member A2 [Source:MGI Symbol;Acc:MGI:1917629]
ENSMUSG00000020524	3.93223845	3.746956072	3.125458919	5.850097939	6.177370215	7.033256983	0.033648047	0	0.011693183	0.056186812	0.073202373	0.011133695	Gria1	glutamate receptor, ionotropic, AMPA1 (alpha 1) [Source:MGI Symbol;Acc:MGI:95808]
ENSMUSG00000020525	8.990676944	10.2721309	8.483603011	8.740446075	10.92596019	10.20947397	17.72993265	18.72002586	18.76826032	29.62712523	28.48936413	29.10416457	Ppm1d	protein phosphatase 1D magnesium-dependent, delta isoform [Source:MGI Symbol;Acc:MGI:1858214]
ENSMUSG00000020526	18.2928598	15.3020563	14.66445227	18.78839204	15.77456028	14.80965104	37.33636218	42.05598343	42.08373409	52.2777725	55.01816835	49.04258598	Znhit3	zinc finger, HIT type 3 [Source:MGI Symbol;Acc:MGI:3051596]
ENSMUSG00000020527	7.891889258	9.335929472	6.425927094	9.345696843	9.859518839	11.98706582	7.965897519	8.320955611	11.21147145	13.32011135	14.48297682	12.84275875	Myo19	myosin XIX [Source:MGI Symbol;Acc:MGI:1913446]
ENSMUSG00000020528	21.13006099	19.19363105	22.05638077	26.91606756	28.75199368	25.09823696	55.80179695	57.5273288	55.12259591	57.65264743	58.22702216	59.21600103	Prpsap2	phosphoribosyl pyrophosphate synthetase-associated protein 2 [Source:MGI Symbol;Acc:MGI:2384838]
ENSMUSG00000020530	68.47335216	76.09961115	63.41931436	80.72171077	84.17094262	93.0748333	49.47555447	46.03811698	53.13170199	75.43542021	72.39679608	75.5765122	Ggnbp2	gametogenetin binding protein 2 [Source:MGI Symbol;Acc:MGI:2387356]
ENSMUSG00000020532	7.855509078	10.34038908	7.796744018	8.249907392	11.41155086	17.58465211	16.48444868	16.80261622	22.5342599	32.16179638	31.7904994	32.30160344	Acaca	acetyl-Coenzyme A carboxylase alpha [Source:MGI Symbol;Acc:MGI:108451]
ENSMUSG00000020534	27.77688425	26.61750839	25.6061289	27.91201352	33.29391305	30.90809843	174.4718717	161.6572553	177.5181399	238.5264325	246.7055858	240.9131169	Shmt1	serine hydroxymethyltransferase 1 (soluble) [Source:MGI Symbol;Acc:MGI:98299]
ENSMUSG00000020536	34.58828445	43.4511137	32.48853452	40.99000862	42.68708521	49.39212982	38.57491596	35.54886401	44.93976906	53.03197674	53.92046927	52.50607646	Llgl1	LLGL1 scribble cell polarity complex component [Source:MGI Symbol;Acc:MGI:102682]
ENSMUSG00000020537	74.47238278	67.42994038	75.09812336	69.08760157	71.13661858	64.06505391	102.207847	102.2731029	100.0775743	138.6263404	135.8934393	132.2573369	Drg2	developmentally regulated GTP binding protein 2 [Source:MGI Symbol;Acc:MGI:1342307]
ENSMUSG00000020538	48.86932622	48.94113139	45.82515795	48.58954276	48.66215986	48.57977274	88.07116271	89.55498698	98.64767132	63.92782955	69.36160265	70.12708802	Srebf1	sterol regulatory element binding transcription factor 1 [Source:MGI Symbol;Acc:MGI:107606]
ENSMUSG00000020541	5.108099384	6.421658323	4.567366532	5.370449602	6.702736676	7.101887313	10.23599533	9.63445806	14.05561202	32.22294748	30.58081592	31.07564861	Tom1l1	target of myb1-like 1 (chicken) [Source:MGI Symbol;Acc:MGI:1919193]
ENSMUSG00000020542	0	0	0	0.042148507	0	0	0.013441167	0	0	0.040400245	0.075192881	0.120082625	Myocd	myocardin [Source:MGI Symbol;Acc:MGI:2137495]
ENSMUSG00000020544	9.001955912	8.630245964	6.393970249	11.89732677	14.43588944	14.79587919	25.84062256	26.28380599	17.35864125	30.62856183	30.00873351	30.57503169	Cox11	cytochrome c oxidase assembly protein 11, copper chaperone [Source:MGI Symbol;Acc:MGI:1917052]
ENSMUSG00000020545	0	0	0	0	0	0.035740481	0	0.046484021	0	0	0	0	Lrrc72	leucine rich repeat containing 72 [Source:MGI Symbol;Acc:MGI:1920830]
ENSMUSG00000020546	11.42886696	13.24570028	8.912196041	13.25854786	15.68175628	20.74659989	4.551289383	4.247999364	5.755566686	11.88550544	12.04108816	12.13467968	Stxbp4	syntaxin binding protein 4 [Source:MGI Symbol;Acc:MGI:1342296]
ENSMUSG00000020547	61.50970863	58.44421251	58.50171948	75.4764621	68.44981284	67.73502949	272.1172511	275.1450395	274.0205104	140.1699903	135.8280945	137.9809051	Bzw2	basic leucine zipper and W2 domains 2 [Source:MGI Symbol;Acc:MGI:1914162]
ENSMUSG00000020548	0.078806754	0.045375014	0	0	0.041399559	0	0.105535313	0.039034659	0.073350094	0.140981562	0.09839801	0	1700086D15	RIKEN cDNA 1700086D15 gene [Source:MGI Symbol;Acc:MGI:1921532]
ENSMUSG00000020549	22.60313745	21.91054767	22.53071933	23.03958211	23.00156847	22.89117797	48.87302692	46.55460191	49.56535908	75.52007467	78.31244378	75.10780428	Elac2	elaC ribonuclease Z 2 [Source:MGI Symbol;Acc:MGI:1890496]
ENSMUSG00000020553	2.322874664	1.765076392	1.779562072	1.636810885	1.361396293	1.275815713	2.76507947	2.974260398	2.897423008	6.402882397	6.852953406	6.679910223	Pctp	phosphatidylcholine transfer protein [Source:MGI Symbol;Acc:MGI:107375]
ENSMUSG00000020561	13.49856909	16.83689227	12.75894604	20.62733845	21.62136965	27.68801877	38.44054963	36.3531477	44.95148913	45.82513722	44.25001272	46.11658306	Twistnb	twist basic helix-loop-helix transcription factor 1 neighbor [Source:MGI Symbol;Acc:MGI:106292]
ENSMUSG00000020562	0.063935346	0.147249675	0.130913076	0.238653657	0.134348643	0.38958717	0.856200314	2.280134498	1.368693143	0.686263888	0.798295887	0.793255075	Efcab10	EF-hand calcium binding domain 10 [Source:MGI Symbol;Acc:MGI:1922290]
ENSMUSG00000020564	9.516782947	11.68620652	7.965422599	7.702396692	9.975210705	12.0927204	9.623847089	7.886228636	9.613475575	7.302224228	7.846673978	7.13493028	Atxn7l1	ataxin 7-like 1 [Source:MGI Symbol;Acc:MGI:3584458]
ENSMUSG00000020566	0.368464691	0.282870689	0.293402237	0.267435152	0.25808745	0.187101926	0.21930459	0.811148386	0.495374852	0.84226758	1.465390719	1.233604909	Atp6v1c2	ATPase, H+ transporting, lysosomal V1 subunit C2 [Source:MGI Symbol;Acc:MGI:1916025]
ENSMUSG00000020570	59.85729989	63.46353267	48.73760489	60.51266947	65.0521497	68.88630674	32.81584045	36.00528835	39.01242616	45.22390196	42.97218825	43.64790554	Sypl	synaptophysin-like protein [Source:MGI Symbol;Acc:MGI:108081]
ENSMUSG00000020571	426.1931126	382.5970523	429.4180102	384.2612068	377.9074219	328.9027565	380.6114749	405.7980701	378.6685059	344.1550382	344.1393522	353.2114258	Pdia6	protein disulfide isomerase associated 6 [Source:MGI Symbol;Acc:MGI:1919103]
ENSMUSG00000020572	47.27286811	55.50942213	38.75356484	56.72411288	65.02535171	66.10884485	80.19366617	78.25822467	92.83663408	91.11595058	84.90059562	87.24317298	Nampt	nicotinamide phosphoribosyltransferase [Source:MGI Symbol;Acc:MGI:1929865]
ENSMUSG00000020573	0.399151093	0.512173631	0.431999273	0	0.047928246	0.060805233	0.050907572	0.033892802	0.031844011	1.264910636	1.347997964	1.142066851	Pik3cg	phosphatidylinositol-4,5-bisphosphate 3-kinase catalytic subunit gamma [Source:MGI Symbol;Acc:MGI:1353576]
ENSMUSG00000020576	11.83115597	13.08308193	9.861931207	12.49706525	14.29951099	17.58855398	8.509998874	7.065516877	10.16848582	12.93940211	13.25671405	12.76054299	Nbas	neuroblastoma amplified sequence [Source:MGI Symbol;Acc:MGI:1918419]
ENSMUSG00000020577	1.844615839	1.430563443	2.35099498	1.791624995	1.384331708	1.86378945	46.07765961	48.33166983	48.73873121	29.59835734	30.70913208	31.26030769	Tspan13	tetraspanin 13 [Source:MGI Symbol;Acc:MGI:1913359]
ENSMUSG00000020580	28.4932764	33.67035802	24.67144182	33.43361186	39.37769537	52.78605676	13.30171794	11.78675937	16.41304476	35.31902321	34.32388975	35.20565736	Rock2	Rho-associated coiled-coil containing protein kinase 2 [Source:MGI Symbol;Acc:MGI:107926]
ENSMUSG00000020581	0	0	0	0	0	0	0	0	0	0	0	0.045074355	Agr2	anterior gradient 2 [Source:MGI Symbol;Acc:MGI:1344405]
ENSMUSG00000020583	0.071381035	0.049319359	0.087695256	0.106578574	0.029998884	0.097865357	4.40993348	3.875077064	4.92973962	2.107005458	2.400467434	2.467125552	Matn3	matrilin 3 [Source:MGI Symbol;Acc:MGI:1328350]
ENSMUSG00000020585	422.2519231	456.7889638	371.9470871	392.9741384	401.4947438	380.6629481	131.0178356	127.2043769	130.4703323	212.2590723	212.5377027	209.1610254	Laptm4a	lysosomal-associated protein transmembrane 4A [Source:MGI Symbol;Acc:MGI:108017]
ENSMUSG00000020589	1.982167851	1.689693324	1.528585409	1.489390144	1.3523278	1.852913521	6.986608219	6.706908732	6.70880193	6.758132294	7.081948756	6.958142944	Fam49a	family with sequence similarity 49, member A [Source:MGI Symbol;Acc:MGI:1261783]
ENSMUSG00000020590	13.96701013	18.69975854	12.61096877	18.68699203	22.96660365	29.76193818	6.895744239	6.017962855	7.752964413	12.34264076	12.94282086	12.21386349	Snx13	sorting nexin 13 [Source:MGI Symbol;Acc:MGI:2661416]
ENSMUSG00000020591	0	0	0.061871138	0	0	0.046030928	0	0	0	0.027028066	0	0	Ntsr2	neurotensin receptor 2 [Source:MGI Symbol;Acc:MGI:108018]
ENSMUSG00000020592	126.5820949	131.080683	124.5282637	129.8048431	128.3841977	127.1928111	60.9221197	60.86072437	59.0731838	68.86211095	68.52030593	69.87117444	Sdc1	syndecan 1 [Source:MGI Symbol;Acc:MGI:1349162]
ENSMUSG00000020593	5.785919437	7.47917217	5.127527395	5.964866891	6.115076811	8.753093473	10.07585994	9.333961867	11.35595128	9.297252573	9.832270096	9.444556782	Lpin1	lipin 1 [Source:MGI Symbol;Acc:MGI:1891340]
ENSMUSG00000020594	47.81782443	54.03820068	38.93241354	58.51015906	64.82822055	88.18375005	46.30554566	44.66135166	58.28782762	81.12474092	80.38675143	80.71107776	Pum2	pumilio RNA-binding family member 2 [Source:MGI Symbol;Acc:MGI:1931751]
ENSMUSG00000020598	2.974403527	3.10997808	2.877034152	5.466185914	5.464109724	6.05999703	0.293243161	0.180771195	0.135874984	2.187187232	2.810060205	2.490444157	Nrcam	neuronal cell adhesion molecule [Source:MGI Symbol;Acc:MGI:104750]
ENSMUSG00000020599	0.305889724	0.140899097	0.146144906	0.228361011	0.12855446	0.201925246	0.619007127	0.404036471	0.474516013	1.769352009	1.544708848	1.138565401	Rgs9	regulator of G-protein signaling 9 [Source:MGI Symbol;Acc:MGI:1338824]
ENSMUSG00000020600	0.377697732	0.200740779	0.237959488	0.325348909	0.763138392	0.951575211	0.051876913	0	0	0.07796347	0.072552752	0.180236552	Slc7a15	solute carrier family 7 (cationic amino acid transporter, y+ system), member 15 [Source:MGI Symbol;Acc:MGI:3045351]
ENSMUSG00000020601	16.71009236	17.97973929	14.11382773	18.95595009	19.99809713	21.91563759	5.268056444	3.724588634	5.224858967	16.18456353	13.23572644	14.76261723	Trib2	tribbles pseudokinase 2 [Source:MGI Symbol;Acc:MGI:2145021]
ENSMUSG00000020604	1.360028498	1.566142728	0.98427371	0.853398167	1.527474516	1.285956123	0.648970287	0.58073362	0.523803617	1.069692798	1.151605261	0.914358478	Arsg	arylsulfatase G [Source:MGI Symbol;Acc:MGI:1921258]
ENSMUSG00000020605	43.62380895	47.91182987	50.18096448	54.31984703	51.84535817	45.62163826	28.58230495	26.60429541	31.92234445	28.69587039	28.90677883	26.11561728	Hs1bp3	HCLS1 binding protein 3 [Source:MGI Symbol;Acc:MGI:1913224]

ENSMUSG00000020607	0.075887986	0.349555666	0.174810517	0.070817421	0.139531847	0.101154385	0.474257458	0.20673912	0.370825477	1.832760303	1.547642768	1.899919854	Lratd1	LRAT domain containing 1 [Source:MGI Symbol;Acc:MGI:2145011]
ENSMUSG00000020608	53.79598309	63.81232219	46.81348092	67.35484541	76.59692897	90.01003932	45.04576909	47.02407885	57.25445239	53.4451992	53.69077428	56.13295951	Smc6	structural maintenance of chromosomes 6 [Source:MGI Symbol;Acc:MGI:1914491]
ENSMUSG00000020609	0.016876123	0.045345354	0.028796085	0	0.01773107	0.038562712	0.010044412	0.005572735	0.005235868	0.266683516	0.243492944	0.209384494	Apob	apolipoprotein B [Source:MGI Symbol;Acc:MGI:88052]
ENSMUSG00000020610	28.08117149	26.19859447	28.91807995	32.87149335	29.70584903	28.48367949	24.2116609	24.6064919	28.00371706	22.41444785	20.88174158	22.4998748	Amz2	archaelysin family metallopeptidase 2 [Source:MGI Symbol;Acc:MGI:104837]
ENSMUSG00000020611	21.70280528	24.823789	19.85278112	26.93927139	32.2989597	37.39777984	22.08012393	20.99356141	24.12913257	32.93838683	34.04476544	33.44310725	Gna13	guanine nucleotide binding protein, alpha 13 [Source:MGI Symbol;Acc:MGI:95768]
ENSMUSG00000020612	254.8610433	278.0941106	242.3779121	260.7971922	266.0202925	274.9707615	97.78531556	99.66889345	110.8818259	179.1961746	178.8950927	186.0518526	Prkar1a	protein kinase, cAMP dependent regulatory, type I, alpha [Source:MGI Symbol;Acc:MGI:104878]
ENSMUSG00000020614	22.2266361	21.87616584	26.18337388	10.70316214	10.05460943	9.188301591	0.678408019	0.63515336	0.618861061	2.081578755	1.699917931	1.809839847	Fam20a	family with sequence similarity 20, member A [Source:MGI Symbol;Acc:MGI:2388266]
ENSMUSG00000020617	0.401453237	0.210133654	0.560461198	0.306515098	0.191723148	0.055596303	0	0	0	0.195867513	0.060758058	0.064686861	1700012B07	RIKEN cDNA 1700012B07 gene [Source:MGI Symbol;Acc:MGI:1916574]
ENSMUSG00000020620	3.035032806	3.574428027	3.234358864	3.585361839	4.123682773	6.157606955	0.067740204	0.068332555	0.102723056	0.758900267	0.625847936	0.67854319	Abca8b	ATP-binding cassette, sub-family A (ABC1), member 8b [Source:MGI Symbol;Acc:MGI:1351668]
ENSMUSG00000020621	13.37371919	10.64644998	10.65588214	15.27460001	14.90652659	13.15389747	3.426189687	3.830566043	3.517830877	4.446920439	4.186702336	3.710228564	Rdh14	retinol dehydrogenase 14 (all-trans and 9-cis) [Source:MGI Symbol;Acc:MGI:1920402]
ENSMUSG00000020622	0.024214118	0.111535207	0	0.045192434	0.025440809	0.129104189	0.021617813	0	0	0	0	0	Nt5c1b	5'-nucleotidase, cytosolic IB [Source:MGI Symbol;Acc:MGI:1918131]
ENSMUSG00000020623	9.758485713	10.86693427	10.45726313	13.30943938	14.81593486	15.68252202	10.07641967	8.97665827	11.35349778	18.12891126	20.68675612	18.389326	Map2k6	mitogen-activated protein kinase kinase 6 [Source:MGI Symbol;Acc:MGI:1346870]
ENSMUSG00000020624	0.029411133	0.033868432	0	0.109783945	0.061802212	0	0	0	0	0	0	0	4933434M1(	RIKEN cDNA 4933434M16 gene [Source:MGI Symbol;Acc:MGI:1918453]
ENSMUSG00000020627	0.31748833	0.233580418	0.189607964	0.172827021	0.129722578	0.100760465	0.165343705	0.078629356	0.123127131	0.189323876	0.190866727	0.179761592	Klhl29	kelch-like 29 [Source:MGI Symbol;Acc:MGI:2683857]
ENSMUSG00000020628	15.22116196	16.03620998	13.48845841	17.15760993	16.87385403	17.03172677	10.78980335	10.39761526	11.19403243	10.48115677	10.8443157	10.25400721	Trappc12	trafficking protein particle complex 12 [Source:MGI Symbol;Acc:MGI:2445089]
ENSMUSG00000020629	49.11251587	41.62159312	47.125663	45.27071175	41.84397838	37.89591409	76.07927844	76.05244269	81.25320881	49.40780799	52.17602512	50.86752123	Adi1	acireductone dioxygenase 1 [Source:MGI Symbol;Acc:MGI:2144929]
ENSMUSG00000020630	16.75916373	16.38886606	16.34086561	15.88762029	16.62995327	16.94421932	30.1915816	29.28468208	24.75990821	17.75681292	17.82540016	18.30025797	Rnaseh1	ribonuclease H1 [Source:MGI Symbol;Acc:MGI:1335073]
ENSMUSG00000020633	0.03498251	0	0	0	0	0	0	0	0	0	0.029119411	0.124009446	Dcdc2c	doublecortin domain containing 2C [Source:MGI Symbol;Acc:MGI:1915761]
ENSMUSG00000020634	4.911826348	8.138815066	3.435893481	6.22212983	7.799368971	11.02060301	11.68716376	10.70049689	15.05980491	31.60943519	32.02428278	33.09053713	Ubxn2a	UBX domain protein 2A [Source:MGI Symbol;Acc:MGI:2442310]
ENSMUSG00000020635	14.29588057	11.19121629	14.27552461	10.44910471	10.21071675	8.957874708	20.30768881	25.81270646	22.28594356	10.45663243	8.03095244	7.988562684	Fkbp1b	FK506 binding protein 1b [Source:MGI Symbol;Acc:MGI:1336205]
ENSMUSG00000020636	0.029839451	0.171808307	0.030549411	0	0	0.02272817	0	0	0	0.026690684	0	0.026444455	Allc	allantoicase [Source:MGI Symbol;Acc:MGI:2136971]
ENSMUSG00000020638	6.786607023	12.4612332	5.921386476	8.683597646	9.752255123	10.01872501	1.145158251	0.9703445	0.933394859	6.216484541	5.105603388	6.241808789	Cmpk2	cytidine monophosphate (UMP-CMP) kinase 2, mitochondrial [Source:MGI Symbol;Acc:MGI:99830]
ENSMUSG00000020639	0.433973654	0.399794377	0.377654187	0.24298625	0.205181475	0.561934797	0.503674335	0.709356013	0.767457266	0.970447967	0.866974293	0.961495328	Pfn4	profilin family, member 4 [Source:MGI Symbol;Acc:MGI:1920121]
ENSMUSG00000020640	15.64672285	19.27220019	12.54821672	20.57060522	24.72355093	31.60560635	10.88763244	8.844293534	14.8346055	21.2266966	21.75827302	22.70301277	Itsn2	intersectin 2 [Source:MGI Symbol;Acc:MGI:1338049]
ENSMUSG00000020641	19.39355416	25.5356968	17.87262722	30.21549207	35.14946752	40.31204538	0.54878363	0.471929028	0.457704589	4.151202087	4.617818589	4.80746977	Rsad2	radical S-adenosyl methionine domain containing 2 [Source:MGI Symbol;Acc:MGI:1929628]
ENSMUSG00000020642	2.579541588	4.443231124	2.330219784	1.820560708	1.70812286	2.410582878	6.831457676	4.681327354	7.081739136	6.805680704	7.343812756	7.357633499	Rnf144a	ring finger protein 144A [Source:MGI Symbol;Acc:MGI:1344401]
ENSMUSG00000020644	14.24030727	13.52960327	12.81577257	7.663315641	8.304887645	6.489209233	6.645028367	6.70313545	6.297936623	16.09396735	14.20898205	14.80069138	Id2	inhibitor of DNA binding 2 [Source:MGI Symbol;Acc:MGI:96397]
ENSMUSG00000020646	7.337614839	8.706729138	7.954090143	9.152937355	8.553770369	9.522717861	13.00869669	13.55003599	14.15781143	11.96841293	10.97673873	10.59174074	Mboat2	membrane bound O-acyltransferase domain containing 2 [Source:MGI Symbol;Acc:MGI:1914466]
ENSMUSG00000020647	3.07232636	5.206090432	2.509821136	3.936615474	5.63640521	6.838530388	6.167978789	4.147465019	8.163922489	9.654029301	10.3753657	10.35499521	Ncoa1	nuclear receptor coactivator 1 [Source:MGI Symbol;Acc:MGI:1276523]
ENSMUSG00000020648	6.409616013	7.744002442	5.916663641	11.37436735	12.08867121	10.68457083	12.94566511	12.80336817	14.13211818	14.8970517	15.91861132	14.38713986	Dus4l	dihydrouridine synthase 4-like (S. cerevisiae) [Source:MGI Symbol;Acc:MGI:1919166]
ENSMUSG00000020649	65.30027554	71.66360402	57.08178899	89.00216302	89.30234716	97.31229895	448.7030798	428.4347967	479.5321822	204.5012016	211.7798878	203.2524101	Rrm2	ribonucleotide reductase M2 [Source:MGI Symbol;Acc:MGI:98181]
ENSMUSG00000020650	13.47586566	12.87875586	9.519413341	15.4728173	18.29169209	19.4266862	6.675796325	6.892279114	9.168789506	10.01821263	9.715344388	9.853761544	Bcap29	B cell receptor associated protein 29 [Source:MGI Symbol;Acc:MGI:101917]
ENSMUSG00000020651	0.124359791	0.057282677	0	0.069630308	0.026131976	0	0.11102559	0.024639218	0.092599194	0.177979035	0.455474717	0.286547839	Slc26a4	solute carrier family 26, member 4 [Source:MGI Symbol;Acc:MGI:1346029]
ENSMUSG00000020652	4.638839494	4.543079014	4.971135539	7.282242832	5.192693493	6.719912544	11.26319651	10.54372354	11.84325035	11.80814686	10.08646519	10.35447639	Cenpo	centromere protein O [Source:MGI Symbol;Acc:MGI:1923800]
ENSMUSG00000020653	12.7722302	16.24350512	11.68052089	19.24712161	20.64264123	25.32537958	19.57781819	18.23047619	22.09323028	18.84638023	18.52511948	20.3795689	Klf11	Kruppel-like factor 11 [Source:MGI Symbol;Acc:MGI:2653368]
ENSMUSG00000020654	1.282040606	2.337531463	1.326216098	0.984520406	1.094428563	1.200290017	2.479916092	2.712072867	3.53009281	5.267906012	7.203393951	6.0477698	Adcy3	adenylate cyclase 3 [Source:MGI Symbol;Acc:MGI:99675]
ENSMUSG00000020656	0.226676261	0.399221384	0.464138986	0.08710082	0.308206695	0.284373281	2.678445047	2.24555132	2.171862881	3.100982726	3.263142668	3.285078247	Grhl1	grainyhead like transcription factor 1 [Source:MGI Symbol;Acc:MGI:2182540]
ENSMUSG00000020657	2.553710754	4.109934796	2.787718371	4.435975921	3.862991807	5.214315046	6.702341361	6.172137404	9.192547471	7.224233945	7.97780244	7.143954972	Dnajc27	DnaJ heat shock protein family (Hsp40) member C27 [Source:MGI Symbol;Acc:MGI:2443036]
ENSMUSG00000020658	1.492649344	1.768445021	1.263673621	1.218801362	1.598426199	2.153596419	2.460190788	1.777261829	2.578214224	3.24798382	4.169476165	3.790395463	Efr3b	EFR3 homolog B [Source:MGI Symbol;Acc:MGI:2444851]
ENSMUSG00000020659	4.046426861	5.25706194	3.368790749	4.619793867	5.372673038	6.231918495	6.113559277	6.152335579	6.994460841	7.968056421	8.88327604	8.1178557	Cbll1	Casitas B-lineage lymphoma-like 1 [Source:MGI Symbol;Acc:MGI:2144842]
ENSMUSG00000020660	0.541920502	1.24809863	2.219256937	0.606853569	0.341624572	0.577879457	2.27392713	2.469507549	1.866270285	1.163364223	0.586422394	1.536842506	Pomc	pro-opiomelanocortin-alpha [Source:MGI Symbol;Acc:MGI:97742]
ENSMUSG00000020661	15.7931584	17.07795267	14.63353578	15.03163757	16.74886493	20.03953671	54.94448147	49.83409131	60.31498723	67.20489137	68.89046187	67.58581515	Dnmt3a	DNA methyltransferase 3A [Source:MGI Symbol;Acc:MGI:1261827]
ENSMUSG00000020664	93.51154849	104.8086048	80.75654247	117.0307247	125.6798536	118.789319	82.46347401	79.72167003	90.48978313	104.0198543	99.94668798	103.9206154	Dld	dihydrolipoamide dehydrogenase [Source:MGI Symbol;Acc:MGI:107450]
ENSMUSG00000020668	9.188228434	13.14082968	9.104323782	12.60540835	12.90610265	16.39772779	3.241017773	3.038729496	3.706314546	4.732333341	4.86069435	5.025363175	Kif3c	kinesin family member 3C [Source:MGI Symbol;Acc:MGI:107979]
ENSMUSG00000020669	7.610648391	6.83539397	6.682224558	6.573494368	6.521177003	5.815653347	4.749632787	4.269904606	3.576226467	4.560394413	3.936370974	4.234564115	Sh3yl1	Sh3 domain YSC-like 1 [Source:MGI Symbol;Acc:MGI:1346118]
ENSMUSG00000020671	38.39860784	46.79911553	32.65358939	47.05717855	52.76234137	57.71138391	24.11179995	20.78510592	26.8362376	34.23708936	31.36143153	32.77063457	Rab10	RAB10, member RAS oncogene family [Source:MGI Symbol;Acc:MGI:105066]
ENSMUSG00000020672	0.175626481	0	0.287688165	0.327783493	0.184523748	0.026754303	0.250872516	0.03479661	0.228852294	0.125674992	0.087714797	0.031128902	Sntg2	syntrophin, gamma 2 [Source:MGI Symbol;Acc:MGI:1919541]
ENSMUSG00000020673	0.107236107	0.030871972	0.027446885	0	0	0	0	0.026558161	0.149716454	0.35970062	0.02231581	0.047517638	Tpo	thyroid peroxidase [Source:MGI Symbol;Acc:MGI:98813]
ENSMUSG00000020674	51.77315613	65.53761344	49.58227064	52.54697968	58.50083121	75.04471819	6.379066261	5.779555828	6.711466587	29.72790821	32.5647643	28.89595794	Pxdn	peroxidasin [Source:MGI Symbol;Acc:MGI:1916925]
ENSMUSG00000020676	421.3371798	387.8325765	413.2035927	149.6663365	143.4800048	119.2828958	1.775178099	2.08795742	1.443546127	12.5566101	10.49348385	14.37913596	Ccl11	chemokine (C-C motif) ligand 11 [Source:MGI Symbol;Acc:MGI:103576]
ENSMUSG00000020677	23.58071345	27.02220116	24.96450892	29.03306428	28.44215973	29.76594617	26.83300106	28.47788758	28.48747011	52.72070954	55.02496505	52.96688949	Ddx52	DEAD (Asp-Glu-Ala-Asp) box polypeptide 52 [Source:MGI Symbol;Acc:MGI:1925644]
ENSMUSG00000020679	0.04661808	0.24157402	0.023863624	0.195764518	0.04897976	0.053262222	0.249717361	0.461818502	0.195255885	0.45868649	0.620877581	0.640368389	Hnf1b	HNF1 homeobox B [Source:MGI Symbol;Acc:MGI:98505]
ENSMUSG00000020680	131.6498256	142.5688517	149.7622073	179.9340335	194.5027474	164.2147905	239.2090606	229.2306762	203.1941614	218.7843557	216.7846994	212.3879135	Taf15	TATA-box binding protein associated factor 15 [Source:MGI Symbol;Acc:MGI:1917689]
ENSMUSG00000020681	1.814365445	1.749605797	1.253457925	1.197584015	0.898896971	1.07861021	1.67250136	2.089645602	2.416404069	2.427763391	2.296110975	2.523020434	Ace	angiotensin I converting enzyme (peptidyl-dipeptidase A) 1 [Source:MGI Symbol;Acc:MGI:87874]
ENSMUSG00000020682	1.322681662	2.403698885	1.608055243	2.314321489	2.757663991	3.636310647	1.27311476	1.146516557	1.308041452	2.421673196	2.528857202	3.186899816	Mmp28	matrix metallopeptidase 28 (epilysin) [Source:MGI Symbol;Acc:MGI:2153062]
ENSMUSG00000020684	6.752357333	5.280911257	6.540650158	4.294252867	3.88781569	3.179838827	0.945900486	0.986927014	0.838956868	1.075003838	1.355802343	1.065086643	Rasl10b	RAS-like, family 10, member B [Source:MGI Symbol;Acc:MGI:2685575]
ENSMUSG00000020686	0	0.019153272	0	0	0	0	0.01484919	0	0	0.014877451	0	0	Gas2l2	growth arrest-specific 2 like 2 [Source:MGI Symbol;Acc:MGI:3652048]
ENSMUSG00000020687	29.40809372	37.45797078	25.89958609	36.71872528	46.70021248	57.67829164	26.33827815	23.56174734	30.51943592	74.41898144	74.74420476	73.9642576	Cdc27	cell division cycle 27 [Source:MGI Symbol;Acc:MGI:102685]
ENSMUSG00000020689	4.38755053	4.984213087	5.046931027	4.394750724	4.636522413	5.025779936	3.1911552	2.936820216	3.48459153	11.41001523	12.62051321	12.01036368	Itgb3	integrin beta 3 [Source:MGI Symbol;Acc:MGI:96612]
ENSMUSG00000020690	0	0	0	0	0	0	0	0	0	0	0	0	Efcab3	EF-hand calcium binding domain 3 [Source:MGI Symbol;Acc:MGI:1918144]
ENSMUSG00000020691	16.18829489	19.05177012	14.48520191	21.54208405	24.69619196	23.6249148	34.3392375	35.79416556	41.22440879	47.06015452	53.25368393	48.77798192	Mettl2	methyltransferase like 2 [Source:MGI Symbol;Acc:MGI:1289171]
ENSMUSG00000020692	26.74873629	23.72404264	27.53265637	28.72586975	25.02603337	23.77579622	63.49598089	63.98634504	61.4146587	78.82301149	82.42678263	85.71391746	Nle1	notchless homolog 1 [Source:MGI Symbol;Acc:MGI:2429770]
ENSMUSG00000020694	34.32184366	39.25697206	30.66002164	40.52635959	46.57826739	47.17472667	38.2083226	35.66737598	40.96914669	59.23685538	55.10827175	53.50907593	Tlk2	tousled-like kinase 2 (Arabidopsis) [Source:MGI Symbol;Acc:MGI:1346023]
ENSMUSG00000020695	233.5582434	256.7626819	243.0331817	225.3489314	236.8793421	253.3735121	5.237600871	4.676334533	5.785881936	31.78080835	35.50964269	33.96669117	Mrc2	mannose receptor, C type 2 [Source:MGI Symbol;Acc:MGI:107818]
ENSMUSG00000020696	4.441549812	5.40181246	3.238901409	5.264599412	6.025592594	7.30026175	5.440133264	4.338233685	6.831999409	11.62584714	11.90868949	11.54621815	Rffl	ring finger and FYVE like domain containing protein [Source:MGI Symbol;Acc:MGI:1914588]
ENSMUSG00000020697	5.688263468	7.141207874	4.502785369	6.484752468	7.732393803	11.61328159	8.572991283	8.002331204	11.43481737	27.60380552	29.8250234	29.50590342	Lig3	ligase III, DNA, ATP-dependent [Source:MGI Symbol;Acc:MGI:109152]
ENSMUSG00000020698	1.458592808	1.056550634	1.228356895	1.339181054	1.45833285	1.272255585	0.861131229	1.608082827	1.926913797	1.64136762	1.351210721	1.438584157	Cct6b	chaperonin containing Tcp1, subunit 6b (zeta) [Source:MGI Symbol;Acc:MGI:1329013]

ENSMUSG00000020700	15.62409744	29.39216126	11.94733264	23.79274201	28.00823836	37.8502025	18.98523315	14.2177474	23.49927721	44.39967843	43.41299946	45.70557021	Map3k3	mitogen-activated protein kinase kinase kinase 3 [Source:MGI Symbol;Acc:MGI:1346874]
ENSMUSG00000020701	2.954298437	3.121859627	3.220297773	1.524405055	0.85815467	0.741254102	0.077574423	0.15494048	0.1617494	0.917120362	1.330837053	0.939461707	Tmem132e	transmembrane protein 132E [Source:MGI Symbol;Acc:MGI:2685490]
ENSMUSG00000020702	0	0	0	0.037041954	0	0	0.177190286	0.078645475	0.073891423	0	0	0.140711825	Ccl1	chemokine (C-C motif) ligand 1 [Source:MGI Symbol;Acc:MGI:98258]
ENSMUSG00000020703	0	0	0	0	0	0	0	0	0	0	0	0	5530401A14	RIKEN cDNA 5530401A14 gene [Source:MGI Symbol;Acc:MGI:1918638]
ENSMUSG00000020704	0.056099723	0.016150429	0	0	0.014735436	0.021365072	0.313028472	0.347342305	0.261076607	0.075269817	0.058371701	0.111863149	Asic2	acid-sensing (proton-gated) ion channel 2 [Source:MGI Symbol;Acc:MGI:1100867]
ENSMUSG00000020705	51.58397262	56.64390318	47.09715439	58.31986278	64.36300979	66.15115386	54.10494258	51.33085699	57.66502607	107.5348253	112.2733655	107.9291279	Ddx42	DEAD (Asp-Glu-Ala-Asp) box polypeptide 42 [Source:MGI Symbol;Acc:MGI:1919297]
ENSMUSG00000020706	85.51808323	75.13577803	90.18403822	131.7173334	74.51968468	66.02165429	155.731894	165.5344818	161.971424	217.5422916	214.9012529	373.761245	Ftsj3	FtsJ RNA methyltransferase homolog 3 (E. coli) [Source:MGI Symbol;Acc:MGI:1860295]
ENSMUSG00000020707	17.59750063	14.62116854	16.72389136	15.97131431	18.64501827	15.41142472	3.34762749	2.684803177	3.006277983	8.733680031	8.622008851	10.39689205	Rnf135	ring finger protein 135 [Source:MGI Symbol;Acc:MGI:1919206]
ENSMUSG00000020708	149.4088821	112.4828857	173.0879558	121.5865089	107.6658622	87.94563766	187.027891	201.8986646	161.4970951	177.1582629	183.0757783	174.6233753	Psmc5	protease (prosome, macropain) 26S subunit, ATPase 5 [Source:MGI Symbol;Acc:MGI:105047]
ENSMUSG00000020709	0.504944534	0.581469588	0.184628039	0.067315135	0.227367905	0.08241581	0.772805111	1.143358865	1.174954152	3.290672733	3.962973649	3.324243158	Adap2	ArfGAP with dual PH domains 2 [Source:MGI Symbol;Acc:MGI:2663075]
ENSMUSG00000020712	0.419151175	0.618426222	0.227972071	0.684503732	0.357813174	0.887941503	0.128634215	0.012975905	0.158489793	0.269474346	0.250772644	0.348245705	Tcam1	testicular cell adhesion molecule 1 [Source:MGI Symbol;Acc:MGI:1923120]
ENSMUSG00000020713	0.349419275	0.057482037	0.204418794	0.139745282	0.157337535	0.114062717	0	0.197799757	0.046460733	0.044649613	0	0.088475417	Gh	growth hormone [Source:MGI Symbol;Acc:MGI:95707]
ENSMUSG00000020715	8.719848307	13.34587175	8.878622276	13.38942426	16.6074773	21.21737445	4.161373286	4.209184666	5.223802373	11.04720921	12.31287576	12.23793662	Ern1	endoplasmic reticulum (ER) to nucleus signalling 1 [Source:MGI Symbol;Acc:MGI:1930134]
ENSMUSG00000020716	13.26966465	15.25869853	12.19652664	14.82576235	18.21782811	23.16884585	6.62707407	6.378734758	8.278365912	15.82586894	14.96611005	14.31677216	Nf1	neurofibromin 1 [Source:MGI Symbol;Acc:MGI:97306]
ENSMUSG00000020717	0.922163306	0.881592676	0.676904775	0.600759676	0.822634179	1.086724502	16.20165499	15.92651972	17.19862471	37.55417018	37.71417054	36.79139126	Pecam1	platelet/endothelial cell adhesion molecule 1 [Source:MGI Symbol;Acc:MGI:97537]
ENSMUSG00000020718	3.985151399	4.043548555	4.165562519	5.409276807	4.626239831	4.627420012	7.488906137	7.868113079	7.781570892	7.179102536	7.353594253	7.853797727	Polg2	polymerase (DNA directed), gamma 2, accessory subunit [Source:MGI Symbol;Acc:MGI:1354947]
ENSMUSG00000020719	458.017838	527.2502473	386.6308441	542.2771145	626.6136913	687.9195164	538.8173984	503.1094579	610.3841488	861.3438314	839.2129218	832.7168683	Ddx5	DEAD (Asp-Glu-Ala-Asp) box polypeptide 5 [Source:MGI Symbol;Acc:MGI:105037]
ENSMUSG00000020720	92.624876	83.25353902	93.4480409	91.02697877	90.39039856	81.39293924	129.4912783	132.2564027	128.7011909	177.5628814	173.4509629	170.8345277	Psmd12	proteasome (prosome, macropain) 26S subunit, non-ATPase, 12 [Source:MGI Symbol;Acc:MGI:1914247]
ENSMUSG00000020721	10.76516366	11.90274933	10.34069765	10.66623657	12.4596063	15.7377368	19.26964895	18.65212773	22.8937836	40.94321666	44.12624779	44.35693972	Helz	helicase with zinc finger domain [Source:MGI Symbol;Acc:MGI:1925705]
ENSMUSG00000020722	0.034450351	0	0	0	0.036195611	0.026240209	0	0	0	0.030815025	0.028676442	0	Cacng1	calcium channel, voltage-dependent, gamma subunit 1 [Source:MGI Symbol;Acc:MGI:1206582]
ENSMUSG00000020723	0.285247497	0.303209683	0.292034295	0.573328597	0.414966716	0.284119506	0.195894207	0.108683956	0.306342311	0.412160782	0.675790103	0.583369271	Cacng4	calcium channel, voltage-dependent, gamma subunit 4 [Source:MGI Symbol;Acc:MGI:1859167]
ENSMUSG00000020728	3.264635782	3.31910631	2.107763167	3.897330043	4.140748229	3.897935469	1.759258525	1.04889304	1.916227346	2.551833724	2.937816171	2.267643683	Cep112	centrosomal protein 112 [Source:MGI Symbol;Acc:MGI:1923673]
ENSMUSG00000020732	0.382098067	0.562229334	0.521585597	0.257521071	0.20072761	0.53356817	0.833869551	0.693958053	0.869345174	0.949382496	0.63611619	1.166373996	Rab37	RAB37, member RAS oncogene family [Source:MGI Symbol;Acc:MGI:1929945]
ENSMUSG00000020733	25.39239593	24.58170775	25.40155726	19.6699894	16.27499461	13.02448093	71.34187185	74.99934865	67.74027915	59.58137132	61.4375703	63.19167063	Slc9a3r1	solute carrier family 9 (sodium/hydrogen exchanger), member 3 regulator 1 [Source:MGI Symbol;Acc:MGI:1349482]
ENSMUSG00000020734	0	0.032406485	0.019207434	0.078783802	0.088701736	0.042869877	0.301490025	0.223026018	0.261930357	0.067125296	0.023425033	0.149638606	Grin2c	glutamate receptor, ionotropic, NMDA2C (epsilon 3) [Source:MGI Symbol;Acc:MGI:95822]
ENSMUSG00000020736	110.4889486	87.65316698	135.1110744	105.0213914	83.54443331	69.69974284	65.6659279	69.75138715	53.18002087	73.93589963	69.92564363	76.834109	Nt5c	5',3'-nucleotidase, cytosolic [Source:MGI Symbol;Acc:MGI:1354954]
ENSMUSG00000020737	333.3598825	259.17717	350.5446456	294.9417831	257.1514397	214.1292328	223.8061279	235.7055573	207.9356076	224.4800719	232.3019772	233.2200588	Jpt1	Jupiter microtubule associated homolog 1 [Source:MGI Symbol;Acc:MGI:1096361]
ENSMUSG00000020738	130.9717834	146.9460161	109.1911225	158.3778764	170.1739661	153.4631867	278.1890854	264.8964114	254.3405831	311.369472	289.5480348	284.2799008	Sumo2	small ubiquitin-like modifier 2 [Source:MGI Symbol;Acc:MGI:2158813]
ENSMUSG00000020739	107.1180075	93.38886402	95.96512497	126.5307238	127.2219219	115.0136635	362.4896081	364.4061007	349.4747522	342.385515	367.4447486	371.4532389	Nup85	nucleoporin 85 [Source:MGI Symbol;Acc:MGI:3046173]
ENSMUSG00000020740	19.02312093	24.62377591	16.59586157	22.31243117	23.36806705	28.08942378	37.6699677	32.82544504	45.01924013	51.85747048	55.14409804	56.00572317	Gga3	golgi associated, gamma adaptin ear containing, ARF binding protein 3 [Source:MGI Symbol;Acc:MGI:2384159]
ENSMUSG00000020741	24.87469053	27.24968212	25.16407289	25.99706804	27.93510918	31.26639319	50.17800197	45.84510156	55.02118242	81.15107774	82.67513585	78.30794228	Cluh	clustered mitochondria (cluA/CLU1) homolog [Source:MGI Symbol;Acc:MGI:1921398]
ENSMUSG00000020743	40.80594197	34.2444483	38.86427858	41.93715505	33.34580506	32.84173556	68.89275482	70.85226131	66.11419848	50.57509631	52.87429242	53.06303989	Mif4gd	MIF4G domain containing [Source:MGI Symbol;Acc:MGI:1916924]
ENSMUSG00000020744	18.47903938	19.63476656	21.24927006	21.74285833	18.05518938	17.98491381	32.87578279	31.92516809	31.24166177	29.70440558	28.53460619	25.27689429	Slc25a19	solute carrier family 25 (mitochondrial thiamine pyrophosphate carrier), member 19 [Source:MGI Symbol;Acc:MGI:1914533]
ENSMUSG00000020745	33.04773266	41.41876925	30.23550314	39.5767672	47.43221049	56.0218044	32.04916102	28.55243048	33.83685944	54.85744435	55.75086363	55.22082751	Pafah1b1	platelet-activating factor acetylhydrolase, isoform 1b, subunit 1 [Source:MGI Symbol;Acc:MGI:109520]
ENSMUSG00000020747	24.77920038	25.42527437	25.34915866	28.07348183	26.98013124	28.35455991	23.26774205	21.96739281	26.93113838	25.82993026	27.75130405	26.84746561	Tmem94	transmembrane protein 94 [Source:MGI Symbol;Acc:MGI:1919797]
ENSMUSG00000020752	13.04545517	11.93692876	11.93415505	12.50233287	13.17206003	11.84333928	12.20544682	12.38087122	13.72593648	16.25241853	16.94789426	17.19447339	Recql5	RecQ protein-like 5 [Source:MGI Symbol;Acc:MGI:2156841]
ENSMUSG00000020755	38.83321026	40.13510766	41.76703972	44.52016855	42.38279845	38.12028275	40.76776388	40.46791416	39.32326065	61.26803147	61.04538714	64.03944681	Sap30bp	SAP30 binding protein [Source:MGI Symbol;Acc:MGI:1927479]
ENSMUSG00000020758	1.467720198	1.653677086	1.362106386	1.665260799	1.653015812	1.407473279	3.949895425	3.525131454	4.108109285	3.891298686	3.955237783	3.630813992	Itgb4	integrin beta 4 [Source:MGI Symbol;Acc:MGI:96613]
ENSMUSG00000020766	80.5483499	74.3242744	98.94523906	82.36317929	67.2015543	58.64824746	124.3428171	127.9381773	105.0222206	87.3233764	87.73351291	88.4162655	Galk1	galactokinase 1 [Source:MGI Symbol;Acc:MGI:95730]
ENSMUSG00000020770	22.56692863	22.7543902	18.25406698	26.44153586	26.88996305	30.11802202	28.54681816	26.136032	30.90439113	29.69968455	32.82300821	30.45579085	Unk	unkempt family zinc finger [Source:MGI Symbol;Acc:MGI:2442456]
ENSMUSG00000020773	53.26152209	50.53150437	54.16498806	58.48945904	49.57609147	42.43572614	2.854943738	3.695827749	2.811046969	5.434716969	6.063137468	6.581153658	Trim47	tripartite motif-containing 47 [Source:MGI Symbol;Acc:MGI:1917374]
ENSMUSG00000020774	7.85110384	8.721481473	7.782280177	8.983402198	8.59861632	9.656819749	0.049535595	0.082448448	0.025821503	0.397038983	0.461855261	0.393376193	Aspa	aspartoacylase [Source:MGI Symbol;Acc:MGI:87914]
ENSMUSG00000020775	115.4507188	100.9194497	135.3936553	117.9249096	108.1677789	89.21607684	134.8709802	141.7762122	116.6989054	126.2458323	122.7889669	130.7208788	Mrpl38	mitochondrial ribosomal protein L38 [Source:MGI Symbol;Acc:MGI:1926269]
ENSMUSG00000020776	33.90542157	32.79045504	35.09623972	42.45691277	42.88462323	43.51319032	19.23387141	19.20999653	20.89760683	28.36699128	29.17620651	25.97027561	Fbf1	Fas (TNFRSF6) binding factor 1 [Source:MGI Symbol;Acc:MGI:1922033]
ENSMUSG00000020777	36.60055863	48.15403947	35.25978067	40.67793142	44.74700898	46.16454907	16.25967497	14.94514415	18.17741081	22.57437048	22.19184366	21.72797345	Acox1	acyl-Coenzyme A oxidase 1, palmitoyl [Source:MGI Symbol;Acc:MGI:1330812]
ENSMUSG00000020778	11.80234838	8.977088357	15.60555422	11.90786337	9.837842556	7.72909596	9.331543499	9.750446944	6.445156976	6.856155953	5.582792332	7.21746273	Ten1	TEN1 telomerase capping complex subunit [Source:MGI Symbol;Acc:MGI:1916785]
ENSMUSG00000020780	80.7155082	77.70205209	83.82780979	88.35895282	81.51887218	73.31991779	72.93727465	76.72652586	76.44395076	78.8339725	78.47410914	79.76554015	Srp68	signal recognition particle 68 [Source:MGI Symbol;Acc:MGI:1917447]
ENSMUSG00000020781	25.70102356	25.44305571	26.0578821	28.47105943	20.86202745	21.15471467	48.70322539	53.83669204	49.61773493	47.86894469	47.54880001	48.89681709	Tsen54	tRNA splicing endonuclease subunit 54 [Source:MGI Symbol;Acc:MGI:1923515]
ENSMUSG00000020782	13.11054014	10.22589649	10.84723256	10.45626418	9.770442584	11.14723111	140.0151679	132.7102344	145.6526272	95.07789035	94.66568329	98.05121551	Llgl2	LLGL2 scribble cell polarity complex component [Source:MGI Symbol;Acc:MGI:1918843]
ENSMUSG00000020783	6.525074142	7.448405772	6.206799598	8.233886641	8.30600987	9.154179073	15.10039824	14.07703289	16.06072308	17.28680613	17.39016808	16.47149778	Ncbp3	nuclear cap binding subunit 3 [Source:MGI Symbol;Acc:MGI:1914124]
ENSMUSG00000020785	5.941502543	7.97013876	6.827047221	8.419991129	8.434016056	8.425198107	3.258843662	2.942952394	3.765179137	7.448827866	8.286680702	7.576166521	Camkk1	calcium/calmodulin-dependent protein kinase kinase 1, alpha [Source:MGI Symbol;Acc:MGI:1891766]
ENSMUSG00000020787	0	0.082445118	0	0	0	0	0.596570113	0.047283248	0.266550093	0.298852762	0.139056089	0.444143649	P2rx1	purinergic receptor P2X, ligand-gated ion channel, 1 [Source:MGI Symbol;Acc:MGI:1098235]
ENSMUSG00000020788	0.731219541	0.130660897	0.219422283	0.18823781	0.079475508	0.307286452	34.77934342	31.46044839	37.8900411	10.82579775	10.57820407	11.56388994	Atp2a3	ATPase, Ca++ transporting, ubiquitous [Source:MGI Symbol;Acc:MGI:1194503]
ENSMUSG00000020790	26.49911349	32.23719144	25.13460405	30.61729116	35.60244344	47.29710561	19.70303732	18.63057061	24.53775029	54.89450075	55.76229192	56.39613629	Ankfy1	ankyrin repeat and FYVE domain containing 1 [Source:MGI Symbol;Acc:MGI:1337008]
ENSMUSG00000020792	39.94247966	42.44212155	44.28342819	49.16752388	47.58076654	42.27179805	15.56753561	16.05615021	15.69272876	22.4419629	25.04048612	22.30163464	Exoc7	exocyst complex component 7 [Source:MGI Symbol;Acc:MGI:1859270]
ENSMUSG00000020793	0.795039249	1.165218173	0.690628782	0.809364615	0.885941289	0.899175258	0.645266306	0.859199618	0.583022363	1.142140099	0.942549355	0.896742439	Galr2	galanin receptor 2 [Source:MGI Symbol;Acc:MGI:1337018]
ENSMUSG00000020794	21.66831728	25.82316879	16.36185465	25.81481729	28.13746897	30.70430709	28.16088912	25.5449823	32.46171461	43.63723531	42.730745	43.49534441	Ube2g1	ubiquitin-conjugating enzyme E2G 1 [Source:MGI Symbol;Acc:MGI:1914378]
ENSMUSG00000020798	0.179693761	0.263361131	0.11707128	0.106710078	0.25745056	0.099541445	0.277100314	0.178012272	0.076023446	0.292239684	0.326349663	0.318498065	Spns3	spinster homolog 3 [Source:MGI Symbol;Acc:MGI:1924827]
ENSMUSG00000020799	0.334260852	0.069985192	0.4044345	0.255212783	0.383121342	0.277745938	5.83276386	5.388436962	5.600093221	9.948160799	9.510695749	8.079005231	Tekt1	tektin 1 [Source:MGI Symbol;Acc:MGI:1333819]
ENSMUSG00000020801	17.14248064	15.77251849	19.31419979	15.95688759	16.1102716	12.95869685	18.14996108	21.67560624	17.55908745	16.7590244	18.57171321	19.73344617	Med31	mediator complex subunit 31 [Source:MGI Symbol;Acc:MGI:1914529]
ENSMUSG00000020802	26.66869819	30.21036548	22.62840196	26.45528618	28.53480771	30.98169138	29.9240709	27.21005447	31.86202029	45.84227505	47.66256086	45.51867236	Ube2o	ubiquitin-conjugating enzyme E2O [Source:MGI Symbol;Acc:MGI:2444266]
ENSMUSG00000020803	204.3853172	161.3535509	232.3473812	189.7509222	170.4585439	135.0877902	137.8009765	157.2784483	116.1938845	149.9550382	133.7163351	135.6162533	Txndc17	thioredoxin domain containing 17 [Source:MGI Symbol;Acc:MGI:1289248]
ENSMUSG00000020804	0.131451592	0.050457755	0.134579176	0	0.138110952	0.066749533	0.156475984	0	0.04078325	0.117580345	0.21884038	0.116495636	Aanat	arylalkylamine N-acetyltransferase [Source:MGI Symbol;Acc:MGI:1328365]
ENSMUSG00000020805	0.163063905	0	0.146075676	0.038042139	0	0.015525353	2.620435332	2.94806905	3.092378824	4.612721692	4.32652925	4.479848585	Slc13a5	solute carrier family 13 (sodium-dependent citrate transporter), member 5 [Source:MGI Symbol;Acc:MGI:3037150]
ENSMUSG00000020806	11.09328202	12.5717149	12.34052991	5.332883775	4.995115532	5.413565909	27.65355008	25.08708745	30.29015069	26.54638752	28.9146651	28.07478362	Rhbdf2	rhomboid 5 homolog 2 [Source:MGI Symbol;Acc:MGI:2442473]
ENSMUSG00000020807	0	0.023121566	0.205563463	0.037474081	0.042191618	0.061174094	0.017925737	0.039781472	0.018688358	0.143678829	0.083567138	0.035588338	4933427D14	RIKEN cDNA 4933427D14 gene [Source:MGI Symbol;Acc:MGI:1921727]
ENSMUSG00000020808	60.73970883	59.80976886	58.79195513	84.10936834	82.25402366	67.61710823	41.03404867	40.09628351	39.4423462	34.98906034	40.56532488	36.1588528	Pimreg	PICALM interacting mitotic regulator [Source:MGI Symbol;Acc:MGI:1924434]

ENSMUSG00000020810	0.147673918	0.510162323	0.415765553	0.017225859	0.252127044	0.12654062	0.049439967	0.054859521	0.05154331	0.247670311	0.199750914	0.13087226	Cygb	cytoglobin [Source:MGI Symbol;Acc:MGI:2149481]
ENSMUSG00000020811	0.752168939	0.866161199	0.543575324	0.701911695	0.69730054	0.657165452	4.977143047	4.470782998	4.90061597	3.443139408	3.056864437	2.882024159	Wscd1	WSC domain containing 1 [Source:MGI Symbol;Acc:MGI:2448493]
ENSMUSG00000020812	8.170485761	7.801211906	6.809603561	9.616911199	7.290272711	7.442955068	27.34492475	24.52613184	22.16460676	18.14222842	17.84008492	19.57586138	Snhg16	small nucleolar RNA host gene 16 [Source:MGI Symbol;Acc:MGI:1913543]
ENSMUSG00000020814	111.7598334	102.8262072	120.6563844	112.465272	107.9798683	94.23256022	27.80827489	27.46160808	23.03712256	24.6595466	25.99100698	29.39270795	Mxra7	matrix-remodelling associated 7 [Source:MGI Symbol;Acc:MGI:1914872]
ENSMUSG00000020817	24.19763976	24.98386018	20.72480042	25.53053264	27.59982674	30.00784822	21.49502619	20.71997286	23.63638577	36.50281024	34.74203333	34.67602993	Rabep1	rabaptin, RAB GTPase binding effector protein 1 [Source:MGI Symbol;Acc:MGI:1860236]
ENSMUSG00000020818	48.68186579	45.78206462	51.63545385	44.54984402	42.73341041	37.45887152	11.00571549	10.16382743	10.42642144	18.75234858	18.32237973	17.55876451	Mfsd11	major facilitator superfamily domain containing 11 [Source:MGI Symbol;Acc:MGI:1917150]
ENSMUSG00000020821	61.51035256	67.68617733	61.66622246	64.21241246	64.95529758	62.19783669	19.17344496	19.44013498	20.29618884	28.88678113	30.21938855	29.10754254	Kif1c	kinesin family member 1C [Source:MGI Symbol;Acc:MGI:1098260]
ENSMUSG00000020823	58.41696687	58.48006826	56.73801362	57.37605095	58.50165133	60.26490631	91.14387005	84.16403708	91.27443322	63.60101369	63.11835772	64.24985156	Sec14l1	SEC14-like lipid binding 1 [Source:MGI Symbol;Acc:MGI:1921386]
ENSMUSG00000020826	80.14347642	85.33627248	71.85376072	46.67387984	49.70462488	48.90618776	2.667505238	2.713254682	3.432784416	10.2448981	9.922505106	10.2469252	Nos2	nitric oxide synthase 2, inducible [Source:MGI Symbol;Acc:MGI:97361]
ENSMUSG00000020827	21.36788851	26.94589505	19.67279361	24.21420212	29.70470537	32.27565618	25.50229808	20.46859433	27.21226862	41.39882097	42.73946486	42.52757318	Mink1	misshapen-like kinase 1 (zebrafish) [Source:MGI Symbol;Acc:MGI:1355329]
ENSMUSG00000020828	21.28140578	23.88994503	22.95579921	24.79173063	24.04749834	24.22581828	6.21539035	5.674219986	6.349784305	9.517857485	9.613187358	9.88401588	Pld2	phospholipase D2 [Source:MGI Symbol;Acc:MGI:892877]
ENSMUSG00000020829	10.72881374	9.094943986	11.12898952	8.215612899	8.357590432	5.088597941	13.29356134	12.03058465	10.98716854	13.24289896	13.24281843	12.06705734	Slc46a1	solute carrier family 46, member 1 [Source:MGI Symbol;Acc:MGI:1098733]
ENSMUSG00000020830	0.256121951	0	0	0	0	0.048770864	0.171494884	0	0	0.286368797	0.213195687	0.283726969	Vmo1	vitelline membrane outer layer 1 homolog (chicken) [Source:MGI Symbol;Acc:MGI:2685587]
ENSMUSG00000020831	1.583870718	1.97590093	0.405388806	0.985361419	1.040068674	0.754003542	1.826473123	1.569049609	0.982801264	1.652857452	1.15361102	1.462151253	0610010K14	RIKEN cDNA 0610010K14 gene [Source:MGI Symbol;Acc:MGI:1915609]
ENSMUSG00000020832	13.91617549	15.61750556	12.90898218	16.49342222	16.93881295	16.88484354	25.62611151	22.95860531	25.70243015	29.28008581	29.58292563	26.91024603	Eral1	Era (G-protein)-like 1 (E. coli) [Source:MGI Symbol;Acc:MGI:1889295]
ENSMUSG00000020834	4.991557325	4.583875895	5.433757734	6.014177297	6.074245417	3.898231529	9.13833533	12.33082525	10.26222752	7.544997813	8.204746806	7.503390937	Dhrs13	dehydrogenase/reductase (SDR family) member 13 [Source:MGI Symbol;Acc:MGI:1917701]
ENSMUSG00000020836	0.87337932	0.60344471	0.625911539	1.276869728	1.468199784	1.197427006	2.703074982	3.085903724	3.278719441	2.838422418	3.344200823	3.276643366	Coro6	coronin 6 [Source:MGI Symbol;Acc:MGI:2183448]
ENSMUSG00000020838	0.144946783	0.071534431	0.063598051	0.135261956	0.065267062	0.047315718	0	0.04102584	0.019272931	0.074086557	0.24130715	0.055052317	Slc6a4	solute carrier family 6 (neurotransmitter transporter, serotonin), member 4 [Source:MGI Symbol;Acc:MGI:96285]
ENSMUSG00000020839	0.217096306	0.349996579	0.133356963	0.243108847	0.228094442	0.165358328	0	0.043012964	0	0.038837504	0.07228431	0	Tmigd1	transmembrane and immunoglobulin domain containing 1 [Source:MGI Symbol;Acc:MGI:1913851]
ENSMUSG00000020840	47.32789708	40.1228292	40.98866445	43.97303328	41.66681939	39.24499034	130.7824648	133.9426118	131.3842669	145.6143444	148.3923786	148.1422197	Blmh	bleomycin hydrolase [Source:MGI Symbol;Acc:MGI:1345186]
ENSMUSG00000020841	36.51837167	46.19883117	32.66305846	32.70698246	39.32567856	49.12745102	7.596379955	7.118865958	8.056335467	51.34357809	52.90573308	52.06808502	Cpd	carboxypeptidase D [Source:MGI Symbol;Acc:MGI:107265]
ENSMUSG00000020843	44.75276239	36.23703192	48.26489707	36.89877881	34.00917209	28.6250326	21.86637868	25.19505934	23.3072931	37.47143538	38.62220452	35.91022172	Timm22	translocase of inner mitochondrial membrane 22 [Source:MGI Symbol;Acc:MGI:1929742]
ENSMUSG00000020844	101.250516	92.56201089	106.6875172	70.51342449	57.66315993	53.44854142	62.13666529	67.83747713	62.75995086	60.63348139	65.16130007	59.33429238	Nxn	nucleoredoxin [Source:MGI Symbol;Acc:MGI:109331]
ENSMUSG00000020846	14.22439475	14.68708592	15.26525868	10.77009571	10.76143341	10.08790328	0.962558986	1.225857237	0.878070805	3.441122278	3.447068312	3.431092791	Rflnb	refilin B [Source:MGI Symbol;Acc:MGI:1923816]
ENSMUSG00000020847	0.601055294	0.570002645	0.361974118	0.296944372	0.40862079	0.107720692	1.736086256	1.96141945	2.20484236	1.739390428	2.207284849	3.039352305	Rph3al	rabphilin 3A-like (without C2 domains) [Source:MGI Symbol;Acc:MGI:1923492]
ENSMUSG00000020848	0.315732999	0.363582779	0.181825385	0.202562832	0.279895573	0.353216916	0.035234904	0.058645973	0.137752184	0.105905892	0.147833928	0.201113691	Doc2b	double C2, beta [Source:MGI Symbol;Acc:MGI:1100497]
ENSMUSG00000020849	772.003166	700.3138327	698.4827624	713.948423	720.2648726	678.2797314	1404.403069	1452.84981	1502.968635	1500.173586	1474.948808	1453.619857	Ywhae	tyrosine 3-monooxygenase/tryptophan 5-monooxygenase activation protein, epsilon polypeptide [Source:MGI Symbol;Acc:MGI:894
ENSMUSG00000020850	52.09049261	59.7527485	45.97531478	61.29903803	69.52608073	92.84344049	103.1540268	90.07161131	128.5564119	199.0457307	212.7351556	204.9643627	Prpf8	pre-mRNA processing factor 8 [Source:MGI Symbol;Acc:MGI:2179381]
ENSMUSG00000020857	0.690033024	0.964881748	0.756911515	1.195865319	1.449980428	0.788378503	1.100081732	0.683576023	0.917506335	2.160263863	1.559039253	1.790892431	Nme2	NME/NM23 nucleoside diphosphate kinase 2 [Source:MGI Symbol;Acc:MGI:97356]
ENSMUSG00000020859	26.6991171	35.14904467	21.70071605	34.76816606	42.23408688	59.9419588	6.375872594	5.795619643	8.218766321	30.3230446	29.54148109	29.29508467	Spag9	sperm associated antigen 9 [Source:MGI Symbol;Acc:MGI:1918084]
ENSMUSG00000020863	45.92572425	50.82669935	42.29108712	49.46840878	54.4830391	63.11714337	76.78929273	76.23770762	83.5112239	105.482538	99.61274101	98.53173479	Luc7l3	LUC7-like 3 (S. cerevisiae) [Source:MGI Symbol;Acc:MGI:1914934]
ENSMUSG00000020864	79.65260666	77.32533946	77.20497457	56.14242713	60.99627489	61.40905413	32.4001572	34.63350126	34.06242269	56.46720324	59.42555049	57.73990079	Ankrd40	ankyrin repeat domain 40 [Source:MGI Symbol;Acc:MGI:1918702]
ENSMUSG00000020865	2.625717779	3.641154779	2.631397842	0.983561769	0.971386299	0.732380349	0.330166344	0.183179406	0.36142337	3.787599613	4.017277396	4.752274327	Abcc3	ATP-binding cassette, sub-family C (CFTR/MRP), member 3 [Source:MGI Symbol;Acc:MGI:1923658]
ENSMUSG00000020866	7.871589409	8.841641325	7.65519776	2.970367237	3.103270103	3.483809591	0.569630459	0.603664954	0.527138116	4.508004059	4.517389993	4.885738705	Cacna1g	calcium channel, voltage-dependent, T type, alpha 1G subunit [Source:MGI Symbol;Acc:MGI:1201678]
ENSMUSG00000020867	0.092667239	0.071140726	0	0.028825182	0	0.047055306	0.0827312	0.061200068	0	0.110518209	0.205696342	0.383245273	Spata20	spermatogenesis associated 20 [Source:MGI Symbol;Acc:MGI:2183449]
ENSMUSG00000020868	39.93451947	41.21168016	43.33382951	37.47459496	36.863714	34.32890375	28.30568195	27.6167464	30.48960609	53.20982749	53.52618307	53.39737358	Xylt2	xylosyltransferase II [Source:MGI Symbol;Acc:MGI:2444797]
ENSMUSG00000020869	141.4092697	164.6106269	126.5279845	153.8310824	161.6436028	170.7017209	214.3078811	190.7662464	227.6968815	255.3292106	265.6692984	273.0621534	Lrrc59	leucine rich repeat containing 59 [Source:MGI Symbol;Acc:MGI:2138133]
ENSMUSG00000020870	0.034616643	0	0.106320797	0.032303683	0.109110986	0.052733742	0.123619804	0.171463587	0.128879007	0.06192754	0	0.398815576	Cdc34b	cell division cycle 34B [Source:MGI Symbol;Acc:MGI:1858429]
ENSMUSG00000020871	1.709594202	1.559608077	1.932116237	1.74039842	1.422965645	2.029351123	0.35679431	0.131968569	0.103325984	0.218455949	0.406589918	0.157411371	Dlx4	distal-less homeobox 4 [Source:MGI Symbol;Acc:MGI:94904]
ENSMUSG00000020872	0	0	0	0.160485523	0.108413233	0	0.12282927	0	0	0	0	0.091445815	Tac4	tachykinin 4 [Source:MGI Symbol;Acc:MGI:1931130]
ENSMUSG00000020873	79.79828652	68.15045678	83.91129155	73.4357723	62.27627169	59.41386629	63.84672003	67.47193005	69.04730722	82.821164	79.1035806	79.36538238	Slc35b1	solute carrier family 35, member B1 [Source:MGI Symbol;Acc:MGI:1343133]
ENSMUSG00000020875	23.62836037	21.76741727	23.44481854	17.80546457	18.03106339	15.00676667	2.109486206	2.745512899	2.364586568	10.0431027	9.597502967	9.635564686	Hoxb9	homeobox B9 [Source:MGI Symbol;Acc:MGI:96190]
ENSMUSG00000020876	21.13272687	23.57135786	22.92619035	20.80426916	20.91363995	21.55454094	20.88085746	17.7799411	19.29893578	27.71608372	31.03945107	30.10647217	Snx11	sorting nexin 11 [Source:MGI Symbol;Acc:MGI:1921729]
ENSMUSG00000020877	12.96957966	10.63030388	16.28527334	13.17091028	10.42035707	8.135395194	20.97832456	24.18487813	18.85292505	16.95790717	17.5591597	18.49175431	Scrn2	secernin 2 [Source:MGI Symbol;Acc:MGI:1343092]
ENSMUSG00000020878	0.869587703	0.519231451	0.923250753	1.021869945	0.541416986	0.490629051	1.380174398	1.24431741	1.289007122	1.065909248	1.018743464	0.94190558	Lrrc46	leucine rich repeat containing 46 [Source:MGI Symbol;Acc:MGI:1916547]
ENSMUSG00000020882	10.74520931	10.48057307	10.61059183	11.78368951	11.26453115	10.74320056	13.27290639	12.9812936	12.32963912	13.40472361	14.04116713	13.9989567	Cacnb1	calcium channel, voltage-dependent, beta 1 subunit [Source:MGI Symbol;Acc:MGI:102522]
ENSMUSG00000020883	28.10463361	31.82265205	26.9486683	30.6314196	29.98111963	39.27810484	19.25113989	17.40345261	19.6508809	37.92737697	38.36008557	39.39995159	Fbxl20	F-box and leucine-rich repeat protein 20 [Source:MGI Symbol;Acc:MGI:1919444]
ENSMUSG00000020884	0.856675776	0.807141431	1.195988693	1.271829615	0.368212548	1.067750899	0.347645738	0.077150856	0.108730728	0.59212256	0.291721158	0.379603576	Asgr1	asialoglycoprotein receptor 1 [Source:MGI Symbol;Acc:MGI:88081]
ENSMUSG00000020886	47.33836358	49.4955695	43.83642535	48.94046645	46.9063989	53.6658977	7.800076521	8.214235213	8.415336139	27.09033235	29.32421378	27.98212059	Dlg4	discs large MAGUK scaffold protein 4 [Source:MGI Symbol;Acc:MGI:1277959]
ENSMUSG00000020887	0	0	0.057409535	0.017442866	0.019638713	0.085423164	0	0.092584378	0.034795093	0	0.03111805	0.049695358	A230052G05	RIKEN cDNA A230052G05 gene [Source:MGI Symbol;Acc:MGI:3045239]
ENSMUSG00000020888	24.46822612	24.97967723	22.91882992	30.00716892	26.79219197	27.08126271	52.17302761	44.26530338	48.33061379	41.1939028	43.59349236	43.85757152	Dvl2	dishevelled segment polarity protein 2 [Source:MGI Symbol;Acc:MGI:106613]
ENSMUSG00000020889	26.04808515	27.64698844	25.15833821	24.92680206	25.97348273	24.23892446	6.406132147	6.718865771	7.456324041	13.16635743	14.09356208	12.43511626	Nr1d1	nuclear receptor subfamily 1, group D, member 1 [Source:MGI Symbol;Acc:MGI:2444210]
ENSMUSG00000020890	0.070250592	0.141570045	0.107883062	0.065556699	0.156845186	0.173902967	0.054878363	0.060894068	0.049039777	0.133529679	0.10233393	0.070040029	Gucy2e	guanylate cyclase 2e [Source:MGI Symbol;Acc:MGI:105123]
ENSMUSG00000020891	0	0.093255048	0.055272585	0.201523101	0.255253993	0.143926063	0.024099658	0	0	0	0.044939618	0.023922776	Alox8	arachidonate 8-lipoxygenase [Source:MGI Symbol;Acc:MGI:1098228]
ENSMUSG00000020892	1.02506891	0.590209772	0.774599975	0.888250502	0.871856395	0.98526551	1.525263633	0.967120765	0.840509703	1.353518954	1.564321845	1.492439265	Aloxe3	arachidonate lipoxygenase 3 [Source:MGI Symbol;Acc:MGI:1345140]
ENSMUSG00000020893	5.927267199	8.110698811	6.538014078	8.51670308	9.341315348	9.520522421	11.81229086	10.95742252	11.99166237	10.62578438	11.10632796	10.54973732	Per1	period circadian clock 1 [Source:MGI Symbol;Acc:MGI:1098283]
ENSMUSG00000020894	44.94263743	47.16158716	45.56284636	47.37291685	45.16642	49.78370697	38.66406999	33.81624248	37.93348736	24.86201349	28.14894384	23.81981257	Vamp2	vesicle-associated membrane protein 2 [Source:MGI Symbol;Acc:MGI:1313277]
ENSMUSG00000020895	26.18815097	22.84372362	28.41844996	28.56690214	24.36600906	17.3925151	40.00750971	44.03964033	32.94258566	22.53109806	22.68996092	21.2481857	Tmem107	transmembrane protein 107 [Source:MGI Symbol;Acc:MGI:1914160]
ENSMUSG00000020897	43.79604742	40.65867914	39.70711744	57.80752528	57.54217225	55.98751899	107.4648489	108.3313779	119.7692359	93.77655605	93.84547542	92.79140186	Aurkb	aurora kinase B [Source:MGI Symbol;Acc:MGI:107168]
ENSMUSG00000020898	35.59309474	31.71574313	34.54813487	37.50531902	37.48869753	35.38789958	91.83428305	92.22524922	102.1294314	70.73836355	73.44656828	76.36328613	Ctc1	CTS telomere maintenance complex component 1 [Source:MGI Symbol;Acc:MGI:1916214]
ENSMUSG00000020899	9.88191233	12.40570343	9.090664057	14.38931953	15.61559517	20.30195435	45.04937214	40.71840407	51.69065785	81.60189024	86.24396311	83.51444585	Pfas	phosphoribosylformylglycinamidine synthase (FGAR amidotransferase) [Source:MGI Symbol;Acc:MGI:2684864]
ENSMUSG00000020900	36.29480889	49.69430317	28.39081709	55.11977412	62.80727085	85.70193578	31.36797337	27.12553945	36.94144139	75.97624531	82.50673101	79.98005334	Myh10	myosin, heavy polypeptide 10, non-muscle [Source:MGI Symbol;Acc:MGI:1930780]
ENSMUSG00000020901	4.478646107	3.928293311	3.363912102	5.663671485	6.090809427	6.137158534	1.251845925	1.098818058	1.266145355	4.39915958	4.35516482	5.323027065	Pik3r5	phosphoinositide-3-kinase regulatory subunit 5 [Source:MGI Symbol;Acc:MGI:2443588]
ENSMUSG00000020902	31.69816678	40.53943644	28.6839622	42.88794407	47.34720653	55.69625681	12.34599253	10.64373019	14.10572266	27.13930436	28.95304414	27.97324191	Ntn1	netrin 1 [Source:MGI Symbol;Acc:MGI:105088]
ENSMUSG00000020903	25.3156841	20.36582371	34.37135671	23.62418044	18.83918523	17.85032156	15.54597324	15.8463411	12.75789944	16.13618349	16.26390103	16.27712334	Stx8	syntaxin 8 [Source:MGI Symbol;Acc:MGI:1890156]
ENSMUSG00000020904	0.221605946	0.042531773	0.075626181	0.137866018	0.155221694	0.196925446	0.857327215	1.097661314	0.790669944	1.123253954	0.953067073	1.276552174	Cfap52	cilia and flagella associated protein 52 [Source:MGI Symbol;Acc:MGI:1919110]
ENSMUSG00000020905	0.30866921	0.266586347	0.284411945	0.244838279	0.324306444	0.129309264	2.769507055	2.537980889	2.944790553	2.968046187	3.417248521	3.255247961	Usp43	ubiquitin specific peptidase 43 [Source:MGI Symbol;Acc:MGI:2444541]

ENSMUSG00000020907	0.455109006	0.142931295	0.084715866	0.270263793	0.347756295	0.315134814	0	0	0.077017599	0	0	0	Rcvrn	recoverin [Source:MGI Symbol;Acc:MGI:97883]
ENSMUSG00000020908	0.37254102	0.321750101	0.337396522	0.300850234	0.331196471	0.240102715	0.940223701	0.823276446	0.893537513	2.03141614	2.272099493	2.247593783	Myh3	myosin, heavy polypeptide 3, skeletal muscle, embryonic [Source:MGI Symbol;Acc:MGI:1339709]
ENSMUSG00000020910	24.67180708	22.92589529	27.61484341	26.26956386	23.27891019	20.99330924	38.51041341	37.69524591	35.31697566	38.96667242	39.51438874	43.63466706	Adprm	ADP-ribose/CDP-alcohol diphosphatase, manganese dependent [Source:MGI Symbol;Acc:MGI:1913608]
ENSMUSG00000020911	1.971773882	0.958697349	2.826162691	0.613342329	0.644517777	1.101367292	25.4664664	29.82073052	22.10452151	25.00542011	25.02075008	26.17268611	Krt19	keratin 19 [Source:MGI Symbol;Acc:MGI:96693]
ENSMUSG00000020912	0.092934099	0	0.071358937	0.021681144	0	0	0	0.046032233	0.021624812	0.062345518	0	0.061770364	Krt12	keratin 12 [Source:MGI Symbol;Acc:MGI:96687]
ENSMUSG00000020913	0.20966778	0.05365405	0.095402816	0.108699182	0.024476621	0.053233385	0	0	0	0	0	0	Krt24	keratin 24 [Source:MGI Symbol;Acc:MGI:1922956]
ENSMUSG00000020914	150.7011897	171.1737099	132.468441	220.5600233	246.4066597	276.1056644	383.9446883	363.9533612	435.7470478	678.1915537	667.8073328	662.0870545	Top2a	topoisomerase (DNA) II alpha [Source:MGI Symbol;Acc:MGI:98790]
ENSMUSG00000020916	0	0	0	0	0	0	0	0	0	0.046065789	0.064303188	0	Krt36	keratin 36 [Source:MGI Symbol;Acc:MGI:109364]
ENSMUSG00000020917	62.47526978	82.19439163	52.99949699	81.94140273	93.85042	108.3894489	164.7018733	146.6415021	191.8426625	216.9409838	222.7620275	219.0521747	Acly	ATP citrate lyase [Source:MGI Symbol;Acc:MGI:103251]
ENSMUSG00000020918	27.65517569	27.3359974	26.07904669	27.68710224	27.68936013	26.55164481	58.62136704	57.0654229	62.78186237	51.00030915	51.86227678	54.27368554	Kat2a	K(lysine) acetyltransferase 2A [Source:MGI Symbol;Acc:MGI:1343101]
ENSMUSG00000020919	7.685753893	9.13429849	5.766315815	7.697816994	8.494281795	10.47481652	5.751228493	4.963522489	6.46552978	7.221085591	6.456218896	7.466437297	Stat5b	signal transducer and activator of transcription 5B [Source:MGI Symbol;Acc:MGI:103035]
ENSMUSG00000020921	30.6737735	29.21731614	34.35978591	31.00961655	29.04472818	24.95007952	38.24581181	41.87555775	37.09723386	33.83045669	34.35898286	33.18275865	Tmem101	transmembrane protein 101 [Source:MGI Symbol;Acc:MGI:1923797]
ENSMUSG00000020922	80.50491271	70.37154399	91.86833694	81.04211089	75.9909658	62.97640931	92.33631445	104.2419379	91.90005825	92.0087217	86.52882472	92.45650213	Lsm12	LSM12 homolog [Source:MGI Symbol;Acc:MGI:1919592]
ENSMUSG00000020923	77.86421943	67.94275233	76.52070332	78.9042814	75.05795657	69.44821264	123.3109442	128.4338881	127.434709	114.3410256	111.9328919	112.6421119	Ubtf	upstream binding transcription factor, RNA polymerase I [Source:MGI Symbol;Acc:MGI:98512]
ENSMUSG00000020925	24.80866085	25.0062976	21.66192605	29.35735942	30.51805243	31.07759398	48.96444016	52.73837807	54.47375606	78.55308162	77.22128653	78.54117321	Ccdc43	coiled-coil domain containing 43 [Source:MGI Symbol;Acc:MGI:1289318]
ENSMUSG00000020926	1.693819838	1.415700446	2.069756517	1.937564645	2.095369676	1.519049845	2.731722956	2.246314514	2.568231304	3.812141431	3.433871871	3.534859736	Adam11	a disintegrin and metallopeptidase domain 11 [Source:MGI Symbol;Acc:MGI:1098667]
ENSMUSG00000020928	0	0	0	0	0	0.111666169	0	0	0.204680502	0.458970638	0.122033647	0	Higd1b	HIG1 domain family, member 1B [Source:MGI Symbol;Acc:MGI:1922939]
ENSMUSG00000020929	101.1789928	104.6156105	99.59767634	111.1424568	110.0341967	114.4782693	221.9610784	211.3567351	232.7202205	315.8284833	316.9709403	312.3010725	Eftud2	elongation factor Tu GTP binding domain containing 2 [Source:MGI Symbol;Acc:MGI:1336880]
ENSMUSG00000020930	2.353793197	1.189981696	1.440000173	2.089373763	1.749221124	1.464882844	0.205015899	0.625596164	0.293889704	1.104057741	1.027435387	0.71228908	Ccdc103	coiled-coil domain containing 103 [Source:MGI Symbol;Acc:MGI:1920543]
ENSMUSG00000020932	0.299448791	0.15915262	0.14149545	0.257945254	0.04840291	0.140359831	0.20564697	0.365103621	0.343033418	0.844757292	0.747782732	0.408275211	Gfap	glial fibrillary acidic protein [Source:MGI Symbol;Acc:MGI:95697]
ENSMUSG00000020935	131.2955221	99.15096994	129.6170506	90.99771392	81.22333087	74.61758158	231.0931316	236.4528155	228.4145005	195.7480218	206.0408937	213.8080788	Dcakd	dephospho-CoA kinase domain containing [Source:MGI Symbol;Acc:MGI:1915337]
ENSMUSG00000020936	89.76595841	90.11247236	87.2704269	85.08856065	88.0064274	78.17101982	101.7696956	102.7098154	105.9596386	130.0383159	136.2183411	136.3246593	Nmt1	N-myristoyltransferase 1 [Source:MGI Symbol;Acc:MGI:102579]
ENSMUSG00000020937	26.8100578	28.73871906	27.92234936	32.80230266	32.54998921	30.3033637	34.9575172	34.98598423	38.07750099	20.36901605	21.87584548	22.7624108	Plcd3	phospholipase C, delta 3 [Source:MGI Symbol;Acc:MGI:107451]
ENSMUSG00000020940	1.090583793	0.685016345	0.862774485	0.370078137	0.26041658	0.075516177	2.25709396	2.111649137	1.43032684	1.197206327	0.86653731	1.274025617	Efcab15	EF-hand calcium binding domain 15 [Source:MGI Symbol;Acc:MGI:1916691]
ENSMUSG00000020941	7.628319172	7.915111914	9.416524396	8.768511088	8.119130119	8.503693259	1.613920462	1.298848545	1.848991298	2.931908796	4.167060305	2.904861161	Map3k14	mitogen-activated protein kinase kinase kinase 14 [Source:MGI Symbol;Acc:MGI:1858204]
ENSMUSG00000020945	0	0	0.119382792	0	0	0	0	0	0	0	0	0	Lyzl6	lysozyme-like 6 [Source:MGI Symbol;Acc:MGI:1916694]
ENSMUSG00000020946	75.75681525	81.37839506	73.41198756	71.22517021	81.0479783	84.00459365	35.57012607	31.83480359	38.07198331	65.48698756	66.63748188	65.05797516	Gosr2	golgi SNAP receptor complex member 2 [Source:MGI Symbol;Acc:MGI:1927204]
ENSMUSG00000020948	3.841603612	6.1409385	2.898004125	5.837289729	7.793621988	9.634888299	3.711743609	2.804167367	4.163700335	6.940267651	6.942478066	8.152938213	Klhl28	kelch-like 28 [Source:MGI Symbol;Acc:MGI:1913939]
ENSMUSG00000020949	52.05412839	52.90574647	50.01999859	62.20425882	63.81188708	59.60431867	140.2016269	146.442916	134.5861509	87.09549273	83.37271233	84.98666304	Fkbp3	FK506 binding protein 3 [Source:MGI Symbol;Acc:MGI:1353460]
ENSMUSG00000020950	34.91983985	34.29418262	32.66723399	37.14638919	39.6098335	39.09461003	0.094015796	0.208643405	0.098015545	0.301423134	0.368161716	0.46662879	Foxg1	forkhead box G1 [Source:MGI Symbol;Acc:MGI:1347464]
ENSMUSG00000020952	19.42763098	23.3548966	16.96060184	19.95872723	20.57792301	20.38442944	17.01995371	16.47406017	19.15650084	21.87394709	20.38219696	20.41133069	Scfd1	Sec1 family domain containing 1 [Source:MGI Symbol;Acc:MGI:1924233]
ENSMUSG00000020953	0.721302826	0.734776827	0.312427306	0.077666301	0.262330667	0.274701656	2.57585095	2.2261081	3.150223395	9.603380391	7.620611804	8.408416121	Coch	cochlin [Source:MGI Symbol;Acc:MGI:1278313]
ENSMUSG00000020954	13.62997397	24.65785899	9.02108049	22.2176683	28.99646465	41.17629394	12.08394277	10.5525455	16.71759172	29.33511875	28.50477979	27.73302159	Strn3	striatin, calmodulin binding protein 3 [Source:MGI Symbol;Acc:MGI:2151064]
ENSMUSG00000020955	5.858789637	3.981657505	4.965711239	3.5403178	4.13736842	3.548080359	10.03245073	11.22734381	8.626658754	8.634019242	8.594451147	10.42696613	Ap4s1	adaptor-related protein complex AP-4, sigma 1 [Source:MGI Symbol;Acc:MGI:1337065]
ENSMUSG00000020956	15.57015192	15.02004333	14.80727202	17.0225594	16.25780129	14.2240716	15.61946248	17.73692992	17.33671324	14.78816158	12.84038992	14.41713846	Dtd2	D-tyrosyl-tRNA deacylase 2 [Source:MGI Symbol;Acc:MGI:1923485]
ENSMUSG00000020961	0.982793534	0.761350531	1.006176811	1.117227166	1.051356241	1.728530158	18.05881014	17.54244812	22.41954163	18.26899733	17.64070362	17.86292213	Ston2	stonin 2 [Source:MGI Symbol;Acc:MGI:1918272]
ENSMUSG00000020962	16.68665747	19.07742675	14.05988194	20.58040624	25.8490768	25.63682102	27.23840698	28.88755184	32.48586228	25.34573395	23.61168237	26.12170405	Gtf2a1	general transcription factor II A, 1 [Source:MGI Symbol;Acc:MGI:1933277]
ENSMUSG00000020963	0.050801048	0.146250046	0.156029221	0.14222011	0.026687319	0.154776957	0.068031028	0.075488514	0.070925298	0.47712355	0.444010853	0.517743098	Tshr	thyroid stimulating hormone receptor [Source:MGI Symbol;Acc:MGI:98849]
ENSMUSG00000020964	42.68686865	54.77090089	38.26552372	48.13780345	56.23712499	70.97686678	19.04800054	17.02880311	20.59546767	31.7654201	31.92626576	31.60998992	Sel1l	sel-1 suppressor of lin-12-like (C. elegans) [Source:MGI Symbol;Acc:MGI:1329016]
ENSMUSG00000020973	1.116963696	1.344706619	1.546376924	1.231847108	1.080198757	0.870106743	1.631781412	1.357991608	0.614323268	0.32924809	0.3592253	0.371205267	Dnaaf2	dynein, axonemal assembly factor 2 [Source:MGI Symbol;Acc:MGI:1923566]
ENSMUSG00000020974	8.558708577	9.62389188	5.223043705	10.1793138	14.59925413	16.20805701	36.22742478	33.14974271	42.17344349	22.78655731	23.2446493	25.40210656	Pole2	polymerase (DNA directed), epsilon 2 (p59 subunit) [Source:MGI Symbol;Acc:MGI:1197514]
ENSMUSG00000020978	34.95353813	34.79380084	29.16763526	35.78435492	35.84800574	37.39414689	25.75656736	26.85240626	27.01112378	21.70246562	21.66368724	20.67912144	Klhdc2	kelch domain containing 2 [Source:MGI Symbol;Acc:MGI:1916804]
ENSMUSG00000020982	38.90047263	44.93570632	34.82912106	46.36231132	46.4584355	54.3582656	47.69338542	44.02105039	52.75097046	58.57903234	54.29126836	56.36008882	Nemf	nuclear export mediator factor [Source:MGI Symbol;Acc:MGI:1918305]
ENSMUSG00000020986	46.77679285	63.71074354	39.74164815	48.3710452	62.55255982	74.66062696	36.03111611	32.96127603	45.78411355	72.91127281	68.27859019	68.57552192	Sec23a	SEC23 homolog A, COPII coat complex component [Source:MGI Symbol;Acc:MGI:1349635]
ENSMUSG00000020988	12.12155018	14.65304527	9.230289285	17.33324621	19.76873907	23.01301239	32.68089197	29.42288717	40.63852236	38.43402388	38.82880614	40.96548205	L2hgdh	L-2-hydroxyglutarate dehydrogenase [Source:MGI Symbol;Acc:MGI:2384968]
ENSMUSG00000020990	0.59928256	1.518230284	0.327221914	0.410109801	0.37778539	0.365170146	0	0.514518125	0	1.250769566	1.596295286	0.885164918	Cdkl1	cyclin-dependent kinase-like 1 (CDC2-related kinase) [Source:MGI Symbol;Acc:MGI:1918341]
ENSMUSG00000020992	0	0.07447322	0	0.040233946	0.203845123	0.0985191	0.019245919	0	0.04012941	0.11569529	0.287109222	0.11462797	4930512B01	RIKEN cDNA 4930512B01 gene [Source:MGI Symbol;Acc:MGI:1921974]
ENSMUSG00000020993	86.32030789	90.97218291	77.15883725	93.42932848	98.55081393	89.65720456	59.71464464	57.07794049	57.89234755	49.9825365	52.25668981	49.33473716	Trappc6b	trafficking protein particle complex 6B [Source:MGI Symbol;Acc:MGI:1925482]
ENSMUSG00000020994	60.97051177	72.92644992	52.00579994	70.4681669	82.78879577	94.359593	103.5405202	92.71866281	116.1662117	106.4881993	106.5460627	104.2968753	Pnn	pinin [Source:MGI Symbol;Acc:MGI:1100514]
ENSMUSG00000021000	24.41447313	30.16259852	20.89843715	25.70652433	28.94266694	31.28079377	31.37784464	31.27363744	34.14241898	40.56693121	39.43389203	40.71211478	Mia2	MIA SH3 domain ER export factor 2 [Source:MGI Symbol;Acc:MGI:2159614]
ENSMUSG00000021003	4.754363839	5.939743881	4.637884576	5.734217995	6.550336606	6.798502544	4.778498178	3.76197544	4.842635417	4.373024671	4.579781159	3.855689807	Galc	galactosylceramidase [Source:MGI Symbol;Acc:MGI:95636]
ENSMUSG00000021007	7.061834448	5.944820211	6.556729939	8.453363755	8.110377429	8.290881282	10.08743197	11.67567079	9.813968653	6.882963992	7.783633381	6.042565542	Spata7	spermatogenesis associated 7 [Source:MGI Symbol;Acc:MGI:2144877]
ENSMUSG00000021009	10.21573508	13.81043666	9.952723923	12.72917965	16.50796088	18.10818427	7.638839964	6.832261147	8.56631195	6.474316006	7.102635318	7.447181058	Ptpn21	protein tyrosine phosphatase, non-receptor type 21 [Source:MGI Symbol;Acc:MGI:1344406]
ENSMUSG00000021010	1.075712196	0.92111279	1.073067633	0.978097537	1.289596261	1.397097675	0.172374345	0.314229005	0.243889064	0.246717733	0.149236981	0.256663782	Npas3	neuronal PAS domain protein 3 [Source:MGI Symbol;Acc:MGI:1351610]
ENSMUSG00000021012	66.44178231	83.07169083	56.07580281	77.9518446	88.73642045	100.2253151	95.88097724	92.67780127	111.6352656	77.22179083	73.15212024	76.08865303	Zc3h14	zinc finger CCCH type containing 14 [Source:MGI Symbol;Acc:MGI:1919824]
ENSMUSG00000021013	19.97604726	29.04952537	12.43037756	26.7945038	31.37431435	39.58501825	7.104367674	5.607594836	7.521146663	10.67683338	12.70150398	9.5968416	Ttc8	tetratricopeptide repeat domain 8 [Source:MGI Symbol;Acc:MGI:1923510]
ENSMUSG00000021018	39.01827612	25.08678247	44.21869447	27.96583532	23.4582206	22.99134697	112.6803465	118.8584721	91.44724397	62.82166803	61.43902318	57.8236993	Polr2h	polymerase (RNA) II (DNA directed) polypeptide H [Source:MGI Symbol;Acc:MGI:2384309]
ENSMUSG00000021022	9.940003667	10.22872038	11.81025474	12.07475004	11.0697759	10.57320952	12.23851249	12.15160448	11.23809971	10.98919147	11.25078796	11.14339574	Ppp2r3c	protein phosphatase 2, regulatory subunit B'', gamma [Source:MGI Symbol;Acc:MGI:1930009]
ENSMUSG00000021023	7.144505015	6.670127103	6.83925648	7.401649526	6.9551259	6.856103754	15.04521724	14.21528035	14.52063514	14.78501147	16.34606934	15.48925678	Prorp	protein only RNase P catalytic subunit [Source:MGI Symbol;Acc:MGI:1913382]
ENSMUSG00000021024	293.4479593	240.9466642	316.6255483	267.4930215	239.7813748	194.3547621	335.3296637	347.4320013	275.9268748	259.5107733	259.3650946	242.9570729	Psma6	proteasome subunit alpha 6 [Source:MGI Symbol;Acc:MGI:1347006]
ENSMUSG00000021025	129.4251621	122.6133189	114.6131352	134.8311519	132.9283185	128.6236564	32.88850664	33.98850488	33.72560128	48.51509445	44.20556474	49.63967593	Nfkbia	nuclear factor of kappa light polypeptide gene enhancer in B cells inhibitor, alpha [Source:MGI Symbol;Acc:MGI:104741]
ENSMUSG00000021027	7.238805883	8.76741372	4.967615654	8.917882196	11.05598801	14.51281409	4.640070616	3.810240798	5.525920059	7.427657044	6.969637241	8.137000748	Ralgapa1	Ral GTPase activating protein, alpha subunit 1 [Source:MGI Symbol;Acc:MGI:1931050]
ENSMUSG00000021028	22.87713719	23.93398549	22.17562369	26.48130948	28.07620676	26.69377246	32.89597798	30.90856151	29.08546977	31.47828019	28.03764461	27.56439604	Mbip	MAP3K12 binding inhibitory protein 1 [Source:MGI Symbol;Acc:MGI:1918320]
ENSMUSG00000021032	1.09288612	0.143830234	0.415587266	0.524501162	0.426493569	0.49945895	0.334527031	0.402130639	0.465011919	0.782048664	0.493846614	0.636470833	Ngb	neuroglobin [Source:MGI Symbol;Acc:MGI:2151886]
ENSMUSG00000021033	19.25599204	16.03896955	17.55444791	17.9188996	18.24245153	15.94411815	25.56966088	25.96131139	25.65058793	14.5630143	16.5014266	14.45263452	Gstz1	glutathione transferase zeta 1 (maleylacetoacetate isomerase) [Source:MGI Symbol;Acc:MGI:1341859]
ENSMUSG00000021036	65.51355983	65.70002253	56.65382056	61.39502352	63.69600696	62.51096442	79.78588749	75.78882591	79.19469608	48.92406684	45.25452115	46.3371638	Sptlc2	serine palmitoyltransferase, long chain base subunit 2 [Source:MGI Symbol;Acc:MGI:108074]
ENSMUSG00000021037	83.61352967	68.55950985	97.34815123	76.19150226	66.93586193	52.31587089	237.0866894	244.1835264	205.6263223	111.1193434	108.9140583	104.1524765	Ahsa1	AHA1, activator of heat shock protein ATPase 1 [Source:MGI Symbol;Acc:MGI:2387603]

ENSMUSG00000021038	25.09800565	22.85246244	22.51612571	24.48861046	25.33922564	24.25597621	28.22230733	29.14192518	28.64068459	21.38452391	23.20420473	23.85634243	Vipas39	VPS33B interacting protein, apical-basolateral polarity regulator, spe-39 homolog [Source:MGI Symbol;Acc:MGI:2144805]
ENSMUSG00000021039	16.1315424	20.55980671	12.8188996	19.67489503	22.83037478	22.39179624	27.82785041	24.28262302	30.275802	26.62158413	24.66373758	24.14496852	Snw1	SNW domain containing 1 [Source:MGI Symbol;Acc:MGI:1913604]
ENSMUSG00000021040	0.122938537	0.047190015	0	0	0	0.031213353	0	0	0	0	0	0.18158526	Slirp	SRA stem-loop interacting RNA binding protein [Source:MGI Symbol;Acc:MGI:1916394]
ENSMUSG00000021044	6.677785841	6.355217908	6.685997785	6.815276058	6.648850582	5.478684083	7.489172064	7.836303767	9.040595128	4.590911765	4.379489691	4.630074789	Adck1	aarF domain containing kinase 1 [Source:MGI Symbol;Acc:MGI:1919363]
ENSMUSG00000021047	6.951736917	7.540143682	6.638308454	8.292566075	9.850231419	8.922176567	2.638174267	2.590404776	2.789985358	3.765127145	3.928530808	3.975317633	Nova1	NOVA alternative splicing regulator 1 [Source:MGI Symbol;Acc:MGI:104297]
ENSMUSG00000021048	31.97347634	32.93137038	27.04130435	34.006907	32.09790603	38.47165807	305.2798903	290.6754922	323.1658457	224.0887827	237.3281272	227.565442	Mthfd1	methylenetetrahydrofolate dehydrogenase (NADP+ dependent), methenyltetrahydrofolate cyclohydrolase, formyltetrahydrofolate sy
ENSMUSG00000021051	27.88215643	28.12978677	24.44540395	30.28780198	32.08653914	38.81704694	23.09635911	21.26591696	25.89856909	30.0501546	30.93491054	29.26830797	Ppp2r5e	protein phosphatase 2, regulatory subunit B', epsilon [Source:MGI Symbol;Acc:MGI:1349473]
ENSMUSG00000021054	19.4037111	22.58740948	19.77084436	22.89560661	24.73845747	25.73092178	7.725366116	7.422281267	9.257222639	8.247421365	7.773865064	9.585831344	Sgpp1	sphingosine-1-phosphate phosphatase 1 [Source:MGI Symbol;Acc:MGI:2135760]
ENSMUSG00000021055	0.053253079	0.015330914	0.040890084	0.074542347	0.097914056	0.111545244	0.118857841	0.118698215	0.210654572	0.369160569	0.52085184	0.283165134	Esr2	estrogen receptor 2 (beta) [Source:MGI Symbol;Acc:MGI:109392]
ENSMUSG00000021056	0.213920039	0.105574252	0.03128711	0	0.064216361	0.023277003	2.128095609	3.057677996	2.133299975	1.722117814	1.449972942	1.435400532	Tex21	testis expressed gene 21 [Source:MGI Symbol;Acc:MGI:1931131]
ENSMUSG00000021057	0.120393083	0.092425883	0.098606044	0.112348847	0.252984434	0.354578101	0.472930492	0.564528108	0.395934533	0.717925133	0.781677752	0.746867188	Akap5	A kinase (PRKA) anchor protein 5 [Source:MGI Symbol;Acc:MGI:2685104]
ENSMUSG00000021061	1.099986591	0.993023274	1.091400234	0.823726072	1.041567694	1.267103528	6.219605535	6.067305448	7.558590062	1.48194158	1.526046454	1.558532794	Sptb	spectrin beta, erythrocytic [Source:MGI Symbol;Acc:MGI:98387]
ENSMUSG00000021062	7.157702564	8.195091972	8.507222314	3.723601673	3.933037256	3.603262307	14.39638891	13.40717389	14.3196775	13.57749483	14.58698753	15.2385795	Rab15	RAB15, member RAS oncogene family [Source:MGI Symbol;Acc:MGI:1916865]
ENSMUSG00000021065	9.187633748	11.08991364	7.803448951	10.25603348	11.08193618	11.9243956	5.604813328	4.433338809	5.07789683	8.501017196	9.266843196	10.02022303	Fut8	fucosyltransferase 8 [Source:MGI Symbol;Acc:MGI:1858901]
ENSMUSG00000021066	3.456286885	5.06716561	2.587641024	6.052859716	6.846836608	8.373263588	0.869975464	1.086008859	1.275450685	2.056504926	2.332025863	1.862116365	Atl1	atlastin GTPase 1 [Source:MGI Symbol;Acc:MGI:1921241]
ENSMUSG00000021067	8.025373103	12.96365902	5.339666691	10.4539571	15.06943945	20.95411136	3.803775563	3.274713012	5.777470429	12.46793277	11.72494387	11.24619596	Sav1	salvador family WW domain containing 1 [Source:MGI Symbol;Acc:MGI:1927144]
ENSMUSG00000021068	8.106816547	9.30976924	6.042363511	9.768185602	11.24358021	17.25216304	1.650327582	1.588540908	2.010749326	8.914785171	9.269377712	8.783200097	Nin	ninein [Source:MGI Symbol;Acc:MGI:105108]
ENSMUSG00000021069	11.57901442	10.82851774	9.973627432	5.075580063	5.511351169	6.352268045	3.819904028	3.137070814	3.464926592	11.04907349	11.42920521	10.1544926	Pygl	liver glycogen phosphorylase [Source:MGI Symbol;Acc:MGI:97829]
ENSMUSG00000021070	2.155963261	2.260565982	2.579008319	2.287223421	2.157886503	2.359522055	1.114354944	1.056653398	1.53141534	6.110167657	5.875025461	6.003519014	Bdkrb2	bradykinin receptor, beta 2 [Source:MGI Symbol;Acc:MGI:102845]
ENSMUSG00000021071	0.108111778	0.093372203	0	0.189165254	0.05679437	0.092640134	0	0	0.025156502	0.072527575	0.101241168	0	Trim9	tripartite motif-containing 9 [Source:MGI Symbol;Acc:MGI:2137354]
ENSMUSG00000021072	49.74391656	60.5048276	44.97531035	63.79877006	74.73745114	75.06766287	41.66235257	40.62391121	49.62738068	48.19338564	48.03187926	51.99190112	Tmx1	thioredoxin-related transmembrane protein 1 [Source:MGI Symbol;Acc:MGI:1919986]
ENSMUSG00000021076	87.02714875	84.3338191	83.51404654	86.27736919	86.64238952	78.76949262	39.98328504	44.3402328	44.24686881	48.73735169	45.74781902	45.94073892	Actr10	ARP10 actin-related protein 10 [Source:MGI Symbol;Acc:MGI:1891654]
ENSMUSG00000021078	0.150712742	0.086776735	0	0.140642654	0	0	0.740040972	0.821163441	0.84166343	0.674044945	0.313632885	0.333913351	Tomm20l	translocase of outer mitochondrial membrane 20-like [Source:MGI Symbol;Acc:MGI:1922516]
ENSMUSG00000021079	23.26463039	21.22821679	22.04880906	21.71017082	20.62542227	23.01944601	38.37529191	40.7381943	32.41877765	24.33739583	26.92720814	23.13108225	Timm9	translocase of inner mitochondrial membrane 9 [Source:MGI Symbol;Acc:MGI:1353436]
ENSMUSG00000021081	0.022054581	0.330160791	0.045158637	0.020580973	0	0	0	0.04369641	0.041054999	0	0	0	Serpina1f	serine (or cysteine) peptidase inhibitor, clade A, member 1F [Source:MGI Symbol;Acc:MGI:1915598]
ENSMUSG00000021086	0.01641056	0.018897603	0	0.03062813	0	0.051725765	0.062498132	0.205113637	0.243854964	0.274936967	0.146788583	0.150261491	0.232695071	Ccdc175	coiled-coil domain containing 175 [Source:MGI Symbol;Acc:MGI:1921186]
ENSMUSG00000021087	0.564308037	0.454880799	0.259980493	0.157980896	0.177868794	0.343858577	2.922047916	3.018748558	2.915052996	4.946650104	4.438943912	4.675969296	Rtn1	reticulon 1 [Source:MGI Symbol;Acc:MGI:1933947]
ENSMUSG00000021090	0.213663232	0.378529531	0.185093488	0.21472448	0.690730609	0.613417499	0.601607722	1.139728548	0.917856796	2.146387735	2.188961068	2.068324089	Lrrc9	leucine rich repeat containing 9 [Source:MGI Symbol;Acc:MGI:1925507]
ENSMUSG00000021091	9.193586789	7.610075801	8.665316657	3.190489518	2.650293857	2.397711781	0.148894783	0.165216464	0.058210976	0.391592681	0.780891181	0.443405868	Serpina3n	serine (or cysteine) peptidase inhibitor, clade A, member 3N [Source:MGI Symbol;Acc:MGI:105045]
ENSMUSG00000021094	35.50562638	29.79533787	35.00841075	35.07446176	32.69369777	27.80365592	24.49900958	26.30946361	25.51475746	16.44022405	15.46529988	14.24301871	Dhrs7	dehydrogenase/reductase (SDR family) member 7 [Source:MGI Symbol;Acc:MGI:1913625]
ENSMUSG00000021095	0.135736555	0	0.104224567	0	0	0.077541058	0.090886879	0.134466397	0.063169009	3.88522058	4.773700281	4.300477422	Gsc	goosecoid homeobox [Source:MGI Symbol;Acc:MGI:95841]
ENSMUSG00000021096	64.57293424	64.57435705	58.83253255	70.91213166	73.8733962	74.688573	62.07135756	60.57777233	68.36654411	71.78897237	75.70651686	72.10812721	Ppm1a	protein phosphatase 1A, magnesium dependent, alpha isoform [Source:MGI Symbol;Acc:MGI:99878]
ENSMUSG00000021097	4.25014253	5.036307296	4.580882937	6.24749186	7.128233672	7.977830408	8.039386974	7.620883899	9.72786271	4.112605216	3.929868276	3.87590118	Clmn	calmin [Source:MGI Symbol;Acc:MGI:2136957]
ENSMUSG00000021098	9.94034533	10.32615108	8.184182185	12.03306969	11.94113094	13.1572603	35.49805578	34.98211734	40.66379924	38.45687755	39.14396811	50.48451598	4930447C04	RIKEN cDNA 4930447C04 gene [Source:MGI Symbol;Acc:MGI:1923051]
ENSMUSG00000021099	0	0	0	0	0	0.024525333	0.019164307	0.031897616	0.009989811	0	0	0.009511825	Six6	sine oculis-related homeobox 6 [Source:MGI Symbol;Acc:MGI:1341840]
ENSMUSG00000021101	0	0	0	0	0	0	0	0	0.04571821	0	0	0	4930408O17	RIKEN cDNA 4930408O17 gene [Source:MGI Symbol;Acc:MGI:1921178]
ENSMUSG00000021102	93.35437461	69.60367769	111.4998171	76.42726153	65.33823848	49.05030791	87.47166096	94.09367867	64.59278695	44.26116133	47.28795587	49.88196181	Glrx5	glutaredoxin 5 [Source:MGI Symbol;Acc:MGI:1920296]
ENSMUSG00000021103	18.25607717	15.40481736	15.53124206	18.8058556	17.55082551	17.62128834	25.51755932	28.28061049	25.54417036	20.23085412	19.38645784	20.45671449	Mnat1	menage a trois 1 [Source:MGI Symbol;Acc:MGI:106207]
ENSMUSG00000021108	0.833733945	0.810073825	0.826896613	0.75371348	0.547481906	0.644962562	0.337278464	0.477492087	0.339502344	0.827322317	0.737374367	0.738875537	Prkch	protein kinase C, eta [Source:MGI Symbol;Acc:MGI:97600]
ENSMUSG00000021109	211.0342813	267.0408308	173.557454	257.9390077	297.4666061	326.842288	29.85164252	27.90409374	34.21384551	88.7348133	83.3496903	89.10341241	Hif1a	hypoxia inducible factor 1, alpha subunit [Source:MGI Symbol;Acc:MGI:106918]
ENSMUSG00000021111	89.07335257	110.4992793	73.40282073	103.1143235	117.3682705	150.536634	197.2990588	187.5894855	229.184809	202.5633892	200.3083977	205.7211946	Papola	poly (A) polymerase alpha [Source:MGI Symbol;Acc:MGI:109301]
ENSMUSG00000021112	11.7401857	14.33385067	9.398392629	11.03494657	14.86138013	17.34588206	8.220930309	7.903017128	9.808224157	20.01576686	19.0740757	20.55817347	Mpp5	membrane protein, palmitoylated 5 (MAGUK p55 subfamily member 5) [Source:MGI Symbol;Acc:MGI:1927339]
ENSMUSG00000021113	13.46237837	14.57050113	12.49600959	14.11335112	15.08435969	15.20260264	11.46740446	12.95656955	12.72849271	14.11860549	14.53952371	14.5358102	Snapc1	small nuclear RNA activating complex, polypeptide 1 [Source:MGI Symbol;Acc:MGI:1922877]
ENSMUSG00000021114	95.11062375	90.59860115	92.65828872	87.0777034	83.05868459	73.59737919	75.5905193	81.78287642	76.86853276	63.43178035	64.67519377	64.02078573	Atp6v1d	ATPase, H+ transporting, lysosomal V1 subunit D [Source:MGI Symbol;Acc:MGI:1921084]
ENSMUSG00000021115	13.85737967	13.13697414	13.84822019	16.69972847	17.10960971	16.06767371	76.19931054	81.66675231	85.54088005	40.58013275	40.35367971	41.86797381	Vrk1	vaccinia related kinase 1 [Source:MGI Symbol;Acc:MGI:1261847]
ENSMUSG00000021116	70.56012563	70.37821185	65.19829476	77.29599428	77.3020178	86.26724972	145.7535678	144.1392923	146.4961574	142.2390525	142.3657087	138.6935696	Eif2s1	eukaryotic translation initiation factor 2, subunit 1 alpha [Source:MGI Symbol;Acc:MGI:95299]
ENSMUSG00000021118	0.625177018	0.313010206	0.278283324	0.431212333	0.314144979	0.393371063	11.42981445	12.41346544	11.15709149	5.373241041	5.068212323	5.034603335	Plek2	pleckstrin 2 [Source:MGI Symbol;Acc:MGI:1351466]
ENSMUSG00000021120	1.668633189	2.792604552	0.888333897	2.657520767	3.506332861	3.304516041	2.1054677	1.630978661	2.402120774	2.905503149	3.981710563	2.602659472	Pigh	phosphatidylinositol glycan anchor biosynthesis, class H [Source:MGI Symbol;Acc:MGI:99463]
ENSMUSG00000021123	1.275496614	0.689436697	0.772846495	0.315786577	0.574334353	0.3172319	1.580284378	1.95980909	1.45368971	1.140901604	1.516746088	1.384134471	Rdh12	retinol dehydrogenase 12 [Source:MGI Symbol;Acc:MGI:1925224]
ENSMUSG00000021124	140.104399	124.9486727	151.5490639	135.2046206	116.6326774	100.1948631	80.52891631	78.936755	65.94336484	65.65354687	68.67743743	66.702319	Vti1b	vesicle transport through interaction with t-SNAREs 1B [Source:MGI Symbol;Acc:MGI:1855688]
ENSMUSG00000021125	0.572507891	0.208191242	0.061697829	0.787323095	0.664828211	0.66557885	0	0.029850033	0.420684365	0.61990422	0.526718757	0.61418544	Arg2	arginase type II [Source:MGI Symbol;Acc:MGI:1330806]
ENSMUSG00000021127	169.3350281	183.5052547	145.6783441	151.439957	166.2347539	175.8189649	99.58517378	89.07026092	110.7470287	96.32682695	96.75530945	100.9462727	Zfp36l1	zinc finger protein 36, C3H type-like 1 [Source:MGI Symbol;Acc:MGI:107946]
ENSMUSG00000021130	1.04771297	1.372908493	1.128122276	0.370854959	0.702228287	0.591638492	0.967628566	0.518954485	1.109674224	0.775576149	0.631531809	0.768421253	Galnt16	polypeptide N-acetylgalactosaminyltransferase 16 [Source:MGI Symbol;Acc:MGI:1917754]
ENSMUSG00000021131	238.180501	202.7187866	255.6405492	221.4948747	202.487678	168.3553833	541.9584214	581.512765	484.7245446	338.3983982	354.2559869	344.1116981	Erh	ERH mRNA splicing and mitosis factor [Source:MGI Symbol;Acc:MGI:108089]
ENSMUSG00000021133	51.93822694	67.81661816	42.30640065	53.40227513	64.68053412	78.16427717	16.27468011	15.16458729	19.61239555	34.20758424	35.01360915	34.43090841	Susd6	sushi domain containing 6 [Source:MGI Symbol;Acc:MGI:2444661]
ENSMUSG00000021134	206.6499744	241.1968589	179.3555901	262.9348351	280.5974947	282.7067306	263.0551003	254.41969	257.9105134	244.3358868	236.8716295	246.3586342	Srsf5	serine and arginine-rich splicing factor 5 [Source:MGI Symbol;Acc:MGI:98287]
ENSMUSG00000021135	0.03215881	0.407357752	0.032923954	0.240080585	0.067575962	0.39191654	0.143553266	0.09557365	0.029932101	0.115061185	0.267689652	0.22799943	Slc10a1	solute carrier family 10 (sodium/bile acid cotransporter family), member 1 [Source:MGI Symbol;Acc:MGI:97379]
ENSMUSG00000021136	2.213965798	2.650322929	2.08735953	3.606809492	3.048933098	2.667652843	0.703527851	0.755865323	0.931375617	10.64013256	10.48476558	10.9410398	Smoc1	SPARC related modular calcium binding 1 [Source:MGI Symbol;Acc:MGI:1929878]
ENSMUSG00000021139	0.224574571	0.155165498	0	0	0.047190311	0	0.040099013	0	0.167219839	0.04017533	0.037387135	0.039804702	Gm20498	predicted gene 20498 [Source:MGI Symbol;Acc:MGI:5141963]
ENSMUSG00000021140	8.485005675	10.09731948	7.488695316	8.626257138	9.568101376	13.24610864	6.212006789	6.286089737	8.32300684	9.683330256	10.38887272	9.910017517	Pcnx	pecanex homolog [Source:MGI Symbol;Acc:MGI:1891924]
ENSMUSG00000021143	25.50588036	30.6762921	26.06078481	27.16357522	28.53952801	32.60654333	23.88848237	21.88260892	27.06151394	18.00341194	18.79541225	18.91655885	Pacs2	phosphofurin acidic cluster sorting protein 2 [Source:MGI Symbol;Acc:MGI:1924399]
ENSMUSG00000021144	62.11466721	60.22947598	61.51421986	63.70325114	60.51474251	59.16605113	102.6646748	101.2018854	110.7372006	52.05310531	53.55964774	55.87652269	Mta1	metastasis associated 1 [Source:MGI Symbol;Acc:MGI:2150037]
ENSMUSG00000021147	16.85875332	25.64748472	15.54971592	22.05410285	26.74798418	33.0803199	24.30271142	18.23319869	26.60346226	27.64160921	27.21670552	27.33178	Wdr37	WD repeat domain 37 [Source:MGI Symbol;Acc:MGI:1920393]
ENSMUSG00000021148	0	0	0	0	0	0	0	0	0	0	0	0	Gm9745	predicted gene 9745 [Source:MGI Symbol;Acc:MGI:3704398]
ENSMUSG00000021149	30.02719339	31.98404069	28.130363	35.03769602	34.87200538	37.65606297	70.92996727	70.50164264	79.83624162	72.90195632	72.30928094	71.73037152	Gtpbp4	GTP binding protein 4 [Source:MGI Symbol;Acc:MGI:1916487]
ENSMUSG00000021156	31.93042977	43.78227692	28.62192649	44.38824353	50.05030217	60.1036138	17.27618847	14.83752185	18.84279752	25.46935917	26.2069925	25.19272228	Zmynd11	zinc finger, MYND domain containing 11 [Source:MGI Symbol;Acc:MGI:1913755]
ENSMUSG00000021171	25.79510433	31.96742943	21.97688113	28.94487367	40.25576223	50.76780584	17.33637458	15.38396931	22.6019964	31.5918472	33.14004735	31.83628644	Esyt2	extended synaptotagmin-like protein 2 [Source:MGI Symbol;Acc:MGI:1261845]

ENSMUSG00000021175	9.416670623	13.27494811	5.935388851	15.54227296	15.52715607	20.19022611	17.74143961	17.29600207	23.2996232	21.07246134	22.39949456	20.58107706	Cdca7l	cell division cycle associated 7 like [Source:MGI Symbol;Acc:MGI:2384982]
ENSMUSG00000021176	1.343563671	0.91244104	1.128640517	1.671722056	1.84597617	2.282898155	4.490447313	4.163613626	3.879861757	2.157051758	2.437497531	2.503521365	Efcab11	EF-hand calcium binding domain 11 [Source:MGI Symbol;Acc:MGI:1926017]
ENSMUSG00000021177	21.48630334	20.68853152	19.82351259	24.35282357	19.52177383	21.64362272	62.98539385	67.62009476	65.82104059	26.74163897	27.83948049	30.01109122	Tdp1	tyrosyl-DNA phosphodiesterase 1 [Source:MGI Symbol;Acc:MGI:1920036]
ENSMUSG00000021178	335.8998082	282.0510889	356.5718118	318.1390936	292.7322748	251.4979127	592.2369438	633.6231778	529.0363725	291.6203606	284.5797402	284.1516446	Psmc1	protease (prosome, macropain) 26S subunit, ATPase 1 [Source:MGI Symbol;Acc:MGI:106054]
ENSMUSG00000021179	16.31161717	16.90240486	16.05642764	18.38804125	18.45830288	17.57391994	39.71629188	37.07353226	39.46432162	28.03416709	27.845947	28.53285684	Nrde2	nrde-2 necessary for RNA interference, domain containing [Source:MGI Symbol;Acc:MGI:2670969]
ENSMUSG00000021180	2.394791155	3.115871239	2.197040306	3.51179676	4.558410126	4.903666261	6.289096095	6.208245979	7.757985261	7.246921026	6.925199993	6.408312497	Rps6ka5	ribosomal protein S6 kinase, polypeptide 5 [Source:MGI Symbol;Acc:MGI:1920336]
ENSMUSG00000021182	3.002373283	3.763012559	2.58131921	4.179429073	4.409292422	6.254101856	36.06020419	30.3507867	42.75100253	20.74255088	21.77456154	20.41889152	Ccdc88c	coiled-coil domain containing 88C [Source:MGI Symbol;Acc:MGI:1915589]
ENSMUSG00000021185	18.50933359	19.9461141	16.86759022	20.74845137	23.93664015	23.32298866	3.202937912	3.735138009	3.445545697	7.296975037	7.893788276	8.059955425	Dglucy	D-glutamate cyclase [Source:MGI Symbol;Acc:MGI:2444813]
ENSMUSG00000021186	37.17682016	44.1795441	33.48552912	30.9920719	32.62707815	34.0531028	1.118655312	0.70381916	0.613181245	8.469432408	8.526803439	8.746184084	Fbln5	fibulin 5 [Source:MGI Symbol;Acc:MGI:1346091]
ENSMUSG00000021187	0.152745786	0.087947312	0.10946601	0.370603629	0.288871044	0.197784292	0.06818398	0.181579756	0.099518658	0.204941248	0.267005462	0.108293659	Tc2n	tandem C2 domains, nuclear [Source:MGI Symbol;Acc:MGI:1921663]
ENSMUSG00000021188	9.135278954	12.82189004	9.095095349	11.4777172	14.42493112	19.01901628	4.586142578	4.052734443	5.20022829	13.48855191	13.67653614	13.59878047	Trip11	thyroid hormone receptor interactor 11 [Source:MGI Symbol;Acc:MGI:1924393]
ENSMUSG00000021189	13.83058537	14.41388559	12.68786008	15.72832081	17.4999942	22.80588533	52.35572853	50.09028186	62.05772903	45.98159116	45.08057196	46.08458182	Atxn3	ataxin 3 [Source:MGI Symbol;Acc:MGI:1099442]
ENSMUSG00000021190	226.3672621	203.0211821	219.1749528	178.5149102	166.986209	142.3468658	71.22079045	79.88543034	73.93889428	80.84720775	79.53624133	77.20614058	Lgmn	legumain [Source:MGI Symbol;Acc:MGI:1330838]
ENSMUSG00000021192	27.2415853	30.27086449	25.03310463	32.53048396	36.58710747	33.50108935	27.48366479	26.60478464	30.49818066	21.65769097	21.75905746	20.61194123	Golga5	golgi autoantigen, golgin subfamily a, 5 [Source:MGI Symbol;Acc:MGI:1351475]
ENSMUSG00000021193	57.50984369	68.02934983	47.25962057	67.47932159	80.30690132	89.28850942	87.04703189	78.06293802	101.8783765	138.2535791	143.5069907	133.8617939	Pitrm1	pitrilysin metallepetidase 1 [Source:MGI Symbol;Acc:MGI:1916867]
ENSMUSG00000021194	2.08456111	2.4910634	3.422706825	2.312311795	2.066193709	1.707604366	29.17983888	32.4174637	26.50419168	12.73550624	12.50644098	13.28028948	Chga	chromogranin A [Source:MGI Symbol;Acc:MGI:88394]
ENSMUSG00000021196	62.30889392	65.80795044	59.48386002	68.06257357	68.83786781	73.98933389	189.7120666	176.6050746	209.3496726	227.0091646	241.0237422	232.9297742	Pfkp	phosphofructokinase, platelet [Source:MGI Symbol;Acc:MGI:1891833]
ENSMUSG00000021198	0	0.011127221	0	0.036068698	0.010152328	0	0.043133678	0.009572389	0.008993746	0.121004161	0.128693018	0.059943933	Unc79	unc-79 homolog [Source:MGI Symbol;Acc:MGI:2684729]
ENSMUSG00000021200	0.044382143	0.178879118	0.136314338	0	0	0	0.019811683	0	0	0.535933518	0.480267541	0.865316042	Asb2	ankyrin repeat and SOCS box-containing 2 [Source:MGI Symbol;Acc:MGI:1929743]
ENSMUSG00000021203	3.959037621	5.49883648	4.319893785	6.432982526	6.111697598	7.618174288	4.960755974	4.936891965	5.818278672	3.354883267	3.555670447	3.062327697	Otub2	OTU domain, ubiquitin aldehyde binding 2 [Source:MGI Symbol;Acc:MGI:1915399]
ENSMUSG00000021207	0	0.139707281	0	0.037738231	0	0.061605302	0.252729303	0.040061887	0.30112144	0.180864504	0.033662477	0.035839196	Akr1c21	aldo-keto reductase family 1, member C21 [Source:MGI Symbol;Acc:MGI:1924587]
ENSMUSG00000021208	1.535325732	1.949340505	2.176414923	0.404106364	0.413616819	0.299853803	0.035146253	0	0.036641493	0.915541744	1.146926479	1.186201976	Ifi27l2b	interferon, alpha-inducible protein 27 like 2B [Source:MGI Symbol;Acc:MGI:1916390]
ENSMUSG00000021209	0.265104427	0.493146838	0.187900593	0.114180506	0.25710892	0.341719646	1.529311726	1.151503943	1.518451243	3.356296594	3.174291808	3.108464268	Ppp4r4	protein phosphatase 4, regulatory subunit 4 [Source:MGI Symbol;Acc:MGI:1921771]
ENSMUSG00000021210	0	0	0	0	0	0	0	0	0	0	0	0	Akr1c6	aldo-keto reductase family 1, member C6 [Source:MGI Symbol;Acc:MGI:1933427]
ENSMUSG00000021211	0.068245024	0.078587654	0.279475021	0.063685135	0.071702329	0.207924001	0.060927604	0	0	0.122087126	0.113614197	0.06048042	Akr1c12	aldo-keto reductase family 1, member C12 [Source:MGI Symbol;Acc:MGI:1351661]
ENSMUSG00000021213	27.62068201	22.96486533	26.90616479	30.72823034	26.31286553	24.80620484	0.460058133	0.765733791	0.287778334	0.921867458	1.372622773	1.278708181	Akr1c13	aldo-keto reductase family 1, member C13 [Source:MGI Symbol;Acc:MGI:1351662]
ENSMUSG00000021214	0.70967124	0.602164252	0.497117407	0.975952586	1.844436599	1.280235181	0.366808502	0.444019247	0.312883996	0.567964782	0.373092453	0.264811838	Akr1c18	aldo-keto reductase family 1, member C18 [Source:MGI Symbol;Acc:MGI:2145420]
ENSMUSG00000021215	16.39484266	19.39373184	14.49903565	26.81859417	27.51373563	28.25514159	53.13722587	50.14619741	58.24791348	58.91597521	60.34970939	56.42199982	Net1	neuroepithelial cell transforming gene 1 [Source:MGI Symbol;Acc:MGI:1927138]
ENSMUSG00000021216	0.019390936	0	0	0	0	0	0	0	0	0	0	0	Tubal3	tubulin, alpha-like 3 [Source:MGI Symbol;Acc:MGI:3588215]
ENSMUSG00000021217	1.151576166	1.36193967	1.354228306	0.653492406	0.572257194	1.209023546	0.972527951	0.462486593	0.825606379	2.644742716	2.189168881	2.303144696	Tshz3	teashirt zinc finger family member 3 [Source:MGI Symbol;Acc:MGI:2442819]
ENSMUSG00000021218	232.3286714	236.3019298	208.6685536	231.9039457	237.5419534	238.3303781	285.7076691	287.8615727	319.1050816	307.4085642	304.2758772	300.3356493	Gdi2	guanosine diphosphate (GDP) dissociation inhibitor 2 [Source:MGI Symbol;Acc:MGI:99845]
ENSMUSG00000021219	0.216631783	0.093548488	0.083169761	0	0	0	0	0	0	0.169550513	0.135243079	0.095992208	Rgs6	regulator of G-protein signaling 6 [Source:MGI Symbol;Acc:MGI:1354730]
ENSMUSG00000021221	0.184107222	0.45430485	0.24234123	0.368155359	0.276334464	0.240396239	0.446138281	0.468988544	0.489598429	0.752820858	0.569216847	0.559406915	Dpf3	D4, zinc and double PHD fingers, family 3 [Source:MGI Symbol;Acc:MGI:1917377]
ENSMUSG00000021222	43.35446122	40.96951292	40.61960733	45.97660124	44.91475784	41.64145046	77.99379463	79.16228859	76.02403871	66.31260007	65.54798315	63.26595551	Dcaf4	DDB1 and CUL4 associated factor 4 [Source:MGI Symbol;Acc:MGI:1921078]
ENSMUSG00000021223	0.073733027	0.012129624	0.053919523	0.127783406	0.09960217	0.064184129	0.225693145	0.062608344	0.117647446	0.329762258	0.526074058	0.382729276	Papln	papilin, proteoglycan-like sulfated glycoprotein [Source:MGI Symbol;Acc:MGI:2386139]
ENSMUSG00000021224	21.48761737	23.05769539	17.74629634	22.01483995	23.94698051	27.1359156	16.42615357	13.63710598	17.99237872	24.19787985	21.76495775	22.62961361	Numb	NUMB endocytic adaptor protein [Source:MGI Symbol;Acc:MGI:107423]
ENSMUSG00000021226	6.152525127	7.130076019	4.513550225	11.97656101	11.38442625	10.82020262	1.976720633	2.193406553	2.352614092	3.014540171	2.83794915	3.525036271	Acot2	acyl-CoA thioesterase 2 [Source:MGI Symbol;Acc:MGI:2159605]
ENSMUSG00000021228	0	0.021760876	0.038693237	0.035268755	0.019854334	0	0.050612456	0.074880716	0.01758856	0.202835132	0.220217943	0.217710918	Acot3	acyl-CoA thioesterase 3 [Source:MGI Symbol;Acc:MGI:2159619]
ENSMUSG00000021234	1.276411036	1.624574283	0.980085192	1.128434974	1.323428523	1.675798869	1.124556618	0.915074795	1.594462707	1.787692427	1.733525589	1.547939925	Fam161b	family with sequence similarity 161, member B [Source:MGI Symbol;Acc:MGI:2443027]
ENSMUSG00000021235	17.74038104	17.19747057	18.92199353	20.97974067	19.24911906	15.821941	28.1920106	31.66582839	28.1905197	24.58151975	24.83553959	26.32714034	Coq6	coenzyme Q6 monooxygenase [Source:MGI Symbol;Acc:MGI:1924408]
ENSMUSG00000021236	10.38284065	12.45123137	9.139706549	11.57775859	13.44306631	17.02052876	4.937991873	4.133498762	5.664168015	5.790554219	6.069483409	5.073740218	Entpd5	ectonucleoside triphosphate diphosphohydrolase 5 [Source:MGI Symbol;Acc:MGI:1321385]
ENSMUSG00000021238	6.74179072	9.84140149	6.333779752	9.085418257	10.18749662	10.6477809	12.39188841	11.00021876	13.04827195	12.20263634	10.18387658	13.10928428	Aldh6a1	aldehyde dehydrogenase family 6, subfamily A1 [Source:MGI Symbol;Acc:MGI:1915077]
ENSMUSG00000021239	0.118790304	0.106394665	0.02702592	0	0.013867582	0.030160142	0.035351093	0.03922624	0	0.023612249	0.054933858	0.16376094	Vsx2	visual system homeobox 2 [Source:MGI Symbol;Acc:MGI:88401]
ENSMUSG00000021240	43.82056611	38.87235958	48.03531776	51.84950982	49.02667117	42.58670792	25.49897731	27.00175518	25.06595515	19.37943082	19.83793374	20.85231929	Abcd4	ATP-binding cassette, sub-family D (ALD), member 4 [Source:MGI Symbol;Acc:MGI:1349217]
ENSMUSG00000021241	37.91956447	34.51470218	40.56526469	47.83455209	39.90021765	33.89819549	34.66459127	36.27133478	33.28623412	24.47387835	20.08202121	21.3805864	Isca2	iron-sulfur cluster assembly 2 [Source:MGI Symbol;Acc:MGI:1921566]
ENSMUSG00000021242	415.2150837	360.4444445	460.778491	335.4190518	306.9624173	256.5951406	95.37057607	100.1026186	88.47387903	128.5744119	135.9242247	125.9082346	Npc2	NPC intracellular cholesterol transporter 2 [Source:MGI Symbol;Acc:MGI:1915213]
ENSMUSG00000021243	13.78881441	12.19244161	12.22623253	94.43822255	13.19384398	10.12759436	14.89331252	13.53782287	19.36568414	13.65524415	11.6315175	103.7062258	Fcf1	FCF1 rRNA processing protein [Source:MGI Symbol;Acc:MGI:1920986]
ENSMUSG00000021244	14.7472086	17.50604032	12.87283082	16.94582897	18.0700503	23.79365022	19.14537661	17.57612046	23.05325609	36.39684338	38.7621692	37.44976014	Ylpm1	YLP motif containing 1 [Source:MGI Symbol;Acc:MGI:1926195]
ENSMUSG00000021245	1.622368879	1.903490996	1.42070335	1.951971296	2.026173149	3.271961747	6.659060547	5.88291282	8.072510978	10.87921648	10.54886397	10.56182895	Mlh3	mutL homolog 3 [Source:MGI Symbol;Acc:MGI:1353455]
ENSMUSG00000021248	138.1594983	135.6323999	136.1447356	134.0550437	135.0571897	136.8070971	76.95031993	76.21796002	76.70324153	75.78765958	74.6700324	72.43455993	Tmed10	transmembrane p24 trafficking protein 10 [Source:MGI Symbol;Acc:MGI:1915831]
ENSMUSG00000021250	121.5200151	138.8475438	102.4658567	155.2086355	156.9043283	166.5412459	35.10808092	35.05312384	41.07605284	9.516255422	8.331031476	10.75543466	Fos	FBJ osteosarcoma oncogene [Source:MGI Symbol;Acc:MGI:95574]
ENSMUSG00000021252	32.16197141	29.98165606	27.3646605	31.78249856	33.67636596	26.2747657	43.91664249	46.85230887	44.56316606	26.90895954	28.92667994	27.40398574	Erg28	ergosterol biosynthesis 28 [Source:MGI Symbol;Acc:MGI:1915571]
ENSMUSG00000021253	17.09438003	16.14057529	18.29542168	12.05909946	10.57043447	10.52101283	0.295236833	0.239400268	0.603756053	3.185524855	2.837399535	2.998330667	Tgfb3	transforming growth factor, beta 3 [Source:MGI Symbol;Acc:MGI:98727]
ENSMUSG00000021254	4.552387035	7.028575492	3.642251094	8.166009011	9.140866876	12.99659439	5.509340357	4.175729594	5.947738412	8.837754034	9.12263678	9.010243545	Gpatch2l	G patch domain containing 2 like [Source:MGI Symbol;Acc:MGI:1917623]
ENSMUSG00000021255	2.214954082	0.375674699	0.281259379	0.36852753	0.306680531	3.007990721	215.0986562	196.2387889	235.212854	160.463796	158.31745	157.1726945	Esrrb	estrogen related receptor, beta [Source:MGI Symbol;Acc:MGI:1346832]
ENSMUSG00000021256	0.463562008	0.781658384	0.220345999	0.942424836	1.095853811	1.90414664	1.788456076	1.246465727	2.57337757	2.191696431	2.287649607	2.3181989	Vash1	vasohibin 1 [Source:MGI Symbol;Acc:MGI:2442543]
ENSMUSG00000021257	13.47045683	16.17672022	14.18498217	17.63448803	18.27742388	17.53029011	45.76796567	39.76633198	47.93002213	19.72594341	18.86953458	21.24937354	Angel1	angel homolog 1 [Source:MGI Symbol;Acc:MGI:1915987]
ENSMUSG00000021258	21.43617253	19.85576754	18.0410978	23.48010523	23.3658338	24.39787125	72.05025507	63.81723596	71.71061138	26.79592475	27.81424545	26.68710281	Ccnk	cyclin K [Source:MGI Symbol;Acc:MGI:1276106]
ENSMUSG00000021259	7.887321779	8.229824884	7.885428607	9.710092316	8.053461186	7.417872141	3.206747715	2.677871671	3.170845043	0.993665225	1.756937954	1.4767476	Cyp46a1	cytochrome P450, family 46, subfamily a, polypeptide 1 [Source:MGI Symbol;Acc:MGI:1341877]
ENSMUSG00000021260	1.443828904	1.360344439	1.441532314	0.85740926	0.626848563	0.699833497	0.319591132	0.141849743	0.355400124	1.088677917	1.052853245	1.543927921	Hhipl1	hedgehog interacting protein-like 1 [Source:MGI Symbol;Acc:MGI:1919265]
ENSMUSG00000021262	36.83832813	35.29294144	33.88417966	39.12653467	36.04525127	32.96261794	42.90968674	41.48116381	40.17718589	20.32676485	20.01531277	20.91945794	Evl	Ena-vasodilator stimulated phosphoprotein [Source:MGI Symbol;Acc:MGI:1194884]
ENSMUSG00000021263	0	0.277255483	0.105640888	0.224679732	0.180688722	0.392973869	0.184243904	0.238513936	0.384164523	0.246126084	0.48672013	0.518192949	Degs2	delta(4)-desaturase, sphingolipid 2 [Source:MGI Symbol;Acc:MGI:1917309]
ENSMUSG00000021264	37.66084937	42.76466753	32.19252052	44.54508246	46.75591962	52.13227594	58.97632949	55.37526719	62.56590686	44.43925331	46.40503797	46.075877	Yy1	YY1 transcription factor [Source:MGI Symbol;Acc:MGI:99150]
ENSMUSG00000021265	19.72641225	15.17482831	22.66366934	19.60809422	17.98202018	13.1891717	2.33143372	2.467602761	1.570554962	1.365586108	1.371140891	1.352988215	Slc25a29	solute carrier family 25 (mitochondrial carrier, palmitoylcarnitine transporter), member 29 [Source:MGI Symbol;Acc:MGI:2444911]
ENSMUSG00000021266	49.22343753	45.23605333	50.59125609	59.44596914	54.69399229	53.16633816	97.04466762	94.67124657	98.17852826	56.48624529	58.62215166	63.11506435	Wars	tryptophanyl-tRNA synthetase [Source:MGI Symbol;Acc:MGI:104630]
ENSMUSG00000021268	25.62925098	21.94257129	43.33515844	19.50481554	11.3062609	12.41605517	6.992270885	7.285660983	5.867356272	33.87453644	42.45551944	47.810734	Meg3	maternally expressed 3 [Source:MGI Symbol;Acc:MGI:1202886]
ENSMUSG00000021270	901.8567879	1007.451113	722.7391942	1026.757607	1139.546945	1249.419726	4764.543174	4502.561823	5282.672188	2972.010907	2790.756714	2836.400045	Hsp90aa1	heat shock protein 90, alpha (cytosolic), class A member 1 [Source:MGI Symbol;Acc:MGI:96250]

ENSMUSG00000021271	7.24628642	6.949977452	7.018765238	7.582318102	8.536840868	8.345994361	7.445820558	7.31392212	7.089954196	5.503272541	6.487032826	6.439146865	Zfp839	zinc finger protein 839 [Source:MGI Symbol;Acc:MGI:1920055]
ENSMUSG00000021273	22.42685173	20.72761777	24.59053858	28.64740454	24.97065959	22.67264153	88.84301765	94.96638278	85.95386491	35.37905573	37.06141492	38.21894464	Fdft1	farnesyl diphosphate farnesyl transferase 1 [Source:MGI Symbol;Acc:MGI:102706]
ENSMUSG00000021275	3.641416535	4.510264811	3.85688683	5.210924742	5.594229852	7.026855457	5.217005406	5.0487204	6.127059006	6.007809044	5.990210394	5.380844895	Tecpr2	tectonin beta-propeller repeat containing 2 [Source:MGI Symbol;Acc:MGI:2144865]
ENSMUSG00000021276	54.74125388	52.14550391	57.69941655	57.66917489	54.27057806	50.91544892	135.1957848	134.7714729	131.9158841	68.12107032	64.65802692	69.22989758	Cinp	cyclin-dependent kinase 2 interacting protein [Source:MGI Symbol;Acc:MGI:1914486]
ENSMUSG00000021277	11.35050688	13.7194409	10.12662802	12.79843627	14.74922599	17.78337072	7.420770265	7.145479084	8.475738013	8.318819948	9.371275211	8.749533186	Traf3	TNF receptor-associated factor 3 [Source:MGI Symbol;Acc:MGI:108041]
ENSMUSG00000021278	2.138842962	2.124930495	2.404411317	1.721981506	0.660940327	1.533287515	5.428999844	6.356486061	4.410871464	2.513346732	2.757829593	2.192827852	Amn	amnionless [Source:MGI Symbol;Acc:MGI:1934943]
ENSMUSG00000021279	55.83612552	82.71163883	42.51585181	73.52215274	84.92530759	110.5211712	48.83283314	40.78035099	62.32543845	62.62684125	65.45248622	66.26824687	Cdc42bpb	CDC42 binding protein kinase beta [Source:MGI Symbol;Acc:MGI:2136459]
ENSMUSG00000021280	2.78714368	4.435655329	2.949641207	3.068497214	4.17864369	3.36327286	0.251625682	0.093069536	0.291478541	0.588244028	0.703825064	0.888102584	Exoc3l4	exocyst complex component 3-like 4 [Source:MGI Symbol;Acc:MGI:1921363]
ENSMUSG00000021281	115.2044298	121.9969135	119.03007	130.017164	134.397709	132.4823574	5.990195048	5.731884823	5.461247935	32.82654203	32.51557256	32.69222511	Tnfaip2	tumor necrosis factor, alpha-induced protein 2 [Source:MGI Symbol;Acc:MGI:104960]
ENSMUSG00000021282	169.7071372	199.7378237	157.3451099	207.081143	227.6431294	250.4187755	234.2852474	224.0439988	265.9821213	240.1850033	241.4133092	239.130521	Eif5	eukaryotic translation initiation factor 5 [Source:MGI Symbol;Acc:MGI:95309]
ENSMUSG00000021285	9.850129806	14.21797071	11.00307791	11.63103914	12.1115667	12.3459706	9.873627497	9.66134354	9.549344379	8.932837163	9.303295399	9.766587103	Ppp1r13b	protein phosphatase 1, regulatory subunit 13B [Source:MGI Symbol;Acc:MGI:1336199]
ENSMUSG00000021286	49.76979314	45.18285084	49.7138001	49.39320101	48.25171799	44.72831458	90.88851031	89.99949484	79.46104604	37.92265964	41.55985062	36.59265272	Zfyve21	zinc finger, FYVE domain containing 21 [Source:MGI Symbol;Acc:MGI:1915770]
ENSMUSG00000021287	19.50565268	24.7233579	18.3703367	25.90815686	33.29658302	30.87013562	40.24792802	39.35373906	46.07314216	19.76301178	21.21518247	24.09018564	Xrcc3	X-ray repair complementing defective repair in Chinese hamster cells 3 [Source:MGI Symbol;Acc:MGI:1921585]
ENSMUSG00000021288	148.4038265	146.259497	145.6730954	149.2743882	150.6849584	144.6202401	61.79571704	59.84167223	62.97020631	58.34650381	55.75501092	60.10051415	Klc1	kinesin light chain 1 [Source:MGI Symbol;Acc:MGI:107978]
ENSMUSG00000021290	136.9771512	123.0082953	220.4536189	142.9919702	145.7918913	91.81019318	281.9885139	254.5451822	140.3469175	104.3712975	95.40739756	92.91773282	Atp5mpl	ATP synthase membrane subunit 6.8PL [Source:MGI Symbol;Acc:MGI:1917507]
ENSMUSG00000021294	0.878371417	1.34865312	0.635484284	0.459021793	0.861345205	0.59767539	8.626089986	6.752379794	9.832426422	4.787425322	4.63065211	4.950842552	Kif26a	kinesin family member 26A [Source:MGI Symbol;Acc:MGI:2447072]
ENSMUSG00000021298	0.606889814	1.572445841	1.366924548	1.444165987	1.243388163	1.178755326	0.216726962	0.030060541	0.141217036	0.461421318	0.202069819	0.430272549	Gpr132	G protein-coupled receptor 132 [Source:MGI Symbol;Acc:MGI:1890220]
ENSMUSG00000021301	0.014342829	0.016516505	0.014684083	0	0.060277758	0.120171409	0.025609903	0.07104308	0.066748586	0.282245087	0.561131049	0.139820651	Hecw1	HECT, C2 and WW domain containing E3 ubiquitin protein ligase 1 [Source:MGI Symbol;Acc:MGI:2444115]
ENSMUSG00000021302	30.78432638	33.08641868	27.42136835	28.27298237	29.749047	33.22443159	20.12354522	18.7112654	23.27836697	27.10025765	26.17498418	27.71340446	Ggps1	geranylgeranyl diphosphate synthase 1 [Source:MGI Symbol;Acc:MGI:1341724]
ENSMUSG00000021303	3.585707319	4.77054398	4.775891203	6.334893348	7.717601271	7.398019221	0.031079979	0.068973864	0.097206678	0.093417394	0.057956109	0	Gng4	guanine nucleotide binding protein (G protein), gamma 4 [Source:MGI Symbol;Acc:MGI:102703]
ENSMUSG00000021306	37.92647406	45.96198511	35.36198468	54.45789098	63.9937266	67.18158375	6.913061357	7.71559153	8.446882955	13.28703141	13.86823405	14.80501022	Gpr137b	G protein-coupled receptor 137B [Source:MGI Symbol;Acc:MGI:1891463]
ENSMUSG00000021311	7.415993944	9.611157568	6.385161722	10.09950447	11.13688476	14.78024499	14.56348761	12.96687633	18.90261415	25.09218514	26.53546225	24.93037344	Mtr	5-methyltetrahydrofolate-homocysteine methyltransferase [Source:MGI Symbol;Acc:MGI:894292]
ENSMUSG00000021313	0.216223557	0.426844356	0.221368091	0.480419692	0.313721281	0.572506223	0.937620272	1.377001541	1.389597265	1.584094333	1.825555032	1.72460373	Ryr2	ryanodine receptor 2, cardiac [Source:MGI Symbol;Acc:MGI:99685]
ENSMUSG00000021314	0.080698589	0.111514289	0.099142349	0.090367917	0.016957359	0.086053318	6.123898342	7.210899217	8.006825867	4.995062402	5.226093234	4.920374799	Amph	amphiphysin [Source:MGI Symbol;Acc:MGI:103574]
ENSMUSG00000021318	9.56047546	11.6234479	9.748948849	9.442998476	13.3130427	16.67229613	1.688938701	1.962122231	2.221670253	8.619759952	7.947562809	8.180177548	Gli3	GLI-Kruppel family member GLI3 [Source:MGI Symbol;Acc:MGI:95729]
ENSMUSG00000021319	0.321108118	0.098606001	0.109582712	0.199768546	0.157441905	0.195665795	6.402475679	6.892239056	6.694783538	8.099706356	6.664433547	6.962575135	Sfrp4	secreted frizzled-related protein 4 [Source:MGI Symbol;Acc:MGI:892010]
ENSMUSG00000021322	2.76576742	3.015812384	3.207444838	0.091361724	0.077147301	0.242356239	6.140258245	5.334292659	7.176042508	7.772029614	6.315831285	7.765403799	Aoah	acyloxyacyl hydrolase [Source:MGI Symbol;Acc:MGI:1350928]
ENSMUSG00000021326	50.82600521	44.59222572	50.1850552	51.50408341	48.40852965	40.46099008	98.16726512	98.20641268	93.15821985	70.20103352	70.90693376	69.35545761	Trim27	tripartite motif-containing 27 [Source:MGI Symbol;Acc:MGI:97904]
ENSMUSG00000021327	18.26495307	22.73381666	14.44886925	24.79347087	26.74222035	30.73658152	6.563636394	5.527379183	7.637137972	16.83665043	15.59987483	14.95065828	Zkscan3	zinc finger with KRAB and SCAN domains 3 [Source:MGI Symbol;Acc:MGI:1919989]
ENSMUSG00000021335	0.034122512	0.432232095	0	0.031842568	0.071702329	0	0	0.13521284	0.095279504	0.061043563	0.028403549	0	Slc17a1	solute carrier family 17 (sodium phosphate), member 1 [Source:MGI Symbol;Acc:MGI:103209]
ENSMUSG00000021336	0.030248498	0	0	0.0141137	0	0	0.013502585	0.04494817	0	0	0	0	Slc17a4	solute carrier family 17 (sodium phosphate), member 4 [Source:MGI Symbol;Acc:MGI:2442850]
ENSMUSG00000021337	0	0	0.186398802	0	0	0	0.108363481	0.180363248	0.112973629	0.271424304	0.10103491	0.107568137	Scgn	secretagogin, EF-hand calcium binding protein [Source:MGI Symbol;Acc:MGI:2384873]
ENSMUSG00000021338	8.61035181	10.98718302	7.933693519	12.68233156	14.45003577	16.90990573	13.83682683	12.05695963	16.00666467	14.61252158	14.79940123	14.84894071	Carmil1	capping protein regulator and myosin 1 linker 1 [Source:MGI Symbol;Acc:MGI:1915982]
ENSMUSG00000021339	16.7400727	15.29999208	15.41204787	16.03864129	17.39602429	18.66169447	10.70102167	9.217487369	10.75919132	10.12171731	10.41699495	10.56846792	Mrs2	MRS2 magnesium transporter [Source:MGI Symbol;Acc:MGI:2685748]
ENSMUSG00000021340	0.543605774	0.650066532	0.149837586	0.370707521	0.417375145	0.493680583	0.05599833	0.020712268	0	0.2618229	0.348074592	0.444698597	Gpld1	glycosylphosphatidylinositol specific phospholipase D1 [Source:MGI Symbol;Acc:MGI:106604]
ENSMUSG00000021342	0.350231878	0.115231432	0.256117734	0.046690091	0.052567812	0.266765284	0	0	0.046568781	0.358027594	0	0	Prl	prolactin [Source:MGI Symbol;Acc:MGI:97762]
ENSMUSG00000021345	6.634142532	5.379969123	6.98331002	0.959149308	1.374411416	0.889631396	0.041709939	0.092564273	0.173937683	0.459682552	0.738893197	0.207019027	Prl8a6	prolactin family 8, subfamily a, member 6 [Source:MGI Symbol;Acc:MGI:1332225]
ENSMUSG00000021346	0.224809236	0.207103514	0.184126438	0	0.047239622	0.239726379	0	0	0	0	0.074852404	0	Prl8a8	prolactin family 8, subfamily a, member 81 [Source:MGI Symbol;Acc:MGI:1921438]
ENSMUSG00000021347	0	0	0	0.08681833	0	0.070862747	0	0	0	0	0	0	Prl7b1	prolactin family 7, subfamily b, member 1 [Source:MGI Symbol;Acc:MGI:1922846]
ENSMUSG00000021348	0.795841329	0.672064947	0.869094948	1.089243542	1.421469936	1.737710095	0	0	0	0	0	0.023509806	Prl7d1	prolactin family 7, subfamily d, member 1 [Source:MGI Symbol;Acc:MGI:97619]
ENSMUSG00000021356	0.065293608	0.060151157	0.066847117	0.097489476	0.10976223	0.179038656	0	0.025873049	0.012154522	0.070084309	0.021740137	0.023145921	Irf4	interferon regulatory factor 4 [Source:MGI Symbol;Acc:MGI:1096873]
ENSMUSG00000021357	23.17087361	26.98566175	17.36480847	28.42071761	31.72190008	39.82686954	17.25827967	15.10706994	21.85241822	28.83162805	30.30096707	28.95456149	Exoc2	exocyst complex component 2 [Source:MGI Symbol;Acc:MGI:1913732]
ENSMUSG00000021359	14.48982753	13.13098437	14.96357547	15.28536492	15.79753828	12.66814268	0.881160635	1.081768707	1.094559534	0.788918784	0.856528501	1.209682189	Tfap2a	transcription factor AP-2, alpha [Source:MGI Symbol;Acc:MGI:104671]
ENSMUSG00000021360	2.572355251	2.547905695	3.296552168	2.736211073	2.211276441	3.439079925	13.39380782	11.58311079	14.31522766	11.85854148	10.81094653	11.3346552	Gcnt2	glucosaminyl (N-acetyl) transferase 2, I-branching enzyme [Source:MGI Symbol;Acc:MGI:1100870]
ENSMUSG00000021361	100.0388803	89.4493444	107.6243706	91.07019645	86.75644462	76.48466742	89.06025796	99.05612748	78.43323985	57.94249642	54.23474361	58.3675423	Tmem14c	transmembrane protein 14C [Source:MGI Symbol;Acc:MGI:1913404]
ENSMUSG00000021362	0.020097379	0	0.041151098	0	0	0.061231239	0.071769928	0.079637268	0.317998868	0.25167283	0.301122727	0.320594248	Gcm2	glial cells missing homolog 2 [Source:MGI Symbol;Acc:MGI:1861438]
ENSMUSG00000021363	0.171884772	0.19793415	0.087987184	0.066833352	0.300987472	0.163651901	0.741698248	0.525018763	0.693260945	1.396534066	1.025383253	1.282098327	Mak	male germ cell-associated kinase [Source:MGI Symbol;Acc:MGI:96913]
ENSMUSG00000021364	0.090135612	0.064872369	0.034605067	0.073598938	0.21307927	0.094400164	3.470312819	3.136387328	3.848661589	2.529583545	2.607250951	2.895664404	Elovl2	elongation of very long chain fatty acids (FEN1/Elo2, SUR4/Elo3, yeast)-like 2 [Source:MGI Symbol;Acc:MGI:1858960]
ENSMUSG00000021365	10.59357572	14.79924228	10.17587535	10.16144903	13.50263162	13.90364289	0.301440738	0.459915963	0.41247287	2.680378237	2.318699148	2.337720984	Nedd9	neural precursor cell expressed, developmentally down-regulated gene 9 [Source:MGI Symbol;Acc:MGI:97302]
ENSMUSG00000021366	9.782642131	14.2503884	9.472977285	14.70881576	17.87259375	26.43252148	5.388891138	3.748975172	6.798140936	13.10012576	12.56898269	13.83449708	Hivep1	human immunodeficiency virus type I enhancer binding protein 1 [Source:MGI Symbol;Acc:MGI:96100]
ENSMUSG00000021367	1.689758288	1.621536276	1.709367352	1.370361796	1.754226472	0.919327367	0.089796293	0.119567595	0.09361653	0.64776381	0.318148964	0.534823348	Edn1	endothelin 1 [Source:MGI Symbol;Acc:MGI:95283]
ENSMUSG00000021368	20.90071985	19.02852121	20.56050841	22.137849	24.10823821	17.50855475	24.06405783	24.10872565	20.51947973	13.50005665	13.78881613	14.39045928	Tbc1d7	TBC1 domain family, member 7 [Source:MGI Symbol;Acc:MGI:1914296]
ENSMUSG00000021371	7.274886769	7.644153909	5.557461087	8.304030377	10.16894471	11.64005172	5.585289549	5.96099535	5.541392035	4.499518081	4.319756347	3.851381051	Mcur1	mitochondrial calcium uniporter regulator 1 [Source:MGI Symbol;Acc:MGI:1923387]
ENSMUSG00000021373	2.531087519	2.609330235	1.999009673	1.439676516	1.722221651	2.423625056	0.51650224	0.955201069	1.032078975	2.587426311	2.468053604	2.969453082	Cap2	CAP, adenylate cyclase-associated protein, 2 (yeast) [Source:MGI Symbol;Acc:MGI:1914502]
ENSMUSG00000021374	39.02663324	57.50579715	27.73400807	52.60169433	68.2796541	97.01943788	110.0533657	90.97806127	144.9155308	203.4844709	200.0677138	204.1231957	Nup153	nucleoporin 153 [Source:MGI Symbol;Acc:MGI:2385621]
ENSMUSG00000021375	10.55550005	17.29043186	10.15095953	16.04810994	20.21165721	26.9924955	24.77694557	20.72546729	29.38556818	26.47904191	29.22214758	27.68524558	Kif13a	kinesin family member 13A [Source:MGI Symbol;Acc:MGI:1098264]
ENSMUSG00000021376	15.07917989	14.26760606	19.17452855	19.18045593	17.91177792	15.43597847	24.65238961	22.12167319	21.4549026	13.27319376	13.59122507	12.76771362	Tpmt	thiopurine methyltransferase [Source:MGI Symbol;Acc:MGI:98812]
ENSMUSG00000021377	45.89957218	71.17033148	26.84131838	81.85809968	114.5092172	130.6356924	172.1079898	140.4519811	220.2175909	209.0650114	200.4046948	206.6006627	Dek	DEK oncogene (DNA binding) [Source:MGI Symbol;Acc:MGI:1926209]
ENSMUSG00000021379	1.665691007	1.892382124	1.133066451	0.751117278	1.127565228	0.936643874	1.586895764	1.417539232	1.206989238	1.829903312	1.563324261	1.654506387	Id4	inhibitor of DNA binding 4 [Source:MGI Symbol;Acc:MGI:99414]
ENSMUSG00000021381	0.67664527	0.425013715	0.850186387	0.25831401	0	0.023426748	0.713928668	1.066407912	0.772928585	0.110044221	0.025601769	0.245315327	Barx1	BarH-like homeobox 1 [Source:MGI Symbol;Acc:MGI:103124]
ENSMUSG00000021384	0.950905808	0.54750846	1.216912989	0.532421806	0.599447315	0.325929487	0.806499699	1.648513447	0.619544885	0.893090932	0.79153316	1.769703864	Susd3	sushi domain containing 3 [Source:MGI Symbol;Acc:MGI:1913579]
ENSMUSG00000021385	4.505933091	5.261895464	4.483193604	5.330107677	5.814403186	6.641814802	7.841616229	7.481527841	9.628335838	8.480976661	9.498339544	8.807688605	Ippk	inositol 1,3,4,5,6-pentakisphosphate 2-kinase [Source:MGI Symbol;Acc:MGI:1922928]
ENSMUSG00000021388	18.71368285	22.57493018	15.36132345	1.332680083	1.870689035	1.907108959	0.033116254	0.073492841	0.034525131	0.547458142	0.524902485	0.378041728	Aspn	asporin [Source:MGI Symbol;Acc:MGI:1913945]
ENSMUSG00000021390	34.17461348	43.11927841	27.38243923	26.28910474	33.96790248	38.38111146	0.089824289	0.066447152	0.156076195	0.449976224	0.23729029	0.312077552	Ogn	osteoglycin [Source:MGI Symbol;Acc:MGI:109278]
ENSMUSG00000021391	2.847078471	3.433206139	2.41984895	5.08811236	4.289428458	3.580183278	10.33508246	11.49461062	10.87476961	8.985330476	9.501980074	8.235945707	Cenpp	centromere protein P [Source:MGI Symbol;Acc:MGI:1913586]
ENSMUSG00000021392	11.28110784	13.07951039	9.324813706	11.59158422	13.76327743	16.99741471	18.61579425	18.85491472	23.85449104	35.2898259	30.31349454	30.96249818	Nol8	nucleolar protein 8 [Source:MGI Symbol;Acc:MGI:1918180]
ENSMUSG00000021395	19.69613879	24.12340268	17.79669544	22.24464969	25.27845406	29.89667704	22.61606902	21.05276609	28.00620244	29.7784829	29.55312613	29.22627554	Spin1	spindlin 1 [Source:MGI Symbol;Acc:MGI:109242]

ENSMUSG00000021396	0.274739854	0.146020184	0.238003316	0.07888699	0.088817914	0.112680818	1.207539617	1.088675874	0.885172799	0.396978042	0.562937139	0.655526358	Nxnl2	nucleoredoxin-like 2 [Source:MGI Symbol;Acc:MGI:1922374]
ENSMUSG00000021400	26.7868234	24.43913917	25.19984017	25.09592365	28.08817316	25.91246459	27.93592528	28.0322693	25.32664939	22.7752067	23.59855712	23.5512952	Wrnip1	Werner helicase interacting protein 1 [Source:MGI Symbol;Acc:MGI:1926153]
ENSMUSG00000021403	102.7794599	98.02782503	97.03011972	120.5411846	112.2593687	103.4136518	1.448523909	1.564447905	1.651096242	4.992405894	4.357809887	4.888833934	Serpinb9b	serine (or cysteine) peptidase inhibitor, clade B, member 9b [Source:MGI Symbol;Acc:MGI:894668]
ENSMUSG00000021404	0.568363864	1.61700051	0.410743592	1.029576355	0.983553158	1.476995491	0.029848371	0	0	0.209336258	0.05565948	0.118517186	Serpinb9c	serine (or cysteine) peptidase inhibitor, clade B, member 9c [Source:MGI Symbol;Acc:MGI:894669]
ENSMUSG00000021408	16.27941382	20.50625172	12.23673617	18.56604197	22.12727818	23.71622168	3.363325831	4.01678171	3.844380579	10.67756873	10.27657187	10.67292269	Ripk1	receptor (TNFRSF)-interacting serine-threonine kinase 1 [Source:MGI Symbol;Acc:MGI:108212]
ENSMUSG00000021411	26.98813673	25.44051746	25.86715892	21.30498995	19.77379276	16.90232511	3.404403877	3.899448618	3.915611727	20.22323676	20.23274613	22.43496794	Pxdc1	PX domain containing 1 [Source:MGI Symbol;Acc:MGI:1914145]
ENSMUSG00000021413	37.63118622	45.01138179	35.93909312	46.68916089	56.84231565	71.30674176	45.45152221	40.35558105	56.63496241	80.75001407	76.39388324	79.62545305	Prpf4b	pre-mRNA processing factor 4B [Source:MGI Symbol;Acc:MGI:109584]
ENSMUSG00000021414	0.243098801	0.111976306	0.124441385	0.045371161	0.306497074	0.203680354	0.065109922	0.0481648	0.248893032	0.086978455	0.344003889	0.150808098	Fam217a	family with sequence similarity 217, member A [Source:MGI Symbol;Acc:MGI:1919114]
ENSMUSG00000021415	0	0	0	0	0	0	0	0	0	0	0	0	4933417A1	RIKEN cDNA 4933417A18 gene [Source:MGI Symbol;Acc:MGI:1914011]
ENSMUSG00000021416	0	0.087931705	0	0	0	0.029080764	0.715804734	0.302579221	0.355360706	0.136603252	0.063561447	0.23685034	Eci3	enoyl-Coenzyme A delta isomerase 3 [Source:MGI Symbol;Acc:MGI:1916373]
ENSMUSG00000021417	71.52239733	61.07573985	77.15033123	68.6402589	61.60741422	55.64573023	25.06209731	25.83384709	23.20138012	22.88010122	22.47333363	21.81936258	Eci2	enoyl-Coenzyme A delta isomerase 2 [Source:MGI Symbol;Acc:MGI:1346064]
ENSMUSG00000021418	5.673243829	4.965987089	6.200673079	8.120124248	6.886968764	5.839469156	14.42482048	15.34150777	14.80659054	10.33774776	9.508622622	9.953622338	Rpp40	ribonuclease P 40 subunit [Source:MGI Symbol;Acc:MGI:1346084]
ENSMUSG00000021420	23.35175941	19.70518993	25.78859487	20.00529145	19.74847358	16.97017795	16.88339672	17.6343587	15.82012747	12.60104237	13.70218546	13.77398515	Fars2	phenylalanine-tRNA synthetase 2 (mitochondrial) [Source:MGI Symbol;Acc:MGI:1917205]
ENSMUSG00000021423	0.766353371	0.545070628	0.576902177	0	0.023681675	0.034336323	0.100615123	0.022328888	0.020979126	0.584678379	0.225145367	0.319605278	Ly86	lymphocyte antigen 86 [Source:MGI Symbol;Acc:MGI:1321404]
ENSMUSG00000021427	81.71916542	84.7694843	75.40451373	76.48856897	80.53964629	82.58106374	63.67409456	61.5849217	66.82893325	64.501801	64.33672276	65.53626803	Ssr1	signal sequence receptor, alpha [Source:MGI Symbol;Acc:MGI:105082]
ENSMUSG00000021428	28.92420873	29.90826619	27.29224648	33.44731834	34.18037098	35.88562204	31.38677266	30.66225219	36.55213621	37.42107888	37.50471161	34.93534303	Riok1	RIO kinase 1 [Source:MGI Symbol;Acc:MGI:1918590]
ENSMUSG00000021431	22.32229063	21.0062031	18.90317215	24.18634369	26.65805063	25.81915804	47.36028488	46.92845674	51.33060266	44.43282127	40.8951367	43.37841693	Snrnp48	small nuclear ribonucleoprotein 48 (U11/U12) [Source:MGI Symbol;Acc:MGI:1915047]
ENSMUSG00000021432	21.74360686	22.46808171	19.31786665	27.42536759	26.8293858	28.11531124	8.463827123	9.603691344	8.823906874	11.98132855	11.78622128	10.19045641	Slc35b3	solute carrier family 35, member B3 [Source:MGI Symbol;Acc:MGI:1913978]
ENSMUSG00000021439	0	0.15667831	0	0	0	0	0	0	0	0.03042527	0	0.060289177	Ctsq	cathepsin Q [Source:MGI Symbol;Acc:MGI:2137385]
ENSMUSG00000021440	0	0	0	0.112474728	0	0	0	0	0	0	0	0	Cts7	cathepsin 7 [Source:MGI Symbol;Acc:MGI:1860262]
ENSMUSG00000021441	0	0	0	0	0	0	0	0	0	0	0	0	Cts6	cathepsin 6 [Source:MGI Symbol;Acc:MGI:1889619]
ENSMUSG00000021448	0.224179683	0.129077214	0.057378379	0.078450299	0.044163124	0.085376804	0	0.069400599	0.091287548	0.075196287	0.279910456	0.099336775	Shc3	src homology 2 domain-containing transforming protein C3 [Source:MGI Symbol;Acc:MGI:106179]
ENSMUSG00000021451	8.593508192	10.07132506	7.737222414	7.905571415	8.196408992	9.980756993	38.63250197	36.58819222	41.5807763	34.07220819	36.09590213	34.78412269	Sema4d	sema domain, immunoglobulin domain (Ig), transmembrane domain (TM) and short cytoplasmic domain, (semaphorin) 4D [Source:N
ENSMUSG00000021453	180.9688314	128.5110498	231.1034801	132.2947057	105.2367369	73.30941741	13.74512876	14.54656643	9.02866084	8.915516512	8.185656133	8.833268511	Gadd45g	growth arrest and DNA-damage-inducible 45 gamma [Source:MGI Symbol;Acc:MGI:1346325]
ENSMUSG00000021456	0.149577594	0.459323436	0.255227406	0.372222274	0	0.037976849	3.204947269	3.556270028	2.459565505	6.332898291	4.565302784	4.683763496	Fbp2	fructose bisphosphatase 2 [Source:MGI Symbol;Acc:MGI:95491]
ENSMUSG00000021457	4.368495574	3.79352741	3.910324567	0.946753292	1.166261749	1.345507643	11.66831212	10.73627454	12.3646895	13.65495395	14.13797886	13.01067206	Syk	spleen tyrosine kinase [Source:MGI Symbol;Acc:MGI:99515]
ENSMUSG00000021458	42.18262867	37.90326195	39.15755573	20.72880394	18.39244405	16.35899833	17.59859981	19.05411825	16.84118167	31.18512147	33.88827449	27.54043116	Aopep	aminopeptidase O [Source:MGI Symbol;Acc:MGI:1919311]
ENSMUSG00000021460	27.37495614	26.82314945	28.29465678	27.60770017	28.99785085	23.38964049	21.89880715	21.73955331	21.50594124	18.59079316	19.10856803	17.62277841	Auh	AU RNA binding protein/enoyl-coenzyme A hydratase [Source:MGI Symbol;Acc:MGI:1338011]
ENSMUSG00000021461	3.949629853	3.882611299	3.616229494	4.764479833	3.947293468	3.326318512	6.478923149	5.980342812	6.24647214	7.008256563	7.724684338	7.541208763	Fancc	Fanconi anemia, complementation group C [Source:MGI Symbol;Acc:MGI:95480]
ENSMUSG00000021464	2.74547441	3.383418418	2.564235459	1.21359392	0.910913877	1.186223881	6.665263859	7.698100853	8.189154864	10.05282704	11.27964287	11.1268401	Ror2	receptor tyrosine kinase-like orphan receptor 2 [Source:MGI Symbol;Acc:MGI:1347521]
ENSMUSG00000021466	2.015046907	2.576831214	1.230955504	1.901186661	2.655184545	2.874616558	10.70447551	9.264091091	13.70893337	13.26415814	14.53062374	13.92122342	Ptch1	patched 1 [Source:MGI Symbol;Acc:MGI:105373]
ENSMUSG00000021468	46.91300409	51.57584612	43.64435899	56.10952806	57.06852532	58.84656329	31.62704421	30.52221193	35.75375641	41.6952969	45.90418995	44.78262418	Sptlc1	serine palmitoyltransferase, long chain base subunit 1 [Source:MGI Symbol;Acc:MGI:1099431]
ENSMUSG00000021469	1.155414392	0.936291195	1.380055576	0.579040705	0.404649294	0.260757996	0.305637825	0.317945159	0.318640688	3.177027557	3.5799065	3.29941592	Msx2	msh homeobox 2 [Source:MGI Symbol;Acc:MGI:97169]
ENSMUSG00000021470	7.929039894	8.691144878	6.252577447	9.050723431	10.44530904	13.95538943	16.31082312	13.57839503	18.36124407	24.92406209	23.41094966	21.77265848	Ercc6l2	excision repair cross-complementing rodent repair deficiency, complementation group 6 like 2 [Source:MGI Symbol;Acc:MGI:192350
ENSMUSG00000021474	17.97376552	18.1395704	15.02867908	17.96246808	17.62448081	19.23755215	36.31888374	36.09880385	40.94200442	27.6155957	25.97222534	26.06326428	Sfxn1	sideroflexin 1 [Source:MGI Symbol;Acc:MGI:2137677]
ENSMUSG00000021476	24.80898169	24.9107998	21.6364011	27.36512187	25.70723167	26.57515157	9.140813354	9.466631216	12.3886027	9.463484105	10.35826661	10.1206916	Habp4	hyaluronic acid binding protein 4 [Source:MGI Symbol;Acc:MGI:1891713]
ENSMUSG00000021477	1687.845251	1579.271479	1674.204537	2034.87544	1947.020279	1738.000815	239.7048694	235.6210624	227.5757566	463.6735489	467.9344558	457.0734617	Ctsl	cathepsin L [Source:MGI Symbol;Acc:MGI:88564]
ENSMUSG00000021478	0.034395274	0.079215852	0.070427259	0.032097105	0.036137744	0.026198258	0.015353659	0.085183549	0.048020565	0.046148641	0	0.030481938	Drd1	dopamine receptor D1 [Source:MGI Symbol;Acc:MGI:99578]
ENSMUSG00000021481	40.15693077	39.21593572	37.94281404	41.91202431	43.13410979	44.46194041	38.53502567	37.24761248	36.49943528	38.12676544	39.29025339	40.55845963	Zfp346	zinc finger protein 346 [Source:MGI Symbol;Acc:MGI:1349417]
ENSMUSG00000021482	23.57184184	23.31518907	21.23406092	26.23647289	28.3287014	25.60235277	12.19752444	12.42574443	12.74709031	12.161844	10.74232226	10.99931528	Prxl2c	peroxiredoxin like 2C [Source:MGI Symbol;Acc:MGI:1913379]
ENSMUSG00000021483	7.317770039	6.663644958	7.514931222	10.02261064	9.439099435	9.55265803	4.482738073	4.282659738	5.176420945	4.229446854	4.441967289	4.010839269	Cdk20	cyclin-dependent kinase 20 [Source:MGI Symbol;Acc:MGI:2145349]
ENSMUSG00000021484	130.0142723	122.1930346	129.5951219	118.8861342	115.5500718	113.9871775	69.56980841	75.54351775	68.9127691	69.70221575	66.33222225	69.89912617	Lman2	lectin, mannose-binding 2 [Source:MGI Symbol;Acc:MGI:1914140]
ENSMUSG00000021485	21.20738465	18.57125269	28.23951697	26.09021539	23.39225981	15.65985297	17.92896121	19.04990341	16.40682356	5.33707933	4.597728865	4.683518405	Mxd3	Max dimerization protein 3 [Source:MGI Symbol;Acc:MGI:104987]
ENSMUSG00000021486	311.6658037	275.3037017	310.609577	289.9857456	257.8800904	213.9850557	196.6465165	203.3811236	169.8758449	135.9881473	135.7467027	139.3714063	Prelid1	PRELI domain containing 1 [Source:MGI Symbol;Acc:MGI:1913744]
ENSMUSG00000021488	14.35724418	16.03511963	14.00522125	11.07079138	14.05471338	16.73286682	48.07969675	42.56872764	54.84788322	88.4514068	90.14813584	84.91437771	Nsd1	nuclear receptor-binding SET-domain protein 1 [Source:MGI Symbol;Acc:MGI:1276545]
ENSMUSG00000021490	0.325797096	6.217137709	0.214424149	1.324695591	0.415652467	0.141802144	0.789488618	1.890383727	0.10829949	0.437126714	0.232451328	0.185611759	Slc34a1	solute carrier family 34 (sodium phosphate), member 1 [Source:MGI Symbol;Acc:MGI:1345284]
ENSMUSG00000021492	0.070596371	0.08129535	0	0.065879373	0	0	0.031513416	0.034967882	0	0.157866966	0.146910892	0.062564241	F12	coagulation factor XII (Hageman factor) [Source:MGI Symbol;Acc:MGI:1891012]
ENSMUSG00000021493	148.7013918	128.8594214	172.1515567	132.2392014	114.7703438	99.18453614	44.5100902	51.79766323	44.45083845	46.02469663	46.65619306	47.54807233	Pdlim7	PDZ and LIM domain 7 [Source:MGI Symbol;Acc:MGI:1914649]
ENSMUSG00000021494	49.78502724	38.00905744	49.81114692	40.69650007	34.64499227	29.67144618	70.79762362	74.71532884	64.63228961	54.82583339	55.64936025	53.08810848	Ddx41	DEAD (Asp-Glu-Ala-Asp) box polypeptide 41 [Source:MGI Symbol;Acc:MGI:1920185]
ENSMUSG00000021495	54.10916558	46.65045043	58.11920277	56.03618847	50.1445377	48.14148478	44.334572	46.27898998	48.77844743	34.76127824	36.71709361	37.34730715	Fam193b	family with sequence similarity 193, member B [Source:MGI Symbol;Acc:MGI:2385851]
ENSMUSG00000021496	9.395211104	7.182406852	12.04364271	10.75671149	10.20297827	5.592631044	5.49790572	7.273768507	4.041657493	3.036665237	3.680266104	3.42846299	Pcbd2	pterin 4 alpha carbinolamine dehydratase/dimerization cofactor of hepatocyte nuclear factor 1 alpha (TCF1) 2 [Source:MGI Symbol;A
ENSMUSG00000021497	51.56985709	47.90335231	49.77831096	40.27020306	38.78753559	36.17663906	9.444183393	9.859882545	8.997753727	15.98337474	17.04573891	17.68872697	Txndc15	thioredoxin domain containing 15 [Source:MGI Symbol;Acc:MGI:1916922]
ENSMUSG00000021499	0.032572663	0.075018192	0.066695307	0.303962739	0.0342228	0	0.058160264	0.096803591	0.151586493	0.029135478	0.13556728	0.144333477	Catsper3	cation channel, sperm associated 3 [Source:MGI Symbol;Acc:MGI:1924106]
ENSMUSG00000021500	20.05922275	22.61389974	17.04353617	21.64991622	25.01850267	30.71800115	47.10478141	43.76903158	57.19706248	59.6796848	59.98157248	60.49863587	Ddx46	DEAD (Asp-Glu-Ala-Asp) box polypeptide 46 [Source:MGI Symbol;Acc:MGI:1920895]
ENSMUSG00000021501	65.00328131	59.65135367	61.20613356	58.57250437	55.66936653	55.75926651	21.57653883	19.90075255	20.4532364	27.03181328	23.11119488	22.93416286	Caml	calcium modulating ligand [Source:MGI Symbol;Acc:MGI:104728]
ENSMUSG00000021504	33.37013873	28.52377539	34.52518726	24.55792781	25.11256524	19.28005123	9.107178519	10.96553964	10.02491372	12.83741123	13.0756437	14.64245836	B4galt7	xylosylprotein beta1,4-galactosyltransferase, polypeptide 7 (galactosyltransferase I) [Source:MGI Symbol;Acc:MGI:2384987]
ENSMUSG00000021506	0.667108338	0.608165889	0.711438149	0.337206209	0.17522604	0.232890328	0.099263189	0.247824696	0.181100814	0.546986602	0.069412549	0.246336594	Pitx1	paired-like homeodomain transcription factor 1 [Source:MGI Symbol;Acc:MGI:107374]
ENSMUSG00000021508	14.15995167	13.17710252	15.78070894	1.487165846	1.509688834	1.313348732	1.096234451	1.035236131	1.021289838	7.828457034	7.067689454	7.385790283	Cxcl14	chemokine (C-X-C motif) ligand 14 [Source:MGI Symbol;Acc:MGI:1888514]
ENSMUSG00000021509	0	0.014685689	0.078338323	0.035702559	0.040197082	0.009713699	0.079698867	0.012633624	0	0.171108327	0.318466587	0.101717884	Slc25a48	solute carrier family 25, member 48 [Source:MGI Symbol;Acc:MGI:2145373]
ENSMUSG00000021510	5.135753164	5.27014342	4.233475481	5.973585109	6.431826413	8.36163454	2.929723823	3.571591206	3.87616617	5.515204483	5.597917953	6.05118597	Zfp729a	zinc finger protein 729a [Source:MGI Symbol;Acc:MGI:3036250]
ENSMUSG00000021514	14.37249387	14.24484524	16.71889951	16.00741024	13.72144926	13.2350104	15.17525776	18.00688602	15.05467071	12.69665075	11.79697502	11.31762528	Zfp369	zinc finger protein 369 [Source:MGI Symbol;Acc:MGI:2176229]
ENSMUSG00000021518	35.63486034	34.99870716	35.64509541	32.09450517	31.74133338	35.49749559	25.61892029	25.69558143	26.144763	28.54072436	29.38497178	27.14955397	Ptdss1	phosphatidylserine synthase 1 [Source:MGI Symbol;Acc:MGI:1276575]
ENSMUSG00000021519	31.01469275	28.94247408	28.15021128	33.11723756	31.26556596	32.77402064	31.23450752	31.37182716	31.62760097	28.64116189	28.28528951	30.38158995	Mterf3	mitochondrial transcription termination factor 3 [Source:MGI Symbol;Acc:MGI:1913660]
ENSMUSG00000021520	83.94466807	70.54049224	113.0941405	80.37996199	77.75830756	53.68980472	123.4862945	109.5871794	74.92832167	62.07059304	49.68777453	51.58342016	Uqcrb	ubiquinol-cytochrome c reductase binding protein [Source:MGI Symbol;Acc:MGI:1914780]
ENSMUSG00000021532	10.17908526	9.318945599	7.505625853	10.78828697	11.2872669	12.02716354	15.63278138	13.04473845	16.21911903	21.43825126	22.90777881	21.11553372	Fastkd3	FAST kinase domains 3 [Source:MGI Symbol;Acc:MGI:1916827]
ENSMUSG00000021534	0.792543137	0.646463366	0.473316579	0.616323528	0.346955628	0.301832884	0.530673349	0.52341793	0.430305542	0.118151854	0.247392079	0.468247487	1700001L19	RIKEN cDNA 1700001L19 gene [Source:MGI Symbol;Acc:MGI:1916565]
ENSMUSG00000021536	0.613086979	0.92956821	0.65905765	0.266990591	0.322073035	0.241271584	0.182450031	0.172082501	0.13314845	1.398399158	1.53950474	1.294943094	Adcy2	adenylate cyclase 2 [Source:MGI Symbol;Acc:MGI:99676]

ENSMUSG00000021537	64.7837877	61.00003394	60.71066414	69.39690707	66.29287018	56.00042742	78.82039386	83.93829393	76.78891938	63.28413518	56.23349778	58.96180221	Cetn3	centrin 3 [Source:MGI Symbol;Acc:MGI:1097706]
ENSMUSG00000021538	0.08027703	0	0	0	0	0	0	0	0	0	0	0	Il9	interleukin 9 [Source:MGI Symbol;Acc:MGI:96563]
ENSMUSG00000021539	0.103683103	0.059698212	0	0.048377684	0.054467853	0	0.046282957	0	0	0	0	0	Lect2	leukocyte cell-derived chemotaxin 2 [Source:MGI Symbol;Acc:MGI:1278342]
ENSMUSG00000021540	22.68010716	24.85281313	24.98237251	27.79369591	30.79407192	35.51035191	11.78830115	11.11978636	12.88285561	17.46145922	17.78430868	16.7116794	Smad5	SMAD family member 5 [Source:MGI Symbol;Acc:MGI:1328787]
ENSMUSG00000021541	0.090017757	0.069106716	0.04607976	0.014000515	0	0.114274828	0.040182902	0	0	0	0	0.013295992	Trpc7	transient receptor potential cation channel, subfamily C, member 7 [Source:MGI Symbol;Acc:MGI:1349470]
ENSMUSG00000021545	0	0	0	0	0	0	0	0	0	0	0	0	1700067P10	RIKEN cDNA 1700067P10 gene [Source:MGI Symbol;Acc:MGI:1915474]
ENSMUSG00000021546	456.4910584	508.1460964	382.0985957	546.5215826	583.9407787	615.4426464	795.5076031	742.1734475	862.4059313	784.8747256	771.8409656	778.0735233	Hnrnpk	heterogeneous nuclear ribonucleoprotein K [Source:MGI Symbol;Acc:MGI:99894]
ENSMUSG00000021548	60.27581489	61.80758995	52.14535671	67.8166254	66.67991005	63.73486477	74.54406442	82.62651271	81.51967168	69.58367198	62.24958003	68.34467364	Ccnh	cyclin H [Source:MGI Symbol;Acc:MGI:1913921]
ENSMUSG00000021549	56.35022164	76.74432428	52.33028066	74.9553746	85.66377541	104.2094481	33.16779205	31.86352688	41.54104872	130.8208526	117.650134	123.3953017	Rasa1	RAS p21 protein activator 1 [Source:MGI Symbol;Acc:MGI:97860]
ENSMUSG00000021550	6.839992329	5.002048775	7.378138266	3.685012715	2.889420543	2.481415525	9.002497758	9.80771717	7.915319033	8.073516381	8.734105328	10.06128754	2210016F16	RIKEN cDNA 2210016F16 gene [Source:MGI Symbol;Acc:MGI:1917403]
ENSMUSG00000021552	29.77467406	28.72931363	26.87225271	35.96144089	31.98517778	31.86912964	13.92082719	14.23313465	16.37903177	13.68165845	12.79394862	13.12772129	Gkap1	G kinase anchoring protein 1 [Source:MGI Symbol;Acc:MGI:1891694]
ENSMUSG00000021553	0	0	0	0	0.11953548	0.103989489	0.121887427	0.135248591	0.148251751	0.346004986	0.435636243	0.463805821	Slc28a3	solute carrier family 28 (sodium-coupled nucleoside transporter), member 3 [Source:MGI Symbol;Acc:MGI:2137361]
ENSMUSG00000021555	63.11929405	66.78412975	56.21033815	79.130436	76.05936357	81.87309692	90.95388417	85.61429713	98.11663587	90.835966	89.81301573	94.97183729	Naa35	N(alpha)-acetyltransferase 35, NatC auxiliary subunit [Source:MGI Symbol;Acc:MGI:1925939]
ENSMUSG00000021556	7.87653334	13.82986027	4.211610689	11.66215645	16.22337478	23.12149983	35.03810659	31.13405822	49.48529053	37.26723341	38.00775821	37.33810921	Golm1	golgi membrane protein 1 [Source:MGI Symbol;Acc:MGI:1917329]
ENSMUSG00000021557	13.78659767	15.85309987	12.24351552	17.29602716	18.09585042	23.09009949	17.46935885	19.24657389	22.16940567	16.64968813	17.31314341	16.37285381	Agtpbp1	ATP/GTP binding protein 1 [Source:MGI Symbol;Acc:MGI:2159437]
ENSMUSG00000021559	2.915058008	4.38477075	2.898738019	0.898084269	1.157684651	1.689163462	20.12265938	17.72737848	25.7041556	24.1781779	25.18210791	24.74621562	Dapk1	death associated protein kinase 1 [Source:MGI Symbol;Acc:MGI:1916885]
ENSMUSG00000021565	0.189924664	0.510318699	0.086419324	0.019692731	0.044343618	0.016073576	0.131880323	0.020905271	0.03928313	0.132131322	0.035131805	0.149614143	Slc6a19	solute carrier family 6 (neurotransmitter transporter), member 19 [Source:MGI Symbol;Acc:MGI:1921588]
ENSMUSG00000021566	0	0	0	0	0.092639997	0.033579939	0.039359482	0	0.041033967	0	0	0	Slc6a19os	solute carrier family 6 (neurotransmitter transporter), member 19, opposite strand [Source:MGI Symbol;Acc:MGI:1918640]
ENSMUSG00000021567	4.862104991	4.705232518	4.159841133	0.617745813	0.887378132	0.956271159	3.301435735	3.165845453	2.698271747	10.51527994	10.67855916	11.16677103	Nkd2	naked cuticle 2 [Source:MGI Symbol;Acc:MGI:1919543]
ENSMUSG00000021569	13.15442342	13.50578226	12.13324834	19.15894514	19.09082701	19.92660455	41.88316661	37.58381886	43.687903	38.86184947	36.12387727	39.28778695	Trip13	thyroid hormone receptor interactor 13 [Source:MGI Symbol;Acc:MGI:1916966]
ENSMUSG00000021572	1.925682607	2.031176082	1.905230828	3.201405529	2.703318021	3.32793741	10.14186821	7.710805846	10.16667066	7.758386602	7.206479271	7.615108082	Cep72	centrosomal protein 72 [Source:MGI Symbol;Acc:MGI:1921720]
ENSMUSG00000021573	0	0.03604912	0	0.029213152	0.049336106	0.023844322	0.293456506	0.372142723	0.276803885	0.420021459	0.208464899	0.2774311	Tppp	tubulin polymerization promoting protein [Source:MGI Symbol;Acc:MGI:1920198]
ENSMUSG00000021575	0.109934818	0.202552951	0.067530276	0.102589366	0.207907443	0.217711941	0.157035683	0.13068732	0.184181075	0.570337775	0.732077169	0.798900916	Ahrr	aryl-hydrocarbon receptor repressor [Source:MGI Symbol;Acc:MGI:1333776]
ENSMUSG00000021576	94.11669241	92.66208511	101.6351513	106.2267737	93.78563524	88.51956027	69.4407483	67.78067827	63.40107371	65.77450044	69.50565866	69.54550895	Pdcd6	programmed cell death 6 [Source:MGI Symbol;Acc:MGI:109283]
ENSMUSG00000021577	263.640177	312.0141353	234.6596518	299.9792897	320.9731967	330.4182392	375.2797417	342.4995156	380.7006894	362.0664333	360.5844195	357.193045	Sdha	succinate dehydrogenase complex, subunit A, flavoprotein (Fp) [Source:MGI Symbol;Acc:MGI:1914195]
ENSMUSG00000021578	24.786637	29.68561191	22.31125293	26.21671247	27.94430034	31.9652766	15.77308935	17.46763095	17.04357096	21.90637892	23.60262547	22.84580576	Ccdc127	coiled-coil domain containing 127 [Source:MGI Symbol;Acc:MGI:1914683]
ENSMUSG00000021579	0	0	0	0	0	0	0	0	0	0	0	0	Lrrc14b	leucine rich repeat containing 14B [Source:MGI Symbol;Acc:MGI:2145269]
ENSMUSG00000021583	15.41318967	21.90190989	12.16368102	15.81244879	19.30825996	23.36701127	1.190816372	1.345821828	1.586332638	9.721415157	9.128985484	8.931242889	Erap1	endoplasmic reticulum aminopeptidase 1 [Source:MGI Symbol;Acc:MGI:1933403]
ENSMUSG00000021585	61.56906486	83.66236776	52.67326976	92.56971152	106.2068172	132.5485512	15.44010809	13.87818297	18.01245297	43.48684221	42.46476417	42.9685385	Cast	calpastatin [Source:MGI Symbol;Acc:MGI:1098236]
ENSMUSG00000021587	0	0	0.114520581	0.069590083	0.01958766	0.028400365	0.166442175	0.424780978	0.381751011	1.183988567	1.241486152	1.156543903	Pcsk1	proprotein convertase subtilisin/kexin type 1 [Source:MGI Symbol;Acc:MGI:97511]
ENSMUSG00000021589	39.35532423	42.3271414	29.39374883	42.135502	45.60211903	57.74470303	8.722536552	7.921179596	11.07049717	16.42779052	16.47326677	16.01050528	Rhobtb3	Rho-related BTB domain containing 3 [Source:MGI Symbol;Acc:MGI:1920546]
ENSMUSG00000021590	1.571122642	0.992157557	1.141518851	1.37155578	1.650715982	1.968757423	0.497718957	0.90372822	1.084959346	0.770665993	0.67499412	0.718641313	Spata9	spermatogenesis associated 9 [Source:MGI Symbol;Acc:MGI:1922821]
ENSMUSG00000021591	36.53932669	39.21107615	40.05182964	24.83004251	22.92183925	22.16232423	11.89822237	11.77395036	10.71585406	46.58084146	50.9180446	50.23237228	Glrx	glutaredoxin [Source:MGI Symbol;Acc:MGI:2135625]
ENSMUSG00000021592	10.14753119	10.74943699	8.573420187	10.36589496	10.14405312	10.37437516	2.132936946	2.220350395	2.063207577	4.780703321	4.674440532	5.020359518	Arsk	arylsulfatase K [Source:MGI Symbol;Acc:MGI:1924291]
ENSMUSG00000021594	4.680291658	5.285281667	5.409925463	6.086421903	4.949121167	4.576863233	5.364600674	3.948499576	4.412432834	4.483510469	4.725314098	4.629468429	Srd5a1	steroid 5 alpha-reductase 1 [Source:MGI Symbol;Acc:MGI:98400]
ENSMUSG00000021595	85.94797379	82.12228144	77.3355526	85.0368777	83.74561822	83.83424278	245.7431545	235.88642	244.479906	213.2072463	212.7009762	218.243863	Nsun2	NOL1/NOP2/Sun domain family member 2 [Source:MGI Symbol;Acc:MGI:107252]
ENSMUSG00000021596	2.169684109	3.438533536	1.935395915	3.167373688	4.784904388	4.745115319	0.095893295	0.170248019	0.039989168	0.84546706	1.019252073	0.723440029	Mctp1	multiple C2 domains, transmembrane 1 [Source:MGI Symbol;Acc:MGI:1926021]
ENSMUSG00000021597	6.194980984	7.876274796	4.70655963	8.187647272	10.74986071	14.11311247	8.233542013	7.025628318	9.210000318	12.61201764	13.04340938	13.86199402	Slf1	SMC5-SMC6 complex localization factor 1 [Source:MGI Symbol;Acc:MGI:2145448]
ENSMUSG00000021598	72.46723049	59.64074554	75.80351406	69.38214309	61.93215927	56.18776012	41.50148912	43.13450937	41.25986573	33.40522777	34.64862417	34.31051329	Med10	mediator complex subunit 10 [Source:MGI Symbol;Acc:MGI:106331]
ENSMUSG00000021600	0	0	0	0	0	0	0	0	0	0	0	0	8030423J24I	RIKEN cDNA 8030423J24 gene [Source:MGI Symbol;Acc:MGI:1924416]
ENSMUSG00000021604	0	0.019923408	0	0.016145347	0	0	0	0.137115714	0.048310195	0.12380528	0.028803277	0.076664465	Irx4	Iroquois homeobox 4 [Source:MGI Symbol;Acc:MGI:1355275]
ENSMUSG00000021606	62.1119487	43.4498282	84.3158583	49.09152012	49.32509607	31.66865738	123.4716895	119.0360941	67.46897533	62.8918034	48.92355673	51.63696874	Ndufs6	NADH:ubiquinone oxidoreductase core subunit S6 [Source:MGI Symbol;Acc:MGI:107932]
ENSMUSG00000021607	63.40161402	46.47270223	61.7757299	48.30884138	43.74621232	32.98601301	70.12077503	68.212385	58.70076155	42.76993934	45.0838549	47.90045337	Mrpl36	mitochondrial ribosomal protein L36 [Source:MGI Symbol;Acc:MGI:2137228]
ENSMUSG00000021608	45.97972129	49.03545725	46.76901721	56.28429861	58.02819883	61.3355879	41.0496481	42.23275631	45.81992264	46.05246005	42.69123832	42.81435062	Lpcat1	lysophosphatidylcholine acyltransferase 1 [Source:MGI Symbol;Acc:MGI:2384812]
ENSMUSG00000021609	0	0	0.050986401	0	0	0	0.255654411	0.074003208	0.115882962	0.044546257	0.114000472	0	Slc6a3	solute carrier family 6 (neurotransmitter transporter, dopamine), member 3 [Source:MGI Symbol;Acc:MGI:94862]
ENSMUSG00000021610	168.8665183	165.8973877	154.8478153	144.4388783	135.6676044	136.3213745	56.02770226	53.2018835	56.4088521	82.05924906	82.10849756	74.93475516	Clptm1l	CLPTM1-like [Source:MGI Symbol;Acc:MGI:2442892]
ENSMUSG00000021611	1.707637265	2.355145413	1.727937825	0.85235809	1.335178761	1.9812629	6.594520404	7.002677333	8.261177079	10.74537132	11.98303402	12.07160711	Tert	telomerase reverse transcriptase [Source:MGI Symbol;Acc:MGI:1202709]
ENSMUSG00000021612	0.169236924	0.876982577	0.04331588	0.138187977	0.044452624	0.016113088	0.037772718	0.230523267	0.078759393	0.018922304	0	0.018747741	Slc6a18	solute carrier family 6 (neurotransmitter transporter), member 18 [Source:MGI Symbol;Acc:MGI:1336892]
ENSMUSG00000021613	0.027850154	0.048106326	0.028512783	0.077967918	0	0.021212958	0	0.055179091	0	0.012455653	0.023182444	0.049362983	Hapln1	hyaluronan and proteoglycan link protein 1 [Source:MGI Symbol;Acc:MGI:1337006]
ENSMUSG00000021614	45.46510747	62.16327906	38.96101202	58.73673476	72.01912027	100.0598864	3.065277931	2.666172713	3.494636388	31.19655241	30.5250128	30.41798498	Vcan	versican [Source:MGI Symbol;Acc:MGI:102889]
ENSMUSG00000021615	9.327854005	8.905052808	7.516607726	12.21451948	10.36946043	9.534260549	4.835436177	3.904885349	4.649062345	4.33326055	4.082622869	4.133286872	Xrcc4	X-ray repair complementing defective repair in Chinese hamster cells 4 [Source:MGI Symbol;Acc:MGI:1333799]
ENSMUSG00000021619	8.140524724	8.035056588	7.024548005	6.800769219	5.620493633	5.75759822	3.737661477	3.379346484	3.247228502	3.398036671	3.484885182	4.156830151	Atg10	autophagy related 10 [Source:MGI Symbol;Acc:MGI:1914045]
ENSMUSG00000021620	0	0.100100032	0	0.054078758	0	0.044140095	0	0	0.080907377	0	0.072357324	0	Acot12	acyl-CoA thioesterase 12 [Source:MGI Symbol;Acc:MGI:1921406]
ENSMUSG00000021621	20.49404267	21.90682908	16.86519202	22.59683167	25.06318904	25.43757669	21.61840114	22.88835616	22.14712494	18.33143726	17.23406142	16.67317381	Zcchc9	zinc finger, CCHC domain containing 9 [Source:MGI Symbol;Acc:MGI:1916335]
ENSMUSG00000021622	0	0	0.029644852	0	0.030422825	0	0	0	0	0	0	0.025661443	Ckmt2	creatine kinase, mitochondrial 2 [Source:MGI Symbol;Acc:MGI:1923972]
ENSMUSG00000021624	0.691220688	1.23376304	0.672283336	0	0	0.026324515	0	0	0.032167993	1.452959373	1.869957354	2.174647577	Cd180	CD180 antigen [Source:MGI Symbol;Acc:MGI:1194924]
ENSMUSG00000021629	26.60474447	28.81476628	24.20937011	23.43088635	25.65779858	27.35098125	26.51576858	25.16322025	27.91595534	27.93531052	30.43432345	29.15597389	Slc30a5	solute carrier family 30 (zinc transporter), member 5 [Source:MGI Symbol;Acc:MGI:1916298]
ENSMUSG00000021631	0.703079866	0.161926522	0.71980801	0.262441032	0.59095841	0.214209285	0.753232433	0.975101091	0.916157061	0.377333004	0.117048613	0.373852007	Mrps36-ps1	mitichondrial ribosomal protein S36, pseudogene 1 [Source:MGI Symbol;Acc:MGI:3809201]
ENSMUSG00000021635	12.20473465	12.67288727	10.21881194	13.43424909	13.14234385	14.83968667	16.6797582	16.622496	18.53575972	18.68597921	17.97501186	19.09480395	Rad17	RAD17 checkpoint clamp loader component [Source:MGI Symbol;Acc:MGI:1333807]
ENSMUSG00000021636	0.587820872	1.005688842	1.066057577	0.297781452	0.617599977	0.46052488	1.574379265	1.197916095	1.610094694	1.772669802	1.593725138	1.667012227	Marveld2	MARVEL (membrane-associating) domain containing 2 [Source:MGI Symbol;Acc:MGI:2446166]
ENSMUSG00000021638	1.308360872	1.633966896	1.150829663	1.582064223	1.606976627	1.824677808	2.829702313	2.555725344	3.378879165	4.45042866	3.988174572	4.033759083	Ocln	occludin [Source:MGI Symbol;Acc:MGI:106183]
ENSMUSG00000021639	28.08864738	26.76176884	27.01275038	35.95468777	30.93442291	30.31180995	40.55984592	42.53929776	43.26121545	45.01333732	39.67128035	41.81069753	Gtf2h2	general transcription factor II H, polypeptide 2 [Source:MGI Symbol;Acc:MGI:1345669]
ENSMUSG00000021640	0.059335998	0.159433054	0.040498506	0.101514194	0.259758207	0.203378202	0.008828971	0	0	0.061920424	0.057623105	0.157755064	Naip1	NLR family, apoptosis inhibitory protein 1 [Source:MGI Symbol;Acc:MGI:1298223]
ENSMUSG00000021643	8.462003172	8.549359839	15.03824497	7.971098568	7.129326641	7.47904367	1.567953227	1.423497697	0.297210772	2.927656069	1.594814493	1.768687602	Serf1	small EDRK-rich factor 1 [Source:MGI Symbol;Acc:MGI:1337114]
ENSMUSG00000021645	65.00706794	52.31216847	67.97991128	69.3297459	61.55161329	56.88296747	148.408465	160.0002593	137.8670432	109.7183933	102.6192134	109.1177183	Smn1	survival motor neuron 1 [Source:MGI Symbol;Acc:MGI:109257]
ENSMUSG00000021646	14.16180328	13.46977124	15.01198041	12.31893106	11.49959166	11.29590032	29.38922816	29.50701884	30.36736635	30.04303394	33.83477654	35.17112568	Mccc2	methylcrotonoyl-Coenzyme A carboxylase 2 (beta) [Source:MGI Symbol;Acc:MGI:1925288]
ENSMUSG00000021647	0.166615635	0.095933235	0.085289933	0.038870743	0.043764103	0	0	0	0	0	0	0	Cartpt	CART prepropeptide [Source:MGI Symbol;Acc:MGI:1351330]

ENSMUSG00000021650	19.74664171	15.93493812	18.79459266	18.66288406	18.78373791	18.13726612	31.40369232	31.5441279	32.12863271	23.01598458	26.14800064	23.36311766	Ptcd2	pentatricopeptide repeat domain 2 [Source:MGI Symbol;Acc:MGI:1916177]
ENSMUSG00000021660	437.6715214	343.2181334	516.093529	407.222786	364.8143048	286.0743146	651.484566	751.0950691	527.4660761	372.7742049	355.6749027	349.2845892	Btf3	basic transcription factor 3 [Source:MGI Symbol;Acc:MGI:1202875]
ENSMUSG00000021661	15.34857645	18.24872405	13.99464778	19.19532111	18.77247853	18.40541037	13.70285953	11.85206497	14.50560732	11.24130237	11.40024939	11.25385724	Ankra2	ankyrin repeat, family A (RFXANK-like), 2 [Source:MGI Symbol;Acc:MGI:1915808]
ENSMUSG00000021662	4.895766102	6.336863901	4.641951312	4.809738114	4.356610684	4.919546778	1.222448073	1.394841609	1.875613221	8.619640622	8.494545671	8.265372917	Arhgef28	Rho guanine nucleotide exchange factor (GEF) 28 [Source:MGI Symbol;Acc:MGI:1346016]
ENSMUSG00000021665	31.91170526	45.9726129	18.77911274	38.50584273	48.40313112	57.23001476	29.94957468	23.02961832	33.5974645	35.35862564	34.21110371	36.74203886	Hexb	hexosaminidase B [Source:MGI Symbol;Acc:MGI:96074]
ENSMUSG00000021666	11.55746268	12.68417613	10.62883212	12.97941339	14.11928564	14.56163927	39.39985033	35.8530144	43.4160411	32.95501786	34.17583327	33.93331619	Gfm2	G elongation factor, mitochondrial 2 [Source:MGI Symbol;Acc:MGI:2444783]
ENSMUSG00000021668	15.03718712	22.61336946	11.88499942	20.3703473	28.06426621	36.52576178	11.21643999	12.53195623	13.18814722	22.32030324	21.71048125	20.88367851	Polk	polymerase (DNA directed), kappa [Source:MGI Symbol;Acc:MGI:1349767]
ENSMUSG00000021669	18.52664554	21.04938338	15.0038272	22.02337424	25.09303126	28.69409942	11.58443011	11.90861809	16.43759341	22.12188371	22.51430546	23.24947845	Col4a3bp	collagen, type IV, alpha 3 (Goodpasture antigen) binding protein [Source:MGI Symbol;Acc:MGI:1915268]
ENSMUSG00000021670	37.87001154	46.41932368	30.10606044	56.46853057	65.23239044	75.33554492	95.88546131	90.11710085	114.799901	66.55959152	70.55384516	69.14839716	Hmgcr	3-hydroxy-3-methylglutaryl-Coenzyme A reductase [Source:MGI Symbol;Acc:MGI:96159]
ENSMUSG00000021671	8.790497803	9.691344611	9.408722039	12.42124416	12.27945037	13.42916922	15.67361719	13.18605736	16.20096263	15.10032807	17.46150633	15.97908136	Poc5	POC5 centriolar protein [Source:MGI Symbol;Acc:MGI:1914713]
ENSMUSG00000021675	0.034395274	0.178235668	0.088034073	0.096291314	0.054206617	0	0.046060976	0.068146839	0.06402742	0.230743204	0.14315298	0.137168722	F2rl2	coagulation factor II (thrombin) receptor-like 2 [Source:MGI Symbol;Acc:MGI:1298208]
ENSMUSG00000021676	6.496184039	9.853596857	6.150151813	5.47547425	6.678501574	8.007289133	21.70189808	21.97030299	28.28146252	34.67665801	35.2354327	32.62344281	Iqgap2	IQ motif containing GTPase activating protein 2 [Source:MGI Symbol;Acc:MGI:2449975]
ENSMUSG00000021678	2.009356601	1.446173386	1.012510676	0.805706414	1.055575474	1.578318396	45.47836609	39.126827	50.46689871	26.86153946	25.78135546	25.55641952	F2rl1	coagulation factor II (thrombin) receptor-like 1 [Source:MGI Symbol;Acc:MGI:101910]
ENSMUSG00000021679	0	0.248368499	0.331219926	0	0	0.164280805	0	0	0	0.192922137	0	0.191142379	S100z	S100 calcium binding protein, zeta [Source:MGI Symbol;Acc:MGI:2685471]
ENSMUSG00000021680	0.37093524	0.170860399	0.126586926	0.32307396	0.155890753	0.527398033	0.176620019	0.073492841	0.161117276	0.331792814	0.473441457	0.306816475	Crhbp	corticotropin releasing hormone binding protein [Source:MGI Symbol;Acc:MGI:88497]
ENSMUSG00000021681	19.94364484	22.45425708	19.56866486	21.04462578	22.55745637	23.09078727	33.3484322	31.48500114	37.40127374	37.69276045	38.25893517	39.05208628	Aggf1	angiogenic factor with G patch and FHA domains 1 [Source:MGI Symbol;Acc:MGI:1913799]
ENSMUSG00000021684	0.644387982	1.5319657	0.532032007	0.581934461	0.873590693	0.585815828	0.575294916	0.535397121	0.580422486	0.966847464	1.159290173	0.865819575	Pde8b	phosphodiesterase 8B [Source:MGI Symbol;Acc:MGI:2443999]
ENSMUSG00000021685	0.025734742	0.059269756	0.02634704	0	0.054076936	0	0.114876956	0	0	0.115095593	0.042843152	0	Otp	orthopedia homeobox [Source:MGI Symbol;Acc:MGI:99835]
ENSMUSG00000021686	67.38313632	78.19899859	60.15801533	73.64642617	86.95790957	92.57495877	59.68997298	58.00809335	71.58413001	84.3764625	86.37759679	83.77879195	Ap3b1	adaptor-related protein complex 3, beta 1 subunit [Source:MGI Symbol;Acc:MGI:1333879]
ENSMUSG00000021687	30.20372136	31.93630787	26.52372129	33.03691037	30.83103488	37.89547269	13.90452781	11.87479931	14.43608957	21.78999194	21.97650522	20.96072295	Scamp1	secretory carrier membrane protein 1 [Source:MGI Symbol;Acc:MGI:1349480]
ENSMUSG00000021690	4.99162872	7.241134487	4.355448466	6.072173767	7.134570768	9.92478817	3.769141049	3.33942611	4.502701216	7.530884569	7.392711022	7.741482346	Jmy	junction-mediating and regulatory protein [Source:MGI Symbol;Acc:MGI:1913096]
ENSMUSG00000021692	1.873004764	2.069420947	1.425219859	13.65218246	2.207229661	3.084613707	3.773694488	3.986770461	4.829456507	3.418637013	4.382300059	22.00492912	Dimt1	DIM1 dimethyladenosine transferase 1-like (S. cerevisiae) [Source:MGI Symbol;Acc:MGI:1913504]
ENSMUSG00000021693	32.92521258	34.42886827	27.61977052	36.62625726	41.18059973	46.63680372	38.9905971	34.67564796	42.43342657	49.62415981	45.64340376	46.91204553	Kif2a	kinesin family member 2A [Source:MGI Symbol;Acc:MGI:108390]
ENSMUSG00000021694	4.769785699	5.984101362	5.063181615	5.224168937	6.25109186	5.124525981	16.6075886	16.26447164	17.47769273	12.75447194	12.28723616	12.30309002	Ercc8	excision repaiross-complementing rodent repair deficiency, complementation group 8 [Source:MGI Symbol;Acc:MGI:1919241]
ENSMUSG00000021696	0.067612332	0.218005411	0.055376807	0.176665207	0.184697718	0.092698361	2.752541398	2.933708563	2.995505362	2.019952981	1.553340294	1.617832223	Elovl7	ELOVL family member 7, elongation of long chain fatty acids (yeast) [Source:MGI Symbol;Acc:MGI:1921809]
ENSMUSG00000021697	3.795668888	3.391221602	3.182481953	3.786335553	4.366126563	5.233874018	21.00401527	20.51875406	21.53212937	19.34642264	18.73917135	18.32699298	Depdc1b	DEP domain containing 1B [Source:MGI Symbol;Acc:MGI:2145425]
ENSMUSG00000021699	3.703833703	4.396795488	3.417443427	2.730948546	3.795384856	5.973161041	6.164338964	5.209322514	6.830912667	12.90430753	11.60908291	12.82578569	Pde4d	phosphodiesterase 4D, cAMP specific [Source:MGI Symbol;Acc:MGI:99555]
ENSMUSG00000021700	1.256608598	1.399344472	1.20168205	0.309269406	0.130576016	0.105179784	0.024656517	0.013679669	0.012852744	0.358199944	0.218395582	0.244755486	Rab3c	RAB3C, member RAS oncogene family [Source:MGI Symbol;Acc:MGI:1914545]
ENSMUSG00000021701	144.9978854	164.4794613	134.3178044	208.4697859	215.0096248	238.5082974	35.49145952	35.6555833	44.86008733	109.3397133	113.239895	110.7533043	Plk2	polo like kinase 2 [Source:MGI Symbol;Acc:MGI:1099790]
ENSMUSG00000021702	0.355999624	0.191310871	0.048595974	0.177180266	0.024935642	0.108463389	0.021188557	0	0	0.509493212	0.671689888	0.546859085	Thbs4	thrombospondin 4 [Source:MGI Symbol;Acc:MGI:1101779]
ENSMUSG00000021703	13.92733596	15.98062787	11.45120293	13.94282602	19.94125128	22.63420197	4.955798092	4.741693371	5.661641351	12.27928166	12.69984229	11.81251045	Serinc5	serine incorporator 5 [Source:MGI Symbol;Acc:MGI:2444223]
ENSMUSG00000021704	7.348174858	8.357119008	5.909826523	10.12320075	11.04738484	13.67507551	2.231848916	2.716647336	2.580631947	7.30458235	7.049870913	7.666862175	Mtx3	metaxin 3 [Source:MGI Symbol;Acc:MGI:2686040]
ENSMUSG00000021706	8.802702997	11.28907932	6.162832967	11.54120675	12.23844374	17.07774186	4.606432356	3.701881395	5.835650349	8.880762589	8.602817839	9.283810371	Zfyve16	zinc finger, FYVE domain containing 16 [Source:MGI Symbol;Acc:MGI:2145181]
ENSMUSG00000021707	6.317068065	5.902943978	5.874182605	8.358111246	8.666266375	8.342143648	16.25824103	17.17151224	15.93127834	11.73281937	11.19319224	10.01282928	Dhfr	dihydrofolate reductase [Source:MGI Symbol;Acc:MGI:94890]
ENSMUSG00000021708	0.153917484	0.295406492	0.262632652	0.255348031	0.539049901	0.40381345	0.09160935	0.135535287	0	0.275351111	0.384362336	0.227342437	Rasgrf2	RAS protein-specific guanine nucleotide-releasing factor 2 [Source:MGI Symbol;Acc:MGI:109137]
ENSMUSG00000021709	17.48254003	24.48884286	14.86430571	24.88353462	29.780017	39.16192281	3.419970522	3.107728043	4.372466442	9.257134978	8.699977447	8.759601114	Erbin	Erbb2 interacting protein [Source:MGI Symbol;Acc:MGI:1890169]
ENSMUSG00000021710	31.53792574	34.14975139	33.58984078	37.62107099	40.91866541	45.52981844	36.01392569	36.69213595	40.98211471	42.55544464	45.70721723	43.59284331	Nln	neurolysin (metallopeptidase M3 family) [Source:MGI Symbol;Acc:MGI:1923055]
ENSMUSG00000021711	23.52559226	25.39516076	23.84631407	25.18809565	24.88721699	23.19897287	20.74658646	22.8251096	22.84941157	21.25191131	20.82341717	20.88078921	Trappc13	trafficking protein particle complex 13 [Source:MGI Symbol;Acc:MGI:1914225]
ENSMUSG00000021712	5.856016124	7.962368687	5.229732376	8.43867208	10.92735823	12.11061404	6.809905949	6.713001149	8.780830013	12.15355302	11.18559106	12.4288799	Trim23	tripartite motif-containing 23 [Source:MGI Symbol;Acc:MGI:1933161]
ENSMUSG00000021713	9.627871597	9.864311768	8.180342571	11.80589499	11.77949909	12.29539796	25.3528397	25.54698442	30.81992422	21.89194239	19.93821048	20.86066053	Ppwd1	peptidylprolyl isomerase domain and WD repeat containing 1 [Source:MGI Symbol;Acc:MGI:2443069]
ENSMUSG00000021714	7.976760667	6.226810787	8.480647097	11.05831074	11.3222259	10.5449389	24.92514596	29.74691511	22.63026922	13.00083713	12.03472554	11.07961402	Cenpk	centromere protein K [Source:MGI Symbol;Acc:MGI:1926210]
ENSMUSG00000021715	21.86204924	19.22475433	23.39957863	22.34870244	21.45567996	18.5820371	47.68587544	51.33805907	44.53502646	28.66597957	28.6204132	26.99246025	Cwc27	CWC27 spliceosome-associated protein [Source:MGI Symbol;Acc:MGI:1914535]
ENSMUSG00000021716	14.76944526	14.97391668	17.58421171	16.26681642	16.05554197	16.68920979	11.31192907	13.00836399	12.15054567	10.23445626	11.03696698	11.31964274	Srek1ip1	splicing regulatory glutamine/lysine-rich protein 1interacting protein 1 [Source:MGI Symbol;Acc:MGI:1914538]
ENSMUSG00000021718	0.598449066	0.390515422	0.306343882	0.223385134	0.335342185	0.638158955	0	0	0	0	0.016604949	0.035357353	Nt5el	5' nucleotidase, ecto-like [Source:MGI Symbol;Acc:MGI:1914013]
ENSMUSG00000021719	0.109440834	0.049010401	0.080921189	0.011347599	0.057492563	0.055572735	0.010856254	0.012046304	0.005659058	0.168592194	0.33908869	0.3232972	Rgs7bp	regulator of G-protein signalling 7 binding protein [Source:MGI Symbol;Acc:MGI:106334]
ENSMUSG00000021720	0.497340035	0.750451014	0.491615387	0.736173767	0.8288492	0.548628484	0.015310823	0.118924246	0.255395147	0.85903791	0.713767945	0.562342556	Rnf180	ring finger protein 180 [Source:MGI Symbol;Acc:MGI:1919066]
ENSMUSG00000021721	0	0	0	0	0	0.007309104	0	0	0.017863136	0	0	0	Htr1a	5-hydroxytryptamine (serotonin) receptor 1A [Source:MGI Symbol;Acc:MGI:96273]
ENSMUSG00000021725	23.83431612	26.43785329	21.08173948	24.32859051	25.2375584	27.664169	0.499110274	0.387675531	0.780516162	6.034059691	6.297813532	5.383857483	Parp8	poly (ADP-ribose) polymerase family, member 8 [Source:MGI Symbol;Acc:MGI:1098713]
ENSMUSG00000021728	71.14157834	62.27480706	63.68964716	58.42691685	54.65303689	55.38656847	58.75964705	57.82708148	58.83468168	59.89577734	59.26584785	59.44470771	Emb	embigin [Source:MGI Symbol;Acc:MGI:95321]
ENSMUSG00000021730	1.176770753	1.660270826	1.053023517	1.307712431	1.665817993	2.045810474	0.020049506	0.004449462	0.016721984	0.188824052	0.205629243	0.163199279	Hcn1	hyperpolarization activated cyclic nucleotide gated potassium channel 1 [Source:MGI Symbol;Acc:MGI:1096392]
ENSMUSG00000021731	10.22243771	7.346853201	12.01200083	9.967895087	8.518613202	7.90589265	13.48457388	13.78965924	11.46028795	9.694946879	10.24919857	10.15164101	Mrps30	mitochondrial ribosomal protein S30 [Source:MGI Symbol;Acc:MGI:1926237]
ENSMUSG00000021732	19.076792	20.24942633	14.25968793	4.409919531	4.625359739	5.228687685	0.954820072	0.615980458	1.088040533	12.72550102	11.44897812	12.34359921	Fgf10	fibroblast growth factor 10 [Source:MGI Symbol;Acc:MGI:1099809]
ENSMUSG00000021733	5.596507768	6.908008412	4.206746477	7.213744672	9.326057171	12.99259135	14.67362519	14.51863671	20.07564439	26.72018068	25.78936826	26.12790126	Slc4a7	solute carrier family 4, sodium bicarbonate cotransporter, member 7 [Source:MGI Symbol;Acc:MGI:2443878]
ENSMUSG00000021737	154.5723615	148.4703112	157.2992819	154.8845782	151.0196342	142.2958378	208.7944047	210.7270624	195.011516	185.7944276	191.0662818	182.5258459	Psmd6	proteasome (prosome, macropain) 26S subunit, non-ATPase, 6 [Source:MGI Symbol;Acc:MGI:1913663]
ENSMUSG00000021738	6.802467261	8.125483269	6.421335506	7.466911473	8.576497693	13.19040166	2.7792097	2.44424743	3.176460035	5.981516746	6.756452395	6.907246401	Atxn7	ataxin 7 [Source:MGI Symbol;Acc:MGI:2179277]
ENSMUSG00000021741	0.129214678	0.148797344	0.396867119	0.120581015	0.271521432	0.098420482	0.115359922	0	0.601338611	0.115579479	0.107558185	0	Gm5457	predicted pseudogene 5457 [Source:MGI Symbol;Acc:MGI:3646665]
ENSMUSG00000021743	0	0	0	0	0	0	0	0	0	0.017671243	0	0.035016441	Fezf2	Fez family zinc finger 2 [Source:MGI Symbol;Acc:MGI:1859823]
ENSMUSG00000021745	11.93801321	17.21028005	9.438657208	16.11525037	19.05355881	26.23772637	8.162986993	7.116846439	10.50353674	21.64183919	21.42944015	21.3479657	Ptprg	protein tyrosine phosphatase, receptor type, G [Source:MGI Symbol;Acc:MGI:97814]
ENSMUSG00000021747	0.322374317	0.468922861	0.347415222	0.696669174	0.445665599	0.762487073	11.5426336	11.07666941	12.99702354	4.765465868	4.674836665	5.02223587	4930452B06	RIKEN cDNA 4930452B06 gene [Source:MGI Symbol;Acc:MGI:1926154]
ENSMUSG00000021748	89.32924702	79.26189999	79.63291382	89.08175415	84.09379497	83.02369913	183.3436217	164.0573491	169.893822	131.8145562	122.0403677	125.5652552	Pdhb	pyruvate dehydrogenase (lipoamide) beta [Source:MGI Symbol;Acc:MGI:1915513]
ENSMUSG00000021749	0.046069045	0.212203493	0.04716515	0.128972627	0.193611642	0.140359831	0.287905759	0.228189763	0.257275063	0.329661382	0.191739162	0.489930253	Oit1	oncoprotein induced transcript 1 [Source:MGI Symbol;Acc:MGI:1201784]
ENSMUSG00000021750	0.057142746	0.197408429	0.019500775	0.088874453	0.080050143	0.159590185	0	0	0	0.034075225	0.015855191	0.033760872	Fam107a	family with sequence similarity 107, member A [Source:MGI Symbol;Acc:MGI:3041256]
ENSMUSG00000021751	0	0.56257092	0.230841486	0.03506854	0.078966495	0.057247198	0	0.297822517	0	0.033613944	0.031281114	0	Acox2	acyl-Coenzyme A oxidase 2, branched chain [Source:MGI Symbol;Acc:MGI:1934852]
ENSMUSG00000021752	4.026485462	4.931768037	3.747532982	5.499540563	6.326472453	5.57618386	11.81367056	10.82417315	11.65190117	6.040859035	5.667323555	6.309527828	Kctd6	potassium channel tetramerisation domain containing 6 [Source:MGI Symbol;Acc:MGI:1918643]
ENSMUSG00000021754	6.293440798	6.737230998	5.154542818	8.809403317	9.967381131	13.0492444	13.75526465	11.75328087	15.53369041	19.18143503	19.92628988	20.11996163	Map3k1	mitogen-activated protein kinase kinase kinase 1 [Source:MGI Symbol;Acc:MGI:1346872]
ENSMUSG00000021756	65.31379741	82.00218503	56.01724999	63.78561957	76.04083624	106.9243786	22.93283101	21.23803656	29.10107005	42.17989006	45.59541105	41.93815589	Il6st	interleukin 6 signal transducer [Source:MGI Symbol;Acc:MGI:96560]
ENSMUSG00000021758	7.765099466	11.19026124	7.247945564	13.35203974	16.09773165	16.80136839	4.045069497	3.381128896	4.716560969	5.079291717	5.123785789	4.847515075	Ddx4	DEAD (Asp-Glu-Ala-Asp) box polypeptide 4 [Source:MGI Symbol;Acc:MGI:102670]

ENSMUSG00000021759	22.24887663	26.41489857	16.88413771	25.63107017	27.66055994	26.40192882	6.853102887	6.445859896	6.837660016	10.21875623	10.7076234	10.70910146	Plpp1	phospholipid phosphatase 1 [Source:MGI Symbol;Acc:MGI:108412]
ENSMUSG00000021760	226.6831925	212.0894278	228.1867662	196.335438	190.4138442	157.6842781	10.8121278	14.49090628	9.813368254	43.79811626	42.49794828	42.93108475	Gpx8	glutathione peroxidase 8 (putative) [Source:MGI Symbol;Acc:MGI:1916840]
ENSMUSG00000021763	1.086055582	1.417402009	1.291916953	0.656354926	0.815053816	0.976917923	2.484037439	2.530909707	3.533182358	2.535024708	3.220074863	2.667470016	BC067074	cDNA sequence BC067074 [Source:MGI Symbol;Acc:MGI:3040697]
ENSMUSG00000021764	92.76543661	72.00069688	119.0108294	95.1260833	83.42338891	58.40242654	112.4371429	112.8209099	78.09828452	68.11573098	67.53291503	67.06932323	Ndufs4	NADH:ubiquinone oxidoreductase core subunit S4 [Source:MGI Symbol;Acc:MGI:1343135]
ENSMUSG00000021765	47.57233631	51.34185516	41.30219724	43.90176048	45.10281469	44.45717502	1.389568817	0.969899685	1.144929069	26.1601405	27.79255799	27.0089535	Fst	follistatin [Source:MGI Symbol;Acc:MGI:95586]
ENSMUSG00000021767	5.181615218	7.155164711	3.510094201	6.875170006	8.26526501	12.28814492	30.18309963	23.87399206	40.1937333	54.74461643	55.44319327	53.07927269	Kat6b	K(lysine) acetyltransferase 6B [Source:MGI Symbol;Acc:MGI:1858746]
ENSMUSG00000021768	0.216223557	0.298791049	0.442736183	0.040355254	0	0.131754857	0.077215787	0.342720384	0.040250404	0.502857852	0.647943476	0.919788656	Dusp13	dual specificity phosphatase 13 [Source:MGI Symbol;Acc:MGI:1351599]
ENSMUSG00000021770	15.66571159	21.49982717	13.55453852	18.84724482	22.08083659	25.95731806	13.68475384	14.83468635	18.21503605	20.50871098	22.24177144	21.72920558	Samd8	sterile alpha motif domain containing 8 [Source:MGI Symbol;Acc:MGI:1914880]
ENSMUSG00000021771	338.8332862	322.0472001	312.4860614	333.0541206	310.7903199	291.5420343	427.7235991	448.7801935	423.8834077	347.0044411	338.9238665	346.573086	Vdac2	voltage-dependent anion channel 2 [Source:MGI Symbol;Acc:MGI:106915]
ENSMUSG00000021772	13.02950997	10.14869597	17.90396675	12.06216925	10.82094688	9.266229128	10.70679065	12.15435818	9.425222005	6.646516281	7.911358038	7.810353972	Nkiras1	NFKB inhibitor interacting Ras-like protein 1 [Source:MGI Symbol;Acc:MGI:1916971]
ENSMUSG00000021773	1.160840499	0.623824837	1.743074648	0.54163864	0.528514513	0.707352963	0.656368909	0.498323759	0.36015424	0.380726286	0.515350726	0.480090527	Comtd1	catechol-O-methyltransferase domain containing 1 [Source:MGI Symbol;Acc:MGI:1916406]
ENSMUSG00000021774	9.21488225	9.59022883	7.934141837	12.23313841	13.65161585	13.94951595	14.28509358	12.22246529	13.09851282	13.69150775	11.87477054	14.93197074	Ube2e1	ubiquitin-conjugating enzyme E2E 1 [Source:MGI Symbol;Acc:MGI:107411]
ENSMUSG00000021775	17.79190305	22.55498034	13.587692	23.09548648	26.67802926	37.75256651	9.217982491	8.165198427	12.18797557	18.37195923	19.18102497	19.53771378	Nr1d2	nuclear receptor subfamily 1, group D, member 2 [Source:MGI Symbol;Acc:MGI:2449205]
ENSMUSG00000021779	0.16501817	0.308793722	0.253416588	0.173241275	0.086689009	0.314228386	0.036831116	0.245211013	0.28798527	0.424363963	0.669634776	0.237645139	Thrb	thyroid hormone receptor beta [Source:MGI Symbol;Acc:MGI:98743]
ENSMUSG00000021782	15.3682176	21.06544013	14.30117	15.83550576	20.29274647	25.74484305	17.13079634	14.07539712	20.58195481	26.52320448	28.59683143	27.59691608	Dlg5	discs large MAGUK scaffold protein 5 [Source:MGI Symbol;Acc:MGI:1918478]
ENSMUSG00000021785	13.47130319	14.50929103	11.59940616	15.45258262	17.05776827	17.71466162	5.001934124	5.204892283	5.70144171	5.991471617	5.617114152	5.980334011	Ngly1	N-glycanase 1 [Source:MGI Symbol;Acc:MGI:1913276]
ENSMUSG00000021786	2.33557554	3.392952218	2.55668592	3.70518522	4.209375779	5.008472465	3.512673504	4.040111661	3.862778289	3.101535495	3.768623224	3.072923008	Oxsm	3-oxoacyl-ACP synthase, mitochondrial [Source:MGI Symbol;Acc:MGI:1918397]
ENSMUSG00000021789	0	0	0	0	0	0.017687005	0	0	0	0	0	0	Sftpa1	surfactant associated protein A1 [Source:MGI Symbol;Acc:MGI:109518]
ENSMUSG00000021790	0.045921545	0	0	0.042853231	0.096495877	0.03497761	0	0	0	0.246454427	0.114675162	0.162787213	Dydc1	DPY30 domain containing 1 [Source:MGI Symbol;Acc:MGI:1916746]
ENSMUSG00000021791	2.524899041	2.362385219	2.584973117	0.785398099	0.552668928	0.681122678	0.187847697	0.260549191	0.293759058	1.03512868	0.744360493	1.025579344	Dydc2	DPY30 domain containing 2 [Source:MGI Symbol;Acc:MGI:1918450]
ENSMUSG00000021792	8.574328073	10.83707511	9.09949275	6.001066554	6.097355209	5.256586542	24.03845668	21.64958	20.87616768	27.54485078	23.28314506	24.97404086	Prxl2a	peroxiredoxin like 2A [Source:MGI Symbol;Acc:MGI:1917814]
ENSMUSG00000021794	157.7233922	173.7943077	149.7483314	174.0095391	186.7005759	200.4886637	353.9471787	346.6453089	401.7243094	406.4290825	418.2409852	413.561186	Glud1	glutamate dehydrogenase 1 [Source:MGI Symbol;Acc:MGI:95753]
ENSMUSG00000021795	0	0	0	0	0	0	0.124561784	0.110572886	0.337638761	0.099839083	0.232275457	0.197836081	Sftpd	surfactant associated protein D [Source:MGI Symbol;Acc:MGI:109515]
ENSMUSG00000021796	30.98866127	39.5031007	25.31629531	29.52309089	38.41636695	48.05351455	25.11931267	23.39559011	30.29115765	43.22647622	41.3058155	38.78951949	Bmpr1a	bone morphogenetic protein receptor, type 1A [Source:MGI Symbol;Acc:MGI:1338938]
ENSMUSG00000021797	0	0.063688323	0	0	0.058108378	0	0	0	0	0.148411181	0.322259805	0.049014016	9230112D13	RIKEN cDNA 9230112D13 gene [Source:MGI Symbol;Acc:MGI:1925493]
ENSMUSG00000021798	0.641439988	0.519052138	0.585706792	0.598581257	0.637506504	0.884713692	0.975074862	0.875873583	1.274731253	3.132380825	2.611947809	2.688661692	Ldb3	LIM domain binding 3 [Source:MGI Symbol;Acc:MGI:1344412]
ENSMUSG00000021799	0.051377299	0	0.026299851	0	0	0	0.091736965	0.152689604	0.119549706	0.091911561	0.192448883	0.182127306	Opn4	opsin 4 (melanopsin) [Source:MGI Symbol;Acc:MGI:1353425]
ENSMUSG00000021803	0.178788731	0.194446391	0.132197427	0.083421353	0.093923099	0.181573522	0.328104709	0.314872375	0.351308347	1.510377258	1.273268427	1.302786217	Cdhr1	cadherin-related family member 1 [Source:MGI Symbol;Acc:MGI:2157782]
ENSMUSG00000021804	0	0.026119934	0	0	0	0	0	0	0	0	0	0	Rgr	retinal G protein coupled receptor [Source:MGI Symbol;Acc:MGI:1929473]
ENSMUSG00000021806	25.52386152	39.90387658	18.73036416	24.42521185	29.80400247	36.68748147	74.39392184	60.69920713	99.76095314	87.91221711	91.91406178	93.68830983	Nid2	nidogen 2 [Source:MGI Symbol;Acc:MGI:1298229]
ENSMUSG00000021807	186.9715087	141.9201936	189.5827683	154.96672	132.5555251	115.2017951	265.0662191	293.4196325	254.6286986	188.5947683	193.8835907	200.6878185	Rtraf	RNA transcription, translation and transport factor [Source:MGI Symbol;Acc:MGI:1915295]
ENSMUSG00000021809	14.04233487	15.33200161	14.97989491	16.85734243	16.24730861	17.48299892	10.18411522	9.102215665	9.42332977	9.366128803	10.64982035	9.402634033	Nudt13	nudix (nucleoside diphosphate linked moiety X)-type motif 13 [Source:MGI Symbol;Acc:MGI:1914975]
ENSMUSG00000021810	22.39106698	21.3023514	20.2088603	20.43596486	22.40193509	22.3814634	46.33839505	46.41810751	47.43396028	47.05785153	45.03811877	42.90899414	Ecd	ecdysoneless cell cycle regulator [Source:MGI Symbol;Acc:MGI:1917851]
ENSMUSG00000021811	61.9738668	55.74320508	60.07872182	73.30257101	69.62587089	59.15833016	100.6956529	98.77108739	94.1453935	64.58941372	62.74635462	65.34528537	Dnajc9	DnaJ heat shock protein family (Hsp40) member C9 [Source:MGI Symbol;Acc:MGI:1915326]
ENSMUSG00000021814	107.724237	104.7729089	99.25855521	110.2364327	112.8278727	102.1915409	79.26137387	76.55068666	82.09221528	118.3655523	107.3232549	110.9542767	Anxa7	annexin A7 [Source:MGI Symbol;Acc:MGI:88031]
ENSMUSG00000021815	1.555246548	1.682404145	1.447500006	1.671229088	1.955891101	2.853816693	1.704054315	2.451102962	1.732685088	0.864187635	0.764982564	1.691547251	Mss51	MSS51 mitochondrial translational activator [Source:MGI Symbol;Acc:MGI:1922093]
ENSMUSG00000021816	35.2267379	43.37760208	28.43151256	43.60211908	50.43077602	50.93059761	37.73955528	37.63748883	46.02333435	50.41517644	52.72690207	51.65466798	Ppp3cb	protein phosphatase 3, catalytic subunit, beta isoform [Source:MGI Symbol;Acc:MGI:107163]
ENSMUSG00000021819	56.57629602	65.11314553	57.60678872	66.3622524	72.6878844	83.94944276	10.8204736	11.09043549	14.02928422	26.44233587	26.20088176	26.50035571	Zswim8	zinc finger SWIM-type containing 8 [Source:MGI Symbol;Acc:MGI:1919156]
ENSMUSG00000021820	55.7521339	59.29341163	51.59214735	56.00430448	56.7985063	65.07063384	27.72696314	23.9983833	30.21917041	37.7591338	38.34727382	35.57775011	Camk2g	calcium/calmodulin-dependent protein kinase II gamma [Source:MGI Symbol;Acc:MGI:88259]
ENSMUSG00000021822	67.61619516	78.25677468	60.903984	75.54802496	79.24611571	76.8368706	5.508161616	5.36769934	4.979656406	26.41211237	24.59804464	24.04995894	Plau	plasminogen activator, urokinase [Source:MGI Symbol;Acc:MGI:97611]
ENSMUSG00000021823	6.502439392	21.69332027	3.068315934	14.50819069	27.62261989	49.81325027	7.405848733	4.546226916	11.3074097	33.36581164	37.84382552	34.44530103	Vcl	vinculin [Source:MGI Symbol;Acc:MGI:98927]
ENSMUSG00000021824	25.66852886	34.72267847	22.4486211	30.52588564	30.84224788	39.37492633	41.82896195	39.19179603	48.08971008	61.22638129	58.19097225	58.78416445	Ap3m1	adaptor-related protein complex 3, mu 1 subunit [Source:MGI Symbol;Acc:MGI:1929212]
ENSMUSG00000021830	14.90750759	21.1983492	11.31175453	20.26995338	24.72020907	28.30092596	3.663358787	4.139518018	5.080601024	6.650370392	6.909554718	7.089450784	Txndc16	thioredoxin domain containing 16 [Source:MGI Symbol;Acc:MGI:1917811]
ENSMUSG00000021831	285.8979388	337.972703	239.0976459	358.3190387	421.3632208	534.241812	91.14886969	89.19650393	132.1587682	134.7573527	128.1059454	130.3772834	Ero1l	ERO1-like (S. cerevisiae) [Source:MGI Symbol;Acc:MGI:1354385]
ENSMUSG00000021832	185.2797606	189.1362519	166.1384395	222.4210818	224.1336205	220.8116021	238.0099692	227.7709423	245.6003653	231.5143675	229.9754127	234.609008	Psmc6	proteasome (prosome, macropain) 26S subunit, ATPase, 6 [Source:MGI Symbol;Acc:MGI:1914339]
ENSMUSG00000021835	3.23290964	2.348948415	1.536706402	1.723941803	1.172666576	2.374504179	139.2967964	135.5791721	134.4046659	48.0927451	49.17612152	52.56064949	Bmp4	bone morphogenetic protein 4 [Source:MGI Symbol;Acc:MGI:88180]
ENSMUSG00000021838	12.78464233	18.13788988	10.89774252	15.50428791	16.58821803	23.02189059	0.653655894	0.613722938	0.943566397	12.47668369	11.48577813	11.31342722	Samd4	sterile alpha motif domain containing 4 [Source:MGI Symbol;Acc:MGI:1921730]
ENSMUSG00000021840	27.85778201	30.27621138	25.02232594	30.92703532	33.92285947	38.43717861	43.61215786	39.36622405	48.91297109	54.68970222	55.67417262	54.25527248	Mapk1ip1l	mitogen-activated protein kinase 1 interacting protein 1-like [Source:MGI Symbol;Acc:MGI:2444022]
ENSMUSG00000021843	43.79903778	51.07064913	36.85212403	53.48470641	62.74935148	72.19973278	28.27062456	27.34694095	32.84303642	44.73487621	44.31803836	44.96170797	Ktn1	kinectin 1 [Source:MGI Symbol;Acc:MGI:109153]
ENSMUSG00000021846	7.321199661	8.15307873	7.607596952	4.459224742	5.66543368	5.543033546	9.510758573	9.011577583	9.24211189	5.391844499	6.07591418	6.293968986	Peli2	pellino 2 [Source:MGI Symbol;Acc:MGI:1891445]
ENSMUSG00000021848	0.289862568	0.296703686	0	0	0	0.343440336	13.22667131	13.40034134	16.54725422	45.25793052	48.65840494	47.20944937	Otx2	orthodenticle homeobox 2 [Source:MGI Symbol;Acc:MGI:97451]
ENSMUSG00000021850	0	0.27604187	0.122708218	0.083886096	0.125928463	0	0.053502582	0.237469904	0.083668144	0.026802205	0.149652683	0.079664843	ccdc198	coiled-coil domain containing 198 [Source:MGI Symbol;Acc:MGI:1914332]
ENSMUSG00000021852	0.046770095	0.053858169	0	0	0	0	0.501063314	0.347493324	0.500614394	0.20917373	0.116794159	0	Slc35f4	solute carrier family 35, member F4 [Source:MGI Symbol;Acc:MGI:1922538]
ENSMUSG00000021866	3.626922346	3.73361612	2.550492136	4.564060699	5.196358431	5.622788555	10.74438447	9.436117839	10.36606391	9.175504967	8.188021342	8.685016606	Anxa11	annexin A11 [Source:MGI Symbol;Acc:MGI:108481]
ENSMUSG00000021867	0	0	0	0	0	0	0	0	0	0	0	0	Tmem254b	transmembrane protein 254b [Source:MGI Symbol;Acc:MGI:3710397]
ENSMUSG00000021868	8.782862605	7.314142254	11.71253433	8.459843556	9.029782377	7.019928205	26.5521856	29.76154237	23.08571327	15.86385298	16.15916853	16.68627761	Ppif	peptidylprolyl isomerase F (cyclophilin F) [Source:MGI Symbol;Acc:MGI:2145814]
ENSMUSG00000021870	31.91836878	41.82612394	25.54497928	37.57020141	47.55071826	64.06217246	27.73300346	25.5828706	33.39158532	49.2067653	50.75603857	51.10731267	Slmap	sarcolemma associated protein [Source:MGI Symbol;Acc:MGI:1933549]
ENSMUSG00000021871	0	0	0	0	0	0	0	0	0	0	0	0	Gm49342	predicted gene, 49342 [Source:MGI Symbol;Acc:MGI:6121535]
ENSMUSG00000021872	0.273196748	0.503360159	0.167818096	0.076482815	0.086111083	0.124853412	0.170732685	0.054128061	0	0.048873608	0.06822262	0.024211368	Rnase10	ribonuclease, RNase A family, 10 (non-active) [Source:MGI Symbol;Acc:MGI:1922269]
ENSMUSG00000021874	0.517524768	0.723661311	0.756911515	0.6899223	0.699097706	0.394189252	0.264019616	0.329581297	0.309658388	0.495978948	0.553869208	0.524163638	4933413J09	RIKEN cDNA 4933413J09 gene [Source:MGI Symbol;Acc:MGI:1918356]
ENSMUSG00000021876	77.90785976	74.74023646	72.89774647	50.88624088	52.46472405	48.84187495	8.462103516	7.557571385	8.902799969	31.4831048	28.74490678	28.86217836	Rnase4	ribonuclease, RNase A family 4 [Source:MGI Symbol;Acc:MGI:1926217]
ENSMUSG00000021877	123.6574755	162.5157881	91.63751294	126.4985505	147.2197368	164.9105483	75.80274463	64.41951419	76.01643464	100.7053556	105.5356055	100.7957481	Arf4	ADP-ribosylation factor 4 [Source:MGI Symbol;Acc:MGI:99433]
ENSMUSG00000021879	0.136381895	0.047115229	0.041888035	0.127269343	0.028658205	0.09349166	0.036527595	0.162126838	0.133285607	0.103691827	0.102171753	0.048345996	Dnah12	dynein, axonemal, heavy chain 12 [Source:MGI Symbol;Acc:MGI:107720]
ENSMUSG00000021880	0.078878988	0.454166046	0.040377865	0.036804288	0.041437505	0.090121047	0	0.117211314	0.036708663	0	0.0984882	0.10485675	Rnase6	ribonuclease, RNase A family, 6 [Source:MGI Symbol;Acc:MGI:1925666]
ENSMUSG00000021884	0.280544338	0.12114796	0.359024044	0.065449842	0.07368919	0.133553466	0.062615899	0.173699461	0.032639895	0.062735072	0.262715468	0.186468972	Hacl1	2-hydroxyacyl-CoA lyase 1 [Source:MGI Symbol;Acc:MGI:1929657]
ENSMUSG00000021886	0.020796756	0.1436912	0	0	0.043700646	0	0	0	0.038713535	0.055806621	0.017311201	0.147444774	Gpr65	G-protein coupled receptor 65 [Source:MGI Symbol;Acc:MGI:108031]
ENSMUSG00000021890	35.77397453	43.57831096	29.75951549	39.68819999	47.01560338	49.44585732	49.69893925	39.95864518	54.2032549	81.9262969	85.12214555	76.35418227	Eaf1	ELL associated factor 1 [Source:MGI Symbol;Acc:MGI:1921677]

ENSMUSG00000021891	42.27525391	39.17700579	43.21683189	45.74743427	46.49433178	39.65871644	26.50652951	27.95086585	28.89030697	30.2626023	32.26392503	30.09467752	Mettl6	methyltransferase like 6 [Source:MGI Symbol;Acc:MGI:1914261]
ENSMUSG00000021892	13.54255224	18.11805149	11.90794846	14.63883564	17.46128439	17.80733624	2.122597571	2.078183257	2.668497095	7.464080152	8.090810983	7.519164305	Sh3bp5	SH3-domain binding protein 5 (BTK-associated) [Source:MGI Symbol;Acc:MGI:1344391]
ENSMUSG00000021893	8.163206024	12.72415162	7.159792438	10.39434001	13.69341794	15.7478007	12.75954373	10.75723225	13.32604746	17.74267953	16.98761381	16.64370574	Capn7	calpain 7 [Source:MGI Symbol;Acc:MGI:1338030]
ENSMUSG00000021895	5.239341937	6.177883465	4.352227417	9.822451195	11.60286406	13.34618339	6.638221229	5.889608559	6.906033055	11.88454521	11.76485775	10.74616672	Arhgef3	Rho guanine nucleotide exchange factor (GEF) 3 [Source:MGI Symbol;Acc:MGI:1918954]
ENSMUSG00000021898	0.258170927	0.660660208	0.0881045	0.347996808	0.361666547	0.633631066	0.28170893	0.142086159	0.347092647	0.359221019	0.501436257	0.177953555	Asb14	ankyrin repeat and SOCS box-containing 14 [Source:MGI Symbol;Acc:MGI:2655107]
ENSMUSG00000021900	16.53184768	12.95455153	15.81437781	13.74863607	13.22950789	11.37412926	5.913899233	5.883328681	5.155630654	6.078391504	6.488392174	6.705520732	Btd	biotinidase [Source:MGI Symbol;Acc:MGI:1347001]
ENSMUSG00000021901	42.4341717	45.75117568	34.92540278	43.78614745	46.61327299	49.29683491	68.65859277	64.91021697	74.12412022	72.82943913	75.9129771	73.13195879	Bap1	Brca1 associated protein 1 [Source:MGI Symbol;Acc:MGI:1206586]
ENSMUSG00000021902	6.68861004	6.590329664	6.34140219	7.010924701	7.200668111	6.188854472	4.331395697	5.039509088	5.21713085	4.613500074	4.31292425	4.508323707	Phf7	PHD finger protein 7 [Source:MGI Symbol;Acc:MGI:1919088]
ENSMUSG00000021903	0.052208479	0.070140871	0.035633772	0.040600079	0.063995597	0.099415634	0	0	0	0.007783209	0.065187438	0.015422813	Galnt15	polypeptide N-acetylgalactosaminyltransferase 15 [Source:MGI Symbol;Acc:MGI:1926004]
ENSMUSG00000021904	0.047388202	0.218279805	0.038812555	0.088443784	0.029873338	0.007218947	0.067691373	0.084500579	0.035285596	0.059342679	0.126226874	0.109191137	Sema3g	sema domain, immunoglobulin domain (Ig), short basic domain, secreted, (semaphorin) 3G [Source:MGI Symbol;Acc:MGI:3041242]
ENSMUSG00000021905	12.05045483	12.84220707	14.08149682	15.11898409	16.01331363	13.94488567	14.90684402	13.74829357	13.11905251	12.82932212	11.52638109	10.95394454	Dph3	diphthamine biosynthesis 3 [Source:MGI Symbol;Acc:MGI:1922658]
ENSMUSG00000021906	17.05349242	18.45605811	16.89343173	24.68149555	26.04662745	25.13677179	30.09750952	34.06157188	32.73742569	25.21138348	25.40040802	26.37817444	Oxnad1	oxidoreductase NAD-binding domain containing 1 [Source:MGI Symbol;Acc:MGI:1916953]
ENSMUSG00000021907	0	0	0.080095	0	0	0	0	0	0.072816639	0	0.130243184	0.138665108	Msmb	beta-microseminoprotein [Source:MGI Symbol;Acc:MGI:97166]
ENSMUSG00000021908	0.96229845	2.057967103	0.539511051	0.534524465	0.674032435	1.012190201	5.850185444	4.380611611	7.165343403	14.03847549	13.35027308	13.44195043	Ncoa4-ps	nuclear receptor coactivator 4, pseudogene [Source:MGI Symbol;Acc:MGI:3648259]
ENSMUSG00000021910	369.4379311	397.8201476	353.7991303	386.165779	390.3388375	446.0135336	169.1156868	155.552326	196.4788877	267.4046364	280.5928181	284.7638794	Nisch	nischarin [Source:MGI Symbol;Acc:MGI:1928323]
ENSMUSG00000021911	20.17168408	23.15206867	17.15288752	24.33230348	25.9965317	29.39351309	35.20536136	33.89843071	36.57918894	49.24633552	46.51206419	45.05543303	Parg	poly (ADP-ribose) glycohydrolase [Source:MGI Symbol;Acc:MGI:1347094]
ENSMUSG00000021913	17.2994413	17.79952285	14.82318991	16.64566304	18.6554978	18.04496096	50.45315809	48.76371133	57.34965654	38.54630357	39.68493517	38.59529686	Ogdhl	oxoglutarate dehydrogenase-like [Source:MGI Symbol;Acc:MGI:3616088]
ENSMUSG00000021916	36.49479966	41.65152236	38.65658868	37.79547159	34.81647336	35.16608422	10.24825805	9.583663745	10.04588616	13.88408183	13.04070802	14.1398858	Glt8d1	glycosyltransferase 8 domain containing 1 [Source:MGI Symbol;Acc:MGI:1923735]
ENSMUSG00000021917	170.6779589	147.1130434	190.1230012	151.3293169	137.5108801	117.4630275	56.04264766	59.696509	46.30235635	68.48931322	62.11389013	63.49424058	Spcs1	signal peptidase complex subunit 1 homolog (S. cerevisiae) [Source:MGI Symbol;Acc:MGI:1916269]
ENSMUSG00000021918	4.259355365	5.595692946	4.176441985	6.018123203	6.413309713	7.688015298	7.150063463	8.037847196	9.227078828	12.6370388	12.85862034	12.67995506	Nek4	NIMA (never in mitosis gene a)-related expressed kinase 4 [Source:MGI Symbol;Acc:MGI:1344404]
ENSMUSG00000021919	0	0	0.084879095	0.077367009	0	0.157871005	0	0	0.038582998	0.037078966	0.03450566	0.073473805	Chat	choline acetyltransferase [Source:MGI Symbol;Acc:MGI:88392]
ENSMUSG00000021922	0.374160764	0.711864497	0.283133555	0.592055061	0.324747844	0.235427747	0.029047149	0.032231265	0.015141456	0.014551216	0.040624055	0.043250932	Itih4	inter alpha-trypsin inhibitor, heavy chain 4 [Source:MGI Symbol;Acc:MGI:109536]
ENSMUSG00000021927	0.178726845	0.308719724	0.365958465	0.166784955	0.187781177	0.136133004	0.47868977	0.442636009	0.956521791	0.799334712	0.371930171	0.871156701	Hnrnpa1l2-p	heterogeneous nuclear ribonucleoprotein A1-like 2, pseudogene 2 [Source:MGI Symbol;Acc:MGI:3645633]
ENSMUSG00000021928	3.856697248	3.211040169	4.784791688	3.415757698	2.797667017	2.506811728	5.532917098	5.983803739	4.576628122	4.594948888	3.903372519	4.308920774	Ebpl	emopamil binding protein-like [Source:MGI Symbol;Acc:MGI:1915427]
ENSMUSG00000021929	28.98647292	33.33784524	26.07287124	38.9071031	40.29684417	48.5836363	59.39477857	58.21718126	69.14526328	88.0024768	87.33368169	86.74276096	Kpna3	karyopherin (importin) alpha 3 [Source:MGI Symbol;Acc:MGI:1100863]
ENSMUSG00000021930	7.216055447	7.202913842	6.919961675	9.615662579	9.369428116	9.000554917	10.21207765	10.51989064	9.751990393	6.99012498	7.672234403	7.330565713	Spryd7	SPRY domain containing 7 [Source:MGI Symbol;Acc:MGI:1913924]
ENSMUSG00000021932	10.62460251	7.590338226	11.5852463	11.22662851	10.31534205	9.1282757	37.71527987	35.47968255	32.2409613	19.15121626	19.54868565	19.81195369	Rnaseh2b	ribonuclease H2, subunit B [Source:MGI Symbol;Acc:MGI:1914403]
ENSMUSG00000021933	0	0.050253058	0	0.020361804	0	0.016619686	0	0	0	0	0	0	Gucy1b2	guanylate cyclase 1, soluble, beta 2 [Source:MGI Symbol;Acc:MGI:2660873]
ENSMUSG00000021936	16.05443579	17.91367181	13.66373924	20.09695718	22.44481465	26.23901954	20.21325403	17.25638547	22.10164656	23.66254126	25.96993992	23.3290429	Mapk8	mitogen-activated protein kinase 8 [Source:MGI Symbol;Acc:MGI:1346861]
ENSMUSG00000021938	16.76985959	21.65805172	13.51776005	22.34490528	24.22113159	28.00424457	42.18621863	36.71668973	47.59664826	56.18445609	54.21123531	55.4403898	Pspc1	paraspeckle protein 1 [Source:MGI Symbol;Acc:MGI:1913895]
ENSMUSG00000021939	810.265646	779.9848675	831.7165706	701.952102	656.8444175	591.317799	122.0445496	116.8222264	117.9078058	303.6159919	307.621381	303.8878508	Ctsb	cathepsin B [Source:MGI Symbol;Acc:MGI:88561]
ENSMUSG00000021940	0.357775675	0.851460634	0.561641771	0.53419261	0.87710152	0.417850596	0.04258853	0.141771112	0.111000974	0.405361065	0.416937021	0.211379738	Ptpn20	protein tyrosine phosphatase, non-receptor type 20 [Source:MGI Symbol;Acc:MGI:1196295]
ENSMUSG00000021943	2.461130398	2.57733565	2.046188973	2.705157528	3.010995483	2.799316542	0.61198328	0.499074225	0.684140976	3.169162682	3.066089532	3.147245493	Gdf10	growth differentiation factor 10 [Source:MGI Symbol;Acc:MGI:95684]
ENSMUSG00000021944	4.783523202	6.150236694	4.659601208	7.172585625	8.831846326	7.729221273	0.829246715	0.736118255	0.864525667	3.925647946	3.943142255	3.498431623	Gata4	GATA binding protein 4 [Source:MGI Symbol;Acc:MGI:95664]
ENSMUSG00000021945	23.11613178	31.1541939	18.90556756	26.05616064	30.92585583	42.04361179	39.40799921	37.60067021	50.40312038	64.279282	63.3554969	64.81016428	Zmym2	zinc finger, MYM-type 2 [Source:MGI Symbol;Acc:MGI:1923257]
ENSMUSG00000021947	5.134339097	6.749617229	6.838100083	4.621678019	3.675861154	4.291424033	20.40408828	22.37069304	22.45628243	11.42039974	13.08620794	13.24786235	Cryl1	crystallin, lambda 1 [Source:MGI Symbol;Acc:MGI:1915881]
ENSMUSG00000021948	46.41861111	51.16021613	42.91150322	44.73296539	47.92330827	49.94652401	27.68409318	26.79674503	31.75246779	33.94999127	36.92196916	33.63679361	Prkcd	protein kinase C, delta [Source:MGI Symbol;Acc:MGI:97598]
ENSMUSG00000021950	10.39483547	11.03952939	9.500906293	5.331258391	4.714079828	5.731208272	5.871700493	5.43866062	5.473038194	15.50486728	14.10405459	14.10225944	Anxa8	annexin A8 [Source:MGI Symbol;Acc:MGI:1201374]
ENSMUSG00000021951	36.31271556	24.50100358	40.15812863	26.8429327	20.23134689	16.29647435	51.57353337	59.87636341	46.18928387	43.21922191	44.27638612	39.76566718	Eef1akmt1	EEF1A alpha lysine methyltransferase 1 [Source:MGI Symbol;Acc:MGI:1915293]
ENSMUSG00000021952	8.01158037	11.8172485	6.967259655	11.87803717	13.27876129	19.01990235	39.20243895	35.09944279	49.53359494	63.48098889	58.82060094	61.55512397	Xpo4	exportin 4 [Source:MGI Symbol;Acc:MGI:1888526]
ENSMUSG00000021953	2.432237236	0.203697906	0.045274666	0.165070824	0.139388443	1.852591095	182.4014654	179.2651272	178.1837379	239.9465612	232.828096	225.9364805	Tdh	L-threonine dehydrogenase [Source:MGI Symbol;Acc:MGI:1926231]
ENSMUSG00000021957	103.6237683	102.8992891	97.87656298	106.7206004	109.6201702	103.2333311	409.7131162	412.7288569	415.9441082	197.0038916	206.4700238	207.6274793	Tkt	transketolase [Source:MGI Symbol;Acc:MGI:105992]
ENSMUSG00000021958	24.18735638	19.8595253	24.1448706	23.37592247	19.8862242	17.20800282	28.36848442	28.23214963	26.7663167	30.62068667	27.02415555	26.44085175	Pinx1	PIN2/TERF1 interacting, telomerase inhibitor 1 [Source:MGI Symbol;Acc:MGI:1919650]
ENSMUSG00000021959	19.99396544	25.86230071	17.85200931	15.13278822	18.17529287	16.9835396	1.213317125	1.072491885	1.029100253	10.75520259	9.20347978	8.104262934	Lats2	large tumor suppressor 2 [Source:MGI Symbol;Acc:MGI:1354386]
ENSMUSG00000021961	0	0	0.043876743	0.039993504	0	0	0.076523614	0	0	0	0	0.151923923	4930578I06R	RIKEN cDNA 4930578I06 gene [Source:MGI Symbol;Acc:MGI:1915000]
ENSMUSG00000021962	12.30036179	14.42680444	10.44754266	14.53942765	17.27919844	17.62753042	24.68800045	23.67099748	29.2153154	31.64203906	32.40393869	31.99611113	Dcp1a	decapping mRNA 1A [Source:MGI Symbol;Acc:MGI:1923151]
ENSMUSG00000021963	215.8306328	189.2919805	234.8223146	166.17974	143.6130204	131.4954613	162.1147802	182.8074245	138.113144	154.9485074	148.17766	153.9837476	Sap18	Sin3-associated polypeptide 18 [Source:MGI Symbol;Acc:MGI:1277978]
ENSMUSG00000021965	20.22516732	16.99425536	18.43095179	25.92554096	24.79919116	26.78125033	72.06753615	66.36069472	77.61591762	60.71397175	61.19949591	60.58719774	Ska3	spindle and kinetochore associated complex subunit 3 [Source:MGI Symbol;Acc:MGI:3041235]
ENSMUSG00000021966	0	0	0	0.037738231	0	0	0	0	0	0.108518702	0.033662477	0.143356785	Prss52	protease, serine 52 [Source:MGI Symbol;Acc:MGI:1920632]
ENSMUSG00000021967	28.5842958	22.03173398	34.81632495	22.46513778	20.63394978	16.21329643	29.79526667	31.60538413	21.10953511	19.833316	20.56622709	21.78381481	Mrpl57	mitochondrial ribosomal protein L57 [Source:MGI Symbol;Acc:MGI:1915090]
ENSMUSG00000021969	42.94641155	47.30478316	40.0188539	43.3703516	47.2348238	58.29618358	23.74136904	24.3111654	28.95303467	46.36175634	45.14258555	47.00694259	Zdhhc20	zinc finger, DHHC domain containing 20 [Source:MGI Symbol;Acc:MGI:1923215]
ENSMUSG00000021972	12.7805526	13.93715597	11.98622031	13.34936271	13.54667457	16.44435769	9.561702523	9.416471167	9.863373713	11.66761185	11.87628679	11.22635383	Hmbox1	homeobox containing 1 [Source:MGI Symbol;Acc:MGI:2445066]
ENSMUSG00000021973	65.57077989	71.97809741	60.01210105	77.48121136	86.09611974	80.0130304	18.92373784	20.03525943	18.47619414	29.05824742	25.3612107	26.1397583	Micu2	mitochondrial calcium uptake 2 [Source:MGI Symbol;Acc:MGI:1915764]
ENSMUSG00000021974	1.540399326	1.093873555	0.473114859	0.574990136	0.512504802	0.469317718	0.091682229	0.050866167	0	0.068892541	0.256445409	0.113761649	Fgf9	fibroblast growth factor 9 [Source:MGI Symbol;Acc:MGI:104723]
ENSMUSG00000021975	9.285475356	9.976524322	9.580438951	9.056465085	9.193624404	9.474170672	25.59268597	25.87639696	27.32765633	21.33332993	20.46678599	21.40569776	Ints9	integrator complex subunit 9 [Source:MGI Symbol;Acc:MGI:1098533]
ENSMUSG00000021977	0	0	0	0	0	0	0	0	0	0	0	0	1700129C05	RIKEN cDNA 1700129C05 gene [Source:MGI Symbol;Acc:MGI:1915182]
ENSMUSG00000021978	25.79039881	26.79055175	27.04520156	18.7018823	18.97433357	21.38796145	11.41171399	8.97065084	12.86236172	20.9934362	22.10886587	21.53676578	Extl3	exostosin-like glycosyltransferase 3 [Source:MGI Symbol;Acc:MGI:1860765]
ENSMUSG00000021981	28.20331721	25.0262782	22.61311574	25.55452936	25.41533633	27.59589174	31.95550361	30.61704289	34.33147443	22.49201588	21.68102742	22.26032507	Cab39l	calcium binding protein 39-like [Source:MGI Symbol;Acc:MGI:1914081]
ENSMUSG00000021982	14.61572026	11.5624779	12.5229676	12.58307084	12.16855127	12.96655433	10.85590448	11.92665017	9.950656562	10.07966887	9.640692281	9.98668125	Cdadc1	cytidine and dCMP deaminase domain containing 1 [Source:MGI Symbol;Acc:MGI:1919141]
ENSMUSG00000021983	0.179659657	0.310330996	0.168606386	0.237511873	0.267411765	0.307897897	0.1069307	0.118652325	0.069674933	0.468712186	0.560807027	0.995117555	Atp8a2	ATPase, aminophospholipid transporter-like, class I, type 8A, member 2 [Source:MGI Symbol;Acc:MGI:1354710]
ENSMUSG00000021986	0	0.03430615	0.030500058	0.0092669	0.020866983	0.022691452	0.026596945	0	0.018485646	0.044412608	0.066128549	0.061604046	Amer2	APC membrane recruitment 2 [Source:MGI Symbol;Acc:MGI:1919375]
ENSMUSG00000021987	16.37567122	23.99445048	12.85339059	23.97615802	26.89558747	37.73456794	8.116524285	7.700995232	10.58166558	18.83991005	19.30288302	21.01813644	Mtmr6	myotubularin related protein 6 [Source:MGI Symbol;Acc:MGI:2145637]
ENSMUSG00000021990	4.573281201	5.36419567	3.841344621	6.223194492	7.140504379	9.134450635	12.84287323	9.734234388	14.65437858	17.29995461	18.05575954	18.39514257	Spata13	spermatogenesis associated 13 [Source:MGI Symbol;Acc:MGI:104838]
ENSMUSG00000021991	2.254649299	1.908156852	2.197050357	0.380241267	0.256865445	0.351741383	0.436532433	0.215282059	0.252835553	0.485959171	0.29395163	0.457402266	Cacna2d3	calcium channel, voltage-dependent, alpha2/delta subunit 3 [Source:MGI Symbol;Acc:MGI:1338890]
ENSMUSG00000021993	7.191904394	6.476829542	7.252888336	8.618657207	7.879164055	7.900879975	15.4071004	14.93427021	16.54888154	10.73539348	10.74506264	11.68501151	Mipep	mitochondrial intermediate peptidase [Source:MGI Symbol;Acc:MGI:1917728]
ENSMUSG00000021994	49.27728261	63.05516205	44.47490589	26.94993563	34.58150475	38.40549213	2.144396564	1.542932996	2.322955583	40.90500395	41.9868126	42.63967188	Wnt5a	wingless-type MMTV integration site family, member 5A [Source:MGI Symbol;Acc:MGI:98958]
ENSMUSG00000021996	1197.667794	1011.430205	1216.720088	1123.366824	1010.298526	860.0996748	173.8683893	183.0491212	154.9526268	255.0998466	245.571787	250.2720419	Esd	esterase D/formylglutathione hydrolase [Source:MGI Symbol;Acc:MGI:95421]

ENSMUSG00000021997	0.153856333	0.039371884	0.052505662	0.047858734	0.017961191	0.039063195	0	0.03387036	0	0	0.014229986	0.015150141	Lrrc63	leucine rich repeat containing 63 [Source:MGI Symbol;Acc:MGI:1918109]
ENSMUSG00000021998	15.66812414	20.30174683	15.18396709	0.561416407	0.384751646	0.876630339	1.261034723	1.477005057	1.339029382	29.9714802	32.44203311	28.5359305	Lcp1	lymphocyte cytosolic protein 1 [Source:MGI Symbol;Acc:MGI:104808]
ENSMUSG00000021999	0.073868649	0	0.189065451	0	0	0.056264413	0.032974124	0.073177421	0.103130862	0	0.092232297	0.032732107	Cpb2	carboxypeptidase B2 (plasma) [Source:MGI Symbol;Acc:MGI:1891837]
ENSMUSG00000022000	11.63926942	14.85023831	10.07094215	13.05284147	14.89607422	18.92965558	17.54165018	15.44086754	21.22959448	28.44022294	30.03757433	27.64916806	Zc3h13	zinc finger CCCH type containing 13 [Source:MGI Symbol;Acc:MGI:1914552]
ENSMUSG00000022002	0.060817651	0	0	0	0	0.023161852	0	0	0	0	0	0	Erich6b	glutamate rich 6B [Source:MGI Symbol;Acc:MGI:1922522]
ENSMUSG00000022003	34.51867547	44.25871312	27.52754353	48.84034908	57.9690693	72.5167049	46.86826207	39.49916064	53.51042622	29.08932649	31.42706344	27.79417844	Slc25a30	solute carrier family 25, member 30 [Source:MGI Symbol;Acc:MGI:1914804]
ENSMUSG00000022008	5.157989077	6.491363524	4.822940734	5.759628571	6.618920612	8.666353573	6.52247012	5.797874627	6.599303498	10.06300698	10.18876788	10.87590917	Gpalpp1	GPALPP motifs containing 1 [Source:MGI Symbol;Acc:MGI:1914717]
ENSMUSG00000022009	7.525112995	7.730422669	8.347092199	7.567930271	7.599183859	6.548206438	26.64100096	28.30639459	26.83716419	16.72589782	17.50286165	16.98420767	Nufip1	nuclear fragile X mental retardation protein interacting protein 1 [Source:MGI Symbol;Acc:MGI:1351474]
ENSMUSG00000022010	189.449809	186.1178087	188.526641	171.3989364	162.8059894	151.9748491	212.5780664	218.5015044	217.6224556	129.4065095	134.9299648	121.9360031	Tsc22d1	TSC22 domain family, member 1 [Source:MGI Symbol;Acc:MGI:109127]
ENSMUSG00000022012	0.829598094	0.403359382	0.47185358	0.756963592	0.34865027	0.75826783	23.65130476	23.13922409	26.64795729	25.82354556	25.35110467	26.01010466	Enox1	ecto-NOX disulfide-thiol exchanger 1 [Source:MGI Symbol;Acc:MGI:2444896]
ENSMUSG00000022013	9.990970414	8.730832724	10.60953741	10.59628625	7.761164574	7.01625802	14.8345546	14.12672186	11.03041678	10.72719359	8.832568305	9.37231128	Dnajc15	DnaJ heat shock protein family (Hsp40) member C15 [Source:MGI Symbol;Acc:MGI:1913398]
ENSMUSG00000022014	2.078080378	1.794762285	1.376632819	0.855542852	0.610055432	0.512094072	0.245549493	0.454110593	0.369771996	5.275694254	3.612213294	4.252035537	Epsti1	epithelial stromal interaction 1 (breast) [Source:MGI Symbol;Acc:MGI:1915168]
ENSMUSG00000022015	1.924350976	3.43478306	2.186851419	2.065138619	2.547517045	2.315874446	0.721567026	0.610030021	0.608976365	1.979479487	1.393590414	1.603082755	Tnfsf11	tumor necrosis factor (ligand) superfamily, member 11 [Source:MGI Symbol;Acc:MGI:1100089]
ENSMUSG00000022016	20.3355512	24.11752664	16.74188747	25.25223044	30.04762797	38.37811355	18.73111829	15.90480015	23.83548118	45.52411709	44.39929287	45.64293177	Akap11	A kinase (PRKA) anchor protein 11 [Source:MGI Symbol;Acc:MGI:2684060]
ENSMUSG00000022018	14.48344525	14.68133796	14.7320706	13.12205158	9.553827625	10.0678364	5.565550753	4.550471759	5.017050572	2.850960472	2.536053275	2.284651152	Rgcc	regulator of cell cycle [Source:MGI Symbol;Acc:MGI:1913464]
ENSMUSG00000022019	10.57306474	11.51602336	10.53150278	10.21012863	10.16327689	10.99736624	8.727329014	9.116744993	12.33452956	7.006126958	7.405104959	7.376469681	Tdrd3	tudor domain containing 3 [Source:MGI Symbol;Acc:MGI:2444023]
ENSMUSG00000022020	6.660020228	6.61481925	4.992101321	6.115137172	7.472242778	7.391818916	13.62251459	13.33677711	15.40862878	10.05282704	10.19662293	10.16034276	Naa16	N(alpha)-acetyltransferase 16, NatA auxiliary subunit [Source:MGI Symbol;Acc:MGI:1914147]
ENSMUSG00000022021	10.77622875	11.96933237	8.254906565	13.67571825	17.34457684	20.01078081	30.53400923	27.08596273	38.18185094	19.56870404	20.65991174	22.70702717	Diaph3	diaphanous related formin 3 [Source:MGI Symbol;Acc:MGI:1927222]
ENSMUSG00000022022	2.259899571	1.743601653	2.151711801	2.129990158	1.804533716	2.13444252	7.02120232	6.42521969	6.457504999	6.165351756	7.11070322	6.669252763	Mtrf1	mitochondrial translational release factor 1 [Source:MGI Symbol;Acc:MGI:2384815]
ENSMUSG00000022023	21.49071394	19.16588993	19.65729283	22.48477651	19.44798237	19.43978548	17.86997951	18.75661618	18.046209	15.79928915	15.48626114	17.22445093	Wbp4	WW domain binding protein 4 [Source:MGI Symbol;Acc:MGI:109568]
ENSMUSG00000022024	49.50683594	42.11456796	51.60342432	46.32150953	44.31151041	43.72647819	183.340069	183.4255705	165.7374857	83.96484627	79.55864457	81.32821432	Sugt1	SGT1, suppressor of G2 allele of SKP1 (S. cerevisiae) [Source:MGI Symbol;Acc:MGI:1915205]
ENSMUSG00000022025	0	0	0.051402859	0.093707066	0.05275183	0.114728194	0.044824801	0	0	0	0.04179332	0	Cnmd	chondromodulin [Source:MGI Symbol;Acc:MGI:1341171]
ENSMUSG00000022026	0.025566541	0.029441186	0	0.023858276	0	0.038947143	0	0	0	0.114343334	0.127689396	0	Olfm4	olfactomedin 4 [Source:MGI Symbol;Acc:MGI:2685142]
ENSMUSG00000022031	24.66764979	24.17389374	22.68419914	22.80773996	23.96279229	23.39684918	47.13752392	47.71997361	47.27066812	46.84844553	48.05378338	49.47224998	Elp3	elongator acetyltransferase complex subunit 3 [Source:MGI Symbol;Acc:MGI:1921445]
ENSMUSG00000022032	0.277440426	0.116177059	0.180753664	0.09414649	0.317995205	0.211321352	0.225174995	0.149915056	0.187803759	0.406086398	0.503871636	0.346459568	Scara5	scavenger receptor class A, member 5 [Source:MGI Symbol;Acc:MGI:1918395]
ENSMUSG00000022033	37.48769647	35.75382682	29.53851579	55.52545891	62.56789303	58.40011102	61.98963111	63.29592234	57.61130845	42.90827805	43.17138471	44.81279867	Pbk	PDZ binding kinase [Source:MGI Symbol;Acc:MGI:1289156]
ENSMUSG00000022034	12.81435957	15.38432542	9.414392179	22.41285716	24.140617	29.58346218	79.94980862	79.77614008	103.3526491	96.81851909	91.70853197	94.87097659	Esco2	establishment of sister chromatid cohesion N-acetyltransferase 2 [Source:MGI Symbol;Acc:MGI:1919238]
ENSMUSG00000022035	22.93184913	24.19860065	20.01986952	25.42664512	27.16003622	25.99373633	31.00733861	28.64440444	31.99663317	25.41622973	25.54381817	27.01080749	Ccdc25	coiled-coil domain containing 25 [Source:MGI Symbol;Acc:MGI:1914429]
ENSMUSG00000022037	43.17385434	39.11510186	46.28462774	67.14855246	60.58321941	51.44930717	24.26884362	25.15309031	23.54240664	39.83157781	38.47894055	38.30467453	Clu	clusterin [Source:MGI Symbol;Acc:MGI:88423]
ENSMUSG00000022039	0.021568164	0	0.066243985	0.040254113	0.113304056	0.032856161	0.019255566	0	0	0.096461069	0.035906642	0.152913904	Adam2	a disintegrin and metallopeptidase domain 2 [Source:MGI Symbol;Acc:MGI:1340894]
ENSMUSG00000022040	1.919962273	1.645918466	1.703557519	0.895838623	0.918959371	0.58496021	6.761166681	6.656986626	6.631837684	11.44907279	12.71436928	12.26983398	Ephx2	epoxide hydrolase 2, cytoplasmic [Source:MGI Symbol;Acc:MGI:99500]
ENSMUSG00000022041	0.015680936	0.054172211	0.032108054	0.014633192	0	0.035831655	0.041998747	0	0.043785516	0.154288495	0.013052797	0.097277818	Chrna2	cholinergic receptor, nicotinic, alpha polypeptide 2 (neuronal) [Source:MGI Symbol;Acc:MGI:87886]
ENSMUSG00000022043	69.83031209	85.46675158	62.70322777	83.74297467	92.93570717	99.34930733	27.42553015	28.14154224	33.25474323	52.15981114	53.4580231	53.44298135	Trim35	tripartite motif-containing 35 [Source:MGI Symbol;Acc:MGI:1914104]
ENSMUSG00000022044	2.473493911	2.907696126	2.690616479	1.923519896	1.786676054	0.981258103	0	0.459440275	0.095926111	0.691400594	0.943678156	0.228340747	Stmn4	stathmin-like 4 [Source:MGI Symbol;Acc:MGI:1931224]
ENSMUSG00000022048	15.44075382	13.88921157	18.23490268	16.62105244	15.19279548	11.57241968	4.121947837	3.885364879	3.242918022	2.775901999	3.312268573	3.239609602	Dpysl2	dihydropyrimidinase-like 2 [Source:MGI Symbol;Acc:MGI:1349763]
ENSMUSG00000022051	153.4891876	163.8214223	138.4450994	177.0697495	176.1493724	201.9010465	113.8294333	109.4924883	122.3911906	133.0685253	129.8808416	128.8265897	Bnip3l	BCL2/adenovirus E1B interacting protein 3-like [Source:MGI Symbol;Acc:MGI:1332659]
ENSMUSG00000022052	52.78506304	57.48294108	52.9401637	61.80030878	67.36269555	69.50640286	56.38681958	56.43338745	67.81112699	71.18759907	69.46252898	71.62492371	Ppp2r2a	protein phosphatase 2, regulatory subunit B, alpha [Source:MGI Symbol;Acc:MGI:1919228]
ENSMUSG00000022053	0.047889246	0.151654054	0.183857472	0.10055128	0.301891942	0.310049166	0.053443036	0.035580841	0.011143337	0.09638055	0.169417608	0.212203142	Ebf2	early B cell factor 2 [Source:MGI Symbol;Acc:MGI:894332]
ENSMUSG00000022054	3.251368484	3.334384285	3.24079856	2.47309701	2.462158199	2.364364762	0.230029066	0.243090092	0.079938426	0.581654468	0.93959297	0.598035289	Nefm	neurofilament, medium polypeptide [Source:MGI Symbol;Acc:MGI:97314]
ENSMUSG00000022055	6.237857703	6.655467483	6.842435312	11.26198136	11.63646064	10.76306595	0.761477877	0.971062211	0.912362328	3.028934632	2.691564015	2.515869156	Nefl	neurofilament, light polypeptide [Source:MGI Symbol;Acc:MGI:97313]
ENSMUSG00000022056	0.022622759	0	0.023161015	0	0	0	0	0	0	0	0.056493494	0	Adam7	a disintegrin and metallopeptidase domain 7 [Source:MGI Symbol;Acc:MGI:107247]
ENSMUSG00000022057	0.176201834	0.162324376	0.16235473	0.361743044	0.185128249	0.402629246	0	0	0	0	0.02933405	0	Adamdec1	ADAM-like, decysin 1 [Source:MGI Symbol;Acc:MGI:1917650]
ENSMUSG00000022061	0.255881461	0.38771139	0.206818076	0.427298356	0.396191835	0.256447736	0.264515427	0.173438504	0.200559131	0.662547152	0.437201297	0.369990625	Nkx3-1	NK3 homeobox 1 [Source:MGI Symbol;Acc:MGI:97352]
ENSMUSG00000022064	4.463306496	6.05298412	3.719799952	5.645238204	6.491550213	7.234728087	12.85983756	13.55695628	14.60858463	6.813343383	6.340492744	6.570693393	Pibf1	progesterone immunomodulatory binding factor 1 [Source:MGI Symbol;Acc:MGI:1261910]
ENSMUSG00000022066	0	0	0	0	0	0	0	0	0	0	0	0	Entpd4b	ectonucleoside triphosphate diphosphohydrolase 4B [Source:MGI Symbol;Acc:MGI:5435040]
ENSMUSG00000022070	10.15593717	9.866886725	10.06293941	11.85213887	13.0743536	13.78180466	84.02076873	82.568788	93.09309014	32.20208581	31.05554369	28.65657786	Bora	bora, aurora kinase A activator [Source:MGI Symbol;Acc:MGI:1924994]
ENSMUSG00000022074	25.03980388	31.51988542	23.55137505	35.69773707	41.06665305	43.23351453	18.62087765	16.8303347	20.18821746	21.02353432	22.26038067	23.2077678	Tnfrsf10b	tumor necrosis factor receptor superfamily, member 10b [Source:MGI Symbol;Acc:MGI:1341090]
ENSMUSG00000022075	6.488166582	6.467828746	5.959556728	6.616939708	6.353357067	6.777723197	7.848195993	7.0243645	8.472513675	4.841072039	4.80066148	5.387619692	Rhobtb2	Rho-related BTB domain containing 2 [Source:MGI Symbol;Acc:MGI:2180557]
ENSMUSG00000022076	0.304339944	0.544336289	0.271804704	0.513626127	0.700745943	0.636245096	0.028905082	0	0	0.052128171	0.048510441	0.005738586	Klhl1	kelch-like 1 [Source:MGI Symbol;Acc:MGI:2136335]
ENSMUSG00000022085	0	0	0	0	0.125695046	0.12149776	0	0.039504956	0	0	0.132778037	0	Pebp4	phosphatidylethanolamine binding protein 4 [Source:MGI Symbol;Acc:MGI:1920773]
ENSMUSG00000022089	60.95190929	61.28306711	64.09885193	76.58373733	67.84472311	57.41297766	18.13882553	21.89059226	19.59608983	28.605179	27.28418189	28.77647158	Bin3	bridging integrator 3 [Source:MGI Symbol;Acc:MGI:1929883]
ENSMUSG00000022090	154.4298948	139.9679973	173.7894643	154.5300511	145.4714638	119.0541128	8.366871625	7.507961811	6.561082588	13.44892007	12.24421595	15.13038548	Pdlim2	PDZ and LIM domain 2 [Source:MGI Symbol;Acc:MGI:2384850]
ENSMUSG00000022091	53.68706525	47.09836699	55.04513491	52.27750339	48.8859463	42.91006806	10.40255667	11.05866624	10.78641494	8.349166761	7.415366305	8.048572041	Sorbs3	sorbin and SH3 domain containing 3 [Source:MGI Symbol;Acc:MGI:700013]
ENSMUSG00000022092	4.794602929	4.459456406	5.160406452	6.921694751	6.673608899	6.211457245	2.835382086	2.537252839	3.318358269	2.126001952	1.654398534	2.179023125	Ppp3cc	protein phosphatase 3, catalytic subunit, gamma isoform [Source:MGI Symbol;Acc:MGI:107162]
ENSMUSG00000022094	40.03569274	50.94756626	38.47345473	36.09597417	42.47920727	49.69866253	54.29501028	47.80024522	65.27696351	67.58123229	70.6701736	67.38314457	Slc39a14	solute carrier family 39 (zinc transporter), member 14 [Source:MGI Symbol;Acc:MGI:2384851]
ENSMUSG00000022095	6.988261723	6.671308451	6.859611486	7.168486001	7.30865945	7.907047379	7.372451538	7.250516123	8.023732812	7.424656047	7.957332117	7.176512437	Fam160b2	family with sequence similarity 160, member B2 [Source:MGI Symbol;Acc:MGI:3036290]
ENSMUSG00000022096	0.669556622	0.698290297	0.788956918	0.848811041	0.690203334	0.942998832	2.345945287	2.190112551	2.563333832	2.779812016	2.902365717	2.664601091	Hr	lysine demethylase and nuclear receptor corepressor [Source:MGI Symbol;Acc:MGI:96223]
ENSMUSG00000022097	0.231335956	0.133197623	0.059210014	0	0.182291606	0.352408824	0	0.057292806	0	0	0.048140962	0.051253904	Sftpc	surfactant associated protein C [Source:MGI Symbol;Acc:MGI:109517]
ENSMUSG00000022098	177.7397393	180.8380177	182.0136385	117.3159664	118.2158273	115.9644612	10.07131006	9.506275194	10.26796428	67.10823146	68.65815763	66.4112385	Bmp1	bone morphogenetic protein 1 [Source:MGI Symbol;Acc:MGI:88176]
ENSMUSG00000022099	2.535474153	1.820536796	1.87813753	2.212964636	2.381859407	2.260669014	28.82778477	28.8851065	28.66603047	18.94381707	19.42925828	19.72072575	Dmtn	dematin actin binding protein [Source:MGI Symbol;Acc:MGI:99670]
ENSMUSG00000022100	9.668727601	11.97977027	8.022889135	11.24127806	12.88711521	16.90892636	19.12293193	16.99633484	21.55733847	28.60692767	28.88417039	27.64518165	Xpo7	exportin 7 [Source:MGI Symbol;Acc:MGI:1929705]
ENSMUSG00000022101	0.682540455	0.294742657	0.553200763	0.212311846	0.149399598	0.303262584	16.90964495	20.45364536	15.88201647	7.707768546	7.314880716	7.863493895	Fgf17	fibroblast growth factor 17 [Source:MGI Symbol;Acc:MGI:1202401]
ENSMUSG00000022102	2.985962678	3.614822314	2.821852326	1.28605445	1.447953257	1.51623583	14.69607407	15.77611445	15.82012747	11.50529956	13.25606779	12.07768156	Dok2	docking protein 2 [Source:MGI Symbol;Acc:MGI:1332623]
ENSMUSG00000022103	0.946133098	0.805298055	0.589609467	0.230325877	0.605082655	0.391658946	0.403980301	0.183380798	0.306303262	1.471815158	1.129977907	0.838486432	Gfra2	glial cell line derived neurotrophic factor family receptor alpha 2 [Source:MGI Symbol;Acc:MGI:1195462]
ENSMUSG00000022105	24.87868612	26.30354676	22.60331012	28.23980859	33.20881542	36.43096969	13.47604771	15.63788496	18.24724655	26.45030994	26.12845608	26.30300104	Rb1	RB transcriptional corepressor 1 [Source:MGI Symbol;Acc:MGI:97874]
ENSMUSG00000022106	14.69743403	15.89324086	12.38163441	15.30900326	16.05968889	17.84766121	4.273871208	3.660775469	4.716263127	11.06810744	11.88458458	11.3877705	Rcbtb2	regulator of chromosome condensation (RCC1) and BTB (POZ) domain containing protein 2 [Source:MGI Symbol;Acc:MGI:1917200]
ENSMUSG00000022108	1364.726552	1286.47863	1286.335452	904.2083499	881.7622446	795.6038527	137.9790168	147.2100625	141.1023757	392.2451706	390.3801705	395.7554334	Itm2b	integral membrane protein 2B [Source:MGI Symbol;Acc:MGI:1309517]

ENSMUSG00000022109	40.86253571	40.33312321	37.29162248	41.78032224	39.63012806	36.33510207	42.2351158	41.13433553	39.75413015	40.61437335	41.18171134	38.88198569	Med4	mediator complex subunit 4 [Source:MGI Symbol;Acc:MGI:1914631]
ENSMUSG00000022110	90.76940984	101.9898423	85.53441006	102.1714902	112.0722078	100.9386163	111.3126427	108.2443482	109.776487	102.2668994	95.86604849	100.1135113	Sucla2	succinate-Coenzyme A ligase, ADP-forming, beta subunit [Source:MGI Symbol;Acc:MGI:1306775]
ENSMUSG00000022111	25.25861085	21.12066374	25.04740775	24.19276651	22.97089332	20.7133744	269.4422397	291.5281059	236.0714367	62.13138524	63.86814521	62.23312543	Uchl3	ubiquitin carboxyl-terminal esterase L3 (ubiquitin thiolesterase) [Source:MGI Symbol;Acc:MGI:1355274]
ENSMUSG00000022112	0.143770604	0.04730264	0.021027327	0.134164365	0.043158299	0.031287848	0.091682229	0.264504067	0.05734962	0.091856722	0.153867245	0.072807455	Gpc5	glypican 5 [Source:MGI Symbol;Acc:MGI:1194894]
ENSMUSG00000022113	0.705385046	0.785210903	0.481445357	0.877671647	1.605759941	1.235741762	2.644957166	1.910010662	2.932560822	1.135710483	0.704594272	1.021044989	Trim52	tripartite motif-containing 52 [Source:MGI Symbol;Acc:MGI:3045276]
ENSMUSG00000022114	38.28648869	37.58355689	33.13945032	36.30551638	39.35961401	39.87217939	78.43109401	74.34503384	81.46078017	44.03893804	41.18854871	40.7640203	Spry2	sprouty RTK signaling antagonist 2 [Source:MGI Symbol;Acc:MGI:1345138]
ENSMUSG00000022116	0	0	0	0	0	0	0.154898605	0.515635254	0.403721286	0.931160477	0.794325867	0.461285137	4930449E01	RIKEN cDNA 4930449E01 gene [Source:MGI Symbol;Acc:MGI:1922114]
ENSMUSG00000022119	10.66657871	12.95579244	7.415801011	12.48322892	14.77893437	18.08221828	59.8595839	58.99102518	71.13808017	39.15677708	39.69711803	39.53066253	Rbm26	RNA binding motif protein 26 [Source:MGI Symbol;Acc:MGI:1921463]
ENSMUSG00000022120	4.857203534	4.863756135	2.97285124	6.429483261	6.462293359	8.324199107	5.821146599	3.68726658	6.314498124	8.901817989	8.789417295	9.006851721	Rnf219	ring finger protein 219 [Source:MGI Symbol;Acc:MGI:1919736]
ENSMUSG00000022122	1.451072193	1.822891879	1.313709802	0.447641893	0.365396107	0.274030273	0.117771292	0.142561364	0.145105621	0.343259455	0.269524983	0.201930097	Ednrb	endothelin receptor type B [Source:MGI Symbol;Acc:MGI:102720]
ENSMUSG00000022123	0.088390484	0.101786187	0	0.032993819	0.018573672	0.013465087	0	0	0	0.031625281	0.044145697	0.094000587	Scel	sciellin [Source:MGI Symbol;Acc:MGI:1891228]
ENSMUSG00000022124	15.97740533	20.36063563	14.1891143	18.77696074	21.67290168	27.60102112	67.10781088	60.75722754	79.3054627	30.18906298	32.13745328	32.56292985	Fbxl3	F-box and leucine-rich repeat protein 3 [Source:MGI Symbol;Acc:MGI:1354702]
ENSMUSG00000022125	37.3206272	42.5071282	29.98892251	28.47619091	30.97362919	33.72953144	55.18635377	50.64149545	58.76016182	33.60683944	34.51159576	30.23950459	Cln5	ceroid-lipofuscinosis, neuronal 5 [Source:MGI Symbol;Acc:MGI:2442253]
ENSMUSG00000022126	9.973816469	10.93754081	8.985113937	0.964418263	0.792826018	1.212001526	0.541879375	0.487523992	0.473322035	6.294829422	6.281266763	6.207682159	Acod1	aconitate decarboxylase 1 [Source:MGI Symbol;Acc:MGI:103206]
ENSMUSG00000022129	0	0	0	0.019838675	0	0.016192698	0.018979671	0	0	0.038031587	0.070784339	0	Dct	dopachrome tautomerase [Source:MGI Symbol;Acc:MGI:102563]
ENSMUSG00000022130	23.88155518	22.22012881	22.55962986	19.00690577	20.21077761	18.9613019	38.78703951	39.82345633	40.65889639	16.06106832	16.33447816	15.67539473	Tgds	TDP-glucose 4,6-dehydratase [Source:MGI Symbol;Acc:MGI:1923605]
ENSMUSG00000022131	24.64671783	23.67806187	25.28011798	16.27554302	16.46788343	15.55673513	23.78647764	21.75623267	22.83335625	11.80297635	13.61996385	12.2025295	Gpr180	G protein-coupled receptor 180 [Source:MGI Symbol;Acc:MGI:1930949]
ENSMUSG00000022132	0	0.517578506	0.061354109	0.13981016	0.062964232	0.296700744	0.107005165	0.14841869	0.027889381	0.16081323	0.049884228	0.026554948	Cldn10	claudin 10 [Source:MGI Symbol;Acc:MGI:1913101]
ENSMUSG00000022136	15.20769546	17.39678578	13.63297609	11.41886493	13.55106372	18.24533108	14.3915598	13.89630826	17.90955054	11.73879231	11.8646448	11.94944747	Dnajc3	DnaJ heat shock protein family (Hsp40) member C3 [Source:MGI Symbol;Acc:MGI:107373]
ENSMUSG00000022139	52.67353449	65.74620665	44.91970508	62.02901876	74.57072794	93.50330207	27.44638573	25.18100619	34.93621423	31.21226285	30.1275523	33.61502527	Mbnl2	muscleblind like splicing factor 2 [Source:MGI Symbol;Acc:MGI:2145597]
ENSMUSG00000022141	6.018417378	6.983100578	5.245317279	7.077886733	7.925728739	11.26552614	6.461587998	6.020906642	8.440795386	12.5965073	11.69189313	11.88946981	Nipbl	NIPBL cohesin loading factor [Source:MGI Symbol;Acc:MGI:1913976]
ENSMUSG00000022142	18.72637274	25.07151535	17.4610008	26.19085287	30.4069016	40.82774549	42.34697344	40.10388285	51.92579646	67.16883569	69.86565838	66.61839211	Nup155	nucleoporin 155 [Source:MGI Symbol;Acc:MGI:2181182]
ENSMUSG00000022144	7.460301237	7.727782377	6.318872034	10.83072851	11.16057287	12.04271308	0.083646933	0.208836488	0.087205556	1.005673591	0.896884198	0.778434364	Gdnf	glial cell line derived neurotrophic factor [Source:MGI Symbol;Acc:MGI:107430]
ENSMUSG00000022146	37.74666966	44.49918633	34.57177224	35.94680234	37.34786287	48.45259914	0.981888034	0.847405619	1.731377014	16.91818781	15.58582243	16.34095442	Osmr	oncostatin M receptor [Source:MGI Symbol;Acc:MGI:1330819]
ENSMUSG00000022148	5.360451346	8.675336069	3.806984585	5.452941434	8.828560486	13.38916219	0.043114314	0.143521373	0.359588341	7.516168517	8.321092283	8.730766057	Fyb	FYN binding protein [Source:MGI Symbol;Acc:MGI:1346327]
ENSMUSG00000022149	0	0.131897557	0.156352263	0.03562864	0.080227717	0.058161527	0	0	0	0	0	0	C9	complement component 9 [Source:MGI Symbol;Acc:MGI:1098282]
ENSMUSG00000022150	49.65859319	57.80346929	44.9924596	61.55751808	73.00166763	81.41469364	3.979466924	4.038471776	4.211311092	32.19685682	35.0524341	31.30431582	Dab2	disabled 2, mitogen-responsive phosphoprotein [Source:MGI Symbol;Acc:MGI:109175]
ENSMUSG00000022151	12.62491805	12.42720971	13.17318093	13.9116953	13.08279309	11.5130603	12.75348451	11.54735585	10.3986141	14.66502899	15.9793938	14.22320561	Ttc33	tetratricopeptide repeat domain 33 [Source:MGI Symbol;Acc:MGI:1914765]
ENSMUSG00000022155	0.017357196	0	0.053310509	0.064789799	0.09118257	0.092544633	0	0	0.032310731	0	0.014448114	0	Mroh2b	maestro heat-like repeat family member 2B [Source:MGI Symbol;Acc:MGI:1921905]
ENSMUSG00000022156	1.127383961	0.324560146	0.673287667	0.131507023	0.098708119	0.035779499	0.209688068	0.372278146	0.218608906	4.66393479	5.943411652	5.203726077	Gzme	granzyme E [Source:MGI Symbol;Acc:MGI:109265]
ENSMUSG00000022157	3.621926585	2.335667403	3.263129644	2.478609743	1.522165601	2.133437931	1.164086487	1.100330524	1.752993164	7.429775775	6.230769584	6.762063907	Mcpt8	mast cell protease 8 [Source:MGI Symbol;Acc:MGI:1261780]
ENSMUSG00000022159	0.437114797	0.629200198	0.167818096	0.611862519	1.23425885	1.560667649	0.780492273	0.405960454	0.813697047	0.977472162	1.728306372	1.573738924	Rab2b	RAB2B, member RAS oncogene family [Source:MGI Symbol;Acc:MGI:1923588]
ENSMUSG00000022160	12.11839429	12.72918099	7.963775012	16.0079041	14.79701381	17.11982619	38.70726019	31.59422965	38.9821903	34.05690649	30.80728395	32.79937758	Mettl3	methyltransferase like 3 [Source:MGI Symbol;Acc:MGI:1927165]
ENSMUSG00000022174	52.77636036	40.95488568	75.44325869	36.40459478	31.09752974	24.8842564	49.03388009	48.82951798	36.76823705	31.82963626	30.65334423	32.02465909	Dad1	defender against cell death 1 [Source:MGI Symbol;Acc:MGI:101912]
ENSMUSG00000022175	145.2628407	140.3262768	148.5482856	124.091885	107.880832	100.7991681	36.80182725	35.08096251	34.66787018	42.46605075	42.82754297	40.99040917	Lrp10	low-density lipoprotein receptor-related protein 10 [Source:MGI Symbol;Acc:MGI:1929480]
ENSMUSG00000022176	0.746878284	0.334471176	0.807128981	0.658253735	1.046287021	0.853325513	4.630527251	4.110496403	4.286842641	4.193963549	3.384815619	3.456598882	Rem2	rad and gem related GTP binding protein 2 [Source:MGI Symbol;Acc:MGI:2155260]
ENSMUSG00000022177	47.06872143	42.49316833	52.0492158	47.37984709	41.2464	37.64652762	91.73875949	100.3942109	87.80729465	47.04084779	41.8704319	42.54892182	Haus4	HAUS augmin-like complex, subunit 4 [Source:MGI Symbol;Acc:MGI:1261794]
ENSMUSG00000022178	28.89011799	33.86884211	27.06560205	34.1576449	35.38042306	36.55833378	37.04582862	35.44052127	42.26141755	37.59640621	40.23335407	40.42957643	Ajuba	ajuba LIM protein [Source:MGI Symbol;Acc:MGI:1341886]
ENSMUSG00000022179	5.584922094	5.622353201	4.710893695	6.293443057	5.978569447	7.196907389	8.811897138	8.455576319	9.415637256	8.388805732	7.455757753	9.525443947	4931414P19	RIKEN cDNA 4931414P19 gene [Source:MGI Symbol;Acc:MGI:1921609]
ENSMUSG00000022180	1.296371108	2.302781976	1.426050407	0.360351724	0.188367993	0.052522469	0.541747942	0.39620179	0.359415462	2.627543858	3.019178927	2.456639052	Slc7a8	solute carrier family 7 (cationic amino acid transporter, y+ system), member 8 [Source:MGI Symbol;Acc:MGI:1355323]
ENSMUSG00000022181	0	0	0	0	0.069981917	0	0	0.021994761	0.020665197	0	0	0	C6	complement component 6 [Source:MGI Symbol;Acc:MGI:88233]
ENSMUSG00000022184	38.40230359	27.03742355	32.38986257	28.96620829	29.21554908	25.15832336	2.264922032	2.808870889	2.453878648	5.650834369	5.54851019	6.877148129	Fbxo4	F-box protein 4 [Source:MGI Symbol;Acc:MGI:2146220]
ENSMUSG00000022185	76.86747449	79.21075205	85.83547746	86.10802345	86.07186741	83.37612034	156.4826185	145.4151877	147.2913205	130.8880967	126.9564248	133.9202702	Acin1	apoptotic chromatin condensation inducer 1 [Source:MGI Symbol;Acc:MGI:1891824]
ENSMUSG00000022186	68.05589585	67.12813713	64.61421091	72.70564701	76.73001431	77.93302162	43.46651841	40.65471023	45.43147969	46.55264141	49.84342643	50.00810839	Oxct1	3-oxoacid CoA transferase 1 [Source:MGI Symbol;Acc:MGI:1914291]
ENSMUSG00000022187	0.061978376	0.089214108	0.015863252	0.028918601	0.048838659	0.05900976	0	0	0	0	0	0	Gm5546	predicted gene 5546 [Source:MGI Symbol;Acc:MGI:3648205]
ENSMUSG00000022191	38.6825714	37.93987024	36.67680521	37.99783162	43.46004398	42.61517333	89.74534834	84.53415383	96.89781064	85.55605264	88.35263824	93.35448596	Drosha	drosha, ribonuclease type III [Source:MGI Symbol;Acc:MGI:1261425]
ENSMUSG00000022193	35.45185528	29.29997639	50.94084123	34.71877584	30.17848321	20.41377895	50.17656366	39.54513597	24.20842273	29.78897791	26.35670206	28.9128594	Psmb5	proteasome (prosome, macropain) subunit, beta type 5 [Source:MGI Symbol;Acc:MGI:1194513]
ENSMUSG00000022194	17.2846005	16.71944903	18.48695566	15.21885124	15.68190249	15.5815996	25.45256399	26.06703541	18.35901558	14.04192346	12.32263013	14.38093476	Pabpn1	poly(A) binding protein, nuclear 1 [Source:MGI Symbol;Acc:MGI:1859158]
ENSMUSG00000022195	10.41725095	11.35470438	8.418054663	10.68415722	12.76915577	14.11017029	19.66805433	17.62149623	22.09030094	23.26566068	23.02805584	22.39781954	6030458C11	RIKEN cDNA 6030458C11 gene [Source:MGI Symbol;Acc:MGI:1925127]
ENSMUSG00000022197	2.282454168	2.161098717	1.4020559	1.703958981	1.634249622	2.498294697	4.347142622	3.768493412	5.350377807	4.249555423	4.461276828	4.225335608	Pdzd2	PDZ domain containing 2 [Source:MGI Symbol;Acc:MGI:1922394]
ENSMUSG00000022199	15.16690428	13.19512865	16.47188562	10.78449541	9.152983742	9.370410825	8.268039727	7.347273338	6.683989721	7.687063051	8.394835384	8.798563508	Slc22a17	solute carrier family 22 (organic cation transporter), member 17 [Source:MGI Symbol;Acc:MGI:1926225]
ENSMUSG00000022200	55.50986408	67.43243284	47.17280774	61.84964347	70.89805044	70.98778307	44.35386202	43.92622258	51.87338395	57.65234673	55.72542829	55.74029036	Golph3	golgi phosphoprotein 3 [Source:MGI Symbol;Acc:MGI:1913879]
ENSMUSG00000022201	80.10197818	101.273356	68.21593174	97.04604263	116.7023172	151.313643	73.97695949	69.4603476	89.46000344	145.6371656	131.268093	136.8601212	Zfr	zinc finger RNA binding protein [Source:MGI Symbol;Acc:MGI:1341890]
ENSMUSG00000022203	6.010262437	6.633743754	6.025513198	1.610796839	2.731290379	1.900861516	4.270380105	4.182236386	4.548840313	10.45444036	10.95799042	11.40853939	Efs	embryonal Fyn-associated substrate [Source:MGI Symbol;Acc:MGI:105311]
ENSMUSG00000022204	100.9627487	74.15516246	109.7547531	95.99833182	77.76447934	58.61292428	140.3518815	149.0449862	124.9998262	115.4638991	114.3572936	117.9126677	Ngdn	neuroguidin, EIF4E binding protein [Source:MGI Symbol;Acc:MGI:1916216]
ENSMUSG00000022205	101.3101083	77.08554675	115.8587819	92.89376567	78.97459328	65.59877505	297.8637833	324.8406319	244.0766402	177.0684769	163.504591	176.8811902	Sub1	SUB1 homolog, transcriptional regulator [Source:MGI Symbol;Acc:MGI:104811]
ENSMUSG00000022206	1.318693725	1.223271243	1.287565769	0.797600298	0.974980759	1.450836328	0.370631396	0.47173895	0.409128676	1.518112181	1.453408965	1.211942487	Npr3	natriuretic peptide receptor 3 [Source:MGI Symbol;Acc:MGI:97373]
ENSMUSG00000022208	0.932678319	0.689300929	0.755344309	0.597560654	0.409522133	0.530152388	1.478928384	1.69620811	1.451150104	2.054517778	2.549243806	2.356319895	Jph4	junctophilin 4 [Source:MGI Symbol;Acc:MGI:2443113]
ENSMUSG00000022209	0	0	0	0	0	0.185688502	0	0	0.037817896	0	0.067642824	0.072016817	Dhrs2	dehydrogenase/reductase member 2 [Source:MGI Symbol;Acc:MGI:1918662]
ENSMUSG00000022210	40.23805412	40.23086483	39.82414836	38.43484649	38.17200421	29.75592016	28.10113839	28.85952335	25.03721275	25.00969021	23.97082717	24.08654291	Dhrs4	dehydrogenase/reductase (SDR family) member 4 [Source:MGI Symbol;Acc:MGI:90169]
ENSMUSG00000022211	0.150554541	0.104022776	0.231204952	0.168594029	0.268908822	0.240816807	1.370998991	1.02910549	1.233143924	0.579070173	0.81458954	1.147456194	Carmil3	capping protein regulator and myosin 1 linker 3 [Source:MGI Symbol;Acc:MGI:2448573]
ENSMUSG00000022212	0	0.021885828	0	0	0	0.014476158	0.050903075	0	0.035379109	0.03399997	0.015820175	0	Cpne6	copine VI [Source:MGI Symbol;Acc:MGI:1334445]
ENSMUSG00000022214	10.63159491	11.8657389	11.7338566	11.22504632	10.20685016	10.15978012	14.32517389	13.58144716	14.36567739	16.98860007	15.30076568	14.97456531	Dcaf11	DDB1 and CUL4 associated factor 11 [Source:MGI Symbol;Acc:MGI:90168]
ENSMUSG00000022215	0.044313582	0.153088102	0	0	0.186234061	0.135011414	0.593432349	1.185270739	0.412452642	0.198187262	0.479525624	0.863979303	Fitm1	fat storage-inducing transmembrane protein 1 [Source:MGI Symbol;Acc:MGI:1915930]
ENSMUSG00000022216	193.0982877	141.7531094	219.9357418	187.1191627	154.6102217	127.3827699	119.3604394	134.0194448	96.32511444	96.36211285	92.93861654	96.13061071	Psme1	proteasome (prosome, macropain) activator subunit 1 (PA28 alpha) [Source:MGI Symbol;Acc:MGI:1096367]
ENSMUSG00000022217	13.61844396	10.2880588	18.24386119	13.74501767	10.50294265	8.717668789	8.967777381	8.611282364	6.697332858	5.399546348	5.547394855	5.692117086	Emc9	ER membrane protein complex subunit 9 [Source:MGI Symbol;Acc:MGI:1934682]
ENSMUSG00000022218	1.013627511	0.904614243	1.06368802	0.236475134	0.479240124	0.849267908	31.42416508	30.22468824	29.01086948	9.859991385	9.681946905	9.499539861	Tgm1	transglutaminase 1, K polypeptide [Source:MGI Symbol;Acc:MGI:98730]

ENSMUSG00000022219	0.491754147	0.707850223	0.503454288	0.573621112	0.387499872	0.436986942	0.292684602	0.852516954	0.41956254	0.769759327	0.44344703	0.980560406	Cideb	cell death-inducing DNA fragmentation factor, alpha subunit-like effector B [Source:MGI Symbol;Acc:MGI:1270844]
ENSMUSG00000022220	1.640144712	1.608901919	1.907203303	2.380865039	2.233822789	2.174652663	0.216930941	0.401184449	0.207313255	1.122943019	1.112430015	1.345867224	Adcy4	adenylate cyclase 4 [Source:MGI Symbol;Acc:MGI:99674]
ENSMUSG00000022221	107.094135	100.6163871	101.4085591	111.4714818	101.3964817	96.48782422	31.88392323	31.68806418	29.09589889	59.74372816	60.02257802	61.00459409	Ripk3	receptor-interacting serine-threonine kinase 3 [Source:MGI Symbol;Acc:MGI:2154952]
ENSMUSG00000022223	0.980706275	1.058750334	1.255049863	1.315569787	1.738781475	0.980419421	4.104151076	4.675484719	4.278755505	4.166788382	2.704123077	4.074028279	Sdr39u1	short chain dehydrogenase/reductase family 39U, member 1 [Source:MGI Symbol;Acc:MGI:1916876]
ENSMUSG00000022225	0	0	0.122141174	0.074220846	0	0	0	0.078790846	0	0	0	0	Cma1	chymase 1, mast cell [Source:MGI Symbol;Acc:MGI:96941]
ENSMUSG00000022226	0	0	0	0	0	0	0	0	0	0	0	0	Mcpt2	mast cell protease 2 [Source:MGI Symbol;Acc:MGI:96938]
ENSMUSG00000022227	0	0	0	0	0	0	0	0	0	0.075913149	0	0.07521283	Mcpt1	mast cell protease 1 [Source:MGI Symbol;Acc:MGI:96937]
ENSMUSG00000022228	5.30843971	6.005276881	4.988050541	6.36910549	6.745229536	6.749454274	5.063860532	5.01177881	4.863525644	8.15848249	7.583631047	7.935915944	Zscan26	zinc finger and SCAN domain containing 26 [Source:MGI Symbol;Acc:MGI:3531417]
ENSMUSG00000022229	0.019540639	0	0.020005563	0.109410021	0.020530571	0.014883751	0.017445438	0.135504511	0.127313379	0.332094169	0.097593666	0.294395722	Atp12a	ATPase, H+/K+ transporting, nongastric, alpha polypeptide [Source:MGI Symbol;Acc:MGI:1926943]
ENSMUSG00000022231	7.570931309	8.343603841	7.762168613	10.20284993	10.56418228	15.82934801	0.41639494	0.494275017	0.353345174	3.434519812	4.017774033	3.691211344	Sema5a	sema domain, seven thrombospondin repeats (type 1 and type 1-like), transmembrane domain (TM) and short cytoplasmic domain,
ENSMUSG00000022234	432.5656952	378.9704544	414.7916769	442.5068796	419.7783607	359.2781019	868.0738889	905.7884008	847.2783799	670.1849557	663.3606632	663.3803786	Cct5	chaperonin containing Tcp1, subunit 5 (epsilon) [Source:MGI Symbol;Acc:MGI:107185]
ENSMUSG00000022235	10.8801386	7.80703931	13.99372624	12.04094126	9.995231757	10.20283997	3.026329037	3.141422067	1.933758269	0.73356988	1.00123413	1.695872508	Cmbl	carboxymethylenebutenolidase-like (Pseudomonas) [Source:MGI Symbol;Acc:MGI:1916824]
ENSMUSG00000022236	5.485345366	2.278138649	4.326971284	4.279681023	3.354013169	2.054797532	1.123945574	0.757198966	0.669578291	0.683694282	1.38476948	0.836772203	Ropn1l	ropporin 1-like [Source:MGI Symbol;Acc:MGI:2182357]
ENSMUSG00000022237	2.484445857	2.860967118	2.056493252	3.379008884	3.41561619	3.764583452	4.801856759	4.267821361	5.485848151	4.574583227	4.323105671	4.157610923	Ankrd33b	ankyrin repeat domain 33B [Source:MGI Symbol;Acc:MGI:1917904]
ENSMUSG00000022240	2.718978065	3.313433867	2.324229152	1.79828459	1.85825603	2.533451902	0.589184878	0.444564056	0.90909117	2.455673928	2.834587078	2.573386206	Ctnnd2	catenin (cadherin associated protein), delta 2 [Source:MGI Symbol;Acc:MGI:1195966]
ENSMUSG00000022241	85.13930648	86.09521879	80.27530901	103.3333577	108.9231225	104.5933941	100.9072648	95.63038225	107.7625866	119.6364689	124.2132637	126.4846762	Tars	threonyl-tRNA synthetase [Source:MGI Symbol;Acc:MGI:106314]
ENSMUSG00000022243	0.115396548	0.033221264	0.073838837	0.094225392	0.075776598	0.07690853	0.012877926	0.01428959	0	0	0.012006998	0.025566815	Slc45a2	solute carrier family 45, member 2 [Source:MGI Symbol;Acc:MGI:2153040]
ENSMUSG00000022244	11.74235369	11.26826991	10.5121569	10.88271829	9.464171318	9.413597205	9.406253988	8.817309306	8.209452244	9.112415714	8.993271538	8.553174884	Amacr	alpha-methylacyl-CoA racemase [Source:MGI Symbol;Acc:MGI:1098273]
ENSMUSG00000022245	0.11889607	0.068457468	0.024344985	0.022190372	0.049967746	0.018112197	0.021229541	0.035335049	0.066398151	0.053174864	0.029690695	0.073758036	Skor1	SKI family transcriptional corepressor 1 [Source:MGI Symbol;Acc:MGI:2443473]
ENSMUSG00000022246	54.86288325	67.16871929	49.74259892	68.21428999	75.45655457	85.47883176	35.4598653	34.969899	42.8828179	75.76096428	72.03662583	73.31867269	Rai14	retinoic acid induced 14 [Source:MGI Symbol;Acc:MGI:1922896]
ENSMUSG00000022247	75.28917135	71.44577625	68.1125656	78.47273523	78.2054524	70.33233227	168.5370658	186.0383323	173.9572877	156.9712789	148.6382548	149.2749854	Brix1	BRX1, biogenesis of ribosomes [Source:MGI Symbol;Acc:MGI:1915082]
ENSMUSG00000022248	7.975107658	8.970170932	9.050965199	10.59605665	10.9123527	8.55452167	21.52556478	21.12919175	21.0027878	14.83851194	15.12954223	14.1172104	Rad1	RAD1 checkpoint DNA exonuclease [Source:MGI Symbol;Acc:MGI:1316678]
ENSMUSG00000022249	0.09576073	0.073515602	0.261437687	0.119149786	0.100611984	0.048626143	0.028497666	0.031621549	0	0.114207615	0.053140765	0	Ttc23l	tetratricopeptide repeat domain 23-like [Source:MGI Symbol;Acc:MGI:1923027]
ENSMUSG00000022253	4.991304588	6.257395973	2.441753297	2.478043204	4.081665314	6.105331844	5.948814545	5.821472914	9.451599759	12.33814236	6.058168652	4.772060644	Nadk2	NAD kinase 2, mitochondrial [Source:MGI Symbol;Acc:MGI:1915896]
ENSMUSG00000022255	65.77732147	80.15088628	54.30900648	76.30294001	85.82350558	105.3054257	66.27736817	58.86617538	79.51433141	110.1414804	102.3291165	108.6949212	Mtdh	metadherin [Source:MGI Symbol;Acc:MGI:1914404]
ENSMUSG00000022257	298.5469738	290.4246438	285.2458632	346.4268803	334.9595779	309.0803402	104.4897187	103.7910196	111.506713	87.61943714	88.9902163	86.06021983	Laptm4b	lysosomal-associated protein transmembrane 4B [Source:MGI Symbol;Acc:MGI:1890494]
ENSMUSG00000022261	42.17450593	45.46136449	37.082827	46.49469903	49.39973318	51.39583446	2.178330256	2.707170367	2.872819772	9.930266345	10.09412368	11.49284879	Sdc2	syndecan 2 [Source:MGI Symbol;Acc:MGI:1349165]
ENSMUSG00000022262	0.022013679	0.015843677	0.019720263	0.064196262	0.046257792	0.077549323	0.002456664	0	0.007683536	0.019690716	0.043519897	0.036579495	Dnah5	dynein, axonemal, heavy chain 5 [Source:MGI Symbol;Acc:MGI:107718]
ENSMUSG00000022263	42.12882947	55.53332519	33.65058333	63.26995372	78.63379093	112.476091	5.760843293	4.963283636	6.95879273	21.35291285	23.46044079	23.61655741	Trio	triple functional domain (PTPRF interacting) [Source:MGI Symbol;Acc:MGI:1927230]
ENSMUSG00000022265	37.58828715	43.6878354	28.20646852	48.80762851	55.50344741	61.14990098	1.661566701	1.418235228	1.791477758	6.317433297	6.143818936	7.344637095	Ank	progressive ankylosis [Source:MGI Symbol;Acc:MGI:3045421]
ENSMUSG00000022269	0.059637544	0.103013546	0.1526412	0.111305552	0	0.136274514	1.09148234	1.270208906	0.666098154	0.240049686	0.446780152	0.290687423	Marchf11	membrane associated ring-CH-type finger 11 [Source:MGI Symbol;Acc:MGI:3608327]
ENSMUSG00000022270	8.322382419	10.48666997	6.914727103	9.04102411	10.07287046	11.4834311	4.561905069	3.658655946	4.261549927	7.534681247	7.832967477	7.666933218	Retreg1	reticulophagy regulator 1 [Source:MGI Symbol;Acc:MGI:1913520]
ENSMUSG00000022272	43.35641316	51.65341449	43.40477564	41.11523778	52.88659561	64.44189578	65.34916656	62.04764113	77.32952665	117.2866825	122.1907233	117.05577	Myo10	myosin X [Source:MGI Symbol;Acc:MGI:107716]
ENSMUSG00000022280	12.90126572	15.96316036	9.093696318	15.55862339	20.4240626	23.13590763	10.26996099	10.01094359	13.45814127	13.81919875	13.6358663	14.31113073	Rnf19a	ring finger protein 19A [Source:MGI Symbol;Acc:MGI:1353623]
ENSMUSG00000022283	848.6318615	927.4367419	756.0081935	927.7292735	980.0084983	1024.29437	2057.236623	1988.86237	2295.366755	2081.543654	2042.119122	2152.679922	Pabpc1	poly(A) binding protein, cytoplasmic 1 [Source:MGI Symbol;Acc:MGI:1349722]
ENSMUSG00000022285	341.1005188	346.6396356	303.6918702	362.0857475	377.0758984	384.6635054	403.3399214	376.190021	419.4621659	391.792707	388.8390116	380.5007632	Ywhaz	tyrosine 3-monooxygenase/tryptophan 5-monooxygenase activation protein, zeta polypeptide [Source:MGI Symbol;Acc:MGI:10948
ENSMUSG00000022286	1.86490688	2.655135589	1.21499508	2.642092513	3.17063856	2.970064912	4.343996501	3.459781177	4.150089382	10.25132595	9.793897393	9.510688995	Grhl2	grainyhead like transcription factor 2 [Source:MGI Symbol;Acc:MGI:2182543]
ENSMUSG00000022288	0.073302364	0	0	0	0	0	0	0	0	0	0	0	4930447A16	RIKEN cDNA 4930447A16 gene [Source:MGI Symbol;Acc:MGI:1922142]
ENSMUSG00000022292	8.069766507	10.92430266	7.126563024	14.05515437	15.95397348	19.44857774	6.352062484	7.170418189	7.683015467	8.154930598	7.640249744	9.116957886	Rrm2b	ribonucleotide reductase M2 B (TP53 inducible) [Source:MGI Symbol;Acc:MGI:2155865]
ENSMUSG00000022295	45.73745558	50.67399219	34.95800821	51.79387112	55.89813301	56.93466396	55.0350435	49.95899134	59.16485196	56.238639	60.51636062	57.00394683	Atp6v1c1	ATPase, H+ transporting, lysosomal V1 subunit C1 [Source:MGI Symbol;Acc:MGI:1913585]
ENSMUSG00000022296	0	0	0	0	0	0	0	0	0	0	0	0	Baalc	brain and acute leukemia, cytoplasmic [Source:MGI Symbol;Acc:MGI:1928704]
ENSMUSG00000022297	0.125814292	0.072440812	0.021467958	0.195679717	0.066094033	0.239576174	0.84243101	0.830913211	0.975856519	2.700909921	2.216736445	2.099911418	Fzd6	frizzled class receptor 6 [Source:MGI Symbol;Acc:MGI:108474]
ENSMUSG00000022299	0.954556897	1.14149908	0.375872442	1.096340692	1.311504116	2.586686782	2.58939716	2.582282583	4.442312029	4.646797988	4.90495607	4.961832735	Slc25a32	solute carrier family 25, member 32 [Source:MGI Symbol;Acc:MGI:1917156]
ENSMUSG00000022300	24.49469734	20.72615919	22.90006012	21.56190454	20.47599355	18.20534031	30.53554641	30.1132838	29.74733548	26.27120771	25.17056817	26.3365791	Dcaf13	DDB1 and CUL4 associated factor 13 [Source:MGI Symbol;Acc:MGI:2684929]
ENSMUSG00000022303	0	0	0	0	0	0	0.15112059	0.100611757	0.189059724	1.059857453	1.183563155	0.990075417	Dcstamp	dendrocyte expressed seven transmembrane protein [Source:MGI Symbol;Acc:MGI:1923016]
ENSMUSG00000022304	0.055094095	0.063443682	0.05640493	0	0.028942585	0.104910437	8.091220864	9.79684976	6.897068988	7.638456834	8.530011318	8.007421727	Dpys	dihydropyrimidinase [Source:MGI Symbol;Acc:MGI:1928679]
ENSMUSG00000022305	7.823361423	10.55871663	5.327419749	7.802969421	8.219694245	10.81077995	5.142271959	3.857301481	6.3130022	9.549766468	9.32015442	10.43168702	Lrp12	low density lipoprotein-related protein 12 [Source:MGI Symbol;Acc:MGI:2443132]
ENSMUSG00000022306	5.627490738	6.931263496	3.642385742	7.767599456	10.22653067	12.30517051	0.459460033	0.421160222	0.406114641	4.513279468	3.967235817	4.283259185	Zfpm2	zinc finger protein, multitype 2 [Source:MGI Symbol;Acc:MGI:1334444]
ENSMUSG00000022307	24.18917863	32.30782943	19.92022147	29.93798491	34.70856119	40.52036281	20.87044185	18.52309098	25.24230478	33.9395321	33.64170208	32.04603562	Oxr1	oxidation resistance 1 [Source:MGI Symbol;Acc:MGI:2179326]
ENSMUSG00000022309	5.068295112	5.870633457	4.443266687	3.1484765	3.435519875	4.20005584	0	0.014723954	0	0.199419515	0.16083571	0.197579817	Angpt1	angiopoietin 1 [Source:MGI Symbol;Acc:MGI:108448]
ENSMUSG00000022311	0.419328237	0.529608152	0.360062403	0.53647369	0.667963207	1.06636628	0	0.00670007	0	0.120993293	0.033778883	0.035963129	Csmd3	CUB and Sushi multiple domains 3 [Source:MGI Symbol;Acc:MGI:2386403]
ENSMUSG00000022312	387.197528	368.605015	372.4253163	400.7108356	369.8300188	339.7558528	564.609018	608.4242064	559.2088777	426.474046	412.5555288	418.2621475	Eif3h	eukaryotic translation initiation factor 3, subunit H [Source:MGI Symbol;Acc:MGI:1915385]
ENSMUSG00000022313	4.864685726	6.588191966	5.962486094	6.361896568	6.946819619	6.653961196	8.563820971	9.672266397	8.704950928	11.36865886	10.20895018	11.32450115	Utp23	UTP23 small subunit processome component [Source:MGI Symbol;Acc:MGI:1925831]
ENSMUSG00000022314	52.02601311	76.55943287	41.50791703	78.37715109	95.08612995	117.7211871	125.2855443	111.0212846	144.7645131	152.3454959	158.382465	150.9882971	Rad21	RAD21 cohesin complex component [Source:MGI Symbol;Acc:MGI:108016]
ENSMUSG00000022315	0	0.025139277	0.067050609	0.04074427	0	0.016628118	0	0	0.040638408	0	0	0	Slc30a8	solute carrier family 30 (zinc transporter), member 8 [Source:MGI Symbol;Acc:MGI:2442682]
ENSMUSG00000022321	0.892002216	0.831531852	0.594321243	0.36995636	0.431405473	0.496085867	0.101124986	0.042078823	0.06589199	0.975180458	1.249288845	0.903444928	Cdh10	cadherin 10 [Source:MGI Symbol;Acc:MGI:107436]
ENSMUSG00000022322	14.12795543	14.2586309	13.74914176	21.53048531	22.20908024	22.09484537	51.74735024	49.51729249	53.8988581	45.33507649	45.96722596	45.13107821	Shcbp1	Shc SH2-domain binding protein 1 [Source:MGI Symbol;Acc:MGI:1338802]
ENSMUSG00000022323	23.38092096	20.98567721	21.96134238	18.15763887	18.31601721	14.74830929	25.64756434	28.36499424	24.61844902	21.11178155	21.04241434	23.24424852	Rida	reactive intermediate imine deaminase A homolog [Source:MGI Symbol;Acc:MGI:1095401]
ENSMUSG00000022324	26.20565937	27.40225225	23.68367389	23.55520786	24.36850202	24.34248225	3.6438311	3.177915842	3.939031004	7.261117912	7.53445648	7.634589248	Matn2	matrilin 2 [Source:MGI Symbol;Acc:MGI:109613]
ENSMUSG00000022325	10.06429037	9.842717341	8.153399528	7.976250173	8.398019815	10.62100465	27.32012334	22.71451952	29.89431581	63.74650973	63.03770084	60.05609328	Pop1	processing of precursor 1, ribonuclease P/MRP family, (S. cerevisiae) [Source:MGI Symbol;Acc:MGI:1914974]
ENSMUSG00000022329	16.51680105	19.95001615	13.62291548	22.68643234	26.36975115	28.34468326	9.067419802	8.381149782	11.35133154	13.34707046	11.29466932	12.23974979	Stk3	serine/threonine kinase 3 [Source:MGI Symbol;Acc:MGI:1928487]
ENSMUSG00000022330	4.009216057	3.218767747	3.52649247	3.688639696	3.797024113	3.50535785	0.050413194	0.083909149	0.052557942	0	0.235018868	0.150129546	Osr2	odd-skipped related 2 [Source:MGI Symbol;Acc:MGI:1930813]
ENSMUSG00000022332	6.37988882	6.259579766	7.234644813	2.482894574	2.164228006	1.874046393	25.28637336	26.15934669	23.08684466	23.15931487	21.50441393	20.38487724	Khdrbs3	KH domain containing, RNA binding, signal transduction associated 3 [Source:MGI Symbol;Acc:MGI:1313312]
ENSMUSG00000022335	2.469570604	2.87285625	2.992715094	3.256958524	2.753537399	3.20542398	2.351011567	2.246750391	3.518227074	3.144404866	3.555467288	3.014900224	Zfat	zinc finger and AT hook domain containing [Source:MGI Symbol;Acc:MGI:2681865]
ENSMUSG00000022336	106.90998	105.5217079	101.5065881	112.1077947	109.8894402	103.8403603	143.443974	150.3961424	140.7696457	131.2533945	128.9567058	130.0765185	Eif3e	eukaryotic translation initiation factor 3, subunit E [Source:MGI Symbol;Acc:MGI:99257]
ENSMUSG00000022337	49.53057043	47.60423834	43.20383649	46.69700756	47.08362637	40.29990686	53.26859765	53.45597049	50.63992715	38.43094716	36.61280605	35.42123147	Emc2	ER membrane protein complex subunit 2 [Source:MGI Symbol;Acc:MGI:1913986]
ENSMUSG00000022338	24.85944647	28.06043453	30.65526252	29.77845574	34.2163568	31.97465428	25.32686644	23.00236294	22.40554666	23.1162481	20.93867644	22.49608859	Eny2	ENY2 transcription and export complex 2 subunit [Source:MGI Symbol;Acc:MGI:1919286]

ENSMUSG00000022339	14.50691279	17.00555381	13.81450107	17.67185364	18.49707776	17.55593568	8.711847794	9.064446752	9.136381135	8.18452044	7.688817818	8.878859369	Ebag9	estrogen receptor-binding fragment-associated gene 9 [Source:MGI Symbol;Acc:MGI:1859920]
ENSMUSG00000022340	0.070239125	0.226475096	0	0.22285639	0.250911333	0.171199585	0	0.041749116	0.065375696	0.150785373	0.292334929	0.19919245	Sybu	syntabulin (syntaxin-interacting) [Source:MGI Symbol;Acc:MGI:2442392]
ENSMUSG00000022342	0	0	0	0	0	0	0	0	0	0.053325897	0.049625044	0.079250928	Kcnv1	potassium channel, subfamily V, member 1 [Source:MGI Symbol;Acc:MGI:1914748]
ENSMUSG00000022346	40.06441289	41.44296456	36.79450289	58.45535005	57.40574064	51.77617956	41.65652086	41.10873946	42.60107794	43.76846233	43.58884961	45.92585192	Myc	myelocytomatosis oncogene [Source:MGI Symbol;Acc:MGI:97250]
ENSMUSG00000022347	0	0	0	0	0	0.097060278	0	0	0	0	0	0	A1bg	alpha-1-B glycoprotein [Source:MGI Symbol;Acc:MGI:2152878]
ENSMUSG00000022350	44.6456138	46.71896602	39.98609241	44.58412316	48.55204845	56.0390425	29.86795329	29.39978792	33.69137882	42.53811316	43.23654692	44.0718237	Washc5	WASH complex subunit 5 [Source:MGI Symbol;Acc:MGI:2146110]
ENSMUSG00000022351	20.49343867	23.83078789	18.08327452	29.89139202	32.11058126	32.73867841	43.86915578	43.94354105	47.0455166	21.34684835	21.34593082	21.13621101	Sqle	squalene epoxidase [Source:MGI Symbol;Acc:MGI:109296]
ENSMUSG00000022353	15.59236491	16.8166448	11.39566494	20.02308649	22.88202965	26.52050628	40.59121673	37.13529222	46.74898286	40.13363035	40.43038002	42.1887294	Mtss1	MTSS I-BAR domain containing 1 [Source:MGI Symbol;Acc:MGI:2384818]
ENSMUSG00000022354	12.66415046	8.954731739	24.86942868	13.33841863	13.61696336	8.292222089	16.19903485	11.5185165	7.513524124	7.021900922	5.918106792	6.103890079	Ndufb9	NADH:ubiquinone oxidoreductase subunit B9 [Source:MGI Symbol;Acc:MGI:1913468]
ENSMUSG00000022357	0	0	0	0	0	0	0	0	0.018105403	0	0	0	Klhl38	kelch-like 38 [Source:MGI Symbol;Acc:MGI:3045310]
ENSMUSG00000022358	0.45026944	0.504302696	0.151555907	0.241749723	0.499002367	0.568471402	0.038547015	0.01833107	0.063150898	0.215170684	0.241312091	0.174921581	Fbxo32	F-box protein 32 [Source:MGI Symbol;Acc:MGI:1914981]
ENSMUSG00000022359	13.99582534	15.51752304	10.00649232	19.48307191	20.59895151	16.87157245	9.293916308	8.536628101	9.366333824	13.55355805	16.51234985	14.40234707	Wdyhv1	WDYHV motif containing 1 [Source:MGI Symbol;Acc:MGI:1924023]
ENSMUSG00000022360	3.373938819	9.341166609	1.984335594	7.971744849	14.51131206	30.80561099	12.21533398	8.291914985	21.19384528	42.99556604	41.5652629	42.54802579	Atad2	ATPase family, AAA domain containing 2 [Source:MGI Symbol;Acc:MGI:1917722]
ENSMUSG00000022361	0	0.015660403	0.013922962	0.088835128	0	0.031075241	0.024282461	0	0	0	0.124521375	0.072312714	Zhx1	zinc fingers and homeoboxes 1 [Source:MGI Symbol;Acc:MGI:109271]
ENSMUSG00000022362	0	0	0	0	0	0	0	0	0	0	0	0	Gm29394	predicted gene 29394 [Source:MGI Symbol;Acc:MGI:5580100]
ENSMUSG00000022364	10.15108692	10.81714778	8.462306278	14.62554298	14.62726106	15.61766711	18.53110918	17.6607092	22.84493854	20.01193557	19.81418698	19.18580416	Tbc1d31	TBC1 domain family, member 31 [Source:MGI Symbol;Acc:MGI:2684931]
ENSMUSG00000022365	72.3893539	74.20150457	75.29049105	71.73106422	67.63325723	69.49456606	28.28764775	29.44520897	30.08647855	39.76581263	42.75661696	43.02706837	Derl1	Der1-like domain family, member 1 [Source:MGI Symbol;Acc:MGI:1915069]
ENSMUSG00000022366	0.025952043	0	0	0	0.109067113	0	0	0	0	0	0	0	Slc22a22	solute carrier family 22 (organic cation transporter), member 22 [Source:MGI Symbol;Acc:MGI:2446114]
ENSMUSG00000022367	4.346517062	5.239127449	3.493404783	3.013643132	3.606422375	4.710733672	0.271995663	0.231388836	0.236306063	5.223172212	4.911400673	5.606985986	Has2	hyaluronan synthase 2 [Source:MGI Symbol;Acc:MGI:107821]
ENSMUSG00000022369	13.11043241	13.16465367	11.18115339	20.67824664	19.16689585	21.97285951	24.43002308	23.34903698	27.23523419	19.73351357	17.2909054	16.51204352	Mtbp	Mdm2, transformed 3T3 cell double minute p53 binding protein [Source:MGI Symbol;Acc:MGI:2146005]
ENSMUSG00000022370	72.16861841	57.58601064	83.2071772	71.57325077	59.48462941	50.70996426	54.6307914	63.54959893	48.32999112	38.48796637	38.72146635	35.94159384	Mrpl13	mitochondrial ribosomal protein L13 [Source:MGI Symbol;Acc:MGI:2137218]
ENSMUSG00000022371	0.807304317	0.958404558	0.664618088	0.566964	0.67331538	0.87481912	0.059442714	0.098938138	0.054225157	0.282890275	0.31868013	0.18439509	Col14a1	collagen, type XIV, alpha 1 [Source:MGI Symbol;Acc:MGI:1341272]
ENSMUSG00000022372	1.331381296	1.314132002	1.393015732	0	0.061487002	0.033431507	0	0.014493658	0.068087643	0.562727832	0.633280355	0.363047035	Sla	src-like adaptor [Source:MGI Symbol;Acc:MGI:104295]
ENSMUSG00000022375	0.082588268	0.095104637	0	0	0	0.06290599	0.073732925	0	0.211391875	0.073873256	0.085933003	0	Lrrc6	leucine rich repeat containing 6 (testis) [Source:MGI Symbol;Acc:MGI:1859553]
ENSMUSG00000022376	0.021368228	0.008202204	0.029168846	0.013293653	0.014967164	0	0	0	0	0.006371125	0.005928965	0.018937049	Adcy8	adenylate cyclase 8 [Source:MGI Symbol;Acc:MGI:1341110]
ENSMUSG00000022377	29.62539737	39.35667969	26.28300433	37.56672377	41.48508569	51.23639903	9.856812849	8.316096934	10.73059257	23.41398247	24.84369657	23.50505545	Asap1	ArfGAP with SH3 domain, ankyrin repeat and PH domain1 [Source:MGI Symbol;Acc:MGI:1342335]
ENSMUSG00000022378	29.03368801	27.58402806	24.52372118	33.35193213	31.9601504	34.47160173	62.20230687	61.84029888	64.96073294	59.41119587	55.32144556	53.27574272	Fam49b	family with sequence similarity 49, member B [Source:MGI Symbol;Acc:MGI:1923520]
ENSMUSG00000022382	0.048564885	0.037283307	0.082867288	0.045319952	0.05102519	0.036990994	0.245693198	0.176404938	0.301348017	1.665205468	1.347512245	1.248142479	Wnt7b	wingless-type MMTV integration site family, member 7B [Source:MGI Symbol;Acc:MGI:98962]
ENSMUSG00000022383	0.228570984	0.493521072	0.204758135	0.346610366	0.600376074	0.435245958	0.306094455	0.339648189	0.159558373	0.281120604	0.404307359	0.430451115	Ppara	peroxisome proliferator activated receptor alpha [Source:MGI Symbol;Acc:MGI:104740]
ENSMUSG00000022385	26.7496186	28.29543018	26.06466097	32.98697785	28.61328885	28.44839062	75.89346311	76.46121312	78.41644891	62.86223529	66.9870922	64.36983011	Gtse1	G two S phase expressed protein 1 [Source:MGI Symbol;Acc:MGI:1352755]
ENSMUSG00000022386	15.71924037	12.80614526	14.84421517	16.20151232	14.88867191	12.86657299	13.40540164	12.31826569	12.18507445	9.19178259	11.32703805	8.899063908	Trmu	tRNA 5-methylaminomethyl-2-thiouridylate methyltransferase [Source:MGI Symbol;Acc:MGI:1919276]
ENSMUSG00000022387	25.54497578	32.21697755	21.79976966	28.67878832	32.02106976	34.50987786	33.87491677	34.31970426	39.11519898	36.05394478	39.18621001	35.1636627	Brd1	bromodomain containing 1 [Source:MGI Symbol;Acc:MGI:1924161]
ENSMUSG00000022388	0.019269363	0	0	0	0.060736657	0.044031376	0.017203249	0.057267149	0.107610797	0.086179952	0.160398009	0.290308724	Ttll8	tubulin tyrosine ligase-like family, member 8 [Source:MGI Symbol;Acc:MGI:1922902]
ENSMUSG00000022389	33.81026352	32.71874287	33.40396139	29.54209363	30.33003458	32.45066878	30.67232534	29.34845187	33.13439316	25.30604131	23.77695328	23.56769374	Tef	thyrotroph embryonic factor [Source:MGI Symbol;Acc:MGI:98663]
ENSMUSG00000022390	18.48185128	23.09578122	16.39870593	19.82761616	22.13668531	30.07326615	29.10142143	24.00501127	36.04805907	29.76908885	32.01069145	30.28308307	Zc3h7b	zinc finger CCCH type containing 7B [Source:MGI Symbol;Acc:MGI:1328310]
ENSMUSG00000022391	88.568366	92.68155646	89.08398345	102.7830825	102.4286301	100.3989037	182.2943909	181.0709995	189.7826663	149.8655945	156.9252988	154.1849632	Rangap1	RAN GTPase activating protein 1 [Source:MGI Symbol;Acc:MGI:103071]
ENSMUSG00000022394	11.82680007	11.39563057	11.5915151	11.81466431	12.40289407	11.93301924	51.16756697	48.21220306	48.15701137	55.50431866	53.89882689	53.70886423	L3mbtl2	L3MBTL2 polycomb repressive complex 1 subunit [Source:MGI Symbol;Acc:MGI:2443584]
ENSMUSG00000022400	112.802288	99.09903125	125.3516041	109.7063485	93.88784213	81.99496753	126.6566532	130.6439372	99.68358281	73.10602753	79.22928579	71.28134377	Rbx1	ring-box 1 [Source:MGI Symbol;Acc:MGI:1891829]
ENSMUSG00000022401	6.869248291	8.559818008	6.393350918	10.16995857	10.64596635	12.22888317	9.172085944	9.099900071	12.52473465	17.13579402	17.67368294	18.90577999	Xpnpep3	X-prolyl aminopeptidase 3, mitochondrial [Source:MGI Symbol;Acc:MGI:2445217]
ENSMUSG00000022403	146.9374579	153.5573932	146.2380391	148.8777553	153.1193618	147.2229816	236.439537	230.9774999	238.4535688	235.0778417	225.8403971	224.7482855	St13	suppression of tumorigenicity 13 [Source:MGI Symbol;Acc:MGI:1917606]
ENSMUSG00000022404	79.31325981	86.17904659	62.931786	81.81062266	82.70426061	83.91240235	80.87898541	82.17743177	79.59838298	88.61307098	89.11993704	97.95026281	Slc25a17	solute carrier family 25 (mitochondrial carrier, peroxisomal membrane protein), member 17 [Source:MGI Symbol;Acc:MGI:1342248]
ENSMUSG00000022407	43.63003741	42.6631109	41.73371071	50.12580539	55.39506113	47.97512889	129.8396795	140.3568209	144.3877305	94.73230539	96.22839818	91.97681698	Adsl	adenylosuccinate lyase [Source:MGI Symbol;Acc:MGI:103202]
ENSMUSG00000022408	0.603807714	0.463543748	0.574018629	0.295147515	0.558873297	0.284705826	0.333707386	0.128176622	0.561999453	0.578669725	0.574410298	0.394961592	Fam83f	family with sequence similarity 83, member F [Source:MGI Symbol;Acc:MGI:2146227]
ENSMUSG00000022412	11.27253275	13.55527707	12.28969267	13.99475928	14.21930879	17.64073058	15.55593782	16.0423399	19.15797897	21.77217263	22.90453204	21.20295589	Mief1	mitochondrial elongation factor 1 [Source:MGI Symbol;Acc:MGI:2146020]
ENSMUSG00000022414	22.83144252	19.02581552	23.24147441	20.78997152	17.35890077	18.00960922	18.46700468	19.36366929	18.31423785	14.45844239	16.59542303	14.72858223	Tab1	TGF-beta activated kinase 1/MAP3K7 binding protein 1 [Source:MGI Symbol;Acc:MGI:1913763]
ENSMUSG00000022415	9.378732486	8.672801612	11.66773602	8.115564212	7.465045962	7.403378506	38.21186937	39.61332082	34.70573765	18.3033922	17.81380927	18.21009913	Syngr1	synaptogyrin 1 [Source:MGI Symbol;Acc:MGI:1328323]
ENSMUSG00000022416	0.125896096	0.080542126	0.028642555	0.078322779	0.05878845	0.063928519	0.074931445	0.12471802	0.02603976	0.137635771	0.244523532	0.309922827	Cacna1i	calcium channel, voltage-dependent, alpha 1I subunit [Source:MGI Symbol;Acc:MGI:2178051]
ENSMUSG00000022419	2.00362605	1.865762158	1.126947476	1.650459135	1.650318031	1.987731634	10.78795988	10.29195518	12.25994434	9.303932084	10.73785603	10.86223298	Deptor	DEP domain containing MTOR-interacting protein [Source:MGI Symbol;Acc:MGI:2146322]
ENSMUSG00000022420	40.40045389	34.72569573	42.54735966	40.90167929	36.64400959	31.14549787	26.44500824	27.9318443	26.20192936	17.33153765	17.83428272	18.90855212	Dnal4	dynein, axonemal, light chain 4 [Source:MGI Symbol;Acc:MGI:1859217]
ENSMUSG00000022421	15.26209031	17.0186504	15.96305862	20.57714517	21.75596147	20.44000137	7.144257988	6.257862056	6.263507401	1.549638745	1.883549738	1.420453311	Nptxr	neuronal pentraxin receptor [Source:MGI Symbol;Acc:MGI:1920590]
ENSMUSG00000022422	3.53108475	2.167030413	3.290389204	4.775008175	3.865477834	3.994082122	11.06196781	12.02321208	12.06394589	6.581725945	5.227327773	5.883897824	Dscc1	DNA replication and sister chromatid cohesion 1 [Source:MGI Symbol;Acc:MGI:1919357]
ENSMUSG00000022425	1.610941758	2.419672494	1.649270355	1.677600539	1.717082086	1.600467638	0.187592885	0.370056193	0.152112892	1.273882772	1.127172424	0.972461487	Enpp2	ectonucleotide pyrophosphatase/phosphodiesterase 2 [Source:MGI Symbol;Acc:MGI:1321390]
ENSMUSG00000022426	21.76440954	24.41465959	21.87245446	22.20113804	24.6674458	25.66663128	13.76904508	13.40731605	14.98350524	12.0834313	13.20226689	14.3330947	Josd1	Josephin domain containing 1 [Source:MGI Symbol;Acc:MGI:1921408]
ENSMUSG00000022427	112.3034769	96.677059	115.2894939	117.3528324	104.8722497	85.57290113	144.8185232	157.1773875	134.4623753	103.5490908	98.84290484	97.5456788	Tomm22	translocase of outer mitochondrial membrane 22 [Source:MGI Symbol;Acc:MGI:2450248]
ENSMUSG00000022428	34.89402794	33.59289195	40.43387763	33.92366837	33.47891111	26.15084828	45.0260154	52.85160517	42.13776438	27.95019201	26.52410075	26.69800498	Cby1	chibby family member 1, beta catenin antagonist [Source:MGI Symbol;Acc:MGI:1920989]
ENSMUSG00000022429	2.23289488	2.745029267	3.212786831	3.519764887	3.265397468	4.274872257	13.84658835	13.12254358	14.60417868	5.532982543	5.073638742	5.268009975	Dmc1	DNA meiotic recombinase 1 [Source:MGI Symbol;Acc:MGI:105393]
ENSMUSG00000022431	0.194824977	0.032050139	0.256449063	0.285697449	0.263179085	0.275590083	0.124239502	0.165430198	0.077715042	0.174266342	0.115837243	0.221989743	Ribc2	RIB43A domain with coiled-coils 2 [Source:MGI Symbol;Acc:MGI:1914997]
ENSMUSG00000022432	6.438569347	6.871830634	5.607015422	9.873505727	11.83830964	11.97627305	5.608007894	6.728350048	5.88313266	3.546792521	3.888876078	3.844603986	Smc1b	structural maintenance of chromosomes 1B [Source:MGI Symbol;Acc:MGI:2154049]
ENSMUSG00000022433	92.28753415	85.71247601	85.70709902	93.41829537	79.82848828	80.51771056	164.8964144	165.1182011	174.4414938	69.00903591	64.91591134	65.33363554	Csnk1e	casein kinase 1, epsilon [Source:MGI Symbol;Acc:MGI:1351660]
ENSMUSG00000022434	17.20479061	18.97560115	18.35792161	22.34172788	23.17663596	21.80742586	25.14802036	24.92777893	27.96986023	31.39885514	25.70128824	27.50953984	Fam118a	family with sequence similarity 118, member A [Source:MGI Symbol;Acc:MGI:1920475]
ENSMUSG00000022435	0	0	0	0	0	0	0.14058501	0	0	0.105639432	0.032769328	0.139553174	Upk3a	uroplakin 3A [Source:MGI Symbol;Acc:MGI:98914]
ENSMUSG00000022436	13.64394232	9.693687057	18.80630608	11.00935356	9.501966547	5.863570238	6.677200089	8.804175089	6.320484277	4.758512206	4.324073694	3.438894677	Sh3bp1	SH3-domain binding protein 1 [Source:MGI Symbol;Acc:MGI:104603]
ENSMUSG00000022437	71.97492509	58.70266251	82.56192637	68.684349	62.04225126	50.24555309	167.7122684	171.619419	140.2157289	102.8958286	103.6475255	100.4767372	Samm50	SAMM50 sorting and assembly machinery component [Source:MGI Symbol;Acc:MGI:1915903]
ENSMUSG00000022438	24.58770733	24.85340784	27.2354785	26.13162843	22.53239994	25.67298283	140.8849529	120.4011047	124.5973603	41.45092886	39.96708486	39.13162362	Parvb	parvin, beta [Source:MGI Symbol;Acc:MGI:2153063]
ENSMUSG00000022439	0.379663128	0.340045695	0.259130884	0.196830774	0.177287523	0.096394178	0.338954595	0.417900468	0.274847118	1.697998516	1.545041687	1.981415636	Parvg	parvin, gamma [Source:MGI Symbol;Acc:MGI:2158329]
ENSMUSG00000022440	35.84753673	34.85597575	37.08121592	26.7511867	24.58803485	22.16168816	1.711300015	0.538491427	1.145022284	3.582656444	4.405666199	3.574959815	C1qtnf6	C1q and tumor necrosis factor related protein 6 [Source:MGI Symbol;Acc:MGI:1919959]
ENSMUSG00000022441	0.26365495	0.333973451	0.242935203	0.049207693	0.110804702	0.281149687	0.423693243	0.208950234	0.122699606	0.660332756	0.285305993	0.514046509	Efcab6	EF-hand calcium binding domain 6 [Source:MGI Symbol;Acc:MGI:1924877]

ENSMUSG00000022442	21.74866062	22.73518937	20.93281865	24.18444942	22.60415917	21.76337811	16.99834039	16.02339674	15.41843848	15.39984611	15.58857958	13.94205474	Ttll1	tubulin tyrosine ligase-like 1 [Source:MGI Symbol;Acc:MGI:2443047]
ENSMUSG00000022443	645.9330589	840.1519892	571.8093915	723.7820123	820.3811338	942.5111868	146.3087601	129.2202473	173.1320866	431.7998785	455.5495686	454.5470924	Myh9	myosin, heavy polypeptide 9, non-muscle [Source:MGI Symbol;Acc:MGI:107717]
ENSMUSG00000022445	0.328461739	0.966614809	0.186820399	0.476801264	0.191723148	0.166788909	0.162912867	0.433850867	0.271749968	0.097933757	0.212653205	0.258747445	Cyp2d26	cytochrome P450, family 2, subfamily d, polypeptide 26 [Source:MGI Symbol;Acc:MGI:1923529]
ENSMUSG00000022449	0.066590386	0.038341126	0.011362458	0.031070527	0.093285155	0.025360346	0.158534347	0.230885427	0.206598667	0.426871951	0.711348829	0.649154426	Adamts20	a disintegrin-like and metallopeptidase (reprolysin type) with thrombospondin type 1 motif, 20 [Source:MGI Symbol;Acc:MGI:266062
ENSMUSG00000022450	138.8754314	117.1005066	169.9851049	141.8265043	139.357752	90.50440561	167.5451525	177.464309	115.1504962	94.63096319	82.28023505	90.3295045	Ndufa6	NADH:ubiquinone oxidoreductase subunit A6 [Source:MGI Symbol;Acc:MGI:1914380]
ENSMUSG00000022451	99.94205668	117.548319	89.82285058	109.6745259	114.5838494	123.1931272	92.7677859	92.35600335	96.21737644	92.60313895	82.27085493	90.15997944	Twf1	twinfilin actin binding protein 1 [Source:MGI Symbol;Acc:MGI:1100520]
ENSMUSG00000022452	130.9181583	105.5221166	184.5299814	100.234978	80.78893927	62.20994658	152.4500598	155.4264706	102.1995015	81.10982543	76.74097209	80.29094932	Smdt1	single-pass membrane protein with aspartate rich tail 1 [Source:MGI Symbol;Acc:MGI:1916279]
ENSMUSG00000022453	73.03995413	61.50564124	69.01342918	73.65487891	65.94010419	57.24633857	27.20860012	32.54506499	26.82764292	15.06251745	15.39308694	15.28978411	Naga	N-acetyl galactosaminidase, alpha [Source:MGI Symbol;Acc:MGI:1261422]
ENSMUSG00000022454	1.697569947	2.181018784	1.781049568	1.885262735	1.444543592	1.69907704	0.789088949	0.875588003	1.005472666	2.120791104	1.891859742	1.914726874	Nell2	NEL-like 2 [Source:MGI Symbol;Acc:MGI:1858510]
ENSMUSG00000022455	0.304518668	0.03506689	0.062352796	0.284171818	0.223961945	0.487087356	1.821511127	0.844673557	1.303794034	1.198492937	0.988576181	0.809615811	Wbp2nl	WBP2 N-terminal like [Source:MGI Symbol;Acc:MGI:1921966]
ENSMUSG00000022456	1.201993009	1.2852884	0.835044317	0.640960072	0.962198994	0.632156035	3.206560815	3.090267642	3.156517762	5.503766392	5.705448405	6.13778712	Septin3	septin 3 [Source:MGI Symbol;Acc:MGI:1345148]
ENSMUSG00000022462	44.17846969	57.39393597	38.79508671	50.66880026	60.91544009	71.88021564	27.71316588	25.36348696	34.31828741	65.95385923	65.03275665	67.24923169	Slc38a2	solute carrier family 38, member 2 [Source:MGI Symbol;Acc:MGI:1915010]
ENSMUSG00000022463	55.64586396	60.76992783	52.31326483	73.07897448	74.9806253	75.05700082	131.8779732	123.1455273	151.0921108	60.12361664	66.12102532	63.58295032	Srebf2	sterol regulatory element binding factor 2 [Source:MGI Symbol;Acc:MGI:107585]
ENSMUSG00000022464	47.81329743	53.75711703	42.73406722	78.97554233	87.67452277	95.234174	94.3206607	82.54556597	106.2849096	101.0211847	103.5836205	101.5695282	Slc38a4	solute carrier family 38, member 4 [Source:MGI Symbol;Acc:MGI:1916604]
ENSMUSG00000022466	31.83904655	33.93107098	32.49243986	35.59705482	35.62515236	35.20172587	56.69899265	59.68962282	55.45024368	40.67168078	38.72399776	40.08612676	Rpap3	RNA polymerase II associated protein 3 [Source:MGI Symbol;Acc:MGI:1277218]
ENSMUSG00000022468	0.023283814	0.34856261	0.238377977	0.08691229	0.17124363	0.266022895	0	0.04613187	0.086686475	0.041653643	0.019381426	0	Endou	endonuclease, polyU-specific [Source:MGI Symbol;Acc:MGI:97746]
ENSMUSG00000022469	23.07177894	19.68565218	22.86879616	27.32043577	24.45346943	21.10346002	21.9771855	23.85943565	24.46793366	5.142300392	5.586505004	5.184638601	Rapgef3	Rap guanine nucleotide exchange factor (GEF) 3 [Source:MGI Symbol;Acc:MGI:2441741]
ENSMUSG00000022471	47.65244473	46.88715359	40.41410416	51.89986837	51.01117712	48.03595263	132.9803754	129.8285769	135.2505674	94.9752802	95.54730663	91.63280082	Xrcc6	X-ray repair complementing defective repair in Chinese hamster cells 6 [Source:MGI Symbol;Acc:MGI:95606]
ENSMUSG00000022472	49.96261301	46.11869745	52.55498231	45.02503948	41.91187847	39.62202372	33.87192096	34.42455102	33.05527396	26.50764351	26.35788447	24.95462145	Desi1	desumoylating isopeptidase 1 [Source:MGI Symbol;Acc:MGI:106313]
ENSMUSG00000022474	112.6499082	79.29098052	122.6460631	91.88144572	75.45445304	64.30395034	337.4400883	361.3842628	297.969488	219.4224987	223.0588331	220.3385276	Pmm1	phosphomannomutase 1 [Source:MGI Symbol;Acc:MGI:1353418]
ENSMUSG00000022475	35.14651101	37.57883754	34.12365436	35.75747017	34.00992757	35.80085679	11.07804507	9.424614033	10.56954672	10.41646796	10.73410947	10.63523213	Hdac7	histone deacetylase 7 [Source:MGI Symbol;Acc:MGI:1891835]
ENSMUSG00000022476	25.96220343	23.32169284	28.67917755	23.93312132	22.27271989	17.97285003	28.58583349	31.7193808	28.80367862	31.71589018	33.4273332	32.68135307	Polr3h	polymerase (RNA) III (DNA directed) polypeptide H [Source:MGI Symbol;Acc:MGI:1926179]
ENSMUSG00000022477	190.9039064	221.8293699	170.0573256	219.2047257	216.1439126	224.1432725	310.0465145	290.4611018	332.289469	252.9274287	263.391792	253.3017681	Aco2	aconitase 2, mitochondrial [Source:MGI Symbol;Acc:MGI:87880]
ENSMUSG00000022479	5.781038331	7.078502232	5.693828257	4.677743591	4.882190842	5.434467708	0.457319691	0.507450568	0.204332406	1.53820954	1.309630651	1.134907878	Vdr	vitamin D (1,25-dihydroxyvitamin D3) receptor [Source:MGI Symbol;Acc:MGI:103076]
ENSMUSG00000022483	0.66489937	0.71097539	0.510539323	0.531834144	0.498988061	0.524529028	1.526418043	1.505548705	1.967094087	2.017856957	2.68824231	2.73580442	Col2a1	collagen, type II, alpha 1 [Source:MGI Symbol;Acc:MGI:88452]
ENSMUSG00000022484	4.598120229	3.971228858	3.784478278	2.250614001	1.75244398	1.733984329	0.362214444	0.290275547	0.293707764	1.673389842	1.688590253	1.797779687	Hoxc10	homeobox C10 [Source:MGI Symbol;Acc:MGI:96192]
ENSMUSG00000022485	2.967481919	2.94103965	3.561035488	1.770475563	1.686686835	1.852686299	0.260586913	0.168672094	0.181115439	1.479469595	1.397040366	1.789163985	Hoxc5	homeobox C5 [Source:MGI Symbol;Acc:MGI:96196]
ENSMUSG00000022487	10.20021374	7.628195185	11.28313765	8.424571644	8.006922868	5.626058038	6.594375495	6.271923095	4.310467262	3.460770818	2.244654322	3.844461781	Gtsf1	gametocyte specific factor 1 [Source:MGI Symbol;Acc:MGI:1921424]
ENSMUSG00000022488	2.465525598	4.537501431	2.674024606	0.850976375	0.910790296	0.951835764	3.045447615	3.479661037	3.604633209	6.646273628	7.675044746	6.634846054	Nckap1l	NCK associated protein 1 like [Source:MGI Symbol;Acc:MGI:1926063]
ENSMUSG00000022489	5.018253827	5.758781477	5.155428796	3.354225146	3.667018559	4.100059079	5.332812672	4.954093679	6.012221294	9.66082126	10.03103777	9.048485858	Pde1b	phosphodiesterase 1B, Ca2+-calmodulin dependent [Source:MGI Symbol;Acc:MGI:97523]
ENSMUSG00000022490	0.323522465	0.791674592	0.041402491	0.452858773	0.382401189	0.061605302	18.01598889	20.39150045	14.6796702	13.16693586	11.07495492	11.89861312	Ppp1r1a	protein phosphatase 1, regulatory inhibitor subunit 1A [Source:MGI Symbol;Acc:MGI:1889595]
ENSMUSG00000022491	0.044359266	0	0	0	0	0	0	0	0	0	0	0	Glycam1	glycosylation dependent cell adhesion molecule 1 [Source:MGI Symbol;Acc:MGI:95759]
ENSMUSG00000022494	0	0	0	0.065753512	0	0	0	0	0.043721781	0.105043576	0	0	Shisa9	shisa family member 9 [Source:MGI Symbol;Acc:MGI:1919805]
ENSMUSG00000022496	0	0	0	0	0.110940659	0	0.094269581	0	0	0	0.087894173	0.04678884	Tnfrsf17	tumor necrosis factor receptor superfamily, member 17 [Source:MGI Symbol;Acc:MGI:1343050]
ENSMUSG00000022498	14.07875825	13.86656618	12.23544877	11.23705955	9.441190102	11.37867103	14.87287354	14.32820446	19.12921072	14.8606877	14.45110126	16.0675732	Txndc11	thioredoxin domain containing 11 [Source:MGI Symbol;Acc:MGI:1923620]
ENSMUSG00000022500	162.3924529	174.7697589	171.2497024	139.0407695	136.9587408	125.9815032	57.26437874	53.05064741	58.20808358	81.57775481	78.72482491	85.82864184	Litaf	LPS-induced TN factor [Source:MGI Symbol;Acc:MGI:1929512]
ENSMUSG00000022501	0	0	0	0	0	0.281927059	0.082612589	0	0	0.08276982	0.077025539	0.082006247	Prm1	protamine 1 [Source:MGI Symbol;Acc:MGI:97765]
ENSMUSG00000022503	7.192261774	7.562062704	7.523458428	11.49743479	11.30207959	9.76551487	23.00424401	21.83955133	20.6648965	13.78965656	15.30546714	15.7686212	Nubp1	nucleotide binding protein 1 [Source:MGI Symbol;Acc:MGI:1347073]
ENSMUSG00000022504	0.024714812	0.028460377	0	0.011531728	0.012983434	0	0.055162054	0.048967085	0.046007067	0.143694303	0.030858882	0.131417248	Ciita	class II transactivator [Source:MGI Symbol;Acc:MGI:108445]
ENSMUSG00000022505	13.41674126	14.73278337	14.18134249	16.48765278	16.7038088	18.08897003	3.636700339	3.408500689	3.598165957	6.402869406	6.26301522	6.037125765	Emp2	epithelial membrane protein 2 [Source:MGI Symbol;Acc:MGI:1098726]
ENSMUSG00000022507	22.00780122	23.6846312	19.99664009	20.96014563	22.18761017	25.67442083	16.15186418	14.76434965	18.28494545	25.35950248	28.48916572	27.57787729	1810013L24	RIKEN cDNA 1810013L24 gene [Source:MGI Symbol;Acc:MGI:1916303]
ENSMUSG00000022508	6.346384635	7.613803286	5.528681441	5.577769246	7.189201604	6.688396274	0.824132026	0.617268913	1.084731645	3.117019535	3.054375544	2.99622984	Bcl6	B cell leukemia/lymphoma 6 [Source:MGI Symbol;Acc:MGI:107187]
ENSMUSG00000022510	0.120595538	0.180533549	0.061732413	0.078776442	0.050681971	0.082669895	0.02153299	0	0	0.064721917	0.060230172	0.021374947	Trp63	transformation related protein 63 [Source:MGI Symbol;Acc:MGI:1330810]
ENSMUSG00000022512	0.151344789	0.174281305	0.166864584	0.206416688	0.195707006	0.336962333	0.031180888	0.023065935	0.021671619	0.333229146	0.2228864	0.299202986	Cldn1	claudin 1 [Source:MGI Symbol;Acc:MGI:1276109]
ENSMUSG00000022514	16.47802416	22.46197985	13.56771921	15.33442576	19.13520405	22.15839019	1.765887917	1.703225173	2.704875509	21.77536994	20.3148049	23.03076422	Il1rap	interleukin 1 receptor accessory protein [Source:MGI Symbol;Acc:MGI:104975]
ENSMUSG00000022515	37.90895103	35.84034841	39.60126741	34.69351484	32.87101523	30.79334328	26.51676896	26.80719034	26.58598066	27.439485	28.47762904	26.89828121	Anks3	ankyrin repeat and sterile alpha motif domain containing 3 [Source:MGI Symbol;Acc:MGI:1919865]
ENSMUSG00000022516	15.60946874	11.49720363	15.90457864	11.36812749	8.337118449	6.753434555	26.50791229	27.5314999	19.14037371	12.86264504	12.7762361	12.47985972	Nudt16l1	nudix (nucleoside diphosphate linked moiety X)-type motif 16-like 1 [Source:MGI Symbol;Acc:MGI:1914161]
ENSMUSG00000022517	40.85331151	46.41128366	38.16496516	32.9207189	34.33310406	36.10664912	28.8390421	25.7092562	34.67358267	37.39214397	39.25080021	37.84485834	Mgrn1	mahogunin, ring finger 1 [Source:MGI Symbol;Acc:MGI:2447670]
ENSMUSG00000022518	0.34110919	0.056114967	0.24944649	0.113684818	0.127996371	0.63098341	0.021752465	0.048273893	0.022677889	0.108969327	0.263657634	0.107964056	4930562C1	RIKEN cDNA 4930562C15 gene [Source:MGI Symbol;Acc:MGI:1926059]
ENSMUSG00000022519	2.15913559	2.816178307	1.872164243	2.138229234	2.731480576	3.842934319	10.70029729	9.123197306	13.14465239	19.23412964	20.76021661	19.21289207	Srl	sarcalumenin [Source:MGI Symbol;Acc:MGI:2146620]
ENSMUSG00000022521	8.841296586	10.29789757	9.362886265	10.02359411	11.40521897	13.31414439	7.452278137	6.888899143	10.12723614	14.88760312	16.71173024	15.76055289	Crebbp	CREB binding protein [Source:MGI Symbol;Acc:MGI:1098280]
ENSMUSG00000022523	0.06895591	0.079406275	0.14119311	0.064348522	0	0.087537448	0	0	0.021393778	0.246717733	0.210462409	0.224071555	Fgf12	fibroblast growth factor 12 [Source:MGI Symbol;Acc:MGI:109183]
ENSMUSG00000022525	0.25854027	0.085063546	0.378130903	0.241265532	0.11641627	0.08439662	0.098922371	0	0	0.165184405	0	0.065464214	Plaat1	phospholipase A and acyltransferase 1 [Source:MGI Symbol;Acc:MGI:1351473]
ENSMUSG00000022526	8.519704289	10.2143154	8.445250038	10.56592255	10.22290248	11.58371196	4.336243705	3.880813669	4.609654376	5.783165931	6.50854399	5.758040382	Zfp251	zinc finger protein 251 [Source:MGI Symbol;Acc:MGI:1918841]
ENSMUSG00000022528	100.3199006	84.60683308	94.3012654	61.57239428	52.51216922	47.5177653	77.63224688	85.96410511	78.59227646	41.59702215	43.94800459	40.2041906	Hes1	hes family bHLH transcription factor 1 [Source:MGI Symbol;Acc:MGI:104853]
ENSMUSG00000022529	7.659790545	9.905956529	7.017483106	11.1297573	11.48662171	13.01302213	13.41689646	11.33973169	15.90163444	16.29339237	16.93135452	17.35798887	Zfp263	zinc finger protein 263 [Source:MGI Symbol;Acc:MGI:1921370]
ENSMUSG00000022533	43.67298232	54.49079566	35.16192615	45.96204164	52.22923968	75.09475048	47.83308002	43.02908788	57.91893454	103.9190263	98.6693507	105.2149518	Atp13a3	ATPase type 13A3 [Source:MGI Symbol;Acc:MGI:2685387]
ENSMUSG00000022534	0	0.017658416	0	0	0	0.023359958	0	0	0	0	0	0	Mefv	Mediterranean fever [Source:MGI Symbol;Acc:MGI:1859396]
ENSMUSG00000022536	31.12668865	40.72361049	29.82916743	36.53090697	38.97835511	40.70217104	52.64586908	46.24608658	59.26639145	59.88147577	57.1696988	57.46399385	Glyr1	glyoxylate reductase 1 homolog (Arabidopsis) [Source:MGI Symbol;Acc:MGI:1921272]
ENSMUSG00000022537	5.459208375	4.972096723	5.91089401	5.310571463	4.884097446	4.29678625	4.903395445	5.063970373	5.235183206	3.107448265	2.382283529	3.005476917	Tmem44	transmembrane protein 44 [Source:MGI Symbol;Acc:MGI:1924489]
ENSMUSG00000022538	39.48816921	33.43024282	41.34777661	34.94369115	34.70739883	34.39197862	81.23039712	81.01608902	79.94621766	72.98097927	77.50499827	76.48302213	Lsg1	large 60S subunit nuclear export GTPase 1 [Source:MGI Symbol;Acc:MGI:107236]
ENSMUSG00000022540	18.05184152	12.67704251	21.57166585	15.90674225	14.79997217	11.72110779	70.54171965	72.99749709	64.56341896	25.88805441	30.24974078	29.47825644	Rogdi	rogdi homolog [Source:MGI Symbol;Acc:MGI:1913299]
ENSMUSG00000022542	0.081493348	0.03128126	0	0.028540605	0.02069067	0.072755405	0.161461544	0.075850666	0.048595917	0.022611664	0.07222141		Septin12	septin 12 [Source:MGI Symbol;Acc:MGI:1918339]
ENSMUSG00000022543	0.438693164	0.336785153	1.160254678	0.54584167	0.576146793	1.085970098	0.228465572	0.688097796	0.136105868	0.294300508	0.517321056	0.421179066	4930451G0	RIKEN cDNA 4930451G09 gene [Source:MGI Symbol;Acc:MGI:1921934]
ENSMUSG00000022544	12.73291987	11.21528925	9.766686268	10.28048227	9.855245651	10.61051317	11.01130789	9.687692659	11.47976607	6.962108945	8.472451992	7.216245414	Eef2kmt	eukaryotic elongation factor 2 lysine methyltransferase [Source:MGI Symbol;Acc:MGI:1917761]
ENSMUSG00000022545	5.708046125	5.714836403	6.439407929	5.530221282	5.825321967	6.161571265	12.49659385	11.97557614	12.31760976	9.268331572	11.74224562	11.30937858	Ercc4	excision repair cross-complementing rodent repair deficiency, complementation group 4 [Source:MGI Symbol;Acc:MGI:1354163]
ENSMUSG00000022546	5.35885929	3.448501087	7.180678146	5.56461506	5.023146483	4.842071425	4.878076707	6.11542992	5.110056574	3.477545191	4.02338528	2.956580522	Gpt	glutamic pyruvic transaminase, soluble [Source:MGI Symbol;Acc:MGI:95802]

ENSMUSG00000022548	127.8630633	107.9954216	140.7669631	28.60935296	23.8884864	18.91954828	9.996592707	11.72210812	10.33086068	21.25585416	17.66423177	19.41311511	Apod	apolipoprotein D [Source:MGI Symbol;Acc:MGI:88056]
ENSMUSG00000022550	27.03071671	21.82271276	27.54096185	25.19433903	21.54725279	16.80719007	15.03567818	15.65519444	14.5276334	15.47065315	15.80156271	14.2351341	Adck5	aarF domain containing kinase 5 [Source:MGI Symbol;Acc:MGI:2679274]
ENSMUSG00000022551	172.7666044	140.810812	199.0226255	171.6637308	148.5556116	118.9167878	374.659281	392.9151812	327.2054731	218.1262753	220.527956	221.8979752	Cyc1	cytochrome c-1 [Source:MGI Symbol;Acc:MGI:1913695]
ENSMUSG00000022552	46.2005793	41.08355328	48.59883977	46.28271643	40.38557493	36.7819451	22.25596055	22.77321959	20.14614734	25.10229897	26.31126932	24.14312266	Sharpin	SHANK-associated RH domain interacting protein [Source:MGI Symbol;Acc:MGI:1913331]
ENSMUSG00000022553	78.78455518	81.49096936	88.62950013	92.57486548	83.85334888	73.00475236	83.15588129	80.67969223	72.37484804	77.60141432	74.54411462	80.02539153	Maf1	MAF1 homolog, negative regulator of RNA polymerase III [Source:MGI Symbol;Acc:MGI:1916127]
ENSMUSG00000022554	16.7819866	16.04556616	18.16818873	15.21088977	14.50164998	14.8183963	21.94575676	25.08934824	19.73909301	21.4388163	24.94780329	22.05650699	Hgh1	HGH1 homolog [Source:MGI Symbol;Acc:MGI:1930628]
ENSMUSG00000022555	48.61391654	45.73801442	56.44194557	50.96402956	46.78408906	36.5556384	36.70650119	36.17049096	33.9358796	28.12582158	30.69402911	33.54962288	Dgat1	diacylglycerol O-acyltransferase 1 [Source:MGI Symbol;Acc:MGI:1333825]
ENSMUSG00000022556	38.71498843	34.86249954	37.56435691	37.56934312	39.30549955	33.90975403	30.54646503	29.93832744	29.21902825	27.69895105	26.06475592	28.43449886	Hsf1	heat shock factor 1 [Source:MGI Symbol;Acc:MGI:96238]
ENSMUSG00000022557	80.27929885	66.53291851	81.72237227	64.91242442	62.66064619	56.25434814	163.3310006	172.9886434	157.0832445	147.3558712	148.2983175	149.8367138	Bop1	block of proliferation 1 [Source:MGI Symbol;Acc:MGI:1334460]
ENSMUSG00000022558	64.66557668	67.46806905	66.18737401	60.72697657	56.87609141	59.89648766	21.24734791	21.35348899	24.77345688	23.93960299	28.6453199	26.89770552	Mroh1	maestro heat-like repeat family member 1 [Source:MGI Symbol;Acc:MGI:2442558]
ENSMUSG00000022559	57.98872366	45.81689483	57.3627397	44.77378696	38.02289027	35.10727045	54.69664653	54.72904783	52.58108801	30.58646334	36.0244236	33.01905489	Fbxl6	F-box and leucine-rich repeat protein 6 [Source:MGI Symbol;Acc:MGI:1354705]
ENSMUSG00000022560	5.103647982	4.580512816	5.07425039	4.86083921	4.123918499	3.614840623	4.734919651	4.805702076	3.751241143	3.501473096	3.293505791	3.68366284	Slc52a2	solute carrier protein 52, member 2 [Source:MGI Symbol;Acc:MGI:1289288]
ENSMUSG00000022561	60.64273932	50.24044512	60.94479634	59.47034018	46.4240799	43.80750567	47.90378217	46.78653801	46.27192805	48.33996573	56.25817991	53.62914484	Gpaa1	GPI anchor attachment protein 1 [Source:MGI Symbol;Acc:MGI:1202392]
ENSMUSG00000022562	5.835370267	6.923356978	5.159544372	4.372878751	3.715752193	3.142714308	6.630509059	6.306289797	7.159471608	9.648353214	9.420328893	10.16006844	Oplah	5-oxoprolinase (ATP-hydrolysing) [Source:MGI Symbol;Acc:MGI:1922725]
ENSMUSG00000022564	573.7489387	522.6176467	584.433843	497.0620049	445.8261718	392.7161326	70.12387327	67.76553094	65.86637264	97.32368554	89.36355091	95.95037614	Grina	glutamate receptor, ionotropic, N-methyl D-aspartate-associated protein 1 (glutamate binding) [Source:MGI Symbol;Acc:MGI:19134
ENSMUSG00000022565	35.56672052	39.36018639	36.91798782	40.26130581	44.22309265	53.99826247	14.63271863	13.67443534	17.37310849	33.76541896	37.39278841	37.06556146	Plec	plectin [Source:MGI Symbol;Acc:MGI:1277961]
ENSMUSG00000022568	41.16962336	42.54398764	41.50037709	46.04738548	46.34019083	49.31387985	81.37461724	77.06970892	86.74367036	56.11647527	60.50361734	54.81254047	Scrib	scribbled planar cell polarity [Source:MGI Symbol;Acc:MGI:2145950]
ENSMUSG00000022570	72.04770055	60.73656712	81.74424334	68.55661623	62.81853538	51.2570439	59.40901851	61.37791636	57.82290288	55.49427709	55.70357088	53.13480324	Tsta3	tissue specific transplantation antigen P35B [Source:MGI Symbol;Acc:MGI:98857]
ENSMUSG00000022571	29.91749289	23.86813859	28.98434087	23.60028906	22.21588758	18.55317972	50.89466103	54.8501386	44.44588456	38.17178442	37.10082621	32.63652634	Pycrl	pyrroline-5-carboxylate reductase-like [Source:MGI Symbol;Acc:MGI:1913444]
ENSMUSG00000022574	4.49876264	2.882728987	4.27150824	2.539216558	1.105431056	1.243533667	10.30010421	11.78859869	11.65004829	4.445911798	6.49294117	4.501354685	Naprt	nicotinate phosphoribosyltransferase [Source:MGI Symbol;Acc:MGI:2442664]
ENSMUSG00000022575	52.93869184	47.45341391	50.6264403	41.80491347	42.71150171	34.50707696	13.27140669	13.42388623	10.63584988	22.11588197	24.95817528	24.60042852	Gsdmd	gasdermin D [Source:MGI Symbol;Acc:MGI:1916396]
ENSMUSG00000022577	0.992701788	0.394188668	0.841093083	0.287495068	0.863165983	0.260732066	0.580654119	0.644304778	0.350469902	1.714658804	1.339214914	1.274130467	Ly6h	lymphocyte antigen 6 complex, locus H [Source:MGI Symbol;Acc:MGI:1346030]
ENSMUSG00000022579	0	0	0	0.128697044	0	0	0	0	0	0	0.038265893	0	Gpihbp1	GPI-anchored HDL-binding protein 1 [Source:MGI Symbol;Acc:MGI:1915703]
ENSMUSG00000022580	4.350394073	3.996811757	5.549123229	5.035822911	3.721568748	3.349830839	3.968827465	3.862231504	2.942838206	2.615480588	4.076395773	3.771154663	Rhpn1	rhophilin, Rho GTPase binding protein 1 [Source:MGI Symbol;Acc:MGI:1098783]
ENSMUSG00000022582	0.336686133	0	0.363638858	0.094701599	0.426493569	0.115945828	0	0	0.250698914	0	0	0	Ly6g	lymphocyte antigen 6 complex, locus G [Source:MGI Symbol;Acc:MGI:109440]
ENSMUSG00000022583	0.101482283	0.175293098	0.363638858	0.186760362	0.367974684	0.038109326	0	0	0	0	0	0	Ly6f	lymphocyte antigen 6 complex, locus F [Source:MGI Symbol;Acc:MGI:109441]
ENSMUSG00000022584	0.147105941	0.25410008	0.225908975	0.171596059	0.23183753	0.168071901	0	0	0	0.032895698	0.061225428	0	Ly6c2	lymphocyte antigen 6 complex, locus C2 [Source:MGI Symbol;Acc:MGI:3712069]
ENSMUSG00000022586	0.775288069	0.836985061	0.545692288	0.406960924	0.101820537	0.553615213	0.129779912	0	0.045100396	0	0.242005915	0	Ly6i	lymphocyte antigen 6 complex, locus I [Source:MGI Symbol;Acc:MGI:1888480]
ENSMUSG00000022587	1162.773571	905.1229913	1280.394488	1040.766148	889.2657728	717.6011054	160.0439579	169.82679	143.481414	204.5863609	202.3268876	198.4976131	Ly6e	lymphocyte antigen 6 complex, locus E [Source:MGI Symbol;Acc:MGI:106651]
ENSMUSG00000022589	0	0	0	0	0	0	0	0	0	0	0	0	Cyp11b2	cytochrome P450, family 11, subfamily b, polypeptide 2 [Source:MGI Symbol;Acc:MGI:88584]
ENSMUSG00000022591	0	0	0	0	0	0	0	0	0	0	0	0	Gm9747	predicted gene 9747 [Source:MGI Symbol;Acc:MGI:3710571]
ENSMUSG00000022594	22.28885664	22.64545226	23.66337482	17.13867956	18.9811929	16.76766096	3.145467145	1.955541213	2.209447964	8.806189751	9.006217818	8.149192175	Lynx1	Ly6/neurotoxin 1 [Source:MGI Symbol;Acc:MGI:1345180]
ENSMUSG00000022595	0	0	0	0	0	0	0.173039883	0.768033292	0.090200792	0.086684609	0	0.08588492	Lypd2	Ly6/Plaur domain containing 2 [Source:MGI Symbol;Acc:MGI:1915561]
ENSMUSG00000022596	0.050237581	0	0	0	0	0.095662641	0.067276452	0.024883741	0	0	0	0	Slurp1	secreted Ly6/Plaur domain containing 1 [Source:MGI Symbol;Acc:MGI:1930923]
ENSMUSG00000022598	0.400265003	0.172847148	0.15367064	0.186760362	0.367974684	0.038109326	0.312679045	0.59477927	0.279412685	0.223767246	0	0	Psca	prostate stem cell antigen [Source:MGI Symbol;Acc:MGI:1919623]
ENSMUSG00000022601	4.064657484	5.281096818	3.712472751	5.761487731	6.424536565	8.186735532	8.47433162	7.19626677	8.834858268	10.92842009	10.07133844	11.25818613	Zbtb11	zinc finger and BTB domain containing 11 [Source:MGI Symbol;Acc:MGI:2443876]
ENSMUSG00000022602	1.000007409	1.086683321	0.894022754	0.880616372	0.976677254	1.019159398	21.01185635	18.44562051	21.5911465	5.681856901	5.123395936	6.740349765	Arc	activity regulated cytoskeletal-associated protein [Source:MGI Symbol;Acc:MGI:88067]
ENSMUSG00000022603	0	0	0	0	0	0	0.357236709	0.44924948	0.372434763	0.238611075	0.222051305	0.260050807	Mroh4	maestro heat-like repeat family member 4 [Source:MGI Symbol;Acc:MGI:1916689]
ENSMUSG00000022604	3.495010768	3.636337805	3.421229264	3.175658026	4.106918838	4.635299677	5.0635896	4.783449243	5.835448156	7.692108705	7.974901024	8.368318236	Cep97	centrosomal protein 97 [Source:MGI Symbol;Acc:MGI:1921451]
ENSMUSG00000022607	42.25393428	43.70497304	37.27016525	44.85168202	46.79307228	46.01714693	41.47202863	39.43547161	46.95483184	47.47639244	50.13683896	51.06666109	Ptk2	PTK2 protein tyrosine kinase 2 [Source:MGI Symbol;Acc:MGI:95481]
ENSMUSG00000022610	11.11898233	10.07658345	10.37315983	8.165768428	8.917237105	6.214034494	11.86513841	9.581039162	10.0429065	6.708911569	6.873116013	6.238869727	Mapk12	mitogen-activated protein kinase 12 [Source:MGI Symbol;Acc:MGI:1353438]
ENSMUSG00000022613	0.321908886	5.066159952	0.164783978	0.450600125	0.169108423	0.122596087	0.335291744	1.753931389	0.549302577	0.383919864	0.580572795	0.332830839	Miox	myo-inositol oxygenase [Source:MGI Symbol;Acc:MGI:1891725]
ENSMUSG00000022614	73.57765437	64.49980051	75.2468855	66.73290436	63.05224203	56.54729423	37.1848218	37.66508094	39.28466649	31.75731215	34.16277101	33.57761928	Lmf2	lipase maturation factor 2 [Source:MGI Symbol;Acc:MGI:2146015]
ENSMUSG00000022615	2.15459291	2.335175994	1.665205334	1.951494505	2.263744954	1.303237934	0.169726896	0.272035356	0.21626935	0.547938647	0.421995587	0.187201299	Tymp	thymidine phosphorylase [Source:MGI Symbol;Acc:MGI:1920212]
ENSMUSG00000022617	0.500747157	0.370694631	0.512661266	0.934578038	0.638854042	0.299679324	0.383190564	0.460628446	0.732403436	0.255946576	0.267956674	0.158490876	Chkb	choline kinase beta [Source:MGI Symbol;Acc:MGI:1328313]
ENSMUSG00000022619	0.202827889	0.066733354	0.088994445	0.162236274	0.091329936	0.187595404	2.030684205	1.765312885	2.036168983	2.993508496	2.532506347	2.670587264	Mapk8ip2	mitogen-activated protein kinase 8 interacting protein 2 [Source:MGI Symbol;Acc:MGI:1926555]
ENSMUSG00000022620	56.02907372	58.39953606	55.21843554	56.9878771	51.95330274	50.14249895	14.95508757	15.52310283	12.80716182	12.22558932	11.85596039	12.3167243	Arsa	arylsulfatase A [Source:MGI Symbol;Acc:MGI:88077]
ENSMUSG00000022621	13.52323903	13.37050422	13.37883154	16.14637204	17.17437702	14.08069035	11.5041309	9.4724106	9.238851886	8.837977463	9.892279894	8.998558461	Rabl2	RAB, member RAS oncogene family-like 2 [Source:MGI Symbol;Acc:MGI:1915958]
ENSMUSG00000022622	0	0	0	0	0	0	0.072480857	0.040213064	0	0.072618804	0.033789505	0.179872192	Acr	acrosin prepropeptide [Source:MGI Symbol;Acc:MGI:87884]
ENSMUSG00000022623	1.118618095	1.358729595	1.537436082	1.115374384	1.69048199	1.412270832	3.338042875	2.641345263	3.622679661	3.673352916	3.507706822	4.345630164	Shank3	SH3 and multiple ankyrin repeat domains 3 [Source:MGI Symbol;Acc:MGI:1930016]
ENSMUSG00000022629	2.984099307	3.73515625	1.802698156	4.185716837	5.014491482	6.945861793	0.852192541	0.725272877	0.974704743	2.486838232	2.491675809	2.702073152	Kif21a	kinesin family member 21A [Source:MGI Symbol;Acc:MGI:109188]
ENSMUSG00000022634	33.78209535	34.82159981	26.88355032	33.65373269	35.77398231	36.10133238	6.326642633	6.226787679	8.899811449	14.41399305	13.59546373	15.33489061	Yaf2	YY1 associated factor 2 [Source:MGI Symbol;Acc:MGI:1914307]
ENSMUSG00000022635	98.31086231	78.48024711	113.6536598	88.9746765	78.83531197	63.97113781	47.05694738	50.05349866	36.03902842	37.73722581	38.42512649	34.21548015	Zcrb1	zinc finger CCHC-type and RNA binding motif 1 [Source:MGI Symbol;Acc:MGI:1914447]
ENSMUSG00000022636	17.57501616	19.54065405	17.74039497	23.01965161	29.36064494	31.71403666	2.124139036	1.778856451	2.695013326	19.71579707	18.96407335	21.90105794	Alcam	activated leukocyte cell adhesion molecule [Source:MGI Symbol;Acc:MGI:1313266]
ENSMUSG00000022637	6.078330208	7.134375004	5.623436402	6.896255992	7.912070958	9.919627368	6.189873057	5.000473696	6.954643079	12.08903462	12.73185612	12.63139494	Cblb	Casitas B-lineage lymphoma b [Source:MGI Symbol;Acc:MGI:2146430]
ENSMUSG00000022639	8.350713934	7.658764637	7.461689787	8.030695567	8.10400966	7.056529875	2.46613878	2.52597616	2.373283053	5.682914541	5.323891121	5.442177509	Dubr	Dppa2 upstream binding RNA [Source:MGI Symbol;Acc:MGI:1915440]
ENSMUSG00000022641	10.11554983	13.00028746	8.400442469	11.12570287	14.20694679	18.98596649	10.5514991	9.222869962	12.24289387	22.61515435	21.09354368	21.2642702	Bbx	bobby sox HMG box containing [Source:MGI Symbol;Acc:MGI:1917758]
ENSMUSG00000022650	0.12364508	0	0.063293463	0	0	0	0	0	0	0	0	0	Retnlb	resistin like beta [Source:MGI Symbol;Acc:MGI:1888505]
ENSMUSG00000022651	0	0	0	0	0	0	0	0	0	0.129807644	0	0	Retnlg	resistin like gamma [Source:MGI Symbol;Acc:MGI:2667763]
ENSMUSG00000022652	2.942948059	2.60688921	2.483215907	2.987744687	3.007092201	3.337752571	40.60916366	39.03765158	43.0441839	42.53780876	41.59118573	43.75037881	Morc1	microrchidia 1 [Source:MGI Symbol;Acc:MGI:1316740]
ENSMUSG00000022656	9.877608841	10.59549391	8.681156223	10.18568306	10.5438688	11.98965744	17.03300133	14.99053659	17.7996229	20.41395281	20.91194931	20.74525947	Nectin3	nectin cell adhesion molecule 3 [Source:MGI Symbol;Acc:MGI:1930171]
ENSMUSG00000022657	0.078806754	0	0	0	0.020699779	0	0.15830297	0.273242613	0.073350094	0.088113476	0.065598673	0.069840485	Cd96	CD96 antigen [Source:MGI Symbol;Acc:MGI:1934368]
ENSMUSG00000022658	0.936910622	0.439552155	0.355260082	0.259054696	0.218749927	0.317167942	2.168580472	3.437568363	3.358961095	4.810995796	4.390455704	3.813290469	Tagln3	transgelin 3 [Source:MGI Symbol;Acc:MGI:1926784]
ENSMUSG00000022659	0	0	0	0	0	0	0	0	0	0	0	0	Gcsam	germinal center associated, signaling and motility [Source:MGI Symbol;Acc:MGI:102969]
ENSMUSG00000022661	44.39521964	56.44194551	39.37200018	62.05537484	71.30337177	70.43004216	1.13603621	1.353942589	1.315963358	6.407635146	6.316009805	6.766189003	Cd200	CD200 antigen [Source:MGI Symbol;Acc:MGI:1196990]
ENSMUSG00000022663	24.45698486	22.67926114	21.18510824	25.42341999	24.07905598	23.90333356	16.50594745	16.0902789	16.39518189	15.71886495	17.3285125	17.21417737	Atg3	autophagy related 3 [Source:MGI Symbol;Acc:MGI:1915091]
ENSMUSG00000022664	7.988643707	9.64343966	5.725100378	7.300632334	9.116914392	11.98077195	3.443072708	3.438448662	4.410021807	7.737017568	8.805172973	8.617743505	Slc35a5	solute carrier family 35, member A5 [Source:MGI Symbol;Acc:MGI:1921352]

ENSMUSG00000022665	61.2853859	65.12827054	57.63368265	38.75619564	36.70760892	39.19676827	0.914639383	0.936831818	1.491451919	11.91294197	10.82378105	9.847508354	Ccdc80	coiled-coil domain containing 80 [Source:MGI Symbol;Acc:MGI:1915146]
ENSMUSG00000022667	1.016250718	2.016839581	0.885472366	0.100888135	0	0.032938714	0.077215787	0.021420024	0.080500807	2.088794155	1.511868111	2.050362212	Cd200r1	CD200 receptor 1 [Source:MGI Symbol;Acc:MGI:1889024]
ENSMUSG00000022668	12.44829906	14.54827846	9.297459841	12.65425468	12.65703098	14.0695365	11.22386619	11.10781243	13.65638692	16.13565855	16.32714711	16.75329337	Gtpbp8	GTP-binding protein 8 (putative) [Source:MGI Symbol;Acc:MGI:1913317]
ENSMUSG00000022671	25.13299923	16.04079941	27.86254074	18.64268614	16.24999461	14.72565443	47.84154061	48.12595708	35.43519397	21.81575974	22.36491533	21.74629933	Mzt2	mitotic spindle organizing protein 2 [Source:MGI Symbol;Acc:MGI:1922845]
ENSMUSG00000022672	2.202094476	2.428588608	1.884348322	2.77061553	3.263286633	3.408831936	6.029982822	5.432014455	6.184547523	6.408944623	6.848752001	7.131411456	Prkdc	protein kinase, DNA activated, catalytic polypeptide [Source:MGI Symbol;Acc:MGI:104779]
ENSMUSG00000022673	28.60275202	35.45161233	23.68126745	39.12191771	50.23146483	58.41227185	182.4273802	160.4013113	205.4930453	152.5675841	154.3553208	145.6005898	Mcm4	minichromosome maintenance complex component 4 [Source:MGI Symbol;Acc:MGI:103199]
ENSMUSG00000022674	7.060325599	8.215611024	6.394273838	9.951980737	9.467031668	10.28536391	21.13588357	16.77643805	20.70240133	22.61140423	22.85046789	21.83398674	Ube2v2	ubiquitin-conjugating enzyme E2 variant 2 [Source:MGI Symbol;Acc:MGI:1917870]
ENSMUSG00000022676	8.963381185	9.144962557	7.389269083	9.417490596	9.260882631	10.6381779	1.045431457	1.497493907	0.911559114	7.560476323	7.283887096	7.264308909	Snai2	snail family zinc finger 2 [Source:MGI Symbol;Acc:MGI:1096393]
ENSMUSG00000022677	19.79629001	16.19333721	23.06278567	16.41387539	16.9023168	13.83060205	15.05309723	16.45524528	14.44395629	12.17123421	10.68255831	9.820584127	Fopnl	Fgfr1op N-terminal like [Source:MGI Symbol;Acc:MGI:1913336]
ENSMUSG00000022678	36.74307645	41.6273547	33.3273201	36.71008366	41.65999104	35.82276674	75.48965509	75.05990478	76.60564448	58.37528039	57.55168	63.10219766	Nde1	nudE neurodevelopment protein 1 [Source:MGI Symbol;Acc:MGI:1914453]
ENSMUSG00000022679	2.724245696	3.501237678	2.589843991	2.928094201	3.220038503	3.612738284	5.841489963	6.337251524	6.701271255	5.526254075	6.499274748	7.199768325	Mpv17l	Mpv17 transgene, kidney disease mutant-like [Source:MGI Symbol;Acc:MGI:2135951]
ENSMUSG00000022680	46.05954325	61.78396393	35.28658821	41.78644425	48.91822392	58.45533383	40.75824386	37.17766383	47.78558893	75.32684776	81.04966158	74.9365578	Pdxdc1	pyridoxal-dependent decarboxylase domain containing 1 [Source:MGI Symbol;Acc:MGI:1920909]
ENSMUSG00000022681	29.59957095	29.9616707	29.32997672	28.24841549	31.92212856	29.06616655	47.30671886	46.28424295	41.92401427	33.48302926	31.11270848	31.53774692	Ntan1	N-terminal Asn amidase [Source:MGI Symbol;Acc:MGI:108471]
ENSMUSG00000022682	26.1271398	25.18839612	20.34222977	22.82891051	26.15964643	26.40929431	120.6793526	108.6434721	128.0305916	105.5882522	100.3061355	105.4686446	Rrn3	RRN3 RNA polymerase I transcription factor homolog (yeast) [Source:MGI Symbol;Acc:MGI:1925255]
ENSMUSG00000022683	0.317163301	0.121743282	0.595300678	0.147985791	0.277692373	0.483155095	27.56053412	28.17285757	22.33699606	15.83964218	15.40037643	16.77098265	Pla2g10	phospholipase A2, group X [Source:MGI Symbol;Acc:MGI:1347522]
ENSMUSG00000022684	26.85985251	20.57536047	29.02830374	14.37762379	13.57172797	13.56474101	22.14934064	21.29841539	22.70996815	20.43608834	21.67542703	21.62335576	Bfar	bifunctional apoptosis regulator [Source:MGI Symbol;Acc:MGI:1914368]
ENSMUSG00000022685	12.40970094	13.10189933	12.65464412	12.68125257	13.86457279	14.13157372	32.25004882	31.93895613	33.00452635	29.76168273	30.06899312	31.66490201	Parn	poly(A)-specific ribonuclease (deadenylation nuclease) [Source:MGI Symbol;Acc:MGI:1921358]
ENSMUSG00000022686	0.351827374	0.081029461	0.054029743	0.049247929	0.073930202	0.415369845	10.08272131	10.19465286	11.47770042	6.797547618	6.340436256	6.111242289	B3gnt5	UDP-GlcNAc:betaGal beta-1,3-N-acetylglucosaminyltransferase 5 [Source:MGI Symbol;Acc:MGI:2137302]
ENSMUSG00000022687	3.161158283	3.640236264	2.682129814	2.435731075	2.610320785	3.107309977	1.570771386	1.254546405	1.457641552	3.761461553	3.556742072	3.649655672	Boc	biregional cell adhesion molecule-related/down-regulated by oncogenes (Cdon) binding protein [Source:MGI Symbol;Acc:MGI:2151
ENSMUSG00000022696	0.019478718	0.022430745	0.039884337	0.054531658	0.020465513	0.044509761	0.017390156	0.038592891	0.126909941	0.034846506	0.097284406	0.120837634	Sidt1	SID1 transmembrane family, member 1 [Source:MGI Symbol;Acc:MGI:2443155]
ENSMUSG00000022698	31.85145525	35.65460266	28.297628	38.06743321	43.84584033	47.81582862	75.20887475	67.73154216	75.68416005	86.70839496	86.85900715	90.69151787	Naa50	N(alpha)-acetyltransferase 50, NatE catalytic subunit [Source:MGI Symbol;Acc:MGI:1919367]
ENSMUSG00000022701	5.934963838	5.173941651	5.477113186	6.669494619	6.718671887	6.28739104	6.007568242	6.583302365	6.185347345	6.934937117	7.132063597	6.630199067	Ccdc191	coiled-coil domain containing 191 [Source:MGI Symbol;Acc:MGI:1922661]
ENSMUSG00000022702	7.679835825	6.34776133	9.478904582	6.916985921	6.269561562	5.013935993	13.54553623	16.11723595	11.70590872	7.970455722	7.75141335	7.493779777	Hira	histone cell cycle regulator [Source:MGI Symbol;Acc:MGI:99430]
ENSMUSG00000022704	6.184522715	7.424850965	6.028557175	9.056021377	10.16150275	11.82671081	29.89779927	31.54573438	35.27264721	50.28741172	46.57836788	46.93729094	Qtrt2	queuine tRNA-ribosyltransferase accessory subunit 2 [Source:MGI Symbol;Acc:MGI:1922194]
ENSMUSG00000022705	0	0	0	0	0	0	0	0.053820515	0.075850666	0.072893876	0.045223328	0.048147607	Drd3	dopamine receptor D3 [Source:MGI Symbol;Acc:MGI:94925]
ENSMUSG00000022706	34.71562959	34.53780727	39.50679299	37.02406299	36.67282904	29.85997489	92.51612495	102.0961577	80.88961449	63.49880731	57.91246716	59.77364204	Mrpl40	mitochondrial ribosomal protein L40 [Source:MGI Symbol;Acc:MGI:1332635]
ENSMUSG00000022707	19.16248446	24.29330503	15.76606849	21.94623282	24.78221176	26.51113086	5.10124378	4.642248191	6.388407166	3.989036191	4.36472855	5.218186957	Gbe1	glucan (1,4-alpha-), branching enzyme 1 [Source:MGI Symbol;Acc:MGI:1921435]
ENSMUSG00000022708	8.609353342	11.61908778	8.739337656	12.94668211	14.15400621	18.83740438	1.044414044	0.633774286	1.054820473	2.321704004	1.962776949	2.186891305	Zbtb20	zinc finger and BTB domain containing 20 [Source:MGI Symbol;Acc:MGI:1929213]
ENSMUSG00000022710	56.46272158	61.65663709	51.97567529	59.20821081	66.04812573	81.74967817	218.8429922	211.9719757	257.8956957	261.6876944	263.136536	259.1657142	Usp7	ubiquitin specific peptidase 7 [Source:MGI Symbol;Acc:MGI:2182061]
ENSMUSG00000022711	26.46794377	25.60752278	25.54290138	27.19638919	22.83210028	21.75754311	16.01156943	15.76081764	16.4908271	19.40569733	20.25609849	19.80347487	Pmm2	phosphomannomutase 2 [Source:MGI Symbol;Acc:MGI:1859214]
ENSMUSG00000022715	0	0	0	0	0	0.046587094	0	0	0.085392647	0.164127788	0.203649612	0.108409111	Tmem114	transmembrane protein 114 [Source:MGI Symbol;Acc:MGI:1921970]
ENSMUSG00000022718	2.775224956	4.836907975	2.27812334	5.29624606	7.223874228	9.102836644	7.321366726	6.489234223	9.634136392	16.1242439	17.1904353	16.08628054	Dgcr8	DGCR8, microprocessor complex subunit [Source:MGI Symbol;Acc:MGI:2151114]
ENSMUSG00000022721	19.08310821	17.79086497	20.15270012	17.49091349	16.12228607	13.8383623	32.8603369	33.30306205	33.7717025	20.92753943	20.8895507	19.46029158	Trmt2a	TRM2 tRNA methyltransferase 2A [Source:MGI Symbol;Acc:MGI:96270]
ENSMUSG00000022722	10.60021094	9.476244479	9.031782253	11.42128908	11.86993124	8.844204921	28.42201116	30.15588739	30.15045524	18.96327679	21.6526654	21.53860176	Arl6	ADP-ribosylation factor-like 6 [Source:MGI Symbol;Acc:MGI:1927136]
ENSMUSG00000022723	1.558842956	2.966350755	1.494063984	2.940324045	2.648380417	4.437797758	6.356414702	4.293250848	6.760609597	4.835718283	4.619751154	4.262028145	Crybg3	beta-gamma crystallin domain containing 3 [Source:MGI Symbol;Acc:MGI:2676311]
ENSMUSG00000022724	7.675936271	9.243848078	5.063795092	13.06501352	14.05660652	15.15180854	21.45151085	20.9112984	24.67852867	24.49014443	24.00540586	22.53961263	Riox2	ribosomal oxygenase 2 [Source:MGI Symbol;Acc:MGI:1914264]
ENSMUSG00000022738	0	0	0	0	0	0	1.133420352	0.89833188	0.970632756	0.283894378	0.641608939	0.522368557	Gsc2	goosecoid homebox 2 [Source:MGI Symbol;Acc:MGI:892006]
ENSMUSG00000022742	13.21050065	18.38882159	10.16590389	19.20972725	22.20759498	29.89910655	42.40668101	36.55698544	48.29138581	46.33099326	49.2059099	46.08369251	Cpox	coproporphyrinogen oxidase [Source:MGI Symbol;Acc:MGI:104841]
ENSMUSG00000022744	41.56208367	49.50126877	33.07135823	49.02868667	53.57207734	52.71926202	34.26290782	30.17428557	36.69547379	41.74838806	46.94207828	42.40289888	Cldnd1	claudin domain containing 1 [Source:MGI Symbol;Acc:MGI:2447860]
ENSMUSG00000022747	1.70193816	2.211134571	1.51904311	1.873285984	3.026114616	2.692389119	1.597372228	1.5130879	1.584094229	9.212129882	8.064245798	9.359191617	St3gal6	ST3 beta-galactoside alpha-2,3-sialyltransferase 6 [Source:MGI Symbol;Acc:MGI:1888707]
ENSMUSG00000022748	14.43681898	12.07891207	14.43389587	14.68259543	13.92392505	12.28488847	46.01726949	48.43592393	36.68985179	45.19687794	42.71737442	42.13111226	Cmss1	cms small ribosomal subunit 1 [Source:MGI Symbol;Acc:MGI:1913747]
ENSMUSG00000022749	17.48865428	27.04654477	15.03890326	18.63380105	25.12506912	30.84494259	18.94561576	16.24219104	20.71935658	35.98410239	34.84045508	37.56587265	Tbc1d23	TBC1 domain family, member 23 [Source:MGI Symbol;Acc:MGI:1914831]
ENSMUSG00000022750	37.13296905	39.31536209	39.87824694	30.08089444	28.35150162	28.48457826	38.83382998	35.36172914	41.16845296	47.09331945	46.55949662	45.07324931	Klhl22	kelch-like 22 [Source:MGI Symbol;Acc:MGI:1337995]
ENSMUSG00000022751	30.24876929	27.47476136	30.5991678	28.56427046	30.26520953	24.60995322	91.73559164	93.26637575	91.17776892	77.75952008	76.13766943	76.85949532	Nit2	nitrilase family, member 2 [Source:MGI Symbol;Acc:MGI:1261838]
ENSMUSG00000022752	38.29914253	39.00916141	33.2424961	42.61674146	42.89718591	48.00216541	77.00427152	73.8839956	86.13809272	103.8224097	101.1539363	102.1281713	Tomm70a	translocase of outer mitochondrial membrane 70A [Source:MGI Symbol;Acc:MGI:106295]
ENSMUSG00000022753	0.016448199	0.018940946	0.134716361	0	0	0.012528295	0	0	0	0.117700203	0.054765865	0	Tmem30c	transmembrane protein 30C [Source:MGI Symbol;Acc:MGI:1918277]
ENSMUSG00000022754	83.3352942	77.60767507	74.92420867	73.61470936	72.21529568	64.16600426	6.209630026	6.07717909	7.5594784	28.86597478	27.61783169	28.82939481	Tmem45a	transmembrane protein 45a [Source:MGI Symbol;Acc:MGI:1913122]
ENSMUSG00000022755	0	0.128556935	0.016327743	0.029765365	0.033512467	0.024295049	0	0	0	0.028530739	0.013275343	0	Adgrg7	adhesion G protein-coupled receptor G7 [Source:MGI Symbol;Acc:MGI:2441732]
ENSMUSG00000022756	1.989428103	1.341829683	1.367540132	0.503907582	0.418042574	0.303062278	2.131339328	2.477592205	1.904583164	3.406464659	2.38936884	3.37503912	Slc7a4	solute carrier family 7 (cationic amino acid transporter, y+ system), member 4 [Source:MGI Symbol;Acc:MGI:2146512]
ENSMUSG00000022757	117.3644417	116.3851227	100.4728144	108.0354027	109.2129268	103.9997596	91.92945365	100.529777	99.5247732	116.0156979	108.8627613	108.8405129	Tfg	Trk-fused gene [Source:MGI Symbol;Acc:MGI:1338041]
ENSMUSG00000022758	1.531522448	1.196746776	1.511962825	0.952794389	0.938647288	0.486055391	0.211607247	0.198679799	0.271519671	0.424019969	0.273179559	0.274686178	P2rx6	purinergic receptor P2X, ligand-gated ion channel, 6 [Source:MGI Symbol;Acc:MGI:1337113]
ENSMUSG00000022759	0.067548647	0.077785739	0	0.105058812	0.260225799	0.205802327	0.040203929	0.133833117	0.188614528	0.221542454	0.149939824	0.259407515	Lrrc74b	leucine rich repeat containing 74B [Source:MGI Symbol;Acc:MGI:1921935]
ENSMUSG00000022760	39.98085184	35.74941043	46.80221059	36.34540491	29.08962621	26.4002015	23.98046112	24.71582766	17.78893885	21.06904282	23.13262786	22.15645362	Thap7	THAP domain containing 7 [Source:MGI Symbol;Acc:MGI:1916259]
ENSMUSG00000022761	57.64303511	57.36077215	57.19639251	58.62733773	54.75161694	56.72256075	30.27963802	31.52236234	35.03623337	40.51913104	46.09181134	46.8534201	Lztr1	leucine-zipper-like transcriptional regulator, 1 [Source:MGI Symbol;Acc:MGI:1914113]
ENSMUSG00000022762	0	0.02583395	0.034451708	0	0	0.017087602	0	0	0	0	0.009337037	0	Ncam2	neural cell adhesion molecule 2 [Source:MGI Symbol;Acc:MGI:97282]
ENSMUSG00000022763	1.738074599	1.590921786	2.075999362	2.432913986	2.481658076	2.138543035	7.639204538	8.896021037	10.05895313	3.58769236	3.746767917	4.265513937	Aifm3	apoptosis-inducing factor, mitochondrion-associated 3 [Source:MGI Symbol;Acc:MGI:1919418]
ENSMUSG00000022765	7.839248725	10.45039102	6.645538179	9.691817104	10.89878519	12.09415557	6.798102983	6.393258851	6.83170905	8.552881416	8.343762985	8.086786576	Snap29	synaptosomal-associated protein 29 [Source:MGI Symbol;Acc:MGI:1914724]
ENSMUSG00000022766	3.08360686	2.009079841	1.931569669	2.21497261	2.493811055	2.194206002	0	0.040194104	0	0	0	0.053936216	Serpind1	serine (or cysteine) peptidase inhibitor, clade D, member 1 [Source:MGI Symbol;Acc:MGI:96051]
ENSMUSG00000022768	0.432644759	0.430274587	0.402671391	0.20186849	0.14463356	0.314558699	0.772510777	1.168898931	0.896895989	1.512781128	1.898883709	1.394256113	Ccdc116	coiled-coil domain containing 116 [Source:MGI Symbol;Acc:MGI:1924122]
ENSMUSG00000022769	20.03853765	17.03823597	24.09355922	14.49584037	11.46528651	9.465421123	57.89964467	66.67021125	47.42282202	33.83328452	35.33198998	34.96663462	Sdf2l1	stromal cell-derived factor 2-like 1 [Source:MGI Symbol;Acc:MGI:2149842]
ENSMUSG00000022770	21.69620087	26.14170981	19.24957369	20.11716731	22.63146988	28.52382545	31.85508841	28.11966916	36.62973142	46.31576461	49.90994328	47.96176454	Dlg1	discs large MAGUK scaffold protein 1 [Source:MGI Symbol;Acc:MGI:107231]
ENSMUSG00000022771	52.51838461	52.92888914	53.2216734	52.77923924	51.93550832	46.85320576	72.40638571	68.79034058	73.32293729	74.35097844	72.01444405	67.34899198	Ppil2	peptidylprolyl isomerase (cyclophilin)-like 2 [Source:MGI Symbol;Acc:MGI:2447857]
ENSMUSG00000022772	15.82992495	19.66936521	13.31812215	15.74493608	18.7929928	23.80774303	6.861567927	6.760106983	9.223205761	17.76463306	18.32491304	18.90633356	Senp5	SUMO/sentrin specific peptidase 5 [Source:MGI Symbol;Acc:MGI:2443596]
ENSMUSG00000022773	1.04209619	2.245212427	1.686375203	1.631235037	1.977863928	2.637284473	4.381694293	5.561341067	4.19264555	4.540377283	5.316567454	4.4984911	Ypel1	yippee like 1 [Source:MGI Symbol;Acc:MGI:1913303]
ENSMUSG00000022774	62.47972205	70.6433031	62.8986291	65.28642392	62.5008574	65.78978853	153.7808542	145.6195977	154.7526224	113.7199765	110.922863	106.9650077	Ncbp2	nuclear cap binding protein subunit 2 [Source:MGI Symbol;Acc:MGI:1915342]
ENSMUSG00000022779	30.49119152	33.64040665	32.68091263	29.07861478	27.33600579	31.91642604	58.89915391	54.59364228	64.23725294	61.13435111	66.13710175	58.49382622	Top3b	topoisomerase (DNA) III beta [Source:MGI Symbol;Acc:MGI:1333803]
ENSMUSG00000022780	0.020821915	0	0.021317324	0.029146004	0.010938379	0.015859676	0.306723974	0.360973789	0.455434339	0.111748269	0.051996432	0.147623149	Meltf	melanotransferrin [Source:MGI Symbol;Acc:MGI:1353421]

ENSMUSG00000022781	19.49111547	22.47343283	17.76993005	21.45816591	25.24822366	27.14568395	28.84418085	24.6858407	33.99814726	41.9527661	38.28133939	38.44995005	Pak2	p21 (RAC1) activated kinase 2 [Source:MGI Symbol;Acc:MGI:1339984]
ENSMUSG00000022783	0	0.266873513	0	0	0.040581973	0.117680505	1.34486473	0.880066874	1.258276753	0.794634853	0.353667532	0.753073522	Spag6l	sperm associated antigen 6-like [Source:MGI Symbol;Acc:MGI:1354388]
ENSMUSG00000022787	12.78635592	8.543019935	13.40332156	11.76912281	8.757392297	8.708715423	17.02565033	21.26969277	16.5720627	15.9650895	13.91264027	10.48074764	Wdr53	WD repeat domain 53 [Source:MGI Symbol;Acc:MGI:1916230]
ENSMUSG00000022788	2.494405091	3.639506582	2.626303454	4.377734244	4.69058639	7.794085277	1.277964513	1.235531817	1.754458878	4.804657199	5.226243688	5.451146454	Fgd4	FYVE, RhoGEF and PH domain containing 4 [Source:MGI Symbol;Acc:MGI:2183747]
ENSMUSG00000022789	22.77290036	26.5949441	22.4606541	27.21968753	31.07687462	35.05928398	44.6736007	45.10510619	54.73384041	67.51103489	65.98101959	65.74683465	Dnm1l	dynamin 1-like [Source:MGI Symbol;Acc:MGI:1921256]
ENSMUSG00000022790	0.475607473	1.030939207	0.257782999	0.496044265	0.646672954	0.490118644	0.424611521	0.582017426	0.546834969	2.627591982	2.002764169	2.057887574	Igsf11	immunoglobulin superfamily, member 11 [Source:MGI Symbol;Acc:MGI:2388477]
ENSMUSG00000022791	84.77009864	81.4778332	85.73300895	92.05386762	84.83627331	83.81523603	97.86492187	95.42719684	99.65465871	67.87192765	70.65993705	70.0036358	Tnk2	tyrosine kinase, non-receptor, 2 [Source:MGI Symbol;Acc:MGI:1858308]
ENSMUSG00000022792	15.74523925	15.48881155	13.41146284	15.6449847	13.328082	12.98822299	30.64651691	28.13305948	30.76368158	29.42191207	28.01675593	31.18424206	Yars2	tyrosyl-tRNA synthetase 2 (mitochondrial) [Source:MGI Symbol;Acc:MGI:1917370]
ENSMUSG00000022793	12.96284097	14.61476249	12.61117257	13.99671704	12.44298352	12.35487529	5.695577733	7.294802012	6.853837445	6.252776871	6.157790895	6.134946811	B4galt4	UDP-Gal:betaGlcNAc beta 1,4-galactosyltransferase, polypeptide 4 [Source:MGI Symbol;Acc:MGI:1928387]
ENSMUSG00000022797	16.6011642	20.11306413	13.79861103	21.59006095	22.84395762	32.37143635	308.7344553	290.5269251	360.8672291	320.9694393	319.2921462	308.7040676	Tfrc	transferrin receptor [Source:MGI Symbol;Acc:MGI:98822]
ENSMUSG00000022798	0	0.037910877	0	0	0	0	0.176349751	0	0.15321022	0	0	0.087527708	Tex55	testis expressed 55 [Source:MGI Symbol;Acc:MGI:1921913]
ENSMUSG00000022799	8.380157524	11.5683048	7.996982183	8.360882858	8.511207806	12.63206422	0.572666735	0.573947456	0.74147263	9.698338244	9.35251906	9.168767658	Arhgap31	Rho GTPase activating protein 31 [Source:MGI Symbol;Acc:MGI:1333857]
ENSMUSG00000022800	61.26845239	77.22617734	56.9695634	65.07742942	70.85638422	82.99269927	103.0926124	101.3510196	115.3926151	142.5128125	140.527725	141.9880985	Fyttd1	forty-two-three domain containing 1 [Source:MGI Symbol;Acc:MGI:1917955]
ENSMUSG00000022801	0.767191581	3.054249307	0.785445113	1.329585359	2.901807495	6.978879585	2.407043836	1.476595845	3.651960327	10.41116156	9.670373919	9.926599234	Lrch3	leucine-rich repeats and calponin homology (CH) domain containing 3 [Source:MGI Symbol;Acc:MGI:1917394]
ENSMUSG00000022802	2.542398238	2.878497184	2.209174414	3.528880624	3.591524794	4.898202687	4.501442682	3.449857584	5.110542175	4.815773349	4.837234424	4.506271756	Lmln	leishmanolysin-like (metallopeptidase M8 family) [Source:MGI Symbol;Acc:MGI:2444736]
ENSMUSG00000022803	0.289998233	0.20871742	0.779357417	0.338276983	0.266603394	0.33132961	0.032362977	0.071821142	0.067479615	0.129698286	0.271568559	0.224878124	Popdc2	popeye domain containing 2 [Source:MGI Symbol;Acc:MGI:1930150]
ENSMUSG00000022805	0.077139634	0.017766051	0.110564988	0.143970878	0	0.117511727	0.592269174	0.473790347	0.445150124	0.607196314	0.796215948	0.601594767	Maats1	MYCBP-associated, testis expressed 1 [Source:MGI Symbol;Acc:MGI:2443598]
ENSMUSG00000022807	7.987596235	9.943921805	6.944598196	8.980722399	8.953324204	11.57086496	9.352652615	8.250527162	11.62239702	15.07199739	15.32171594	14.69128808	Osbpl11	oxysterol binding protein-like 11 [Source:MGI Symbol;Acc:MGI:2146553]
ENSMUSG00000022808	55.04635702	62.55084625	48.11070823	59.42246457	60.41834732	65.28049825	51.78725517	51.47412862	57.28313043	67.39321459	66.03671152	67.26066279	Snx4	sorting nexin 4 [Source:MGI Symbol;Acc:MGI:1916400]
ENSMUSG00000022809	0.050562265	0.077633397	0.155295829	0.031455917	0.070831678	0.141211996	0.060187785	0.050089128	0.047061283	0.075377921	0.112234627	0.059746032	Nr1i2	nuclear receptor subfamily 1, group I, member 2 [Source:MGI Symbol;Acc:MGI:1337040]
ENSMUSG00000022811	13.28641692	16.50714741	10.3828533	15.74289329	16.18790776	22.12478504	9.130329339	9.15308571	11.59496681	18.29541291	17.36053086	19.00163721	Zfp148	zinc finger protein 148 [Source:MGI Symbol;Acc:MGI:1332234]
ENSMUSG00000022812	21.71974367	26.92275298	19.66329316	25.06273286	29.74581123	40.09459542	14.19740749	13.95283993	17.78817935	27.82671485	25.15865757	27.73811583	Gsk3b	glycogen synthase kinase 3 beta [Source:MGI Symbol;Acc:MGI:1861437]
ENSMUSG00000022814	12.09222575	11.02564576	10.96099133	12.25725254	11.5158305	12.02876289	52.72960909	53.74837238	48.99630239	44.09510804	42.63308937	41.84027042	Umps	uridine monophosphate synthetase [Source:MGI Symbol;Acc:MGI:1298388]
ENSMUSG00000022816	695.5617873	801.8793418	657.0859363	373.801035	403.6987333	451.450173	118.8897673	111.8928502	133.7407797	379.190749	380.815739	381.0159117	Fstl1	follistatin-like 1 [Source:MGI Symbol;Acc:MGI:102793]
ENSMUSG00000022817	157.3003728	144.3579464	166.2726343	63.45483264	59.6042633	51.28951135	45.84566034	47.56711217	47.15284181	79.20672968	85.24016175	83.56218919	Itgb5	integrin beta 5 [Source:MGI Symbol;Acc:MGI:96614]
ENSMUSG00000022818	0.026742379	0.030795224	0.109514606	0.024955549	0	0.061107559	0	0	0.074672128	0.07176128	0.15582233	0.16589828	Cyp2ab1	cytochrome P450, family 2, subfamily ab, polypeptide 1 [Source:MGI Symbol;Acc:MGI:3644957]
ENSMUSG00000022820	25.65369338	23.39489788	34.6284144	22.56727109	21.51687051	18.0879405	43.61787861	45.21577262	31.32958942	24.2620345	24.89046157	22.05916132	Ndufb4	NADH:ubiquinone oxidoreductase subunit B4 [Source:MGI Symbol;Acc:MGI:1915444]
ENSMUSG00000022821	0.188556038	0.998807063	0.154433831	0.175957396	0.039621664	0.603202834	0.033667707	0.149433296	0	0.033731785	0.062781552	0.1002618	Hgd	homogentisate 1, 2-dioxygenase [Source:MGI Symbol;Acc:MGI:96078]
ENSMUSG00000022822	30.44665213	33.00632785	24.31532388	23.80666618	25.09950254	31.4503526	51.8197143	50.43025708	61.29223943	75.33858792	80.23597307	75.79351169	Abcc5	ATP-binding cassette, sub-family C (CFTR/MRP), member 5 [Source:MGI Symbol;Acc:MGI:1351644]
ENSMUSG00000022824	0.029112644	0	0.059610623	0	0.061174991	0.033261861	0.038986658	0.100940776	0	0.130202863	0.036350009	0.051600683	Muc13	mucin 13, epithelial transmembrane [Source:MGI Symbol;Acc:MGI:103190]
ENSMUSG00000022827	4.369931058	4.745276552	3.944099015	4.274690959	4.772548529	4.525588596	8.555646786	8.126442234	9.187221842	7.046126583	6.828339735	8.051223529	Rabl3	RAB, member RAS oncogene family-like 3 [Source:MGI Symbol;Acc:MGI:1914907]
ENSMUSG00000022828	5.777146578	6.451084859	4.647185941	7.21152261	7.239111715	9.591234752	23.46644094	21.02181446	24.90705733	27.58248331	26.92771196	26.53012898	Gtf2e1	general transcription factor II E, polypeptide 1 (alpha subunit) [Source:MGI Symbol;Acc:MGI:1921447]
ENSMUSG00000022829	0	0	0	0	0	0.012649178	0.014826266	0	0.077285125	0	0.082941433	0.058869787	Stxbp5l	syntaxin binding protein 5-like [Source:MGI Symbol;Acc:MGI:2443815]
ENSMUSG00000022831	10.74839761	8.398904108	12.96915395	0.455919456	0.183326514	0.212645714	0.560800789	0.241995891	0.649621274	10.61306453	10.37033621	10.32959462	Hcls1	hematopoietic cell specific Lyn substrate 1 [Source:MGI Symbol;Acc:MGI:104568]
ENSMUSG00000022832	0	0	0	0	0.052203601	0	0	0.049221533	0	0.044443379	0	0.088066754	Ropn1	ropporin, rhophilin associated protein 1 [Source:MGI Symbol;Acc:MGI:1923628]
ENSMUSG00000022833	2.74787488	2.233637068	1.875502763	2.775447003	3.056911083	3.480136426	9.716754974	9.020496195	11.66470404	10.06296942	8.709788653	9.483088995	Ccdc14	coiled-coil domain containing 14 [Source:MGI Symbol;Acc:MGI:2443448]
ENSMUSG00000022836	2.845313828	3.276525137	2.725075204	3.888583391	4.590264406	4.865767569	0.213051665	0.309146506	0.119600708	0.607531892	0.336165214	0.439244209	Mylk	myosin, light polypeptide kinase [Source:MGI Symbol;Acc:MGI:894806]
ENSMUSG00000022837	17.42619113	16.99524449	18.69205785	20.65867338	18.52740261	19.65913476	34.67234413	36.7913919	37.17928479	23.30350299	24.19513569	23.30344175	Iqcb1	IQ calmodulin-binding motif containing 1 [Source:MGI Symbol;Acc:MGI:2443764]
ENSMUSG00000022838	2.518740751	1.611366362	2.829372167	2.056641548	1.249660832	1.065821809	2.84207457	3.708102198	2.767624292	1.314223242	1.980119945	1.581120438	Eaf2	ELL associated factor 2 [Source:MGI Symbol;Acc:MGI:2146616]
ENSMUSG00000022840	1.165767755	1.369290326	1.312854923	0.739755213	0.894122796	0.852426438	0.156115636	0.21942322	0.119355369	0.761208763	0.504599709	0.774849052	Adcy5	adenylate cyclase 5 [Source:MGI Symbol;Acc:MGI:99673]
ENSMUSG00000022841	315.6479281	293.5939194	295.2891887	299.2490769	276.8652591	257.1208166	277.80005	274.1515047	280.6813167	200.8666708	206.6445205	209.2091571	Ap2m1	adaptor-related protein complex 2, mu 1 subunit [Source:MGI Symbol;Acc:MGI:1298405]
ENSMUSG00000022842	0.019269363	0	0.059183498	0.107891118	0	0.073385626	0.997788417	0.553582438	0.77121071	0.258539855	0.144358208	0.187846821	Ece2	endothelin converting enzyme 2 [Source:MGI Symbol;Acc:MGI:1101356]
ENSMUSG00000022843	12.58154579	14.30460993	12.20725005	14.55051884	14.79011712	15.32390492	29.4786832	26.44856815	31.93910555	17.21079126	19.58476046	20.83350075	Clcn2	chloride channel, voltage-sensitive 2 [Source:MGI Symbol;Acc:MGI:105061]
ENSMUSG00000022844	88.32853034	76.91493339	86.85225369	47.20581455	40.02198335	38.49699683	27.22189659	29.5135311	28.21737173	37.86278113	41.52575817	41.19127979	Pdia5	protein disulfide isomerase associated 5 [Source:MGI Symbol;Acc:MGI:1919849]
ENSMUSG00000022847	0.982749413	1.192858709	1.577179328	1.264090969	0.948816558	0.910389462	4.386881483	3.394280465	3.658811374	1.948156323	1.613970315	1.953722895	Thpo	thrombopoietin [Source:MGI Symbol;Acc:MGI:101875]
ENSMUSG00000022848	10.27796285	9.53193633	10.22816722	8.395117443	7.790631698	8.147884306	8.716065164	8.579757207	8.406753967	7.211136238	6.57108044	6.48641558	Slc49a4	solute carrier family 49 member 4 [Source:MGI Symbol;Acc:MGI:2387188]
ENSMUSG00000022849	5.009785129	4.62804014	5.185969946	3.81276232	4.210865357	3.875220551	24.19191586	27.6709578	24.95170941	19.10954915	20.45222359	20.72890886	Hspbap1	Hspb associated protein 1 [Source:MGI Symbol;Acc:MGI:1913917]
ENSMUSG00000022853	0.606238339	1.213389681	0.85710517	1.279631129	0.86443279	0.901532939	2.048964038	1.78739717	1.773394163	3.227773094	3.460335504	3.197996032	Ehhadh	enoyl-Coenzyme A, hydratase/3-hydroxyacyl Coenzyme A dehydrogenase [Source:MGI Symbol;Acc:MGI:1277964]
ENSMUSG00000022855	20.73662064	24.89636528	17.51475008	24.04550078	26.30595588	27.75773814	51.40836562	51.3184012	61.90551579	61.17542892	60.15828617	61.03646993	Senp2	SUMO/sentrin specific peptidase 2 [Source:MGI Symbol;Acc:MGI:1923076]
ENSMUSG00000022856	15.08703268	13.36122945	17.93951698	13.51076141	12.36831351	10.15170136	20.65704417	21.61452183	18.26140557	15.49201162	13.60027583	14.00005227	Tmem41a	transmembrane protein 41a [Source:MGI Symbol;Acc:MGI:1913914]
ENSMUSG00000022857	0	0	0	0	0	0	0	0	0	0	0	0	Tmprss15	transmembrane protease, serine 15 [Source:MGI Symbol;Acc:MGI:1197523]
ENSMUSG00000022858	64.51646282	65.3400206	58.06398546	71.62909782	75.76119284	82.73539398	227.2761604	227.3118313	243.6934635	192.0403841	188.2681916	199.4877047	Tra2b	transformer 2 beta [Source:MGI Symbol;Acc:MGI:106016]
ENSMUSG00000022860	0.096368394	0.05548658	0.073995941	0	0	0	0	0	0	0	0	0	Chodl	chondrolectin [Source:MGI Symbol;Acc:MGI:2179069]
ENSMUSG00000022861	0.042956893	0.016489024	0.014659651	0	0	0.065438976	0.012783645	0.028369949	0.066637523	0.268967485	0.21454368	0.329935282	Dgkg	diacylglycerol kinase, gamma [Source:MGI Symbol;Acc:MGI:105060]
ENSMUSG00000022863	11.29398387	11.02118	7.328470458	8.436436193	9.972013536	9.389968944	12.89962752	13.70960721	12.33800109	14.79759151	14.32233653	12.1000899	Btg3	BTG anti-proliferation factor 3 [Source:MGI Symbol;Acc:MGI:109532]
ENSMUSG00000022864	6.40513787	6.567533678	5.838899394	9.023069448	9.863968318	11.04051429	14.74240525	15.55878209	17.19892192	14.92742905	15.32296182	16.68703374	D16Ertd472	DNA segment, Chr 16, ERATO Doi 472, expressed [Source:MGI Symbol;Acc:MGI:1196400]
ENSMUSG00000022865	4.119748835	4.74410256	2.910507012	6.070234611	7.543157261	9.413814438	15.3358292	15.41785981	16.79552883	16.78730448	16.58485779	16.41892705	Cxadr	coxsackie virus and adenovirus receptor [Source:MGI Symbol;Acc:MGI:1201679]
ENSMUSG00000022867	16.73535097	21.75759177	13.37435383	18.63038723	18.97896384	26.96970223	9.70255105	7.6587608	10.32141512	14.54026517	12.91680629	14.14449509	Usp25	ubiquitin specific peptidase 25 [Source:MGI Symbol;Acc:MGI:1353655]
ENSMUSG00000022868	0.496776502	0.176019594	0.11736834	0.142641129	0.240896901	0.174639542	0.989370132	0.832831838	0.995893644	0.581079421	0.699796857	0.643450434	Ahsg	alpha-2-HS-glycoprotein [Source:MGI Symbol;Acc:MGI:107189]
ENSMUSG00000022871	65.64683517	54.8895294	68.88815958	111.275767	95.22250632	82.94422232	3.266952399	2.781305312	2.906793256	6.405255401	6.407124353	6.485948598	Fetub	fetuin beta [Source:MGI Symbol;Acc:MGI:1890221]
ENSMUSG00000022875	0.512854444	0.32481804	0.393792463	0.263223037	0.377185016	0.23437917	0.503651245	0.330236007	0.453476686	0.344052143	0.213449794	0.295427748	Kng1	kininogen 1 [Source:MGI Symbol;Acc:MGI:1097705]
ENSMUSG00000022876	0.224273578	0.848576962	0.524822043	0.089695036	0.033662188	0.073210768	0.143018816	0.063478552	0.268385974	1.089011707	1.040102862	1.107359109	Samsn1	SAM domain, SH3 domain and nuclear localization signals, 1 [Source:MGI Symbol;Acc:MGI:1914992]
ENSMUSG00000022877	0	0	0	0	0	0	0.206690222	0.491458659	0.184700161	0.207083601	0.027530266	0.029310457	Hrg	histidine-rich glycoprotein [Source:MGI Symbol;Acc:MGI:2146636]
ENSMUSG00000022878	0.234871658	0.216373431	0.336643833	0.526028094	0.296124356	0.107338496	0.209688068	0.046534768	0.043721781	0	0.117304177	0.083259617	Adipoq	adiponectin, C1Q and collagen domain containing [Source:MGI Symbol;Acc:MGI:106675]
ENSMUSG00000022881	18.67555895	13.19026458	16.97847143	23.70170567	22.7611325	18.09399056	75.76262886	78.936755	69.76154287	49.04542937	45.55873397	47.13650718	Rfc4	replication factor C (activator 1) 4 [Source:MGI Symbol;Acc:MGI:2146571]
ENSMUSG00000022883	2.729779106	4.365945785	2.498317297	1.749718996	2.694247745	3.759403841	7.594349555	7.074716151	10.34271584	22.71542264	22.65380077	21.83386757	Robo1	roundabout guidance receptor 1 [Source:MGI Symbol;Acc:MGI:1274781]
ENSMUSG00000022884	257.5705295	288.0324866	211.942547	289.3665049	324.6588071	340.8803021	812.5366994	776.1869477	878.5852468	557.4847669	545.8491823	542.3031134	Eif4a2	eukaryotic translation initiation factor 4A2 [Source:MGI Symbol;Acc:MGI:106906]

ENSMUSG00000022885	10.62049467	11.99928998	9.847412407	12.46083796	14.66112272	15.8319888	1.854061543	2.562603884	3.001143023	5.26317237	5.883551095	6.409298544	St6gal1	beta galactoside alpha 2,6 sialyltransferase 1 [Source:MGI Symbol;Acc:MGI:108470]
ENSMUSG00000022886	0.58409712	0.448411906	0.548161484	0.590492321	0.255703158	0.18537342	0	0.144657854	0	0.174153694	0.16206731	0	Prl2a1	prolactin family 2, subfamily a, member 1 [Source:MGI Symbol;Acc:MGI:1861446]
ENSMUSG00000022887	58.3058725	71.71601085	52.80954518	37.22221339	43.12178058	42.9572005	0.764820624	0.565772941	0.519491257	5.770292394	5.629137896	5.314450065	Masp1	mannan-binding lectin serine peptidase 1 [Source:MGI Symbol;Acc:MGI:88492]
ENSMUSG00000022889	36.70705633	33.23228692	31.7651428	35.99279099	36.51317692	33.61139803	67.99620018	71.7590566	66.22748265	57.63634425	58.00859464	59.6215678	Mrpl39	mitochondrial ribosomal protein L39 [Source:MGI Symbol;Acc:MGI:1351620]
ENSMUSG00000022890	194.8459826	162.2470417	246.0276615	177.8323838	158.5780635	119.4593377	319.2452281	309.7868211	218.1751376	167.2873187	161.943883	157.1101836	Atp5j	ATP synthase, H+ transporting, mitochondrial F0 complex, subunit F [Source:MGI Symbol;Acc:MGI:107777]
ENSMUSG00000022892	225.0908901	270.8088796	184.3924459	199.6136307	211.0879549	220.9098204	13.74617732	14.04246102	15.82965127	81.64386852	87.29839847	83.5152657	App	amyloid beta (A4) precursor protein [Source:MGI Symbol;Acc:MGI:88059]
ENSMUSG00000022893	24.81477059	35.52324867	19.19502496	11.26053054	15.57497285	20.25985445	0.50679227	0.626614422	0.920843665	5.193626823	6.466748343	4.72888264	Adamts1	a disintegrin-like and metallopeptidase (reprolysin type) with thrombospondin type 1 motif, 1 [Source:MGI Symbol;Acc:MGI:109249]
ENSMUSG00000022894	8.122419582	9.073287265	7.278765833	2.70517824	3.121137723	3.523108511	0.007119795	0.051351684	0.044536171	0.135533567	0.149361449	0.137817003	Adamts5	a disintegrin-like and metallopeptidase (reprolysin type) with thrombospondin type 1 motif, 5 (aggrecanase-2) [Source:MGI Symbol;
ENSMUSG00000022895	39.82476308	45.60485201	36.7102085	31.0924983	31.38171927	36.22786932	22.94770492	20.09225238	22.77709477	29.89099794	31.03718935	29.15660238	Ets2	E26 avian leukemia oncogene 2, 3' domain [Source:MGI Symbol;Acc:MGI:95456]
ENSMUSG00000022897	10.65076105	12.99133226	8.606184577	12.40335865	15.78360654	17.20831632	15.17995768	14.2134603	17.65549026	19.67046082	20.51563273	19.9830462	Dyrk1a	dual-specificity tyrosine-(Y)-phosphorylation regulated kinase 1a [Source:MGI Symbol;Acc:MGI:1330299]
ENSMUSG00000022898	64.05578383	65.45429842	62.8605669	61.3869013	65.3474149	59.0067244	44.93589411	42.05574891	46.47679313	50.40656501	49.96675221	51.78542612	Vps26c	VPS26 endosomal protein sorting factor C [Source:MGI Symbol;Acc:MGI:1206040]
ENSMUSG00000022899	1.233657774	1.93720956	1.148190709	1.779172345	1.76748421	1.936258387	6.908666197	6.814210232	8.316027119	10.06505463	8.619699833	9.905941357	Slc15a2	solute carrier family 15 (H+/peptide transporter), member 2 [Source:MGI Symbol;Acc:MGI:1890457]
ENSMUSG00000022900	0.186836682	0.086060817	0	0.017435292	0	0.256158216	9.457760425	7.459060627	10.22531528	13.95460353	13.46826494	15.15050273	Ildr1	immunoglobulin-like domain containing receptor 1 [Source:MGI Symbol;Acc:MGI:2146574]
ENSMUSG00000022901	0.948889469	1.344855386	1.046194237	0	0.03834463	0.083394455	0.260660587	0.036154239	0.135874984	4.439663636	5.194814001	4.075272257	Cd86	CD86 antigen [Source:MGI Symbol;Acc:MGI:101773]
ENSMUSG00000022902	0.182711201	0	0	0.511509272	0	0	0	0	0	0.081715428	0	0.080961581	Stfa2	stefin A2 [Source:MGI Symbol;Acc:MGI:106197]
ENSMUSG00000022905	27.16617939	34.28717248	25.38467081	24.83466786	29.95431749	33.59688337	54.88172354	50.87432772	66.14363849	87.44051148	78.12597089	79.65176846	Kpna1	karyopherin (importin) alpha 1 [Source:MGI Symbol;Acc:MGI:103560]
ENSMUSG00000022906	36.63029444	44.06032165	30.97750058	33.25507228	37.60217948	37.80273589	15.63448149	12.97967163	17.41473769	35.36423924	33.39796327	32.73375527	Parp9	poly (ADP-ribose) polymerase family, member 9 [Source:MGI Symbol;Acc:MGI:1933117]
ENSMUSG00000022911	10.78134971	12.88956994	7.267629115	12.66100652	13.64395193	18.41693277	11.72345209	9.912429674	13.19186579	15.77659883	16.47656941	16.91932933	Arl13b	ADP-ribosylation factor-like 13B [Source:MGI Symbol;Acc:MGI:1915396]
ENSMUSG00000022912	54.17630429	68.05559985	45.43888277	45.40356937	48.13646003	55.1272246	27.1833925	24.82207354	31.41870483	45.96655032	45.44320279	46.02724694	Pros1	protein S (alpha) [Source:MGI Symbol;Acc:MGI:1095733]
ENSMUSG00000022913	38.49054057	28.45791749	42.37300305	36.32690752	28.26237945	24.94490262	130.0345251	150.4632845	114.9568403	83.33353837	86.33368842	86.00496293	Psmg1	proteasome (prosome, macropain) assembly chaperone 1 [Source:MGI Symbol;Acc:MGI:1860263]
ENSMUSG00000022914	13.31065432	17.00095932	12.75592102	14.69463906	15.66563828	20.03105218	23.63587884	23.46456964	25.73417513	34.8649518	34.97398175	35.54475734	Brwd1	bromodomain and WD repeat domain containing 1 [Source:MGI Symbol;Acc:MGI:1890651]
ENSMUSG00000022931	0	0	0	0	0	0	0	0	0	0	0	0	Krtap15	keratin associated protein 15 [Source:MGI Symbol;Acc:MGI:1347350]
ENSMUSG00000022935	0.167426023	0.096399836	0.214261917	0.013019934	0.043976963	0.085016915	0	0	0.012986106	0.074879312	0.023227546	0.02472951	Grik1	glutamate receptor, ionotropic, kainate 1 [Source:MGI Symbol;Acc:MGI:95814]
ENSMUSG00000022938	0.893325007	0.342903222	1.625919269	1.065201834	1.407871506	1.474263904	0.265846741	0.344153326	0.415735137	0.93223448	0.908848051	0.92363437	Fam3b	family with sequence similarity 3, member B [Source:MGI Symbol;Acc:MGI:1270150]
ENSMUSG00000022940	25.84031995	20.72921706	25.50393433	22.54230335	22.45163448	16.01106001	21.41070814	21.91693384	16.70065833	14.75112341	11.89062263	12.556584	Pigp	phosphatidylinositol glycan anchor biosynthesis, class P [Source:MGI Symbol;Acc:MGI:1860433]
ENSMUSG00000022941	0.084314476	0.161820756	0.028773514	0.131134807	0.029528621	0.406731804	11.19074129	10.55205505	10.41120987	1.759737195	1.520638084	1.369895335	Ripply3	ripply transcriptional repressor 3 [Source:MGI Symbol;Acc:MGI:2181192]
ENSMUSG00000022945	30.04195446	24.16954308	27.35474638	33.44541062	29.17699979	26.75641086	126.657013	127.4072816	125.2916718	63.75232885	68.1903634	62.98389931	Chaf1b	chromatin assembly factor 1, subunit B (p60) [Source:MGI Symbol;Acc:MGI:1314881]
ENSMUSG00000022946	6.889336783	8.988158318	7.073637584	7.525293162	9.811523047	11.01061498	14.39890412	13.59056412	13.73272618	11.41637503	11.75111741	12.44039695	Dop1b	DOP1 leucine zipper like protein B [Source:MGI Symbol;Acc:MGI:1917278]
ENSMUSG00000022947	37.97711786	27.07508218	41.654529	23.39786435	22.8212749	17.27893938	29.7233396	30.16108541	24.23465199	20.45571745	19.30365221	16.60429575	Cbr3	carbonyl reductase 3 [Source:MGI Symbol;Acc:MGI:1309992]
ENSMUSG00000022948	5.748080435	4.538886927	5.968911763	5.555586151	3.968669168	4.284390102	23.9726284	24.48545832	25.0307197	22.2554462	19.4805525	22.26845197	Setd4	SET domain containing 4 [Source:MGI Symbol;Acc:MGI:2136890]
ENSMUSG00000022949	1.623716522	1.198585284	1.641036897	1.418095734	1.181059115	1.189187977	0.631884392	0.412441681	0.329383443	0.726188045	0.76242986	0.608798188	Clic6	chloride intracellular channel 6 [Source:MGI Symbol;Acc:MGI:2146607]
ENSMUSG00000022951	17.32247051	15.77544505	15.1624217	15.00511034	14.2543745	13.55680141	15.86649166	13.28291628	12.67689799	17.10313441	16.11429186	17.39066704	Rcan1	regulator of calcineurin 1 [Source:MGI Symbol;Acc:MGI:1890564]
ENSMUSG00000022952	19.94738021	20.69767932	18.38595316	20.87812135	22.60415917	25.33789831	0.47077027	0.567150635	0.687117796	4.891852869	5.392596798	5.15381695	Runx1	runt related transcription factor 1 [Source:MGI Symbol;Acc:MGI:99852]
ENSMUSG00000022956	5.11935322	3.581892696	7.032437532	3.38643789	4.765937175	2.122413987	7.17385829	7.575075344	3.92047417	2.724298825	3.182523574	2.641737373	Atp5o	ATP synthase, H+ transporting, mitochondrial F1 complex, O subunit [Source:MGI Symbol;Acc:MGI:106341]
ENSMUSG00000022957	28.88599488	33.99905655	25.90064645	27.79721338	30.27043228	34.26179809	35.29605754	32.56011937	39.59464347	38.77356019	38.03686514	40.57966898	Itsn1	intersectin 1 (SH3 domain protein 1A) [Source:MGI Symbol;Acc:MGI:1338069]
ENSMUSG00000022960	7.500123766	6.877434383	6.654762134	8.683934845	7.2671861	9.140333709	20.26141754	22.17975032	21.51122922	13.46706118	13.73481216	11.76728757	Donson	downstream neighbor of SON [Source:MGI Symbol;Acc:MGI:1890621]
ENSMUSG00000022961	29.78895311	40.96450493	28.59228134	33.95628342	44.83056391	61.20663131	32.0989196	29.16450233	40.15741907	96.10168059	97.67998702	97.1081736	Son	Son DNA binding protein [Source:MGI Symbol;Acc:MGI:98353]
ENSMUSG00000022962	23.33127374	28.83648177	18.0711147	25.50558031	27.59343774	36.58873986	280.7365169	245.1061874	312.8609019	252.0155981	261.6233374	250.6379207	Gart	phosphoribosylglycinamide formyltransferase [Source:MGI Symbol;Acc:MGI:95654]
ENSMUSG00000022964	16.07289518	20.35964533	16.28390572	17.18631347	18.39996148	17.7515318	22.39122623	21.03096299	22.78282472	22.79326257	24.30530383	20.53538525	Tmem50b	transmembrane protein 50B [Source:MGI Symbol;Acc:MGI:1925225]
ENSMUSG00000022965	39.1133347	41.83008376	41.25621301	37.58707712	37.38264407	34.03959534	6.683809497	5.827235283	7.092592746	18.72295213	18.93382099	18.71948184	Ifngr2	interferon gamma receptor 2 [Source:MGI Symbol;Acc:MGI:107654]
ENSMUSG00000022967	41.61102229	45.03998405	36.96847127	38.09087596	38.92307476	42.0151599	26.87538367	21.13442365	26.74415702	43.47356559	42.5362893	38.68648119	Ifnar1	interferon (alpha and beta) receptor 1 [Source:MGI Symbol;Acc:MGI:107658]
ENSMUSG00000022969	13.35977038	12.7015208	13.97956946	10.89840797	10.69010955	9.052728113	1.500785937	1.197847674	1.070539348	4.933001858	3.535044598	3.868176761	Il10rb	interleukin 10 receptor, beta [Source:MGI Symbol;Acc:MGI:109380]
ENSMUSG00000022971	23.72954509	23.28795933	21.70609928	19.20163154	16.19274574	18.1571801	4.489417564	5.439305897	5.515296925	10.45472239	8.846745617	8.81657809	Ifnar2	interferon (alpha and beta) receptor 2 [Source:MGI Symbol;Acc:MGI:1098243]
ENSMUSG00000022972	7.386864863	8.506354614	9.453272575	8.042184402	7.567057578	6.939277617	18.85020736	20.48967786	19.82404353	18.06016162	15.83321105	15.43864382	Cfap298	cilia and flagella associate protien 298 [Source:MGI Symbol;Acc:MGI:1915251]
ENSMUSG00000022973	6.825313719	9.002608942	4.98675224	7.950901573	8.614927946	11.35111574	9.09251514	7.275739086	11.34110111	12.31942714	13.1675951	13.19360613	Synj1	synaptojanin 1 [Source:MGI Symbol;Acc:MGI:1354961]
ENSMUSG00000022974	19.00075784	26.18750478	14.36752805	26.13606068	31.12394657	41.43474932	33.68646105	29.96852018	40.77326692	49.75600103	44.53447382	45.95571893	Paxbp1	PAX3 and PAX7 binding protein 1 [Source:MGI Symbol;Acc:MGI:1914617]
ENSMUSG00000022978	11.5603204	13.37836922	10.69001271	15.28509056	15.97360582	12.08268894	35.39288555	38.76110418	37.85979791	25.94088933	20.55888652	24.60843449	Mis18a	MIS18 kinetochore protein A [Source:MGI Symbol;Acc:MGI:1913828]
ENSMUSG00000022982	83.83651163	65.4070796	107.3988511	63.7475639	57.6376652	48.37934356	288.0614296	309.6566352	222.5454586	182.5925204	168.2740802	169.2995135	Sod1	superoxide dismutase 1, soluble [Source:MGI Symbol;Acc:MGI:98351]
ENSMUSG00000022983	18.88006612	19.04226762	15.9389451	18.30083548	20.06246212	25.45268769	37.41656701	31.97471134	42.29078319	49.49002373	53.75216245	49.54800209	Scaf4	SR-related CTD-associated factor 4 [Source:MGI Symbol;Acc:MGI:2146350]
ENSMUSG00000022986	0.052698699	0.091027892	0.067440677	0	0	0	0	0	0	0	0	0	Krt75	keratin 75 [Source:MGI Symbol;Acc:MGI:1923500]
ENSMUSG00000022987	0.131616551	0.233174191	0.145113295	0.028343631	0.106372514	0.123384548	0.054232735	0	0.01884666	0.163007858	0.050565001	0.170476515	Zfp641	zinc finger protein 641 [Source:MGI Symbol;Acc:MGI:2442788]
ENSMUSG00000022991	0	0	0	0	0	0	0	0	0	0	0	0	Lalba	lactalbumin, alpha [Source:MGI Symbol;Acc:MGI:96742]
ENSMUSG00000022992	74.07259775	77.28808973	64.21658274	73.18504551	73.37738138	75.32726406	52.78715513	52.2732682	57.62268221	54.02537173	53.83284656	53.45651997	Kansl2	KAT8 regulatory NSL complex subunit 2 [Source:MGI Symbol;Acc:MGI:1916862]
ENSMUSG00000022993	1.079567795	0.35519366	1.263146959	0.287838551	0.48611095	1.115961276	0.688438245	0.152780816	0	0.827698202	0.641879489	0.341692694	4930415O2	RIKEN cDNA 4930415O20 gene [Source:MGI Symbol;Acc:MGI:1921113]
ENSMUSG00000022994	12.55514234	18.47397286	11.6478212	15.2311283	21.28504038	26.42228321	1.854319325	1.68906457	2.640127784	9.247648981	10.21864747	10.28874121	Adcy6	adenylate cyclase 6 [Source:MGI Symbol;Acc:MGI:87917]
ENSMUSG00000022995	48.15237489	55.44993498	45.51546982	41.01897683	48.19558179	57.24451732	105.7155065	100.600013	120.3314259	155.597464	154.1517821	151.6018766	Enah	ENAH actin regulator [Source:MGI Symbol;Acc:MGI:108360]
ENSMUSG00000022996	0.885546715	1.176637574	0.790383381	0.995891007	1.335971413	1.05499486	0.081086763	0.067481553	0.105670584	0.142171907	0.113404355	0.080491619	Wnt10b	wingless-type MMTV integration site family, member 10B [Source:MGI Symbol;Acc:MGI:108061]
ENSMUSG00000022997	0.144755216	0.229202974	0.27787374	0.151968587	0.133077472	0.110257429	0.01615427	0.017925083	0.033683054	0	0.045185293	0.016035704	Wnt1	wingless-type MMTV integration site family, member 1 [Source:MGI Symbol;Acc:MGI:98953]
ENSMUSG00000022999	36.34753963	29.99531676	35.38022682	33.55178537	33.88534767	28.47280906	39.44445737	43.57133785	38.6426194	29.77303868	30.55358232	28.51157107	Lmbr1l	limb region 1 like [Source:MGI Symbol;Acc:MGI:1289247]
ENSMUSG00000023000	0.052303672	0	0.053548116	0.016269643	0	0	0.420259749	0.414513916	0.373229532	0.1091636	0.101587572	0.108156537	Dhh	desert hedgehog [Source:MGI Symbol;Acc:MGI:94891]
ENSMUSG00000023004	324.7797843	215.2238872	370.1941519	316.2749708	256.9107078	193.9009239	576.0874283	605.2494815	430.6254143	314.1770999	320.1113523	294.5808884	Tuba1b	tubulin, alpha 1B [Source:MGI Symbol;Acc:MGI:107804]
ENSMUSG00000023007	36.83293025	30.28897078	37.54664363	34.60484324	35.24150555	30.88986017	32.07316139	30.03593488	30.79729057	23.50689085	26.57929525	26.80968457	Prpf40b	pre-mRNA processing factor 40B [Source:MGI Symbol;Acc:MGI:1925583]
ENSMUSG00000023008	24.08446279	27.07290822	24.48723114	23.58981551	22.15938303	24.37898865	13.64631675	13.07532152	17.3227356	19.32512378	19.89686582	18.23572849	Fmnl3	formin-like 3 [Source:MGI Symbol;Acc:MGI:109569]
ENSMUSG00000023009	34.36560955	31.11248655	32.08057556	28.6810556	28.83719974	27.43871495	11.51169381	11.81061694	10.85717862	13.91192937	16.06404714	15.07579953	Nckap5l	NCK-associated protein 5-like [Source:MGI Symbol;Acc:MGI:3609653]
ENSMUSG00000023010	831.2643461	752.4695475	874.5187447	731.6029758	670.2644812	589.6626264	374.5053057	375.1250013	360.8955948	313.2888785	321.9833929	322.5212031	Tmbim6	transmembrane BAX inhibitor motif containing 6 [Source:MGI Symbol;Acc:MGI:99682]
ENSMUSG00000023011	0.279260695	0.28299308	0.388830869	0.343993969	0.492925589	0.37436576	0.089754332	0.055329501	0.093572784	0.399667356	0.399824934	0.415779335	Faim2	Fas apoptotic inhibitory molecule 2 [Source:MGI Symbol;Acc:MGI:1919643]
ENSMUSG00000023013	0.151615531	1.04755847	0	0.594237516	0.22301496	0.069289684	0.351933124	0.450590356	0.028223504	0.027123303	0.126204635	0	Aqp2	aquaporin 2 [Source:MGI Symbol;Acc:MGI:1096865]

ENSMUSG00000023015	72.61464171	81.70797584	66.67817901	100.5659343	107.0363362	106.0073466	125.2405606	124.2017892	133.9616854	94.67690366	99.32274244	89.56328976	Racgap1	Rac GTPase-activating protein 1 [Source:MGI Symbol;Acc:MGI:1349423]
ENSMUSG00000023017	10.41692944	9.780220641	12.03390259	11.95408882	11.48695548	10.72214416	13.61486104	12.87607395	13.08039355	6.58954277	7.928926633	6.882219985	Asic1	acid-sensing (proton-gated) ion channel 1 [Source:MGI Symbol;Acc:MGI:1194915]
ENSMUSG00000023018	33.54953214	30.12507411	30.41248149	31.55164867	29.09329767	29.3061711	130.4039365	129.5042016	131.7835914	81.38218735	78.71635947	80.23454187	Smarcd1	SWI/SNF related, matrix associated, actin dependent regulator of chromatin, subfamily d, member 1 [Source:MGI Symbol;Acc:MGI:19
ENSMUSG00000023019	0.347219331	0.645896852	0.109378647	0.697887883	0.420934063	0.345846276	0.572288329	0.502725702	0.422616745	0.597268255	0.51135204	0.37872531	Gpd1	glycerol-3-phosphate dehydrogenase 1 (soluble) [Source:MGI Symbol;Acc:MGI:95679]
ENSMUSG00000023020	65.86907024	46.8121925	82.74345794	50.53744368	48.64690141	35.9753261	48.04163496	50.20225938	36.26548469	21.13910723	21.82277201	21.55470834	Cox14	cytochrome c oxidase assembly protein 14 [Source:MGI Symbol;Acc:MGI:1913629]
ENSMUSG00000023021	63.75986842	68.38100269	56.25270494	63.6665319	69.5184518	68.15406178	47.50084718	51.3865509	51.08695676	46.70945245	47.13635971	43.88881483	Cers5	ceramide synthase 5 [Source:MGI Symbol;Acc:MGI:1919199]
ENSMUSG00000023022	110.2696163	135.4993292	98.98423289	113.5575354	135.9095595	154.5537109	26.56484824	24.44019549	32.91206302	77.16231515	79.09198653	75.97034862	Lima1	LIM domain and actin binding 1 [Source:MGI Symbol;Acc:MGI:1920992]
ENSMUSG00000023025	17.48464061	23.17496075	15.21319236	23.29274169	30.98293955	39.66089119	38.08593631	38.15492397	49.47920504	75.91864647	73.89546589	81.02732084	Larp4	La ribonucleoprotein domain family, member 4 [Source:MGI Symbol;Acc:MGI:2443114]
ENSMUSG00000023026	9.199982125	9.426252474	7.883188199	9.025200695	11.96249327	13.69936653	12.61364232	12.34055086	16.86000137	16.38166431	16.56471034	16.58502827	Dip2b	disco interacting protein 2 homolog B [Source:MGI Symbol;Acc:MGI:2145977]
ENSMUSG00000023027	60.1408404	64.69316554	52.44583751	70.037524	79.95281877	87.38341819	111.3637777	112.7241088	131.670548	119.6965911	121.9239418	120.4941272	Atf1	activating transcription factor 1 [Source:MGI Symbol;Acc:MGI:1298366]
ENSMUSG00000023030	65.16749385	73.4941438	65.0451319	70.85907853	74.25878651	73.30838272	32.40806378	33.13580417	35.39712797	48.46102095	55.12545169	52.95448045	Slc11a2	solute carrier family 11 (proton-coupled divalent metal ion transporters), member 2 [Source:MGI Symbol;Acc:MGI:1345279]
ENSMUSG00000023031	16.69963221	14.96565965	18.6825662	17.97166614	17.40414134	13.43786136	9.137805663	9.205582132	5.640725567	4.517366945	5.268836142	6.027266625	Cela1	chymotrypsin-like elastase family, member 1 [Source:MGI Symbol;Acc:MGI:95314]
ENSMUSG00000023032	1.071526545	0.983278326	1.079880063	1.124922336	1.178582618	1.173233424	8.415394102	7.06560744	8.259163544	9.210155377	11.19103152	10.66831069	Slc4a8	solute carrier family 4 (anion exchanger), member 8 [Source:MGI Symbol;Acc:MGI:1928745]
ENSMUSG00000023033	0.282743946	0.162797094	0.223682274	0.179899094	0.135030819	0.264306618	4.187999324	4.417911933	4.36617094	1.563429936	2.182390262	2.118494705	Scn8a	sodium channel, voltage-gated, type VIII, alpha [Source:MGI Symbol;Acc:MGI:103169]
ENSMUSG00000023034	38.4977281	37.56529966	35.33738258	43.63839997	42.35214508	41.99759632	50.69682082	49.45359995	52.18913045	34.31250097	35.72544759	36.64847126	Nr4a1	nuclear receptor subfamily 4, group A, member 1 [Source:MGI Symbol;Acc:MGI:1352454]
ENSMUSG00000023036	0.24836068	0.314600099	0.203415874	0.173824579	0.300084076	0.387802264	0.022173076	0.049207328	0.03467459	0.211045126	0.485827748	0.363170521	Pcdhgc4	protocadherin gamma subfamily C, 4 [Source:MGI Symbol;Acc:MGI:1935203]
ENSMUSG00000023039	0.414621403	0.769237662	0.754642402	0.601872259	0.242014552	0.263174684	0.308470456	0.251008739	0.343033418	1.092003329	0.843652313	0.796136661	Krt7	keratin 7 [Source:MGI Symbol;Acc:MGI:96704]
ENSMUSG00000023041	0	0	0	0	0	0	0	0	0	0.034441133	0	0	Krt6b	keratin 6B [Source:MGI Symbol;Acc:MGI:1333768]
ENSMUSG00000023043	0.299432762	0.379293439	0.429179891	0.33531088	0.314602076	0.182458013	9.276238248	9.492185988	7.998682315	15.85586367	13.78339049	14.91349508	Krt18	keratin 18 [Source:MGI Symbol;Acc:MGI:96692]
ENSMUSG00000023044	50.5611828	54.72912494	49.65587396	56.79137986	53.74893265	52.50447639	84.81264381	81.2253244	90.08537106	46.4860954	52.19159311	47.35395973	Csad	cysteine sulfinic acid decarboxylase [Source:MGI Symbol;Acc:MGI:2180098]
ENSMUSG00000023045	0.314076553	0.387509246	0.367484882	0.942078469	0.824969313	0.751854488	3.745348129	4.978201191	3.967329921	2.167205614	2.035474154	1.749580611	Soat2	sterol O-acyltransferase 2 [Source:MGI Symbol;Acc:MGI:1332226]
ENSMUSG00000023046	277.7891314	221.6806117	312.129208	338.7769146	281.2510309	225.4687571	1.865469532	1.993295036	1.080462721	3.807262861	3.993968129	2.846250979	Igfbp6	insulin-like growth factor binding protein 6 [Source:MGI Symbol;Acc:MGI:96441]
ENSMUSG00000023047	0.398822235	0.408234938	0.907358397	0.330821651	0.884611791	0.810068481	10.7609066	14.44274345	12.6622961	6.540179662	6.528925808	7.108193356	Amhr2	anti-Mullerian hormone type 2 receptor [Source:MGI Symbol;Acc:MGI:105062]
ENSMUSG00000023048	199.355317	168.162065	224.9607433	185.6748058	159.9644285	119.5287811	78.43830622	83.38176788	68.39899956	68.26529629	68.8841005	67.14128582	Prr13	proline rich 13 [Source:MGI Symbol;Acc:MGI:1913401]
ENSMUSG00000023050	10.71659701	15.1614466	9.809877399	14.01758079	16.53916711	17.20327412	9.985610874	8.992644303	11.38274336	11.29345518	10.73456795	10.69445213	Map3k12	mitogen-activated protein kinase kinase kinase 12 [Source:MGI Symbol;Acc:MGI:1346881]
ENSMUSG00000023051	50.95262728	38.20229831	51.57644179	49.00410129	43.95732481	36.12022313	99.48274232	107.0527015	100.6578866	76.05780377	80.69049905	80.12276348	Tarbp2	TARBP2, RISC loading complex RNA binding subunit [Source:MGI Symbol;Acc:MGI:103027]
ENSMUSG00000023052	1.790367039	1.786806389	2.321755212	2.673185764	2.069174071	1.500059197	1.225439173	0.354722727	0.33328004	1.067627361	0.39741332	0.264444554	Npff	neuropeptide FF-amide peptide precursor [Source:MGI Symbol;Acc:MGI:1891708]
ENSMUSG00000023055	49.68671867	53.42862143	50.22225955	50.88563063	51.73380039	49.21114414	29.9800329	26.74869709	29.95725524	39.21770763	38.92757659	41.79459645	Calcoco1	calcium binding and coiled coil domain 1 [Source:MGI Symbol;Acc:MGI:1914738]
ENSMUSG00000023057	0	0.051346648	0	0.041609822	0.140543995	0	0	0	0.083003423	0	0	0.039515963	Fabp2	fatty acid binding protein 2, intestinal [Source:MGI Symbol;Acc:MGI:95478]
ENSMUSG00000023064	6.817894198	6.51355135	9.565336017	4.571464026	4.510219554	3.423577273	19.61321775	15.35931367	15.04193484	7.498829129	6.768212385	7.429650444	Sncg	synuclein, gamma [Source:MGI Symbol;Acc:MGI:1298397]
ENSMUSG00000023066	5.152278478	5.795666876	4.297283769	7.369824642	7.733276833	8.636704467	8.063034558	7.764486359	10.75753908	10.17804751	12.51851958	12.02341239	Rttn	rotatin [Source:MGI Symbol;Acc:MGI:2179288]
ENSMUSG00000023067	371.1016211	371.3927853	402.285524	352.4899697	333.8805967	294.5009761	111.4548782	113.2050547	105.395496	169.9042944	166.8319047	168.1474357	Cdkn1a	cyclin-dependent kinase inhibitor 1A (P21) [Source:MGI Symbol;Acc:MGI:104556]
ENSMUSG00000023068	45.70736893	53.78402566	43.8307965	41.55093066	46.16982551	48.17248741	37.6869624	35.85120601	40.46729812	53.59441876	53.36520542	55.69527495	Nus1	NUS1 dehydrodolichyl diphosphate synthase subunit [Source:MGI Symbol;Acc:MGI:1196365]
ENSMUSG00000023070	0	0.03083355	0.164476354	0	0	0.020394537	0	0.053050215	0	0.071850591	0.066864111	0.0474585	Rgn	regucalcin [Source:MGI Symbol;Acc:MGI:108024]
ENSMUSG00000023072	19.54132198	15.31923889	20.46794507	21.00605522	18.75454255	17.28865368	33.55009089	37.7862318	33.15859882	32.37022849	29.99858828	29.17416988	Cep89	centrosomal protein 89 [Source:MGI Symbol;Acc:MGI:1919390]
ENSMUSG00000023073	0	0.011933891	0	0	0	0	0	0	0	0.009269741	0	0	Slc10a2	solute carrier family 10, member 2 [Source:MGI Symbol;Acc:MGI:1201406]
ENSMUSG00000023074	15.62907285	15.42658354	12.72123638	13.11272626	15.73239279	15.52745479	5.438312675	5.337246582	5.014614573	7.315535627	7.232059459	8.818099787	Mospd1	motile sperm domain containing 1 [Source:MGI Symbol;Acc:MGI:1917630]
ENSMUSG00000023075	99.97884139	114.0920014	82.65568957	114.2031131	126.6843861	128.0729024	60.83023014	61.71960617	66.86053318	68.2078845	66.4526935	71.60245625	Akirin1	akirin 1 [Source:MGI Symbol;Acc:MGI:1915300]
ENSMUSG00000023078	0	0	0	0	0	0	0	0.037065955	0	0	0	0	Cxcl13	chemokine (C-X-C motif) ligand 13 [Source:MGI Symbol;Acc:MGI:1888499]
ENSMUSG00000023079	24.54859645	32.078451	22.18962762	30.46639808	32.28823161	37.53546372	26.07158707	22.89500727	26.62865112	29.89653804	30.06262666	28.08409577	Gtf2ird1	general transcription factor II I repeat domain-containing 1 [Source:MGI Symbol;Acc:MGI:1861942]
ENSMUSG00000023083	0.077319834	0	0.079159479	0	0	0.088339678	0	0	0	0.069160766	0	0.10278411	H2-M10.2	histocompatibility 2, M region locus 10.2 [Source:MGI Symbol;Acc:MGI:1276525]
ENSMUSG00000023084	0.165494184	0.245025077	0.701931459	0.48537171	0.794871531	0.720308146	0.105535313	0.163945568	0.022005028	0.084588937	0.295194029	0.419042909	Lrrc71	leucine rich repeat containing 71 [Source:MGI Symbol;Acc:MGI:1921735]
ENSMUSG00000023087	17.20932794	16.72281274	16.02092405	32.71693414	31.61215823	32.52209446	26.07743877	26.87793664	30.80407955	26.79272797	26.51636085	26.66463741	Noct	nocturnin [Source:MGI Symbol;Acc:MGI:109382]
ENSMUSG00000023088	32.96739328	40.74561333	27.34225594	34.88857472	39.31158117	46.36160673	20.7658523	17.68658932	24.50114131	58.52911558	58.9917795	54.89519487	Abcc1	ATP-binding cassette, sub-family C (CFTR/MRP), member 1 [Source:MGI Symbol;Acc:MGI:102676]
ENSMUSG00000023089	78.18085553	58.61927282	113.5682288	78.99171651	68.11735058	50.13983119	104.2900374	112.7654892	62.58162213	64.814032	59.69479247	58.63209623	Ndufa5	NADH:ubiquinone oxidoreductase subunit A5 [Source:MGI Symbol;Acc:MGI:1915452]
ENSMUSG00000023092	7.127055372	6.436328834	6.236950887	6.67844786	5.313142569	6.419653527	13.83462786	11.7184461	14.75350187	36.82147711	36.75229903	38.10669651	Fhl1	four and a half LIM domains 1 [Source:MGI Symbol;Acc:MGI:1298387]
ENSMUSG00000023093	0	0	0	0	0	0	0	0	0	0	0	0	Pate12	prostate and testis expressed 12 [Source:MGI Symbol;Acc:MGI:3647831]
ENSMUSG00000023094	15.57521132	11.95710432	15.38204714	6.680011852	4.958864901	5.931678324	24.6501166	27.04052861	22.25766373	24.34522187	25.86410569	23.35378989	Msrb2	methionine sulfoxide reductase B2 [Source:MGI Symbol;Acc:MGI:1923717]
ENSMUSG00000023104	69.4402832	57.97561889	74.25609654	75.38298583	69.2520378	58.02067475	122.8442488	133.0758172	120.2017217	68.5012789	73.52651267	72.85554961	Rfc2	replication factor C (activator 1) 2 [Source:MGI Symbol;Acc:MGI:1341868]
ENSMUSG00000023106	100.46347	82.79199345	111.0664216	98.30854656	83.65115596	72.21922747	121.1151899	139.8787329	107.6527288	95.9636549	94.80002425	95.4410747	Denr	density-regulated protein [Source:MGI Symbol;Acc:MGI:1915434]
ENSMUSG00000023110	60.19328171	62.07105387	61.42664852	73.05903548	69.24053812	66.2875273	197.6908826	194.5092834	206.2459013	172.1190193	181.1532222	180.2364818	Prmt5	protein arginine N-methyltransferase 5 [Source:MGI Symbol;Acc:MGI:1351645]
ENSMUSG00000023118	72.49493169	73.30426968	70.14907728	72.53709239	71.67979478	67.85691555	84.14012868	80.864314	93.54573087	80.0218697	84.77425148	80.41751629	Sympk	symplekin [Source:MGI Symbol;Acc:MGI:1915438]
ENSMUSG00000023120	0	0.037395861	0	0	0.068238971	0	0.115969371	0.160852255	0.030225775	0.029047522	0.054063208	0.201456855	Ldc1	leucine decarboxylase 1 [Source:MGI Symbol;Acc:MGI:2685699]
ENSMUSG00000023122	0.06992712	0.134207789	0	0.195764518	0.02448988	0.017754074	0	0	0.021695098	0.041698772	0.038804849	0	Sult1c2	sulfotransferase family, cytosolic, 1C, member 2 [Source:MGI Symbol;Acc:MGI:1916333]
ENSMUSG00000023132	0.045921545	0	0	0	0	0	0	0	0	0	0	0.12209041	Gzma	granzyme A [Source:MGI Symbol;Acc:MGI:109266]
ENSMUSG00000023140	0	0	0	0	0	0	0.33068741	0.366936996	0.632052607	0.441755712	0.61664635	0.164130149	Reg2	regenerating islet-derived 2 [Source:MGI Symbol;Acc:MGI:97896]
ENSMUSG00000023143	15.14307297	15.3114383	15.37732339	12.60899626	12.2883841	9.049193109	10.57919801	11.9218215	11.8314017	11.31513174	11.70837775	10.74704181	Nagpa	N-acetylglucosamine-1-phosphodiester alpha-N-acetylglucosaminidase [Source:MGI Symbol;Acc:MGI:1351598]
ENSMUSG00000023147	15.5303339	15.70456834	13.81975507	12.83041271	14.06545998	14.07629347	28.07812749	29.77743565	32.89936768	28.00709067	29.93234354	27.74871784	Get1	guided entry of tail-anchored proteins factor 1 [Source:MGI Symbol;Acc:MGI:2136882]
ENSMUSG00000023150	36.81802687	48.32403849	33.43352765	39.45683204	44.1736503	49.7235972	131.3819206	126.1939087	147.6008597	113.5174793	110.9014217	111.7242025	Ivns1abp	influenza virus NS1A binding protein [Source:MGI Symbol;Acc:MGI:2152389]
ENSMUSG00000023151	0	0.048293875	0.171743665	0	0	0	0.037441378	0.124636982	0.156137043	0.187563189	0	0.037166574	Lrrc69	leucine rich repeat containing 69 [Source:MGI Symbol;Acc:MGI:1920564]
ENSMUSG00000023153	0.098463359	0.113385619	0	0	0	0	0.96696429	1.560671769	0.824810901	0.352292598	0.12294122	0.218151629	Tmem52	transmembrane protein 52 [Source:MGI Symbol;Acc:MGI:1916921]
ENSMUSG00000023156	21.26007981	22.38164361	20.32709808	23.80188783	22.43136852	24.68869935	31.42062769	25.741391	25.9108826	26.61259662	26.01017017	25.78409747	Rpp14	ribonuclease P 14 subunit [Source:MGI Symbol;Acc:MGI:1914303]
ENSMUSG00000023159	0	0.009100003	0.008090404	0	0	0	0	0	0	0	0	0	Psg29	pregnancy-specific glycoprotein 29 [Source:MGI Symbol;Acc:MGI:1891361]
ENSMUSG00000023165	0	0	0	0	0	0	0	0	0	0	0	0	Ssxb2	synovial sarcoma, X member B2 [Source:MGI Symbol;Acc:MGI:2446771]
ENSMUSG00000023169	5.111175538	5.44760449	4.580002112	4.424176215	4.862271333	5.342228029	21.44768143	19.16329339	25.85390972	22.7211465	22.92485481	22.71204553	Slc38a1	solute carrier family 38, member 1 [Source:MGI Symbol;Acc:MGI:2145895]
ENSMUSG00000023170	49.26784965	38.0003804	44.33615299	49.68507676	45.01408282	37.66724176	34.53623293	38.55098149	35.01612174	28.524916	32.43139208	29.73629302	Gps2	G protein pathway suppressor 2 [Source:MGI Symbol;Acc:MGI:1891751]
ENSMUSG00000023175	1664.81205	1420.076004	1793.775984	1427.08041	1304.802655	1058.681408	653.6334622	680.3214218	587.6020596	553.5646354	550.2866833	523.5525269	Bsg	basigin [Source:MGI Symbol;Acc:MGI:88208]

ENSMUSG00000023176	0.028370432	0.010890003	0.02904544	0	0	0.064827733	0	0	0	0.093047831	0.070846567	0.041904291	Cpn2	carboxypeptidase N, polypeptide 2 [Source:MGI Symbol;Acc:MGI:1919006]
ENSMUSG00000023185	0	0	0	0	0	0	0	0	0	0	0	0	Ceacam14	carcinoembryonic antigen-related cell adhesion molecule 14 [Source:MGI Symbol;Acc:MGI:1914334]
ENSMUSG00000023186	51.71581125	67.14026507	44.70885555	73.17944131	85.8222954	109.1801216	9.395502927	7.653014662	11.53176788	34.05507052	37.97656513	35.75605697	Vwa5a	von Willebrand factor A domain containing 5A [Source:MGI Symbol;Acc:MGI:1915026]
ENSMUSG00000023191	97.54807585	86.92068368	92.2091488	76.62450172	66.36965885	64.29767512	6.792354975	5.941974368	6.229215057	21.06531395	23.09833716	22.35377171	P3h3	prolyl 3-hydroxylase 3 [Source:MGI Symbol;Acc:MGI:1315208]
ENSMUSG00000023192	0	0	0	0	0	0.013869666	0.130054521	0.036077738	0.305071827	0.309467356	0.136416369	0.161374967	Grm2	glutamate receptor, metabotropic 2 [Source:MGI Symbol;Acc:MGI:1351339]
ENSMUSG00000023206	4.306593647	5.692372552	4.715776131	4.228521165	4.24934585	3.850733495	0.167166467	0.185491069	0.139422634	1.875827778	2.213227292	2.356341369	Il15ra	interleukin 15 receptor, alpha chain [Source:MGI Symbol;Acc:MGI:104644]
ENSMUSG00000023210	0	0	0	0	0	0	0	0	0	0	0	0	Lcn9	lipocalin 9 [Source:MGI Symbol;Acc:MGI:1924954]
ENSMUSG00000023216	0	0	0	0.022050235	0.049652189	0	0	0	0	0	0.03933759	0	Epb42	erythrocyte membrane protein band 4.2 [Source:MGI Symbol;Acc:MGI:95402]
ENSMUSG00000023224	150.5848186	146.5225469	156.7284555	78.08392576	76.49435181	60.64894892	4.067455137	4.336331911	4.3513611	9.961591296	11.50105898	12.16558413	Serping1	serine (or cysteine) peptidase inhibitor, clade G, member 1 [Source:MGI Symbol;Acc:MGI:894696]
ENSMUSG00000023232	6.764447856	5.927702789	6.65513289	2.401818459	2.010799436	1.60854089	5.243745418	5.720493365	5.343981875	10.1532672	9.338755876	10.81991928	Serinc2	serine incorporator 2 [Source:MGI Symbol;Acc:MGI:1919132]
ENSMUSG00000023235	9.784454708	9.759080964	9.386244894	11.10781079	10.11824493	10.29872985	4.677189036	4.846448217	4.410067715	7.752723754	7.567397147	6.622903769	Ccl25	chemokine (C-C motif) ligand 25 [Source:MGI Symbol;Acc:MGI:1099448]
ENSMUSG00000023236	1.002883734	0.769914782	1.760134303	1.381529204	0.752635711	0.472877101	0.383722183	0.520404356	0.311147681	0.042716944	0.278266513	0.211614343	Scg5	secretogranin V [Source:MGI Symbol;Acc:MGI:98289]
ENSMUSG00000023243	1.425066082	2.081912421	1.458972201	1.647423955	2.033592175	2.52730955	23.01473215	19.15318614	22.33091593	24.90397824	26.57495309	23.78829744	Kcnk5	potassium channel, subfamily K, member 5 [Source:MGI Symbol;Acc:MGI:1336175]
ENSMUSG00000023247	0.796823849	0.250250079	1.112430561	0.608386028	0.45664968	0.49657607	0.064671472	0.215282059	0	0.323972781	0.241191081	0	Guca2a	guanylate cyclase activator 2a (guanylin) [Source:MGI Symbol;Acc:MGI:102738]
ENSMUSG00000023249	38.74289646	44.31634486	33.27476118	33.76542454	32.45570448	32.44540412	2.38808919	3.07726708	2.221978326	12.42626187	11.53992913	11.7508483	Parp3	poly (ADP-ribose) polymerase family, member 3 [Source:MGI Symbol;Acc:MGI:1891258]
ENSMUSG00000023257	0	0	0	0	0	0.049136463	0	0	0	0	0	0	Cypt1	cysteine-rich perinuclear theca 1 [Source:MGI Symbol;Acc:MGI:3616441]
ENSMUSG00000023259	2.081091216	2.440862839	1.617682273	2.157822365	2.469957384	2.274954411	1.840747598	2.061617354	1.919059209	1.62018309	2.117253723	1.69062139	Slc26a6	solute carrier family 26, member 6 [Source:MGI Symbol;Acc:MGI:2159728]
ENSMUSG00000023262	16.37235818	19.06737184	19.84061657	17.49569138	18.76203721	13.48853136	66.45537741	70.43011074	63.85749095	29.65466261	32.47932367	29.97331853	Acy1	aminoacylase 1 [Source:MGI Symbol;Acc:MGI:87913]
ENSMUSG00000023263	0	0.123410998	0	0	0	0	0	0	0.04987441	0	0	0	9530002B09	RIKEN cDNA 9530002B09 gene [Source:MGI Symbol;Acc:MGI:1924682]
ENSMUSG00000023266	8.74596301	5.985847525	8.785108381	7.0451452	8.452178407	6.347413393	13.70117518	12.75097266	11.67300294	5.867293052	5.906522131	5.776604929	Frs3	fibroblast growth factor receptor substrate 3 [Source:MGI Symbol;Acc:MGI:2135965]
ENSMUSG00000023267	0.166518916	0.191755091	0.034096169	0	0	0.025366889	0.059465718	0.098976427	0	0	0.08316612	0.029514632	Gabrr2	gamma-aminobutyric acid (GABA) C receptor, subunit rho 2 [Source:MGI Symbol;Acc:MGI:95626]
ENSMUSG00000023272	62.33775799	53.24940625	68.91629478	51.26476233	46.10189419	36.94874947	60.3782516	67.9017896	56.61413733	50.3499238	50.29045913	49.1596231	Creld2	cysteine-rich with EGF-like domains 2 [Source:MGI Symbol;Acc:MGI:1923987]
ENSMUSG00000023274	0.032771126	0.113212907	0.100652514	0	0	0	0.321830461	0.064928938	0.183012117	0.205190986	0.245507905	0.319468356	Cd4	CD4 antigen [Source:MGI Symbol;Acc:MGI:88335]
ENSMUSG00000023277	38.68345425	39.15383293	42.75659578	33.57932999	31.20260404	29.78580111	120.2535579	129.6327251	112.4909991	67.42940775	67.77114674	69.42431765	Twf2	twinfilin actin binding protein 2 [Source:MGI Symbol;Acc:MGI:1346078]
ENSMUSG00000023279	0	0	0	0	0	0	0	0	0	0.075466601	0.023409723	0	Bmp15	bone morphogenetic protein 15 [Source:MGI Symbol;Acc:MGI:1316745]
ENSMUSG00000023284	11.179457	11.58634748	9.995689566	13.84043087	14.43429564	12.96201163	3.548716311	2.830238156	4.347136539	5.666530846	4.983756923	5.504172155	Zfp605	zinc finger protein 605 [Source:MGI Symbol;Acc:MGI:2444933]
ENSMUSG00000023286	77.35043007	67.31766224	75.81805249	69.45296869	65.37546661	55.17813631	109.4223109	113.5912238	104.284236	75.56270273	78.12555964	69.53296839	Ube2j2	ubiquitin-conjugating enzyme E2J 2 [Source:MGI Symbol;Acc:MGI:2153608]
ENSMUSG00000023289	0	0	0	0.061965244	0	0	0	0	0	0	0	0	Sva	seminal vesicle antigen [Source:MGI Symbol;Acc:MGI:102785]
ENSMUSG00000023307	52.12421341	56.00409371	38.24505029	54.99961551	55.29379342	59.43806395	41.74094518	41.26261856	49.76466332	55.84057524	57.60873419	56.30701137	Marchf5	membrane associated ring-CH-type finger 5 [Source:MGI Symbol;Acc:MGI:1915207]
ENSMUSG00000023328	4.475227933	3.855547826	4.921091124	3.804990581	5.085065084	4.620682416	0.384740449	0.722471995	0.64794467	1.512239049	1.407288636	0.998858829	Ache	acetylcholinesterase [Source:MGI Symbol;Acc:MGI:87876]
ENSMUSG00000023330	13.57583306	11.92368023	15.8170799	15.64421214	12.81305279	13.42007262	12.41366178	14.29545236	13.12535218	13.2017547	11.98456185	13.37127827	Dtwd1	DTW domain containing 1 [Source:MGI Symbol;Acc:MGI:1916435]
ENSMUSG00000023333	0	0	0	0	0.021860889	0	0	0.082448448	0.096830637	0.223334428	0.190515295	0.092197545	Gcm1	glial cells missing homolog 1 [Source:MGI Symbol;Acc:MGI:108045]
ENSMUSG00000023336	0.11264002	0.216184623	0.230640053	0.035037939	0.039448794	0.171591731	0.569853856	0.409148625	0.104840711	0.335846129	0.281284362	0.199648716	Wfdc1	WAP four-disulfide core domain 1 [Source:MGI Symbol;Acc:MGI:1915116]
ENSMUSG00000023341	57.70879544	55.99095265	60.19610424	70.50478314	66.96149741	61.48020695	0.926475892	1.328923487	1.696199359	9.237111929	7.753300104	7.357407493	Mx2	MX dynamin-like GTPase 2 [Source:MGI Symbol;Acc:MGI:97244]
ENSMUSG00000023345	8.841470102	5.392374988	6.034554822	7.395084959	6.571376564	6.360255146	23.00798337	22.9024722	22.92006236	16.40278628	16.57280083	16.51265051	Poc1a	POC1 centriolar protein A [Source:MGI Symbol;Acc:MGI:1917485]
ENSMUSG00000023348	153.1389422	131.9548259	175.452739	157.7704247	133.9757245	111.544199	45.21576708	46.65060585	43.91655886	35.55594318	35.77844536	36.3326388	Trip6	thyroid hormone receptor interactor 6 [Source:MGI Symbol;Acc:MGI:1343458]
ENSMUSG00000023349	50.54358048	45.43358142	54.98321324	1.154572845	0.577742088	0.349031104	1.513684346	1.271058291	1.535430729	30.9051402	30.40047897	31.83833576	Clec4n	C-type lectin domain family 4, member n [Source:MGI Symbol;Acc:MGI:1861231]
ENSMUSG00000023350	0	0	0	0	0	0	0	0	0	0	0	0	Phf8-ps	PHD finger protein 8, pseudogene [Source:MGI Symbol;Acc:MGI:1921292]
ENSMUSG00000023353	72.05806163	66.39905118	75.04486873	73.10777627	65.01621369	59.42188919	42.18306864	41.39469243	42.03524024	26.82621743	29.60991831	26.76576634	Agap3	ArfGAP with GTPase domain, ankyrin repeat and PH domain 3 [Source:MGI Symbol;Acc:MGI:2183446]
ENSMUSG00000023367	268.5330393	226.0405743	283.0103145	242.6353622	221.8334733	189.7590606	10.8257061	12.08521045	9.815633208	59.78617492	58.23683255	56.75973772	Tmem176a	transmembrane protein 176A [Source:MGI Symbol;Acc:MGI:1913308]
ENSMUSG00000023387	0	0.032555529	0	0	0	0.064600566	0	0	0	0	0	0.125272354	Kcnk16	potassium channel, subfamily K, member 16 [Source:MGI Symbol;Acc:MGI:1921821]
ENSMUSG00000023391	2.220825178	1.854110907	1.705248395	1.7874774	2.245832589	2.579632592	0.099135107	0.137502735	0.103352649	0.297971353	0.277291939	0.049203748	Dlx2	distal-less homeobox 2 [Source:MGI Symbol;Acc:MGI:94902]
ENSMUSG00000023393	3.683530002	3.402741901	3.729729556	2.153102763	1.616101691	2.343203733	31.62088176	33.24254727	31.6098195	31.68106357	31.16708355	27.01230219	Slc17a9	solute carrier family 17, member 9 [Source:MGI Symbol;Acc:MGI:1919107]
ENSMUSG00000023403	4.054607507	4.24982384	4.269679635	5.096402956	5.407610387	5.899323715	3.575536417	3.770748066	4.405406667	2.516520878	3.044434416	3.299962906	Stk31	serine threonine kinase 31 [Source:MGI Symbol;Acc:MGI:1924735]
ENSMUSG00000023411	41.02064738	37.3167269	39.63029345	28.71529234	26.93365901	27.72365609	17.84216771	16.99945635	17.14529601	22.05279028	23.63175289	20.62859792	Nfatc4	nuclear factor of activated T cells, cytoplasmic, calcineurin dependent 4 [Source:MGI Symbol;Acc:MGI:1920431]
ENSMUSG00000023433	0	0	0	0	0.047537664	0	0	0	0	0	0	0	Cela3b	chymotrypsin-like elastase family, member 3B [Source:MGI Symbol;Acc:MGI:1915118]
ENSMUSG00000023439	0.342583502	0.236701508	0.315661028	0.127877318	0.071987768	0.052187931	0.183510449	0	0.063772534	0.061286571	0.285166206	0.030360593	Gnb3	guanine nucleotide binding protein (G protein), beta 3 [Source:MGI Symbol;Acc:MGI:95785]
ENSMUSG00000023443	0.335374028	0.11586013	0.412024164	0.031296553	0.070472827	0.17881383	2.245607214	1.495060909	1.342255273	2.219882706	2.289153382	1.84274364	Esx1	extraembryonic, spermatogenesis, homeobox 1 [Source:MGI Symbol;Acc:MGI:1096388]
ENSMUSG00000023452	20.22728914	17.27496708	19.69707864	20.28296524	22.50855246	19.05051834	24.85940103	22.35603372	19.21391318	13.99985242	14.45659989	14.99971055	Pisd	phosphatidylserine decarboxylase [Source:MGI Symbol;Acc:MGI:2445114]
ENSMUSG00000023456	1121.0908	1034.930449	1162.463202	1225.299127	1129.983488	976.3859177	731.5607314	750.4181013	667.5919141	606.8256624	624.7857345	603.1209265	Tpi1	triosephosphate isomerase 1 [Source:MGI Symbol;Acc:MGI:98797]
ENSMUSG00000023460	70.51891061	76.25980139	60.80128283	66.46934222	73.65973398	68.87665274	32.01237839	29.3792735	33.33952805	46.20560546	41.81744576	39.78797868	Rab12	RAB12, member RAS oncogene family [Source:MGI Symbol;Acc:MGI:894284]
ENSMUSG00000023467	0.428636277	0.949224437	1.012695407	0.83076161	0.623563012	0.85388253	8.418887559	6.761341359	5.76953294	3.421152566	2.662250515	3.185047212	Tulp2	tubby-like protein 2 [Source:MGI Symbol;Acc:MGI:1861600]
ENSMUSG00000023473	0.055794201	0.089949847	0.045697355	0.031239739	0.046896596	0.20398768	0.637590939	0.663265265	1.381363369	1.5171605	1.22609636	1.374604189	Celsr3	cadherin, EGF LAG seven-pass G-type receptor 3 [Source:MGI Symbol;Acc:MGI:1858236]
ENSMUSG00000023484	3.161276657	2.325793591	3.446264471	2.567637358	2.675594351	1.98427744	19.54715023	20.35601707	17.95400738	2.880051905	3.849704983	3.086949427	Prph	peripherin [Source:MGI Symbol;Acc:MGI:97774]
ENSMUSG00000023495	282.0257667	232.461811	302.1288456	244.3318182	214.655294	179.5000385	243.0841586	251.054173	222.6876071	135.5395895	136.5418293	139.1197477	Pcbp4	poly(rC) binding protein 4 [Source:MGI Symbol;Acc:MGI:1890471]
ENSMUSG00000023505	35.53392452	31.90233112	33.21096852	41.37130429	37.80418302	32.94442282	159.2185849	155.0265882	139.0267773	93.51842089	90.90782346	88.6573512	Cdca3	cell division cycle associated 3 [Source:MGI Symbol;Acc:MGI:1315198]
ENSMUSG00000023571	13.20735529	13.48847925	16.2748591	10.54028793	7.409141063	7.920388321	16.91320658	17.05033908	14.1840745	10.63764626	11.98870415	11.1220972	C1qtnf12	C1q and tumor necrosis factor related 12 [Source:MGI Symbol;Acc:MGI:1914639]
ENSMUSG00000023572	42.75139545	40.88860673	38.90539116	44.80788281	45.37467831	39.98008405	30.18057532	29.25380164	30.54346514	33.95580474	32.88430702	33.04617867	Ccndbp1	cyclin D-type binding-protein 1 [Source:MGI Symbol;Acc:MGI:109595]
ENSMUSG00000023707	32.34923542	23.31440497	37.03669019	26.69977895	19.96272175	19.48817153	12.35472556	13.00374878	12.300515	7.16425434	7.222637765	9.069873918	Ogfod2	2-oxoglutarate and iron-dependent oxygenase domain containing 2 [Source:MGI Symbol;Acc:MGI:1913877]
ENSMUSG00000023723	50.22488997	44.94561173	46.2523045	49.3407428	45.26082511	36.4663319	72.41266917	72.67682279	62.05060178	54.31228704	53.16372458	50.75529474	Mrps23	mitochondrial ribosomal protein S23 [Source:MGI Symbol;Acc:MGI:1928138]
ENSMUSG00000023737	0	0.039138638	0	0.031716807	0	0.025887852	0	0.033669706	0.063268802	0	0.028291371	0	1700082M2	RIKEN cDNA 1700082M22 gene [Source:MGI Symbol;Acc:MGI:1920784]
ENSMUSG00000023755	11.11972157	9.059615216	10.57433994	10.58181541	10.20535072	11.51610122	46.53730023	48.11191186	42.29910386	26.92978239	25.7559554	23.95478691	Rhebl1	Ras homolog enriched in brain like 1 [Source:MGI Symbol;Acc:MGI:1916409]
ENSMUSG00000023764	12.58694167	11.96169578	13.64333007	13.14301981	13.45763085	11.8819903	2.986721902	3.094402174	3.079380637	6.331997904	6.354110641	6.617565934	Sfi1	Sfi1 homolog, spindle assembly associated (yeast) [Source:MGI Symbol;Acc:MGI:1926137]
ENSMUSG00000023781	0.404358371	0.383467783	0.365275706	0.488323114	0.599778685	0.271758048	0.14864786	0.353448157	0.354221351	0.085103298	0.079197071	0.274034141	Hes7	hes family bHLH transcription factor 7 [Source:MGI Symbol;Acc:MGI:2135679]
ENSMUSG00000023791	44.83685444	39.13738008	45.79473723	44.1241555	35.20548895	36.90622101	65.10106914	66.44152528	60.34600211	40.86670348	44.05165773	40.48969731	Pigx	phosphatidylinositol glycan anchor biosynthesis, class X [Source:MGI Symbol;Acc:MGI:1919334]
ENSMUSG00000023795	1.130277307	1.615745075	0.71824321	2.218625134	1.310386039	1.870256612	0.382756698	0.193051846	0.362764054	1.255042382	1.330155702	0.75989484	Pisd-ps2	phosphatidylserine decarboxylase, pseudogene 2 [Source:MGI Symbol;Acc:MGI:3612472]
ENSMUSG00000023800	5.002009168	6.566803275	4.418135155	6.472149635	7.242752402	9.17800643	2.164040949	2.290219756	2.660379977	5.338936397	5.504905642	5.128261034	Tiam2	T cell lymphoma invasion and metastasis 2 [Source:MGI Symbol;Acc:MGI:1344338]

ENSMUSG00000023802	0.024011433	0.110601597	0.049165458	0	13.63301104	16.40183905	0	3.158781104	0	0.022348857	0.064432979	0.099935478	0.063838568	Nox3	NADPH oxidase 3 [Source:MGI Symbol;Acc:MGI:2681162]
ENSMUSG00000023805	9.701383197	11.98943481	8.958459368	13.63301104	16.40183905	19.19303832	3.158781104	3.404540149	4.922044263	5.762418779	6.238660008	5.911555516	Synj2	synaptojanin 2 [Source:MGI Symbol;Acc:MGI:1201671]
ENSMUSG00000023806	8.746857221	8.621093228	7.329138287	9.903113163	9.110522268	7.37271466	2.813038523	2.771803805	3.448872451	2.435091018	2.475917579	2.256252709	Rsph3b	radial spoke 3B homolog (Chlamydomonas) [Source:MGI Symbol;Acc:MGI:3630308]
ENSMUSG00000023809	6.983013067	8.755147464	7.130491346	10.92310711	10.66992937	12.74853854	12.89176459	12.60713631	14.81479207	15.7865896	17.96914431	16.07722041	Rps6ka2	ribosomal protein S6 kinase, polypeptide 2 [Source:MGI Symbol;Acc:MGI:1342290]
ENSMUSG00000023826	0.443287993	0.544500172	0.605113327	0.330935257	0.124198677	0.315134814	0.870666335	1.0246598	1.07274513	0.607982985	0.762584574	0.209521454	Prkn	parkin RBR E3 ubiquitin protein ligase [Source:MGI Symbol;Acc:MGI:1355296]
ENSMUSG00000023827	58.6921627	45.04013392	56.22269852	55.03158122	46.04097427	42.07715542	49.77699401	49.22985227	47.59904574	29.64782675	32.94531666	30.65505447	Agpat4	1-acylglycerol-3-phosphate O-acyltransferase 4 (lysophosphatidic acid acyltransferase, delta) [Source:MGI Symbol;Acc:MGI:1915512
ENSMUSG00000023828	0	0.012984674	0	0	0	0.051531479	0	0	0	0	0.009385974	0.0099929	Slc22a3	solute carrier family 22 (organic cation transporter), member 3 [Source:MGI Symbol;Acc:MGI:1333817]
ENSMUSG00000023829	0.185068765	0.603829223	0.126314696	0.460541681	0.19444438	0.117469608	0.220300238	0.152780816	0.028709069	0	0.02567518	0.082006247	Slc22a1	solute carrier family 22 (organic cation transporter), member 1 [Source:MGI Symbol;Acc:MGI:108111]
ENSMUSG00000023830	119.198785	161.7839559	109.2261711	153.6064505	183.877191	242.6088961	24.86758669	21.2871836	32.09252661	77.60345393	85.83289272	79.83501241	Igf2r	insulin-like growth factor 2 receptor [Source:MGI Symbol;Acc:MGI:96435]
ENSMUSG00000023832	32.03119511	28.61761096	29.66150336	37.08652594	34.29138601	32.40815319	77.80023618	76.61132087	77.53346314	35.25425357	34.30170801	35.33230946	Acat2	acetyl-Coenzyme A acetyltransferase 2 [Source:MGI Symbol;Acc:MGI:87871]
ENSMUSG00000023845	18.4454944	24.29344861	16.73354903	24.3466384	30.52023741	39.97056556	11.80333706	11.47367526	13.70803014	24.07465586	22.44963668	23.54063518	Lnpep	leucyl/cystinyl aminopeptidase [Source:MGI Symbol;Acc:MGI:2387123]
ENSMUSG00000023852	14.68411138	19.51574535	11.61302275	19.03330164	22.74570056	31.70686405	27.79484275	23.31476391	35.54769711	52.46606311	50.97793235	50.14345639	Chd1	chromodomain helicase DNA binding protein 1 [Source:MGI Symbol;Acc:MGI:88393]
ENSMUSG00000023861	3.680903089	4.969567939	2.728900455	4.500979816	5.000920171	5.365658246	5.49593045	3.960808857	5.020911327	3.122180163	4.014870998	3.318350113	Mpc1	mitochondrial pyruvate carrier 1 [Source:MGI Symbol;Acc:MGI:1915240]
ENSMUSG00000023868	6.173466204	6.500185543	4.535435922	6.307736638	7.897567403	10.47114176	0.446535999	0.509641487	0.35912557	2.262494204	2.807300768	2.076983185	Pde10a	phosphodiesterase 10A [Source:MGI Symbol;Acc:MGI:1345143]
ENSMUSG00000023873	0.805411287	1.152314317	1.074445127	0.979353118	1.230856087	0.929495764	0.239684285	0.531916421	0.477046048	0.851407084	0.406317362	0.800293519	1700010I14R	RIKEN cDNA 1700010I14 gene [Source:MGI Symbol;Acc:MGI:1914181]
ENSMUSG00000023882	2.991401169	3.170170521	2.840648879	3.964776653	3.985620006	4.292818824	1.838494275	1.610548402	1.815830548	2.423675464	2.129164386	2.612632261	Zfp54	zinc finger protein 54 [Source:MGI Symbol;Acc:MGI:99201]
ENSMUSG00000023883	23.64003816	29.51442767	24.66555565	29.46089089	35.92588157	37.59710707	27.61993012	24.55392208	24.38872647	25.59571134	26.47989042	26.0009224	Phf10	PHD finger protein 10 [Source:MGI Symbol;Acc:MGI:1919307]
ENSMUSG00000023885	268.3790498	342.7028778	246.1939804	89.95474683	100.5479602	119.2301979	7.48303499	6.774041992	8.732293523	143.3039819	147.8192675	146.3656109	Thbs2	thrombospondin 2 [Source:MGI Symbol;Acc:MGI:98738]
ENSMUSG00000023886	51.92914917	54.01880447	54.27724634	7.72646597	7.122416962	7.942231098	0.808490615	0.512637976	0.385319558	2.221794812	1.723000624	1.926135895	Smoc2	SPARC related modular calcium binding 2 [Source:MGI Symbol;Acc:MGI:1929881]
ENSMUSG00000023892	4.229295812	5.024251584	3.542663477	5.912264219	7.165887291	7.945217124	3.745970618	4.239400548	4.87000172	5.951130775	5.913819766	4.992536472	Zfp51	zinc finger protein 51 [Source:MGI Symbol;Acc:MGI:99198]
ENSMUSG00000023902	0.555772446	0.101052717	0.329418594	0.30026394	0.030733051	1.002604302	73.90485182	75.42833553	78.21972286	80.37731658	79.69315666	81.21712467	Zscan10	zinc finger and SCAN domain containing 10 [Source:MGI Symbol;Acc:MGI:3040700]
ENSMUSG00000023903	0.14833756	0.207422252	0.19525745	0.118651006	0.100190806	0.080704311	0.690540347	0.472337638	0.808675308	1.099385397	1.005447871	0.985952965	Mmp25	matrix metallopeptidase 25 [Source:MGI Symbol;Acc:MGI:2443938]
ENSMUSG00000023904	269.7799552	192.8107997	316.3483739	263.5038297	217.0606104	163.1222396	49.29476576	58.41743692	38.22140501	35.43992608	34.32650885	34.60113231	Hcfc1r1	host cell factor C1 regulator 1 (XPO1-dependent) [Source:MGI Symbol;Acc:MGI:2663619]
ENSMUSG00000023905	300.8649965	262.4455562	336.839939	364.9181354	315.3455885	272.3385264	37.27037008	33.29454166	27.18362098	55.08964099	62.16918801	59.37273502	Tnfrsf12a	tumor necrosis factor receptor superfamily, member 12a [Source:MGI Symbol;Acc:MGI:1351484]
ENSMUSG00000023906	0.312479941	0.035983671	0.063982934	0.116640458	0.065662046	0.595025792	27.73011253	29.93405572	24.77986717	22.83563437	21.53694473	20.63109222	Cldn6	claudin 6 [Source:MGI Symbol;Acc:MGI:1859284]
ENSMUSG00000023908	9.962561812	7.718650871	7.696628941	11.95858786	10.93818687	11.43676762	17.97058514	19.05246223	21.14337308	11.53545583	11.31148482	10.79710085	Pkmyt1	protein kinase, membrane associated tyrosine/threonine 1 [Source:MGI Symbol;Acc:MGI:2137630]
ENSMUSG00000023909	8.094752213	8.164596637	8.19918466	11.03617936	9.801670089	11.06314506	2.870222586	3.042672251	3.419807468	2.387845812	1.696463081	1.042994725	Paqr4	progestin and adipoQ receptor family member IV [Source:MGI Symbol;Acc:MGI:1923748]
ENSMUSG00000023911	6.457469941	2.944993499	9.36028496	3.162160959	2.75414718	1.947935848	10.33148378	11.97070611	8.866751229	8.692675912	8.728035035	8.442500444	Flywch2	FLYWCH family member 2 [Source:MGI Symbol;Acc:MGI:1924167]
ENSMUSG00000023912	1.77564777	2.805586777	1.881310109	2.755253038	2.95025494	3.192478978	2.672818541	3.211256281	4.15096772	4.284648848	3.368573701	3.659587782	Slc25a27	solute carrier family 25, member 27 [Source:MGI Symbol;Acc:MGI:1921261]
ENSMUSG00000023913	127.6772584	111.8017253	124.1472506	89.14072079	89.71796269	72.16243453	18.36928477	21.04166846	19.22359273	54.44297789	47.21315404	53.940727	Pla2g7	phospholipase A2, group VII (platelet-activating factor acetylhydrolase, plasma) [Source:MGI Symbol;Acc:MGI:1351327]
ENSMUSG00000023914	0.233006252	2.1763626	0.079516697	0.700632285	0.136005771	0.039439255	0.39293172	0.795066955	0.072290887	0.370521939	0.107752333	0.045887972	Mep1a	meprin 1 alpha [Source:MGI Symbol;Acc:MGI:96963]
ENSMUSG00000023915	14.50563097	18.26001824	12.71731627	6.897343525	7.132753973	7.675792196	11.19161469	9.99925296	12.91028267	11.40222386	13.89038871	12.5234057	Tnfrsf21	tumor necrosis factor receptor superfamily, member 21 [Source:MGI Symbol;Acc:MGI:2151075]
ENSMUSG00000023918	0.131184414	0.194227195	0.03837304	0.069953794	0.177210307	0.085646395	0	0	0.03488602	0.016763052	0.031199369	0.066433632	Adgrf4	adhesion G protein-coupled receptor F4 [Source:MGI Symbol;Acc:MGI:1925499]
ENSMUSG00000023919	12.52251396	12.50483764	10.89256952	17.66050119	18.10311216	17.73765377	15.10255751	15.60771375	16.59220865	13.98031164	13.63705981	14.46324613	Cenpq	centromere protein Q [Source:MGI Symbol;Acc:MGI:1933744]
ENSMUSG00000023921	12.10933053	18.59268879	11.07504977	17.64718984	19.91116835	25.24152997	24.90118589	22.85241272	26.48654017	30.14770349	32.59134073	33.08905895	Mmut	methylmalonyl-Coenzyme A mutase [Source:MGI Symbol;Acc:MGI:97239]
ENSMUSG00000023923	9.696377324	11.7354116	9.354107574	13.17801677	14.97371706	16.10943241	2.428525257	2.024276494	2.616641479	5.029280542	5.113601097	5.421193757	Tbc1d5	TBC1 domain family, member 5 [Source:MGI Symbol;Acc:MGI:1919488]
ENSMUSG00000023926	0.119689813	0	0	0.027923142	0.03143833	0.068374174	0	0	0	0.053529856	0.049814848	0.053036029	Rhag	Rhesus blood group-associated A glycoprotein [Source:MGI Symbol;Acc:MGI:1202713]
ENSMUSG00000023927	1.157475744	1.363890327	0.606286834	0.565188146	1.032282527	1.19942459	8.290975699	8.066511686	9.796195336	4.201532769	3.641064914	4.460965391	Satb1	special AT-rich sequence binding protein 1 [Source:MGI Symbol;Acc:MGI:105084]
ENSMUSG00000023930	0.039367326	0.1360005	0.24182388	0.183684711	0.248170092	0.059970761	0.175731263	0	0.036641493	0.070426288	0	0	Crisp2	cysteine-rich secretory protein 2 [Source:MGI Symbol;Acc:MGI:98815]
ENSMUSG00000023931	0.231555648	0.219592662	0.139449985	0.076264915	0.085865752	0.197121365	0.753947753	0.526245033	0.950834557	0.938136566	0.986409676	0.869126033	Efhb	EF hand domain family, member B [Source:MGI Symbol;Acc:MGI:3045296]
ENSMUSG00000023932	80.50590203	81.47593196	79.46988257	93.5006501	88.03216374	90.91695532	211.3501487	210.1518094	223.4816873	182.5640885	179.8757695	179.167575	Cdc5l	cell division cycle 5-like (S. pombe) [Source:MGI Symbol;Acc:MGI:1918952]
ENSMUSG00000023935	0.967571816	0.720958561	0.932322756	0.770139456	0.687692673	0.715306006	2.693104849	2.875553218	2.410365601	3.08005551	3.316377359	2.421136806	Spats1	spermatogenesis associated, serine-rich 1 [Source:MGI Symbol;Acc:MGI:1918270]
ENSMUSG00000023938	18.99988754	15.51206412	16.7030478	18.15506269	18.88660576	17.67821314	40.40001976	40.77745029	43.12008065	23.54869228	23.33495007	25.40849281	Aars2	alanyl-tRNA synthetase 2, mitochondrial [Source:MGI Symbol;Acc:MGI:2681839]
ENSMUSG00000023939	48.61759746	37.7352183	69.52730592	46.51230262	40.54269119	26.12590969	35.20222393	36.10303115	29.64492355	20.68214563	19.43798402	17.57370519	Mrpl14	mitochondrial ribosomal protein L14 [Source:MGI Symbol;Acc:MGI:1333864]
ENSMUSG00000023940	9.634950431	10.46959865	7.054214151	17.26199346	17.30231985	20.33949188	37.13861307	33.61919018	46.7816667	38.64140677	36.69742325	39.57714095	Sgo1	shugoshin 1 [Source:MGI Symbol;Acc:MGI:1919665]
ENSMUSG00000023942	42.02692947	43.64970759	54.22725517	52.44755968	42.05142593	41.31228385	203.1825943	192.7130984	180.0956921	106.5051961	99.75496832	100.2994395	Slc29a1	solute carrier family 29 (nucleoside transporters), member 1 [Source:MGI Symbol;Acc:MGI:1927073]
ENSMUSG00000023943	0	0	0.084933644	0	0	0.084251981	0	0	0	0	0	0	Sult1c1	sulfotransferase family, cytosolic, 1C, member 1 [Source:MGI Symbol;Acc:MGI:102928]
ENSMUSG00000023944	3573.757675	3321.223606	3453.376399	3714.811991	3647.043816	3315.905028	8525.394175	8540.410337	8521.104774	5566.993819	5594.872402	5437.682955	Hsp90ab1	heat shock protein 90 alpha (cytosolic), class B member 1 [Source:MGI Symbol;Acc:MGI:96247]
ENSMUSG00000023945	0	0	0.02761031	0	0.042502336	0.010270768	0	0	0	0.205044008	0.202038157	0.298753562	Slc5a7	solute carrier family 5 (choline transporter), member 7 [Source:MGI Symbol;Acc:MGI:1927126]
ENSMUSG00000023947	15.36376941	15.74755672	16.71239657	21.64487205	19.6905796	19.18017906	3.089151405	2.624138373	3.235983771	1.717816121	2.494364933	1.834018117	Nfkbie	nuclear factor of kappa light polypeptide gene enhancer in B cells inhibitor, epsilon [Source:MGI Symbol;Acc:MGI:1194908]
ENSMUSG00000023949	0	0	0	0	0	0	0	0	0	0.012915425	0.048076343	0.012796277	Tcte1	t-complex-associated testis expressed 1 [Source:MGI Symbol;Acc:MGI:98640]
ENSMUSG00000023951	140.5349591	172.0231614	113.8304613	168.776981	197.5113814	221.1603261	57.59212816	56.84561636	65.44924134	71.62870351	66.34837216	73.96107338	Vegfa	vascular endothelial growth factor A [Source:MGI Symbol;Acc:MGI:103178]
ENSMUSG00000023952	68.38529489	77.32942553	60.12479994	92.96277963	94.99360111	90.12073105	29.24225281	27.8065463	34.15844538	34.29729955	36.19317503	34.52721456	Gtpbp2	GTP binding protein 2 [Source:MGI Symbol;Acc:MGI:1860138]
ENSMUSG00000023953	6.380354434	8.261239314	5.5921663	9.700731716	11.1998908	12.49324331	7.877476405	6.598142067	8.965795585	9.284438904	8.186714395	7.847241507	Polh	polymerase (DNA directed), eta (RAD 30 related) [Source:MGI Symbol;Acc:MGI:1891457]
ENSMUSG00000023959	0.011167529	0.1543198	0.022866468	0.156320313	0.023466555	0.017012209	0.029910346	0.110630282	0.04157711	0.229749086	0.167325138	0.227629589	Clic5	chloride intracellular channel 5 [Source:MGI Symbol;Acc:MGI:1917912]
ENSMUSG00000023960	11.4517825	15.17550996	11.46187074	10.64885337	13.23654211	14.39385671	2.143524773	1.723741177	1.832970571	5.043250766	5.142360178	4.709832115	Enpp5	ectonucleotide pyrophosphatase/phosphodiesterase 5 [Source:MGI Symbol;Acc:MGI:1933830]
ENSMUSG00000023961	2.197768643	3.026008616	1.927224803	2.568110508	2.700652373	3.588183916	2.081551053	1.590304778	2.356878782	2.384664139	2.147580808	2.48968998	Enpp4	ectonucleotide pyrophosphatase/phosphodiesterase 4 [Source:MGI Symbol;Acc:MGI:2682634]
ENSMUSG00000023963	3.573373191	4.553198733	3.138858122	4.3803794	5.487201293	5.669019786	3.001455429	3.351418001	2.932345738	2.288473676	2.728066683	2.398531597	Cyp39a1	cytochrome P450, family 39, subfamily a, polypeptide 1 [Source:MGI Symbol;Acc:MGI:1927096]
ENSMUSG00000023964	0	0	0	0.065868566	0	0.021505263	0.063016493	0.069924291	0.013139485	0.138900141	0.305524528	0.187661933	Calcr	calcitonin receptor [Source:MGI Symbol;Acc:MGI:101950]
ENSMUSG00000023965	10.86577975	11.51150363	9.283041229	10.96493439	11.40049547	9.817365987	2.542257845	1.900420935	2.106381707	4.906511909	5.239668304	5.193300499	Fbxl17	F-box and leucine-rich repeat protein 17 [Source:MGI Symbol;Acc:MGI:1354704]
ENSMUSG00000023966	2.531087519	2.040274173	3.303918765	1.358132339	1.130207959	1.73509521	1.694772974	2.319347595	2.002457576	2.03759822	2.106875028	2.243112041	Rsph9	radial spoke head 9 homolog (Chlamydomonas) [Source:MGI Symbol;Acc:MGI:1922814]
ENSMUSG00000023967	62.99154801	45.7698765	73.88073309	61.13230638	50.21571829	38.86991998	44.27542742	51.26682414	40.8024271	34.58688723	33.08476272	32.19580668	Mrps18a	mitochondrial ribosomal protein S18A [Source:MGI Symbol;Acc:MGI:1915815]
ENSMUSG00000023968	0.229485268	0.330330104	0.234945334	0.910145498	0.361666547	0.480685636	0.819516887	0.454675709	0.42719095	0.256586442	0.859605011	0.610126475	Crip3	cysteine-rich protein 3 [Source:MGI Symbol;Acc:MGI:2152434]
ENSMUSG00000023971	53.67237692	41.55204994	54.94938577	47.51494877	42.11511762	32.91776634	52.46660332	63.37790513	51.18562611	39.43328484	39.93267433	40.00610001	Rrp36	ribosomal RNA processing 36 homolog (S. cerevisiae) [Source:MGI Symbol;Acc:MGI:2385053]
ENSMUSG00000023972	50.2751342	61.33631563	45.11390068	34.7230245	37.21002216	38.99058423	42.11801551	37.94872747	47.60023907	73.85499189	75.81763321	72.55739251	Ptk7	PTK7 protein tyrosine kinase 7 [Source:MGI Symbol;Acc:MGI:1918711]
ENSMUSG00000023973	81.93980327	74.51020032	92.23698165	82.15265962	72.35027308	59.49130388	71.24405863	75.29500114	62.96658344	41.30415903	40.05038984	40.24345007	Cnpy3	canopy FGF signaling regulator 3 [Source:MGI Symbol;Acc:MGI:1919279]
ENSMUSG00000023977	29.56278633	36.06833696	26.21143249	33.33803573	41.8088785	50.57731859	18.84185666	17.22323643	23.30473248	30.38207642	34.46466271	32.51707598	Ubr2	ubiquitin protein ligase E3 component n-recognin 2 [Source:MGI Symbol;Acc:MGI:1861099]

ENSMUSG00000023978	0.021248636	0	0	0.019828878	0	0.048554105	0.720871336	0.673593643	0.731762275	0.513172885	0.176873459	0.320128089	Prph2	peripherin 2 [Source:MGI Symbol;Acc:MGI:102791]
ENSMUSG00000023979	0.053385221	0.092213739	0	0	0	0	0.071491664	0	0.024844387	0.071627729	0	0.094622592	Guca1b	guanylate cyclase activator 1B [Source:MGI Symbol;Acc:MGI:1194489]
ENSMUSG00000023980	14.28623412	14.13306364	13.75701613	15.79883175	15.46868615	14.7058176	37.48371612	37.5318907	38.5140093	29.35412542	28.79079168	28.33098556	Taf8	TATA-box binding protein associated factor 8 [Source:MGI Symbol;Acc:MGI:1926879]
ENSMUSG00000023982	0	0	0	0	0	0	0	0	0	0	0	0	Guca1a	guanylate cyclase activator 1a (retina) [Source:MGI Symbol;Acc:MGI:102770]
ENSMUSG00000023984	0	0.039247141	0.069785743	0	0	0.051919241	0.182565643	0.101289143	0.0634442	0.106699313	0.014184901	0.075510702	Gm20517	predicted gene 20517 [Source:MGI Symbol;Acc:MGI:5141982]
ENSMUSG00000023987	0.595708564	0.474915485	0.234570022	0.384857615	0.288871044	0.174515552	8.468450259	9.215172613	5.928468649	5.574401945	5.416396505	4.142232458	Pgc	progastricsin (pepsinogen C) [Source:MGI Symbol;Acc:MGI:98909]
ENSMUSG00000023988	14.43586787	13.58947535	13.16766794	12.83577704	12.34020522	11.42073423	23.22434094	24.03372264	23.52653841	23.91766462	24.03280747	23.56839101	Bysl	bystin-like [Source:MGI Symbol;Acc:MGI:1858419]
ENSMUSG00000023990	29.41077631	22.69558198	34.876554	26.83665202	24.50117152	20.91042166	57.7193864	56.28853094	51.50912107	25.60675619	24.19178243	24.86928567	Tfeb	transcription factor EB [Source:MGI Symbol;Acc:MGI:103270]
ENSMUSG00000023991	54.74395198	55.12941603	53.88573548	54.54281223	55.82028096	52.47452053	81.77095814	78.80874945	84.00700402	41.49303282	43.36387477	42.14086764	Foxp4	forkhead box P4 [Source:MGI Symbol;Acc:MGI:1921373]
ENSMUSG00000023992	4.893797972	2.977224115	5.033867646	3.683607677	2.498099136	2.33848967	0.515220682	0.548830657	0.193370367	1.98221286	2.017581505	2.37307776	Trem2	triggering receptor expressed on myeloid cells 2 [Source:MGI Symbol;Acc:MGI:1913150]
ENSMUSG00000023993	0.312650229	0.090008203	0.040011126	0.145880028	0.246366857	0.238140023	0	0	0	0.034957281	0	0	Treml1	triggering receptor expressed on myeloid cells-like 1 [Source:MGI Symbol;Acc:MGI:1918576]
ENSMUSG00000023994	3.64869067	8.163215172	2.348030295	5.228950778	9.885041141	13.79645327	6.65454165	4.23418475	8.708446636	15.17605458	14.20960233	13.90430565	Nfya	nuclear transcription factor-Y alpha [Source:MGI Symbol;Acc:MGI:97316]
ENSMUSG00000023995	0	0	0	0	0	0	0.528644781	0.703913081	0.404165749	0.035310061	0.131438075	0	Tspo2	translocator protein 2 [Source:MGI Symbol;Acc:MGI:1917276]
ENSMUSG00000023999	1.221628992	0.781538101	0.972762623	1.52000547	1.226471728	1.199301702	0.290838012	0.510972307	0.050535207	0.485652573	0.632727138	0.769875678	Kif6	kinesin family member 6 [Source:MGI Symbol;Acc:MGI:1098238]
ENSMUSG00000024002	63.23333529	74.59942623	59.04293152	79.70046156	83.7086943	98.24926311	71.89772555	65.82294385	88.12236672	98.51961894	105.4775955	104.9120741	Brd4	bromodomain containing 4 [Source:MGI Symbol;Acc:MGI:1888520]
ENSMUSG00000024006	97.16319666	101.9037208	88.17099436	107.8537567	110.9906865	118.604852	103.7056098	96.60786464	121.6889691	120.4019778	130.562648	123.4930521	Stk38	serine/threonine kinase 38 [Source:MGI Symbol;Acc:MGI:2442572]
ENSMUSG00000024007	54.79661749	42.05189155	56.18318	55.51293787	45.88814268	42.19963184	241.3203803	255.0339722	212.2153349	116.8746422	126.0659762	124.9712769	Ppil1	peptidylprolyl isomerase (cyclophilin)-like 1 [Source:MGI Symbol;Acc:MGI:1916066]
ENSMUSG00000024008	1.19251314	0.733648865	1.271059611	0.746970544	1.081292352	0.945643724	15.32802264	12.75215186	15.47837367	6.984528446	6.771755501	5.226263703	Cpne5	copine V [Source:MGI Symbol;Acc:MGI:2385908]
ENSMUSG00000024011	0.846277077	0.757968989	1.097455534	1.21092219	0.968185199	1.088647539	0.100738254	0.149041426	0	0.100929982	0.172196517	0.049999438	Pi16	peptidase inhibitor 16 [Source:MGI Symbol;Acc:MGI:1921366]
ENSMUSG00000024012	358.9350994	283.7177038	355.8092449	303.0830255	272.7464133	234.7417152	113.6772125	128.238501	98.68924867	98.0717234	98.65495032	89.06484436	Mtch1	mitochondrial carrier 1 [Source:MGI Symbol;Acc:MGI:1929261]
ENSMUSG00000024013	0.719425354	0.670654272	0.175367238	0.415601283	0.575902145	0.52187931	0.734041798	0.814506644	1.020360548	2.604679253	1.996163442	2.125241503	Fgd2	FYVE, RhoGEF and PH domain containing 2 [Source:MGI Symbol;Acc:MGI:1347084]
ENSMUSG00000024014	29.92420665	31.28839884	28.53037021	30.6181878	27.81514113	27.72020096	77.24436347	78.41578459	72.16258065	39.43755382	39.32400207	39.25013705	Pim1	proviral integration site 1 [Source:MGI Symbol;Acc:MGI:97584]
ENSMUSG00000024018	0.470850456	0.875875276	0.741620374	0.54078758	0.68497452	1.00694592	0.468868168	0.322923089	0.556238216	0.777534674	0.798945455	0.83455852	Ccdc167	coiled-coil domain containing 167 [Source:MGI Symbol;Acc:MGI:1915847]
ENSMUSG00000024019	0.910334721	1.048297228	1.708655685	0.906143364	1.083979424	1.040078229	0.514726536	1.262542739	0.762571997	0.434278887	0.580950731	1.156357476	Cmtr1	cap methyltransferase 1 [Source:MGI Symbol;Acc:MGI:1921407]
ENSMUSG00000024026	65.02785685	53.63006397	64.89069208	45.60569027	46.33852631	37.22421854	128.7274228	136.4862927	120.9916279	84.95652577	81.60629276	80.73334086	Glo1	glyoxalase 1 [Source:MGI Symbol;Acc:MGI:95742]
ENSMUSG00000024027	0.073570937	0.060514797	0.010760198	0.068655189	0.121468369	0.104069924	1.379331595	1.322296692	1.535837027	1.946020772	1.977189511	2.132983677	Glp1r	glucagon-like peptide 1 receptor [Source:MGI Symbol;Acc:MGI:99571]
ENSMUSG00000024028	0	0	0	0	0	0	0	0	0.068577314	0.263616208	0.061330266	0	Tff2	trefoil factor 2 (spasmolytic protein 1) [Source:MGI Symbol;Acc:MGI:1306805]
ENSMUSG00000024029	0	0	0	0	0.183400886	0	0	0.086462166	0	0.078068897	0	0	Tff3	trefoil factor 3, intestinal [Source:MGI Symbol;Acc:MGI:104638]
ENSMUSG00000024030	25.08720367	27.60579921	23.49155038	7.688096061	7.71457337	8.372438995	8.837952714	7.403778524	9.64108574	31.56834215	32.051465	31.49014882	Abcg1	ATP binding cassette subfamily G member 1 [Source:MGI Symbol;Acc:MGI:107704]
ENSMUSG00000024032	0	0.101535893	0	0	0	0	0	0	0	0	0	0	Tff1	trefoil factor 1 [Source:MGI Symbol;Acc:MGI:88135]
ENSMUSG00000024033	1.62871917	1.046820753	1.202130067	0.565541941	0.397960549	0.663558516	7.168969246	7.729687171	6.839379749	5.217558822	4.193348978	5.303696251	Rsph1	radial spoke head 1 homolog (Chlamydomonas) [Source:MGI Symbol;Acc:MGI:1194909]
ENSMUSG00000024034	0.016091431	0	0	0	0.016906626	0	0	0	0.119817955	0.273474705	0.281284362	0.213909338	Tmprss3	transmembrane protease, serine 3 [Source:MGI Symbol;Acc:MGI:2155445]
ENSMUSG00000024036	0.61601271	0.567496242	0.788336618	0.718566175	0.291249009	0.516126309	12.97910603	13.12033966	13.27326926	10.9650756	12.94740309	14.68539923	Slc37a1	solute carrier family 37 (glycerol-3-phosphate transporter), member 1 [Source:MGI Symbol;Acc:MGI:2446181]
ENSMUSG00000024037	24.44455378	24.23275535	25.36812714	29.30042066	26.32100398	25.28017259	40.23122332	35.55663513	38.69251125	20.91442068	24.77102186	24.27373326	Wdr4	WD repeat domain 4 [Source:MGI Symbol;Acc:MGI:1889002]
ENSMUSG00000024038	122.1057272	96.40463362	165.0842851	114.0580817	102.4582994	77.20773894	94.03673034	96.88049809	63.48805587	61.79358288	50.34169134	49.76607653	Ndufv3	NADH:ubiquinone oxidoreductase core subunit V3 [Source:MGI Symbol;Acc:MGI:1890894]
ENSMUSG00000024039	0.17330231	0.028509503	0.101386076	0.115516334	0.026011691	0.320574425	24.26899296	23.83908168	25.11713185	19.19969324	21.18512543	18.93480825	Cbs	cystathionine beta-synthase [Source:MGI Symbol;Acc:MGI:88285]
ENSMUSG00000024041	0	0	0.096079063	0	0	0	0	0.278904129	0.131022306	0.58760254	0.117176256	0.083168822	Cryaa	crystallin, alpha A [Source:MGI Symbol;Acc:MGI:88515]
ENSMUSG00000024042	16.56711831	18.35524108	14.8625482	16.20193807	19.4723238	20.63313404	52.08508897	46.14864503	58.91528075	32.63150868	37.5755067	36.61277917	Sik1	salt inducible kinase 1 [Source:MGI Symbol;Acc:MGI:104754]
ENSMUSG00000024043	13.75728188	16.50230967	11.85075575	14.08046322	17.75694154	21.70380997	3.252138484	3.169003718	4.474765531	11.47857699	11.68242737	12.70004346	Arhgap28	Rho GTPase activating protein 28 [Source:MGI Symbol;Acc:MGI:2147003]
ENSMUSG00000024044	84.49290843	101.9539724	66.79384108	92.82192208	106.5699569	119.0688027	5.496324828	5.291143508	7.557612507	40.3037173	37.34040847	38.89969164	Epb41l3	erythrocyte membrane protein band 4.1 like 3 [Source:MGI Symbol;Acc:MGI:103008]
ENSMUSG00000024045	44.45004043	55.87758493	42.94442734	53.7359659	61.79361857	69.66903499	85.12640739	75.79329724	91.49295809	111.7441548	114.4621154	107.6107562	Akap8	A kinase (PRKA) anchor protein 8 [Source:MGI Symbol;Acc:MGI:1928488]
ENSMUSG00000024048	157.3016102	136.6143456	237.7984192	155.737829	151.8560303	97.38763894	55.16811144	52.34832194	36.58132636	43.85270931	37.97269586	40.02545645	Myl12a	myosin, light chain 12A, regulatory, non-sarcomeric [Source:MGI Symbol;Acc:MGI:1914518]
ENSMUSG00000024049	0.724141498	0.433620803	0.845162659	0.797393198	0.578228239	0.573628096	0.129299408	0.243904211	0.188720337	0.544091076	0.590719252	0.397886249	Myom1	myomesin 1 [Source:MGI Symbol;Acc:MGI:1341430]
ENSMUSG00000024050	22.98627113	27.27086123	23.63254174	28.54519796	28.55262211	29.33644479	19.06301029	17.43493319	20.80749151	19.09929158	20.76301579	20.9587619	Wiz	widely-interspaced zinc finger motifs [Source:MGI Symbol;Acc:MGI:1332638]
ENSMUSG00000024052	25.64627145	30.38363539	25.76016777	28.49949097	32.86334301	38.68178401	6.368127633	6.471628165	8.486810543	15.65122238	18.23509379	17.51167726	Lpin2	lipin 2 [Source:MGI Symbol;Acc:MGI:1891341]
ENSMUSG00000024053	62.76563251	72.97450631	62.24606179	65.98154969	71.0302928	70.88557949	2.633004058	2.52209175	2.064631127	19.81893523	21.29708765	21.66896166	Emilin2	elastin microfibril interfacer 2 [Source:MGI Symbol;Acc:MGI:2389136]
ENSMUSG00000024054	25.13017286	34.68711753	21.55655476	34.16131282	39.78421669	59.22722123	39.17667567	34.34159768	48.56729034	59.5733421	57.07260178	58.21081484	Smchd1	SMC hinge domain containing 1 [Source:MGI Symbol;Acc:MGI:1921605]
ENSMUSG00000024055	31.21674607	28.82292085	36.8061829	34.11733411	30.04038583	26.31204053	25.44251773	27.39569732	23.90733664	13.66783991	14.94320622	15.78486251	Cyp4f13	cytochrome P450, family 4, subfamily f, polypeptide 13 [Source:MGI Symbol;Acc:MGI:2158641]
ENSMUSG00000024056	15.03062883	13.97326859	13.1207098	21.09250002	21.8782007	22.89711605	21.58194837	20.57217551	23.27537579	23.92586735	22.26539598	23.2689568	Ndc80	NDC80 kinetochore complex component [Source:MGI Symbol;Acc:MGI:1914302]
ENSMUSG00000024059	2.602722072	3.492113476	3.031342411	2.116859264	2.960367128	3.255576967	8.143437434	8.63398136	9.601128445	7.859917661	7.135548444	6.538883212	Clip4	CAP-GLY domain containing linker protein family, member 4 [Source:MGI Symbol;Acc:MGI:1919100]
ENSMUSG00000024063	81.81223933	88.50829508	79.98212426	105.4647718	99.52201144	105.0577679	7.421307686	7.533987174	8.983426824	65.08828136	67.4063922	62.98758713	Lbh	limb-bud and heart [Source:MGI Symbol;Acc:MGI:1925139]
ENSMUSG00000024064	0.080389515	0.162002153	0	0	0.021115515	0.091846858	0.035884964	0.039818634	0.018705816	0.143813046	0.167290404	0.053432375	Galnt14	polypeptide N-acetylgalactosaminyltransferase 14 [Source:MGI Symbol;Acc:MGI:1918935]
ENSMUSG00000024065	1.197231158	1.502753813	1.213459313	0.826754969	0.566047392	0.720408356	0.908531607	0.62859486	1.214623683	3.694587757	3.767050343	3.596843264	Ehd3	EH-domain containing 3 [Source:MGI Symbol;Acc:MGI:1928900]
ENSMUSG00000024066	126.4906542	156.8294388	115.8821781	184.3580639	203.3050897	242.0633901	0.989609824	1.023219997	1.617910688	8.224887427	8.450937247	9.265313493	Xdh	xanthine dehydrogenase [Source:MGI Symbol;Acc:MGI:98973]
ENSMUSG00000024067	64.08795911	50.19406217	67.54678364	66.33485119	56.30824692	47.16261506	353.9163629	379.681941	298.382459	216.2210143	199.8756368	207.9638412	Dpy30	dpy-30, histone methyltransferase complex regulatory subunit [Source:MGI Symbol;Acc:MGI:1913560]
ENSMUSG00000024068	17.92403759	18.23654276	14.48141725	18.17066626	19.493842	20.98579071	21.78609039	21.35749291	24.11994069	31.85599559	30.933437	28.24482401	Spast	spastin [Source:MGI Symbol;Acc:MGI:1858896]
ENSMUSG00000024069	21.40595074	21.40771858	18.21959677	21.7610291	22.60415917	21.72103717	24.1381927	22.52876179	26.17306127	21.47189399	23.0766446	24.28093512	Slc30a6	solute carrier family 30 (zinc transporter), member 6 [Source:MGI Symbol;Acc:MGI:2386741]
ENSMUSG00000024070	17.86134276	20.79335153	14.32135902	22.2656849	25.50509045	32.24360633	11.94328938	10.55358864	14.90756322	19.87779978	19.35250257	20.22494775	Prkd3	protein kinase D3 [Source:MGI Symbol;Acc:MGI:1922542]
ENSMUSG00000024072	71.68637711	68.06462988	59.27650317	68.02380079	69.40986728	67.13439271	43.69550197	43.9462999	40.74700702	44.82190082	44.34635405	45.84808095	Yipf4	Yip1 domain family, member 4 [Source:MGI Symbol;Acc:MGI:1915114]
ENSMUSG00000024073	16.86840128	21.88036392	14.40848588	20.57632879	25.81698146	33.68632994	18.72946913	19.17354136	26.97532475	42.19716094	41.73851833	42.86936206	Birc6	baculoviral IAP repeat-containing 6 [Source:MGI Symbol;Acc:MGI:1276108]
ENSMUSG00000024074	25.39348426	32.92696544	24.25348606	24.59910761	29.19552715	36.96502702	3.370805815	3.489435366	4.328479726	25.98812925	22.99638875	26.17684147	Crim1	cysteine rich transmembrane BMP regulator 1 (chordin like) [Source:MGI Symbol;Acc:MGI:1354756]
ENSMUSG00000024076	0	0	0	0.075287771	0.028255199	0.020483763	0.024009284	0	0	0.072164957	0	0	Vit	vitrin [Source:MGI Symbol;Acc:MGI:1921449]
ENSMUSG00000024077	6.685452368	8.190299896	5.863682868	7.686689489	10.7550283	14.07886898	5.931431969	4.97232783	7.647333144	10.38578597	10.95656597	10.86215605	Strn	striatin, calmodulin binding protein [Source:MGI Symbol;Acc:MGI:1333757]
ENSMUSG00000024078	23.38139096	21.88856632	21.76780463	22.41562406	21.73816715	22.54975618	60.01006914	63.72214293	64.53581023	50.40449075	51.16296443	51.84763194	Ttc27	tetratricopeptide repeat domain 27 [Source:MGI Symbol;Acc:MGI:1921446]
ENSMUSG00000024079	45.71257999	60.35162868	45.52538011	56.67966807	65.12323672	80.91283937	25.71695375	24.86279348	28.81993605	59.40264906	63.38780792	62.38592253	Eif2ak2	eukaryotic translation initiation factor 2-alpha kinase 2 [Source:MGI Symbol;Acc:MGI:1353449]
ENSMUSG00000024081	32.73020046	36.10539349	27.37607255	36.75185027	38.94128844	44.79245593	86.27411836	83.38302672	94.92692846	95.51308716	90.96383008	93.54760564	Cebpz	CCAAT/enhancer binding protein zeta [Source:MGI Symbol;Acc:MGI:109386]
ENSMUSG00000024082	24.94111217	20.73514132	25.036293	26.6049905	25.86375988	23.59976604	47.97330975	46.402313	47.25875373	27.38776118	27.61376451	27.38668555	Ndufaf7	NADH:ubiquinone oxidoreductase complex assembly factor 7 [Source:MGI Symbol;Acc:MGI:1920944]

ENSMUSG00000024083	21.54900037	28.26826739	17.51719988	27.03154739	32.29165596	43.4314393	7.686517074	6.945268515	9.797392249	18.00487141	15.73859472	16.8795087	Pja2	praja ring finger ubiquitin ligase 2 [Source:MGI Symbol;Acc:MGI:2159342]
ENSMUSG00000024084	7.166519474	8.015860207	5.472702755	2.713443212	2.931597436	2.997760249	2.359956905	1.949441499	2.077635164	4.755168541	5.427540175	5.414091587	Qpct	glutaminyl-peptide cyclotransferase (glutaminyl cyclase) [Source:MGI Symbol;Acc:MGI:1917786]
ENSMUSG00000024085	43.18348959	60.83024108	39.92631898	51.30561451	72.81210063	103.6466596	8.206054922	7.078716063	10.63264578	29.10167198	27.0561916	28.06394967	Man2a1	mannosidase 2, alpha 1 [Source:MGI Symbol;Acc:MGI:104669]
ENSMUSG00000024087	51.37246439	66.88662407	41.55847632	17.02381716	20.22871456	23.26057412	0.636199029	0.936972864	1.10946159	4.485047572	4.885590061	4.351812971	Cyp1b1	cytochrome P450, family 1, subfamily b, polypeptide 1 [Source:MGI Symbol;Acc:MGI:88590]
ENSMUSG00000024088	0.068299187	0	0.20977262	0.127471358	0	0	0	0	0	0	0	0	4930583I09R	RIKEN cDNA 4930583I09 gene [Source:MGI Symbol;Acc:MGI:1925307]
ENSMUSG00000024091	81.70266686	85.15040955	76.3923566	90.55772853	84.9683481	77.59332301	110.5089684	106.7410832	110.6049244	79.7554225	76.57628207	79.08981934	Vapa	vesicle-associated membrane protein, associated protein A [Source:MGI Symbol;Acc:MGI:1353561]
ENSMUSG00000024095	23.30709757	27.87160254	17.46648412	24.39217552	30.57242428	33.58686837	36.92856507	33.61860012	41.09540912	39.53162154	38.94374556	41.38630727	Hnrnpll	heterogeneous nuclear ribonucleoprotein L-like [Source:MGI Symbol;Acc:MGI:1919942]
ENSMUSG00000024096	70.41705573	77.06744952	66.34651943	82.44847449	77.02449138	81.07057742	63.06716912	61.28237814	68.72202232	73.74071238	75.5372713	75.11629573	Ralbp1	ralA binding protein 1 [Source:MGI Symbol;Acc:MGI:108466]
ENSMUSG00000024097	68.88163072	63.48624354	62.29531252	72.35315485	80.64990844	75.00170851	255.6392443	253.1531494	259.3967486	212.248121	214.8798185	215.9495374	Srsf7	serine and arginine-rich splicing factor 7 [Source:MGI Symbol;Acc:MGI:1926232]
ENSMUSG00000024098	52.641782	62.75088	48.05221557	58.791024	65.48994743	79.27240714	46.53842536	43.72077688	55.52812466	47.63862982	50.30195594	49.34042506	Twsg1	twisted gastrulation BMP signaling modulator 1 [Source:MGI Symbol;Acc:MGI:2137520]
ENSMUSG00000024099	105.3814172	81.78529704	113.2508006	101.1210355	92.50390428	71.6351364	170.9924564	179.31482	146.9169294	104.3577178	104.1432969	103.8351392	Ndufv2	NADH:ubiquinone oxidoreductase core subunit V2 [Source:MGI Symbol;Acc:MGI:1920150]
ENSMUSG00000024101	60.57408481	40.07256943	61.20268935	52.66732869	41.43364322	33.53768714	39.01158967	39.06679634	38.454962	32.77082185	35.22572317	32.87574696	Washc1	WASH complex subunit 1 [Source:MGI Symbol;Acc:MGI:1916017]
ENSMUSG00000024104	45.9434873	58.04541578	36.7102085	50.9621778	60.57589419	71.48194669	47.94947613	42.03808122	59.30539824	61.6315097	65.0630292	62.75469027	Washc2	WASH complex subunit 2` [Source:MGI Symbol;Acc:MGI:106463]
ENSMUSG00000024105	0	0	0.017316136	0	0	0.038648609	0.015100178	0.016755443	0	0.030257835	0.042236881	0.074946747	Themis3	thymocyte selection associated family member 3 [Source:MGI Symbol;Acc:MGI:1921806]
ENSMUSG00000024107	0.055023642	0.047521914	0	0	0.014452787	0	0.012280964	0	0	0	0	0	Lhcgr	luteinizing hormone/choriogonadotropin receptor [Source:MGI Symbol;Acc:MGI:96783]
ENSMUSG00000024109	0.027345718	0.173195002	0.027996346	0.076555725	0.057462114	0.031243108	0.085447721	0.02708983	0.089082955	0.183450745	0.284531899	0.169641139	Nrxn1	neurexin I [Source:MGI Symbol;Acc:MGI:1096391]
ENSMUSG00000024112	2.179705379	2.255861244	1.708984378	2.149929721	2.377096572	2.647981148	1.798194516	1.311985053	1.913216921	1.665878634	2.583775884	1.858352521	Cacna1h	calcium channel, voltage-dependent, T type, alpha 1H subunit [Source:MGI Symbol;Acc:MGI:1928842]
ENSMUSG00000024114	0.979510292	0.322273272	0.429778051	0.293805935	0	0.266455452	7.401886516	8.767755665	7.944709733	4.474617229	4.076717534	3.379257407	Prss41	protease, serine 41 [Source:MGI Symbol;Acc:MGI:1918253]
ENSMUSG00000024116	0	0	0	0.059574893	0	0	0	0	0	0.028551904	0.132851912	0	Prss21	protease, serine 21 [Source:MGI Symbol;Acc:MGI:1916698]
ENSMUSG00000024118	15.9182209	12.28721142	14.15965185	14.1781822	14.40938988	13.14052714	17.83546796	16.48735619	17.67668076	11.10030297	10.57128805	10.56106727	Tedc2	tubulin epsilon and delta complex 2 [Source:MGI Symbol;Acc:MGI:1919266]
ENSMUSG00000024120	46.94985571	48.27335177	41.45580752	50.75367778	53.29162837	57.99978986	83.52709726	79.72025801	90.05604156	103.5305326	112.2049219	109.8847149	Lrpprc	leucine-rich PPR-motif containing [Source:MGI Symbol;Acc:MGI:1919666]
ENSMUSG00000024121	146.9893681	124.8219888	135.4422962	112.5400713	93.83277066	89.70239799	75.88724362	88.43085578	72.19851948	65.11604909	68.43518799	69.34275995	Atp6v0c	ATPase, H+ transporting, lysosomal V0 subunit C [Source:MGI Symbol;Acc:MGI:88116]
ENSMUSG00000024122	21.13114116	30.99747111	13.58312119	30.59491798	40.03923228	62.69144903	22.42966644	19.7748778	29.50199915	49.02075567	51.02310284	50.76643965	Pdpk1	3-phosphoinositide dependent protein kinase 1 [Source:MGI Symbol;Acc:MGI:1338068]
ENSMUSG00000024124	0	0	0	0	0	0	0	0	0	0.037258438	0	0	Prss30	protease, serine 30 [Source:MGI Symbol;Acc:MGI:1353645]
ENSMUSG00000024125	0	0	0	0	0	0	0	0	0	0	0	0	Sbpl	spermine binding protein-like [Source:MGI Symbol;Acc:MGI:3694550]
ENSMUSG00000024127	11.55923626	15.05718171	10.01187504	13.69893932	15.37729317	18.17803188	18.44226906	17.41607669	22.86769735	26.83149852	26.65229192	29.48581088	Prepl	prolyl endopeptidase-like [Source:MGI Symbol;Acc:MGI:2441932]
ENSMUSG00000024128	0	0	0	0	0	0	0	0	0	0	0	0	Sbp	spermine binding protein [Source:MGI Symbol;Acc:MGI:106021]
ENSMUSG00000024130	8.214944968	11.77557527	6.995020079	10.8646824	14.05707403	17.20505544	3.631356359	3.101296407	3.902824909	4.639813259	5.734884735	5.619692539	Abca3	ATP-binding cassette, sub-family A (ABC1), member 3 [Source:MGI Symbol;Acc:MGI:1351617]
ENSMUSG00000024131	0.170522409	0.545459221	0.426750112	0.495067098	0.398135785	0.606124556	0.11840774	0.262774931	0.017635029	0.186423439	0.11039988	0.016791239	Slc3a1	solute carrier family 3, member 1 [Source:MGI Symbol;Acc:MGI:1195264]
ENSMUSG00000024132	43.07989582	34.80897872	47.52590434	42.40821315	40.61727049	30.15053272	46.63021713	48.2787379	43.11145224	26.02651012	31.96559855	25.74084964	Eci1	enoyl-Coenzyme A delta isomerase 1 [Source:MGI Symbol;Acc:MGI:94871]
ENSMUSG00000024134	0.153204178	0.403251399	0.201663404	0	0.045990151	0	0.097698001	0.260178114	0.142596165	1.859794916	1.384579113	1.241356002	Six2	sine oculis-related homeobox 2 [Source:MGI Symbol;Acc:MGI:102778]
ENSMUSG00000024135	14.46437868	19.49087585	15.02629721	20.72894936	20.88011314	24.81197043	36.380169	34.61844976	39.37035234	34.57393589	32.04800934	29.51529911	Srbd1	S1 RNA binding domain 1 [Source:MGI Symbol;Acc:MGI:1925836]
ENSMUSG00000024136	1.322304028	0.90538982	2.19529486	1.734203362	2.665939039	1.578814948	0.287154225	0.814281144	0.399160979	0.63933499	0.565216474	0.570093259	Dnase1l2	deoxyribonuclease 1-like 2 [Source:MGI Symbol;Acc:MGI:1913955]
ENSMUSG00000024137	17.91528075	15.84485435	18.42296046	21.486935	19.80475314	17.93181166	15.0357585	16.70365936	14.63903829	9.461921492	9.516960906	9.145553389	E4f1	E4F transcription factor 1 [Source:MGI Symbol;Acc:MGI:109530]
ENSMUSG00000024140	16.4667845	15.33594789	13.46877964	3.501756788	4.746564204	6.187161215	28.06228738	25.24896314	31.48871387	14.93975439	14.27040353	14.37156668	Epas1	endothelial PAS domain protein 1 [Source:MGI Symbol;Acc:MGI:109169]
ENSMUSG00000024142	3.252388047	4.815375462	3.102271141	5.429202637	7.025330222	9.047476119	3.643098837	2.72165037	4.155354501	4.083699718	5.481831646	4.439887494	Mlst8	MTOR associated protein, LST8 homolog (S. cerevisiae) [Source:MGI Symbol;Acc:MGI:1929514]
ENSMUSG00000024143	66.91979627	77.42285504	53.31730562	61.25488486	67.82515512	77.45918282	8.510301379	7.865341499	10.0852883	29.91829579	29.69004036	28.7868142	Rhoq	ras homolog family member Q [Source:MGI Symbol;Acc:MGI:1931553]
ENSMUSG00000024145	7.055278369	9.227845015	8.60534847	9.875804771	8.60239256	10.08431404	15.00823246	14.36680899	13.49834763	9.738857888	10.51305049	10.42093549	Pigf	phosphatidylinositol glycan anchor biosynthesis, class F [Source:MGI Symbol;Acc:MGI:99462]
ENSMUSG00000024146	76.99574059	67.5105023	79.53737694	72.31322533	66.12171763	56.7982452	47.43284053	51.45513721	41.57000538	44.77713924	45.50268185	45.46109236	Cript	cysteine-rich PDZ-binding protein [Source:MGI Symbol;Acc:MGI:1929655]
ENSMUSG00000024150	201.439655	206.0877927	178.4875655	171.7314564	180.6040223	172.346143	46.32379462	41.32891521	45.65440802	84.70475358	84.96518433	78.62494446	Mcfd2	multiple coagulation factor deficiency 2 [Source:MGI Symbol;Acc:MGI:2183439]
ENSMUSG00000024151	30.46667038	30.69844495	28.71834276	35.98960992	33.80172812	38.81361889	218.0563117	214.3680346	223.9207844	170.1198619	170.4731867	171.4217631	Msh2	mutS homolog 2 [Source:MGI Symbol;Acc:MGI:101816]
ENSMUSG00000024154	0.249958021	0.16447972	0.036557884	0.099967165	0.150069107	0.299181931	0.095638641	0.318367327	0.232650673	0.255521768	0.535023866	0.632911281	Gtf2a1l	general transcription factor IIA, 1-like [Source:MGI Symbol;Acc:MGI:1919078]
ENSMUSG00000024155	0.575046612	0.547031259	0.17917824	0.53662405	0.577909938	0.742700061	0.558031	0.520129461	0.418875495	0.514365617	0.16649332	0.576092691	Meiob	meiosis specific with OB domains [Source:MGI Symbol;Acc:MGI:1922428]
ENSMUSG00000024158	32.56792813	30.79769392	42.39615057	32.60583163	30.47879107	26.77777125	59.45155987	59.29159267	57.06251288	30.43346714	34.20107659	32.4464189	Hagh	hydroxyacyl glutathione hydrolase [Source:MGI Symbol;Acc:MGI:95745]
ENSMUSG00000024160	1.39952352	1.706424645	1.798051028	1.971822572	1.26860077	1.546733117	0.88197369	0.652436444	1.200452884	1.129111258	1.416228495	0.972778181	Spsb3	splA/ryanodine receptor domain and SOCS box containing 3 [Source:MGI Symbol;Acc:MGI:1891471]
ENSMUSG00000024163	51.85839302	56.96042742	50.93851382	65.10571945	70.72797055	75.35178498	27.98637648	25.63857678	33.32491315	32.79338145	34.71091856	34.09761178	Mapk8ip3	mitogen-activated protein kinase 8 interacting protein 3 [Source:MGI Symbol;Acc:MGI:1353598]
ENSMUSG00000024164	388.4695512	440.6548105	387.4853897	370.4140265	403.0510062	430.1713087	4.654949324	4.423678256	3.916023814	14.17613159	14.93265054	16.08124295	C3	complement component 3 [Source:MGI Symbol;Acc:MGI:88227]
ENSMUSG00000024165	48.85692104	56.40796079	38.70467875	60.1856348	63.8471291	71.12763028	149.6217336	143.2745925	169.475203	177.7682533	176.3537052	169.1283234	Jpt2	Jupiter microtubule associated homolog 2 [Source:MGI Symbol;Acc:MGI:1196260]
ENSMUSG00000024168	2.061781708	2.133381923	1.988469627	0.920183867	0.941839966	0.796590779	0.160061892	0.325614114	0.36155484	0.721649369	1.144150189	1.297578007	Tmem204	transmembrane protein 204 [Source:MGI Symbol;Acc:MGI:3039635]
ENSMUSG00000024169	10.43321496	11.66101913	10.24511524	14.55643115	17.12327747	16.86745357	8.827502124	9.305404946	11.26580728	10.76565145	13.18299418	11.04403856	Ift140	intraflagellar transport 140 [Source:MGI Symbol;Acc:MGI:2146906]
ENSMUSG00000024170	14.29533646	13.18038992	15.21893412	16.73061576	15.26903467	14.68681278	18.78342202	20.67777049	20.22349539	17.18364503	19.94438596	16.35169257	Telo2	telomere maintenance 2 [Source:MGI Symbol;Acc:MGI:1918968]
ENSMUSG00000024171	0	0	0	0	0.13141953	0	0	0	0	0	0	0	Prss28	protease, serine 28 [Source:MGI Symbol;Acc:MGI:2149951]
ENSMUSG00000024172	0	0	0	0	0	0	0	0.047031093	0	0.070775959	0.039518436	0.042073819	St6gal2	beta galactoside alpha 2,6 sialyltransferase 2 [Source:MGI Symbol;Acc:MGI:2445190]
ENSMUSG00000024173	0	0	0	0	0	0	0	0	0	0	0	0	Tpsab1	tryptase alpha/beta 1 [Source:MGI Symbol;Acc:MGI:96943]
ENSMUSG00000024174	0.950905808	1.073958902	0.449321719	1.177471301	1.364126903	2.451435458	1.812175436	1.032585346	2.161598913	3.500393032	3.440124887	2.933300361	Pot1b	protection of telomeres 1B [Source:MGI Symbol;Acc:MGI:1920086]
ENSMUSG00000024175	0.065692348	0.037824058	0	0	0	0	0	0	0	0	0	0.029109088	Tekt4	tektin 4 [Source:MGI Symbol;Acc:MGI:1919090]
ENSMUSG00000024176	0.438171974	0.470939049	0.777568571	0.354375568	0.521752486	0.244748287	0.156475984	0.405133651	0.353454834	0.705482072	0.461996357	0.310655028	Sox8	SRY (sex determining region Y)-box 8 [Source:MGI Symbol;Acc:MGI:98370]
ENSMUSG00000024177	19.62702954	15.15823998	22.75066869	17.08283925	14.72244578	12.07463918	79.31487962	85.2516954	60.16155437	40.47144709	41.82562762	42.14689466	Nme4	NME/NM23 nucleoside diphosphate kinase 4 [Source:MGI Symbol;Acc:MGI:1931148]
ENSMUSG00000024180	4.081427225	5.099566124	3.77252373	4.348418107	4.079859759	3.788394859	20.40235913	19.77420277	20.77818883	18.81535376	16.97312763	18.43676152	Tmem8	transmembrane protein 8 [Source:MGI Symbol;Acc:MGI:1926283]
ENSMUSG00000024181	149.9545026	106.2214539	169.4212187	135.7411779	107.2002833	83.68051391	155.5265292	170.0625858	122.8394865	72.99579144	73.5597877	76.66015172	Mrpl28	mitochondrial ribosomal protein L28 [Source:MGI Symbol;Acc:MGI:1915861]
ENSMUSG00000024182	46.30125433	45.08015415	42.14067035	52.27195798	54.22353152	51.53399149	38.17136645	36.14156468	37.47163421	34.30326793	34.69923529	33.44763312	Axin1	axin 1 [Source:MGI Symbol;Acc:MGI:1096327]
ENSMUSG00000024184	0.074897281	0.258744207	0.038339643	0	0.118037385	0.057047904	0.20059976	0.148392855	0	0.234478472	0.124688862	0.19912744	Pdia2	protein disulfide isomerase associated 2 [Source:MGI Symbol;Acc:MGI:1916441]
ENSMUSG00000024186	5.056956301	4.393049839	6.146121505	6.181703804	7.332758165	5.969151525	4.488333465	5.302596862	6.165642571	4.708493481	4.824816216	4.796097291	Rgs11	regulator of G-protein signaling 11 [Source:MGI Symbol;Acc:MGI:1354739]
ENSMUSG00000024187	58.91087409	55.64597703	61.9385478	51.4141689	48.10456615	43.16103524	24.95254204	24.93892405	26.45588838	18.92556344	21.18316691	20.98361365	Fam234a	family with sequence similarity 234, member A [Source:MGI Symbol;Acc:MGI:2146854]
ENSMUSG00000024188	47.47274479	47.97282264	45.66083523	52.32310257	55.27819416	56.16205414	79.65521384	80.76008286	83.19025632	71.47478366	71.85795675	69.88048731	Luc7l	Luc7-like [Source:MGI Symbol;Acc:MGI:1914228]
ENSMUSG00000024190	103.7525494	111.7436163	86.95377182	111.9617738	115.8519933	117.2006003	45.92800704	40.68441592	47.58686859	25.18603024	24.85350502	23.31905022	Dusp1	dual specificity phosphatase 1 [Source:MGI Symbol;Acc:MGI:105120]
ENSMUSG00000024191	25.68632595	20.4006327	26.4568507	25.77144344	23.52010195	22.52917482	26.54572043	26.03199382	23.4730917	12.44871271	14.04684986	13.40997951	Bnip1	BCL2/adenovirus E1B interacting protein 1 [Source:MGI Symbol;Acc:MGI:109328]

ENSMUSG00000024193	16.02543768	12.55484892	18.23182967	14.65698253	12.38151937	9.762170124	3.953731165	3.624963062	2.986657179	3.440921547	3.467660862	2.993424703	Phf1	PHD finger protein 1 [Source:MGI Symbol;Acc:MGI:98647]
ENSMUSG00000024194	124.681695	90.11884185	158.5035655	110.3150452	86.22354107	66.80589281	59.50616521	73.39098511	47.22213388	36.14914539	31.082022	32.44142638	Cuta	cutA divalent cation tolerance homolog [Source:MGI Symbol;Acc:MGI:1914925]
ENSMUSG00000024197	58.79605632	60.66518643	59.35747591	65.13489537	64.9762142	59.95589612	43.45406497	44.10478633	45.83572094	38.48796637	38.71082454	40.97837334	Plin3	perilipin 3 [Source:MGI Symbol;Acc:MGI:1914155]
ENSMUSG00000024201	17.06639932	18.05082536	17.75329755	18.74194662	17.72511935	18.81969036	23.63318208	21.42938791	23.28905578	19.90998625	21.65974983	20.54245275	Kdm4b	lysine (K)-specific demethylase 4B [Source:MGI Symbol;Acc:MGI:2442355]
ENSMUSG00000024205	3.043815859	3.186464027	2.549647921	2.840438947	4.651659766	0.421530812	10.49923664	7.538333195	2.44673273	1.732577264	3.224670461	2.329662987	Rpl36-ps2	ribosomal protein L36, pseudogene 2 [Source:MGI Symbol;Acc:MGI:3650081]
ENSMUSG00000024206	1.627053383	2.158754072	1.575234594	2.492057194	2.675708114	2.869242251	19.4837361	18.06298766	22.3208588	14.37959779	14.08826545	15.29704685	Rfx2	regulatory factor X, 2 (influences HLA class II expression) [Source:MGI Symbol;Acc:MGI:106583]
ENSMUSG00000024207	0	0.018991765	0.016884726	0	0.017327834	0	0.323927478	0.196056026	0.153503839	0.221279991	0.192194809	0.263086349	Acsbg2	acyl-CoA synthetase bubblegum family member 2 [Source:MGI Symbol;Acc:MGI:3587728]
ENSMUSG00000024208	213.869407	166.4355525	346.509613	233.2386434	216.589754	140.8571814	114.4111038	121.0674964	67.93347201	46.53476013	47.68518925	46.10546464	Uqcc2	ubiquinol-cytochrome c reductase complex assembly factor 2 [Source:MGI Symbol;Acc:MGI:1914517]
ENSMUSG00000024209	0.760724647	0.602259306	0.535441715	0.510237564	0.749309144	0.615644587	8.44702316	7.818663777	7.700057862	6.017731759	7.618506663	7.057747553	Acsbg3	acyl-CoA synthetase bubblegum family member 3 [Source:MGI Symbol;Acc:MGI:1925875]
ENSMUSG00000024210	0.293960634	0.211569238	0.188096713	0.102869713	0.077213182	0.097958217	0	0	0.034200813	0.082169014	0.030586572	0.016282197	Ip6k3	inositol hexaphosphate kinase 3 [Source:MGI Symbol;Acc:MGI:3045325]
ENSMUSG00000024211	0.366267661	0.403437951	0.114125001	0.029721301	0	0.084906789	0	0	0	0.056977004	0.159068285	0.254031193	Grm8	glutamate receptor, metabotropic 8 [Source:MGI Symbol;Acc:MGI:1351345]
ENSMUSG00000024212	48.2188192	53.23898067	50.74373124	54.02631009	56.38416473	57.59247111	37.35651483	36.91277802	42.79265259	42.09889993	41.95081543	43.0166423	Mllt1	myeloid/lymphoid or mixed-lineage leukemia; translocated to, 1 [Source:MGI Symbol;Acc:MGI:1927238]
ENSMUSG00000024213	103.433865	92.53477918	126.0383825	107.7623251	101.4289194	81.93505159	79.7026139	77.1910383	64.52363301	52.67423603	46.11040059	46.03651955	Nudt3	nudix (nucleotide diphosphate linked moiety X)-type motif 3 [Source:MGI Symbol;Acc:MGI:1928484]
ENSMUSG00000024215	0.640304878	0.110601597	0.819424297	0.507893693	0.235459991	0.585250369	0.228659846	0.412303586	0.148992379	0.114547519	0.213195687	0.141863485	Spdef	SAM pointed domain containing ets transcription factor [Source:MGI Symbol;Acc:MGI:1353422]
ENSMUSG00000024217	79.76497	57.48150581	84.69152462	68.72857981	55.66955569	46.31075056	125.88798	142.6183442	107.3251505	68.4252455	72.03804611	67.10921656	Snrpc	U1 small nuclear ribonucleoprotein C [Source:MGI Symbol;Acc:MGI:109489]
ENSMUSG00000024218	33.91982006	27.9356143	35.29203793	31.39584119	27.09921292	23.45339752	30.47450647	29.01474309	26.11901186	25.7317979	26.62651541	24.48877201	Taf11	TATA-box binding protein associated factor 11 [Source:MGI Symbol;Acc:MGI:1916026]
ENSMUSG00000024219	10.55045631	11.01996717	9.88342572	11.38836835	11.02606006	11.13100082	8.556092538	8.133750164	10.06771246	11.36717209	12.0944643	11.62240273	Anks1	ankyrin repeat and SAM domain containing 1 [Source:MGI Symbol;Acc:MGI:2446180]
ENSMUSG00000024220	22.18967823	21.42130372	24.32736205	23.4969142	22.47618915	18.82439888	10.37972125	9.98186346	9.996828867	8.161112787	9.530275153	7.575553891	Zfp523	zinc finger protein 523 [Source:MGI Symbol;Acc:MGI:2687278]
ENSMUSG00000024222	22.11492651	23.9983409	23.41923045	28.23327159	30.1388789	30.3463154	148.843879	139.997371	150.6578338	84.62966509	86.4298961	84.60941929	Fkbp5	FK506 binding protein 5 [Source:MGI Symbol;Acc:MGI:104670]
ENSMUSG00000024223	0	0	0	0.066534346	0	0	0	0	0.165903693	0.095661888	0.089022889	0.031593127	Armc12	armadillo repeat containing 12 [Source:MGI Symbol;Acc:MGI:1914895]
ENSMUSG00000024224	0	0	0	0	0	0	0	0	0.076722512	0.073731736	0	0	Clpsl2	colipase-like 2 [Source:MGI Symbol;Acc:MGI:2685595]
ENSMUSG00000024225	0.174912552	0	0.268611282	0.408063799	0	0	0.078078972	0	0.081400714	0.078227574	0.218395582	0.232517712	Clps	colipase, pancreatic [Source:MGI Symbol;Acc:MGI:88421]
ENSMUSG00000024227	0	0	0	0.020559896	0	0	0.019669664	0.043651662	0.10253239	0.0394142	0.055018242	0.05857589	Pdzph1	PDZ and pleckstrin homology domains 1 [Source:MGI Symbol;Acc:MGI:1916489]
ENSMUSG00000024228	2.918567382	3.755173598	2.119983242	3.362606518	4.676722588	5.4147302	2.314498597	2.19670909	2.443316936	2.8876913	2.062965166	2.586506229	Nudt12	nudix (nucleoside diphosphate linked moiety X)-type motif 12 [Source:MGI Symbol;Acc:MGI:1915243]
ENSMUSG00000024231	31.92844634	34.70167156	27.65418355	32.41423468	34.3663038	38.54443428	63.54747562	60.541665	68.52942112	55.30639601	53.54087124	54.8896887	Cul2	cullin 2 [Source:MGI Symbol;Acc:MGI:1918995]
ENSMUSG00000024232	1.009142698	0.85935305	1.224155948	0.735962444	0.739513288	0.768645734	3.626471246	4.250823598	3.449246988	2.389379071	2.477675068	2.690496626	Bambi	BMP and activin membrane-bound inhibitor [Source:MGI Symbol;Acc:MGI:1915260]
ENSMUSG00000024233	0	0	0	0	0	0	0	0	0	0	0	0	Lyzl1	lysozyme-like 1 [Source:MGI Symbol;Acc:MGI:1914578]
ENSMUSG00000024234	12.10410071	12.38737891	11.31951299	15.27577961	15.01702401	14.26382376	15.44945218	16.12369022	17.13407178	12.684295	11.80399635	11.60566664	Mtpap	mitochondrial poly(A) polymerase [Source:MGI Symbol;Acc:MGI:1914690]
ENSMUSG00000024235	5.349105551	6.72961658	4.077125018	5.54144382	6.833239794	6.281986431	1.13603621	0.793690483	0.877308905	3.519983781	2.903010718	3.550160897	Map3k8	mitogen-activated protein kinase kinase kinase 8 [Source:MGI Symbol;Acc:MGI:1346878]
ENSMUSG00000024236	12.9644549	19.47238871	10.44337483	16.39017911	20.70339266	26.14529619	4.803033973	3.876977077	5.184101987	10.09613239	10.48427294	10.82566895	Svil	supervillin [Source:MGI Symbol;Acc:MGI:2147319]
ENSMUSG00000024238	53.56253583	68.53017685	46.10768353	67.90933809	79.76559856	102.5320888	6.682768393	6.605042069	9.458612975	19.2218127	20.83815912	21.57057166	Zeb1	zinc finger E-box binding homeobox 1 [Source:MGI Symbol;Acc:MGI:1344313]
ENSMUSG00000024240	10.26837755	12.76440985	10.08518653	12.82360739	13.77986236	16.35086782	7.422014152	5.753643316	7.578776358	10.83163765	9.368948901	10.29437039	Epc1	enhancer of polycomb homolog 1 [Source:MGI Symbol;Acc:MGI:1278322]
ENSMUSG00000024241	5.671517624	8.146096207	5.667909246	7.278714781	8.447072492	11.65714987	7.205187783	6.124228297	8.599546188	8.220303325	8.586833478	8.520619018	Sos1	SOS Ras/Rac guanine nucleotide exchange factor 1 [Source:MGI Symbol;Acc:MGI:98354]
ENSMUSG00000024242	10.64667923	14.09159736	8.095844749	10.95219775	13.73887293	16.72349924	20.57469084	20.08754014	25.21917823	28.89890425	28.68291876	28.78890751	Map4k3	mitogen-activated protein kinase kinase kinase kinase 3 [Source:MGI Symbol;Acc:MGI:2154405]
ENSMUSG00000024245	0.050741141	0.029215516	0.129871021	0.0236754	0	0	0.588906961	0.427263804	0.802872141	0.385787399	0	0.33726036	Tmem178	transmembrane protein 178 [Source:MGI Symbol;Acc:MGI:1915277]
ENSMUSG00000024246	6.002009108	5.500093581	5.257709626	5.699585017	5.506710683	5.167220346	8.88673987	8.793151294	9.068103562	5.633307455	7.318182809	6.705098172	Thumpd2	THUMP domain containing 2 [Source:MGI Symbol;Acc:MGI:1919417]
ENSMUSG00000024247	21.84924079	17.23088692	22.82638224	12.57401296	13.3747793	9.922930124	7.277554534	7.6697027	5.092755427	5.826465497	5.01931992	4.585184906	Pkdcc	protein kinase domain containing, cytoplasmic [Source:MGI Symbol;Acc:MGI:2147077]
ENSMUSG00000024248	224.8645515	165.3614646	234.2150989	173.0723418	146.9148162	117.6675919	189.7070848	214.0969604	176.7791196	103.1061413	107.6829521	104.5872208	Cox7a2l	cytochrome c oxidase subunit 7A2 like [Source:MGI Symbol;Acc:MGI:106015]
ENSMUSG00000024251	5.871896549	7.426884892	5.190347152	7.635448805	7.416726351	11.48679321	14.60967605	12.07891285	17.63536463	26.62012588	27.89763344	27.55464925	Thada	thyroid adenoma associated [Source:MGI Symbol;Acc:MGI:3039623]
ENSMUSG00000024253	22.61037422	16.63200524	21.21197262	21.50076336	21.22668294	16.20693328	13.16313181	11.83814751	13.68312669	12.91903766	12.41603975	13.14270941	Dync2li1	dynein cytoplasmic 2 light intermediate chain 1 [Source:MGI Symbol;Acc:MGI:1913996]
ENSMUSG00000024254	0	0.071064203	0	0.014397088	0.016209508	0.011751173	0	0	0	0	0.012842193	0.027345217	Abcg8	ATP binding cassette subfamily G member 8 [Source:MGI Symbol;Acc:MGI:1914720]
ENSMUSG00000024256	0	0	0.048065739	0	0	0	0	0.069764072	0.043697929	0.062991762	0.117240182	0.145624841	Adcyap1	adenylate cyclase activating polypeptide 1 [Source:MGI Symbol;Acc:MGI:105094]
ENSMUSG00000024258	65.21739444	50.81558919	70.14591756	62.39878437	60.01522401	46.28711858	106.5889505	123.7684312	85.8661251	73.9809342	73.83850636	76.88585661	Polr2d	polymerase (RNA) II (DNA directed) polypeptide D [Source:MGI Symbol;Acc:MGI:1916491]
ENSMUSG00000024259	21.74348836	24.51077105	19.57245601	26.99371678	27.82274936	29.84266651	11.67206254	11.97432596	12.5824425	13.85666209	14.09776274	13.95046206	Slc25a46	solute carrier family 25, member 46 [Source:MGI Symbol;Acc:MGI:1914703]
ENSMUSG00000024260	18.83582721	21.61763664	17.65002019	20.8066681	25.41826493	26.80416598	37.86465969	33.04559842	42.16706479	38.77065434	39.34200314	39.54730916	Sap130	Sin3A associated protein [Source:MGI Symbol;Acc:MGI:1919782]
ENSMUSG00000024261	0.32061309	0.43285805	0.181098665	0.278557015	0.360086811	0.218942584	0.049351046	0.032856512	0.061740727	0.286780839	0.220864597	0.28413521	Syt4	synaptotagmin IV [Source:MGI Symbol;Acc:MGI:101759]
ENSMUSG00000024266	2.814947802	2.855657442	3.316498587	2.355837485	2.628936477	2.501443943	2.991811065	3.983724084	3.098298846	2.318070664	3.310178523	2.930254358	Adad2	adenosine deaminase domain containing 2 [Source:MGI Symbol;Acc:MGI:1923023]
ENSMUSG00000024268	30.87342579	31.42528236	32.80157516	38.84390631	34.70583396	35.09270449	11.87333171	12.40491303	13.22244942	13.63397103	9.974092268	12.95332487	Celf4	CUGBP, Elav-like family member 4 [Source:MGI Symbol;Acc:MGI:1932407]
ENSMUSG00000024269	11.80106206	11.23749563	11.82626337	11.66907505	12.0293231	11.20124756	8.823665057	9.004035867	9.600389962	9.317498188	8.340269264	8.859464407	Tpgs2	tubulin polyglutamylase complex subunit 2 [Source:MGI Symbol;Acc:MGI:1913898]
ENSMUSG00000024270	75.45654389	85.82515847	64.19520437	73.51722889	90.50252964	96.8068602	15.1567157	13.25477595	15.72544265	46.89730671	47.86168921	51.41542374	Slc39a6	solute carrier family 39 (metal ion transporter), member 6 [Source:MGI Symbol;Acc:MGI:2147279]
ENSMUSG00000024271	93.34595447	90.36694448	92.43750861	98.95747816	97.08671645	95.63566678	122.5916655	122.229036	129.448487	117.935121	117.7014945	113.2526432	Elp2	elongator acetyltransferase complex subunit 2 [Source:MGI Symbol;Acc:MGI:1889642]
ENSMUSG00000024273	14.71468041	14.55899348	16.75073217	15.06994724	13.78574646	13.0691465	22.43237775	21.52342186	18.24461347	19.00640315	16.71454189	19.06645234	2700062C07	RIKEN cDNA 2700062C07 gene [Source:MGI Symbol;Acc:MGI:1915296]
ENSMUSG00000024274	1.467200896	1.916997654	1.271015743	1.474488366	1.126502259	1.912713336	0.352660955	0.363367882	0.577758251	0.984282419	1.620566825	1.225253987	Zscan30	zinc finger and SCAN domain containing 30 [Source:MGI Symbol;Acc:MGI:2685600]
ENSMUSG00000024276	9.96395035	12.28369817	9.498711581	13.33114669	18.10855318	21.86676387	7.104221717	6.693736147	7.438383663	13.54518705	14.00412708	12.75149742	Zfp397	zinc finger protein 397 [Source:MGI Symbol;Acc:MGI:1916506]
ENSMUSG00000024277	27.39086615	29.88188564	23.92227826	25.31780478	28.21049554	31.18793495	25.8839966	22.88982236	29.44516492	30.55396493	31.41684983	29.43810557	Mapre2	microtubule-associated protein, RP/EB family, member 2 [Source:MGI Symbol;Acc:MGI:106271]
ENSMUSG00000024283	71.98063558	78.74657592	62.96519371	88.70001839	96.98639232	112.2189426	36.78343359	34.36655936	42.65354227	56.08465438	52.92911857	51.08847682	Wac	WW domain containing adaptor with coiled-coil [Source:MGI Symbol;Acc:MGI:2387357]
ENSMUSG00000024286	41.27222302	47.58899751	37.20864781	44.33438973	49.7649252	56.52528924	24.30327502	21.81889497	25.97106577	31.91567724	34.78756919	33.05063501	Ccny	cyclin Y [Source:MGI Symbol;Acc:MGI:1915224]
ENSMUSG00000024287	24.12203106	29.34750925	18.32476523	31.35953434	31.82017999	35.12147115	23.1759037	22.36976305	27.47173204	32.73597691	35.42219917	30.56935335	Thoc1	THO complex 1 [Source:MGI Symbol;Acc:MGI:1919668]
ENSMUSG00000024290	16.86597896	22.98016106	13.82762698	23.75967785	26.63732524	35.2611053	12.3351554	11.50857788	15.89909872	25.35691983	23.36127344	23.9511471	Rock1	Rho-associated coiled-coil containing protein kinase 1 [Source:MGI Symbol;Acc:MGI:107927]
ENSMUSG00000024292	0.102570889	0	0.026252831	0.167505569	0.053883574	0.019531598	0.343398576	0.330236007	0.381875104	0.298178524	0.192104815	0.204526902	Cyp4f14	cytochrome P450, family 4, subfamily f, polypeptide 14 [Source:MGI Symbol;Acc:MGI:1927669]
ENSMUSG00000024293	10.02951127	13.71954958	7.331323391	14.0879112	14.73945172	16.84154053	13.77700606	11.42912023	15.61039921	19.7777579	17.42913649	18.61182647	Esco1	establishment of sister chromatid cohesion N-acetyltransferase 1 [Source:MGI Symbol;Acc:MGI:1925055]
ENSMUSG00000024294	17.73921125	22.75313757	14.46088533	22.76434503	26.94415774	35.52703837	19.25864684	15.87954913	22.99355286	28.34767016	25.66398518	28.49290638	Mib1	mindbomb E3 ubiquitin protein ligase 1 [Source:MGI Symbol;Acc:MGI:2443157]
ENSMUSG00000024295	0.50489751	0.387610292	0.459475882	0.994675461	1.002009664	0.640952164	2.253804998	1.333794452	2.036397535	0.903237803	1.447618175	1.590942569	Cyp4f41-ps	cytochrome P450, family 4, subfamily f, polypeptide 41 pseudogene [Source:MGI Symbol;Acc:MGI:1925125]
ENSMUSG00000024298	8.062486469	11.15900083	7.201692038	11.35363173	13.96218673	20.85085542	5.76758263	5.287913803	6.834353416	15.05950894	14.72043628	15.86972736	Zfp871	zinc finger protein 871 [Source:MGI Symbol;Acc:MGI:1921793]
ENSMUSG00000024299	18.24066823	18.70818584	18.72142692	20.2614506	19.38870553	19.06599077	7.394791442	6.983640299	8.743923032	8.689073795	10.87433799	9.405037002	Adamts10	a disintegrin-like and metallopeptidase (reprolysin type) with thrombospondin type 1 motif, 10 [Source:MGI Symbol;Acc:MGI:244911
ENSMUSG00000024300	1.977509494	1.931556826	1.518419785	0.164766015	0.092754038	0.295867236	2.93195029	3.30582077	3.714037031	4.864297761	5.775967199	5.194962805	Myo1f	myosin IF [Source:MGI Symbol;Acc:MGI:107711]
ENSMUSG00000024301	11.55158326	11.69956218	8.477980227	14.76262635	16.27986882	14.85873388	32.82347369	33.34237467	35.4505158	20.97646038	24.94630896	23.08531103	Kifc5b	kinesin family member C5B [Source:MGI Symbol;Acc:MGI:2137414]

ENSMUSG00000024302	7.182576373	9.138704438	5.656514617	10.90549709	12.19041425	14.42311959	1.076213811	0.646851385	1.075249593	2.396137984	2.27165397	2.537247744	Dtna	dystrobrevin alpha [Source:MGI Symbol;Acc:MGI:106039]
ENSMUSG00000024304	18.06054235	22.82032538	15.78732032	17.20576022	20.2548664	23.55658383	1.087122091	0.896712048	1.364058254	16.64631052	15.4820754	14.64960576	Cdh2	cadherin 2 [Source:MGI Symbol;Acc:MGI:88355]
ENSMUSG00000024306	0.016454489	0.056844569	0.084229924	0.030710117	0	0.062665431	0	0	0	0	0	0.058329491	Ccdc178	coiled coil domain containing 178 [Source:MGI Symbol;Acc:MGI:1918200]
ENSMUSG00000024308	250.1939562	227.3847635	262.9456688	203.2976083	185.4986067	161.5355154	27.08334917	25.65336405	25.78507016	42.7058257	44.25592011	43.49588854	Tapbp	TAP binding protein [Source:MGI Symbol;Acc:MGI:1201689]
ENSMUSG00000024309	76.94825943	60.43005697	100.6312066	91.5347054	83.20330312	53.27719942	99.83007101	102.0865484	70.10183237	72.84136448	70.84159257	72.41034982	Pfdn6	prefoldin subunit 6 [Source:MGI Symbol;Acc:MGI:95908]
ENSMUSG00000024312	46.82193134	47.21973151	53.33614172	52.69511827	53.0656835	47.60626004	72.31410817	76.30420428	77.4754367	83.27748895	82.15928112	84.33423254	Wdr46	WD repeat domain 46 [Source:MGI Symbol;Acc:MGI:1931871]
ENSMUSG00000024313	0	0.17775611	0.039508745	0.216072526	0	0.117574962	5.443539859	4.31992896	4.849000409	6.523969188	7.709461987	6.326984186	Mep1b	meprin 1 beta [Source:MGI Symbol;Acc:MGI:96964]
ENSMUSG00000024317	9.76870311	10.34172082	8.29022454	13.36336143	14.43183134	13.69119965	28.06819461	27.34945454	31.38679952	28.52265774	25.8872207	27.16382475	Rnf138	ring finger protein 138 [Source:MGI Symbol;Acc:MGI:1929211]
ENSMUSG00000024319	76.10082435	69.58354716	79.25172634	77.18855107	71.05198472	62.84529978	41.82882429	40.37340098	40.0812165	35.37564883	35.47275818	36.60636727	Vps52	VPS52 GARP complex subunit [Source:MGI Symbol;Acc:MGI:1330304]
ENSMUSG00000024325	16.77381729	18.23614134	22.10612313	23.64971788	21.37269772	17.53767206	10.9291655	10.37263364	8.92136063	5.451685309	5.528633927	5.909215254	Ring1	ring finger protein 1 [Source:MGI Symbol;Acc:MGI:1101770]
ENSMUSG00000024327	0	0.110849028	0	0.149714682	0.26969914	0.048879971	0	0.031786613	0	0.20090661	0.106836318	0.028436171	Slc39a7	solute carrier family 39 (zinc transporter), member 7 [Source:MGI Symbol;Acc:MGI:95909]
ENSMUSG00000024330	5.105490855	4.16916917	5.20183445	4.867435277	4.642040675	3.935499911	0.482103132	0.291791313	0.274152777	1.042885569	1.297416767	1.120276436	Col11a2	collagen, type XI, alpha 2 [Source:MGI Symbol;Acc:MGI:88447]
ENSMUSG00000024331	0.325378396	0.413450978	0.229737941	0.31410804	0.247555329	0.341841154	0.450761	0.277873844	0.543039343	2.47888597	2.941933709	2.495791154	Dsc2	desmocollin 2 [Source:MGI Symbol;Acc:MGI:103221]
ENSMUSG00000024334	0.035269252	0	0.324975616	0.131650747	0.074111997	0.107455805	1.322478583	1.22287268	1.313086935	0.283927621	0.264222852	0.093769438	H2-Oa	histocompatibility 2, O region alpha locus [Source:MGI Symbol;Acc:MGI:95924]
ENSMUSG00000024335	184.1117553	195.0580425	161.3574183	198.0955798	212.977591	214.1734479	153.9758885	153.4314417	173.8508796	195.83009	201.9862959	195.6909139	Brd2	bromodomain containing 2 [Source:MGI Symbol;Acc:MGI:99495]
ENSMUSG00000024338	9.120751784	6.466561889	12.63343834	2.436578456	1.728663877	1.498396621	0.542853299	0.67322619	0.832276632	3.711225353	3.42389084	3.740384666	Psmb8	proteasome (prosome, macropain) subunit, beta type 8 (large multifunctional peptidase 7) [Source:MGI Symbol;Acc:MGI:1346527]
ENSMUSG00000024339	45.51323149	40.93069143	54.07258598	39.5596588	32.82378607	27.62711458	2.948745841	2.941783855	1.94605032	6.748946216	6.078779571	7.116351931	Tap2	transporter 2, ATP-binding cassette, sub-family B (MDR/TAP) [Source:MGI Symbol;Acc:MGI:98484]
ENSMUSG00000024340	0	0	0.017174367	0	0	0	0	0	0	0	0	0	Btnl2	butyrophilin-like 2 [Source:MGI Symbol;Acc:MGI:1859549]
ENSMUSG00000024346	119.7207296	94.72868451	162.9882797	113.3772312	107.2467667	80.039189	172.4488123	194.4285516	140.2867186	105.0152759	99.45029343	96.97027309	Pfdn1	prefoldin 1 [Source:MGI Symbol;Acc:MGI:1914449]
ENSMUSG00000024347	0.621100867	0.476819717	0.41214348	0.203933729	0.447128516	0.665818115	1.18088966	1.048269978	1.284656023	1.018526776	0.842524737	0.764492877	Psd2	pleckstrin and Sec7 domain containing 2 [Source:MGI Symbol;Acc:MGI:1921252]
ENSMUSG00000024349	51.03027679	55.33029245	48.72797149	63.08322702	65.47274877	54.59461859	4.953494341	2.991287558	4.145438491	13.67335647	12.47306979	12.27611932	Sting1	stimulator of interferon response cGAMP interactor 1 [Source:MGI Symbol;Acc:MGI:1919762]
ENSMUSG00000024350	25.69292614	23.59238868	24.15295405	23.15175553	25.31364773	24.40773346	11.44771178	11.04676519	12.64593075	12.34519676	12.44248837	11.68111176	Dnajc18	DnaJ heat shock protein family (Hsp40) member C18 [Source:MGI Symbol;Acc:MGI:1923844]
ENSMUSG00000024352	0.711214675	0.98280031	0.87376364	1.526495852	1.942836822	2.437737072	0.571460639	0.281823786	1.059151115	0.890630627	0.532813024	0.819384729	Spata24	spermatogenesis associated 24 [Source:MGI Symbol;Acc:MGI:1918492]
ENSMUSG00000024353	0.066814422	0.153880483	0.205212346	0.436450846	0.140398504	0.610696037	0	0.132378409	0	0.119527846	0	0.059212585	Mzb1	marginal zone B and B1 cell-specific protein 1 [Source:MGI Symbol;Acc:MGI:1917066]
ENSMUSG00000024354	0.546850347	1.524600481	0.235731105	0.67146284	0.876950657	1.424957419	0.976431073	1.083466363	0.991183014	2.085301188	1.509339903	2.21910549	Slc23a1	solute carrier family 23 (nucleobase transporters), member 1 [Source:MGI Symbol;Acc:MGI:1341903]
ENSMUSG00000024357	64.34444378	50.80537892	69.42797036	55.55798028	48.69790053	40.47667065	31.64357219	32.65689945	27.17620996	13.96740715	13.53448751	13.57496261	Sil1	endoplasmic reticulum chaperone SIL1 homolog (S. cerevisiae) [Source:MGI Symbol;Acc:MGI:1932040]
ENSMUSG00000024359	485.4256424	544.1836898	425.3663846	646.9559048	701.5733991	693.7784647	684.8257087	660.7932237	731.7060012	668.6406391	670.0338513	640.6394248	Hspa9	heat shock protein 9 [Source:MGI Symbol;Acc:MGI:96245]
ENSMUSG00000024360	65.23299732	69.66856942	56.98643744	72.06840171	77.69151285	80.1103454	125.2806888	115.2817143	138.0934481	136.2073461	139.0088764	136.5443805	Etf1	eukaryotic translation termination factor 1 [Source:MGI Symbol;Acc:MGI:2385071]
ENSMUSG00000024365	0.318434693	0.275020438	0.326011102	0.074289506	0.334566648	0	0	0	0.074096488	0	0	0.035275583	Cyp21a1	cytochrome P450, family 21, subfamily a, polypeptide 1 [Source:MGI Symbol;Acc:MGI:88591]
ENSMUSG00000024366	0.495570966	0.322555685	0.61765799	0.583100079	0.339571745	0.508760461	0	0.192104471	0.120327946	0.424003636	0.286965452	0.22914114	Gfra3	glial cell line derived neurotrophic factor family receptor alpha 3 [Source:MGI Symbol;Acc:MGI:1201403]
ENSMUSG00000024369	94.15857378	71.31813127	101.6980295	86.81719528	85.49857696	62.77116296	95.67548906	105.9849791	79.21856227	45.65413584	47.46859962	46.86837135	Nelfe	negative elongation factor complex member E, Rdbp [Source:MGI Symbol;Acc:MGI:102744]
ENSMUSG00000024370	17.72857566	24.25163082	12.99863365	22.91378757	27.09561906	30.68017416	49.58365572	41.31037758	55.66116511	70.18271291	67.23178521	66.72742174	Cdc23	CDC23 cell division cycle 23 [Source:MGI Symbol;Acc:MGI:1098815]
ENSMUSG00000024371	1.513564898	2.199434111	2.988469235	1.715098299	1.135887396	1.015610355	1.415622763	1.213801358	1.173970103	0.612455076	0.749934579	0.702616334	C2	complement component 2 (within H-2S) [Source:MGI Symbol;Acc:MGI:88226]
ENSMUSG00000024376	26.01606836	26.43595545	26.04939419	32.07636206	33.8670408	29.78078638	82.73626952	76.7758043	79.1491613	31.27981544	30.91017057	32.26969507	Epb41l4a	erythrocyte membrane protein band 4.1 like 4a [Source:MGI Symbol;Acc:MGI:103007]
ENSMUSG00000024378	14.39523001	19.75774005	11.63384061	23.42984771	28.78608997	34.53588083	39.75817099	37.44222426	46.71704262	23.48248053	23.90907293	22.46975438	Stard4	StAR-related lipid transfer (START) domain containing 4 [Source:MGI Symbol;Acc:MGI:2156764]
ENSMUSG00000024379	1.903915391	1.403172124	1.169528766	1.030487484	1.560287093	1.189145882	0.883881598	0.754439782	0.815159721	2.179849423	1.267853999	2.260977535	Tslp	thymic stromal lymphopoietin [Source:MGI Symbol;Acc:MGI:1855696]
ENSMUSG00000024381	132.8643662	98.96554656	149.9105669	113.3427103	97.96759504	76.97303661	13.78464052	16.44402314	12.3643124	26.8337438	26.63108198	25.74381981	Bin1	bridging integrator 1 [Source:MGI Symbol;Acc:MGI:108092]
ENSMUSG00000024382	35.61698216	35.67757457	38.51632938	35.28284734	35.49105244	34.82537855	34.27700953	34.84937088	38.30028028	39.88854739	39.62274434	40.14963764	Ercc3	excision repair cross-complementing rodent repair deficiency, complementation group 3 [Source:MGI Symbol;Acc:MGI:95414]
ENSMUSG00000024383	5.870166649	7.870473786	5.175607366	8.853162939	9.775479852	13.42626546	3.199382049	3.022934513	3.846266339	6.433254282	6.637368809	6.816026442	Map3k2	mitogen-activated protein kinase kinase kinase 2 [Source:MGI Symbol;Acc:MGI:1346873]
ENSMUSG00000024384	18.1069425	20.22606367	16.37681761	22.79209324	26.12071372	30.22467495	20.79570762	20.55121459	24.96406467	34.13211033	34.42385755	34.63226663	Iws1	IWS1, SUPT6 interacting protein [Source:MGI Symbol;Acc:MGI:1920723]
ENSMUSG00000024386	0	0.54993913	0.097785239	0.133696375	0	0.036375162	1.10853075	1.230046659	1.111241718	1.23879137	1.113066053	1.058071717	Proc	protein C [Source:MGI Symbol;Acc:MGI:97771]
ENSMUSG00000024387	291.0302005	207.0540321	302.3142606	242.640883	208.4791283	167.6945458	230.9998093	258.5254515	213.6151937	159.2958372	149.0341776	151.2361139	Csnk2b	casein kinase 2, beta polypeptide [Source:MGI Symbol;Acc:MGI:88548]
ENSMUSG00000024388	0.012519199	0.057666006	0.064085322	0.023365422	0.013153424	0.047678238	0.011176856	0.012402051	0.023304714	0.011198128	0.020841941	0.033284467	Myo7b	myosin VIIB [Source:MGI Symbol;Acc:MGI:107709]
ENSMUSG00000024391	0.976954808	1.222840958	1.82645065	1.189145444	1.517357181	0.420594539	2.351155925	1.220285867	0.869774268	0.531916613	0.282857852	1.091662622	Apom	apolipoprotein M [Source:MGI Symbol;Acc:MGI:1930124]
ENSMUSG00000024392	197.7474014	190.2384189	216.213771	212.2874148	194.2055027	191.4375811	202.0935565	194.8104836	207.4626764	160.7961986	167.4709625	164.7377649	Bag6	BCL2-associated athanogene 6 [Source:MGI Symbol;Acc:MGI:1919439]
ENSMUSG00000024393	142.8786781	169.3154782	139.1525206	168.0113833	183.1320014	208.5509714	124.2042537	110.0984497	142.651123	170.742489	185.0230635	183.526898	Prrc2a	proline-rich coiled-coil 2A [Source:MGI Symbol;Acc:MGI:1915467]
ENSMUSG00000024395	3.228891419	2.263273144	3.665032235	3.078651645	1.253738029	1.657413768	8.14670641	9.109275773	8.035963519	3.70438828	3.155157057	2.92373005	Lims2	LIM and senescent cell antigen like domains 2 [Source:MGI Symbol;Acc:MGI:2385067]
ENSMUSG00000024397	5.378560978	2.615113325	5.078245509	2.174980049	2.448783911	1.092467355	0.213415856	0.118405133	0.278119108	2.191675863	2.885248302	3.601440998	Aif1	allograft inflammatory factor 1 [Source:MGI Symbol;Acc:MGI:1343098]
ENSMUSG00000024399	0.407677318	0.085356616	0.720923991	0.380437774	0.46726944	0.112916522	2.051439236	1.982597567	1.621283481	1.193425314	1.172300834	1.54370932	Ltb	lymphotoxin B [Source:MGI Symbol;Acc:MGI:104796]
ENSMUSG00000024400	33.31612232	33.46374661	32.86091865	38.74521737	35.28850749	38.74231127	48.43311637	48.31823833	50.67370161	52.09027823	53.66089438	49.62078515	Wdr33	WD repeat domain 33 [Source:MGI Symbol;Acc:MGI:1921570]
ENSMUSG00000024401	2.774241639	2.396011394	2.385808521	0.388331507	0.087443556	0.169047173	0.222910178	0.192379712	0.025821503	3.201126797	2.794224328	1.942294952	Tnf	tumor necrosis factor [Source:MGI Symbol;Acc:MGI:104798]
ENSMUSG00000024402	2.675668802	2.722894568	3.280825575	2.467856555	2.647775695	2.511963982	0.083329401	0.061642585	0.028958172	0.361781318	0.258979577	0.165435595	Lta	lymphotoxin A [Source:MGI Symbol;Acc:MGI:104797]
ENSMUSG00000024403	3.294616688	2.468333804	2.682148075	2.444768946	3.378112349	2.08616921	2.019969264	1.101036656	1.884231299	0.603593569	1.453821514	1.336762341	Atp6v1g2	ATPase, H+ transporting, lysosomal V1 subunit G2 [Source:MGI Symbol;Acc:MGI:1913487]
ENSMUSG00000024404	127.3214845	133.5225234	115.2259809	149.6202066	149.2933558	155.5926236	114.9633407	111.307759	120.7924629	107.0675492	106.2182742	101.6133039	Riok3	RIO kinase 3 [Source:MGI Symbol;Acc:MGI:1914128]
ENSMUSG00000024406	7.675351883	2.812269806	2.73838312	1.627843696	1.344031086	7.839191423	660.5797541	571.7047817	456.0732431	301.3503745	256.4778691	252.6047281	Pou5f1	POU domain, class 5, transcription factor 1 [Source:MGI Symbol;Acc:MGI:101893]
ENSMUSG00000024409	1.122926135	0.646553585	1.358669638	0.619211402	0.643534781	0.233267051	5.923998848	6.826203368	5.795962617	4.154691506	4.971025422	3.890189565	Psors1c2	psoriasis susceptibility 1 candidate 2 (human) [Source:MGI Symbol;Acc:MGI:1930025]
ENSMUSG00000024410	39.69655663	41.05059787	32.97499827	41.69141287	39.78783721	43.74417575	24.24517851	25.23478577	27.47751945	32.43115901	30.09094701	29.46489222	Rmc1	regulator of MON1-CCZ1 [Source:MGI Symbol;Acc:MGI:1916528]
ENSMUSG00000024411	0	0.221822118	0.049303022	0.157288385	0.025298443	0	0.021496841	0	0.022411389	0.021537754	0.040086039	0	Aqp4	aquaporin 4 [Source:MGI Symbol;Acc:MGI:107387]
ENSMUSG00000024413	9.898425719	17.96080047	9.318745234	13.92841974	16.23956283	22.43997712	8.614071051	5.567962689	10.24479384	18.21056174	19.67588878	18.02872816	Npc1	NPC intracellular cholesterol transporter 1 [Source:MGI Symbol;Acc:MGI:1097712]
ENSMUSG00000024414	44.98091485	36.83785978	60.27393395	40.5739331	33.53147521	29.62817223	44.046351	40.02217463	32.71030177	32.44273605	29.94115769	29.68110906	Mrpl27	mitochondrial ribosomal protein L27 [Source:MGI Symbol;Acc:MGI:2137224]
ENSMUSG00000024420	7.039388594	8.894034394	6.064096366	3.74653789	4.161435162	4.717264895	0.739365392	1.854848603	1.055688931	2.061280207	1.978170529	2.217771444	Zfp521	zinc finger protein 521 [Source:MGI Symbol;Acc:MGI:95459]
ENSMUSG00000024421	0.427612301	0.812488952	0.394007704	0.74820145	0.892934487	1.131823321	0.429482841	0.481857409	0.611931135	0.655012595	0.760830523	0.658443944	Lama3	laminin, alpha 3 [Source:MGI Symbol;Acc:MGI:99909]
ENSMUSG00000024422	77.50622269	66.11874711	74.14700372	70.62910227	68.19131818	63.76940328	222.1500659	246.7400426	214.5010821	159.4501464	163.5056087	163.9745335	Dhx16	DEAH (Asp-Glu-Ala-His) box polypeptide 16 [Source:MGI Symbol;Acc:MGI:1916442]
ENSMUSG00000024423	120.4888629	151.267443	104.2166342	108.1936734	109.4366269	131.8582691	48.97284779	45.19421273	53.76577424	181.4548943	180.8260014	189.7777117	Impact	impact, RWD domain protein [Source:MGI Symbol;Acc:MGI:1098233]
ENSMUSG00000024424	56.41367752	56.71752851	55.9064723	63.02288403	61.10097452	58.2207185	5.61552495	5.476461971	7.13065318	18.65021335	18.47911633	17.51374682	Ttc39c	tetratricopeptide repeat domain 39C [Source:MGI Symbol;Acc:MGI:1919997]
ENSMUSG00000024425	116.4865514	125.8717534	101.9323159	134.0358959	133.5496352	132.6819223	70.39545839	67.85231092	74.63253795	83.42242835	81.74335291	81.80123103	Ndfip1	Nedd4 family interacting protein 1 [Source:MGI Symbol;Acc:MGI:1929601]
ENSMUSG00000024426	33.47127967	27.8776629	31.29347715	30.56693322	27.86814145	25.33412554	12.93024442	13.47176987	15.24375728	10.73294561	11.17963139	9.701130351	Atat1	alpha tubulin acetyltransferase 1 [Source:MGI Symbol;Acc:MGI:1913869]

ENSMUSG00000024427	4.479306304	4.585023528	4.243097916	4.500905086	4.14528429	5.066015808	46.29979573	43.72847215	51.4701083	41.11049026	42.70154934	40.41847311	Spry4	sprouty RTK signaling antagonist 4 [Source:MGI Symbol;Acc:MGI:1345144]
ENSMUSG00000024429	152.0874633	124.5143871	174.4341958	135.5270421	112.3275835	93.17833408	79.50927704	86.63581794	69.91141724	45.52364994	46.04719169	45.97502941	Gnl1	guanine nucleotide binding protein-like 1 [Source:MGI Symbol;Acc:MGI:95764]
ENSMUSG00000024430	2.371423964	2.665013789	2.515599945	2.906194611	3.542218835	2.58971345	0.892775493	0.820816456	0.771198801	1.150038836	1.165356506	1.291353346	Cabyr	calcium-binding tyrosine-(Y)-phosphorylation regulated (fibrousheathin 2) [Source:MGI Symbol;Acc:MGI:1918382]
ENSMUSG00000024431	31.10590947	38.34522727	26.12536242	36.01535045	42.23880253	58.38452374	7.39583827	6.17905833	8.708309278	19.99223923	20.68698357	21.44885919	Nr3c1	nuclear receptor subfamily 3, group C, member 1 [Source:MGI Symbol;Acc:MGI:95824]
ENSMUSG00000024436	18.62336248	23.33803211	24.48731871	29.13540895	31.07672237	30.91976204	44.8807206	38.4657579	45.60051906	44.58506996	35.41159259	40.29035333	Mrps18b	mitochondrial ribosomal protein S18B [Source:MGI Symbol;Acc:MGI:1914223]
ENSMUSG00000024437	2.568013199	4.008647816	3.856032511	1.917142178	2.278403312	2.912280348	0.713273153	0.6218625	1.115427297	3.573153377	4.227721376	3.28731937	Gm8615	predicted pseudogene 8615 [Source:MGI Symbol;Acc:MGI:3647532]
ENSMUSG00000024440	0.288862576	0.44352014	0.468247694	0.280793551	0.202330935	0.146680932	0	0.143079769	0.02240512	0.043063459	0.040074826	0.042666187	Pcdh12	protocadherin 12 [Source:MGI Symbol;Acc:MGI:1855700]
ENSMUSG00000024442	13.13041767	14.51302381	12.30707413	14.90057208	15.99296807	15.32040018	20.24654397	19.34918023	21.17560801	12.94861421	13.06422804	13.0064472	Dele1	DAP3 binding cell death enhancer 1 [Source:MGI Symbol;Acc:MGI:1914089]
ENSMUSG00000024446	38.32224697	31.06522366	53.25848217	40.70245117	29.93285141	26.24482121	64.14980514	64.02202516	51.00009139	25.93427422	24.48419222	22.86484715	Rpp21	ribonuclease P 21 subunit [Source:MGI Symbol;Acc:MGI:1914926]
ENSMUSG00000024448	0.149274893	0	0	0.139300877	0	0.056849992	0	0	0	0	0.031064072	0	H2-M10.1	histocompatibility 2, M region locus 10.1 [Source:MGI Symbol;Acc:MGI:1276522]
ENSMUSG00000024451	3.979822806	2.959406989	3.453287137	2.581413963	2.343857235	2.677927028	2.453706979	2.793398564	3.205925443	5.922453556	5.689698593	5.852001134	Arap3	ArfGAP with RhoGAP domain, ankyrin repeat and PH domain 3 [Source:MGI Symbol;Acc:MGI:2147274]
ENSMUSG00000024454	89.30789949	81.05058975	85.69623856	92.91041217	88.69494398	76.60021408	77.32523144	78.1561151	72.69363805	50.47408366	49.3472062	48.53278779	Hdac3	histone deacetylase 3 [Source:MGI Symbol;Acc:MGI:1343091]
ENSMUSG00000024456	22.78617035	21.78290558	21.41514544	23.93007771	24.57386876	27.34688525	40.12624712	39.49668768	45.62618383	45.08775214	46.43448665	44.2123731	Diaph1	diaphanous related formin 1 [Source:MGI Symbol;Acc:MGI:1194490]
ENSMUSG00000024457	46.52938655	47.51034445	41.69751781	53.71524849	54.74611872	53.30041767	48.17302656	44.58171678	49.01000709	38.99774076	40.5947179	38.58386146	Trim26	tripartite motif-containing 26 [Source:MGI Symbol;Acc:MGI:1337056]
ENSMUSG00000024459	0.935401909	1.118592744	0.957657613	1.074340544	0.83159114	1.205733201	29.93532107	29.86660566	31.44327197	26.09844542	23.02941241	25.28377376	H2-M5	histocompatibility 2, M region locus 5 [Source:MGI Symbol;Acc:MGI:95917]
ENSMUSG00000024462	28.20273501	34.7569554	27.39051247	41.55051242	47.99895528	56.95911443	62.06305548	54.22715866	73.17622747	36.50093332	37.14342642	37.19135149	Gabbr1	gamma-aminobutyric acid (GABA) B receptor, 1 [Source:MGI Symbol;Acc:MGI:1860139]
ENSMUSG00000024471	0	0	0.082033985	0.037386851	0	0.045773772	0.089420051	0.119066614	0.316962559	0.430033144	0.500235691	0.532582468	Myot	myotilin [Source:MGI Symbol;Acc:MGI:1889800]
ENSMUSG00000024472	11.41825415	13.41256025	10.59521616	15.07425882	17.58379899	22.44805895	21.79427155	19.85170941	25.84934142	43.21890344	43.78766006	44.31776232	Dcp2	decapping mRNA 2 [Source:MGI Symbol;Acc:MGI:1917890]
ENSMUSG00000024474	119.00414	119.6439524	124.4077221	128.6810642	123.2285869	117.0950597	128.5037912	127.1529352	122.811478	105.1663057	105.4387399	96.89647178	Ik	IK cytokine [Source:MGI Symbol;Acc:MGI:1345142]
ENSMUSG00000024477	36.09743576	33.58659013	41.31964438	37.82341109	37.47382867	37.11875074	22.29176124	25.73756787	24.80282621	22.85403506	21.96672803	23.76868396	Pggt1b	protein geranylgeranyltransferase type I, beta subunit [Source:MGI Symbol;Acc:MGI:1917514]
ENSMUSG00000024479	0.131273353	0.218353798	0.074664831	0.108890787	0	0.055549188	0.143241829	0.072247199	0.040727951	0.665385181	0.52207649	0.323160209	Mal2	mal, T cell differentiation protein 2 [Source:MGI Symbol;Acc:MGI:2146021]
ENSMUSG00000024480	120.3854804	120.4684937	102.7593326	116.0211546	116.6680805	111.6405882	63.32329033	59.60825714	59.3956042	60.91372367	56.82896677	61.11139805	Ap3s1	adaptor-related protein complex 3, sigma 1 subunit [Source:MGI Symbol;Acc:MGI:1337062]
ENSMUSG00000024481	1.168524705	1.303565758	1.121556582	0.704247702	0.856847901	0.964214469	0	0.084407619	0.101963894	0.500833509	0.44581118	0.496213187	Lvrn	laeverin [Source:MGI Symbol;Acc:MGI:1921824]
ENSMUSG00000024483	4.138554412	9.938641365	3.704732496	7.821097906	13.78755383	30.79395656	12.79257344	9.806623251	19.86004324	38.46936416	40.17929821	40.9343554	Ankhd1	ankyrin repeat and KH domain containing 1 [Source:MGI Symbol;Acc:MGI:1921733]
ENSMUSG00000024485	0.031146209	0.125532613	0.03188726	0.130793087	0	0.05930876	1.084458421	1.342181958	1.203068974	0.598980295	0.401854195	0.455441858	Slc4a9	solute carrier family 4, sodium bicarbonate cotransporter, member 9 [Source:MGI Symbol;Acc:MGI:2443384]
ENSMUSG00000024486	30.16965927	28.05376388	26.35268539	33.82677439	35.093432	35.51092677	28.589206	27.98440632	30.54168388	32.42368596	34.55651274	33.04584703	Hbegf	heparin-binding EGF-like growth factor [Source:MGI Symbol;Acc:MGI:96070]
ENSMUSG00000024487	76.35041169	86.42198874	70.53207597	67.71411806	67.06626044	71.29589221	36.06346342	33.74301974	34.7055921	53.49668503	50.3998124	52.68844946	Yipf5	Yip1 domain family, member 5 [Source:MGI Symbol;Acc:MGI:1914430]
ENSMUSG00000024491	7.832092853	9.099495057	7.017251706	9.125790417	10.59569961	13.52061363	15.41115392	14.72183295	17.88721236	25.78881188	26.4629147	25.95327969	Rbm27	RNA binding motif protein 27 [Source:MGI Symbol;Acc:MGI:2147194]
ENSMUSG00000024493	118.4097663	114.0180612	122.3385451	140.9255366	141.0240982	144.9885185	182.7047938	179.179131	194.7217367	219.7601377	232.7343818	225.0771447	Lars	leucyl-tRNA synthetase [Source:MGI Symbol;Acc:MGI:1913808]
ENSMUSG00000024497	0	0	0	0	0	0	0.220190962	0.545039499	0.406142189	0.237580039	0.221091824	0.319455551	Pou4f3	POU domain, class 4, transcription factor 3 [Source:MGI Symbol;Acc:MGI:102523]
ENSMUSG00000024498	35.50438067	44.45425992	30.74685123	45.10035439	50.89023814	59.60540961	79.74946605	72.56769998	92.23440957	108.5425659	100.1911703	103.5063046	Tcerg1	transcription elongation regulator 1 (CA150) [Source:MGI Symbol;Acc:MGI:1926421]
ENSMUSG00000024500	0.029411133	0.067736863	0	0	0.154505531	0	0.02625759	0	0.082124029	0.368305898	0.048963603	0.208518959	Ppp2r2b	protein phosphatase 2, regulatory subunit B, beta [Source:MGI Symbol;Acc:MGI:1920180]
ENSMUSG00000024501	128.4632712	150.999518	114.9976726	194.5075835	221.4656029	255.246383	11.05750657	9.438167814	11.87177741	62.36685378	65.46735712	64.25558138	Dpysl3	dihydropyrimidinase-like 3 [Source:MGI Symbol;Acc:MGI:1349762]
ENSMUSG00000024502	0.327872834	0.15546688	0.276437249	0.089989865	0.141845911	0.176283392	0.051656012	0.076424648	0.017951211	0.845320642	0.481625399	0.786245458	Jakmip2	janus kinase and microtubule interacting protein 2 [Source:MGI Symbol;Acc:MGI:1923467]
ENSMUSG00000024503	0	0.067969157	0.060428327	0.495721949	0.062014154	0	0.052695273	0.877075056	0	2.111822572	1.031761845	1.098478736	Spink1	serine peptidase inhibitor, Kazal type 1 [Source:MGI Symbol;Acc:MGI:106202]
ENSMUSG00000024505	2.822251977	2.774972873	2.489329981	2.978083371	2.62308608	3.373309894	8.101619073	6.667009479	7.072251783	6.835400988	7.860918685	7.1956137	Dtwd2	DTW domain containing 2 [Source:MGI Symbol;Acc:MGI:1916107]
ENSMUSG00000024507	110.6099546	114.4957734	92.29402702	115.0915127	112.3396913	114.1227197	89.27293771	93.92135934	94.57935218	95.45885445	93.48811566	94.29096771	Hsd17b4	hydroxysteroid (17-beta) dehydrogenase 4 [Source:MGI Symbol;Acc:MGI:105089]
ENSMUSG00000024510	0	0	0	0	0	0	0	0	0	0	0	0	Ftmt	ferritin mitochondrial [Source:MGI Symbol;Acc:MGI:1914884]
ENSMUSG00000024511	2.919045591	4.170196418	2.314400291	5.099829626	4.992400369	6.46118795	0.039188834	0.065227005	0.040856059	0.265028078	0.264904205	0.34038553	Rab27b	RAB27B, member RAS oncogene family [Source:MGI Symbol;Acc:MGI:1931295]
ENSMUSG00000024512	12.46948015	11.56829508	11.14791638	17.14307666	14.35594762	11.42408719	0	0.069593221	0.098079555	0.251349984	0.61400358	0.34241792	Dynap	dynactin associated protein [Source:MGI Symbol;Acc:MGI:1922827]
ENSMUSG00000024513	98.5464403	92.26626294	91.75782105	107.0243132	101.7514128	89.66238818	35.04383023	31.40419252	31.050644	30.61964538	28.59823809	30.77843898	Mbd2	methyl-CpG binding domain protein 2 [Source:MGI Symbol;Acc:MGI:1333813]
ENSMUSG00000024515	50.78455781	63.41003832	48.89569132	60.60562324	71.95460093	86.09002279	49.27559815	46.0690725	54.99644143	57.97663745	59.41797293	57.35622388	Smad4	SMAD family member 4 [Source:MGI Symbol;Acc:MGI:894293]
ENSMUSG00000024516	30.04638851	26.89225848	34.75233071	29.8920335	27.00907224	22.46561073	22.72058036	30.8946315	25.15908237	16.87549295	18.00027588	17.8931322	Sec11c	SEC11 homolog C, signal peptidase complex subunit [Source:MGI Symbol;Acc:MGI:1913536]
ENSMUSG00000024517	0	0.154520319	0.04579236	0	0	0.034068629	0	0.177238452	0.041631135	0.320066248	0.335085114	0.19819597	Grp	gastrin releasing peptide [Source:MGI Symbol;Acc:MGI:95833]
ENSMUSG00000024518	0.439066202	0.126401826	0.168567284	0.025608085	0	0	0.048998538	0.380587926	0.255415507	0.122729485	0	0.121597273	Rax	retina and anterior neural fold homeobox [Source:MGI Symbol;Acc:MGI:109632]
ENSMUSG00000024519	0	0	0	0.068404562	0	0	0	0	0	0	0	0	Cplx4	complexin 4 [Source:MGI Symbol;Acc:MGI:2685803]
ENSMUSG00000024521	8.539848134	9.241660016	6.9944271	5.415246652	6.572610606	6.661386308	36.1735283	36.02098177	39.78100799	29.32153536	30.54112338	29.34282336	Pmaip1	phorbol-12-myristate-13-acetate-induced protein 1 [Source:MGI Symbol;Acc:MGI:1930146]
ENSMUSG00000024524	0.383693522	0.483923083	0.579881	0.375106799	0.959836908	0.473170574	0.864538117	0.651605315	0.816288439	0.882526617	1.460050975	0.971538973	Gnal	guanine nucleotide binding protein, alpha stimulating, olfactory type [Source:MGI Symbol;Acc:MGI:95774]
ENSMUSG00000024525	26.94176449	28.09309338	26.49436374	33.80933277	30.98151335	33.45592484	105.4035658	107.3683576	97.44078348	54.2034689	59.61716595	57.29853234	Impa2	inositol (myo)-1(or 4)-monophosphatase 2 [Source:MGI Symbol;Acc:MGI:2149728]
ENSMUSG00000024526	0	0	0	0	0	0	0.206716704	0.305835679	0.251429651	0.207110133	0.385473099	0.273599316	Cidea	cell death-inducing DNA fragmentation factor, alpha subunit-like effector A [Source:MGI Symbol;Acc:MGI:1270845]
ENSMUSG00000024527	38.95594818	38.2617996	40.12722921	41.5688093	43.05413608	40.65732782	52.43176231	50.05476502	56.15246571	51.40103081	52.63818221	52.04693453	Afg3l2	AFG3-like AAA ATPase 2 [Source:MGI Symbol;Acc:MGI:1916847]
ENSMUSG00000024528	41.40315727	37.23601771	41.24972136	45.30238304	41.74803951	40.42997973	67.13053028	66.09972407	73.21112291	69.47728323	69.74677839	64.09815291	Srfbp1	serum response factor binding protein 1 [Source:MGI Symbol;Acc:MGI:1914472]
ENSMUSG00000024529	328.8665923	407.4896213	277.1895769	211.9371188	248.4739495	308.3320078	8.417065745	7.94201462	10.5928049	82.52555482	82.35936904	85.69759623	Lox	lysyl oxidase [Source:MGI Symbol;Acc:MGI:96817]
ENSMUSG00000024530	0.73823386	0.566742826	0.75579841	1.016958997	0.701763112	0.83006098	2.228312614	2.507402806	3.108390028	3.144441697	3.072526083	3.52043973	Prelid3a	PRELI domain containing 3A [Source:MGI Symbol;Acc:MGI:2442865]
ENSMUSG00000024532	0.092534382	0	0.094736022	0	0	0	0	0	0	0	0	0	1700034E13	RIKEN cDNA 1700034E13 gene [Source:MGI Symbol;Acc:MGI:1925664]
ENSMUSG00000024533	8.647882358	9.569857429	6.62223369	9.500365344	11.0361483	13.01321408	10.60802343	9.500270631	13.02251822	13.9738989	12.7231842	13.39636358	Spire1	spire type actin nucleation factor 1 [Source:MGI Symbol;Acc:MGI:1915416]
ENSMUSG00000024534	0.082366937	0.094849762	0.084326666	0.015372694	0.103847592	0.062737406	0.161777716	0.048957712	0.153327533	0.029470112	0.054849733	0.058396485	Sncaip	synuclein, alpha interacting protein (synphilin) [Source:MGI Symbol;Acc:MGI:1915097]
ENSMUSG00000024535	49.66133773	48.17858041	38.21924474	51.22345838	53.24932359	50.94222773	16.58823244	18.53297726	17.25437753	18.02697239	17.41294737	16.9186504	Snx24	sorting nexing 24 [Source:MGI Symbol;Acc:MGI:1916476]
ENSMUSG00000024537	54.61465534	39.69618664	56.56471719	52.72292998	44.04692482	35.76765145	63.74453425	70.9754838	57.95885772	38.37810538	38.91805119	40.708962	Psmg2	proteasome (prosome, macropain) assembly chaperone 2 [Source:MGI Symbol;Acc:MGI:1922901]
ENSMUSG00000024538	157.2088586	152.4980816	153.7589945	135.7719878	136.0760716	120.350502	38.57913713	36.57984364	37.53715912	41.03920007	41.96931097	40.17137073	Ppic	peptidylprolyl isomerase C [Source:MGI Symbol;Acc:MGI:97751]
ENSMUSG00000024539	24.41727498	22.71401086	22.60968849	27.38749228	28.02191513	27.50352194	42.93564096	43.72891954	41.72711967	37.18369148	34.93414287	35.57191479	Ptpn2	protein tyrosine phosphatase, non-receptor type 2 [Source:MGI Symbol;Acc:MGI:97806]
ENSMUSG00000024542	22.16219715	25.82442851	17.76481759	28.65470241	34.96202261	45.94051081	53.12191918	45.80265922	70.1831226	91.35734744	97.15715993	95.36145996	Cep192	centrosomal protein 192 [Source:MGI Symbol;Acc:MGI:1918049]
ENSMUSG00000024544	4.50062721	3.999110382	4.543934602	2.775719966	2.667012628	4.80400954	0.889812038	1.326754579	0.594287084	2.493429598	2.774090247	2.304811829	Ldlrad4	low density lipoprotein receptor class A domain containing 4 [Source:MGI Symbol;Acc:MGI:1277150]
ENSMUSG00000024546	0	0	0	0	0	0.024403589	0	0	0	0	0	0	4930546C1C	RIKEN cDNA 4930546C10 gene [Source:MGI Symbol;Acc:MGI:1926181]
ENSMUSG00000024548	1.599572038	2.225322363	1.279121904	1.375699381	1.621558289	2.601374089	0.23929679	0.192722109	0.317882344	0.41376594	0.582973358	0.513393874	Setbp1	SET binding protein 1 [Source:MGI Symbol;Acc:MGI:1933199]
ENSMUSG00000024552	0.098613799	0.264970672	0.168266807	0.21472448	0.069073061	0.087631071	0.073366795	0.162818364	0	0.029402572	0.013681007	0.029131325	Slc14a2	solute carrier family 14 (urea transporter), member 2 [Source:MGI Symbol;Acc:MGI:1351653]
ENSMUSG00000024553	0	0	0	0	0	0	0	0	0	0	0.035972757	0.038298866	Galr1	galanin receptor 1 [Source:MGI Symbol;Acc:MGI:1096364]

ENSMUSG00000024556	47.88817586	51.46154399	44.32174632	54.06075298	50.73968906	55.584739	58.10133693	58.65316644	61.3458768	60.33101929	66.25700589	62.53796369	Me2	malic enzyme 2, NAD(+)-dependent, mitochondrial [Source:MGI Symbol;Acc:MGI:2147351]
ENSMUSG00000024558	0.077273549	0.311445623	0.171409534	0.12018401	0.054125493	0.156954304	3.817339584	4.108208009	3.99172926	0.956151215	1.082760977	1.244084589	Mapk4	mitogen-activated protein kinase 4 [Source:MGI Symbol;Acc:MGI:2444559]
ENSMUSG00000024560	65.58278497	61.35516679	65.92626117	72.95842554	63.00441052	61.79079858	39.9801002	39.79982465	39.93851851	42.66104007	39.55192258	43.90024317	Cxxc1	CXXC finger 1 (PHD domain) [Source:MGI Symbol;Acc:MGI:1921572]
ENSMUSG00000024561	39.70980127	46.43070746	37.53033018	44.6743038	48.73520843	52.22633164	8.105261763	8.596968397	8.911646305	20.81352791	21.22657913	18.66078315	Mbd1	methyl-CpG binding domain protein 1 [Source:MGI Symbol;Acc:MGI:1333811]
ENSMUSG00000024563	20.53370197	25.46110052	18.18779231	25.2439425	28.886459	33.59410337	23.06951022	21.63671268	28.84684194	30.92107397	33.94441148	33.17460475	Smad2	SMAD family member 2 [Source:MGI Symbol;Acc:MGI:108051]
ENSMUSG00000024565	0.963200422	0.927499617	0.731087132	0.968580612	0.881134727	1.207996515	0.429961701	0.49354513	0.502353309	0.943259693	0.870885046	1.192113192	Sall3	spalt like transcription factor 3 [Source:MGI Symbol;Acc:MGI:109295]
ENSMUSG00000024566	44.8983352	49.9801607	42.29556537	48.15953937	57.9202688	60.99050875	17.4595119	16.99803743	18.2637235	28.90532853	28.65716264	28.42422037	Atp9b	ATPase, class II, type 9B [Source:MGI Symbol;Acc:MGI:1354757]
ENSMUSG00000024570	13.92507697	14.48534923	15.63450951	13.47112363	14.67929215	14.31874688	32.65469796	31.72797725	25.66256311	29.89356611	27.780295	26.86169014	Rbfa	ribosome binding factor A [Source:MGI Symbol;Acc:MGI:1915981]
ENSMUSG00000024571	142.8696293	114.2632312	154.5680702	131.9510949	106.6959437	91.26183966	100.2817017	106.6662707	92.16398542	87.22511298	89.91231539	85.54138079	Gm16286	predicted gene 16286 [Source:MGI Symbol;Acc:MGI:3833940]
ENSMUSG00000024575	0.037678185	0.014462789	0.012858217	0.058601106	0.145152219	0.057397584	0.123340162	0.037325611	0.116897699	0.168511236	0.052272148	0.166956676	Pde6a	phosphodiesterase 6A, cGMP-specific, rod, alpha [Source:MGI Symbol;Acc:MGI:97524]
ENSMUSG00000024576	146.5068485	175.6511297	127.8172158	166.2930973	182.2019843	213.3495442	186.1375871	180.5001441	213.0042989	202.457486	207.5533715	202.4507443	Csnk1a1	casein kinase 1, alpha 1 [Source:MGI Symbol;Acc:MGI:1934950]
ENSMUSG00000024578	0	0	0.057811352	0	0	0	1.461982635	0.950970356	0.420463533	0.656618849	0.940075472	0.800690912	Il17b	interleukin 17B [Source:MGI Symbol;Acc:MGI:1928397]
ENSMUSG00000024579	19.8898463	15.0061872	19.180468	19.36933704	16.34629632	16.4145053	31.19595532	31.50450656	33.86706773	20.56781424	21.69444974	23.80107474	Pcyox1l	prenylcysteine oxidase 1 like [Source:MGI Symbol;Acc:MGI:3606062]
ENSMUSG00000024580	14.21612584	15.81032248	12.88878557	16.01882561	18.29099806	17.90411163	16.31613237	16.99009053	17.80276771	15.63998633	16.0479852	15.69782119	Grpel2	GrpE-like 2, mitochondrial [Source:MGI Symbol;Acc:MGI:1334416]
ENSMUSG00000024581	45.2843503	51.81051294	41.18671548	53.05716829	55.56672915	66.91627709	42.5174113	39.31510225	45.84266956	54.10735467	54.00350254	50.57237651	Napg	N-ethylmaleimide sensitive fusion protein attachment protein gamma [Source:MGI Symbol;Acc:MGI:104561]
ENSMUSG00000024583	128.415048	123.1017711	135.8693694	137.352194	131.2385955	123.7613867	132.2483043	138.3528754	138.470225	114.428104	111.3316335	123.6618772	Txnl1	thioredoxin-like 1 [Source:MGI Symbol;Acc:MGI:1860078]
ENSMUSG00000024587	174.859829	204.7912092	157.0987578	244.3010647	254.5845219	250.0215003	154.8590655	139.886862	156.8795684	184.1569498	180.7621536	177.1251418	Nars	asparaginyl-tRNA synthetase [Source:MGI Symbol;Acc:MGI:1917473]
ENSMUSG00000024588	28.87505966	32.87438852	24.80252742	33.77455399	35.85549699	41.15441247	12.85106763	12.53701403	14.19909894	17.06469257	15.42175191	17.04459965	Fech	ferrochelatase [Source:MGI Symbol;Acc:MGI:95513]
ENSMUSG00000024589	45.61177468	45.14410498	47.68625801	43.28184229	42.97069306	46.35225833	22.95736467	23.24691052	25.40239492	18.82065083	19.15595494	18.78683434	Nedd4l	neural precursor cell expressed, developmentally down-regulated gene 4-like [Source:MGI Symbol;Acc:MGI:1933754]
ENSMUSG00000024590	38.85096474	38.54165636	28.50439719	47.5833618	48.92619262	51.21057708	258.3733503	236.2314255	271.6068957	185.5159544	195.591641	194.693414	Lmnb1	lamin B1 [Source:MGI Symbol;Acc:MGI:96795]
ENSMUSG00000024592	10.22459117	10.71748853	9.964598864	12.47724216	11.50005966	12.53050903	5.178544684	4.674883525	6.283416004	8.559395415	9.41113636	9.438837794	C330018D2(	RIKEN cDNA C330018D20 gene [Source:MGI Symbol;Acc:MGI:1924672]
ENSMUSG00000024593	0.212778237	0.226875072	0.201704442	0.13972822	0.099358941	0.11404879	0.02814275	0.046841591	0.073350094	0.44409192	0.400151906	0.419042909	Megf10	multiple EGF-like-domains 10 [Source:MGI Symbol;Acc:MGI:2685177]
ENSMUSG00000024594	23.81202371	25.4320473	23.04502068	20.19965773	21.51239366	24.30182483	20.54018728	20.47200577	23.19853913	35.92210195	36.04834508	36.74507448	Prrc1	proline-rich coiled-coil 1 [Source:MGI Symbol;Acc:MGI:1916106]
ENSMUSG00000024597	10.3424838	12.93692808	8.770402539	14.25083432	16.25855522	21.80366209	3.680841472	3.479819203	4.645701577	13.01593045	13.43458438	14.10917473	Slc12a2	solute carrier family 12, member 2 [Source:MGI Symbol;Acc:MGI:101924]
ENSMUSG00000024598	2.139269451	2.65010592	1.708995017	1.497248326	2.1284519	2.259000292	0.318315175	0.032109866	0.271519671	2.507878788	2.805992505	2.901260545	Fbn2	fibrillin 2 [Source:MGI Symbol;Acc:MGI:95490]
ENSMUSG00000024600	3.012859912	3.449523623	2.570453231	5.009085766	5.221242401	6.924088867	0	0	0.032232718	0.32525042	0.201785214	0.276214199	Slc27a6	solute carrier family 27 (fatty acid transporter), member 6 [Source:MGI Symbol;Acc:MGI:3036230]
ENSMUSG00000024601	23.20201598	25.28857154	19.98041646	30.19657395	30.94058938	36.1282599	32.95918273	31.80603153	41.29504487	34.34070579	32.11892035	32.32184888	Isoc1	isochorismatase domain containing 1 [Source:MGI Symbol;Acc:MGI:1913557]
ENSMUSG00000024603	47.50705056	57.34714856	41.83647466	67.74899547	73.10329167	81.44539989	20.71876841	22.18327233	26.54652424	34.59700164	35.85254966	36.84155134	Dctn4	dynactin 4 [Source:MGI Symbol;Acc:MGI:1914915]
ENSMUSG00000024604	40.06916955	41.5070908	37.9927935	53.49194399	52.20410351	55.69780261	47.97893741	46.55422706	53.51822655	40.57912823	38.15704272	40.38835847	Rbm22	RNA binding motif protein 22 [Source:MGI Symbol;Acc:MGI:1914060]
ENSMUSG00000024608	1864.658583	1246.499603	2174.801042	1524.942682	1234.001107	907.4566252	2630.444976	3075.198617	2129.702349	1195.867398	1234.718775	1252.714189	Rps14	ribosomal protein S14 [Source:MGI Symbol;Acc:MGI:98107]
ENSMUSG00000024610	1.051448011	0.884812779	0.703842343	0.528335235	0.552357273	0.338829161	2.274563728	2.604022651	1.39268666	3.870500378	5.184021451	4.838291478	Cd74	CD74 antigen (invariant polypeptide of major histocompatibility complex, class II antigen-associated) [Source:MGI Symbol;Acc:MGI:9
ENSMUSG00000024613	23.45825856	22.20324281	23.23212389	26.1125741	25.80078363	26.48783798	97.14041701	93.25567554	105.9008803	88.62445733	92.55600372	89.88195909	Tcof1	treacle ribosome biogenesis factor 1 [Source:MGI Symbol;Acc:MGI:892003]
ENSMUSG00000024614	37.73496339	46.90556914	31.36328683	47.29540077	56.19769218	73.43388517	23.47785301	23.37682359	30.15558642	53.1111117	51.86722432	56.44574232	Tmx3	thioredoxin-related transmembrane protein 3 [Source:MGI Symbol;Acc:MGI:2442418]
ENSMUSG00000024617	1.267407594	0.851366248	1.492196988	0.512513708	0.532645872	0.57921686	2.979649948	2.218134441	2.890144955	2.62632662	3.516629895	3.575544819	Camk2a	calcium/calmodulin-dependent protein kinase II alpha [Source:MGI Symbol;Acc:MGI:88256]
ENSMUSG00000024619	0.36881561	0.028314009	0.075518143	0.114724222	0.025833325	0.018728012	0.175610761	0.219128645	0.228852294	1.869415509	1.187073587	1.176672488	Cdx1	caudal type homeobox 1 [Source:MGI Symbol;Acc:MGI:88360]
ENSMUSG00000024620	34.05726664	35.47617213	30.84064275	12.22771652	14.46833162	15.11902189	1.957987022	1.8498752	1.89336709	12.7653532	13.72484148	12.30252715	Pdgfrb	platelet derived growth factor receptor, beta polypeptide [Source:MGI Symbol;Acc:MGI:97531]
ENSMUSG00000024621	8.294141	9.57537117	8.469928732	0.825071462	0.751997468	0.52913047	0.394673158	0.50049919	0.842521289	7.983805157	8.165686875	9.570538212	Csf1r	colony stimulating factor 1 receptor [Source:MGI Symbol;Acc:MGI:1339758]
ENSMUSG00000024622	7.378603691	10.94430121	5.569824738	8.806572026	11.46007697	15.1601481	10.65687005	8.978044343	14.07132351	18.81952751	18.25892907	17.61529334	Hmgxb3	HMG box domain containing 3 [Source:MGI Symbol;Acc:MGI:2441817]
ENSMUSG00000024639	18.77069284	23.76419448	17.77978325	20.84394792	25.62257631	32.29556778	10.35832046	9.883193541	13.20641836	18.85563316	16.54742723	17.45370391	Gnaq	guanine nucleotide binding protein, alpha q polypeptide [Source:MGI Symbol;Acc:MGI:95776]
ENSMUSG00000024640	167.7482362	175.3538053	150.3569092	223.5492731	216.3173999	218.9102401	143.8997983	148.3254944	151.842197	175.8512684	184.4917953	176.9527728	Psat1	phosphoserine aminotransferase 1 [Source:MGI Symbol;Acc:MGI:2183441]
ENSMUSG00000024642	2.169399415	2.48389966	1.738737619	2.625998118	2.826333933	4.86275443	26.34034938	24.95411194	33.78389964	37.45674169	37.89097285	40.47295329	Tle4	transducin-like enhancer of split 4 [Source:MGI Symbol;Acc:MGI:104633]
ENSMUSG00000024644	139.13488	118.9025383	144.7275819	111.9410278	97.10865751	92.82378871	133.9149395	137.0266765	122.3541101	116.001971	123.0616001	118.8830935	Cndp2	CNDP dipeptidase 2 (metallopeptidase M20 family) [Source:MGI Symbol;Acc:MGI:1913304]
ENSMUSG00000024645	12.97492971	13.62090472	13.40720658	16.69776463	16.54890756	15.32978385	20.09641033	19.66886942	19.82798105	12.33450255	12.28222448	12.03352532	Timm21	translocase of inner mitochondrial membrane 21 [Source:MGI Symbol;Acc:MGI:1920595]
ENSMUSG00000024646	165.8801421	140.1860265	183.0581139	98.18447584	93.39857525	70.60504564	118.7285949	119.9950247	98.25957906	97.60258139	98.92882568	101.0140196	Cyb5a	cytochrome b5 type A (microsomal) [Source:MGI Symbol;Acc:MGI:1926952]
ENSMUSG00000024647	0.188473446	0.086814745	0.212253505	0.123116226	0.158417235	0.114845451	0	0.03734196	0.087711677	0.573189162	0.423589855	0.718228165	Cbln2	cerebellin 2 precursor protein [Source:MGI Symbol;Acc:MGI:88282]
ENSMUSG00000024650	0.010803035	0.099522	0.033180204	0.090730932	0	0	0.057868221	0.128423342	0.010055022	0	0.008992437	0	Slc22a6	solute carrier family 22 (organic anion transporter), member 6 [Source:MGI Symbol;Acc:MGI:892001]
ENSMUSG00000024653	0	0.312286863	0	0	0	0.275411938	0	0	0	0	0	0	Scgb1a1	secretoglobin, family 1A, member 1 (uteroglobin) [Source:MGI Symbol;Acc:MGI:98919]
ENSMUSG00000024654	6.345653874	6.144315489	7.47210444	6.988625682	6.587040185	6.908960949	28.1391358	30.8461626	27.26109207	10.36345596	9.675949532	9.676773422	Asrgl1	asparaginase like 1 [Source:MGI Symbol;Acc:MGI:1913764]
ENSMUSG00000024658	0	0	0	0	0	0.016248895	0.03809108	0	0.038163576	0	0	0	Gm9750	predicted gene 9750 [Source:MGI Symbol;Acc:MGI:3642669]
ENSMUSG00000024659	1035.850307	933.6115444	977.5404092	984.368593	961.8510069	853.3659767	33.71693361	34.73729796	31.59722019	184.3173714	175.5957207	174.2805806	Anxa1	annexin A1 [Source:MGI Symbol;Acc:MGI:96819]
ENSMUSG00000024660	70.22897289	72.44060249	73.55864515	83.85418543	85.08068022	73.73561341	145.0640579	151.3973809	146.4795542	117.5541218	116.8133289	118.871944	Incenp	inner centromere protein [Source:MGI Symbol;Acc:MGI:1313288]
ENSMUSG00000024661	6085.546255	4639.341496	6790.94039	3620.052169	3086.860103	2478.530764	1120.937767	1298.314249	1077.193272	1465.276607	1461.893003	1494.505139	Fth1	ferritin heavy polypeptide 1 [Source:MGI Symbol;Acc:MGI:95588]
ENSMUSG00000024663	5.611646614	5.935408045	5.11481564	3.775674531	2.846312766	2.612810312	1.507696644	1.76010246	2.177652147	3.147012786	3.821833401	3.133570663	Rab3il1	RAB3A interacting protein (rabin3)-like 1 [Source:MGI Symbol;Acc:MGI:1922010]
ENSMUSG00000024664	76.56748032	77.37422716	79.14324608	70.34128013	67.91664416	62.72157866	1.922007182	2.553622212	2.030141321	14.06242353	14.78418757	15.91590624	Fads3	fatty acid desaturase 3 [Source:MGI Symbol;Acc:MGI:1928740]
ENSMUSG00000024665	51.53994093	57.72298124	49.41932791	64.86119623	71.10795042	67.56697478	50.01030284	45.9227134	50.27388357	30.53220604	34.59110972	32.70399013	Fads2	fatty acid desaturase 2 [Source:MGI Symbol;Acc:MGI:1930079]
ENSMUSG00000024666	10.81228668	7.840802472	12.97362973	10.35459648	7.485040254	6.338711684	6.782402807	7.61956545	7.129629172	5.188121474	3.279933652	4.413918638	Tmem138	transmembrane protein 138 [Source:MGI Symbol;Acc:MGI:1920232]
ENSMUSG00000024667	11.94029682	11.44988807	15.42495542	14.50549452	11.49595986	10.88057799	12.9083213	15.48466718	13.37665909	11.02985111	11.99919542	10.54330564	Tmem216	transmembrane protein 216 [Source:MGI Symbol;Acc:MGI:1920020]
ENSMUSG00000024668	47.60936684	42.59733743	44.70286805	44.62595752	41.84203741	38.84477769	22.7179537	24.50002569	22.18106854	27.11921323	28.14655384	25.64877835	Sdhaf2	succinate dehydrogenase complex assembly factor 2 [Source:MGI Symbol;Acc:MGI:1913322]
ENSMUSG00000024669	0.07007897	0	0.143492672	0.043597696	0.073629183	0.035585256	0	0	0	0.188051953	0.058333673	0.020701903	Cd5	CD5 antigen [Source:MGI Symbol;Acc:MGI:88340]
ENSMUSG00000024670	0.180412947	0.166203826	0.03694109	0.084179199	0.056865809	0.068708639	0.209389142	0.232342147	0.235089359	1.258725944	1.021193934	1.039261553	Cd6	CD6 antigen [Source:MGI Symbol;Acc:MGI:103566]
ENSMUSG00000024672	6.427039954	5.049032921	8.224945448	0.152481561	0.200290018	0.103715255	0.729395963	0.539567692	0.405561028	6.065508624	6.778003651	7.843793686	Ms4a7	membrane-spanning 4-domains, subfamily A, member 7 [Source:MGI Symbol;Acc:MGI:1918846]
ENSMUSG00000024673	0	0.051506773	0.01526412	0	0	0	0	0	0	0.040008281	0	0.013213065	Ms4a1	membrane-spanning 4-domains, subfamily A, member 1 [Source:MGI Symbol;Acc:MGI:88321]
ENSMUSG00000024675	1.164190688	0.482625152	0.715133932	0.173824579	0.097853503	0.248288035	0.041574517	0.09226374	0	0.333229146	0.077525705	0.165077509	Ms4a4c	membrane-spanning 4-domains, subfamily A, member 4C [Source:MGI Symbol;Acc:MGI:1927656]
ENSMUSG00000024677	1.235160141	1.525794061	0.965654754	0.14669851	0.023595156	0.13684351	0.100247532	0.044494622	0.250829759	2.289993824	1.962824596	2.268868024	Ms4a6b	membrane-spanning 4-domains, subfamily A, member 6B [Source:MGI Symbol;Acc:MGI:1917024]
ENSMUSG00000024678	80.48289528	83.24842612	73.69216474	78.05114231	82.28929857	78.26340817	0.812475433	0.957884218	0.741160768	4.146423343	3.195821672	2.847996186	Ms4a4d	membrane-spanning 4-domains, subfamily A, member 4D [Source:MGI Symbol;Acc:MGI:1913857]
ENSMUSG00000024679	24.70456906	22.79654521	21.16071334	0.66159089	0.343789493	0.747696288	1.071136615	1.512704354	2.84252533	28.19524026	27.8273063	27.35516357	Ms4a6d	membrane-spanning 4-domains, subfamily A, member 6D [Source:MGI Symbol;Acc:MGI:1916024]
ENSMUSG00000024680	0.152044127	0.393944912	0.233492492	0.177356351	0.079873354	0.11580926	0	0	0	0	0.031640349	0	Ms4a2	membrane-spanning 4-domains, subfamily A, member 2 [Source:MGI Symbol;Acc:MGI:95495]

ENSMUSG00000024681	0	0.0427151	0	0	0.038972688	0	0	0	0	0	0	0	Ms4a3	membrane-spanning 4-domains, subfamily A, member 3 [Source:MGI Symbol;Acc:MGI:2158468]
ENSMUSG00000024682	0	0	0	0.026124579	0.02941335	0	0	0	0	0	0.023303107	0.124049788	Cblif	cobalamin binding intrinsic factor [Source:MGI Symbol;Acc:MGI:1202394]
ENSMUSG00000024683	10.54945551	11.31125526	9.886243408	11.53055951	12.34937654	11.34120309	15.31402966	16.76644106	12.35106555	18.83532717	17.85657933	18.12785286	Mrpl16	mitochondrial ribosomal protein L16 [Source:MGI Symbol;Acc:MGI:2137219]
ENSMUSG00000024687	14.37159163	16.67107143	12.23019246	16.32800736	17.18480194	18.46824826	9.817700004	8.728421131	10.81541258	11.94601987	12.26616257	12.5410355	Osbp	oxysterol binding protein [Source:MGI Symbol;Acc:MGI:97447]
ENSMUSG00000024691	32.12222696	34.92950213	28.2510698	46.277133	49.78861367	52.76698879	40.08090889	38.39811521	46.04299894	19.9759797	19.62097587	18.27342891	Fam111a	family with sequence similarity 111, member A [Source:MGI Symbol;Acc:MGI:1915508]
ENSMUSG00000024694	0	0.459500918	0.03404347	0.217213559	0.10481063	0.025327682	0.059373808	0.428234946	0.030949885	0	0.221433542	0	Keg1	kidney expressed gene 1 [Source:MGI Symbol;Acc:MGI:1928492]
ENSMUSG00000024695	17.62966764	21.94349909	14.5985565	21.01626094	24.89680838	26.61942949	7.584214009	6.780412006	9.306460798	12.42993572	11.95574353	12.32292501	Zfp91	zinc finger protein 91 [Source:MGI Symbol;Acc:MGI:104854]
ENSMUSG00000024696	1.649087092	1.548422363	2.000013718	0.568209592	0.69305205	0.541080529	0.181202142	0.502663298	0.212524979	2.360111145	1.309343325	1.843689967	Lpxn	leupaxin [Source:MGI Symbol;Acc:MGI:2147677]
ENSMUSG00000024697	0.184355132	0.084917765	0.037748286	0	0.077477838	0.084251981	0.95461077	0.858361115	1.183972346	0.890467088	0.82866807	0.457464152	Gna14	guanine nucleotide binding protein, alpha 14 [Source:MGI Symbol;Acc:MGI:95769]
ENSMUSG00000024712	46.08993614	56.09671511	40.05689365	61.69005782	68.07757712	72.21046924	33.51913772	31.86107146	36.60473661	38.698612	36.88102033	37.23846596	Rfk	riboflavin kinase [Source:MGI Symbol;Acc:MGI:1914688]
ENSMUSG00000024713	12.29385367	14.68842355	11.83041983	2.256587557	2.880066613	2.994794679	0.271919816	0.201151576	0.206173238	2.782163163	2.527617339	2.895548747	Pcsk5	proprotein convertase subtilisin/kexin type 5 [Source:MGI Symbol;Acc:MGI:97515]
ENSMUSG00000024725	105.5498334	93.43538224	115.2318286	98.08886686	89.52383328	74.74673408	48.25420628	49.10518062	44.98638983	42.86421629	42.78305413	44.43133778	Ostf1	osteoclast stimulating factor 1 [Source:MGI Symbol;Acc:MGI:700012]
ENSMUSG00000024726	7.468415989	7.608813474	5.96519535	8.912614671	8.036099399	7.976180918	18.17957496	16.85992115	19.54935316	20.36334005	19.05011106	18.41877853	Carnmt1	carnosine N-methyltransferase 1 [Source:MGI Symbol;Acc:MGI:1914633]
ENSMUSG00000024727	0.210783062	0.110330696	0.117708083	0.178817537	0.342258478	0.284610734	0.017107483	0.018982787	0.02675294	0.274240687	0.27115871	0.331147469	Trpm6	transient receptor potential cation channel, subfamily M, member 6 [Source:MGI Symbol;Acc:MGI:2675603]
ENSMUSG00000024728	0	0	0	0	0	0	0	0	0	0	0	0	Ms4a19	membrane-spanning 4-domains, subfamily A, member 19 [Source:MGI Symbol;Acc:MGI:1916666]
ENSMUSG00000024729	0.023158497	0	0	0	0	0	0	0	0	0	0	0	Ms4a20	membrane-spanning 4-domains, subfamily A, member 20 [Source:MGI Symbol;Acc:MGI:1916619]
ENSMUSG00000024730	0	0	0	0	0	0	0	0	0	0.034090316	0.031724425	0	Ms4a8a	membrane-spanning 4-domains, subfamily A, member 8A [Source:MGI Symbol;Acc:MGI:1927657]
ENSMUSG00000024731	0	0	0	0	0	0	0	0	0.158088756	1.01284122	1.178186693	1.455071363	Ms4a10	membrane-spanning 4-domains, subfamily A, member 10 [Source:MGI Symbol;Acc:MGI:1917076]
ENSMUSG00000024732	9.745768611	10.50606101	12.10640406	11.00854718	8.738491515	8.726810229	11.17591908	11.50421465	10.36117102	12.44975638	13.03395173	13.8266252	Ccdc86	coiled-coil domain containing 86 [Source:MGI Symbol;Acc:MGI:1277220]
ENSMUSG00000024734	0	0.040547885	0.144197218	0.06571764	0	0.053639968	0	0	0	0.062991762	0.146550227	0.093615969	Zp1	zona pellucida glycoprotein 1 [Source:MGI Symbol;Acc:MGI:103073]
ENSMUSG00000024735	105.4764463	118.7913984	102.5907578	140.1806351	136.9814814	127.5637937	188.2868904	165.2828338	175.1813162	169.5073204	174.1494129	173.6646737	Prpf19	pre-mRNA processing factor 19 [Source:MGI Symbol;Acc:MGI:106247]
ENSMUSG00000024736	20.524977	23.72073932	16.88638039	18.04922124	20.51567863	20.18271232	6.702376774	6.722684188	7.89968223	13.3971864	12.62133128	12.48701047	Tmem132a	transmembrane protein 132A [Source:MGI Symbol;Acc:MGI:2147810]
ENSMUSG00000024737	7.347378242	10.07366216	8.007494653	0.562993179	0.452762327	0.492348833	1.308067139	0.768417885	0.782131652	15.84231766	15.19123362	17.1473953	Slc15a3	solute carrier family 15, member 3 [Source:MGI Symbol;Acc:MGI:1929691]
ENSMUSG00000024738	0.163399321	0	0	0.101654374	0	0.082972204	1.458791927	1.348919231	1.216683084	3.994953471	3.85371445	5.020027959	Pga5	pepsinogen 5, group I [Source:MGI Symbol;Acc:MGI:1915935]
ENSMUSG00000024740	835.3389401	895.0659866	858.8581042	832.3135333	876.8873817	903.61138	625.0436353	572.2857119	661.4768619	822.6061458	836.3371143	828.7489432	Ddb1	damage specific DNA binding protein 1 [Source:MGI Symbol;Acc:MGI:1202384]
ENSMUSG00000024742	0.057576032	0.022100587	0.137540478	0.053729007	0.423449904	0.233891316	0.616830841	0.304198735	0.464441543	0.635171613	0.527188622	0.476236098	Fen1	flap structure specific endonuclease 1 [Source:MGI Symbol;Acc:MGI:102779]
ENSMUSG00000024743	0.268775336	0.112548587	0.300185691	0.228015206	0.077015875	0.241943366	5.671699068	6.245013462	5.867507719	3.387640425	2.644062358	3.399696784	Syt7	synaptotagmin VII [Source:MGI Symbol;Acc:MGI:1859545]
ENSMUSG00000024747	0.18744259	0.119916543	0.298514764	0.136047602	0.043764103	0.1110446	0	0.041264131	0.019384875	0.055887657	0.104018031	0.110744157	Aldh1a7	aldehyde dehydrogenase family 1, subfamily A7 [Source:MGI Symbol;Acc:MGI:1347050]
ENSMUSG00000024749	0.016323402	0	0	0	0	0	0.014573162	0	0	0.058403591	0	0.043398602	Tmc1	transmembrane channel-like gene family 1 [Source:MGI Symbol;Acc:MGI:2151016]
ENSMUSG00000024750	31.32612372	35.16911783	27.19190568	38.2650717	40.49445595	45.67270698	20.01566035	18.85982742	23.54374625	24.99574301	22.75125166	24.1752233	Zfand5	zinc finger, AN1-type domain 5 [Source:MGI Symbol;Acc:MGI:1278334]
ENSMUSG00000024754	6.935803479	10.17443853	6.863376763	6.225025825	7.47283291	10.50686647	25.70521678	21.8380883	30.17049787	28.58936722	28.00261877	29.7742033	Cemip2	cell migration inducing hyaluronidase 2 [Source:MGI Symbol;Acc:MGI:1890373]
ENSMUSG00000024757	0	0.020020006	0	0	0	0	0.015521153	0.017222565	0	0.04665208	0	0	Slc22a19	solute carrier family 22 (organic anion transporter), member 19 [Source:MGI Symbol;Acc:MGI:2442751]
ENSMUSG00000024758	73.7545988	76.0556209	67.75201875	73.95990011	74.50722017	73.0826123	52.90298539	52.79174593	56.19764468	54.81813513	53.97362766	53.42413602	Rtn3	reticulon 3 [Source:MGI Symbol;Acc:MGI:1339970]
ENSMUSG00000024759	40.36286067	51.38749001	36.11779137	49.95000512	58.32996792	78.93337743	17.0553138	16.6428262	19.66466943	36.898406	36.83043142	37.06839148	Atl3	atlastin GTPase 3 [Source:MGI Symbol;Acc:MGI:1924270]
ENSMUSG00000024761	0.939113822	1.081437841	0.611836808	1.354383181	1.076388532	0.780333825	0.609759588	1.26862642	0.874088625	0.458190076	0.994913208	0.075660525	Gm16437	predicted pseudogene 16437 [Source:MGI Symbol;Acc:MGI:3644508]
ENSMUSG00000024764	16.07586726	17.30584225	13.13490598	17.97070288	19.15952175	20.33230496	24.87093347	22.93365416	27.4457666	27.12782721	27.91498591	27.71426617	Naa40	N(alpha)-acetyltransferase 40, NatD catalytic subunit [Source:MGI Symbol;Acc:MGI:1918249]
ENSMUSG00000024766	1.680175241	1.750540716	1.441650434	0.716759738	0.975114341	1.243194535	1.614309598	1.870528088	2.36809784	3.292016462	3.18342627	3.984881449	Lipo3	lipase, member O3 [Source:MGI Symbol;Acc:MGI:2147592]
ENSMUSG00000024767	166.3133302	145.0594471	189.8146757	157.3822431	145.8127451	115.6652559	103.5346017	108.237062	91.057427	88.28225687	90.76731321	87.42946659	Otub1	OTU domain, ubiquitin aldehyde binding 1 [Source:MGI Symbol;Acc:MGI:2147616]
ENSMUSG00000024768	0	0	0	0	0	0	0	0	0	0	0	0	Lipf	lipase, gastric [Source:MGI Symbol;Acc:MGI:1914967]
ENSMUSG00000024769	4.366988933	6.427450647	3.576712988	3.09460676	3.613224302	4.469657112	13.73090407	11.73461332	18.49399449	24.93844443	27.77426595	25.42193646	Cdc42bpg	CDC42 binding protein kinase gamma (DMPK-like) [Source:MGI Symbol;Acc:MGI:2652845]
ENSMUSG00000024770	0.118318482	0.726665674	0.242267187	0.110412863	0.082875011	0.180242093	0	0	0.036708663	0.317499264	0.262635201	0.279618	Lipn	lipase, family member N [Source:MGI Symbol;Acc:MGI:1917416]
ENSMUSG00000024771	0.102497589	0.236062485	0.041974512	0.019129813	0.064614081	0.015614112	0	0	0.01908011	0.018336335	0	0	Lipk	lipase, family member K [Source:MGI Symbol;Acc:MGI:2679259]
ENSMUSG00000024772	60.69670375	60.38001084	48.694759	53.97680627	50.39615816	52.34632804	38.05499595	38.53296771	47.86514518	57.32633867	60.25399427	61.82829277	Ehd1	EH-domain containing 1 [Source:MGI Symbol;Acc:MGI:1341878]
ENSMUSG00000024773	13.97308339	19.6367838	14.22079181	14.90885191	17.30753242	18.6883796	13.92335226	11.07058376	15.99500549	22.8351074	24.48887562	23.62215335	Atg2a	autophagy related 2A [Source:MGI Symbol;Acc:MGI:1916291]
ENSMUSG00000024774	0.026610073	0	0.163459184	0	0	0.081073644	0	0.026361068	0	0	0.066450604	0.070747504	Ankrd22	ankyrin repeat domain 22 [Source:MGI Symbol;Acc:MGI:1277101]
ENSMUSG00000024776	29.11238736	31.74991001	22.83921071	29.86622318	28.14622575	28.18746348	4.508189486	4.955401578	6.685891411	12.36280132	11.84028941	11.74451445	Stambpl1	STAM binding protein like 1 [Source:MGI Symbol;Acc:MGI:1923880]
ENSMUSG00000024777	48.4219204	47.54013435	36.17562666	49.55346251	52.4999826	47.45436536	15.15350926	15.16458729	18.89877012	20.07759352	24.26304468	20.59528309	Ppp2r5b	protein phosphatase 2, regulatory subunit B', beta [Source:MGI Symbol;Acc:MGI:2388480]
ENSMUSG00000024778	42.13690655	34.72930014	44.60786416	29.69910388	28.95776067	26.86879169	2.007262645	2.726518186	2.525622168	10.19411001	10.486923	10.34054442	Fas	Fas (TNF receptor superfamily member 6) [Source:MGI Symbol;Acc:MGI:95484]
ENSMUSG00000024780	13.30181747	15.3177266	12.15778948	13.25856325	15.00867558	18.57488177	27.45147502	25.01785864	32.10032799	45.4026951	46.53626294	44.36879636	Cdc37l1	cell division cycle 37-like 1 [Source:MGI Symbol;Acc:MGI:1914322]
ENSMUSG00000024781	42.71713842	49.54951562	38.76088006	35.4853697	38.07704961	42.11769946	9.172866746	6.394905275	10.29724928	26.32703151	27.4198656	25.14601096	Lipa	lysosomal acid lipase A [Source:MGI Symbol;Acc:MGI:96789]
ENSMUSG00000024782	27.11028443	26.04564159	24.88407132	22.41518829	21.85387025	22.44931408	8.485115625	9.209446878	9.256985536	13.5880871	12.79637313	13.87697135	Ak3	adenylate kinase 3 [Source:MGI Symbol;Acc:MGI:1860835]
ENSMUSG00000024784	0.376342459	0.144459229	0.128432216	0.1755983	0.329506694	0.04777554	1.23196325	1.429146497	0.992471685	0.224419629	1.096434964	0.555873246	Gpha2	glycoprotein hormone alpha 2 [Source:MGI Symbol;Acc:MGI:2156541]
ENSMUSG00000024785	16.40402763	15.12818725	14.83538361	12.8909213	12.52790167	12.4768777	54.1016139	50.94759671	50.36550021	48.96570993	48.98598696	47.68681031	Rcl1	RNA terminal phosphate cyclase-like 1 [Source:MGI Symbol;Acc:MGI:1913275]
ENSMUSG00000024786	0.368552358	0.31830524	0.031443433	0.45856934	0.516297711	0.257326358	1.17904263	1.338713275	0.828997426	0.274717818	0.664695762	0.653240337	Majin	membrane anchored junction protein [Source:MGI Symbol;Acc:MGI:1923913]
ENSMUSG00000024787	48.17133498	40.66613633	45.63181295	30.23508889	28.5178635	26.41942965	32.59905943	31.41745747	31.22025491	23.10300239	23.11686784	21.57319708	Snx15	sorting nexin 15 [Source:MGI Symbol;Acc:MGI:1916274]
ENSMUSG00000024789	10.66145585	12.16705003	9.369809145	10.45498163	11.81581048	13.95810563	2.60970476	2.421923165	3.047218051	9.108565164	8.290615693	9.354242677	Jak2	Janus kinase 2 [Source:MGI Symbol;Acc:MGI:96629]
ENSMUSG00000024790	24.02075777	17.24955398	23.92018856	19.35275111	15.39340105	10.36404536	23.92270385	27.25452953	22.8574561	21.85967022	21.58451095	24.83134362	Sac3d1	SAC3 domain containing 1 [Source:MGI Symbol;Acc:MGI:1913656]
ENSMUSG00000024791	9.740106105	10.49346846	11.39979894	14.49082831	13.06669385	12.89005242	47.87848101	46.74795831	44.35481395	32.89463144	36.59087603	32.17914485	Cdca5	cell division cycle associated 5 [Source:MGI Symbol;Acc:MGI:1915099]
ENSMUSG00000024792	29.79649271	29.51525817	33.06427435	34.89419543	28.67999239	24.89780383	18.76990022	20.39049662	18.92983813	19.419327	17.21494471	17.5897795	Zfpl1	zinc finger like protein 1 [Source:MGI Symbol;Acc:MGI:1891017]
ENSMUSG00000024793	1.784591661	1.187361665	1.380439177	1.036218783	1.374999544	1.057226472	6.302160391	5.42153639	5.019985813	1.880057344	2.673886557	1.405821371	Tnfrsf25	tumor necrosis factor receptor superfamily, member 25 [Source:MGI Symbol;Acc:MGI:1934667]
ENSMUSG00000024795	16.48451872	19.03259424	13.2001715	20.85396813	25.34302411	32.03252696	54.21342151	47.91925362	62.60070995	77.18878726	73.20040162	74.3102758	Kif20b	kinesin family member 20B [Source:MGI Symbol;Acc:MGI:2444576]
ENSMUSG00000024797	30.16282702	28.86273003	28.49004885	27.70278703	28.687006	26.49505544	21.01543512	22.56384851	22.75217578	21.35378865	24.07623856	21.87468951	Vps51	VPS51 GARP complex subunit [Source:MGI Symbol;Acc:MGI:1915755]
ENSMUSG00000024798	0.128924308	0.055673613	0.115492791	0	0.033863909	0.012274914	0.014387586	0	0	0	0.01341456	0	Htr7	5-hydroxytryptamine (serotonin) receptor 7 [Source:MGI Symbol;Acc:MGI:99841]
ENSMUSG00000024799	11.37181464	11.85649124	11.68171278	12.61966446	12.43228755	10.88565685	43.90269037	46.66007971	44.59043924	10.51546224	9.210053695	10.35035984	Tm7sf2	transmembrane 7 superfamily member 2 [Source:MGI Symbol;Acc:MGI:1920416]
ENSMUSG00000024800	24.5657736	18.4318894	22.63523293	24.67952384	24.80206582	23.06753103	30.01910095	34.67865576	31.04613084	22.21383965	20.89583993	22.72326524	Rpp30	ribonuclease P/MRP 30 subunit [Source:MGI Symbol;Acc:MGI:1859683]
ENSMUSG00000024803	177.7755944	172.9443083	165.8938659	240.0798436	241.1191243	239.3806019	2.331298878	1.808508039	2.301428149	13.02224426	12.17619881	12.22628577	Ankrd1	ankyrin repeat domain 1 (cardiac muscle) [Source:MGI Symbol;Acc:MGI:1097717]
ENSMUSG00000024805	11.2140317	12.28994278	10.81093502	16.54988651	17.99324259	21.15617169	7.93678684	7.063328722	8.127436623	7.568425479	8.789878198	8.198474527	Pcgf5	polycomb group ring finger 5 [Source:MGI Symbol;Acc:MGI:1923505]

ENSMUSG00000024806	0.365977418	0	0	0	0.054931128	0.119467876	0.980208913	1.191244832	0.486623955	2.478568916	2.132474968	2.038697213	Mlana	melan-A [Source:MGI Symbol;Acc:MGI:108454]
ENSMUSG00000024807	29.07535434	27.81696519	28.6479524	24.79484341	25.77863178	22.67814201	15.69668349	14.54452884	16.26555166	15.82162344	17.95899085	19.36247348	Syvn1	synovial apoptosis inhibitor 1, synoviolin [Source:MGI Symbol;Acc:MGI:1921376]
ENSMUSG00000024810	161.4799086	198.5760146	126.8812332	364.9341635	433.0481021	488.1104141	0.263717534	0.780335885	1.252490627	2.78898307	3.005229827	2.123342519	Il33	interleukin 33 [Source:MGI Symbol;Acc:MGI:1924375]
ENSMUSG00000024811	77.95688862	111.3675864	64.91632553	108.4240193	127.0603096	168.2910592	144.8387394	129.8452831	180.7494081	259.0092461	259.8870767	257.3971067	Tnks2	tankyrase, TRF1-interacting ankyrin-related ADP-ribose polymerase 2 [Source:MGI Symbol;Acc:MGI:1921743]
ENSMUSG00000024812	13.70148325	18.22858197	12.96795577	17.70996888	20.19978723	20.89454839	45.29881674	46.39459643	55.24395156	56.18043601	61.28422698	58.82698101	Tjp2	tight junction protein 2 [Source:MGI Symbol;Acc:MGI:1341872]
ENSMUSG00000024815	0.135127597	0.155606375	0.039526469	0.126098854	0.162255068	0.073517319	0	0	0	0	0	0	Trpd52l3	tumor protein D52-like 3 [Source:MGI Symbol;Acc:MGI:1913995]
ENSMUSG00000024816	59.36290186	64.13679853	58.36004302	61.54633194	59.62217432	57.38383351	31.68424198	34.01430874	37.13735386	41.65324609	45.46480618	48.5410515	Frmd8	FERM domain containing 8 [Source:MGI Symbol;Acc:MGI:1914707]
ENSMUSG00000024817	47.8707608	61.91522565	41.04693634	69.0920776	71.67429597	82.09748843	36.01812557	32.16200987	36.61636711	48.96836305	54.34140525	51.91856119	Uhrf2	ubiquitin-like, containing PHD and RING finger domains 2 [Source:MGI Symbol;Acc:MGI:1923718]
ENSMUSG00000024818	9.295934628	8.365086402	9.346143638	10.20719068	11.43366913	10.11203612	0.54665381	0.937437789	0.777150418	1.17007401	0.949865391	1.455266091	Slc25a45	solute carrier family 25, member 45 [Source:MGI Symbol;Acc:MGI:2147731]
ENSMUSG00000024824	19.96951672	21.39755357	20.07792465	29.58237493	28.79031304	30.11390242	30.26019202	33.8877707	36.67352065	22.98865005	23.66671793	23.96698692	Rad9a	RAD9 checkpoint clamp component A [Source:MGI Symbol;Acc:MGI:1328356]
ENSMUSG00000024826	80.34572018	80.9010777	76.21152821	92.82904541	87.47460052	80.68913199	85.54235546	80.39037127	87.53010436	59.44307693	59.88710816	61.20429483	Dpf2	D4, zinc and double PHD fingers family 2 [Source:MGI Symbol;Acc:MGI:109529]
ENSMUSG00000024827	1.177250009	1.220097512	0.828616197	1.098590365	0.850361665	1.546790304	43.68310184	40.43915921	47.81518056	80.34560422	83.27056071	76.26580938	Gldc	glycine decarboxylase [Source:MGI Symbol;Acc:MGI:1341155]
ENSMUSG00000024829	56.91817827	47.23538989	71.52439524	56.97602006	52.4118416	41.68783394	106.1685253	123.6032479	103.8637336	78.08616254	74.19209921	73.75155193	Mrpl21	mitochondrial ribosomal protein L21 [Source:MGI Symbol;Acc:MGI:2660674]
ENSMUSG00000024830	21.63545092	17.20275437	22.73964746	20.04846182	18.72006422	16.64089358	16.63741919	17.47059392	16.86576944	10.76036806	12.48546011	10.71480913	Rps6kb2	ribosomal protein S6 kinase, polypeptide 2 [Source:MGI Symbol;Acc:MGI:1927343]
ENSMUSG00000024831	5.68736164	5.064366897	6.183990958	6.042021744	5.590429272	5.303808387	4.859418449	5.593802096	5.306196732	5.390743555	5.988310411	5.509422823	Ighmbp2	immunoglobulin mu binding protein 2 [Source:MGI Symbol;Acc:MGI:99954]
ENSMUSG00000024833	35.69157901	33.44592305	33.85810768	47.95251872	45.15035897	42.79530772	99.04137377	96.07210289	101.6632308	50.5771353	55.67687374	52.23719059	Pola2	polymerase (DNA directed), alpha 2 [Source:MGI Symbol;Acc:MGI:99690]
ENSMUSG00000024835	147.667997	161.425466	139.3140868	168.8213832	177.2791603	159.6101676	92.97683234	98.11987584	101.9700095	87.06187781	88.04043291	89.65629478	Coro1b	coronin, actin binding protein 1B [Source:MGI Symbol;Acc:MGI:1345963]
ENSMUSG00000024837	21.6611444	21.40686709	22.2038654	27.74104335	27.36071408	25.55193664	7.296676192	7.382130049	7.532521981	5.877119632	4.935658818	6.012264302	Dmrt1	doublesex and mab-3 related transcription factor 1 [Source:MGI Symbol;Acc:MGI:1354733]
ENSMUSG00000024841	34.87079472	24.56300775	34.194404	35.62958173	31.57908174	25.18531967	49.24981291	50.80656595	43.65886526	41.32895845	35.23245094	37.1551379	Eif1ad	eukaryotic translation initiation factor 1A domain containing [Source:MGI Symbol;Acc:MGI:1917110]
ENSMUSG00000024842	0.166938847	0.264328163	0.170910767	0.097365368	0.131546998	0.254308599	0.298078402	0.289409247	0.330182142	0.895937142	0.781648592	0.573288092	Cabp4	calcium binding protein 4 [Source:MGI Symbol;Acc:MGI:1920910]
ENSMUSG00000024843	36.5706664	34.70372892	35.35804579	49.97662112	49.65707535	50.01686712	46.14428595	46.14089836	49.34037854	38.63607839	39.51867459	39.51650651	Chka	choline kinase alpha [Source:MGI Symbol;Acc:MGI:107760]
ENSMUSG00000024844	157.9352276	116.447031	181.7533343	148.485172	129.2109602	98.8845451	342.5251233	370.9521712	285.9344457	163.6839485	165.2739803	165.0535222	Banf1	BAF nuclear assembly factor 1 [Source:MGI Symbol;Acc:MGI:1346330]
ENSMUSG00000024845	32.94191518	24.40498531	14.69697548	1.480637462	35.20612676	15.68203797	6.981452847	17.62666749	15.08435997	10.23867762	4.716884391	11.04816378	Tmem134	transmembrane protein 134 [Source:MGI Symbol;Acc:MGI:1914240]
ENSMUSG00000024846	7.732105316	5.217382793	9.332633081	9.671266414	8.009796631	5.641429782	0.484424389	0.671908066	0.505033437	0.218405862	0.112915749	0.120217228	Cst6	cystatin E/M [Source:MGI Symbol;Acc:MGI:1920970]
ENSMUSG00000024847	88.24672058	84.72588931	96.06304941	89.24697936	73.10403641	61.75359359	60.60358658	62.74568167	56.91463391	39.02660203	39.91804443	37.89032896	Aip	aryl-hydrocarbon receptor-interacting protein [Source:MGI Symbol;Acc:MGI:109622]
ENSMUSG00000024851	39.90779848	44.98133656	39.42229643	42.59674416	43.09655916	43.02723299	9.397118574	9.562651758	10.97843982	11.89886115	12.94426723	11.76565344	Pitpnm1	phosphatidylinositol transfer protein, membrane-associated 1 [Source:MGI Symbol;Acc:MGI:1197524]
ENSMUSG00000024853	194.2200589	198.4025309	186.600969	219.3252551	219.3567304	209.2443147	284.1483876	278.9735672	280.4542371	216.8720141	228.4988562	217.2316709	Sf3b2	splicing factor 3b, subunit 2 [Source:MGI Symbol;Acc:MGI:2441856]
ENSMUSG00000024854	0	0	0	0	0	0	0	0	0	0	0	0	Pold4	polymerase (DNA-directed), delta 4 [Source:MGI Symbol;Acc:MGI:1916995]
ENSMUSG00000024855	23.76534388	25.6904308	21.05151779	25.647224	27.60507935	28.01402493	18.17756499	17.81809251	19.24277296	19.77492078	18.43981772	18.52056225	Pacs1	phosphofurin acidic cluster sorting protein 1 [Source:MGI Symbol;Acc:MGI:1277113]
ENSMUSG00000024856	67.58595198	47.84301324	66.37639175	49.4386609	41.76069752	33.94308164	42.24216426	51.22441968	39.99989057	24.71183081	28.10721594	27.76713355	Cdk2ap2	CDK2-associated protein 2 [Source:MGI Symbol;Acc:MGI:1098779]
ENSMUSG00000024857	0	0	0	0	0	0	0.286678015	0.381724009	0	0	0.053458023	0	Cabp2	calcium binding protein 2 [Source:MGI Symbol;Acc:MGI:1352749]
ENSMUSG00000024858	67.76551322	66.46723148	68.54553911	60.3114991	56.89170966	53.36282847	68.06842564	68.65580193	68.53215132	53.0053572	57.32633511	55.75843756	Grk2	G protein-coupled receptor kinase 2 [Source:MGI Symbol;Acc:MGI:87940]
ENSMUSG00000024862	28.66874484	28.57277493	28.02616625	28.2447518	27.8287054	25.19889323	15.5154334	14.80343414	15.2309568	15.99883036	14.82514539	14.77200376	Klc2	kinesin light chain 2 [Source:MGI Symbol;Acc:MGI:107953]
ENSMUSG00000024863	0.86926238	0.136500043	0.283164143	0.294975044	0.124540822	0.060191039	0.211652089	0.078284385	0.110328241	0.388767337	0.131558771	0.140065766	Mbl2	mannose-binding lectin (protein C) 2 [Source:MGI Symbol;Acc:MGI:96924]
ENSMUSG00000024866	7.156597666	7.268627292	8.510093788	6.636938485	5.697742602	3.927465283	7.222627522	6.120860362	6.247336653	4.612194317	4.329105817	4.648432596	Acy3	aspartoacylase (aminoacylase) 3 [Source:MGI Symbol;Acc:MGI:1918920]
ENSMUSG00000024867	0.237890741	0.376672199	0.091331548	0.166496798	0.187456745	0.158547439	1.061917182	0.972116775	0.830321041	4.175957651	3.910895817	3.267781743	Pip5k1b	phosphatidylinositol-4-phosphate 5-kinase, type 1 beta [Source:MGI Symbol;Acc:MGI:107930]
ENSMUSG00000024868	0.088645422	0.020415952	0	0	0	0	0.031656246	0.035126368	0.066006018	2.584894322	2.789200439	3.220949922	Dkk1	dickkopf WNT signaling pathway inhibitor 1 [Source:MGI Symbol;Acc:MGI:1329040]
ENSMUSG00000024869	0.132259696	0.291915691	0.259529138	0.329126868	0.416879985	0.394564285	0.334555594	0.272962652	0.420598159	0.226747753	0.284406542	0.185585346	Gm49405	predicted gene, 49405 [Source:MGI Symbol;Acc:MGI:6155029]
ENSMUSG00000024870	214.8937196	183.4000376	236.5155976	192.7212202	160.2325657	140.9788075	135.969339	138.8009299	129.754621	112.9425294	109.5209661	112.929238	Rab1b	RAB1B, member RAS oncogene family [Source:MGI Symbol;Acc:MGI:1923558]
ENSMUSG00000024871	2.584394355	1.198155214	3.951644909	2.098504692	2.080570033	1.022590347	1.797887086	1.196981683	1.374541862	0.300218146	0.642580449	0.624641964	Doc2g	double C2, gamma [Source:MGI Symbol;Acc:MGI:1926250]
ENSMUSG00000024873	15.21058904	11.64863289	14.73484946	11.62611502	9.729361512	6.741760511	7.868902692	10.05778429	7.303691419	3.093674047	2.476534173	2.90034193	Cnih2	cornichon family AMPA receptor auxiliary protein 2 [Source:MGI Symbol;Acc:MGI:1277225]
ENSMUSG00000024875	57.11495421	50.94790271	60.96035752	53.44983174	49.84397846	43.87340674	30.17535574	30.71627407	27.61620702	21.21726086	21.02514609	22.02596752	Yif1a	Yip1 interacting factor homolog A (S. cerevisiae) [Source:MGI Symbol;Acc:MGI:1915340]
ENSMUSG00000024878	18.84562749	19.90683065	15.52225281	24.02156468	25.15984347	23.27639007	40.77625015	40.66066043	40.66461598	35.06587496	39.15873543	33.90521712	Cbwd1	COBW domain containing 1 [Source:MGI Symbol;Acc:MGI:2385089]
ENSMUSG00000024883	41.20591836	57.44276259	43.07871981	81.93380008	90.08361041	97.35634491	5.730392596	4.199784258	6.785491602	20.41350703	19.22765967	20.96256363	Rin1	Ras and Rab interactor 1 [Source:MGI Symbol;Acc:MGI:2385695]
ENSMUSG00000024885	12.50790259	11.02767418	13.6058426	11.21622507	10.0272803	8.435402738	3.140654231	3.03317917	3.60775854	3.583663788	3.958564587	3.290803281	Aldh3b1	aldehyde dehydrogenase 3 family, member B1 [Source:MGI Symbol;Acc:MGI:1914939]
ENSMUSG00000024887	7.149307934	9.088150985	5.866390902	6.807772136	8.751795291	10.58790802	0.49735631	0.551875957	0.753082072	3.879643959	4.063073421	3.597000257	Asah2	N-acylsphingosine amidohydrolase 2 [Source:MGI Symbol;Acc:MGI:1859310]
ENSMUSG00000024889	43.57143405	32.51198501	43.17213163	40.95570294	37.12691549	28.98312445	40.91929251	40.65367388	37.43816583	26.34665206	28.02077325	31.03561328	Rce1	Ras converting CAAX endopeptidase 1 [Source:MGI Symbol;Acc:MGI:1336895]
ENSMUSG00000024891	4.940410084	6.197662313	5.510063367	6.902586312	5.770657698	4.982323856	12.98564984	13.67089407	13.71792618	11.4303582	10.13164983	11.47166921	Slc29a2	solute carrier family 29 (nucleoside transporters), member 2 [Source:MGI Symbol;Acc:MGI:1345278]
ENSMUSG00000024892	31.50377224	34.7593587	32.07623645	13.84185216	13.99413271	14.60831975	14.6130877	15.80797769	17.71017756	25.7231132	27.59159302	26.13732764	Pcx	pyruvate carboxylase [Source:MGI Symbol;Acc:MGI:97520]
ENSMUSG00000024896	35.2563906	32.66597697	32.20457949	32.64901018	29.95856082	28.34812725	30.22844073	30.7489021	29.39219781	24.07965076	26.42897649	23.8140533	Minpp1	multiple inositol polyphosphate histidine phosphatase 1 [Source:MGI Symbol;Acc:MGI:1336159]
ENSMUSG00000024897	0.957750699	0.870177322	0.88158475	0.647768991	0.618533251	0.582262568	0.062756551	0.078340337	0.04906982	0.290801461	0.292561776	0.171313846	Apba1	amyloid beta (A4) precursor protein binding, family A, member 1 [Source:MGI Symbol;Acc:MGI:1860297]
ENSMUSG00000024899	7.10664057	8.966863956	7.460461727	8.756048715	8.983330356	9.811061662	0.23544588	0.39875793	0.322977028	1.862320953	1.975705067	1.59912181	Papss2	3'-phosphoadenosine 5'-phosphosulfate synthase 2 [Source:MGI Symbol;Acc:MGI:1330223]
ENSMUSG00000024900	134.0279249	177.0725596	120.3697972	155.6218573	185.7817515	218.5008524	60.63317507	55.18319202	69.76129231	70.26654401	70.03651192	67.73743678	Cpt1a	carnitine palmitoyltransferase 1a, liver [Source:MGI Symbol;Acc:MGI:1098296]
ENSMUSG00000024901	4.461982422	5.533448445	5.718167357	7.104748798	6.294414157	6.678389992	3.342844444	2.472855559	3.07846995	2.121164209	3.818042564	3.207699017	Peli3	pellino 3 [Source:MGI Symbol;Acc:MGI:1924963]
ENSMUSG00000024902	42.83001694	36.65932798	57.82873804	41.15361059	36.15831366	28.99472859	80.72789444	91.46468796	67.24119536	58.0870046	52.00716052	54.70172213	Mrpl11	mitochondrial ribosomal protein L11 [Source:MGI Symbol;Acc:MGI:2137215]
ENSMUSG00000024903	0	0.031479997	0	0	0	0.020822122	0	0.027081225	0.025444188	0	0	0	Lao1	L-amino acid oxidase 1 [Source:MGI Symbol;Acc:MGI:2140628]
ENSMUSG00000024905	1.691896851	0.98617968	1.240306074	0.760187202	0.614481997	1.113680313	1.15617522	0.88976284	0.797094763	0.709972195	0.886728471	0.61086692	Tesmin	testis expressed metallothionein like [Source:MGI Symbol;Acc:MGI:1340029]
ENSMUSG00000024906	30.06374756	25.46808126	26.83406659	28.05499524	23.19456632	22.92958533	10.53541707	11.13361694	12.14176702	13.93034604	14.5673113	15.04684579	Mus81	MUS81 structure-specific endonuclease subunit [Source:MGI Symbol;Acc:MGI:1918961]
ENSMUSG00000024907	1.237805814	0.339380244	0.482764386	0.605052406	0.557362829	0.404063268	0.526230878	0.350349433	0.054861851	0.896295107	1.275669538	0.888026548	Gal	galanin and GMAP prepropeptide [Source:MGI Symbol;Acc:MGI:95637]
ENSMUSG00000024908	34.53262274	36.99473971	34.26286126	38.23925423	43.57918976	51.006415	66.36079521	64.93081455	76.05430088	88.64946008	93.93862949	88.38989732	Ppp6r3	protein phosphatase 6, regulatory subunit 3 [Source:MGI Symbol;Acc:MGI:1921807]
ENSMUSG00000024909	334.2819735	294.0885595	366.7169287	286.2416367	249.9797669	210.1298274	11.11511761	13.76605245	11.48951068	45.99627457	47.29589049	43.82158391	Efemp2	epidermal growth factor-containing fibulin-like extracellular matrix protein 2 [Source:MGI Symbol;Acc:MGI:1891209]
ENSMUSG00000024910	0.487574933	0.748623466	0.54077361	0.075832819	0.08537926	0.185688502	1.41471134	1.04652695	0.907629496	1.744497059	1.691070608	1.944454063	Ctsw	cathepsin W [Source:MGI Symbol;Acc:MGI:1338045]
ENSMUSG00000024911	69.84277358	55.92404272	80.62847983	67.81394842	56.6759318	45.39321471	35.40054983	39.12554999	29.01371014	27.70712178	24.50972318	27.48630904	Fibp	fibroblast growth factor (acidic) intracellular binding protein [Source:MGI Symbol;Acc:MGI:1926233]
ENSMUSG00000024912	10.35343305	16.13367231	10.59976881	19.98809989	24.79970664	23.65806788	0.72080852	0.611629161	0.839882869	6.414661062	5.297418669	6.650109206	Fosl1	fos-like antigen 1 [Source:MGI Symbol;Acc:MGI:107179]
ENSMUSG00000024913	14.82170224	17.8245102	13.50624772	12.04113861	12.56297935	13.71811613	19.45768667	15.14293653	20.53008215	17.30077967	18.55010815	18.57736989	Lrp5	low density lipoprotein receptor-related protein 5 [Source:MGI Symbol;Acc:MGI:1278315]
ENSMUSG00000024914	219.5499667	167.9354262	306.5607936	208.5821505	187.2006422	124.6755638	138.0672156	152.4924692	90.20032974	84.86079141	70.25794268	71.33440941	Drap1	Dr1 associated protein 1 (negative cofactor 2 alpha) [Source:MGI Symbol;Acc:MGI:1913806]
ENSMUSG00000024921	37.29723433	41.46048951	27.71319838	30.58137144	36.06783868	38.25874404	1.338222375	1.426684793	1.887562469	11.67257997	12.25700452	11.92955625	Smarca2	SWI/SNF related, matrix associated, actin dependent regulator of chromatin, subfamily a, member 2 [Source:MGI Symbol;Acc:MGI:99

ENSMUSG00000024922	0.11629321	0.187484654	0.023812027	0.043409165	0.171058502	0.141725495	0.31147179	0.207368989	0.41131561	0.686542103	0.696977036	0.803882856	Ovol1	ovo like zinc finger 1 [Source:MGI Symbol;Acc:MGI:1330290]
ENSMUSG00000024924	12.25250662	14.76010555	10.04650933	15.57522795	18.20206796	21.98231877	1.107410912	1.146883795	1.847238251	3.78469112	4.462768918	4.543702929	Vldlr	very low density lipoprotein receptor [Source:MGI Symbol;Acc:MGI:98935]
ENSMUSG00000024925	75.51811414	55.15076522	102.2139892	63.95459731	55.49484876	43.1762098	106.7821596	115.0897888	78.47312009	49.97857662	49.2092724	49.2964507	Rnaseh2c	ribonuclease H2, subunit C [Source:MGI Symbol;Acc:MGI:1915459]
ENSMUSG00000024926	19.02853446	19.97773299	17.55069729	26.61967614	23.70284739	25.64469184	30.48700769	24.38673996	29.42256391	23.52212766	23.14540718	22.15050798	Kat5	K(lysine) acetyltransferase 5 [Source:MGI Symbol;Acc:MGI:1932051]
ENSMUSG00000024927	96.35559733	88.1737692	91.96809939	98.93553637	86.78514883	81.42463652	21.47453318	20.78883556	20.3528475	30.0963278	30.41498606	32.60050786	Rela	v-rel reticuloendotheliosis viral oncogene homolog A (avian) [Source:MGI Symbol;Acc:MGI:103290]
ENSMUSG00000024935	0.058462619	0.29173175	0.039902401	0.018185452	0.081899127	0.089059839	1.774599237	1.872602911	1.650576444	2.004581582	1.930347909	2.504198904	Slc1a1	solute carrier family 1 (neuronal/epithelial high affinity glutamate transporter, system Xag), member 1 [Source:MGI Symbol;Acc:MGI:[illegible]
ENSMUSG00000024936	0.619672192	0.490589264	0.436160893	0.216850465	0.28484089	0.79648835	0.069153653	0.115101299	0.360478412	0.554282144	0.128953647	0.240261326	Kcnk7	potassium channel, subfamily K, member 7 [Source:MGI Symbol;Acc:MGI:1341841]
ENSMUSG00000024937	47.2098407	42.91747558	52.53315645	43.37812457	40.98091784	38.75915532	11.93156634	12.50483248	12.87976013	16.99285351	18.95260408	18.23443627	Ehbp1l1	EH domain binding protein 1-like 1 [Source:MGI Symbol;Acc:MGI:3612340]
ENSMUSG00000024939	0	0	0.037269247	0.067941587	0.038247308	0	0	0	0	0	0	0	Fam89b	family with sequence similarity 89, member B [Source:MGI Symbol;Acc:MGI:106595]
ENSMUSG00000024940	123.4420286	121.3589644	113.207642	114.1246586	111.0443518	106.9355357	5.907007711	6.361747119	7.84882869	21.44882146	25.44977986	23.4162862	Ltbp3	latent transforming growth factor beta binding protein 3 [Source:MGI Symbol;Acc:MGI:1101355]
ENSMUSG00000024941	138.1939495	130.2300563	140.5867324	120.5964317	117.633499	108.7009643	58.51678448	53.59812348	56.46218229	47.22189947	52.34540819	50.79005849	Scyl1	SCY1-like 1 (S. cerevisiae) [Source:MGI Symbol;Acc:MGI:1931787]
ENSMUSG00000024942	53.87800224	54.41327176	55.7274478	67.07847862	59.54739846	52.96120881	39.07474114	38.07560384	37.81327865	28.71249516	30.21362195	30.85724005	Capn1	calpain 1 [Source:MGI Symbol;Acc:MGI:88263]
ENSMUSG00000024943	33.3051672	39.78976255	27.72812631	46.15042582	49.67043808	63.44823389	45.19981531	42.47822572	51.17969493	50.16764794	48.23649334	50.03499312	Smc5	structural maintenance of chromosomes 5 [Source:MGI Symbol;Acc:MGI:2385088]
ENSMUSG00000024944	59.69348946	44.79118912	73.80500013	47.54681661	40.83100498	34.77687745	29.84773185	31.15746487	24.9512571	18.7552471	18.47695957	17.25059974	Arl2	ADP-ribosylation factor-like 2 [Source:MGI Symbol;Acc:MGI:1928393]
ENSMUSG00000024947	16.17871142	19.63482139	13.43887313	19.68055794	20.25332662	17.56687506	18.39815411	15.64084438	17.45075029	16.64732839	18.45285425	18.3648069	Men1	multiple endocrine neoplasia 1 [Source:MGI Symbol;Acc:MGI:1316736]
ENSMUSG00000024948	14.90339808	17.39575313	13.18005329	21.0508125	19.71009567	23.78545837	12.78769401	13.95967789	16.24635392	12.81203193	12.96068877	12.43687727	Map4k2	mitogen-activated protein kinase kinase kinase kinase 2 [Source:MGI Symbol;Acc:MGI:1346883]
ENSMUSG00000024949	70.31272438	84.74169181	54.29350782	97.46147383	100.4696994	105.6100854	159.2507967	141.1191306	192.5813647	155.897012	154.8766105	161.7051005	Sf1	splicing factor 1 [Source:MGI Symbol;Acc:MGI:1095403]
ENSMUSG00000024952	63.30335875	69.0546056	53.87778902	67.79235623	71.31829246	70.00182251	12.25643793	12.78145803	13.13280892	17.33948263	17.69681661	17.88360006	Rps6ka4	ribosomal protein S6 kinase, polypeptide 4 [Source:MGI Symbol;Acc:MGI:1930076]
ENSMUSG00000024953	353.9895894	249.0294561	438.9445109	223.5809572	182.137792	131.2265264	90.20801553	101.5385865	66.19068724	97.69433056	104.2787837	100.8150525	Prdx5	peroxiredoxin 5 [Source:MGI Symbol;Acc:MGI:1859821]
ENSMUSG00000024955	32.16547642	30.15433753	35.86419443	32.89934634	31.09871581	26.96692723	20.95355677	20.18989196	17.64759948	16.98195158	18.6654244	17.06817332	Esrra	estrogen related receptor, alpha [Source:MGI Symbol;Acc:MGI:1346831]
ENSMUSG00000024957	0.82747092	0.9528753	0.988351767	0.804356527	0.941839966	1.129233083	1.046558524	1.092970454	0.930629322	0.801832633	1.693265748	1.374984544	Kcnk4	potassium channel, subfamily K, member 4 [Source:MGI Symbol;Acc:MGI:1298234]
ENSMUSG00000024958	48.56771706	37.21499893	49.97392537	40.21059516	34.59754662	29.1143327	11.83046359	10.8838402	10.36834364	9.827297246	12.07482146	9.926488703	Gpr137	G protein-coupled receptor 137 [Source:MGI Symbol;Acc:MGI:2147529]
ENSMUSG00000024959	59.84672926	45.17306094	70.30470793	47.65422902	43.05117016	32.94038622	17.51093348	21.24801139	13.66143646	11.80037142	12.03592465	13.04648617	Bad	BCL2-associated agonist of cell death [Source:MGI Symbol;Acc:MGI:1096330]
ENSMUSG00000024960	75.42321519	78.33028359	73.40357745	91.70372834	90.18333689	88.06138802	109.0550129	105.2203408	120.6296978	68.12634873	74.44734725	72.54872578	Plcb3	phospholipase C, beta 3 [Source:MGI Symbol;Acc:MGI:104778]
ENSMUSG00000024962	89.17498202	71.04148979	98.77554237	77.27258312	62.85173037	49.94136478	39.28972652	42.1404602	33.70596293	19.36351103	21.31956874	22.57973238	Vegfb	vascular endothelial growth factor B [Source:MGI Symbol;Acc:MGI:106199]
ENSMUSG00000024963	25.73091261	22.04563571	28.92701408	24.12655321	23.4772812	20.20830171	30.8857625	35.97889863	34.20506933	14.37104324	15.3208094	14.4566806	Dnajc4	DnaJ heat shock protein family (Hsp40) member C4 [Source:MGI Symbol;Acc:MGI:1927346]
ENSMUSG00000024965	3.49572147	3.287845358	4.215974604	0.871045244	0.999928777	0.599439765	1.192804912	0.906547165	0.868782104	3.257807447	4.067675778	3.779228751	Fermt3	fermitin family member 3 [Source:MGI Symbol;Acc:MGI:2147790]
ENSMUSG00000024966	153.5186228	156.8049425	148.4151238	154.823277	153.0177189	139.6938214	338.3896704	334.6573906	335.9721041	280.827045	275.5418736	274.2928054	Stip1	stress-induced phosphoprotein 1 [Source:MGI Symbol;Acc:MGI:109130]
ENSMUSG00000024968	35.70201558	31.60455591	36.10498614	39.88217055	36.25829316	30.36025831	99.64501674	105.6717805	103.5324687	78.90033106	83.27423549	83.94392127	Rcor2	REST corepressor 2 [Source:MGI Symbol;Acc:MGI:1859854]
ENSMUSG00000024969	43.31022197	42.3235446	44.57668175	47.82566021	48.46439366	51.59957416	38.57491596	36.96777387	40.45441079	48.29323399	48.4167406	50.52609871	Mark2	MAP/microtubule affinity regulating kinase 2 [Source:MGI Symbol;Acc:MGI:99638]
ENSMUSG00000024970	28.26521585	24.42814228	28.84967485	24.74481479	20.78197179	19.28012007	34.80626352	37.03138268	31.19084486	25.43641748	26.70158805	25.86228979	Spindoc	spindlin interactor and repressor of chromatin binding [Source:MGI Symbol;Acc:MGI:2147611]
ENSMUSG00000024972	0.033022631	0.095068142	0.033808327	0.046224265	0.138782251	0.113187331	0.442227791	0.310779396	0.461041652	0.51691436	0.590992189	0.512145688	Lgals12	lectin, galactose binding, soluble 12 [Source:MGI Symbol;Acc:MGI:1929094]
ENSMUSG00000024973	0	0	0	0	0	0	0	0	0.128913578	0.123888304	0	0	Plaat5	phospholipase A and acyltransferase 5 [Source:MGI Symbol;Acc:MGI:1913977]
ENSMUSG00000024974	61.66844591	67.30928718	48.9054642	75.38285021	90.68707529	102.324871	132.6710414	124.2439339	153.7080845	128.9711416	126.8767284	130.1004984	Smc3	structural maintenance of chromosomes 3 [Source:MGI Symbol;Acc:MGI:1339795]
ENSMUSG00000024975	30.02183092	31.47281937	26.89052667	26.73410707	26.83047428	29.37918723	21.7725883	21.68780916	20.42898087	24.42168029	24.99014569	23.47595761	Pdcd4	programmed cell death 4 [Source:MGI Symbol;Acc:MGI:107490]
ENSMUSG00000024976	30.66647268	37.6616278	24.26870437	38.29152221	44.79816223	54.48020164	22.0536889	22.49458797	28.11918754	36.47045201	35.23918394	34.36574273	Shoc2	Shoc2, leucine rich repeat scaffold protein [Source:MGI Symbol;Acc:MGI:1927197]
ENSMUSG00000024978	8.033323282	10.92748944	7.491966705	9.464413679	10.11516341	13.54748753	7.36247349	6.627647848	7.885991038	8.465556197	8.598975647	7.942501152	Gpam	glycerol-3-phosphate acyltransferase, mitochondrial [Source:MGI Symbol;Acc:MGI:109162]
ENSMUSG00000024979	0	0	0.032178415	0	0	0	0	0	0	0	0	0	Tectb	tectorin beta [Source:MGI Symbol;Acc:MGI:109574]
ENSMUSG00000024981	45.08122303	42.0034426	47.73000687	43.17419819	37.82567554	34.36761981	6.449466326	4.927391569	6.319682626	18.21999142	17.05409025	18.15686012	Acsl5	acyl-CoA synthetase long-chain family member 5 [Source:MGI Symbol;Acc:MGI:1919129]
ENSMUSG00000024982	26.24068921	31.83492196	29.26165013	33.84958986	31.43500685	30.26077968	22.09351711	23.521517	23.51071952	25.00720717	25.05325487	26.59424337	Zdhhc6	zinc finger, DHHC domain containing 6 [Source:MGI Symbol;Acc:MGI:1914230]
ENSMUSG00000024983	8.728441271	10.16903688	9.634248062	9.083849383	10.37068793	11.40078848	5.600897899	5.083351885	6.061321665	7.593901447	8.443360107	8.641846863	Vti1a	vesicle transport through interaction with t-SNAREs 1A [Source:MGI Symbol;Acc:MGI:1855699]
ENSMUSG00000024985	9.902728538	10.50260068	10.49666057	9.759792699	12.45932601	12.21577133	4.319373544	4.731673046	5.882817951	5.874479078	5.312550908	6.20343904	Tcf7l2	transcription factor 7 like 2, T cell specific, HMG box [Source:MGI Symbol;Acc:MGI:1202879]
ENSMUSG00000024986	0.523856485	0.374429589	0.295900925	0.42142609	0.664270001	0.577879457	0.967628566	0.966329041	1.143300715	0.953312349	1.187881261	1.056579223	Hhex	hematopoietically expressed homeobox [Source:MGI Symbol;Acc:MGI:96086]
ENSMUSG00000024987	2.037267179	1.941532041	2.625154502	2.687824639	3.542855969	2.702184559	0.219513452	0.173983052	0.326931849	9.739811898	12.07540373	10.95737343	Cyp26a1	cytochrome P450, family 26, subfamily a, polypeptide 1 [Source:MGI Symbol;Acc:MGI:1096359]
ENSMUSG00000024989	10.94746797	14.83404915	9.080114699	17.91640116	19.80431354	20.15657128	46.31460885	44.9701674	56.86558148	49.34779428	47.80722117	50.55208599	Cep55	centrosomal protein 55 [Source:MGI Symbol;Acc:MGI:1921357]
ENSMUSG00000024990	5.127747254	5.840681246	6.847500031	3.120736619	3.630719867	1.995309547	2.537768857	3.644178689	2.17883985	3.240567117	2.876484818	3.852806432	Rbp4	retinol binding protein 4, plasma [Source:MGI Symbol;Acc:MGI:97879]
ENSMUSG00000024991	158.5824091	214.7215396	125.1091344	212.9880129	254.3039469	304.6780314	215.2950686	187.4529681	252.9740411	341.5664859	332.76886	330.7695477	Eif3a	eukaryotic translation initiation factor 3, subunit A [Source:MGI Symbol;Acc:MGI:95301]
ENSMUSG00000024992	0	0	0	0.043947623	0	0	0	0	0	0	0	0	Pde6c	phosphodiesterase 6C, cGMP specific, cone, alpha prime [Source:MGI Symbol;Acc:MGI:105956]
ENSMUSG00000024993	25.33868934	33.27856932	19.13581909	33.06756998	39.3052283	42.47699507	21.23122187	20.66540774	24.93040266	27.76507464	24.91206077	27.13919073	Fam45a	family with sequence similarity 45, member A [Source:MGI Symbol;Acc:MGI:1915144]
ENSMUSG00000024997	53.74194082	54.31002389	47.39044507	58.84467726	58.33035392	50.02583935	54.52933954	55.97887036	50.40015009	49.16460116	45.53444454	46.51801567	Prdx3	peroxiredoxin 3 [Source:MGI Symbol;Acc:MGI:88034]
ENSMUSG00000024998	0.040577601	0.046727193	0.03323444	0.106025781	0.127899807	0.055633004	1.760620434	1.519480425	1.956380657	0.704136691	0.898461795	0.798331275	Plce1	phospholipase C, epsilon 1 [Source:MGI Symbol;Acc:MGI:1921305]
ENSMUSG00000024999	6.123742547	7.911476596	5.427609403	8.703117399	9.719163236	11.89984556	13.81273355	12.46513977	16.92799188	27.27158392	25.18078548	27.79665343	Noc3l	NOC3 like DNA replication regulator [Source:MGI Symbol;Acc:MGI:1932610]
ENSMUSG00000025001	40.73071734	52.29001649	34.13839351	65.27173733	76.93748774	97.11361105	204.3093892	186.3017819	263.973047	304.0622747	294.1628826	308.8934975	Hells	helicase, lymphoid specific [Source:MGI Symbol;Acc:MGI:106209]
ENSMUSG00000025002	13.26442106	14.38053611	10.84193225	18.01371562	20.34940846	19.68084046	0.019255566	0.02136634	0.060224288	0.115753282	0.125673247	0.133799666	Cyp2c55	cytochrome P450, family 2, subfamily c, polypeptide 55 [Source:MGI Symbol;Acc:MGI:1919332]
ENSMUSG00000025003	0	0.027269959	0	0	0	0	0	0	0	0	0	0	Cyp2c39	cytochrome P450, family 2, subfamily c, polypeptide 39 [Source:MGI Symbol;Acc:MGI:1306818]
ENSMUSG00000025004	0	0	0.079301981	0	0	0	0.034576826	0.076734199	0	0	0.064476824	0	Cyp2c40	cytochrome P450, family 2, subfamily c, polypeptide 40 [Source:MGI Symbol;Acc:MGI:1306815]
ENSMUSG00000025006	0.543517741	1.642957623	0.324595528	0.549468644	1.046929478	1.638701032	4.913057652	2.647289489	5.227468199	7.798877396	9.218132689	9.733925671	Sorbs1	sorbin and SH3 domain containing 1 [Source:MGI Symbol;Acc:MGI:700014]
ENSMUSG00000025007	71.30601709	79.56121411	64.34732481	98.96907342	104.0158869	101.1420488	36.87930096	32.57585109	39.47934652	41.77352448	45.59614433	40.97478783	Aldh18a1	aldehyde dehydrogenase 18 family, member A1 [Source:MGI Symbol;Acc:MGI:1888908]
ENSMUSG00000025008	12.87615351	17.12784386	10.61233631	14.50966326	16.818402	15.99635386	5.880316433	6.097047224	6.004860235	7.219511885	7.257748293	6.291690046	Tctn3	tectonic family member 3 [Source:MGI Symbol;Acc:MGI:1914840]
ENSMUSG00000025010	0.862612787	1.921334423	0.906377081	1.133321585	1.645673419	2.048916616	1.986101661	2.338743424	2.672760574	3.086347079	3.070557776	3.62116742	Ccnj	cyclin J [Source:MGI Symbol;Acc:MGI:2443297]
ENSMUSG00000025013	0.01078949	0.012424653	0.022092402	0.050342876	0.079352615	0	0	0	0	0.048254722	0.035924649	0.038247648	Tll2	tolloid-like 2 [Source:MGI Symbol;Acc:MGI:1346044]
ENSMUSG00000025014	0.04994601	0.057515398	0.051134359	0	0.052476284	0	0	0	0	0	0	0.066395075	Dntt	deoxynucleotidyltransferase, terminal [Source:MGI Symbol;Acc:MGI:98659]
ENSMUSG00000025016	53.2719109	61.80275107	47.72001255	59.42828772	67.78438785	77.76048035	19.10854271	18.88007989	22.32457585	35.91208082	36.01398592	36.94146297	Tm9sf3	transmembrane 9 superfamily member 3 [Source:MGI Symbol;Acc:MGI:1914262]
ENSMUSG00000025017	1.355511325	1.699077341	1.240389537	0.347019184	0.201654055	0.182737779	0.149932522	0.261435364	0.212136571	3.508660441	3.794372089	3.848372316	Pik3ap1	phosphoinositide-3-kinase adaptor protein 1 [Source:MGI Symbol;Acc:MGI:1933177]
ENSMUSG00000025019	2.44998536	3.66652709	1.949753812	3.304470168	3.76214913	5.507667368	4.454281143	3.773242397	5.206943628	7.434973679	7.884996792	8.491559689	Lcor	ligand dependent nuclear receptor corepressor [Source:MGI Symbol;Acc:MGI:2443930]
ENSMUSG00000025020	0.771527853	1.354528205	1.256045935	0.944232283	1.116254774	1.367992631	0.158085819	0.125296437	0.235444747	0.701426783	0.45271183	0.717373868	Slit1	slit guidance ligand 1 [Source:MGI Symbol;Acc:MGI:1315203]
ENSMUSG00000025024	47.57333496	50.2292072	39.12734157	54.25401326	56.27784486	56.53406166	70.29549428	67.18394926	78.66993824	69.90132713	72.27246067	70.61649016	Smndc1	survival motor neuron domain containing 1 [Source:MGI Symbol;Acc:MGI:1923729]

ENSMUSG00000025025	21.94634818	22.28680645	20.722253	27.16514357	29.84418056	25.75844011	17.98919699	18.85720641	19.05086489	18.67439383	16.92404158	17.69592511	Mxi1	MAX interactor 1, dimerization protein [Source:MGI Symbol;Acc:MGI:97245]
ENSMUSG00000025026	11.92277934	14.6957881	10.50514853	17.60008084	20.51071061	25.32705532	16.04579956	15.71098923	21.09825943	19.28006114	19.17753963	18.50167929	Add3	adducin 3 (gamma) [Source:MGI Symbol;Acc:MGI:1351615]
ENSMUSG00000025027	133.9767573	128.119665	131.1653843	135.4050343	123.5989685	111.068565	143.4405774	148.6370442	146.1690037	112.976506	117.0817078	113.6725826	Xpnpep1	X-prolyl aminopeptidase (aminopeptidase P) 1, soluble [Source:MGI Symbol;Acc:MGI:2180003]
ENSMUSG00000025034	71.26795701	77.89193801	70.46591753	100.5750935	91.27861166	89.9846668	28.52683594	30.09260799	31.08682258	30.47366329	29.72459209	32.41201231	Trim8	tripartite motif-containing 8 [Source:MGI Symbol;Acc:MGI:1933302]
ENSMUSG00000025035	110.5084034	83.83523647	129.2267876	101.9532879	87.93730069	69.2575862	69.07501765	76.44799057	54.72293633	38.98197761	40.20517411	34.5287445	Arl3	ADP-ribosylation factor-like 3 [Source:MGI Symbol;Acc:MGI:1929699]
ENSMUSG00000025036	2.996254331	2.882563271	2.912224561	3.096896704	4.064564541	3.495510354	5.299184365	5.579496151	5.577581261	5.546745264	5.367006462	5.982950229	Sfxn2	sideroflexin 2 [Source:MGI Symbol;Acc:MGI:2137678]
ENSMUSG00000025037	15.63358065	17.01592437	16.21697907	17.73999345	19.81050859	18.89992119	9.735081809	10.13732063	10.64901845	16.07128905	16.43433307	16.19756211	Maoa	monoamine oxidase A [Source:MGI Symbol;Acc:MGI:96915]
ENSMUSG00000025038	0.589662128	1.396854003	0.914161809	1.131969618	1.362975925	1.539891338	1.669557088	1.51877531	2.054204718	1.627525594	1.374335864	1.418412665	Efhc2	EF-hand domain (C-terminal) containing 2 [Source:MGI Symbol;Acc:MGI:1921655]
ENSMUSG00000025040	47.15262404	53.55991305	36.87440751	60.03778795	63.88864585	71.23724826	18.3278537	18.43034523	21.62398277	29.67549271	26.58611524	27.85854379	Fundc1	FUN14 domain containing 1 [Source:MGI Symbol;Acc:MGI:1919268]
ENSMUSG00000025041	21.19371568	22.4818344	18.33687345	15.0462242	17.08323362	17.63730591	22.11238778	23.34318432	23.03504246	20.74701212	20.358215	18.36921413	Nt5c2	5'-nucleotidase, cytosolic II [Source:MGI Symbol;Acc:MGI:2178563]
ENSMUSG00000025043	0	0	0	0	0	0	0	0	0	0	0	0	Dusp21	dual specificity phosphatase 21 [Source:MGI Symbol;Acc:MGI:1920797]
ENSMUSG00000025044	13.84115922	17.67190293	13.2106232	1.197236724	1.062810236	1.766489644	0.726886574	0.586594234	0.597063039	20.83293593	20.68095964	20.35649901	Msr1	macrophage scavenger receptor 1 [Source:MGI Symbol;Acc:MGI:98257]
ENSMUSG00000025047	20.59756925	21.96406917	21.8791335	22.85944914	23.79934829	23.40280426	42.70398691	41.2801138	47.00841117	62.21694359	64.26076992	61.3395569	Pdcd11	programmed cell death 11 [Source:MGI Symbol;Acc:MGI:1341788]
ENSMUSG00000025049	5.015590606	4.833357659	4.621432383	5.185464469	5.380621938	6.092348621	11.28878227	11.66326876	13.95577795	11.74709403	11.36032185	11.01877185	Taf5	TATA-box binding protein associated factor 5 [Source:MGI Symbol;Acc:MGI:2442144]
ENSMUSG00000025050	10.19695409	10.56152517	8.65103766	11.64202777	13.12756994	14.24642998	29.39600925	28.49874637	31.86611659	31.03155982	32.41853999	30.56017428	Pcgf6	polycomb group ring finger 6 [Source:MGI Symbol;Acc:MGI:1918291]
ENSMUSG00000025051	0	0	0	0	0	0	0	0	0	0	0	0	Samt4	spermatogenesis associated multipass transmembrane protein 4 [Source:MGI Symbol;Acc:MGI:1922435]
ENSMUSG00000025056	0.713968271	0	0	0.088835128	0.050009202	0.725088952	55.73128442	58.20974445	61.13697909	42.681622	41.93823307	43.78534853	Nr0b1	nuclear receptor subfamily 0, group B, member 1 [Source:MGI Symbol;Acc:MGI:1352460]
ENSMUSG00000025058	0.080234261	0.065995625	0.082143248	0.053480924	0.084298942	0.008730426	0	0	0.010668394	0.071767652	0.095409897	0.091421455	5430427O19	RIKEN cDNA 5430427O19 gene [Source:MGI Symbol;Acc:MGI:1918648]
ENSMUSG00000025059	4.060793633	4.859593501	3.065071442	5.260670301	6.659108328	7.483926992	11.00410698	10.58547143	13.62145457	12.94802422	14.02452045	13.32252481	Gk	glycerol kinase [Source:MGI Symbol;Acc:MGI:106594]
ENSMUSG00000025060	37.66633205	46.48370681	34.74273327	49.47888249	59.23373127	76.78688844	34.58173791	32.15047598	41.35794732	52.44090233	52.50020441	51.10972323	Slk	STE20-like kinase [Source:MGI Symbol;Acc:MGI:103241]
ENSMUSG00000025064	1.723843581	1.673707126	1.141967209	1.435179294	1.385015252	1.287274966	1.191977012	1.088248272	1.462910877	1.647756612	1.927302412	1.407892003	Col17a1	collagen, type XVII, alpha 1 [Source:MGI Symbol;Acc:MGI:88450]
ENSMUSG00000025066	190.9412311	185.3518918	182.7732034	221.6568815	216.4236291	195.3738129	162.8578051	179.1313617	168.1995373	156.3718668	154.62031	156.0460208	Sfr1	SWI5 dependent recombination repair 1 [Source:MGI Symbol;Acc:MGI:1915038]
ENSMUSG00000025068	550.8895153	497.7719175	601.3904531	332.9776916	300.8785782	254.5832026	91.32237377	96.79726963	86.87808686	363.8439191	360.6373907	351.3575982	Gsto1	glutathione S-transferase omega 1 [Source:MGI Symbol;Acc:MGI:1342273]
ENSMUSG00000025069	29.4771753	24.8926206	27.49598109	31.32811782	29.16388187	22.2963128	1.169624482	0.851705806	0.609692126	3.259209331	2.896702584	3.084012273	Gsto2	glutathione S-transferase omega 2 [Source:MGI Symbol;Acc:MGI:1915464]
ENSMUSG00000025075	0.211813934	0.592364111	0.061958158	0.225898609	0.031792066	0	0.297161318	0.119903932	0.112655841	0.108264322	0.050375352	0.187714721	Habp2	hyaluronic acid binding protein 2 [Source:MGI Symbol;Acc:MGI:1196378]
ENSMUSG00000025076	11.93693533	14.46526182	11.79463473	14.60420847	14.43749521	16.59845561	8.023747746	7.631401766	8.817272876	9.125372672	9.474141256	8.88742698	Casp7	caspase 7 [Source:MGI Symbol;Acc:MGI:109383]
ENSMUSG00000025077	6.513567882	7.908355435	5.073891425	8.456841074	8.740398858	11.31114904	6.115404577	5.067408468	7.462881793	7.20363007	7.867631224	7.717560307	Dclre1a	DNA cross-link repair 1A [Source:MGI Symbol;Acc:MGI:1930042]
ENSMUSG00000025078	8.69404789	10.70434053	8.054113896	9.963816256	12.19577832	13.49476385	6.486387549	6.275627206	8.013329575	9.399201923	10.74975686	9.670664557	Nhlrc2	NHL repeat containing 2 [Source:MGI Symbol;Acc:MGI:1914116]
ENSMUSG00000025081	2.508348662	2.492808532	1.758923945	2.436944951	2.481989927	2.892017792	0.115037495	0.110628073	0.095945262	0.445658225	0.514836302	0.395869643	Tdrd1	tudor domain containing 1 [Source:MGI Symbol;Acc:MGI:1933218]
ENSMUSG00000025082	0.085015535	0.195799518	0.054398926	0.109085763	0.29029792	0.12950959	0.16128736	0.189495001	0.316515892	0.884016022	1.008427712	0.922950027	Vwa2	von Willebrand factor A domain containing 2 [Source:MGI Symbol;Acc:MGI:2684334]
ENSMUSG00000025083	6.258689139	7.51608237	3.844560017	6.528851201	8.431057812	8.597859959	1.117523028	0.752872115	1.165066226	2.91908732	2.642076009	3.169488182	Afap1l2	actin filament associated protein 1-like 2 [Source:MGI Symbol;Acc:MGI:2147658]
ENSMUSG00000025085	2.723322014	3.818596327	2.837319168	1.764002377	1.699940489	2.184121256	3.389545947	2.491533168	3.384645345	16.80803595	17.26837444	16.3264484	Ablim1	actin-binding LIM protein 1 [Source:MGI Symbol;Acc:MGI:1194500]
ENSMUSG00000025086	7.140279697	7.12289226	6.608900054	7.747897315	8.265293128	8.521561565	17.69714696	17.29297536	20.28538789	17.54516557	18.05529902	15.81972891	Trub1	TruB pseudouridine (psi) synthase family member 1 [Source:MGI Symbol;Acc:MGI:1919383]
ENSMUSG00000025089	4.983347982	6.920054007	3.601351841	3.790644623	5.367800329	5.310826702	0.130853518	0.103712525	0.21437502	1.779249054	1.969492423	1.874171958	Gfra1	glial cell line derived neurotrophic factor family receptor alpha 1 [Source:MGI Symbol;Acc:MGI:1100842]
ENSMUSG00000025090	4.339783107	4.093683158	5.341099005	4.17906368	4.171583024	4.923136147	0.164870618	0.137207664	0.300798348	2.849430987	2.075226691	2.413992893	Ccdc172	coiled-coil domain containing 172 [Source:MGI Symbol;Acc:MGI:1922895]
ENSMUSG00000025091	0.054743633	0.063040096	0.11209224	0.025542925	0.086275417	0	0.195495441	0.298272471	0.331195273	0.465185344	0.364548351	0.388121167	Pnliprp2	pancreatic lipase-related protein 2 [Source:MGI Symbol;Acc:MGI:1336202]
ENSMUSG00000025092	9.072079097	12.61728572	8.764664479	14.24896986	14.49888161	16.7641427	16.99721828	14.74658131	18.19605103	9.74342727	11.00830471	8.747637379	Hspa12a	heat shock protein 12A [Source:MGI Symbol;Acc:MGI:1920692]
ENSMUSG00000025094	1.504786931	1.369306741	0.732588167	1.581000228	1.636300102	1.514866691	0.770363911	0.823536798	0.989230693	0.545928601	0.551837407	0.708755382	Slc18a2	solute carrier family 18 (vesicular monoamine), member 2 [Source:MGI Symbol;Acc:MGI:106677]
ENSMUSG00000025102	38.87455367	34.47707644	47.4223677	35.98453751	33.18570545	23.93876553	37.64515754	45.88684151	34.21320959	22.15336498	19.5720631	20.35144094	3110040N11	RIKEN cDNA 3110040N11 gene [Source:MGI Symbol;Acc:MGI:1914540]
ENSMUSG00000025103	90.42964009	93.34434796	87.38973289	87.16493651	91.2659817	101.9325219	83.71879384	85.36089911	94.1206362	53.24552137	57.04395625	51.35404842	Btbd1	BTB (POZ) domain containing 1 [Source:MGI Symbol;Acc:MGI:1933765]
ENSMUSG00000025104	9.116303842	8.92723595	6.993936228	11.53147833	13.509828	14.50900832	3.070274167	2.644627235	2.712622021	4.264854578	4.22117064	3.936233185	Hdgfl3	HDGF like 3 [Source:MGI Symbol;Acc:MGI:1352760]
ENSMUSG00000025105	13.0188758	16.62238024	10.79656645	18.04332562	20.2085047	23.59309306	0.090291324	0.081972784	0.111247643	1.875054772	1.423491205	2.468861137	Bnc1	basonuclin 1 [Source:MGI Symbol;Acc:MGI:1097164]
ENSMUSG00000025127	0.168833753	0.444390275	0.296315584	0.382628432	0.152045914	0.349050668	0.08613196	0.310614361	0.22449076	0.237313694	0.341304307	0.235124412	Gcgr	glucagon receptor [Source:MGI Symbol;Acc:MGI:99572]
ENSMUSG00000025128	0.231612674	0.459340605	0.237123356	0.204129522	0.108153872	0.2254194	0.022975391	0.025493928	0.011976421	0.230191186	0.417720737	0.330698037	Bhlhe22	basic helix-loop-helix family, member e22 [Source:MGI Symbol;Acc:MGI:1930001]
ENSMUSG00000025129	0.502278068	0.257066229	1.142730226	0.156239213	1.172719023	0.382576116	0	0	0	0.049919541	0.185820366	0.296754122	Ppp1r27	protein phosphatase 1, regulatory subunit 27 [Source:MGI Symbol;Acc:MGI:1915951]
ENSMUSG00000025130	1315.720504	1237.818946	1266.89435	1079.407698	1027.348973	940.1621691	417.7472892	418.9538992	434.0638752	667.3722003	667.6339986	661.0740725	P4hb	prolyl 4-hydroxylase, beta polypeptide [Source:MGI Symbol;Acc:MGI:97464]
ENSMUSG00000025132	428.2501812	392.0306376	478.2157912	428.2771145	383.6743792	316.5327638	242.4017447	244.4037529	214.6387329	254.089546	261.3080856	253.3209288	Arhgdia	Rho GDP dissociation inhibitor (GDI) alpha [Source:MGI Symbol;Acc:MGI:2178103]
ENSMUSG00000025133	30.10645293	32.49454128	26.34849322	32.17313146	35.37302903	38.79457741	54.28657121	53.3691648	67.01641719	68.02355937	65.84017486	66.60706738	Ints4	integrator complex subunit 4 [Source:MGI Symbol;Acc:MGI:1917164]
ENSMUSG00000025134	77.47240933	62.90263862	83.66952861	83.56064448	74.5978881	62.30886551	94.35103201	99.69581322	77.9839832	76.5270246	79.48312146	81.64982907	Alyref	Aly/REF export factor [Source:MGI Symbol;Acc:MGI:1341044]
ENSMUSG00000025135	109.2494406	78.41115804	129.5372754	100.4607153	84.05741033	61.85991619	88.2755662	95.52659603	66.19377026	48.87282686	47.97720852	47.93432904	Anapc11	anaphase promoting complex subunit 11 [Source:MGI Symbol;Acc:MGI:1913406]
ENSMUSG00000025137	63.78051281	48.75672337	62.07591371	58.05045653	51.04664975	45.28433251	80.86875566	83.644092	80.28138488	47.54913331	46.54147134	50.46617558	Pcyt2	phosphate cytidylyltransferase 2, ethanolamine [Source:MGI Symbol;Acc:MGI:1915921]
ENSMUSG00000025138	42.81517379	37.65342087	49.82396853	42.82658472	40.14959717	34.58448353	16.02432652	15.99978703	17.92568255	22.18782197	25.28996094	21.28096947	Sirt7	sirtuin 7 [Source:MGI Symbol;Acc:MGI:2385849]
ENSMUSG00000025139	38.68856432	42.34185674	40.21191212	39.52258183	40.25900423	41.56137843	26.47957503	21.8422362	29.27059123	30.54848218	31.13326987	29.78348004	Tollip	toll interacting protein [Source:MGI Symbol;Acc:MGI:1891808]
ENSMUSG00000025140	12.84702538	15.83584183	14.3753348	21.27560222	20.95019631	18.57837671	22.29289261	20.86479677	21.62452084	14.76073395	16.83905248	14.49627664	Pycr1	pyrroline-5-carboxylate reductase 1 [Source:MGI Symbol;Acc:MGI:2384795]
ENSMUSG00000025141	0.074123149	0	0	0.034585252	0	0.169374784	0.231614107	0.146859079	0.068990786	0	0	0	Myadml2	myeloid-associated differentiation marker-like 2 [Source:MGI Symbol;Acc:MGI:1915765]
ENSMUSG00000025142	48.85373248	44.87077865	53.77481736	59.33943994	54.77458366	50.18638427	81.19999871	83.31195262	79.36930825	93.48328578	95.02270036	97.69658858	Aspscr1	alveolar soft part sarcoma chromosome region, candidate 1 (human) [Source:MGI Symbol;Acc:MGI:1916188]
ENSMUSG00000025144	36.56828786	26.0032582	39.92614412	31.19361088	25.40632767	21.30762774	76.7238821	86.66081433	69.72744838	50.52208258	46.81847773	53.785254	Cenpx	centromere protein X [Source:MGI Symbol;Acc:MGI:894324]
ENSMUSG00000025145	49.06658382	44.57831832	47.98084431	44.33997159	43.7260784	40.98364417	17.10405634	17.44165834	16.6236806	27.61038374	28.55955448	27.60572248	Lrrc45	leucine rich repeat containing 45 [Source:MGI Symbol;Acc:MGI:2387183]
ENSMUSG00000025147	21.8026386	19.4279267	24.74248369	19.35010091	17.36217588	13.6411976	18.20328541	18.17026752	17.85032558	13.77518367	13.85143241	12.62577407	Mob2	MOB kinase activator 2 [Source:MGI Symbol;Acc:MGI:1919891]
ENSMUSG00000025150	2.277978767	2.113141107	3.174353323	1.94862466	1.19669078	0.337379624	0.169477297	0.06268507	0.058895811	0	0.105343751	0.224311204	Cbr2	carbonyl reductase 2 [Source:MGI Symbol;Acc:MGI:107200]
ENSMUSG00000025151	374.1812822	363.0860635	343.2096603	278.5097034	270.0249105	270.6922659	106.5950241	105.5745996	115.6774525	169.5208687	166.8835321	162.1017477	Maged1	melanoma antigen, family D, 1 [Source:MGI Symbol;Acc:MGI:1930187]
ENSMUSG00000025153	56.78605912	65.36436941	58.64194178	73.39400365	78.03425772	90.35297361	81.86508169	75.03006954	99.93259497	125.8657615	138.7528538	128.882397	Fasn	fatty acid synthase [Source:MGI Symbol;Acc:MGI:95485]
ENSMUSG00000025154	5.559352944	5.759957699	4.180027942	7.128085876	8.12040172	9.444334378	33.63989847	29.55153307	38.7590528	32.23501945	30.23616484	32.28479115	Arhgap19	Rho GTPase activating protein 19 [Source:MGI Symbol;Acc:MGI:1918335]
ENSMUSG00000025155	39.41711832	30.98053644	42.30391756	36.86178089	32.03113772	28.46669977	66.95731559	73.40964142	66.75727144	70.54040421	73.45139331	70.08812996	Dus1l	dihydrouridine synthase 1-like (S. cerevisiae) [Source:MGI Symbol;Acc:MGI:1915980]
ENSMUSG00000025156	127.3792149	129.1788381	127.2655851	138.4093565	129.464629	119.4704026	105.7170137	104.4901818	100.7148093	130.6148938	141.0467007	142.8265608	Gps1	G protein pathway suppressor 1 [Source:MGI Symbol;Acc:MGI:2384801]
ENSMUSG00000025157	31.99268395	41.07731865	24.87925011	35.88847112	45.85247833	48.26950967	26.02457083	22.36200559	28.37679519	31.1592992	32.66202116	31.26700605	Zdhhc16	zinc finger, DHHC domain containing 16 [Source:MGI Symbol;Acc:MGI:1921418]
ENSMUSG00000025158	11.89710479	12.44680375	12.92942189	13.01091759	12.6772563	10.79127151	12.46430503	12.13897887	12.75005696	12.33696262	13.40204063	13.52030158	Rfng	RFNG O-fucosylpeptide 3-beta-N-acetylglucosaminyltransferase [Source:MGI Symbol;Acc:MGI:894275]

ENSMUSG00000025159	41.09011033	39.67580939	39.81508165	47.45411017	45.85048196	48.58908173	61.06776924	55.68693334	66.34766092	48.73476261	50.35294507	50.20419757	Mms19	MMS19 cytosolic iron-sulfur assembly component [Source:MGI Symbol;Acc:MGI:1919449]
ENSMUSG00000025161	32.50793906	27.59517009	37.21360851	19.66117899	17.5160302	17.27737828	165.7851917	166.5606377	151.9070016	201.4767864	206.954818	206.8697747	Slc16a3	solute carrier family 16 (monocarboxylic acid transporters), member 3 [Source:MGI Symbol;Acc:MGI:1933438]
ENSMUSG00000025162	133.5599359	139.0139188	136.8318113	142.7393218	145.574649	143.3513253	72.8623621	67.30876378	75.50577214	110.8859145	119.4150955	114.7789567	Csnk1d	casein kinase 1, delta [Source:MGI Symbol;Acc:MGI:1355272]
ENSMUSG00000025163	0.382718443	0.440719961	0.615723954	0	0.057443861	0.166576979	0.292870552	0.054162449	0.050888376	0.586855904	0.091021285	0.290721001	Cd7	CD7 antigen [Source:MGI Symbol;Acc:MGI:88344]
ENSMUSG00000025165	0.292426616	0.336744281	0.299384225	0.389839591	0.438915712	0.190916625	0	0	0	0.037366958	0	0	Sectm1a	secreted and transmembrane 1A [Source:MGI Symbol;Acc:MGI:2384805]
ENSMUSG00000025169	6.842450664	5.454992546	7.308362609	5.495009581	6.463268755	4.710639052	4.845910491	5.50746809	3.643615467	3.795831546	3.477632405	3.61504601	Ogfod3	2-oxoglutarate and iron-dependent oxygenase domain containing 3 [Source:MGI Symbol;Acc:MGI:1913429]
ENSMUSG00000025170	2.96355148	2.559511403	2.742325509	3.084638032	3.113685502	2.778195127	0.254403007	0.338748459	0.212180935	0.662706706	0.521835272	0.505071585	Rab40b	Rab40B, member RAS oncogene family [Source:MGI Symbol;Acc:MGI:2183451]
ENSMUSG00000025171	86.3123376	72.29034179	97.16767353	74.94521531	68.90803024	52.06239084	34.15920258	38.4096412	36.00858431	27.79051973	26.14525628	25.57280849	Ubtd1	ubiquitin domain containing 1 [Source:MGI Symbol;Acc:MGI:2385092]
ENSMUSG00000025172	0.384526254	1.151284545	0.787350316	0.574133731	0.848413481	0.497907373	0	0.076185608	0.286321012	0.309554689	0.640158632	0.102232988	Ankrd2	ankyrin repeat domain 2 (stretch responsive muscle) [Source:MGI Symbol;Acc:MGI:1861447]
ENSMUSG00000025173	57.94421185	57.46612868	54.48402414	58.28268716	58.78371209	65.40778156	72.77450243	65.88460197	73.32822536	92.32170536	93.6041596	89.83807717	Wdr45b	WD repeat domain 45B [Source:MGI Symbol;Acc:MGI:1914090]
ENSMUSG00000025175	0.212248146	0.133317082	0.237052467	0.126042307	0.141909519	0.146968702	0.103358352	0.07645892	0.053877782	0.276146844	0.256982066	0.37619906	Fn3k	fructosamine 3 kinase [Source:MGI Symbol;Acc:MGI:1926834]
ENSMUSG00000025176	5.786911595	7.470046282	5.685702574	5.966406143	5.736847339	5.296452359	0.374982307	1.155798473	0.825307895	2.629871889	2.058887636	2.109303839	Hoga1	4-hydroxy-2-oxoglutarate aldolase 1 [Source:MGI Symbol;Acc:MGI:1914682]
ENSMUSG00000025178	25.01079781	24.18881751	25.21851073	23.82666953	24.79740688	23.06503999	21.08274283	21.61019325	22.21039968	20.46934294	21.02530956	21.61018765	Pi4k2a	phosphatidylinositol 4-kinase type 2 alpha [Source:MGI Symbol;Acc:MGI:1934031]
ENSMUSG00000025184	13.54059052	12.09598735	14.80689852	11.64933623	11.03621781	11.51288127	10.96419776	10.36122278	10.1954714	9.979106806	10.27886285	10.10631609	R3hcc1l	R3H domain and coiled-coil containing 1 like [Source:MGI Symbol;Acc:MGI:1196316]
ENSMUSG00000025185	14.2266342	19.29295064	13.09299791	19.27366936	23.37098381	29.25599918	0.291758385	0.2805752	0.932790365	6.937577736	6.945753574	5.966110151	Loxl4	lysyl oxidase-like 4 [Source:MGI Symbol;Acc:MGI:1914823]
ENSMUSG00000025188	19.86675677	17.98959892	20.89505381	20.65552779	18.02223502	18.70481268	14.88142996	16.35910912	16.16398188	12.82932212	16.61773952	14.41148965	Hps1	HPS1, biogenesis of lysosomal organelles complex 3 subunit 1 [Source:MGI Symbol;Acc:MGI:2177763]
ENSMUSG00000025189	0.820706852	1.170106636	1.100305977	0.437640085	0.492733715	0.535815051	0.994389956	0.987247154	1.091257535	0.996282508	0.845811773	0.536839258	Cnnm1	cyclin M1 [Source:MGI Symbol;Acc:MGI:1891366]
ENSMUSG00000025190	41.09703451	39.91145523	37.82707768	51.93583667	48.10195136	49.28362704	234.3848952	225.0701451	234.9350476	129.4449808	127.8153337	126.7610024	Got1	glutamic-oxaloacetic transaminase 1, soluble [Source:MGI Symbol;Acc:MGI:95791]
ENSMUSG00000025192	5.07728254	5.755751815	4.550852293	3.650316167	4.545739999	4.559471191	2.116520885	2.602955805	2.758206028	6.361647334	5.985924093	5.777712832	Entpd7	ectonucleoside triphosphate diphosphohydrolase 7 [Source:MGI Symbol;Acc:MGI:2135885]
ENSMUSG00000025193	5.459158795	5.247001654	4.664873647	5.856551213	5.871210175	4.845709345	10.43840191	9.41090144	9.642203314	5.421381877	5.474507441	3.847575798	Cutc	cutC copper transporter [Source:MGI Symbol;Acc:MGI:1913638]
ENSMUSG00000025194	0.166133158	0.212567635	0.056695303	0.051677578	0.077577552	0.098420482	1.219519177	0.548595208	0.738787437	3.533429774	3.672343732	3.451755851	Abcc2	ATP-binding cassette, sub-family C (CFTR/MRP), member 2 [Source:MGI Symbol;Acc:MGI:1352447]
ENSMUSG00000025195	8.212258562	9.612653974	6.777269261	10.03351297	11.43877624	13.41404038	7.071529741	5.897916034	7.837388385	7.671524986	7.996849557	8.154206034	Dnmbp	dynamin binding protein [Source:MGI Symbol;Acc:MGI:1917352]
ENSMUSG00000025196	0.362120937	0.392471411	0.785089607	0.536704902	0.626649957	0.697664796	2.738484647	3.123081911	2.26019964	5.144431149	5.088833298	5.795446406	Cpn1	carboxypeptidase N, polypeptide 1 [Source:MGI Symbol;Acc:MGI:2135874]
ENSMUSG00000025197	0.371575888	0.142629579	0.025361111	0.161815973	0.234239991	0.339627157	0.287503226	0.343558934	0.415017114	0.376681306	0.536119034	0.790319268	Cyp2c23	cytochrome P450, family 2, subfamily c, polypeptide 23 [Source:MGI Symbol;Acc:MGI:1888897]
ENSMUSG00000025198	24.37702637	30.53555499	20.51626975	32.38183696	37.21786961	41.40803683	23.72530302	21.11239903	28.9871883	30.08165651	28.45130834	29.75289136	Erlin1	ER lipid raft associated 1 [Source:MGI Symbol;Acc:MGI:2387613]
ENSMUSG00000025199	29.45048947	42.9284072	22.41303319	41.56413954	53.91531567	63.51251553	37.25807051	33.82791552	47.53638131	59.44628181	57.07331475	58.89787412	Chuk	conserved helix-loop-helix ubiquitous kinase [Source:MGI Symbol;Acc:MGI:99484]
ENSMUSG00000025200	11.38517752	10.30999238	9.521852838	13.71702659	14.71376324	13.63887815	19.61304972	18.16231772	19.65144702	17.11638753	18.70931197	17.40849997	Cwf19l1	CWF19-like 1, cell cycle control (S. pombe) [Source:MGI Symbol;Acc:MGI:1919752]
ENSMUSG00000025201	1.394441735	1.60577134	0.917755212	1.39421798	1.360435506	0.910389462	2.223081833	3.1574702	1.854127385	1.603665265	0.663275472	0.617894289	Bloc1s2-ps	biogenesis of lysosomal organelles complex-1, subunit 2, pseudogene [Source:MGI Symbol;Acc:MGI:3702045]
ENSMUSG00000025202	0.436099539	0.526104896	0.531518463	0.523235474	0.174549492	0.25308124	1.223639174	1.748647227	1.353011876	1.114516401	1.175457303	1.067426869	Scd3	stearoyl-coenzyme A desaturase 3 [Source:MGI Symbol;Acc:MGI:1353437]
ENSMUSG00000025203	184.3981273	213.5140765	165.8288064	214.8226868	236.599294	271.7809956	146.9259808	132.1949341	161.3946127	114.0702126	117.4316083	115.1891889	Scd2	stearoyl-Coenzyme A desaturase 2 [Source:MGI Symbol;Acc:MGI:98240]
ENSMUSG00000025204	54.72921697	52.66447055	70.71545426	65.63097693	68.67169995	50.598488	102.0464764	95.16033544	53.04756035	59.64509408	42.67654023	49.05987738	Ndufb8	NADH:ubiquinone oxidoreductase subunit B8 [Source:MGI Symbol;Acc:MGI:1914514]
ENSMUSG00000025207	9.904494484	10.45507333	9.773445303	11.01568447	9.948120722	11.66010217	12.24881883	11.12314013	13.07431584	6.630572563	6.805595232	6.997243097	Sema4g	sema domain, immunoglobulin domain (Ig), transmembrane domain (TM) and short cytoplasmic domain, (semaphorin) 4G [Source:M
ENSMUSG00000025208	10.7458815	10.3033998	15.55868398	13.17470433	9.589643286	6.986311609	12.56410588	10.15537437	9.123004213	7.601071729	6.774142593	5.737866536	Mrpl43	mitochondrial ribosomal protein L43 [Source:MGI Symbol;Acc:MGI:2137229]
ENSMUSG00000025209	13.11142521	11.50692481	13.39238007	12.98418231	12.59851799	11.72807142	15.51733022	16.6333797	16.31840868	18.41788679	18.00925935	18.87860552	Twnk	twinkle mtDNA helicase [Source:MGI Symbol;Acc:MGI:2137410]
ENSMUSG00000025212	72.7061314	77.53115833	71.55695464	73.88647571	78.25399267	76.15384744	13.21119981	14.49711572	14.66266525	23.51771042	22.74917028	23.2523613	Sfxn3	sideroflexin 3 [Source:MGI Symbol;Acc:MGI:2137679]
ENSMUSG00000025213	5.095855511	4.681324829	6.213624113	3.793608684	3.880155068	2.573076803	0.638969629	0.510489194	0.13323071	0.563363447	0.762568207	0.329825523	Kazald1	Kazal-type serine peptidase inhibitor domain 1 [Source:MGI Symbol;Acc:MGI:2147606]
ENSMUSG00000025215	0	0	0.061354109	0	0	0	0.321015494	0.118734952	0.055778763	0.080406615	0.024942114	0	Tlx1	T cell leukemia, homeobox 1 [Source:MGI Symbol;Acc:MGI:98769]
ENSMUSG00000025216	0	0	0	0	0	0	0.386622927	0.326043118	0.225719856	0.030988701	0.014419032	0.046054233	Lbx1	ladybird homeobox 1 [Source:MGI Symbol;Acc:MGI:104867]
ENSMUSG00000025217	13.92663111	13.25007137	11.85870842	13.53387311	12.16991784	13.31444588	19.13882908	18.40142622	17.39635019	15.82473974	15.22217208	16.24053173	Btrc	beta-transducin repeat containing protein [Source:MGI Symbol;Acc:MGI:1338871]
ENSMUSG00000025218	8.016925658	7.37699329	8.473044681	8.172373071	7.820027528	6.035835126	14.95931279	15.31522497	13.37269431	8.992670284	9.231630126	8.991752052	Poll	polymerase (DNA directed), lambda [Source:MGI Symbol;Acc:MGI:1889000]
ENSMUSG00000025219	0.140462964	0	0.431414855	0.218462883	0.196771788	0.178313496	1.128619216	1.901697231	1.481687869	12.77348177	12.27673099	12.6141491	Fgf8	fibroblast growth factor 8 [Source:MGI Symbol;Acc:MGI:99604]
ENSMUSG00000025220	41.52844202	47.54049775	38.51024676	52.69097906	60.24920638	73.28514429	52.89866605	50.13665451	62.60051157	62.71248784	65.49914402	65.96313373	Oga	O-GlcNAcase [Source:MGI Symbol;Acc:MGI:1932139]
ENSMUSG00000025221	0.615155206	0.372833075	0.331469151	0.18127981	0.850419833	0.46855259	9.134005934	8.403289765	8.708957705	3.35937103	4.015586491	4.275246651	Kcnip2	Kv channel-interacting protein 2 [Source:MGI Symbol;Acc:MGI:2135916]
ENSMUSG00000025223	133.6267287	123.9914528	144.6169121	121.7317837	111.2673334	94.86306237	101.9666047	108.2179173	96.96332833	93.93501027	96.16689311	99.78948515	Ldb1	LIM domain binding 1 [Source:MGI Symbol;Acc:MGI:894762]
ENSMUSG00000025224	52.00565435	56.79776611	51.18301012	54.13979116	56.83563764	63.24914414	29.38690275	25.88728041	34.86293046	44.62573559	44.96435087	38.86447121	Gbf1	golgi-specific brefeldin A-resistance factor 1 [Source:MGI Symbol;Acc:MGI:1861607]
ENSMUSG00000025225	41.54311219	43.41723991	44.78025286	54.97325206	53.1110578	51.16502451	9.635455502	9.768097306	9.148208684	11.23684659	11.23305606	11.93141197	Nfkb2	nuclear factor of kappa light polypeptide gene enhancer in B cells 2, p49/p100 [Source:MGI Symbol;Acc:MGI:1099800]
ENSMUSG00000025226	9.927097115	10.01695455	13.52839165	10.06935208	7.778900457	5.968108696	3.290161113	5.426901906	3.028408063	3.68249061	2.984739623	2.589271306	Fbxl15	F-box and leucine-rich repeat protein 15 [Source:MGI Symbol;Acc:MGI:1915681]
ENSMUSG00000025227	1.077468939	0.728272685	0.695435632	0.502738155	0.344538082	0.553072749	29.42280875	31.11663678	31.0015751	14.26799407	13.14130325	13.4928623	Mfsd13a	major facilitator superfamily domain containing 13a [Source:MGI Symbol;Acc:MGI:1922396]
ENSMUSG00000025228	176.3437673	182.8145849	175.2120616	184.9218159	196.4355785	187.882752	78.70426609	75.15292667	83.75200923	88.73463251	93.2150649	92.2309293	Actr1a	ARP1 actin-related protein 1A, centractin alpha [Source:MGI Symbol;Acc:MGI:1858964]
ENSMUSG00000025229	0.486197603	0	0.248882769	0.056713952	0	0.277745938	1.682006322	2.167415985	1.301031759	0.869784551	0.910598529	0.484740314	Pitx3	paired-like homeodomain transcription factor 3 [Source:MGI Symbol;Acc:MGI:1100498]
ENSMUSG00000025231	22.52326775	25.64287601	21.39680724	24.02714846	25.34590355	26.34289206	18.80360512	16.36097227	18.7183905	23.98495371	22.55208116	22.09858358	Sufu	SUFU negative regulator of hedgehog signaling [Source:MGI Symbol;Acc:MGI:1345643]
ENSMUSG00000025232	118.1989974	103.0709363	138.0715818	77.65151525	69.0128988	57.71577281	33.27619373	35.00188596	29.61550483	25.8000496	26.88060094	24.41575761	Hexa	hexosaminidase A [Source:MGI Symbol;Acc:MGI:96073]
ENSMUSG00000025234	48.43939663	57.24337152	44.26901906	56.13583533	63.15322765	76.90874608	65.10992215	54.72142723	76.55102992	90.14896005	93.56644671	87.16986031	Arih1	ariadne RBR E3 ubiquitin protein ligase 1 [Source:MGI Symbol;Acc:MGI:1344363]
ENSMUSG00000025235	2.927424118	4.548281672	4.019886762	4.076054985	4.540360266	4.477228521	8.213975277	9.183430471	9.277044278	6.982698003	7.252337098	7.16536222	Bbs4	Bardet-Biedl syndrome 4 (human) [Source:MGI Symbol;Acc:MGI:2143311]
ENSMUSG00000025236	13.09618551	12.94976887	11.48674266	12.64090969	12.54436313	14.15508777	24.60019329	22.08955967	27.6324791	28.9004622	29.66553038	28.01880094	Adpgk	ADP-dependent glucokinase [Source:MGI Symbol;Acc:MGI:1919391]
ENSMUSG00000025237	20.06356918	18.54567585	21.42197995	20.51269096	18.03166069	16.42446634	15.27813625	14.22485432	16.4773652	11.30446555	10.41759407	10.72079378	Parp6	poly (ADP-ribose) polymerase family, member 6 [Source:MGI Symbol;Acc:MGI:1914537]
ENSMUSG00000025239	30.55345465	34.49347309	30.21804944	34.96752491	37.67359862	42.5921758	8.975817993	8.177949344	9.937168893	16.74824689	18.92574449	16.98201704	Limd1	LIM domains containing 1 [Source:MGI Symbol;Acc:MGI:1352502]
ENSMUSG00000025240	2.254389287	9.335777755	1.309361593	6.007850336	12.41235945	22.95006987	3.851161693	2.190345826	5.830833647	15.29823953	13.29825553	14.98421444	Sacm1l	SAC1 suppressor of actin mutations 1-like (yeast) [Source:MGI Symbol;Acc:MGI:1933169]
ENSMUSG00000025241	6.793971762	8.542599718	6.12137761	6.794292364	7.849747357	11.87312651	2.513121294	2.086213402	3.201174187	8.348840713	8.711974877	8.628202733	Fyco1	FYVE and coiled-coil domain containing 1 [Source:MGI Symbol;Acc:MGI:107277]
ENSMUSG00000025243	0	0.158756013	0.047047615	0.028589162	0.112658679	0.023335009	0.054702533	0.075873705	0.014257441	0.082209967	0.025501513	0.122177338	Slc6a20b	solute carrier family 6 (neurotransmitter transporter), member 20B [Source:MGI Symbol;Acc:MGI:1336891]
ENSMUSG00000025245	6.909537137	8.606212686	5.403412712	8.158524991	10.54014164	10.23420963	4.244337809	4.569905957	4.949895132	7.820120508	8.149345263	7.962207627	Lztfl1	leucine zipper transcription factor-like 1 [Source:MGI Symbol;Acc:MGI:1934860]
ENSMUSG00000025246	16.55765008	21.64360483	16.08509701	19.10081785	21.85290179	27.861252	13.7921746	12.22975373	16.6064083	28.00232326	27.52465742	26.68354963	Tbl1x	transducin (beta)-like 1 X-linked [Source:MGI Symbol;Acc:MGI:1336172]
ENSMUSG00000025255	4.523294395	5.538098351	3.50778551	3.769846819	5.511743984	7.187632607	0.09283435	0.036053762	0.096783836	1.469574356	1.20745629	1.216419386	Zfhx4	zinc finger homeodomain 4 [Source:MGI Symbol;Acc:MGI:2137668]
ENSMUSG00000025257	3.9162451	3.526861672	3.546797274	3.888843244	5.327934835	3.480088539	13.58191457	13.72781093	11.58948609	5.928134844	4.973405113	6.095925254	Ribc1	RIB43A domain with coiled-coils 1 [Source:MGI Symbol;Acc:MGI:1913861]
ENSMUSG00000025260	82.91293166	67.23838237	85.80532241	76.15748354	64.74510728	51.59000326	108.6282251	114.7961243	88.66831981	64.64210636	64.49280814	69.2999865	Hsd17b10	hydroxysteroid (17-beta) dehydrogenase 10 [Source:MGI Symbol;Acc:MGI:1333871]
ENSMUSG00000025261	64.10768599	82.48170037	59.85122585	71.48268889	87.22566421	128.1575496	122.8513189	104.5723772	160.9606113	242.5578896	265.2033966	255.742159	Huwe1	HECT, UBA and WWE domain containing 1 [Source:MGI Symbol;Acc:MGI:1926884]
ENSMUSG00000025262	0.137251955	0.482938749	0.093678363	0.249046912	0.520740863	1.283547503	0.047652663	0.045322539	0.12774849	0.531997409	0.488729295	0.682513446	Fam120c	family with sequence similarity 120, member C [Source:MGI Symbol;Acc:MGI:2387687]

ENSMUSG00000025264	0.262689181	0.403333461	0.14343427	5.883293456	0.202397844	0.253441755	0.891187091	1.092970454	1.548501992	0.892883225	0.714296662	11.09687703	Tsr2	TSR2 20S rRNA accumulation [Source:MGI Symbol;Acc:MGI:1916749]
ENSMUSG00000025265	5.126208991	5.697966378	3.667643646	6.353632187	6.508833323	6.814101807	13.76502821	11.4505497	16.69021678	15.75634318	16.93640662	22.04832622	Fgd1	FYVE, RhoGEF and PH domain containing 1 [Source:MGI Symbol;Acc:MGI:104566]
ENSMUSG00000025266	1.253716248	3.704636682	1.017149262	14.98857144	4.697290911	10.55831561	10.15807851	7.56907576	19.57322469	37.74740627	44.02778096	93.07866785	Gnl3l	guanine nucleotide binding protein-like 3 (nucleolar)-like [Source:MGI Symbol;Acc:MGI:2448557]
ENSMUSG00000025268	123.2998763	124.4764183	118.6136602	100.6278711	98.05317696	90.10641214	18.6623514	17.85677404	19.15864816	41.16652079	41.770221	41.19899635	Maged2	melanoma antigen, family D, 2 [Source:MGI Symbol;Acc:MGI:1933391]
ENSMUSG00000025269	6.762257164	5.731090241	6.420478374	5.394061597	6.260695539	5.524666342	9.404466349	10.36904698	10.49005266	11.91769498	11.01629002	10.34414375	Apex2	apurinic/apyrimidinic endonuclease 2 [Source:MGI Symbol;Acc:MGI:1924872]
ENSMUSG00000025270	0.138133187	0.238601199	0.141419744	0.547840388	0.217696557	0.157820324	0.154152705	0.068420301	0.160710881	0.370670623	0.632400691	0.550876633	Alas2	aminolevulinic acid synthase 2, erythroid [Source:MGI Symbol;Acc:MGI:87990]
ENSMUSG00000025271	3.997359519	3.143624858	3.393753414	3.245031819	4.507173733	4.183390852	9.313571115	10.17354069	8.106625837	7.558059862	7.222889541	7.891552783	Pfkfb1	6-phosphofructo-2-kinase/fructose-2,6-biphosphatase 1 [Source:MGI Symbol;Acc:MGI:107816]
ENSMUSG00000025272	6.416985287	5.452244162	6.108010507	4.936239718	7.179394369	7.199452337	10.43595794	9.123065348	10.8476541	25.81377511	26.00007769	26.51320459	Tro	trophinin [Source:MGI Symbol;Acc:MGI:1928994]
ENSMUSG00000025277	7.764539157	6.892225857	6.316837166	8.647446086	7.842259305	6.389349888	5.199002807	6.638826978	5.592255315	4.216726713	5.020517991	4.648855727	Abhd6	abhydrolase domain containing 6 [Source:MGI Symbol;Acc:MGI:1913332]
ENSMUSG00000025278	60.07733505	80.43459147	60.76073502	67.5617571	78.13795536	104.2635094	58.63144249	50.42732178	74.26396074	134.5908976	150.2504707	139.6909468	Flnb	filamin, beta [Source:MGI Symbol;Acc:MGI:2446089]
ENSMUSG00000025279	0.098350829	0.226512071	0.268508954	0.15296563	0.103333299	0.09988273	0.01463423	0.016238418	0.030513639	0.087972495	0.040933572	0.043580463	Dnase1l3	deoxyribonuclease 1-like 3 [Source:MGI Symbol;Acc:MGI:1314633]
ENSMUSG00000025280	12.73236385	13.78350815	12.34727485	14.89865218	15.07580055	17.8880577	18.08043997	16.55372726	19.42443018	30.7871238	29.13428411	28.34615667	Polr3a	polymerase (RNA) III (DNA directed) polypeptide A [Source:MGI Symbol;Acc:MGI:2681836]
ENSMUSG00000025283	174.9797483	156.2053263	169.6606933	152.9397911	155.5235971	141.0295212	113.6130433	105.5771479	102.8289026	114.4236838	119.8275004	114.5827532	Sat1	spermidine/spermine N1-acetyl transferase 1 [Source:MGI Symbol;Acc:MGI:98233]
ENSMUSG00000025287	64.50675475	64.42265618	64.347225	67.17985715	62.5961331	56.47763924	59.3246116	64.36645564	64.72826914	74.85201487	76.15578122	76.23392568	Acot9	acyl-CoA thioesterase 9 [Source:MGI Symbol;Acc:MGI:1928939]
ENSMUSG00000025288	0	0	0	0	0	0	0	0	0	0	0	0	4933436I01I	RIKEN cDNA 4933436I01 gene [Source:MGI Symbol;Acc:MGI:1914030]
ENSMUSG00000025289	129.3918718	109.986819	139.864774	118.4997347	101.3622926	88.91995223	62.17500796	67.6896727	56.26437259	63.89863796	65.76750787	61.21436785	Prdx4	peroxiredoxin 4 [Source:MGI Symbol;Acc:MGI:1859815]
ENSMUSG00000025290	1041.23121	744.5830425	1321.157891	971.3883197	852.5379324	624.9781332	1613.910957	1823.867421	1266.778699	903.1495744	850.7340525	868.385843	Rps24	ribosomal protein S24 [Source:MGI Symbol;Acc:MGI:98147]
ENSMUSG00000025314	2.92632617	3.257827204	2.579595502	2.433794116	2.72160207	3.151477637	2.959845958	2.566133835	3.735836201	7.275309033	7.337050514	6.440363192	Ptprj	protein tyrosine phosphatase, receptor type, J [Source:MGI Symbol;Acc:MGI:104574]
ENSMUSG00000025316	35.50825747	33.37341654	36.35309353	33.03641458	35.36908006	32.26066005	39.9349802	39.18204467	37.80402184	37.94856371	36.67317341	37.34698332	Banp	BTG3 associated nuclear protein [Source:MGI Symbol;Acc:MGI:1889023]
ENSMUSG00000025317	0.151153939	0.05802051	0	0.047018124	0	0.076754147	0.404840382	0.199652682	0.328271734	0.045067876	0.251640811	0.089304226	Car5a	carbonic anhydrase 5a, mitochondrial [Source:MGI Symbol;Acc:MGI:101946]
ENSMUSG00000025318	0.427496855	0.577899318	0.704074841	0.381588126	0.6249098	0.580447018	1.427074493	1.233663843	1.435887506	1.197033943	0.928299148	0.955381629	Jph3	junctophilin 3 [Source:MGI Symbol;Acc:MGI:1891497]
ENSMUSG00000025321	0.84369584	1.302302663	0.661610766	1.180984642	1.282505485	1.784317769	0.056091777	0.026674498	0	0.160567236	0.254020393	0.15908596	Itgb8	integrin beta 8 [Source:MGI Symbol;Acc:MGI:1338035]
ENSMUSG00000025323	1.254259762	2.022082428	1.193459453	1.96911774	2.387544933	3.55164284	3.240759294	2.42657432	3.474772063	4.514020747	5.023697555	4.289297921	Sp4	trans-acting transcription factor 4 [Source:MGI Symbol;Acc:MGI:107595]
ENSMUSG00000025324	7.636359005	9.880699088	6.79391285	11.01788292	12.58253785	15.47483227	1.08175424	0.851400363	1.337594452	6.502878405	6.227492102	6.230621951	Atp10a	ATPase, class V, type 10A [Source:MGI Symbol;Acc:MGI:1330809]
ENSMUSG00000025326	17.90872606	20.21218731	17.50326516	22.72036108	23.12451275	26.64775343	18.00475205	16.34775835	20.59617967	32.60490706	30.91926405	33.33995672	Ube3a	ubiquitin protein ligase E3A [Source:MGI Symbol;Acc:MGI:105098]
ENSMUSG00000025328	1.537213291	1.242231667	1.573787691	1.283501955	1.558418997	1.725489021	0.770464011	0.534325888	1.016603345	2.327852557	2.24486841	2.306377501	Padi3	peptidyl arginine deiminase, type III [Source:MGI Symbol;Acc:MGI:1338891]
ENSMUSG00000025329	0	0.02639825	0	0.032088554	0.06021353	0	0.020466092	0	0.010668394	0.051262608	0.038163959	0	Padi1	peptidyl arginine deiminase, type I [Source:MGI Symbol;Acc:MGI:1338893]
ENSMUSG00000025330	0.585783869	0.674560237	0.436160893	1.093287763	0.30772989	0.547585741	4.873171463	3.95660715	4.584834797	2.072062546	2.748324604	2.454097827	Padi4	peptidyl arginine deiminase, type IV [Source:MGI Symbol;Acc:MGI:1338898]
ENSMUSG00000025332	14.01915471	17.20605183	13.71895182	14.95136551	15.8388523	19.70139294	39.59601602	34.37723514	42.87349225	48.9758015	49.01203468	47.43754857	Kdm5c	lysine (K)-specific demethylase 5C [Source:MGI Symbol;Acc:MGI:99781]
ENSMUSG00000025333	0	0.045752092	0.040676131	0.11122847	0	0	0.177353897	0.157436187	0.073959652	0.142153154	0.132287629	0.352104383	Gpr143	G protein-coupled receptor 143 [Source:MGI Symbol;Acc:MGI:107193]
ENSMUSG00000025337	113.6163261	105.3611173	108.3198888	115.3858222	105.8928586	94.66263139	67.96836757	70.50262908	62.35874022	69.89225795	74.97372953	80.14928311	Sbds	SBDS ribosome maturation factor [Source:MGI Symbol;Acc:MGI:1913961]
ENSMUSG00000025340	39.85720899	48.96853303	39.44041496	53.26893542	56.11283735	56.59969058	28.02289606	24.63302757	22.94273002	32.62129143	34.04702987	34.65176012	Rabgef1	RAB guanine nucleotide exchange factor (GEF) 1 [Source:MGI Symbol;Acc:MGI:1929459]
ENSMUSG00000025347	4.792603929	5.156372358	6.697374624	7.606309216	6.946644039	5.009362504	0	0.242586125	0.032560286	0	0.029119411	0.062004723	Mettl7b	methyltransferase like 7B [Source:MGI Symbol;Acc:MGI:1918914]
ENSMUSG00000025348	85.72946632	83.31286806	88.67930323	93.24513682	86.69706864	86.68069177	1.559428654	1.684022254	1.364487221	6.110086724	5.789897232	6.247217441	Itga7	integrin alpha 7 [Source:MGI Symbol;Acc:MGI:102700]
ENSMUSG00000025350	3.375834039	4.319384924	5.156801393	4.450385464	4.109847123	4.489591868	1.052461755	1.008581701	0.997488207	0.718953295	1.15969958	1.044737115	Rdh5	retinol dehydrogenase 5 [Source:MGI Symbol;Acc:MGI:1201412]
ENSMUSG00000025351	1979.275113	1593.151175	2212.124613	1810.895477	1569.782765	1232.443098	103.8273396	111.1841692	88.38433439	208.4290855	216.4597869	206.5688876	Cd63	CD63 antigen [Source:MGI Symbol;Acc:MGI:99529]
ENSMUSG00000025352	24.61797285	27.00704766	23.91314911	24.43608991	27.75715066	27.24713631	0.775484499	0.829010824	0.788757292	3.013090425	3.456478382	2.778764103	Gdf11	growth differentiation factor 11 [Source:MGI Symbol;Acc:MGI:1338027]
ENSMUSG00000025353	10.37140842	11.6470973	7.634553321	13.75776531	12.42778473	10.28268576	18.71002355	15.19925645	14.75914947	17.32725179	18.69326967	16.9774984	Ormdl2	ORM1-like 2 (S. cerevisiae) [Source:MGI Symbol;Acc:MGI:1914094]
ENSMUSG00000025354	27.99215911	37.15669172	26.1215321	36.99414925	41.31344784	45.94125354	12.95129366	11.12956199	13.66072157	20.94422082	20.7974033	21.23709461	Dnajc14	DnaJ heat shock protein family (Hsp40) member C14 [Source:MGI Symbol;Acc:MGI:1921580]
ENSMUSG00000025355	23.28194732	31.67594285	17.85484489	21.24352334	27.11140133	31.34549369	1.270230646	1.025070486	1.223947015	11.28029075	9.869379516	10.75592452	Mmp19	matrix metallopeptidase 19 [Source:MGI Symbol;Acc:MGI:1927899]
ENSMUSG00000025357	19.76491618	20.37353879	17.96041646	25.67037369	24.26789497	26.28236491	17.91212849	17.74023719	21.71451106	20.00780988	20.44115321	19.58811636	Dgka	diacylglycerol kinase, alpha [Source:MGI Symbol;Acc:MGI:102952]
ENSMUSG00000025358	23.77138239	26.12371541	21.91887084	30.3950629	29.73862818	31.38123357	60.38563382	57.47602129	64.55022607	43.58221247	46.80452254	44.04105725	Cdk2	cyclin-dependent kinase 2 [Source:MGI Symbol;Acc:MGI:104772]
ENSMUSG00000025359	2.542247517	1.529306038	2.408500452	2.86810556	2.431840758	2.109791452	14.97298545	13.1185369	12.78464978	14.25480236	13.13917125	13.24899863	Pmel	premelanosome protein [Source:MGI Symbol;Acc:MGI:98301]
ENSMUSG00000025362	1552.458371	1232.535682	1916.727343	1559.965375	1375.278086	1072.796931	2175.168456	2512.227918	1743.2674	1275.185208	1151.362283	1156.78553	Rps26	ribosomal protein S26 [Source:MGI Symbol;Acc:MGI:1351628]
ENSMUSG00000025364	85.75720591	85.55655913	85.24795041	102.0836543	107.2304486	97.65463812	248.1525461	248.2395964	245.166912	228.2258027	221.6577053	216.0864599	Pa2g4	proliferation-associated 2G4 [Source:MGI Symbol;Acc:MGI:894684]
ENSMUSG00000025366	39.92571844	38.94863594	38.47352364	39.26809126	36.30405779	34.40490903	37.56780425	36.11003725	37.22088903	20.45963409	21.96398383	22.31615757	Esyt1	extended synaptotagmin-like protein 1 [Source:MGI Symbol;Acc:MGI:1344426]
ENSMUSG00000025369	80.02793509	93.74651997	75.84573332	95.14252635	103.1209704	109.6743156	19.69606999	17.35063859	21.58421982	27.25038402	29.11610582	27.37211241	Smarcc2	SWI/SNF related, matrix associated, actin dependent regulator of chromatin, subfamily c, member 2 [Source:MGI Symbol;Acc:MGI:19
ENSMUSG00000025370	0	0.015407188	0	0	0	0.020381854	0.215008512	0.172305976	0.062265472	0.418867793	0.42320935	0.332002902	Cdh9	cadherin 9 [Source:MGI Symbol;Acc:MGI:107433]
ENSMUSG00000025371	71.21156746	68.18066772	75.10255593	75.65304854	72.26019617	53.71052439	39.0878906	43.77479238	37.24247244	49.27704589	48.89824105	47.43486661	Chmp6	charged multivesicular body protein 6 [Source:MGI Symbol;Acc:MGI:3583942]
ENSMUSG00000025372	27.92960244	27.29704582	29.80072119	31.41724312	27.52217003	25.9143419	22.90994398	24.16566146	23.160207	22.8938756	25.09957786	23.80596841	Baiap2	brain-specific angiogenesis inhibitor 1-associated protein 2 [Source:MGI Symbol;Acc:MGI:2137336]
ENSMUSG00000025373	24.51643149	25.89412912	23.79631748	24.85309224	23.84237181	24.85898327	10.89009658	10.32837413	9.848152014	15.58908834	15.83905009	16.60405166	Rnf41	ring finger protein 41 [Source:MGI Symbol;Acc:MGI:1914838]
ENSMUSG00000025374	43.18844503	34.75220055	49.89313096	36.3719855	30.1388789	21.60567408	43.98429388	41.62226516	33.05171613	18.64782509	15.40081263	17.15271921	Nabp2	nucleic acid binding protein 2 [Source:MGI Symbol;Acc:MGI:1917167]
ENSMUSG00000025375	0	0.059358509	0.158318958	0	0.08123687	0.00981552	0.264612652	0.165958676	0.191909681	0.599393307	0.57924866	0.513920548	Aatk	apoptosis-associated tyrosine kinase [Source:MGI Symbol;Acc:MGI:1197518]
ENSMUSG00000025377	21.48957162	20.99603902	21.64723533	22.24097453	19.72679488	20.7468571	15.28664491	14.8848287	15.06435975	22.95153958	22.07748919	24.51088656	Tepsin	TEPSIN, adaptor related protein complex 4 accessory protein [Source:MGI Symbol;Acc:MGI:1926027]
ENSMUSG00000025380	0.260515567	0.099999022	0.533427853	0.526735834	0.27371333	0.429931653	0.15505491	0.043012964	0.282890071	0.116512512	0.036142155	0.115437653	Fscn2	fascin actin-bundling protein 2 [Source:MGI Symbol;Acc:MGI:2443337]
ENSMUSG00000025381	160.7576263	132.6796711	193.3858729	133.1881274	121.2949974	92.3671006	61.49433536	73.09711018	52.37196738	60.8456975	59.23560175	64.07744064	Cnpy2	canopy FGF signaling regulator 2 [Source:MGI Symbol;Acc:MGI:1928477]
ENSMUSG00000025383	0.251139034	0.096399836	0.142841278	0.286438558	0.26386178	0.085016915	0.797195415	1.050442421	0.389583186	0.499195413	1.091694648	0.642967265	Il23a	interleukin 23, alpha subunit p19 [Source:MGI Symbol;Acc:MGI:1932410]
ENSMUSG00000025384	9.849603232	9.307951754	8.894689438	13.12101835	11.41649884	10.58058934	12.57449104	12.53842427	13.22207646	17.07930566	15.61196766	16.70727376	Faap100	Fanconi anemia core complex associated protein 100 [Source:MGI Symbol;Acc:MGI:1919135]
ENSMUSG00000025386	0.532036944	0.038291743	0.20426082	0.248244067	0.10481063	0.253276821	0.059373808	0.098823449	0.185699312	0.237947242	0.166075157	0.206283101	Pde6g	phosphodiesterase 6G, cGMP-specific, rod, gamma [Source:MGI Symbol;Acc:MGI:97526]
ENSMUSG00000025389	0.08027703	0.184886252	0.082187034	0.093641506	0.168687755	0.107004732	1.809654973	1.272413364	1.270215627	2.064419838	1.871030809	2.169876106	Mip	major intrinsic protein of lens fiber [Source:MGI Symbol;Acc:MGI:96990]
ENSMUSG00000025393	1946.608789	1841.656397	1941.672152	2106.428502	1979.142677	1762.605558	2734.215653	2702.965897	2566.303859	1982.416179	2014.914147	1956.719186	Atp5b	ATP synthase, H+ transporting mitochondrial F1 complex, beta subunit [Source:MGI Symbol;Acc:MGI:107801]
ENSMUSG00000025395	29.8037577	29.47642112	30.85064934	42.82182397	42.34849502	40.22932501	73.14777325	73.10400973	76.72035544	48.86619117	49.22817837	48.85397693	Prim1	DNA primase, p49 subunit [Source:MGI Symbol;Acc:MGI:97757]
ENSMUSG00000025396	0.086402586	0.149245529	0.088458334	0	0	0	0	0	0	0.077285073	0.035960718	0.038286049	Hsd17b6	hydroxysteroid (17-beta) dehydrogenase 6 [Source:MGI Symbol;Acc:MGI:1351670]
ENSMUSG00000025400	0.071594822	0	0	0	0	0.109064961	0.063918226	0.283699486	0.19991257	0.128079755	0.297977333	0.380694556	Tac2	tachykinin 2 [Source:MGI Symbol;Acc:MGI:98476]
ENSMUSG00000025401	1.758605455	1.342745648	1.506896164	1.890834585	1.526358149	1.849089569	1.587108587	1.306611946	1.458920669	1.880798001	1.432038558	1.626281142	Myo1a	myosin IA [Source:MGI Symbol;Acc:MGI:107732]
ENSMUSG00000025402	34.15007449	34.02167446	40.39157025	34.38495003	31.58214916	24.16507015	6.492651392	7.054277612	5.781743706	8.998594955	9.307342994	10.44626755	Nab2	Ngfi-A binding protein 2 [Source:MGI Symbol;Acc:MGI:107563]
ENSMUSG00000025403	132.8853819	114.5859361	123.8802672	193.0148129	179.9721626	164.778128	205.0122602	220.1412827	215.8839977	155.8845945	161.2844756	155.9322155	Shmt2	serine hydroxymethyltransferase 2 (mitochondrial) [Source:MGI Symbol;Acc:MGI:1277989]

ENSMUSG00000025404	47.455375	52.15997718	42.80424907	54.98152656	56.62274465	59.84679514	40.01865514	38.64291561	46.02069499	34.81515845	36.24411455	37.10945695	R3hdm2	R3H domain containing 2 [Source:MGI Symbol;Acc:MGI:1919000]
ENSMUSG00000025405	0	0	0	0	0	0	0.351292372	0.596165702	0.366237534	0.248443032	0.578002294	0.307688849	Inhbc	inhibin beta-C [Source:MGI Symbol;Acc:MGI:105932]
ENSMUSG00000025407	2.630312877	2.963093958	1.609882898	4.402208533	3.995153715	5.661957054	16.31035997	12.71694725	15.40761378	13.45160818	13.99356995	12.8967764	Gli1	GLI-Kruppel family member GLI1 [Source:MGI Symbol;Acc:MGI:95727]
ENSMUSG00000025408	105.3148476	77.94984775	131.8344171	91.61848418	80.4928595	59.59276191	18.17678461	18.60982131	18.16863947	13.04835933	8.167994774	11.16085015	Ddit3	DNA-damage inducible transcript 3 [Source:MGI Symbol;Acc:MGI:109247]
ENSMUSG00000025409	31.65636492	33.96900093	33.73509832	36.32741927	34.03093573	35.23946853	20.47893173	19.79515294	22.23390709	16.98566219	17.17198243	18.45448998	Mbd6	methyl-CpG binding domain protein 6 [Source:MGI Symbol;Acc:MGI:106378]
ENSMUSG00000025410	234.1169208	206.5758176	261.2939258	221.4404669	203.6956674	176.5254181	150.470369	160.4718699	146.1351751	116.431228	114.624913	123.7504241	Dctn2	dynactin 2 [Source:MGI Symbol;Acc:MGI:107733]
ENSMUSG00000025413	103.02771	102.7782142	101.7832126	128.3981991	124.5081555	108.9728285	58.93735085	61.08197655	58.04616658	52.85136366	56.36754174	58.2473684	Ttc4	tetratricopeptide repeat domain 4 [Source:MGI Symbol;Acc:MGI:1919604]
ENSMUSG00000025417	26.38660843	30.51736047	25.10992911	31.58063687	33.00041862	30.95384723	22.63755285	22.170544	22.44190898	16.66489266	17.19972338	17.3354442	Pip4k2c	phosphatidylinositol-5-phosphate 4-kinase, type II, gamma [Source:MGI Symbol;Acc:MGI:2152214]
ENSMUSG00000025418	0.015612659	0.071915117	0	0	0	0.01189188	0	0.077332815	0.014531622	0.027930309	0	0	Bsnd	barttin CLCNK type accessory beta subunit [Source:MGI Symbol;Acc:MGI:2153465]
ENSMUSG00000025420	4.966011857	5.541021102	5.115745184	6.764205944	6.8055533	5.849986479	3.745104053	3.78896424	3.732178995	3.145253166	2.978320828	3.225579035	Katnal2	katanin p60 subunit A-like 2 [Source:MGI Symbol;Acc:MGI:1924234]
ENSMUSG00000025421	3.056753757	4.022867524	2.826113913	4.351536743	4.669942272	5.250495513	0.891102088	0.633439564	0.798370183	1.520546696	1.415019727	1.478876695	Hdhd2	haloacid dehalogenase-like hydrolase domain containing 2 [Source:MGI Symbol;Acc:MGI:1924237]
ENSMUSG00000025422	5.042796866	5.875129061	4.790920025	6.566126234	6.753735819	6.144324638	0.339353835	0.351449863	0.400963237	0.528888427	0.653900554	0.688697925	Agap2	ArfGAP with GTPase domain, ankyrin repeat and PH domain 2 [Source:MGI Symbol;Acc:MGI:3580016]
ENSMUSG00000025423	17.85699415	20.4841573	14.95948321	17.64125263	20.3671953	27.63427997	38.55281644	35.80503169	46.72933721	60.11249018	58.92776441	57.77758943	Pias2	protein inhibitor of activated STAT 2 [Source:MGI Symbol;Acc:MGI:1096566]
ENSMUSG00000025425	0	0.055362587	0	0	0	0.048825357	0.085843249	0.079377742	0.074579426	0.157678819	0.333490461	0.312448381	St8sia5	ST8 alpha-N-acetyl-neuraminide alpha-2,8-sialyltransferase 5 [Source:MGI Symbol;Acc:MGI:109243]
ENSMUSG00000025427	0.957349041	0.601329073	1.425639165	0.844652446	1.170441899	1.286035599	6.04505592	5.207526701	7.225696432	6.92845673	7.737140577	6.370910047	Rnf165	ring finger protein 165 [Source:MGI Symbol;Acc:MGI:2444521]
ENSMUSG00000025428	507.1911289	522.4831257	457.5266706	540.5193496	533.2170455	503.4526557	862.3110343	860.9000374	913.9216377	678.0296742	677.5836503	697.4762487	Atp5a1	ATP synthase, H+ transporting, mitochondrial F1 complex, alpha subunit 1 [Source:MGI Symbol;Acc:MGI:88115]
ENSMUSG00000025429	0.025889584	0.208692304	0.026505566	0.144958404	0.19040808	0.019719627	2.634953889	2.026138368	2.988023339	3.357855069	3.706680254	3.028606149	Pstpip2	proline-serine-threonine phosphatase-interacting protein 2 [Source:MGI Symbol;Acc:MGI:1335088]
ENSMUSG00000025431	0	0	0	0.056713952	0	0	0.949519698	0.571956996	1.187898562	0.163084603	0.531182475	0.484740314	Crisp1	cysteine-rich secretory protein 1 [Source:MGI Symbol;Acc:MGI:102553]
ENSMUSG00000025432	3.990751546	3.859088316	3.090467017	3.795721151	4.354189996	3.292990183	0.274065546	0.152054154	0.190483479	0.480527523	0.574943899	0.294725185	Avil	advillin [Source:MGI Symbol;Acc:MGI:1333798]
ENSMUSG00000025433	0	0	0	0	0	0	0	0.060163511	0	0	0	0	Crisp3	cysteine-rich secretory protein 3 [Source:MGI Symbol;Acc:MGI:102552]
ENSMUSG00000025436	8.309608082	7.451675213	9.36293664	9.62613897	8.680398529	7.402345394	15.32756941	16.65293939	14.31279333	6.920144897	7.73183383	8.908963859	Atp23	ATP23 metallopeptidase and ATP synthase assembly factor homolog [Source:MGI Symbol;Acc:MGI:1916984]
ENSMUSG00000025437	2.664560466	8.557898925	1.172169212	5.924920532	12.9697788	20.01006969	7.917139736	3.938818051	12.12906089	23.48008011	22.7342722	24.88693199	Usp33	ubiquitin specific peptidase 33 [Source:MGI Symbol;Acc:MGI:2159711]
ENSMUSG00000025439	45.32088124	31.98113256	44.24108409	37.98966519	35.40810727	29.0410336	79.67612415	86.1130873	73.77088045	73.98268612	73.03747419	71.61887243	Clns1a	chloride channel, nucleotide-sensitive, 1A [Source:MGI Symbol;Acc:MGI:109638]
ENSMUSG00000025450	0.485466203	0.708116432	0.248508369	0.634240718	0.646076005	0.838147199	0.80903792	0.609169693	0.617531107	0.62240788	0.956373885	0.717053492	Gm9752	predicted gene 9752 [Source:MGI Symbol;Acc:MGI:3641896]
ENSMUSG00000025451	94.15911402	92.55685964	79.28083138	93.62116212	100.5571372	90.18387674	43.02578929	43.55319172	40.86034964	46.25485531	43.58212455	40.04956231	Paip1	polyadenylate binding protein-interacting protein 1 [Source:MGI Symbol;Acc:MGI:2384993]
ENSMUSG00000025453	0.199632527	0.088418122	0.0943303	0.186293794	0	0.187146442	0.685489901	0.745419892	0.371619536	0.247246037	0.191739162	0.095264216	Nnt	nicotinamide nucleotide transhydrogenase [Source:MGI Symbol;Acc:MGI:109279]
ENSMUSG00000025461	0.058482484	0	0	0	0	0	0	0	0	0.0130778	0	0.012957154	Cd163l1	CD163 molecule-like 1 [Source:MGI Symbol;Acc:MGI:2443796]
ENSMUSG00000025464	7.527788544	6.476179033	8.066749696	9.238853307	8.155346741	6.693128789	27.90241613	28.72674556	30.94335397	16.76807248	17.55490153	16.56146575	Paox	polyamine oxidase (exo-N4-amino) [Source:MGI Symbol;Acc:MGI:1916983]
ENSMUSG00000025465	24.36893856	23.51924784	24.89761607	25.81627859	26.23306674	22.51708342	38.25138661	44.04396101	37.81817364	21.23165959	22.47387699	22.0389012	Echs1	enoyl Coenzyme A hydratase, short chain, 1, mitochondrial [Source:MGI Symbol;Acc:MGI:2136460]
ENSMUSG00000025466	8.620064339	5.294105175	6.030525069	1.675854668	1.132094783	1.477293084	12.76219693	17.79042058	14.44176416	6.104101511	6.338211687	7.830295954	Fuom	fucose mutarotase [Source:MGI Symbol;Acc:MGI:1916314]
ENSMUSG00000025467	0	0	0	0	0	0	0	0	0	0	0	0	Prap1	proline-rich acidic protein 1 [Source:MGI Symbol;Acc:MGI:893573]
ENSMUSG00000025468	0.03817967	0	0.117264198	0.071257281	0.040113858	0.058161527	0.647632855	0.642980844	0.461968917	0.341508131	0.699175919	0.406029154	Caly	calcyon neuron-specific vesicular protein [Source:MGI Symbol;Acc:MGI:1915816]
ENSMUSG00000025469	0.01944351	0	0	0	0	0	0.121511061	0.250400371	0.180972217	0.26087641	0.242771411	0.465245561	Msx3	msh homeobox 3 [Source:MGI Symbol;Acc:MGI:106587]
ENSMUSG00000025470	26.78303834	19.94339915	30.017463	30.502075	27.21725288	19.06244057	30.57508793	29.77043331	24.02040947	12.30567632	13.7663501	12.97037574	Zfp511	zinc finger protein 511 [Source:MGI Symbol;Acc:MGI:1917002]
ENSMUSG00000025473	39.45144582	37.45550625	38.45256832	24.37543169	23.99165469	23.87165254	3.564839044	3.110924603	4.316559106	42.95249606	44.76676655	43.45934715	Adam8	a disintegrin and metallopeptidase domain 8 [Source:MGI Symbol;Acc:MGI:107825]
ENSMUSG00000025474	23.63933969	21.59128806	21.44851692	27.32472246	25.94340996	27.43105311	27.38401529	29.19348922	30.59699164	25.33342542	26.84700238	26.64970044	Tubgcp2	tubulin, gamma complex associated protein 2 [Source:MGI Symbol;Acc:MGI:1921487]
ENSMUSG00000025475	0.489850727	0.722033015	0.501505581	0.09142413	0.10293333	0.164168592	0.087465515	0	0.054711956	0.858793421	0.326201052	0.486211899	Adgra1	adhesion G protein-coupled receptor A1 [Source:MGI Symbol;Acc:MGI:1277167]
ENSMUSG00000025477	23.69836472	25.27175903	23.80696075	22.74956921	23.71719072	22.97169957	16.46350889	15.91019483	17.28565202	15.76953759	16.28633608	19.37939716	Inpp5a	inositol polyphosphate-5-phosphatase A [Source:MGI Symbol;Acc:MGI:2686961]
ENSMUSG00000025478	0.452748152	0.300786214	0.231760118	0.324997797	0.182955558	0.159161573	0.730675088	0.517513327	0.648306783	0.560731198	0.724744546	0.555558304	Dpysl4	dihydropyrimidinase-like 4 [Source:MGI Symbol;Acc:MGI:1349764]
ENSMUSG00000025479	0.068900701	0.357042146	0	0.160742505	0.036195611	0.157441252	0.061512977	0.068255961	0.064129946	0.092445075	0	0.18318449	Cyp2e1	cytochrome P450, family 2, subfamily e, polypeptide 1 [Source:MGI Symbol;Acc:MGI:88607]
ENSMUSG00000025480	11.06587976	10.41051821	9.963597347	11.15630498	12.24932621	11.43892339	18.70022747	19.8202851	20.50737265	14.22861673	17.27105362	16.85553192	Syce1	synaptonemal complex central element protein 1 [Source:MGI Symbol;Acc:MGI:1921325]
ENSMUSG00000025481	0.832396342	0.958547177	1.114417044	0.776778589	1.412759948	0.731562961	24.86675822	22.13753102	15.55480439	10.5956455	7.51514798	8.171336719	Urah	urate (5-hydroxyiso-) hydrolase [Source:MGI Symbol;Acc:MGI:1916142]
ENSMUSG00000025482	0.151295667	0	0	0	0	0.069143503	0	0	0.197147722	0.135330402	0.025187676	0.026816389	Odf3	outer dense fiber of sperm tails 3 [Source:MGI Symbol;Acc:MGI:1916537]
ENSMUSG00000025484	35.62738375	30.32924088	40.23752391	25.91161559	26.38492601	22.74353745	17.72642732	16.6862749	11.68694101	16.29917437	14.36074011	15.06317587	Bet1l	Bet1 golgi vesicular membrane trafficking protein like [Source:MGI Symbol;Acc:MGI:1913128]
ENSMUSG00000025485	47.24039765	44.45126179	44.5667648	49.5107121	49.60846057	41.55993718	26.85621333	25.6513722	24.32351138	23.43955683	25.9562785	23.81715905	Ric8a	RIC8 guanine nucleotide exchange factor A [Source:MGI Symbol;Acc:MGI:2141866]
ENSMUSG00000025486	8.379727389	8.312464328	6.458426178	10.60215826	9.562663983	10.94857874	15.32671421	15.04807461	16.884325	9.713228566	8.490506587	7.426320607	Sirt3	sirtuin 3 [Source:MGI Symbol;Acc:MGI:1927665]
ENSMUSG00000025487	165.2499852	141.2877514	175.8137164	146.6022291	128.2329668	119.7575131	260.2864888	280.6166225	242.4111654	170.4905629	176.8703731	173.8982114	Psmd13	proteasome (prosome, macropain) 26S subunit, non-ATPase, 13 [Source:MGI Symbol;Acc:MGI:1345192]
ENSMUSG00000025488	0	0	0	0.111537438	0	0	0	0	0	0	0	0	Cox8b	cytochrome c oxidase subunit 8B [Source:MGI Symbol;Acc:MGI:105958]
ENSMUSG00000025489	0.84369584	0.323853043	0.345507845	0.472393857	0.236383364	0.385576713	0.301292973	0.055720062	0.209407328	0.503110672	0.374555561	0.398775474	Ifitm5	interferon induced transmembrane protein 5 [Source:MGI Symbol;Acc:MGI:1934923]
ENSMUSG00000025491	13.86868616	12.62625398	19.72035994	7.521863617	7.347908492	6.365202355	99.95269882	114.9019063	78.88469239	125.801576	115.3441527	110.9320864	Ifitm1	interferon induced transmembrane protein 1 [Source:MGI Symbol;Acc:MGI:1915963]
ENSMUSG00000025492	1405.533805	1116.691149	1634.74132	1122.461453	993.7828564	763.6153451	420.523668	458.2592978	341.1266225	331.1781649	304.9717141	309.6751817	Ifitm3	interferon induced transmembrane protein 3 [Source:MGI Symbol;Acc:MGI:1913391]
ENSMUSG00000025494	8.050264471	5.505501736	9.357504127	8.824579688	6.832956613	7.577653464	52.47519282	57.87921477	48.98168287	15.78509732	17.64507836	17.16603798	Sigirr	single immunoglobulin and toll-interleukin 1 receptor (TIR) domain [Source:MGI Symbol;Acc:MGI:1344402]
ENSMUSG00000025495	49.10209148	48.76145176	52.54741574	55.68002145	46.3317855	49.03071676	80.38098193	83.1331145	72.21586567	69.97464264	72.95107143	67.16849613	Ptdss2	phosphatidylserine synthase 2 [Source:MGI Symbol;Acc:MGI:1351664]
ENSMUSG00000025496	1.334398154	0.620042905	1.006634662	1.201545855	0.811683626	0.677591286	0.459807829	0.278297156	0.52294866	0.20940134	0.233842495	0.352698248	Drd4	dopamine receptor D4 [Source:MGI Symbol;Acc:MGI:94926]
ENSMUSG00000025497	0.091290993	0.280336722	0.280389146	0.113588339	0.127887746	0.139069394	0.353177583	0.482329253	0.084969911	0.163315275	0.075990542	0.215744864	Cdhr5	cadherin-related family member 5 [Source:MGI Symbol;Acc:MGI:1919290]
ENSMUSG00000025498	186.8342235	172.0448595	206.4825021	117.6828997	101.9736504	91.29905749	7.480787372	7.78803082	7.829157441	36.6937905	36.30161515	36.3266092	Irf7	interferon regulatory factor 7 [Source:MGI Symbol;Acc:MGI:1859212]
ENSMUSG00000025499	65.29240625	50.36393896	80.77694004	52.53479349	42.86022029	32.29997169	49.2245566	56.94656313	41.89230774	33.68013362	34.00366088	31.46403378	Hras	Harvey rat sarcoma virus oncogene [Source:MGI Symbol;Acc:MGI:96224]
ENSMUSG00000025500	0.742581765	1.425201792	1.203728793	1.28968505	0.975251355	1.53971909	3.867267188	4.740746245	5.241331911	2.435480135	2.266456166	3.162508395	Lmntd2	lamin tail domain containing 2 [Source:MGI Symbol;Acc:MGI:1919250]
ENSMUSG00000025503	290.3370372	219.0457061	354.9611354	260.4868474	222.9451316	170.732765	403.8070228	437.9784997	361.2652915	235.8611873	237.5408826	236.0359639	Taldo1	transaldolase 1 [Source:MGI Symbol;Acc:MGI:1274789]
ENSMUSG00000025504	0.755516927	1.160022105	0.220997911	0.537170272	0.604793557	0.465851464	4.753677594	3.528393748	6.194893839	5.502878135	5.420446874	5.739065923	Eps8l2	EPS8-like 2 [Source:MGI Symbol;Acc:MGI:2138828]
ENSMUSG00000025505	17.64796964	13.7613874	17.08303082	18.81654808	18.42540232	16.68290646	2.84065129	3.005433802	3.615787541	7.412987504	6.990912067	6.852774789	Tmem80	transmembrane protein 80 [Source:MGI Symbol;Acc:MGI:1918698]
ENSMUSG00000025507	10.19357474	10.19238881	10.18156784	10.67253456	11.38915262	10.99621417	22.87725901	22.01897603	23.17173559	15.07791287	15.53536278	16.01111946	Pidd1	p53 induced death domain protein 1 [Source:MGI Symbol;Acc:MGI:1889507]
ENSMUSG00000025508	1293.66889	921.3992544	2002.59558	1212.413875	999.9519778	689.3262172	1922.924614	2135.254168	1247.934319	802.8152572	751.1680339	748.4813168	Rplp2	ribosomal protein, large P2 [Source:MGI Symbol;Acc:MGI:1914436]
ENSMUSG00000025509	40.52683156	34.98554301	44.1660803	37.55958392	35.3956601	30.0640241	29.82858824	32.21721047	30.03686364	19.14453108	20.31473945	19.75619481	Pnpla2	patatin-like phospholipase domain containing 2 [Source:MGI Symbol;Acc:MGI:1914103]
ENSMUSG00000025510	251.4024008	255.9973953	269.6231697	284.2324836	273.3279227	230.4559326	75.88797525	70.23032925	65.49406699	55.11759842	51.58508113	55.7386993	Cd151	CD151 antigen [Source:MGI Symbol;Acc:MGI:1096360]
ENSMUSG00000025511	149.0242937	133.5682595	165.4227519	114.3501217	101.5894019	86.73490816	55.11696454	56.72066462	50.13017365	53.76865783	53.56139159	55.454144	Tspan4	tetraspanin 4 [Source:MGI Symbol;Acc:MGI:1928097]
ENSMUSG00000025512	19.0245084	20.77774106	21.36639092	16.59166851	16.04910689	13.28586065	8.655763579	8.182769947	9.078534688	10.45384519	10.26474103	10.98040754	Chid1	chitinase domain containing 1 [Source:MGI Symbol;Acc:MGI:1915288]

ENSMUSG00000025515	0.435646684	0.308719724	0.754789334	0.781804475	0.52813456	0.918897775	0.747952766	0.630756313	0.62381856	0.779351344	1.311053853	1.395830623	Muc2	mucin 2 [Source:MGI Symbol;Acc:MGI:1339364]
ENSMUSG00000025519	0.217836736	0.44888983	0.070427259	0.267475872	0.3252397	0.541430663	0.12282927	0.090862452	0.181411025	0.584549449	0.400828343	0.487711011	Tktl2	transketolase-like 2 [Source:MGI Symbol;Acc:MGI:1921669]
ENSMUSG00000025521	34.16829537	25.12751997	38.88950017	27.75119881	25.10472881	20.10235658	32.65052219	36.04288546	29.91623367	29.17160211	29.4616358	28.10892098	Tmem192	transmembrane protein 192 [Source:MGI Symbol;Acc:MGI:1920317]
ENSMUSG00000025525	8.814081603	7.717791046	8.879642056	9.460243469	8.225073626	6.11295542	15.36091351	18.43958464	15.33295395	12.97205673	11.81394322	12.0787473	Apool	apolipoprotein O-like [Source:MGI Symbol;Acc:MGI:1915367]
ENSMUSG00000025527	0	0	0.045532042	0.020751151	0	0	0.019852638	0.022028862	0	0	0	0	Satl1	spermidine/spermine N1-acetyl transferase-like 1 [Source:MGI Symbol;Acc:MGI:1921059]
ENSMUSG00000025528	0	0	0	0	0	0	0	0	0	0	0.141345207	0	2010106E10	RIKEN cDNA 2010106E10 gene [Source:MGI Symbol;Acc:MGI:1914965]
ENSMUSG00000025529	0.590884164	0.854903615	0.698011007	0.763481621	1.066534271	1.2232557	0.175842642	0.180109216	0.084610883	1.179032773	1.046760797	1.235291279	Zfp711	zinc finger protein 711 [Source:MGI Symbol;Acc:MGI:3045342]
ENSMUSG00000025531	5.755500328	8.496217193	5.735725213	7.742150476	10.45372	15.2735564	4.796732045	4.352052042	7.223379517	14.39020016	13.40425222	14.827202	Chm	choroidermia (RAB escort protein 1) [Source:MGI Symbol;Acc:MGI:892979]
ENSMUSG00000025532	44.55956285	43.89539912	45.94406462	52.32269136	47.75995349	42.66654711	55.28910899	60.6699182	48.74694271	36.92955792	35.81687548	36.1677734	Crcp	calcitonin gene-related peptide-receptor component protein [Source:MGI Symbol;Acc:MGI:1100818]
ENSMUSG00000025533	44.29124609	37.18940168	48.83561999	43.0110621	38.67417402	34.12056881	45.71452187	53.25885446	48.04135197	30.01666049	29.45760632	30.77490474	Asl	argininosuccinate lyase [Source:MGI Symbol;Acc:MGI:88084]
ENSMUSG00000025534	66.81141386	63.75684119	64.19936936	54.56876668	47.59193229	50.5007273	18.27436482	18.13113664	18.69593092	29.7780119	31.33976604	31.01687093	Gusb	glucuronidase, beta [Source:MGI Symbol;Acc:MGI:95872]
ENSMUSG00000025537	1.099149952	1.116818534	1.224592981	1.478227208	0.645347895	1.009567878	4.386970563	5.476348578	5.085128931	4.886901816	5.435769231	3.753125856	Phkg1	phosphorylase kinase gamma 1 [Source:MGI Symbol;Acc:MGI:97579]
ENSMUSG00000025538	64.61970578	60.54082142	71.27953543	61.70275038	57.94590357	47.69528764	63.53225866	64.81568971	58.17515391	47.43865623	47.02967192	43.48788325	Sumf2	sulfatase modifying factor 2 [Source:MGI Symbol;Acc:MGI:1915152]
ENSMUSG00000025544	89.09340939	98.30305509	70.0084527	92.51656167	101.9845713	107.4416259	159.3896871	137.023881	173.6382318	90.64434995	88.87434679	88.51734502	Tm9sf2	transmembrane 9 superfamily member 2 [Source:MGI Symbol;Acc:MGI:1915309]
ENSMUSG00000025545	19.27144629	16.29968622	19.15167325	18.82097378	17.21057988	14.17254946	68.99841743	71.62459042	62.31586125	14.3252508	13.99485636	16.16599589	Clybl	citrate lyase beta like [Source:MGI Symbol;Acc:MGI:1916884]
ENSMUSG00000025551	0.02535562	0.102194051	0.012979449	0.106476518	0.053280281	0.164159797	0.01131846	0.062795886	0.023599971	0.045360008	0	0	Fgf14	fibroblast growth factor 14 [Source:MGI Symbol;Acc:MGI:109189]
ENSMUSG00000025555	40.77848065	49.62964961	37.52159316	45.54335168	52.08095758	55.30063859	22.59331849	17.716727	24.43969786	31.10159655	31.83936801	29.54015312	Farp1	FERM, RhoGEF (Arhgef) and pleckstrin domain protein 1 (chondrocyte-derived) [Source:MGI Symbol;Acc:MGI:2446173]
ENSMUSG00000025557	0.108418237	0.124849173	0.12487252	0.075880588	0.071194202	0.123870314	6.884425816	6.806710168	8.993714972	2.327461273	2.673574642	2.534186737	Slc15a1	solute carrier family 15 (oligopeptide transporter), member 1 [Source:MGI Symbol;Acc:MGI:1861376]
ENSMUSG00000025558	4.216255755	6.493431971	3.271729171	5.858521323	7.300049489	10.70220176	11.78030983	9.936499715	15.09279237	8.667630184	9.224518721	8.002976643	Dock9	dedicator of cytokinesis 9 [Source:MGI Symbol;Acc:MGI:106321]
ENSMUSG00000025571	7.655044697	7.225809507	7.334000033	7.074450953	8.15603052	9.84092405	6.281258411	6.189451833	7.657653444	14.01369078	15.28844255	14.66007617	Tnrc6c	trinucleotide repeat containing 6C [Source:MGI Symbol;Acc:MGI:2443265]
ENSMUSG00000025572	27.94204965	28.6063548	31.97465612	28.6860962	27.92745852	24.65251464	32.5749861	32.77470715	34.52391709	38.79235275	44.31472818	41.51728375	Tmc6	transmembrane channel-like gene family 6 [Source:MGI Symbol;Acc:MGI:1098686]
ENSMUSG00000025573	0	0	0.041131886	0.037491576	0	0.030601326	0	0.159200178	0	0	0.234097225	0	6030468B19	RIKEN cDNA 6030468B19 gene [Source:MGI Symbol;Acc:MGI:1924977]
ENSMUSG00000025574	66.24896622	56.68609126	70.83698209	84.73591722	76.61099709	62.59031288	129.5319464	139.8065634	122.4474514	73.53762763	75.81293059	75.50106562	Tk1	thymidine kinase 1 [Source:MGI Symbol;Acc:MGI:98763]
ENSMUSG00000025575	42.26329967	41.31175496	43.63322549	40.71808422	40.59026432	39.02250017	19.92234367	18.13978382	19.97488699	29.97222526	30.9435289	29.28569231	Cant1	calcium activated nucleotidase 1 [Source:MGI Symbol;Acc:MGI:1923275]
ENSMUSG00000025576	0.103870822	0.209322033	0.053171093	0.024232636	0.163699403	0.039558263	0.695501281	0.231522408	0.362545125	0.766507478	0.67008035	1.035594877	Rbfox3	RNA binding protein, fox-1 homolog (C. elegans) 3 [Source:MGI Symbol;Acc:MGI:106368]
ENSMUSG00000025577	10.35696644	11.60130891	8.964681112	12.38870979	12.08852626	12.73668723	11.16300355	10.1294684	11.56780744	13.6939679	14.21702672	14.96945974	Cbx2	chromobox 2 [Source:MGI Symbol;Acc:MGI:88289]
ENSMUSG00000025578	9.111295464	8.551385193	9.813353165	10.51755723	10.95623872	9.687791902	0.141058216	0.260868101	0.465687808	0.895068985	1.53438267	1.633600568	Cbx8	chromobox 8 [Source:MGI Symbol;Acc:MGI:1353589]
ENSMUSG00000025579	270.5445563	271.9131206	278.3656876	228.7431729	211.4044723	211.6207767	64.995624	61.17275001	69.1832679	99.40095687	98.5698028	103.7454641	Gaa	glucosidase, alpha, acid [Source:MGI Symbol;Acc:MGI:95609]
ENSMUSG00000025580	243.4132225	194.1713112	254.058914	215.313346	199.3275854	167.887855	410.3933997	438.3534145	380.5221036	325.9814121	329.1107112	314.9903986	Eif4a3	eukaryotic translation initiation factor 4A3 [Source:MGI Symbol;Acc:MGI:1923731]
ENSMUSG00000025582	0.0907252	0.13296784	0.227987494	0.161629871	0.129983664	0.182182595	0.596435342	0.351334378	0.452923124	1.792711487	2.361703118	1.944288412	Nptx1	neuronal pentraxin 1 [Source:MGI Symbol;Acc:MGI:107811]
ENSMUSG00000025583	17.53531571	20.40801621	17.34666964	20.44731933	19.24175982	22.73897849	18.74166605	16.69242389	20.14778162	35.8691688	37.16503149	36.46295121	Rptor	regulatory associated protein of MTOR, complex 1 [Source:MGI Symbol;Acc:MGI:1921620]
ENSMUSG00000025584	4.206901897	4.249529642	2.720207559	2.410586148	2.998379033	4.234950637	13.57368772	12.50260713	18.41024461	14.25969252	11.87752303	11.44641221	Pde8a	phosphodiesterase 8A [Source:MGI Symbol;Acc:MGI:1277116]
ENSMUSG00000025586	31.08560857	32.92822688	29.97369601	33.7849643	35.24169464	41.45484767	4.508642667	5.374710376	6.224927596	13.7958452	13.2360539	14.57578117	Cpeb1	cytoplasmic polyadenylation element binding protein 1 [Source:MGI Symbol;Acc:MGI:108442]
ENSMUSG00000025588	0	0	0.034469679	0.031418997	0	0	0	0	0.062674729	0.481852474	0.560514483	0.238703629	Nat1	N-acetyl transferase 1 [Source:MGI Symbol;Acc:MGI:97279]
ENSMUSG00000025591	7.186837035	7.17491194	6.015737393	8.001911712	7.437497535	8.246366483	20.85967882	20.71707867	21.31648387	30.14200951	28.37107341	29.76826753	Tma16	translation machinery associated 16 [Source:MGI Symbol;Acc:MGI:1913532]
ENSMUSG00000025592	0.928569624	0.75673231	0.628899986	1.159811611	1.470921381	2.121824045	0.153047227	0.155672087	0.172854904	0.638910464	0.297284823	0.582375038	Dach2	dachshund family transcription factor 2 [Source:MGI Symbol;Acc:MGI:1890446]
ENSMUSG00000025597	0.05140799	0.162797094	0.013157781	0.047973092	0.013503082	0.068523938	0.011473971	0	0	0.06897485	0.117677906	0.011389756	Klhl4	kelch-like 4 [Source:MGI Symbol;Acc:MGI:2442829]
ENSMUSG00000025602	1.658667088	1.896098516	1.61136537	3.231259049	3.345466439	3.799351858	0.756623462	0.515731978	0.540900189	1.364514283	1.491529986	1.105146084	Zfp202	zinc finger protein 202 [Source:MGI Symbol;Acc:MGI:1933401]
ENSMUSG00000025607	39.88812444	40.98461856	31.13552127	34.67800358	40.78010279	42.59363758	43.55486976	39.92420947	47.66413104	41.27230709	42.16852369	40.5741918	Copg2	coatomer protein complex, subunit gamma 2 [Source:MGI Symbol;Acc:MGI:1858683]
ENSMUSG00000025608	1.74399182	1.731289854	1.154409072	1.402986647	1.216296475	1.316915574	9.234598044	7.193670314	9.43156119	15.49201162	16.03124301	16.17517341	Podxl	podocalyxin-like [Source:MGI Symbol;Acc:MGI:1351317]
ENSMUSG00000025609	22.33644432	26.19496686	22.44700647	25.07816291	24.4278493	29.15565742	15.87705221	14.40470083	19.52856716	17.90929787	17.03392804	18.13539416	Mkln1	muskelin 1, intracellular mediator containing kelch motifs [Source:MGI Symbol;Acc:MGI:1351638]
ENSMUSG00000025610	0.599064903	1.079771527	0.453322187	0.34028371	0.492584582	0.595169866	0.046507087	0.20642057	0.072728483	0.069893401	0.151766422	0.300077337	Map3k7cl	Map3k7 C-terminal like [Source:MGI Symbol;Acc:MGI:2446584]
ENSMUSG00000025612	10.46517501	15.91308383	9.33466262	15.20075988	18.92331488	26.01167052	8.94207981	8.854429741	11.81686865	16.27100875	16.10139287	15.92188087	Bach1	BTB and CNC homology 1, basic leucine zipper transcription factor 1 [Source:MGI Symbol;Acc:MGI:894680]
ENSMUSG00000025613	336.2205562	342.1348456	322.5093164	338.2663639	344.7480964	340.8766236	799.327485	788.796106	798.1984269	781.6048058	765.1187756	757.9792098	Cct8	chaperonin containing Tcp1, subunit 8 (theta) [Source:MGI Symbol;Acc:MGI:107183]
ENSMUSG00000025616	22.84607806	26.54438337	17.98800216	19.59872132	25.10676251	30.33547743	21.40256155	20.93233592	23.33816627	40.11454114	35.85951462	34.34231238	Usp16	ubiquitin specific peptidase 16 [Source:MGI Symbol;Acc:MGI:1921362]
ENSMUSG00000025626	7.659616649	9.197563365	7.227176777	8.896584518	11.52668751	14.96657179	10.93158427	10.38160181	14.47865732	24.49234224	23.62550772	24.70173784	Phf6	PHD finger protein 6 [Source:MGI Symbol;Acc:MGI:1918248]
ENSMUSG00000025630	65.72776768	63.04205246	56.3896143	68.37617056	63.13031267	61.73415214	181.8223621	192.1635384	185.5186065	151.8916097	144.3511777	146.9699538	Hprt	hypoxanthine guanine phosphoribosyl transferase [Source:MGI Symbol;Acc:MGI:96217]
ENSMUSG00000025644	0.363922534	0.542333007	0.745162441	0.599305639	0.472325714	0.342415141	0.095559338	0.106034447	0.059774853	0.057444726	0.017819341	0.113829566	Gm7628	predicted gene 7628 [Source:MGI Symbol;Acc:MGI:3643956]
ENSMUSG00000025645	6.908243459	6.331687776	5.658087181	6.394033495	5.717696882	5.691425602	8.005192395	8.351984576	7.00728929	4.388536139	5.116696497	6.297176921	Ccdc51	coiled-coil domain containing 51 [Source:MGI Symbol;Acc:MGI:1913908]
ENSMUSG00000025646	10.23901546	11.29454863	10.4196071	11.81435185	11.55539277	11.33102764	12.95150302	14.45254064	12.54758825	11.54455453	11.52903717	10.78341082	Atrip	ATR interacting protein [Source:MGI Symbol;Acc:MGI:1925349]
ENSMUSG00000025647	406.4945683	414.2439354	417.719637	402.6473432	382.5056457	353.0843483	75.52340958	72.63573292	73.0367398	117.3058925	117.1942601	117.0401621	Shisa5	shisa family member 5 [Source:MGI Symbol;Acc:MGI:1915044]
ENSMUSG00000025648	7.283273792	7.242072474	8.270930857	5.938353741	7.051557455	6.721419599	3.661727858	4.77724694	4.00260151	5.158410282	4.448658711	4.670234428	Pfkfb4	6-phosphofructo-2-kinase/fructose-2,6-biphosphatase 4 [Source:MGI Symbol;Acc:MGI:2687284]
ENSMUSG00000025650	93.70325424	91.38202898	100.5314021	81.97549546	77.12905664	76.05565803	6.229435795	6.725091512	6.995071398	28.43688596	30.81945333	30.05542792	Col7a1	collagen, type VII, alpha 1 [Source:MGI Symbol;Acc:MGI:88462]
ENSMUSG00000025651	365.7421465	282.6257079	374.9219168	325.0167083	287.7739917	246.7649146	599.4300494	638.5555406	573.5021123	363.2980166	355.0221529	362.3039534	Uqcrc1	ubiquinol-cytochrome c reductase core protein 1 [Source:MGI Symbol;Acc:MGI:107876]
ENSMUSG00000025652	0	0	0	0	0	0	0	0	0	0	0	0	Tmem89	transmembrane protein 89 [Source:MGI Symbol;Acc:MGI:1916634]
ENSMUSG00000025656	2.920780933	3.307059063	2.639459815	3.228114259	3.634495066	4.039270651	0.946896289	1.26083281	1.382052631	3.167193289	2.947387933	3.340425098	Arhgef9	CDC42 guanine nucleotide exchange factor (GEF) 9 [Source:MGI Symbol;Acc:MGI:2442233]
ENSMUSG00000025658	0.020455663	0.011777874	0.073298253	0.104988889	0.042983901	0.179178149	0.091311752	0.172246116	0.180873278	0.896558285	0.876904724	0.915479766	Cnksr2	connector enhancer of kinase suppressor of Ras 2 [Source:MGI Symbol;Acc:MGI:2661175]
ENSMUSG00000025665	0.449853506	0.56983238	0.069083508	0.54573467	0.827125532	1.644697324	11.58670716	7.286279246	20.47468384	36.61688385	36.49089927	37.03655876	Rps6ka6	ribosomal protein S6 kinase polypeptide 6 [Source:MGI Symbol;Acc:MGI:1914321]
ENSMUSG00000025666	15.70913352	17.34581823	11.30764001	17.42936039	19.36893226	24.0230034	9.319793315	9.401289732	11.95272659	27.95841011	26.06850017	25.66052807	Tmem47	transmembrane protein 47 [Source:MGI Symbol;Acc:MGI:2177570]
ENSMUSG00000025701	0.998962628	1.290644235	0.997785961	0.136421782	0.127996371	0.111350013	0.565564103	0.458601985	0.703014551	3.639575529	3.894020437	3.433256991	Alox5	arachidonate 5-lipoxygenase [Source:MGI Symbol;Acc:MGI:87999]
ENSMUSG00000025702	24.54054421	34.46922826	22.3922883	30.78262144	37.95301918	45.01189024	8.473864869	7.522208418	10.14032225	19.34241788	21.06875028	19.87104688	Marchf8	membrane associated ring-CH-type finger 8 [Source:MGI Symbol;Acc:MGI:1919029]
ENSMUSG00000025716	0	0	0.055481423	0	0	0	0	0.035789964	0	0.032315673	0.150364717	0.032017552	Myo3a	myosin IIIA [Source:MGI Symbol;Acc:MGI:2183924]
ENSMUSG00000025722	14.59463457	13.53564718	15.99368915	18.04650689	17.74680475	15.65051547	37.38973858	40.23113716	41.06163007	32.27151113	34.33288977	28.70679342	Wdr73	WD repeat domain 73 [Source:MGI Symbol;Acc:MGI:1919218]
ENSMUSG00000025723	3.827905013	2.530535394	3.628686178	2.910631013	3.351522777	3.239606653	6.202068697	4.704994721	4.024708802	2.092426508	2.006217078	1.570547969	Nmb	neuromedin B [Source:MGI Symbol;Acc:MGI:1915289]
ENSMUSG00000025724	92.07692372	76.72032982	97.74222274	92.63576999	81.79946428	67.73297598	66.11683335	64.31900212	52.04509456	43.69197309	45.09899532	43.93340287	Sec11a	SEC11 homolog A, signal peptidase complex subunit [Source:MGI Symbol;Acc:MGI:1929464]
ENSMUSG00000025726	0.179722191	0.068986447	0	0.125785347	0.015735579	0.06844557	3.87758708	3.501461906	4.070432386	4.21987797	4.151416476	4.180948478	Slc28a1	solute carrier family 28 (sodium-coupled nucleoside transporter), member 1 [Source:MGI Symbol;Acc:MGI:3605073]
ENSMUSG00000025727	0.254205324	0.312245865	0.017350236	0.07907341	0.195861167	0.064541199	0.09077949	0.050365318	0.157735926	0.106110975	0.084640115	0.045056603	A930017K11	RIKEN cDNA A930017K11 gene [Source:MGI Symbol;Acc:MGI:2442713]

ENSMUSG00000025728	34.84137927	35.69124623	29.39967771	36.76427155	36.8004766	39.17409763	32.86420992	30.85647916	32.71540132	35.76242369	36.25241688	34.83241723	Pigq	phosphatidylinositol glycan anchor biosynthesis, class Q [Source:MGI Symbol;Acc:MGI:1333114]
ENSMUSG00000025730	20.78473059	18.89425313	21.7771683	20.49475549	18.34150691	18.54140013	23.97214867	22.28918509	21.27536723	18.15642911	17.83386364	18.91900209	Rab40c	Rab40C, member RAS oncogene family [Source:MGI Symbol;Acc:MGI:2183454]
ENSMUSG00000025731	18.55448088	15.4663228	20.32005529	19.82142779	16.04503322	12.99594113	29.26634547	29.91201105	26.25187158	13.21494337	12.87751246	12.38768349	Mettl26	methyltransferase like 26 [Source:MGI Symbol;Acc:MGI:1915597]
ENSMUSG00000025732	13.23953472	10.94042012	20.89658022	15.44364532	10.56148748	11.76503305	26.3212889	33.63920175	24.13218105	9.484926186	8.367475183	11.78752182	Mcrip2	MAPK regulated corepressor interacting protein 2 [Source:MGI Symbol;Acc:MGI:1915491]
ENSMUSG00000025733	18.17352281	18.87600595	20.70364554	19.28697072	24.05494372	19.10121493	21.07647273	19.45962612	22.88522944	18.00886211	17.90843774	19.06645234	Rhot2	ras homolog family member T2 [Source:MGI Symbol;Acc:MGI:2384892]
ENSMUSG00000025735	1.266578546	1.17492719	0.792436226	1.313278098	1.071987925	1.768671596	0.251282774	0.139414056	0.229226542	0.157350639	0.410005116	0.062359615	Rhbdl1	rhomboid like 1 [Source:MGI Symbol;Acc:MGI:2384891]
ENSMUSG00000025736	2.338878635	4.198440173	1.795895092	2.022117589	2.782606325	3.196187464	2.917195153	2.180698843	3.297412156	4.030314624	5.582966202	3.200603511	Jmjd8	jumonji domain containing 8 [Source:MGI Symbol;Acc:MGI:1919356]
ENSMUSG00000025737	22.05860872	21.66769248	20.32994385	22.94799971	23.22583572	20.15476077	17.09439876	18.45244342	19.73819569	20.02729163	21.1000405	20.05228651	Wdr24	WD repeat domain 24 [Source:MGI Symbol;Acc:MGI:2446285]
ENSMUSG00000025738	1.517965649	1.90488866	1.43453732	1.089646617	1.104138033	0.933859475	4.239359744	3.16175442	4.347262038	2.471863964	2.251716731	1.793678013	Fbxl16	F-box and leucine-rich repeat protein 16 [Source:MGI Symbol;Acc:MGI:2448488]
ENSMUSG00000025739	0.16145774	0.185926888	0	0	0.084818609	0	0.360364485	0.319893791	0.150278242	0.288840273	0	0	Gng13	guanine nucleotide binding protein (G protein), gamma 13 [Source:MGI Symbol;Acc:MGI:1925616]
ENSMUSG00000025742	16.65877239	17.66167006	13.52930904	17.3767959	20.9948356	22.96774085	30.69255674	29.93131884	37.96189933	36.69699539	36.23354457	34.45979568	Prps2	phosphoribosyl pyrophosphate synthetase 2 [Source:MGI Symbol;Acc:MGI:97776]
ENSMUSG00000025743	70.4125729	82.67069244	66.70545359	36.12860372	39.41650532	45.2926857	7.929044613	6.977896777	9.430316224	68.11867832	66.81143142	67.44503071	Sdc3	syndecan 3 [Source:MGI Symbol;Acc:MGI:1349163]
ENSMUSG00000025745	175.7222671	180.2965036	167.209676	187.1972353	187.1612978	177.2400851	109.7705666	105.3283084	112.1931779	101.1704488	108.444285	98.6983441	Hadha	hydroxyacyl-CoA dehydrogenase trifunctional multienzyme complex subunit alpha [Source:MGI Symbol;Acc:MGI:2135593]
ENSMUSG00000025746	15.22027295	12.66913105	11.7818175	6.676499844	8.048853541	8.097110981	0.33142227	0.635208711	0.848099679	5.010982288	4.691308396	4.007693512	Il6	interleukin 6 [Source:MGI Symbol;Acc:MGI:96559]
ENSMUSG00000025747	37.78250963	28.65355067	40.17823486	45.2629477	41.56394359	35.00116927	69.74049124	79.31645078	68.59851337	51.20617511	48.75008435	49.1586746	Tyms	thymidylate synthase [Source:MGI Symbol;Acc:MGI:98878]
ENSMUSG00000025754	0	0	0	0	0.014370095	0	0	0	0	0	0	0	Agbl1	ATP/GTP binding protein-like 1 [Source:MGI Symbol;Acc:MGI:3646469]
ENSMUSG00000025757	4.989295476	8.078306244	3.736727838	6.827653624	9.235162309	13.2626387	1.733900028	1.260530905	1.698582691	3.204834554	3.804671987	4.021018354	Hspa4l	heat shock protein 4 like [Source:MGI Symbol;Acc:MGI:107422]
ENSMUSG00000025758	6.734715745	8.462548101	5.972831259	10.27715084	11.09776036	15.15525597	14.16342146	11.49802838	18.19549938	16.27773649	16.18745562	17.79656494	Plk4	polo like kinase 4 [Source:MGI Symbol;Acc:MGI:101783]
ENSMUSG00000025759	1.678583866	2.776950876	1.403862918	1.566426883	2.334935124	3.151348138	0.992031946	1.007094204	1.452331868	1.628337039	1.790843774	2.137118834	Mfsd8	major facilitator superfamily domain containing 8 [Source:MGI Symbol;Acc:MGI:1919425]
ENSMUSG00000025762	9.883646802	7.793084023	7.927471506	7.490224468	9.623716635	8.990678668	13.65736582	12.34808756	13.65245399	9.263014584	8.069932442	7.754899417	Larp1b	La ribonucleoprotein domain family, member 1B [Source:MGI Symbol;Acc:MGI:1914604]
ENSMUSG00000025764	43.70045797	50.78760867	36.53057499	45.75531649	52.29197469	69.84739224	82.24898219	76.1540392	103.5732352	162.3266546	159.6750243	163.5883746	Jade1	jade family PHD finger 1 [Source:MGI Symbol;Acc:MGI:1925835]
ENSMUSG00000025766	2.200471035	2.10654133	2.30711107	2.003962849	1.726996758	2.01934816	4.520786893	5.35777753	5.107932449	5.21709957	4.700551126	4.722559361	D3Ertd751e	DNA segment, Chr 3, ERATO Doi 751, expressed [Source:MGI Symbol;Acc:MGI:1289213]
ENSMUSG00000025774	0.085999643	0	0	0	0	0	0	0	0	0	0	0	Crisp4	cysteine-rich secretory protein 4 [Source:MGI Symbol;Acc:MGI:1925331]
ENSMUSG00000025776	1.536731708	2.088158158	1.25863572	1.534436474	2.018228498	2.633626658	0.137195907	0.076117585	0	1.250858907	1.496633725	1.389127242	Crispld1	cysteine-rich secretory protein LCCL domain containing 1 [Source:MGI Symbol;Acc:MGI:1934666]
ENSMUSG00000025777	0.031731923	0.036540941	0.03248691	0	0.050009202	0.060424079	0.028329538	0.062869982	0.044302159	0.127725552	0.118861312	0.12654725	Gdap1	ganglioside-induced differentiation-associated-protein 1 [Source:MGI Symbol;Acc:MGI:1338002]
ENSMUSG00000025779	9.561886184	9.806674967	14.8370426	9.480682267	12.32240622	11.15227091	0.66692455	0.444019247	0.278119108	2.806414215	1.92764434	1.919885826	Ly96	lymphocyte antigen 96 [Source:MGI Symbol;Acc:MGI:1341909]
ENSMUSG00000025780	0.332245286	0.627209059	0.462827439	0.11690253	0.160232014	0.253065223	0.048626398	0.053956769	0.096320744	0.375135875	0.421641699	0.453733296	Itih5	inter-alpha (globulin) inhibitor H5 [Source:MGI Symbol;Acc:MGI:1925751]
ENSMUSG00000025781	265.9826465	210.8312227	280.7893924	240.2835102	208.6848267	181.6226977	403.3940777	414.1642385	378.7584935	272.6230951	268.8042292	268.5948644	Atp5c1	ATP synthase, H+ transporting, mitochondrial F1 complex, gamma polypeptide 1 [Source:MGI Symbol;Acc:MGI:1261437]
ENSMUSG00000025782	6.314761012	7.516043326	6.465006002	7.019625818	8.486203103	8.737253131	8.973663295	9.149120136	11.01085888	9.55996131	10.30906655	9.804364573	Taf3	TATA-box binding protein associated factor 3 [Source:MGI Symbol;Acc:MGI:2388097]
ENSMUSG00000025783	0.098576146	0	0	0	0	0	0	0	0	0.132261053	0	0	4930412O13	RIKEN cDNA 4930412O13 gene [Source:MGI Symbol;Acc:MGI:1921185]
ENSMUSG00000025784	3.6869168	2.047913448	4.041083435	1.457182663	0.501301917	0.561651563	0	0.042969605	0.161488514	0.077596706	0.036105721	0.076880856	Clec3b	C-type lectin domain family 3, member b [Source:MGI Symbol;Acc:MGI:104540]
ENSMUSG00000025785	23.71761777	16.95657862	25.67574949	23.50365938	18.59526167	17.95612455	76.38190801	75.9173091	61.75772575	34.93079379	32.77497598	33.65601913	Exosc7	exosome component 7 [Source:MGI Symbol;Acc:MGI:1913696]
ENSMUSG00000025786	35.36420248	38.46686674	32.10314451	38.25929181	41.99097158	45.22255064	34.08751996	30.54361271	37.58985023	32.60286049	31.54914692	30.96844679	Zdhhc3	zinc finger, DHHC domain containing 3 [Source:MGI Symbol;Acc:MGI:1926134]
ENSMUSG00000025787	0.306525292	0.705959261	0.666863983	1.072666371	0.603851091	0.67123996	0.4789029	0.037957122	0.320963815	0	0.095681769	0.203737692	Tgm4	transglutaminase 4 (prostate) [Source:MGI Symbol;Acc:MGI:3027002]
ENSMUSG00000025789	0.055664279	0.240376046	0.056988681	0.051944991	0.058484241	0.137795041	0.0248479	0.04135755	0.038857521	0.211609129	0.254841934	0.308319087	St8sia2	ST8 alpha-N-acetyl-neuraminide alpha-2,8-sialyltransferase 2 [Source:MGI Symbol;Acc:MGI:106020]
ENSMUSG00000025790	47.73092176	51.42936701	44.92531291	43.70044417	43.5699648	46.72988899	3.110602566	4.055007029	3.900596864	24.99756366	25.35169555	25.13403231	Slco3a1	solute carrier organic anion transporter family, member 3a1 [Source:MGI Symbol;Acc:MGI:1351867]
ENSMUSG00000025791	136.6818291	132.1860392	122.7774198	143.7780028	140.2621794	131.9800569	27.97518184	27.55786312	29.91820555	23.0896341	21.32619026	23.09479557	Pgm1	phosphoglucomutase 1 [Source:MGI Symbol;Acc:MGI:97565]
ENSMUSG00000025792	49.63304432	38.93826825	60.28841962	45.94768374	41.34400437	35.72609235	9.497790207	9.206869678	8.944801305	17.82898442	15.83448263	14.40498514	Slc25a10	solute carrier family 25 (mitochondrial carrier, dicarboxylate transporter), member 10 [Source:MGI Symbol;Acc:MGI:1353497]
ENSMUSG00000025793	67.77824715	64.51234119	66.48748847	76.57790291	73.37887023	74.3061079	36.89575259	36.1642902	38.777369	50.06433493	51.95485652	51.95579704	Hgs	HGF-regulated tyrosine kinase substrate [Source:MGI Symbol;Acc:MGI:104681]
ENSMUSG00000025794	1329.952636	987.4170301	1811.860579	1255.954456	1066.199066	787.7976427	2058.703742	2312.554704	1549.594094	907.5666013	914.1913073	856.2945412	Rpl14	ribosomal protein L14 [Source:MGI Symbol;Acc:MGI:1914365]
ENSMUSG00000025795	13.78779766	17.18683057	13.6376952	13.83295839	14.82764571	15.76060738	24.92705255	24.03864464	28.06653611	20.52449363	22.00734869	19.70529997	Rassf3	Ras association (RalGDS/AF-6) domain family member 3 [Source:MGI Symbol;Acc:MGI:2179722]
ENSMUSG00000025804	2.111175365	2.343676348	1.508319191	0.212602248	0.367028352	0.323922548	0.14915757	0.15046187	0.197913209	3.763207443	3.628465677	3.634269067	Ccr1	chemokine (C-C motif) receptor 1 [Source:MGI Symbol;Acc:MGI:104618]
ENSMUSG00000025808	0.068444042	0	0.070072508	0	0.095881905	0.208530354	0	0	0	0.06122158	0.113945521	0.161751451	Cccdc7a	coiled-coil domain containing 7A [Source:MGI Symbol;Acc:MGI:1921953]
ENSMUSG00000025809	455.57822	566.5218493	399.1731113	440.6352456	514.8961265	576.2454475	183.4447089	167.7404091	212.5319892	531.5757572	530.5363604	538.7664327	Itgb1	integrin beta 1 (fibronectin receptor beta) [Source:MGI Symbol;Acc:MGI:96610]
ENSMUSG00000025810	8.65115961	9.654302764	9.158158913	7.187198021	9.72161476	14.48249141	0.310374831	0.30465957	0.261352449	5.59737982	7.26799866	6.304134647	Nrp1	neuropilin 1 [Source:MGI Symbol;Acc:MGI:106206]
ENSMUSG00000025812	10.76474571	15.50397672	9.272514748	14.22873063	16.91707448	20.11502613	21.80744019	20.14985146	27.1630319	24.26296675	25.46018859	23.78239272	Pard3	par-3 family cell polarity regulator [Source:MGI Symbol;Acc:MGI:2135608]
ENSMUSG00000025813	0.510607341	0.520145531	0.723816069	0.311551403	0.268237398	0.224377138	2.156561867	1.225663352	1.681662227	1.282346666	1.258740033	1.723029468	Homer2	homer scaffolding protein 2 [Source:MGI Symbol;Acc:MGI:1347354]
ENSMUSG00000025815	2.264657254	3.394726343	2.338526893	3.06070067	3.446006117	3.98522574	13.7345304	11.73951432	16.29949588	16.97382183	16.52711541	16.43659685	Dhtkd1	dehydrogenase E1 and transketolase domain containing 1 [Source:MGI Symbol;Acc:MGI:2445096]
ENSMUSG00000025816	11.64055709	16.66012699	8.512512116	12.80255815	18.25804548	27.23251956	24.27373388	17.66810499	32.65843854	37.52111891	39.76192263	39.60664014	Sec61a2	Sec61, alpha subunit 2 (S. cerevisiae) [Source:MGI Symbol;Acc:MGI:1931071]
ENSMUSG00000025817	17.68948944	20.70680375	16.9410814	19.11008112	22.96694198	22.17304112	34.33623994	32.73244107	33.64623162	31.64566124	29.1399024	30.88294503	Nudt5	nudix (nucleoside diphosphate linked moiety X)-type motif 5 [Source:MGI Symbol;Acc:MGI:1858232]
ENSMUSG00000025821	3.548637037	5.19245394	4.885493913	4.500284052	5.0561935	4.889920842	2.482162798	2.19338831	2.663042735	3.192258489	3.383078934	3.252407049	Zfp282	zinc finger protein 282 [Source:MGI Symbol;Acc:MGI:2141413]
ENSMUSG00000025823	198.6894485	203.8639576	184.422676	180.4713123	178.3966998	172.2724741	111.9150883	111.9200153	120.4683145	165.5010586	169.4845156	157.6419279	Pdia4	protein disulfide isomerase associated 4 [Source:MGI Symbol;Acc:MGI:104864]
ENSMUSG00000025825	175.423623	147.461358	197.2629766	143.2967746	121.9997494	88.89901436	87.58392959	96.21723504	75.6540088	56.88259343	60.79383909	59.62774504	Iscu	iron-sulfur cluster assembly enzyme [Source:MGI Symbol;Acc:MGI:1913633]
ENSMUSG00000025838	0.226465725	0	0	0	0	0	0	0	0.084314003	1.134382167	1.357271071	0.802797993	Pramel6	preferentially expressed antigen in melanoma like 6 [Source:MGI Symbol;Acc:MGI:2156390]
ENSMUSG00000025839	0	0	0	0	0	0	0.120928187	0	0.084048587	0.969266729	0.826832383	1.000338528	Pramel7	preferentially expressed antigen in melanoma like 7 [Source:MGI Symbol;Acc:MGI:2156391]
ENSMUSG00000025854	32.22842327	32.66213081	31.45647207	34.32280815	31.67289369	29.5195146	3.086223306	2.807690567	2.737891097	6.645127926	6.559278094	6.831194004	Fam20c	family with sequence similarity 20, member C [Source:MGI Symbol;Acc:MGI:2136853]
ENSMUSG00000025855	18.79497145	19.80138636	20.26567272	8.545675115	6.554365857	6.091825397	3.855766022	3.604974108	4.503668526	16.81165817	15.67820479	15.94777612	Prkar1b	protein kinase, cAMP dependent regulatory, type I beta [Source:MGI Symbol;Acc:MGI:97759]
ENSMUSG00000025856	114.8076516	96.76306134	130.0000674	126.3942657	109.3934739	95.46956329	43.84796127	48.11349083	42.44556494	26.55425422	23.86709291	26.68957608	Pdgfa	platelet derived growth factor, alpha [Source:MGI Symbol;Acc:MGI:97527]
ENSMUSG00000025857	9.175341849	11.36241848	10.10181624	14.97924067	14.64193762	14.95363117	21.05097677	18.86420494	21.7761344	19.21668676	19.39022337	18.35427753	Dnaaf5	dynein, axonemal assembly factor 5 [Source:MGI Symbol;Acc:MGI:3616079]
ENSMUSG00000025858	106.6199783	95.09032161	106.9189139	101.309151	90.45916569	82.44377346	136.7829	147.9674299	127.2304654	89.39490966	88.48082315	88.4267263	Get4	golgi to ER traffic protein 4 [Source:MGI Symbol;Acc:MGI:1914854]
ENSMUSG00000025860	19.54651756	26.00182725	15.07226792	29.48980733	36.43040421	53.57770618	13.21349584	11.78654555	18.72854579	25.14406797	27.38233324	29.63961416	Xiap	X-linked inhibitor of apoptosis [Source:MGI Symbol;Acc:MGI:107572]
ENSMUSG00000025862	26.58747174	33.8624419	24.06201876	32.1315494	40.09194753	46.85921655	24.0162734	20.92513411	27.87992244	38.85211744	35.60833472	39.92298646	Stag2	stromal antigen 2 [Source:MGI Symbol;Acc:MGI:1098583]
ENSMUSG00000025867	2.147931819	2.191922566	2.351002801	1.914786382	1.935664605	2.241242889	0.124723552	0.259491768	0.243805741	0.905966741	1.279174124	1.168439247	Cplx2	complexin 2 [Source:MGI Symbol;Acc:MGI:104726]
ENSMUSG00000025868	155.3142485	133.1260906	192.1167578	120.2745378	103.811694	77.07964113	127.6938205	135.2449736	96.66184102	76.56016767	65.49845284	72.85267902	Higd2a	HIG1 domain family, member 2A [Source:MGI Symbol;Acc:MGI:1914294]
ENSMUSG00000025869	51.88258076	41.52194047	51.84547882	45.16331585	35.61102172	27.76942158	80.15613405	88.62524945	73.75905187	71.34966578	66.66474309	64.76202808	Nop16	NOP16 nucleolar protein [Source:MGI Symbol;Acc:MGI:107862]
ENSMUSG00000025870	7.787364361	15.80720744	5.87813768	11.4548265	17.19580207	18.90035546	13.37449674	10.39495366	14.74201897	16.88275826	16.20548814	17.42884294	Arl10	ADP-ribosylation factor-like 10 [Source:MGI Symbol;Acc:MGI:1930788]
ENSMUSG00000025871	87.20236575	93.99899237	77.15984196	89.96231724	86.80638945	91.80797085	127.3450708	117.7411392	138.8749638	127.5874378	125.5974196	127.8315119	4833439L19	RIKEN cDNA 4833439L19 gene [Source:MGI Symbol;Acc:MGI:1921162]

ENSMUSG00000025872	42.9576007	44.44762975	47.13663345	43.19641026	44.76144865	41.87191599	98.37873254	105.6517098	102.432138	82.33381274	78.83252988	78.71586421	Thoc3	THO complex 3 [Source:MGI Symbol;Acc:MGI:1920916]
ENSMUSG00000025873	89.90194967	100.0116076	84.94932559	95.17962857	103.923624	103.329825	47.24555381	49.13893766	52.28790997	62.74641175	62.276409	70.12708802	Faf2	Fas associated factor family member 2 [Source:MGI Symbol;Acc:MGI:1923827]
ENSMUSG00000025875	96.79988741	82.80070973	109.4032105	100.8876022	87.60977942	71.95564372	24.00294052	26.29626272	22.16723182	13.01574556	15.28249773	13.04679302	Tspan17	tetraspanin 17 [Source:MGI Symbol;Acc:MGI:1921507]
ENSMUSG00000025876	1.161747252	1.009669193	0.785445113	0.736385737	0.598785673	0.751314788	0.919764718	0.99887366	1.060904676	0.470560974	0.602117621	0.291387453	Unc5a	unc-5 netrin receptor A [Source:MGI Symbol;Acc:MGI:894682]
ENSMUSG00000025877	4.939389333	5.735558725	5.458924376	0.135002087	0.173711118	0.236124068	0.166058447	0.757519868	0.327010363	8.965736643	8.377914677	8.809763283	Hk3	hexokinase 3 [Source:MGI Symbol;Acc:MGI:2670962]
ENSMUSG00000025878	26.16479765	26.6266084	23.40553899	25.41025643	27.26198591	26.56681976	21.4385928	19.33232891	19.29640937	18.52476361	18.65010031	17.81461456	Uimc1	ubiquitin interaction motif containing 1 [Source:MGI Symbol;Acc:MGI:103185]
ENSMUSG00000025880	31.81403906	33.76572044	30.50815109	32.01392503	33.11934105	33.8038882	95.00753188	93.13078	99.74040077	70.95255415	74.10540225	74.24521184	Smad7	SMAD family member 7 [Source:MGI Symbol;Acc:MGI:1100518]
ENSMUSG00000025885	0.293138117	0.434010356	0.171492945	0.214933458	0.307988543	0.382762285	3.495658254	3.360285804	4.24852313	2.547135487	2.126354651	3.488557704	Myo5b	myosin VB [Source:MGI Symbol;Acc:MGI:106598]
ENSMUSG00000025887	65.20120888	67.99616334	50.37694722	65.09019529	66.59233092	66.53290333	0.213772143	0.237205608	0.378873442	2.313133204	2.551229862	2.843522108	Casp12	caspase 12 [Source:MGI Symbol;Acc:MGI:1312922]
ENSMUSG00000025888	2.030756265	1.491230292	1.295654418	0.30211235	0.061844485	0.044834502	0.1576533	0.233246773	0.219147204	1.658510179	1.102434608	1.278052209	Casp1	caspase 1 [Source:MGI Symbol;Acc:MGI:96544]
ENSMUSG00000025889	5.886885823	5.3964819	6.026950522	4.626143261	4.88298668	3.687446066	1.03730479	0.537139395	0.216287047	1.7321317	1.418490118	0.995368349	Snca	synuclein, alpha [Source:MGI Symbol;Acc:MGI:1277151]
ENSMUSG00000025892	0.112615453	0.054034368	0.067255344	0.026272723	0.069020333	0.021444288	0.092162136	0.018593609	0.183431228	0.528842286	0.42183452	0.407527226	Gria4	glutamate receptor, ionotropic, AMPA4 (alpha 4) [Source:MGI Symbol;Acc:MGI:95811]
ENSMUSG00000025893	1.74235074	1.482996066	2.501206107	2.438899622	1.79082247	1.976250364	0.963752941	1.069398468	0.546445289	1.202748949	0.851281371	0.855976294	Kbtbd3	kelch repeat and BTB (POZ) domain containing 3 [Source:MGI Symbol;Acc:MGI:1916399]
ENSMUSG00000025894	12.77901859	9.731641284	12.77579943	13.79723027	11.86635375	11.61047354	21.40739665	22.97163892	23.39140972	21.90027455	22.94436406	21.61424554	Aasdhppt	aminoadipate-semialdehyde dehydrogenase-phosphopantetheinyl transferase [Source:MGI Symbol;Acc:MGI:1914868]
ENSMUSG00000025898	2.446028988	3.252465064	1.715602709	2.875311855	3.379265002	5.023153917	9.398609083	8.246709228	12.57825111	15.6417803	16.26608101	15.92863167	Cwf19l2	CWF19-like 2, cell cycle control (S. pombe) [Source:MGI Symbol;Acc:MGI:1918023]
ENSMUSG00000025899	3.059725872	3.523431627	2.631320918	3.556870446	3.01266323	4.820883978	11.44172614	9.491655644	13.50702794	14.63825133	15.96550687	15.44839739	Alkbh8	alkB homolog 8, tRNA methyltransferase [Source:MGI Symbol;Acc:MGI:1914917]
ENSMUSG00000025900	0.036658989	0.010553677	0.009382801	0	0.01925807	0	0	0	0	0	0	0.016244049	Rp1	retinitis pigmentosa 1 (human) [Source:MGI Symbol;Acc:MGI:1341105]
ENSMUSG00000025902	0	0.115231432	0.025611773	0.046690091	0	0.019054663	0.156339522	0.322172105	0.512256589	1.723007797	1.499311067	2.039666995	Sox17	SRY (sex determining region Y)-box 17 [Source:MGI Symbol;Acc:MGI:107543]
ENSMUSG00000025903	37.0605977	42.81463	33.23775882	52.46127063	53.45366351	54.09304377	58.55468048	63.58404882	70.7386858	73.95988545	72.15535594	78.72514373	Lypla1	lysophospholipase 1 [Source:MGI Symbol;Acc:MGI:1344588]
ENSMUSG00000025905	0.053286053	0	0.027276935	0.024862835	0	0.020293511	0.083252006	0.026393714	0.247982362	0.38130493	0.365930926	0.236117057	Oprk1	opioid receptor, kappa 1 [Source:MGI Symbol;Acc:MGI:97439]
ENSMUSG00000025907	37.28852157	42.59691561	29.05620258	43.60540725	46.54848589	62.11518931	12.26086746	11.66735159	15.43396156	30.596412	29.26932413	30.70979972	Rb1cc1	RB1-inducible coiled-coil 1 [Source:MGI Symbol;Acc:MGI:1341850]
ENSMUSG00000025909	0	0.036246902	0	0	0.016535596	0	0.028101576	0.01559102	0	0.098542708	0.052402159	0.027895322	Sntg1	syntrophin, gamma 1 [Source:MGI Symbol;Acc:MGI:1918346]
ENSMUSG00000025911	1.861683507	1.962656012	1.154324655	3.425647104	3.608951678	3.59495656	1.147061456	1.220849995	1.708373313	0.7270731	0.91670248	0.604177649	Adhfe1	alcohol dehydrogenase, iron containing, 1 [Source:MGI Symbol;Acc:MGI:1923437]
ENSMUSG00000025912	7.103546361	8.440611494	4.632202965	11.4422634	15.3308966	19.33357053	12.13844758	13.58110605	16.52922395	17.17995975	16.38311339	17.44249584	Mybl1	myeloblastosis oncogene-like 1 [Source:MGI Symbol;Acc:MGI:99925]
ENSMUSG00000025915	7.256240428	8.59036888	5.567942404	7.436331819	9.105832287	9.425715296	2.245951252	3.097180553	3.275395292	8.519641086	8.08572924	8.698786977	Sgk3	serum/glucocorticoid regulated kinase 3 [Source:MGI Symbol;Acc:MGI:2182368]
ENSMUSG00000025916	0.270053271	0.57013041	0.092159519	0.252009275	0.141867108	0.034282448	0.040182902	0.089175414	0.041892418	0	0.037465351	0.039887976	Ppp1r42	protein phosphatase 1, regulatory subunit 42 [Source:MGI Symbol;Acc:MGI:1921138]
ENSMUSG00000025917	98.17505065	100.8165188	97.80057626	113.9189657	114.8620359	110.6478201	115.8770425	113.1764275	107.9314222	90.75022227	86.00395317	82.74245524	Cops5	COP9 signalosome subunit 5 [Source:MGI Symbol;Acc:MGI:1349415]
ENSMUSG00000025920	12.44039939	16.53798311	10.06308446	19.30221366	20.36039998	23.72458364	4.229463777	3.898592919	5.173232403	7.17374319	8.352613354	7.586910816	Stau2	staufen double-stranded RNA binding protein 2 [Source:MGI Symbol;Acc:MGI:1352508]
ENSMUSG00000025921	5.555066225	7.147423819	5.099449085	7.254559104	8.428669523	10.08158659	1.925591315	1.675520158	2.137495285	1.943135699	2.234518376	2.392760698	Rdh10	retinol dehydrogenase 10 (all-trans) [Source:MGI Symbol;Acc:MGI:1924238]
ENSMUSG00000025922	0.371851129	0.183516725	0.326312964	0.594866338	0.558127387	0.485541046	0.474257458	0.78936755	0.64276416	0.902804149	0.663275472	0.470776601	Rpl7-ps8	ribosomal protein L7, pseudogene 8 [Source:MGI Symbol;Acc:MGI:3647181]
ENSMUSG00000025925	15.77636944	15.47299737	16.1517313	20.04554771	21.14091738	20.07854294	78.12280266	81.38865897	75.76362015	66.43210353	69.1297229	66.96461926	Terf1	telomeric repeat binding factor 1 [Source:MGI Symbol;Acc:MGI:109634]
ENSMUSG00000025927	0.089381986	0.164684722	0.018301724	0	0.037564037	0	0.063838561	0	0	0.06396006	0	0.031685006	Tfap2b	transcription factor AP-2 beta [Source:MGI Symbol;Acc:MGI:104672]
ENSMUSG00000025929	0	0	0	0	0	0	0	0	0	0	0	0	Il17a	interleukin 17A [Source:MGI Symbol;Acc:MGI:107364]
ENSMUSG00000025930	0.310115228	0	0	0	0	0.029526145	7.544538908	7.757136248	5.66460972	2.531190581	1.613372769	2.2673619	Msc	musculin [Source:MGI Symbol;Acc:MGI:1333884]
ENSMUSG00000025931	9.051643645	9.10904112	7.351100357	12.43494502	13.42862757	15.3457629	3.211112108	3.287002444	4.755991159	2.599896556	2.817181754	3.093446617	Paqr8	progestin and adipoQ receptor family member VIII [Source:MGI Symbol;Acc:MGI:1921479]
ENSMUSG00000025932	2.701085935	3.480211809	2.146531946	4.706311932	4.84233085	5.855586655	1.70320468	1.234989442	1.951473143	1.723341778	1.808139017	1.774402062	Eya1	EYA transcriptional coactivator and phosphatase 1 [Source:MGI Symbol;Acc:MGI:109344]
ENSMUSG00000025933	3.824754474	4.812216332	3.770727589	5.651230214	4.539436081	5.260028003	5.817558147	3.859130244	6.065056692	5.416824897	5.718908513	4.519455371	Tmem14a	transmembrane protein 14A [Source:MGI Symbol;Acc:MGI:1922962]
ENSMUSG00000025934	1.433307888	1.246316844	1.108044363	0.191077053	0.399529666	0.401041721	2.063068302	3.303430753	2.286967184	4.604950429	3.968831205	3.758851924	Gsta3	glutathione S-transferase, alpha 3 [Source:MGI Symbol;Acc:MGI:95856]
ENSMUSG00000025935	131.6485901	129.2050661	111.3278758	132.4197717	141.9534464	137.0993656	74.28397321	70.87380336	76.96713334	83.30939556	76.96761179	84.89753166	Tram1	translocating chain-associating membrane protein 1 [Source:MGI Symbol;Acc:MGI:1919515]
ENSMUSG00000025936	0	0.122344483	0	0	0	0	0	0	0	0	0	0	Gm4956	predicted gene 4956 [Source:MGI Symbol;Acc:MGI:3647976]
ENSMUSG00000025937	17.35558541	23.59018623	15.42818897	26.00868916	28.65472078	30.52390398	30.75386151	31.77341488	36.15389281	28.40591225	28.45443642	29.81129819	Lactb2	lactamase, beta 2 [Source:MGI Symbol;Acc:MGI:2442551]
ENSMUSG00000025938	0.093580878	0.010776319	0.019161483	0.026198441	0.039328681	0.021383659	0.459507824	0.463525965	0.766490938	1.121658872	1.230766926	1.434752612	Slco5a1	solute carrier organic anion transporter family, member 5A1 [Source:MGI Symbol;Acc:MGI:2443431]
ENSMUSG00000025939	59.03081801	62.38051967	53.15395478	65.04199715	70.69767323	68.01587387	19.22506471	17.33999131	18.13060762	22.68987549	24.07525514	24.82665696	Ube2w	ubiquitin-conjugating enzyme E2W (putative) [Source:MGI Symbol;Acc:MGI:1914049]
ENSMUSG00000025940	46.68529982	39.77062161	39.51807147	44.85511263	42.51887165	46.08169663	28.33050144	28.05497247	30.55685971	33.61791208	33.71994793	33.91992088	Tmem70	transmembrane protein 70 [Source:MGI Symbol;Acc:MGI:1915068]
ENSMUSG00000025945	0	0	0	0.06571764	0	0	0	0	0	0	0	0	Crygf	crystallin, gamma F [Source:MGI Symbol;Acc:MGI:88526]
ENSMUSG00000025946	0.904078536	0.878422142	1.01236294	1.160047948	1.602921333	0.946852861	0.025223148	0.027988081	0.026296226	0.126355766	0.070551954	0.100152081	Pth2r	parathyroid hormone 2 receptor [Source:MGI Symbol;Acc:MGI:2180917]
ENSMUSG00000025949	10.62273792	13.88329676	8.779002865	11.33422084	14.87221895	19.56497901	14.07706729	12.66603862	17.59446663	18.29380436	19.21881123	17.51255349	Pikfyve	phosphoinositide kinase, FYVE type zinc finger containing [Source:MGI Symbol;Acc:MGI:1335106]
ENSMUSG00000025950	168.7979945	193.3794857	145.0491064	185.4427591	196.3451184	210.4753619	102.3456296	99.25174446	114.2319377	77.15248305	82.41167312	79.06455468	Idh1	isocitrate dehydrogenase 1 (NADP+), soluble [Source:MGI Symbol;Acc:MGI:96413]
ENSMUSG00000025952	0	0	0	0	0	0	0	0.06106855	0	0	0	0.054631669	Crygc	crystallin, gamma C [Source:MGI Symbol;Acc:MGI:88523]
ENSMUSG00000025955	0.135736555	0.390769051	0.416898267	0.190000684	0.035653248	0.103388078	0.060591252	0	0.063169009	0.151766429	0.112986989	0.150366343	Akr1cl	aldo-keto reductase family 1, member C-like [Source:MGI Symbol;Acc:MGI:1918111]
ENSMUSG00000025956	31.19487922	31.24937054	30.25618912	39.19950752	39.77290498	34.75603484	10.42649387	10.1577794	10.61057434	9.42110048	8.844627998	9.361641828	Mettl21a	methyltransferase like 21A [Source:MGI Symbol;Acc:MGI:1914349]
ENSMUSG00000025958	16.25837927	19.2772483	13.59389222	21.88372869	26.83247763	30.47671268	15.39143469	16.21231951	16.82695582	18.61342546	18.85176897	20.78251214	Creb1	cAMP responsive element binding protein 1 [Source:MGI Symbol;Acc:MGI:88494]
ENSMUSG00000025959	6.059412554	8.63397626	5.249184873	10.42673101	12.07514043	14.51551332	2.401671471	2.216385767	3.557445524	6.003693919	6.096961162	6.030923645	Klf7	Kruppel-like factor 7 (ubiquitous) [Source:MGI Symbol;Acc:MGI:1935151]
ENSMUSG00000025961	0.048951636	0	0	0.068521293	0	0.130499513	0.021851453	0.072740354	0	0.131358247	0.142615545	0.086764288	4933402D24	RIKEN cDNA 4933402D24 gene [Source:MGI Symbol;Acc:MGI:1921676]
ENSMUSG00000025962	12.19258634	12.08126575	11.07958846	15.47926493	14.74670022	15.37013379	22.29791365	21.7927865	23.68640098	26.06022105	23.01249001	22.46930628	Fastkd2	FAST kinase domains 2 [Source:MGI Symbol;Acc:MGI:1922869]
ENSMUSG00000025963	0.050062231	0	0.128133363	0.023358626	0	0	0.06704163	0.173578205	0.046595871	0.134338452	0.125015272	0.177465525	Mdh1b	malate dehydrogenase 1B, NAD (soluble) [Source:MGI Symbol;Acc:MGI:1923918]
ENSMUSG00000025964	46.34036963	49.96828605	42.94861701	29.54849954	32.4086194	35.06909241	56.86279637	52.26266023	59.25775453	37.52373419	36.48715809	37.17756781	Adam23	a disintegrin and metallopeptidase domain 23 [Source:MGI Symbol;Acc:MGI:1345162]
ENSMUSG00000025967	1109.160958	853.7430845	1368.468907	1166.748196	1025.396634	788.981554	1427.656517	1547.413703	1151.413106	832.8049141	817.6404634	838.4021843	Eef1b2	eukaryotic translation elongation factor 1 beta 2 [Source:MGI Symbol;Acc:MGI:1929520]
ENSMUSG00000025968	69.04571303	78.51101545	59.04367488	84.602444	97.32054268	97.4328443	90.14090176	83.83083381	97.51502708	97.65202631	96.37238356	93.52907786	Ndufs1	NADH:ubiquinone oxidoreductase core subunit S1 [Source:MGI Symbol;Acc:MGI:2443241]
ENSMUSG00000025969	32.46674298	38.58414054	29.24646835	34.76522239	42.05944059	53.0302263	3.407277658	2.66082703	4.272568625	26.55676466	27.45476242	25.80745692	Nrp2	neuropilin 2 [Source:MGI Symbol;Acc:MGI:1100492]
ENSMUSG00000025971	4.622979914	3.191803835	5.120406548	4.199555562	4.169438441	3.497869091	2.2827938	2.55329537	1.646898791	2.287138482	2.017732229	2.003178497	Maip1	matrix AAA peptidase interacting protein 1 [Source:MGI Symbol;Acc:MGI:1915365]
ENSMUSG00000025977	1.884459321	1.653372784	1.378068306	1.50732555	2.35705518	3.520046012	0.120171598	0.400034024	0.542897779	2.247472489	3.510726064	2.584607721	Boll	boule homolog, RNA binding protein [Source:MGI Symbol;Acc:MGI:1922638]
ENSMUSG00000025978	34.8133794	31.14261613	34.40291666	26.84161937	27.72271323	23.63868681	0.568547421	0.483250744	0.395156897	3.227900485	3.198250844	3.480309505	Rftn2	raftlin family member 2 [Source:MGI Symbol;Acc:MGI:1921263]
ENSMUSG00000025979	67.1424281	69.22691961	67.56021128	82.01170816	79.43198978	81.94575506	37.16028666	35.69323106	34.94818186	42.13313628	37.66503561	42.59129132	Mob4	MOB family member 4, phocein [Source:MGI Symbol;Acc:MGI:104899]
ENSMUSG00000025980	415.4906618	428.5974624	380.5961915	520.0996897	531.4022629	534.2603726	795.9219253	774.9117521	823.4811376	734.0875537	720.1699998	709.794067	Hspd1	heat shock protein 1 (chaperonin) [Source:MGI Symbol;Acc:MGI:96242]
ENSMUSG00000025981	36.53636822	35.6702873	27.59971832	35.53501286	34.50269492	34.81408146	9.746664599	11.36304736	11.51616333	16.53576363	18.32040451	17.15722708	Coq10b	coenzyme Q10B [Source:MGI Symbol;Acc:MGI:1915126]
ENSMUSG00000025982	115.9626674	148.8747065	96.43969794	151.916768	177.3066494	213.3167169	153.3406208	143.7959095	181.7832649	213.5842582	204.2899462	205.1265462	Sf3b1	splicing factor 3b, subunit 1 [Source:MGI Symbol;Acc:MGI:1932339]

ENSMUSG00000025983	0.37268769	0.097538417	0.173434056	0.426828308	0.551755066	0.412901047	0.211735416	0.302072936	0.299580267	0.287902111	0.197415849	0.435375683	Ccdc150	coiled-coil domain containing 150 [Source:MGI Symbol;Acc:MGI:1925266]
ENSMUSG00000025986	14.24005934	17.39669514	10.41629561	17.24459706	19.47622134	29.55973021	38.62129305	30.17978738	49.64444757	53.93141369	53.26817889	52.76787975	Slc39a10	solute carrier family 39 (zinc transporter), member 10 [Source:MGI Symbol;Acc:MGI:1914515]
ENSMUSG00000025991	0.007745902	0.008919805	0.03965099	0.014456698	0.081383111	0.076698878	0.124476575	0.115101299	0.194658342	0.207855804	0.232116565	0.322636637	Cps1	carbamoyl-phosphate synthetase 1 [Source:MGI Symbol;Acc:MGI:891996]
ENSMUSG00000025993	6.54967055	7.148771943	5.21863189	4.623894867	4.697356705	5.097216975	1.728795551	1.269466014	1.192727875	9.450056601	9.29200211	9.160982397	Slc40a1	solute carrier family 40 (iron-regulated transporter), member 1 [Source:MGI Symbol;Acc:MGI:1315204]
ENSMUSG00000025995	57.38147491	57.51533719	51.29029697	66.74236011	67.7804603	78.54623643	85.15474026	81.14608967	96.66254135	111.8658739	112.3462758	109.0514654	Wdr75	WD repeat domain 75 [Source:MGI Symbol;Acc:MGI:1920924]
ENSMUSG00000025997	2.140022631	2.423946953	1.508515702	1.456852641	3.151497121	3.353558573	0.250565701	0.225901353	0.212245809	0.941409695	0.89067648	1.181451592	Ikzf2	IKAROS family zinc finger 2 [Source:MGI Symbol;Acc:MGI:1342541]
ENSMUSG00000026000	20.61821008	19.15943568	19.21612843	22.18035163	21.84110777	20.70467503	21.72519147	20.33183792	20.98147132	19.13430682	17.60224349	19.44259893	Lancl1	LanC (bacterial lantibiotic synthetase component C)-like 1 [Source:MGI Symbol;Acc:MGI:1336997]
ENSMUSG00000026003	160.4764725	153.205712	156.8095297	189.2502576	191.791798	171.0992909	33.59428392	36.0475797	33.43152087	33.29824059	32.49481142	30.94026016	Acadl	acyl-Coenzyme A dehydrogenase, long-chain [Source:MGI Symbol;Acc:MGI:87866]
ENSMUSG00000026004	3.049378354	3.899808555	2.041262701	4.172678276	4.990628669	7.101968356	0.693692424	0.624501346	1.214437644	1.429365702	1.659657603	2.117772901	Kansl1l	KAT8 regulatory NSL complex subunit 1-like [Source:MGI Symbol;Acc:MGI:1915941]
ENSMUSG00000026005	23.69459128	31.6665984	20.11279055	32.84529573	35.11007971	42.19612791	19.44578187	19.48358292	21.53625521	31.80132899	31.18697948	32.92758574	Rpe	ribulose-5-phosphate-3-epimerase [Source:MGI Symbol;Acc:MGI:1913896]
ENSMUSG00000026009	0	0	0.02174346	0	0	0	0	0	0	0.037994044	0	0	Icos	inducible T cell co-stimulator [Source:MGI Symbol;Acc:MGI:1858745]
ENSMUSG00000026011	0	0	0	0	0	0	0	0	0	0.107010101	0	0	Ctla4	cytotoxic T-lymphocyte-associated protein 4 [Source:MGI Symbol;Acc:MGI:88556]
ENSMUSG00000026012	0	0.021885828	0.019457708	0.017735635	0	0.014476158	0.016967692	0.037655342	0	0.186999837	0.189842096	0.11790209	Cd28	CD28 antigen [Source:MGI Symbol;Acc:MGI:88327]
ENSMUSG00000026014	30.80082901	38.80365896	25.94641958	42.26745094	49.03540602	69.35190607	12.46787815	12.12598898	15.93340458	34.78077966	35.68830757	36.20515689	Raph1	Ras association (RalGDS/AF-6) and pleckstrin homology domains 1 [Source:MGI Symbol;Acc:MGI:1924550]
ENSMUSG00000026017	3.470883291	4.900722259	3.732030925	4.996804903	5.497566074	6.735479717	2.242293874	2.004296041	2.353922566	4.430718464	4.500701823	4.142528762	Carf	calcium response factor [Source:MGI Symbol;Acc:MGI:2182269]
ENSMUSG00000026018	0.567137662	0.709878447	0.328182953	0.483222369	0.259073458	0.770048622	0.484313346	0.341983878	0.252458835	0.92635793	1.067322364	0.9396647	Ica1l	islet cell autoantigen 1-like [Source:MGI Symbol;Acc:MGI:1917625]
ENSMUSG00000026019	19.74060468	18.89253996	17.28678573	22.19530659	21.46742899	21.27744538	37.385602	43.55708015	38.81102854	40.5662531	38.17907666	38.30580739	Wdr12	WD repeat domain 12 [Source:MGI Symbol;Acc:MGI:1927241]
ENSMUSG00000026020	87.78743302	104.1191291	94.30520383	141.3911714	149.6454776	146.1523612	269.8249843	261.062713	280.808668	254.3539618	257.6432783	256.2248074	Nop58	NOP58 ribonucleoprotein [Source:MGI Symbol;Acc:MGI:1933184]
ENSMUSG00000026021	270.9242159	231.2097888	266.5274674	260.9458618	224.7599126	199.6427778	299.0517184	330.679901	293.7625908	240.5497898	226.9940639	228.2568844	Sumo1	small ubiquitin-like modifier 1 [Source:MGI Symbol;Acc:MGI:1197010]
ENSMUSG00000026023	16.89040108	12.76142054	17.13577761	16.01146674	12.125144	14.69882808	0.102348634	0.113567978	0.177838151	0.751985133	0.445325272	0.270926499	Cdk15	cyclin-dependent kinase 15 [Source:MGI Symbol;Acc:MGI:3583944]
ENSMUSG00000026024	6.597701467	10.76325589	6.534417113	9.848755813	12.93460219	16.78758168	15.78210255	11.87603479	18.16674012	20.39862218	20.07734853	19.95622012	Als2	alsin Rho guanine nucleotide exchange factor [Source:MGI Symbol;Acc:MGI:1921268]
ENSMUSG00000026027	13.71832292	15.17408969	13.72503586	15.24938563	14.32580964	14.99962435	7.136576663	6.537200639	7.362688717	4.859873838	5.198385411	5.368493113	Stradb	STE20-related kinase adaptor beta [Source:MGI Symbol;Acc:MGI:2144047]
ENSMUSG00000026028	34.65245566	48.74572966	29.29901099	41.56126591	47.66863499	66.11399767	22.83583162	20.78874414	26.86709171	37.92404012	37.55992593	38.25263123	Trak2	trafficking protein, kinesin binding 2 [Source:MGI Symbol;Acc:MGI:1918077]
ENSMUSG00000026029	33.75417717	37.404296	30.27350126	38.60369448	41.10671152	40.51164656	5.911458747	5.469782963	6.002354038	12.28914014	13.33931753	12.4433689	Casp8	caspase 8 [Source:MGI Symbol;Acc:MGI:1261423]
ENSMUSG00000026031	69.71555542	81.14748542	67.73936178	86.27512287	105.1155162	124.935283	8.060821838	7.220772015	7.703443353	37.09987578	38.6156805	36.19500298	Cflar	CASP8 and FADD-like apoptosis regulator [Source:MGI Symbol;Acc:MGI:1336166]
ENSMUSG00000026032	74.721817	59.09575258	87.29620223	73.72873191	70.80464014	49.13955388	88.10746347	91.11763774	61.67464356	59.27045935	48.06878177	50.47737056	Ndufb3	NADH:ubiquinone oxidoreductase subunit B3 [Source:MGI Symbol;Acc:MGI:1913745]
ENSMUSG00000026034	52.21876576	57.64055345	51.33194928	60.76786946	65.67165252	71.11384491	74.29548198	69.49321741	77.90096227	77.25246287	77.92685318	77.76024065	Clk1	CDC-like kinase 1 [Source:MGI Symbol;Acc:MGI:107403]
ENSMUSG00000026035	29.16790118	23.05503493	34.06643643	27.48030858	28.24294075	21.754556	16.37422739	15.16407597	14.81210485	17.57422326	16.89841739	17.90840318	Ppil3	peptidylprolyl isomerase (cyclophilin)-like 3 [Source:MGI Symbol;Acc:MGI:1917475]
ENSMUSG00000026036	25.34590228	22.15119742	26.5055657	29.61983383	28.61561426	27.35112312	29.70101533	31.05890588	28.53080349	38.25639016	36.80819694	38.43117651	Nif3l1	Ngg1 interacting factor 3-like 1 (S. pombe) [Source:MGI Symbol;Acc:MGI:1929485]
ENSMUSG00000026037	21.68180033	24.35026819	20.99686692	28.67658814	32.35704405	35.99016285	64.65303711	58.09597623	71.73913435	81.74196596	80.0858641	80.75028649	Orc2	origin recognition complex, subunit 2 [Source:MGI Symbol;Acc:MGI:1328306]
ENSMUSG00000026039	4.450126788	4.246055513	3.89430136	4.844916065	5.488229733	5.996330296	9.232429838	8.607041397	9.546313389	6.387808258	6.323737926	6.788990419	Sgo2a	shugoshin 2A [Source:MGI Symbol;Acc:MGI:1098767]
ENSMUSG00000026042	292.5387749	361.7314437	241.961721	140.7935286	164.5181746	189.1113852	4.892214279	4.339930058	6.096191411	78.20454726	79.67329156	77.45746265	Col5a2	collagen, type V, alpha 2 [Source:MGI Symbol;Acc:MGI:88458]
ENSMUSG00000026043	1654.526273	1917.001697	1477.943248	1311.628455	1404.808485	1504.92212	3.229567378	3.654317696	3.167604352	21.3514548	21.37522602	21.44975889	Col3a1	collagen, type III, alpha 1 [Source:MGI Symbol;Acc:MGI:88453]
ENSMUSG00000026047	11.97992495	14.93832184	8.539508138	15.50131517	17.55204891	18.9337011	11.81346418	10.86128135	14.09747728	14.01291692	15.22364724	13.44389062	Poglut2	protein O-glucosyltransferase 2 [Source:MGI Symbol;Acc:MGI:1919300]
ENSMUSG00000026048	29.04190593	31.16910675	26.3076613	31.04739755	33.29597528	34.34901407	25.34716613	24.92645414	26.55526894	30.63243112	29.10604622	29.2585624	Ercc5	excision repair cross-complementing rodent repair deficiency, complementation group 5 [Source:MGI Symbol;Acc:MGI:103582]
ENSMUSG00000026049	22.45418661	20.02425813	25.39419763	24.31624447	25.84116255	18.49504805	27.73888128	32.64188095	23.62122286	17.09538312	15.30041283	15.36422859	Tex30	testis expressed 30 [Source:MGI Symbol;Acc:MGI:1922873]
ENSMUSG00000026051	0.906365043	0.769061218	0.830253053	0.311612356	0.300720521	0.072669669	0.08517706	0.141771112	0	0.213347929	0.317666301	0.507311371	Ecrg4	ECRG4 augurin precursor [Source:MGI Symbol;Acc:MGI:1926146]
ENSMUSG00000026058	0.036981941	0.170346422	0.056792759	0.155299227	0.038855452	0.070421187	0.049524952	0	0.10326382	0.264635781	0.215486144	0.147484376	Khdrbs2	KH domain containing, RNA binding, signal transduction associated 2 [Source:MGI Symbol;Acc:MGI:2159649]
ENSMUSG00000026062	0.046200953	0.035468515	0	0.143713235	0.097083003	0.082111004	0.068745265	0.015256209	0.028667968	0.096426544	0.205107376	0.163777687	Slc9a2	solute carrier family 9 (sodium/hydrogen exchanger), member 2 [Source:MGI Symbol;Acc:MGI:105075]
ENSMUSG00000026063	0	0	0	0	0	0.022587195	0	0	0	0	0	0	Pih1d3	PIH1 domain containing 3 [Source:MGI Symbol;Acc:MGI:1921958]
ENSMUSG00000026064	0.066325222	0.171848031	0.067903276	0.154734019	0.365847663	0.606224659	0.296068239	0.312096765	0.601894763	0.415284416	0.234638491	0.279200458	Ptp4a1	protein tyrosine phosphatase 4a1 [Source:MGI Symbol;Acc:MGI:1277096]
ENSMUSG00000026065	2.186653814	1.734652721	1.243711186	4.285153761	4.467225133	4.182342554	0	0	0	0	0	0	Slc9a4	solute carrier family 9 (sodium/hydrogen exchanger), member 4 [Source:MGI Symbol;Acc:MGI:105074]
ENSMUSG00000026068	10.75254251	11.83366465	9.639359984	16.63232607	18.82236498	21.8074901	0.637794512	0.671416077	0.903620703	5.062912562	4.726790719	5.292178343	Il18rap	interleukin 18 receptor accessory protein [Source:MGI Symbol;Acc:MGI:1338888]
ENSMUSG00000026069	75.27407491	91.95752283	67.69960669	82.3414352	92.76914077	103.8535895	4.339918467	3.473109124	4.264048656	35.49694673	36.16020739	35.02587582	Il1rl1	interleukin 1 receptor-like 1 [Source:MGI Symbol;Acc:MGI:98427]
ENSMUSG00000026070	0.153429632	0.078525381	0.08726674	0.07954334	0.017911378	0.155819433	0.015219831	0	0	0	0.014190521	0	Il18r1	interleukin 18 receptor 1 [Source:MGI Symbol;Acc:MGI:105383]
ENSMUSG00000026072	28.95255586	34.22230569	24.67543788	23.16296901	25.57903233	29.07059166	0.818439448	0.632260416	0.831657138	8.127313287	8.345679723	6.910817678	Il1r1	interleukin 1 receptor, type I [Source:MGI Symbol;Acc:MGI:96545]
ENSMUSG00000026073	3.298850733	2.367365746	3.817861684	1.427679212	1.35624955	1.310960825	0.042683171	0	0.177996229	2.651393239	2.507181284	1.906645234	Il1r2	interleukin 1 receptor, type II [Source:MGI Symbol;Acc:MGI:96546]
ENSMUSG00000026074	153.3198992	191.7053673	148.9674943	177.8383687	202.5124808	232.8537659	87.53288128	74.57073586	95.63921518	141.2464127	144.4037614	140.2764921	Map4k4	mitogen-activated protein kinase kinase kinase kinase 4 [Source:MGI Symbol;Acc:MGI:1349394]
ENSMUSG00000026077	0.671154923	1.059117318	0.941613667	0.069590083	0.261168795	0.151468611	0.2219229	0.098499937	0.370182799	0.667035812	0.682817383	0.5727646	Npas2	neuronal PAS domain protein 2 [Source:MGI Symbol;Acc:MGI:109232]
ENSMUSG00000026078	79.88896883	74.63013464	81.54802694	80.72005729	76.58748033	63.05289978	49.79703306	51.83513578	44.5265258	40.28301556	40.28784347	39.88523982	Pdcl3	phosducin-like 3 [Source:MGI Symbol;Acc:MGI:1916083]
ENSMUSG00000026080	1.915132824	1.618331922	1.861958702	1.517166309	1.896346367	1.679104483	2.779941409	3.111973511	3.590702026	3.019388972	3.188309761	3.211320504	Chst10	carbohydrate sulfotransferase 10 [Source:MGI Symbol;Acc:MGI:2138283]
ENSMUSG00000026082	12.23144832	20.66659154	8.161965138	19.15952749	22.94838495	33.46721058	18.68742901	15.57899002	22.83682583	40.53935544	42.21058168	41.74617485	Rev1	REV1, DNA directed polymerase [Source:MGI Symbol;Acc:MGI:1929074]
ENSMUSG00000026083	109.6763315	100.7959325	129.8004006	106.8925917	98.29049253	89.24667263	110.1075465	119.9112643	104.6685751	103.538404	101.1423705	101.2400014	Eif5b	eukaryotic translation initiation factor 5B [Source:MGI Symbol;Acc:MGI:2441772]
ENSMUSG00000026085	0.123822987	0	0.190153598	0.34664873	0	0	0	0	0	0	0	0	Lyg1	lysozyme G-like 1 [Source:MGI Symbol;Acc:MGI:1916791]
ENSMUSG00000026087	129.2505344	103.2175971	151.3662357	124.516859	103.4819563	91.28109584	109.7426905	119.4831946	97.61340512	85.88427279	79.42002824	82.45874914	Mrpl30	mitochondrial ribosomal protein L30 [Source:MGI Symbol;Acc:MGI:1333820]
ENSMUSG00000026088	44.37734325	45.71810062	41.29035363	46.23733811	53.05009562	48.93842789	35.36414466	34.92023469	35.36210349	32.17384859	28.44391365	29.68549007	Mitd1	MIT, microtubule interacting and transport, domain containing 1 [Source:MGI Symbol;Acc:MGI:1916278]
ENSMUSG00000026090	0.356813695	0.522950033	0.398512629	0.18162146	0.10224271	0.259424965	0.695029399	0.739083677	0.784981485	0.725366875	0.810031108	0.488699118	2010300C02	RIKEN cDNA 2010300C02 gene [Source:MGI Symbol;Acc:MGI:1919347]
ENSMUSG00000026094	26.5431317	30.27069664	21.92774538	32.04307313	35.60575743	40.58123151	13.26885282	11.06368156	15.00600963	23.13098128	21.11684768	21.57395104	Stk17b	serine/threonine kinase 17b (apoptosis-inducing) [Source:MGI Symbol;Acc:MGI:2138162]
ENSMUSG00000026095	1.962211171	1.694690274	3.94604847	2.942844467	2.429763038	1.601336513	2.408748993	3.262890622	1.304532623	1.567099608	1.020839285	0.77632135	Asnsd1	asparagine synthetase domain containing 1 [Source:MGI Symbol;Acc:MGI:1917646]
ENSMUSG00000026096	4.699435272	3.458170776	3.70818089	4.513789996	5.925011355	4.696969393	5.157455102	4.99610362	5.803606401	5.085250751	4.961314664	5.302444392	Osgepl1	O-sialoglycoprotein endopeptidase-like 1 [Source:MGI Symbol;Acc:MGI:1919335]
ENSMUSG00000026097	8.698900066	7.532798404	9.117914602	8.488119863	9.447867573	7.677508244	7.057361879	7.745490978	7.261218006	5.11011507	5.674956203	4.894717008	Ormdl1	ORM1-like 1 (S. cerevisiae) [Source:MGI Symbol;Acc:MGI:2181669]
ENSMUSG00000026098	2.282454168	3.666729054	2.509853155	4.680628065	5.210650969	6.954016167	16.47788436	14.23653067	16.44454355	15.14817799	15.41040418	17.23097854	Pms1	PMS1 homolog 1, mismatch repair system component [Source:MGI Symbol;Acc:MGI:1202302]
ENSMUSG00000026100	0.033316677	0	0	0	0.017502253	0	0	0	0	0	0	0	Mstn	myostatin [Source:MGI Symbol;Acc:MGI:95691]
ENSMUSG00000026102	13.3089425	18.21577868	11.40277774	19.44522944	21.35989353	25.68658498	4.07811688	3.743029877	4.487814489	9.054508471	8.660830309	7.596594381	Inpp1	inositol polyphosphate-1-phosphatase [Source:MGI Symbol;Acc:MGI:104848]
ENSMUSG00000026103	80.84494264	103.4860878	66.68855041	103.4480963	122.413616	145.8719854	40.8353446	40.91247848	52.73174541	65.97937092	59.94122398	66.59276946	Gls	glutaminase [Source:MGI Symbol;Acc:MGI:95752]
ENSMUSG00000026104	30.76996133	26.80881018	32.69559456	27.09865253	22.56704233	21.19009955	5.18821305	6.474369314	4.80063721	7.220443609	7.689747896	8.828800593	Stat1	signal transducer and activator of transcription 1 [Source:MGI Symbol;Acc:MGI:103063]
ENSMUSG00000026107	23.42477523	27.42122486	20.01344185	34.21055641	37.5475351	43.20659177	18.29278765	15.54353091	18.98511902	40.04003366	39.61214285	42.94246024	Nabp1	nucleic acid binding protein 1 [Source:MGI Symbol;Acc:MGI:1923258]

ENSMUSG00000026109	0.466252964	0.715885675	0.545538702	0.455818634	0.349909585	0.52424904	0.198219061	0.109973807	0.103325984	0.436911899	0.332664478	0.41320485	Tmeff2	transmembrane protein with EGF-like and two follistatin-like domains 2 [Source:MGI Symbol;Acc:MGI:1861735]
ENSMUSG00000026110	3.836595819	3.789080607	3.546001564	3.07056007	4.2129114	4.616773805	4.864295763	4.143813058	4.748862239	5.076121881	4.380082295	5.253604516	Mgat4a	mannoside acetylglucosaminyltransferase 4, isoenzyme A [Source:MGI Symbol;Acc:MGI:2662992]
ENSMUSG00000026111	69.66741992	68.36854132	63.00777388	71.13909245	69.07791344	62.16630918	36.64380701	35.3265807	34.86166538	37.34726923	41.83222339	36.79343932	Unc50	unc-50 homolog [Source:MGI Symbol;Acc:MGI:1914637]
ENSMUSG00000026112	47.83622993	54.38225986	44.03578822	51.34723511	58.67282871	70.09182366	30.0744093	26.64911928	33.40422504	43.6369544	47.11337881	45.82788546	Coa5	cytochrome C oxidase assembly factor 5 [Source:MGI Symbol;Acc:MGI:1923428]
ENSMUSG00000026113	6.366539159	6.955886531	6.788275292	6.738133235	7.733216491	10.15982812	5.046195194	4.676383147	6.30149046	6.222522171	5.855302992	6.068787791	Inpp4a	inositol polyphosphate-4-phosphatase, type I [Source:MGI Symbol;Acc:MGI:1931123]
ENSMUSG00000026114	0	0.023361394	0.041539133	0.056794169	0.021314624	0.03090431	1.394598663	1.46708481	1.831573643	2.050514003	2.566791642	2.247342332	Cnga3	cyclic nucleotide gated channel alpha 3 [Source:MGI Symbol;Acc:MGI:1341818]
ENSMUSG00000026116	35.94144277	40.57230926	31.66579397	35.85469374	45.13740334	53.37666969	71.90356803	63.72146331	87.27573922	86.54509772	91.87379706	88.81228272	Tmem131	transmembrane protein 131 [Source:MGI Symbol;Acc:MGI:1927110]
ENSMUSG00000026117	0.284250952	0.245497187	0.181883775	0.298415607	0.298651153	0.433017614	11.73699092	10.31327281	8.466212048	3.877400411	4.673052292	4.093540553	Zap70	zeta-chain (TCR) associated protein kinase [Source:MGI Symbol;Acc:MGI:99613]
ENSMUSG00000026121	18.23173979	20.53936723	15.18345021	19.52315237	20.17338103	18.67637205	4.078047468	4.720969345	4.969334058	7.269556148	6.732124113	6.694287496	Sema4c	sema domain, immunoglobulin domain (Ig), transmembrane domain (TM) and short cytoplasmic domain, (semaphorin) 4C [Source:M
ENSMUSG00000026123	63.09583973	76.03400397	58.92351532	70.82896991	75.45318012	86.58103913	19.04701336	18.66168981	19.75171341	30.33359284	30.21212485	30.17108942	Plekhb2	pleckstrin homology domain containing, family B (evectins) member 2 [Source:MGI Symbol;Acc:MGI:2385825]
ENSMUSG00000026124	0	0	0	0	0	0.031513481	0	0.040986392	0	0.3700766	0.344393033	0.4033288	Cfc1	cripto, FRL-1, cryptic family 1 [Source:MGI Symbol;Acc:MGI:109448]
ENSMUSG00000026125	0	0	0	0	0	0	0	0.140370519	0	0.126744126	0.196579997	0.167433171	Prss39	protease, serine 39 [Source:MGI Symbol;Acc:MGI:1270856]
ENSMUSG00000026126	0.471360587	0.193855695	0.172348397	0.282770971	0.141497084	0.179513415	1.021991423	0.667071169	0.532735192	0.542084033	0.868797449	0.596759072	Ptpn18	protein tyrosine phosphatase, non-receptor type 18 [Source:MGI Symbol;Acc:MGI:108410]
ENSMUSG00000026127	27.12365921	29.42498461	23.90279047	32.02503517	32.87087849	35.16751093	47.91397878	43.3904369	48.20778713	68.76549533	68.69681306	61.7064172	Imp4	IMP4, U3 small nucleolar ribonucleoprotein [Source:MGI Symbol;Acc:MGI:106572]
ENSMUSG00000026131	25.97606078	33.35974856	23.2756092	33.65148666	39.40402751	55.78410535	13.13543418	11.26935159	17.18341035	35.01381351	37.79236409	37.21867417	Dst	dystonin [Source:MGI Symbol;Acc:MGI:104627]
ENSMUSG00000026134	18.57018948	16.89445727	17.52009401	24.12884046	24.18696757	22.22933574	35.58428588	35.60282474	37.2081362	28.27148102	27.70275495	28.42952025	Prim2	DNA primase, p58 subunit [Source:MGI Symbol;Acc:MGI:97758]
ENSMUSG00000026135	2.824879114	5.210908105	1.758826207	4.388042134	6.317441482	8.046801115	2.205751036	2.01768779	3.412296508	8.324933661	8.860235506	9.032923091	Zfp142	zinc finger protein 142 [Source:MGI Symbol;Acc:MGI:1924514]
ENSMUSG00000026141	0.019792313	0.022791866	0	0.018469861	0.010397497	0.007537723	0	0	0	0	0.016475104	0.017540435	Col19a1	collagen, type XIX, alpha 1 [Source:MGI Symbol;Acc:MGI:1095415]
ENSMUSG00000026142	5.789197296	7.50880012	4.5943286	6.270004318	7.606354917	7.90315623	2.40162009	2.149099207	2.607637063	5.843606534	6.160378756	5.405182687	Rhbdd1	rhomboid domain containing 1 [Source:MGI Symbol;Acc:MGI:1924117]
ENSMUSG00000026147	0	0	0.020498953	0.037369454	0.105184547	0	0	0	0	0.071638839	0.066667055	0.08872244	Col9a1	collagen, type IX, alpha 1 [Source:MGI Symbol;Acc:MGI:88465]
ENSMUSG00000026149	0	0.04692189	0	0	0	0	0	0	0	0	0.135669983	0.288885642	Tm4sf20	transmembrane 4 L six family member 20 [Source:MGI Symbol;Acc:MGI:1913511]
ENSMUSG00000026150	58.70530053	54.40051715	50.09018099	55.40056767	53.58608364	53.88232344	47.54845574	49.39232842	52.39997378	49.20000177	48.74100677	47.86612861	Mff	mitochondrial fission factor [Source:MGI Symbol;Acc:MGI:1922984]
ENSMUSG00000026153	10.90169866	14.73591543	9.20852013	14.72026268	18.59009488	26.23233736	5.349738042	4.570973088	7.650223968	13.69145844	13.76547917	14.64469288	Fam135a	family with sequence similarity 135, member A [Source:MGI Symbol;Acc:MGI:1915437]
ENSMUSG00000026154	18.76197127	15.44516433	26.98696409	17.36366609	16.61711161	12.75529452	14.88142996	16.66632243	12.70027147	10.12476232	8.002328936	9.344279347	Sdhaf4	succinate dehydrogenase complex assembly factor 4 [Source:MGI Symbol;Acc:MGI:1915252]
ENSMUSG00000026155	13.5702647	20.30717853	7.04973559	18.086305	21.77481063	27.50356923	12.235176	11.61313103	17.13265811	18.02715053	15.96521147	15.83661615	Smap1	small ArfGAP 1 [Source:MGI Symbol;Acc:MGI:2138261]
ENSMUSG00000026156	0.174439816	0.619368945	0.253002788	0.501918473	0.473465496	1.018651993	0.532097641	0.331214357	0.378843325	0.520107654	0.54451331	0.373599404	B3gat2	beta-1,3-glucuronyltransferase 2 (glucuronosyltransferase S) [Source:MGI Symbol;Acc:MGI:2389490]
ENSMUSG00000026158	9.014681212	11.51903591	10.9389152	14.91642068	14.62780043	15.51087313	5.84210058	6.178637726	5.376895987	6.950698085	7.305224108	6.675146088	Ogfrl1	opioid growth factor receptor-like 1 [Source:MGI Symbol;Acc:MGI:1917405]
ENSMUSG00000026159	23.44418767	28.76857014	22.39796634	28.35066806	29.77972642	37.89462234	39.26682204	37.56041264	47.6718798	53.58503362	57.65556619	54.22673239	Agfg1	ArfGAP with FG repeats 1 [Source:MGI Symbol;Acc:MGI:1333754]
ENSMUSG00000026162	8.467144295	9.041235409	12.1360403	9.840834461	8.329976368	6.214751677	10.72042439	11.66682415	7.307716378	8.399878171	9.354675885	8.254170782	Nhej1	non-homologous end joining factor 1 [Source:MGI Symbol;Acc:MGI:1922820]
ENSMUSG00000026163	0.010308694	0.023741982	0	0.067339326	0.010830934	0.023555837	0	0	0	0.110650598	0.060066634	0.146173089	Sphkap	SPHK1 interactor, AKAP domain containing [Source:MGI Symbol;Acc:MGI:1924879]
ENSMUSG00000026166	3.289904502	2.791522007	3.279543777	0.121187559	0.090962411	0.131887407	0.386467345	0.214415733	0.201454485	0.271042017	0.396363813	0.882350913	Ccl20	chemokine (C-C motif) ligand 20 [Source:MGI Symbol;Acc:MGI:1329031]
ENSMUSG00000026167	0.304969952	0	0.056768364	0.051744172	0.029129071	0.042234563	1.039577236	0.466906837	0.77414597	0.173592632	0.346168255	0.515973581	Wnt10a	wingless-type MMTV integration site family, member 10A [Source:MGI Symbol;Acc:MGI:108071]
ENSMUSG00000026170	3.226164851	2.327529189	2.427450101	1.88616156	1.71522075	1.125034132	4.129510058	4.08160782	3.94341239	4.867493489	4.691460654	4.684801299	Cyp27a1	cytochrome P450, family 27, subfamily a, polypeptide 1 [Source:MGI Symbol;Acc:MGI:88594]
ENSMUSG00000026171	102.3582635	80.06193488	94.82733376	90.46626934	83.75945461	68.26824111	43.35170279	53.92426516	44.71351054	39.56995739	41.85827616	34.457444	Rnf25	ring finger protein 25 [Source:MGI Symbol;Acc:MGI:1890215]
ENSMUSG00000026172	19.538446	17.26287789	18.23737472	18.05735847	18.02401613	16.74532687	16.2955139	15.93809028	16.22983108	14.81169551	14.72355523	15.98135583	Bcs1l	BCS1-like (yeast) [Source:MGI Symbol;Acc:MGI:1914071]
ENSMUSG00000026173	0.329900783	0.596982116	0.337750002	0.30785799	0.478656894	0.406833407	0.378678737	0.31125117	0.497141713	0.491814101	0.588448118	0.431588187	Plcd4	phospholipase C, delta 4 [Source:MGI Symbol;Acc:MGI:107469]
ENSMUSG00000026174	55.69327673	61.02157097	54.6584262	63.93897394	72.82325172	72.69213198	78.41455742	75.80261586	86.75672109	71.40655337	77.65469123	71.64008141	Cnot9	CCR4-NOT transcription complex, subunit 9 [Source:MGI Symbol;Acc:MGI:1928902]
ENSMUSG00000026175	0.836858758	1.325068084	0.892468602	0.423010707	0.549533854	0.504624305	3.486599397	3.039768718	2.985836256	2.339219998	2.263951611	2.317640074	Vil1	villin 1 [Source:MGI Symbol;Acc:MGI:98930]
ENSMUSG00000026176	53.8530038	59.19674662	60.69657027	62.13629751	58.84706885	56.16598428	13.97745293	12.44880179	12.50691575	14.85728244	14.56840622	13.45218614	Ctdsp1	CTD (carboxy-terminal domain, RNA polymerase II, polypeptide A) small phosphatase 1 [Source:MGI Symbol;Acc:MGI:2654470]
ENSMUSG00000026177	12.25872837	9.748108788	12.01098087	1.114463875	0.885713992	1.0434178	2.508725164	2.505355946	3.007773012	14.45262411	14.0636058	14.0080058	Slc11a1	solute carrier family 11 (proton-coupled divalent metal ion transporters), member 1 [Source:MGI Symbol;Acc:MGI:1345275]
ENSMUSG00000026179	29.02404166	25.58508501	34.43870284	28.16125952	27.39163525	22.423405	36.04610972	37.3004737	29.2047432	27.3027236	24.98539142	25.6195869	Pnkd	paroxysmal nonkinesiogenic dyskinesia [Source:MGI Symbol;Acc:MGI:1930773]
ENSMUSG00000026180	0.016342001	0.018818654	0.01673082	0	0.051509668	0.037342219	0.014589766	0.04856724	0	0	0	0.014482683	Cxcr2	chemokine (C-X-C motif) receptor 2 [Source:MGI Symbol;Acc:MGI:105303]
ENSMUSG00000026181	17.53607778	22.72947523	16.76848821	15.88439784	18.86353814	21.60857169	25.44248647	21.89411303	26.68946071	34.97991116	35.85701393	33.91649087	Ppm1f	protein phosphatase 1F (PP2C domain containing) [Source:MGI Symbol;Acc:MGI:1918464]
ENSMUSG00000026182	0	0	0.403224258	0	0	0.149995518	0	0	0	0	0	0	Tnp1	transition protein 1 [Source:MGI Symbol;Acc:MGI:98784]
ENSMUSG00000026185	6.248499302	8.42842655	5.369510279	2.935013154	3.330861713	4.173208304	0.52275268	0.480618034	0.389280244	7.893624518	7.394541555	7.116560751	Igfbp5	insulin-like growth factor binding protein 5 [Source:MGI Symbol;Acc:MGI:96440]
ENSMUSG00000026187	24.62870292	25.21213094	22.62235995	32.7812426	34.90387231	35.2683949	63.52240483	62.79327505	67.59178103	55.5954069	56.73932647	54.21484762	Xrcc5	X-ray repair complementing defective repair in Chinese hamster cells 5 [Source:MGI Symbol;Acc:MGI:104517]
ENSMUSG00000026188	2.468264056	1.995099085	1.94383895	2.56911386	3.067083925	3.579291829	0.402582585	0.282134679	0.353439837	0.509493212	0.434622868	0.652024294	Tmem169	transmembrane protein 169 [Source:MGI Symbol;Acc:MGI:2442781]
ENSMUSG00000026189	18.66612608	14.12817146	17.00183587	18.19582244	18.36875664	17.18800471	58.24818504	53.26060604	52.81430878	27.31783357	29.03282371	26.21151799	Pecr	peroxisomal trans-2-enoyl-CoA reductase [Source:MGI Symbol;Acc:MGI:2148199]
ENSMUSG00000026192	49.02260644	57.28872233	40.64295316	63.11177497	65.26791927	71.1589782	101.1903473	97.54761301	112.502049	114.832061	118.1688096	113.8800207	Atic	5-aminoimidazole-4-carboxamide ribonucleotide formyltransferase/IMP cyclohydrolase [Source:MGI Symbol;Acc:MGI:1351352]
ENSMUSG00000026193	1855.279718	2347.769071	1746.564986	2019.545013	2359.031126	2966.000741	126.9589303	109.6421095	160.0495828	502.1897795	555.0037863	539.8335142	Fn1	fibronectin 1 [Source:MGI Symbol;Acc:MGI:95566]
ENSMUSG00000026196	2.828520493	3.441060194	2.895818532	4.569506932	5.943627646	6.364843379	11.24817339	9.332642746	12.15841002	8.957174715	8.734279583	8.834111689	Bard1	BRCA1 associated RING domain 1 [Source:MGI Symbol;Acc:MGI:1328361]
ENSMUSG00000026197	24.40143682	21.82538188	31.56124419	26.5517045	23.33806045	18.97629983	22.82441005	24.49602287	19.98123252	10.82994725	11.93895849	10.73003812	Zfand2b	zinc finger, AN1 type domain 2B [Source:MGI Symbol;Acc:MGI:1916068]
ENSMUSG00000026198	0.39946872	0.262862152	0.155799293	0.266269747	0.219845934	0.130400613	0.169826941	0.131910227	0.318693513	0.238210225	0.1266733	0.252870721	Abcb6	ATP-binding cassette, sub-family B (MDR/TAP), member 6 [Source:MGI Symbol;Acc:MGI:1921354]
ENSMUSG00000026199	10.05835191	12.10404453	10.76116267	12.08645399	12.96688728	12.48891088	13.10955221	11.15369848	14.19857842	12.09571496	12.02634337	10.86770339	Ankzf1	ankyrin repeat and zinc finger domain containing 1 [Source:MGI Symbol;Acc:MGI:1098746]
ENSMUSG00000026200	12.58779953	15.36662193	9.975263406	14.57046031	18.24864608	18.85312861	1.377747931	0.449639744	0.873082769	3.16673141	3.375148604	3.164333863	Glb1l	galactosidase, beta 1-like [Source:MGI Symbol;Acc:MGI:1921827]
ENSMUSG00000026201	92.87519428	88.75411096	94.04730931	90.53878407	88.52892319	68.70613748	52.76901261	54.26352561	54.0277633	42.27907227	46.32418156	44.17537781	Stk16	serine/threonine kinase 16 [Source:MGI Symbol;Acc:MGI:1313271]
ENSMUSG00000026202	159.0135112	123.3213502	146.022253	141.6735916	125.2340208	109.8744826	172.306764	183.1887846	166.4478459	104.7361212	107.6656506	100.7938356	Tuba4a	tubulin, alpha 4A [Source:MGI Symbol;Acc:MGI:1095410]
ENSMUSG00000026203	36.73248435	37.49260034	47.00806003	44.37332595	39.54357353	36.74823494	17.63915692	16.60926321	13.85694167	12.8189508	13.37703838	14.61196186	Dnajb2	DnaJ heat shock protein family (Hsp40) member B2 [Source:MGI Symbol;Acc:MGI:1928739]
ENSMUSG00000026204	65.28460222	63.85297186	61.93355767	41.26115998	37.1019992	36.03527786	14.26837437	15.2865109	15.74345956	57.03044475	57.87160176	61.80160415	Ptprn	protein tyrosine phosphatase, receptor type, N [Source:MGI Symbol;Acc:MGI:102765]
ENSMUSG00000026205	0.603062198	0.347228561	0.463057991	0.16883032	0.285126044	0.229670782	0.08076003	0.089612854	0.084195834	0.215769959	0.150596533	0.240501852	Slc23a3	solute carrier family 23 (nucleobase transporters), member 3 [Source:MGI Symbol;Acc:MGI:104516]
ENSMUSG00000026207	59.4805567	57.454474	66.05874417	66.83299453	68.47730573	66.78948145	9.706427031	9.117828385	9.869510157	9.862112595	9.305364708	9.091401073	Speg	SPEG complex locus [Source:MGI Symbol;Acc:MGI:109282]
ENSMUSG00000026208	18.00029704	14.52258237	18.6560853	14.62011717	13.01672691	12.20826979	32.28930923	33.37969868	31.31021465	8.696441724	9.480257551	7.976153079	Des	desmin [Source:MGI Symbol;Acc:MGI:94885]
ENSMUSG00000026209	119.86753	104.6837488	120.148903	107.2043293	101.547062	91.64150615	86.62534239	85.66057643	79.04185061	55.80908953	57.83380373	60.04941826	Dnpep	aspartyl aminopeptidase [Source:MGI Symbol;Acc:MGI:1278328]
ENSMUSG00000026211	33.56134594	34.21635759	29.95015791	30.03349562	28.96732187	31.59162457	38.6100515	37.12434093	38.6449892	16.09532334	17.81743958	17.32256951	Obsl1	obscurin-like 1 [Source:MGI Symbol;Acc:MGI:2138628]
ENSMUSG00000026213	15.5590341	18.66558264	16.33711618	20.62464212	23.04478606	23.44652159	23.79399587	23.36943405	25.450338	19.86294171	19.18262483	19.43811808	Stk11ip	serine/threonine kinase 11 interacting protein [Source:MGI Symbol;Acc:MGI:1918978]
ENSMUSG00000026219	89.36298662	106.4425632	83.03608627	97.89367783	114.025817	144.2363685	144.3104034	128.343036	176.4099521	265.4378742	274.8847409	264.3871477	Trip12	thyroid hormone receptor interactor 12 [Source:MGI Symbol;Acc:MGI:1309481]
ENSMUSG00000026220	0.048022866	0.110601597	0.024582729	0	0.025227856	0.073156296	0.450174071	0.285440944	0.648116849	0.730240433	0.899419306	0.702224249	Slc16a14	solute carrier family 16 (monocarboxylic acid transporters), member 14 [Source:MGI Symbol;Acc:MGI:1919031]

ENSMUSG00000026222	34.61891459	34.80100648	32.3698855	31.80665682	29.31672026	33.22670048	0.822417456	0.618192543	0.331899046	7.203203651	7.519565018	7.505440495	Sp100	nuclear antigen Sp100 [Source:MGI Symbol;Acc:MGI:109561]
ENSMUSG00000026223	269.8533927	255.040148	282.9350505	166.4156571	156.6796356	134.069532	49.72245225	52.65208876	48.04283298	102.9449638	107.2099216	104.5323375	Itm2c	integral membrane protein 2C [Source:MGI Symbol;Acc:MGI:1927594]
ENSMUSG00000026224	0.028571373	0	0.146255811	0.026662336	0.060037607	0	0	0	0.026593062	0	0	0.025320654	4933407L21	RIKEN cDNA 4933407L21 gene [Source:MGI Symbol;Acc:MGI:1918391]
ENSMUSG00000026226	0	0.17406153	0	0	0	0	0	0	0	0.135203629	0	0.044652113	Spata3	spermatogenesis associated 3 [Source:MGI Symbol;Acc:MGI:1917310]
ENSMUSG00000026227	0.358885544	0.261015886	0.232057508	0.264399547	0.198456182	0.244582244	0.168634127	0.280679419	0.298874265	0.152059569	0.251567168	0.184136063	2810459M1	RIKEN cDNA 2810459M11 gene [Source:MGI Symbol;Acc:MGI:1920042]
ENSMUSG00000026228	1.123631787	1.255863299	1.353371742	0.246718758	0.208333264	0.050344118	0.265540466	0.098216239	0.061519434	0.591213001	0.550182419	0.732198631	Htr2b	5-hydroxytryptamine (serotonin) receptor 2B [Source:MGI Symbol;Acc:MGI:109323]
ENSMUSG00000026229	80.86761029	108.4540367	65.366835	95.67077741	105.540166	122.1320468	182.9738383	163.0675825	212.9821353	238.0009636	241.9503218	226.2515845	Psmd1	proteasome (prosome, macropain) 26S subunit, non-ATPase, 1 [Source:MGI Symbol;Acc:MGI:1917497]
ENSMUSG00000026234	332.1381615	301.515869	321.6808206	360.9197663	354.8535435	330.2376732	1164.203936	1218.801644	1179.924044	1086.068476	1049.164567	1037.867601	Ncl	nucleolin [Source:MGI Symbol;Acc:MGI:97286]
ENSMUSG00000026235	3.016896605	3.778096283	2.31650746	1.489772622	1.941461524	1.934078927	9.965641372	9.414297652	11.87551528	16.26996417	17.51605304	15.70768204	Epha4	Eph receptor A4 [Source:MGI Symbol;Acc:MGI:98277]
ENSMUSG00000026237	0	0	0.038642325	0.035222349	0.03965642	0	0	0.037391094	0	0	0.031418312	0	Nmur1	neuromedin U receptor 1 [Source:MGI Symbol;Acc:MGI:1341898]
ENSMUSG00000026238	1015.024958	998.107548	984.7576204	1202.626872	1229.777579	1126.242679	6425.31789	6489.741579	5537.630697	3540.629651	3331.508388	3328.569607	Ptma	prothymosin alpha [Source:MGI Symbol;Acc:MGI:97803]
ENSMUSG00000026239	57.76012874	47.06209014	58.95747943	61.07682623	53.42388624	43.6328829	46.55294063	45.53604614	41.49671122	27.20434571	23.23062406	24.88593177	Pde6d	phosphodiesterase 6D, cGMP-specific, rod, delta [Source:MGI Symbol;Acc:MGI:1270843]
ENSMUSG00000026240	11.25147499	12.91111	11.47868836	12.41879163	12.69406365	10.45274371	27.8048691	25.28950799	27.64422132	22.25085556	21.41440947	22.08063415	Cops7b	COP9 signalosome subunit 7B [Source:MGI Symbol;Acc:MGI:1349388]
ENSMUSG00000026241	0.454235476	0.28531391	0.169106527	0.038535008	0.086772204	0.125811979	1.142860342	1.10450853	0.538088408	0.36936628	0.687464021	0.402554656	Nppc	natriuretic peptide type C [Source:MGI Symbol;Acc:MGI:97369]
ENSMUSG00000026245	56.27075347	47.58715857	60.9534848	53.60423031	52.29543096	50.56717706	156.1633548	158.0180971	164.4592633	144.0123164	137.4162302	136.6429085	Farsb	phenylalanyl-tRNA synthetase, beta subunit [Source:MGI Symbol;Acc:MGI:1346035]
ENSMUSG00000026246	0	0	0.027810764	0.050698836	0	0	0.024251802	0.10764103	0.151701332	0.461661213	0.180893311	0.168516624	Alppl2	alkaline phosphatase, placental-like 2 [Source:MGI Symbol;Acc:MGI:108009]
ENSMUSG00000026247	0.504061093	0.316610323	0.304941029	0.106904893	0.048145174	0.244321773	0.695476591	0.749016493	0.490484816	0.368894246	0.839160022	0.38579616	Ecel1	endothelin converting enzyme-like 1 [Source:MGI Symbol;Acc:MGI:1343461]
ENSMUSG00000026248	18.62705101	19.13340603	19.07023818	20.19841612	16.6350955	14.69865168	33.42591198	33.65781221	31.69257532	31.19024807	26.48278066	31.62885082	Mrpl44	mitochondrial ribosomal protein L44 [Source:MGI Symbol;Acc:MGI:1916413]
ENSMUSG00000026249	128.181401	127.0954924	120.7747098	63.75305169	61.12493242	55.81447353	23.82113609	24.1328621	25.38038223	125.7798106	125.1048274	120.0287852	Serpine2	serine (or cysteine) peptidase inhibitor, clade E, member 2 [Source:MGI Symbol;Acc:MGI:101780]
ENSMUSG00000026251	0	0.069559451	0.020614062	0.018789648	0.105775195	0	0.862851191	0.578450905	0.562224869	0.306174744	0.083801768	0.196285424	Chrnd	cholinergic receptor, nicotinic, delta polypeptide [Source:MGI Symbol;Acc:MGI:87893]
ENSMUSG00000026253	0	0.079342699	0.246890113	0.032148501	0.108586834	0	0.092269465	0.136511922	0.192389839	0.154075126	0.114705766	0.122122993	Chrng	cholinergic receptor, nicotinic, gamma polypeptide [Source:MGI Symbol;Acc:MGI:87895]
ENSMUSG00000026254	57.20562897	52.5856989	63.08034421	52.05229849	50.49889503	45.76181686	91.01098275	92.61648275	80.96325207	76.18336287	76.72474833	79.50260541	Eif4e2	eukaryotic translation initiation factor 4E member 2 [Source:MGI Symbol;Acc:MGI:1914440]
ENSMUSG00000026255	0.229747837	0.566928096	0.168010108	0.122512518	0.413806118	0.324990289	2.021834425	1.983353707	2.443884151	1.614674409	1.994379794	1.948821216	Efhd1	EF hand domain containing 1 [Source:MGI Symbol;Acc:MGI:1921607]
ENSMUSG00000026258	0	0.095655436	0	0	0	0.316351551	0.1483199	0.246867844	0	0.297204374	0	0.073615646	Snorc	secondary ossification center associated regulator of chondrocyte maturation [Source:MGI Symbol;Acc:MGI:1920484]
ENSMUSG00000026259	2.77170519	2.110680666	2.906304299	4.484674148	4.485604574	3.217812935	0.658540356	0.420722653	0.249657048	0.579818714	0.781459101	0.495232528	Ngef	neuronal guanine nucleotide exchange factor [Source:MGI Symbol;Acc:MGI:1858414]
ENSMUSG00000026260	125.5609457	115.3900552	141.7289688	121.8765402	108.5719682	87.84481527	163.536273	183.548161	158.9664274	120.192796	126.0721419	123.7258751	Ndufa10	NADH:ubiquinone oxidoreductase subunit A10 [Source:MGI Symbol;Acc:MGI:1914523]
ENSMUSG00000026269	9.11109683	7.848899565	9.588959634	5.646582821	5.163881191	5.315183304	13.47417565	14.51483607	13.22744067	11.30169271	13.27694522	13.06709449	Rnpepl1	arginyl aminopeptidase (aminopeptidase B)-like 1 [Source:MGI Symbol;Acc:MGI:1914170]
ENSMUSG00000026270	15.3128757	13.80364928	18.08908825	13.99875677	11.39307862	11.82186896	21.4343751	24.98519897	22.3462657	20.63847472	20.04245447	17.74623101	Capn10	calpain 10 [Source:MGI Symbol;Acc:MGI:1344392]
ENSMUSG00000026271	2.548912079	2.853099698	1.496389609	2.0958319	2.584402623	2.185839819	0.190960335	0.176577662	0.464530341	1.291435491	1.275994735	1.153149148	Gpr35	G protein-coupled receptor 35 [Source:MGI Symbol;Acc:MGI:1929509]
ENSMUSG00000026272	0	0	0	0	0	0	0	0	0	0.039966736	0.074386034	0	Agxt	alanine-glyoxylate aminotransferase [Source:MGI Symbol;Acc:MGI:1329033]
ENSMUSG00000026273	10.00293259	8.288725861	11.3246252	8.569038628	9.731187836	6.853116246	11.74180964	11.37737878	11.69947538	12.59261876	12.02706889	11.18659012	Mterf4	mitochondrial transcription termination factor 4 [Source:MGI Symbol;Acc:MGI:1918355]
ENSMUSG00000026274	3.650606999	3.512559931	2.973360779	4.269713706	6.597141478	6.512786152	21.06153764	16.89282275	23.18078717	18.92470141	17.7058536	18.80763172	Pask	PAS domain containing serine/threonine kinase [Source:MGI Symbol;Acc:MGI:2155936]
ENSMUSG00000026275	10.11968386	9.891633617	9.293087282	8.9676453	9.882250258	9.796816809	20.00162405	19.90403686	20.25134868	19.50246176	18.79985063	18.4990283	Ppp1r7	protein phosphatase 1, regulatory subunit 7 [Source:MGI Symbol;Acc:MGI:1913635]
ENSMUSG00000026276	1.67537322	0.988166978	1.255049863	1.372768473	1.245053402	2.676700641	1.203884316	1.133450841	1.67346882	2.412351169	2.789158394	2.317669421	Septin2	septin 2 [Source:MGI Symbol;Acc:MGI:97298]
ENSMUSG00000026277	64.64806464	65.34835139	62.92408321	64.50604609	59.37396033	53.81438027	57.12857735	53.79100242	54.1423352	48.16884408	50.04279481	49.05469134	Stk25	serine/threonine kinase 25 (yeast) [Source:MGI Symbol;Acc:MGI:1891699]
ENSMUSG00000026278	9.982609176	9.286994928	9.767013187	7.296460544	7.129997637	5.768227632	21.07329138	22.55516284	18.0335608	19.3539488	20.3030517	23.0104842	Bok	BCL2-related ovarian killer [Source:MGI Symbol;Acc:MGI:1858494]
ENSMUSG00000026279	18.40550862	12.63939131	20.42902996	15.96085034	14.92794237	10.37758504	18.07522085	17.72185739	14.7285612	10.92198941	10.12662833	9.070737892	Thap4	THAP domain containing 4 [Source:MGI Symbol;Acc:MGI:1914276]
ENSMUSG00000026280	55.36859763	53.31958746	58.67494529	48.74820219	49.21507566	43.29678312	40.63109565	39.38257668	38.54228545	41.99564893	40.18916964	41.83141384	Atg4b	autophagy related 4B, cysteine peptidase [Source:MGI Symbol;Acc:MGI:1913865]
ENSMUSG00000026281	67.41433633	48.97213779	65.00506835	70.09844436	56.79773894	48.08476545	275.2115022	287.0471545	236.6887885	176.4167696	176.9975891	179.4751012	Dtymk	deoxythymidylate kinase [Source:MGI Symbol;Acc:MGI:108396]
ENSMUSG00000026283	3.941047684	4.24072431	4.259707076	4.811182479	4.792353266	6.200490391	13.17410311	12.86455764	15.00941174	19.08217192	18.84419395	19.53595658	Ing5	inhibitor of growth family, member 5 [Source:MGI Symbol;Acc:MGI:1922816]
ENSMUSG00000026285	0	0	0	0	0.023423999	0	0	0	0.083003423	0.099709757	0.0556739	0.098789909	Pdcd1	programmed cell death 1 [Source:MGI Symbol;Acc:MGI:104879]
ENSMUSG00000026288	1.921329669	1.962710378	1.649778185	0.563912724	0.553504798	1.050373726	28.35810257	26.73900853	30.57407318	33.70642212	35.12040041	34.37042148	Inpp5d	inositol polyphosphate-5-phosphatase D [Source:MGI Symbol;Acc:MGI:107357]
ENSMUSG00000026289	22.51958241	22.53653982	25.76086193	20.46093997	20.38096417	20.60956438	24.82348381	25.40098357	28.98082794	31.9448408	32.58108299	32.80414097	Atg16l1	autophagy related 16-like 1 (S. cerevisiae) [Source:MGI Symbol;Acc:MGI:1924290]
ENSMUSG00000026295	0.43400492	1.285145938	0.063475865	2.372179526	0.586275022	0.141674441	1.162409129	2.333980133	0.288538556	0.499123483	0.154827992	0.109893097	Spp2	secreted phosphoprotein 2 [Source:MGI Symbol;Acc:MGI:1922646]
ENSMUSG00000026301	0	0.131693065	0	0.053360103	0	0.021776758	0.05104964	0	0.106442928	0.179013797	0	0.025337478	Iqca	IQ motif containing with AAA domain [Source:MGI Symbol;Acc:MGI:1922168]
ENSMUSG00000026303	0.215250064	0.570104482	0.440742873	0.120520694	0.158308268	0.245928119	0.115302213	0.127941514	0.020034593	0.75089079	0.483769704	0.457823768	Mlph	melanophilin [Source:MGI Symbol;Acc:MGI:2176380]
ENSMUSG00000026304	0.115564426	0.133078377	0	0.035947608	0	0	0.171955479	0.114483029	0.179271045	0.930326851	1.218479202	0.614496226	Rab17	RAB17, member RAS oncogene family [Source:MGI Symbol;Acc:MGI:104640]
ENSMUSG00000026305	69.82452233	79.42876503	60.67091684	79.84086771	94.16869098	100.6046402	17.85855955	15.58577136	20.7102693	47.86982701	50.03553063	52.80621624	Lrrfip1	leucine rich repeat (in FLII) interacting protein 1 [Source:MGI Symbol;Acc:MGI:1342770]
ENSMUSG00000026307	5.072464012	5.89155826	5.551299822	6.039344227	4.180850579	4.729584279	13.35150416	14.42521065	15.18123325	12.98577727	11.35657027	11.93590919	Scly	selenocysteine lyase [Source:MGI Symbol;Acc:MGI:1355310]
ENSMUSG00000026308	0.320380453	0.785991022	0.541918261	0.493956671	0.321975721	0.297077558	0.024872032	0	0	0.012459685	0.034784923	0	Klhl30	kelch-like 30 [Source:MGI Symbol;Acc:MGI:1918038]
ENSMUSG00000026309	54.308223	48.09512701	68.0402164	52.50839409	47.4504313	43.52177639	84.4893501	83.95738828	75.31510479	49.00593289	51.73145881	47.12578606	Ilkap	integrin-linked kinase-associated serine/threonine phosphatase 2C [Source:MGI Symbol;Acc:MGI:1914694]
ENSMUSG00000026311	8.908336958	10.06167135	8.520599726	7.607068404	9.103206474	11.52574747	9.173371279	8.486484709	9.677219823	10.80632068	11.23467507	10.57685218	Asb1	ankyrin repeat and SOCS box-containing 1 [Source:MGI Symbol;Acc:MGI:1929735]
ENSMUSG00000026312	0	0	0	0	0	0	0.079314909	0	0.041344616	0.145687416	0.123251464	0.131221282	Cdh7	cadherin 7, type 2 [Source:MGI Symbol;Acc:MGI:2442792]
ENSMUSG00000026313	8.709985642	9.963277827	8.46246099	9.713969728	8.867161184	10.76776621	13.17277761	11.68958391	15.2431241	13.47424316	13.63227577	15.6776215	Hdac4	histone deacetylase 4 [Source:MGI Symbol;Acc:MGI:3036234]
ENSMUSG00000026315	3.405402841	2.816544395	2.099560678	3.107636894	2.747685287	3.124064932	0.117579352	0.074553297	0.087558267	1.279005441	1.284208041	1.61735538	Serpinb8	serine (or cysteine) peptidase inhibitor, clade B, member 8 [Source:MGI Symbol;Acc:MGI:894657]
ENSMUSG00000026317	26.64242881	24.95539261	28.6846116	29.81671601	28.37246676	31.21160238	28.52566842	29.33365963	25.65122324	30.13415651	28.74590511	27.69608052	Cln8	CLN8 transmembrane ER and ERGIC protein [Source:MGI Symbol;Acc:MGI:1349447]
ENSMUSG00000026319	13.28622587	17.47435896	10.68508733	15.82852614	17.07712339	24.56767299	17.55161436	16.55259211	18.37678544	24.29628285	25.62759194	24.99856957	Relch	RAB11 binding and LisH domain, coiled-coil and HEAT repeat containing [Source:MGI Symbol;Acc:MGI:1922832]
ENSMUSG00000026321	0.831188219	0.756820993	0.712435313	0.090191999	0.487421222	0.412251832	0.569492446	0.823410355	0.575729043	2.69726988	1.802106942	2.432557262	Tnfrsf11a	tumor necrosis factor receptor superfamily, member 11a, NFKB activator [Source:MGI Symbol;Acc:MGI:1314891]
ENSMUSG00000026322	0	0	0	0.056554194	0.106122813	0	0	0.04002427	0	0.144555742	0.235416083	0.089513861	Htr4	5 hydroxytryptamine (serotonin) receptor 4 [Source:MGI Symbol;Acc:MGI:109246]
ENSMUSG00000026327	0	0	0	0	0	0.046954184	0	0	0	0	0	0	Serpinb11	serine (or cysteine) peptidase inhibitor, clade B (ovalbumin), member 11 [Source:MGI Symbol;Acc:MGI:1914207]
ENSMUSG00000026331	0	0	0	0	0	0	0	0	0	0	0	0	Slco6c1	solute carrier organic anion transporter family, member 6c1 [Source:MGI Symbol;Acc:MGI:1921691]
ENSMUSG00000026333	4.621265228	3.764075843	3.490715307	5.4432023	5.092227083	4.915029945	6.415054216	6.559970013	8.445204185	7.309436966	6.497232814	7.616591965	Gin1	gypsy retrotransposon integrase 1 [Source:MGI Symbol;Acc:MGI:2182036]
ENSMUSG00000026335	41.59154389	51.6025877	36.17896615	48.77021056	53.06455673	56.16376697	16.53477949	14.94672582	19.34346774	22.46916528	21.48569525	22.02605626	Pam	peptidylglycine alpha-amidating monooxygenase [Source:MGI Symbol;Acc:MGI:97475]
ENSMUSG00000026336	0	0	0	0	0	0	0	0	0	0	0	0	Slco6d1	solute carrier organic anion transporter family, member 6d1 [Source:MGI Symbol;Acc:MGI:1918116]
ENSMUSG00000026339	11.04961168	13.99661544	8.966828873	12.90430878	15.72389018	20.00106395	7.920887584	7.485279147	9.271197086	14.56379996	14.7298253	13.1621884	Ccdc93	coiled-coil domain containing 93 [Source:MGI Symbol;Acc:MGI:1918079]
ENSMUSG00000026341	190.33598	216.7570893	153.3598354	211.5202333	218.9224882	233.6752681	202.6349375	190.9440776	221.731201	246.5421333	254.2552041	242.3000047	Actr3	ARP3 actin-related protein 3 [Source:MGI Symbol;Acc:MGI:1921367]
ENSMUSG00000026342	31.42459534	37.52328479	25.23693051	32.48568542	36.4434403	39.48648405	11.81564754	12.08560842	15.09140768	17.33641634	17.98675452	18.42719811	Slc35f5	solute carrier family 35, member F5 [Source:MGI Symbol;Acc:MGI:1921400]

ENSMUSG00000026343	0.067584012	0.181595083	0.138384032	0.021022763	0.047338553	0.257387597	0	0.022317198	0.041936284	0.100753839	0.131266036	0.119789229	Gpr39	G protein-coupled receptor 39 [Source:MGI Symbol;Acc:MGI:1918361]
ENSMUSG00000026344	0	0	0.062016307	0	0.084858411	0.046138931	0.090133398	0	0	0.072243954	0.016807544	0.017894371	Lypd1	Ly6/Plaur domain containing 1 [Source:MGI Symbol;Acc:MGI:1919835]
ENSMUSG00000026347	0.043006984	0	0	0.040133411	0.067778588	0	0.057593484	0	0.020014568	0.19234366	0.053698464	0.171512315	Tmem163	transmembrane protein 163 [Source:MGI Symbol;Acc:MGI:1919410]
ENSMUSG00000026348	0.071714146	0.247747578	0.055065313	0.033461232	0.018836799	0.06827921	0.064024757	0.01776077	0	0.032073305	0.044771094	0.079443551	Acmsd	amino carboxymuconate semialdehyde decarboxylase [Source:MGI Symbol;Acc:MGI:2386323]
ENSMUSG00000026349	11.00702038	12.70161148	8.81045004	13.04854006	15.13784545	21.26591993	26.05440036	22.69584521	32.88414978	37.06971821	37.42361382	38.34673406	Ccnt2	cyclin T2 [Source:MGI Symbol;Acc:MGI:1920199]
ENSMUSG00000026353	80.74304358	84.61125565	77.22377104	75.13364377	72.70149459	80.42543382	55.08351572	50.0526476	53.63057273	63.12745886	65.13028625	64.09789987	Ubxn4	UBX domain protein 4 [Source:MGI Symbol;Acc:MGI:1915062]
ENSMUSG00000026354	0.075999684	0.014586257	0.025935973	0.035460828	0.039924921	0.048239654	0	0.062740429	0	0.07930991	0.06326207	0.123480115	Lct	lactase [Source:MGI Symbol;Acc:MGI:104576]
ENSMUSG00000026355	43.11119516	41.21586331	35.6694097	52.34809484	51.31285341	55.79930568	214.0045186	216.2836741	236.3881442	160.1291748	157.4668879	158.4137247	Mcm6	minichromosome maintenance complex component 6 [Source:MGI Symbol;Acc:MGI:1298227]
ENSMUSG00000026356	43.44447321	44.84904798	35.34827085	49.47005517	52.20126669	54.81479705	121.4884267	116.6222588	131.1095486	115.0220433	112.4498399	111.3803391	Dars	aspartyl-tRNA synthetase [Source:MGI Symbol;Acc:MGI:2442544]
ENSMUSG00000026357	0	0	0.043379862	0	0	0	0	0	0	0	0.052905281	0.018775433	Rgs18	regulator of G-protein signaling 18 [Source:MGI Symbol;Acc:MGI:1927498]
ENSMUSG00000026358	0.404213131	0.512019419	0.289681307	0.018860253	0.042469064	0.046182274	0.144348911	0.180193814	0.263357502	1.428158452	1.429983286	1.737384572	Rgs1	regulator of G-protein signaling 1 [Source:MGI Symbol;Acc:MGI:1354694]
ENSMUSG00000026360	11.61358071	10.84881911	8.277333636	12.08440655	12.95779825	11.37696895	0.703456723	0.814506644	1.11601935	2.665965824	2.680562337	3.248583441	Rgs2	regulator of G-protein signaling 2 [Source:MGI Symbol;Acc:MGI:1098271]
ENSMUSG00000026361	9.233782246	10.44426065	7.198080098	11.30683444	11.9299729	14.28061901	20.22219851	19.0957297	25.61967781	32.52400862	33.41737892	34.16591951	Cdc73	cell division cycle 73, Paf1/RNA polymerase II complex component [Source:MGI Symbol;Acc:MGI:2384876]
ENSMUSG00000026365	3.492131361	4.333984016	2.94355443	3.776983289	5.004419525	6.400661902	0.209315236	0.256708575	0.045941097	0.331126754	0.349232511	0.306200554	Cfh	complement component factor h [Source:MGI Symbol;Acc:MGI:88385]
ENSMUSG00000026368	0	0.317399032	0	0.228631902	0	0	0.136707666	0.091016045	0.114018937	0.082180711	0.050984876	0	F13b	coagulation factor XIII, beta subunit [Source:MGI Symbol;Acc:MGI:88379]
ENSMUSG00000026374	34.33444432	39.86157953	32.80792875	34.00772854	33.879869	37.66794812	53.88476497	55.29624667	56.32671147	59.87815207	60.10148587	56.67442904	Tsn	translin [Source:MGI Symbol;Acc:MGI:109263]
ENSMUSG00000026377	59.33094785	52.04619664	51.30891932	59.49095699	47.53006338	49.2245465	112.3038902	117.7993388	108.582874	117.8507497	114.7389441	117.5764561	Nifk	nucleolar protein interacting with the FHA domain of MKI67 [Source:MGI Symbol;Acc:MGI:1915199]
ENSMUSG00000026380	0.670940721	0.874283616	0.289222816	0.716732165	0.834786346	1.344851073	29.84756922	27.74965424	34.99576043	35.00557138	37.24984444	36.30214972	Tfcp2l1	transcription factor CP2-like 1 [Source:MGI Symbol;Acc:MGI:2444691]
ENSMUSG00000026383	4.267943667	5.298436405	3.443612479	4.412144688	5.317645115	5.70403309	10.42527013	8.990407406	11.29705048	11.18308167	12.12385387	11.50173895	Epb41l5	erythrocyte membrane protein band 4.1 like 5 [Source:MGI Symbol;Acc:MGI:103006]
ENSMUSG00000026384	4.985007736	6.070090329	4.248942092	6.365795267	6.390311075	9.301717609	13.2663271	12.23957268	15.38473504	20.0973749	19.13939466	20.52497253	Ptpn4	protein tyrosine phosphatase, non-receptor type 4 [Source:MGI Symbol;Acc:MGI:1099792]
ENSMUSG00000026385	303.5710637	248.430347	420.2307174	276.015639	246.9548886	181.6119451	282.515541	318.0995839	208.8389811	129.8590046	118.0969812	128.2106323	Dbi	diazepam binding inhibitor [Source:MGI Symbol;Acc:MGI:94865]
ENSMUSG00000026387	0.494064056	1.008575736	0.965654754	0.607750969	0.235951557	0.478952285	0.060148519	0.022247311	0.04180496	0.281227327	0.149548541	0.059707053	Sctr	secretin receptor [Source:MGI Symbol;Acc:MGI:2441720]
ENSMUSG00000026388	3.357318318	2.367015078	4.068519923	3.644503561	1.727706434	2.76596034	11.86700906	11.60671968	8.864382264	4.412633087	4.163426608	3.582549963	3110009E18	RIKEN cDNA 3110009E18 gene [Source:MGI Symbol;Acc:MGI:1920353]
ENSMUSG00000026389	53.31229642	52.22518947	50.58666731	59.26653328	55.47060661	53.98973666	2.701844736	3.097478266	2.990336647	4.695531897	5.456104031	5.249629879	Steap3	STEAP family member 3 [Source:MGI Symbol;Acc:MGI:1915678]
ENSMUSG00000026390	67.99100777	57.44808824	78.43449425	0.629584147	0.433180751	0.542427171	1.539271156	2.190701232	2.930425721	57.0949536	60.65157311	57.1329234	Marco	macrophage receptor with collagenous structure [Source:MGI Symbol;Acc:MGI:1309998]
ENSMUSG00000026393	19.74248265	23.39183641	17.65398062	17.30770684	20.18814516	24.06162767	6.154069238	6.177813722	8.260450777	16.46456434	16.11983532	15.91177775	Nek7	NIMA (never in mitosis gene a)-related expressed kinase 7 [Source:MGI Symbol;Acc:MGI:1890645]
ENSMUSG00000026394	0.02280259	0.105033419	0	0.042558005	0.023957773	0	0	0.022589214	0	0	0.018980856	0.020208217	Atp6v1g3	ATPase, H+ transporting, lysosomal V1 subunit G3 [Source:MGI Symbol;Acc:MGI:2450548]
ENSMUSG00000026395	1.954494285	2.148396066	0.803430578	0.069087195	0.155568886	0.18044884	0.118972363	0.146682201	0.151596926	3.920315914	3.734519363	3.81853932	Ptprc	protein tyrosine phosphatase, receptor type, C [Source:MGI Symbol;Acc:MGI:97810]
ENSMUSG00000026398	0.70991089	0.999165301	0.787368358	0.276032157	0.145031269	0.826109594	19.26024306	16.46818956	19.89609544	32.01450915	30.77756257	34.3580617	Nr5a2	nuclear receptor subfamily 5, group A, member 2 [Source:MGI Symbol;Acc:MGI:1346834]
ENSMUSG00000026399	25.67870447	28.24233857	22.24914126	7.078873996	7.64691031	7.886065716	6.726603393	6.270747101	6.917363872	16.77974472	15.08191243	16.10257857	Cd55	CD55 molecule, decay accelerating factor for complement [Source:MGI Symbol;Acc:MGI:104850]
ENSMUSG00000026401	0	0	0	0	0	0	0	0.084157646	0	0.037994044	0	0.075287077	Cd55b	CD55 molecule, decay accelerating factor for complement B [Source:MGI Symbol;Acc:MGI:104849]
ENSMUSG00000026404	8.977451045	10.5201637	8.907624121	13.39679454	12.42397719	12.74210545	11.54564088	15.27948585	14.50309347	12.80573881	15.60404318	12.75769973	Ddx59	DEAD (Asp-Glu-Ala-Asp) box polypeptide 59 [Source:MGI Symbol;Acc:MGI:1915247]
ENSMUSG00000026405	0.020363695	0	0.020848202	0	0	0	0	0	0	0	0	0.018046808	C4bp	complement component 4 binding protein [Source:MGI Symbol;Acc:MGI:88229]
ENSMUSG00000026407	0	0	0	0	0.010810215	0	0.06430033	0.101926943	0.086188992	0.13804866	0.239806914	0.072946733	Cacna1s	calcium channel, voltage-dependent, L type, alpha 1S subunit [Source:MGI Symbol;Acc:MGI:88294]
ENSMUSG00000026409	2.589758519	2.538934107	2.221422946	2.939254173	2.904804514	4.398302891	2.062123114	1.300097022	2.003271914	3.568628033	3.335528461	3.132511894	Pfkfb2	6-phosphofructo-2-kinase/fructose-2,6-biphosphatase 2 [Source:MGI Symbol;Acc:MGI:107815]
ENSMUSG00000026411	87.83603813	84.28182033	89.29052252	75.09472537	71.59432723	57.92177878	48.46181591	53.48724159	44.24661067	31.75257226	33.61276006	31.38631386	Tmem9	transmembrane protein 9 [Source:MGI Symbol;Acc:MGI:1913491]
ENSMUSG00000026413	0.047880365	0	0.081699277	0.04468117	0.050305992	0.072939215	0.014248833	0.031621549	0.014855027	0.028551904	0.092996338	0.070721262	Pkp1	plakophilin 1 [Source:MGI Symbol;Acc:MGI:1328359]
ENSMUSG00000026414	8.619324957	5.64935095	9.591740938	2.670540094	2.935140225	2.413288931	0.364986737	0.269997452	0.190257252	0.700889332	0.964191422	0.905769708	Tnnt2	troponin T2, cardiac [Source:MGI Symbol;Acc:MGI:104597]
ENSMUSG00000026415	0	0	0	0	0	0.036194391	0	0	0	0.085009313	0	0.02105627	Fcamr	Fc receptor, IgA, IgM, high affinity [Source:MGI Symbol;Acc:MGI:1927803]
ENSMUSG00000026416	0	0	0	0	0	0	0	0	0	0.046595601	0	0	Il20	interleukin 20 [Source:MGI Symbol;Acc:MGI:1890473]
ENSMUSG00000026417	0.02402484	0.110663351	0	0	0	0	0	0	0.044722671	0	0	0	Pigr	polymeric immunoglobulin receptor [Source:MGI Symbol;Acc:MGI:103080]
ENSMUSG00000026418	5.177701855	2.261596149	4.478340788	3.707115692	3.048278727	2.413854217	0.75713924	0.265306106	0.456992393	0.678729697	0.557316527	0.791139101	Tnni1	troponin I, skeletal, slow 1 [Source:MGI Symbol;Acc:MGI:105073]
ENSMUSG00000026420	0.204410869	0.058847406	0.470867282	0.429193944	0.429532716	0.428164165	0	0	0	0.137130522	0	0.181153942	Il24	interleukin 24 [Source:MGI Symbol;Acc:MGI:2135548]
ENSMUSG00000026421	81.03777089	73.0760381	88.9488754	54.95265121	52.16534872	46.23542013	34.08108631	35.13247579	33.46933473	74.42552533	70.53531447	72.82006498	Csrp1	cysteine and glycine-rich protein 1 [Source:MGI Symbol;Acc:MGI:88549]
ENSMUSG00000026424	0	0	0	0	0	0	0.033860603	0.206648006	0.105903442	0.441025617	0.078926566	0.21847852	Gpr37l1	G protein-coupled receptor 37-like 1 [Source:MGI Symbol;Acc:MGI:1928503]
ENSMUSG00000026425	25.0415073	25.7486457	23.54662463	26.58291126	30.53620353	34.48656534	24.4006144	22.74332142	29.0289152	40.09316919	43.320219	41.55133862	Srgap2	SLIT-ROBO Rho GTPase activating protein 2 [Source:MGI Symbol;Acc:MGI:109605]
ENSMUSG00000026426	118.6254996	113.1517484	111.8748674	109.7010762	103.9874579	96.02848404	47.75327164	51.3786691	43.62628568	61.24052108	56.06693215	56.77799897	Arl8a	ADP-ribosylation factor-like 8A [Source:MGI Symbol;Acc:MGI:1915974]
ENSMUSG00000026427	22.69935057	20.70161966	24.2100714	26.30393082	25.07529183	21.01148018	70.77448538	72.95160969	70.83269426	56.7697504	58.18724601	57.05393494	Eif2d	eukaryotic translation initiation factor 2D [Source:MGI Symbol;Acc:MGI:109342]
ENSMUSG00000026429	9.558286176	7.31303092	8.425656288	10.28025788	10.07655288	10.19252298	42.69602757	45.28996318	42.79348344	27.41687966	27.07842092	25.81436846	Ube2t	ubiquitin-conjugating enzyme E2T [Source:MGI Symbol;Acc:MGI:1914446]
ENSMUSG00000026430	3.170908272	4.352035434	3.718206208	3.71600309	3.544611079	4.675984949	0.872316738	0.876624117	0.617724702	3.347496647	3.514166446	3.104221033	Rassf5	Ras association (RalGDS/AF-6) domain family member 5 [Source:MGI Symbol;Acc:MGI:1926375]
ENSMUSG00000026432	0.03704562	0	0	0	0.019461179	0.014108489	0	0	0	0.149113946	0.061673483	0	Avpr1b	arginine vasopressin receptor 1B [Source:MGI Symbol;Acc:MGI:1347010]
ENSMUSG00000026433	21.13006099	18.24926357	22.22278693	17.6119607	14.53124518	11.1929133	11.91889446	14.17690024	12.82824385	9.579036273	8.794322104	9.320430945	Rab29	RAB29, member RAS oncogene family [Source:MGI Symbol;Acc:MGI:2385107]
ENSMUSG00000026434	33.18227924	32.5258779	29.37291279	38.95951534	39.12988899	41.05756431	101.9843605	97.94025575	105.2228677	97.37679404	96.38374442	95.28296256	Nucks1	nuclear casein kinase and cyclin-dependent kinase substrate 1 [Source:MGI Symbol;Acc:MGI:1934811]
ENSMUSG00000026435	1.922593919	1.852993056	1.412068243	1.306596899	1.405212421	1.639496904	2.145560087	1.821794365	2.256290227	1.925332946	2.365757681	2.11129244	Slc45a3	solute carrier family 45, member 3 [Source:MGI Symbol;Acc:MGI:1922082]
ENSMUSG00000026436	20.4789507	18.2585229	23.34740221	20.7781675	17.8508785	16.87311148	9.192216235	9.497387292	9.187280639	11.89904827	12.3718146	10.40673866	Elk4	ELK4, member of ETS oncogene family [Source:MGI Symbol;Acc:MGI:102853]
ENSMUSG00000026437	29.1998701	28.04210743	27.94864273	24.575368	24.86168083	23.24815292	22.15863221	22.78654471	20.51172933	22.39792665	25.70468464	23.68048499	Cdk18	cyclin-dependent kinase 18 [Source:MGI Symbol;Acc:MGI:97518]
ENSMUSG00000026439	14.38696106	16.71978527	11.25026697	17.33806581	18.43108363	21.14344511	56.89338388	50.36407851	67.79602522	49.32380921	48.91299456	48.34078994	Rbbp5	retinoblastoma binding protein 5, histone lysine methyltransferase complex subunit [Source:MGI Symbol;Acc:MGI:1918367]
ENSMUSG00000026442	1.362837219	1.926053731	1.090841784	0.878682498	0.663871073	0.839875565	1.393686039	1.448272395	1.499104434	2.947825815	2.85668716	3.535501097	Nfasc	neurofascin [Source:MGI Symbol;Acc:MGI:104753]
ENSMUSG00000026443	0.484412736	0.315293051	0.603750859	0.23585009	0.088513594	0.048126315	0.357260023	0.479879832	0.431268396	1.752021964	1.349926291	1.493212127	Lrrn2	leucine rich repeat protein 2, neuronal [Source:MGI Symbol;Acc:MGI:106037]
ENSMUSG00000026447	1.957789051	2.139301571	1.770283053	2.107024078	1.921841497	2.830038315	8.77762192	7.913599756	10.24172922	6.889742237	7.81523985	7.421415526	Pik3c2b	phosphatidylinositol-4-phosphate 3-kinase catalytic subunit type 2 beta [Source:MGI Symbol;Acc:MGI:2685045]
ENSMUSG00000026450	0.101602096	0.029250009	0.078014611	0	0.026687319	0.116082718	0.634956266	0.956187847	1.087521234	0.886086593	0.930308454	0.900422779	Chit1	chitinase 1 (chitotriosidase) [Source:MGI Symbol;Acc:MGI:1919134]
ENSMUSG00000026452	0.10822832	0.106826121	0.174119566	0.245278161	0.211177915	0.200200629	0.151837368	0.122532081	0.04317192	0.401060375	0.321746995	0.424764659	Syt2	synaptotagmin II [Source:MGI Symbol;Acc:MGI:99666]
ENSMUSG00000026455	6.158605604	6.354513562	6.207489341	5.721679869	6.613756516	8.167559924	21.50647942	19.2612681	22.53557386	17.42805317	17.9651459	18.04007587	Klhl12	kelch-like 12 [Source:MGI Symbol;Acc:MGI:2385619]
ENSMUSG00000026456	74.73368939	64.92695151	69.75605862	84.02352077	86.64243952	78.30671174	177.3288227	180.8697494	191.1601969	124.3000656	133.5494931	126.3714772	Cyb5r1	cytochrome b5 reductase 1 [Source:MGI Symbol;Acc:MGI:1919267]
ENSMUSG00000026457	70.93784309	69.07950803	70.20286577	58.97706369	58.76489395	53.01637734	76.86324359	76.67442967	80.67517865	71.01206192	69.24179026	69.92419581	Adipor1	adiponectin receptor 1 [Source:MGI Symbol;Acc:MGI:1919924]
ENSMUSG00000026458	1.149360541	0.745298731	0.776855035	1.093390864	0.832414575	0.764953801	10.52025049	10.29166603	11.97527793	8.074887136	8.842755564	8.91020413	Ppfia4	protein tyrosine phosphatase, receptor type, f polypeptide (PTPRF), interacting protein (liprin), alpha 4 [Source:MGI Symbol;Acc:MGI:
ENSMUSG00000026459	0.143907986	0	0.058932776	0.026858514	0	0.043844844	0	0.085536818	0	0.051488918	0.095831105	0.229562637	Myog	myogenin [Source:MGI Symbol;Acc:MGI:97276]

ENSMUSG00000026463	3.332607618	4.305418114	2.353359858	2.868720901	3.35594897	5.34397247	1.21978082	1.390072699	1.91610401	10.39612737	11.02707923	10.62747118	Atp2b4	ATPase, Ca++ transporting, plasma membrane 4 [Source:MGI Symbol;Acc:MGI:88111]
ENSMUSG00000026466	26.96289379	31.49833231	22.68753282	22.84084518	26.27973633	29.4687653	8.183560981	7.824801504	7.941758092	14.69738772	14.95582846	15.68526278	Tor1aip1	torsin A interacting protein 1 [Source:MGI Symbol;Acc:MGI:3582693]
ENSMUSG00000026468	0.293892186	0.138449438	0.164118908	0.087263069	0.224567865	0.162801717	0	0	0	0.011949074	0.066718799	0.047355361	Lhx4	LIM homeobox protein 4 [Source:MGI Symbol;Acc:MGI:101776]
ENSMUSG00000026469	9.279324536	10.84180366	7.637111933	10.15702631	12.0650516	16.82175895	6.518517397	6.426907428	9.194367857	11.65658661	12.50344826	12.14002639	Xpr1	xenotropic and polytropic retrovirus receptor 1 [Source:MGI Symbol;Acc:MGI:97932]
ENSMUSG00000026470	40.11657308	42.87563211	38.75556046	40.31177013	37.69340078	35.2719092	27.43557581	26.6341532	26.33981647	32.29246587	31.95750125	31.56863332	Stx6	syntaxin 6 [Source:MGI Symbol;Acc:MGI:1926235]
ENSMUSG00000026471	10.40421516	14.14840361	10.09865274	11.54676762	14.97788757	15.78303383	0.451207277	0.517932536	0.502844754	2.291507111	3.046392811	2.563816192	Mr1	major histocompatibility complex, class I-related [Source:MGI Symbol;Acc:MGI:1195463]
ENSMUSG00000026473	45.40343683	49.34842181	45.91159842	44.42304407	46.78620608	45.2504944	215.4287555	198.0441171	199.2055199	140.581046	138.3252383	135.2603843	Glul	glutamate-ammonia ligase (glutamine synthetase) [Source:MGI Symbol;Acc:MGI:95739]
ENSMUSG00000026475	10.44898721	11.26991976	12.24196778	10.02549425	9.567386739	8.869583182	6.290247588	5.467188675	4.999723063	35.26280972	34.02526606	31.13888326	Rgs16	regulator of G-protein signaling 16 [Source:MGI Symbol;Acc:MGI:108407]
ENSMUSG00000026478	59.63395595	81.34599624	56.16465589	62.18063078	71.41866691	100.950994	123.7184271	107.5333885	154.2891778	157.810953	162.6922512	164.9876879	Lamc1	laminin, gamma 1 [Source:MGI Symbol;Acc:MGI:99914]
ENSMUSG00000026479	6.494866087	6.668928518	4.654577379	6.161917353	6.217328477	6.967055959	4.42938569	4.396628316	4.93687654	6.390454794	6.472567435	7.082967202	Lamc2	laminin, gamma 2 [Source:MGI Symbol;Acc:MGI:99913]
ENSMUSG00000026480	2.270088087	2.590358371	2.408612241	0.308132204	0.108413233	0.220065366	1.25285855	0.960870428	1.402200509	4.910215374	3.813595375	4.974652314	Ncf2	neutrophil cytosolic factor 2 [Source:MGI Symbol;Acc:MGI:97284]
ENSMUSG00000026482	10.0388538	13.0004228	9.481788338	8.406050152	8.860546291	11.54685645	1.267418595	1.406351613	1.19549706	5.895642924	6.977678805	7.473809678	Rgl1	ral guanine nucleotide dissociation stimulator,-like 1 [Source:MGI Symbol;Acc:MGI:107484]
ENSMUSG00000026483	13.2756271	17.49647745	12.37645002	5.962495265	7.568551051	10.76350133	15.68380668	13.57519781	19.34381725	14.76318402	14.49675974	14.85935831	Fam129a	family with sequence similarity 129, member A [Source:MGI Symbol;Acc:MGI:2137237]
ENSMUSG00000026484	32.1207059	37.64791628	25.82054161	42.26237898	48.56411789	53.429916	95.57981549	91.13215198	111.9300866	93.52468019	90.04382561	91.05966736	Rnf2	ring finger protein 2 [Source:MGI Symbol;Acc:MGI:1101759]
ENSMUSG00000026489	2.621483502	3.539251116	3.701869765	4.555226477	4.368871101	4.09675258	18.07757839	15.53694239	19.98250732	13.5435596	13.39370554	14.62028384	Coq8a	coenzyme Q8A [Source:MGI Symbol;Acc:MGI:1914676]
ENSMUSG00000026490	11.01918641	16.2961574	10.33592529	14.16085529	17.34646322	23.5444458	6.216789029	5.513668235	7.758942348	15.33714205	15.23879244	15.92557504	Cdc42bpa	CDC42 binding protein kinase alpha [Source:MGI Symbol;Acc:MGI:2441841]
ENSMUSG00000026491	24.82756251	31.81946894	22.21813366	30.13594351	34.4551567	43.1953157	60.37303342	56.28593671	75.75922934	106.628762	110.6174984	103.0332406	Ahctf1	AT hook containing transcription factor 1 [Source:MGI Symbol;Acc:MGI:1915033]
ENSMUSG00000026492	11.92203955	16.00086797	8.853427845	11.94810804	13.62865402	15.73152991	18.55156367	18.63061441	21.80236737	30.22713457	30.71515468	31.69681443	Tfb2m	transcription factor B2, mitochondrial [Source:MGI Symbol;Acc:MGI:107937]
ENSMUSG00000026494	6.424777604	7.916353996	6.398249995	3.826217109	4.571765599	5.952441918	0.219006905	0.324018932	0.250069359	2.889079056	3.35950332	2.831369467	Kif26b	kinesin family member 26B [Source:MGI Symbol;Acc:MGI:2447076]
ENSMUSG00000026495	1.664544984	1.852915478	1.704148944	2.17465644	1.573984004	2.028566074	2.996903509	3.27045511	2.427221304	2.158899468	2.216905252	2.188155072	Efcab2	EF-hand calcium binding domain 2 [Source:MGI Symbol;Acc:MGI:1915476]
ENSMUSG00000026496	107.7468461	117.6548192	96.37553513	119.518056	110.1606778	128.3203237	788.6804734	734.2954703	853.8642571	559.1751686	596.6799072	580.2427293	Parp1	poly (ADP-ribose) polymerase family, member 1 [Source:MGI Symbol;Acc:MGI:1340806]
ENSMUSG00000026497	0	0.151197301	0.019203248	0	0	0.200015819	0.636340216	0.538862068	0.418997226	17.56621656	17.47126576	17.96934175	Mixl1	Mix1 homeobox-like 1 (Xenopus laevis) [Source:MGI Symbol;Acc:MGI:1351322]
ENSMUSG00000026499	15.48781082	21.67791309	12.47729927	17.54433208	20.80610106	28.09068757	11.37166987	9.724021507	13.64174224	21.72601965	22.67045414	21.89392056	Acbd3	acyl-Coenzyme A binding domain containing 3 [Source:MGI Symbol;Acc:MGI:2181074]
ENSMUSG00000026500	0	0	0	0	0	0	0	0	0	0	0	0	Cox20	cytochrome c oxidase assembly protein 20 [Source:MGI Symbol;Acc:MGI:1913609]
ENSMUSG00000026502	0.298178735	1.015175373	0.225636714	0.641197447	1.743496459	2.527914819	1.076402961	0.693520872	1.83412806	2.632349613	3.259022716	3.400825486	Desi2	desumoylating isopeptidase 2 [Source:MGI Symbol;Acc:MGI:1926075]
ENSMUSG00000026504	17.20296231	17.13961687	18.13027446	21.50306285	21.10902861	18.86794427	3.331420466	3.070063504	2.845236144	5.053784903	5.650678052	5.081896166	Sdccag8	serologically defined colon cancer antigen 8 [Source:MGI Symbol;Acc:MGI:1924066]
ENSMUSG00000026509	158.3135891	155.6199613	147.5517125	169.7056469	176.684048	160.4984944	34.09487805	31.19016894	38.6556107	53.21936651	55.359921	53.20915142	Capn2	calpain 2 [Source:MGI Symbol;Acc:MGI:88264]
ENSMUSG00000026510	9.341488098	10.05437374	8.105752893	9.1919506	10.95473435	11.41537992	20.58479178	16.45915317	19.48806231	17.01477473	17.75320305	17.86446953	Trp53bp2	transformation related protein 53 binding protein 2 [Source:MGI Symbol;Acc:MGI:2138319]
ENSMUSG00000026511	23.86928088	18.70112847	25.39157637	17.8561646	18.68644858	16.85414288	79.50166493	81.04743562	69.36677442	57.79777846	52.11212441	57.80809211	Srp9	signal recognition particle 9 [Source:MGI Symbol;Acc:MGI:1350930]
ENSMUSG00000026514	0.059064499	0.204047422	0.181409404	0.137795051	0.279255227	0.044988361	0	0.029255901	0	0	0	0	Cnih3	cornichon family AMPA receptor auxiliary protein 3 [Source:MGI Symbol;Acc:MGI:1920228]
ENSMUSG00000026516	13.52768162	15.57782078	10.27499922	15.14329585	16.08429986	21.85652493	39.46230246	34.82604574	45.98284644	55.20317384	54.9843164	54.85676401	Nvl	nuclear VCP-like [Source:MGI Symbol;Acc:MGI:1914709]
ENSMUSG00000026519	48.15771154	47.82676756	48.61063701	43.11180526	41.87757304	42.18841233	25.50390146	23.14781133	25.95901129	20.13608837	22.91924234	23.07545805	Tmem63a	transmembrane protein 63a [Source:MGI Symbol;Acc:MGI:2384789]
ENSMUSG00000026520	38.35392698	28.60122564	39.12231906	32.79551069	26.92380216	24.96659687	132.2898967	142.8300656	118.889366	89.4945972	89.40154652	87.02188393	Pycr2	pyrroline-5-carboxylate reductase family, member 2 [Source:MGI Symbol;Acc:MGI:1277956]
ENSMUSG00000026523	0.014919725	0.034361661	0	0	0	0.011364085	0.452879694	0.280822159	0.388826714	0.13345342	0.372574988	0.224777871	Wdr64	WD repeat domain 64 [Source:MGI Symbol;Acc:MGI:1923070]
ENSMUSG00000026525	1.19211179	1.087861817	0.759918587	1.2593877	1.181607902	1.199258466	0.261052328	0.222821995	0.376834672	0.301787504	0.149783065	0.318936997	Opn3	opsin 3 [Source:MGI Symbol;Acc:MGI:1338022]
ENSMUSG00000026526	67.82109272	55.25950742	58.35874288	60.76559646	58.94303602	50.34713837	155.927722	160.882128	158.0987934	133.6082261	125.7001773	131.794582	Fh1	fumarate hydratase 1 [Source:MGI Symbol;Acc:MGI:95530]
ENSMUSG00000026527	0.077435791	0.118895058	0.05285213	0	0.027119567	0.039320961	0.069132911	0.102281578	0.024024686	0.069264487	0.257829937	0.137251007	Rgs7	regulator of G protein signaling 7 [Source:MGI Symbol;Acc:MGI:1346089]
ENSMUSG00000026531	0.107705852	0	0	0	0	0	0	0	0	0	0	0	Mptx1	mucosal pentraxin 1 [Source:MGI Symbol;Acc:MGI:1913539]
ENSMUSG00000026532	0.308207792	0.200605326	0.09603418	0.043767416	0.070396011	0.107171354	0.562285313	0.404885816	0.573734341	1.977737294	1.868367064	1.59728299	Spta1	spectrin alpha, erythrocytic 1 [Source:MGI Symbol;Acc:MGI:98385]
ENSMUSG00000026535	135.5765023	140.1518415	137.3744891	145.5415687	149.7442295	139.1495691	5.568682441	5.400446232	6.702391887	35.97048595	34.6138337	35.68359025	Ifi202b	interferon activated gene 202B [Source:MGI Symbol;Acc:MGI:1347083]
ENSMUSG00000026536	12.07690331	15.87805207	10.71074319	14.14932076	18.71649706	19.64605159	0.215209267	0.214920241	0.381420491	5.217976393	5.056500068	4.785305686	Ifi211	interferon activated gene 211 [Source:MGI Symbol;Acc:MGI:3041120]
ENSMUSG00000026542	0	0	0	0	0	0	0	0	0	0	0	0	Apcs	serum amyloid P-component [Source:MGI Symbol;Acc:MGI:98229]
ENSMUSG00000026544	2.16668272	1.721933522	2.062020222	2.847764385	2.052008776	1.882762888	0.299690351	0.423235367	0.369247496	0.873485762	0.812865259	0.486803039	Dusp23	dual specificity phosphatase 23 [Source:MGI Symbol;Acc:MGI:1915690]
ENSMUSG00000026546	0.231751281	0.489268108	1.186326307	0.576710633	0.649311574	0.764923282	3.517338524	3.941169042	3.19961803	1.692917731	1.061002595	1.882683804	Cfap45	cilia and flagella associated protein 45 [Source:MGI Symbol;Acc:MGI:1919120]
ENSMUSG00000026547	381.8721258	322.2557575	404.7855228	351.4174471	319.2388037	266.9536082	330.3351555	372.0156167	339.0799849	237.2132137	239.8173402	239.5943375	Tagln2	transgelin 2 [Source:MGI Symbol;Acc:MGI:1312985]
ENSMUSG00000026548	1.179760519	1.434030155	1.61044022	0.336396235	0.482038429	0.22465056	0.321830461	0.194786814	0.305020194	2.19847485	1.936784584	2.700959738	Slamf9	SLAM family member 9 [Source:MGI Symbol;Acc:MGI:1923692]
ENSMUSG00000026553	141.5867288	159.136021	130.1298066	145.6223412	156.0499227	169.67234	92.70403215	83.04755428	102.0182812	143.0902308	145.4177701	144.7279086	Copa	coatomer protein complex subunit alpha [Source:MGI Symbol;Acc:MGI:1334462]
ENSMUSG00000026554	41.12457244	41.71646669	40.50822363	43.43230165	42.35324756	42.59568008	39.58924221	36.43601221	42.54618229	40.26838316	42.8909245	41.13936057	Dcaf8	DDB1 and CUL4 associated factor 8 [Source:MGI Symbol;Acc:MGI:91860]
ENSMUSG00000026556	6.07532479	7.153459898	5.986627718	7.710374847	7.452271327	7.577473885	15.30899056	14.26378525	17.88287211	15.69135233	15.52528171	15.15624803	Vangl2	VANGL planar cell polarity 2 [Source:MGI Symbol;Acc:MGI:2135272]
ENSMUSG00000026558	107.5386222	114.9023237	104.7575859	104.9770091	111.9933341	115.4042787	177.8142109	172.8930217	181.829216	193.3728735	191.1590059	187.51567	Uck2	uridine-cytidine kinase 2 [Source:MGI Symbol;Acc:MGI:1931744]
ENSMUSG00000026560	0	0	0	0	0.081780605	0	0	0	0	0	0.012958349	0	Fmo9	flavin containing monooxygenase 9 [Source:MGI Symbol;Acc:MGI:3606068]
ENSMUSG00000026563	2.926451713	5.295650773	1.679121644	4.111380018	6.554868281	7.495403823	0.717766332	0.732731313	0.68843833	3.883314993	3.53350341	3.163492093	Tada1	transcriptional adaptor 1 [Source:MGI Symbol;Acc:MGI:1196415]
ENSMUSG00000026564	0.085373984	0.110601597	0.185736174	0.089628299	0.022424761	0.12192716	4.439812003	4.006745109	4.598898104	9.431079061	9.718170083	9.958816626	Dusp27	dual specificity phosphatase 27 (putative) [Source:MGI Symbol;Acc:MGI:2685055]
ENSMUSG00000026565	5.142219418	4.915364732	3.255525814	4.357534545	4.529861459	6.851559574	11.39401963	12.34503579	13.89188279	14.77251173	15.04690308	15.22015736	Pou2f1	POU domain, class 2, transcription factor 1 [Source:MGI Symbol;Acc:MGI:101898]
ENSMUSG00000026566	89.06991135	90.04567336	79.09162207	89.13720627	84.62957625	84.30166577	91.161727	95.65165605	100.7363231	69.52257383	72.14318135	71.09241749	Mpzl1	myelin protein zero-like 1 [Source:MGI Symbol;Acc:MGI:1915731]
ENSMUSG00000026567	0.040051338	0.076868625	0.013668089	0.062292085	0.014026782	0	0	0.013225519	0.012426048	0.023883318	0.022225799	0	Adcy10	adenylate cyclase 10 [Source:MGI Symbol;Acc:MGI:2660854]
ENSMUSG00000026568	21.03773796	21.01601825	25.58738121	18.83166984	16.79666756	12.77569797	12.69859794	12.72166772	9.890322026	8.886756355	8.17084867	7.284524982	Mpc2	mitochondrial pyruvate carrier 2 [Source:MGI Symbol;Acc:MGI:1917706]
ENSMUSG00000026571	24.18964262	26.94879803	19.9072346	19.38898578	21.87225538	25.16109703	9.590193508	9.201262291	11.33253795	16.23682861	15.47974768	16.78492004	Dcaf6	DDB1 and CUL4 associated factor 6 [Source:MGI Symbol;Acc:MGI:1921356]
ENSMUSG00000026572	0	0	0	0	0	0	0	0	0.030432486	0	0.081649412	0.028976371	Tbx19	T-box 19 [Source:MGI Symbol;Acc:MGI:1891158]
ENSMUSG00000026573	0	0	0	0	0	0	0	0	0	0	0	0	Xcl1	chemokine (C motif) ligand 1 [Source:MGI Symbol;Acc:MGI:104593]
ENSMUSG00000026574	156.0157062	162.990315	170.8685598	96.75031724	101.0369203	83.24677284	1.0918375	0.890098908	0.88275392	4.37566207	4.071988164	2.963926467	Dpt	dermatopontin [Source:MGI Symbol;Acc:MGI:1928392]
ENSMUSG00000026575	12.17546841	10.93759678	10.9967469	10.3804176	12.18950213	10.17208492	29.82130894	27.84888716	30.43735516	24.09061599	23.08198642	24.32031921	Nme7	NME/NM23 family member 7 [Source:MGI Symbol;Acc:MGI:2449121]
ENSMUSG00000026576	8.806765266	19.5419585	5.681505166	9.156494011	8.914911375	9.801576265	107.1307706	96.3662856	133.0979279	70.64520183	71.23206637	73.62858046	Atp1b1	ATPase, Na+/K+ transporting, beta 1 polypeptide [Source:MGI Symbol;Acc:MGI:88108]
ENSMUSG00000026577	7.981090484	8.861559483	6.917122778	7.371629944	9.414825311	9.048614806	10.89757245	9.26121359	11.11420951	14.46037446	14.73113427	14.81539324	Blzf1	basic leucine zipper nuclear factor 1 [Source:MGI Symbol;Acc:MGI:1201607]
ENSMUSG00000026578	7.91147157	5.840042439	6.761293509	7.109416191	6.204598956	6.86726467	10.66520307	11.87896856	9.860189217	5.685493199	5.609872063	5.233536421	Ccdc181	coiled-coil domain containing 181 [Source:MGI Symbol;Acc:MGI:1922145]
ENSMUSG00000026579	0	0.026675379	0.00592897	0.010808473	0.01216913	0.013233117	0.103404722	0.045895933	0.123974493	0.574988461	0.520622147	0.656932948	F5	coagulation factor V [Source:MGI Symbol;Acc:MGI:88382]
ENSMUSG00000026580	0.188034178	0.108265511	0.224592681	0.11698027	0.098780011	0.11935186	0	0	0	0.112128088	0.026086581	0.027773419	Selp	selectin, platelet [Source:MGI Symbol;Acc:MGI:98280]

ENSMUSG00000026581	0	0	0	0	0	0	0	0	0.032921621	0.063276558	0	0.015673204	Sell	selectin, lymphocyte [Source:MGI Symbol;Acc:MGI:98279]
ENSMUSG00000026582	0.251196529	0.357328238	0.226917498	0.248201443	0.667567888	0.619014813	0	0.029275994	0.096271999	0.026434043	0.184496268	0.039285273	Sele	selectin, endothelial cell [Source:MGI Symbol;Acc:MGI:98278]
ENSMUSG00000026584	4.596469404	5.700230093	3.871687584	5.336582263	6.089151846	7.306534076	5.928980693	4.264107666	7.054030618	8.731639388	9.110973684	8.065266015	Scyl3	SCY1-like 3 (S. cerevisiae) [Source:MGI Symbol;Acc:MGI:1921385]
ENSMUSG00000026585	11.09505617	12.47766139	10.73905069	11.18121474	13.88403243	13.96128801	13.0237844	12.58742675	14.41326589	17.69150301	17.97594881	17.17370324	Kifap3	kinesin-associated protein 3 [Source:MGI Symbol;Acc:MGI:107566]
ENSMUSG00000026586	37.71646026	44.29054922	31.49703193	33.6864036	38.69885737	45.21225608	3.42141204	3.353542694	4.409171803	43.42988981	44.89072023	44.30285018	Prrx1	paired related homeobox 1 [Source:MGI Symbol;Acc:MGI:97712]
ENSMUSG00000026587	0.033111572	0.076259355	0.169496923	0.06179835	0	0	0.029561257	0.049202594	0.015409447	0.266557674	0.08268613	0.249426464	Astn1	astrotactin 1 [Source:MGI Symbol;Acc:MGI:1098567]
ENSMUSG00000026589	3.773509286	4.224684645	2.843811523	4.242770039	4.529091576	4.690557156	0.058487902	0.012979856	0.07317141	0.574272336	0.436259141	0.719926946	Sec16b	SEC16 homolog B (S. cerevisiae) [Source:MGI Symbol;Acc:MGI:2148802]
ENSMUSG00000026592	0.207366206	0.238792846	0.10615	0.193510737	0.054467853	0.157947087	0.092565913	0	0.04825199	0.046371044	0.172611448	0.045943259	Tex35	testis expressed 35 [Source:MGI Symbol;Acc:MGI:1920685]
ENSMUSG00000026594	3.611582153	2.615792887	3.346164086	3.111017652	3.193599397	3.634642623	17.59525788	16.49667254	18.7240811	19.07588155	20.06452386	17.91507904	Ralgps2	Ral GEF with PH domain and SH3 binding motif 2 [Source:MGI Symbol;Acc:MGI:1925505]
ENSMUSG00000026596	13.19071894	17.13418857	11.86869949	21.9538109	23.31258555	32.70496818	11.86742792	12.74172693	16.07450801	25.51335564	27.81774185	25.77009045	Abl2	v-abl Abelson murine leukemia viral oncogene 2 (arg, Abelson-related gene) [Source:MGI Symbol;Acc:MGI:87860]
ENSMUSG00000026600	69.86803964	75.03562787	63.41779385	72.39552786	74.95181097	86.42931086	15.44201065	15.3832886	18.33933919	47.70983554	46.79244277	47.02428989	Soat1	sterol O-acyltransferase 1 [Source:MGI Symbol;Acc:MGI:104665]
ENSMUSG00000026601	1.917779432	2.457257837	2.544134977	2.470207676	3.263626246	3.271229932	0.12057393	0.321098664	0.100562841	0.217446138	0.3147748	0.215440139	Axdnd1	axonemal dynein light chain domain containing 1 [Source:MGI Symbol;Acc:MGI:1924602]
ENSMUSG00000026602	0.449917648	0.35325225	0.418747625	0.725205398	0.58014477	0.373848144	0.01825801	0.283632066	0	0	0.085116149	0.018124004	Nphs2	nephrosis 2, podocin [Source:MGI Symbol;Acc:MGI:2157018]
ENSMUSG00000026603	22.05253412	25.15467742	22.15056648	23.20410826	27.54824887	28.64750956	11.13069622	12.04119992	13.25274586	15.12803844	14.28049757	13.91127758	Smyd2	SET and MYND domain containing 2 [Source:MGI Symbol;Acc:MGI:1915889]
ENSMUSG00000026604	16.99097171	20.62359775	16.53722413	20.75167731	24.64263457	32.70406542	9.162988138	8.177157016	10.42902131	22.46842861	26.36573091	23.57362331	Ptpn14	protein tyrosine phosphatase, non-receptor type 14 [Source:MGI Symbol;Acc:MGI:102467]
ENSMUSG00000026605	9.751536141	11.73220388	8.493468903	13.05111503	15.50039205	19.83372467	29.74434273	25.27141278	35.17231757	46.48853995	43.62565922	47.78339072	Cenpf	centromere protein F [Source:MGI Symbol;Acc:MGI:1313302]
ENSMUSG00000026608	13.60305956	16.95747986	11.68133091	19.34578969	20.0529616	24.10322391	41.98128166	40.76355338	48.33602086	36.386075	39.42464328	38.98904393	Kctd3	potassium channel tetramerisation domain containing 3 [Source:MGI Symbol;Acc:MGI:2444629]
ENSMUSG00000026609	0	0.049371777	0.035115385	0.080018888	0.144147687	0.071844206	0	0.008494589	0.015962197	0.061359851	0.014275359	0.030396895	Ush2a	usherin [Source:MGI Symbol;Acc:MGI:1341292]
ENSMUSG00000026610	0.156895124	0.180672801	0.026771346	0.378230876	0.068684774	0.358512532	0.046690798	0.051808991	0.012169296	0.105254238	0.054416402	0.03476108	Esrrg	estrogen-related receptor gamma [Source:MGI Symbol;Acc:MGI:1347056]
ENSMUSG00000026611	0.322828955	0.318646403	0.661019831	0.602517352	0.339183525	0.386403244	0.494081724	0.365494943	0.171700542	0.206259198	0.115166802	0.081742561	Spata17	spermatogenesis associated 17 [Source:MGI Symbol;Acc:MGI:1921967]
ENSMUSG00000026614	0.01718047	0.019784195	0.061562338	0.06413013	0.054152508	0.104688427	0.667217469	0.570160071	0.519703503	0.453341988	0.414729243	0.342579499	Slc30a10	solute carrier family 30, member 10 [Source:MGI Symbol;Acc:MGI:2685058]
ENSMUSG00000026615	57.81399702	60.95134175	56.96880504	59.56925415	66.09158475	73.16833825	118.5380168	111.0961737	132.7417865	163.3098791	166.0399598	164.4710364	Eprs	glutamyl-prolyl-tRNA synthetase [Source:MGI Symbol;Acc:MGI:97838]
ENSMUSG00000026616	0.219941155	0.138149207	0.020470376	0.186586793	0.273098577	0.228443452	0.053552306	0.01980755	0.055830602	0.643850739	0.699028239	1.027745572	Cr2	complement receptor 2 [Source:MGI Symbol;Acc:MGI:88489]
ENSMUSG00000026617	24.79607773	24.68144422	20.82319171	21.85377607	21.83791649	26.97221661	52.9922422	52.2186703	58.15046295	73.60551761	74.0620137	74.93726185	Bpnt1	bisphosphate 3'-nucleotidase 1 [Source:MGI Symbol;Acc:MGI:1338800]
ENSMUSG00000026618	20.11703892	22.65031452	16.37377143	23.01356327	25.12681754	28.37246553	28.3751586	26.33879618	30.3398837	36.6436502	35.65879343	36.17336387	Iars2	isoleucine-tRNA synthetase 2, mitochondrial [Source:MGI Symbol;Acc:MGI:1919586]
ENSMUSG00000026620	8.023960434	9.35550295	6.400754302	3.385122257	3.947143123	3.512689848	3.507183647	2.732426135	3.438563515	7.162290556	6.999179629	6.39992806	Mark1	MAP/microtubule affinity regulating kinase 1 [Source:MGI Symbol;Acc:MGI:2664902]
ENSMUSG00000026621	0.309044388	0.054750846	0.194706078	0.110921209	0.024976971	0.108643162	0	0	0	0.04252814	0.019788329	0.105339516	Mtarc1	mitochondrial amidoxime reducing component 1 [Source:MGI Symbol;Acc:MGI:1913362]
ENSMUSG00000026622	2.518611426	3.938316399	1.004271878	3.958445135	4.42891104	6.138818406	13.04164177	10.53170975	18.01348158	17.1690004	18.16222336	15.34244409	Nek2	NIMA (never in mitosis gene a)-related expressed kinase 2 [Source:MGI Symbol;Acc:MGI:109359]
ENSMUSG00000026623	27.60860003	34.76941397	25.10445297	33.08432154	37.75847031	50.79494646	16.98779529	15.43564281	21.08553576	29.96184616	30.70658286	30.91040209	Lpgat1	lysophosphatidylglycerol acyltransferase 1 [Source:MGI Symbol;Acc:MGI:2446186]
ENSMUSG00000026626	47.63654563	42.96818219	41.9691118	45.57056733	45.64845497	47.88471304	112.6265819	110.9220599	113.769669	54.52015821	56.49003726	54.65362765	Ppp2r5a	protein phosphatase 2, regulatory subunit B', alpha [Source:MGI Symbol;Acc:MGI:2388479]
ENSMUSG00000026627	7.394922663	8.05234782	6.884389946	9.350075204	8.534428753	10.02482288	6.225737703	4.972382946	5.723854692	5.20941308	4.95950502	5.331136274	Pacc1	proton activated chloride channel 1 [Source:MGI Symbol;Acc:MGI:1914200]
ENSMUSG00000026628	7.322751502	7.623002404	7.40701552	11.56223358	11.01741182	9.950451466	1.804373677	0.957558185	2.181029355	8.226835562	7.533978573	7.086646004	Atf3	activating transcription factor 3 [Source:MGI Symbol;Acc:MGI:109384]
ENSMUSG00000026630	3.842869008	2.749025901	4.232354214	4.727134738	4.159899388	3.991423352	0.831715379	1.211289312	0.975486776	0.416649162	1.066266929	0.722409561	Batf3	basic leucine zipper transcription factor, ATF-like 3 [Source:MGI Symbol;Acc:MGI:1925491]
ENSMUSG00000026632	11.24030238	9.195359282	11.4556679	13.05225343	10.09973069	11.04089348	12.35087507	14.46054171	12.3555893	8.643869516	10.20315247	10.05158126	Tatdn3	TatD DNase domain containing 3 [Source:MGI Symbol;Acc:MGI:1916222]
ENSMUSG00000026634	11.74383638	15.41738526	10.59710155	15.02144495	15.59117814	20.83743938	14.90648339	14.83471498	17.32341896	21.49372942	21.72525406	21.87841114	Angel2	angel homolog 2 [Source:MGI Symbol;Acc:MGI:1196310]
ENSMUSG00000026637	1.696013389	1.844544012	1.254042159	1.993019338	2.804895664	1.818120853	0.50472071	0.466706362	0.701590976	0.393307686	0.94117337	0.807192873	Traf5	TNF receptor-associated factor 5 [Source:MGI Symbol;Acc:MGI:107548]
ENSMUSG00000026638	0.434033348	0.363499427	0.302972835	0.312979882	0.26946728	0.420757716	4.139152089	4.612425519	5.068118212	3.89997275	4.089134339	3.094692403	Irf6	interferon regulatory factor 6 [Source:MGI Symbol;Acc:MGI:1859211]
ENSMUSG00000026639	0.468209878	0.350459033	0.287609903	0.152924018	0.221368262	0.588434538	0.438907473	0.301488585	0.653685825	1.277348177	1.890226834	1.680503417	Lamb3	laminin, beta 3 [Source:MGI Symbol;Acc:MGI:99915]
ENSMUSG00000026640	2.942204652	3.492171229	2.529020665	2.82058269	3.724279201	5.545196024	0.654442088	0.557070588	0.953328694	3.359276411	4.271277333	3.604160186	Plxna2	plexin A2 [Source:MGI Symbol;Acc:MGI:107684]
ENSMUSG00000026641	90.38503644	75.9694722	94.12726898	84.58110517	72.93373234	62.28344156	89.73469818	99.19667487	89.56304393	79.00557159	78.7341667	82.78266321	Usf1	upstream transcription factor 1 [Source:MGI Symbol;Acc:MGI:99542]
ENSMUSG00000026643	37.63191157	43.09535468	38.41251292	45.4734027	52.40934561	62.03523221	37.47128118	36.15344507	42.12091634	53.98628405	52.7988998	54.02752815	Nmt2	N-myristoyltransferase 2 [Source:MGI Symbol;Acc:MGI:1202298]
ENSMUSG00000026644	0.403644351	0	0.247948875	0.075334855	0.169637217	0	3.964009332	4.398539631	3.982373416	3.105032934	3.427130674	4.149546852	Acbd7	acyl-Coenzyme A binding domain containing 7 [Source:MGI Symbol;Acc:MGI:1925495]
ENSMUSG00000026645	0.216223557	0.342364744	0.415065171	0.428774575	0.482752143	0.288213749	0.024129933	0	0.125782511	0	0	0	Olah	oleoyl-ACP hydrolase [Source:MGI Symbol;Acc:MGI:2139018]
ENSMUSG00000026646	3.47134142	4.071798845	3.669643356	4.670761024	4.851624408	5.029859077	11.20012819	10.52450574	11.12058992	10.45601788	10.5098674	11.34686432	Suv39h2	suppressor of variegation 3-9 2 [Source:MGI Symbol;Acc:MGI:1890396]
ENSMUSG00000026648	2.905403279	3.345721229	2.135092491	3.659389032	4.738071476	6.340293139	2.864405026	2.63100717	4.396447868	7.860218484	9.332566146	8.419568434	Dclre1c	DNA cross-link repair 1C [Source:MGI Symbol;Acc:MGI:2441769]
ENSMUSG00000026649	1.591192154	0.610779854	0.950279665	1.175525545	0.557267406	0.757488921	3.137114431	4.006435608	2.900323762	0.948855874	0.993380214	1.05761523	Cfap126	cilia and flagella associated protein 126 [Source:MGI Symbol;Acc:MGI:1922722]
ENSMUSG00000026650	0.220282361	0	0.225523465	0.20556388	0	0	0	0	0	0	0.183362844	0	Meig1	meiosis expressed gene 1 [Source:MGI Symbol;Acc:MGI:1202878]
ENSMUSG00000026655	62.45725822	68.76175532	54.01601516	80.00755111	85.80965811	92.32118489	6.365344502	6.286164781	7.200220282	18.49463173	18.45741222	17.78693069	Fam107b	family with sequence similarity 107, member B [Source:MGI Symbol;Acc:MGI:1913790]
ENSMUSG00000026656	10.85815942	12.10038265	9.626947601	0.603433606	0.509548986	0.595145021	1.804078933	1.094339233	1.680208611	13.80939557	14.26395291	13.68200024	Fcgr2b	Fc receptor, IgG, low affinity IIb [Source:MGI Symbol;Acc:MGI:95499]
ENSMUSG00000026657	26.54141995	33.32428345	21.11683015	35.11618783	39.60937231	49.52226766	6.297264655	6.543366601	8.92478086	22.6454247	23.41375708	23.68978047	Frmd4a	FERM domain containing 4A [Source:MGI Symbol;Acc:MGI:1919850]
ENSMUSG00000026659	8.922788393	7.515222164	11.24898934	6.640635152	6.856788644	4.690026946	9.282766794	10.59254142	9.300188571	9.036591933	7.028332892	7.450130479	Dusp12	dual specificity phosphatase 12 [Source:MGI Symbol;Acc:MGI:1890614]
ENSMUSG00000026662	20.51629946	22.42462408	21.40943041	21.34359541	21.02668547	22.95414066	40.63016952	36.21326719	40.95289335	36.81527581	37.43334959	38.41973807	Sephs1	selenophosphate synthetase 1 [Source:MGI Symbol;Acc:MGI:1923580]
ENSMUSG00000026663	6.621186082	7.783925193	6.363312609	7.288897498	9.407903368	10.99257229	5.318473153	4.576843056	6.643386901	11.75425462	11.58329728	12.29144635	Atf6	activating transcription factor 6 [Source:MGI Symbol;Acc:MGI:1926157]
ENSMUSG00000026664	17.86947789	19.35345787	16.0142886	16.34482745	16.96641594	14.84470346	80.6259996	80.53777813	78.40210369	33.82413045	29.5805254	29.38476773	Phyh	phytanoyl-CoA hydroxylase [Source:MGI Symbol;Acc:MGI:891978]
ENSMUSG00000026667	7.612476938	9.518454301	7.079421062	8.002796856	9.475598378	12.15320734	4.81421357	4.798972818	6.168146471	9.894358828	10.07868074	9.66079379	Uhmk1	U2AF homology motif (UHM) kinase 1 [Source:MGI Symbol;Acc:MGI:1341908]
ENSMUSG00000026668	0	0	0	0	0	0	1.310892646	1.138375755	0.297100531	1.427595192	1.541082179	1.244694218	Ucma	upper zone of growth plate and cartilage matrix associated [Source:MGI Symbol;Acc:MGI:1915777]
ENSMUSG00000026669	16.84850427	15.11450175	15.05400007	18.36153012	19.41036231	22.88437914	92.58695113	93.25863174	102.6670746	94.40722967	95.38744001	95.10254543	Mcm10	minichromosome maintenance 10 replication initiation factor [Source:MGI Symbol;Acc:MGI:1917274]
ENSMUSG00000026670	46.90948869	49.93813928	39.90615606	48.15268048	49.07318635	47.19458972	46.57989364	45.86875533	52.26806912	39.75580526	37.97993384	37.92354757	Uap1	UDP-N-acetylglucosamine pyrophosphorylase 1 [Source:MGI Symbol;Acc:MGI:1334459]
ENSMUSG00000026672	3.313473575	2.713937678	3.655094512	2.025094056	2.157446863	2.577132859	10.08285758	11.02631744	11.11993795	9.601119594	9.090183148	8.995560488	Optn	optineurin [Source:MGI Symbol;Acc:MGI:1918898]
ENSMUSG00000026674	35.82115626	45.90981832	33.11885922	49.31481668	63.12057359	86.54931271	1.357453107	1.304630842	2.395817356	19.76872174	18.69573133	18.23886009	Ddr2	discoidin domain receptor family, member 2 [Source:MGI Symbol;Acc:MGI:1345277]
ENSMUSG00000026675	10.47449516	11.58369373	8.172696283	11.19560776	14.81087534	18.01253145	10.79323514	9.713665026	12.08990472	13.74422821	14.42277399	14.57842415	Hsd17b7	hydroxysteroid (17-beta) dehydrogenase 7 [Source:MGI Symbol;Acc:MGI:1330808]
ENSMUSG00000026676	0.483466155	0.556736131	0.362977342	0.300775115	0.15238759	0.466446736	14.87676371	13.170908	14.89468444	5.362368348	5.406067722	5.327181067	Ccdc3	coiled-coil domain containing 3 [Source:MGI Symbol;Acc:MGI:1921436]
ENSMUSG00000026678	0.267812165	0.596238985	0.127952594	0.2832403	0.168827745	0.122392608	0.223156829	0.336054401	0.149561147	1.085967516	1.278112569	1.32911369	Rgs5	regulator of G-protein signaling 5 [Source:MGI Symbol;Acc:MGI:1098434]
ENSMUSG00000026679	2.86175216	2.295048823	3.191433803	3.195110469	2.630887885	1.985124765	0.273740053	0.506245222	0.428078817	0.411391565	0.127613571	1.041635164	Enkur	enkurin, TRPC channel interacting protein [Source:MGI Symbol;Acc:MGI:1918483]
ENSMUSG00000026683	16.0110933	17.19214342	14.32946532	22.71867548	24.13041339	22.93696861	30.10331098	29.95784072	32.58804789	27.58082952	26.95533211	24.65764956	Nuf2	NUF2, NDC80 kinetochore complex component [Source:MGI Symbol;Acc:MGI:1914227]
ENSMUSG00000026686	0.131424825	0.075671221	0.026910354	0.012264349	0	0	0.023466618	0.078117009	0.085627386	0.105800763	0.076578536	0.139766297	Lmx1a	LIM homeobox transcription factor 1 alpha [Source:MGI Symbol;Acc:MGI:1888519]
ENSMUSG00000026687	30.55489946	33.11579992	25.09910997	32.53874144	30.25218296	31.78833581	138.9289083	133.3971809	148.7539914	103.3098045	109.2758807	103.4398843	Aldh9a1	aldehyde dehydrogenase 9, subfamily A1 [Source:MGI Symbol;Acc:MGI:1861622]

ENSMUSG00000026688	44.40132017	31.39189914	49.83486098	40.66591967	32.55658295	25.27513351	26.64877297	28.36542281	22.63488965	24.45406797	20.17760168	20.82188688	Mgst3	microsomal glutathione S-transferase 3 [Source:MGI Symbol;Acc:MGI:1913697]
ENSMUSG00000026691	2.656722614	3.179327274	3.653243509	4.253546381	4.077505711	4.543129979	0.046507087	0	0.145456967	0.326169207	0.41193743	0.392408825	Fmo3	flavin containing monooxygenase 3 [Source:MGI Symbol;Acc:MGI:1100496]
ENSMUSG00000026692	0.123912132	0.356727974	0.222005581	0.231265531	0.162736927	0.401121923	0.027656482	0	0	0.387927667	0.283646962	0.164720971	Fmo4	flavin containing monooxygenase 4 [Source:MGI Symbol;Acc:MGI:2429497]
ENSMUSG00000026694	9.658683447	7.676685519	8.162850939	8.553821863	8.71533602	8.972465157	14.7437292	15.40309021	16.04081333	18.53249789	20.30463716	17.74052828	Eef1aknmt	EEF1A lysine and N-terminal methyltransferase [Source:MGI Symbol;Acc:MGI:1918699]
ENSMUSG00000026696	23.1455099	22.86082802	18.84352333	24.90204956	24.73845747	24.24058465	14.50861441	14.11714412	14.52426311	13.18722167	15.57701022	15.24770652	Vamp4	vesicle-associated membrane protein 4 [Source:MGI Symbol;Acc:MGI:1858730]
ENSMUSG00000026697	0.062239857	0.047781597	0.148681654	0	0.130785878	0	0	0	0.038620204	0.074229443	0.051808402	0	Myoc	myocilin [Source:MGI Symbol;Acc:MGI:1202864]
ENSMUSG00000026698	10.51455455	10.51188527	9.629461042	8.130478715	7.59366599	8.476299861	3.571008064	3.06011608	3.501766585	3.967466391	3.263571719	3.948413969	Pigc	phosphatidylinositol glycan anchor biosynthesis, class C [Source:MGI Symbol;Acc:MGI:1914542]
ENSMUSG00000026700	0.140157941	0.242098611	0	0.043597696	0.171801427	0.035585256	0	0	0	0.041789323	0	0.062105708	Tnfsf4	tumor necrosis factor (ligand) superfamily, member 4 [Source:MGI Symbol;Acc:MGI:104511]
ENSMUSG00000026701	324.8582748	277.579209	321.9229708	203.4993505	189.0594706	159.0473438	136.2450877	140.1864311	136.3065268	159.5240948	167.1832004	162.1553502	Prdx6	peroxiredoxin 6 [Source:MGI Symbol;Acc:MGI:894320]
ENSMUSG00000026705	5.677119872	8.66598223	5.455843539	8.131499277	8.045441313	11.59203854	6.525703244	6.028257959	7.551806535	10.11315602	10.77003954	9.658208496	Klhl20	kelch-like 20 [Source:MGI Symbol;Acc:MGI:2444855]
ENSMUSG00000026707	4.239571595	4.396305063	3.325513005	5.058550884	5.074855344	5.189219934	5.611581624	4.304374817	5.791421421	4.999662298	4.652682354	4.635764884	Nsun6	NOL1/NOP2/Sun domain family member 6 [Source:MGI Symbol;Acc:MGI:1921705]
ENSMUSG00000026708	3.808884123	3.815015726	3.513618849	4.146786093	3.772570595	4.502839165	8.111573612	8.312924655	8.345881276	8.0737781	8.041871073	8.63220664	Cenpl	centromere protein L [Source:MGI Symbol;Acc:MGI:1917704]
ENSMUSG00000026709	7.917730258	7.641032211	6.001441843	7.369701703	8.104991795	7.627633942	30.78276386	30.93096044	32.24392426	26.28979349	27.10832581	28.44635321	Dars2	aspartyl-tRNA synthetase 2 (mitochondrial) [Source:MGI Symbol;Acc:MGI:2442510]
ENSMUSG00000026712	0.635832034	1.21144705	0.532603777	0.356008804	0.510142227	0.704438918	0.402519965	0.561167796	0.473445101	3.371057775	3.242957291	2.878921606	Mrc1	mannose receptor, C type 1 [Source:MGI Symbol;Acc:MGI:97142]
ENSMUSG00000026715	0.107638494	0.309878146	0.082649625	0.075334855	0.254455826	0.163972586	0.120121495	0.106631264	0.225417363	0.312910296	0.111997734	0.095391882	Serpinc1	serine (or cysteine) peptidase inhibitor, clade C (antithrombin), member 1 [Source:MGI Symbol;Acc:MGI:88095]
ENSMUSG00000026718	27.51254356	36.59268706	21.77627652	37.70831083	42.05048357	55.33428678	28.30085369	23.94681951	33.56955746	41.84273837	42.5739696	41.50225985	Stam	signal transducing adaptor molecule (SH3 domain and ITAM motif) 1 [Source:MGI Symbol;Acc:MGI:1329014]
ENSMUSG00000026721	8.58159197	10.46507977	7.675209978	11.33745257	14.76551798	16.56143788	4.325706257	4.561085104	7.022624329	14.89926317	13.26327994	14.29182814	Rabgap1l	RAB GTPase activating protein 1-like [Source:MGI Symbol;Acc:MGI:1352507]
ENSMUSG00000026723	3.375273595	3.512516506	2.073348208	4.013014635	4.413130437	4.475243957	7.723149046	6.464466153	7.469946333	7.491847028	7.492199403	6.935269708	Trdmt1	tRNA aspartic acid methyltransferase 1 [Source:MGI Symbol;Acc:MGI:1274787]
ENSMUSG00000026725	0.684663821	0.606481219	0.37743666	0.131059903	0.193670886	0.167146168	0.015673135	0.017391207	0.032679846	0.109920753	0.051146089	0.101127654	Tnn	tenascin N [Source:MGI Symbol;Acc:MGI:2665790]
ENSMUSG00000026726	0.453568038	0.670293869	0.417923667	0.931176928	0.778358257	0.96157088	0.492671178	0.584120893	0.37291023	1.7580589	1.547953508	1.62795078	Cubn	cubilin (intrinsic factor-cobalamin receptor) [Source:MGI Symbol;Acc:MGI:1931256]
ENSMUSG00000026727	61.22078421	64.88392503	54.99962848	60.48300538	60.18788043	60.27031628	33.22159348	31.21375859	34.58187173	51.03672678	48.13560204	50.1110338	Rsu1	Ras suppressor protein 1 [Source:MGI Symbol;Acc:MGI:103040]
ENSMUSG00000026728	2471.927386	2191.393051	2478.050018	2470.261372	2291.323955	2001.85655	348.1072035	367.7731803	341.1574466	731.7624768	728.9660251	728.4690892	Vim	vimentin [Source:MGI Symbol;Acc:MGI:98932]
ENSMUSG00000026729	0	0.067783195	0	0.054929518	0.061844485	0.089669003	0	0	0	0.15795335	0	0	4930562F07	RIKEN cDNA 4930562F07 gene [Source:MGI Symbol;Acc:MGI:1922505]
ENSMUSG00000026730	16.65015399	18.52721019	15.89711638	22.22158308	20.94745682	23.7967889	5.320815191	5.58636886	5.447679617	9.753472223	8.965342123	8.526612229	Pter	phosphotriesterase related [Source:MGI Symbol;Acc:MGI:107372]
ENSMUSG00000026734	0	0	0.025001277	0	0	0	0	0	0	0	0	0	4921504E06	RIKEN cDNA 4921504E06 gene [Source:MGI Symbol;Acc:MGI:1918087]
ENSMUSG00000026735	0	0	0	0	0	0	0	0	0	0	0	0	Ptf1a	pancreas specific transcription factor, 1a [Source:MGI Symbol;Acc:MGI:1328312]
ENSMUSG00000026736	0.235611159	0.271318361	0.060304244	0.192384904	0.061886815	0.381354107	0.026293535	0.029175803	0	0.079030732	0.147091891	0.026100551	4930426L09	RIKEN cDNA 4930426L09 gene [Source:MGI Symbol;Acc:MGI:1921879]
ENSMUSG00000026737	35.6629889	38.92218379	34.63377181	29.0691619	32.26980775	31.88703767	14.55501913	13.95866731	13.90068656	17.34302138	17.61763767	19.27285508	Pip4k2a	phosphatidylinositol-5-phosphate 4-kinase, type II, alpha [Source:MGI Symbol;Acc:MGI:1298206]
ENSMUSG00000026739	35.0779425	46.17024652	30.95185858	40.82217176	46.71406725	53.28052522	6.884819207	6.321200254	8.384596354	11.41514625	12.27033323	12.15656438	Bmi1	Bmi1 polycomb ring finger oncogene [Source:MGI Symbol;Acc:MGI:88174]
ENSMUSG00000026740	25.84773353	28.35361398	24.60293526	19.81122762	22.857105	23.7219895	8.226171701	8.130566067	8.515028147	15.40693262	16.34167716	16.71951365	Dnajc1	DnaJ heat shock protein family (Hsp40) member C1 [Source:MGI Symbol;Acc:MGI:103268]
ENSMUSG00000026743	15.50975359	16.71211778	12.06786669	18.11248537	20.7185393	22.86755817	29.57271207	28.29285154	36.41956827	23.20772844	25.3779715	25.46775354	Mllt10	myeloid/lymphoid or mixed-lineage leukemia; translocated to, 10 [Source:MGI Symbol;Acc:MGI:1329038]
ENSMUSG00000026748	9.276847682	11.08197352	10.2215985	3.726781056	4.560168754	3.950204228	1.101811799	1.277539103	1.290659089	7.367660981	8.183746283	7.778965088	Plxdc2	plexin domain containing 2 [Source:MGI Symbol;Acc:MGI:1914698]
ENSMUSG00000026749	40.45962288	43.27125054	40.30312252	36.6341511	38.23110567	37.68648102	15.99297354	15.16066987	16.77511301	12.03894788	12.46595603	11.29818993	Nek6	NIMA (never in mitosis gene a)-related expressed kinase 6 [Source:MGI Symbol;Acc:MGI:1891638]
ENSMUSG00000026750	43.50062086	46.92994023	38.99887486	44.07870559	48.11698322	41.69901578	81.69622831	78.38478826	69.93121171	72.0041705	72.49121232	74.8393915	Psmb7	proteasome (prosome, macropain) subunit, beta type 7 [Source:MGI Symbol;Acc:MGI:107637]
ENSMUSG00000026751	0.032231077	0.018557871	0.164989701	0.060155023	0.067727818	0.024549828	0.028775172	0	0	0.028829937	0.04024368	0	Nr5a1	nuclear receptor subfamily 5, group A, member 1 [Source:MGI Symbol;Acc:MGI:1346833]
ENSMUSG00000026753	39.35856736	37.91242296	35.99324769	36.57986794	35.26137068	35.0021679	39.64944765	42.17798651	44.67782355	41.65168672	37.43284081	44.23699759	Ppp6c	protein phosphatase 6, catalytic subunit [Source:MGI Symbol;Acc:MGI:1915107]
ENSMUSG00000026754	10.34598861	13.0885513	8.05599931	12.18008254	13.10100759	15.96683057	4.386021074	4.186283059	5.037629122	7.764626017	7.554986797	7.490062556	Golga1	golgi autoantigen, golgin subfamily a, 1 [Source:MGI Symbol;Acc:MGI:1924149]
ENSMUSG00000026755	63.22797239	57.77335884	69.87176351	65.97439483	62.0044644	52.57499144	57.08874147	59.43937652	47.69742699	39.82435409	40.07012944	36.91477023	Arpc5l	actin related protein 2/3 complex, subunit 5-like [Source:MGI Symbol;Acc:MGI:1921442]
ENSMUSG00000026761	50.83765077	53.64117254	42.05350926	57.09664226	64.28442364	65.13167441	56.47192704	53.88185648	60.03738504	61.4689844	55.1553881	62.77049828	Orc4	origin recognition complex, subunit 4 [Source:MGI Symbol;Acc:MGI:1347043]
ENSMUSG00000026764	5.923821854	6.418491698	4.796678057	6.935143856	6.25221424	8.367835056	9.25514319	10.00293673	10.52605985	20.49640762	20.91185533	20.64140335	Kif5c	kinesin family member 5C [Source:MGI Symbol;Acc:MGI:1098269]
ENSMUSG00000026765	0.080804672	0.325677567	0.124090846	0	0.042449125	0	0.108210857	0.12007281	0.112814511	1.120307002	1.042556939	0.823527519	Lypd6b	LY6/PLAUR domain containing 6B [Source:MGI Symbol;Acc:MGI:1919147]
ENSMUSG00000026766	76.23120625	74.59239852	70.65523246	75.43986745	71.9712514	66.47025398	69.13842243	70.95081576	60.8972488	59.52305622	60.54757521	55.67239007	Mmadhc	methylmalonic aciduria (cobalamin deficiency) cblD type, with homocystinuria [Source:MGI Symbol;Acc:MGI:1923786]
ENSMUSG00000026767	31.94804052	37.4338339	25.15737765	44.98102838	47.26491122	51.14451149	51.77111528	54.15664159	68.45118993	43.20907354	37.70809961	41.63332567	Mindy3	MINDY lysine 48 deubiquitinase 3 [Source:MGI Symbol;Acc:MGI:1914210]
ENSMUSG00000026768	3.619598693	4.9643182	3.330983002	2.656657324	4.059348056	4.894418961	0.599097441	0.765492539	0.889560549	3.383157725	3.994698777	3.586223078	Itga8	integrin alpha 8 [Source:MGI Symbol;Acc:MGI:109442]
ENSMUSG00000026770	0.065326652	0.200605326	0.089174596	0.020320586	0	0	0	0.021571785	0	0.253210305	0.326267084	0.173682258	Il2ra	interleukin 2 receptor, alpha chain [Source:MGI Symbol;Acc:MGI:96549]
ENSMUSG00000026771	6.189267901	6.665493998	4.819330451	6.556665951	7.76674513	9.123076247	6.584069397	6.632222657	7.497045382	8.530355594	6.966004955	8.575268275	Spopl	speckle-type BTB/POZ protein-like [Source:MGI Symbol;Acc:MGI:1924107]
ENSMUSG00000026773	18.1572045	24.94283622	16.55695157	17.2527381	20.34476561	23.98388304	7.505751677	5.76441812	8.857546402	22.89085291	24.76888404	24.97351696	Pfkfb3	6-phosphofructo-2-kinase/fructose-2,6-biphosphatase 3 [Source:MGI Symbol;Acc:MGI:2181202]
ENSMUSG00000026774	0.239490656	0.0919286	0.245188777	0.049664166	0.167748862	0.20268411	0	0.052722137	0.024767564	0.142812491	0.044300403	0.117912507	Potegl	POTE ankyrin domain family, member G like [Source:MGI Symbol;Acc:MGI:1918231]
ENSMUSG00000026775	86.84161279	96.88387643	73.28221386	94.80944708	102.8888139	119.2974351	118.9278688	116.4939344	139.5595139	151.4841076	148.6189645	154.3036773	Yme1l1	YME1-like 1 (S. cerevisiae) [Source:MGI Symbol;Acc:MGI:1351651]
ENSMUSG00000026778	0.40460148	0.627978326	0.666366826	0.426815382	0.258755706	0.428768872	0.04711552	0	0	0.047205192	0	0.140309134	Prkcq	protein kinase C, theta [Source:MGI Symbol;Acc:MGI:97601]
ENSMUSG00000026779	4.941434115	6.492795775	4.540131793	8.170215896	11.84788084	14.09977743	8.744017137	7.945277266	10.30711379	15.84398616	14.22761043	13.0253738	Mastl	microtubule associated serine/threonine kinase-like [Source:MGI Symbol;Acc:MGI:1914371]
ENSMUSG00000026781	19.64381834	22.94144577	15.39067698	23.39715389	26.85443577	31.03791198	11.24640289	11.06582864	12.25928334	22.62899438	21.52198826	22.1889405	Acbd5	acyl-Coenzyme A binding domain containing 5 [Source:MGI Symbol;Acc:MGI:1921409]
ENSMUSG00000026782	12.46460163	13.58897651	12.81943789	16.58898092	19.71385663	23.62677414	19.15661374	18.06148132	25.03954888	22.19676367	22.88300378	21.79023125	Abi2	abl-interactor 2 [Source:MGI Symbol;Acc:MGI:106913]
ENSMUSG00000026784	6.117599892	5.579048515	6.052980943	8.199278871	8.498128545	9.976061625	17.08141412	17.04220438	17.08203314	15.64491763	17.15655677	15.97361179	Pdss1	prenyl (solanesyl) diphosphate synthase, subunit 1 [Source:MGI Symbol;Acc:MGI:1889278]
ENSMUSG00000026785	4.441650356	4.581998219	3.765756872	3.605177849	4.66666512	5.42709589	14.56046888	14.80446108	15.11532493	9.332297222	9.24306464	10.29178395	Pkn3	protein kinase N3 [Source:MGI Symbol;Acc:MGI:2388285]
ENSMUSG00000026786	26.43859703	29.47978442	27.28647213	28.09362549	28.900847	28.49098087	1.65872316	1.482213275	2.127180765	14.98633463	14.2747425	13.90095188	Apbb1ip	amyloid beta (A4) precursor protein-binding, family B, member 1 interacting protein [Source:MGI Symbol;Acc:MGI:1861354]
ENSMUSG00000026787	0	0.030652345	0	0.012419882	0.027966791	0	0	0	0	0.035714166	0.033235579	0.011794898	Gad2	glutamic acid decarboxylase 2 [Source:MGI Symbol;Acc:MGI:95634]
ENSMUSG00000026788	9.11564014	14.45034286	8.189419092	12.58100835	14.77762226	19.82688623	14.6696538	10.87933138	16.11582821	19.78060859	19.47594623	20.85347073	Zbtb43	zinc finger and BTB domain containing 43 [Source:MGI Symbol;Acc:MGI:1919084]
ENSMUSG00000026790	30.17552187	33.20190902	27.4556005	36.37153025	42.21551794	47.41472953	88.552648	81.05334765	98.31226055	86.64971116	91.3542373	93.21832782	Odf2	outer dense fiber of sperm tails 2 [Source:MGI Symbol;Acc:MGI:1098824]
ENSMUSG00000026791	17.95942366	15.75340904	19.38712969	18.9604911	15.25824997	14.37231915	23.37379923	20.7287795	20.79294241	15.22037824	16.91274543	15.28899546	Slc2a8	solute carrier family 2, (facilitated glucose transporter), member 8 [Source:MGI Symbol;Acc:MGI:1860103]
ENSMUSG00000026792	42.02782532	39.88780327	36.72334274	49.77881957	53.53829472	53.85140958	14.6031422	14.29757861	16.69311682	17.69482533	17.90843774	17.77034288	Lrsam1	leucine rich repeat and sterile alpha motif containing 1 [Source:MGI Symbol;Acc:MGI:2684789]
ENSMUSG00000026796	358.4841057	385.8363922	387.0820673	328.5032587	319.3056894	306.3338788	167.7791124	150.7535373	161.3714733	252.6125627	269.2600829	260.6132339	Fam129b	family with sequence similarity 129, member B [Source:MGI Symbol;Acc:MGI:2442910]
ENSMUSG00000026797	30.67662148	37.27417357	32.1513867	34.80073344	34.96844758	38.18695366	13.06679071	12.521238	14.33139123	18.60075135	18.28167691	19.83870739	Stxbp1	syntaxin binding protein 1 [Source:MGI Symbol;Acc:MGI:107363]
ENSMUSG00000026798	9.606360073	6.798654469	9.664175819	8.466469745	7.850126597	5.894242471	11.46489831	9.747771373	8.879114214	7.041209352	7.191140116	8.276909545	Coq4	coenzyme Q4 [Source:MGI Symbol;Acc:MGI:1098826]
ENSMUSG00000026799	19.16936719	15.79643959	22.68630868	18.27147357	16.75294631	12.10048991	20.51583487	23.51971161	21.27952193	13.47603182	12.73597343	12.00095502	Med27	mediator complex subunit 27 [Source:MGI Symbol;Acc:MGI:1916225]
ENSMUSG00000026803	6.878647997	8.507165979	5.781752792	8.196150569	10.64232446	12.34183837	13.60129133	12.81540175	16.08994909	16.59343838	17.15001096	15.91659585	Ttf1	transcription termination factor, RNA polymerase I [Source:MGI Symbol;Acc:MGI:105044]
ENSMUSG00000026805	0.133925929	0.066095397	0.235049801	0.196393177	0.180913679	0.218590622	0.0854043	0.189532449	0.106845224	0.051340106	0.047777068	0.016955493	Barhl1	BarH like homeobox 1 [Source:MGI Symbol;Acc:MGI:1859288]

ENSMUSG00000026806	13.58794352	12.01089074	11.85394131	12.68004563	12.16502562	10.39828082	16.08924577	15.799054	18.67377043	19.28677929	19.88646385	22.02040975	Ddx31	DEAD/H (Asp-Glu-Ala-Asp/His) box polypeptide 31 [Source:MGI Symbol;Acc:MGI:2682639]
ENSMUSG00000026807	1.025650675	1.012362408	0.60003065	1.048274677	0.872351205	1.153228876	1.177299727	1.209587052	0.909174836	0.961106992	0.731786332	0.822389545	Ak8	adenylate kinase 8 [Source:MGI Symbol;Acc:MGI:1916120]
ENSMUSG00000026809	1.56061873	1.454821702	3.195500014	2.843337809	2.498559909	1.698135784	0.663468982	1.766874516	1.383390381	1.130043918	1.29905766	1.580638536	Spaca9	sperm acrosome associated 9 [Source:MGI Symbol;Acc:MGI:1917237]
ENSMUSG00000026810	62.65711163	51.6947428	74.98651248	59.1489985	45.25605681	36.40788776	39.34784419	43.34603098	35.862387	29.2622342	24.50827382	27.09973058	Dpm2	dolichol-phosphate (beta-D) mannosyltransferase 2 [Source:MGI Symbol;Acc:MGI:1330238]
ENSMUSG00000026811	52.47107985	53.51220881	48.56175758	52.61313027	53.36490554	46.99077431	26.59300838	26.23548656	27.36563904	23.29470006	22.41132708	22.03071845	St6galnac6	ST6 (alpha-N-acetyl-neuraminyl-2,3-beta-galactosyl-1,3)-N-acetylgalactosaminide alpha-2,6-sialyltransferase 6 [Source:MGI Sym
ENSMUSG00000026812	6.459957937	8.367357551	6.222129021	7.600514181	9.730159318	11.42327743	8.913944039	8.426873086	10.49570606	11.21448552	10.51362523	10.9186698	Tsc1	TSC complex subunit 1 [Source:MGI Symbol;Acc:MGI:1929183]
ENSMUSG00000026814	14.60132517	13.47175296	14.65383812	4.817590286	4.632572272	3.965960273	20.23604311	18.65703942	22.76738583	21.30513072	24.60335319	25.23212282	Eng	endoglin [Source:MGI Symbol;Acc:MGI:95392]
ENSMUSG00000026815	0	0.048340991	0	0	0.132317029	0	0	0	0	0.037549235	0.279546345	0.260419837	Gfi1b	growth factor independent 1B [Source:MGI Symbol;Acc:MGI:1276578]
ENSMUSG00000026816	45.63222875	41.51549796	47.77738229	46.1655967	42.08687511	39.89328036	51.79597857	51.22376888	52.32177747	29.61990895	31.78782432	28.45915773	Gtf3c5	general transcription factor IIIC, polypeptide 5 [Source:MGI Symbol;Acc:MGI:1917489]
ENSMUSG00000026817	194.3308377	186.4464278	205.6000044	154.9624498	150.4600331	130.8667154	53.94807656	60.69045656	55.65150371	116.8705355	116.1402572	121.9007552	Ak1	adenylate kinase 1 [Source:MGI Symbol;Acc:MGI:87977]
ENSMUSG00000026818	0.264790694	0.038115012	0	0.030887291	0	0.025210785	0	0.065578227	0	0	0	0	Cel	carboxyl ester lipase [Source:MGI Symbol;Acc:MGI:88374]
ENSMUSG00000026819	14.93381749	14.02986813	13.96267835	12.79056901	13.4454745	11.49601146	14.34722759	14.68148584	14.99991993	11.24344712	10.55774776	9.649055122	Slc25a25	solute carrier family 25 (mitochondrial carrier, phosphate carrier), member 25 [Source:MGI Symbol;Acc:MGI:1915913]
ENSMUSG00000026820	17.25675338	14.7200286	14.90219868	14.60301374	14.21018536	11.87214164	10.84156639	11.58066873	10.30493262	10.27206386	10.26281339	10.04939989	Ptges2	prostaglandin E synthase 2 [Source:MGI Symbol;Acc:MGI:1917592]
ENSMUSG00000026821	53.46825827	53.12837179	49.45234801	49.14001408	49.48540238	51.12813901	16.90175421	15.11201213	14.44295444	20.02705473	19.74847051	19.53196085	Ralgds	ral guanine nucleotide dissociation stimulator [Source:MGI Symbol;Acc:MGI:107485]
ENSMUSG00000026822	141.6315668	108.2491038	175.4478388	20.68381094	17.33148274	14.369603	5.102621005	5.063545623	5.795449572	7.398334789	6.304698383	6.835920279	Lcn2	lipocalin 2 [Source:MGI Symbol;Acc:MGI:96757]
ENSMUSG00000026824	0.070654331	0.040681047	0.024111795	0.065933461	0.012372282	0	0.441550047	0.466621212	0.372652204	2.127687249	2.185868427	2.765522644	Kcnj3	potassium inwardly-rectifying channel, subfamily J, member 3 [Source:MGI Symbol;Acc:MGI:104742]
ENSMUSG00000026825	64.53271113	63.67355082	67.8121504	70.43222987	71.65528987	69.13532346	17.57027792	17.15357821	18.18720202	16.63569296	16.41537283	18.15175994	Dnm1	dynamin 1 [Source:MGI Symbol;Acc:MGI:107384]
ENSMUSG00000026826	3.275330739	3.556185332	2.222732067	3.982163091	3.795217678	5.345914632	0.768631269	0.482067003	1.010374419	1.506705948	1.19960827	1.44304598	Nr4a2	nuclear receptor subfamily 4, group A, member 2 [Source:MGI Symbol;Acc:MGI:1352456]
ENSMUSG00000026827	10.48938588	12.26489886	9.477316698	14.49796557	16.55429046	21.66683465	31.05408081	27.51132959	38.39693627	32.28107647	32.77616563	31.46322173	Gpd2	glycerol phosphate dehydrogenase 2, mitochondrial [Source:MGI Symbol;Acc:MGI:99778]
ENSMUSG00000026828	0.010893288	0.137985993	0	0	0.011445144	0.033188882	0	0	0	0.009743789	0.009067563	0.0193078	Galnt5	polypeptide N-acetylgalactosaminyltransferase 5 [Source:MGI Symbol;Acc:MGI:2179403]
ENSMUSG00000026829	0.126927693	0.584655052	0.129947641	0.315858233	0.266715742	0.580071162	0.52881805	0.209566606	0.630075147	1.703007362	1.831344666	1.762287631	Gbgt1	globoside alpha-1,3-N-acetylgalactosaminyltransferase 1 [Source:MGI Symbol;Acc:MGI:2449143]
ENSMUSG00000026830	0	0	0.012118913	0	0	0.02704871	0	0	0	0	0	0	Ermn	ermin, ERM-like protein [Source:MGI Symbol;Acc:MGI:1925017]
ENSMUSG00000026831	3.490610557	3.63311581	2.680246112	1.064912829	1.622139348	1.585015039	27.38781328	29.45904919	23.55464918	19.81439766	16.93059793	19.6316046	1700007K13	RIKEN cDNA 1700007K13 gene [Source:MGI Symbol;Acc:MGI:1916577]
ENSMUSG00000026832	2.536683144	3.760943338	1.753000376	3.994634862	4.997234895	4.299024078	0	0.062823652	0	0.425438095	0.448700724	0.449614204	Cytip	cytohesin 1 interacting protein [Source:MGI Symbol;Acc:MGI:2183535]
ENSMUSG00000026833	7.985543644	6.970193461	7.653697961	2.021547255	1.606613485	2.102972696	4.00075726	4.670749353	4.269800952	5.623118483	6.240551354	6.832302272	Olfm1	olfactomedin 1 [Source:MGI Symbol;Acc:MGI:1860437]
ENSMUSG00000026834	0	0	0.013264755	0	0.013612863	0.039474882	0	0	0	0.02317854	0	0	Acvr1c	activin A receptor, type IC [Source:MGI Symbol;Acc:MGI:2661081]
ENSMUSG00000026835	0	0	0	0	0	0	0	0	0	0	0	0.047547263	Fcnb	ficolin B [Source:MGI Symbol;Acc:MGI:1341158]
ENSMUSG00000026836	16.30315127	22.46057704	14.58907922	18.73720721	20.67553904	20.82005112	4.566909461	4.309123302	4.583061011	7.252483756	6.879504995	7.077639811	Acvr1	activin A receptor, type 1 [Source:MGI Symbol;Acc:MGI:87911]
ENSMUSG00000026837	194.4036458	225.3194654	184.4187943	113.5830706	119.898675	134.072209	9.080460057	8.063061999	10.01508539	65.75968549	67.2304469	68.04043849	Col5a1	collagen, type V, alpha 1 [Source:MGI Symbol;Acc:MGI:88457]
ENSMUSG00000026839	3.702918566	3.20633957	4.496327072	3.723371194	2.412718791	2.995357456	0.281896861	0.085308568	0.187020721	0.821624366	0.525664617	0.483339019	Upp2	uridine phosphorylase 2 [Source:MGI Symbol;Acc:MGI:1923904]
ENSMUSG00000026840	0.500747157	0.384424061	0.451630163	0.767689102	0.538641643	0.60843984	0.042576729	0.047243943	0.044388087	0.15996661	0.099243213	0.073962409	Lamc3	laminin gamma 3 [Source:MGI Symbol;Acc:MGI:1344394]
ENSMUSG00000026841	0.099623946	0	0.037088824	0.025354753	0.009515537	0.068983415	0.024256906	0.017943948	0.008429626	0.05670717	0.045232846	0.048157741	Fibcd1	fibrinogen C domain containing 1 [Source:MGI Symbol;Acc:MGI:2138953]
ENSMUSG00000026842	65.54500174	76.65250369	56.34795859	80.72622893	89.12756417	99.30923121	34.18767736	32.02930163	40.06523552	47.623062	51.36629009	49.40431748	Abl1	c-abl oncogene 1, non-receptor tyrosine kinase [Source:MGI Symbol;Acc:MGI:87859]
ENSMUSG00000026843	41.06578487	50.87083604	31.06842909	52.74898983	61.13433646	78.97963991	97.04805239	88.57202459	113.7395903	115.8150174	122.9335705	117.8775054	Fubp3	far upstream element (FUSE) binding protein 3 [Source:MGI Symbol;Acc:MGI:2443699]
ENSMUSG00000026848	39.49805496	35.35731224	36.77957637	35.42131759	33.9905407	34.66451611	21.83400209	20.20011579	21.74722698	33.6841476	34.10706939	32.63387816	Tor1b	torsin family 1, member B [Source:MGI Symbol;Acc:MGI:1353605]
ENSMUSG00000026849	57.55519953	52.93751669	64.76057294	64.4343202	55.59270771	48.35568643	40.83630321	42.67138814	37.13959778	31.82658758	33.59745371	34.62924037	Tor1a	torsin family 1, member A (torsin A) [Source:MGI Symbol;Acc:MGI:1353568]
ENSMUSG00000026851	51.03224959	45.62668695	47.23511839	44.9990551	40.6214636	37.08175125	49.75328909	46.88515608	43.12748754	39.43463426	38.34965991	39.4225648	BC005624	cDNA sequence BC005624 [Source:MGI Symbol;Acc:MGI:2385132]
ENSMUSG00000026853	38.9409375	38.15923538	38.42991805	41.08524817	39.864623	35.31703747	12.5635237	9.922332931	12.54621716	9.573148996	11.6878854	10.45267438	Crat	carnitine acetyltransferase [Source:MGI Symbol;Acc:MGI:109501]
ENSMUSG00000026854	21.7902538	20.64284344	18.47160525	22.25928785	21.48997712	20.89039533	7.651844666	7.339359327	8.626387127	11.27871533	10.44759636	11.50939122	Usp20	ubiquitin specific peptidase 20 [Source:MGI Symbol;Acc:MGI:1921520]
ENSMUSG00000026856	15.11033853	13.34025421	13.62820449	17.45803384	12.70066469	12.71500466	33.88601258	32.96731532	34.49049002	23.23437435	25.84445112	20.34180033	Dolpp1	dolichyl pyrophosphate phosphatase 1 [Source:MGI Symbol;Acc:MGI:1914093]
ENSMUSG00000026857	52.95407766	40.36917592	57.61926222	47.0017397	37.94170522	31.20569327	53.73366091	60.46071158	48.35382221	31.40429158	37.44291768	29.66412463	Ntmt1	N-terminal Xaa-Pro-Lys N-methyltransferase 1 [Source:MGI Symbol;Acc:MGI:1913867]
ENSMUSG00000026858	17.59491723	17.43337141	15.77476981	14.12749416	16.43617516	15.37483376	10.99283549	9.798279299	12.46251939	17.27294191	17.95044346	17.29248219	Miga2	mitoguardin 2 [Source:MGI Symbol;Acc:MGI:1922035]
ENSMUSG00000026860	86.7781235	78.61487395	87.78808359	83.79995574	76.48973256	67.87596453	50.83146692	54.83868036	48.7429817	39.50161592	39.0755537	43.33699623	Sh3glb2	SH3-domain GRB2-like endophilin B2 [Source:MGI Symbol;Acc:MGI:2385131]
ENSMUSG00000026864	660.6905962	661.5104938	649.7551572	610.174288	584.4562003	555.7022413	404.3312391	419.705279	414.2178112	506.1791854	505.9828276	492.3124726	Hspa5	heat shock protein 5 [Source:MGI Symbol;Acc:MGI:95835]
ENSMUSG00000026866	0.054743623	0.220640337	0.02802306	0.102171699	0.057516944	0.041697227	0	0	0	0.048966878	0.068352816	0.048515146	Kynu	kynureninase [Source:MGI Symbol;Acc:MGI:1918039]
ENSMUSG00000026867	22.14495465	26.27218255	21.50795712	27.00150627	30.80972257	38.08808551	28.02907919	23.58846223	31.74362716	41.49679762	43.60766164	42.34229156	Gapvd1	GTPase activating protein and VPS9 domains 1 [Source:MGI Symbol;Acc:MGI:1913941]
ENSMUSG00000026869	73.19413483	72.45708127	70.76881468	70.59862871	74.29530921	77.68288351	60.6243524	59.11991623	64.37845399	63.97139985	65.94508181	66.35163992	Psmd5	proteasome (prosome, macropain) 26S subunit, non-ATPase, 5 [Source:MGI Symbol;Acc:MGI:1914248]
ENSMUSG00000026870	0.775567553	0.785933197	0.794020371	0.231599007	0.358537492	0.496218049	0.747801774	0.921972193	0.952863735	0.887970385	1.11040061	1.484626426	Cutal	cutA divalent cation tolerance homolog-like [Source:MGI Symbol;Acc:MGI:1925246]
ENSMUSG00000026872	25.96651119	35.71471422	22.96670576	30.61914664	36.42547283	48.8611279	1.207787528	1.320181111	1.616249193	19.74066037	19.46313553	21.16904094	Zeb2	zinc finger E-box binding homeobox 2 [Source:MGI Symbol;Acc:MGI:1344407]
ENSMUSG00000026873	5.54579808	6.336572961	4.330110852	8.699248959	7.11906317	8.234595957	9.016124225	9.512789482	10.40394207	6.234510099	6.771796417	6.941948045	Phf19	PHD finger protein 19 [Source:MGI Symbol;Acc:MGI:1921266]
ENSMUSG00000026874	0.241131003	0.462791242	0.274297946	0.22501949	0.309646016	0.469366423	0.071758756	0	0.024937205	0.143790659	0.26762298	0.189952203	Hc	hemolytic complement [Source:MGI Symbol;Acc:MGI:96031]
ENSMUSG00000026875	3.326572168	2.942436222	4.61499764	5.691221228	5.622547156	4.975775682	1.807757839	1.647721844	1.929538953	1.595579558	1.745696452	1.858578548	Traf1	TNF receptor-associated factor 1 [Source:MGI Symbol;Acc:MGI:101836]
ENSMUSG00000026878	81.80340603	94.85790333	76.44751556	92.82012507	104.8865686	117.0895843	67.68331432	60.61143745	73.79762279	79.80627144	82.73072142	76.150882	Rab14	RAB14, member RAS oncogene family [Source:MGI Symbol;Acc:MGI:1915615]
ENSMUSG00000026879	678.2671791	612.2442895	698.9401975	488.5110145	449.1044611	408.5168077	33.32627011	33.4102389	34.35925834	181.2394902	182.6242745	186.555487	Gsn	gelsolin [Source:MGI Symbol;Acc:MGI:95851]
ENSMUSG00000026880	65.81790987	66.44323302	60.05382884	59.01840706	63.54730991	62.69657673	15.12362934	16.64239187	17.48747617	36.54159287	36.26482988	35.25064599	Stom	stomatin [Source:MGI Symbol;Acc:MGI:95403]
ENSMUSG00000026882	0	0	0	0	0	0	0	0	0	0	0	0	4930568D16	RIKEN cDNA 4930568D16 gene [Source:MGI Symbol;Acc:MGI:1923109]
ENSMUSG00000026883	13.75740767	15.66871923	13.249275	12.5402018	13.85791989	15.9917763	14.72133864	13.31233957	17.8904373	22.47709931	23.27986155	21.93958709	Dab2ip	disabled 2 interacting protein [Source:MGI Symbol;Acc:MGI:1916851]
ENSMUSG00000026885	3.927600342	3.399327235	3.739318912	2.497919842	3.022649192	2.381832895	0.067002652	0.099129878	0.093137562	0.492287942	0.56224165	0.509916749	Ttll11	tubulin tyrosine ligase-like family, member 11 [Source:MGI Symbol;Acc:MGI:1921660]
ENSMUSG00000026887	20.32062551	16.62825423	17.91299213	16.92531576	18.10779734	14.39129864	20.53297342	22.66841427	20.29554431	13.95774694	15.24277042	14.21598815	Mrrf	mitochondrial ribosome recycling factor [Source:MGI Symbol;Acc:MGI:1915121]
ENSMUSG00000026888	43.71303195	42.68015096	40.2811106	29.29378724	29.45390438	26.07024369	3.492259461	1.71149237	3.731852756	14.92866574	13.26852433	12.62430254	Grb14	growth factor receptor bound protein 14 [Source:MGI Symbol;Acc:MGI:1355324]
ENSMUSG00000026889	27.25470343	27.30396743	25.29682581	29.64929077	32.33286341	28.62974193	24.19868418	24.7744196	25.22817782	19.33328241	17.99153954	21.54376402	Rbm18	RNA binding motif protein 18 [Source:MGI Symbol;Acc:MGI:1915139]
ENSMUSG00000026890	5.371685954	5.742423626	4.880095889	8.999032527	7.377411984	8.238888869	1.947749312	1.652727797	2.320700727	1.033123938	1.403984893	1.511018379	Lhx6	LIM homeobox protein 6 [Source:MGI Symbol;Acc:MGI:1306803]
ENSMUSG00000026893	2.109694177	1.761997862	1.613983305	1.947097524	1.75377097	1.960084208	6.188599875	7.096652446	6.387149165	14.95141366	13.99096698	15.36821805	Gca	grancalcin [Source:MGI Symbol;Acc:MGI:1918521]
ENSMUSG00000026894	1.145015783	0.208191242	0.863769612	0.224949456	0.063316972	0.229509948	1.076046333	1.49250167	1.065733724	0.323428289	0.351145838	0.267037148	Morn5	MORN repeat containing 5 [Source:MGI Symbol;Acc:MGI:1922745]
ENSMUSG00000026895	156.9215468	109.9757542	193.0931385	122.4657756	99.76283956	74.72172185	143.6653079	167.2854116	116.6588525	69.15317535	78.24801716	69.26813349	Ndufa8	NADH:ubiquinone oxidoreductase subunit A8 [Source:MGI Symbol;Acc:MGI:1915625]
ENSMUSG00000026896	13.31458376	16.75332072	12.00089644	16.56213807	19.23501641	21.71456452	0.473941181	0.198986952	0.667708428	7.340819195	7.547937747	6.573761829	Ifih1	interferon induced with helicase C domain 1 [Source:MGI Symbol;Acc:MGI:1918836]
ENSMUSG00000026904	0.055538545	0.047966617	0.11371991	0.129569144	0.029176069	0.116332439	0	0	0	0.024838959	0	0.086134344	Slc4a10	solute carrier family 4, sodium bicarbonate cotransporter-like, member 10 [Source:MGI Symbol;Acc:MGI:2150150]

ENSMUSG00000026914	187.7890608	175.1133099	197.5637926	187.8879718	187.7883993	162.5857802	238.1832461	238.0613381	220.938788	222.1018205	223.7904919	225.0892937	Psmd14	proteasome (prosome, macropain) 26S subunit, non-ATPase, 14 [Source:MGI Symbol;Acc:MGI:1913284]
ENSMUSG00000026915	12.11887417	12.42886376	10.87290971	14.02547802	16.59354217	19.46026075	43.48365933	40.02775517	48.04288706	30.34181944	30.87496232	29.37229765	Strbp	spermatid perinuclear RNA binding protein [Source:MGI Symbol;Acc:MGI:104626]
ENSMUSG00000026917	52.72504847	56.56505119	49.70164922	57.89363557	57.3398315	58.11375639	217.4753451	192.3060853	220.938314	157.8320459	163.0743137	152.3902811	Wdr5	WD repeat domain 5 [Source:MGI Symbol;Acc:MGI:2155884]
ENSMUSG00000026918	22.56924679	25.89965601	19.92192208	23.27822398	27.1282753	31.5597271	32.61006682	27.49854904	35.393965	37.48137953	39.81098401	42.66231255	Brd3	bromodomain containing 3 [Source:MGI Symbol;Acc:MGI:1914632]
ENSMUSG00000026919	0	0	0	0	0	0	0	0	0	0	0.046275033	0	Lcn4	lipocalin 4 [Source:MGI Symbol;Acc:MGI:102668]
ENSMUSG00000026921	6.589505565	4.473613411	11.12633976	6.470446143	4.856665057	3.595778264	22.21476133	25.52679337	14.6923173	9.896905638	9.53752227	9.326218975	Egfl7	EGF-like domain 7 [Source:MGI Symbol;Acc:MGI:2449923]
ENSMUSG00000026922	31.70757541	31.78648172	31.07669223	29.71525718	25.49483781	23.77318897	46.58908403	46.55779172	41.51846002	31.66986751	32.06248602	31.85736377	Agpat2	1-acylglycerol-3-phosphate O-acyltransferase 2 (lysophosphatidic acid acyltransferase, beta) [Source:MGI Symbol;Acc:MGI:1914762
ENSMUSG00000026923	6.91175183	12.75435195	6.380181069	7.665904261	13.00594807	20.54040003	16.28648191	11.11246589	23.2016151	27.41539145	33.31747535	29.97411995	Notch1	notch 1 [Source:MGI Symbol;Acc:MGI:97363]
ENSMUSG00000026924	22.75571265	26.9427455	22.71051326	23.4249839	26.74658276	36.25509131	15.56568913	14.60955655	20.15155468	36.16941418	42.67590934	40.65978392	Sec16a	SEC16 homolog A, endoplasmic reticulum export factor [Source:MGI Symbol;Acc:MGI:2139207]
ENSMUSG00000026925	7.098909527	10.56570554	6.336559886	10.01991657	10.4918202	13.46506179	3.265968751	2.742471819	4.067491952	6.137557137	6.106645589	6.09846086	Inpp5e	inositol polyphosphate-5-phosphatase E [Source:MGI Symbol;Acc:MGI:1927753]
ENSMUSG00000026926	59.50119647	73.79262627	56.34150454	78.72149646	90.49424754	94.72113947	110.2324954	100.1433006	109.1124523	99.20975366	100.5946923	97.67260738	Pmpca	peptidase (mitochondrial processing) alpha [Source:MGI Symbol;Acc:MGI:1918568]
ENSMUSG00000026927	47.94800898	39.29651601	51.3233543	43.51576583	41.38635907	30.71574881	38.10555573	44.41050477	37.08254771	25.96853126	26.47334275	24.22452641	Entr1	endosome associated trafficking regulator 1 [Source:MGI Symbol;Acc:MGI:1915362]
ENSMUSG00000026928	1.18863226	0.54750846	0.608456494	0.194112117	0.156106072	0.090535969	0.822417456	1.471887006	0.636139838	1.275844189	1.484124675	1.685432251	Card9	caspase recruitment domain family, member 9 [Source:MGI Symbol;Acc:MGI:2685628]
ENSMUSG00000026930	21.48641177	25.5760113	19.00572502	22.57009847	21.69765344	23.97633722	8.324046645	7.940649507	8.963134817	10.43108532	8.780463006	8.410944695	Gpsm1	G-protein signalling modulator 1 (AGS3-like, C. elegans) [Source:MGI Symbol;Acc:MGI:1915089]
ENSMUSG00000026931	0.088082882	0	0	0	0	0	0	0.174517288	0	0.118182087	0.476580738	0.156122435	1700019N1[illegible]	RIKEN cDNA 1700019N19 gene [Source:MGI Symbol;Acc:MGI:1914757]
ENSMUSG00000026932	21.95742122	24.171627	20.2318621	21.9189865	25.44026154	26.08419245	5.287437779	4.969024381	5.981123285	6.558810749	6.656975452	6.373336598	Nacc2	nucleus accumbens associated 2, BEN and BTB (POZ) domain containing [Source:MGI Symbol;Acc:MGI:1915241]
ENSMUSG00000026933	16.50218495	20.71781388	16.65907428	20.49933171	24.48313495	25.75853281	17.58339557	14.41644796	21.41765185	26.37723275	27.74114758	27.01817649	Camsap1	calmodulin regulated spectrin-associated protein 1 [Source:MGI Symbol;Acc:MGI:3036242]
ENSMUSG00000026934	0	0	0	0.02333148	0.183880435	0.057130774	0	0.02476807	0.023270861	0	0.020811665	0	Lhx3	LIM homeobox protein 3 [Source:MGI Symbol;Acc:MGI:102673]
ENSMUSG00000026936	0	0.072440812	0	0	0	0	0	0	0	0	0	0	Lcn3	lipocalin 3 [Source:MGI Symbol;Acc:MGI:102669]
ENSMUSG00000026937	0	0	0	0	0	0	0	0	0	0	0	0	Lcn5	lipocalin 5 [Source:MGI Symbol;Acc:MGI:1277241]
ENSMUSG00000026938	2.180347367	0.819847208	1.412223119	1.577902955	1.870044192	1.491268778	0.039725806	0.132241513	0	0.358212717	0.222235008	0.157736938	Fcna	ficolin A [Source:MGI Symbol;Acc:MGI:1340905]
ENSMUSG00000026939	36.36749416	32.18073524	48.2066439	39.22466073	32.82027382	25.42610854	9.7745981	12.36301277	7.553754558	8.697458593	8.093849085	6.64951007	Tmem141	transmembrane protein 141 [Source:MGI Symbol;Acc:MGI:1098773]
ENSMUSG00000026940	0.441506502	0.444865254	0.696850473	0.274671075	0.09664027	0.336287514	0	0.054671887	0.017122339	0.148093928	0.015312901	0.03260616	Ccdc183	coiled-coil domain containing 183 [Source:MGI Symbol;Acc:MGI:1924308]
ENSMUSG00000026941	1.296157096	0.904600924	1.608480153	1.099593186	1.134850529	2.049311195	0.403259536	0.428009425	0.383857682	0.632390137	1.291434579	1.131281974	Mamdc4	MAM domain containing 4 [Source:MGI Symbol;Acc:MGI:2685841]
ENSMUSG00000026942	8.60847002	9.194755477	8.99778564	9.909009028	9.612593463	9.682273494	6.633492842	6.646556149	5.857704508	5.765755272	6.000249758	5.1720209	Traf2	TNF receptor-associated factor 2 [Source:MGI Symbol;Acc:MGI:101835]
ENSMUSG00000026943	0	0	0	0	0	0	0	0	0	0	0	0	Lcn12	lipocalin 12 [Source:MGI Symbol;Acc:MGI:1924951]
ENSMUSG00000026944	32.49296638	38.46722255	29.63609298	35.00135413	39.2213427	46.87514429	15.87241175	15.75575771	21.167827	18.03203133	19.98493111	18.47167779	Abca2	ATP-binding cassette, sub-family A (ABC1), member 2 [Source:MGI Symbol;Acc:MGI:99606]
ENSMUSG00000026946	6.737031808	4.926637553	6.645596601	2.753381337	1.498332837	1.648065038	3.775631372	3.848505107	3.753177628	7.653607027	9.864990846	8.323868338	Nmi	N-myc (and STAT) interactor [Source:MGI Symbol;Acc:MGI:1928368]
ENSMUSG00000026950	0.21841872	0.203611739	0.069214316	0.092206439	0.284122861	0.221820783	0.584997778	0.571847848	0.784138572	0.762874847	0.922044293	0.884882487	Neb	nebulin [Source:MGI Symbol;Acc:MGI:97292]
ENSMUSG00000026955	4.854984246	4.969567939	4.710601975	5.033990584	4.10075454	4.44721224	20.36893811	21.4700988	21.08782757	11.94943499	14.97652537	12.99218434	Sapcd2	suppressor APC domain containing 2 [Source:MGI Symbol;Acc:MGI:1919330]
ENSMUSG00000026956	137.4849908	109.4310066	144.5476675	106.8146642	87.96969134	76.5696507	27.0301502	28.63155875	26.72312506	23.66651348	27.93525603	27.88066856	Uap1l1	UDP-N-acteylglucosamine pyrophosphorylase 1-like 1 [Source:MGI Symbol;Acc:MGI:2443318]
ENSMUSG00000026958	84.11875548	77.59105149	84.09975038	77.8076401	70.71899103	66.21909002	26.14475326	24.50724413	27.15863838	24.83908067	24.37659584	24.39131741	Dpp7	dipeptidylpeptidase 7 [Source:MGI Symbol;Acc:MGI:1933213]
ENSMUSG00000026959	0.220057047	0.0675752	0.195253751	0.232734103	0.138723104	0.100568082	2.3706405	2.107312621	2.225711455	1.482829935	1.453190652	1.547158424	Grin1	glutamate receptor, ionotropic, NMDA1 (zeta 1) [Source:MGI Symbol;Acc:MGI:95819]
ENSMUSG00000026960	18.09121086	21.59282406	13.6519753	26.83740436	26.02728104	31.05678761	39.34761091	37.36911377	43.22237503	35.04495341	36.11439585	34.59982406	Arl6ip6	ADP-ribosylation factor-like 6 interacting protein 6 [Source:MGI Symbol;Acc:MGI:1929507]
ENSMUSG00000026961	0	0	0	0	0	0	0	0.10746012	0	0.097028487	0.030098215	0.128177831	Lrrc26	leucine rich repeat containing 26 [Source:MGI Symbol;Acc:MGI:2385129]
ENSMUSG00000026963	0	0.470779246	0	0	0	0	0	0	0	0.091420348	0.340302855	0	Tmem210	transmembrane protein 210 [Source:MGI Symbol;Acc:MGI:1925467]
ENSMUSG00000026965	138.3463533	130.9471094	129.5123256	129.7786271	116.4627176	113.5094375	90.300217	89.24591832	87.42794278	88.89424151	91.87579186	91.64738367	Anapc2	anaphase promoting complex subunit 2 [Source:MGI Symbol;Acc:MGI:2139135]
ENSMUSG00000026966	94.01538894	74.72140272	118.9030526	92.53618998	76.25768126	65.54804127	71.33510509	81.88788005	59.4564469	38.91508314	36.17018621	40.62494408	Ssna1	SS nuclear autoantigen 1 [Source:MGI Symbol;Acc:MGI:1915725]
ENSMUSG00000026969	0.249440509	0.215432677	0.063843841	0.407354123	0.393115812	0.332490064	0.556737016	0.247106363	0.290211243	0.111559323	0.259542576	0	Fam166a	family with sequence similarity 166, member A [Source:MGI Symbol;Acc:MGI:3605773]
ENSMUSG00000026970	127.6712157	153.5749983	113.4788861	179.3214068	211.7770114	237.5510567	47.4109374	43.71163347	56.07539375	86.59001802	87.05531443	87.85921508	Rbms1	RNA binding motif, single stranded interacting protein 1 [Source:MGI Symbol;Acc:MGI:1861774]
ENSMUSG00000026971	0.066163743	0.12698492	0.022579318	0.102904864	0	0	0	0.087392819	0	0.039454604	0.055074642	0	Itgb6	integrin beta 6 [Source:MGI Symbol;Acc:MGI:96615]
ENSMUSG00000026972	13.49725647	12.51139049	14.84742137	13.48871002	8.900859119	9.733774761	23.33093473	23.4228796	22.76431193	15.71199517	14.34268651	14.97320952	Arrdc1	arrestin domain containing 1 [Source:MGI Symbol;Acc:MGI:2446136]
ENSMUSG00000026974	17.00166778	16.33174207	16.94858847	15.977972	16.58059778	14.53422409	39.67335109	42.31930944	38.88111794	32.5217934	30.85145167	29.76895556	Zmynd19	zinc finger, MYND domain containing 19 [Source:MGI Symbol;Acc:MGI:1914437]
ENSMUSG00000026975	11.90391646	13.00947635	14.08895755	15.91988108	17.36636722	18.0184924	18.31051635	19.56657855	16.54894455	19.66785947	20.92210435	19.21764007	Dph7	diphthamine biosynthesis 7 [Source:MGI Symbol;Acc:MGI:1914478]
ENSMUSG00000026976	0.243442647	0.560673444	0.062308699	0.425956271	0.543522922	0.139069394	1.575715372	2.622665311	1.812691441	1.524275896	1.975754093	1.537182155	Pax8	paired box 8 [Source:MGI Symbol;Acc:MGI:97492]
ENSMUSG00000026977	39.88380333	53.89945082	34.53115334	60.64177201	67.85277014	88.2590939	47.64195137	45.43717812	57.59296433	77.64484125	76.61363739	84.23565443	Marchf7	membrane associated ring-CH-type finger 7 [Source:MGI Symbol;Acc:MGI:1931053]
ENSMUSG00000026979	1.271420071	1.652347714	1.357456422	0.542385517	0.515248879	0.650223288	3.372853376	3.274759089	4.141849778	6.319889793	7.151279908	7.629800263	Psd4	pleckstrin and Sec7 domain containing 4 [Source:MGI Symbol;Acc:MGI:2674093]
ENSMUSG00000026980	5.637822407	6.426581205	4.575246128	6.3918324	7.645799741	10.09767739	10.93203898	10.01216698	14.05733843	13.87912917	14.77883664	14.84385059	Ly75	lymphocyte antigen 75 [Source:MGI Symbol;Acc:MGI:106662]
ENSMUSG00000026981	27.09310992	30.68652761	25.76297115	0.858453664	0.872988216	1.084933097	1.245171132	1.264076863	2.402949091	36.94844772	34.35949917	36.34460295	Il1rn	interleukin 1 receptor antagonist [Source:MGI Symbol;Acc:MGI:96547]
ENSMUSG00000026983	0	0.167171105	0	0	0	0	0	0	0	0	0	0	Il1f5	interleukin 1 family, member 5 (delta) [Source:MGI Symbol;Acc:MGI:1859325]
ENSMUSG00000026984	0	0.14551987	0	0	0	0.240631576	0	0.37555813	0	0.169550513	0.420756246	0.11199091	Il1f6	interleukin 1 family, member 6 [Source:MGI Symbol;Acc:MGI:1859324]
ENSMUSG00000026985	0	0	0	0	0	0.026930173	0	0	0	0	0	0	Il1f8	interleukin 1 family, member 8 [Source:MGI Symbol;Acc:MGI:1916927]
ENSMUSG00000026986	0.271377989	0.416674273	0.246964262	0.056276773	0.095042015	0.045934156	0.02692001	0	0	0.377597428	0.301193066	0.427558847	Hnmt	histamine N-methyltransferase [Source:MGI Symbol;Acc:MGI:2153181]
ENSMUSG00000026987	11.2539072	13.5032059	9.769246157	11.38324475	13.53975698	19.19685434	9.327063334	8.770750556	12.1411008	20.46708043	20.78263145	20.31313853	Baz2b	bromodomain adjacent to zinc finger domain, 2B [Source:MGI Symbol;Acc:MGI:2442782]
ENSMUSG00000026988	23.7802412	26.68050841	28.93372646	30.83977173	28.19501038	26.29678816	21.59414874	17.9583453	20.61701883	18.62908668	19.0740757	18.59261152	Wdsub1	WD repeat, SAM and U-box domain containing 1 [Source:MGI Symbol;Acc:MGI:1919387]
ENSMUSG00000026989	0	0	0	0	0	0	0.062159958	0.137947727	0.064804452	0	0	0.061703729	Dapl1	death associated protein-like 1 [Source:MGI Symbol;Acc:MGI:1923997]
ENSMUSG00000026991	28.24897721	29.76795514	25.42638455	33.00781175	35.47651054	38.19887997	44.29568722	40.70658783	47.96624365	49.19924003	48.73368177	46.70136183	Pkp4	plakophilin 4 [Source:MGI Symbol;Acc:MGI:109281]
ENSMUSG00000026994	1.433307888	1.583159235	0.688784333	0.846198377	0.645394076	0.623842677	1.723576049	1.072166122	1.769676988	3.924673661	4.187969125	4.614314775	Galnt3	polypeptide N-acetylgalactosaminyltransferase 3 [Source:MGI Symbol;Acc:MGI:894695]
ENSMUSG00000026999	22.71536755	21.80504136	19.16887734	25.18658216	24.71981939	26.77352261	46.36275491	45.67055111	44.1921672	43.89144934	44.22707777	43.67438591	Nup35	nucleoporin 35 [Source:MGI Symbol;Acc:MGI:1916732]
ENSMUSG00000027001	3.165394012	3.925507301	3.311931502	5.031357368	5.372370895	8.001418134	3.291814496	2.894560394	2.072066045	2.986939993	3.185009137	3.329307766	Dusp19	dual specificity phosphatase 19 [Source:MGI Symbol;Acc:MGI:1915332]
ENSMUSG00000027002	81.11072829	92.57994365	70.34117724	91.1251356	107.2251179	130.0070491	65.1689801	59.94798159	76.93321518	97.68028107	98.20289253	100.9400418	Nckap1	NCK-associated protein 1 [Source:MGI Symbol;Acc:MGI:1355333]
ENSMUSG00000027004	4.06500287	5.876223963	3.962488837	1.815986435	1.794702085	1.663405068	0.250951308	0.278460312	0.503130044	5.415392253	4.571601192	5.940301735	Frzb	frizzled-related protein [Source:MGI Symbol;Acc:MGI:892032]
ENSMUSG00000027006	106.3361708	137.1303037	98.0326475	118.6960709	129.4092118	145.2648654	51.5918841	47.33855659	60.57043894	98.98854002	104.7853026	105.2100623	Dnajc10	DnaJ heat shock protein family (Hsp40) member C10 [Source:MGI Symbol;Acc:MGI:1914111]
ENSMUSG00000027007	6.599717178	8.860562765	5.294382384	8.40625269	11.04191541	13.19855156	5.445284621	4.823424007	6.792926112	8.617126467	8.947709866	9.590975301	Itprid2	ITPR interacting domain containing 2 [Source:MGI Symbol;Acc:MGI:1917849]
ENSMUSG00000027009	3.341449327	5.116966327	3.36093462	1.630209318	2.291211775	4.07219439	0.54429766	0.557504275	0.698404822	13.30823687	14.14132223	14.74403045	Itga4	integrin alpha 4 [Source:MGI Symbol;Acc:MGI:96603]
ENSMUSG00000027010	30.23066709	35.22730375	27.10096541	32.82444687	34.52173202	37.67737501	37.49528844	35.02411065	40.4805607	43.2327426	42.99739809	42.85672951	Slc25a12	solute carrier family 25 (mitochondrial carrier, Aralar), member 12 [Source:MGI Symbol;Acc:MGI:1926080]
ENSMUSG00000027011	77.52766002	77.35392824	70.81377497	85.69243199	87.20657859	79.02611485	56.10690225	53.72370154	54.14929659	43.80188678	43.55247285	45.42854918	Ube2e3	ubiquitin-conjugating enzyme E2E 3 [Source:MGI Symbol;Acc:MGI:107412]

ENSMUSG00000027012	78.7543952	91.99644224	63.40825805	89.71766017	96.95463255	100.6361782	41.68281273	39.72489677	46.8971987	71.1478953	70.59778859	70.42665766	Dync1i2	dynein cytoplasmic 1 intermediate chain 2 [Source:MGI Symbol;Acc:MGI:107750]
ENSMUSG00000027014	34.67602968	38.26849529	30.7023943	37.62445135	41.40400452	40.43877611	35.14255107	35.27580478	35.85195082	44.78671977	44.47061721	41.53868296	Cwc22	CWC22 spliceosome-associated protein [Source:MGI Symbol;Acc:MGI:2136773]
ENSMUSG00000027015	0.145067013	0.111368082	0.074259272	0.12033258	0.110078318	0.085940492	0.086341684	0.103790227	0.067511213	0.129759018	0.134170727	0.199985265	Cybrd1	cytochrome b reductase 1 [Source:MGI Symbol;Acc:MGI:2654575]
ENSMUSG00000027016	0.491902087	1.073274707	0.689144708	1.304625634	0.870436936	1.124018758	0.115568153	0.102589284	0.072290887	0.486310044	0.280156066	0.34415979	Zfp385b	zinc finger protein 385B [Source:MGI Symbol;Acc:MGI:2444734]
ENSMUSG00000027018	54.21085472	54.66407031	45.69695354	65.13917049	59.77029498	60.58155352	341.4872423	340.1018405	332.3845028	252.087727	240.2824648	244.4860756	Hat1	histone aminotransferase 1 [Source:MGI Symbol;Acc:MGI:96013]
ENSMUSG00000027022	0.523899752	0.356044424	0.38688603	0.496909307	0.50532252	0.673797231	0.176355617	0.042540766	0.119907639	0.153644576	0.092938	0.068502224	Xirp2	xin actin-binding repeat containing 2 [Source:MGI Symbol;Acc:MGI:2685198]
ENSMUSG00000027030	20.87202768	21.59471427	21.96037547	25.97989947	24.9995253	24.36039146	1.920742704	2.385775058	2.988742651	6.692310571	5.933840564	7.48429398	Stk39	serine/threonine kinase 39 [Source:MGI Symbol;Acc:MGI:1858416]
ENSMUSG00000027035	17.08202217	21.49168857	15.64885057	17.48330716	19.28149881	26.2776112	13.17569605	12.46022831	14.22681441	23.80853599	22.81431267	23.24918187	Cers6	ceramide synthase 6 [Source:MGI Symbol;Acc:MGI:2442564]
ENSMUSG00000027048	0.055420515	0	0.03404347	0.020687006	0.046582502	0.033770243	0.009895635	0	0	0.039657874	0.027679193	0.049115024	Abcb11	ATP-binding cassette, sub-family B (MDR/TAP), member 11 [Source:MGI Symbol;Acc:MGI:1351619]
ENSMUSG00000027067	241.9514658	221.120502	256.3882132	276.6024232	253.1877081	232.8640111	356.6238789	353.9870826	338.2032321	256.0768666	259.6444525	252.7938354	Ssrp1	structure specific recognition protein 1 [Source:MGI Symbol;Acc:MGI:107912]
ENSMUSG00000027068	2.613269525	4.004702734	2.510803328	5.815266587	8.131372373	11.45291634	0.089733366	0.169268725	0.205812038	1.825054233	1.338635851	1.08671207	Dhrs9	dehydrogenase/reductase (SDR family) member 9 [Source:MGI Symbol;Acc:MGI:2442798]
ENSMUSG00000027070	0.897385575	1.536829896	0.730277944	1.223929538	0.958964329	1.291097783	2.095355677	2.271858906	2.405804646	7.354563271	8.229581549	7.646972865	Lrp2	low density lipoprotein receptor-related protein 2 [Source:MGI Symbol;Acc:MGI:95794]
ENSMUSG00000027071	0.933840303	0.49632236	0.919287358	1.943991414	2.113243596	2.708370654	0.384789857	0.32022757	0.468020135	0.706790701	0.388663924	0.795761784	P2rx3	purinergic receptor P2X, ligand-gated ion channel, 3 [Source:MGI Symbol;Acc:MGI:1097160]
ENSMUSG00000027072	0.060179703	0	0	0	0	0	0	0	0	0	0	0	Prg3	proteoglycan 3 [Source:MGI Symbol;Acc:MGI:1858200]
ENSMUSG00000027073	0	0.0591283	0.052568318	0.143747534	0.053947874	0.03910981	0	0	0	0	0.042740902	0	Prg2	proteoglycan 2, bone marrow [Source:MGI Symbol;Acc:MGI:103294]
ENSMUSG00000027074	52.89740705	59.50900959	47.3221837	66.10756067	76.69200961	72.70967047	2.723321598	2.453030435	2.271344707	13.57832342	14.24908224	16.06098154	Slc43a3	solute carrier family 43, member 3 [Source:MGI Symbol;Acc:MGI:1931054]
ENSMUSG00000027075	1.176760071	1.119430198	1.431741782	1.002643323	1.110945596	0.79239606	2.877688236	2.145653055	3.015988422	3.111987768	3.435467248	3.189077641	Slc43a1	solute carrier family 43, member 1 [Source:MGI Symbol;Acc:MGI:1931352]
ENSMUSG00000027076	24.63672589	20.70490127	30.21127207	25.36009126	21.48656917	14.79273978	35.78034255	34.2638393	30.02089508	27.43878942	23.53517177	26.69911509	Timm10	translocase of inner mitochondrial membrane 10 [Source:MGI Symbol;Acc:MGI:1353429]
ENSMUSG00000027077	0.024434121	0	0	0.022801521	0	0.037222056	1.941466219	1.186068448	1.182598455	1.070931489	0.752540825	0.887818905	Smtnl1	smoothelin-like 1 [Source:MGI Symbol;Acc:MGI:1915928]
ENSMUSG00000027078	150.6475168	139.509414	171.2164124	123.9850166	108.600427	91.87650452	13.06105038	11.7931512	12.63773226	24.7605917	24.3156788	22.49841377	Ube2l6	ubiquitin-conjugating enzyme E2L 6 [Source:MGI Symbol;Acc:MGI:1914500]
ENSMUSG00000027079	8.128864719	8.367200527	8.368765209	9.344424129	8.922380508	8.163238913	29.65748364	29.15203093	25.5088791	25.22535843	25.47817844	25.13350816	Clp1	CLP1, cleavage and polyadenylation factor I subunit [Source:MGI Symbol;Acc:MGI:2138968]
ENSMUSG00000027080	21.80231342	21.16068109	17.44705047	24.98627268	22.65194809	22.05721614	29.56238241	33.46384379	32.76748761	27.44878222	27.18447843	26.33562128	Med19	mediator complex subunit 19 [Source:MGI Symbol;Acc:MGI:1914234]
ENSMUSG00000027082	31.93661943	36.54745602	27.39835073	11.05993607	13.89709029	12.67960428	33.16471632	34.41615962	35.18195018	36.22206423	36.23860488	37.411298	Tfpi	tissue factor pathway inhibitor [Source:MGI Symbol;Acc:MGI:1095418]
ENSMUSG00000027086	4.478219621	5.192962336	3.92751139	5.567130661	5.428946527	7.120120204	13.96522533	13.6191755	16.83288574	13.47358939	14.68908976	16.27725517	Fastkd1	FAST kinase domains 1 [Source:MGI Symbol;Acc:MGI:2444596]
ENSMUSG00000027087	70.79572821	89.06551187	60.83057086	80.19008484	99.60868147	126.8170628	15.68539987	14.85258228	18.95049249	67.2619221	69.84663035	74.25213832	Itgav	integrin alpha V [Source:MGI Symbol;Acc:MGI:96608]
ENSMUSG00000027088	17.27895194	17.71335341	17.58365784	18.84012819	17.52641327	16.84220505	6.866423192	7.078567704	8.295204693	12.24828398	11.33335915	11.97410051	Phospho2	phosphatase, orphan 2 [Source:MGI Symbol;Acc:MGI:1920623]
ENSMUSG00000027091	132.7682918	131.4162668	127.240844	148.6940273	149.3392878	147.6073714	178.7560111	186.2514017	175.4265651	189.0622313	184.5946659	178.022412	Zc3h15	zinc finger CCCH-type containing 15 [Source:MGI Symbol;Acc:MGI:1919747]
ENSMUSG00000027099	49.4381719	54.45953887	50.64296898	57.9676827	60.2289578	56.99368226	36.2751666	38.07062434	37.79249596	38.03927246	36.83756364	35.44087968	Mtx2	metaxin 2 [Source:MGI Symbol;Acc:MGI:1859652]
ENSMUSG00000027102	3.233656055	3.573571127	2.910118346	2.263196023	1.890529676	1.211645611	0.442352865	0.594178483	0.291266557	1.912735298	2.496327685	1.802646403	Hoxd8	homeobox D8 [Source:MGI Symbol;Acc:MGI:96209]
ENSMUSG00000027104	22.01209419	24.69285779	18.96795236	26.46479221	28.69420537	38.46207381	24.8585378	24.81810719	29.67278045	46.13784729	44.11987843	45.36555066	Atf2	activating transcription factor 2 [Source:MGI Symbol;Acc:MGI:109349]
ENSMUSG00000027107	0.765212506	0.813398341	0.582542296	0.732393577	0.907052443	0.866800363	0.245238466	0.242965388	0.319589672	0.754666007	0.710457858	0.599902057	Chrna1	cholinergic receptor, nicotinic, alpha polypeptide 1 (muscle) [Source:MGI Symbol;Acc:MGI:87885]
ENSMUSG00000027108	83.05652172	75.93795498	72.54274764	98.73755584	89.27343784	84.48414209	155.5727998	156.6540732	153.1688239	122.6601166	119.6640437	112.3240503	Ola1	Obg-like ATPase 1 [Source:MGI Symbol;Acc:MGI:1914309]
ENSMUSG00000027109	33.840794	42.94165849	24.98860545	50.10031201	60.20332668	71.67449194	31.77398834	31.07864808	41.26645096	49.98171317	47.22057671	55.34486927	Sp3	trans-acting transcription factor 3 [Source:MGI Symbol;Acc:MGI:1277166]
ENSMUSG00000027111	14.36710829	18.99782529	11.39057337	27.00121722	33.71013835	41.74769451	79.67465078	69.66611249	98.67378763	77.69259399	79.57621509	73.82904738	Itga6	integrin alpha 6 [Source:MGI Symbol;Acc:MGI:96605]
ENSMUSG00000027115	3.038356358	4.665098044	2.363481752	5.045244879	6.196778839	7.226393612	11.63482081	10.40194622	14.29237633	14.28144685	13.37708849	14.6248731	Kif18a	kinesin family member 18A [Source:MGI Symbol;Acc:MGI:2446977]
ENSMUSG00000027120	0	0	0	0	0	0	0	0	0	0	0	0	Fshb	follicle stimulating hormone beta [Source:MGI Symbol;Acc:MGI:95582]
ENSMUSG00000027122	19.87464139	21.53439299	16.28708323	28.94689442	35.31463385	39.22081563	15.35011677	14.70613695	17.6735957	26.18053737	28.71620758	27.84369663	Arl14ep	ADP-ribosylation factor-like 14 effector protein [Source:MGI Symbol;Acc:MGI:1926020]
ENSMUSG00000027130	7.900061045	8.18464964	6.674583363	8.56512094	9.173286225	10.31125605	6.037272075	5.597333536	6.936617747	9.501931092	9.597986239	9.68616563	Slc12a6	solute carrier family 12, member 6 [Source:MGI Symbol;Acc:MGI:2135960]
ENSMUSG00000027131	59.9434872	52.07995407	64.35186193	61.51779858	59.65135592	47.2148302	56.34482753	55.838848	48.23021335	47.31002992	50.32228467	49.63617998	Emc4	ER membrane protein complex subunit 4 [Source:MGI Symbol;Acc:MGI:1915282]
ENSMUSG00000027132	12.9644275	11.71170369	11.34679172	16.04400868	15.40214136	17.91930219	22.30057113	21.95877003	23.89079255	24.20909746	20.06365232	22.30197153	Katnbl1	katanin p80 subunit B like 1 [Source:MGI Symbol;Acc:MGI:1919675]
ENSMUSG00000027133	47.18001912	38.87112587	65.42295408	45.98279247	39.5419917	29.02081899	154.733231	155.8207273	105.8828785	93.84261593	84.4231853	87.92230594	Nop10	NOP10 ribonucleoprotein [Source:MGI Symbol;Acc:MGI:1913431]
ENSMUSG00000027134	66.39545222	53.77298049	73.89784326	64.00563163	58.99328009	46.68425962	28.65923647	32.81216396	29.0690678	23.50539247	26.50071103	23.02047937	Lpcat4	lysophosphatidylcholine acyltransferase 4 [Source:MGI Symbol;Acc:MGI:2138993]
ENSMUSG00000027157	0	0	0	0	0	0	0	0	0	0	0	0	A26c3	ANKRD26-like family C, member 3 [Source:MGI Symbol;Acc:MGI:1914825]
ENSMUSG00000027160	22.50400332	20.51565901	23.59941975	27.36853271	23.72615739	23.31081976	24.83478459	27.35072552	24.04888518	18.12568876	18.51550343	19.38961255	Ccdc34	coiled-coil domain containing 34 [Source:MGI Symbol;Acc:MGI:1915451]
ENSMUSG00000027162	24.67174252	27.79315412	21.62758243	27.86686882	33.37453659	40.37780427	33.61106653	29.99406252	37.23105682	51.20895888	48.02946511	50.47588349	Lin7c	lin-7 homolog C (C. elegans) [Source:MGI Symbol;Acc:MGI:1330839]
ENSMUSG00000027163	85.08584659	74.49632036	99.49996095	62.25183282	56.35342461	45.69017113	47.28495058	54.39235774	37.96325821	37.28521742	37.99324812	39.52316684	Commd9	COMM domain containing 9 [Source:MGI Symbol;Acc:MGI:1923751]
ENSMUSG00000027164	7.52998537	9.190095972	7.709143785	10.10619628	10.88970641	13.77394651	6.579841559	6.393877155	8.510444699	14.95309504	14.67765877	13.95200533	Traf6	TNF receptor-associated factor 6 [Source:MGI Symbol;Acc:MGI:108072]
ENSMUSG00000027165	21.28633879	17.05889044	20.39283192	15.82319254	17.38413089	12.04723004	14.9752821	14.0430494	13.06688418	11.33437993	13.16573707	11.79534764	B230118H07	RIKEN cDNA B230118H07 gene [Source:MGI Symbol;Acc:MGI:1915420]
ENSMUSG00000027166	19.94782794	19.58420677	20.16061376	26.37122913	18.60728705	20.98900876	22.54660826	26.53823207	20.41138035	24.07642473	21.92652704	23.34436364	Dnajc24	DnaJ heat shock protein family (Hsp40) member C24 [Source:MGI Symbol;Acc:MGI:1919522]
ENSMUSG00000027167	14.02172116	12.61335099	9.862444074	13.45108213	12.59535238	14.620583	20.25857976	20.95947569	23.64427519	17.04193536	16.15620254	18.90835572	Elp4	elongator acetyltransferase complex subunit 4 [Source:MGI Symbol;Acc:MGI:1925016]
ENSMUSG00000027168	0	0.169496633	0	0.297602743	0.077323235	0.07474121	0.350420562	0.291624956	0.342495595	0.570517175	0.408402229	0.543513465	Pax6	paired box 6 [Source:MGI Symbol;Acc:MGI:97490]
ENSMUSG00000027170	133.3128237	112.3122354	128.3311604	135.9582671	124.283219	109.76422	214.7188159	219.8596355	212.1199797	163.2700047	166.5798893	166.1122848	Eif3m	eukaryotic translation initiation factor 3, subunit M [Source:MGI Symbol;Acc:MGI:1351744]
ENSMUSG00000027171	1.685994832	3.199389254	1.555929367	3.456921933	5.738362704	15.22942902	2.607630823	1.387927712	4.066801258	12.80808152	14.50860188	13.5948435	Prrg4	proline rich Gla (G-carboxyglutamic acid) 4 (transmembrane) [Source:MGI Symbol;Acc:MGI:2442211]
ENSMUSG00000027173	5.068800922	5.318141729	2.959880685	6.341866918	6.193460716	6.03430921	26.2467982	29.86784965	27.39837242	20.0500139	19.90868209	20.39744378	Depdc7	DEP domain containing 7 [Source:MGI Symbol;Acc:MGI:2139258]
ENSMUSG00000027175	5.364233432	7.285917055	5.257167845	6.524672744	7.105196544	10.70350274	4.911862002	5.268618363	4.011316189	6.254038222	5.457446131	5.424339665	Tcp11l1	t-complex 11 like 1 [Source:MGI Symbol;Acc:MGI:2444263]
ENSMUSG00000027176	35.06149438	37.58795287	32.2820396	39.9338885	38.3211136	45.37153482	64.49493859	58.14432038	64.23508443	86.26716838	85.06507926	80.61534382	Cstf3	cleavage stimulation factor, 3' pre-RNA, subunit 3 [Source:MGI Symbol;Acc:MGI:1351825]
ENSMUSG00000027177	14.47127252	17.46558797	12.0979314	14.78287244	16.11530353	23.01518912	3.77459387	2.756895622	4.134427334	11.11555447	9.961011638	10.70946502	Hipk3	homeodomain interacting protein kinase 3 [Source:MGI Symbol;Acc:MGI:1314882]
ENSMUSG00000027180	11.23170087	14.39121961	9.509177958	12.08400357	14.83991542	15.38864204	13.6187114	12.6937267	16.73272061	14.86759415	14.45654874	15.74183986	Fbxo3	F-box protein 3 [Source:MGI Symbol;Acc:MGI:1929084]
ENSMUSG00000027184	220.2633912	290.2494123	192.6726502	263.6783896	304.8444805	364.7001687	402.3802639	358.3293322	450.8581187	581.2018086	583.62273	565.2691176	Caprin1	cell cycle associated protein 1 [Source:MGI Symbol;Acc:MGI:1858234]
ENSMUSG00000027185	21.62334251	22.56362324	20.77514842	22.21472836	22.03103141	24.21488239	70.36229863	68.65095958	73.11204132	61.38167051	62.71585752	62.6911141	Nat10	N-acetyltransferase 10 [Source:MGI Symbol;Acc:MGI:2138939]
ENSMUSG00000027186	0	0.02931924	0.130332101	0	0.053500968	0	0.090922732	0.025222395	0.142186337	0.091095778	0.105967087	0.270766187	Elf5	E74-like factor 5 [Source:MGI Symbol;Acc:MGI:1335079]
ENSMUSG00000027187	52.89763981	64.18648928	45.24706359	46.47480955	52.56533242	57.45115764	57.66758164	50.66846047	65.90635725	49.56233405	47.43201788	48.31816756	Cat	catalase [Source:MGI Symbol;Acc:MGI:88271]
ENSMUSG00000027188	6.115159769	6.017641432	6.078527547	4.274737175	3.621338162	3.235058368	0.099263189	0.110144309	0.062091708	0.656383923	0.518280369	0.63062168	Pamr1	peptidase domain containing associated with muscle regeneration 1 [Source:MGI Symbol;Acc:MGI:2445082]
ENSMUSG00000027189	49.52977028	55.9303691	46.03879983	55.01269139	61.45472936	77.70688296	18.60914828	16.09120796	23.47837288	45.39468651	43.74287861	44.30224492	Trim44	tripartite motif-containing 44 [Source:MGI Symbol;Acc:MGI:1931835]
ENSMUSG00000027193	81.30948373	87.65837344	72.31397368	91.1773536	99.85734042	117.4079017	127.1742559	116.9801924	137.1327782	150.5339352	149.609672	151.5875318	Api5	apoptosis inhibitor 5 [Source:MGI Symbol;Acc:MGI:1888993]
ENSMUSG00000027194	12.46942789	13.88742747	11.04910649	13.619448	13.71987049	15.49476905	2.628805842	2.409673033	2.645317357	4.809564378	5.722601051	5.752270954	Ttc17	tetratricopeptide repeat domain 17 [Source:MGI Symbol;Acc:MGI:1921819]
ENSMUSG00000027195	93.77312578	96.46052924	81.10167597	104.8644077	103.1613466	106.9197885	76.10773751	74.83723613	83.45402678	92.79909824	92.89388535	93.25690003	Hsd17b12	hydroxysteroid (17-beta) dehydrogenase 12 [Source:MGI Symbol;Acc:MGI:1926967]

ENSMUSG00000027196	0.416404721	0.831153165	0.511574518	0.699447678	1.079166309	0.993792884	0.594810644	0.247504922	0.981850166	1.018068788	0.57769154	0.36902811	Alkbh3os1	alkB homolog 3, opposite strand 1 [Source:MGI Symbol;Acc:MGI:1918102]
ENSMUSG00000027198	145.7867114	145.7462111	141.7630634	132.7870128	128.4145952	128.4824874	40.69209313	43.91936319	43.12985549	54.26884743	56.11394747	57.69721076	Ext2	exostosin glycosyltransferase 2 [Source:MGI Symbol;Acc:MGI:108050]
ENSMUSG00000027199	1.990766076	5.429532962	1.394511167	1.401460671	0.293560509	0.399034342	0.779522201	1.522351704	0.747670848	5.185878586	4.477109435	5.076133416	Gatm	glycine amidinotransferase (L-arginine:glycine amidinotransferase) [Source:MGI Symbol;Acc:MGI:1914342]
ENSMUSG00000027200	2.096382388	1.884976011	1.068230316	1.750852426	2.484194579	3.332086192	0.162376469	0.227590733	0.151465089	3.150961429	3.378499489	2.850424021	Sema6d	sema domain, transmembrane domain (TM), and cytoplasmic domain, (semaphorin) 6D [Source:MGI Symbol;Acc:MGI:2387661]
ENSMUSG00000027201	11.63663507	18.84335877	8.681156223	18.87297637	26.04454063	33.52981566	32.90630896	26.30828001	40.43536976	57.25589295	59.58966956	58.95011303	Myef2	myelin basic protein expression factor 2, repressor [Source:MGI Symbol;Acc:MGI:104592]
ENSMUSG00000027202	0.616857094	1.819474165	0.432102052	0.74732328	0.500293261	0.486334813	0.299512192	0.493156186	0.120872691	0.212961585	0.081074416	0.182224645	Slc12a1	solute carrier family 12, member 1 [Source:MGI Symbol;Acc:MGI:103150]
ENSMUSG00000027203	78.79867529	59.57024712	73.65306324	91.98252425	80.26267144	67.99158428	328.6206389	348.1177105	294.7886288	121.7769299	122.8388646	125.4156763	Dut	deoxyuridine triphosphatase [Source:MGI Symbol;Acc:MGI:1346051]
ENSMUSG00000027204	15.80338776	22.6368981	13.22958923	9.725106382	12.34875354	18.38078049	1.55794923	1.270966527	1.62928955	10.42554642	11.52113266	11.25920382	Fbn1	fibrillin 1 [Source:MGI Symbol;Acc:MGI:95489]
ENSMUSG00000027206	104.0342672	111.8385853	91.58947445	112.916146	122.2971628	134.7218858	110.8522581	107.5130312	119.6276572	140.9163861	129.6092744	141.9731878	Cops2	COP9 signalosome subunit 2 [Source:MGI Symbol;Acc:MGI:1330276]
ENSMUSG00000027207	53.44118965	48.84851726	48.48043618	39.16683244	40.52927423	34.9459401	25.48595307	26.59751331	26.36146683	32.84230042	33.33663019	28.96169976	Galk2	galactokinase 2 [Source:MGI Symbol;Acc:MGI:1917226]
ENSMUSG00000027208	68.1747182	80.37407232	53.14925592	75.95371337	77.90674582	83.66935057	3.411127982	2.772770682	3.080703963	21.93635254	22.13360865	21.49774493	Fgf7	fibroblast growth factor 7 [Source:MGI Symbol;Acc:MGI:95521]
ENSMUSG00000027209	0.023971302	0.110416748	0	0.134217753	0.125928463	0.200843581	0.107005165	0	0	0	0	0	Fam227b	family with sequence similarity 227, member B [Source:MGI Symbol;Acc:MGI:1923073]
ENSMUSG00000027210	12.39595039	15.69351784	10.16784469	14.39637865	18.35778792	19.14633508	3.454256638	3.832908413	5.436225035	4.210310182	4.000166505	3.756506075	Meis2	Meis homeobox 2 [Source:MGI Symbol;Acc:MGI:108564]
ENSMUSG00000027215	53.72893369	47.40880947	49.90112641	53.05844173	51.06777373	43.34182919	2.793071582	3.233994768	2.700891146	8.881838393	10.34122643	12.61720977	Cd82	CD82 antigen [Source:MGI Symbol;Acc:MGI:104651]
ENSMUSG00000027217	0.613182382	0.608716408	0.303062164	0.572211723	0.622030916	0.273787887	2.170863989	1.612869913	1.672814683	0.81325924	0.633615488	0.580894371	Tspan18	tetraspanin 18 [Source:MGI Symbol;Acc:MGI:1917186]
ENSMUSG00000027219	0.187324718	0.30199966	0.095890836	0	0	0.02853637	0.016723924	0	0.034870833	0.167557538	0.265079183	0.13280942	Slc28a2	solute carrier family 28 (sodium-coupled nucleoside transporter), member 2 [Source:MGI Symbol;Acc:MGI:1913105]
ENSMUSG00000027220	3.626703175	3.596654551	3.642714653	1.345210903	1.069805991	1.167699751	0.980544002	0.884024494	1.043556727	8.698423668	9.189918861	8.253173203	Syt13	synaptotagmin XIII [Source:MGI Symbol;Acc:MGI:1933945]
ENSMUSG00000027221	1.075712196	0.795010885	1.002681814	0.524392434	0.657882245	0.39133159	0.444350924	0.477155012	0.418423971	1.335589878	1.470095635	1.408640882	Chst1	carbohydrate sulfotransferase 1 [Source:MGI Symbol;Acc:MGI:1924219]
ENSMUSG00000027222	18.35104434	16.53372784	18.0067592	17.41798413	14.33089549	11.55121895	14.14019133	17.46815239	13.69772857	10.07349276	10.71891894	11.05416841	Pex16	peroxisomal biogenesis factor 16 [Source:MGI Symbol;Acc:MGI:1338829]
ENSMUSG00000027223	17.43353996	20.48343238	18.75475523	20.50486242	21.19192776	20.72132244	18.29484072	17.54150111	16.5410637	7.458573356	8.455087328	7.789212855	Mapk8ip1	mitogen-activated protein kinase 8 interacting protein 1 [Source:MGI Symbol;Acc:MGI:1309464]
ENSMUSG00000027224	0.388226958	0.223531649	0.960537786	0.392477603	0.611841902	0.517484536	0.375483536	0.416643624	0.090336432	0.289383206	0.430879705	0.229370855	Duoxa1	dual oxidase maturation factor 1 [Source:MGI Symbol;Acc:MGI:2384861]
ENSMUSG00000027225	0	0	0	0	0	0	0	0	0	0.024175858	0.134988224	0.071858489	Duoxa2	dual oxidase maturation factor 2 [Source:MGI Symbol;Acc:MGI:1914061]
ENSMUSG00000027227	32.96655402	32.48526917	30.82908326	34.00753735	38.0580231	35.53305809	9.277056595	9.768423431	10.16553718	14.13843663	14.20796974	15.74279356	Sord	sorbitol dehydrogenase [Source:MGI Symbol;Acc:MGI:98266]
ENSMUSG00000027229	0.738398387	0.618402691	0.9277775	1.753974071	0.740541876	1.022590347	2.906584122	2.826206751	2.93652125	2.942137835	3.073210845	3.807341498	Terb2	telomere repeat binding bouquet formation protein 2 [Source:MGI Symbol;Acc:MGI:1921651]
ENSMUSG00000027230	22.36555668	21.31980695	22.08535786	12.37152871	10.99380158	9.367603286	10.25675128	10.79156114	10.24692083	10.80856835	11.61330884	10.2400693	Creb3l1	cAMP responsive element binding protein 3-like 1 [Source:MGI Symbol;Acc:MGI:1347062]
ENSMUSG00000027233	0.318205744	0.061071712	0.180987059	0.263950553	0.315752429	0.430882769	0.88382573	0.823095662	0.773340231	1.707765147	1.294940445	1.598009975	Patl2	protein associated with topoisomerase II homolog 2 (yeast) [Source:MGI Symbol;Acc:MGI:1914828]
ENSMUSG00000027236	8.307763961	9.682278852	6.423064443	10.62650296	12.65652321	12.51391534	18.6191836	15.80732168	21.69106842	27.31569384	24.65689031	25.94662356	Eif3j1	eukaryotic translation initiation factor 3, subunit J1 [Source:MGI Symbol;Acc:MGI:1925905]
ENSMUSG00000027238	3.862906638	4.473048543	3.295679566	5.747654932	6.538859013	8.124034043	4.29173432	5.081086085	4.394420616	5.259702138	5.162631927	5.230198897	Frmd5	FERM domain containing 5 [Source:MGI Symbol;Acc:MGI:2442557]
ENSMUSG00000027239	55.96821424	35.307022	70.44103776	20.27071707	15.77923189	16.82241397	474.7110952	559.8503548	369.0035954	91.2927125	86.2546494	83.40421545	Mdk	midkine [Source:MGI Symbol;Acc:MGI:96949]
ENSMUSG00000027242	3.058897239	3.824404049	2.013220642	4.14583979	5.264611209	6.667971023	21.6522451	20.76458864	26.58645435	23.36117932	23.20700733	23.36511763	Wdr76	WD repeat domain 76 [Source:MGI Symbol;Acc:MGI:1926186]
ENSMUSG00000027243	5.340772494	5.598979224	5.596802279	4.654794269	5.16137124	5.583864172	9.2438554	8.98437071	9.9888399	9.914932789	9.101009343	8.684836021	Harbi1	harbinger transposase derived 1 [Source:MGI Symbol;Acc:MGI:2443194]
ENSMUSG00000027244	26.72053781	29.5063545	26.96436667	28.10267132	27.85969322	29.25557595	26.81748709	26.41993526	29.92997089	44.85697148	43.27341361	43.40275451	Atg13	autophagy related 13 [Source:MGI Symbol;Acc:MGI:1196429]
ENSMUSG00000027245	0.67672589	0.746814456	0.577355835	0.736761061	0.651758194	0.579881099	0.629339025	0.530728117	0.393667938	0.479207969	0.211239764	0.524764743	Hypk	huntingtin interacting protein K [Source:MGI Symbol;Acc:MGI:1914943]
ENSMUSG00000027246	0.030756603	0.177089048	0.157441924	0	0.032314738	0	0.10983518	0.030468797	0.143134923	0.055022111	0.051203539	0.109029034	Ell3	elongation factor RNA polymerase II-like 3 [Source:MGI Symbol;Acc:MGI:2673679]
ENSMUSG00000027247	63.6889919	70.70075444	54.29115189	66.14852981	63.4300385	66.94149453	37.28244341	35.78734719	43.43198802	47.73904219	49.1162255	52.18414508	Arhgap1	Rho GTPase activating protein 1 [Source:MGI Symbol;Acc:MGI:2445003]
ENSMUSG00000027248	1253.627741	1181.993631	1219.527722	1066.479148	1013.807284	924.9525461	515.2305147	551.5571011	531.334897	642.120324	644.1774951	637.4819728	Pdia3	protein disulfide isomerase associated 3 [Source:MGI Symbol;Acc:MGI:95834]
ENSMUSG00000027249	0.18117258	0.312944309	0.31532137	0.219787458	0.323596384	0.110396701	0.663161691	0.664065838	0.455295617	0.388931029	0.497666059	0.610126475	F2	coagulation factor II [Source:MGI Symbol;Acc:MGI:88380]
ENSMUSG00000027253	1.282162011	1.77677556	0.967814588	1.013976713	1.324284073	1.91181787	2.958719821	2.604912915	3.246408495	3.761323986	4.061054821	3.938474247	Lrp4	low density lipoprotein receptor-related protein 4 [Source:MGI Symbol;Acc:MGI:2442252]
ENSMUSG00000027254	13.91832172	18.37063478	13.37768859	14.08536184	16.80070955	20.932899	8.631568787	6.54691216	9.594963097	11.7081607	11.39741904	11.41983661	Map1a	microtubule-associated protein 1 A [Source:MGI Symbol;Acc:MGI:1306776]
ENSMUSG00000027255	19.1047853	25.85705462	12.98124291	24.3025815	26.82336283	26.29210551	11.71668305	12.83176829	15.17350697	21.33804649	20.90977242	20.86860312	Arfgap2	ADP-ribosylation factor GTPase activating protein 2 [Source:MGI Symbol;Acc:MGI:1924288]
ENSMUSG00000027257	124.6984189	96.75168035	140.7766554	136.6706944	114.8132987	99.60467034	25.04284514	26.99783351	27.26311963	23.34645952	20.87780795	20.61768791	Pacsin3	protein kinase C and casein kinase substrate in neurons 3 [Source:MGI Symbol;Acc:MGI:1891410]
ENSMUSG00000027259	4.664156696	5.623176582	3.788717701	5.374231385	4.808416557	6.855024164	7.682970814	7.093461678	7.663349095	12.26130634	11.35568113	10.18818064	Adal	adenosine deaminase-like [Source:MGI Symbol;Acc:MGI:1923144]
ENSMUSG00000027261	0.299502235	0.076642716	0.238488593	0.217381551	0.244747276	0.253472704	0	0	0.061947643	0.059532817	0	0.206442639	Hao1	hydroxyacid oxidase 1, liver [Source:MGI Symbol;Acc:MGI:96011]
ENSMUSG00000027263	3.761459522	6.409639212	1.981126967	7.12171335	9.091920517	13.48057241	6.075719719	3.467792562	8.499567276	12.87470123	13.97202015	12.87154135	Tubgcp4	tubulin, gamma complex associated protein 4 [Source:MGI Symbol;Acc:MGI:1196293]
ENSMUSG00000027270	0	0	0.074349789	0.101654374	0.076300959	0.110629606	0	0	0	0	0	0	Lamp5	lysosomal-associated membrane protein family, member 5 [Source:MGI Symbol;Acc:MGI:1923411]
ENSMUSG00000027272	8.333754014	11.21304023	6.241858081	9.35277236	12.12006904	16.9883685	13.86224092	11.42772471	16.0441555	29.68006785	31.74594621	29.87271891	Ubr1	ubiquitin protein ligase E3 component n-recognin 1 [Source:MGI Symbol;Acc:MGI:1277977]
ENSMUSG00000027273	0	0.018557871	0.01649897	0	0	0	0.100713101	0.175612107	0.104997776	0.115319749	0.201218402	0.071409934	Snap25	synaptosomal-associated protein 25 [Source:MGI Symbol;Acc:MGI:98331]
ENSMUSG00000027274	2.777835653	2.705042881	3.836439467	2.296472736	2.761860003	3.109839913	5.592457092	5.35593407	4.928058506	4.118946141	4.236571493	4.79139617	Mkks	McKusick-Kaufman syndrome [Source:MGI Symbol;Acc:MGI:1891836]
ENSMUSG00000027276	3.478277987	4.075440658	3.274658508	2.678411749	3.782267448	4.835511242	1.93268627	2.072257152	2.76201045	11.12865732	11.44965692	10.61642485	Jag1	jagged 1 [Source:MGI Symbol;Acc:MGI:1095416]
ENSMUSG00000027281	2.543193848	2.471550831	1.775936291	3.237519907	3.830428066	4.400474927	2.887348102	3.131041085	3.762732036	6.27221044	6.50993432	5.875620094	Slx4ip	SLX4 interacting protein [Source:MGI Symbol;Acc:MGI:1921493]
ENSMUSG00000027282	89.64498298	78.72620282	84.91148932	88.11099915	82.44210973	79.44388966	125.840064	130.8031144	128.0853557	91.41817798	93.94176	91.50429534	Mtch2	mitochondrial carrier 2 [Source:MGI Symbol;Acc:MGI:1929260]
ENSMUSG00000027284	7.890347037	7.990146651	7.873770014	8.065075998	7.644931465	8.360122995	9.031533654	9.16995582	10.37305887	7.661892699	6.938481407	9.128497698	Cdan1	congenital dyserythropoietic anemia, type I (human) [Source:MGI Symbol;Acc:MGI:1916218]
ENSMUSG00000027285	9.582542262	14.22651057	8.460938864	13.02701205	15.12862814	17.2366118	15.66167263	14.38161947	19.33244588	23.6279228	23.91543139	21.68752788	Haus2	HAUS augmin-like complex, subunit 2 [Source:MGI Symbol;Acc:MGI:1913546]
ENSMUSG00000027286	13.09018043	15.54282477	12.08711668	16.10230443	15.23395298	15.40903736	26.67232226	25.12877485	27.48642641	19.91626207	21.40149546	19.23297158	Lrrc57	leucine rich repeat containing 57 [Source:MGI Symbol;Acc:MGI:1913856]
ENSMUSG00000027287	26.97690748	34.42270892	23.22337538	25.02491508	29.01680597	32.65200269	43.2202863	40.30888055	48.96553001	53.79144667	58.67246914	55.11241102	Snap23	synaptosomal-associated protein 23 [Source:MGI Symbol;Acc:MGI:109356]
ENSMUSG00000027288	76.54866346	87.39459614	65.49588252	84.53647933	98.60317216	130.1559045	170.9469616	155.5814782	212.0576544	260.1172375	257.0113669	256.7946033	Zfp106	zinc finger protein 106 [Source:MGI Symbol;Acc:MGI:1270153]
ENSMUSG00000027291	17.67276374	19.54065405	13.32975858	18.27977501	20.3345003	24.34598989	20.94403675	19.19228309	28.29460728	19.28769964	22.19637354	21.4313508	Vps39	VPS39 HOPS complex subunit [Source:MGI Symbol;Acc:MGI:2443189]
ENSMUSG00000027293	32.27136587	37.58795287	31.93788139	36.20086986	43.8308774	42.29897038	41.95622343	36.63158787	45.90008225	62.45273918	63.15522497	58.30163631	Ehd4	EH-domain containing 4 [Source:MGI Symbol;Acc:MGI:1919619]
ENSMUSG00000027296	0.923053369	0.735883895	0.920784108	1.104331074	1.168751905	1.261925987	13.88259578	13.22386034	12.00593376	6.986264523	5.555752962	5.768178652	Itpka	inositol 1,4,5-trisphosphate 3-kinase A [Source:MGI Symbol;Acc:MGI:1333822]
ENSMUSG00000027297	0.034770495	0.060060019	0.017798889	0.048670882	0.109595923	0.013242029	1.2261711	1.377805178	1.521058684	0.761983981	0.622272988	0.677918306	Ltk	leukocyte tyrosine kinase [Source:MGI Symbol;Acc:MGI:96840]
ENSMUSG00000027298	22.85378807	23.60778221	22.68201946	24.93559235	24.81929334	26.42150337	47.62859546	42.94894563	48.93932593	35.82068987	36.74768762	38.64905121	Tyro3	TYRO3 protein tyrosine kinase 3 [Source:MGI Symbol;Acc:MGI:104294]
ENSMUSG00000027300	3.737245518	4.375838891	3.980238287	4.82169422	4.453632061	4.833475501	7.591160321	8.522684792	7.622295524	6.394095258	5.511894565	5.923881725	Ubox5	U box domain containing 5 [Source:MGI Symbol;Acc:MGI:2154658]
ENSMUSG00000027301	0.560892765	0	0.656271884	0.149547403	0.33674725	0.061031696	0.286144164	0	0.149158851	0.215016572	0.066698092	0	Oxt	oxytocin [Source:MGI Symbol;Acc:MGI:97453]
ENSMUSG00000027303	29.3106901	41.76478668	21.26834557	30.55256026	37.71680229	43.67363759	10.85924487	9.45883203	13.27317063	23.87842868	23.94637432	21.18737652	Ptpra	protein tyrosine phosphatase, receptor type, A [Source:MGI Symbol;Acc:MGI:97808]
ENSMUSG00000027304	36.34573312	41.7323115	34.83075399	40.6957593	43.68194787	49.73442542	65.58115971	62.64351098	72.77193892	59.39763503	59.93664982	60.09034152	Rtf1	RTF1, Paf1/RNA polymerase II complex component [Source:MGI Symbol;Acc:MGI:1309480]
ENSMUSG00000027305	18.67352821	16.25993814	18.04859183	14.93085632	16.67879706	14.47784407	31.03025106	35.50774498	32.23373458	30.26723569	28.79259505	31.17272403	Ndufaf1	NADH:ubiquinone oxidoreductase complex assembly factor 1 [Source:MGI Symbol;Acc:MGI:1916952]
ENSMUSG00000027306	17.19735647	16.86677367	13.36658815	24.9785637	26.82016059	22.97211126	55.16782469	57.90068926	60.54194738	50.45920985	47.17154563	47.2566013	Nusap1	nucleolar and spindle associated protein 1 [Source:MGI Symbol;Acc:MGI:2675669]

ENSMUSG00000027309	74.60760218	100.4604785	71.7322252	102.4886311	122.1572564	150.1253946	17.44362137	15.28736114	19.14135152	58.16330421	57.9596293	56.82712694	4930402H24	RIKEN cDNA 4930402H24 gene [Source:MGI Symbol;Acc:MGI:1923029]
ENSMUSG00000027312	11.64205614	14.78280154	9.801155025	13.06275	16.18032761	20.82515895	4.360660518	3.130904946	5.690744821	13.59003513	14.19664616	14.01465728	Atrn	attractin [Source:MGI Symbol;Acc:MGI:1341628]
ENSMUSG00000027313	9.699152113	7.809270025	9.660817239	36.30247233	30.82008752	27.20824316	2.182395497	2.551822281	2.422031766	2.703797271	2.84434564	2.515793345	Chac1	ChaC, cation transport regulator 1 [Source:MGI Symbol;Acc:MGI:1916315]
ENSMUSG00000027314	0.232586421	0.424072431	0.138903492	0.307481587	0.30546161	0.295261447	0.069215953	0	0.036080317	0.052010765	0	0.120238889	Dll4	delta like canonical Notch ligand 4 [Source:MGI Symbol;Acc:MGI:1859388]
ENSMUSG00000027315	6.336001153	7.425941805	6.775051568	7.305410234	5.029721282	6.091506453	47.06322436	47.25666623	47.1258995	17.95937174	24.96398716	23.15925102	Spint1	serine protease inhibitor, Kunitz type 1 [Source:MGI Symbol;Acc:MGI:1338033]
ENSMUSG00000027316	14.98696832	13.64836957	17.36590701	16.79072576	16.24250959	13.96657366	1.955247064	2.041956776	2.118368294	1.843734916	2.144723083	2.892316124	Gfra4	glial cell line derived neurotrophic factor family receptor alpha 4 [Source:MGI Symbol;Acc:MGI:1341873]
ENSMUSG00000027317	0.763496615	0.3768024	0.390831117	0.458024462	0.229192995	0.45692551	3.067345762	4.646163374	3.857712948	1.121955927	1.271067824	0.918282876	Ppp1r14d	protein phosphatase 1, regulatory inhibitor subunit 14D [Source:MGI Symbol;Acc:MGI:1919362]
ENSMUSG00000027318	15.64217966	14.01313247	17.24228608	7.111969071	5.696983207	6.432995921	1.583887711	1.410960116	2.209447964	6.660519151	5.636685978	6.798374994	Adam33	a disintegrin and metallopeptidase domain 33 [Source:MGI Symbol;Acc:MGI:1341813]
ENSMUSG00000027322	1.425393378	3.166340067	1.346328655	0.017163273	0.077295693	0.112071881	0.082100564	0.081990303	0.03423736	3.808490795	3.513559702	3.805955651	Siglec1	sialic acid binding Ig-like lectin 1, sialoadhesin [Source:MGI Symbol;Acc:MGI:99668]
ENSMUSG00000027323	20.19259625	20.3344434	16.43240999	29.98160251	34.70074847	31.21730655	83.22812906	80.58147903	84.6130269	67.49409682	70.52227097	70.421501	Rad51	RAD51 recombinase [Source:MGI Symbol;Acc:MGI:97890]
ENSMUSG00000027324	1.319794712	1.611366362	1.302357729	1.706448615	1.921269996	2.507103574	6.587025975	5.592082754	7.74046794	8.092998103	8.510809657	7.623762541	Rpusd2	RNA pseudouridylate synthase domain containing 2 [Source:MGI Symbol;Acc:MGI:1918066]
ENSMUSG00000027326	2.2407586	3.76253491	1.566436977	3.878094556	5.278970874	7.120210494	7.473226945	6.03352788	8.379175542	15.66360458	15.56255154	15.44911899	Knl1	kinetochore scaffold 1 [Source:MGI Symbol;Acc:MGI:1923714]
ENSMUSG00000027327	70.32108665	48.51825712	65.92556769	65.54289197	52.73557056	46.54625528	146.3114809	157.0692782	139.5568055	60.91702903	61.19751568	57.29383896	1700037H04	RIKEN cDNA 1700037H04 gene [Source:MGI Symbol;Acc:MGI:1914576]
ENSMUSG00000027329	4.348868556	3.073659793	2.756210306	2.319024388	1.934045705	1.61241171	1.581788109	1.595619967	1.670499368	1.440726014	1.16835797	1.447826863	Spef1	sperm flagellar 1 [Source:MGI Symbol;Acc:MGI:3513546]
ENSMUSG00000027330	19.53181358	19.2359902	18.19983449	29.62986997	28.5795789	26.28852713	26.31591589	24.14606176	23.95689426	18.72073289	19.55861101	18.92244591	Cdc25b	cell division cycle 25B [Source:MGI Symbol;Acc:MGI:99701]
ENSMUSG00000027331	28.39590443	25.35867466	32.59421542	38.970505	38.20635658	33.04989623	50.63776219	53.38995065	44.19565458	30.58303052	30.66141613	29.81941789	Knstrn	kinetochore-localized astrin/SPAG5 binding [Source:MGI Symbol;Acc:MGI:1289298]
ENSMUSG00000027332	36.18120492	35.13256616	35.88059927	28.28135727	24.94545626	25.213159	50.53253403	53.46292762	51.02307157	44.35901209	47.85701159	47.50455076	Ivd	isovaleryl coenzyme A dehydrogenase [Source:MGI Symbol;Acc:MGI:1929242]
ENSMUSG00000027333	26.36094719	25.82469179	27.34842299	27.34616037	25.04103879	24.31856699	14.13780875	13.84943899	12.2976205	22.94970188	23.11453377	24.58083893	Smox	spermine oxidase [Source:MGI Symbol;Acc:MGI:2445356]
ENSMUSG00000027335	6.231086968	6.131719943	4.964287279	2.833368104	2.237799855	3.486230737	0.040457982	0.112232353	0.021089601	0.729629694	0.509244675	0.502012963	Adra1d	adrenergic receptor, alpha 1d [Source:MGI Symbol;Acc:MGI:106673]
ENSMUSG00000027338	0	0	0	0	0	0.029132463	0	0.037889642	0	0	0	0	Prnd	prion like protein doppel [Source:MGI Symbol;Acc:MGI:1346999]
ENSMUSG00000027339	3.240738226	4.480975987	3.039338867	4.172076587	4.336916741	4.657550486	3.780241275	3.011226734	4.513510754	3.427735323	2.884646651	2.45274868	Rassf2	Ras association (RalGDS/AF-6) domain family member 2 [Source:MGI Symbol;Acc:MGI:2442060]
ENSMUSG00000027340	21.35762136	27.77527448	15.83082188	27.87623974	31.68471815	47.0028476	27.96581038	23.04786074	34.40357292	59.14563727	59.1416971	58.12050265	Slc23a2	solute carrier family 23 (nucleobase transporters), member 2 [Source:MGI Symbol;Acc:MGI:1859682]
ENSMUSG00000027341	22.72566606	22.99125778	18.58797728	23.21059909	22.55582526	22.42555938	44.84927368	42.26126454	43.4745957	28.61222304	29.12833565	30.95750423	Tmem230	transmembrane protein 230 [Source:MGI Symbol;Acc:MGI:1917862]
ENSMUSG00000027342	120.8236118	121.4815538	102.9678567	161.8171787	160.4619335	151.1387966	545.823487	549.168524	549.5312545	372.0218636	354.6705472	357.9245005	Pcna	proliferating cell nuclear antigen [Source:MGI Symbol;Acc:MGI:97503]
ENSMUSG00000027344	0.666638544	0.977032702	0.682499651	0.706927427	0.923268473	0.392365036	0.297579855	0.630382255	0.620479812	0.704709243	1.135041829	0.993603855	Fsip1	fibrous sheath-interacting protein 1 [Source:MGI Symbol;Acc:MGI:1918563]
ENSMUSG00000027345	0.160106001	0.230462863	0	0	0.04205425	0.060974922	0.035734748	0	0	0	0	0	4921508D12	RIKEN cDNA 4921508D12 gene [Source:MGI Symbol;Acc:MGI:1918095]
ENSMUSG00000027346	6.778219236	8.636699532	6.615047391	9.282616603	11.69053077	14.45597146	9.462251289	9.086770316	12.30642913	10.56686802	9.789555164	10.56300183	Gpcpd1	glycerophosphocholine phosphodiesterase 1 [Source:MGI Symbol;Acc:MGI:104898]
ENSMUSG00000027347	0.045937887	0.105799677	0.172446531	0.014289494	0.064353478	0.163286936	0.095695366	0.166862749	0.199533139	4.232306622	4.588638851	3.786149611	Rasgrp1	RAS guanyl releasing protein 1 [Source:MGI Symbol;Acc:MGI:1314635]
ENSMUSG00000027349	28.39764993	30.31342522	26.71441679	33.48810802	34.0424084	34.59479956	42.99172424	38.17495597	39.96873154	41.50208422	44.25565739	43.28875927	Fam98b	family with sequence similarity 98, member B [Source:MGI Symbol;Acc:MGI:1915465]
ENSMUSG00000027350	0.047111483	0.090418824	0.032154927	0.087927324	0.032998773	0.08372925	0.056080079	0.093341272	0.043849436	0.112373624	0.052287409	0.055668474	Chgb	chromogranin B [Source:MGI Symbol;Acc:MGI:88395]
ENSMUSG00000027351	20.85635126	27.20704358	18.5874972	27.60551601	32.6450625	38.16309646	23.49406311	23.03945363	28.12464557	28.48398584	30.35979788	28.04732421	Spred1	sprouty protein with EVH-1 domain 1, related sequence [Source:MGI Symbol;Acc:MGI:2150016]
ENSMUSG00000027353	4.361114688	4.818725976	3.850278741	6.920172627	6.67829077	7.827033242	12.29527541	12.08636059	14.08375989	9.997079488	11.66951134	11.23488944	Mcm8	minichromosome maintenance 8 homologous recombination repair factor [Source:MGI Symbol;Acc:MGI:1913884]
ENSMUSG00000027355	0	0	0.053526428	0.048789159	0	0.199113127	0.046676615	0	0.097324791	0.046765451	0	0	Tmco5	transmembrane and coiled-coil domains 5 [Source:MGI Symbol;Acc:MGI:1914606]
ENSMUSG00000027356	0.047301379	0.090783283	0.016142268	0.029427246	0.016565892	0.012009535	0.112612251	0.156195851	0.102727761	1.142369101	1.076212455	1.187723305	Fermt1	fermitin family member 1 [Source:MGI Symbol;Acc:MGI:2443583]
ENSMUSG00000027357	28.91029174	26.07460807	25.9070007	29.83997687	28.49858753	23.45767804	24.96815104	26.2698059	26.43808515	20.43448802	20.53089495	22.27654416	Crls1	cardiolipin synthase 1 [Source:MGI Symbol;Acc:MGI:1913836]
ENSMUSG00000027358	0.786737471	1.184728222	0.805456051	0.508271871	0.165318745	0.405641887	0.237729055	0.059951966	0.202780514	1.916278494	2.045239303	2.51001398	Bmp2	bone morphogenetic protein 2 [Source:MGI Symbol;Acc:MGI:88177]
ENSMUSG00000027359	0.74187048	4.476542446	0.334189484	0.636917235	0.280603355	0.926713687	7.285575772	6.67314995	7.650768946	8.653156577	7.657400964	7.389893943	Slc27a2	solute carrier family 27 (fatty acid transporter), member 2 [Source:MGI Symbol;Acc:MGI:1347099]
ENSMUSG00000027360	1.153922498	0.797280645	0.997607573	0.670022276	0.754370032	0.742200706	0.137359431	0.304833237	0.334140716	1.123903666	0.619004403	0.590855496	Hdc	histidine decarboxylase [Source:MGI Symbol;Acc:MGI:96062]
ENSMUSG00000027361	19.01206809	19.42361594	14.7069558	20.93151181	22.06428829	22.95542775	31.09488967	29.67874443	30.60869732	27.25731367	28.65721968	28.8273672	Gabpb1	GA repeat binding protein, beta 1 [Source:MGI Symbol;Acc:MGI:95611]
ENSMUSG00000027363	22.38666543	30.18969468	20.05439087	28.4996503	33.19427404	42.05205249	24.11092983	19.5246168	28.0578983	36.15179295	36.36495315	35.71130477	Usp8	ubiquitin specific peptidase 8 [Source:MGI Symbol;Acc:MGI:1934029]
ENSMUSG00000027364	0	0.076465302	0.033990934	0.247860974	0.244180732	0.202308769	1.185643644	0.822257865	1.143378554	1.544270256	2.376737107	1.971377017	Usp50	ubiquitin specific peptidase 50 [Source:MGI Symbol;Acc:MGI:1922333]
ENSMUSG00000027365	28.84208203	35.89430096	23.50438853	33.93844708	41.79493861	58.60555788	41.46032698	38.79953419	53.11888302	69.17824493	68.13319224	66.30070887	Trpm7	transient receptor potential cation channel, subfamily M, member 7 [Source:MGI Symbol;Acc:MGI:1929996]
ENSMUSG00000027366	23.99037463	27.89699422	19.99942728	27.71602038	30.82073328	36.44654223	7.815958769	7.13235411	9.911952169	16.38631304	15.77117201	16.24672496	Sppl2a	signal peptide peptidase like 2A [Source:MGI Symbol;Acc:MGI:1913802]
ENSMUSG00000027367	36.59575968	43.02797709	35.2607067	45.09367683	48.70080646	45.9258272	58.72141856	55.21846222	66.66264349	50.38124353	49.49799959	50.18923078	Stard7	START domain containing 7 [Source:MGI Symbol;Acc:MGI:2139090]
ENSMUSG00000027368	0.797495141	0.91835665	0.593796127	0.608898569	0.533206788	0.552216017	0.647259546	0.574569135	0.742275765	1.394256575	1.659585637	1.606272312	Dusp2	dual specificity phosphatase 2 [Source:MGI Symbol;Acc:MGI:101911]
ENSMUSG00000027371	8.678524343	8.106831893	7.145287409	5.833297908	5.866241591	5.084826897	10.9447116	9.919978661	9.998166177	7.92558969	8.486932413	8.282746576	Fahd2a	fumarylacetoacetate hydrolase domain containing 2A [Source:MGI Symbol;Acc:MGI:1915376]
ENSMUSG00000027374	36.40872047	34.1063642	34.8795668	36.31919348	32.82699509	33.99109302	116.36542	115.3272272	107.9574277	83.84149173	82.63057678	82.63815154	Mrps5	mitochondrial ribosomal protein S5 [Source:MGI Symbol;Acc:MGI:1924971]
ENSMUSG00000027375	0.21092396	0.425057119	0.188949603	0.344453854	0.110804702	0.02008212	0.40015473	0.861919715	0.171779449	0.141499876	0.065839845	0.070097251	Mal	myelin and lymphocyte protein, T cell differentiation protein [Source:MGI Symbol;Acc:MGI:892970]
ENSMUSG00000027376	0	0.039862844	0	0.016151841	0.072740657	0	0.278144559	0.051439076	0.096659255	0.154818851	0.201704045	0.306781212	Prom2	prominin 2 [Source:MGI Symbol;Acc:MGI:2138997]
ENSMUSG00000027377	0	0.024700656	0	0	0	0.016337996	0.478749428	0.467481879	0.359364271	0.153491391	0.410662082	0.266131937	Mall	mal, T cell differentiation protein-like [Source:MGI Symbol;Acc:MGI:2385152]
ENSMUSG00000027378	26.55968595	26.3436534	23.2370255	29.00439109	25.79737025	25.24762759	9.409901624	10.76769848	11.25946918	8.329685137	8.748589626	8.33245099	Nphp1	nephronophthisis 1 (juvenile) homolog (human) [Source:MGI Symbol;Acc:MGI:1858233]
ENSMUSG00000027379	26.27587787	30.09792871	22.38195911	37.36414093	45.09876669	49.29339776	140.4407347	130.700903	158.3526629	131.9720656	127.7603571	131.1550147	Bub1	BUB1, mitotic checkpoint serine/threonine kinase [Source:MGI Symbol;Acc:MGI:1100510]
ENSMUSG00000027380	0.208749486	0.210337544	0.187001669	0.19480159	0.219324771	0.11925053	0.559100362	0.129247567	0.291443189	0.676865388	0.434407222	0.369997862	Acoxl	acyl-Coenzyme A oxidase-like [Source:MGI Symbol;Acc:MGI:1921371]
ENSMUSG00000027381	16.77038853	21.71140036	18.03926183	23.38982559	27.18450361	29.7231245	13.83440443	12.16449909	14.78071648	24.35580759	23.94527359	23.16300558	Bcl2l11	BCL2-like 11 (apoptosis facilitator) [Source:MGI Symbol;Acc:MGI:1197519]
ENSMUSG00000027384	18.28037009	15.51654978	20.46268407	17.43616574	17.22446367	15.25805136	24.38019966	26.02935376	21.82218903	14.7443462	16.07646812	14.568521	Ndufaf5	NADH:ubiquinone oxidoreductase complex assembly factor 5 [Source:MGI Symbol;Acc:MGI:1916737]
ENSMUSG00000027386	0.196413052	0.27464681	0.186722939	0.248749944	0.250584093	0	0.050100886	0.166778667	0.195871299	0.163137777	0.186850345	0.062166457	Fbln7	fibulin 7 [Source:MGI Symbol;Acc:MGI:1917620]
ENSMUSG00000027387	7.895621535	7.749739513	7.323957853	8.381791248	11.16289141	10.47396349	14.40557028	15.48599147	14.89512778	11.37575361	9.704079563	10.96555818	Zc3h8	zinc finger CCCH type containing 8 [Source:MGI Symbol;Acc:MGI:1930128]
ENSMUSG00000027394	14.81339454	16.66823163	13.75910177	18.95258206	20.25079527	21.31888394	8.755091413	7.850167052	8.601983114	10.08499206	11.42191698	10.87605156	Ttl	tubulin tyrosine ligase [Source:MGI Symbol;Acc:MGI:1916987]
ENSMUSG00000027395	19.79506917	17.44980088	16.92189748	17.46401262	18.39813412	19.60455116	60.47269836	60.55692633	68.66875067	72.80200945	76.04953013	75.0034187	Polr1b	polymerase (RNA) I polypeptide B [Source:MGI Symbol;Acc:MGI:108014]
ENSMUSG00000027397	44.41244504	40.94354712	41.91404156	44.35667815	42.22086807	43.9670195	122.0064751	117.557601	122.2553046	106.6859419	99.72172173	102.2229445	Slc20a1	solute carrier family 20, member 1 [Source:MGI Symbol;Acc:MGI:108392]
ENSMUSG00000027398	38.07595148	38.08199159	34.40219044	1.043592865	1.510674004	1.541352456	4.65922735	5.592004773	3.766999401	34.96307836	32.314978	36.57549645	Il1b	interleukin 1 beta [Source:MGI Symbol;Acc:MGI:96543]
ENSMUSG00000027399	11.24756825	13.88089267	10.28778487	1.505246889	1.007682881	1.477653413	1.498451755	1.27402483	1.257876086	6.512148312	5.806180422	6.278213791	Il1a	interleukin 1 alpha [Source:MGI Symbol;Acc:MGI:96542]
ENSMUSG00000027400	0.037943993	0.131083375	0	0	0	0.028901253	8.367256575	7.931264431	7.487142965	2.884900478	2.874193711	2.992794107	Pdyn	prodynorphin [Source:MGI Symbol;Acc:MGI:97535]
ENSMUSG00000027401	0.176900675	0.035948863	0.09588156	0.08739572	0.163996318	0.103038033	1.198431965	1.257642907	1.685260548	2.178037275	1.810333005	1.899728746	Tgm3	transglutaminase 3, E polypeptide [Source:MGI Symbol;Acc:MGI:98732]
ENSMUSG00000027403	0.134464024	0.077421118	0	0.012547962	0	0.030725644	0	0	0	0	0.022385547	0.011916533	Tgm6	transglutaminase 6 [Source:MGI Symbol;Acc:MGI:3044321]
ENSMUSG00000027404	214.3247032	181.606822	248.0879212	215.3752608	182.1424736	147.9131592	338.3983616	348.4904146	274.9437678	182.3389176	191.1745987	190.215901	Snrpb	small nuclear ribonucleoprotein B [Source:MGI Symbol;Acc:MGI:98342]
ENSMUSG00000027405	166.1333126	134.1811976	176.7432165	172.8996769	164.5127331	139.8704705	429.4436672	452.9791577	413.4925458	342.011537	339.9930269	343.3740009	Nop56	NOP56 ribonucleoprotein [Source:MGI Symbol;Acc:MGI:1914384]

ENSMUSG00000027406	109.6974179	102.5849268	100.8700603	119.5188109	106.2433344	100.9297101	123.770472	127.8775431	119.0405685	90.61111681	89.87215991	93.73540642	Idh3b	isocitrate dehydrogenase 3 (NAD+) beta [Source:MGI Symbol;Acc:MGI:2158650]
ENSMUSG00000027408	175.4804142	145.2279689	192.1384798	37.90952937	33.43810529	30.46841892	12.77969504	12.77934685	13.27072476	20.74959397	20.67535613	18.92856216	Cpxm1	carboxypeptidase X 1 (M14 family) [Source:MGI Symbol;Acc:MGI:1934569]
ENSMUSG00000027409	0	0	0	0	0	0.110910391	0	0	0	0	0.242415401	0.064522681	1700020A23	RIKEN cDNA 1700020A23 gene [Source:MGI Symbol;Acc:MGI:1922906]
ENSMUSG00000027411	78.29431927	75.11725792	81.48170817	79.80065576	78.79665186	71.01567614	95.98124548	94.26472624	99.17117734	67.34460847	73.40548777	67.79594015	Vps16	VSP16 CORVET/HOPS core subunit [Source:MGI Symbol;Acc:MGI:2136772]
ENSMUSG00000027412	5.689083743	5.738985238	6.797799122	5.824114568	5.864514568	5.55966993	5.106464921	4.52690756	4.924076007	4.979020596	5.284457038	5.218473058	Lpin3	lipin 3 [Source:MGI Symbol;Acc:MGI:1891342]
ENSMUSG00000027416	0	0	0	0	0.095275697	0	0	0	0	0.081112679	0	0	Otor	otoraplin [Source:MGI Symbol;Acc:MGI:1888678]
ENSMUSG00000027419	0	0	0.020952319	0	0.021502173	0	0	0	0.028572522	0.036611621	0.008517687	0	Pcsk2	proprotein convertase subtilisin/kexin type 2 [Source:MGI Symbol;Acc:MGI:97512]
ENSMUSG00000027420	1.898874132	1.793053963	2.29398302	2.959236893	2.593592844	2.415384575	0.542486907	0.470276343	0.159065474	0.203819769	0.158062116	0.252424347	Bfsp1	beaded filament structural protein 1, in lens-CP94 [Source:MGI Symbol;Acc:MGI:101770]
ENSMUSG00000027422	406.5743394	398.8708813	421.9780037	354.9241276	354.7686804	327.1466045	120.4101161	118.7066692	126.851621	196.4322718	204.030379	195.4363688	Rrbp1	ribosome binding protein 1 [Source:MGI Symbol;Acc:MGI:1932395]
ENSMUSG00000027423	199.1269767	184.4744357	185.8842278	240.2574957	236.6297402	226.1694077	487.4467926	496.2245186	485.999762	432.1199956	426.9482941	423.130715	Snx5	sorting nexin 5 [Source:MGI Symbol;Acc:MGI:1916428]
ENSMUSG00000027424	5.279674566	4.673735287	4.296062769	5.424410675	6.45058739	7.361710471	17.84095141	16.01450713	15.91081399	13.41387157	13.79994723	14.25030611	Mgme1	mitochondrial genome maintenance exonuclease 1 [Source:MGI Symbol;Acc:MGI:1921778]
ENSMUSG00000027425	32.05376358	32.51006038	28.86449111	33.05510576	31.45099368	30.41037095	45.03693973	44.38786603	46.10759837	39.43577909	42.36912711	38.50182461	Kat14	lysine acetyltransferase 14 [Source:MGI Symbol;Acc:MGI:1917264]
ENSMUSG00000027427	12.30166425	15.20483866	11.41888061	16.30118954	16.74488539	22.3213152	17.74307428	18.44231403	22.74710385	33.96428544	34.42880089	32.68188591	Polr3f	polymerase (RNA) III (DNA directed) polypeptide F [Source:MGI Symbol;Acc:MGI:1924086]
ENSMUSG00000027428	19.50981125	24.89444263	16.02176387	23.72075709	27.58050616	27.5265584	15.66817029	15.91482651	17.32975708	18.69946744	20.02185586	21.52706421	Rbbp9	retinoblastoma binding protein 9, serine hydrolase [Source:MGI Symbol;Acc:MGI:1347074]
ENSMUSG00000027429	38.40435166	47.40571244	32.37056487	45.78561255	51.80185536	53.19774738	74.71193182	73.23435941	88.53852898	93.14463878	95.85325713	96.17049375	Sec23b	SEC23 homolog B, COPII coat complex component [Source:MGI Symbol;Acc:MGI:1350925]
ENSMUSG00000027430	14.45315873	11.05335349	15.81362828	15.75251823	15.47515513	12.22723078	29.59913756	32.78911362	30.80703963	21.85919116	19.51560523	20.6308792	Dtd1	D-tyrosyl-tRNA deacylase 1 [Source:MGI Symbol;Acc:MGI:1913294]
ENSMUSG00000027431	0	0	0	0	0	0	0	0	0	0	0	0	Scp2d1	SCP2 sterol-binding domain containing 1 [Source:MGI Symbol;Acc:MGI:1913578]
ENSMUSG00000027433	91.70403163	114.8176377	77.71214185	113.056934	128.5634303	146.0317473	138.3182185	122.9697362	158.100636	158.8480802	156.359983	146.4217572	Xrn2	5'-3' exoribonuclease 2 [Source:MGI Symbol;Acc:MGI:894687]
ENSMUSG00000027434	0	0	0	0	0	0	0	0	0	0.237335867	0	0	Nkx2-2	NK2 homeobox 2 [Source:MGI Symbol;Acc:MGI:97347]
ENSMUSG00000027435	0.650872738	0.645620467	0.468430397	0.372849427	0.270830123	0.441764544	0.074793036	0.044687874	0.065978833	1.683164023	1.63608612	1.661925305	Cd93	CD93 antigen [Source:MGI Symbol;Acc:MGI:106664]
ENSMUSG00000027438	2.185851553	2.290579589	1.409851035	2.243780829	2.618109368	2.747123904	1.668514109	1.656529006	2.268468577	2.443990753	2.51091126	2.15024253	Napb	N-ethylmaleimide sensitive fusion protein attachment protein beta [Source:MGI Symbol;Acc:MGI:104562]
ENSMUSG00000027439	21.00172884	25.31248416	20.17215268	23.72222407	27.49833657	31.2125268	19.01423428	16.42753686	19.69595647	24.83686131	23.60614609	24.44624012	Gzf1	GDNF-inducible zinc finger protein 1 [Source:MGI Symbol;Acc:MGI:1921783]
ENSMUSG00000027442	0	0	0.063567461	0.115883053	0.065235669	0	0	0	0	0	0	0	Cst8	cystatin 8 (cystatin-related epididymal spermatogenic) [Source:MGI Symbol;Acc:MGI:107161]
ENSMUSG00000027443	0	0	0	0	0	0	0	0	0	0	0	0	Cst12	cystatin 12 [Source:MGI Symbol;Acc:MGI:1916612]
ENSMUSG00000027444	0	0	0	0	0	0	0	0	0	0	0	0	Cstdc1	cystatin domain containing 1 [Source:MGI Symbol;Acc:MGI:1925859]
ENSMUSG00000027445	0	0	0	0	0	0	0.111833636	0	0	0	0	0	Cst9	cystatin 9 [Source:MGI Symbol;Acc:MGI:1340053]
ENSMUSG00000027446	0	0.537607764	0.079660489	0	0	0.05926586	0	0	0	0	0.06476831	0	Cstdc2	cystatin domain containing 2 [Source:MGI Symbol;Acc:MGI:1924955]
ENSMUSG00000027447	997.5610567	772.494858	1178.795755	714.5383947	628.7632831	465.3022478	101.1282604	111.1225036	77.74601854	151.9810961	153.8399531	158.7339587	Cst3	cystatin C [Source:MGI Symbol;Acc:MGI:102519]
ENSMUSG00000027452	1.72260556	3.334251903	1.463405245	1.949530014	1.27076193	1.116661691	14.49555396	14.77744465	17.0908219	11.83488574	10.67794414	11.69322461	Acss1	acyl-CoA synthetase short-chain family member 1 [Source:MGI Symbol;Acc:MGI:1915988]
ENSMUSG00000027454	15.56486406	14.66487906	16.06688946	20.10674899	18.31308561	18.27310867	83.43315839	80.85530752	72.1363641	49.13112598	48.82659258	50.15987463	Gins1	GINS complex subunit 1 (Psf1 homolog) [Source:MGI Symbol;Acc:MGI:1916520]
ENSMUSG00000027455	132.2897126	121.8132193	149.19824	124.188416	115.950155	100.6992878	180.0742724	191.4040724	161.2036447	149.4107321	145.4151289	145.7458794	Nsfl1c	NSFL1 (p97) cofactor (p47) [Source:MGI Symbol;Acc:MGI:3042273]
ENSMUSG00000027456	2.860364094	1.947979528	2.330140468	2.238721423	1.874254799	1.522738629	2.773338285	2.711722977	2.919952434	4.539324125	3.456238878	3.652472644	Sdcbp2	syndecan binding protein (syntenin) 2 [Source:MGI Symbol;Acc:MGI:2385156]
ENSMUSG00000027457	0.919848908	1.035714341	1.004516869	0.362430441	0.451009352	0.716201876	0.182493369	0.020249809	0.247334427	1.060476033	1.684499132	0.996346678	Snph	syntaphilin [Source:MGI Symbol;Acc:MGI:2139270]
ENSMUSG00000027459	18.66071989	15.10022253	20.27327492	14.46615118	14.50235999	10.57422134	11.65953621	12.99023366	10.37670798	8.096481513	8.15325543	9.480296477	Fam110a	family with sequence similarity 110, member A [Source:MGI Symbol;Acc:MGI:1921097]
ENSMUSG00000027460	8.937394688	8.445699023	9.03684276	3.232913847	3.426926059	2.989664409	0.263228122	0.18255181	0.222971722	0.329661382	0.644243585	0.685902354	Angpt4	angiopoietin 4 [Source:MGI Symbol;Acc:MGI:1336887]
ENSMUSG00000027463	0.396243923	0.331851088	0.331913145	0.285730064	0.132464725	0.21949951	8.956497998	9.117542141	9.538705407	3.753744732	3.748103336	3.703153574	Slc52a3	solute carrier protein family 52, member 3 [Source:MGI Symbol;Acc:MGI:1916948]
ENSMUSG00000027465	77.43173079	81.54145161	73.9957729	73.89972837	70.21280561	72.15495628	37.56409921	38.09942321	40.59496619	40.89209829	40.39313725	38.6959118	Tbc1d20	TBC1 domain family, member 20 [Source:MGI Symbol;Acc:MGI:1914481]
ENSMUSG00000027466	79.19550293	73.40121987	84.36235694	84.4060812	74.07831002	67.15987835	111.8095529	117.2051997	108.3296722	69.66264554	68.05740125	66.7467907	Rbck1	RanBP-type and C3HC4-type zinc finger containing 1 [Source:MGI Symbol;Acc:MGI:1344372]
ENSMUSG00000027468	0	0	0	0	0	0	0	0	0	0	0	0	Defb22	defensin beta 22 [Source:MGI Symbol;Acc:MGI:3045368]
ENSMUSG00000027469	27.64861575	27.00124748	22.81791391	34.82264438	33.62922701	34.74206844	111.6510158	114.2087153	130.3233419	91.09447596	94.53138581	89.72448162	Tpx2	TPX2, microtubule-associated [Source:MGI Symbol;Acc:MGI:1919369]
ENSMUSG00000027470	0.131228868	0.067163017	0.074639529	0.013606736	0.107237624	0.088848582	0.091123002	0.057778174	0.040714149	0.260846943	0.376253182	0.323050701	Mylk2	myosin, light polypeptide kinase 2, skeletal muscle [Source:MGI Symbol;Acc:MGI:2139434]
ENSMUSG00000027472	169.7897848	144.8871238	193.5083516	123.86029	122.4914436	91.0495785	78.11954996	77.59771107	62.85085823	72.98666908	74.82328294	76.26580938	Pdrg1	p53 and DNA damage regulated 1 [Source:MGI Symbol;Acc:MGI:1915809]
ENSMUSG00000027474	0.055592362	0	0.113830104	0.025938939	0	0.063515544	0.694842322	0.578257624	0.181100814	1.864727053	2.036101448	1.502653221	Ccm2l	cerebral cavernous malformation 2-like [Source:MGI Symbol;Acc:MGI:2385159]
ENSMUSG00000027475	9.659456451	11.33439674	8.669733003	10.98787273	13.4782564	15.06383557	5.078805019	4.236109088	5.529412579	9.323171978	9.089286945	8.959709993	Kif3b	kinesin family member 3B [Source:MGI Symbol;Acc:MGI:107688]
ENSMUSG00000027478	2.264657254	2.41157801	1.844859374	2.965494543	2.379385176	3.876497065	52.19811241	45.52064211	57.51257611	65.02876265	69.70811249	66.97536486	Dnmt3b	DNA methyltransferase 3B [Source:MGI Symbol;Acc:MGI:1261819]
ENSMUSG00000027479	84.79812317	86.43657132	77.94934649	87.68092886	84.28156353	88.91844795	71.38636259	67.26680302	73.84459345	84.65453301	88.94250538	87.62368899	Mapre1	microtubule-associated protein, RP/EB family, member 1 [Source:MGI Symbol;Acc:MGI:891995]
ENSMUSG00000027480	0.06034851	0.138988824	0	0	0	0	0	0	0	0	0	0	Sun5	Sad1 and UNC84 domain containing 5 [Source:MGI Symbol;Acc:MGI:1923657]
ENSMUSG00000027481	0	0	0	0	0	0.086171833	0	0	0	0	0	0	Bpifb2	BPI fold containing family B, member 2 [Source:MGI Symbol;Acc:MGI:1913807]
ENSMUSG00000027482	0	0	0	0	0	0	0	0	0	0	0	0	Bpifa3	BPI fold containing family A, member 3 [Source:MGI Symbol;Acc:MGI:1920638]
ENSMUSG00000027483	0	0	0	0	0	0	0	0	0	0	0	0	Bpifa1	BPI fold containing family A, member 1 [Source:MGI Symbol;Acc:MGI:1338036]
ENSMUSG00000027484	0	0	0	0	0	0	0	0	0	0	0	0	Bpifa5	BPI fold containing family A, member 5 [Source:MGI Symbol;Acc:MGI:1914385]
ENSMUSG00000027485	0.056270908	0.021599614	0	0.035007391	0	0	0.133966361	0.167233055	0.017458218	0	0.03122657	0.016622888	Bpifb1	BPI fold containing family B, member 1 [Source:MGI Symbol;Acc:MGI:2137431]
ENSMUSG00000027487	8.067841468	8.271319459	7.388510318	10.16543743	8.47655969	8.219433973	20.87895945	18.00807173	19.23246438	15.65096101	16.97334526	16.41162987	Cdk5rap1	CDK5 regulatory subunit associated protein 1 [Source:MGI Symbol;Acc:MGI:1914221]
ENSMUSG00000027488	3.831303711	2.845572815	4.572335769	1.882855388	1.857876097	1.295074577	15.62500914	16.73143126	12.66042725	7.198750296	8.038982589	7.554252984	Snta1	syntrophin, acidic 1 [Source:MGI Symbol;Acc:MGI:101772]
ENSMUSG00000027489	2.39313058	2.755812882	3.283093174	2.411888546	2.765803681	2.807118564	9.315281632	12.61231979	7.840545793	3.724641907	4.701213912	3.775115147	Necab3	N-terminal EF-hand calcium binding protein 3 [Source:MGI Symbol;Acc:MGI:1861721]
ENSMUSG00000027490	27.97478946	27.97091826	27.08314362	31.73939265	31.83034659	28.91614576	20.28539488	20.39640599	19.58672061	15.14832496	14.41028809	13.79955334	E2f1	E2F transcription factor 1 [Source:MGI Symbol;Acc:MGI:101941]
ENSMUSG00000027495	8.826966538	8.822197418	7.770344381	8.958765998	9.086659508	9.541344686	4.29073847	4.348614266	4.185396537	5.022482756	5.020502037	5.67196647	Fam210b	family with sequence similarity 210, member B [Source:MGI Symbol;Acc:MGI:1914267]
ENSMUSG00000027496	36.1464098	36.48386525	32.72180832	44.52175965	40.84382652	39.07853325	98.83245237	97.5050911	93.1113919	72.42836323	75.06577145	71.48545965	Aurka	aurora kinase A [Source:MGI Symbol;Acc:MGI:894678]
ENSMUSG00000027498	26.67641102	24.81078509	26.81998364	27.54104272	26.36238826	26.89598494	35.6962071	39.87779256	37.00130044	35.48430055	38.03915374	35.17543268	Cstf1	cleavage stimulation factor, 3' pre-RNA, subunit 1 [Source:MGI Symbol;Acc:MGI:1914587]
ENSMUSG00000027499	8.515170832	11.33677281	7.733038003	7.259833271	10.55157989	12.38991576	1.060765201	1.373219288	1.421863368	2.985917181	2.025148778	3.309364509	Pkia	protein kinase inhibitor, alpha [Source:MGI Symbol;Acc:MGI:104747]
ENSMUSG00000027500	1.845224152	0.585109258	1.451068527	1.14795524	1.770120607	1.711011643	28.05310972	31.18124471	28.69898801	107.9050443	103.6441095	106.1985991	Stmn2	stathmin-like 2 [Source:MGI Symbol;Acc:MGI:98241]
ENSMUSG00000027502	73.60944882	61.96638829	73.420417	68.73892991	65.90188788	56.63272733	52.50030056	53.15544698	46.43794474	45.38372701	48.45937974	49.18690742	Rtf2	replication termination factor 2 [Source:MGI Symbol;Acc:MGI:1913654]
ENSMUSG00000027505	1.479489553	0.496914913	0.883569488	0.575264725	0.194305093	0.751266949	0.330213645	0	0.28688504	0.055140353	0.205254301	0.109263337	Fam209	family with sequence similarity 209 [Source:MGI Symbol;Acc:MGI:1923676]
ENSMUSG00000027506	38.73400492	38.37438183	34.23113933	39.94529389	35.10017199	39.88957275	135.012154	138.9552909	136.084058	92.29129979	95.14800557	97.93953421	Tpd52	tumor protein D52 [Source:MGI Symbol;Acc:MGI:107749]
ENSMUSG00000027508	3.006008732	4.962609911	2.464747229	3.835370835	5.072834485	7.203192789	0.664986655	0.764234673	1.151335732	1.30870983	1.140382743	1.555963963	Pag1	phosphoprotein associated with glycosphingolipid microdomains 1 [Source:MGI Symbol;Acc:MGI:2443160]
ENSMUSG00000027509	101.2435008	93.21252593	104.3636931	108.233769	101.5231786	93.8992702	206.9173801	204.9187234	204.6802657	127.7605158	125.2970973	130.1444809	Rae1	ribonucleic acid export 1 [Source:MGI Symbol;Acc:MGI:1913929]
ENSMUSG00000027510	22.50720902	21.8780169	23.92376049	26.09976059	23.68220369	19.48793689	64.80290383	63.15183282	61.24439479	35.73459647	38.26895388	36.05026143	Rbm38	RNA binding motif protein 38 [Source:MGI Symbol;Acc:MGI:1889294]

ENSMUSG00000027513	0.398846398	1.837168518	0.240197656	1.313636134	0.27115131	0.196572643	0.523648502	1.185342548	0.262044612	0.33577288	0.156235008	0.124753232	Pck1	phosphoenolpyruvate carboxykinase 1, cytosolic [Source:MGI Symbol;Acc:MGI:97501]
ENSMUSG00000027514	48.96930469	43.36804252	48.80937189	54.60714589	49.7336086	45.88039674	1.439609916	1.513343705	1.658840596	8.141685194	7.611969098	7.897347125	Zbp1	Z-DNA binding protein 1 [Source:MGI Symbol;Acc:MGI:1927449]
ENSMUSG00000027517	0.151153939	0	0.051583431	0	0	0	0.044982265	0.099826341	0	0	0	0	Ankrd60	ankyrin repeat domain 60 [Source:MGI Symbol;Acc:MGI:1917315]
ENSMUSG00000027518	0	0.23010611	0.136384676	0.124314173	0.069981917	0	0	0	0	0.476631162	0.221776319	0	1700021F07	RIKEN cDNA 1700021F07 gene [Source:MGI Symbol;Acc:MGI:1919471]
ENSMUSG00000027519	18.39176194	19.91463786	18.3638866	21.48563288	21.4595307	23.01238052	5.000976816	4.588907049	4.830489767	8.916071955	8.70430048	9.289954053	Rab22a	RAB22A, member RAS oncogene family [Source:MGI Symbol;Acc:MGI:105072]
ENSMUSG00000027520	0.463502562	0.413392305	0.334962722	0.309557913	0.25304054	0.640320395	8.097586728	7.630247318	10.48070519	14.41317233	13.88570598	14.84803248	Zdbf2	zinc finger, DBF-type containing 2 [Source:MGI Symbol;Acc:MGI:1921134]
ENSMUSG00000027522	48.51119334	56.54401314	40.3376632	55.77847265	61.10605928	68.49586074	49.66817485	49.36184987	58.51028445	53.75333717	57.6291513	58.06872057	Stx16	syntaxin 16 [Source:MGI Symbol;Acc:MGI:1923396]
ENSMUSG00000027523	4.589705368	7.191032267	3.546770913	5.703853005	8.786642387	9.243954539	2.698889748	2.972879634	2.998551858	3.552735357	3.342908378	3.16796439	Gnas	GNAS (guanine nucleotide binding protein, alpha stimulating) complex locus [Source:MGI Symbol;Acc:MGI:95777]
ENSMUSG00000027524	0.024559639	0	0	0	0.025803835	0.037413266	0.021926287	0	0.022859105	0.043936035	0.183990799	0.108826783	Edn3	endothelin 3 [Source:MGI Symbol;Acc:MGI:95285]
ENSMUSG00000027525	0.100260554	0.01924253	0.15396903	0.062374335	0.052669885	0.1527333	0.507225258	0.562826727	0.388826714	0.986487682	0.40337452	0.592355801	Phactr3	phosphatase and actin regulator 3 [Source:MGI Symbol;Acc:MGI:1921439]
ENSMUSG00000027528	0	0.086625027	0.077014423	0	0	0	0	0	0	0	0	0	Fabp9	fatty acid binding protein 9, testis [Source:MGI Symbol;Acc:MGI:1194881]
ENSMUSG00000027530	0	0	0	0	0	0	0	0	0	0	0	0	Fabp12	fatty acid binding protein 12 [Source:MGI Symbol;Acc:MGI:1922747]
ENSMUSG00000027531	16.14147256	20.16115921	16.83374469	18.39107085	19.87900758	16.75743789	34.87936427	32.46263428	34.0353123	27.13629491	27.41205432	26.22919924	Impa1	inositol (myo)-1(or 4)-monophosphatase 1 [Source:MGI Symbol;Acc:MGI:1933158]
ENSMUSG00000027533	167.8986664	137.2519238	209.0240703	119.5482864	99.27430059	78.15820634	11.7989779	13.25375388	9.211494387	24.49896582	24.8667091	26.2220116	Fabp5	fatty acid binding protein 5, epidermal [Source:MGI Symbol;Acc:MGI:101790]
ENSMUSG00000027534	2.940243431	4.118598188	3.526875717	4.115680288	4.518526464	5.281280225	4.172546618	3.890885639	4.023295691	5.829131062	4.986235087	5.911475281	Snx16	sorting nexin 16 [Source:MGI Symbol;Acc:MGI:1921968]
ENSMUSG00000027536	0.57360233	0.593522019	0.417032508	0.29478983	0.384305643	0.417906755	6.865096603	6.604700513	7.350597749	11.12403356	10.71184761	10.8151283	Chmp4c	charged multivesicular body protein 4C [Source:MGI Symbol;Acc:MGI:1913621]
ENSMUSG00000027540	45.84875552	49.96664925	47.31047436	43.5579519	46.07823187	44.83517556	40.35523155	39.88323453	37.88172901	44.29703406	47.08099206	46.15847092	Ptpn1	protein tyrosine phosphatase, non-receptor type 1 [Source:MGI Symbol;Acc:MGI:97805]
ENSMUSG00000027544	1.167483103	1.189291792	0.965402822	0.733301187	0.813820497	1.111563445	3.287261188	3.047086426	3.677876686	3.042487297	3.074289599	2.944779491	Nfatc2	nuclear factor of activated T cells, cytoplasmic, calcineurin dependent 2 [Source:MGI Symbol;Acc:MGI:102463]
ENSMUSG00000027546	17.60622582	17.95474021	17.71576916	9.662400565	8.465977219	6.935326464	30.91532498	30.59242721	29.00563549	24.28922222	24.69955879	26.0289209	Atp9a	ATPase, class II, type 9A [Source:MGI Symbol;Acc:MGI:1330826]
ENSMUSG00000027547	7.833682481	7.347447963	8.81045004	7.861182204	7.204702977	10.18675364	78.71497539	81.96759315	84.98028405	91.27436458	90.08085938	94.71851312	Sall4	spalt like transcription factor 4 [Source:MGI Symbol;Acc:MGI:2139360]
ENSMUSG00000027550	6.706155075	8.424526146	6.175856105	9.806311701	11.42850106	12.81877243	5.872516842	6.675188678	8.392104085	9.743970606	9.481723188	10.53560118	Lrrcc1	leucine rich repeat and coiled-coil domain containing 1 [Source:MGI Symbol;Acc:MGI:1918960]
ENSMUSG00000027551	31.10967546	30.41294248	27.65696489	36.11118141	33.84417731	32.57405712	62.43878661	62.85947156	61.28326542	44.3116489	43.64020765	42.39057495	Zfp64	zinc finger protein 64 [Source:MGI Symbol;Acc:MGI:107342]
ENSMUSG00000027552	8.947000019	9.376508906	7.787139978	10.98817552	11.96163437	11.28986093	22.04773211	21.10764356	24.1429446	17.46677681	16.33574207	17.58650675	E2f5	E2F transcription factor 5 [Source:MGI Symbol;Acc:MGI:105091]
ENSMUSG00000027555	12.18423035	12.95313566	10.0022943	13.55332701	16.02643734	16.35137089	9.023062722	8.510336709	9.9295417	12.75677702	10.92110905	12.00879643	Car13	carbonic anhydrase 13 [Source:MGI Symbol;Acc:MGI:1931322]
ENSMUSG00000027556	0.04707712	0	0	0.08786319	0	0	0	0	0	0	0	0	Car1	carbonic anhydrase 1 [Source:MGI Symbol;Acc:MGI:88268]
ENSMUSG00000027559	0	0.038321358	0	0.062109014	0.069927793	0.02534727	0.029709864	0.395599515	0.371685861	0.982291485	0.720215594	0.707803335	Car3	carbonic anhydrase 3 [Source:MGI Symbol;Acc:MGI:88270]
ENSMUSG00000027560	2.604099659	2.550342716	2.940138871	0.908449725	0.869390737	0.630269628	0.47801289	0.313425351	0.385087995	2.133382751	2.977986819	1.962723035	Dok5	docking protein 5 [Source:MGI Symbol;Acc:MGI:1924079]
ENSMUSG00000027562	2.57774351	1.37003269	0.771421893	0.629132832	0.541666487	1.147845884	72.29781752	71.0692705	73.12199923	63.56722188	65.69178083	62.45361441	Car2	carbonic anhydrase 2 [Source:MGI Symbol;Acc:MGI:88269]
ENSMUSG00000027564	0	0	0	0	0	0	0	0	0	0	0	0	Cypt12	cysteine-rich perinuclear theca 12 [Source:MGI Symbol;Acc:MGI:1922689]
ENSMUSG00000027566	328.9929356	262.2093985	375.6033964	306.1462533	246.050058	203.4655109	370.4092568	409.1265574	314.0217563	245.429843	236.6227312	241.6148279	Psma7	proteasome subunit alpha 7 [Source:MGI Symbol;Acc:MGI:1347070]
ENSMUSG00000027568	0.039645413	0.01521791	0.067647804	0.061660746	0.069423093	0.100657312	0.035394522	0	0	0.023641257	0	0.02342316	Ntsr1	neurotensin receptor 1 [Source:MGI Symbol;Acc:MGI:97386]
ENSMUSG00000027569	23.5533537	24.13435606	22.01496133	26.4976321	26.99209276	24.55144577	18.12682673	18.8175942	15.97500367	13.12843455	14.10527737	14.68880262	Mrgbp	MRG/MORF4L binding protein [Source:MGI Symbol;Acc:MGI:1920497]
ENSMUSG00000027570	4.100554749	4.218319627	4.030193157	3.494934221	4.222123759	3.643871418	2.660240847	2.383147775	2.340865273	3.594492412	3.049213033	3.076797265	Col9a3	collagen, type IX, alpha 3 [Source:MGI Symbol;Acc:MGI:894686]
ENSMUSG00000027573	37.62609275	36.88767485	39.23708548	34.84515248	38.66413046	39.50310316	64.34629914	70.67579622	67.94158128	68.61887062	65.02059079	69.08420621	Gid8	GID complex subunit 8 [Source:MGI Symbol;Acc:MGI:1923675]
ENSMUSG00000027574	1.158756405	0.711662702	1.107237886	0.072088829	0.081163947	0.294201262	3.241468835	4.209015482	2.804159622	2.418453901	2.314914753	2.73844917	Nkain4	Na+/K+ transporting ATPase interacting 4 [Source:MGI Symbol;Acc:MGI:1915372]
ENSMUSG00000027575	72.71461337	68.07975652	75.50338779	72.91645623	67.78597792	62.41664305	27.71904183	25.01082857	25.44740929	34.6752663	35.56493966	35.60710268	Arfgap1	ADP-ribosylation factor GTPase activating protein 1 [Source:MGI Symbol;Acc:MGI:2183559]
ENSMUSG00000027577	1.254779718	1.655664252	2.381926043	0.780626544	1.09862256	1.015476946	0.980208913	0.776898804	1.192228691	0.771629945	1.13151733	0.880346524	Chrna4	cholinergic receptor, nicotinic, alpha polypeptide 4 [Source:MGI Symbol;Acc:MGI:87888]
ENSMUSG00000027579	0	0	0.017194477	0.015672708	0.088228568	0	0.419834471	0.216290406	0.297007759	0.015022625	0.237660766	0.074420189	Srms	src-related kinase lacking C-terminal regulatory tyrosine and N-terminal myristylation sites [Source:MGI Symbol;Acc:MGI:101865]
ENSMUSG00000027580	8.671447509	11.55576128	7.757698369	11.0442181	13.02910077	16.75235198	2.169977353	1.655061722	2.86925284	7.327368347	8.294764875	7.842463615	Helz2	helicase with zinc finger 2, transcriptional coactivator [Source:MGI Symbol;Acc:MGI:2385169]
ENSMUSG00000027581	0	0	0	0	0	0.115401481	7.033705675	9.268120055	7.509215911	2.032815125	1.923265321	2.450441939	Stmn3	stathmin-like 3 [Source:MGI Symbol;Acc:MGI:1277137]
ENSMUSG00000027582	5.596035099	6.588701744	5.692454173	7.180426007	8.122044689	8.716058844	6.965594632	6.396542381	8.013169125	8.807792221	10.39122357	9.664362672	Zgpat	zinc finger, CCCH-type with G patch domain [Source:MGI Symbol;Acc:MGI:2449939]
ENSMUSG00000027583	4.262635243	4.07510035	3.870010765	4.668310881	5.965807991	6.174993047	3.661976743	3.282588645	4.266918947	3.539454104	3.387539999	3.962971029	Zbtb46	zinc finger and BTB domain containing 46 [Source:MGI Symbol;Acc:MGI:1919397]
ENSMUSG00000027584	0.040901604	0	0.104686906	0.171759173	0.171894747	0.093462036	0.38341822	0.3038915	0.380695357	1.060980062	1.481020992	1.613036368	Oprl1	opioid receptor-like 1 [Source:MGI Symbol;Acc:MGI:97440]
ENSMUSG00000027589	20.96581089	26.0919882	17.93532983	25.44320882	27.72425641	34.03915035	15.50023973	15.89543629	21.23509272	27.13499741	26.3996198	26.7735762	Pcmtd2	protein-L-isoaspartate (D-aspartate) O-methyltransferase domain containing 2 [Source:MGI Symbol;Acc:MGI:1923927]
ENSMUSG00000027593	294.434158	241.3811146	320.4307802	291.6982151	270.9824052	221.7995006	359.6968204	376.5703587	312.3698403	192.7651739	203.6384653	207.2328354	Raly	hnRNP-associated with lethal yellow [Source:MGI Symbol;Acc:MGI:97850]
ENSMUSG00000027596	1.830999482	0.862564102	2.72663831	0.621330411	0.349774223	0.380356139	0.297213571	0.164896896	0.154929019	0.297779237	0.069278289	0.590064289	a	nonagouti [Source:MGI Symbol;Acc:MGI:87853]
ENSMUSG00000027597	48.95434098	49.85376704	34.73944994	52.05863179	53.06028943	59.53947082	193.0851876	180.1129021	205.3397286	182.1426128	175.5341082	179.5926011	Ahcy	S-adenosylhomocysteine hydrolase [Source:MGI Symbol;Acc:MGI:87968]
ENSMUSG00000027598	15.24138106	20.57152257	13.59011173	17.51360632	22.88738095	29.35566698	19.91640419	18.88066671	26.14790523	32.25794676	31.438314	31.25012846	Itch	itchy, E3 ubiquitin protein ligase [Source:MGI Symbol;Acc:MGI:1202301]
ENSMUSG00000027599	12.59341803	12.49192798	12.05350162	12.73585671	13.68680915	13.82166021	22.23522455	20.47154787	23.93353869	21.94222749	22.52572348	22.74685385	Armc1	armadillo repeat containing 1 [Source:MGI Symbol;Acc:MGI:1921502]
ENSMUSG00000027601	34.59593023	39.46977064	30.72496735	35.0669717	38.13399133	41.41932861	70.33181554	64.70108179	76.27841378	63.18106551	56.60774433	61.71733903	Mtfr1	mitochondrial fission regulator 1 [Source:MGI Symbol;Acc:MGI:1914722]
ENSMUSG00000027602	101.9986777	75.75931405	122.5278434	74.16325414	61.75999774	49.85949369	45.42608973	47.75026655	39.61145588	42.31573133	39.06583795	43.3423179	Map1lc3a	microtubule-associated protein 1 light chain 3 alpha [Source:MGI Symbol;Acc:MGI:1915661]
ENSMUSG00000027603	5.219968195	5.30142248	6.271971595	3.416597524	3.275413124	2.705858486	2.006504593	3.016487959	2.159347681	6.063394871	4.82788549	5.397074969	Ggt7	gamma-glutamyltransferase 7 [Source:MGI Symbol;Acc:MGI:1913385]
ENSMUSG00000027605	5.692310369	5.754969783	5.322980232	5.019184729	5.415579802	5.58182539	14.80572501	13.0319649	14.72640676	4.851087428	5.876206133	5.10424818	Acss2	acyl-CoA synthetase short-chain family member 2 [Source:MGI Symbol;Acc:MGI:1890410]
ENSMUSG00000027606	0.231335956	0.159837147	0.142104033	0.215878913	0.29166657	0.211445294	0	0	0	0.04138491	0	0	Dnajc5b	DnaJ heat shock protein family (Hsp40) member C5 beta [Source:MGI Symbol;Acc:MGI:1913576]
ENSMUSG00000027610	20.86913915	21.5061241	28.36647895	20.52823979	19.52023341	13.93018718	30.75492065	30.25987839	23.8073292	20.45574824	19.08980395	18.69484233	Gss	glutathione synthetase [Source:MGI Symbol;Acc:MGI:95852]
ENSMUSG00000027611	51.57799147	47.60820997	52.43532766	43.82422488	37.27999386	33.59948571	11.21627735	12.72413364	10.34852143	27.21443891	23.85564281	24.90021761	Procr	protein C receptor, endothelial [Source:MGI Symbol;Acc:MGI:104596]
ENSMUSG00000027612	0.104227262	0	0.017784518	0.113473696	0.054753716	0.145544702	0.372206903	0.120460611	0.24252615	0.512758535	0.578391207	0.600396965	Mmp24	matrix metallopeptidase 24 [Source:MGI Symbol;Acc:MGI:1341867]
ENSMUSG00000027613	290.6086401	227.0593783	322.1036954	324.7981706	263.443319	214.973924	284.0026003	308.1585124	238.4885685	210.3942698	235.3021021	229.780235	Eif6	eukaryotic translation initiation factor 6 [Source:MGI Symbol;Acc:MGI:1196288]
ENSMUSG00000027615	5.392276799	7.003720241	4.493490002	6.58253735	6.884181082	8.727799303	5.079997096	4.084006537	5.091859337	8.118226787	7.828825241	8.140576374	Hps3	HPS3, biogenesis of lysosomal organelles complex 2 subunit 1 [Source:MGI Symbol;Acc:MGI:2153839]
ENSMUSG00000027618	22.07396408	20.98804686	20.45027225	22.72099234	21.94257403	21.58289164	25.45374477	25.64481894	25.33190235	25.62735322	23.32464818	26.35200731	Nfs1	nitrogen fixation gene 1 (S. cerevisiae) [Source:MGI Symbol;Acc:MGI:1316706]
ENSMUSG00000027620	191.6988299	216.4332903	158.1334159	256.4794904	282.0738983	317.6196332	306.2138368	298.5612463	344.9580471	308.26191	295.383166	308.805553	Rbm39	RNA binding motif protein 39 [Source:MGI Symbol;Acc:MGI:2157953]
ENSMUSG00000027624	18.47160761	22.36437516	18.59296578	24.67203663	25.57421419	25.39740055	8.653938039	7.849654602	9.576447064	9.697268959	9.864908733	10.57929933	Epb41l1	erythrocyte membrane protein band 4.1 like 1 [Source:MGI Symbol;Acc:MGI:103010]
ENSMUSG00000027628	25.75291099	22.69608018	26.00971889	24.80581539	23.01156789	20.32844785	24.85386146	24.92910049	24.94049088	25.54701559	27.02404402	25.90383146	Aar2	AAR2 splicing factor homolog [Source:MGI Symbol;Acc:MGI:1915545]
ENSMUSG00000027630	21.64307172	26.05429246	18.67934112	24.44974118	28.54282674	31.59651091	21.99652946	20.61100121	28.02841068	27.62721538	28.01544527	27.04410718	Tbl1xr1	transducin (beta)-like 1X-linked receptor 1 [Source:MGI Symbol;Acc:MGI:2441730]
ENSMUSG00000027634	41.01891559	41.92651322	42.47895555	40.23621165	39.55727856	40.58486209	36.62075374	34.77988123	40.02164524	28.54876626	25.48508448	25.7449487	Ndrg3	N-myc downstream regulated gene 3 [Source:MGI Symbol;Acc:MGI:1352499]
ENSMUSG00000027635	13.77468163	15.06113005	8.903041673	16.23018506	20.13725417	25.11703441	17.17333412	17.33290331	19.00473075	26.44686452	23.74279539	22.81192358	Dsn1	DSN1 homolog, MIS12 kinetochore complex component [Source:MGI Symbol;Acc:MGI:1914184]
ENSMUSG00000027636	0.092072371	0	0	0.042960212	0.032245591	0.116883098	0.027400039	0	0	0	0.07664096	0.0407984	Sla2	Src-like-adaptor 2 [Source:MGI Symbol;Acc:MGI:1925049]

ENSMUSG00000027637	45.35328708	48.09751517	41.43009245	44.30855384	41.68935584	43.00839888	51.50123293	46.88062554	49.03453808	43.52805721	43.52471956	50.18038831	Rab5if	RAB5 interacting factor [Source:MGI Symbol;Acc:MGI:1914638]
ENSMUSG00000027639	47.92697415	49.65544724	47.46624073	47.38582526	48.01118576	54.49491085	46.48382128	42.73975964	49.98116986	54.3274822	54.78775928	53.88743987	Samhd1	SAM domain and HD domain, 1 [Source:MGI Symbol;Acc:MGI:1927468]
ENSMUSG00000027641	5.075658762	7.372093078	4.056561852	7.425643161	11.19886425	14.86553752	10.30535469	8.77495571	11.64290402	10.48606694	9.676553116	10.05559473	Rbl1	RB transcriptional corepressor like 1 [Source:MGI Symbol;Acc:MGI:103300]
ENSMUSG00000027642	334.1425555	299.0652841	331.981759	301.232498	282.6528193	247.413288	213.8208787	219.5939859	218.7274828	198.1209429	201.1612836	192.5510662	Rpn2	ribophorin II [Source:MGI Symbol;Acc:MGI:98085]
ENSMUSG00000027643	0.337478336	0.097155913	0.431884806	0	0	0.064262786	0.45193946	0	0	0.226399802	0.210687503	0.224311204	Ghrh	growth hormone releasing hormone [Source:MGI Symbol;Acc:MGI:95709]
ENSMUSG00000027646	37.36586432	43.88671384	38.82700494	46.81095923	48.77018585	51.303402	32.96027739	30.87144727	35.07334785	27.64135767	29.59931398	26.61049453	Src	Rous sarcoma oncogene [Source:MGI Symbol;Acc:MGI:98397]
ENSMUSG00000027649	47.20094725	42.14211329	50.77133078	40.43739131	39.59152728	35.88589734	140.9708736	149.2765798	128.6427291	86.88949984	90.8807992	90.20935635	Ctnnbl1	catenin, beta like 1 [Source:MGI Symbol;Acc:MGI:1913892]
ENSMUSG00000027650	12.78818694	15.13772352	11.24410582	13.17974731	15.33289119	16.27241584	31.73254991	28.83951584	37.93116755	20.82521957	23.23087553	23.38307049	Tti1	TELO2 interacting protein 1 [Source:MGI Symbol;Acc:MGI:1922675]
ENSMUSG00000027651	9.532416992	11.73162933	8.257800345	11.95286632	12.78552876	13.43996278	17.04850667	15.47080259	20.91036193	19.39748849	19.83044556	19.53654617	Rprd1b	regulation of nuclear pre-mRNA domain containing 1B [Source:MGI Symbol;Acc:MGI:1917720]
ENSMUSG00000027652	12.34309063	15.56561755	11.92002064	15.35782582	20.49368324	25.47267436	12.32736994	11.42735492	16.43067818	21.20450913	21.07771571	20.51254372	Ralgapb	Ral GTPase activating protein, beta subunit (non-catalytic) [Source:MGI Symbol;Acc:MGI:2444531]
ENSMUSG00000027654	5.907379094	5.686590817	4.362700502	6.158685017	6.675379148	7.171147883	12.25100101	12.43571388	14.16110506	12.57704729	13.63781637	12.67915673	Fam83d	family with sequence similarity 83, member D [Source:MGI Symbol;Acc:MGI:1919128]
ENSMUSG00000027655	16.66407081	17.69826538	15.30947026	19.76891839	20.84563004	21.49573756	30.36540424	25.75462916	33.02178763	22.24914808	25.07791452	22.75847974	Dhx35	DEAH (Asp-Glu-Ala-His) box polypeptide 35 [Source:MGI Symbol;Acc:MGI:1918965]
ENSMUSG00000027656	54.54870082	47.84873133	53.66242835	3.951028711	3.103545882	2.599922308	0.263717534	0.195083971	0.244388415	4.286226613	4.371243384	4.043076851	Ccn5	cellular communication network factor 5 [Source:MGI Symbol;Acc:MGI:1328326]
ENSMUSG00000027660	10.58920591	13.55964793	8.98236126	10.78267783	12.90091036	17.39421193	17.08055585	14.98142598	21.24558648	37.92472784	38.87441363	40.53248747	Skil	SKI-like [Source:MGI Symbol;Acc:MGI:106203]
ENSMUSG00000027661	4.76143899	5.154564603	3.886302542	2.989499115	2.397585471	4.011098905	0.313430732	0.239104704	0.285919491	1.943043687	2.703160414	2.722389932	Slc2a10	solute carrier family 2 (facilitated glucose transporter), member 10 [Source:MGI Symbol;Acc:MGI:2156687]
ENSMUSG00000027663	22.55701501	27.2014883	23.47785713	27.19785357	30.44754111	32.22235896	20.21629412	19.13884137	23.54542492	28.43473159	28.26271801	27.83214844	Zmat3	zinc finger matrin type 3 [Source:MGI Symbol;Acc:MGI:1195270]
ENSMUSG00000027665	10.61379696	12.20448998	10.50940431	10.09259184	11.19219261	12.56907885	6.072783917	5.287938254	6.612364412	10.08975136	11.47894101	10.74504786	Pik3ca	phosphatidylinositol-4,5-bisphosphate 3-kinase catalytic subunit alpha [Source:MGI Symbol;Acc:MGI:1206581]
ENSMUSG00000027667	24.49313923	24.92721786	23.58489703	23.07280611	23.5778768	23.26955465	33.7755217	35.34666448	34.87356886	31.14564663	33.25459131	30.33032573	Zfp639	zinc finger protein 639 [Source:MGI Symbol;Acc:MGI:1915028]
ENSMUSG00000027668	35.70654874	40.7836343	37.71189635	40.84577918	41.07382447	47.3921183	66.89483209	63.33165678	68.78006457	53.54847495	60.0885812	60.60101795	Mfn1	mitofusin 1 [Source:MGI Symbol;Acc:MGI:1914664]
ENSMUSG00000027669	17.83316447	20.6931681	17.10325554	22.53950681	23.72584234	25.17575623	7.823271194	7.953214934	8.187897195	16.34860025	17.44633037	16.72761401	Gnb4	guanine nucleotide binding protein (G protein), beta 4 [Source:MGI Symbol;Acc:MGI:104581]
ENSMUSG00000027670	0.047064247	0.013549225	0.024092015	0	0.074172795	0.053771978	0.115549192	0.128215566	0.054756838	0.052622322	0.166499011	0.072991614	Ocstamp	osteoclast stimulatory transmembrane protein [Source:MGI Symbol;Acc:MGI:1921864]
ENSMUSG00000027671	32.53579856	37.02815327	23.86705001	36.60007272	39.47392989	42.37742098	167.1065038	154.7020687	168.5057221	174.8799337	173.5551252	161.0805885	Actl6a	actin-like 6A [Source:MGI Symbol;Acc:MGI:1861453]
ENSMUSG00000027673	32.42106737	22.31205823	43.87709215	29.2968715	24.17701319	19.15618701	66.44318696	63.13371081	43.31359528	31.71622676	28.73182755	30.89295956	Ndufb5	NADH:ubiquinone oxidoreductase subunit B5 [Source:MGI Symbol;Acc:MGI:1913296]
ENSMUSG00000027674	0.060710388	0.017477783	0.031077425	0.042490453	0.04783949	0.069363007	0.054200852	0	0.02825337	0	0.012633819	0	Pex5l	peroxisomal biogenesis factor 5-like [Source:MGI Symbol;Acc:MGI:1916672]
ENSMUSG00000027676	2.405986339	3.413795884	2.815121331	3.50816706	4.130365581	3.321580724	2.263081768	2.660125293	3.539041348	2.978349869	2.82529522	3.274518026	Ccdc39	coiled-coil domain containing 39 [Source:MGI Symbol;Acc:MGI:1289263]
ENSMUSG00000027677	16.7865084	18.82921699	13.17467135	18.75237289	23.61956252	29.16635831	24.81186042	21.80456151	27.16405421	32.0395576	30.22184758	31.18842589	Ttc14	tetratricopeptide repeat domain 14 [Source:MGI Symbol;Acc:MGI:1914370]
ENSMUSG00000027678	10.92498342	13.74048661	9.810075223	13.11906542	14.40317035	20.94724466	59.54552153	52.75572243	76.53085587	61.62328816	62.86583573	62.08374079	Ncoa3	nuclear receptor coactivator 3 [Source:MGI Symbol;Acc:MGI:1276535]
ENSMUSG00000027679	15.88175946	13.52510384	19.17123678	14.68273095	13.19384398	11.33727924	53.61592508	55.35871894	41.21796796	31.68236888	30.95930782	29.85168161	Dnajc19	DnaJ heat shock protein family (Hsp40) member C19 [Source:MGI Symbol;Acc:MGI:1914963]
ENSMUSG00000027680	43.92765602	49.01916913	38.50642665	43.20285001	48.87198941	47.90820142	140.2670818	137.6541137	146.8060641	140.6517426	130.4705449	131.8131293	Fxr1	fragile X mental retardation gene 1, autosomal homolog [Source:MGI Symbol;Acc:MGI:104860]
ENSMUSG00000027684	6.346384635	9.135235181	6.042565293	10.76385634	12.43562149	16.30241661	0.226636307	0.282914918	0.073836931	0.510902208	0.435824812	0.506189	Mecom	MDS1 and EVI1 complex locus [Source:MGI Symbol;Acc:MGI:95457]
ENSMUSG00000027690	0.148990609	0.171570345	0	0.180746264	0.0313077	0	0.093110796	0.177115711	0.055469739	0.013326858	0	0.026407829	Slc2a2	solute carrier family 2 (facilitated glucose transporter), member 2 [Source:MGI Symbol;Acc:MGI:1095438]
ENSMUSG00000027692	1.551241621	1.772157211	1.28564507	1.562481541	2.496482244	2.419369764	1.220042576	1.024483893	1.397996133	3.590007736	3.894252556	3.622353178	Tnik	TRAF2 and NCK interacting kinase [Source:MGI Symbol;Acc:MGI:1916264]
ENSMUSG00000027694	13.3956613	16.04905695	7.896158054	9.470159867	8.103377817	12.05836608	7.368887102	10.05326597	18.76516848	8.835287878	12.84001196	17.98721919	Gm8325	predicted pseudogene 8325 [Source:MGI Symbol;Acc:MGI:3644852]
ENSMUSG00000027695	4.453385766	5.272761867	3.050265138	4.024127699	5.3215331	6.294427433	0.811975778	0.761175852	1.065447544	4.263972951	4.281317477	4.377501814	Pld1	phospholipase D1 [Source:MGI Symbol;Acc:MGI:109585]
ENSMUSG00000027698	30.57862079	38.30296132	26.54635237	36.39727868	39.53678893	47.818223	10.58567407	9.056833485	10.01954842	24.00264763	25.81724019	24.22365809	Nceh1	neutral cholesterol ester hydrolase 1 [Source:MGI Symbol;Acc:MGI:2443191]
ENSMUSG00000027699	15.79376225	20.40596415	12.61418773	21.66378904	29.88897111	34.29586571	61.71009725	57.77699202	82.511436	81.67788994	82.44415119	83.91768577	Ect2	ect2 oncogene [Source:MGI Symbol;Acc:MGI:95281]
ENSMUSG00000027702	0.045508713	0.104811244	0	0.021233991	0	0.207978978	10.19791472	10.21126865	12.30489531	6.777627077	6.515602943	5.222856856	Lrrc34	leucine rich repeat containing 34 [Source:MGI Symbol;Acc:MGI:1919077]
ENSMUSG00000027703	0.205681108	0.023685237	0.315862215	0.383876461	0.151270664	0.250661726	1.817911354	2.282072152	1.799531665	2.023745842	1.36967019	2.169127944	Lrriq4	leucine-rich repeats and IQ motif containing 4 [Source:MGI Symbol;Acc:MGI:1915557]
ENSMUSG00000027706	91.05738098	98.03375985	90.20344549	79.39702793	77.96530439	78.82142441	46.76299784	49.04077771	51.24243559	68.90262893	65.39025146	69.92878977	Sec62	SEC62 homolog (S. cerevisiae) [Source:MGI Symbol;Acc:MGI:1916526]
ENSMUSG00000027708	18.53785767	18.09198991	13.94199454	16.43679952	16.76391843	18.5712549	17.30245796	15.26775467	15.86280354	22.24667671	21.06475747	23.02909468	Dcun1d1	DCN1, defective in cullin neddylation 1, domain containing 1 (S. cerevisiae) [Source:MGI Symbol;Acc:MGI:2150386]
ENSMUSG00000027709	5.715664426	5.909787878	6.490392335	5.277421549	6.195527257	4.522140359	8.462767197	7.75559156	7.755074242	7.848808858	7.8736817	7.045134402	Mccc1	methylcrotonoyl-Coenzyme A carboxylase 1 (alpha) [Source:MGI Symbol;Acc:MGI:1919289]
ENSMUSG00000027710	10.56840052	16.75054476	7.995988734	14.19731605	17.81488388	21.45128206	24.15832928	23.64267666	27.29395617	37.39721429	33.47258606	36.46040887	Acad9	acyl-Coenzyme A dehydrogenase family, member 9 [Source:MGI Symbol;Acc:MGI:1914272]
ENSMUSG00000027712	461.437138	427.9037257	425.8384186	358.6532511	336.7107872	314.1365524	81.56019973	81.26966292	76.39949606	250.0083506	248.0945993	240.3749342	Anxa5	annexin A5 [Source:MGI Symbol;Acc:MGI:106008]
ENSMUSG00000027713	0.708482236	0.435121981	1.063830411	0.661143982	0.198499751	0.755493341	0.210838936	0.093580346	0.351693976	0.549224548	0.353843995	0.293008049	1810062G17	RIKEN cDNA 1810062G17 gene [Source:MGI Symbol;Acc:MGI:1919532]
ENSMUSG00000027714	24.29309427	22.46030034	20.46944526	25.83391913	22.98977034	24.12125321	35.29423229	34.31536561	31.98076031	34.76735874	35.55501368	31.68965191	Exosc9	exosome component 9 [Source:MGI Symbol;Acc:MGI:1355319]
ENSMUSG00000027715	18.15857903	22.48538937	15.32364913	24.17716764	28.60443921	34.74161929	91.57160623	84.44885424	112.769359	103.7974467	98.84951685	98.23880154	Ccna2	cyclin A2 [Source:MGI Symbol;Acc:MGI:108069]
ENSMUSG00000027716	0.173501967	0.299694651	0.177630041	0.026984864	0	0.066076655	0.077449303	0.057292806	0.134573762	1.163950596	1.179453561	0.922570275	Trpc3	transient receptor potential cation channel, subfamily C, member 3 [Source:MGI Symbol;Acc:MGI:109526]
ENSMUSG00000027718	0	0	0	0	0	0	0	0.043078168	0	0.019448189	0	0	Il21	interleukin 21 [Source:MGI Symbol;Acc:MGI:1890474]
ENSMUSG00000027719	1.678079363	2.442838519	1.426268586	1.418224383	1.630027667	1.881069617	0.113068003	0	0.088408723	0.45313279	0.316263801	0.168357195	Adad1	adenosine deaminase domain containing 1 (testis specific) [Source:MGI Symbol;Acc:MGI:103258]
ENSMUSG00000027720	0	0.086625027	0.192536059	0	0	0	0	0	0	0	0	0	Il2	interleukin 2 [Source:MGI Symbol;Acc:MGI:96548]
ENSMUSG00000027722	28.02099085	27.35294399	30.40679709	32.74303924	32.74846476	33.73326027	41.88156041	39.57619356	42.65397439	51.06696026	47.61023692	45.9454998	Spata5	spermatogenesis associated 5 [Source:MGI Symbol;Acc:MGI:1927170]
ENSMUSG00000027737	2.982765442	2.924414619	3.078261358	2.34750288	3.184216187	4.115000921	0.331531313	0.344139639	0.457131184	4.178815948	4.048343943	3.492685313	Slc7a11	solute carrier family 7 (cationic amino acid transporter, y+ system), member 11 [Source:MGI Symbol;Acc:MGI:1347355]
ENSMUSG00000027739	11.781291	13.25842671	11.01991573	10.54434826	11.02779141	12.1347494	4.063799124	3.97876612	4.18684347	7.903564967	7.689372769	8.328966737	Rab33b	RAB33B, member RAS oncogene family [Source:MGI Symbol;Acc:MGI:1330805]
ENSMUSG00000027742	14.97993441	17.0025545	13.15941733	14.04591396	14.13230607	17.01483026	24.20925007	24.40158189	27.30725046	22.88762464	23.08905743	22.18949701	Cog6	component of oligomeric golgi complex 6 [Source:MGI Symbol;Acc:MGI:1914792]
ENSMUSG00000027744	0	0	0.072394824	0	0.037147345	0	0	0	0	0.031625281	0.088291394	0.062667058	Stoml3	stomatin (Epb7.2)-like 3 [Source:MGI Symbol;Acc:MGI:2388072]
ENSMUSG00000027746	55.19473886	54.30229665	51.52568133	46.76428664	43.26665829	46.28714582	43.94197338	36.88951315	40.13515543	40.30629549	41.89318148	39.00638568	Ufm1	ubiquitin-fold modifier 1 [Source:MGI Symbol;Acc:MGI:1915140]
ENSMUSG00000027748	0	0.052322614	0.031011792	0.014133572	0	0.023072172	0	0	0	0.081283984	0.07564282	0.013422353	Trpc4	transient receptor potential cation channel, subfamily C, member 4 [Source:MGI Symbol;Acc:MGI:109525]
ENSMUSG00000027750	389.5827658	477.9622507	342.4263703	112.5003375	127.0999579	134.582374	6.889345258	7.232341632	8.678783165	94.70577396	89.37688004	92.40734222	Postn	periostin, osteoblast specific factor [Source:MGI Symbol;Acc:MGI:1926321]
ENSMUSG00000027751	26.1335813	28.43268582	23.63430622	30.29426803	31.29618577	34.33384423	60.87372765	58.78632322	71.58596311	54.80736369	53.85122435	53.82741008	Supt20	SPT20 SAGA complex component [Source:MGI Symbol;Acc:MGI:1929651]
ENSMUSG00000027752	43.63635748	33.49967252	38.5846147	39.74829642	39.27786286	33.13780896	86.63693123	94.92961239	87.08972909	52.89668032	58.80328598	55.41007341	Exosc8	exosome component 8 [Source:MGI Symbol;Acc:MGI:1916889]
ENSMUSG00000027761	0.057371317	0.396396125	0	0.05353797	0	0.131096083	0.307318833	0.170503391	0.213595475	0.205269154	0.095511668	0.050843873	Aadac	arylacetamide deacetylase [Source:MGI Symbol;Acc:MGI:1915008]
ENSMUSG00000027762	0	0.051134691	0.045461559	0.082876115	0	0	0	0.087979046	0	0	0	0	Sucnr1	succinate receptor 1 [Source:MGI Symbol;Acc:MGI:1934135]
ENSMUSG00000027763	46.46415274	50.31116488	43.58200842	50.25185463	56.19189356	65.64599949	4.65833414	4.058737847	5.403728386	24.14125645	24.63748352	24.92804316	Mbnl1	muscleblind like splicing factor 1 [Source:MGI Symbol;Acc:MGI:1928482]
ENSMUSG00000027765	0.465676275	0.612857336	0.326918369	0.595969986	0.894660598	0.699260181	0.190054937	0.237249616	0.185756732	0.452239555	0.465154227	0.436276275	P2ry1	purinergic receptor P2Y, G-protein coupled 1 [Source:MGI Symbol;Acc:MGI:105049]
ENSMUSG00000027770	21.12059835	22.66515493	15.5779421	23.08613047	29.95329467	33.65045469	31.00762676	31.00198001	40.10551908	48.33341027	48.1549195	53.06672697	Dhx36	DEAH (Asp-Glu-Ala-His) box polypeptide 36 [Source:MGI Symbol;Acc:MGI:1919412]
ENSMUSG00000027774	20.32929416	21.70456407	15.94145543	21.56262493	24.316006	23.73437037	39.72069464	37.56356975	44.40906346	48.55710732	48.37744068	50.55399283	Gfm1	G elongation factor, mitochondrial 1 [Source:MGI Symbol;Acc:MGI:107339]
ENSMUSG00000027775	58.67101584	69.10364082	52.67373616	58.86306081	66.63681884	76.63673137	19.01174815	18.07884479	20.658061	55.63729616	55.75141529	53.38606656	Mfsd1	major facilitator superfamily domain containing 1 [Source:MGI Symbol;Acc:MGI:1914118]

ENSMUSG00000027776	0.62700893	0.541524761	0.601806697	0	0	0.149244174	0	0	0	0.070105585	0	0	Il12a	interleukin 12a [Source:MGI Symbol;Acc:MGI:96539]
ENSMUSG00000027777	0.094933233	0.355291618	0.048595974	0.155032733	0.249356417	0.488085249	0.233074128	0.07053367	0.287169868	0.467035444	0.375356114	0.441693877	Schip1	schwannomin interacting protein 1 [Source:MGI Symbol;Acc:MGI:1353557]
ENSMUSG00000027778	12.31092846	14.94350471	10.34878259	13.68723709	17.47337384	20.66584079	11.95128793	11.95891838	16.03555313	18.48033306	19.27289014	17.46244921	Ift80	intraflagellar transport 80 [Source:MGI Symbol;Acc:MGI:1915509]
ENSMUSG00000027782	20.49684599	25.94634382	19.27730533	23.40665248	27.56299548	32.41112491	22.12731041	21.98648107	26.66248011	28.79272778	28.67002489	29.2484217	Kpna4	karyopherin (importin) alpha 4 [Source:MGI Symbol;Acc:MGI:1100848]
ENSMUSG00000027784	5.48461711	7.048593068	4.637895355	7.097551364	8.659621543	11.37858604	1.433793849	1.440873725	1.396079578	2.466482352	2.446645719	2.725697061	Ppm1l	protein phosphatase 1 (formerly 2C)-like [Source:MGI Symbol;Acc:MGI:2139740]
ENSMUSG00000027787	34.76540586	34.08704968	31.18218413	41.72858499	39.54404427	38.41545042	74.82799736	73.84653592	75.97145839	77.65195089	80.021778	78.27515447	Nmd3	NMD3 ribosome export adaptor [Source:MGI Symbol;Acc:MGI:2140103]
ENSMUSG00000027788	0.019911378	0	0	0.037161942	0.020920092	0	0	0	0	0.053430772	0.082871068	0	Otol1	otolin 1 [Source:MGI Symbol;Acc:MGI:2685260]
ENSMUSG00000027790	0.012504646	0.043199229	0	0.058345652	0.052552535	0.085721065	0	0	0	0.022370222	0.041635426	0.033245776	Sis	sucrase isomaltase (alpha-glucosidase) [Source:MGI Symbol;Acc:MGI:1917233]
ENSMUSG00000027792	0.622539824	0.82231111	0.58111479	1.315667146	1.26968043	1.534103094	0.702910096	0.580437075	0.852109607	3.848796637	4.496160964	4.186506132	Bche	butyrylcholinesterase [Source:MGI Symbol;Acc:MGI:894278]
ENSMUSG00000027793	0.112935664	0	0.115622704	0.070259804	0	0	0.26887037	0.298343641	0.350386278	0.976510083	1.00274717	0.733966669	Ccna1	cyclin A1 [Source:MGI Symbol;Acc:MGI:108042]
ENSMUSG00000027794	10.010858	10.31233574	8.124695891	13.74118594	13.69253635	14.0301645	30.71238333	31.96938578	35.17006707	14.71790254	14.16614999	14.3562966	Sohlh2	spermatogenesis and oogenesis specific basic helix-loop-helix 2 [Source:MGI Symbol;Acc:MGI:1921684]
ENSMUSG00000027796	0.248487914	0.147688571	0.155922644	0.119682497	0.269498172	0.329693948	0.014312539	0.031762927	0.014921442	0.308305245	0.313597829	0.319668538	Smad9	SMAD family member 9 [Source:MGI Symbol;Acc:MGI:1859993]
ENSMUSG00000027797	17.86942033	21.01417775	16.16584849	17.26896304	19.57142378	23.38339733	0.120114666	0.339262006	0.216297294	2.417805733	3.084853118	2.709842573	Dclk1	doublecortin-like kinase 1 [Source:MGI Symbol;Acc:MGI:1330861]
ENSMUSG00000027799	4.539608405	6.495965206	3.909900675	6.426477673	7.008370883	11.26704968	1.415284097	1.131930405	1.695861201	6.822866766	7.454708533	7.580967415	Nbea	neurobeachin [Source:MGI Symbol;Acc:MGI:1347075]
ENSMUSG00000027800	241.9996673	209.758623	240.5787951	227.3512612	212.9928889	180.9567393	15.06102517	13.89248915	13.4139371	87.5294581	89.44526991	92.33987203	Tm4sf1	transmembrane 4 superfamily member 1 [Source:MGI Symbol;Acc:MGI:104678]
ENSMUSG00000027801	0.258861224	0.298092006	0.559487135	0.295246161	0.483511426	0.460063124	0.15407027	0.113972855	0.053541646	0.540272255	0.57460228	0.407838553	Tm4sf4	transmembrane 4 superfamily member 4 [Source:MGI Symbol;Acc:MGI:2385173]
ENSMUSG00000027803	26.926603	31.08252389	23.84453326	28.25099513	31.74868034	36.72962833	14.50857869	14.84284664	17.44285906	20.5574945	20.58209182	21.68989703	Wwtr1	WW domain containing transcription regulator 1 [Source:MGI Symbol;Acc:MGI:1917649]
ENSMUSG00000027804	70.07496589	69.2884247	59.0839568	69.49010042	73.1821186	77.21291719	253.5693959	245.6640692	260.4992974	226.214986	223.4680647	221.7934252	Ppid	peptidylprolyl isomerase D (cyclophilin D) [Source:MGI Symbol;Acc:MGI:1914988]
ENSMUSG00000027805	60.222486	66.47782049	53.87792056	58.17383918	60.10512945	58.50295193	14.44439931	14.24691406	14.54615409	25.80561088	24.32844372	25.82450756	Pfn2	profilin 2 [Source:MGI Symbol;Acc:MGI:97550]
ENSMUSG00000027806	23.55263458	31.29385764	20.89794591	26.36589103	30.33101508	38.54495722	6.645963982	5.650337693	7.993983201	16.23618542	16.52662599	16.61451244	Tsc22d2	TSC22 domain family, member 2 [Source:MGI Symbol;Acc:MGI:1919283]
ENSMUSG00000027808	37.67208965	43.87566087	33.39984097	34.18836564	35.89409097	38.36074374	23.48546381	22.80589162	24.50198578	37.7033494	37.31604203	36.80970696	Serp1	stress-associated endoplasmic reticulum protein 1 [Source:MGI Symbol;Acc:MGI:92638]
ENSMUSG00000027809	27.8030229	30.46478461	25.07589627	28.61486855	27.54726651	31.45945827	18.13096684	16.48823999	19.16637966	21.33755938	21.03749444	22.06260914	Etfdh	electron transferring flavoprotein, dehydrogenase [Source:MGI Symbol;Acc:MGI:106100]
ENSMUSG00000027810	30.14235215	31.56200917	27.05083489	33.02205287	35.36609071	34.73704791	85.47305031	83.3646136	95.61734926	84.23251807	83.64732794	82.52198905	Eif2a	eukaryotic translation initiation factor 2A [Source:MGI Symbol;Acc:MGI:1098684]
ENSMUSG00000027811	5.242049279	5.634056599	4.785371341	5.523651012	6.127218781	5.589883723	6.844474	5.431009022	6.54610497	4.141852219	5.59979576	5.323121213	4930579G24	RIKEN cDNA 4930579G24 gene [Source:MGI Symbol;Acc:MGI:1923189]
ENSMUSG00000027820	11.19061792	13.31181017	8.991261514	11.17705017	13.69744571	17.878962	13.90388375	13.23309899	18.41065473	14.67712747	14.3534792	15.96459666	Mme	membrane metallo endopeptidase [Source:MGI Symbol;Acc:MGI:97004]
ENSMUSG00000027822	9.660827997	10.44776979	9.477856251	9.658157886	10.55625708	10.03312463	4.889981424	5.226285114	5.28567482	7.499217188	6.824925902	6.953559738	Slc33a1	solute carrier family 33 (acetyl-CoA transporter), member 1 [Source:MGI Symbol;Acc:MGI:1332247]
ENSMUSG00000027823	8.70125744	8.895070356	7.321206788	9.298960876	9.287088223	9.830184351	20.35679189	20.52141366	23.40688843	20.00139597	20.18648824	20.32500287	Gmps	guanine monophosphate synthetase [Source:MGI Symbol;Acc:MGI:2448526]
ENSMUSG00000027824	0.062723743	0.05056073	0.089902551	0.117065533	0.072491473	0.119438851	0.089597327	0.093205206	0.110923306	0.033662944	0.020884475	0.027793662	Vmn2r1	vomeronasal 2, receptor 1 [Source:MGI Symbol;Acc:MGI:3645892]
ENSMUSG00000027827	4.502318594	5.840936279	3.967619885	4.11285071	5.029799657	5.45510189	0.525766215	0.432845253	0.495088849	3.177593692	3.352396292	3.232458146	Kcnab1	potassium voltage-gated channel, shaker-related subfamily, beta member 1 [Source:MGI Symbol;Acc:MGI:109155]
ENSMUSG00000027828	222.0559491	262.1755693	188.0397385	208.19718	238.0516902	265.4429525	141.716051	132.6247373	159.8946113	222.9578423	218.4123039	214.8846688	Ssr3	signal sequence receptor, gamma [Source:MGI Symbol;Acc:MGI:1914687]
ENSMUSG00000027829	34.74696015	43.51258229	31.21772174	41.80782948	46.29927836	52.12207402	37.23911987	37.48264654	41.93492676	37.423261	36.15417389	38.58179115	Ccnl1	cyclin L1 [Source:MGI Symbol;Acc:MGI:1922664]
ENSMUSG00000027831	1.016420972	1.05666618	0.79490609	0.935333637	1.438716168	1.537626296	0.012603299	0.167818298	0.052557942	0	0.117509434	0.062553978	Veph1	ventricular zone expressed PH domain-containing 1 [Source:MGI Symbol;Acc:MGI:1920039]
ENSMUSG00000027832	78.0484309	79.94759719	69.33515644	48.90287378	50.63214383	47.35442152	0.224211211	0.331718659	0.181805487	1.447666699	1.13814974	1.582688651	Ptx3	pentraxin related gene [Source:MGI Symbol;Acc:MGI:104641]
ENSMUSG00000027833	8.011014867	8.800627846	6.59148461	6.925385669	7.771389962	8.273053753	0.17551047	0.243437166	0.388826714	1.868347882	2.63870756	1.916445239	Shox2	short stature homeobox 2 [Source:MGI Symbol;Acc:MGI:1201673]
ENSMUSG00000027834	8.597755067	9.237657735	7.846870594	5.002971396	6.425547786	6.684871768	2.464081549	2.478475685	2.809169835	5.73678502	5.421289874	5.032769147	Serpini1	serine (or cysteine) peptidase inhibitor, clade I, member 1 [Source:MGI Symbol;Acc:MGI:1194506]
ENSMUSG00000027835	63.92663083	64.53356159	57.12158903	62.82892408	68.51157523	60.66760292	80.34996968	83.39627477	84.3647075	79.35663169	79.59305662	77.35781696	Pdcd10	programmed cell death 10 [Source:MGI Symbol;Acc:MGI:1928396]
ENSMUSG00000027840	0.837130113	0.985420544	0.799911158	0.520797378	0.547268878	0.637626861	0.315556519	0.073715258	0.259722124	2.030061348	1.64141323	1.961874474	Wnt2b	wingless-type MMTV integration site family, member 2B [Source:MGI Symbol;Acc:MGI:1261834]
ENSMUSG00000027843	0.88807132	1.253582952	1.173162455	2.833734122	2.588492262	3.120296239	0.204606413	0.255414533	0.239974943	0.538114044	0.858460398	0.863194913	Ptpn22	protein tyrosine phosphatase, non-receptor type 22 (lymphoid) [Source:MGI Symbol;Acc:MGI:107170]
ENSMUSG00000027845	1.312935828	1.924253034	1.099778784	2.533957339	2.570792028	2.863749376	1.891438723	1.980535226	2.388506956	3.336335504	2.980599901	2.789890045	Dclre1b	DNA cross-link repair 1B [Source:MGI Symbol;Acc:MGI:2156057]
ENSMUSG00000027848	20.88405286	21.62394508	22.24663136	6.789019612	7.124039784	6.878048083	0.527011346	0.695416129	0.534582668	3.553394003	3.665353219	3.817532171	Olfml3	olfactomedin-like 3 [Source:MGI Symbol;Acc:MGI:1914877]
ENSMUSG00000027849	0.089344867	0.123462248	0.091470619	0.050025097	0	0.040831421	0.031906025	0	0.049895122	0.031966749	0.163615295	0.047507772	Syt6	synaptotagmin VI [Source:MGI Symbol;Acc:MGI:1859544]
ENSMUSG00000027852	83.98829836	82.82685304	83.2170466	103.2645284	102.7887357	114.151526	90.2692244	90.83215031	99.94514589	100.2053562	94.36369117	99.5629834	Nras	neuroblastoma ras oncogene [Source:MGI Symbol;Acc:MGI:97376]
ENSMUSG00000027854	24.5611662	26.743046	22.72989594	28.15597928	30.52927888	37.13256915	15.60810781	15.1104581	16.65256171	21.72010883	20.63027284	19.82384164	Sike1	suppressor of IKBKE 1 [Source:MGI Symbol;Acc:MGI:1913891]
ENSMUSG00000027855	7.167783542	7.551597065	5.576234202	9.67749643	11.62826619	12.761125	2.256264847	2.43884597	3.082263813	7.385792028	6.202213374	7.800351136	Sycp1	synaptonemal complex protein 1 [Source:MGI Symbol;Acc:MGI:105931]
ENSMUSG00000027857	0.072014206	0.124392089	0	0.268809893	0	0.109703835	0.096438964	0.071340331	0.033513934	0.064415006	0.239778246	0.03191038	Tshb	thyroid stimulating hormone, beta subunit [Source:MGI Symbol;Acc:MGI:98848]
ENSMUSG00000027858	3.651379614	4.477001412	3.523104259	3.235811401	5.050746189	5.262251177	3.447486904	4.762228406	5.158956637	6.156194865	6.822261996	6.960768316	Tspan2	tetraspanin 2 [Source:MGI Symbol;Acc:MGI:1917997]
ENSMUSG00000027859	37.91184998	38.38263724	42.10656757	35.24229368	31.99909283	26.20436984	1.435662723	2.124051301	1.587452212	12.9891438	9.532237528	11.14189061	Ngf	nerve growth factor [Source:MGI Symbol;Acc:MGI:97321]
ENSMUSG00000027860	20.33636911	24.85780169	19.93425847	25.30342067	27.30178332	29.86280875	31.09671707	31.90985247	34.12008719	34.70367471	32.23518793	32.61535032	Vangl1	VANGL planar cell polarity 1 [Source:MGI Symbol;Acc:MGI:2159344]
ENSMUSG00000027861	0.876282407	0.835104196	0.556840241	0.592151007	0.666695706	0.52935567	0.863255148	0.927950336	1.265598187	2.02710497	1.886422515	2.249412917	Casq2	calsequestrin 2 [Source:MGI Symbol;Acc:MGI:1309469]
ENSMUSG00000027863	0.603420002	0.481063258	1.188032637	0.04331551	0.243842062	0.282839445	1.367519077	0.64375606	0.993668269	1.494678306	1.313671808	1.768840239	Cd2	CD2 antigen [Source:MGI Symbol;Acc:MGI:88320]
ENSMUSG00000027864	26.1037218	31.40886557	24.18623412	19.61502169	21.95286302	25.47935361	23.95470282	22.59181196	27.33463104	26.96090692	31.01667567	28.73827948	Ptgfrn	prostaglandin F2 receptor negative regulator [Source:MGI Symbol;Acc:MGI:1277114]
ENSMUSG00000027865	13.31646168	14.91031296	11.53172054	14.80889733	15.34594293	17.9004284	8.716765052	8.264460991	12.78633461	12.14666095	12.13611561	11.96463615	Gdap2	ganglioside-induced differentiation-associated-protein 2 [Source:MGI Symbol;Acc:MGI:1338001]
ENSMUSG00000027867	0.034897395	0	0.035727697	0.081414189	0.073330606	0.039871071	0	0.051856262	0	0	0	0	Spag17	sperm associated antigen 17 [Source:MGI Symbol;Acc:MGI:1921612]
ENSMUSG00000027868	37.21382731	37.07231393	36.18266562	25.96609213	28.10246816	26.53032125	1.164853848	1.152049938	1.452012745	8.372464669	8.263616623	9.149886161	Tbx15	T-box 15 [Source:MGI Symbol;Acc:MGI:1277234]
ENSMUSG00000027869	0.785908453	0.641051572	0.905183223	0.336140225	0.550481806	0.448959187	0	0	0	0	0	0.087061426	Hsd3b6	hydroxy-delta-5-steroid dehydrogenase, 3 beta- and steroid delta-isomerase 6 [Source:MGI Symbol;Acc:MGI:109598]
ENSMUSG00000027870	0.083999	0.411098958	0	0.215562839	0.022063601	0.01599513	0.093740493	0.041606489	0.039091412	0	0	0.03722099	Hao2	hydroxyacid oxidase 2 [Source:MGI Symbol;Acc:MGI:96012]
ENSMUSG00000027871	0	0.112676556	0.150263503	0.022827446	0.025701147	0.018632189	0	0	0	0.021880595	0.040724134	0	Hsd3b1	hydroxy-delta-5-steroid dehydrogenase, 3 beta- and steroid delta-isomerase 1 [Source:MGI Symbol;Acc:MGI:96233]
ENSMUSG00000027875	1.006514335	1.210187688	1.454769872	0.980700697	0.59095841	0.541160299	0.198219061	0.395905706	0.123991181	0.344233617	0.07392544	0.170528986	Hmgcs2	3-hydroxy-3-methylglutaryl-Coenzyme A synthase 2 [Source:MGI Symbol;Acc:MGI:101939]
ENSMUSG00000027876	0.042143475	0	0.04314618	0.039327598	0	0.096299767	0	0	0	0.113089029	0	0.149394338	Reg4	regenerating islet-derived family, member 4 [Source:MGI Symbol;Acc:MGI:1914959]
ENSMUSG00000027878	11.19795916	16.45254972	11.60653833	14.50526571	19.43855573	23.49386475	2.844856086	2.530866724	3.405276356	8.96065432	10.3844668	10.42225565	Notch2	notch 2 [Source:MGI Symbol;Acc:MGI:97364]
ENSMUSG00000027879	84.57877518	84.40809943	80.99823748	67.38227386	75.08834952	69.95380954	54.88246142	52.52624997	50.57738254	59.41346265	55.45062201	57.38446822	Sec22b	SEC22 homolog B, vesicle trafficking protein [Source:MGI Symbol;Acc:MGI:1338759]
ENSMUSG00000027880	0.134801027	0.031046062	0	0	0	0.020535101	0	0	0	0	0	0	Slc25a54	solute carrier family 25, member 54 [Source:MGI Symbol;Acc:MGI:1921936]
ENSMUSG00000027881	47.74479239	56.58358496	40.01075935	53.02740796	59.6810924	70.07045282	70.78275381	67.34513456	83.3470995	84.03794492	86.87019099	83.37314941	Prpf38b	PRP38 pre-mRNA processing factor 38 (yeast) domain containing B [Source:MGI Symbol;Acc:MGI:1914171]
ENSMUSG00000027882	42.62859482	54.15448919	32.30225409	58.0284926	68.77882634	72.24904921	25.66940528	22.58060705	28.10140279	35.59063805	35.1511343	38.2746627	Stxbp3	syntaxin binding protein 3 [Source:MGI Symbol;Acc:MGI:107362]
ENSMUSG00000027883	44.69388744	46.18359276	34.06568163	51.38122756	57.49241117	58.86897546	31.67860004	29.29264415	35.39889459	26.32747147	26.19780939	28.55449735	Gpsm2	G-protein signalling modulator 2 (AGS3-like, C. elegans) [Source:MGI Symbol;Acc:MGI:1923373]
ENSMUSG00000027884	25.08372631	26.64314075	23.78688623	27.67743495	27.03634725	27.78129653	23.29226446	20.25209959	23.49791847	16.95095955	14.47801301	14.81029104	Clcc1	chloride channel CLIC-like 1 [Source:MGI Symbol;Acc:MGI:2385186]
ENSMUSG00000027886	0.685167004	0.631204021	0.280587581	1.086957203	0.503914376	0.939382757	0.06117015	0	0.127545069	0.551579136	0.171099724	0.242884743	1700013F07	RIKEN cDNA 1700013F07 gene [Source:MGI Symbol;Acc:MGI:1922754]

ENSMUSG00000027887	0.085036537	0.058754366	0.087059783	0.158709399	0.178689005	0.168404059	0.151837368	0.303266901	0.300764379	0.228189524	0.198196149	0.120578355	Sypl2	synaptophysin-like 2 [Source:MGI Symbol;Acc:MGI:1328311]
ENSMUSG00000027889	42.05352209	42.13954274	37.30910616	38.27317902	39.67678695	35.90950096	32.32186362	30.64940107	35.1488777	39.18563344	40.10912944	36.84644718	Ampd2	adenosine monophosphate deaminase 2 [Source:MGI Symbol;Acc:MGI:88016]
ENSMUSG00000027890	4.777307394	3.541942562	4.857472455	1.862632812	1.856463263	1.545238996	10.51661404	12.38256564	10.59855873	9.102477218	8.552470647	7.394593685	Gstm4	glutathione S-transferase, mu 4 [Source:MGI Symbol;Acc:MGI:95862]
ENSMUSG00000027893	74.38823319	94.11608561	68.64186782	87.04457314	99.96912882	122.4674421	67.10662643	63.55345315	78.19766535	79.60703101	81.4884506	77.25683402	Ahcyl1	S-adenosylhomocysteine hydrolase-like 1 [Source:MGI Symbol;Acc:MGI:2385184]
ENSMUSG00000027894	3.547334708	3.633238363	4.068241893	0.572939042	0.447961934	0.974257451	0.319743137	0.118264342	0.190483479	2.654342508	2.342364033	2.327573255	Slc6a17	solute carrier family 6 (neurotransmitter transporter), member 17 [Source:MGI Symbol;Acc:MGI:2442535]
ENSMUSG00000027895	0.067954024	0	0	0.101461725	0.014279317	0.01035187	0.412541073	0.323127083	0.17709669	0.218819771	0.42989301	0.277023629	Kcnc4	potassium voltage gated channel, Shaw-related subfamily, member 4 [Source:MGI Symbol;Acc:MGI:96670]
ENSMUSG00000027896	2.164212922	1.427352302	1.409994291	1.615686351	1.901767393	2.442691067	1.826769467	1.812621781	1.757987447	1.689458057	2.210918239	2.162085131	Slc16a4	solute carrier family 16 (monocarboxylic acid transporters), member 4 [Source:MGI Symbol;Acc:MGI:2385183]
ENSMUSG00000027900	29.57987925	29.38826891	24.86295991	36.26659483	35.64002784	35.35507558	12.29023223	12.48353374	11.43322783	24.12207244	20.9494932	22.52312664	Dram2	DNA-damage regulated autophagy modulator 2 [Source:MGI Symbol;Acc:MGI:1914421]
ENSMUSG00000027901	1.395518524	1.101100347	1.137685741	1.181693942	1.276150728	1.121993499	0.553727626	0.204808878	0.312696078	0.878404037	1.032558572	1.2596455	Dennd2d	DENN/MADD domain containing 2D [Source:MGI Symbol;Acc:MGI:2181193]
ENSMUSG00000027902	0.025796456	0	0	0	0.108413233	0	0	0	0	0	0	0	Chil6	chitinase-like 6 [Source:MGI Symbol;Acc:MGI:2682303]
ENSMUSG00000027905	16.91409279	18.0895057	16.82294149	20.75158728	22.5408422	23.96083862	41.03323348	40.93434581	42.38628688	47.41818079	47.65550659	50.6302432	Ddx20	DEAD (Asp-Glu-Ala-Asp) box polypeptide 20 [Source:MGI Symbol;Acc:MGI:1858415]
ENSMUSG00000027907	1746.990288	1400.767716	2118.818406	1343.844967	1172.850639	859.2437465	232.4436963	235.1512029	195.7784354	298.9407799	285.9041782	315.4359846	S100a11	S100 calcium binding protein A11 [Source:MGI Symbol;Acc:MGI:1338798]
ENSMUSG00000027908	0.062480863	0	0.021322483	0	0.021882051	0	0	0	0	0	0	0.018457359	Tchhl1	trichohyalin-like 1 [Source:MGI Symbol;Acc:MGI:1918575]
ENSMUSG00000027912	0.305708617	0	0.312982239	0.071320565	0	0	0	0	0	0	0	0	Lce1m	late cornified envelope 1M [Source:MGI Symbol;Acc:MGI:1913453]
ENSMUSG00000027913	50.62604765	37.85616644	54.37349048	33.40442129	28.32235903	23.48155638	1.173968376	0.940808184	1.291908114	2.548439199	3.648578147	4.402440609	Crct1	cysteine-rich C-terminal 1 [Source:MGI Symbol;Acc:MGI:1921425]
ENSMUSG00000027919	2.000243218	1.553444274	1.00010514	0.130227496	0	0.248019616	1.702712451	1.981525893	0.952520361	6.823812416	6.388956167	6.884535225	Lce1g	late cornified envelope 1G [Source:MGI Symbol;Acc:MGI:1913445]
ENSMUSG00000027923	0	0	0	0	0	0	0	0	0	0	0	0	Lce1b	late cornified envelope 1B [Source:MGI Symbol;Acc:MGI:1915970]
ENSMUSG00000027925	0.190391539	0	0	0.059223419	0	0	0	0	0	0	0	0	Sprr2j-ps	small proline-rich protein 2J, pseudogene [Source:MGI Symbol;Acc:MGI:1330345]
ENSMUSG00000027927	0.401666164	0.925078471	0.668237168	0.234267665	0.15825549	0.114728194	0	0	0.093463597	0.269460675	0.083586641	0.133487414	Lelp1	late cornified envelope-like proline-rich 1 [Source:MGI Symbol;Acc:MGI:1916582]
ENSMUSG00000027931	1.818945085	1.785568131	1.068488397	1.224361071	1.879763757	2.362091577	12.0861703	11.43188015	11.60120952	7.201490589	8.091684966	8.350656883	Npr1	natriuretic peptide receptor 1 [Source:MGI Symbol;Acc:MGI:97371]
ENSMUSG00000027932	21.50068736	20.38804896	18.04282459	20.84843462	17.31988504	16.66580634	15.95172579	15.63216343	17.48838186	12.41703767	13.47364503	12.61485505	Slc27a3	solute carrier family 27 (fatty acid transporter), member 3 [Source:MGI Symbol;Acc:MGI:1347358]
ENSMUSG00000027933	38.93629216	38.77269481	38.41313791	39.25115249	39.49378373	41.5856554	54.57005755	50.99022953	58.67554262	49.79342038	54.27147745	53.61865892	Ints3	integrator complex subunit 3 [Source:MGI Symbol;Acc:MGI:2140050]
ENSMUSG00000027935	42.36046564	38.82509991	54.59260595	40.59351595	42.68986408	31.39721246	10.80527402	11.28903729	7.534360926	9.665927628	9.64929522	9.054388328	Rab13	RAB13, member RAS oncogene family [Source:MGI Symbol;Acc:MGI:1927232]
ENSMUSG00000027936	31.19331008	33.38385121	28.86430119	33.11131015	34.91579271	33.14888689	23.93605286	20.05922245	24.64898759	18.23776184	21.34186371	21.57956861	Crtc2	CREB regulated transcription coactivator 2 [Source:MGI Symbol;Acc:MGI:1921593]
ENSMUSG00000027937	60.6798889	52.78997146	69.87598307	51.80323835	50.04630723	40.76293173	53.35142634	50.12718594	42.66829936	34.91833334	35.43357955	34.0067521	Jtb	jumping translocation breakpoint [Source:MGI Symbol;Acc:MGI:1346082]
ENSMUSG00000027938	1.42555504	1.60809787	1.667968905	2.144776707	2.170243815	1.839245242	2.675273813	3.199100132	1.868418834	1.561377946	1.937356348	2.372026526	Creb3l4	cAMP responsive element binding protein 3-like 4 [Source:MGI Symbol;Acc:MGI:1916603]
ENSMUSG00000027939	0.694982598	0.635027149	0.494969103	0.78248526	0.93654873	1.035695877	0.236046329	0.224504114	0.224995233	0.682458673	0.723128631	0.609215998	Nup210l	nucleoporin 210-like [Source:MGI Symbol;Acc:MGI:1924845]
ENSMUSG00000027940	540.1310179	534.1667319	504.1387424	609.9446724	583.6562349	569.3042114	376.1792961	365.7323488	389.8440067	398.9105236	403.7604271	404.0244615	Tpm3	tropomyosin 3, gamma [Source:MGI Symbol;Acc:MGI:1890149]
ENSMUSG00000027942	11.91729473	8.550390962	13.11305118	11.22495843	9.712572277	6.786682444	3.778510237	3.898481325	3.144497661	3.486830431	3.924713707	2.763730797	4933434E20	RIKEN cDNA 4933434E20 gene [Source:MGI Symbol;Acc:MGI:1914027]
ENSMUSG00000027944	92.98623863	86.67947733	115.4512219	105.8070215	95.17231694	73.55000995	129.4131577	140.0563567	112.1376243	110.0655869	111.4974682	116.5282139	Hax1	HCLS1 associated X-1 [Source:MGI Symbol;Acc:MGI:1346319]
ENSMUSG00000027947	2.643045155	3.812773611	2.536809601	3.887333858	4.557807425	4.86331236	0.987462448	0.611888316	0.835610739	1.62534727	1.548417751	1.705828486	Il6ra	interleukin 6 receptor, alpha [Source:MGI Symbol;Acc:MGI:105304]
ENSMUSG00000027950	0.306080595	0.333916616	0.272131085	0.578775326	0.35543792	0.858922293	0.158204191	0.287257678	0.329869462	0.439491941	0.382171827	0.299809434	Chrnb2	cholinergic receptor, nicotinic, beta polypeptide 2 (neuronal) [Source:MGI Symbol;Acc:MGI:87891]
ENSMUSG00000027951	18.62109035	22.8399672	14.88546511	24.32153515	26.7977689	32.96130075	15.3513074	12.43862832	19.42193139	33.51752043	32.84236925	31.87184491	Adar	adenosine deaminase, RNA-specific [Source:MGI Symbol;Acc:MGI:1889575]
ENSMUSG00000027952	10.72468842	9.561313568	14.92020936	11.50124742	10.35929305	10.70508212	127.2516171	141.7148786	104.9730524	20.26903958	18.75436394	21.11681456	Pmvk	phosphomevalonate kinase [Source:MGI Symbol;Acc:MGI:1915853]
ENSMUSG00000027953	60.28822957	51.72078653	70.8795756	58.74137993	52.87849371	42.56940209	28.14211781	28.49705548	23.89449377	20.32510584	19.03526079	18.33807102	Slc50a1	solute carrier family 50 (sugar transporter), member 1 [Source:MGI Symbol;Acc:MGI:107417]
ENSMUSG00000027954	10.43548044	9.613593729	12.14729823	9.284657998	8.891469921	7.469427959	3.420325877	5.409659077	3.539227344	2.250459163	2.165671541	2.128348169	Efna1	ephrin A1 [Source:MGI Symbol;Acc:MGI:103236]
ENSMUSG00000027955	52.10921414	56.96743865	49.00152726	41.4631954	44.20004808	48.73616562	2.95581258	2.162783463	3.159718394	22.81165779	23.27547721	23.87691774	Gask1b	golgi associated kinase 1B [Source:MGI Symbol;Acc:MGI:1915909]
ENSMUSG00000027956	7.329773952	6.252304811	5.072262247	6.301689345	4.706228724	6.461754857	1.635963817	1.411897164	1.610809722	8.407860162	7.400647772	6.315386423	Tmem144	transmembrane protein 144 [Source:MGI Symbol;Acc:MGI:1917902]
ENSMUSG00000027957	9.525854583	14.93720678	7.428500033	14.1662529	16.65233394	20.09975265	9.373007258	8.653669505	12.82780171	17.5851754	18.25429075	17.71033633	Slc35a3	solute carrier family 35 (UDP-N-acetylglucosamine (UDP-GlcNAc) transporter), member 3 [Source:MGI Symbol;Acc:MGI:1917648]
ENSMUSG00000027959	1.109955504	1.414146439	0.846228736	2.071584477	2.878246393	4.047285754	4.891463307	4.468461532	5.077581779	7.837011813	7.155511896	8.204225378	Sass6	SAS-6 centriolar assembly protein [Source:MGI Symbol;Acc:MGI:1920026]
ENSMUSG00000027961	0.115357876	0.132840524	0.103339729	0.121106334	0.106051685	0.109832509	0.090115274	0.228556824	0.161055569	0.116083008	0.08402082	0.17890773	Lrrc39	leucine rich repeat containing 39 [Source:MGI Symbol;Acc:MGI:1924557]
ENSMUSG00000027962	109.7940935	123.5263939	93.85892717	104.1323403	113.8715797	121.8080944	8.122402724	8.242045828	8.674853116	39.56733369	39.45142926	38.49875544	Vcam1	vascular cell adhesion molecule 1 [Source:MGI Symbol;Acc:MGI:98926]
ENSMUSG00000027963	23.91326813	27.03506666	21.43508417	27.72673067	27.90137716	31.36690705	3.848357474	3.177239691	4.346419545	11.24573853	13.07091699	12.05154412	Extl2	exostosin-like glycosyltransferase 2 [Source:MGI Symbol;Acc:MGI:1889574]
ENSMUSG00000027965	0.083740164	0.096431104	0	0	0.014663743	0	0	0	0	0.0374518	0	0.012368766	Olfm3	olfactomedin 3 [Source:MGI Symbol;Acc:MGI:2387329]
ENSMUSG00000027966	11.7217772	15.12249723	8.43200569	9.516404506	12.3496725	15.0656764	0.448704023	0.62637832	1.192118678	4.843625007	4.62893304	5.287456264	Col11a1	collagen, type XI, alpha 1 [Source:MGI Symbol;Acc:MGI:88446]
ENSMUSG00000027967	0	0	0.118685901	0.0540909	0.121800588	0	0	0	0.071933815	0	0.016083015	0	Neurog2	neurogenin 2 [Source:MGI Symbol;Acc:MGI:109619]
ENSMUSG00000027968	48.53643	41.92978771	44.73347056	50.12285506	47.30561147	47.1900847	68.01805347	64.92751544	68.09043889	59.88119167	58.09472586	59.62361386	Larp7	La ribonucleoprotein domain family, member 7 [Source:MGI Symbol;Acc:MGI:107634]
ENSMUSG00000027971	0.203084308	0.087698258	0.142942405	0.106602154	0.09335051	0.164353496	0	0	0	0	0	0	Ndst4	N-deacetylase/N-sulfotransferase (heparin glucosaminyl) 4 [Source:MGI Symbol;Acc:MGI:1932545]
ENSMUSG00000027973	0	0	0	0	0	0	0	0	0	0	0	0	1700006A11	RIKEN cDNA 1700006A11 gene [Source:MGI Symbol;Acc:MGI:1919074]
ENSMUSG00000027977	0.474847489	0.434644874	0.274235853	0.40903373	0.588450097	0.695543732	0.152181086	0.024123287	0.056662637	0.620773651	0.668905994	0.658208032	Ndst3	N-deacetylase/N-sulfotransferase (heparan glucosaminyl) 3 [Source:MGI Symbol;Acc:MGI:1932544]
ENSMUSG00000027978	1.835573462	1.713857189	1.693014996	1.682063445	1.754819429	1.624845758	0.014763587	0.131055642	0.01539168	0.576875745	0.481779647	0.351725485	Prss12	protease, serine 12 neurotrypsin (motopsin) [Source:MGI Symbol;Acc:MGI:1100881]
ENSMUSG00000027981	4.909578332	6.591789074	5.289781912	7.342464557	9.190330785	11.10430196	10.06786908	9.875943787	11.81320264	11.33352672	10.77897	10.92497269	Rnpc3	RNA-binding region (RNP1, RRM) containing 3 [Source:MGI Symbol;Acc:MGI:1914475]
ENSMUSG00000027983	4.58473722	3.059533344	4.610890646	4.610998019	3.40424084	3.344035991	0.462829567	0.353075546	0.452362404	0.579638048	0.512440237	0.502504392	Cyp2u1	cytochrome P450, family 2, subfamily u, polypeptide 1 [Source:MGI Symbol;Acc:MGI:1918769]
ENSMUSG00000027984	39.7353328	35.91892685	40.71254892	44.29928818	40.34475197	38.48876864	65.09722925	63.38478941	64.613595	44.10137374	44.32499969	41.50567063	Hadh	hydroxyacyl-Coenzyme A dehydrogenase [Source:MGI Symbol;Acc:MGI:96009]
ENSMUSG00000027985	0.387961775	0.223378964	0.162487808	0.213932464	0.018527999	0.188047659	1.857767533	1.642143313	1.329500522	3.706832827	3.522971359	3.547610395	Lef1	lymphoid enhancer binding factor 1 [Source:MGI Symbol;Acc:MGI:96770]
ENSMUSG00000027993	12.58856934	16.17712496	11.73864593	13.62022732	16.77986506	20.63074358	8.845690468	9.064596445	11.45567707	21.44078492	20.7004251	20.64599536	Trim2	tripartite motif-containing 2 [Source:MGI Symbol;Acc:MGI:1933163]
ENSMUSG00000027994	42.14923393	34.298643	50.91607462	42.20474107	39.2036375	34.02973024	2.011505769	2.014248231	1.585598592	9.683434707	8.416737022	9.788906568	Mcub	mitochondrial calcium uniporter dominant negative beta subunit [Source:MGI Symbol;Acc:MGI:1914065]
ENSMUSG00000027995	9.864859021	9.633714716	9.456471758	4.04998582	3.464871114	3.523152849	1.210819886	1.527402638	1.448360158	5.376056351	5.644663521	5.655410881	Tlr2	toll-like receptor 2 [Source:MGI Symbol;Acc:MGI:1346060]
ENSMUSG00000027996	24.99544318	26.39320933	22.22535638	3.793393886	4.67985607	5.039623274	8.845890499	6.04044676	9.056340797	9.360892324	8.567252627	7.243335665	Sfrp2	secreted frizzled-related protein 2 [Source:MGI Symbol;Acc:MGI:108078]
ENSMUSG00000027997	34.4650787	37.07082926	31.14504913	44.42040146	45.45824148	41.97409891	10.14642952	10.39463239	10.15988918	9.834762906	10.20824952	9.345840891	Casp6	caspase 6 [Source:MGI Symbol;Acc:MGI:1312921]
ENSMUSG00000027998	46.56860276	44.62050622	43.69981583	48.68271322	45.41119786	41.01042373	84.55865036	83.08373905	83.09375193	72.9457792	70.45503553	74.92531086	Plrg1	pleiotropic regulator 1 [Source:MGI Symbol;Acc:MGI:1858197]
ENSMUSG00000027999	28.75666266	20.18424399	26.97966667	26.29570048	23.97895245	19.26111114	32.24783012	33.61231979	29.14467957	20.38719011	18.79499406	16.80113127	Pla2g12a	phospholipase A2, group XIIA [Source:MGI Symbol;Acc:MGI:1913600]
ENSMUSG00000028001	0.036855236	0.021220349	0	0.01719635	0	0.014035983	0	0.091275905	0.051455013	0.049449207	0.030678266	0.032662017	Fga	fibrinogen alpha chain [Source:MGI Symbol;Acc:MGI:1316726]
ENSMUSG00000028003	0	0	0	0.022511761	0.008448574	0	0.007179005	0	0.029937695	0.043156008	0.046854444	0.021378941	Lrat	lecithin-retinol acyltransferase (phosphatidylcholine-retinol-O-acyltransferase) [Source:MGI Symbol;Acc:MGI:1891259]
ENSMUSG00000028004	0	0.020415952	0	0	0	0	0	0	0	0.063432989	0	0	Npy2r	neuropeptide Y receptor Y2 [Source:MGI Symbol;Acc:MGI:108418]
ENSMUSG00000028005	0.110103602	0.126789958	0.022544652	0.123296247	0.046272588	0.083863922	0.235915173	0.065443983	0.1229759	1.634852205	1.118131732	0.878188695	Gucy1b1	guanylate cyclase 1, soluble, beta 1 [Source:MGI Symbol;Acc:MGI:1860604]
ENSMUSG00000028007	18.38662255	19.96047546	14.20970772	15.58576012	14.2949455	15.8495998	3.741828664	3.713852614	4.116652873	8.797787712	8.870944196	8.884612155	Snx7	sorting nexin 7 [Source:MGI Symbol;Acc:MGI:1923811]

ENSMUSG00000028008	0	0	0	0.029524616	0	0	0	0	0	0	0	0	0.019435731	Asic5	acid-sensing (proton-gated) ion channel family member 5 [Source:MGI Symbol;Acc:MGI:1929259]
ENSMUSG00000028009	0	0	0	0	0	0.033408788	0	0	0	0	0	0	1700061I17F	RIKEN cDNA 1700061I17 gene [Source:MGI Symbol;Acc:MGI:1920612]
ENSMUSG00000028010	36.06898124	32.13397825	41.85991426	40.02858366	39.16179366	33.43663692	140.6951498	138.9483309	84.52052662	74.34223053	65.78496103	65.53166358	Gar1	GAR1 ribonucleoprotein [Source:MGI Symbol;Acc:MGI:1930948]
ENSMUSG00000028011	0.136338681	0.314002	0.111666033	0.229012231	0.114596498	0.31154012	0	0	0.02537969	0.170732424	0.02269764	0.120826694	Tdo2	tryptophan 2,3-dioxygenase [Source:MGI Symbol;Acc:MGI:1928486]
ENSMUSG00000028012	0.111472766	0.06418331	0.08559375	0.052012277	0.351359987	0.339627157	0.149280521	0.082822243	0.077815709	0.074782318	0	0.049394954	Rrh	retinal pigment epithelium derived rhodopsin homolog [Source:MGI Symbol;Acc:MGI:1097709]
ENSMUSG00000028013	27.63534322	21.11654027	30.43465785	27.69288477	26.37604168	23.23294421	34.03380829	37.25284169	32.76530224	26.17458938	25.88446463	26.48245542	Ppa2	pyrophosphatase (inorganic) 2 [Source:MGI Symbol;Acc:MGI:1922026]
ENSMUSG00000028015	18.1369244	20.2236166	14.80179148	19.52687499	19.65972962	20.42629342	0.641530629	0.626431964	0.775835267	5.013462553	3.899900269	4.024715391	Ctso	cathepsin O [Source:MGI Symbol;Acc:MGI:2139628]
ENSMUSG00000028016	6.933301261	7.17758283	5.563890975	8.496015818	9.374250908	9.068332533	9.328607141	10.05981106	9.777624883	9.872564291	9.024173705	9.14842147	Ints12	integrator complex subunit 12 [Source:MGI Symbol;Acc:MGI:1919043]
ENSMUSG00000028017	0.365829249	2.186598058	0.695561845	0.601489344	1.171389625	1.08804441	0.979812067	0.552237703	0.599926561	0.763526458	0.754039484	0.926305377	Egf	epidermal growth factor [Source:MGI Symbol;Acc:MGI:95290]
ENSMUSG00000028018	6.207667216	9.572065518	4.766523119	10.89143248	14.49614556	17.65004076	16.17067968	13.31004731	15.43396156	16.16342461	16.65201572	14.48824294	Gstcd	glutathione S-transferase, C-terminal domain containing [Source:MGI Symbol;Acc:MGI:1914803]
ENSMUSG00000028019	21.97416837	24.86723231	20.04549288	24.45724218	25.15834479	26.15693809	1.616369835	2.039445038	1.57641723	4.440416887	4.703471602	4.412392433	Pdgfc	platelet-derived growth factor, C polypeptide [Source:MGI Symbol;Acc:MGI:1859631]
ENSMUSG00000028020	1.432664098	2.097186045	1.814793603	1.903438001	1.68383127	1.923531685	0.672043158	0.938153533	0.497224229	0.890522924	0.889358082	1.226622781	Glrb	glycine receptor, beta subunit [Source:MGI Symbol;Acc:MGI:95751]
ENSMUSG00000028023	3.741607637	4.574255463	2.991040216	3.802503261	3.688826453	3.533113601	1.304137393	1.472483125	1.335766817	5.639094537	6.335683155	6.518251129	Pitx2	paired-like homeodomain transcription factor 2 [Source:MGI Symbol;Acc:MGI:109340]
ENSMUSG00000028024	0.222630934	0.466129028	0.165765758	0.906569584	0.170115967	0.385395351	0.072276301	0.160398298	0.018837795	0.108620789	0.101082433	0.125555189	Enpep	glutamyl aminopeptidase [Source:MGI Symbol;Acc:MGI:106645]
ENSMUSG00000028028	2.131442122	2.318105994	1.666923812	2.403407342	3.281374328	4.723878447	0.052858416	0.073315872	0.123991181	0.860584043	1.010314341	0.773939242	Alpk1	alpha-kinase 1 [Source:MGI Symbol;Acc:MGI:1918731]
ENSMUSG00000028029	100.5707094	86.20071554	108.8991006	101.8006597	88.30332392	74.65026699	141.511731	153.6249616	128.9942424	109.8194677	112.342207	111.1831071	Aimp1	aminoacyl tRNA synthetase complex-interacting multifunctional protein 1 [Source:MGI Symbol;Acc:MGI:102774]
ENSMUSG00000028030	6.556004275	6.545445146	5.852325458	7.283280318	8.087052194	8.298050759	3.719426701	3.465949588	4.158218134	4.458325852	4.310211935	4.636625389	Tbck	TBC1 domain containing kinase [Source:MGI Symbol;Acc:MGI:2445052]
ENSMUSG00000028031	34.57376741	40.46878118	39.2011522	8.041533213	9.127077497	10.30546128	1.565625632	1.852297295	1.372804924	20.33992933	19.12166793	18.52610754	Dkk2	dickkopf WNT signaling pathway inhibitor 2 [Source:MGI Symbol;Acc:MGI:1890663]
ENSMUSG00000028032	65.14694321	74.97035538	58.36812689	77.89049763	84.20446056	82.24734166	19.30375315	20.15856288	18.6588073	21.67602118	21.15961851	20.00351971	Papss1	3'-phosphoadenosine 5'-phosphosulfate synthase 1 [Source:MGI Symbol;Acc:MGI:1330587]
ENSMUSG00000028033	0.856224736	1.103716564	0.667260101	0.906345208	0.953308762	1.245938414	0.1483199	0.139258784	0.178419148	2.514806238	2.340276984	2.627512292	Kcnq5	potassium voltage-gated channel, subfamily Q, member 5 [Source:MGI Symbol;Acc:MGI:1924937]
ENSMUSG00000028034	33.63883438	42.22480462	29.24047912	41.16770647	43.35505809	57.70398996	106.7662528	96.7421413	119.4343641	101.6783542	97.63250156	101.9245962	Fubp1	far upstream element (FUSE) binding protein 1 [Source:MGI Symbol;Acc:MGI:1196294]
ENSMUSG00000028035	18.89853037	23.49460315	16.22390369	19.81230365	21.34455557	24.53900836	5.838123719	5.29044209	5.741559584	11.67897257	10.92287692	11.45532547	Dnajb4	DnaJ heat shock protein family (Hsp40) member B4 [Source:MGI Symbol;Acc:MGI:1914285]
ENSMUSG00000028036	1.26959278	1.16255529	1.221498584	0.585244433	0.369637868	0.629149347	0.040968561	0.030306326	0.028474335	0.506240581	0.432909266	0.406678685	Ptgfr	prostaglandin F receptor [Source:MGI Symbol;Acc:MGI:97796]
ENSMUSG00000028037	39.89597911	49.80001874	36.79186922	40.7015789	45.00261821	51.28189781	1.165263521	0.732699101	1.316074241	9.729010711	10.97321868	10.04410685	Ifi44	interferon-induced protein 44 [Source:MGI Symbol;Acc:MGI:2443016]
ENSMUSG00000028039	11.75483141	10.69534233	14.56027157	9.107323388	11.89291343	8.9534424	9.555128121	8.266395422	6.112054322	6.685093653	6.130544454	5.401625622	Efna3	ephrin A3 [Source:MGI Symbol;Acc:MGI:106644]
ENSMUSG00000028040	17.67962258	12.30216242	22.42559829	13.15848805	10.48923459	8.547071048	7.855945767	9.27691995	5.535248584	5.752735436	5.68948491	6.582039834	Efna4	ephrin A4 [Source:MGI Symbol;Acc:MGI:106643]
ENSMUSG00000028041	116.559163	103.0844065	135.7264107	92.79165046	80.51205315	62.75457733	30.04475081	32.95443688	27.90940271	30.33295309	31.99010247	29.5495678	Adam15	a disintegrin and metallopeptidase domain 15 (metargidin) [Source:MGI Symbol;Acc:MGI:1333882]
ENSMUSG00000028042	38.49917332	39.61477565	37.95780355	36.67149705	35.57747359	34.61396566	16.38648662	17.30673516	17.02339874	19.48513586	20.87971237	22.87974281	Zbtb7b	zinc finger and BTB domain containing 7B [Source:MGI Symbol;Acc:MGI:102755]
ENSMUSG00000028044	155.0464204	122.7442408	166.2336432	149.9958184	133.9760194	112.5174964	265.342723	290.2185375	230.0695939	161.0879322	162.2645985	154.270201	Cks1b	CDC28 protein kinase 1b [Source:MGI Symbol;Acc:MGI:1889208]
ENSMUSG00000028047	23.90821542	27.02371094	22.83235223	23.33957643	27.20441997	28.62328449	7.283636314	6.459407375	8.736930566	12.98898426	12.55950465	10.85922396	Thbs3	thrombospondin 3 [Source:MGI Symbol;Acc:MGI:98739]
ENSMUSG00000028048	87.12505869	85.78040258	88.57314136	70.59607912	70.1209874	60.71329213	19.77956316	21.22223056	23.71705072	34.39350186	34.62297963	34.80641726	Gba	glucosidase, beta, acid [Source:MGI Symbol;Acc:MGI:95665]
ENSMUSG00000028049	46.52639624	51.49073073	53.19924044	49.67075662	42.24166083	38.23100742	50.11614655	51.01742007	43.65788996	39.84503473	37.64104543	41.12079144	Scamp3	secretory carrier membrane protein 3 [Source:MGI Symbol;Acc:MGI:1346346]
ENSMUSG00000028051	0.481873711	0.42433709	0.203139494	0.079354699	0.059563002	0.151131847	3.163278497	3.453873035	3.667214797	2.763628679	2.52464142	2.687892491	Hcn3	hyperpolarization-activated, cyclic nucleotide-gated K+ 3 [Source:MGI Symbol;Acc:MGI:1298211]
ENSMUSG00000028053	6.921073351	8.588781523	6.124628225	9.026376149	10.95856167	15.18868245	3.225321783	2.578331444	3.88077845	7.511118324	7.566490095	7.722974678	Ash1l	ASH1 like histone lysine methyltransferase [Source:MGI Symbol;Acc:MGI:2183158]
ENSMUSG00000028057	51.5223323	41.7809888	58.76019076	49.67810458	45.9428158	33.49286593	7.364667393	7.583314904	7.808120524	11.56827584	12.33660049	12.66966533	Rit1	Ras-like without CAAX 1 [Source:MGI Symbol;Acc:MGI:108053]
ENSMUSG00000028059	96.85201931	106.0278119	86.81036849	135.6064575	139.5295513	153.4525091	43.19978302	40.64732715	48.77901945	43.16893525	44.76057235	45.10078563	Arhgef2	rho/rac guanine nucleotide exchange factor (GEF) 2 [Source:MGI Symbol;Acc:MGI:103264]
ENSMUSG00000028060	49.01858791	54.88096656	44.36987596	54.29516304	60.20253597	68.47461771	46.14895947	43.2168883	55.17586932	51.65883451	52.34468491	51.60526203	Khdc4	KH domain containing 4, pre-mRNA splicing factor [Source:MGI Symbol;Acc:MGI:1921450]
ENSMUSG00000028062	121.7744284	98.48514344	147.4385931	106.7442897	95.85763784	68.73843266	157.5348971	172.6158222	117.099466	81.13127071	78.60694795	78.15558165	Lamtor2	late endosomal/lysosomal adaptor, MAPK and MTOR activator 2 [Source:MGI Symbol;Acc:MGI:1932697]
ENSMUSG00000028063	360.4015656	321.7892498	398.9348807	354.6417977	317.80883	286.7415266	90.89359279	95.06883795	84.07928878	124.620732	125.4855225	126.3709134	Lmna	lamin A [Source:MGI Symbol;Acc:MGI:96794]
ENSMUSG00000028064	2.040987402	1.738579496	1.688812711	1.174078299	1.850632915	1.810131094	24.41177861	27.00467934	24.2272645	7.477519133	6.888755778	7.259870743	Sema4a	sema domain, immunoglobulin domain (Ig), transmembrane domain (TM) and short cytoplasmic domain, (semaphorin) 4A [Source:N
ENSMUSG00000028066	47.14541105	35.17403887	47.17941611	45.52379894	43.25487249	29.09874466	138.9969566	156.6894587	125.5038226	51.35688517	52.65670274	48.88230375	Pmf1	polyamine-modulated factor 1 [Source:MGI Symbol;Acc:MGI:1914287]
ENSMUSG00000028068	14.02631034	15.714632	12.52683786	21.72237326	23.10298993	26.37833376	22.63472959	19.79441162	25.70620192	16.4289743	18.28105971	17.45554149	Iqgap3	IQ motif containing GTPase activating protein 3 [Source:MGI Symbol;Acc:MGI:3028642]
ENSMUSG00000028069	18.60055014	15.2512189	22.0562566	18.53871241	17.93490058	14.16770249	30.32198468	33.06273002	29.17397153	31.22211226	31.87260944	26.63043481	Gpatch4	G patch domain containing 4 [Source:MGI Symbol;Acc:MGI:1913864]
ENSMUSG00000028070	92.6439358	79.39927889	105.7156973	91.53931621	82.30104651	65.36756759	70.56825616	72.01327133	53.76642699	49.42397444	45.63890411	45.69229108	Naxe	NAD(P)HX epimerase [Source:MGI Symbol;Acc:MGI:2180167]
ENSMUSG00000028071	0.110653716	0	0.048551341	0.029502923	0.016608493	0.084282933	0	0	0	0.014139591	0	0.028018299	Sh2d2a	SH2 domain containing 2A [Source:MGI Symbol;Acc:MGI:1351596]
ENSMUSG00000028072	1.030821579	0.459500918	0.74895634	0.651640676	0.768611286	0.430570596	0.326555947	0.263529198	0.154749426	0.743585131	0.705819416	0.898804941	Ntrk1	neurotrophic tyrosine kinase, receptor, type 1 [Source:MGI Symbol;Acc:MGI:97383]
ENSMUSG00000028073	36.42912823	38.9713146	38.00885865	48.24606701	46.54975978	47.96752616	1.27309343	1.774023719	1.743951492	6.333087337	6.68029585	6.451000061	Pear1	platelet endothelial aggregation receptor 1 [Source:MGI Symbol;Acc:MGI:1920432]
ENSMUSG00000028076	9.542179081	9.640301473	8.534442536	8.87150211	10.80825418	8.348864284	7.568961355	8.222958254	9.343701468	8.884279129	9.448804743	10.1226672	Cd1d1	CD1d1 antigen [Source:MGI Symbol;Acc:MGI:107674]
ENSMUSG00000028078	9.930781076	11.48655552	9.986580448	13.31137828	13.25843136	17.98151286	17.19422113	16.89607688	19.25203323	16.08373876	17.91455192	17.06802255	Dclk2	doublecortin-like kinase 2 [Source:MGI Symbol;Acc:MGI:1918012]
ENSMUSG00000028080	1.874011644	2.82921617	2.016795405	2.788435961	2.961175859	4.591285127	5.309048762	4.136870679	6.008746156	6.922818368	7.007382531	7.022417633	Lrba	LPS-responsive beige-like anchor [Source:MGI Symbol;Acc:MGI:1933162]
ENSMUSG00000028081	1279.183653	1021.977778	1444.165324	1212.495295	1066.860786	823.7114399	561.2815168	663.1220667	652.6331028	454.8434593	494.5808261	501.9336277	Rps3a1	ribosomal protein S3A1 [Source:MGI Symbol;Acc:MGI:1202063]
ENSMUSG00000028082	9.131149151	12.05236269	7.477260984	11.01272588	13.02917687	17.16734414	12.20581476	10.75017231	15.77512622	13.8702278	13.89411894	12.77423836	Sh3d19	SH3 domain protein D19 [Source:MGI Symbol;Acc:MGI:1350923]
ENSMUSG00000028085	7.299880443	6.526543154	6.684429956	7.637199947	6.002368927	5.629257496	12.83193756	12.59910462	12.36501728	8.780447544	8.888170891	8.739627787	Gatb	glutamyl-tRNA(Gln) amidotransferase, subunit B [Source:MGI Symbol;Acc:MGI:2442496]
ENSMUSG00000028086	5.667435098	5.987761159	4.774204865	4.929327202	4.957306008	6.318009733	11.29848649	11.96467208	11.90719658	14.01463993	13.76339013	13.26361902	Fbxw7	F-box and WD-40 domain protein 7 [Source:MGI Symbol;Acc:MGI:1354695]
ENSMUSG00000028088	0.515311231	0.784145457	0.263785929	0.549577113	0.522080665	0.658844726	0.722948494	0.510489194	0.68518651	1.03710089	1.011083739	0.897052933	Fmo5	flavin containing monooxygenase 5 [Source:MGI Symbol;Acc:MGI:1310004]
ENSMUSG00000028089	6.864544802	7.23653391	5.798505491	7.003513576	8.137497303	7.652352725	24.44256761	23.94977861	26.45106752	21.94709152	22.02321367	21.60273391	Chd1l	chromodomain helicase DNA binding protein 1-like [Source:MGI Symbol;Acc:MGI:1915308]
ENSMUSG00000028093	39.70137799	30.35598578	35.40556317	36.89940417	34.18353886	32.45724572	182.3920135	191.3250874	186.2211105	92.91481547	91.25980094	87.9466694	Acp6	acid phosphatase 6, lysophosphatidic [Source:MGI Symbol;Acc:MGI:1931010]
ENSMUSG00000028096	8.872540901	10.19157996	9.652792808	10.29257106	10.63037977	11.26342311	8.33810786	10.11124759	8.754930798	8.891018588	8.53311607	8.611927312	Gpr89	G protein-coupled receptor 89 [Source:MGI Symbol;Acc:MGI:1914799]
ENSMUSG00000028098	28.28381204	29.28654022	21.95491995	29.14155961	32.59091053	31.60856516	30.44625557	30.5684285	33.10096898	30.5456759	30.70293582	30.20224671	Rnf115	ring finger protein 115 [Source:MGI Symbol;Acc:MGI:1915095]
ENSMUSG00000028099	33.98372406	30.74092398	35.72128043	39.47059556	38.96526486	34.13403102	34.70403143	35.17357365	35.36312835	43.148414	43.56501589	42.02401741	Polr3c	polymerase (RNA) III (DNA directed) polypeptide C [Source:MGI Symbol;Acc:MGI:1921664]
ENSMUSG00000028100	2.235246121	0.643500203	1.251484381	1.759973866	0.990766717	1.170500737	11.03803437	11.27924217	10.36986959	3.248984174	2.703708944	3.3737716	Nudt17	nudix (nucleoside diphosphate linked moiety X)-type motif 17 [Source:MGI Symbol;Acc:MGI:1925623]
ENSMUSG00000028101	33.76081353	38.54587737	32.53089245	42.51702703	42.06339185	44.590206	19.83696812	20.72842226	19.93081521	21.54811228	19.81308095	20.02296333	Pias3	protein inhibitor of activated STAT 3 [Source:MGI Symbol;Acc:MGI:1913126]
ENSMUSG00000028102	13.50552992	11.45762113	15.59696804	12.84667716	10.45378097	9.367555557	12.84378366	12.9590333	9.625156204	5.923470185	5.756768462	6.013376934	Pex11b	peroxisomal biogenesis factor 11 beta [Source:MGI Symbol;Acc:MGI:1338882]
ENSMUSG00000028104	28.04582974	22.34947173	28.27641339	24.18164836	21.62380531	18.11302751	53.12388741	55.83088106	52.00930706	12.01854712	10.69624162	10.96261944	Polr3gl	polymerase (RNA) III (DNA directed) polypeptide G like [Source:MGI Symbol;Acc:MGI:1917120]
ENSMUSG00000028106	7.619748314	8.468752737	6.772723199	9.179169068	10.29832634	12.55736557	11.21421015	10.94524718	13.14196735	17.60718612	16.81769039	17.09688321	Rprd2	regulation of nuclear pre-mRNA domain containing 2 [Source:MGI Symbol;Acc:MGI:1922387]
ENSMUSG00000028107	30.52204729	28.03795255	29.10884524	30.13726813	28.27599014	27.27550945	71.11977398	71.19990749	67.98630799	55.45212521	54.60821633	56.23130316	Tars2	threonyl-tRNA synthetase 2, mitochondrial (putative) [Source:MGI Symbol;Acc:MGI:1919057]
ENSMUSG00000028108	261.4048008	229.4380494	275.5334898	196.4502616	165.8267544	147.2094058	23.01683323	22.33630015	23.03453287	76.85540675	73.05446209	75.98717646	Ecm1	extracellular matrix protein 1 [Source:MGI Symbol;Acc:MGI:103060]

ENSMUSG00000028109	5.244469431	5.11279628	4.087951196	7.090814992	7.075540129	5.92383614	12.50344973	14.55300756	14.1725183	11.35448315	10.91372937	13.15109871	Hormad1	HORMA domain containing 1 [Source:MGI Symbol;Acc:MGI:1915231]
ENSMUSG00000028111	53.27336589	49.1562655	52.55969534	9.188560404	9.71739647	9.602441232	2.413631704	3.072895105	2.569290805	11.88748697	11.03879892	11.2229779	Ctsk	cathepsin K [Source:MGI Symbol;Acc:MGI:107823]
ENSMUSG00000028114	8.971957871	8.991169117	8.18258557	11.40618423	10.69428052	12.0239891	19.12504612	18.26822044	18.76271698	14.84408865	13.87298311	13.46163247	Mettl14	methyltransferase like 14 [Source:MGI Symbol;Acc:MGI:2442926]
ENSMUSG00000028115	0.286141232	0.370694631	0.476042604	0.534044593	0.413376145	0.299679324	1.149571693	1.098421678	0.798985567	2.655445726	2.947523418	2.250570435	Bnipl	BCL2/adenovirus E1B 19kD interacting protein like [Source:MGI Symbol;Acc:MGI:2384749]
ENSMUSG00000028116	0.526572403	0.9528753	0.500593752	0.210595163	0.51373089	0.143243097	0	0	0.035008	0	0.062616915	0.066665917	Myoz2	myozenin 2 [Source:MGI Symbol;Acc:MGI:1913063]
ENSMUSG00000028121	23.85274869	26.39050289	22.3852467	30.11510838	35.46232218	32.47715451	54.45583933	54.67228292	60.40021493	23.46232008	23.29394619	21.79516563	Bcar3	breast cancer anti-estrogen resistance 3 [Source:MGI Symbol;Acc:MGI:1352501]
ENSMUSG00000028124	53.04065674	61.18198501	48.10969429	55.47519964	50.93762739	58.92060774	63.36854737	62.02001802	70.80712177	52.26793771	53.79972501	49.67312584	Gclm	glutamate-cysteine ligase, modifier subunit [Source:MGI Symbol;Acc:MGI:104995]
ENSMUSG00000028125	1.09272154	1.32955097	0.823209275	1.135148631	1.213064604	1.272015176	0.349727948	0.265517978	0.441365829	1.641317205	1.098361299	0.968396717	Abca4	ATP-binding cassette, sub-family A (ABC1), member 4 [Source:MGI Symbol;Acc:MGI:109424]
ENSMUSG00000028126	33.00225046	44.08957335	26.97624729	40.97543492	48.0633733	54.69949786	20.1611059	16.22604133	19.29196933	32.57060917	30.37258561	31.10754812	Pip5k1a	phosphatidylinositol-4-phosphate 5-kinase, type 1 alpha [Source:MGI Symbol;Acc:MGI:107929]
ENSMUSG00000028127	29.14571157	32.67463342	25.68329304	31.61139364	32.00834238	38.66406869	26.12934885	25.39454983	28.26109938	30.10049445	29.90371207	30.23651546	Abcd3	ATP-binding cassette, sub-family D (ALD), member 3 [Source:MGI Symbol;Acc:MGI:1349216]
ENSMUSG00000028128	69.37781443	86.23687475	60.13055406	46.34438862	49.42198565	48.19037181	11.47760884	9.839590279	13.01248564	47.80822304	46.4246609	47.43361315	F3	coagulation factor III [Source:MGI Symbol;Acc:MGI:88381]
ENSMUSG00000028132	0.466079807	0.193217349	0.171780872	0.069590083	0.137113617	0.142001823	0.049932653	0	0.034704637	0.450249173	0.310371538	0.54522784	Tlcd4	TLC domain containing 4 [Source:MGI Symbol;Acc:MGI:1923195]
ENSMUSG00000028133	4.724696703	4.352584902	4.56070355	5.574247511	7.197873431	4.832561474	7.381677841	7.956824904	8.072652503	4.618555965	4.859858398	4.097417994	Rwdd3	RWD domain containing 3 [Source:MGI Symbol;Acc:MGI:1920420]
ENSMUSG00000028134	23.71138054	27.67844773	19.68613237	28.04413565	29.74639179	39.31085409	18.56235238	18.14300988	21.1362897	22.2380779	23.51786615	21.84997143	Ptbp2	polypyrimidine tract binding protein 2 [Source:MGI Symbol;Acc:MGI:1860489]
ENSMUSG00000028136	35.24764467	42.86620645	31.95094036	44.2005675	50.70300526	66.01762951	40.78731294	38.32956661	51.14973156	49.17484241	48.92967426	46.95019688	Snx27	sorting nexin family member 27 [Source:MGI Symbol;Acc:MGI:1923992]
ENSMUSG00000028137	0.376823101	0.542413964	0.385788726	0.175822563	0.420657587	0.717548345	0.126157156	0.233310606	0.394572921	0.273860735	0.411688224	0.20871869	Celf3	CUGBP, Elav-like family member 3 [Source:MGI Symbol;Acc:MGI:1926034]
ENSMUSG00000028138	105.3891093	91.75690809	102.0322721	96.94742232	92.35106553	77.00076956	312.0978596	324.8278743	305.6048567	244.3467405	246.6880203	242.1530096	Adh5	alcohol dehydrogenase 5 (class III), chi polypeptide [Source:MGI Symbol;Acc:MGI:87929]
ENSMUSG00000028139	4.454714034	3.497612868	7.566621799	4.629459797	5.744115743	5.629420015	0.090387892	0.300888337	0.376933191	0.633919446	1.601225021	0.628071371	Riiad1	regulatory subunit of type II PKA R-subunit (RIIa) domain containing 1 [Source:MGI Symbol;Acc:MGI:1913603]
ENSMUSG00000028140	25.07835059	24.06584155	22.1588126	27.15197522	24.91904295	19.71870669	35.98499884	35.17916346	31.12253341	23.47534093	25.86474668	22.94291479	Mrpl9	mitochondrial ribosomal protein L9 [Source:MGI Symbol;Acc:MGI:2137211]
ENSMUSG00000028141	0.078878988	0.272499628	0.484534374	0.110412863	0.082875011	0.210282442	0	0.117211314	0	0.105833088	0.0656588	0	Oaz3	ornithine decarboxylase antizyme 3 [Source:MGI Symbol;Acc:MGI:1858170]
ENSMUSG00000028145	4.458373438	2.925212368	3.715250017	5.079656835	4.439130055	5.528148059	4.142324626	2.696213258	3.45001727	4.544362294	4.768391254	4.203808168	Them4	thioesterase superfamily member 4 [Source:MGI Symbol;Acc:MGI:1923028]
ENSMUSG00000028148	0	0	0.03349981	0.030534964	0	0	0.584256336	1.102113172	0.791846342	1.81464176	1.98831324	1.53691555	Them5	thioesterase superfamily member 5 [Source:MGI Symbol;Acc:MGI:1913448]
ENSMUSG00000028149	40.48419463	47.76142441	39.8385567	46.00191917	49.17878805	53.05116471	16.35136615	15.32677219	17.56938865	25.93754255	25.78815034	25.46614852	Rap1gds1	RAP1, GTP-GDP dissociation stimulator 1 [Source:MGI Symbol;Acc:MGI:2385189]
ENSMUSG00000028150	0.603021303	0.985614235	0.936010741	1.179916844	0.572257194	0.755639716	0.138932564	0.115621648	0.126737821	0.139196985	0.145728698	0	Rorc	RAR-related orphan receptor gamma [Source:MGI Symbol;Acc:MGI:104856]
ENSMUSG00000028152	31.31989086	30.53925076	30.65043887	34.99573533	32.62639764	31.48152405	9.630766232	10.51837819	10.04049151	15.09161949	15.43656887	15.23167855	Tspan5	tetraspanin 5 [Source:MGI Symbol;Acc:MGI:1928096]
ENSMUSG00000028156	110.1048916	119.438281	94.97173341	131.5853249	135.2248814	137.0529672	152.4303502	140.499901	155.7527588	135.1925844	130.6133437	131.2197639	Eif4e	eukaryotic translation initiation factor 4E [Source:MGI Symbol;Acc:MGI:95305]
ENSMUSG00000028158	2.896148219	3.269348014	2.351595584	2.955594191	4.017184787	4.009818838	0.980750585	1.215458522	1.327889639	1.761054164	1.781343276	2.111470518	Mttp	microsomal triglyceride transfer protein [Source:MGI Symbol;Acc:MGI:106926]
ENSMUSG00000028159	5.02104954	5.635616991	4.750094925	4.65590548	4.040034358	3.461362593	10.97972565	9.759241349	10.11581228	10.09101039	10.60750891	10.33587594	Dapp1	dual adaptor for phosphotyrosine and 3-phosphoinositides 1 [Source:MGI Symbol;Acc:MGI:1347063]
ENSMUSG00000028161	98.91216086	79.93839556	96.49777016	100.4742323	95.46243548	91.9852586	18.44952405	18.76594535	19.46018524	35.27701991	32.84896077	35.14503899	Ppp3ca	protein phosphatase 3, catalytic subunit, alpha isoform [Source:MGI Symbol;Acc:MGI:107164]
ENSMUSG00000028163	42.94901149	48.85395537	39.38176782	51.48604417	57.31619822	59.63975828	28.21157366	26.28095302	29.30822651	28.46431794	29.10865388	28.66659071	Nfkb1	nuclear factor of kappa light polypeptide gene enhancer in B cells 1, p105 [Source:MGI Symbol;Acc:MGI:97312]
ENSMUSG00000028164	24.1876115	30.05222101	23.03619888	23.22486662	25.9145316	26.90766487	110.8120791	107.2582351	112.6234276	98.52577782	92.56225068	93.18869607	Manba	mannosidase, beta A, lysosomal [Source:MGI Symbol;Acc:MGI:88175]
ENSMUSG00000028165	19.5725956	22.42649279	19.65869124	22.65348137	22.55290258	24.12346281	10.45302152	12.10148783	11.64240641	11.95657912	13.49833266	12.03650869	Cisd2	CDGSH iron sulfur domain 2 [Source:MGI Symbol;Acc:MGI:1914256]
ENSMUSG00000028167	11.3833451	9.775292539	11.99611729	7.506051161	6.959624688	5.51180166	21.94069608	23.32565793	19.27333036	6.14911235	5.583355104	6.191047268	Bdh2	3-hydroxybutyrate dehydrogenase, type 2 [Source:MGI Symbol;Acc:MGI:1917022]
ENSMUSG00000028172	0.011437663	0.013171057	0	0	0	0.008711861	0	0	0.010645707	0.010230719	0.028562102	0.06081803	Tacr3	tachykinin receptor 3 [Source:MGI Symbol;Acc:MGI:892968]
ENSMUSG00000028173	98.04617114	122.7490708	85.13221196	106.5296351	120.0157523	134.1810174	4.735858728	3.603934645	4.954018327	37.23762844	38.6959496	38.05349423	Wls	wntless WNT ligand secretion mediator [Source:MGI Symbol;Acc:MGI:1915401]
ENSMUSG00000028174	0.094360719	0.108661218	0	0	0	0.047915235	0	0	0	0.056268957	0.052363853	0	Rpe65	retinal pigment epithelium 65 [Source:MGI Symbol;Acc:MGI:98001]
ENSMUSG00000028175	3.37878922	5.573868083	3.137395467	5.323488844	6.918292691	7.098244791	7.702613179	6.850026657	9.37600662	10.7395202	9.876457628	11.3368095	Depdc1a	DEP domain containing 1a [Source:MGI Symbol;Acc:MGI:1923381]
ENSMUSG00000028176	0	0.06923086	0.026378593	0.032058665	0	0.111209252	0.007667636	0	0.03197537	0.015364458	0.021447231	0	Lrrc7	leucine rich repeat containing 7 [Source:MGI Symbol;Acc:MGI:2676665]
ENSMUSG00000028177	0	0	0	0	0.099468247	0	0.126781697	0.046893122	0	0.254045983	0.078805007	0.08390078	1810013D15	RIKEN cDNA 1810013D15 gene [Source:MGI Symbol;Acc:MGI:1916292]
ENSMUSG00000028179	4.644344718	4.08980129	4.055603986	9.772804129	9.854935005	8.809101843	49.6344305	51.55697769	53.90742935	52.94640877	53.47895376	50.8842252	Cth	cystathionase (cystathionine gamma-lyase) [Source:MGI Symbol;Acc:MGI:1339968]
ENSMUSG00000028180	51.33361875	55.76597599	47.43309844	56.24439359	62.22376537	71.40699993	60.46455662	59.62917298	68.44797542	72.00574959	68.48989838	66.66248595	Zranb2	zinc finger, RAN-binding domain containing 2 [Source:MGI Symbol;Acc:MGI:1858211]
ENSMUSG00000028182	0.920161145	0.782095206	0.785045192	1.267574148	0.966776665	1.535239256	0.078237992	0.151925119	0.061174875	0.50951483	0.237077078	0.310655028	Lrriq3	leucine-rich repeats and IQ motif containing 3 [Source:MGI Symbol;Acc:MGI:1921685]
ENSMUSG00000028184	17.73051538	23.93981129	17.71571659	18.20609403	21.82956182	26.74085343	8.746967622	8.353748686	12.66918784	12.85112216	15.20518163	13.99610435	Adgrl2	adhesion G protein-coupled receptor L2 [Source:MGI Symbol;Acc:MGI:2139714]
ENSMUSG00000028185	0	0	0	0.015290738	0	0.024961173	0	0	0	0.029312998	0	0	Dnase2b	deoxyribonuclease II beta [Source:MGI Symbol;Acc:MGI:1913283]
ENSMUSG00000028186	1.618108905	1.597144786	1.318524756	2.003051465	1.838864829	1.484011679	0.088445558	0.098140862	0	0.561221305	0.412320132	0.204858275	Uox	urate oxidase [Source:MGI Symbol;Acc:MGI:98907]
ENSMUSG00000028187	30.07947582	31.37618231	24.71897425	38.42272448	33.83537933	33.00518536	64.75732845	66.87848518	57.65696814	56.34445234	53.3323381	53.16491963	Rpf1	ribosome production factor 1 homolog [Source:MGI Symbol;Acc:MGI:1917535]
ENSMUSG00000028188	2.390471546	2.907835424	2.998862103	3.770279609	4.138789708	5.898297924	1.743397133	1.701031481	1.974253948	2.349030811	1.961800691	1.90962903	Spata1	spermatogenesis associated 1 [Source:MGI Symbol;Acc:MGI:1918201]
ENSMUSG00000028189	5.81814384	4.982514408	5.070627002	7.43964737	8.666378528	9.396060688	3.205347259	3.064245791	3.992919258	4.133709696	4.061391528	4.226111388	Ctbs	chitobiase [Source:MGI Symbol;Acc:MGI:1921495]
ENSMUSG00000028191	35.84410499	38.05068288	34.78508002	45.96229922	44.55431013	43.54065189	23.54906967	22.17366279	24.35780222	33.10568295	34.21925716	32.24862283	Bcl10	B cell leukemia/lymphoma 10 [Source:MGI Symbol;Acc:MGI:1337994]
ENSMUSG00000028194	37.31195623	40.23118212	31.95172187	31.32259626	34.51496836	37.65776159	34.51819473	30.19012014	38.30553945	52.01403355	50.735215	51.46572908	Ddah1	dimethylarginine dimethylaminohydrolase 1 [Source:MGI Symbol;Acc:MGI:1916469]
ENSMUSG00000028195	289.3074404	285.7134526	265.6008131	301.9764277	293.0367817	277.0776415	23.68820868	21.99907544	25.37041347	122.0405947	123.8955217	119.0449224	Ccn1	cellular communication network factor 1 [Source:MGI Symbol;Acc:MGI:88613]
ENSMUSG00000028197	4.881676091	5.079427655	2.588158946	2.277749958	2.985800604	2.562960285	0.52921598	0.449056742	0.924960145	9.450448793	10.8263327	9.687735511	Col24a1	collagen, type XXIV, alpha 1 [Source:MGI Symbol;Acc:MGI:1918605]
ENSMUSG00000028199	20.77032123	24.08942565	21.93473039	31.62850018	29.70117734	27.01703499	27.15074748	26.76643825	27.58572263	19.27060004	19.32030628	18.7236254	Cryz	crystallin, zeta [Source:MGI Symbol;Acc:MGI:88527]
ENSMUSG00000028207	21.47021149	31.21436944	16.08457109	30.73557247	38.62045043	52.03865168	13.0984746	11.89964312	16.29507025	37.07007158	35.37704483	34.66769599	Asph	aspartate-beta-hydroxylase [Source:MGI Symbol;Acc:MGI:1914186]
ENSMUSG00000028211	18.99088727	24.62341537	14.50468104	20.34655459	25.36581576	36.33771989	42.85004492	38.44658774	51.02168937	84.89923431	82.39850523	87.2258165	Trp53inp1	transformation related protein 53 inducible nuclear protein 1 [Source:MGI Symbol;Acc:MGI:1926609]
ENSMUSG00000028212	5.735266583	5.702382487	3.694889646	7.753955083	9.082815585	10.29249153	16.28510069	15.07702285	17.86327565	16.76653932	14.39195647	15.76887346	Ccne2	cyclin E2 [Source:MGI Symbol;Acc:MGI:1329034]
ENSMUSG00000028214	8.175412687	10.70269537	7.290647408	7.930311872	7.956664029	9.684723098	1.325312465	1.534530517	1.521867758	1.616494588	1.934111276	2.001977496	Gem	GTP binding protein (gene overexpressed in skeletal muscle) [Source:MGI Symbol;Acc:MGI:99844]
ENSMUSG00000028217	0.535465626	1.101100347	0.652625929	0.678147626	0.428641833	0.611781719	0	0	0.189862644	0.022807684	0.010612408	0.022597277	Cdh17	cadherin 17 [Source:MGI Symbol;Acc:MGI:1095414]
ENSMUSG00000028218	35.09866542	32.0183884	32.13142355	30.16194369	33.32619424	30.4803489	24.70379807	23.40112634	23.13881771	20.97983237	21.75192827	22.56542805	Fam92a	family with sequence similarity 92, member A [Source:MGI Symbol;Acc:MGI:1915349]
ENSMUSG00000028221	16.66687045	17.75224202	14.63385994	17.73345131	17.29860835	18.81808881	12.99108575	10.98904924	13.81772778	15.61568195	15.31290102	14.62386298	Pip4p2	phosphatidylinositol-4,5-bisphosphate 4-phosphatase 2 [Source:MGI Symbol;Acc:MGI:1919769]
ENSMUSG00000028222	0.045629361	0.788168329	0.023357503	0.276773707	0.023970476	0.017377529	0.142578992	0.38422026	0.063704907	0.204071932	0.113945521	0.101094657	Calb1	calbindin 1 [Source:MGI Symbol;Acc:MGI:88248]
ENSMUSG00000028223	33.89054518	34.00529351	36.79774402	35.54570169	32.57559154	29.72129889	27.5373246	26.2361775	25.8737812	17.58056884	19.32296412	17.95826808	Decr1	2,4-dienoyl CoA reductase 1, mitochondrial [Source:MGI Symbol;Acc:MGI:1914710]
ENSMUSG00000028224	9.035380985	8.832438156	8.284207573	8.412931194	8.284612357	10.29254125	21.85147322	21.97926351	26.350585	14.13440482	13.4710227	13.57695113	Nbn	nibrin [Source:MGI Symbol;Acc:MGI:1351625]
ENSMUSG00000028226	5.784742762	7.298787571	5.703029901	6.781106008	6.47173022	9.01625547	0.175337508	0.176870737	0.049853716	1.405369726	1.114633054	1.867088279	Mmp16	matrix metallopeptidase 16 [Source:MGI Symbol;Acc:MGI:1276107]
ENSMUSG00000028228	31.11265373	41.73288081	25.62703478	43.97250609	54.25688932	72.77134529	16.21406178	13.69302441	21.44725379	28.87332542	26.76004906	27.456274	Cpne3	copine III [Source:MGI Symbol;Acc:MGI:1917818]
ENSMUSG00000028229	8.571816867	9.246641892	9.469499452	8.979202917	7.083823111	10.29671987	4.325452073	4.161893791	4.414867097	3.363908872	3.66629434	3.573083195	Rmdn1	regulator of microtubule dynamics 1 [Source:MGI Symbol;Acc:MGI:1913552]
ENSMUSG00000028232	10.48257824	11.03655104	8.994426955	12.73234796	12.4471472	14.904195	8.37818163	8.933955705	9.818701495	12.91862135	11.80045549	11.32485336	Tmem68	transmembrane protein 68 [Source:MGI Symbol;Acc:MGI:1919348]

ENSMUSG00000028233	17.71387119	21.46047018	14.71664234	24.21673164	27.93359508	35.68080739	38.38646172	32.79638393	46.41320738	53.76367808	51.40899644	54.28231665	Tgs1	trimethylguanosine synthase 1 [Source:MGI Symbol;Acc:MGI:2151797]
ENSMUSG00000028234	523.0112695	380.9738383	729.1198061	504.9801756	417.1315024	298.6532631	870.1924819	938.2748313	590.724985	434.5050854	400.9923065	411.0250464	Rps20	ribosomal protein S20 [Source:MGI Symbol;Acc:MGI:1914677]
ENSMUSG00000028236	0	0	0.086717028	0.03952114	0	0	0	0	0	0	0	0	Sdr16c5	short chain dehydrogenase/reductase family 16C, member 5 [Source:MGI Symbol;Acc:MGI:2668443]
ENSMUSG00000028238	0.620177244	0.782181784	0.362818807	0.275590102	0.155141793	0.224941803	0.10546288	0.555862118	0.137437033	0.369822601	0.540817612	0.418755302	Atp6v0d2	ATPase, H+ transporting, lysosomal V0 subunit D2 [Source:MGI Symbol;Acc:MGI:1924415]
ENSMUSG00000028240	0	0	0	0	0	0	0.017461297	0.058126156	0	0	0	0	Cyp7a1	cytochrome P450, family 7, subfamily a, polypeptide 1 [Source:MGI Symbol;Acc:MGI:106091]
ENSMUSG00000028243	1.418735145	2.103728306	1.366269729	2.254930327	2.307380295	2.753373007	3.822977799	3.061205865	3.913263977	4.357565599	4.195352171	4.110683493	Ubxn2b	UBX domain protein 2B [Source:MGI Symbol;Acc:MGI:1915303]
ENSMUSG00000028244	0	0	0	0	0	0	0	0	0	0	0	0	1700025O08	RIKEN cDNA 1700025O08 gene [Source:MGI Symbol;Acc:MGI:1917234]
ENSMUSG00000028245	15.2104033	18.78042129	13.34768513	15.46421399	18.89115634	21.11578133	8.825610203	7.735101109	9.467779439	21.31575496	24.22906282	22.93899483	Nsmaf	neutral sphingomyelinase (N-SMase) activation associated factor [Source:MGI Symbol;Acc:MGI:1341864]
ENSMUSG00000028246	2.363672538	3.110731923	1.613273737	2.895972056	4.426225489	7.127982067	1.134070804	1.322102152	1.227216153	2.747085791	4.04211375	3.163492093	Faxc	failed axon connections homolog [Source:MGI Symbol;Acc:MGI:1923382]
ENSMUSG00000028247	12.56746687	11.72269028	14.18267567	14.9841027	13.72789179	11.93054288	13.30465678	14.2952538	14.43234668	16.99102906	14.45778754	16.85753409	Coq3	coenzyme Q3 methyltransferase [Source:MGI Symbol;Acc:MGI:101813]
ENSMUSG00000028248	65.69679903	90.56565899	53.72832322	88.60518994	108.6429152	132.4470162	71.47201816	67.6092638	88.3975908	73.88994109	74.22243535	77.53618847	Pnisr	PNN interacting serine/arginine-rich [Source:MGI Symbol;Acc:MGI:1913875]
ENSMUSG00000028249	135.5996202	157.0245342	109.607262	132.0730483	154.5615012	164.7378471	42.03988103	36.89610035	46.79198219	112.3545522	110.7818633	106.3993471	Sdcbp	syndecan binding protein [Source:MGI Symbol;Acc:MGI:1337026]
ENSMUSG00000028251	15.36344622	12.01294932	16.20057564	13.83120427	14.20588845	10.31927602	13.59518135	14.60230551	11.87855817	6.859001564	6.17998985	6.387501667	Tstd3	thiosulfate sulfurtransferase (rhodanese)-like domain containing 3 [Source:MGI Symbol;Acc:MGI:1924282]
ENSMUSG00000028252	23.45445541	26.1039971	20.26604661	32.84242793	33.80302342	37.86222475	25.91687073	23.86755513	31.68271918	30.51357608	29.68384886	31.88624736	Ccnc	cyclin C [Source:MGI Symbol;Acc:MGI:1858199]
ENSMUSG00000028255	1.136065685	1.504473742	0.668780036	1.37820518	2.178354613	4.088640198	0.177494375	0.225086985	0.211480668	0.533496564	0.378264031	0.729936789	Clca1	chloride channel accessory 1 [Source:MGI Symbol;Acc:MGI:1346342]
ENSMUSG00000028256	15.13267219	16.37180491	12.12953072	20.47877624	20.62381982	25.00095817	16.46694308	15.5245578	17.14244033	15.92024141	13.33607066	14.62795405	Odf2l	outer dense fiber of sperm tails 2-like [Source:MGI Symbol;Acc:MGI:1098600]
ENSMUSG00000028259	0	0	0	0	0	0	0	0	0	0.101373046	0	0	Fhl5	four and a half LIM domains 5 [Source:MGI Symbol;Acc:MGI:1913192]
ENSMUSG00000028261	14.27825103	14.09963704	13.58657874	17.84398137	16.52821004	15.62705441	12.59163159	13.28099352	12.8929056	14.57378646	13.40109119	14.54235492	Ndufaf4	NADH:ubiquinone oxidoreductase complex assembly factor 4 [Source:MGI Symbol;Acc:MGI:1915743]
ENSMUSG00000028262	0	0.139871604	0.031088391	0.028336964	0.031904247	0.069387482	0.108439955	0.060163511	0.056526678	0.081484756	0.17693587	0.026911012	Clca3a2	chloride channel accessory 3A2 [Source:MGI Symbol;Acc:MGI:1931471]
ENSMUSG00000028264	0.974229913	1.355599956	1.122085618	0.833374068	1.407428779	0.803891072	0	0.040213064	0.113346655	0.181547011	0.101368516	0.431693261	Spaca1	sperm acrosome associated 1 [Source:MGI Symbol;Acc:MGI:1914902]
ENSMUSG00000028266	82.38787979	87.06374838	91.13996983	67.57958285	62.76986148	55.92190774	8.867511534	8.260823198	8.451371338	20.61973737	19.71316402	21.97322415	Lmo4	LIM domain only 4 [Source:MGI Symbol;Acc:MGI:109360]
ENSMUSG00000028268	52.60915964	57.59685018	43.47527246	49.61991662	57.7908562	53.63606914	1.232696693	1.004931576	1.652322558	15.9295316	16.13753132	16.48180556	Gbp3	guanylate binding protein 3 [Source:MGI Symbol;Acc:MGI:1926263]
ENSMUSG00000028270	43.20724119	45.55026688	39.37251561	42.25051407	43.46437728	38.84256333	1.048714117	0.733619931	0.903185613	13.93332907	13.77408624	12.5374654	Gbp2	guanylate binding protein 2 [Source:MGI Symbol;Acc:MGI:102772]
ENSMUSG00000028271	21.68059116	22.17809076	19.90677286	19.6800336	21.0700281	17.89337639	19.73546627	20.25093967	19.16441357	24.76588214	22.66248178	22.85025861	Gtf2b	general transcription factor IIB [Source:MGI Symbol;Acc:MGI:2385191]
ENSMUSG00000028273	58.95860112	71.20092975	49.27921428	71.85479747	84.04672533	106.4361092	32.73918539	28.40102117	37.55893046	63.45135357	63.15457051	65.17180973	Pdlim5	PDZ and LIM domain 5 [Source:MGI Symbol;Acc:MGI:1927489]
ENSMUSG00000028274	13.0721983	17.35152333	12.13684444	17.37624992	21.26216828	22.57394279	17.79983266	17.10174316	20.80475404	20.51144405	23.22448605	23.66504011	Rngtt	RNA guanylyltransferase and 5'-phosphatase [Source:MGI Symbol;Acc:MGI:1329041]
ENSMUSG00000028277	48.62341514	53.15348283	42.92454123	46.12510436	51.95007082	56.19925522	5.681839448	5.85434235	6.704693388	17.2187123	16.56221223	18.12901198	Ube2j1	ubiquitin-conjugating enzyme E2J 1 [Source:MGI Symbol;Acc:MGI:1926245]
ENSMUSG00000028278	2.684196783	3.318466075	2.379273573	1.930142872	2.344044592	2.823362836	2.365267818	1.887831224	2.606493523	8.096712342	7.834639249	7.538019506	Rragd	Ras-related GTP binding D [Source:MGI Symbol;Acc:MGI:1098604]
ENSMUSG00000028280	0	0	0.019270451	0	0	0	0.016804398	0.037292955	0.017519314	0.370400376	0.250686793	0.316940153	Gabrr1	gamma-aminobutyric acid (GABA) C receptor, subunit rho 1 [Source:MGI Symbol;Acc:MGI:95625]
ENSMUSG00000028282	8.764546258	12.9650287	6.64778128	12.49594955	16.96775938	22.34109891	17.04285948	15.22548899	21.37338138	31.37118243	29.35638337	30.26675822	Casp8ap2	caspase 8 associated protein 2 [Source:MGI Symbol;Acc:MGI:1349399]
ENSMUSG00000028284	46.07834224	52.52525469	42.59101983	53.30430263	63.72415544	72.73452345	27.76492595	24.27470587	32.26026277	50.54233293	48.49785025	46.84062804	Map3k7	mitogen-activated protein kinase kinase kinase 7 [Source:MGI Symbol;Acc:MGI:1346877]
ENSMUSG00000028287	1.539174843	1.575501292	2.871450331	1.436332673	1.114036462	1.302624032	0	0	0.031835574	0.275351111	0.170827705	0.06062465	1700009N14	RIKEN cDNA 1700009N14 gene [Source:MGI Symbol;Acc:MGI:1922721]
ENSMUSG00000028289	1.953866753	2.116645975	1.437291715	2.066425196	2.38738849	2.987473795	0.71066834	0.65953212	0.86214117	2.226683557	1.614349584	1.410904647	Epha7	Eph receptor A7 [Source:MGI Symbol;Acc:MGI:95276]
ENSMUSG00000028291	89.37597747	75.53002382	75.72618216	86.31707219	85.68408547	78.88035637	51.53728356	52.52882242	47.80890448	45.30906601	43.9735193	40.26890761	Akirin2	akirin 2 [Source:MGI Symbol;Acc:MGI:1889364]
ENSMUSG00000028292	28.934666	32.51686963	27.12476517	36.43556322	36.26083868	35.84658507	70.33830919	65.02415193	70.68860656	50.87182592	44.52236605	46.87169535	Rars2	arginyl-tRNA synthetase 2, mitochondrial [Source:MGI Symbol;Acc:MGI:1923596]
ENSMUSG00000028293	56.74964649	54.20277251	47.37094397	54.61535995	50.71356248	58.58060242	37.10660827	36.59928306	38.93323089	33.8805318	34.19051581	33.09574081	Slc35a1	solute carrier family 35 (CMP-sialic acid transporter), member 1 [Source:MGI Symbol;Acc:MGI:1345622]
ENSMUSG00000028294	0.157038277	0.090418824	0.160774636	0	0.494981588	0.149516518	0.52575074	0.97230492	0.43849436	1.299320033	0.947709296	0.243549574	Cfap206	cilia and flagella associated protein 206 [Source:MGI Symbol;Acc:MGI:1916579]
ENSMUSG00000028295	24.21821992	18.20877009	30.63196086	28.25005613	26.92735902	22.52094587	19.83717874	19.50773085	17.17955407	16.35212779	15.02155843	14.95101591	Smim8	small integral membrane protein 8 [Source:MGI Symbol;Acc:MGI:1913541]
ENSMUSG00000028298	0	0	0.065946482	0	0	0	0	0	0.179861459	0.115233432	0	0.057085187	Cga	glycoprotein hormones, alpha subunit [Source:MGI Symbol;Acc:MGI:88390]
ENSMUSG00000028300	15.9231744	17.75731111	16.10591451	19.39522469	24.05072473	23.38135473	10.79549321	9.156136456	11.67821669	14.32857513	12.49704003	13.30513703	C9orf72	C9orf72, member of C9orf72-SMCR8 complex [Source:MGI Symbol;Acc:MGI:1920455]
ENSMUSG00000028307	2.237481367	12.53602745	0.572679253	5.661640375	0.632916457	0.753802475	2.765869493	4.347836465	1.241523697	0.885223227	0.752154385	0.57199357	Aldob	aldolase B, fructose-bisphosphate [Source:MGI Symbol;Acc:MGI:87995]
ENSMUSG00000028309	42.20778098	51.60170265	31.71281881	49.89096974	60.70479368	70.83474978	30.71094873	26.5511411	34.44008779	43.8783791	47.82089642	46.90243372	Rnf20	ring finger protein 20 [Source:MGI Symbol;Acc:MGI:1925927]
ENSMUSG00000028310	0	0.016374592	0.014557915	0	0	0.010830807	0	0	0	0	0	0	Ppp3r2	protein phosphatase 3, regulatory subunit B, alpha isoform (calcineurin B, type II) [Source:MGI Symbol;Acc:MGI:107171]
ENSMUSG00000028312	18.98176416	25.92855108	13.42406304	31.1715851	43.88207505	63.08428101	41.37309366	35.92013129	52.06242836	85.3876775	82.33809768	85.9982999	Smc2	structural maintenance of chromosomes 2 [Source:MGI Symbol;Acc:MGI:106067]
ENSMUSG00000028314	0.032062957	0	0.03282582	0.149603122	0.101061814	0.061054435	0.014312539	0	0.014921442	0.028679558	0.053378354	0.142074906	Toporsl	topoisomerase I binding, arginine/serine-rich like [Source:MGI Symbol;Acc:MGI:1915524]
ENSMUSG00000028318	18.94439548	14.83450276	19.76617046	17.55561703	16.58099884	17.89270805	42.09162035	45.72650278	41.21444077	37.42704234	38.77876105	38.57087833	Polr1e	polymerase (RNA) I polypeptide E [Source:MGI Symbol;Acc:MGI:1929022]
ENSMUSG00000028322	50.37481502	50.11823316	57.95683419	56.68726283	53.25111817	48.99645553	147.8172721	151.5449793	130.7774302	83.65748788	81.50009255	84.90999724	Exosc3	exosome component 3 [Source:MGI Symbol;Acc:MGI:1913612]
ENSMUSG00000028327	0.435420609	0.565692446	0.354338286	0.062511901	0.046920932	0.093542872	0.109642811	0.14378205	0.103915806	0.99864988	0.910756045	1.157641372	Stra6l	STRA6-like [Source:MGI Symbol;Acc:MGI:1921402]
ENSMUSG00000028328	0.890778776	0.891980479	0.475811883	0.795118373	1.709045338	1.887972386	0.069153653	0.230202598	0.036047841	0.34642634	0.451337765	0.205938279	Tmod1	tropomodulin 1 [Source:MGI Symbol;Acc:MGI:98775]
ENSMUSG00000028329	40.70262362	37.24184958	49.66508517	43.09909976	42.12461066	33.57544456	10.67903279	9.65527567	8.762362624	11.49190244	11.06312756	10.64973014	Xpa	xeroderma pigmentosum, complementation group A [Source:MGI Symbol;Acc:MGI:99135]
ENSMUSG00000028330	76.31353467	78.01783789	74.10195673	76.8902392	83.83567896	77.21024552	88.87824887	89.41835703	92.01165825	81.84782722	81.4065693	81.92406739	Ncbp1	nuclear cap binding protein subunit 1 [Source:MGI Symbol;Acc:MGI:1891840]
ENSMUSG00000028331	5.006771049	4.795114415	3.857109464	4.718496243	4.401040208	3.926841182	9.9356391	11.39308372	10.542893	7.050215038	7.056089524	6.501923841	Trmo	tRNA methyltransferase O [Source:MGI Symbol;Acc:MGI:1922003]
ENSMUSG00000028332	0	0.063470772	0	0	0.019303296	0.013994031	0	0.036401236	0	0	0	0	Hemgn	hemogen [Source:MGI Symbol;Acc:MGI:2136910]
ENSMUSG00000028333	314.7147732	253.3204549	359.9654151	339.5496019	294.6444245	238.2992165	470.1790094	507.7344563	386.6983634	224.7598549	220.1402014	218.7308746	Anp32b	acidic (leucine-rich) nuclear phosphoprotein 32 family, member B [Source:MGI Symbol;Acc:MGI:1914878]
ENSMUSG00000028334	96.06360073	80.08584502	106.1231362	98.04919006	83.14990974	71.26444022	140.2588858	147.5868954	118.5312439	87.21657496	82.48660721	84.71660358	Nans	N-acetylneuraminic acid synthase (sialic acid synthase) [Source:MGI Symbol;Acc:MGI:2149820]
ENSMUSG00000028337	0.486197603	0.581415438	0.459475882	0.401360274	0.373297718	0.29911101	3.372360071	3.297436285	3.568046963	2.977339424	2.661749547	2.386413853	Coro2a	coronin, actin binding protein 2A [Source:MGI Symbol;Acc:MGI:1345966]
ENSMUSG00000028339	0.309716622	0.611407905	0.113244859	0.51611154	0.069730054	0.219055151	0.098752838	0.438312059	0.205908234	0.296822363	0.478785996	0.725407441	Col15a1	collagen, type XV, alpha 1 [Source:MGI Symbol;Acc:MGI:88449]
ENSMUSG00000028341	0.199021683	0.397251744	0.43468147	0.235250101	0.306686094	0.252652025	0	0	0.024698829	0.083076091	0.099399284	0.14110233	Nr4a3	nuclear receptor subfamily 4, group A, member 3 [Source:MGI Symbol;Acc:MGI:1352457]
ENSMUSG00000028343	126.8252951	117.6616843	115.9154849	121.9726557	118.7782477	107.9041591	50.06494857	46.93490267	48.78137485	57.6365879	57.78976539	59.75036899	Erp44	endoplasmic reticulum protein 44 [Source:MGI Symbol;Acc:MGI:1923549]
ENSMUSG00000028344	6.202304552	7.979257584	5.523067405	7.14442511	9.94447243	11.09690939	1.845758754	2.192095021	2.345220585	4.594284274	4.678781031	4.666414012	Invs	inversin [Source:MGI Symbol;Acc:MGI:1335082]
ENSMUSG00000028345	32.62521857	39.25386517	23.49936373	36.25700548	41.68881111	50.56255157	56.31651304	50.52471641	65.49704989	98.39330438	93.70511501	93.91064028	Tex10	testis expressed gene 10 [Source:MGI Symbol;Acc:MGI:1344413]
ENSMUSG00000028347	48.16117173	64.62672538	41.56644465	35.27769838	37.52290423	40.56955555	68.79002797	65.33933512	76.49387941	94.54294025	95.14497136	93.07152523	Tmeff1	transmembrane protein with EGF-like and two follistatin-like domains 1 [Source:MGI Symbol;Acc:MGI:1926810]
ENSMUSG00000028348	5.111465935	5.379523848	4.954269618	6.822572427	6.184779677	6.781382069	1.701923915	1.141393195	1.676838866	1.424092232	2.075077012	2.246388251	Cavin4	caveolae associated 4 [Source:MGI Symbol;Acc:MGI:1915266]
ENSMUSG00000028351	1.93673873	1.325528443	1.384232066	0.511509272	0.575902145	0.250502069	0.228368559	0.271502215	0.408144219	2.386090484	2.205285327	1.538270041	Brinp1	bone morphogenic protein/retinoic acid inducible neural specific 1 [Source:MGI Symbol;Acc:MGI:1928478]
ENSMUSG00000028354	0.252366498	0.494042091	0.465067744	0.117752134	0.344696855	0.403668289	2.613561919	2.425048227	3.265004142	4.243834415	4.159379088	2.706206139	Fmn2	formin 2 [Source:MGI Symbol;Acc:MGI:1859252]
ENSMUSG00000028356	0.052346092	0	0	0	0	0	0	0	0	0	0.087145682	0	Ambp	alpha 1 microglobulin/bikunin precursor [Source:MGI Symbol;Acc:MGI:88002]
ENSMUSG00000028357	0.045556896	0.078691661	0.023320408	0.06376942	0.023932408	0.017349932	0	0.31591417	0.04240249	0.020374784	0.01896076	0.040373642	Kif12	kinesin family member 12 [Source:MGI Symbol;Acc:MGI:1098232]

ENSMUSG00000028358	7.412955578	7.975182843	7.037855769	9.131774542	9.621084739	11.99597536	0.778602109	0.775015413	0.835600777	4.015138072	4.430403375	4.455469043	Zfp618	zinc finger protein 618 [Source:MGI Symbol;Acc:MGI:1919950]
ENSMUSG00000028359	0.333985675	0.064100279	0.056988681	0.051944991	0.058484241	0.084796949	0.049695801	0	0	0	0.046334897	0	Orm3	orosomucoid 3 [Source:MGI Symbol;Acc:MGI:97445]
ENSMUSG00000028360	0.20112644	0.043426394	0.102955887	0.082113452	0.118864991	0.15319437	0.022445138	0	0.02340003	0.202390708	0.125563104	0.0445608	Slc44a5	solute carrier family 44, member 5 [Source:MGI Symbol;Acc:MGI:3035141]
ENSMUSG00000028362	0.146932057	0.195230558	0.092571054	0.09492548	0.15437566	0.060262187	0	0	0	0.010109789	0.047040814	0.03004957	Tnfsf8	tumor necrosis factor (ligand) superfamily, member 8 [Source:MGI Symbol;Acc:MGI:88328]
ENSMUSG00000028364	384.8292161	441.1659978	368.3788553	182.6004869	211.2538241	248.3157148	29.02911536	26.2427824	34.89596464	518.8488751	533.2781285	519.0771647	Tnc	tenascin C [Source:MGI Symbol;Acc:MGI:101922]
ENSMUSG00000028367	502.8724224	385.0341523	584.9602319	515.5003582	442.8350499	335.0371815	427.4611973	460.1644795	342.6850805	223.2741494	219.2633462	217.041899	Txn1	thioredoxin 1 [Source:MGI Symbol;Acc:MGI:98874]
ENSMUSG00000028369	15.97294913	20.60398788	14.54168538	6.456018837	8.128027756	9.153333925	0.044703089	0.016534464	0.069907363	1.142098304	1.062835909	0.961458031	Svep1	sushi, von Willebrand factor type A, EGF and pentraxin domain containing 1 [Source:MGI Symbol;Acc:MGI:1928849]
ENSMUSG00000028370	2.567583657	3.675660087	2.101340673	0.670013596	0.791258315	1.096727452	0.041804502	0.023193533	0.032687255	0.649203024	0.782793778	0.82303721	Pappa	pregnancy-associated plasma protein A [Source:MGI Symbol;Acc:MGI:97479]
ENSMUSG00000028373	0.133989478	0.133723003	0.164612934	0.100029424	0.131392248	0.081645889	0.039874252	0.008849045	0.041570637	0.071910255	0.066919634	0.071246864	Astn2	astrotactin 2 [Source:MGI Symbol;Acc:MGI:1889277]
ENSMUSG00000028378	60.64468058	50.21852041	63.44982204	50.26180109	44.87358777	37.37037719	35.8215168	37.18622515	33.8743259	22.61335655	22.0579538	21.2587622	Ptgr1	prostaglandin reductase 1 [Source:MGI Symbol;Acc:MGI:1914353]
ENSMUSG00000028381	124.576912	141.217742	110.6931788	106.287071	114.2198377	125.9234964	33.13186825	30.34335065	36.40355096	57.34127275	53.50248393	56.4876427	Ugcg	UDP-glucose ceramide glucosyltransferase [Source:MGI Symbol;Acc:MGI:1332243]
ENSMUSG00000028382	47.56710915	53.01119411	39.90852255	53.20794186	65.21145099	75.58976175	44.62437843	43.94939	58.62903186	67.64391898	65.01008221	67.49001761	Ptbp3	polypyrimidine tract binding protein 3 [Source:MGI Symbol;Acc:MGI:1923334]
ENSMUSG00000028383	19.41337519	23.64922051	16.28668049	21.42916676	22.66317787	24.66180874	11.65484607	11.04042844	14.3675024	13.74713786	14.4576735	12.94328358	Hsdl2	hydroxysteroid dehydrogenase like 2 [Source:MGI Symbol;Acc:MGI:1919729]
ENSMUSG00000028385	18.66827761	23.0044838	17.30451162	21.90858501	28.34424064	33.47057366	10.92338833	9.496707732	13.26655661	19.88568435	20.19867034	20.55459978	Snx30	sorting nexin family member 30 [Source:MGI Symbol;Acc:MGI:2443882]
ENSMUSG00000028386	0.015188312	0	0	0.028346966	0	0.023137325	0	0.015046187	0.014136658	0	0	0	Slc46a2	solute carrier family 46, member 2 [Source:MGI Symbol;Acc:MGI:1353616]
ENSMUSG00000028389	4.909578332	6.715230817	4.609353533	6.822289956	8.469520528	10.05919119	4.019491457	3.610560094	4.988683548	5.25446078	4.586416043	5.737985658	Zfp37	zinc finger protein 37 [Source:MGI Symbol;Acc:MGI:99181]
ENSMUSG00000028391	1.435509859	1.271587227	0.942092605	1.889171327	1.353542466	1.289653507	18.36946401	19.32566234	19.52781554	12.93906825	15.19689499	15.2009697	Wdr31	WD repeat domain 31 [Source:MGI Symbol;Acc:MGI:1918604]
ENSMUSG00000028392	1.10329456	1.303077334	1.158507566	1.715960591	1.605029054	1.810005084	8.385096352	8.435489913	7.609602097	10.67844958	10.54961224	10.80557654	Bspry	B-box and SPRY domain containing [Source:MGI Symbol;Acc:MGI:2177191]
ENSMUSG00000028393	64.46627743	48.31694061	73.70220111	74.91320492	59.47448277	46.37745209	47.97078792	57.3191925	46.39667163	33.40000493	31.28567162	32.84796054	Alad	aminolevulinate, delta-, dehydratase [Source:MGI Symbol;Acc:MGI:96853]
ENSMUSG00000028394	72.74308848	63.08586881	73.03735395	78.24108833	74.36440772	64.2655796	42.00580782	45.28224111	38.59809531	36.45200873	32.93595289	34.84463247	Pole3	polymerase (DNA directed), epsilon 3 (p17 subunit) [Source:MGI Symbol;Acc:MGI:1933378]
ENSMUSG00000028396	0.038939808	0	0	0	0	0	0	0	0	0	0	0	2310002L09	RIKEN cDNA 2310002L09 gene [Source:MGI Symbol;Acc:MGI:1916780]
ENSMUSG00000028397	14.04791654	18.76122583	11.86362004	18.44562974	21.67459997	24.72022419	44.86870368	36.55476408	55.27345285	47.15451447	43.57659976	42.55050799	Kdm4c	lysine (K)-specific demethylase 4C [Source:MGI Symbol;Acc:MGI:1924054]
ENSMUSG00000028398	36.40872047	30.35827273	43.87390101	34.08439617	29.04070113	25.12232621	61.43266137	64.3126825	53.8312482	34.12495804	34.0284485	30.46515462	Dmac1	distal membrane arm assembly complex 1 [Source:MGI Symbol;Acc:MGI:1914178]
ENSMUSG00000028399	0.377328741	0.569362441	0.372985487	0.279265192	0.369103295	0.57480895	0.394951629	0.335129132	0.230097937	1.373323263	1.635424311	1.695052008	Ptprd	protein tyrosine phosphatase, receptor type, D [Source:MGI Symbol;Acc:MGI:97812]
ENSMUSG00000028402	19.78071005	30.20418119	15.86155369	27.47754536	35.37124168	50.73658236	1.555136171	1.377168299	2.026621136	10.00777016	9.815134425	9.292019593	Mpdz	multiple PDZ domain protein [Source:MGI Symbol;Acc:MGI:1343489]
ENSMUSG00000028403	9.648643414	13.23033081	7.494306984	15.8349911	18.72204499	24.95753354	6.983387276	6.69913679	8.630163889	10.67585846	9.540334298	9.860679826	Zdhhc21	zinc finger, DHHC domain containing 21 [Source:MGI Symbol;Acc:MGI:1915518]
ENSMUSG00000028405	52.07092916	62.65410517	49.12188013	65.32474307	66.66530433	69.69457569	59.11878543	57.55555847	64.81149324	47.9517694	54.37815834	49.74449682	Aco1	aconitase 1 [Source:MGI Symbol;Acc:MGI:87879]
ENSMUSG00000028407	0.385329742	0.073954501	0.065749627	0.239722256	0.269900408	0.244582244	0.172006809	0.381724009	0	0.229778904	0.106916046	0.227659132	Smim27	small integral membrane protein 27 [Source:MGI Symbol;Acc:MGI:1913684]
ENSMUSG00000028409	121.117225	113.4467024	118.7334218	128.7750426	133.7983563	116.8057267	174.742316	175.7778012	165.32074	139.8914468	143.9910752	137.9589431	Smu1	smu-1 suppressor of mec-8 and unc-52 homolog (C. elegans) [Source:MGI Symbol;Acc:MGI:1915546]
ENSMUSG00000028410	249.6794012	253.2804704	231.0003568	263.7832673	264.6773353	259.4927018	153.2771789	164.4947721	157.7834731	195.4397231	188.3504347	189.3575914	Dnaja1	DnaJ heat shock protein family (Hsp40) member A1 [Source:MGI Symbol;Acc:MGI:1270129]
ENSMUSG00000028411	12.82805658	10.35017162	12.32638957	13.30103514	12.03323516	11.44408515	5.868520185	6.229420455	5.556312526	5.39971469	6.434754324	5.424205071	Aptx	aprataxin [Source:MGI Symbol;Acc:MGI:1913658]
ENSMUSG00000028412	29.58334147	33.92787945	29.29958164	36.29004185	41.11190837	47.20928521	43.68994379	42.68557565	50.24382111	39.04524192	38.82810275	37.52733778	Slc44a1	solute carrier family 44, member 1 [Source:MGI Symbol;Acc:MGI:2140592]
ENSMUSG00000028413	65.08497362	77.00560378	64.34763455	71.21363656	73.39230332	80.81747935	21.20078492	20.01740142	22.88947059	32.45371516	35.16638367	34.20371733	B4galt1	UDP-Gal:betaGlcNAc beta 1,4- galactosyltransferase, polypeptide 1 [Source:MGI Symbol;Acc:MGI:95705]
ENSMUSG00000028414	5.869287861	8.761804592	4.228140545	7.463033482	9.770632489	12.9156879	4.554606682	3.186349501	4.992577155	7.318624138	7.021013531	7.500849138	Fktn	fukutin [Source:MGI Symbol;Acc:MGI:2179507]
ENSMUSG00000028415	1.249214163	0.479511442	0.99472823	0.777164087	0.29166657	0.740058531	0.247837768	0.137502735	0.258381623	0.24830946	0.115538308	0.12300937	Spink4	serine peptidase inhibitor, Kazal type 4 [Source:MGI Symbol;Acc:MGI:1341848]
ENSMUSG00000028416	257.1325321	201.8974607	289.329598	222.5096193	186.3905554	161.2007259	163.5093789	182.2367501	152.0720045	97.58411987	99.56335069	83.59906028	Bag1	BCL2-associated athanogene 1 [Source:MGI Symbol;Acc:MGI:108047]
ENSMUSG00000028417	0.18827679	0.316876741	0.133446736	0.13515139	0.152165326	0.154438334	0.232738934	0.573892261	0.161760289	0.129545494	0.168776929	0.128350403	Tal2	T cell acute lymphocytic leukemia 2 [Source:MGI Symbol;Acc:MGI:99540]
ENSMUSG00000028419	108.1235932	112.7993581	95.15170039	111.8428998	118.8690082	110.4693783	54.9289421	55.8148855	54.36560266	64.05370572	61.37677872	59.72230432	Chmp5	charged multivesicular body protein 5 [Source:MGI Symbol;Acc:MGI:1924209]
ENSMUSG00000028420	27.64562203	21.89202883	23.80901549	18.68863387	18.0299617	17.24366684	27.36977377	33.78439868	33.46434162	17.84855102	19.78082077	19.16290994	Tmem38b	transmembrane protein 38B [Source:MGI Symbol;Acc:MGI:1098718]
ENSMUSG00000028423	50.48209671	54.99614758	48.23514136	65.86338216	65.18273788	75.5708919	79.29430528	80.20149873	91.45526945	79.57691593	77.33258658	79.83771106	Nfx1	nuclear transcription factor, X-box binding 1 [Source:MGI Symbol;Acc:MGI:1921414]
ENSMUSG00000028426	148.9304988	199.0256676	135.4796574	214.2239965	262.4079814	290.8341163	215.4833221	184.2373654	219.9250821	260.5610439	255.8127701	248.9117135	Rad23b	RAD23 homolog B, nucleotide excision repair protein [Source:MGI Symbol;Acc:MGI:105128]
ENSMUSG00000028427	0.475452904	0.342192787	0.334651072	0.388224233	0.343433358	0.430045851	0.106118381	0.264939661	0.248924284	0.053160175	0	0.342353426	Aqp7	aquaporin 7 [Source:MGI Symbol;Acc:MGI:1314647]
ENSMUSG00000028430	47.31293714	51.03267156	43.81005701	52.68764227	50.37261617	58.66089216	109.8003068	97.49002675	117.5297031	135.6600892	145.1053868	141.0707824	Nol6	nucleolar protein family 6 (RNA-associated) [Source:MGI Symbol;Acc:MGI:2140151]
ENSMUSG00000028431	39.29094274	48.1776235	38.83868313	52.68781538	59.3942076	63.51968169	59.01956407	53.8492658	68.09660291	94.02597648	94.96722033	94.62839703	Elp1	elongator complex protein 1 [Source:MGI Symbol;Acc:MGI:1914544]
ENSMUSG00000028433	113.8089064	127.3987882	109.4801896	139.3514321	146.2140175	163.0122597	144.970956	128.1922306	164.4434289	167.7197085	178.747843	175.5137318	Ubap2	ubiquitin-associated protein 2 [Source:MGI Symbol;Acc:MGI:1916176]
ENSMUSG00000028434	5.849654713	5.085415091	5.397051609	6.192395561	6.461801548	5.899675934	6.233898941	6.001059698	7.510560925	8.789567817	10.43135224	10.1018388	Epb41l4b	erythrocyte membrane protein band 4.1 like 4b [Source:MGI Symbol;Acc:MGI:1859149]
ENSMUSG00000028435	6.436394301	7.13732635	6.142095826	9.343191518	6.595488734	6.930056072	23.76542219	27.47261468	24.55460444	8.84903382	8.929414939	6.86603547	Aqp3	aquaporin 3 [Source:MGI Symbol;Acc:MGI:1333777]
ENSMUSG00000028436	19.36406962	19.26125854	17.22670795	20.00674448	19.63580358	21.2431261	29.72073108	28.76811279	32.76537208	26.26619204	26.24350958	27.02096297	Dcaf12	DDB1 and CUL4 associated factor 12 [Source:MGI Symbol;Acc:MGI:1916220]
ENSMUSG00000028437	77.2470751	72.82938974	77.17356916	81.01474579	81.06847595	76.05794755	40.83694318	40.08274628	42.43852463	38.38359223	39.65353062	39.29628144	Ubap1	ubiquitin-associated protein 1 [Source:MGI Symbol;Acc:MGI:2149543]
ENSMUSG00000028438	2.331809668	3.090511711	1.599033622	3.264009701	4.067824146	8.126482622	7.875437875	7.518362708	9.295661959	12.848999	10.73041361	10.89790068	Kif24	kinesin family member 24 [Source:MGI Symbol;Acc:MGI:1918345]
ENSMUSG00000028439	6.907000969	9.669284973	5.130185612	8.593999753	10.06210323	11.75974302	2.038198193	1.571636471	2.467056546	3.14964473	3.849025737	3.069150153	Fam219a	family with sequence similarity 219, member A [Source:MGI Symbol;Acc:MGI:1919151]
ENSMUSG00000028441	5.069448279	2.562905981	4.873596645	3.576890267	2.663133696	2.825346607	0.110387512	0.428708238	0.230167537	0.276494011	0.51461028	0.164365968	1110017D15	RIKEN cDNA 1110017D15 gene [Source:MGI Symbol;Acc:MGI:1920971]
ENSMUSG00000028443	36.3068779	27.03233929	35.76312856	29.46172338	33.86611304	25.48228587	33.09587428	35.76535624	32.18150755	16.53388378	17.92963116	17.73518526	Nudt2	nudix (nucleoside diphosphate linked moiety X)-type motif 2 [Source:MGI Symbol;Acc:MGI:1913651]
ENSMUSG00000028444	9.097451712	8.824532783	8.936313612	3.671175116	3.315276679	3.246188711	63.62231546	64.01996364	62.32410818	6.927833947	5.969479247	6.500752323	Cntfr	ciliary neurotrophic factor receptor [Source:MGI Symbol;Acc:MGI:99605]
ENSMUSG00000028445	8.273405067	4.997902285	9.858817223	2.468062467	3.099388256	1.601252387	5.358100213	6.3149404	3.881832653	2.396019971	2.681326376	2.824659607	Enho	energy homeostasis associated [Source:MGI Symbol;Acc:MGI:1916888]
ENSMUSG00000028447	162.2654691	120.4984815	184.3112539	144.6947532	119.0567839	98.76857445	85.6222088	89.95563772	69.45854208	63.65720869	58.60499653	61.19967537	Dctn3	dynactin 3 [Source:MGI Symbol;Acc:MGI:1859251]
ENSMUSG00000028451	0.163234021	0.075188946	0	0.182792767	0.034300697	0.29839776	0.029146323	0	0	0.058403591	0.054350342	0.057864802	1700022I11	RIKEN cDNA 1700022I11 gene [Source:MGI Symbol;Acc:MGI:1914567]
ENSMUSG00000028452	423.3436572	434.2005702	475.5467602	455.4693264	460.0085924	419.7232602	365.7157342	348.4092951	353.9158353	373.9193046	371.4342642	359.3908573	Vcp	valosin containing protein [Source:MGI Symbol;Acc:MGI:99919]
ENSMUSG00000028453	14.75490315	19.83817493	15.67753853	20.87683139	19.56652889	21.41398012	21.29016009	18.21178479	21.97321381	22.5434613	23.56810157	22.79492449	Fancg	Fanconi anemia, complementation group G [Source:MGI Symbol;Acc:MGI:1926471]
ENSMUSG00000028454	8.899777159	8.599870967	9.131334236	11.69939462	9.818173454	11.56816826	17.63559623	17.15383709	17.07131099	14.76179645	16.12081491	13.88833309	Pigo	phosphatidylinositol glycan anchor biosynthesis, class O [Source:MGI Symbol;Acc:MGI:1861452]
ENSMUSG00000028455	130.974519	125.9887074	139.3555329	147.5395472	141.9100139	122.7826724	217.6997889	236.1056101	210.5022476	187.9339236	188.0822754	183.1774531	Stoml2	stomatin (Epb7.2)-like 2 [Source:MGI Symbol;Acc:MGI:1913842]
ENSMUSG00000028456	1.978876053	3.265321417	2.32244056	2.420919163	2.877814993	3.942808428	7.400730439	6.060377113	8.097430804	11.55934155	12.66547391	12.71453272	Unc13b	unc-13 homolog B [Source:MGI Symbol;Acc:MGI:1342278]
ENSMUSG00000028457	0.798245225	0.353546312	0.801521498	0.744909328	0.387085137	0.549546547	0.411147207	0.182486683	0.328623077	0.631625563	0.587790322	0.489755465	Atp8b5	ATPase, class I, type 8B, member 5 [Source:MGI Symbol;Acc:MGI:2444287]
ENSMUSG00000028458	8.019067775	9.296344395	6.28137151	8.236610838	8.142586419	8.157636156	1.729749527	1.25291735	1.502782421	3.94748373	2.822342908	2.90945239	Tesk1	testis specific protein kinase 1 [Source:MGI Symbol;Acc:MGI:1201675]
ENSMUSG00000028459	7.113110454	7.295908805	9.073092182	0.326451039	0.245031536	0.621729389	0.659333539	0.154022937	0.217068572	10.32603976	9.965309528	9.920755691	Cd72	CD72 antigen [Source:MGI Symbol;Acc:MGI:88345]
ENSMUSG00000028460	0.042020008	0	0.172079102	0.117637142	0	0.128023518	0.150058024	0.124880413	0	0.187929523	0.454706427	0.558587471	Sit1	suppression inducing transmembrane adaptor 1 [Source:MGI Symbol;Acc:MGI:1889342]
ENSMUSG00000028461	62.13290415	43.59822427	81.56503006	51.37044128	39.83944923	31.53559611	27.26884378	29.62525178	22.6458765	15.94575665	14.45242344	11.11566452	Ccdc107	coiled-coil domain containing 107 [Source:MGI Symbol;Acc:MGI:1913423]

ENSMUSG00000028463	33.71823023	29.26608818	35.44789256	19.81844169	18.08332734	17.63280642	3.459583349	3.090997143	3.700447918	2.340788598	1.968880874	1.962395095	Car9	carbonic anhydrase 9 [Source:MGI Symbol;Acc:MGI:2447188]
ENSMUSG00000028464	285.6529915	204.3965644	362.6865914	229.3771321	192.8466727	142.8356502	22.41612697	25.37016266	19.91566198	58.49453164	55.85428833	60.38450642	Tpm2	tropomyosin 2, beta [Source:MGI Symbol;Acc:MGI:98810]
ENSMUSG00000028465	372.1886528	404.7438172	358.858854	422.2155106	442.1003599	514.5245165	74.40277693	66.96515544	86.38761023	163.5622783	174.3001738	166.825722	Tln1	talin 1 [Source:MGI Symbol;Acc:MGI:1099832]
ENSMUSG00000028466	116.84846	93.59989425	122.9388809	103.3777978	91.30512388	74.98241253	11.76871252	14.04435444	12.9638872	23.67121169	24.47141434	23.32058564	Creb3	cAMP responsive element binding protein 3 [Source:MGI Symbol;Acc:MGI:99946]
ENSMUSG00000028467	8.423468015	9.678643501	8.205064752	9.005901036	8.166411871	9.291165747	6.391408305	6.097301464	6.524861765	9.297654798	9.395351519	7.975940307	Gba2	glucosidase beta 2 [Source:MGI Symbol;Acc:MGI:2654325]
ENSMUSG00000028468	30.55480386	26.75146721	30.25663195	34.38675495	32.49347881	35.49792528	13.20510609	13.2638941	14.61900544	20.24200891	22.12358864	21.52582186	Rgp1	RAB6A GEF compex partner 1 [Source:MGI Symbol;Acc:MGI:1915956]
ENSMUSG00000028469	64.62547883	63.96969517	60.00707158	62.6754606	61.20510792	60.12772248	4.038484659	4.071314941	4.672401011	18.87390659	21.6278825	19.06645234	Npr2	natriuretic peptide receptor 2 [Source:MGI Symbol;Acc:MGI:97372]
ENSMUSG00000028470	32.49949407	24.41101749	37.15336599	36.55080038	26.69724509	24.219627	30.58148252	30.17882203	22.60523071	21.97518471	17.93765216	22.14569995	Hint2	histidine triad nucleotide binding protein 2 [Source:MGI Symbol;Acc:MGI:1916167]
ENSMUSG00000028475	0.016280169	0.074989808	0.100005108	0.030384773	0.068419702	0	0	0	0	0.043686681	0.013551599	0.014427887	Spaar	small regulatory polypeptide of amino acid response [Source:MGI Symbol;Acc:MGI:1918656]
ENSMUSG00000028476	8.60293135	11.65870956	6.103027912	7.731254652	9.60549612	12.44169668	2.852404786	3.110275612	4.402703253	6.890966657	6.930812934	6.93771265	Reck	reversion-inducing-cysteine-rich protein with kazal motifs [Source:MGI Symbol;Acc:MGI:1855698]
ENSMUSG00000028478	405.2089117	337.4007151	458.6074977	362.3884948	312.7538679	252.8477328	322.9610014	370.3031418	293.5375102	212.52051	227.3892437	224.5568961	Clta	clathrin, light polypeptide (Lca) [Source:MGI Symbol;Acc:MGI:894297]
ENSMUSG00000028479	22.03829067	21.82266356	22.5336395	20.98871718	19.8511839	22.18017986	14.79439739	13.72221167	13.28819774	14.54383983	16.43473483	14.51008487	Gne	glucosamine (UDP-N-acetyl)-2-epimerase/N-acetylmannosamine kinase [Source:MGI Symbol;Acc:MGI:1354951]
ENSMUSG00000028480	124.2166694	110.3842449	129.0679806	132.4016273	123.4484399	107.3872166	11.05262968	11.35727373	11.34855067	35.95684286	37.82594586	39.22083572	Glipr2	GLI pathogenesis-related 2 [Source:MGI Symbol;Acc:MGI:1917770]
ENSMUSG00000028483	16.68222769	20.39818517	19.0074326	18.7899026	19.64759641	21.86073289	31.80886041	32.63020858	33.18298641	20.65794964	18.72033669	19.91097994	Snapc3	small nuclear RNA activating complex, polypeptide 3 [Source:MGI Symbol;Acc:MGI:1916338]
ENSMUSG00000028484	59.11046188	66.44300241	50.93345048	83.34281063	84.90264066	96.14803629	162.2356284	159.3148659	168.1664444	144.1788869	140.9171791	132.7307952	Psip1	PC4 and SFRS1 interacting protein 1 [Source:MGI Symbol;Acc:MGI:2142116]
ENSMUSG00000028487	6.451141552	7.248509927	6.123711636	7.481288447	10.19983893	15.33747836	6.374517269	4.46733775	9.939418245	11.12061328	10.16636204	9.519349569	Bnc2	basonuclin 2 [Source:MGI Symbol;Acc:MGI:2443805]
ENSMUSG00000028488	0.132872325	0.408024833	0.158705997	0.165325694	0.349009148	0.691577378	11.6450587	11.05683337	13.37719987	15.27237369	15.4290915	16.32865502	Sh3gl2	SH3-domain GRB2-like 2 [Source:MGI Symbol;Acc:MGI:700009]
ENSMUSG00000028492	0	0	0.114619905	0	0	0	0.433124981	0.184847562	0.486286315	0.367187884	0.559153303	0.661455415	Saxo1	stabilizer of axonemal microtubules 1 [Source:MGI Symbol;Acc:MGI:1923061]
ENSMUSG00000028494	906.2968952	860.239442	915.7853964	875.8681385	822.045798	733.0570144	80.44364912	77.13248631	77.02833324	350.6830385	341.9866821	345.4991748	Plin2	perilipin 2 [Source:MGI Symbol;Acc:MGI:87920]
ENSMUSG00000028495	1444.205859	1119.782842	1536.194791	1391.579665	1270.953953	987.2924219	2110.664619	2329.728426	1875.236885	1269.634097	1157.002868	1167.024803	Rps6	ribosomal protein S6 [Source:MGI Symbol;Acc:MGI:98159]
ENSMUSG00000028496	26.02847328	26.89948725	20.76067745	27.28163024	34.08503935	40.29212445	4.653922787	4.09932174	4.551797821	7.146391758	5.889952462	8.334627378	Mllt3	myeloid/lymphoid or mixed-lineage leukemia; translocated to, 3 [Source:MGI Symbol;Acc:MGI:1917372]
ENSMUSG00000028497	7.807314768	10.86680762	7.270219897	8.679435884	10.47109677	14.06521555	0.545493352	0.484231782	0.429684806	2.4776097	2.836868332	2.972176541	Hacd4	3-hydroxyacyl-CoA dehydratase 4 [Source:MGI Symbol;Acc:MGI:1914025]
ENSMUSG00000028514	25.8978059	32.6991548	24.33416667	34.84859566	41.07055829	50.82517107	10.88959237	9.732809986	13.1273658	16.38103614	17.37558642	16.76639195	Usp24	ubiquitin specific peptidase 24 [Source:MGI Symbol;Acc:MGI:1919936]
ENSMUSG00000028517	43.39625268	53.51786873	35.53185613	44.34804817	50.83252509	54.45737079	7.271947684	8.082583974	9.673156889	15.09547019	17.06375359	17.0445272	Plpp3	phospholipid phosphatase 3 [Source:MGI Symbol;Acc:MGI:1915166]
ENSMUSG00000028518	1.052899376	2.213530877	1.155341987	2.499200026	3.193911812	3.409419389	1.56667431	1.497708289	2.060503466	2.439004018	2.579857764	2.76103476	Prkaa2	protein kinase, AMP-activated, alpha 2 catalytic subunit [Source:MGI Symbol;Acc:MGI:1336173]
ENSMUSG00000028519	2.575014233	1.364020179	2.319927	2.52310902	2.380809105	3.314667557	19.97756325	21.78484377	21.4026884	9.858078759	9.666912532	9.08252308	Dab1	disabled 1 [Source:MGI Symbol;Acc:MGI:108554]
ENSMUSG00000028520	0.039804337	0.02291837	0	0.037144753	0.146372909	0.090954729	0	0	0.055572366	0.035604039	0.033133095	0	4921539E11	RIKEN cDNA 4921539E11 gene [Source:MGI Symbol;Acc:MGI:1918191]
ENSMUSG00000028521	12.29385367	14.64331378	11.49746116	15.8366134	16.95928191	18.04894266	4.663962654	4.121583238	5.168102069	5.79208218	5.819754031	5.447557813	Slc35d1	solute carrier family 35 (UDP-glucuronic acid/UDP-N-acetylgalactosamine dual transporter), member D1 [Source:MGI Symbol;Acc:M
ENSMUSG00000028522	60.23581983	78.18436318	54.5765366	85.63421123	105.8275932	129.8426852	23.41528641	21.3733004	30.65140445	38.19721686	37.64323174	39.37519479	Mier1	MEIR1 treanscription regulator [Source:MGI Symbol;Acc:MGI:1918398]
ENSMUSG00000028523	0	0	0.385156286	0.087767165	0.148223995	0	0	0	0	0	0	0.083350611	Tctex1d1	Tctex1 domain containing 1 [Source:MGI Symbol;Acc:MGI:1914594]
ENSMUSG00000028524	6.858301847	8.132271896	6.117724383	9.019218851	8.822875385	9.016734768	0.181869378	0.089691421	0.063202238	1.154031606	1.111623174	1.123325967	Sgip1	SH3-domain GRB2-like (endophilin) interacting protein 1 [Source:MGI Symbol;Acc:MGI:1920344]
ENSMUSG00000028525	12.36201515	16.1585366	9.614225976	13.47893964	15.85936974	17.41119846	0.832580003	0.558604859	0.8478147	6.61511922	7.311408553	7.322901556	Pde4b	phosphodiesterase 4B, cAMP specific [Source:MGI Symbol;Acc:MGI:99557]
ENSMUSG00000028527	50.67620783	58.42578526	49.86269667	67.23547739	72.86616699	78.4706322	23.10640895	23.48608447	26.59953001	63.04249684	60.54358652	59.46450151	Ak4	adenylate kinase 4 [Source:MGI Symbol;Acc:MGI:87979]
ENSMUSG00000028528	0.380783078	0.822171166	0.389842922	0.632950287	0.812649528	0.996997309	3.760746223	2.687691724	5.36056121	12.37873476	12.76768598	13.87801509	Dnajc6	DnaJ heat shock protein family (Hsp40) member C6 [Source:MGI Symbol;Acc:MGI:1919935]
ENSMUSG00000028530	295.1385928	379.3140741	261.0224381	374.7553772	433.3665879	515.763615	54.25911295	48.33140864	63.95397582	88.26773299	89.55612523	87.2457119	Jak1	Janus kinase 1 [Source:MGI Symbol;Acc:MGI:96628]
ENSMUSG00000028532	16.75879684	22.26903571	16.5094678	21.61996747	25.13974893	29.36282246	3.630973702	2.539679047	3.667612626	6.836957616	6.151703144	7.573287434	Cachd1	cache domain containing 1 [Source:MGI Symbol;Acc:MGI:2444177]
ENSMUSG00000028533	0	0	0	0	0	0	0	0	0	0	0	0	Izumo3	IZUMO family member 3 [Source:MGI Symbol;Acc:MGI:1916564]
ENSMUSG00000028536	0	0.234000074	0	0.094813407	0.053374638	0.038694239	1.451328607	1.509770285	1.182088298	2.590099272	1.691469916	1.575739863	2610528J11	RIKEN cDNA 2610528J11 gene [Source:MGI Symbol;Acc:MGI:1913701]
ENSMUSG00000028538	35.02678943	32.30973214	40.74899208	31.93971902	30.23831731	25.77488966	8.272904986	7.82454073	7.543751397	9.512322823	9.367821061	9.012816105	St3gal3	ST3 beta-galactoside alpha-2,3-sialyltransferase 3 [Source:MGI Symbol;Acc:MGI:1316659]
ENSMUSG00000028539	0.343008317	1.18497513	1.053508198	0.711310502	0.800855949	0.522526458	1.190894502	2.114302455	1.880075315	0.681806313	1.046906015	0.844395587	Artn	artemin [Source:MGI Symbol;Acc:MGI:1333791]
ENSMUSG00000028540	10.34254728	11.33206468	10.0301523	11.89129364	11.60010603	10.10476904	7.928420693	8.213905743	9.849816678	10.22702555	10.40723613	10.17135287	Dph2	DPH2 homolog [Source:MGI Symbol;Acc:MGI:1914978]
ENSMUSG00000028541	66.91721351	67.22604294	70.47377819	73.70423774	69.62685819	65.21920495	7.02345158	7.318149995	7.34457684	9.37527386	9.643004937	9.926458467	B4galt2	UDP-Gal:betaGlcNAc beta 1,4- galactosyltransferase, polypeptide 2 [Source:MGI Symbol;Acc:MGI:1858493]
ENSMUSG00000028542	5.810830043	8.062404716	3.453371392	9.839982711	11.88281885	13.86094944	2.075110694	0.730086983	1.081696451	1.724098625	1.816798032	2.009639246	Slc6a9	solute carrier family 6 (neurotransmitter transporter, glycine), member 9 [Source:MGI Symbol;Acc:MGI:95760]
ENSMUSG00000028544	0	0.305233156	0.045228183	0.12367601	0.092830222	0.050473338	0.019720151	0	0.020559113	0.019757683	0.036772973	0.039150826	Slc5a9	solute carrier family 5 (sodium/glucose cotransporter), member 9 [Source:MGI Symbol;Acc:MGI:2140201]
ENSMUSG00000028545	7.728856532	5.985498211	7.357466157	5.947101234	4.41635883	3.373796242	2.582511198	2.865603207	3.239269608	3.436425569	3.348426384	3.084279056	Bend5	BEN domain containing 5 [Source:MGI Symbol;Acc:MGI:1914871]
ENSMUSG00000028546	0.016794882	0.25142221	0.103166862	0.015672708	0.105874282	0.076754147	1.049586177	0.815248453	0.87539129	0.781176523	0.699002254	0.952578415	Elavl4	ELAV like RNA binding protein 4 [Source:MGI Symbol;Acc:MGI:107427]
ENSMUSG00000028549	5.233559021	4.908353272	5.496174163	6.394346941	6.43403653	4.705486348	10.11551016	11.06401313	8.913760997	5.887814291	5.187271621	5.8813132	Itgb3bp	integrin beta 3 binding protein (beta3-endonexin) [Source:MGI Symbol;Acc:MGI:1914983]
ENSMUSG00000028550	16.45437616	20.05468089	12.30812642	20.09504041	24.025934	29.00963905	7.266255442	6.158793856	7.976973207	8.385254296	8.537933398	7.803862445	Atg4c	autophagy related 4C, cysteine peptidase [Source:MGI Symbol;Acc:MGI:2651854]
ENSMUSG00000028551	58.46967798	65.19818881	58.6296298	78.98272135	72.82860452	67.43497483	1.889607132	1.953783964	1.67897505	0.559355576	1.041071842	0.809977853	Cdkn2c	cyclin dependent kinase inhibitor 2C [Source:MGI Symbol;Acc:MGI:105388]
ENSMUSG00000028552	88.32284981	106.2878379	74.94326928	114.2248854	135.2848602	151.2958685	28.27960778	26.68410133	34.21716868	43.57557504	43.13036967	42.35395701	Eps15	epidermal growth factor receptor pathway substrate 15 [Source:MGI Symbol;Acc:MGI:104583]
ENSMUSG00000028553	0.028308216	0.032598366	0.086945231	0.079250285	0.148711574	0.064685567	0.050545861	0.028043321	0	0	0.070691202	0.075262312	Angptl3	angiopoietin-like 3 [Source:MGI Symbol;Acc:MGI:1353627]
ENSMUSG00000028555	1.791298713	0.859488562	1.260818956	1.636785306	1.607581138	1.563374792	4.364567115	3.696951232	3.021922101	3.805401705	4.162585702	3.406495389	Ttc39a	tetratricopeptide repeat domain 39A [Source:MGI Symbol;Acc:MGI:2444350]
ENSMUSG00000028556	146.4748856	193.1460986	121.6278234	222.3793645	268.3607194	358.7950135	9.848507102	9.621127796	12.82697243	30.65388086	30.45823692	29.14554112	Dock7	dedicator of cytokinesis 7 [Source:MGI Symbol;Acc:MGI:1914549]
ENSMUSG00000028557	132.1589269	154.9257092	109.0026029	180.4200743	191.0894283	202.813178	14.66874482	14.07457235	17.84760032	33.61213236	34.69055864	32.13716496	Rnf11	ring finger protein 11 [Source:MGI Symbol;Acc:MGI:1352759]
ENSMUSG00000028558	2.589026083	2.462892863	2.016780585	3.886667573	2.040139939	3.601069632	0.125620844	0.195147766	0.314316428	1.006879434	0.726176023	1.596145128	Calr4	calreticulin 4 [Source:MGI Symbol;Acc:MGI:2140435]
ENSMUSG00000028559	160.6047278	175.663998	136.169207	204.7853126	219.1979977	227.1687409	41.88919302	40.05571962	44.95575059	46.63487394	47.44185238	46.84061096	Osbpl9	oxysterol binding protein-like 9 [Source:MGI Symbol;Acc:MGI:1923784]
ENSMUSG00000028560	52.89998328	66.1406436	42.43788561	86.93872987	96.95277911	118.6083909	77.08040749	73.05454019	98.33353419	79.3138062	77.06381943	77.72067924	Usp1	ubiquitin specific peptidase 1 [Source:MGI Symbol;Acc:MGI:2385198]
ENSMUSG00000028563	62.65647656	63.14887442	59.38937415	69.69460066	69.9666223	62.00828047	20.06163281	21.82746712	17.60737792	17.65458178	15.6101503	16.71645758	Tm2d1	TM2 domain containing 1 [Source:MGI Symbol;Acc:MGI:2137022]
ENSMUSG00000028565	8.031429546	7.930797713	8.336128584	8.103184115	7.928215726	8.415379574	0.111455089	0.061836336	0.109741389	0.564539803	0.577319076	0.528599259	Nfia	nuclear factor I/A [Source:MGI Symbol;Acc:MGI:108056]
ENSMUSG00000028567	239.0526436	197.4585175	252.5291449	245.6231113	229.8305782	190.6988939	80.04932278	89.6562032	76.32788924	48.38613453	47.53651901	43.95572481	Txndc12	thioredoxin domain containing 12 (endoplasmic reticulum) [Source:MGI Symbol;Acc:MGI:1913323]
ENSMUSG00000028568	126.1298938	140.5597321	112.9796783	185.9023639	179.2604595	194.8022297	46.26231311	46.92265483	49.48088747	57.54311475	55.05127248	56.26389327	Btf3l4	basic transcription factor 3-like 4 [Source:MGI Symbol;Acc:MGI:1915312]
ENSMUSG00000028571	0	0	0	0.038758183	0.021818686	0	0.315179787	0.102861602	0.212616152	0.780161481	0.518584104	0.294462584	Cyp2j13	cytochrome P450, family 2, subfamily j, polypeptide 13 [Source:MGI Symbol;Acc:MGI:2385197]
ENSMUSG00000028572	5.530388225	7.112513053	3.413057223	7.066047451	7.331078651	11.10183041	38.85322178	33.53745375	50.19974728	88.88061904	85.96050115	83.36562947	Hook1	hook microtubule tethering protein 1 [Source:MGI Symbol;Acc:MGI:1925213]
ENSMUSG00000028573	87.38422254	79.229922	82.71303097	111.1112655	107.1054786	98.38728384	6.154021398	8.482755761	9.524126581	10.2634577	9.30169602	8.613103845	Fggy	FGGY carbohydrate kinase domain containing [Source:MGI Symbol;Acc:MGI:1922828]
ENSMUSG00000028575	1.345841889	1.461245769	1.732170696	0.430600299	1.373621827	1.464433451	0.034329628	0	0	0.034394965	0.096023795	0.170388314	Eqtn	equatorin, sperm acrosome associated [Source:MGI Symbol;Acc:MGI:1915003]
ENSMUSG00000028576	68.8103834	68.86943583	63.93131607	83.07144187	84.68470267	84.73882773	16.34006063	16.56219031	18.01802318	20.33277024	20.16682968	18.94948024	Ift74	intraflagellar transport 74 [Source:MGI Symbol;Acc:MGI:1914944]
ENSMUSG00000028577	158.4738699	172.2533043	137.7998074	192.8267663	218.5691424	230.9465669	90.36788281	87.11374542	104.4729637	114.3278907	109.8398277	117.6627466	Plaa	phospholipase A2, activating protein [Source:MGI Symbol;Acc:MGI:104810]

ENSMUSG00000028578	18.10859363	21.35060429	16.0745418	26.02006813	27.65252949	28.27562565	11.33522954	13.08741952	13.77108558	15.314198	14.01157539	16.83245865	Caap1	caspase activity and apoptosis inhibitor 1 [Source:MGI Symbol;Acc:MGI:1915020]
ENSMUSG00000028580	13.84949371	17.72198839	11.96161492	15.34949782	19.54475955	26.04691461	36.05152412	32.30314521	42.17704268	55.33183309	54.24614027	56.81546873	Pum1	pumilio RNA-binding family member 1 [Source:MGI Symbol;Acc:MGI:1931749]
ENSMUSG00000028581	35.31921709	35.44166743	31.2648609	0.892299507	1.255786621	2.275973655	170.5192689	149.72329	179.4980722	119.2592402	120.6332264	114.0279775	Laptm5	lysosomal-associated protein transmembrane 5 [Source:MGI Symbol;Acc:MGI:108046]
ENSMUSG00000028582	94.1784495	90.5937369	89.42230275	105.5315764	105.8080727	96.26784979	43.77761148	40.80423028	42.81608008	39.31035882	43.08739507	42.1254524	Cc2d1b	coiled-coil and C2 domain containing 1B [Source:MGI Symbol;Acc:MGI:2443076]
ENSMUSG00000028583	118.6785734	164.9345892	90.59737967	140.0080181	169.0931287	166.6901933	12.89428823	9.483425031	11.89056204	42.90364003	42.89695552	43.33553355	Pdpn	podoplanin [Source:MGI Symbol;Acc:MGI:103098]
ENSMUSG00000028584	0	0	0	0	0	0	0.016529627	0	0	0	0	0.04922492	Lrrc38	leucine rich repeat containing 38 [Source:MGI Symbol;Acc:MGI:2442845]
ENSMUSG00000028587	16.070881	18.95418218	14.63985423	21.28618102	22.92188832	25.76511863	47.86429107	49.90134379	54.28348851	53.94498098	53.25782388	54.28961732	Orc1	origin recognition complex, subunit 1 [Source:MGI Symbol;Acc:MGI:1328337]
ENSMUSG00000028589	0	0	0	0	0	0	0	0	0	0.039515366	0	0	1700012P22	RIKEN cDNA 1700012P22 gene [Source:MGI Symbol;Acc:MGI:1916614]
ENSMUSG00000028590	0.112199447	0	0	0	0	0	0.100169111	0.166724307	0.417722571	0.150539634	0.093394721	0.248584776	Pramef12os	PRAME family member 12, opposite strand [Source:MGI Symbol;Acc:MGI:1925973]
ENSMUSG00000028591	2.8666922	1.909484926	2.014145992	2.334199561	1.801245865	2.633053258	25.14153088	23.74908432	25.76643648	23.85700855	23.06691001	25.25588854	Pramef12	PRAME family member 12 [Source:MGI Symbol;Acc:MGI:1924882]
ENSMUSG00000028593	0	0	0	0	0	0	0	0	0	0	0	0	Aadacl4fm1	AADACL4 family member 1 [Source:MGI Symbol;Acc:MGI:2685880]
ENSMUSG00000028597	31.55422441	24.22420764	41.33297549	8.278556541	5.469648393	5.462336773	14.93910992	17.52395961	13.49804737	19.29149919	19.63295397	18.8781417	Gpx7	glutathione peroxidase 7 [Source:MGI Symbol;Acc:MGI:1914555]
ENSMUSG00000028599	21.11412207	24.9756023	21.59547601	9.746361573	10.56369861	9.768127276	13.72089924	11.54577086	13.57889687	30.2103923	30.19753736	31.45919176	Tnfrsf1b	tumor necrosis factor receptor superfamily, member 1b [Source:MGI Symbol;Acc:MGI:1314883]
ENSMUSG00000028600	1.277855757	2.181903914	1.669158482	0.740156273	1.157407024	0.906194119	0.295044962	0.305561632	0.41012956	1.714517703	1.888959639	1.405821371	Podn	podocan [Source:MGI Symbol;Acc:MGI:2674939]
ENSMUSG00000028601	33.95925152	28.1399474	36.41790239	36.43904604	33.61508449	27.63149281	88.07527432	100.8745178	87.97682466	61.96832993	68.06883946	62.42365428	Echdc2	enoyl Coenzyme A hydratase domain containing 2 [Source:MGI Symbol;Acc:MGI:1289238]
ENSMUSG00000028602	0.038776048	0.133957837	0.052931511	0.13267896	0.108641198	0.009845005	0.876998771	0.755459602	1.215068881	1.745774383	1.656893609	1.672395339	Tnfrsf8	tumor necrosis factor receptor superfamily, member 8 [Source:MGI Symbol;Acc:MGI:99908]
ENSMUSG00000028603	263.5277124	256.3736072	270.5557229	261.729147	258.8496527	241.0947186	112.4062177	112.6478627	105.392504	88.85733937	86.60578494	88.38497139	Scp2	sterol carrier protein 2, liver [Source:MGI Symbol;Acc:MGI:98254]
ENSMUSG00000028607	107.8134475	95.14318455	108.9357589	115.9806699	110.4063947	98.82524544	27.5234369	28.35905937	30.37504737	22.84853684	24.23229753	21.27168174	Cpt2	carnitine palmitoyltransferase 2 [Source:MGI Symbol;Acc:MGI:109176]
ENSMUSG00000028608	93.77582653	81.08532375	99.03349377	100.4986377	99.34614233	89.4405525	33.28309876	34.85720561	29.46364105	23.57755192	23.23995737	20.449134	Czib	CXXC motif containing zinc binding protein [Source:MGI Symbol;Acc:MGI:1921348]
ENSMUSG00000028609	194.5063276	157.0753352	219.1374691	215.0396287	196.9791014	158.4635045	177.8320285	188.5541324	153.1811935	118.1475838	113.4810187	119.5868644	Magoh	mago homolog, exon junction complex core component [Source:MGI Symbol;Acc:MGI:1330312]
ENSMUSG00000028610	2.919434795	2.139377698	2.615283936	4.55093388	2.335356461	3.512404679	1.243965976	1.347463189	1.327766781	1.750801863	2.485365299	1.264236624	Dmrtb1	DMRT-like family B with proline-rich C-terminal, 1 [Source:MGI Symbol;Acc:MGI:1927125]
ENSMUSG00000028612	0	0	0	0	0	0	0	0	0	0	0	0	Lrp8os1	low density lipoprotein receptor-related protein 8, apolipoprotein e receptor, opposite strand 1 [Source:MGI Symbol;Acc:MGI:192535
ENSMUSG00000028613	11.4940306	13.33639172	10.32111902	16.39017113	20.19439271	22.45159906	8.128315292	6.462126891	8.603992828	4.087493794	4.515220216	4.822645425	Lrp8	low density lipoprotein receptor-related protein 8, apolipoprotein e receptor [Source:MGI Symbol;Acc:MGI:1340044]
ENSMUSG00000028614	61.06887217	67.14186658	50.86216227	79.95699417	88.8820424	95.81139152	52.5649127	51.11191096	59.63281919	65.21768247	64.32250803	63.75891633	Ndc1	NDC1 transmembrane nucleoporin [Source:MGI Symbol;Acc:MGI:1920037]
ENSMUSG00000028617	231.5791121	211.8814445	257.614558	245.2195362	218.1218863	190.7432053	82.62464963	81.51804451	78.83154156	61.7586711	61.61199748	61.79200614	Lrrc42	leucine rich repeat containing 42 [Source:MGI Symbol;Acc:MGI:1925059]
ENSMUSG00000028618	700.136669	625.3853454	692.8772206	693.8431987	623.1484546	561.2740173	77.14894516	79.54463361	71.40804394	92.7904711	98.19049243	95.84281308	Tmem59	transmembrane protein 59 [Source:MGI Symbol;Acc:MGI:1929278]
ENSMUSG00000028619	7.694749628	5.92051149	7.807175055	3.767407303	3.516605357	3.390415928	6.222775078	6.631447036	6.872909724	7.530925256	7.238053425	5.534928427	Tceanc2	transcription elongation factor A (SII) N-terminal and central domain containing 2 [Source:MGI Symbol;Acc:MGI:1913776]
ENSMUSG00000028621	8.012350527	8.852580798	10.64171117	10.34654285	9.828884513	8.626478507	2.648633622	2.123784058	2.579915146	4.048306478	3.118429521	3.166547194	Cyb5rl	cytochrome b5 reductase-like [Source:MGI Symbol;Acc:MGI:1919657]
ENSMUSG00000028622	97.44776768	93.01523692	103.6744228	111.312622	98.91926577	95.13537573	74.75765072	73.75572512	69.15513531	58.1007129	61.44030378	57.4293994	Mrpl37	mitochondrial ribosomal protein L37 [Source:MGI Symbol;Acc:MGI:1926268]
ENSMUSG00000028626	0.278802297	0.178363987	0.285435746	0.231265531	0.227831698	0.448312737	3.954876985	3.252944461	3.286971399	7.564589503	7.452179275	6.973187754	Col9a2	collagen, type IX, alpha 2 [Source:MGI Symbol;Acc:MGI:88466]
ENSMUSG00000028629	25.86024448	25.66210587	26.60175281	29.13715834	27.39701814	25.21439697	12.24542268	11.20607887	11.76228662	16.54210589	13.88032209	14.39544468	Exo5	exonuclease 5 [Source:MGI Symbol;Acc:MGI:1920422]
ENSMUSG00000028630	15.77339644	21.52280342	15.10254173	19.46992902	22.21375919	27.41074178	6.253195158	6.736209093	7.91990993	12.54681114	12.00081838	11.54195431	Dyrk2	dual-specificity tyrosine-(Y)-phosphorylation regulated kinase 2 [Source:MGI Symbol;Acc:MGI:1330301]
ENSMUSG00000028631	0.388143576	0.361830655	0.643374788	0.189728632	0.194193807	0.098547313	0.379528197	0.494371945	0.49889407	0.611707369	0.492328185	0.425882956	Kcnq4	potassium voltage-gated channel, subfamily Q, member 4 [Source:MGI Symbol;Acc:MGI:1926803]
ENSMUSG00000028633	135.6427802	149.6553585	120.3284976	133.8060742	143.7283329	146.484175	103.4524844	101.7390516	117.3573831	102.1411415	103.9937001	101.5586068	Ctps	cytidine 5'-triphosphate synthase [Source:MGI Symbol;Acc:MGI:1858304]
ENSMUSG00000028634	3.141664824	2.997089528	2.853778556	2.126955877	2.443255573	3.120218142	0.893688445	0.732298028	0.530357411	1.033141545	0.884525558	0.73699959	Hivep3	human immunodeficiency virus type I enhancer binding protein 3 [Source:MGI Symbol;Acc:MGI:106589]
ENSMUSG00000028635	0.441468753	0.372807573	0.482103969	0.219718673	0.742133817	0.896690031	0.4204088	0.262402619	0.191753803	0.236930026	0.53896803	0.208661585	Edn2	endothelin 2 [Source:MGI Symbol;Acc:MGI:95284]
ENSMUSG00000028636	11.14218268	11.01938961	12.14540333	10.3977018	9.709631207	8.486798946	18.25656431	17.95285132	17.05062887	17.17741683	18.49492885	20.67831541	Ppcs	phosphopantothenoylcysteine synthetase [Source:MGI Symbol;Acc:MGI:1915237]
ENSMUSG00000028637	1.034062458	0.562310925	0.882221299	0.482485047	0.181074706	0.546962961	2.179748061	1.764220666	1.89819076	2.800526258	3.729928288	3.43654348	Ccdc30	coiled-coil domain containing 30 [Source:MGI Symbol;Acc:MGI:1920582]
ENSMUSG00000028639	1607.02048	1547.883788	1600.696534	1724.597008	1554.830847	1454.630062	2445.28722	2473.389342	2283.825912	1804.111119	1820.248421	1550.526816	Ybx1	Y box protein 1 [Source:MGI Symbol;Acc:MGI:99146]
ENSMUSG00000028640	0.226863732	0.108852187	0.15484095	0.035284251	0.019863057	0.172798	6.481240757	5.150311124	6.91534119	13.93413194	12.77825259	14.4254969	Tfap2c	transcription factor AP-2, gamma [Source:MGI Symbol;Acc:MGI:106032]
ENSMUSG00000028641	92.11838003	89.36147426	92.34373143	74.976436	69.89599133	66.49801289	33.87188002	34.34778452	37.86676298	37.64011552	39.11825704	38.8402977	P3h1	prolyl 3-hydroxylase 1 [Source:MGI Symbol;Acc:MGI:1888921]
ENSMUSG00000028642	0.116450576	0.435821179	0.029805311	0.380344174	0.275287459	0.133047445	0.181937739	0.057680443	0.298065441	0.182284009	0.024233339	0.283803756	Tmem269	transmembrane protein 269 [Source:MGI Symbol;Acc:MGI:1922430]
ENSMUSG00000028643	130.0177474	105.6230508	167.7656528	120.1927437	93.27983019	68.77081997	75.16506447	69.40908867	51.66340546	33.09965108	36.77199216	37.3702466	Svbp	small vasohibin binding protein [Source:MGI Symbol;Acc:MGI:1916466]
ENSMUSG00000028644	0.321748413	0.29640787	0.043920489	0.140116822	0.112682748	0.408449908	0	0.021249176	0.059894045	0.191864239	0.035709746	0.076037696	Ermap	erythroblast membrane-associated protein [Source:MGI Symbol;Acc:MGI:1349816]
ENSMUSG00000028645	152.9835982	163.0393904	144.6175041	154.9211265	161.7605279	162.539293	668.0430369	649.7141425	642.1094521	395.1572487	382.8190476	387.1552718	Slc2a1	solute carrier family 2 (facilitated glucose transporter), member 1 [Source:MGI Symbol;Acc:MGI:95755]
ENSMUSG00000028646	146.9939682	138.7524844	135.4176015	135.181506	131.583988	130.9129875	28.99892266	30.0345533	33.61667607	38.40733516	36.54221723	37.94650083	Rragc	Ras-related GTP binding C [Source:MGI Symbol;Acc:MGI:1858751]
ENSMUSG00000028647	25.95716509	21.62435626	23.93072391	26.91876083	27.18017919	25.53906389	20.98553066	19.99035177	19.31159494	18.71540989	17.89021959	19.74531891	Mycbp	MYC binding protein [Source:MGI Symbol;Acc:MGI:1891750]
ENSMUSG00000028648	46.14186635	36.95529737	75.59987279	32.10788302	27.51081339	17.6335176	15.75080288	11.30889837	9.807955475	22.99281108	20.53323659	19.1725736	Ndufs5	NADH:ubiquinone oxidoreductase core subunit S5 [Source:MGI Symbol;Acc:MGI:1890889]
ENSMUSG00000028649	25.47588016	31.24200902	21.9956602	29.30148673	35.96656254	49.44661371	15.79454443	14.05532793	19.72360703	28.22095976	29.75327166	29.31785701	Macf1	microtubule-actin crosslinking factor 1 [Source:MGI Symbol;Acc:MGI:108559]
ENSMUSG00000028651	38.1932775	35.09523014	50.7025899	43.84522778	43.97676371	34.48526349	62.22247078	63.43074499	47.77713763	32.37183097	31.71074106	32.46274519	Ppie	peptidylprolyl isomerase E (cyclophilin E) [Source:MGI Symbol;Acc:MGI:1917118]
ENSMUSG00000028653	27.99546308	26.26091665	25.8157221	30.09525662	28.25147383	25.6877459	21.8536711	21.80465248	23.20580368	16.94789818	18.81743557	18.60141692	Trit1	tRNA isopentenyltransferase 1 [Source:MGI Symbol;Acc:MGI:1914216]
ENSMUSG00000028654	1.830465974	2.512125792	1.360588147	2.433544111	3.056040168	2.864861944	8.77542121	6.657183673	9.24211189	16.01422377	17.40750242	16.86647707	Mycl	v-myc avian myelocytomatosis viral oncogene lung carcinoma derived [Source:MGI Symbol;Acc:MGI:96799]
ENSMUSG00000028655	1.159134774	0.728074515	0.61133735	0.721123684	0.774999743	1.070172102	0.376308775	0.382762714	0.261545479	1.445262411	2.456014319	1.7432185	Mfsd2a	major facilitator superfamily domain containing 2A [Source:MGI Symbol;Acc:MGI:1923824]
ENSMUSG00000028656	401.2304596	435.3518335	382.4617554	447.4205862	448.5044465	454.9113893	156.544429	150.8343596	165.5346865	259.4682659	267.051399	251.4501279	Cap1	CAP, adenylate cyclase-associated protein 1 (yeast) [Source:MGI Symbol;Acc:MGI:88262]
ENSMUSG00000028657	77.9001451	82.81410912	74.24397748	68.70906387	66.43061922	63.36065571	19.08892917	17.0216509	18.84533827	28.86057826	30.09180004	29.53535691	Ppt1	palmitoyl-protein thioesterase 1 [Source:MGI Symbol;Acc:MGI:1298204]
ENSMUSG00000028661	0.401708777	0.294374377	0.15889842	0.238552383	0.134291631	0.208618846	0.187469052	0.081398818	0.135961473	0.253156579	0.212788566	0.291276166	Epha8	Eph receptor A8 [Source:MGI Symbol;Acc:MGI:109378]
ENSMUSG00000028664	11.04516024	12.56598169	12.07920867	8.435952089	8.066262774	9.256720041	8.416848819	7.846931284	8.692439427	7.858124957	7.949059388	8.237257218	Ephb2	Eph receptor B2 [Source:MGI Symbol;Acc:MGI:99611]
ENSMUSG00000028668	8.015289682	9.935162011	6.849245358	7.411506423	8.719021466	10.50467229	8.265558023	8.836624332	10.4801518	13.50512329	12.35484405	13.32383777	Eloa	elongin A [Source:MGI Symbol;Acc:MGI:1351315]
ENSMUSG00000028669	26.06040855	23.44753864	28.05708792	24.08013626	22.60415917	22.53213919	91.1209485	92.73659128	78.13160364	42.38258202	42.53253963	42.33206384	Pithd1	PITH (C-terminal proteasome-interacting domain of thioredoxin-like) domain containing 1 [Source:MGI Symbol;Acc:MGI:1913443]
ENSMUSG00000028670	61.94127418	46.61940305	64.50369204	49.3324282	39.18320266	31.9062178	66.14949717	65.08624583	59.1016605	45.82547805	46.65993663	46.84995881	Lypla2	lysophospholipase 2 [Source:MGI Symbol;Acc:MGI:1347000]
ENSMUSG00000028671	22.56315071	19.75538651	23.52953342	20.31600666	19.34700514	14.30978366	43.65891866	47.15166902	41.82814959	20.43239818	21.34266686	20.69835888	Gale	galactose-4-epimerase, UDP [Source:MGI Symbol;Acc:MGI:1921496]
ENSMUSG00000028672	56.51370463	45.87161343	61.67315028	51.41300976	46.32687468	38.21383231	75.16638456	76.81441421	62.46841882	32.45686236	30.20433417	29.52351693	Hmgcl	3-hydroxy-3-methylglutaryl-Coenzyme A lyase [Source:MGI Symbol;Acc:MGI:96158]
ENSMUSG00000028673	128.7100786	108.60908	137.6812967	91.21013558	77.84507598	68.22857731	54.41755228	60.38274391	46.77496323	62.49572729	55.26813392	61.75285001	Fuca1	fucosidase, alpha-L- 1, tissue [Source:MGI Symbol;Acc:MGI:95593]
ENSMUSG00000028675	51.94729236	51.02452877	48.81203026	49.09614875	53.73160659	53.29843488	56.5422604	53.24706223	58.24126886	55.75875172	54.53484569	54.52963328	Pnrc2	proline-rich nuclear receptor coactivator 2 [Source:MGI Symbol;Acc:MGI:106512]
ENSMUSG00000028676	59.00268972	68.37375424	50.79234622	75.3689125	82.50779117	96.2027257	131.9900673	121.5279653	150.8780102	149.2186456	146.98981	146.432996	Srsf10	serine and arginine-rich splicing factor 10 [Source:MGI Symbol;Acc:MGI:1333805]
ENSMUSG00000028677	93.90139998	82.7858061	99.38865372	104.5534788	87.59981071	84.4561301	144.0261529	144.8295148	132.0389719	86.53871208	86.84214732	84.71371442	Rnf220	ring finger protein 220 [Source:MGI Symbol;Acc:MGI:1913993]
ENSMUSG00000028678	53.75795652	51.46016533	50.92243832	57.25397354	61.5174032	56.11181939	98.88450868	100.1543054	101.4441233	95.08209687	90.87726503	91.42747842	Kif2c	kinesin family member 2C [Source:MGI Symbol;Acc:MGI:1921054]

ENSMUSG00000028680	39.36648886	41.15772532	36.72270559	60.98202655	55.4619497	54.79537323	17.42563168	16.66942725	17.17852967	12.00496994	11.56808872	11.69949969	Plk3	polo like kinase 3 [Source:MGI Symbol;Acc:MGI:109604]
ENSMUSG00000028681	3.047684133	3.509564946	2.853337734	1.683976237	2.022366524	2.015922984	13.98042918	13.10953378	17.20657861	12.9488871	14.32007417	12.59842937	Ptch2	patched 2 [Source:MGI Symbol;Acc:MGI:1095405]
ENSMUSG00000028683	26.55798057	24.44661532	31.56591703	29.83589545	29.40037657	24.35261666	54.87472863	51.65913993	48.72204725	42.13285325	43.59696499	42.70380254	Eif2b3	eukaryotic translation initiation factor 2B, subunit 3 [Source:MGI Symbol;Acc:MGI:1313286]
ENSMUSG00000028684	142.4503726	113.5134358	155.5845382	117.4185034	105.4860761	89.33403504	118.7368217	133.302651	116.2032199	87.70591125	89.92206465	90.47898294	Urod	uroporphyrinogen decarboxylase [Source:MGI Symbol;Acc:MGI:98916]
ENSMUSG00000028687	7.423337869	5.87941169	7.531180947	9.027479794	8.928731102	7.880092394	22.8210023	24.42417815	21.60340648	12.58896012	11.85507822	11.93699827	Mutyh	mutY DNA glycosylase [Source:MGI Symbol;Acc:MGI:1917853]
ENSMUSG00000028688	8.442840739	9.32754826	9.345746307	10.99821355	10.40151548	10.54383333	11.47854205	9.807341453	8.815600086	13.60879289	12.27191749	11.96400894	Toe1	target of EGR1, member 1 (nuclear) [Source:MGI Symbol;Acc:MGI:1915526]
ENSMUSG00000028689	25.14586832	23.36152949	30.56477429	30.6643556	24.47248898	24.53282336	29.33660567	29.87282924	27.13458229	16.48844194	15.63600501	13.89476038	Ccdc163	coiled-coil domain containing 163 [Source:MGI Symbol;Acc:MGI:1915644]
ENSMUSG00000028690	6.327718798	7.938660785	6.785137608	10.31807635	8.572784826	9.335015166	18.34517412	20.38914387	22.75238175	18.79714147	19.73809237	21.07344733	Mmachc	methylmalonic aciduria cblC type, with homocystinuria [Source:MGI Symbol;Acc:MGI:1914346]
ENSMUSG00000028691	2147.895964	1830.00767	2210.775547	1997.411022	1779.878936	1500.729037	1446.795862	1539.070011	1336.763053	1202.021408	1135.536084	1176.312798	Prdx1	peroxiredoxin 1 [Source:MGI Symbol;Acc:MGI:99523]
ENSMUSG00000028692	918.8397472	817.4635209	927.1581781	833.6291376	761.0633442	644.5283591	328.5320477	342.9653627	316.3448017	292.3091915	294.0155543	293.7858371	Akr1a1	aldo-keto reductase family 1, member A1 (aldehyde reductase) [Source:MGI Symbol;Acc:MGI:1929955]
ENSMUSG00000028693	103.1195385	91.87624824	92.73012271	113.540841	109.5163833	108.6056953	501.4336696	506.5604137	536.050101	488.2194968	462.4336925	460.1621735	Nasp	nuclear autoantigenic sperm protein (histone-binding) [Source:MGI Symbol;Acc:MGI:1355328]
ENSMUSG00000028696	3.353661073	4.423645939	3.82882099	4.457282613	5.231949927	6.517648884	5.697810193	5.214971448	5.429431501	4.308761469	4.957177381	4.575228054	Ipp	IAP promoted placental gene [Source:MGI Symbol;Acc:MGI:96581]
ENSMUSG00000028698	10.5004085	10.35619206	9.563306048	9.010576435	10.28410937	10.62191123	2.39538351	2.313412058	3.129321693	5.762824834	4.839000498	4.99044942	Pik3r3	phosphoinositide-3-kinase regulatory subunit 3 [Source:MGI Symbol;Acc:MGI:109277]
ENSMUSG00000028699	0.069066594	0.238601199	0.035354936	0.032225905	0.072565519	0.052606775	3.329698427	3.626275968	3.085648913	1.482682493	0.948601036	1.315983067	Tspan1	tetraspanin 1 [Source:MGI Symbol;Acc:MGI:1914055]
ENSMUSG00000028700	52.69467525	58.54789464	53.11724744	55.30573362	50.51283212	48.85184208	28.44873627	24.15297149	30.20216085	19.72054409	19.45008391	19.13390442	Pomgnt1	protein O-linked mannose beta 1,2-N-acetylglucosaminyltransferase [Source:MGI Symbol;Acc:MGI:1915523]
ENSMUSG00000028701	1.0406761	1.661770276	0.880760888	1.37254713	1.297497689	1.79670542	1.610426001	1.347092252	1.885577595	1.563845135	1.859566898	1.832248565	Lurap1	leucine rich adaptor protein 1 [Source:MGI Symbol;Acc:MGI:1915325]
ENSMUSG00000028702	8.4599755	9.182741842	8.143242863	10.89772188	12.35467214	12.70263076	39.58934397	40.23992302	44.28765533	29.25519927	31.97574302	27.26341257	Rad54l	RAD54 like (S. cerevisiae) [Source:MGI Symbol;Acc:MGI:894697]
ENSMUSG00000028703	48.10790069	72.10270037	31.10158868	56.24221527	64.19478576	76.4479142	11.55497881	10.01538079	15.50143095	26.95468246	25.08400927	26.10004714	Lrrc41	leucine rich repeat containing 41 [Source:MGI Symbol;Acc:MGI:2441984]
ENSMUSG00000028706	14.22672916	18.54250242	15.82699151	17.42650937	18.69135952	19.18280162	11.86411433	11.33327333	12.71797464	12.7015082	13.0912241	10.85101958	Nsun4	NOL1/NOP2/Sun domain family, member 4 [Source:MGI Symbol;Acc:MGI:1919431]
ENSMUSG00000028707	0.027964788	0.032202891	0.038173527	0.017397521	0.029381489	0.028400365	0.19973061	0.184687382	0.112790071	0.175096901	0.162945057	0.181742613	Dmbx1	diencephalon/mesencephalon homeobox 1 [Source:MGI Symbol;Acc:MGI:2153518]
ENSMUSG00000028708	30.33470042	35.77104069	29.3681668	34.74957841	35.49779721	37.91504348	23.07630271	21.08497814	23.52262999	20.17016419	20.4303033	20.22199491	Mknk1	MAP kinase-interacting serine/threonine kinase 1 [Source:MGI Symbol;Acc:MGI:894316]
ENSMUSG00000028709	12.75652558	12.48252006	12.21417997	12.49013711	13.47221776	11.60431913	6.018917228	7.628127891	5.690547643	6.059933261	7.069844085	6.824091239	Mob3c	MOB kinase activator 3C [Source:MGI Symbol;Acc:MGI:2140623]
ENSMUSG00000028710	16.35862847	16.91230229	13.18143756	17.2940821	16.66031939	19.78328189	20.9739132	17.69635258	18.96109441	24.35410825	22.96548363	23.93185534	Atpaf1	ATP synthase mitochondrial F1 complex assembly factor 1 [Source:MGI Symbol;Acc:MGI:2180560]
ENSMUSG00000028712	0.132558496	0.122118338	0	0.024740282	0	0	0.047338083	0.052527231	0	0.023714089	0.022068315	0.070485961	Cyp4a31	cytochrome P450, family 4, subfamily a, polypeptide 31 [Source:MGI Symbol;Acc:MGI:3028580]
ENSMUSG00000028713	0.510219223	0.391695776	0.348239132	0.063483759	0	0.103633267	0.030367474	0	0	0.212976889	0.084941207	0.060289177	Cyp4b1	cytochrome P450, family 4, subfamily b, polypeptide 1 [Source:MGI Symbol;Acc:MGI:103225]
ENSMUSG00000028715	0.200665833	0.561187052	0.058697202	0.107004604	0.120475199	0	0.102371363	0.227186397	0.213453173	0.02564155	0.119310045	0.076214999	Cyp4a14	cytochrome P450, family 4, subfamily a, polypeptide 14 [Source:MGI Symbol;Acc:MGI:1096550]
ENSMUSG00000028716	0.279043371	1.028264916	0.114273023	0.781196067	0.175907853	0.085016915	1.893339111	1.548020409	0.415555398	0.399356331	0.232275457	0.593508244	Pdzk1ip1	PDZK1 interacting protein 1 [Source:MGI Symbol;Acc:MGI:1914432]
ENSMUSG00000028717	1.15321739	1.259002659	0.965990868	1.257853465	1.557822317	1.471579764	0.374387718	0.163203733	0.264856902	0.294721636	0.32413462	0.371639865	Tal1	T cell acute lymphocytic leukemia 1 [Source:MGI Symbol;Acc:MGI:98480]
ENSMUSG00000028718	5.726687903	7.877410858	4.294737984	10.39572242	10.58884155	14.26571448	9.168593812	8.242376699	12.18323703	10.43160901	10.93921561	11.0758194	Stil	Scl/Tal1 interrupting locus [Source:MGI Symbol;Acc:MGI:107477]
ENSMUSG00000028719	134.6639528	139.3495897	120.8541334	159.4125834	156.4052065	154.4212015	90.96971789	87.76364472	94.40416497	89.50284111	81.47283824	81.69361177	Cmpk1	cytidine monophosphate (UMP-CMP) kinase 1 [Source:MGI Symbol;Acc:MGI:1913838]
ENSMUSG00000028729	30.85475253	26.18504564	30.69060761	112.6480746	28.26960264	26.23035466	37.07669858	44.50906272	39.09641045	31.52285436	33.96060581	142.9740938	Ebna1bp2	EBNA1 binding protein 2 [Source:MGI Symbol;Acc:MGI:1916322]
ENSMUSG00000028730	0.110755438	0.06377029	0.079373424	0.051677578	0.116366328	0.101232496	0.02966398	0.032915713	0.010308662	0.059440875	0.119850549	0.058892517	Cfap57	cilia and flagella associated protein 57 [Source:MGI Symbol;Acc:MGI:2686209]
ENSMUSG00000028736	0.295182238	0.562171285	0.546294396	0.889945155	0.834982133	0.648562414	0.040543382	0	0.021134117	0.101551362	0.16065617	0.070430167	Pax7	paired box 7 [Source:MGI Symbol;Acc:MGI:97491]
ENSMUSG00000028737	3.933308139	3.506637302	4.270454037	3.167285019	2.316238942	2.23486687	12.8851888	13.10355952	11.26336256	9.150290568	9.228159219	10.93527455	Aldh4a1	aldehyde dehydrogenase 4 family, member A1 [Source:MGI Symbol;Acc:MGI:2443883]
ENSMUSG00000028738	0.432447114	0.248992541	0.300428124	0.417965132	0.243404442	0.211748877	0	0.015300017	0	0	0	0.027374662	Tas1r2	taste receptor, type 1, member 2 [Source:MGI Symbol;Acc:MGI:1933546]
ENSMUSG00000028741	64.7326431	45.48807992	72.43900915	57.87569378	47.83074287	41.94909325	175.3131865	204.260052	168.5801611	116.0948494	118.697035	121.2751395	Mrto4	mRNA turnover 4, ribosome maturation factor [Source:MGI Symbol;Acc:MGI:1917152]
ENSMUSG00000028743	32.69300181	26.44300784	46.66439368	26.5537572	21.94534448	17.81984439	45.51870648	46.48145201	37.07062166	26.84487303	25.01781755	25.02591634	Akr7a5	aldo-keto reductase family 7, member A5 (aflatoxin aldehyde reductase) [Source:MGI Symbol;Acc:MGI:107796]
ENSMUSG00000028744	9.680142727	10.24256772	10.81848547	7.307081656	7.02885515	6.330906224	8.506469453	8.284175348	8.090022952	7.72932658	7.888993775	6.939380182	Pqlc2	PQ loop repeat containing 2 [Source:MGI Symbol;Acc:MGI:2384837]
ENSMUSG00000028745	341.7341101	297.9785213	361.8515876	258.1182308	234.3460667	198.6079895	372.4882363	396.5711233	328.4581199	275.5405687	265.847064	262.322919	Capzb	capping protein (actin filament) muscle Z-line, beta [Source:MGI Symbol;Acc:MGI:104652]
ENSMUSG00000028747	0.44143697	0.187282186	0.451939932	0.260173722	0.317337008	0.212358665	0.497816683	0.506354562	0.389246613	0.519545982	0.328772615	0.391212305	Htr6	5-hydroxytryptamine (serotonin) receptor 6 [Source:MGI Symbol;Acc:MGI:1196627]
ENSMUSG00000028749	0.017455776	0	0	0	0.018340089	0.039887246	0.015584119	0	0.048741361	0.124910236	0.116241381	0.092818429	Pla2g2f	phospholipase A2, group IIF [Source:MGI Symbol;Acc:MGI:1349661]
ENSMUSG00000028750	0	0	0	0	0.023375552	0.033892472	0.238354834	0.110201261	0.124247626	0.179106359	0.148156672	0.078868469	Pla2g2c	phospholipase A2, group IIC [Source:MGI Symbol;Acc:MGI:106638]
ENSMUSG00000028751	2.553619456	1.396796434	2.549017445	1.965971467	1.945165033	2.188176452	0.45596266	0.25297239	0.178260318	0.285519038	0.425126118	0.792078139	Pla2g2e	phospholipase A2, group IIE [Source:MGI Symbol;Acc:MGI:1349660]
ENSMUSG00000028753	0	0.042440699	0.0943303	0.085981751	0	0.014035983	0	0	0	0.131864553	0.092034798	0.016331008	Vwa5b1	von Willebrand factor A domain containing 5B1 [Source:MGI Symbol;Acc:MGI:1922968]
ENSMUSG00000028755	13.46357537	11.39069325	15.07790939	15.58960059	13.85695582	11.3851004	1.030283443	2.613078786	0.664928442	0.589853891	0.823376448	1.168824665	Cda	cytidine deaminase [Source:MGI Symbol;Acc:MGI:1919519]
ENSMUSG00000028756	22.2817791	20.32869317	24.19301957	16.03968655	14.4910988	14.15483378	8.679680542	10.59885943	9.157184375	8.841830112	9.138143365	9.296183792	Pink1	PTEN induced putative kinase 1 [Source:MGI Symbol;Acc:MGI:1916193]
ENSMUSG00000028757	135.0526217	122.3078374	139.0347198	117.7898484	111.2934969	100.9501673	107.9058755	107.6101589	103.3343674	88.92172608	92.4987704	85.96092052	Ddost	dolichyl-di-phosphooligosaccharide-protein glycotransferase [Source:MGI Symbol;Acc:MGI:1194508]
ENSMUSG00000028758	0.733077941	0.532198501	0.587363336	0.698967948	0.736728151	0.801142727	1.308950583	0.852516954	1.260806622	0.270841245	0.23877917	0.409575643	Kif17	kinesin family member 17 [Source:MGI Symbol;Acc:MGI:1098229]
ENSMUSG00000028759	53.28641668	68.64343725	48.10072815	70.60654769	77.21202324	88.09322744	21.79405051	18.98576201	24.61619823	36.45675432	35.68651378	35.32238123	Hp1bp3	heterochromatin protein 1, binding protein 3 [Source:MGI Symbol;Acc:MGI:109369]
ENSMUSG00000028760	31.3725985	35.62584299	31.0982432	32.3167351	33.65540932	37.80136714	16.23604309	14.34309693	17.68488969	30.59944063	31.45666185	28.72413317	Eif4g3	eukaryotic translation initiation factor 4 gamma, 3 [Source:MGI Symbol;Acc:MGI:1923935]
ENSMUSG00000028763	39.09692165	49.60254521	38.97224489	34.5561636	38.87008188	50.26372877	26.67712475	21.11618917	31.88394658	46.25249524	53.20542947	51.07513602	Hspg2	perlecan (heparan sulfate proteoglycan 2) [Source:MGI Symbol;Acc:MGI:96257]
ENSMUSG00000028766	5.704534371	4.191688822	2.558590289	2.281447604	1.369949041	3.269125796	134.8885217	134.7127485	144.9253387	123.9195887	125.4495108	119.4542128	Alpl	alkaline phosphatase, liver/bone/kidney [Source:MGI Symbol;Acc:MGI:87983]
ENSMUSG00000028771	70.61667396	97.53018115	59.97615067	99.65973127	120.3567103	141.9342811	46.28702733	39.10181104	52.80006872	63.5711529	67.09757675	62.54869591	Ptpn12	protein tyrosine phosphatase, non-receptor type 12 [Source:MGI Symbol;Acc:MGI:104673]
ENSMUSG00000028772	44.46277076	33.56979684	50.29298564	32.1562435	25.31665828	21.74010036	46.89813435	54.5966066	46.55713865	38.48796637	37.9957354	37.62446596	Zcchc17	zinc finger, CCHC domain containing 17 [Source:MGI Symbol;Acc:MGI:1919955]
ENSMUSG00000028773	6.639876007	3.190916559	5.72732779	2.195512154	1.318347072	2.787583772	174.5705398	193.2406291	135.2814596	46.29779673	41.73558151	42.16396509	Fabp3	fatty acid binding protein 3, muscle and heart [Source:MGI Symbol;Acc:MGI:95476]
ENSMUSG00000028776	22.83722817	17.48379687	20.34917268	19.95915919	15.98507889	15.66722948	7.086781278	7.426758238	7.476232532	11.0166873	10.01613941	10.07758316	Tinagl1	tubulointerstitial nephritis antigen-like 1 [Source:MGI Symbol;Acc:MGI:2137617]
ENSMUSG00000028777	0.031615347	0.109220093	0.064735122	0	0.066433973	0	0	0	0.029426269	0.169675091	0.078949762	0.056036598	Gnat3	guanine nucleotide binding protein, alpha transducing 3 [Source:MGI Symbol;Acc:MGI:3588268]
ENSMUSG00000028778	0.171257663	0.271166503	0.175332339	0.25969911	0	0.11413838	0	0.021206889	0.039849902	0.019148242	0.035638682	0.018971594	Hcrtr1	hypocretin (orexin) receptor 1 [Source:MGI Symbol;Acc:MGI:2385650]
ENSMUSG00000028779	38.41031198	30.59318045	33.09638186	29.21727383	27.73020335	23.65856885	25.6246805	26.41671115	24.47319761	21.96456938	23.43700911	21.54190057	Pef1	penta-EF hand domain containing 1 [Source:MGI Symbol;Acc:MGI:1915148]
ENSMUSG00000028780	32.931312	40.6009643	26.40094596	39.9533263	46.47373523	61.91625755	2.224172916	2.010344811	2.625922128	23.06621604	22.35808025	24.41792593	Sema3c	sema domain, immunoglobulin domain (Ig), short basic domain, secreted, (semaphorin) 3C [Source:MGI Symbol;Acc:MGI:107557]
ENSMUSG00000028782	2.304306248	1.905096378	1.814716795	1.254365425	1.412274964	1.271357478	3.916653505	3.263152776	3.712073777	4.069445123	5.004280233	3.482098403	Adgrb2	adhesion G protein-coupled receptor B2 [Source:MGI Symbol;Acc:MGI:2451244]
ENSMUSG00000028784	0.90172543	1.411136125	1.254577786	0.906204228	0.947406994	0.704438918	0.288988693	0.22904808	0.086080928	0.165450688	0.250198485	0.348339269	Spocd1	SPOC domain containing 1 [Source:MGI Symbol;Acc:MGI:3652045]
ENSMUSG00000028785	0.107302962	0.411882923	0.622517251	0.400533445	0.112738949	0.245192174	2.267210839	3.188966162	2.829740548	1.535679456	1.756604866	2.313966785	Hpca	hippocalcin [Source:MGI Symbol;Acc:MGI:1336200]
ENSMUSG00000028786	0.14923175	0.171848031	0.254637284	0.324941439	0	0.151556165	0.799384247	0.689898113	0.555595166	1.601811317	1.573458114	1.631118466	Tmem54	transmembrane protein 54 [Source:MGI Symbol;Acc:MGI:1913510]
ENSMUSG00000028788	228.4756498	223.5121837	219.1103939	233.4612949	234.210584	225.7792292	172.8110888	170.3487252	180.0468024	189.1719857	179.0583855	181.5602233	Ptp4a2	protein tyrosine phosphatase 4a2 [Source:MGI Symbol;Acc:MGI:1277117]
ENSMUSG00000028789	41.50439212	36.13573151	41.0987154	29.79129001	27.76551367	25.22832582	18.41774787	18.44154322	17.42809377	24.80660198	25.28250034	23.78181153	Azin2	antizyme inhibitor 2 [Source:MGI Symbol;Acc:MGI:2442093]
ENSMUSG00000028790	62.14376452	67.26935573	59.78659456	79.0226152	81.5597427	83.99525769	108.7937338	102.4270148	113.4221861	98.79318956	98.84248355	100.8463712	Khdrbs1	KH domain containing, RNA binding, signal transduction associated 1 [Source:MGI Symbol;Acc:MGI:893579]

ENSMUSG00000028792	87.83501234	72.53271037	92.33679303	75.90998392	74.95800718	68.49364024	381.2515528	393.232252	347.9063173	220.8684434	223.1524546	223.1641581	Ak2	adenylate kinase 2 [Source:MGI Symbol;Acc:MGI:87978]
ENSMUSG00000028793	44.43840187	50.27111051	43.06321303	34.84191341	35.826495	32.77402064	42.0745216	42.26373492	40.29216822	48.83394277	49.03086837	49.58665943	Rnf19b	ring finger protein 19B [Source:MGI Symbol;Acc:MGI:1922484]
ENSMUSG00000028794	0.171988868	0.522142425	0.144066225	0.452310252	0.837799504	0.607367388	0.013958886	0	0.014552744	0.055941812	0.065074265	0.055425734	A3galt2	alpha 1,3-galactosyltransferase 2 (isoglobotriaosylceramide synthase) [Source:MGI Symbol;Acc:MGI:2685279]
ENSMUSG00000028795	7.618158501	8.772701127	6.643945931	7.043314965	6.29951977	6.124980906	16.37354436	19.56596288	12.73694327	5.930932523	7.163375096	8.501762357	Ccdc28b	coiled coil domain containing 28B [Source:MGI Symbol;Acc:MGI:1913514]
ENSMUSG00000028796	91.10445216	83.24433722	97.62101438	91.61615239	78.7601365	70.61069487	21.75057069	22.52585448	20.55949474	33.25789429	32.75933733	29.74012253	Phc2	polyhomeotic 2 [Source:MGI Symbol;Acc:MGI:1860454]
ENSMUSG00000028797	116.6355964	108.8454461	128.6348067	111.4418378	104.0709257	85.09599775	100.814617	107.8087747	88.73834842	76.92709015	81.67883913	81.15340249	Tmem234	transmembrane protein 234 [Source:MGI Symbol;Acc:MGI:1924049]
ENSMUSG00000028798	147.0006703	124.0119383	156.8849654	125.8714505	118.3147068	86.59529802	316.2085153	343.145997	280.2323995	204.4226095	199.2230481	206.6122996	Eif3i	eukaryotic translation initiation factor 3, subunit I [Source:MGI Symbol;Acc:MGI:1860763]
ENSMUSG00000028799	18.32290373	18.36098802	15.6033915	16.14745048	14.81445739	14.0301645	5.113313909	5.385329884	5.28567482	5.144753542	5.878573471	6.15116229	Zfp362	zinc finger protein 362 [Source:MGI Symbol;Acc:MGI:2652839]
ENSMUSG00000028800	124.3922915	130.0273466	118.170399	107.0181245	111.214416	109.8248229	402.567758	392.3787536	402.4377483	331.304082	334.8452387	345.1316136	Hdac1	histone deacetylase 1 [Source:MGI Symbol;Acc:MGI:108086]
ENSMUSG00000028801	0.058107343	0	0	0.027112409	0.030525536	0.044259312	0.778153695	0.978581244	0.486755387	0.85759817	0.386948013	0.540709655	Stpg1	sperm tail PG rich repeat containing 1 [Source:MGI Symbol;Acc:MGI:1926056]
ENSMUSG00000028803	2.148925641	1.956659154	1.688413771	2.168567618	1.680216245	1.789058037	8.477145498	7.69839642	8.395902265	10.30368902	10.37899005	11.05012743	Nipal3	NIPA-like domain containing 3 [Source:MGI Symbol;Acc:MGI:1921802]
ENSMUSG00000028804	0.109464035	0.073531187	0.037356159	0.017025007	0.028752375	0.048636452	0.048863499	0.027109931	0.093394248	0.089753579	0.121490356	0.064673148	Csmd2	CUB and Sushi multiple domains 2 [Source:MGI Symbol;Acc:MGI:2386401]
ENSMUSG00000028807	1.334791522	1.707867837	1.63226778	1.314805823	0.973897422	1.101410431	7.365191754	7.107368832	8.07539979	7.561616831	7.962734919	7.60686552	Zbtb8a	zinc finger and BTB domain containing 8a [Source:MGI Symbol;Acc:MGI:1920930]
ENSMUSG00000028809	73.18842298	85.98696697	66.54804117	79.82397234	85.37426549	93.62725961	199.6354226	179.4527213	234.5141677	189.5148306	187.3287211	180.9488681	Srrm1	serine/arginine repetitive matrix 1 [Source:MGI Symbol;Acc:MGI:1858303]
ENSMUSG00000028811	79.92559754	64.13027528	80.71293396	76.65048383	71.81490031	62.81226721	178.6323105	190.8459117	170.2595464	101.9314523	109.2022106	105.8139876	Yars	tyrosyl-tRNA synthetase [Source:MGI Symbol;Acc:MGI:2147627]
ENSMUSG00000028813	0	0	0.018996227	0	0	0	0.016565267	0	0	0	0	0	CK137956	cDNA sequence CK137956 [Source:MGI Symbol;Acc:MGI:3616080]
ENSMUSG00000028820	165.8316517	173.9487074	141.7505114	177.8412599	187.4338354	195.9490793	289.6154129	282.8047677	312.5205813	370.1248968	358.0518971	364.3154263	Sfpq	splicing factor proline/glutamine rich (polypyrimidine tract binding protein associated) [Source:MGI Symbol;Acc:MGI:1918764]
ENSMUSG00000028821	65.81076184	54.46552847	69.19405507	60.03299928	50.8482559	42.23920931	68.86749379	75.11863534	58.77547385	60.75654416	54.00678179	56.93714649	Syf2	SYF2 homolog, RNA splicing factor (S. cerevisiae) [Source:MGI Symbol;Acc:MGI:1915842]
ENSMUSG00000028822	217.1552062	184.7547947	232.9457347	168.6696507	149.9111596	127.3485334	100.9488057	104.4820674	92.83743024	100.4207854	98.47779173	101.0006665	Tmem50a	transmembrane protein 50A [Source:MGI Symbol;Acc:MGI:1919067]
ENSMUSG00000028825	6.368837549	5.232214195	7.037227694	6.885524178	4.937009495	5.383458263	9.014315937	8.617499896	6.180870858	6.18308485	6.368162878	7.192939603	Rhd	Rh blood group, D antigen [Source:MGI Symbol;Acc:MGI:1202882]
ENSMUSG00000028826	31.40511276	29.38769738	27.51623297	25.69685714	29.43259925	26.44822969	35.28863459	37.21361821	39.23008068	41.5545406	40.60873696	40.82999126	Maco1	macoilin 1 [Source:MGI Symbol;Acc:MGI:1913396]
ENSMUSG00000028830	27.54163591	30.40909627	26.48362042	30.25407064	29.74153046	31.06726544	10.68626791	11.26761037	11.08809732	12.33035706	14.33147226	13.87565154	AU040320	expressed sequence AU040320 [Source:MGI Symbol;Acc:MGI:2140475]
ENSMUSG00000028832	90.83791875	76.6340529	88.77963067	95.22989571	86.7791858	70.61273667	258.3067774	297.1832728	248.1206054	225.0292593	228.2125054	229.7227477	Stmn1	stathmin 1 [Source:MGI Symbol;Acc:MGI:96739]
ENSMUSG00000028833	12.43720956	12.36111591	13.16897656	13.80541835	13.14715406	11.68515435	13.89993306	13.8421039	12.59496285	15.00078689	16.93391498	15.76519381	Ncdn	neurochondrin [Source:MGI Symbol;Acc:MGI:1347351]
ENSMUSG00000028834	0.571897623	0.564488153	0.641266933	0.203308749	0.400580036	0.352631868	5.130084942	6.07013654	4.41047618	4.043672416	4.715132974	4.995893209	Trim63	tripartite motif-containing 63 [Source:MGI Symbol;Acc:MGI:2447992]
ENSMUSG00000028836	0.86926238	0.750750237	0.889944488	0.659084863	0.45664968	0.896595682	1.552115316	1.614615443	1.887838792	6.641442009	7.672891254	5.890057248	Slc30a2	solute carrier family 30 (zinc transporter), member 2 [Source:MGI Symbol;Acc:MGI:106637]
ENSMUSG00000028837	189.813613	148.1565659	207.8266442	147.7420163	138.7029682	113.8955327	286.5584646	305.3340864	254.6671578	226.5057766	214.952057	211.1643403	Psmb2	proteasome (prosome, macropain) subunit, beta type 2 [Source:MGI Symbol;Acc:MGI:1347045]
ENSMUSG00000028838	1.193853998	1.288860233	0.967621681	0.835563701	0.783959278	0.966170551	4.68527989	4.903204446	6.412494203	4.360486363	4.306306416	4.011669742	Extl1	exostosin-like glycosyltransferase 1 [Source:MGI Symbol;Acc:MGI:1888742]
ENSMUSG00000028840	0.107196033	0.098753394	0.043898605	0.060020146	0.022525321	0.065319423	0.057421307	0.106192944	0.079818937	0.057530592	0.035691954	0.018999953	Zfp593	zinc finger protein 593 [Source:MGI Symbol;Acc:MGI:1915290]
ENSMUSG00000028841	0.839265329	0.520400014	0.528759192	0.692820698	0.474806044	0.221279959	2.07492085	2.014574948	2.22328373	2.945065694	1.827117429	2.631828381	Cnksr1	connector enhancer of kinase suppressor of Ras 1 [Source:MGI Symbol;Acc:MGI:2670958]
ENSMUSG00000028842	5.749137942	7.114965862	5.772461632	6.11480844	8.049001947	9.633831565	4.947180177	4.99543096	6.086026889	10.01232351	10.41295511	9.772631079	Ago3	argonaute RISC catalytic subunit 3 [Source:MGI Symbol;Acc:MGI:2446634]
ENSMUSG00000028843	163.6350412	112.9692456	174.3430675	108.5548904	92.33153784	72.18734564	121.6824107	127.4065393	107.3877801	76.90505251	77.63621075	72.05222875	Sh3bgrl3	SH3 domain binding glutamic acid-rich protein-like 3 [Source:MGI Symbol;Acc:MGI:1920973]
ENSMUSG00000028845	2.591539246	2.675570937	2.790433294	1.292583399	1.032796473	0.782764771	0.638253027	1.150853624	0.665406464	1.558702688	1.264562582	1.663117338	Tekt2	tektin 2 [Source:MGI Symbol;Acc:MGI:1346335]
ENSMUSG00000028847	80.49688176	69.57000458	77.44190175	63.19235856	61.94891068	53.56987944	62.12315359	59.35556806	53.83656434	53.4451992	52.32648636	48.50270134	Trappc3	trafficking protein particle complex 3 [Source:MGI Symbol;Acc:MGI:1351486]
ENSMUSG00000028848	25.91774413	20.09025195	25.20338291	20.97686561	18.46226646	14.66206186	40.66133677	49.39779037	37.19965049	31.28553992	28.27648064	29.99342503	Gpn2	GPN-loop GTPase 2 [Source:MGI Symbol;Acc:MGI:2140368]
ENSMUSG00000028849	99.88123124	100.2555646	99.07220022	85.19520515	83.81642912	77.72767732	42.05415604	43.56225076	43.75163601	46.87523021	50.27476297	49.12774677	Map7d1	MAP7 domain containing 1 [Source:MGI Symbol;Acc:MGI:2384297]
ENSMUSG00000028850	9.155738106	8.498784233	11.19126745	9.323663543	7.278685559	7.053308648	11.18879245	14.24310283	11.95642145	10.43160901	10.77983631	12.2173384	Gpatch3	G patch domain containing 3 [Source:MGI Symbol;Acc:MGI:2442492]
ENSMUSG00000028851	312.5541546	250.7756041	342.75098	264.8456476	238.7250366	192.7294491	909.1515464	992.4718206	768.1093519	514.7230947	509.893019	508.120955	Nudc	nudC nuclear distribution protein [Source:MGI Symbol;Acc:MGI:106014]
ENSMUSG00000028854	35.95992268	39.76235576	35.23605671	32.56253352	34.68721688	35.61557523	31.15259352	32.56681454	38.45658422	33.94668742	36.72747401	37.11370711	Slc9a1	solute carrier family 9 (sodium/hydrogen exchanger), member 1 [Source:MGI Symbol;Acc:MGI:102462]
ENSMUSG00000028857	58.11963863	49.40589384	59.56630353	53.71255079	51.43265203	44.6486658	58.79142884	60.35572928	53.92124893	50.64793256	56.63219007	54.43610307	Tmem222	transmembrane protein 222 [Source:MGI Symbol;Acc:MGI:1098568]
ENSMUSG00000028859	0.354229682	0.287939074	0.213328088	0.136113484	0.021892648	0.095227179	0	0.082568228	0	1.081017942	1.005994566	0.757118204	Csf3r	colony stimulating factor 3 receptor (granulocyte) [Source:MGI Symbol;Acc:MGI:1339755]
ENSMUSG00000028860	4.215895929	3.428717248	4.127369431	4.27844773	2.851473846	2.729495901	2.752319612	2.636381272	2.256290227	6.999954692	8.181074436	7.005432582	Sytl1	synaptotagmin-like 1 [Source:MGI Symbol;Acc:MGI:1933365]
ENSMUSG00000028861	117.0801227	95.15832662	145.0220753	110.5136789	97.3717626	76.14511705	104.6266364	116.9848884	96.3372268	72.61028336	67.5710806	64.73147576	Mrps15	mitochondrial ribosomal protein S15 [Source:MGI Symbol;Acc:MGI:1913657]
ENSMUSG00000028862	4.485491553	6.155027222	5.93962924	6.165889855	7.054981016	7.283115697	4.268317119	3.485634238	4.09991314	5.645862732	4.829241638	5.331941729	Map3k6	mitogen-activated protein kinase kinase kinase 6 [Source:MGI Symbol;Acc:MGI:1855691]
ENSMUSG00000028863	34.40037959	33.5491512	33.11566725	40.81573138	39.75349521	36.24488046	37.71458329	41.92281723	41.99598528	25.65864425	27.14247595	26.15016901	Meaf6	MYST/Esa1-associated factor 6 [Source:MGI Symbol;Acc:MGI:1917338]
ENSMUSG00000028864	3.746501369	6.517724028	2.97961782	4.651561334	8.109115399	13.59460497	0.08613196	0.09557365	0.134694456	1.366351571	1.030605161	1.282496794	Hgf	hepatocyte growth factor [Source:MGI Symbol;Acc:MGI:96079]
ENSMUSG00000028865	2.21724897	1.516007724	2.340940832	1.163868923	1.455984488	1.108300215	8.598493906	11.80619292	7.867949252	3.780621495	2.999158655	4.175583766	Cd164l2	CD164 sialomucin-like 2 [Source:MGI Symbol;Acc:MGI:1916905]
ENSMUSG00000028868	47.08522772	54.53249645	39.73285923	54.08479133	64.36005095	76.54824682	92.74132903	81.68837815	104.0623459	109.0532699	111.3452333	105.6983719	Wasf2	WAS protein family, member 2 [Source:MGI Symbol;Acc:MGI:1098641]
ENSMUSG00000028869	115.12778	111.6593811	109.5864785	131.5446911	125.4761489	121.6264676	126.4265444	127.8317581	133.52585	119.060519	124.147215	123.94218	Gnl2	guanine nucleotide binding protein-like 2 (nucleolar) [Source:MGI Symbol;Acc:MGI:2385207]
ENSMUSG00000028871	0.15097715	0.17385795	0.038642325	0.387445839	0.079312839	0.258742268	2.392504069	3.066069747	2.142980915	2.363296181	1.193895849	1.471796321	Rspo1	R-spondin 1 [Source:MGI Symbol;Acc:MGI:2183426]
ENSMUSG00000028873	49.34714113	46.42605046	53.45595404	57.64152549	55.73317756	51.36787762	115.27208	116.9157384	112.258116	78.40958192	81.28070309	79.87149951	Cdca8	cell division cycle associated 8 [Source:MGI Symbol;Acc:MGI:1196274]
ENSMUSG00000028874	0.221588655	0.18557871	0.185613414	0	0	0.030687285	0.791317219	0.898016454	0.824982522	0.900935542	1.106701209	1.196116392	Fgr	FGR proto-oncogene, Src family tyrosine kinase [Source:MGI Symbol;Acc:MGI:95527]
ENSMUSG00000028876	0.048970206	0.018797237	0.217253131	0.076163653	0	0.04973296	0.349755879	0.355754419	0.425408286	0.423426034	0.584266178	0.76670863	Epha10	Eph receptor A10 [Source:MGI Symbol;Acc:MGI:3586824]
ENSMUSG00000028878	26.52207922	26.45440241	23.59408655	28.42503261	28.58085211	31.73710247	22.46292314	24.29914143	26.20164828	19.46142932	20.01293212	20.50990128	Fam76a	family with sequence similarity 76, member A [Source:MGI Symbol;Acc:MGI:2385211]
ENSMUSG00000028879	41.92764776	42.99068096	34.79098736	42.9003509	48.65300111	51.65827279	38.37212342	35.89295518	42.58841919	41.12090289	39.64159188	39.53266649	Stx12	syntaxin 12 [Source:MGI Symbol;Acc:MGI:1931027]
ENSMUSG00000028882	44.10731202	47.35156218	44.56201587	49.60135497	53.36063805	48.54474031	114.9111643	110.901193	104.0814497	104.8490887	103.2368764	98.66015745	Ppp1r8	protein phosphatase 1, regulatory subunit 8 [Source:MGI Symbol;Acc:MGI:2140494]
ENSMUSG00000028883	24.20883709	30.48146813	21.7563464	30.01595419	35.34858238	41.57936794	0.303554754	0.235781062	0.411409696	4.242648209	4.401046335	3.585788746	Sema3a	sema domain, immunoglobulin domain (Ig), short basic domain, secreted, (semaphorin) 3A [Source:MGI Symbol;Acc:MGI:107558]
ENSMUSG00000028884	55.61689789	45.98794893	53.44043467	57.41997965	43.89710609	44.38751345	234.3160771	241.994409	231.0527972	135.2660306	137.3507167	135.0279904	Rpa2	replication protein A2 [Source:MGI Symbol;Acc:MGI:1339939]
ENSMUSG00000028885	3.710831702	4.248512805	3.645400565	4.710138851	3.806753145	5.457743475	5.971018546	5.310605337	9.689144077	11.39140905	10.28409842	Smpdl3b	sphingomyelin phosphodiesterase, acid-like 3B [Source:MGI Symbol;Acc:MGI:1916022]	
ENSMUSG00000028886	16.78805815	16.80180721	14.09407329	15.96790576	14.46394564	18.41154145	21.8833397	20.85673284	25.46121424	27.73503848	31.6339809	27.93740032	Eya3	EYA transcriptional coactivator and phosphatase 3 [Source:MGI Symbol;Acc:MGI:109339]
ENSMUSG00000028889	34.71054608	32.41169988	33.18879038	40.32130846	43.93284855	33.69867953	57.441647	55.18644232	50.38576639	31.28906775	31.62514084	31.75749743	Yrdc	yrdC domain containing (E.coli) [Source:MGI Symbol;Acc:MGI:2387201]
ENSMUSG00000028890	7.00607943	8.796109978	6.664965808	9.581290965	10.7483754	13.00571366	7.273359978	7.370463579	8.690781296	8.285449821	8.918092338	8.121099875	Mtf1	metal response element binding transcription factor 1 [Source:MGI Symbol;Acc:MGI:101786]
ENSMUSG00000028893	15.86648918	13.87386437	13.53893618	16.90490304	16.01480285	16.26911816	23.32527783	23.01182454	24.91691096	30.73499473	31.931131	30.47340326	Sesn2	sestrin 2 [Source:MGI Symbol;Acc:MGI:2651874]
ENSMUSG00000028894	32.55464951	38.8637482	33.29785744	41.03942646	45.07961139	48.72948691	26.49394562	24.96873499	34.9468041	32.46138089	34.66971577	33.13898692	Inpp5b	inositol polyphosphate-5-phosphatase B [Source:MGI Symbol;Acc:MGI:103257]
ENSMUSG00000028896	31.91982594	29.51110857	30.73580202	35.85600594	33.94317366	36.12104072	147.4452487	146.9616645	143.6076193	105.7475743	109.5282393	106.0493526	Rcc1	regulator of chromosome condensation 1 [Source:MGI Symbol;Acc:MGI:1913989]
ENSMUSG00000028898	34.8894296	29.42978797	43.27453077	33.26713107	29.8158266	25.78939329	69.46645743	73.74862931	62.47263611	38.58503564	41.91432591	40.63342303	Trnau1ap	tRNA selenocysteine 1 associated protein 1 [Source:MGI Symbol;Acc:MGI:1919037]
ENSMUSG00000028899	19.25806852	16.39278607	18.97381563	18.64805712	14.50504097	15.03676977	32.42002666	32.35520025	30.47435437	20.56535531	18.51209294	19.35209208	Taf12	TATA-box binding protein associated factor 12 [Source:MGI Symbol;Acc:MGI:1913714]

ENSMUSG00000028901	9.690478391	8.831788413	9.231342675	9.623294367	10.27214299	11.10448551	14.3265353	13.21041928	13.03888474	16.56326774	15.42814427	15.69097844	Gmeb1	glucocorticoid modulatory element binding protein 1 [Source:MGI Symbol;Acc:MGI:2135604]
ENSMUSG00000028902	138.7131802	122.765108	143.8771614	139.7769517	138.8541206	119.080822	150.254313	162.3964094	141.1715358	128.944624	120.6738963	123.8170821	Sf3a3	splicing factor 3a, subunit 3 [Source:MGI Symbol;Acc:MGI:1922312]
ENSMUSG00000028906	6.89474231	7.564363924	6.109968903	7.55550648	7.960944107	9.013825321	11.62901736	10.61358385	14.20915459	14.94881723	14.72518644	15.37049389	Epb41	erythrocyte membrane protein band 4.1 [Source:MGI Symbol;Acc:MGI:95401]
ENSMUSG00000028907	45.05263622	41.65620362	46.70543276	56.09466611	49.55837036	45.32999789	38.6475884	40.31682103	36.25261756	24.46989737	22.80996311	25.29056865	Utp11	UTP11 small subunit processome component [Source:MGI Symbol;Acc:MGI:1914455]
ENSMUSG00000028909	1.626982243	2.331976399	0.726525446	0.662225499	1.018369199	1.186509194	3.214114902	3.480710814	4.043578854	3.173786447	3.400153907	3.850104214	Ptpru	protein tyrosine phosphatase, receptor type, U [Source:MGI Symbol;Acc:MGI:1321151]
ENSMUSG00000028910	30.25987152	29.74313743	39.69477829	31.06725724	29.34252641	22.24903027	45.44196152	47.88188041	48.56909297	35.72392543	34.50650199	33.89591528	Mecr	mitochondrial trans-2-enoyl-CoA reductase [Source:MGI Symbol;Acc:MGI:1349441]
ENSMUSG00000028911	4.645256894	6.81064572	2.598518932	5.921353159	6.717095715	7.587085467	11.8215995	8.871489727	15.37780442	14.13581403	13.05512156	12.43510414	Srsf4	serine and arginine-rich splicing factor 4 [Source:MGI Symbol;Acc:MGI:1890577]
ENSMUSG00000028914	10.49609345	11.89694628	8.607910958	12.97423996	13.30845131	14.41973194	14.3013154	12.43934381	16.41861767	16.27013649	15.94148289	16.5339989	Casp9	caspase 9 [Source:MGI Symbol;Acc:MGI:1277950]
ENSMUSG00000028917	29.50730005	28.57777959	34.30097346	30.41659444	28.99065131	28.49043062	7.456498371	7.53894303	7.506431626	10.25346824	10.13948255	8.928783578	Plekhm2	pleckstrin homology domain containing, family M (with RUN domain) member 2 [Source:MGI Symbol;Acc:MGI:1916832]
ENSMUSG00000028919	26.38102081	21.4388389	24.99194087	20.58136072	20.45331459	17.50497512	9.414053107	11.24955047	10.89308161	7.87724985	7.394866571	7.736118905	Arhgef19	Rho guanine nucleotide exchange factor (GEF) 19 [Source:MGI Symbol;Acc:MGI:1925912]
ENSMUSG00000028920	14.61145457	15.09153193	13.06554304	13.99269185	16.01793042	17.95175094	21.51326184	19.48722359	25.05992349	25.22929328	28.63590536	27.1976474	Fbxo42	F-box protein 42 [Source:MGI Symbol;Acc:MGI:1924992]
ENSMUSG00000028923	80.80849554	78.41432283	86.90012762	75.91582876	64.2233839	58.4487266	82.08095002	85.117229	83.29886615	63.37766071	65.57011589	64.39689267	Necap2	NECAP endocytosis associated 2 [Source:MGI Symbol;Acc:MGI:1913397]
ENSMUSG00000028926	19.30740552	27.96536151	14.43193917	25.81943017	31.29649512	39.98637854	0.451326799	0.616370143	0.741986088	7.774117021	6.668121418	7.202690538	Cdk14	cyclin-dependent kinase 14 [Source:MGI Symbol;Acc:MGI:894318]
ENSMUSG00000028927	0.779098331	1.538008762	1.230637098	1.495628766	1.286320491	1.237715213	6.081192626	5.689326801	7.230809044	5.555169487	5.002874485	5.030465957	Padi2	peptidyl arginine deiminase, type II [Source:MGI Symbol;Acc:MGI:1338892]
ENSMUSG00000028931	41.69951938	44.46751402	46.38835437	54.47394895	52.00537321	52.44607265	1.231245323	1.167491167	0.863530656	4.283917003	4.049227181	4.355506596	Kcnab2	potassium voltage-gated channel, shaker-related subfamily, beta member 2 [Source:MGI Symbol;Acc:MGI:109239]
ENSMUSG00000028932	111.4845705	90.14965246	119.7611772	105.1842812	98.48541427	81.10940376	88.44273832	92.60474513	84.51020787	75.52975809	77.20050701	75.06092682	Psmc2	proteasome (prosome, macropain) 26S subunit, ATPase 2 [Source:MGI Symbol;Acc:MGI:109555]
ENSMUSG00000028933	6.080997649	7.002581388	4.697053807	8.182696114	8.613824695	9.346282226	12.36061551	9.816046947	11.31988636	13.25831523	13.73921785	13.83370359	Xrcc2	X-ray repair complementing defective repair in Chinese hamster cells 2 [Source:MGI Symbol;Acc:MGI:1927345]
ENSMUSG00000028936	340.5830252	281.5437274	429.0369318	318.7982943	298.9518169	225.9208067	723.7544454	757.2301305	522.8418934	364.1749161	348.948569	348.985296	Rpl22	ribosomal protein L22 [Source:MGI Symbol;Acc:MGI:99262]
ENSMUSG00000028937	111.284637	91.49691378	117.673819	95.70828963	80.01253056	72.85111071	119.9455581	129.8239288	109.284808	72.19569217	72.22252151	73.93256315	Acot7	acyl-CoA thioesterase 7 [Source:MGI Symbol;Acc:MGI:1917275]
ENSMUSG00000028938	0	0	0	0	0	0	0	0	0	0.024943595	0.046424984	0.049426967	Galntl5	UDP-N-acetyl-alpha-D-galactosamine:polypeptide N-acetylgalactosaminyltransferase-like 5 [Source:MGI Symbol;Acc:MGI:191515
ENSMUSG00000028940	0.054363219	0.031301021	0	0.101461725	0	0	0.728013659	0.511617881	0.581889125	0.364699618	0.362015166	0.289068134	Hes2	hes family bHLH transcription factor 2 [Source:MGI Symbol;Acc:MGI:1098624]
ENSMUSG00000028943	3.494929919	2.195231705	3.554849304	2.308401416	2.265184727	2.0051616	12.88599535	13.42174635	13.1173645	5.765075132	5.799462433	6.355484115	Espn	espin [Source:MGI Symbol;Acc:MGI:1861630]
ENSMUSG00000028944	19.8759466	17.47759495	17.43734904	16.93253699	20.42818408	20.08350546	9.852085419	8.359810648	10.41508107	8.07362513	7.719154198	7.971749472	Prkag2	protein kinase, AMP-activated, gamma 2 non-catalytic subunit [Source:MGI Symbol;Acc:MGI:1336153]
ENSMUSG00000028945	80.90892443	79.10226244	77.60275861	89.98681193	91.70830294	84.56867825	53.71219775	54.08154427	55.19273694	45.77318858	44.78708403	44.1422419	Rheb	Ras homolog enriched in brain [Source:MGI Symbol;Acc:MGI:97912]
ENSMUSG00000028946	0.061645398	0	0.189336319	0.172579418	0	0	0.137589019	0.091602825	0.14344252	0.110280706	0.076970363	0.027315834	Hes3	hes family bHLH transcription factor 3 [Source:MGI Symbol;Acc:MGI:104877]
ENSMUSG00000028948	13.39105175	13.64457918	14.22553147	15.01836964	14.39029864	16.18929621	56.94068327	54.98401575	63.29982998	64.11018955	68.95193381	65.26437165	Nol9	nucleolar protein 9 [Source:MGI Symbol;Acc:MGI:1921285]
ENSMUSG00000028949	10.06534563	11.9890709	9.207061938	12.81425297	12.50133562	9.958665435	6.083381232	5.448158991	6.149025676	4.950220755	4.693047189	3.801029085	Smarcd3	SWI/SNF related, matrix associated, actin dependent regulator of chromatin, subfamily d, member 3 [Source:MGI Symbol;Acc:MGI:19
ENSMUSG00000028950	0.375729024	0.190375366	0.461602342	0.322574219	0.694783796	0.377765519	0.630631555	0.744426261	0.825323067	0.779707317	0.888228487	0.839110372	Tas1r1	taste receptor, type 1, member 1 [Source:MGI Symbol;Acc:MGI:1927505]
ENSMUSG00000028952	13.60056502	12.66353066	15.99349211	13.74681168	10.54620802	11.58572067	19.20742704	19.68391056	19.35496415	12.1040977	13.45984493	12.8425308	Zbtb48	zinc finger and BTB domain containing 48 [Source:MGI Symbol;Acc:MGI:2140248]
ENSMUSG00000028953	63.29492234	62.70357154	59.91380389	61.9885921	59.37441265	59.22032067	91.28948389	93.14967263	97.35576149	91.70293584	96.28312264	89.16479504	Abcf2	ATP-binding cassette, sub-family F (GCN20), member 2 [Source:MGI Symbol;Acc:MGI:1351657]
ENSMUSG00000028954	28.88312937	24.15521484	31.24132607	27.44968013	27.01828972	23.68496237	27.02747811	26.50491738	24.52011834	18.90333386	16.91715866	18.36465261	Nub1	negative regulator of ubiquitin-like proteins 1 [Source:MGI Symbol;Acc:MGI:1889001]
ENSMUSG00000028955	86.93902131	87.2195128	91.32400531	83.3130984	87.35439766	87.54344015	67.14040003	64.09073536	69.67552921	69.05193967	66.39848574	66.27376483	Vamp3	vesicle-associated membrane protein 3 [Source:MGI Symbol;Acc:MGI:1321389]
ENSMUSG00000028957	3.32488196	3.202460943	2.510967755	3.217640962	3.655048872	4.41624652	2.684367337	2.826135383	3.104215179	3.295296906	2.86159153	2.892025684	Per3	period circadian clock 3 [Source:MGI Symbol;Acc:MGI:1277134]
ENSMUSG00000028958	17.40011657	12.82202378	17.38904613	15.6035006	11.26242317	8.136006877	7.750365295	8.749516107	6.815381926	5.030444728	4.367145343	4.147789633	Tmub1	transmembrane and ubiquitin-like domain containing 1 [Source:MGI Symbol;Acc:MGI:1923764]
ENSMUSG00000028959	101.1529672	90.65223406	104.2301719	104.4784937	94.166794	85.57660944	57.27146052	58.26181314	52.11875883	40.36257052	43.42182849	46.37471971	Fastk	Fas-activated serine/threonine kinase [Source:MGI Symbol;Acc:MGI:1913837]
ENSMUSG00000028960	33.61216794	37.36250346	36.69623696	30.14376214	33.68041224	36.15744713	58.11571644	53.92108898	60.35952047	62.36442125	61.8020016	64.54656274	Ube4b	ubiquitination factor E4B [Source:MGI Symbol;Acc:MGI:1927086]
ENSMUSG00000028961	151.6459113	141.203763	156.8743473	112.6750647	106.8765232	97.64778343	160.6047453	164.736677	151.8322259	134.5399174	137.9753576	131.8485595	Pgd	phosphogluconate dehydrogenase [Source:MGI Symbol;Acc:MGI:97553]
ENSMUSG00000028962	80.50393386	106.5419416	87.91810159	104.008268	116.2408524	123.6075193	14.49361561	11.78373223	19.29024717	28.63526412	31.21978709	29.28542539	Slc4a2	solute carrier family 4 (anion exchanger), member 2 [Source:MGI Symbol;Acc:MGI:109351]
ENSMUSG00000028963	0	0	0	0	0	0	0	0.1584604	0	0	0.133148236	0	Uts2	urotensin 2 [Source:MGI Symbol;Acc:MGI:1346329]
ENSMUSG00000028964	235.5084509	166.255327	276.4402442	192.2516937	165.7215041	120.601031	515.9568111	569.2825643	417.7036506	277.3620161	271.4939283	266.5554307	Park7	Parkinson disease (autosomal recessive, early onset) 7 [Source:MGI Symbol;Acc:MGI:2135637]
ENSMUSG00000028965	122.9938691	132.8348717	126.7133056	148.6533009	147.2784905	129.9964632	0.880209422	0.662758679	0.786562714	1.291331114	0.498270772	0.717722429	Tnfrsf9	tumor necrosis factor receptor superfamily, member 9 [Source:MGI Symbol;Acc:MGI:1101059]
ENSMUSG00000028967	50.06348969	58.52587715	43.77293339	74.86950555	85.17509254	92.68160546	12.96153364	10.25105305	14.69194417	55.58791882	56.87226695	58.9436611	Errfi1	ERBB receptor feedback inhibitor 1 [Source:MGI Symbol;Acc:MGI:1921405]
ENSMUSG00000028969	107.6119662	88.35391848	111.6741229	103.2355041	86.73489865	75.97613875	26.55572298	23.81557779	21.95876774	20.4102852	24.45451441	25.96359704	Cdk5	cyclin-dependent kinase 5 [Source:MGI Symbol;Acc:MGI:101765]
ENSMUSG00000028970	10.96059841	14.68365217	8.527137964	18.40578713	21.40696537	25.41743088	13.34589193	12.25560312	14.92373805	37.7114122	37.03636556	34.50234441	Abcb1b	ATP-binding cassette, sub-family B (MDR/TAP), member 1B [Source:MGI Symbol;Acc:MGI:97568]
ENSMUSG00000028971	0.762691069	0.439138986	1.626744837	0.948974365	1.602658258	0.871391981	8.341186925	8.248133011	7.986167586	1.421269068	1.798779566	2.478357173	Cort	cortistatin [Source:MGI Symbol;Acc:MGI:109538]
ENSMUSG00000028972	2.025319587	0.968107765	2.699471815	2.531880041	2.69001539	2.008354728	0.272929691	0.378559926	0.391243931	1.26470227	0.667987909	0.745047871	Car6	carbonic anhydrase 6 [Source:MGI Symbol;Acc:MGI:1333786]
ENSMUSG00000028973	30.85797056	28.0940943	33.75913543	36.38502846	36.51778725	33.0497891	22.82513659	20.47144271	19.54502281	13.05352387	12.19396537	13.42673149	Abcb8	ATP-binding cassette, sub-family B (MDR/TAP), member 8 [Source:MGI Symbol;Acc:MGI:1351667]
ENSMUSG00000028974	12.98760131	13.75291674	11.59117607	12.96293379	13.58622691	12.6881051	24.22353987	24.27771378	24.64967462	24.62320683	23.80741133	24.62124555	Dffa	DNA fragmentation factor, alpha subunit [Source:MGI Symbol;Acc:MGI:1196227]
ENSMUSG00000028975	11.81393745	9.800294567	15.64901015	9.561595893	7.235684004	6.311717702	11.54695028	12.47991637	10.57902116	8.26351913	7.6434191	6.202489377	Pex14	peroxisomal biogenesis factor 14 [Source:MGI Symbol;Acc:MGI:1927868]
ENSMUSG00000028976	5.864525906	6.298024191	6.037789269	6.333550103	5.192379596	5.847126193	2.529615199	2.349970164	3.219878185	4.037405354	3.894331025	4.321339888	Slc2a5	solute carrier family 2 (facilitated glucose transporter), member 5 [Source:MGI Symbol;Acc:MGI:1928369]
ENSMUSG00000028977	1.39882656	1.443026869	1.685710895	1.278166921	0.949175665	1.520501465	5.918983611	5.730599911	7.744011351	5.669578521	5.82191	6.133806206	Casz1	castor zinc finger 1 [Source:MGI Symbol;Acc:MGI:1196251]
ENSMUSG00000028978	0.186841454	0.101250607	0.157530397	0.143588426	0.115474632	0.058599781	2.845544746	2.417097877	2.782469787	0.747148262	0.640403904	0.506490688	Nos3	nitric oxide synthase 3, endothelial cell [Source:MGI Symbol;Acc:MGI:97362]
ENSMUSG00000028979	0.656012981	0.412054184	0.518980079	0.584354147	0.469940939	0.363398704	0.745402574	0.88619226	0.582835885	0.720149059	0.918381423	1.136323563	Masp2	mannan-binding lectin serine peptidase 2 [Source:MGI Symbol;Acc:MGI:1330832]
ENSMUSG00000028980	40.42019725	44.6902195	41.02472103	30.68097785	34.70043895	36.26654175	9.181001803	8.903700234	10.80863201	14.72647671	14.06400091	15.0617612	H6pd	hexose-6-phosphate dehydrogenase (glucose 1-dehydrogenase) [Source:MGI Symbol;Acc:MGI:2140356]
ENSMUSG00000028982	3.283572998	4.673987165	4.458918806	4.660101561	3.425961592	3.473664084	10.44346589	9.510059291	7.322181916	6.037328058	6.054893099	4.344766565	Slc25a33	solute carrier family 25, member 33 [Source:MGI Symbol;Acc:MGI:1917806]
ENSMUSG00000028986	16.75242459	22.68895915	16.76502779	25.02517704	30.82776756	33.21100758	12.146411	12.0651447	14.78385822	16.31889774	16.71454189	16.57510257	Klhl7	kelch-like 7 [Source:MGI Symbol;Acc:MGI:1196453]
ENSMUSG00000028988	9.261270657	11.35384911	9.215283294	9.977677987	9.484453729	9.412128055	21.5299696	21.54486619	22.78491631	13.61273297	13.98893686	11.06638105	Ctnnbip1	catenin beta interacting protein 1 [Source:MGI Symbol;Acc:MGI:1915756]
ENSMUSG00000028989	0.159586418	0.505372899	0.020422925	0.130307995	0.188629979	0.075971304	0	0.118569813	0.148536492	0.160589568	0.249074238	0.212144115	Angptl7	angiopoietin-like 7 [Source:MGI Symbol;Acc:MGI:3605801]
ENSMUSG00000028990	38.4268301	35.09757357	37.87269843	32.84777562	36.66896933	35.59622797	52.60700849	53.10470193	51.22953966	47.30812533	47.35786869	46.50095877	Lzic	leucine zipper and CTNNBIP1 domain containing [Source:MGI Symbol;Acc:MGI:1916401]
ENSMUSG00000028991	17.6057188	20.91344512	16.60310688	21.08089979	24.08484466	30.68003674	23.81249909	20.67332981	27.51359909	34.33986028	34.81136784	35.48734983	Mtor	mechanistic target of rapamycin kinase [Source:MGI Symbol;Acc:MGI:1928394]
ENSMUSG00000028992	5.465862956	6.049446862	6.46638535	7.169251865	5.519434774	5.550998555	11.68440516	10.709105	10.59875217	7.496597543	7.127987922	6.943041221	Nmnat1	nicotinamide nucleotide adenylyltransferase 1 [Source:MGI Symbol;Acc:MGI:1913704]
ENSMUSG00000028995	29.24086485	37.40189244	25.25662345	39.35904344	51.70276064	67.05635104	8.856069584	8.158495581	10.73145006	12.11750167	12.53975769	10.52960207	Fam126a	family with sequence similarity 126, member A [Source:MGI Symbol;Acc:MGI:2149839]
ENSMUSG00000028996	0.251139034	0	0.514228602	0.078119607	0.439769634	0.191288058	3.587379369	4.478201898	2.415451753	2.99517248	2.647940211	3.264295343	Rbp7	retinol binding protein 7, cellular [Source:MGI Symbol;Acc:MGI:1890409]
ENSMUSG00000028998	39.50971371	36.95292765	65.62806341	45.86419468	46.21294765	29.42378742	91.92247748	78.73467691	48.02338258	42.25123419	35.68952659	36.43810893	Tomm7	translocase of outer mitochondrial membrane 7 [Source:MGI Symbol;Acc:MGI:1913419]
ENSMUSG00000028999	17.10689088	15.95839732	12.0480811	20.35234357	20.88557474	25.26402858	7.991263202	7.764475954	9.303921156	11.86746165	12.48106537	12.70425705	Rint1	RAD50 interactor 1 [Source:MGI Symbol;Acc:MGI:1916233]
ENSMUSG00000029001	3.849315563	3.379373519	4.409126902	0.960269178	1.281369519	0.841848183	12.18114948	13.40316735	10.7483551	3.545392828	3.616584415	4.154426286	Fbxo44	F-box protein 44 [Source:MGI Symbol;Acc:MGI:1354744]
ENSMUSG00000029003	22.89440296	18.88490973	25.82182213	23.33447391	19.84616743	15.95414589	75.09016758	73.88480268	61.10644125	33.74353781	34.28508458	31.46564211	Mad2l2	MAD2 mitotic arrest deficient-like 2 [Source:MGI Symbol;Acc:MGI:1919140]

ENSMUSG00000029004	8.690715434	16.7340662	7.108321382	13.50202932	20.79284894	34.45289473	6.058105417	4.772597657	7.942422292	14.51924381	15.66087311	15.83638742	Kmt2e	lysine (K)-specific methyltransferase 2E [Source:MGI Symbol;Acc:MGI:1924825]
ENSMUSG00000029005	1.373601727	1.55318674	1.21143688	0.34748202	0.36514411	0.49161031	0.354598653	0.36068025	0.415895535	0.48109958	0.495925968	0.542660567	Draxin	dorsal inhibitory axon guidance protein [Source:MGI Symbol;Acc:MGI:1917683]
ENSMUSG00000029007	25.05524167	23.13752319	25.67883662	22.07940614	19.8138702	17.96032677	75.53643999	78.36884343	71.18462032	50.00556229	54.12724823	48.3793398	Agtrap	angiotensin II, type I receptor-associated protein [Source:MGI Symbol;Acc:MGI:1339977]
ENSMUSG00000029009	18.52615448	18.27520715	17.57331277	17.59812917	18.03449119	20.74929316	7.469554958	6.265604928	9.085224188	17.41758663	17.17607107	16.40362219	Mthfr	methylenetetrahydrofolate reductase [Source:MGI Symbol;Acc:MGI:106639]
ENSMUSG00000029012	14.32661353	13.61281209	13.89550911	16.61523221	13.4168271	14.91412471	31.66130991	34.19224301	31.42352872	32.35251893	27.02969404	28.28172468	Orc5	origin recognition complex, subunit 5 [Source:MGI Symbol;Acc:MGI:1347044]
ENSMUSG00000029014	55.40590494	45.26546289	48.50777958	55.37206079	54.05823385	51.112502	95.88046775	100.0397818	96.78000509	85.89157911	84.00118757	89.82694025	Dnajc2	DnaJ heat shock protein family (Hsp40) member C2 [Source:MGI Symbol;Acc:MGI:99470]
ENSMUSG00000029015	0.145788681	0.119916543	0.234547315	0.077741487	0.131292309	0.079317572	0.018593831	0	0.019384875	0.204921409	0.190699724	0.239945673	Slc26a5	solute carrier family 26, member 5 [Source:MGI Symbol;Acc:MGI:1933154]
ENSMUSG00000029016	11.06935651	12.86514263	10.82476764	15.39083604	15.36903069	18.66099306	26.56200049	24.4907952	31.51382539	25.41889151	29.79280457	25.06309799	Clcn6	chloride channel, voltage-sensitive 6 [Source:MGI Symbol;Acc:MGI:1347049]
ENSMUSG00000029017	122.4018105	102.5146688	113.4102507	114.6662276	107.4528596	94.13493979	110.9017065	117.6912192	116.0615577	98.30703911	94.28265751	98.3113949	Pmpcb	peptidase (mitochondrial processing) beta [Source:MGI Symbol;Acc:MGI:1920328]
ENSMUSG00000029019	1.487540552	1.015098912	1.184503526	2.159341958	2.662717854	1.426781948	0.786988048	0.764099703	1.025586467	0.689925133	0.917205518	0.683560391	Nppb	natriuretic peptide type B [Source:MGI Symbol;Acc:MGI:97368]
ENSMUSG00000029020	20.91687369	23.59402961	17.96411695	21.7823881	21.62055507	23.1849371	11.28724888	10.35173923	12.16578038	18.48634605	19.34081589	20.19637472	Mfn2	mitofusin 2 [Source:MGI Symbol;Acc:MGI:2442230]
ENSMUSG00000029022	16.2249713	11.24166876	17.09854138	15.20436038	10.54265131	10.51877658	22.4111955	23.04828445	25.80241778	15.82114033	14.38337766	16.48909001	Miip	migration and invasion inhibitory protein [Source:MGI Symbol;Acc:MGI:106506]
ENSMUSG00000029026	0	0	0.047641222	0.028949876	0.065188635	0.023629431	1.232488108	1.367592086	1.227172992	1.526199099	1.846363804	1.622091836	Trp73	transformation related protein 73 [Source:MGI Symbol;Acc:MGI:1336991]
ENSMUSG00000029027	7.26202818	5.500532872	5.009551918	5.762096549	6.569078749	6.625794786	17.99396143	16.69640605	19.19323056	12.01880538	12.05748606	11.9423447	Dffb	DNA fragmentation factor, beta subunit [Source:MGI Symbol;Acc:MGI:1196287]
ENSMUSG00000029028	9.791820104	15.28624353	7.079364548	13.84765101	17.44696391	20.91386185	29.064611	26.72553149	27.99917221	34.19898478	29.3047163	29.34332607	Lrrc47	leucine rich repeat containing 47 [Source:MGI Symbol;Acc:MGI:1920196]
ENSMUSG00000029029	14.18664602	14.53812164	16.14982675	14.33185234	11.59608408	11.61861833	14.64086997	15.13694652	14.10079019	11.31588122	12.35064672	11.46524721	Wrap73	WD repeat containing, antisense to Trp73 [Source:MGI Symbol;Acc:MGI:1891749]
ENSMUSG00000029030	95.45776484	90.32014968	85.80612212	77.03357433	71.79277512	71.92472572	80.54592772	77.37518006	72.39319455	81.45225057	84.98721649	80.65938318	Tprgl	transformation related protein 63 regulated like [Source:MGI Symbol;Acc:MGI:1915058]
ENSMUSG00000029032	0.642525589	0.716033463	0.594153664	0.715644836	0.892842511	0.836716326	2.997690925	2.689781334	2.874433322	3.948909844	4.52024151	4.794165763	Arhgef16	Rho guanine nucleotide exchange factor (GEF) 16 [Source:MGI Symbol;Acc:MGI:2446219]
ENSMUSG00000029033	32.04343719	34.67185744	34.49052147	35.40146418	33.94712484	33.45151977	11.29615089	9.802843528	12.95646969	15.5344195	17.58451251	14.95755796	Acap3	ArfGAP with coiled-coil, ankyrin repeat and PH domains 3 [Source:MGI Symbol;Acc:MGI:2153589]
ENSMUSG00000029034	21.93132134	29.37749957	19.17945303	30.32593035	32.28122247	30.55025833	64.75912799	55.57198561	66.99660162	56.17750827	55.40332516	59.23229056	Ints11	integrator complex subunit 11 [Source:MGI Symbol;Acc:MGI:1919207]
ENSMUSG00000029036	51.73719157	45.66750787	51.09869283	50.02800013	47.42200097	49.14319961	190.235039	206.6820211	205.1701908	145.1988895	154.5309154	148.9631424	Atad3a	ATPase family, AAA domain containing 3A [Source:MGI Symbol;Acc:MGI:1919214]
ENSMUSG00000029038	46.96724941	41.240443	41.34134249	38.88709892	35.9580009	32.496702	64.47785506	71.54584591	60.4742188	50.65608497	52.4854983	49.89543914	Ssu72	Ssu72 RNA polymerase II CTD phosphatase homolog (yeast) [Source:MGI Symbol;Acc:MGI:1916241]
ENSMUSG00000029044	0	0	0	0	0	0	0	0	0	0	0	0	4930584F24	RIKEN cDNA 4930584F24 gene [Source:MGI Symbol;Acc:MGI:1923192]
ENSMUSG00000029047	4.709585732	5.382244234	4.602473901	3.795602382	3.823589114	3.723085263	18.60221789	20.04051049	19.95815926	14.07395785	13.8099893	14.13383781	Pex10	peroxisomal biogenesis factor 10 [Source:MGI Symbol;Acc:MGI:2684988]
ENSMUSG00000029048	127.2369843	111.7919064	136.4323071	107.9064822	108.1475046	87.5277544	103.5703584	103.0887645	90.4849565	78.9049239	77.30404389	79.02642539	Rer1	retention in endoplasmic reticulum sorting receptor 1 [Source:MGI Symbol;Acc:MGI:1915080]
ENSMUSG00000029049	0.822438516	1.420620344	1.301282803	1.151229165	0.62843883	0.854231641	2.970392713	4.147780141	3.305533792	1.504742386	2.022673246	1.325209546	Morn1	MORN repeat containing 1 [Source:MGI Symbol;Acc:MGI:1924116]
ENSMUSG00000029050	27.37106271	29.89238904	23.33038852	22.27784318	22.89129428	26.97756008	25.95273608	21.9585176	29.15440481	30.13148198	30.20320846	30.40497384	Ski	ski sarcoma viral oncogene homolog (avian) [Source:MGI Symbol;Acc:MGI:98310]
ENSMUSG00000029053	4.714425623	4.954951562	4.251999802	2.548872941	2.987680169	2.393928464	31.83422255	32.3585783	29.67119748	18.34035267	18.34446927	18.86749806	Prkcz	protein kinase C, zeta [Source:MGI Symbol;Acc:MGI:97602]
ENSMUSG00000029054	0	0.117624963	0	0	0	0	0.045596266	0	0.023768042	0.068524569	0.042512612	0.045261608	Gabrd	gamma-aminobutyric acid (GABA) A receptor, subunit delta [Source:MGI Symbol;Acc:MGI:95622]
ENSMUSG00000029055	0.409795122	0.707850223	0.786647325	0.394364514	0.309461703	0.643775405	0.240092838	0.304470341	0.178790855	0.652920858	1.002019731	0.749039199	Plch2	phospholipase C, eta 2 [Source:MGI Symbol;Acc:MGI:2443078]
ENSMUSG00000029056	24.50056838	22.27240484	18.37028272	23.26672283	20.98862269	23.42384227	29.90595255	27.44369403	30.04277757	22.3471181	18.1305743	20.51924168	Pank4	pantothenate kinase 4 [Source:MGI Symbol;Acc:MGI:2387466]
ENSMUSG00000029059	12.51232279	8.645149737	13.02352469	3.762361413	4.820273039	3.176803293	22.58967186	29.54198492	18.56883116	17.72063072	19.38393099	18.29640177	Prxl2b	peroxiredoxin like 2B [Source:MGI Symbol;Acc:MGI:1913719]
ENSMUSG00000029060	27.19011228	28.50964592	26.59184198	25.07070161	25.04631708	20.23672254	36.65336893	36.84244123	32.9298614	26.52091653	30.59733081	30.75543318	Mib2	mindbomb E3 ubiquitin protein ligase 2 [Source:MGI Symbol;Acc:MGI:2679684]
ENSMUSG00000029061	16.56385627	11.77949226	19.69620783	4.028484145	3.40171643	2.501833636	3.39324229	5.856986707	3.755972771	3.98730295	4.608932722	4.033687846	Mmp23	matrix metallopeptidase 23 [Source:MGI Symbol;Acc:MGI:1347361]
ENSMUSG00000029062	85.31135565	84.03740953	90.33096791	76.56702885	81.63610411	69.90213499	136.9898659	144.3123465	136.5719441	109.8782809	104.6059648	109.6630754	Cdk11b	cyclin-dependent kinase 11B [Source:MGI Symbol;Acc:MGI:88353]
ENSMUSG00000029063	127.5213488	129.5068238	114.4479344	117.6270805	119.9003226	118.4817054	126.5800929	122.0138306	141.6135714	143.9663917	152.9939663	150.7105891	Nadk	NAD kinase [Source:MGI Symbol;Acc:MGI:2183149]
ENSMUSG00000029064	291.1944556	290.5053455	274.7765218	273.6541961	276.0416908	268.3395674	254.8341651	246.3878078	251.0295041	217.1729423	223.2094959	215.3599173	Gnb1	guanine nucleotide binding protein (G protein), beta 1 [Source:MGI Symbol;Acc:MGI:95781]
ENSMUSG00000029066	82.73598498	75.90387209	119.2958588	74.58036907	66.52297851	60.10403449	147.213971	150.5493446	123.305693	122.0507658	123.2557242	127.898786	Mrpl20	mitochondrial ribosomal protein L20 [Source:MGI Symbol;Acc:MGI:2137221]
ENSMUSG00000029068	45.66734431	47.9623827	39.42332234	47.47835055	48.74021822	54.85807751	57.98242036	52.15006055	62.70195285	59.35566064	58.9439439	60.01463789	Ccnl2	cyclin L2 [Source:MGI Symbol;Acc:MGI:1927119]
ENSMUSG00000029070	291.6636255	291.0222643	271.1425618	208.5263036	201.6113324	186.5543052	23.86250215	23.91887087	24.17814497	84.95924455	92.31838756	85.96555478	Mxra8	matrix-remodelling associated 8 [Source:MGI Symbol;Acc:MGI:1922011]
ENSMUSG00000029071	38.9543084	40.51859717	38.94139782	45.69862504	47.1032778	46.95320022	25.31757411	24.63110361	28.3881336	19.28719572	20.41508284	19.48031995	Dvl1	dishevelled segment polarity protein 1 [Source:MGI Symbol;Acc:MGI:94941]
ENSMUSG00000029072	0.29312623	0.379743662	0.27509211	0.250745533	0.295143719	0.213966073	0.021808035	0.012099304	0.034103734	0.043699082	0.05083292	0.03247196	Tas1r3	taste receptor, type 1, member 3 [Source:MGI Symbol;Acc:MGI:1933547]
ENSMUSG00000029073	18.49730396	17.99729533	14.75159643	16.92292169	15.20800901	15.1940862	6.328884004	5.226157572	6.352624035	11.08925315	11.58216203	12.1265559	Cptp	ceramide-1-phosphate transfer protein [Source:MGI Symbol;Acc:MGI:1933107]
ENSMUSG00000029074	0.031755342	0	0	0	0.033364073	0.072562407	0.311854904	0.094374571	0.354678833	0.426066048	0.185031829	0.281423651	Ttll10	tubulin tyrosine ligase-like family, member 10 [Source:MGI Symbol;Acc:MGI:1921855]
ENSMUSG00000029075	2.751821896	1.786087191	3.227083445	2.334504525	2.155280293	2.057903621	0.759363394	1.288688419	0.791669274	0.939822435	1.08283577	1.55192054	Tnfrsf4	tumor necrosis factor receptor superfamily, member 4 [Source:MGI Symbol;Acc:MGI:104512]
ENSMUSG00000029076	149.500796	131.2134165	162.508301	118.5461928	108.8129842	97.54084235	62.59310218	66.79339699	59.02817622	74.17998513	72.93470444	71.89415969	Sdf4	stromal cell derived factor 4 [Source:MGI Symbol;Acc:MGI:108079]
ENSMUSG00000029082	24.08290601	24.63390322	22.48529258	7.48280225	7.253893153	6.397455702	0.922150635	1.184799304	1.037280614	4.692468421	5.927998342	5.853629715	Bst1	bone marrow stromal cell antigen 1 [Source:MGI Symbol;Acc:MGI:105370]
ENSMUSG00000029084	6.736155368	7.422880231	5.602020256	3.790983457	3.810913156	5.872801386	6.161920234	5.790216306	7.195729055	10.40064987	10.47246793	10.02917435	Cd38	CD38 antigen [Source:MGI Symbol;Acc:MGI:107474]
ENSMUSG00000029086	0.234807573	0.202794743	0.360591407	0.383457496	0.061675741	0.059616227	1.275254364	0.911057227	1.129171166	3.447989711	3.257560298	3.57225028	Prom1	prominin 1 [Source:MGI Symbol;Acc:MGI:1100886]
ENSMUSG00000029088	0.189645637	0.2183867	0.129438541	0.196637991	0.354227764	0.320999223	0.075249469	0.104372791	0.235352506	0.320418917	0.701603829	0.16806863	Kcnip4	Kv channel interacting protein 4 [Source:MGI Symbol;Acc:MGI:1933131]
ENSMUSG00000029089	8.775866726	7.810347378	8.910003147	9.301088086	9.450326434	9.70259142	5.360716924	5.900187959	8.100336862	7.980273253	9.409518134	9.737894305	5730480H06	RIKEN cDNA 5730480H06 gene [Source:MGI Symbol;Acc:MGI:1917842]
ENSMUSG00000029090	34.15057553	41.78402719	34.17137165	43.32880678	43.97329599	52.28292724	19.552636	20.07184058	22.48625977	38.07292719	36.38335374	37.36531207	Adgra3	adhesion G protein-coupled receptor A3 [Source:MGI Symbol;Acc:MGI:1917943]
ENSMUSG00000029092	0.410024828	0.361067098	0.592631169	0.855287084	0.886934497	1.157378531	0	0	0	0.258887666	0.140537067	0.085499786	D5Ertd615e	DNA segment, Chr 5, ERATO Doi 615, expressed [Source:MGI Symbol;Acc:MGI:1277236]
ENSMUSG00000029093	22.07992802	26.45582704	22.0261251	24.52120784	29.27612473	35.06736814	2.020119553	1.753480588	1.800343537	7.083875066	7.85297906	6.872979005	Sorcs2	sortilin-related VPS10 domain containing receptor 2 [Source:MGI Symbol;Acc:MGI:1932289]
ENSMUSG00000029094	31.73792567	34.95315585	26.70622459	33.66562526	35.72125253	44.17227609	4.819203368	4.619549997	6.484148342	16.31788656	16.65572929	16.10473413	Afap1	actin filament associated protein 1 [Source:MGI Symbol;Acc:MGI:1917542]
ENSMUSG00000029095	0.098802498	0.085332116	0.177018227	0.092200868	0.233567661	0.112884112	2.954988775	2.740582631	2.712858714	1.502400524	1.377572134	1.597991988	Ablim2	actin-binding LIM protein 2 [Source:MGI Symbol;Acc:MGI:2385758]
ENSMUSG00000029096	13.78944452	15.41106982	15.29688373	8.536566154	7.902556436	6.99351149	0.453718749	0.604145873	0.37841718	2.515355283	2.425394717	3.152720466	Htra3	HtrA serine peptidase 3 [Source:MGI Symbol;Acc:MGI:1925808]
ENSMUSG00000029097	5.929362174	6.032928283	6.507010058	5.874269999	6.379087865	6.929099219	5.130440633	5.069237197	5.632207246	5.903062327	6.473743893	8.116064188	Trmt44	tRNA methyltransferase 44 [Source:MGI Symbol;Acc:MGI:1926140]
ENSMUSG00000029098	19.91416202	22.06880254	18.45963395	20.68253937	20.78852548	21.88184718	4.89777145	4.510767564	6.093326071	8.701911649	9.681059416	9.010580114	Acox3	acyl-Coenzyme A oxidase 3, pristanoyl [Source:MGI Symbol;Acc:MGI:1933156]
ENSMUSG00000029101	27.8285778	28.61346963	24.92644511	28.26244262	30.95968919	34.76715251	9.304073593	8.385412662	9.805794371	19.55945832	21.30829451	22.40383771	Rgs12	regulator of G-protein signaling 12 [Source:MGI Symbol;Acc:MGI:1918979]
ENSMUSG00000029102	0.830247668	0.290978699	0.702175129	0.774773482	1.592910546	0.769859545	0.161136133	0.286079515	0.100794844	0.064577125	0.210334	0.447869686	Hgfac	hepatocyte growth factor activator [Source:MGI Symbol;Acc:MGI:1859281]
ENSMUSG00000029103	136.3870617	120.6473999	131.9481369	95.67746613	87.74053261	81.53410659	36.47440367	38.72324444	36.82498936	66.52616129	71.71855808	70.00563244	Lrpap1	low density lipoprotein receptor-related protein associated protein 1 [Source:MGI Symbol;Acc:MGI:96829]
ENSMUSG00000029104	12.61329527	15.74984604	12.17993598	15.93375899	17.8141759	23.81159925	16.22262001	13.77490184	19.22642163	19.58381135	21.56783092	20.43246457	Htt	huntingtin [Source:MGI Symbol;Acc:MGI:96067]
ENSMUSG00000029106	197.2823957	220.4019755	207.2525328	216.6670885	226.1816278	232.9205824	65.42622797	59.04241062	70.79416356	79.36031889	78.8636933	77.89979548	Add1	adducin 1 (alpha) [Source:MGI Symbol;Acc:MGI:87918]
ENSMUSG00000029108	36.89914374	45.15582288	30.60724134	50.32967388	56.17418047	71.52911255	1.076846596	0.975419856	1.603798974	17.46050191	18.53338894	17.37577722	Pcdh7	protocadherin 7 [Source:MGI Symbol;Acc:MGI:1860487]
ENSMUSG00000029110	141.5214426	155.8407052	123.555669	163.0380776	178.8588574	184.5196306	156.2993581	151.8049618	179.6424369	178.3745442	173.333434	173.2267404	Rnf4	ring finger protein 4 [Source:MGI Symbol;Acc:MGI:1201691]
ENSMUSG00000029111	16.84470762	17.66012474	16.60024874	19.17671645	18.27997249	17.80532679	42.08071897	37.53647663	42.71553977	27.62318758	28.9491923	29.01011924	Nelfa	negative elongation factor complex member A, Whsc2 [Source:MGI Symbol;Acc:MGI:1346098]

ENSMUSG00000029112	0.142360588	0.409838839	0.364369315	0.03321214	0.03739315	0.027108371	0	0.141028446	0	0.063669092	0.088875622	0	Nkx1-1	NK1 homeobox 1 [Source:MGI Symbol;Acc:MGI:109346]
ENSMUSG00000029119	59.3901114	67.68793624	59.91394421	70.71218028	57.7374104	58.53908541	8.131458472	7.162444676	9.722789104	24.33581725	24.62043814	27.02380971	Man2b2	mannosidase 2, alpha B2 [Source:MGI Symbol;Acc:MGI:1195262]
ENSMUSG00000029120	3.226491289	3.210166579	2.69545862	3.275868618	3.606902424	3.637188853	3.687088573	3.426432869	4.18029536	11.31319947	11.1511448	12.00946308	Ppp2r2c	protein phosphatase 2, regulatory subunit B, gamma [Source:MGI Symbol;Acc:MGI:2442660]
ENSMUSG00000029121	12.45037264	8.258252604	11.18301722	5.948663383	5.825454602	5.61406835	21.78620196	20.88534976	21.13705219	34.00364313	36.89469812	33.24859745	Crmp1	collapsin response mediator protein 1 [Source:MGI Symbol;Acc:MGI:107793]
ENSMUSG00000029122	38.00070257	36.21961876	37.73170995	43.62326206	42.53021688	42.16092763	15.61647329	17.23566885	19.15394203	19.53682641	19.69928151	18.77631068	Evc	EvC ciliary complex subunit 1 [Source:MGI Symbol;Acc:MGI:1890596]
ENSMUSG00000029123	0.099545374	0.114631615	0.025478456	0.06967058	0.039220635	0.009477739	0.01110898	0.049306938	0.011581594	0.055650617	0.248584445	0.11027445	Stk32b	serine/threonine kinase 32B [Source:MGI Symbol;Acc:MGI:1927552]
ENSMUSG00000029125	17.93488298	14.20711775	16.88019853	16.09692519	15.1227826	16.53202811	6.867080108	5.620576378	6.52127777	10.21804417	10.84015169	10.02254221	Stx18	syntaxin 18 [Source:MGI Symbol;Acc:MGI:1918366]
ENSMUSG00000029126	77.80870323	82.84636798	66.66480035	111.3093747	108.7118028	99.85779877	2.3318921	1.634217803	1.748684997	7.992708671	7.247291098	7.391042229	Nsg1	neuron specific gene family member 1 [Source:MGI Symbol;Acc:MGI:109149]
ENSMUSG00000029127	6.34228122	7.303462851	5.165578927	8.998779416	7.084700848	9.176725778	4.167748551	3.129788269	4.805898183	4.618555965	5.043801095	5.578896193	Zbtb49	zinc finger and BTB domain containing 49 [Source:MGI Symbol;Acc:MGI:1922329]
ENSMUSG00000029128	34.51185311	36.05640691	35.61792545	46.90632663	44.47726534	40.85092999	16.47415799	16.59816321	17.25273927	16.00760826	14.29431576	14.94730934	Rab28	RAB28, member RAS oncogene family [Source:MGI Symbol;Acc:MGI:1917285]
ENSMUSG00000029130	2.252085802	2.357629609	2.235800169	4.012163527	3.836074594	4.028527517	4.691425477	3.88736914	4.573416193	3.326874175	2.243880383	2.661138472	Rnf32	ring finger protein 32 [Source:MGI Symbol;Acc:MGI:1861747]
ENSMUSG00000029131	170.0470807	196.3588883	142.3522495	178.6742092	186.7064065	191.8854505	167.7923921	154.6350348	172.7447573	264.7098124	259.0858263	254.0250922	Dnajb6	DnaJ heat shock protein family (Hsp40) member B6 [Source:MGI Symbol;Acc:MGI:1344381]
ENSMUSG00000029134	0.423385267	0.704238742	0.288972487	0.438995749	0.477784706	0.621082024	0.027999165	0.015534201	0.058380687	0.252472083	0.404636713	0.305730285	Plb1	phospholipase B1 [Source:MGI Symbol;Acc:MGI:1922406]
ENSMUSG00000029135	43.7116546	57.0358701	40.23927694	56.43080317	65.90805541	81.2174642	1.898608665	1.713766476	2.184499174	15.65144446	17.37187251	18.69049413	Fosl2	fos-like antigen 2 [Source:MGI Symbol;Acc:MGI:102858]
ENSMUSG00000029136	5.564586396	3.725527491	4.835810924	4.709731235	4.962717653	4.977708397	2.830568195	3.012653898	2.288522944	2.025682441	1.72351882	1.777624128	Rbks	ribokinase [Source:MGI Symbol;Acc:MGI:1918586]
ENSMUSG00000029138	0.058970061	0.045271371	0.020124372	0.018343297	0.041304996	0.029944286	0.42117702	0.584182472	0.402504035	0.685715253	0.605401733	0.57486791	4930548H24	RIKEN cDNA 4930548H24 gene [Source:MGI Symbol;Acc:MGI:1914906]
ENSMUSG00000029141	32.70165075	34.79477097	30.83657514	35.98941347	37.20477162	35.00750945	26.8619424	30.18541511	25.5721249	30.10614258	30.93516801	31.43846142	Slc4a1ap	solute carrier family 4 (anion exchanger), member 1, adaptor protein [Source:MGI Symbol;Acc:MGI:1196608]
ENSMUSG00000029145	60.17970326	50.04451578	59.0590607	53.99258858	49.0923497	38.20907301	83.89098002	90.70235325	77.01759908	69.1322313	71.3475022	66.323064	Eif2b4	eukaryotic translation initiation factor 2B, subunit 4 delta [Source:MGI Symbol;Acc:MGI:95300]
ENSMUSG00000029146	111.2598525	100.4444409	120.4345459	104.3554311	99.57053203	80.49848668	55.49413389	55.5557984	51.26539954	44.31042831	46.65363377	44.70040263	Snx17	sorting nexin 17 [Source:MGI Symbol;Acc:MGI:2387801]
ENSMUSG00000029147	137.0867475	126.3761225	134.7203264	140.3894506	132.721946	117.4603362	232.2107266	240.3299467	234.053135	172.3334093	167.8960959	161.9436869	Ppm1g	protein phosphatase 1G (formerly 2C), magnesium-dependent, gamma isoform [Source:MGI Symbol;Acc:MGI:106065]
ENSMUSG00000029148	129.0854635	115.7241977	131.3701033	111.7429021	107.284778	92.65437413	47.76583829	50.51803943	49.94851886	56.79145414	56.11534908	57.91614096	Nrbp1	nuclear receptor binding protein 1 [Source:MGI Symbol;Acc:MGI:2183436]
ENSMUSG00000029149	5.733624906	6.966007698	5.06223902	6.03774789	5.029287249	5.889707865	16.67148312	20.58338611	15.37339068	7.481157278	8.538252712	7.08582092	Krtcap3	keratinocyte associated protein 3 [Source:MGI Symbol;Acc:MGI:1917065]
ENSMUSG00000029151	1.813461173	1.661142765	1.94099953	1.15383557	1.342392595	0.941782202	3.642787886	3.878788823	3.260706781	2.396281431	2.984739623	2.593329725	Slc30a3	solute carrier family 30 (zinc transporter), member 3 [Source:MGI Symbol;Acc:MGI:1345280]
ENSMUSG00000029152	181.1247872	173.3830859	175.4581102	190.6638355	188.7961022	175.9741449	107.1998931	108.43296	108.5748101	97.78192755	97.13001703	91.1846893	Ociad1	OCIA domain containing 1 [Source:MGI Symbol;Acc:MGI:1915345]
ENSMUSG00000029153	10.17249576	8.994314249	11.58765712	9.754676409	8.427420214	9.20878732	3.507443198	3.891925225	2.829559618	5.773194956	4.846957606	5.761384513	Ociad2	OCIA domain containing 2 [Source:MGI Symbol;Acc:MGI:1916377]
ENSMUSG00000029154	0	0.052600335	0	0	0.047991845	0	0.713651748	0.384628137	0.340120183	0.388148281	0.399232688	0.667933044	Cwh43	cell wall biogenesis 43 C-terminal homolog [Source:MGI Symbol;Acc:MGI:2444131]
ENSMUSG00000029155	0	0	0	0	0	0	0	0	0	0	0.021370451	0	Spata18	spermatogenesis associated 18 [Source:MGI Symbol;Acc:MGI:1920722]
ENSMUSG00000029156	17.75811004	18.56066562	16.66729843	22.1080325	21.72126731	23.81321782	4.736325537	4.152259047	4.91897814	6.158074619	5.899250079	5.598807653	Sgcb	sarcoglycan, beta (dystrophin-associated glycoprotein) [Source:MGI Symbol;Acc:MGI:1346523]
ENSMUSG00000029158	0.053187253	0	0.108905439	0	0.167645185	0.040511768	0.474843684	0.526895522	0.396036109	0.380597937	0.398457206	0.37708682	Yipf7	Yip1 domain family, member 7 [Source:MGI Symbol;Acc:MGI:1922831]
ENSMUSG00000029161	4.776662928	4.12542968	5.126940489	1.042480624	0.566621716	0.498798882	10.83319519	10.72324369	9.788199074	11.88750976	12.50544444	12.25578584	Cgref1	cell growth regulator with EF hand domain 1 [Source:MGI Symbol;Acc:MGI:1915817]
ENSMUSG00000029162	27.40407495	20.91253321	32.5366905	28.89370981	22.77605392	15.95085415	10.99132232	13.08759392	9.783870667	7.792715625	11.57579626	11.59936264	Khk	ketohexokinase [Source:MGI Symbol;Acc:MGI:1096353]
ENSMUSG00000029163	104.5600035	107.0018654	110.6165545	72.71192171	74.57328752	70.88196868	4.876533012	4.933190331	4.910185304	19.06998695	20.32429571	19.79049484	Emilin1	elastin microfibril interfacer 1 [Source:MGI Symbol;Acc:MGI:1926189]
ENSMUSG00000029165	5.403392747	5.398746855	4.698093165	5.135047719	5.635385944	7.020842833	8.725811728	8.344117925	10.37284118	6.556814216	7.226211719	6.637167621	Agbl5	ATP/GTP binding protein-like 5 [Source:MGI Symbol;Acc:MGI:2441745]
ENSMUSG00000029166	42.50412633	41.5221389	45.41013318	36.98725378	35.55091414	33.7137775	5.617656079	5.77511485	6.918885675	13.07763158	13.40244373	13.33968279	Mapre3	microtubule-associated protein, RP/EB family, member 3 [Source:MGI Symbol;Acc:MGI:2140967]
ENSMUSG00000029167	4.147153597	5.395029497	3.680681431	6.08906358	6.781247752	9.972358253	0.063182326	0.056086642	0.079044378	0.037981546	0.035345601	0.012543719	Ppargc1a	peroxisome proliferative activated receptor, gamma, coactivator 1 alpha [Source:MGI Symbol;Acc:MGI:1342774]
ENSMUSG00000029168	0.653183876	0.940218513	0.689622513	0.990503248	1.372548565	1.181606057	5.448786227	5.702319284	5.642598673	10.16973305	9.973674529	8.79155203	Dpysl5	dihydropyrimidinase-like 5 [Source:MGI Symbol;Acc:MGI:1929772]
ENSMUSG00000029169	67.54966371	81.2378882	53.62972765	83.1035273	96.86281103	114.0690149	120.3785945	111.2283885	143.0272992	156.795447	157.2402971	160.4094282	Dhx15	DEAH (Asp-Glu-Ala-His) box polypeptide 15 [Source:MGI Symbol;Acc:MGI:1099786]
ENSMUSG00000029171	42.39102875	47.48495247	33.20234413	52.01362546	51.61283011	59.08427609	66.08777673	65.47803827	67.04524625	49.74926024	45.53386114	48.51352874	Pgm2	phosphoglucomutase 2 [Source:MGI Symbol;Acc:MGI:97564]
ENSMUSG00000029173	5.451048323	6.381039748	4.037133441	6.529900708	9.206845306	9.923490525	5.487836296	5.463870265	6.776810605	9.440444582	9.761412604	9.42187672	Sepsecs	Sep (O-phosphoserine) tRNA:Sec (selenocysteine) tRNA synthase [Source:MGI Symbol;Acc:MGI:1098791]
ENSMUSG00000029174	19.87641724	22.34781371	16.59077851	24.97336384	27.88344139	32.82109622	20.97863763	19.86185722	26.05898541	20.36622639	21.36048075	20.14547881	Tbc1d1	TBC1 domain family, member 1 [Source:MGI Symbol;Acc:MGI:1889508]
ENSMUSG00000029175	40.23767594	40.43585835	37.9753425	40.96976344	39.91286189	35.12182076	6.972686484	6.478468624	8.044723079	11.77366638	11.35530176	11.94191158	Slc35f6	solute carrier family 35, member F6 [Source:MGI Symbol;Acc:MGI:1922169]
ENSMUSG00000029176	150.3022755	160.7505023	142.7130502	154.1375439	146.8332847	154.4154779	66.80998123	77.15867731	77.66213346	89.95897025	85.06920563	87.96927229	Anapc4	anaphase promoting complex subunit 4 [Source:MGI Symbol;Acc:MGI:1098673]
ENSMUSG00000029177	74.02480252	60.88220951	76.26407922	74.81015236	71.85028225	70.52706611	150.4207683	154.4564314	144.3842115	85.71443596	90.22982487	91.92640129	Cenpa	centromere protein A [Source:MGI Symbol;Acc:MGI:88375]
ENSMUSG00000029178	51.40531046	64.04538456	40.95921986	67.96189706	81.23069116	100.5534846	34.04209941	34.75820453	36.21465829	43.76982559	41.05604609	40.54663642	Klf3	Kruppel-like factor 3 (basic) [Source:MGI Symbol;Acc:MGI:1342773]
ENSMUSG00000029179	14.9957334	14.8187734	13.71376952	20.14552853	19.20744999	22.07393656	13.95194406	15.60652672	15.19240941	18.68823428	16.5672772	17.76922431	Zcchc4	zinc finger, CCHC domain containing 4 [Source:MGI Symbol;Acc:MGI:1926046]
ENSMUSG00000029182	0	0	0	0	0	0	0.054566554	0.060548079	0	0.109340814	0	0	Fam166c	family with sequence similarity 166, member C [Source:MGI Symbol;Acc:MGI:1922684]
ENSMUSG00000029184	0	0	0	0	0	0	0	0	0	0	0	0	Olfr109	olfactory receptor 109 [Source:MGI Symbol;Acc:MGI:2177492]
ENSMUSG00000029185	120.4895618	133.8147135	106.6330222	108.5537961	128.1116773	130.8908952	9.576383656	9.413106948	10.84996934	27.07520002	24.9107694	27.43311982	Fam114a1	family with sequence similarity 114, member A1 [Source:MGI Symbol;Acc:MGI:1915553]
ENSMUSG00000029186	40.15688324	49.49702673	36.53910159	52.70143966	60.31766627	72.63056268	21.99494558	20.77106985	25.98528105	36.49017998	35.57025504	36.98164644	Pi4k2b	phosphatidylinositol 4-kinase type 2 beta [Source:MGI Symbol;Acc:MGI:1914323]
ENSMUSG00000029188	0.077389367	0.022279458	0	0	0.060982444	0	0.017272866	0.057498895	0.036015424	0.051917221	0.064418841	0.085730451	Slc34a2	solute carrier family 34 (sodium phosphate), member 2 [Source:MGI Symbol;Acc:MGI:1342284]
ENSMUSG00000029189	0.12887604	0.241162	0.082463965	0.045099376	0.101553692	0.110432866	0.086293195	0.191505119	0.089964399	0.302601009	0.442514748	0.49968239	Sel1l3	sel-1 suppressor of lin-12-like 3 (C. elegans) [Source:MGI Symbol;Acc:MGI:1916941]
ENSMUSG00000029190	21.74758712	24.82849915	20.39481308	24.22647082	25.97341872	29.46310315	7.916293138	6.76307221	7.061657024	11.62868522	9.37876039	10.05612082	D5Ertd579e	DNA segment, Chr 5, ERATO Doi 579, expressed [Source:MGI Symbol;Acc:MGI:1261849]
ENSMUSG00000029191	21.84151085	28.821957	20.21974976	31.88111782	35.31152379	42.33310999	59.08316345	55.42487869	68.74839469	71.00673426	63.2006771	67.04783539	Rfc1	replication factor C (activator 1) 1 [Source:MGI Symbol;Acc:MGI:97891]
ENSMUSG00000029192	21.79790027	23.31741912	18.36255054	22.76449707	26.61605355	30.91261581	26.16184027	24.36071519	29.93531914	30.19314603	30.25908446	29.50852564	Tbc1d14	TBC1 domain family, member 14 [Source:MGI Symbol;Acc:MGI:1098708]
ENSMUSG00000029193	0.117617734	0.045147622	0.020069362	0.091465781	0.123576269	0.0895873	0.052503217	0	0.054736881	0.035068762	0.146857348	0.156353596	Cckar	cholecystokinin A receptor [Source:MGI Symbol;Acc:MGI:99478]
ENSMUSG00000029195	0.158451878	0.121643655	0.090123261	0.164294099	0.184976753	0.174329897	0.141462229	0	0.081933616	0.173227345	0.278445431	0.280847907	Klb	klotho beta [Source:MGI Symbol;Acc:MGI:1932466]
ENSMUSG00000029196	5.841504412	10.42943639	4.636171871	8.373162632	10.36466141	11.34781231	4.223229262	3.818982443	5.562382156	6.459834731	6.950379592	6.683701604	Tada2b	transcriptional adaptor 2B [Source:MGI Symbol;Acc:MGI:3035274]
ENSMUSG00000029198	33.41781651	30.32239349	37.63420694	33.6319521	29.60068463	25.06357927	38.20797135	39.42407626	30.99041487	29.37871129	29.54283096	30.30744466	Grpel1	GrpE-like 1, mitochondrial [Source:MGI Symbol;Acc:MGI:1334417]
ENSMUSG00000029199	33.11138373	29.97764478	30.99354677	31.44696179	36.77674419	31.45709638	46.97737651	47.73190072	49.64394446	35.54082111	38.74956885	38.27745702	Lias	lipoic acid synthetase [Source:MGI Symbol;Acc:MGI:1934604]
ENSMUSG00000029201	73.86615715	87.51181827	65.28593907	92.98008931	100.3602488	102.5561545	25.66225329	24.08377822	28.24398462	35.04198737	33.03469791	32.95705663	Ugdh	UDP-glucose dehydrogenase [Source:MGI Symbol;Acc:MGI:1306785]
ENSMUSG00000029202	28.99137637	37.09053337	26.32312927	38.13710015	44.37536162	54.74966453	51.01625851	46.78371579	61.8823053	77.86582792	77.36600379	80.5778033	Pds5a	PDS5 cohesin associated factor A [Source:MGI Symbol;Acc:MGI:1918771]
ENSMUSG00000029203	66.52268638	61.44052664	61.93695944	67.22303951	64.1595817	61.94015701	68.15211231	70.96800095	64.52657218	53.41878908	56.06736782	57.57715992	Ube2k	ubiquitin-conjugating enzyme E2K [Source:MGI Symbol;Acc:MGI:1858216]
ENSMUSG00000029204	0.191359222	0.141659987	0.44779924	0.140307578	0.100526756	0.072877428	0.061014698	0.027081225	0.012722094	0.122261647	0.295819175	0.314947752	Rhoh	ras homolog family member H [Source:MGI Symbol;Acc:MGI:1921984]
ENSMUSG00000029205	0	0	0	0	0	0.545476072	25.0415831	22.87961589	24.05170958	12.54462149	12.96560957	13.2222277	Chrna9	cholinergic receptor, nicotinic, alpha polypeptide 9 [Source:MGI Symbol;Acc:MGI:1202403]
ENSMUSG00000029206	1.418692896	1.525981459	1.372642579	1.600319769	1.588843072	1.899943225	0.18093953	0.077220739	0.130595059	0.446237291	0.454199508	0.414488094	Nsun7	NOL1/NOP2/Sun domain family, member 7 [Source:MGI Symbol;Acc:MGI:1918168]
ENSMUSG00000029207	22.55002706	26.69591691	21.22473356	20.6448789	24.04132181	28.07653146	8.866052318	9.790942599	10.75925386	20.49579687	21.32426986	20.82524674	Apbb2	amyloid beta (A4) precursor protein-binding, family B, member 2 [Source:MGI Symbol;Acc:MGI:108405]
ENSMUSG00000029208	13.03991866	14.66127771	11.34100523	18.64646992	18.21624286	22.86645767	13.10420013	11.93780808	13.02767349	12.14264148	12.63648528	13.03676764	Guf1	GUF1 homolog, GTPase [Source:MGI Symbol;Acc:MGI:2140726]

ENSMUSG00000029209	5.16846094	10.08893946	2.688735974	8.842391848	11.74356707	14.89873692	2.71980168	2.788995894	3.871939454	9.095788928	8.8143092	9.347030349	Gnpda2	glucosamine-6-phosphate deaminase 2 [Source:MGI Symbol;Acc:MGI:1915230]
ENSMUSG00000029211	0.197197469	0.181666418	0.100944661	0.257630014	0.580125075	0.465625408	0.105632046	0.058605657	0.128480321	0.723192769	0.869979102	0.908758499	Gabra4	gamma-aminobutyric acid (GABA) A receptor, subunit alpha 4 [Source:MGI Symbol;Acc:MGI:95616]
ENSMUSG00000029212	0.230890345	0.368144447	0.363667448	0.110493885	0.08086243	0.108224614	0.02642739	0.058648662	0.01102068	0.190639349	0.152768731	0.225607444	Gabrb1	gamma-aminobutyric acid (GABA) A receptor, subunit beta 1 [Source:MGI Symbol;Acc:MGI:95619]
ENSMUSG00000029213	34.90814414	37.24274873	34.25755249	41.3293389	39.71663543	37.50172643	27.62369658	27.46177173	27.40890956	27.82237482	27.42593719	27.40027045	Commd8	COMM domain containing 8 [Source:MGI Symbol;Acc:MGI:1343485]
ENSMUSG00000029217	2.199820441	2.001232993	1.734163224	0.882719605	0.993843399	1.139383131	1.924670603	1.656219031	1.986077555	4.742126736	4.596132794	4.795856111	Tec	tec protein tyrosine kinase [Source:MGI Symbol;Acc:MGI:98662]
ENSMUSG00000029219	0.024406403	0.056210454	0	0.022775654	0	0	0	0.0379251	0	0.065492853	0.223474549	0.2379251	Slc10a4	solute carrier family 10 (sodium/bile acid cotransporter family), member 4 [Source:MGI Symbol;Acc:MGI:3606480]
ENSMUSG00000029221	42.17313716	43.37252075	35.94829851	43.64390819	47.72158403	52.84921252	22.57260967	22.33261226	24.78783401	26.53179892	25.80070073	25.32343502	Slc30a9	solute carrier family 30 (zinc transporter), member 9 [Source:MGI Symbol;Acc:MGI:1923690]
ENSMUSG00000029223	116.7574127	77.73554051	141.4083358	109.6821008	77.21253815	58.30037885	84.46993642	98.53151443	75.36370238	98.54846466	101.373215	90.16124892	Uchl1	ubiquitin carboxy-terminal hydrolase L1 [Source:MGI Symbol;Acc:MGI:103149]
ENSMUSG00000029227	57.57541036	60.87928711	60.37190128	68.42641242	75.15494728	76.77290212	52.04289111	47.58282803	54.82685611	47.83612307	46.04524718	45.50501678	Fip1l1	FIP1 like 1 (S. cerevisiae) [Source:MGI Symbol;Acc:MGI:1914149]
ENSMUSG00000029228	0.12477192	0.164207177	0.036497308	0.166335865	0.093637776	0.3937227	2.323350743	1.394963533	2.057205795	2.646645575	3.17514969	3.996530607	Lnx1	ligand of numb-protein X 1 [Source:MGI Symbol;Acc:MGI:1278335]
ENSMUSG00000029229	20.99217616	18.54955448	18.74160312	23.01556489	19.63155742	20.9150844	7.329644368	8.158136801	6.348299948	8.714398648	8.109613489	8.566844341	Chic2	cysteine-rich hydrophobic domain 2 [Source:MGI Symbol;Acc:MGI:1921527]
ENSMUSG00000029231	206.0978353	252.2963861	178.1508304	201.6450608	245.458935	299.288297	1.707326848	2.034238998	3.122228935	21.97109046	22.49482453	23.0603358	Pdgfra	platelet derived growth factor receptor, alpha polypeptide [Source:MGI Symbol;Acc:MGI:97530]
ENSMUSG00000029233	27.97911524	25.7104219	23.29199436	24.75801466	27.16953122	25.67029203	7.506350796	7.384280677	7.200955816	12.1657365	12.26242781	13.39973991	Srd5a3	steroid 5 alpha-reductase 3 [Source:MGI Symbol;Acc:MGI:1930252]
ENSMUSG00000029234	113.3879396	131.8590416	97.46625368	120.2708067	123.3487883	145.9933647	83.1792708	77.90414668	90.78438126	100.0141842	99.75443832	91.57299662	Tmem165	transmembrane protein 165 [Source:MGI Symbol;Acc:MGI:894407]
ENSMUSG00000029235	0.038590572	0	0.118526234	0	0	0.176362443	0.17226392	0.344065138	0.359185215	3.693464037	4.304449609	3.077992307	Pdcl2	phosducin-like 2 [Source:MGI Symbol;Acc:MGI:1890655]
ENSMUSG00000029236	0	0	0	0	0	0	0.046394751	0	0	0.139449154	0.173028384	0.046054233	Nmu	neuromedin U [Source:MGI Symbol;Acc:MGI:1860476]
ENSMUSG00000029238	6.220986192	8.944613398	5.604360195	8.125811014	9.398012677	14.26877925	3.012314471	2.820252125	3.786554453	8.213176406	7.504209565	7.343134393	Clock	circadian locomotor output cycles kaput [Source:MGI Symbol;Acc:MGI:99698]
ENSMUSG00000029245	0.316536099	0.258682806	0.167260561	0.152457438	0.354028122	0.256654647	0.009116007	0	0.009503833	0.018266714	0.033997983	0.054294596	Epha5	Eph receptor A5 [Source:MGI Symbol;Acc:MGI:99654]
ENSMUSG00000029246	8.899390098	9.898240985	8.450152141	11.04545507	11.91722661	12.75670177	46.33742327	42.24962663	52.82151994	61.64415679	63.64634536	62.14126481	Ppat	phosphoribosyl pyrophosphate amidotransferase [Source:MGI Symbol;Acc:MGI:2387203]
ENSMUSG00000029247	140.243472	139.454188	106.847228	175.0791394	179.2186906	180.7914725	487.0454713	454.9845918	495.6082501	383.7142672	384.5586731	386.7173921	Paics	phosphoribosylaminoimidazole carboxylase, phosphoribosylaminoribosylaminoimidazole, succinocarboxamide synthetase [Source:M
ENSMUSG00000029248	0	0.072387897	0	0	0.132091507	0	0.084181565	0.249091878	0.175525865	0.168683563	0.104651207	0.167127413	Thegl	theg spermatid protein like [Source:MGI Symbol;Acc:MGI:1919118]
ENSMUSG00000029249	8.501895355	10.78648272	7.217636053	9.871159399	11.06188605	14.38216644	29.21933975	25.83013746	34.03372756	44.96526341	44.42136036	43.46077894	Rest	RE1-silencing transcription factor [Source:MGI Symbol;Acc:MGI:104897]
ENSMUSG00000029250	90.89539826	100.7109674	84.30366976	96.43649981	113.3085433	127.9670212	149.7472954	139.8754833	172.120717	212.5820491	212.4442253	207.1690055	Polr2b	polymerase (RNA) II (DNA directed) polypeptide B [Source:MGI Symbol;Acc:MGI:2388280]
ENSMUSG00000029253	14.28800719	18.13422311	11.04989553	19.3764991	23.39255077	27.3403918	15.45404067	14.56162499	18.59903262	27.78562694	25.37813012	24.74175087	Cenpc1	centromere protein C1 [Source:MGI Symbol;Acc:MGI:99700]
ENSMUSG00000029254	0.965847089	0.889778058	0.964106486	0.157729711	0.355171974	0.14713365	0.215571572	0.382723661	0.314639799	4.427628006	3.899255804	3.274037271	Stap1	signal transducing adaptor family member 1 [Source:MGI Symbol;Acc:MGI:1926193]
ENSMUSG00000029255	0	0	0.053396667	0	0	0.019863043	0	0	0	0	0.065121592	0.023110851	Gnrhr	gonadotropin releasing hormone receptor [Source:MGI Symbol;Acc:MGI:95790]
ENSMUSG00000029260	0.023372345	0.080743371	0	0.021810689	0.024556392	0	0	0.011576819	0.02175402	0.010453006	0	0	Ugt2b34	UDP glucuronosyltransferase 2 family, polypeptide B34 [Source:MGI Symbol;Acc:MGI:2140962]
ENSMUSG00000029263	3.299981933	4.339119943	2.40807786	4.335308866	6.18433074	7.567349339	3.708804241	4.393580712	5.184497178	5.380150861	5.435359401	6.263876647	Pigg	phosphatidylinositol glycan anchor biosynthesis, class G [Source:MGI Symbol;Acc:MGI:3576484]
ENSMUSG00000029265	34.9425975	45.94692221	24.68699927	54.70574497	63.23602024	71.13868797	33.1081041	29.60922978	42.40103123	45.13941022	46.5539254	48.1566548	Dr1	down-regulator of transcription 1 [Source:MGI Symbol;Acc:MGI:1100515]
ENSMUSG00000029267	18.10894954	18.56808369	13.71437978	22.77047328	25.5185319	29.32141154	94.70831947	89.7421542	117.5446795	146.0041408	138.7434626	148.9141891	Mtf2	metal response element binding transcription factor 2 [Source:MGI Symbol;Acc:MGI:105050]
ENSMUSG00000029268	0	0	0	0	0	0	0	0	0	0	0	0	Ugt2a2	UDP glucuronosyltransferase 2 family, polypeptide A2 [Source:MGI Symbol;Acc:MGI:3576095]
ENSMUSG00000029269	0	0	0.020556346	0	0.084383235	0	0	0.019890736	0	0.017959854	0	0	Sult1b1	sulfotransferase family 1B, member 1 [Source:MGI Symbol;Acc:MGI:2136282]
ENSMUSG00000029270	32.87350432	37.59316503	30.25676489	35.29375283	34.94924459	35.55063963	8.020045177	8.222081062	9.421547749	6.288502826	7.260909704	6.980456078	Dipk1a	divergent protein kinase domain 1A [Source:MGI Symbol;Acc:MGI:1914516]
ENSMUSG00000029272	0	0	0	0.064659385	0	0	0	0	0	0	0	0	Sult1e1	sulfotransferase family 1E, member 1 [Source:MGI Symbol;Acc:MGI:98431]
ENSMUSG00000029273	0.23164731	1.933964944	0	0.513402476	0.060845651	0.022055196	0.180958263	0.487644287	0.053901954	0.077701143	0.096411509	0.230952989	Sult1d1	sulfotransferase family 1D, member 1 [Source:MGI Symbol;Acc:MGI:1926341]
ENSMUSG00000029275	1.421946406	2.130920772	1.71502649	1.817721948	2.107947845	2.388690504	0.017390156	0.038592891	0	0.017423253	0	0.034525038	Gfi1	growth factor independent 1 transcription repressor [Source:MGI Symbol;Acc:MGI:103170]
ENSMUSG00000029276	8.954486555	8.264689278	6.455045236	12.11176611	9.619540849	11.95498181	6.22781734	7.109845213	6.742491605	7.319613246	7.984120333	6.776541129	Glmn	glomulin, FKBP associated protein [Source:MGI Symbol;Acc:MGI:2141180]
ENSMUSG00000029279	8.327424527	8.106798164	7.228651094	9.224447611	9.272941746	10.50287148	0.574739612	0.288012484	0.521876009	0.984489487	0.777876157	0.975407311	Brdt	bromodomain, testis-specific [Source:MGI Symbol;Acc:MGI:1891374]
ENSMUSG00000029280	0	0	0	0	0	0	0	0.077360885	0	0	0	0	Smr3a	submaxillary gland androgen regulated protein 3A [Source:MGI Symbol;Acc:MGI:102763]
ENSMUSG00000029281	0	0	0	0	0	0	0	0	0	0	0	0	Smr2	submaxillary gland androgen regulated protein 2 [Source:MGI Symbol;Acc:MGI:102762]
ENSMUSG00000029282	0	0	0	0	0	0	0	0	0	0	0	0	Amtn	amelotin [Source:MGI Symbol;Acc:MGI:1918671]
ENSMUSG00000029283	13.63187007	15.24929058	11.71561999	20.40558206	23.61744907	26.65714523	29.10918684	28.97455251	34.61144345	21.91491533	20.5947034	22.05791297	Cdc7	cell division cycle 7 (S. cerevisiae) [Source:MGI Symbol;Acc:MGI:1309511]
ENSMUSG00000029286	0	0	0	0	0.009365717	0	0	0	0	0	0	0.007899918	Enam	enamelin [Source:MGI Symbol;Acc:MGI:1333772]
ENSMUSG00000029287	67.18895243	83.89879774	59.50580522	86.29783118	99.72810256	123.2245929	2.292589544	1.799344372	2.914785409	15.22431566	14.72819115	15.15325033	Tgfbr3	transforming growth factor, beta receptor III [Source:MGI Symbol;Acc:MGI:104637]
ENSMUSG00000029288	0.075400388	0	0	0.023454134	0	0	0	0	0	0.022481289	0.020921072	0	Ambn	ameloblastin [Source:MGI Symbol;Acc:MGI:104655]
ENSMUSG00000029290	22.66277537	22.68860594	18.78948481	22.87223421	28.43869448	28.04821249	30.38092752	28.85514181	33.30449529	26.13877298	25.36946546	25.28902045	Zfp326	zinc finger protein 326 [Source:MGI Symbol;Acc:MGI:1927246]
ENSMUSG00000029291	9.812376606	11.59828466	9.481279632	12.62269149	13.68348271	14.41661594	14.29154202	14.55673502	14.77878603	12.20067083	11.82645417	11.02447716	Rufy3	RUN and FYVE domain containing 3 [Source:MGI Symbol;Acc:MGI:106484]
ENSMUSG00000029298	16.01691345	20.25746759	11.18677016	19.20273577	25.599032	40.50744885	0.751331531	0.726118541	1.099140752	6.750570758	6.587141452	6.086829581	Gbp9	guanylate-binding protein 9 [Source:MGI Symbol;Acc:MGI:3605620]
ENSMUSG00000029299	0.090522765	0.121615221	0.030892181	0	0	0.022983184	0.094286107	0.029891899	0.084254877	0.539803175	0.464664934	0.574935099	Abcg3	ATP binding cassette subfamily G member 3 [Source:MGI Symbol;Acc:MGI:1351624]
ENSMUSG00000029304	833.0849558	821.8787013	778.1873511	1677.909205	1644.255883	1459.723431	345.5529959	343.1721586	316.2865729	548.4625725	511.8477473	523.5292672	Spp1	secreted phosphoprotein 1 [Source:MGI Symbol;Acc:MGI:98389]
ENSMUSG00000029306	0.041613625	0.071880342	0	0	0	0.031696345	0	0	0	0	0	0	Ibsp	integrin binding sialoprotein [Source:MGI Symbol;Acc:MGI:96389]
ENSMUSG00000029307	0.124539762	0.089633711	0.111565033	0.087163845	0.049068363	0.118574604	0	0	0	0	0	0.013796275	Dmp1	dentin matrix protein 1 [Source:MGI Symbol;Acc:MGI:94910]
ENSMUSG00000029309	4.70912635	5.210142201	4.324872203	8.336585795	8.052125371	7.736356803	0.556438337	0.84611393	0.709024678	1.156290835	0.960753098	0.961505644	Sparcl1	SPARC-like 1 [Source:MGI Symbol;Acc:MGI:108110]
ENSMUSG00000029310	77.29980267	57.5624736	86.989867	81.41128674	72.01501097	55.48868841	75.73093344	82.06296312	61.10910139	33.23768273	36.44731553	33.09885787	Nudt9	nudix (nucleoside diphosphate linked moiety X)-type motif 9 [Source:MGI Symbol;Acc:MGI:1921417]
ENSMUSG00000029311	34.10484577	33.48863815	35.32860262	33.05244407	31.64582284	29.86794984	45.15164621	43.48676384	43.22546354	24.7422641	24.70129343	25.07755062	Hsd17b11	hydroxysteroid (17-beta) dehydrogenase 11 [Source:MGI Symbol;Acc:MGI:2149821]
ENSMUSG00000029312	4.787128012	5.128022316	3.989207651	6.354603866	5.906908348	5.737926856	15.30630667	14.63138196	15.87113853	11.96628881	13.23633561	12.80963034	Klhl8	kelch-like 8 [Source:MGI Symbol;Acc:MGI:2179430]
ENSMUSG00000029313	17.87398227	20.53712058	15.99407424	18.79012448	23.63588428	34.31526089	8.76687541	7.507180185	10.12769	24.79803274	26.03287422	25.58016428	Aff1	AF4/FMR2 family, member 1 [Source:MGI Symbol;Acc:MGI:1100819]
ENSMUSG00000029314	7.803208509	6.892513765	8.53356315	10.42625081	9.572704194	9.320587013	0.277077773	0.219607665	0.144432798	0.733670662	0.996450941	0.766194376	Gpat3	glycerol-3-phosphate acyltransferase 3 [Source:MGI Symbol;Acc:MGI:3603816]
ENSMUSG00000029319	61.10842916	53.52980627	58.84219208	72.07776711	66.62523538	60.75513609	36.43674624	36.9628875	34.8934935	29.41098975	30.91095948	28.66840414	Coq2	coenzyme Q2 4-hydroxybenzoate polyprenyltransferase [Source:MGI Symbol;Acc:MGI:1919133]
ENSMUSG00000029320	0.146521525	0	0	0	0	0	0.784866485	0.532218303	0.409128676	0.611613541	0.284583574	0.302985622	1700016H13	RIKEN cDNA 1700016H13 gene [Source:MGI Symbol;Acc:MGI:1921468]
ENSMUSG00000029321	0.303873502	0.723180313	0.497765539	0.207951156	0.276698747	0.185163958	0.036172179	0.100343333	0.056566598	0.144964092	0.505888072	0.215440139	Slc10a6	solute carrier family 10 (sodium/bile acid cotransporter family), member 6 [Source:MGI Symbol;Acc:MGI:1923000]
ENSMUSG00000029322	179.8255504	147.7476466	196.9192795	30.61050843	27.18370311	19.36646674	4.340379616	5.092958427	4.889117199	39.73757567	38.65431626	42.93666021	Plac8	placenta-specific 8 [Source:MGI Symbol;Acc:MGI:2445289]
ENSMUSG00000029326	31.34984821	26.0565731	26.60322489	27.10955755	32.28617816	25.96347352	50.92656227	50.36285483	50.817244	58.07471771	59.208728	56.9567813	Enoph1	enolase-phosphatase 1 [Source:MGI Symbol;Acc:MGI:1915120]
ENSMUSG00000029328	131.5533097	141.9519059	123.8015822	167.4543675	176.3406807	176.953656	242.9668624	248.2513879	252.0299246	161.0768383	158.4200131	154.6104142	Hnrnpdl	heterogeneous nuclear ribonucleoprotein D-like [Source:MGI Symbol;Acc:MGI:1355299]
ENSMUSG00000029330	3.127238685	3.067668531	2.351824932	1.423115634	1.642832564	1.970264139	2.378308597	2.600769532	2.928672812	5.542681564	5.206221469	4.447983499	Cds1	CDP-diacylglycerol synthase 1 [Source:MGI Symbol;Acc:MGI:1921846]
ENSMUSG00000029331	0	0	0	0	0	0	0	0	0	0	0	0	4930522N0	RIKEN cDNA 4930522N08 gene [Source:MGI Symbol;Acc:MGI:1922344]
ENSMUSG00000029334	28.57139984	31.11356651	24.85422176	63.0338101	66.94390158	68.25105329	0.756056172	0.519340225	0.731919826	2.993909287	2.651858634	2.859074391	Prkg2	protein kinase, cGMP-dependent, type II [Source:MGI Symbol;Acc:MGI:108173]

ENSMUSG00000029335	0.040600574	0.028052189	0.008313314	0.015155115	0.059720368	0.061849444	0.014498907	0	0	0.014526502	0	0.007196245	Bmp3	bone morphogenetic protein 3 [Source:MGI Symbol;Acc:MGI:88179]
ENSMUSG00000029337	0	0	0.085207447	0	0	0.021130897	0.09907119	0.054965632	0.07746451	3.573350843	2.909688143	3.343697639	Fgf5	fibroblast growth factor 5 [Source:MGI Symbol;Acc:MGI:95519]
ENSMUSG00000029338	37.77265911	45.67534209	33.52701458	55.59505018	66.15888467	73.76354242	1.456670641	1.487613477	1.303613321	9.531583618	8.930180698	7.511414447	Antxr2	anthrax toxin receptor 2 [Source:MGI Symbol;Acc:MGI:1919164]
ENSMUSG00000029343	0.257964555	0.059411889	0.316922663	0.048145657	0.054206617	0.039297387	0	0	0	0	0.171783575	0.045722907	Crybb1	crystallin, beta B1 [Source:MGI Symbol;Acc:MGI:104992]
ENSMUSG00000029344	87.69959018	74.13973148	90.27840101	70.8480824	66.21292808	57.40030989	35.58918045	38.06162751	34.12008719	48.37872868	47.35564542	44.5593998	Tpst2	protein-tyrosine sulfotransferase 2 [Source:MGI Symbol;Acc:MGI:1309516]
ENSMUSG00000029345	40.69291229	55.5492482	40.2509426	54.37624765	55.7474879	59.32166157	29.88713072	25.30260827	34.50302685	42.63941716	42.84565689	41.50303614	Tfip11	tuftelin interacting protein 11 [Source:MGI Symbol;Acc:MGI:1930075]
ENSMUSG00000029346	20.32760696	14.67473664	17.80048959	16.94727122	14.79840637	13.00743505	12.98919526	13.18641332	12.58138813	9.46047135	11.08004062	7.681448427	Srrd	SRR1 domain containing [Source:MGI Symbol;Acc:MGI:1917368]
ENSMUSG00000029348	23.58401672	21.34171547	27.02234235	21.68925722	20.03051704	16.0254258	2.23775849	2.294948552	2.156220868	4.484034917	3.951552899	4.308042189	Asphd2	aspartate beta-hydroxylase domain containing 2 [Source:MGI Symbol;Acc:MGI:1920148]
ENSMUSG00000029352	0.782336142	0.831600262	0.924173081	0.224634841	0.569055755	0.41254012	3.438532392	3.338527932	2.52057597	1.830197002	1.602993028	1.919978418	Crybb3	crystallin, beta B3 [Source:MGI Symbol;Acc:MGI:102717]
ENSMUSG00000029359	0.731203061	0.388623652	0.460677126	0.57737027	0.472766728	0.342734856	3.3644382	3.231763616	2.774647099	2.616175492	1.310944462	2.193265106	Tesc	tescalcin [Source:MGI Symbol;Acc:MGI:1930803]
ENSMUSG00000029360	0.200532234	0.280406713	0.029329061	0.120300033	0.33108622	0.349124055	0.038363702	0	0.119987471	0.230620304	0.095384489	0.368127242	Gm9754	predicted gene 9754 [Source:MGI Symbol;Acc:MGI:3704256]
ENSMUSG00000029361	0.518687222	0.740126413	0.461763629	0.599776792	0.639740354	0.772972185	4.499853189	3.730878703	4.796242727	7.392997733	8.015621504	8.424014322	Nos1	nitric oxide synthase 1, neuronal [Source:MGI Symbol;Acc:MGI:97360]
ENSMUSG00000029363	27.00485825	26.12962738	25.46770715	32.57241757	31.99312633	29.21069938	149.9329754	155.7952532	157.3024291	114.0356347	112.9770584	109.4334921	Rfc5	replication factor C (activator 1) 5 [Source:MGI Symbol;Acc:MGI:1919401]
ENSMUSG00000029364	97.36649036	107.8371553	80.87159715	91.02359334	95.57893791	91.01434109	53.96248758	50.60379352	55.82526999	45.01531743	46.61033931	48.28450229	Wsb2	WD repeat and SOCS box-containing 2 [Source:MGI Symbol;Acc:MGI:2144041]
ENSMUSG00000029366	15.87351603	17.74528574	10.51015718	22.37387221	26.60540849	29.52856862	28.06708681	25.74172537	33.57635382	34.02502107	31.97607149	31.89155189	Dck	deoxycytidine kinase [Source:MGI Symbol;Acc:MGI:102726]
ENSMUSG00000029368	129.75554	126.3815788	138.0369175	5.404416928	4.626435248	4.338274144	0	0.142244208	0.044548556	0.385307783	0.398407959	0.424170241	Alb	albumin [Source:MGI Symbol;Acc:MGI:87991]
ENSMUSG00000029369	0.019106789	0.066007348	0.09780695	0.053490423	0.080298967	0.02910659	0	0.056783989	0	0	0	0	Afm	afamin [Source:MGI Symbol;Acc:MGI:2429409]
ENSMUSG00000029370	3.224617355	2.630263124	2.0977262	0.877671647	0.599954264	0.69078732	0.509799	0.399305365	0.46895962	2.253393816	1.92924622	2.560054491	Rassf6	Ras association (RalGDS/AF-6) domain family member 6 [Source:MGI Symbol;Acc:MGI:1920496]
ENSMUSG00000029371	317.6698889	328.8312241	309.416445	32.84222794	30.32297327	29.79976396	2.145332165	1.859766479	1.770643116	9.269353158	7.105034635	8.385246057	Cxcl5	chemokine (C-X-C motif) ligand 5 [Source:MGI Symbol;Acc:MGI:1096868]
ENSMUSG00000029372	4.211467876	3.065390163	3.091385979	0.852751607	0.584410394	0.696031833	0.141883118	0.314872375	0.036979826	2.523218479	1.818954895	2.006994984	Ppbp	pro-platelet basic protein [Source:MGI Symbol;Acc:MGI:1888712]
ENSMUSG00000029373	177.7181031	134.6478007	224.1510744	2.836448765	2.993928367	2.02576728	4.324851121	5.739904436	3.53634892	70.17894089	57.16468206	67.76377103	Pf4	platelet factor 4 [Source:MGI Symbol;Acc:MGI:1888711]
ENSMUSG00000029375	0.283215247	0.046590988	0.020710978	0.075511947	0	0	0.054181741	0	0	0	0	0	Cxcl15	chemokine (C-X-C motif) ligand 15 [Source:MGI Symbol;Acc:MGI:1339941]
ENSMUSG00000029376	17.82673418	18.28799157	17.00018488	16.82561611	18.27823176	17.09349552	2.28227051	2.935849658	2.210915305	4.714877058	4.368830237	4.891918372	Mthfd2l	methylenetetrahydrofolate dehydrogenase (NADP+ dependent) 2-like [Source:MGI Symbol;Acc:MGI:1915871]
ENSMUSG00000029377	15.59365741	20.21351364	10.73560167	25.08619654	30.91644445	34.91767024	0.977122015	0.939668639	1.387361596	5.762006593	5.778594736	5.145355599	Ereg	epiregulin [Source:MGI Symbol;Acc:MGI:107508]
ENSMUSG00000029378	6.268347609	4.689871849	6.780058261	13.25230009	12.24159402	11.03257156	0.948514915	0.94724106	1.28552832	2.850960472	1.532903313	1.663410657	Areg	amphiregulin [Source:MGI Symbol;Acc:MGI:88068]
ENSMUSG00000029379	35.87931506	38.00186599	37.18189483	1.19197298	1.28193725	1.016473835	1.072279932	1.303138454	1.579253206	19.78114355	17.37681819	17.58810535	Cxcl3	chemokine (C-X-C motif) ligand 3 [Source:MGI Symbol;Acc:MGI:3037818]
ENSMUSG00000029380	193.5063015	205.3216827	190.1547202	67.39641818	62.4015669	63.15730181	8.705915937	8.241399732	10.74973685	25.78584716	25.74655001	24.17064426	Cxcl1	chemokine (C-X-C motif) ligand 1 [Source:MGI Symbol;Acc:MGI:108068]
ENSMUSG00000029381	4.548858476	4.396383668	4.88281414	4.548128557	3.87708879	4.295624064	10.53611289	9.541384464	12.25884223	9.839965513	11.81338692	11.00383172	Shroom3	shroom family member 3 [Source:MGI Symbol;Acc:MGI:1351655]
ENSMUSG00000029384	0	0.067598248	0.120197136	0.054779642	0.061675741	0.04471217	0	0.116305178	0	0.052507458	0.097726809	0	2010109A12	RIKEN cDNA 2010109A12 gene [Source:MGI Symbol;Acc:MGI:1922860]
ENSMUSG00000029385	37.83726693	44.77215193	29.86480162	46.88288452	53.30975854	59.82375498	18.57719849	17.75180432	19.6910013	17.79655588	19.45429481	18.07511232	Ccng2	cyclin G2 [Source:MGI Symbol;Acc:MGI:1095734]
ENSMUSG00000029386	19.70853555	21.98485468	19.49000863	20.01746554	20.97391404	20.04315897	12.15147219	11.39805459	12.39817681	10.37275855	9.501819771	9.762409594	Tctn2	tectonic family member 2 [Source:MGI Symbol;Acc:MGI:1915228]
ENSMUSG00000029387	31.51712247	30.62693001	30.76760293	33.34734034	33.48308398	29.02586852	64.48674618	66.65185308	66.71223538	52.34782628	54.07884943	54.64245362	Gtf2h3	general transcription factor IIH, polypeptide 3 [Source:MGI Symbol;Acc:MGI:1277143]
ENSMUSG00000029388	67.64731884	62.09369679	66.18989998	67.29519576	59.8008768	56.33812661	104.6926339	103.2732563	100.984696	94.46603392	86.45921714	90.21569476	Eif2b1	eukaryotic translation initiation factor 2B, subunit 1 (alpha) [Source:MGI Symbol;Acc:MGI:2384802]
ENSMUSG00000029389	15.8124151	15.57108419	16.84424907	18.870066	18.58017892	19.45429845	15.15044903	13.27330346	15.56575494	15.99442678	18.0417003	16.98191176	Ddx55	DEAD (Asp-Glu-Ala-Asp) box polypeptide 55 [Source:MGI Symbol;Acc:MGI:1915098]
ENSMUSG00000029390	163.2979641	189.7224875	167.9285026	179.3698122	180.4083832	178.5629677	74.44683186	69.58749761	71.44106164	91.55361174	81.63150661	81.53417121	Tmed2	transmembrane p24 trafficking protein 2 [Source:MGI Symbol;Acc:MGI:1929269]
ENSMUSG00000029392	54.68037963	48.54203934	55.61494803	51.47215689	49.42831849	42.92124514	17.9292988	20.64320166	20.24665978	21.52053578	22.70829628	20.5819005	Rilpl1	Rab interacting lysosomal protein-like 1 [Source:MGI Symbol;Acc:MGI:1922945]
ENSMUSG00000029394	69.8426411	54.95593071	77.59935187	54.92309229	52.02262055	39.70485965	84.52132122	86.45039753	70.63787355	43.59660533	40.85299408	42.25074367	Cdk2ap1	CDK2 (cyclin-dependent kinase 2)-associated protein 1 [Source:MGI Symbol;Acc:MGI:1202069]
ENSMUSG00000029397	43.75521809	39.96041161	43.83524404	51.14552771	47.78528794	45.71295489	38.41485407	34.8866016	30.55121763	22.39940056	22.86129913	22.9709827	Rchy1	ring finger and CHY zinc finger domain containing 1 [Source:MGI Symbol;Acc:MGI:1915348]
ENSMUSG00000029401	21.07005396	16.5016789	22.75769299	16.27405318	13.69641602	13.53325807	10.55201557	10.29295604	9.041617242	8.067271228	7.748534103	8.33515466	Rilpl2	Rab interacting lysosomal protein-like 2 [Source:MGI Symbol;Acc:MGI:1933112]
ENSMUSG00000029402	14.10770093	10.72219027	14.39521618	12.37517022	11.90732757	10.27993871	10.91569624	10.80786521	7.572134659	8.034100084	8.376843008	7.376529104	Snrnp35	small nuclear ribonucleoprotein 35 (U11/U12) [Source:MGI Symbol;Acc:MGI:1923417]
ENSMUSG00000029403	2.993120332	2.788120728	2.400720768	4.696729352	4.827294959	4.603174365	4.544424474	4.173820267	5.820169118	9.873519065	10.15631383	9.951387178	Cdkl2	cyclin-dependent kinase-like 2 (CDC2-related kinase) [Source:MGI Symbol;Acc:MGI:1858227]
ENSMUSG00000029404	123.830967	90.15725977	132.5751573	109.4005203	95.26257584	74.88663603	98.82573042	112.8399306	89.77096108	64.23364808	66.67673427	73.00614453	Arl6ip4	ADP-ribosylation factor-like 6 interacting protein 4 [Source:MGI Symbol;Acc:MGI:1929500]
ENSMUSG00000029405	78.21597021	96.57350339	63.44557279	88.2760717	102.1214516	126.2915915	237.2294845	215.1096004	277.4176003	300.8033348	299.7987182	299.0431578	G3bp2	GTPase activating protein (SH3 domain) binding protein 2 [Source:MGI Symbol;Acc:MGI:2442040]
ENSMUSG00000029406	4.558004236	5.18199786	5.105786411	3.928763465	4.350234407	5.388720374	4.286724956	4.216626983	4.242403382	6.369706564	7.182683384	7.606024116	Pitpnm2	phosphatidylinositol transfer protein, membrane-associated 2 [Source:MGI Symbol;Acc:MGI:1336192]
ENSMUSG00000029407	60.00233925	71.05213561	46.97675318	65.82813347	75.15764455	85.32467777	34.84450076	33.51088656	41.51846002	56.3468131	56.62870774	55.6804363	Uso1	USO1 vesicle docking factor [Source:MGI Symbol;Acc:MGI:1929095]
ENSMUSG00000029408	1.370334007	1.472809382	1.426320203	0.703325249	0.935840985	0.626255171	0.346630849	0.475128876	0.7440129	0.776296562	0.627365653	0.809615811	Abcb9	ATP-binding cassette, sub-family B (MDR/TAP), member 9 [Source:MGI Symbol;Acc:MGI:1861729]
ENSMUSG00000029409	11.09258314	8.653137864	10.89858165	6.42789562	6.485184961	3.67941188	0.958374738	1.949622972	0.499573616	4.720977164	4.244411439	3.488249078	U90926	cDNA sequence U90926 [Source:MGI Symbol;Acc:MGI:1930915]
ENSMUSG00000029410	0.175536921	0.101069894	0.044928353	0.30714032	0.207483358	0.150416209	1.782637287	1.565048972	1.85847669	2.571098212	2.1004287	1.80844474	Ppef2	protein phosphatase, EF hand calcium-binding domain 2 [Source:MGI Symbol;Acc:MGI:1342304]
ENSMUSG00000029413	16.75615428	18.19026195	18.10943364	19.32178188	19.36264495	18.56985618	4.039803009	5.134662342	4.390346757	7.727029577	9.268101622	8.895247365	Naaa	N-acylethanolamine acid amidase [Source:MGI Symbol;Acc:MGI:1914361]
ENSMUSG00000029414	16.47474285	19.56334558	16.77902562	29.68213292	35.45868101	47.8452856	61.07630415	55.26934732	79.27446516	68.49683199	68.5678074	71.98945842	Kntc1	kinetochore associated 1 [Source:MGI Symbol;Acc:MGI:2673709]
ENSMUSG00000029415	18.88923554	29.44924042	21.36118547	29.04182359	35.37053335	43.43073049	47.81320516	43.69819607	49.41914169	83.63265649	81.77060251	85.23543602	Sdad1	SDA1 domain containing 1 [Source:MGI Symbol;Acc:MGI:2140779]
ENSMUSG00000029416	29.03378548	30.86822575	26.94458022	24.68984074	26.92016368	22.06147667	19.23850701	17.69179601	17.49720212	18.87031361	18.05332801	17.95578522	Slc15a4	solute carrier family 15, member 4 [Source:MGI Symbol;Acc:MGI:2140796]
ENSMUSG00000029417	14.12284723	16.35438	14.75615252	0.27097439	0.416027469	0.281494656	0	0.052301654	0.073710095	0.118061247	0.021973543	0.14036652	Cxcl9	chemokine (C-X-C motif) ligand 9 [Source:MGI Symbol;Acc:MGI:1352449]
ENSMUSG00000029419	2.398569848	2.319533839	2.130028728	3.054483839	3.035236649	3.865860765	0.331264525	0.157533161	0.357691837	0.616376425	0.551537965	0.563714021	Ajm1	apical junction component 1 [Source:MGI Symbol;Acc:MGI:2685842]
ENSMUSG00000029420	0.112443784	0.129484797	0.051164054	0.034976897	0.026253379	0.085646395	0.803097995	0.928263234	1.046580615	1.631603685	1.143976859	1.06293811	Rimbp2	RIMS binding protein 2 [Source:MGI Symbol;Acc:MGI:2443235]
ENSMUSG00000029422	49.62944903	59.21542492	46.69427278	60.40766268	66.72793453	70.07929791	87.79152256	84.41747742	96.43147974	85.82038966	85.58514753	84.37868115	Rsrc2	arginine/serine-rich coiled-coil 2 [Source:MGI Symbol;Acc:MGI:1913489]
ENSMUSG00000029423	0.266430265	0.272718352	0.227307793	0.151939545	0.233273056	0.236757631	0.951451494	0.395905706	0.909268662	0.834104534	1.022635248	0.865762542	Piwil1	piwi-like RNA-mediated gene silencing 1 [Source:MGI Symbol;Acc:MGI:1928897]
ENSMUSG00000029426	65.01571274	76.22322992	62.83269554	66.34986444	76.51568319	85.90766015	30.89394236	27.3154942	33.55276509	29.93508496	30.34751303	30.21403945	Scarb2	scavenger receptor class B, member 2 [Source:MGI Symbol;Acc:MGI:1196458]
ENSMUSG00000029427	37.34652085	39.09964983	35.12884567	46.12502069	43.75933379	44.3499728	78.23332781	76.99976847	85.43818792	71.07937892	73.99858312	76.58358358	Zcchc8	zinc finger, CCHC domain containing 8 [Source:MGI Symbol;Acc:MGI:1917900]
ENSMUSG00000029428	65.00760305	66.46572882	61.528467	69.96491903	71.70031475	76.93538466	13.01523146	14.03668796	12.25358655	19.92592209	17.98582943	19.93985077	Stx2	syntaxin 2 [Source:MGI Symbol;Acc:MGI:108059]
ENSMUSG00000029430	401.248351	360.6826316	411.1105282	411.812727	387.0220475	339.6686984	795.1599746	843.7425402	733.2865828	468.032961	476.2618657	463.2146817	Ran	RAN, member RAS oncogene family [Source:MGI Symbol;Acc:MGI:1333112]
ENSMUSG00000029431	0	0	0	0	0	0	0	0	0	0	0	0	B3gnt4	UDP-GlcNAc:betaGal beta-1,3-N-acetylglucosaminyltransferase 4 [Source:MGI Symbol;Acc:MGI:2680208]
ENSMUSG00000029432	57.69083558	64.22631109	52.77546806	67.5850617	71.39333442	70.68588279	63.35915288	55.74419852	62.22157782	49.15789764	48.16956026	48.7673286	Nipsnap2	nipsnap homolog 2 [Source:MGI Symbol;Acc:MGI:1278343]
ENSMUSG00000029433	0.983508293	1.344915424	0.692249646	1.204604335	1.969791014	3.675372314	0.850614626	1.461457635	1.802211819	2.281786578	2.507181284	2.805492274	Diablo	diablo, IAP-binding mitochondrial protein [Source:MGI Symbol;Acc:MGI:1913843]
ENSMUSG00000029434	39.05885629	48.21484854	40.64079648	46.39957439	45.05610179	50.45212874	22.94543809	21.09764179	25.08128681	38.46204855	36.90223534	36.02724935	Vps33a	VPS33A CORVET/HOPS core subunit [Source:MGI Symbol;Acc:MGI:1924823]
ENSMUSG00000029436	1.570649386	1.724558693	1.745137246	1.340721671	1.701388325	1.78986587	11.06573895	10.21621194	11.29852973	4.356306324	4.408698595	4.49955327	Mmp17	matrix metallopeptidase 17 [Source:MGI Symbol;Acc:MGI:1346076]
ENSMUSG00000029437	0	0	0	0	0	0	0	0	0	0	0	0.174921581	Il31	interleukin 31 [Source:MGI Symbol;Acc:MGI:1923649]

ENSMUSG00000029438	4.009754801	5.112164359	3.508932288	4.721842301	5.025375525	5.417493981	7.815935474	6.752796818	8.388373427	5.832545159	5.753587275	5.829503291	Bcl7a	B cell CLL/lymphoma 7A [Source:MGI Symbol;Acc:MGI:1924295]
ENSMUSG00000029439	81.20118414	68.78106278	80.31705108	76.61377865	70.59929167	73.58654862	58.28633436	58.53059974	59.40754766	51.7943083	55.53601632	54.69945037	Sfswap	splicing factor SWAP [Source:MGI Symbol;Acc:MGI:101760]
ENSMUSG00000029440	40.91548173	33.512284	42.47895555	40.75099983	34.64625588	32.2131557	23.18039185	28.16350652	20.79933613	26.1454712	23.23832992	23.37910228	Psmd9	proteasome (prosome, macropain) 26S subunit, non-ATPase, 9 [Source:MGI Symbol;Acc:MGI:1914401]
ENSMUSG00000029442	0.615643484	0.593981046	0.919884575	0.853998949	0.384602863	0.367535421	0.029709864	0.098899879	0.046460733	0.506028947	0.332407197	0.486614793	Wdr66	WD repeat domain 66 [Source:MGI Symbol;Acc:MGI:1918495]
ENSMUSG00000029445	0	0.415219405	0.061525489	0.056080276	0	0	0.107304062	0.059533307	0	0	0.300141415	0	Hpd	4-hydroxyphenylpyruvic acid dioxygenase [Source:MGI Symbol;Acc:MGI:96213]
ENSMUSG00000029446	41.29695914	33.89705294	39.88634324	54.89796416	51.48472472	44.74280282	38.1056802	41.89683421	40.17002421	26.79443937	30.70017898	31.38100205	Psph	phosphoserine phosphatase [Source:MGI Symbol;Acc:MGI:97788]
ENSMUSG00000029447	245.1039935	252.1553571	220.8017694	277.2560693	283.5920584	293.7597801	616.6801727	623.3485417	626.4129559	566.6152759	567.7597347	555.3055507	Cct6a	chaperonin containing Tcp1, subunit 6a (zeta) [Source:MGI Symbol;Acc:MGI:107943]
ENSMUSG00000029449	0.961010895	1.106653447	1.063220736	0.607509391	0.781700029	0.814628035	2.29714185	2.50288659	2.452577723	1.358725038	2.206298886	1.978075748	Rhof	ras homolog family member F (in filopodia) [Source:MGI Symbol;Acc:MGI:1345629]
ENSMUSG00000029451	0	0	0	0	0	0	0	0	0	0	0	0	Gm13103	predicted gene 13103 [Source:MGI Symbol;Acc:MGI:3650203]
ENSMUSG00000029452	1.305023461	1.760424732	0.992511703	1.530981824	1.527837449	1.505219319	1.597844883	1.846873817	0.902320572	2.501384296	1.831192074	1.982646691	Tmem116	transmembrane protein 116 [Source:MGI Symbol;Acc:MGI:1924712]
ENSMUSG00000029454	1.421646074	2.128228094	0.727735411	2.034207027	1.941766978	1.804736819	2.369198048	2.769741205	3.21981064	1.907443267	1.617281822	1.385887505	Mapkapk5	MAP kinase-activated protein kinase 5 [Source:MGI Symbol;Acc:MGI:1333110]
ENSMUSG00000029455	130.0242669	121.5855023	130.0422426	96.33914428	90.8440608	90.57547521	278.5426146	293.5138371	272.6837502	156.3311216	159.469754	159.3539421	Aldh2	aldehyde dehydrogenase 2, mitochondrial [Source:MGI Symbol;Acc:MGI:99600]
ENSMUSG00000029456	4.297870554	4.184860749	3.601649457	4.552688964	3.905385002	5.068408128	4.755560415	3.73161104	5.498456592	3.963087937	4.79307975	4.176531019	Acad10	acyl-Coenzyme A dehydrogenase family, member 10 [Source:MGI Symbol;Acc:MGI:1919235]
ENSMUSG00000029458	34.8551016	38.25113706	31.8981168	41.54025415	40.950078	42.78115881	16.90072661	15.9261409	17.74548354	18.26214731	18.21768863	16.94130616	Brap	BRCA1 associated protein [Source:MGI Symbol;Acc:MGI:1919649]
ENSMUSG00000029461	32.19313226	40.2927652	27.29237137	38.18230711	46.19468166	55.56686226	28.28730163	26.89303482	35.5345199	41.1121459	41.09172441	40.78487488	Fam168a	family with sequence similarity 168, member A [Source:MGI Symbol;Acc:MGI:2442372]
ENSMUSG00000029462	134.5221469	123.6060652	124.4668316	142.5565285	140.7357874	128.3444815	81.23639963	80.29108137	81.03045287	81.29503121	79.99995379	76.74128201	Vps29	VPS29 retromer complex component [Source:MGI Symbol;Acc:MGI:1928344]
ENSMUSG00000029463	13.23715007	12.63726839	12.09215225	14.84058655	15.86199642	13.69519594	5.092459041	5.619978479	5.049424724	5.157609326	3.741676473	4.588036803	Fam216a	family with sequence similarity 216, member A [Source:MGI Symbol;Acc:MGI:1916198]
ENSMUSG00000029464	28.43309715	28.65514157	26.21088473	31.89205793	29.20314911	28.15710577	76.08206038	79.68149658	79.65290006	63.49266	65.6919338	61.42260273	Gpn3	GPN-loop GTPase 3 [Source:MGI Symbol;Acc:MGI:1289326]
ENSMUSG00000029465	353.0141393	284.6151897	373.9820401	314.9380471	283.8323651	243.3986707	194.5994426	208.0022135	181.7615338	167.7321723	169.9673545	168.4247734	Arpc3	actin related protein 2/3 complex, subunit 3 [Source:MGI Symbol;Acc:MGI:1928375]
ENSMUSG00000029466	42.35616771	42.66456619	43.53601289	47.61503371	47.90769896	43.43655079	51.34128097	48.16815937	45.56931917	42.28951216	42.2327332	43.75069868	Anapc7	anaphase promoting complex subunit 7 [Source:MGI Symbol;Acc:MGI:1929711]
ENSMUSG00000029467	93.65760793	106.3767666	72.47029837	99.8575814	112.1213098	132.3260361	83.56566767	75.44678511	96.74765915	127.6166831	128.6904623	124.7615869	Atp2a2	ATPase, Ca++ transporting, cardiac muscle, slow twitch 2 [Source:MGI Symbol;Acc:MGI:88110]
ENSMUSG00000029468	3.794564523	3.965040172	3.45319762	3.278727096	3.986797808	3.871509242	1.714762131	0.904958117	2.245543063	6.474023739	7.038591207	7.037046646	P2rx7	purinergic receptor P2X, ligand-gated ion channel, 7 [Source:MGI Symbol;Acc:MGI:1339957]
ENSMUSG00000029469	28.87964441	29.69620167	22.75226109	33.63659893	34.89027443	37.23439031	16.46350889	13.43993338	16.62225985	14.11984565	13.41240561	13.2804368	Ift81	intraflagellar transport 81 [Source:MGI Symbol;Acc:MGI:1098597]
ENSMUSG00000029470	54.14688393	59.46621101	58.60549529	47.91446533	43.99987118	42.41079572	3.923107098	3.009227082	4.007660125	12.82052889	13.47735753	12.5977108	P2rx4	purinergic receptor P2X, ligand-gated ion channel 4 [Source:MGI Symbol;Acc:MGI:1338859]
ENSMUSG00000029471	18.60571352	22.42256202	21.4805864	25.71231475	26.4298373	28.14033557	14.68803244	14.58252684	14.65162458	14.49753119	15.19167783	14.16702341	Camkk2	calcium/calmodulin-dependent protein kinase kinase 2, beta [Source:MGI Symbol;Acc:MGI:2444812]
ENSMUSG00000029472	252.5726259	237.3767493	256.9206974	282.9924242	268.4652368	260.4823953	261.8053191	260.8991231	272.1882309	208.9139394	217.8728604	218.6590129	Anapc5	anaphase-promoting complex subunit 5 [Source:MGI Symbol;Acc:MGI:1929722]
ENSMUSG00000029474	14.05550359	13.99783945	12.26475159	16.35031232	15.19265645	13.66700779	23.80904283	23.1601601	25.10030633	22.56408167	20.92490395	22.46505137	Rnf34	ring finger protein 34 [Source:MGI Symbol;Acc:MGI:2153340]
ENSMUSG00000029475	10.53216313	17.45066116	7.202459158	16.69880851	19.25221162	25.26590374	35.40228234	30.57148488	46.02606862	39.36601884	39.10235883	40.94212533	Kdm2b	lysine (K)-specific demethylase 2B [Source:MGI Symbol;Acc:MGI:1354737]
ENSMUSG00000029477	0.521908882	0.400669938	0.356217657	0.324691195	0.426493569	0.706717426	0.414176324	0.402130639	0.539745977	0.414964597	0.386165773	0.668096713	Morn3	MORN repeat containing 3 [Source:MGI Symbol;Acc:MGI:1922140]
ENSMUSG00000029478	36.19669406	44.04023523	39.2852142	42.72565212	41.96068349	51.80409714	19.37880422	16.43206437	21.96243793	32.26261926	36.51338301	35.1667213	Ncor2	nuclear receptor co-repressor 2 [Source:MGI Symbol;Acc:MGI:1337080]
ENSMUSG00000029480	21.12212525	26.27990713	19.80477834	24.88247108	23.64785061	26.20328746	31.99681952	33.43254519	38.92991643	36.36013834	38.6891887	38.59624496	Dhx37	DEAH (Asp-Glu-Ala-His) box polypeptide 37 [Source:MGI Symbol;Acc:MGI:3028576]
ENSMUSG00000029482	102.8154393	118.9188375	103.1492455	117.4078299	116.3254975	120.171409	52.29811712	49.65537346	53.96096205	49.11154539	51.0233384	48.34627916	Aacs	acetoacetyl-CoA synthetase [Source:MGI Symbol;Acc:MGI:1926144]
ENSMUSG00000029484	387.0035791	373.6301313	395.4867115	451.7856055	425.8400469	395.6375018	13.99366012	13.77451013	16.18908123	84.75493417	83.29212288	81.95661889	Anxa3	annexin A3 [Source:MGI Symbol;Acc:MGI:1201378]
ENSMUSG00000029486	11.21824946	9.453755089	9.751552222	11.8090165	11.67537385	11.05518616	20.4627262	20.26450174	19.20839488	17.67525371	16.96456365	17.33801078	Mrpl1	mitochondrial ribosomal protein L1 [Source:MGI Symbol;Acc:MGI:2137202]
ENSMUSG00000029490	2.495143081	2.345539201	2.102691232	2.312586889	2.906885953	3.193366113	0.530382601	0.235409021	0.758327129	1.184245119	0.664060413	0.676915468	Mfsd7a	major facilitator superfamily domain containing 7A [Source:MGI Symbol;Acc:MGI:2442629]
ENSMUSG00000029491	0.222780632	0.348165975	0.114040589	0.237594543	0.217347684	0.193929116	0	0.094583982	0	0.042701146	0.013245886	0.028204811	Pde6b	phosphodiesterase 6B, cGMP, rod receptor, beta polypeptide [Source:MGI Symbol;Acc:MGI:97525]
ENSMUSG00000029499	3.852322886	2.781991014	4.010827029	4.508888255	3.978880826	3.13317648	32.70217471	31.8885325	22.36432134	18.63074548	18.75086804	17.9380887	Pxmp2	peroxisomal membrane protein 2 [Source:MGI Symbol;Acc:MGI:107487]
ENSMUSG00000029500	42.25144967	44.25528596	33.90929417	49.61024124	51.19527398	51.02291098	63.70680088	58.98339648	71.41948014	54.61873934	57.18841969	59.48044501	Pgam5	phosphoglycerate mutase family member 5 [Source:MGI Symbol;Acc:MGI:1919792]
ENSMUSG00000029501	19.72394054	22.46644112	16.98173514	21.26192336	24.40477498	27.28448873	16.69379505	16.06806492	19.12731525	25.00622851	25.57620411	24.34159456	Ankle2	ankyrin repeat and LEM domain containing 2 [Source:MGI Symbol;Acc:MGI:1261856]
ENSMUSG00000029502	20.8232755	26.3058499	20.6113083	25.26445102	29.47890345	33.66713263	10.57109488	10.0186299	12.38502958	20.83594287	21.08925515	20.50455459	Golga3	golgi autoantigen, golgin subfamily a, 3 [Source:MGI Symbol;Acc:MGI:96958]
ENSMUSG00000029503	0.023971302	0.303646057	0.122708218	0.20132663	0.151114156	0.365170146	2.140103291	2.469686998	2.365019533	1.114971728	1.157314082	1.465833105	P2rx2	purinergic receptor P2X, ligand-gated ion channel, 2 [Source:MGI Symbol;Acc:MGI:2665170]
ENSMUSG00000029504	24.56569112	26.44297216	21.20566772	28.13044507	25.68065648	27.84383129	25.70162873	25.09917393	26.90981671	34.43515043	36.12475693	36.00226961	Ddx51	DEAD (Asp-Glu-Ala-Asp) box polypeptide 51 [Source:MGI Symbol;Acc:MGI:1916913]
ENSMUSG00000029505	20.92678646	25.41295353	19.23456056	27.49303042	31.81584898	41.15748341	29.13274868	22.83919712	34.9217872	48.16448754	55.41374398	51.98334148	Ep400	E1A binding protein p400 [Source:MGI Symbol;Acc:MGI:1276124]
ENSMUSG00000029507	25.93277795	25.82939649	28.2934542	25.69942532	26.33494273	22.15122381	43.57891212	40.46112952	34.904675	26.24310179	27.87243036	29.1336531	Pus1	pseudouridine synthase 1 [Source:MGI Symbol;Acc:MGI:1929237]
ENSMUSG00000029510	0.136219985	0.313728631	0.052297883	0.174787596	0.357802282	0.402055655	0.805693417	0.640990191	1.489758703	0.852918922	0.751204749	0.663968265	Gpc2	glypican 2 (cerebroglycan) [Source:MGI Symbol;Acc:MGI:1919201]
ENSMUSG00000029512	37.58772298	45.19435388	33.21750246	40.2153953	38.30681717	43.63973295	75.31917803	73.88042069	90.6278024	114.0098433	118.0548517	122.4251466	Ulk1	unc-51 like kinase 1 [Source:MGI Symbol;Acc:MGI:1270126]
ENSMUSG00000029513	65.19840394	63.42338	60.07887836	77.85950559	75.43330833	70.66703116	45.36611338	42.30107934	43.71078823	37.13172375	40.79712674	34.28329717	Prkab1	protein kinase, AMP-activated, beta 1 non-catalytic subunit [Source:MGI Symbol;Acc:MGI:1336167]
ENSMUSG00000029516	7.241878617	8.732022405	6.339055971	10.37245149	13.16844519	16.48569596	15.37811749	15.76683495	19.49778027	21.44590963	22.04639372	21.93699588	Cit	citron [Source:MGI Symbol;Acc:MGI:105313]
ENSMUSG00000029517	0	0.04220572	0	0.171011405	0	0	0	0	0	0	0	0	Ankrd7	ankyrin repeat domain 7 [Source:MGI Symbol;Acc:MGI:1922446]
ENSMUSG00000029518	57.96230531	54.94159181	55.71108662	62.59530538	60.4688125	56.80483978	49.98192582	45.08026788	47.39043815	42.89995195	44.93665043	41.96029607	Rab35	RAB35, member RAS oncogene family [Source:MGI Symbol;Acc:MGI:1924657]
ENSMUSG00000029521	22.51104286	19.51370886	21.4733459	21.12320987	20.4659279	25.27648889	47.46236796	49.003267	55.43482942	60.74114384	61.78065297	60.21759852	Chek2	checkpoint kinase 2 [Source:MGI Symbol;Acc:MGI:1355321]
ENSMUSG00000029522	4.107731535	4.37549422	5.572241672	4.216594331	4.423725662	2.815906308	39.79008751	49.34018171	41.29172663	69.90296518	72.40308719	67.61992407	Pla2g1b	phospholipase A2, group IB, pancreas [Source:MGI Symbol;Acc:MGI:101842]
ENSMUSG00000029524	20.55396902	20.09272856	21.37768267	20.97285167	20.04965306	19.9507571	13.64402224	13.52044932	14.1484182	10.23421708	8.945715338	9.162036929	Sirt4	sirtuin 4 [Source:MGI Symbol;Acc:MGI:1922637]
ENSMUSG00000029526	0.246581592	0.04732523	0.50489685	0.115052945	0.129536729	0.250422316	0	0	0	0	0	0	1700123K08	RIKEN cDNA 1700123K08 gene [Source:MGI Symbol;Acc:MGI:1923908]
ENSMUSG00000029528	114.6754022	119.1252938	118.2068714	165.0893511	165.010362	162.0607041	60.82351895	57.63369824	62.47250459	48.25027868	50.12497105	49.47347166	Pxn	paxillin [Source:MGI Symbol;Acc:MGI:108295]
ENSMUSG00000029530	0	0.065317052	0.046456367	0	0.047675527	0.043203296	0	0	0.010558701	0.030441313	0.009442888	0.020106989	Ccr9	chemokine (C-C motif) receptor 9 [Source:MGI Symbol;Acc:MGI:1341902]
ENSMUSG00000029534	9.511560467	8.501653733	9.4364557	7.544627151	8.732343597	6.020070106	0.930043835	1.144167491	1.285788549	3.605714744	3.5062836	3.572451071	St7	suppression of tumorigenicity 7 [Source:MGI Symbol;Acc:MGI:1927450]
ENSMUSG00000029535	37.68947509	28.96460656	35.79245329	33.43662768	28.29675073	23.22497234	33.68361285	35.22117379	32.83441467	19.95148257	21.29918974	19.59218173	Triap1	TP53 regulated inhibitor of apoptosis 1 [Source:MGI Symbol;Acc:MGI:1916326]
ENSMUSG00000029536	12.02020083	13.37313026	11.05596263	14.59110797	15.4971197	16.46908216	8.016455357	6.262228043	7.822622251	8.631413716	8.949235999	8.551786639	Gatc	glutamyl-tRNA(Gln) amidotransferase, subunit C [Source:MGI Symbol;Acc:MGI:1923776]
ENSMUSG00000029538	137.2419626	111.4410351	137.7641341	115.3125517	99.97993481	87.78755528	121.1545398	129.2827908	102.2867066	83.32010302	76.84884546	81.57368622	Srsf9	serine and arginine-rich splicing factor 9 [Source:MGI Symbol;Acc:MGI:104896]
ENSMUSG00000029541	0	0	0	0	0	0	0	0.136913858	0.042879177	0.041207673	0.038347832	0	Cyp2w1	cytochrome P450, family 2, subfamily w, polypeptide 1 [Source:MGI Symbol;Acc:MGI:3616076]
ENSMUSG00000029544	2.940377044	1.072790595	1.609486814	2.037558077	2.355237153	2.43920316	48.3174653	54.10425594	42.81303072	18.09819799	18.73237128	19.91786361	Cabp1	calcium binding protein 1 [Source:MGI Symbol;Acc:MGI:1352750]
ENSMUSG00000029545	76.52148269	62.28858668	80.84352225	65.51856912	56.65301093	47.73944489	102.0659648	103.0348763	97.53294945	47.92783825	52.31297586	51.39521618	Acads	acyl-Coenzyme A dehydrogenase, short chain [Source:MGI Symbol;Acc:MGI:87868]
ENSMUSG00000029546	0.256260621	0.107308101	0.11925352	0.173918691	0.244766207	0.070977847	0.020798513	0	0	0.020838098	0	0.020645861	Uncx	UNC homeobox [Source:MGI Symbol;Acc:MGI:108013]
ENSMUSG00000029547	54.38060424	54.16079627	54.34013304	58.2207802	57.77059469	61.69227414	51.45027207	51.10236614	57.36627806	59.04979915	65.56469304	64.19652743	Ints1	integrator complex subunit 1 [Source:MGI Symbol;Acc:MGI:1915760]
ENSMUSG00000029550	36.18358302	38.78615403	34.60385205	41.67327486	41.95966794	44.02829212	30.04913983	26.46871069	29.76774565	29.85298105	30.94436581	29.64033239	Sppl3	signal peptide peptidase 3 [Source:MGI Symbol;Acc:MGI:1891433]
ENSMUSG00000029551	3.824040367	2.849374685	6.711470531	4.108309532	3.578851191	2.765843414	5.10667963	3.56541818	2.243231041	1.868347882	2.193415825	2.192855609	Psmg3	proteasome (prosome, macropain) assembly chaperone 3 [Source:MGI Symbol;Acc:MGI:1913756]

ENSMUSG00000029552	78.01845241	83.23729334	66.71143538	91.70831484	91.46955591	94.47298218	7.328899315	6.167862997	7.402543864	34.46370428	30.82949094	33.63556509	Tes	testis derived transcript [Source:MGI Symbol;Acc:MGI:105081]
ENSMUSG00000029553	3.565749764	3.922286951	3.841290834	0.769794566	1.28607461	0.709394386	0.190054937	0.184527479	0.198140515	1.951770713	2.569423349	3.466627699	Tfec	transcription factor EC [Source:MGI Symbol;Acc:MGI:1333760]
ENSMUSG00000029554	34.56179566	33.39681009	35.31942527	41.30324702	42.38694753	36.86475743	39.40201992	36.64821414	36.19714062	33.03409155	32.24044738	34.1572274	Mad1l1	MAD1 mitotic arrest deficient 1-like 1 [Source:MGI Symbol;Acc:MGI:1341857]
ENSMUSG00000029556	0.040383377	0.116258835	0.062016307	0.075370207	0.063643808	0.076898218	1.315947605	1.040142694	0.883298978	1.282330179	0.840377182	1.019979149	Hnf1a	HNF1 homeobox A [Source:MGI Symbol;Acc:MGI:98504]
ENSMUSG00000029557	9.989288581	10.02665181	11.29544877	9.934020504	7.95766657	8.335457778	13.01792352	13.05656596	11.04612772	8.695133082	9.407204301	9.196293023	Mrm2	mitochondrial rRNA methyltransferase 2 [Source:MGI Symbol;Acc:MGI:1915267]
ENSMUSG00000029559	72.24688404	58.23247836	74.00778033	63.74842624	54.3360931	50.44077843	54.87836296	53.01843533	47.33428289	38.19472472	38.68222552	42.34568273	2210016L21	RIKEN cDNA 2210016L21 gene [Source:MGI Symbol;Acc:MGI:1919607]
ENSMUSG00000029560	30.93499525	27.91350327	34.19061485	28.79394536	25.94056643	22.95793943	29.79159347	29.0463956	27.11815241	19.6463789	18.08465853	18.2925373	Snx8	sorting nexin 8 [Source:MGI Symbol;Acc:MGI:2443816]
ENSMUSG00000029561	166.2817768	172.9755681	158.2961882	187.7961226	194.0076854	190.6223911	3.037319034	3.273973242	4.071261638	38.8647311	38.21725343	39.65477513	Oasl2	2'-5' oligoadenylate synthetase-like 2 [Source:MGI Symbol;Acc:MGI:1344390]
ENSMUSG00000029563	0.471662547	0.296260183	0.395087446	0.240080585	0.360405129	0.228617982	0	0	0	0.038353728	0.017845977	0.22799943	Foxp2	forkhead box P2 [Source:MGI Symbol;Acc:MGI:2148705]
ENSMUSG00000029564	0.060603504	0	0	0.056554194	0	0	0	0	0.112814511	0	0	0.080562475	4930519G04	RIKEN cDNA 4930519G04 gene [Source:MGI Symbol;Acc:MGI:1914843]
ENSMUSG00000029569	18.48165102	24.41236109	15.11367095	21.73200197	26.39155818	29.41817012	13.79256729	10.486412	15.17820237	21.009721	25.19564778	22.18503431	Tmem168	transmembrane protein 168 [Source:MGI Symbol;Acc:MGI:1921794]
ENSMUSG00000029570	7.773097952	9.700169725	8.457499282	9.014046045	7.996029096	7.679475735	1.509880886	0.934353427	1.816288051	3.170966239	3.167478784	2.709367378	Lfng	LFNG O-fucosylpeptide 3-beta-N-acetylglucosaminyltransferase [Source:MGI Symbol;Acc:MGI:1095413]
ENSMUSG00000029571	77.35273957	101.9043155	68.06801255	92.72081714	110.2599825	147.2954553	13.46474847	11.74108909	16.20309184	32.3181397	31.78405935	35.27051108	Tmem106b	transmembrane protein 106B [Source:MGI Symbol;Acc:MGI:1919150]
ENSMUSG00000029575	15.49234565	15.25294743	15.32601054	15.87122894	14.68515502	14.12844848	15.11404095	15.11444751	15.95157462	10.79626015	10.5254206	12.0626857	Mmab	methylmalonic aciduria (cobalamin deficiency) cblB type homolog (human) [Source:MGI Symbol;Acc:MGI:1924947]
ENSMUSG00000029576	4.439169743	3.159737593	4.008003267	3.457569984	4.113185747	3.860465469	2.23124457	2.822101208	3.399785812	3.017131401	3.506038583	3.608841102	Radil	Ras association and DIL domains [Source:MGI Symbol;Acc:MGI:2443088]
ENSMUSG00000029577	34.68322953	39.7782802	35.53710649	37.30497209	38.10268289	41.94605375	25.0765367	22.5127728	26.92533259	28.14268156	28.05441565	26.28229487	Ube3b	ubiquitin protein ligase E3B [Source:MGI Symbol;Acc:MGI:1891295]
ENSMUSG00000029578	44.21737272	44.61738176	42.97552145	45.3593485	47.41828928	47.27446406	45.79028833	45.16256127	47.28438612	50.2129579	48.23795982	49.72271419	Wipi2	WD repeat domain, phosphoinositide interacting 2 [Source:MGI Symbol;Acc:MGI:1923831]
ENSMUSG00000029580	3959.013834	3207.652376	4122.304975	2920.065417	2719.053275	2295.341256	2207.187711	2329.87239	2222.179291	2026.681407	1917.797425	2038.23858	Actb	actin, beta [Source:MGI Symbol;Acc:MGI:87904]
ENSMUSG00000029581	553.3083172	516.0267235	583.023013	432.6666645	380.6074168	342.3768546	394.7250061	404.9848035	402.2247658	381.0875713	382.6403216	386.6311358	Fscn1	fascin actin-bundling protein 1 [Source:MGI Symbol;Acc:MGI:1352745]
ENSMUSG00000029586	0.031731923	0	0.06497382	0	0.066678936	0.048339263	0.056659077	0.031434991	0.029534773	0	0.05282725	0.028121611	Spdye4b	speedy/RINGO cell cycle regulator family, member E4B [Source:MGI Symbol;Acc:MGI:3612701]
ENSMUSG00000029587	8.16517115	10.3558003	7.210378089	10.19874771	11.70375115	11.32718027	3.10625491	2.529476453	2.898895219	6.927081003	5.500407027	6.502611396	Zfp12	zinc finger protein 12 [Source:MGI Symbol;Acc:MGI:99157]
ENSMUSG00000029591	17.35100089	14.95671937	18.12114579	15.74965498	15.63694441	12.66527912	71.17653727	73.44685461	67.26679621	44.64247316	47.04337005	46.90391463	Ung	uracil DNA glycosylase [Source:MGI Symbol;Acc:MGI:109352]
ENSMUSG00000029592	36.82742473	36.85468376	37.63310725	39.63910182	40.93723623	40.46240178	18.69994498	18.22434161	19.71995852	19.04368552	20.847773	21.73789283	Usp30	ubiquitin specific peptidase 30 [Source:MGI Symbol;Acc:MGI:2140991]
ENSMUSG00000029594	19.70747255	19.26731341	22.63234873	23.26464736	21.69488813	17.78632454	34.75500559	31.04437179	35.32917389	34.35940541	34.7299837	36.49133293	Rbm19	RNA binding motif protein 19 [Source:MGI Symbol;Acc:MGI:1921361]
ENSMUSG00000029595	0.128013352	0.055280233	0.081911956	0.074662473	0.084061581	0.07312909	0.128573331	0.253630927	0.20851176	0.128818035	0.226436178	0.226897164	Lhx5	LIM homeobox protein 5 [Source:MGI Symbol;Acc:MGI:107792]
ENSMUSG00000029596	8.474061656	6.537125388	11.07623499	7.754304515	6.094046737	6.36054129	0.293803855	0.285259019	0.114863723	0.588726063	0.924527379	0.492837114	Sdsl	serine dehydratase-like [Source:MGI Symbol;Acc:MGI:2182607]
ENSMUSG00000029597	1.454342829	2.136750674	0.718801285	0.889179579	1.369949041	0.954953981	0	0	0.186709331	0.04485777	0.250467661	0.222219724	Sds	serine dehydratase [Source:MGI Symbol;Acc:MGI:98270]
ENSMUSG00000029598	67.59648773	67.26949687	71.05367315	64.32046464	60.28214994	56.21542158	22.97501619	21.48064432	22.31826806	20.11335579	20.74267898	21.01699877	Plbd2	phospholipase B domain containing 2 [Source:MGI Symbol;Acc:MGI:1919022]
ENSMUSG00000029599	33.17608961	39.83900693	33.85907359	39.50714419	42.44306105	40.27841409	57.53725829	53.72400604	62.77321738	51.90067417	52.5938747	50.05615094	Ddx54	DEAD (Asp-Glu-Ala-Asp) box polypeptide 54 [Source:MGI Symbol;Acc:MGI:1919240]
ENSMUSG00000029600	19.53493347	18.77926642	24.75736461	21.40180369	21.02186803	15.4037897	27.92759891	30.22172602	24.9506214	21.1683815	20.37980215	20.3629711	Rita1	RBPJ interacting and tubulin associated 1 [Source:MGI Symbol;Acc:MGI:1922021]
ENSMUSG00000029601	1.006204638	0.853776269	0.759054157	1.260201458	0.612051079	0.584890214	0.898316588	1.023020345	1.0104709	0.59212256	0.308576158	0.469328058	Iqcd	IQ motif containing D [Source:MGI Symbol;Acc:MGI:1922982]
ENSMUSG00000029602	0.68823557	0.570627819	0.648241686	0.25690005	0.433861021	0.125811979	5.99694459	5.781624896	5.380884082	7.288827925	6.897555674	7.514353579	Rasal1	RAS protein activator like 1 (GAP1 like) [Source:MGI Symbol;Acc:MGI:1330842]
ENSMUSG00000029603	0.744770029	1.30029438	0.836279457	0.425972127	0.378629132	0.658774284	11.43222625	10.66241193	11.27011299	8.337984898	9.279190521	8.729475669	Dtx1	deltex 1, E3 ubiquitin ligase [Source:MGI Symbol;Acc:MGI:1352744]
ENSMUSG00000029605	15.02485168	14.91057106	17.85226037	17.95364871	18.35284464	15.53800269	2.590071779	3.479043638	3.268738414	5.62705541	6.48211728	4.65497488	Oas1b	2'-5' oligoadenylate synthetase 1B [Source:MGI Symbol;Acc:MGI:97430]
ENSMUSG00000029607	1.137772515	0.56576971	0.476526745	0.844574354	0.842222277	1.102971848	0.184686798	0.10246598	0.240679997	0.624504262	0.710310631	0.664575863	Ankrd61	ankyrin repeat domain 61 [Source:MGI Symbol;Acc:MGI:1913979]
ENSMUSG00000029608	0.237071558	0.085312527	0.197203599	0.165923462	0.202378836	0.146715657	0	0	0.04137309	0.238561775	0.209671792	0.091918159	Rph3a	rabphilin 3A [Source:MGI Symbol;Acc:MGI:102788]
ENSMUSG00000029610	30.55022636	36.06103637	32.15814264	40.51039764	41.18980116	36.70690311	44.04903267	45.08867446	42.76359401	45.50132913	44.45272212	42.66648336	Aimp2	aminoacyl tRNA synthetase complex-interacting multifunctional protein 2 [Source:MGI Symbol;Acc:MGI:2385237]
ENSMUSG00000029613	24.30205926	25.35040008	25.2542614	25.71042616	26.03821374	25.42256471	22.29709358	21.8271767	21.83197715	28.42743897	28.53507104	29.34086768	Eif2ak1	eukaryotic translation initiation factor 2 alpha kinase 1 [Source:MGI Symbol;Acc:MGI:1353448]
ENSMUSG00000029614	2858.26737	2173.192782	3086.794956	2666.996332	2228.349552	1801.237309	3820.491243	4382.779748	3630.443943	2371.037742	2382.915467	2370.558624	Rpl6	ribosomal protein L6 [Source:MGI Symbol;Acc:MGI:108057]
ENSMUSG00000029616	59.06539273	40.46751942	76.73054887	43.76830299	35.01731825	25.95249239	42.4956542	46.67814291	31.90087258	25.54314801	25.13759104	24.92301837	Erp29	endoplasmic reticulum protein 29 [Source:MGI Symbol;Acc:MGI:1914647]
ENSMUSG00000029617	76.02280198	74.06348651	68.45041954	75.85268323	74.12699972	73.08473914	84.38825838	82.28341775	83.43843471	81.15195501	78.21709568	78.08754329	Ccz1	CCZ1 vacuolar protein trafficking and biogenesis associated [Source:MGI Symbol;Acc:MGI:2141070]
ENSMUSG00000029618	0	0.456677563	0.101502881	0.092519534	0.104166632	0	0	0.098216239	0.092279151	0.1773639	0.165054726	0.263591507	Ocm	oncomodulin [Source:MGI Symbol;Acc:MGI:97401]
ENSMUSG00000029620	0	0	0	0.035787413	0	0	0	0	0	0	0	0.135946184	1700018F24	RIKEN cDNA 1700018F24 gene [Source:MGI Symbol;Acc:MGI:1916646]
ENSMUSG00000029621	236.2402783	192.9234366	262.9871969	222.657511	198.7221564	158.9363796	122.3492449	124.1649693	118.8986101	82.68705033	82.3788136	83.81038407	Arpc1a	actin related protein 2/3 complex, subunit 1A [Source:MGI Symbol;Acc:MGI:1928896]
ENSMUSG00000029622	898.5439626	824.777956	994.930603	1009.096133	875.3589874	756.2894845	137.8861654	138.999256	129.1814108	195.8005275	206.1890399	203.3094264	Arpc1b	actin related protein 2/3 complex, subunit 1B [Source:MGI Symbol;Acc:MGI:1343142]
ENSMUSG00000029623	175.1497985	160.2778788	205.6723128	188.9439389	170.6133944	145.9847845	235.5502607	238.7845363	196.3532915	143.3115638	144.2473732	143.7190468	Pdap1	PDGFA associated protein 1 [Source:MGI Symbol;Acc:MGI:2448536]
ENSMUSG00000029624	10.57783659	14.54042333	9.22999528	13.64740229	13.61631493	15.95782671	10.74701275	9.489325619	11.41877594	14.29553037	12.98361736	14.54951899	Ptcd1	pentatricopeptide repeat domain 1 [Source:MGI Symbol;Acc:MGI:1919049]
ENSMUSG00000029625	37.54163368	34.22211817	44.07912771	41.86839147	38.0643559	31.45426142	62.39191064	61.61112459	53.78168548	45.15702217	46.24070074	44.3449154	Cpsf4	cleavage and polyadenylation specific factor 4 [Source:MGI Symbol;Acc:MGI:1861602]
ENSMUSG00000029627	5.11204124	5.650123871	6.68016212	5.106084047	5.721888651	4.304647487	3.027319843	3.231929945	2.821373062	3.837307692	3.848977663	3.665312033	Zkscan14	zinc finger with KRAB and SCAN domains 14 [Source:MGI Symbol;Acc:MGI:1914485]
ENSMUSG00000029629	19.04692837	18.51111357	16.66790179	21.94312559	20.75812011	22.380869	18.84029985	20.5166982	22.26885923	19.79572206	20.56903101	19.712492	Phf14	PHD finger protein 14 [Source:MGI Symbol;Acc:MGI:1923539]
ENSMUSG00000029630	0	0	0	0	0	0	0	0	0	0	0	0	Cyp3a25	cytochrome P450, family 3, subfamily a, polypeptide 25 [Source:MGI Symbol;Acc:MGI:1930638]
ENSMUSG00000029632	257.2207711	208.279904	308.7826497	237.310854	209.2845652	157.7185126	319.8612218	347.9990342	247.6225141	212.2301742	191.2869722	200.5285556	Ndufa4	Ndufa4, mitochondrial complex associated [Source:MGI Symbol;Acc:MGI:107686]
ENSMUSG00000029633	0	0.200443024	0.071281958	0.324866326	0.146305237	0.63638875	0.062159958	0.034486932	0.129608905	0.186834788	0.347736655	0.246814917	Gm5578	predicted pseudogene 5578 [Source:MGI Symbol;Acc:MGI:3645010]
ENSMUSG00000029634	40.2463571	52.87770029	33.29498383	53.21028984	61.89538124	67.60541859	15.16820038	13.19180346	18.15159284	38.29468092	34.22051756	34.85342701	Rnf6	ring finger protein (C3H2C3 type) 6 [Source:MGI Symbol;Acc:MGI:1921382]
ENSMUSG00000029635	30.121974	31.38682119	28.07108433	37.00492927	40.48888597	41.98542464	38.36041639	39.32370362	43.22735533	44.68749237	45.62971808	46.94111933	Cdk8	cyclin-dependent kinase 8 [Source:MGI Symbol;Acc:MGI:1196224]
ENSMUSG00000029636	2.193491349	2.024194598	1.584281344	1.878689256	2.14676372	2.35734925	19.93172945	16.59490928	23.17089528	19.31117586	19.72179212	19.83869692	Wasf3	WAS protein family, member 3 [Source:MGI Symbol;Acc:MGI:2658986]
ENSMUSG00000029638	0.095280088	0.065831952	0	0.106696575	0.300320981	0.232233981	0.017012779	0	0.124155917	0.136361263	0.301382035	0.10132747	Glcci1	glucocorticoid induced transcript 1 [Source:MGI Symbol;Acc:MGI:2179717]
ENSMUSG00000029640	15.34212476	20.23948794	12.33703728	16.05864841	19.40807929	21.9388936	19.12870807	15.94101886	21.62490046	16.60187894	19.81671389	18.41527569	Usp12	ubiquitin specific peptidase 12 [Source:MGI Symbol;Acc:MGI:1270128]
ENSMUSG00000029641	7.119447865	5.206289685	6.065164882	2.81268323	2.948368588	2.533257634	13.64005688	12.35531817	13.34971717	5.996313601	5.320622807	4.651477504	Rasl11a	RAS-like, family 11, member A [Source:MGI Symbol;Acc:MGI:1916145]
ENSMUSG00000029642	127.9832559	105.8358127	142.8334536	124.6538016	105.8720913	84.98642593	143.024368	163.1121108	127.4075963	94.87378509	93.51203943	96.66597415	Polr1d	polymerase (RNA) I polypeptide D [Source:MGI Symbol;Acc:MGI:108403]
ENSMUSG00000029644	0.019939058	0.091843402	0.102067308	0	0	0	0.053403412	0.079009912	0.222701491	0.017835017	0.099583528	0.070681936	Pdx1	pancreatic and duodenal homeobox 1 [Source:MGI Symbol;Acc:MGI:102851]
ENSMUSG00000029646	0	0	0	0.035518335	0	0	0	0.056557958	0.017713026	1.497983654	1.362340244	1.129988772	Cdx2	caudal type homeobox 2 [Source:MGI Symbol;Acc:MGI:88361]
ENSMUSG00000029647	30.80906035	34.08744883	26.83865766	35.91051265	41.92405967	47.5647179	21.33218919	18.55643673	22.46014607	27.15551617	27.51808375	28.94071302	Pan3	PAN3 poly(A) specific ribonuclease subunit [Source:MGI Symbol;Acc:MGI:1919837]
ENSMUSG00000029648	9.717038904	12.56558476	9.741899425	4.903318638	5.581087143	5.635947343	1.709326059	1.739830518	2.103618866	14.84664611	14.93068814	14.25040299	Flt1	FMS-like tyrosine kinase 1 [Source:MGI Symbol;Acc:MGI:95558]
ENSMUSG00000029649	143.9595087	120.6840936	168.711572	136.2881271	119.5987258	100.6341621	161.9927973	173.4729163	124.7739439	98.96905638	99.52290532	94.02081262	Pomp	proteasome maturation protein [Source:MGI Symbol;Acc:MGI:1913787]
ENSMUSG00000029650	1.467547334	0.743580726	1.171922072	0.93125392	0.832622507	0.849531231	1.126765842	1.192128073	0.601010459	2.074044573	1.465902134	1.326588089	Slc46a3	solute carrier family 46, member 3 [Source:MGI Symbol;Acc:MGI:1918956]
ENSMUSG00000029651	0.613440763	0.832552961	0.740185467	0.265781676	0.460369841	0.600745796	5.046350484	3.472574151	3.813233877	5.467486058	5.015092035	5.785949897	Mtus2	microtubule associated tumor suppressor candidate 2 [Source:MGI Symbol;Acc:MGI:1915388]

ENSMUSG00000029655	20.60784672	24.24870128	18.69288271	23.6292458	25.93345394	29.04041128	22.2306385	20.42937493	23.10891385	28.95353588	29.28605996	28.60931777	N4bp2l2	NEDD4 binding protein 2-like 2 [Source:MGI Symbol;Acc:MGI:2687207]
ENSMUSG00000029656	0.739593406	0.973348373	0.351552641	0.862720518	0.52729162	0.744406853	0.731037739	0.235501921	0.516287405	0.661548839	0.417753612	0.608628313	C8b	complement component 8, beta polypeptide [Source:MGI Symbol;Acc:MGI:88236]
ENSMUSG00000029657	76.53192262	88.53762961	64.73214577	65.66444449	67.75442883	73.7457547	82.25553499	77.32639655	90.48598744	118.4093059	114.4594757	115.1821328	Hsph1	heat shock 105kDa/110kDa protein 1 [Source:MGI Symbol;Acc:MGI:105053]
ENSMUSG00000029658	0.127021366	0.109703725	0.325108858	0.118534252	0.100092218	0.024187469	0	0.094374571	0.029556569	0.397661645	0.898726027	0.562847302	Wdr95	WD40 repeat domain 95 [Source:MGI Symbol;Acc:MGI:1923042]
ENSMUSG00000029659	69.67838997	61.65451557	79.33194476	65.19543175	62.49442373	47.53290219	0.132180143	0.183336979	0.155028974	0.380741173	0.770255387	0.72165497	Medag	mesenteric estrogen dependent adipogenesis [Source:MGI Symbol;Acc:MGI:1917967]
ENSMUSG00000029660	3.144389495	3.335063552	2.965055302	3.165947291	2.564702676	3.434969471	0.443248316	0.696768664	0.269561597	0.51810724	0.792103977	0.623326327	Tex26	testis expressed 26 [Source:MGI Symbol;Acc:MGI:1923110]
ENSMUSG00000029661	1541.578324	1686.416032	1435.16378	1039.918917	1059.253819	1088.31808	26.24231508	24.55457486	31.59471962	283.6805354	304.5060585	294.3133583	Col1a2	collagen, type I, alpha 2 [Source:MGI Symbol;Acc:MGI:88468]
ENSMUSG00000029663	0	0	0.042357933	0.05791367	0.065204305	0	0	0	0	0.01850383	0	0	Gngt1	guanine nucleotide binding protein (G protein), gamma transducing activity polypeptide 1 [Source:MGI Symbol;Acc:MGI:109165]
ENSMUSG00000029664	25.09577695	35.30186678	23.6928284	15.7528778	15.31504149	14.5365563	16.94904504	18.50924469	18.13634452	147.4998665	141.2660933	146.2723178	Tfpi2	tissue factor pathway inhibitor 2 [Source:MGI Symbol;Acc:MGI:108543]
ENSMUSG00000029669	0.801546767	1.006933425	0.559512069	0.543992926	0.344517244	0.166506455	0.422856141	0.649674224	0.712135632	1.62946513	2.244241139	2.744535901	Tspan12	tetraspanin 12 [Source:MGI Symbol;Acc:MGI:1889818]
ENSMUSG00000029670	5.277770228	5.711840813	4.252630627	6.11080753	7.008444582	7.723576698	15.05181676	14.30543781	18.19382238	15.23345404	14.60335979	14.85472607	Ing3	inhibitor of growth family, member 3 [Source:MGI Symbol;Acc:MGI:1919027]
ENSMUSG00000029671	0.325562329	0.156209066	0.055551387	0.37976177	0.171027686	0.247974935	0.217990975	0.080628968	0	0.388277088	0.158082048	0.21639101	Wnt16	wingless-type MMTV integration site family, member 16 [Source:MGI Symbol;Acc:MGI:2136018]
ENSMUSG00000029672	97.71118601	98.58708053	91.64818694	94.20509671	102.8947998	97.37875062	49.6983315	46.52666328	46.10824825	64.22235569	61.16623606	58.45169228	Fam3c	family with sequence similarity 3, member C [Source:MGI Symbol;Acc:MGI:107892]
ENSMUSG00000029673	0.182066944	0.244602686	0.155332335	0.05663396	0.302876604	0.346692743	0.514726536	0.300605414	0.833180515	1.601403396	1.80599901	1.774874266	Auts2	autism susceptibility candidate 2 [Source:MGI Symbol;Acc:MGI:1919847]
ENSMUSG00000029674	50.7818311	59.40423233	47.31148589	56.26917359	56.51938925	59.04277624	18.29060474	16.6501919	20.35911521	22.88763314	21.92608248	19.97654554	Limk1	LIM-domain containing, protein kinase [Source:MGI Symbol;Acc:MGI:104572]
ENSMUSG00000029675	0.642918368	0.74035355	0.593999	0.80482554	0.642538052	0.489699288	0.461986219	0.264081418	0.145951718	0.210393402	0.182739161	0.375214441	Eln	elastin [Source:MGI Symbol;Acc:MGI:95317]
ENSMUSG00000029676	8.181987499	9.562187226	7.229854305	10.58942879	10.92470398	13.81813547	21.21839138	22.60351969	25.11288052	27.35760371	26.37111205	27.2778673	Pot1a	protection of telomeres 1A [Source:MGI Symbol;Acc:MGI:2141503]
ENSMUSG00000029678	0	0	0	0	0	0	0	0	0	0	0	0	Hyal5	hyaluronoglucosaminidase 5 [Source:MGI Symbol;Acc:MGI:1921718]
ENSMUSG00000029679	0	0	0	0	0	0	0	0	0	0	0	0	Hyal6	hyaluronoglucosaminidase 6 [Source:MGI Symbol;Acc:MGI:1921659]
ENSMUSG00000029680	0	0	0	0	0	0	0	0	0	0	0	0	Hyal4	hyaluronoglucosaminidase 4 [Source:MGI Symbol;Acc:MGI:1924292]
ENSMUSG00000029681	65.40990856	51.45160835	66.67024814	60.80824187	50.97866992	48.46906507	25.9539897	26.67183561	24.675197	18.65299714	19.48960499	20.09113692	Bcl7b	B cell CLL/lymphoma 7B [Source:MGI Symbol;Acc:MGI:1332238]
ENSMUSG00000029682	0	0	0	0	0	0	0	0	0	0	0	0	Spam1	sperm adhesion molecule 1 [Source:MGI Symbol;Acc:MGI:109335]
ENSMUSG00000029683	0	0	0	0.040641172	0	0.033172086	0	0	0	0	0	0.038596057	Lmod2	leiomodin 2 (cardiac) [Source:MGI Symbol;Acc:MGI:2135672]
ENSMUSG00000029684	35.96787288	42.55397932	31.02667808	43.61904172	45.85862447	54.16356527	32.4387553	32.33841616	37.27536905	46.48493328	44.88081573	47.8438948	Wasl	Wiskott-Aldrich syndrome-like (human) [Source:MGI Symbol;Acc:MGI:1920428]
ENSMUSG00000029685	0.026643027	0.092042444	0.204577013	0.024862835	0.055985534	0.030440267	0	0.013196857	0.037197354	0	0.022177632	0	Asb15	ankyrin repeat and SOCS box-containing 15 [Source:MGI Symbol;Acc:MGI:1926160]
ENSMUSG00000029686	103.8241203	114.904454	93.88043144	119.5567838	121.3370032	138.9528683	95.28537678	88.08785748	103.0227195	130.7159797	120.9783309	120.5925127	Cul1	cullin 1 [Source:MGI Symbol;Acc:MGI:1349658]
ENSMUSG00000029687	39.02440521	35.14929848	29.64145683	47.62656605	48.47677945	52.33989981	139.5253898	141.3640318	150.2774891	91.93582232	89.40117738	89.25883918	Ezh2	enhancer of zeste 2 polycomb repressive complex 2 subunit [Source:MGI Symbol;Acc:MGI:107940]
ENSMUSG00000029695	1.513089682	2.359500744	1.613635538	0.32358114	0.794871531	0.888380047	13.64923386	12.55354636	14.99275929	4.314035791	5.589006943	4.832961547	Aass	aminoadipate-semialdehyde synthase [Source:MGI Symbol;Acc:MGI:1353573]
ENSMUSG00000029697	0	0	0	0	0	0	0	0	0	0.03195348	0	0	Fezf1	Fez family zinc finger 1 [Source:MGI Symbol;Acc:MGI:1920441]
ENSMUSG00000029699	4.822747841	3.584265439	4.237140122	3.383361407	4.024905926	3.986029537	7.389820894	6.132971728	6.940155033	4.925725426	7.260177462	6.153401949	Ssc4d	scavenger receptor cysteine rich family, 4 domains [Source:MGI Symbol;Acc:MGI:1924709]
ENSMUSG00000029700	0.070250592	0.060672876	0.01798051	0.245837619	0.073809499	0	0	0.086991526	0.032693185	0.047128122	0.014619133	0.031128902	Slc13a1	solute carrier family 13 (sodium/sulfate symporters), member 1 [Source:MGI Symbol;Acc:MGI:1859937]
ENSMUSG00000029701	63.06042389	67.22526041	56.67507785	68.87885702	69.83564438	76.66968253	113.6862005	110.5856667	124.8151277	123.4688013	124.7709571	120.4636384	Rbm28	RNA binding motif protein 28 [Source:MGI Symbol;Acc:MGI:2655711]
ENSMUSG00000029703	35.53692075	32.12976404	36.08744603	38.96590288	36.12629011	31.74983249	59.31579309	58.18237917	56.67670104	36.49054401	36.05672063	36.1751886	Lrwd1	leucine-rich repeats and WD repeat domain containing 1 [Source:MGI Symbol;Acc:MGI:1918985]
ENSMUSG00000029705	11.47902509	12.49019092	10.66572147	13.13057765	13.56142346	15.11630783	7.688436527	7.479992245	8.57585578	10.80620161	11.62753202	10.86927945	Cux1	cut-like homeobox 1 [Source:MGI Symbol;Acc:MGI:88568]
ENSMUSG00000029706	0	0.098117853	0.087232179	0	0	0	0	0	0.118957876	0	0	0	Pax4	paired box 4 [Source:MGI Symbol;Acc:MGI:97488]
ENSMUSG00000029707	0.037318723	0	0	0.243776535	0.039209296	0.028424996	0	0	0	0	0	0	Fscn3	fascin actin-bundling protein 3 [Source:MGI Symbol;Acc:MGI:1890386]
ENSMUSG00000029708	9.033159581	11.5398825	9.248082516	10.83805212	12.37191093	14.12938097	6.732500198	5.712742475	6.437141037	12.11992193	11.58090163	10.81087504	Gcc1	golgi coiled coil 1 [Source:MGI Symbol;Acc:MGI:1921625]
ENSMUSG00000029710	35.862437	39.91385814	34.7607509	37.6207354	37.23966189	39.64586092	16.76998491	16.86542516	18.97117624	13.36667593	13.51950872	14.63139873	Ephb4	Eph receptor B4 [Source:MGI Symbol;Acc:MGI:104757]
ENSMUSG00000029711	0.056691025	0	0	0	0.238252007	0	0.657961927	0.730086983	0.527656805	0.253543915	0.424706033	0.401927849	Epo	erythropoietin [Source:MGI Symbol;Acc:MGI:95407]
ENSMUSG00000029712	0.041294134	0	0.126829895	0.038535008	0.043386102	0	0.073732925	0	0.115304659	0.073873256	0.171866005	0	Actl6b	actin-like 6B [Source:MGI Symbol;Acc:MGI:1933548]
ENSMUSG00000029713	364.1934195	265.9687356	444.6488186	328.4714683	267.8387013	200.9362982	123.7404753	140.4471773	106.5661725	76.20817626	69.11756109	77.78715683	Gnb2	guanine nucleotide binding protein (G protein), beta 2 [Source:MGI Symbol;Acc:MGI:95784]
ENSMUSG00000029714	29.10669885	30.05540179	26.25705277	33.37581677	32.48536468	35.75442775	27.91021784	26.79416719	32.03676543	27.00543652	27.39408121	26.75630422	Gigyf1	GRB10 interacting GYF protein 1 [Source:MGI Symbol;Acc:MGI:1888677]
ENSMUSG00000029715	29.95887227	25.17221382	37.17350311	31.65038841	28.140365	21.52219109	23.33651028	25.9858109	19.7461592	16.58893096	17.0082275	17.37392235	Pop7	processing of precursor 7, ribonuclease P family, (S. cerevisiae) [Source:MGI Symbol;Acc:MGI:1921347]
ENSMUSG00000029716	1.882012949	2.018793926	2.111551837	1.347270676	1.598137078	2.336793562	3.015186269	2.656134308	2.351597873	1.360569213	1.609506088	1.507951893	Tfr2	transferrin receptor 2 [Source:MGI Symbol;Acc:MGI:1354956]
ENSMUSG00000029718	1067.837982	862.7066165	1165.182018	790.5416716	694.5353948	571.9394341	189.3468157	194.5017431	190.5138138	150.4494605	151.5412002	155.3153208	Pcolce	procollagen C-endopeptidase enhancer protein [Source:MGI Symbol;Acc:MGI:105099]
ENSMUSG00000029720	0	0.011099802	0.009868336	0	0	0.007341851	0.008605478	0.019097602	0	0	0.016046987	0	Gm20605	predicted gene 20605 [Source:MGI Symbol;Acc:MGI:5306917]
ENSMUSG00000029722	20.64317358	21.71054761	19.3263061	21.89177411	21.06211171	20.64963253	25.58859664	24.49272225	28.00997231	13.08548163	13.50830578	14.02224948	Agfg2	ArfGAP with FG repeats 2 [Source:MGI Symbol;Acc:MGI:2443267]
ENSMUSG00000029723	76.19513273	73.214009	79.02330669	67.32519747	59.82432396	53.54628724	34.33235357	38.42329934	36.94675246	30.80022917	31.57479996	29.66163841	Tsc22d4	TSC22 domain family, member 4 [Source:MGI Symbol;Acc:MGI:1926079]
ENSMUSG00000029725	89.46210779	66.03631489	125.659836	91.06750998	69.19474625	55.88088411	113.3100012	143.3639122	91.83933128	39.11097302	40.5838697	47.52896214	Ppp1r35	protein phosphatase 1, regulatory subunit 35 [Source:MGI Symbol;Acc:MGI:1922853]
ENSMUSG00000029726	82.46396547	73.26466058	82.36245556	77.03579658	71.81769514	65.11416931	47.84253211	48.22537352	47.67561928	53.69502437	53.61589303	52.34537222	Mepce	methylphosphate capping enzyme [Source:MGI Symbol;Acc:MGI:106477]
ENSMUSG00000029727	0.407577016	0.519632945	0.566300916	0.692769746	1.024681099	1.053292274	0.051982211	0.057680443	0.040645282	0.182284009	0.218100054	0.387005122	Cyp3a13	cytochrome P450, family 3, subfamily a, polypeptide 13 [Source:MGI Symbol;Acc:MGI:88610]
ENSMUSG00000029729	5.293557381	7.201339581	5.296644895	7.814187815	9.120116283	11.08547987	1.916576233	1.347330792	1.818159497	4.526242094	4.334208204	4.579020938	Zkscan1	zinc finger with KRAB and SCAN domains 1 [Source:MGI Symbol;Acc:MGI:1921820]
ENSMUSG00000029730	120.9471165	113.1566185	115.3278011	149.4476352	140.0272632	126.8612372	537.1245467	535.417366	540.1010575	313.4174565	325.7055193	321.4875223	Mcm7	minichromosome maintenance complex component 7 [Source:MGI Symbol;Acc:MGI:1298398]
ENSMUSG00000029735	2.518740751	3.142164405	1.960862356	3.329800601	3.087397351	3.177481269	6.53520993	5.796075632	7.008601516	5.937472861	5.285173089	5.04563434	Tpk1	thiamine pyrophosphokinase [Source:MGI Symbol;Acc:MGI:1352500]
ENSMUSG00000029736	0.938315934	0.578849482	1.303727031	1.157070623	1.161896032	1.072047404	0.029918111	0.099593102	0.530245776	0.389675672	0.557895257	0.089095572	Nobox	NOBOX oogenesis homeobox [Source:MGI Symbol;Acc:MGI:108011]
ENSMUSG00000029752	51.41576667	42.4566408	43.56029613	90.33513389	87.2038628	81.61895698	150.0324301	153.063577	157.2691554	302.9413231	300.2435948	300.1853228	Asns	asparagine synthetase [Source:MGI Symbol;Acc:MGI:1350929]
ENSMUSG00000029754	0	0	0	0	0	0	0	0	0	0	0	0	Dlx6	distal-less homeobox 6 [Source:MGI Symbol;Acc:MGI:101927]
ENSMUSG00000029755	0.031202674	0.071862967	0	0	0	0	0	0	0	0	0.051946157	0	Dlx5	distal-less homeobox 5 [Source:MGI Symbol;Acc:MGI:101926]
ENSMUSG00000029757	0.192413584	0.1661806	0.172367664	0	0	0.018319743	1.588987815	1.572557825	1.768520386	0.43027352	0.400412247	0.404988926	Dync1i1	dynein cytoplasmic 1 intermediate chain 1 [Source:MGI Symbol;Acc:MGI:107743]
ENSMUSG00000029759	8.131061443	10.03534494	7.607576662	2.468749089	2.779534944	4.504205368	2.778651289	3.006162858	4.562561566	10.19617916	11.10776518	12.03289669	Pon3	paraoxonase 3 [Source:MGI Symbol;Acc:MGI:106686]
ENSMUSG00000029761	406.2754533	486.1722509	366.8024098	412.867293	491.4857546	594.4186112	36.52183737	31.80495057	47.58453195	306.8692112	302.7943321	307.6066966	Cald1	caldesmon 1 [Source:MGI Symbol;Acc:MGI:88250]
ENSMUSG00000029762	122.6545754	105.7864206	124.8147089	120.3513208	105.8136727	102.3594413	63.08526307	60.15495536	58.8403295	58.01084786	58.00776572	58.82276874	Akr1b8	aldo-keto reductase family 1, member B8 [Source:MGI Symbol;Acc:MGI:107673]
ENSMUSG00000029763	49.13759637	57.19416404	51.51590641	59.72663551	69.77014961	81.95831758	42.30542614	38.73243142	52.27428296	70.32751451	66.71804159	69.78700541	Exoc4	exocyst complex component 4 [Source:MGI Symbol;Acc:MGI:1096376]
ENSMUSG00000029765	0.084228024	0.069280643	0.06159431	0.039300104	0.018963221	0.018329989	0.042969635	0.101319828	0.07839599	0.629626967	0.946503001	0.655809183	Plxna4	plexin A4 [Source:MGI Symbol;Acc:MGI:2179061]
ENSMUSG00000029766	0	0	0	0	0	0	0	0	0	0	0	0	1700012A03	RIKEN cDNA 1700012A03 gene [Source:MGI Symbol;Acc:MGI:1923632]
ENSMUSG00000029767	550.3201682	649.8170764	482.4091468	521.670386	592.8563167	635.4529776	173.8349837	157.9304899	189.4795887	320.8128871	308.8230408	300.6021385	Calu	calumenin [Source:MGI Symbol;Acc:MGI:1097158]
ENSMUSG00000029769	25.63927194	22.97706996	26.27046732	24.42782457	24.43986456	23.73399765	15.06320083	17.77954628	15.80026593	18.67988757	17.7105069	17.75626364	Ccdc136	coiled-coil domain containing 136 [Source:MGI Symbol;Acc:MGI:1918128]

ENSMUSG00000029771	6.27868289	5.596276703	6.173852048	5.528137509	4.733269934	5.944175219	5.003748511	4.849436919	5.117575009	4.664246543	4.192907105	4.338285942	Irf5	interferon regulatory factor 5 [Source:MGI Symbol;Acc:MGI:1350924]
ENSMUSG00000029772	8.321946711	10.48537978	6.373701554	10.41382029	13.30441652	18.08054977	12.74193486	10.29984804	14.7227934	13.08818148	15.4054868	14.48750119	Ahcyl2	S-adenosylhomocysteine hydrolase-like 2 [Source:MGI Symbol;Acc:MGI:1921590]
ENSMUSG00000029775	26.32138948	30.70733051	25.59190589	28.2022319	32.34338796	33.69976237	31.75865465	32.70617319	35.84500366	35.37258292	38.54995072	38.58304016	Klhdc10	kelch domain containing 10 [Source:MGI Symbol;Acc:MGI:1924038]
ENSMUSG00000029776	22.76877337	22.89886231	19.2848406	21.40470424	19.37780399	19.12530969	37.94678796	33.55533442	35.7967612	27.46747818	28.63167983	30.1678071	Hibadh	3-hydroxyisobutyrate dehydrogenase [Source:MGI Symbol;Acc:MGI:1889802]
ENSMUSG00000029777	214.2968302	195.4265854	208.807	232.6959294	216.9179175	205.0172296	242.1907013	247.4103435	251.9796956	209.8074176	201.5274536	196.1697274	Gars	glycyl-tRNA synthetase [Source:MGI Symbol;Acc:MGI:2449057]
ENSMUSG00000029778	1.132328627	1.166678346	0.976227151	0.444913882	0.709641193	0.665769579	0.922240264	0.767501413	1.05392504	2.025682441	2.16620992	1.989389764	Adcyap1r1	adenylate cyclase activating polypeptide 1 receptor 1 [Source:MGI Symbol;Acc:MGI:108449]
ENSMUSG00000029780	22.87062205	24.84684731	20.80838588	25.39288802	22.23144268	22.18842522	27.795811	24.97188362	25.59822721	20.86786925	19.24592533	19.97261739	Nt5c3	5'-nucleotidase, cytosolic III [Source:MGI Symbol;Acc:MGI:1927186]
ENSMUSG00000029781	232.3876959	242.7341364	216.1011734	172.418447	172.4980691	163.6674533	112.8044956	111.7520819	118.1888957	140.0191784	140.9521896	139.7992155	Fkbp9	FK506 binding protein 9 [Source:MGI Symbol;Acc:MGI:1350921]
ENSMUSG00000029782	21.54487644	25.06871555	18.83772066	30.83451691	37.56989342	46.21339123	49.18997451	43.61714648	59.26590149	48.26065835	43.24543485	43.5623643	Tmem209	transmembrane protein 209 [Source:MGI Symbol;Acc:MGI:1919899]
ENSMUSG00000029784	0.267812165	0.102799825	0.137092065	0.208264927	0.375172766	0.237985627	0.239096602	0.221088422	0.083089526	0.319402211	0.483007657	0.316455641	Ssmem1	serine-rich single-pass membrane protein 1 [Source:MGI Symbol;Acc:MGI:1922897]
ENSMUSG00000029787	9.107946465	13.48799968	8.231017132	12.78405588	16.5593187	21.19748286	9.168702104	6.646364755	10.58790722	15.72374236	16.59806425	16.70805841	Avl9	AVL9 cell migration associated [Source:MGI Symbol;Acc:MGI:1926187]
ENSMUSG00000029788	0	0	0	0	0	0	0	0	0	0	0	0	Cpa5	carboxypeptidase A5 [Source:MGI Symbol;Acc:MGI:1921899]
ENSMUSG00000029790	6.326857298	7.117050917	5.847643832	8.85617891	7.355199513	8.544894421	10.95699378	10.59117387	12.67723312	13.23105719	13.21493976	12.48599534	Cep41	centrosomal protein 41 [Source:MGI Symbol;Acc:MGI:1891414]
ENSMUSG00000029797	0.078635729	0.12451049	0.10566502	0.077967918	0.056800857	0.029947706	0.048265379	0.053556176	0.059467615	0.057149465	0.143185682	0.174222294	Sspo	SCO-spondin [Source:MGI Symbol;Acc:MGI:2674311]
ENSMUSG00000029798	13.83058537	14.53086902	10.76931267	16.46131008	17.1236739	16.84861624	0.173910305	0.364506746	0.322327175	2.652339735	2.234050583	2.570326574	Herc6	hect domain and RLD 6 [Source:MGI Symbol;Acc:MGI:1914388]
ENSMUSG00000029802	43.6520891	52.45974652	38.94474293	48.33118842	53.31202149	60.62318844	51.57142881	49.97401061	58.46992831	55.25122251	57.296086	56.34129396	Abcg2	ATP binding cassette subfamily G member 2 (Junior blood group) [Source:MGI Symbol;Acc:MGI:1347061]
ENSMUSG00000029804	12.37027234	19.71200622	10.98739107	20.27953426	28.34740708	35.11684107	12.47661927	11.16154676	18.11080512	19.55798757	19.20252065	20.38495604	Herc3	hect domain and RLD 3 [Source:MGI Symbol;Acc:MGI:1921248]
ENSMUSG00000029810	494.737214	398.1137027	535.1978212	450.2925729	386.5976047	312.9023882	18.56179924	18.40254559	14.38740527	78.51221712	80.17411938	80.39153331	Tmem176b	transmembrane protein 176B [Source:MGI Symbol;Acc:MGI:1916348]
ENSMUSG00000029811	0.021930932	0	0.044905454	0.020465585	0	0	0.019579436	0.065177137	0	0.039233401	0.127787018	0.019435731	Aoc1	amine oxidase, copper-containing 1 [Source:MGI Symbol;Acc:MGI:1923757]
ENSMUSG00000029813	0	0	0	0	0	0	0	0	0.032232718	0	0	0	Doxl1	diamine oxidase-like protein 1 [Source:MGI Symbol;Acc:MGI:1917011]
ENSMUSG00000029814	4.574387784	7.095683063	2.929688484	5.145882596	7.329892392	10.50041438	15.2540537	10.34607954	18.04155952	13.54552215	14.13549503	13.06884986	Igf2bp3	insulin-like growth factor 2 mRNA binding protein 3 [Source:MGI Symbol;Acc:MGI:1890359]
ENSMUSG00000029815	24.88103437	17.35102337	19.53755939	19.2173136	16.49707056	12.85661581	27.71261052	34.36989404	26.44649234	16.88068701	16.66427259	17.47423632	Malsu1	mitochondrial assembly of ribosomal large subunit 1 [Source:MGI Symbol;Acc:MGI:1922843]
ENSMUSG00000029816	91.30669088	102.6323672	82.33721416	14.40603884	16.92478548	15.24585361	13.56825486	12.5621579	13.25303399	95.78035977	96.75544802	99.27301594	Gpnmb	glycoprotein (transmembrane) nmb [Source:MGI Symbol;Acc:MGI:1934765]
ENSMUSG00000029817	44.93971718	47.80467474	40.56191585	53.60224172	53.90987942	53.24630804	103.6847502	98.53922456	104.682296	93.5785849	92.50184648	92.57796428	Tra2a	transformer 2 alpha [Source:MGI Symbol;Acc:MGI:1933972]
ENSMUSG00000029819	12.99977282	11.73080405	16.34447445	1.418850807	0.559113478	0.868569452	1.96825224	1.65683507	1.556680801	4.555996019	3.796841924	3.907612141	Npy	neuropeptide Y [Source:MGI Symbol;Acc:MGI:97374]
ENSMUSG00000029821	2.563205655	4.06945264	3.602439917	4.939571295	4.541547666	4.332131737	2.694591456	3.260419087	2.724528109	3.961398019	4.25459536	3.763557742	Gsdme	gasdermin E [Source:MGI Symbol;Acc:MGI:1889850]
ENSMUSG00000029822	13.67430757	14.88775893	12.82877548	17.54007853	18.98192103	21.99242833	4.942435	4.663228331	5.738938508	8.287663021	9.312939502	8.813460252	Osbpl3	oxysterol binding protein-like 3 [Source:MGI Symbol;Acc:MGI:1918970]
ENSMUSG00000029823	55.39430325	73.51965517	45.88221527	78.30534726	95.62378817	124.3382973	54.61930724	51.99451966	67.60562965	75.95850161	71.60669894	73.74569672	Luc7l2	LUC7-like 2 (S. cerevisiae) [Source:MGI Symbol;Acc:MGI:2183260]
ENSMUSG00000029826	30.24821068	34.05354244	29.04583326	40.58874589	48.27121255	53.23779938	12.66961447	13.19206452	16.61659918	25.29358224	26.74132573	25.81463304	Zc3hav1	zinc finger CCCH type, antiviral 1 [Source:MGI Symbol;Acc:MGI:1926031]
ENSMUSG00000029828	0.108566412	0.10418317	0.148199328	0.11819779	0.133077472	0.082693071	0.33923967	0.197175915	0.387355124	0.436995413	0.52716175	0.705570987	4921507P07Rik	RIKEN cDNA 4921507P07 gene [Source:MGI Symbol;Acc:MGI:1918071]
ENSMUSG00000029829	0	0	0	0.254538687	0	0	0.36527595	0	0.063469337	0.060995192	0	0	Tmem213	transmembrane protein 213 [Source:MGI Symbol;Acc:MGI:1924772]
ENSMUSG00000029830	0.141607623	0.11647747	0.165687824	0.113267921	0.042508997	0	0.03612116	0.140282527	0.131802567	0.018094954	0.185230668	0.089640114	Svopl	SV2 related protein homolog (rat)-like [Source:MGI Symbol;Acc:MGI:2444335]
ENSMUSG00000029831	0	0	0.05425154	0.049450096	0	0.201810472	0	0	0.049321615	0	0	0	Npvf	neuropeptide VF precursor [Source:MGI Symbol;Acc:MGI:1926488]
ENSMUSG00000029832	0.650660894	0.358346267	0.231701513	0.686384237	0.475564164	0.474048951	1.565891487	1.597418356	1.737833005	2.45452514	2.802240751	2.732732815	Nfe2l3	nuclear factor, erythroid derived 2, like 3 [Source:MGI Symbol;Acc:MGI:1339958]
ENSMUSG00000029833	25.88323681	25.65670712	22.72112419	29.02680619	30.41320122	34.37825061	58.30604065	51.98904968	63.78378218	54.13537988	54.49323167	58.43343162	Trim24	tripartite motif-containing 24 [Source:MGI Symbol;Acc:MGI:109275]
ENSMUSG00000029836	56.79883069	71.6578526	40.81819334	81.79518151	90.19107844	93.46319071	106.7292239	106.3671367	123.9439885	107.0695173	99.86857938	114.9151255	Cbx3	chromobox 3 [Source:MGI Symbol;Acc:MGI:108515]
ENSMUSG00000029837	0	0	0	0	0	0	0	0	0	0	0	0	1700111E14Rik	RIKEN cDNA 1700111E14 gene [Source:MGI Symbol;Acc:MGI:1920835]
ENSMUSG00000029838	23.72136194	25.84365916	25.42613099	18.69080871	18.96322078	18.55518618	0.18415558	0.224776762	0.057597054	0.959431568	0.721145144	0.694655023	Ptn	pleiotrophin [Source:MGI Symbol;Acc:MGI:97804]
ENSMUSG00000029840	114.0796476	142.4350149	99.23348992	120.6258254	142.1217111	178.5546644	54.16758142	47.21032102	63.67540155	151.4858173	146.3164442	146.1790433	Mtpn	myotrophin [Source:MGI Symbol;Acc:MGI:99445]
ENSMUSG00000029843	0.122880022	0.188670217	0.041934555	0.19111603	0.043035048	0.124793986	0.347397538	0.284037062	0.07624779	1.318959343	1.380850506	0.980093695	Slc13a4	solute carrier family 13 (sodium/sulfate symporters), member 4 [Source:MGI Symbol;Acc:MGI:2442367]
ENSMUSG00000029844	0.961978303	1.453944799	1.661961931	2.300362261	1.431842407	1.709683424	0.518877861	0.615464033	0.484992054	0.448159832	0.383692658	0.266415263	Hoxa1	homeobox A1 [Source:MGI Symbol;Acc:MGI:96170]
ENSMUSG00000029847	0.247824264	0.285382379	0.222005581	0.115632766	0.292926469	0.165167851	0.359534271	0.245505242	0.317163907	1.219201239	0.850940886	1.043232814	Slc23a4	solute carrier family 23 member 4 [Source:MGI Symbol;Acc:MGI:1917272]
ENSMUSG00000029848	36.78241701	28.69076792	35.31285217	58.99970695	55.70831487	43.98062124	6.164964484	6.909512409	5.781288809	3.383216399	2.744034815	3.382757674	Stra8	stimulated by retinoic acid gene 8 [Source:MGI Symbol;Acc:MGI:107917]
ENSMUSG00000029851	5.974413376	7.373875479	5.173048583	6.479715589	8.53081635	9.851570457	1.191598132	1.023146271	1.050033146	6.637326549	6.811554975	5.900179861	Tcaf2	TRPM8 channel-associated factor 2 [Source:MGI Symbol;Acc:MGI:2385258]
ENSMUSG00000029859	3.716746457	3.29232662	3.018530433	2.601305042	2.328003104	2.749315685	1.898408486	1.894088747	2.428229287	8.327338239	7.64529651	7.126165743	Epha1	Eph receptor A1 [Source:MGI Symbol;Acc:MGI:107381]
ENSMUSG00000029860	345.2752492	323.7637045	352.686068	303.4451804	269.9966707	250.3589487	24.57096143	23.63299196	27.72841863	99.66795889	97.90914642	98.90319296	Zyx	zyxin [Source:MGI Symbol;Acc:MGI:103072]
ENSMUSG00000029861	3.407041093	3.813381567	3.531569145	4.367953558	4.560977357	4.899125926	0.170564226	0.231319351	0.138304914	1.139258995	1.139708174	1.081717819	Fam131b	family with sequence similarity 131, member B [Source:MGI Symbol;Acc:MGI:1923406]
ENSMUSG00000029862	0.358570732	0.619368945	0.312036772	0.568840936	0.82881917	0.628168729	0.576222811	0.461780017	0.817670177	0.625429454	0.492482038	0.492550019	Clcn1	chloride channel, voltage-sensitive 1 [Source:MGI Symbol;Acc:MGI:88417]
ENSMUSG00000029863	11.49535582	16.79987662	10.50919694	16.96025167	17.02698918	22.43842263	23.47574415	17.86524499	26.14319613	25.78442192	23.84865483	22.46227474	Casp2	caspase 2 [Source:MGI Symbol;Acc:MGI:97295]
ENSMUSG00000029864	4.879519238	3.933312065	5.358933529	2.773487644	1.724441009	2.196197259	3.801882465	4.921747363	4.087490722	5.515285903	4.283255212	5.267844565	Gstk1	glutathione S-transferase kappa 1 [Source:MGI Symbol;Acc:MGI:1923513]
ENSMUSG00000029865	0	0	0	0	0	0	0	0	0	0	0	0	Sval1	seminal vesicle antigen-like 1 [Source:MGI Symbol;Acc:MGI:1918828]
ENSMUSG00000029866	0	0	0	0	0	0	0.085082733	0.047204704	0	0.042622333	0.079328628	0.04222913	Kel	Kell blood group [Source:MGI Symbol;Acc:MGI:1346053]
ENSMUSG00000029867	0	0	0	0	0	0	0	0	0	0	0	0	Llcfc1	LLLL and CFNLAS motif containing 1 [Source:MGI Symbol;Acc:MGI:1923856]
ENSMUSG00000029868	0	0.035672797	0.11100279	0.014454096	0	0	0.013828241	0	0	0.01385456	0.10314435	0.041180243	Trpv6	transient receptor potential cation channel, subfamily V, member 6 [Source:MGI Symbol;Acc:MGI:1927259]
ENSMUSG00000029869	7.775862443	9.591370524	6.953962353	6.682685954	5.715623106	6.437754054	2.634161422	2.131292385	2.374342555	6.487971474	6.472621069	5.610984547	Ephb6	Eph receptor B6 [Source:MGI Symbol;Acc:MGI:1096338]
ENSMUSG00000029875	14.39582596	12.38321788	16.79519784	19.78674907	16.37036183	14.77418062	0.090327168	0.157502292	0.215245692	0.271497243	0.348904733	0.20494675	Ccdc184	coiled-coil domain containing 184 [Source:MGI Symbol;Acc:MGI:2146066]
ENSMUSG00000029878	0	0	0.031707474	0	0.010846526	0	0.009216616	0	0	0	0.042966501	0.009148969	Dbpht2	DNA binding protein with his-thr domain [Source:MGI Symbol;Acc:MGI:2679513]
ENSMUSG00000029882	0.140615973	0.04626472	0.041131886	0.206203668	0.147739602	0.091803979	0	0.059700067	0	0	0	0	2210010C04Rik	RIKEN cDNA 2210010C04 gene [Source:MGI Symbol;Acc:MGI:1914623]
ENSMUSG00000029883	0	0	0	0	0	0	0	0	0	0	0	0	1700074P13Rik	RIKEN cDNA 1700074P13 gene [Source:MGI Symbol;Acc:MGI:1920731]
ENSMUSG00000029885	0	0	0	0	0.09722219	0	0	0	0	0	0	0	Moxd2	monooxygenase, DBH-like 2 [Source:MGI Symbol;Acc:MGI:2388042]
ENSMUSG00000029909	0	0	0	0	0	0	0	0	0	0	0.058524306	0	Prss37	protease, serine 37 [Source:MGI Symbol;Acc:MGI:1914940]
ENSMUSG00000029910	16.80848363	19.00165485	14.23274989	21.64097376	23.75133237	22.68880557	44.82648268	44.80436672	48.46548501	39.57465306	38.50506127	37.54687363	Mad2l1	MAD2 mitotic arrest deficient-like 1 [Source:MGI Symbol;Acc:MGI:1860374]
ENSMUSG00000029911	24.82610246	24.83388611	23.65574295	27.91097952	26.35353641	25.4604862	70.43487265	78.8425584	67.98319575	49.64855805	45.90373824	44.23052907	Ssbp1	single-stranded DNA binding protein 1 [Source:MGI Symbol;Acc:MGI:1920040]
ENSMUSG00000029913	10.73615291	11.51808916	10.86278684	12.32782214	12.18333336	12.84128294	23.58806829	21.81147178	24.37689583	11.77896827	11.92150388	10.92697269	Prdm5	PR domain containing 5 [Source:MGI Symbol;Acc:MGI:1918029]
ENSMUSG00000029915	0.932202131	0.790984286	0.753459239	0	0.051548824	0	0	0.048604159	0	1.206863256	1.388567578	1.173989084	Clec5a	C-type lectin domain family 5, member a [Source:MGI Symbol;Acc:MGI:1345151]
ENSMUSG00000029916	8.881918074	9.323096465	8.630047908	10.9549887	10.40767041	11.40833369	14.00962304	13.79973487	18.10744704	17.29509059	18.53280497	17.19884744	Agk	acylglycerol kinase [Source:MGI Symbol;Acc:MGI:1917173]
ENSMUSG00000029917	0	0	0	0	0.102358457	0.07420533	0	0.160852255	0	0.029047522	0	0.057559101	Qrfprl	pyroglutamylated RFamide peptide receptor like [Source:MGI Symbol;Acc:MGI:2441881]

ENSMUSG00000029918	45.17675301	44.01976058	57.0544562	49.82333153	48.72673373	35.61347323	52.46081393	52.13998698	34.99154942	45.1569532	36.55319891	39.19681833	Mrps33	mitochondrial ribosomal protein S33 [Source:MGI Symbol;Acc:MGI:1338046]
ENSMUSG00000029919	0.745871322	0.343563668	0.458170553	0.077337207	0.174146065	0.138872969	0.177572515	0.098518908	0.200544303	1.363980318	1.779806216	1.557044644	Hpgds	hematopoietic prostaglandin D synthase [Source:MGI Symbol;Acc:MGI:1859384]
ENSMUSG00000029920	19.86136946	20.67400152	17.99511032	20.56580376	25.6679334	34.69829168	59.79376411	57.45570227	71.14290252	94.7174188	94.94492163	100.8699884	Smarcad1	SWI/SNF-related, matrix-associated actin-dependent regulator of chromatin, subfamily a, containing DEAD/H box 1 [Source:MGI Sy
ENSMUSG00000029922	41.87988343	35.40704607	40.86627739	36.88293447	36.26567358	39.01028192	606.4866694	599.5043933	558.0291427	282.166056	284.9660237	285.5400255	Mkrn1	makorin, ring finger protein, 1 [Source:MGI Symbol;Acc:MGI:1859353]
ENSMUSG00000029923	0.273196748	0.283140089	0.195787779	0.076482815	0.114814777	0.041617804	0.463417287	0.92017703	0.610272785	1.14852979	1.591861132	1.380047979	Rab19	RAB19, member RAS oncogene family [Source:MGI Symbol;Acc:MGI:103292]
ENSMUSG00000029924	40.87184575	49.95007759	39.10215903	46.74293297	49.77666095	47.90252976	53.41434843	48.51834564	58.01784926	53.11152676	54.9269303	56.25913586	Slc37a3	solute carrier family 37 (glycerol-3-phosphate transporter), member 3 [Source:MGI Symbol;Acc:MGI:1919394]
ENSMUSG00000029925	1.189923614	1.247913727	1.435777043	0.019828878	0.022325095	0.21040112	0.094851492	0.126298808	0.079109435	0.760256126	0.77824322	0.828566818	Tbxas1	thromboxane A synthase 1, platelet [Source:MGI Symbol;Acc:MGI:98497]
ENSMUSG00000029992	32.77882468	41.5651238	31.11022994	34.13326934	43.92027191	55.79696647	14.46164053	12.44332313	18.35040525	31.4231505	32.83910629	30.01118375	Gfpt1	glutamine fructose-6-phosphate transaminase 1 [Source:MGI Symbol;Acc:MGI:95698]
ENSMUSG00000029993	52.07572521	48.25345183	61.15905963	64.59296887	49.39258926	46.45733911	90.62659235	97.90222364	74.69932286	61.10061462	59.52663812	58.88733269	Nfu1	NFU1 iron-sulfur cluster scaffold [Source:MGI Symbol;Acc:MGI:1913290]
ENSMUSG00000029994	99.95529576	108.6881593	92.83738678	106.8547096	109.6581937	109.7433115	32.54150861	32.3803788	34.61548565	30.72145525	28.61403275	30.80571433	Anxa4	annexin A4 [Source:MGI Symbol;Acc:MGI:88030]
ENSMUSG00000029998	155.0491252	184.9768211	138.123276	124.3357489	133.681694	148.0279975	52.7464298	49.8511536	62.26619908	82.3761097	81.94599005	73.88443577	Pcyox1	prenylcysteine oxidase 1 [Source:MGI Symbol;Acc:MGI:1914131]
ENSMUSG00000029999	0.827889892	0.903181044	0.646843167	0.71158062	0.595147482	0.547616547	1.089231305	1.230214339	1.115292827	3.390838556	2.593323136	2.548629579	Tgfa	transforming growth factor alpha [Source:MGI Symbol;Acc:MGI:98724]
ENSMUSG00000030000	0.71170483	0.71711911	0.834897919	0.996226879	1.261844861	1.185827763	0.225035327	0.176261259	0.138005346	0.716178561	0.65413315	0.643870548	Add2	adducin 2 (beta) [Source:MGI Symbol;Acc:MGI:87919]
ENSMUSG00000030001	1.077636547	1.684151694	1.654914587	1.292956352	2.102712481	1.348483854	0	0.152507505	0	0	0	0.068216287	Figla	folliculogenesis specific basic helix-loop-helix [Source:MGI Symbol;Acc:MGI:1349421]
ENSMUSG00000030002	49.80694824	56.91406692	45.16864284	61.49532633	68.21501734	87.34500979	42.72994727	38.97637444	48.30586016	63.53150613	65.62338487	65.87067509	Dusp11	dual specificity phosphatase 11 (RNA/RNP complex 1-interacting) [Source:MGI Symbol;Acc:MGI:1919352]
ENSMUSG00000030004	0.076631323	0.044122454	0	0	0	0.058368692	0.03420735	0.151828487	0.106987938	0	0.127575692	0	Nat8	N-acetyltransferase 8 (GCN5-related) [Source:MGI Symbol;Acc:MGI:1915646]
ENSMUSG00000030007	249.6359016	217.9490065	249.0914034	237.3950721	224.5279841	194.9257134	520.5540333	536.6602304	481.7029975	379.7252424	393.6462411	396.5292129	Cct7	chaperonin containing Tcp1, subunit 7 (eta) [Source:MGI Symbol;Acc:MGI:107184]
ENSMUSG00000030008	14.95198419	13.2642204	15.25103643	14.00462354	11.57844962	10.92467355	21.60526542	22.43754403	21.44201677	11.73957275	9.495851157	10.30619046	Pradc1	protease-associated domain containing 1 [Source:MGI Symbol;Acc:MGI:1920577]
ENSMUSG00000030016	30.25730594	33.64578843	29.43784968	32.13317575	36.33438183	41.11598447	40.50297815	40.12493515	46.11353987	70.0686877	67.50816608	70.21192287	Zfp638	zinc finger protein 638 [Source:MGI Symbol;Acc:MGI:1203484]
ENSMUSG00000030017	2.40718813	2.044350645	2.43365578	0.758142588	0.821969425	0.504215702	0	0	0.0280064	0	0	0.026666367	Reg3g	regenerating islet-derived 3 gamma [Source:MGI Symbol;Acc:MGI:109406]
ENSMUSG00000030019	13.18591527	13.27786548	11.37359818	14.66286298	14.44896304	15.6293254	15.54485657	14.99025761	17.12530374	16.22391833	17.37051976	17.14243302	Fbxl14	F-box and leucine-rich repeat protein 14 [Source:MGI Symbol;Acc:MGI:2141676]
ENSMUSG00000030020	1.542650971	1.747079218	1.344409413	1.725112381	2.036048708	2.893824637	0.284554475	0.208393033	0.35005925	1.887335832	2.026969842	1.926417446	Prickle2	prickle planar cell polarity protein 2 [Source:MGI Symbol;Acc:MGI:1925144]
ENSMUSG00000030022	9.640598919	10.7824286	7.956944261	9.849147033	11.25084134	14.31077494	1.659052395	1.698524592	2.111873655	7.778407902	8.11028748	8.170686808	Adamts9	a disintegrin-like and metallopeptidase (reprolysin type) with thrombospondin type 1 motif, 9 [Source:MGI Symbol;Acc:MGI:1916320
ENSMUSG00000030029	80.57225887	108.1247037	67.34632658	111.7594126	126.7580275	154.4502675	8.513818398	7.326317573	10.35810703	44.92900351	44.97727702	42.44480797	Lrig1	leucine-rich repeats and immunoglobulin-like domains 1 [Source:MGI Symbol;Acc:MGI:107935]
ENSMUSG00000030030	1.304531171	1.550693887	1.701773969	1.276271912	1.3927257	1.105822701	0.732606019	0.854601348	0.822525361	0.997487637	1.190976789	0.969638652	1700003E16Rik	RIKEN cDNA 1700003E16 gene [Source:MGI Symbol;Acc:MGI:1919087]
ENSMUSG00000030031	0.97448352	1.315645085	0.516035731	0.83097787	1.182724455	1.458898224	7.79997037	7.057149326	10.1022069	18.93589911	17.70564793	18.0911672	Kbtbd8	kelch repeat and BTB (POZ) domain containing 8 [Source:MGI Symbol;Acc:MGI:2661430]
ENSMUSG00000030032	6.575782193	4.554385157	6.976159223	6.514379294	5.907620781	4.754592141	3.530933431	2.785077535	2.638897581	2.045871967	2.300530208	2.977153688	Wdr54	WD repeat domain 54 [Source:MGI Symbol;Acc:MGI:1922909]
ENSMUSG00000030034	19.66779482	13.8088814	25.0634789	16.99843469	10.02691728	10.93520843	13.18774161	15.45546648	12.01088344	11.24576067	8.634733722	9.854984047	Ino80b	INO80 complex subunit B [Source:MGI Symbol;Acc:MGI:1917270]
ENSMUSG00000030035	71.23654957	60.48031408	75.63601751	53.8583069	49.81853187	38.26477748	30.85979952	29.27477574	24.5045797	24.95109579	25.56750945	22.81625411	Wbp1	WW domain binding protein 1 [Source:MGI Symbol;Acc:MGI:104710]
ENSMUSG00000030036	37.01642429	35.15955814	36.37108417	37.32511508	36.62082601	32.81186593	22.91139969	22.83879138	20.81076003	20.44395442	19.21124557	19.41871936	Mogs	mannosyl-oligosaccharide glucosidase [Source:MGI Symbol;Acc:MGI:1929872]
ENSMUSG00000030037	0	0	0	0	0	0	0	0	0	0	0	0	Mrpl53	mitochondrial ribosomal protein L53 [Source:MGI Symbol;Acc:MGI:1915749]
ENSMUSG00000030041	10.90233752	9.261591705	11.80216049	11.67494683	12.44050297	9.120911247	7.898381211	8.498611379	8.151229178	13.66862357	10.45123781	12.03780169	M1ap	meiosis 1 associated protein [Source:MGI Symbol;Acc:MGI:1315200]
ENSMUSG00000030042	43.02848783	40.15624252	45.56589387	53.47453683	42.74435787	40.9675258	111.0116458	111.7665888	99.41110479	79.43843296	76.81107182	78.75077355	Pole4	polymerase (DNA-directed), epsilon 4 (p12 subunit) [Source:MGI Symbol;Acc:MGI:1914229]
ENSMUSG00000030043	0.030734634	0.011797504	0	0	0.021527771	0.023410015	0	0	0.028606537	0.009163802	0.017055655	0	Tacr1	tachykinin receptor 1 [Source:MGI Symbol;Acc:MGI:98475]
ENSMUSG00000030045	14.44078031	13.00372654	15.2571208	16.43354532	16.0985719	14.05286056	26.17831763	26.01216365	24.53743137	24.68862233	21.78714328	22.15428755	Mrpl19	mitochondrial ribosomal protein L19 [Source:MGI Symbol;Acc:MGI:1926274]
ENSMUSG00000030046	0.009583182	0	0	0	0	0	0	0	0	0	0.007977032	0.025478555	Bmp10	bone morphogenetic protein 10 [Source:MGI Symbol;Acc:MGI:1338820]
ENSMUSG00000030047	0.467350617	1.132030132	1.072433057	0.195503825	0.101591727	0.110474227	0.100713101	0.095788422	0.089998093	1.369422024	1.086579368	1.071149008	Arhgap25	Rho GTPase activating protein 25 [Source:MGI Symbol;Acc:MGI:2443687]
ENSMUSG00000030048	0	0	0	0	0	0.076574815	0	0	0.046786392	0.044962578	0	0	Gkn3	gastrokine 3 [Source:MGI Symbol;Acc:MGI:1916138]
ENSMUSG00000030049	0	0	0	0	0	0	0	0	0	0	0	0	Gkn2	gastrokine 2 [Source:MGI Symbol;Acc:MGI:1913534]
ENSMUSG00000030050	0	0	0	0.064245565	0.072333309	0	0	0	0	0.092371119	0	0	Gkn1	gastrokine 1 [Source:MGI Symbol;Acc:MGI:1913533]
ENSMUSG00000030051	5.570577082	5.384549167	5.513012088	6.175034646	5.905990589	4.770169343	5.380189448	5.066940703	6.221837585	6.356780637	5.269253945	6.343017492	Aplf	aprataxin and PNKP like factor [Source:MGI Symbol;Acc:MGI:1919353]
ENSMUSG00000030054	0	0	0	0	0	0	0	0	0	0	0.081309592	0	Gp9	glycoprotein 9 (platelet) [Source:MGI Symbol;Acc:MGI:1860137]
ENSMUSG00000030055	0.449618473	1.009629629	0.713489946	0.440555399	0.708594331	1.318495083	0.18063411	0.111352789	0.272016181	0.864672181	1.253777773	0.77700276	Rab43	RAB43, member RAS oncogene family [Source:MGI Symbol;Acc:MGI:1917084]
ENSMUSG00000030056	15.54989907	14.10444133	17.08296831	13.45075248	15.18134123	12.8446038	27.57501901	28.48757148	24.94811007	16.8623021	16.22398072	16.32918938	Isy1	ISY1 splicing factor homolog [Source:MGI Symbol;Acc:MGI:1923310]
ENSMUSG00000030057	326.3367728	314.8720337	307.8405383	375.8628827	363.8899067	329.6695265	481.6741799	480.7001335	464.1762316	375.8169235	375.7569224	389.0579218	Cnbp	cellular nucleic acid binding protein [Source:MGI Symbol;Acc:MGI:88431]
ENSMUSG00000030058	137.928593	152.6381757	135.257275	140.1528204	145.0688098	148.387331	82.79958411	74.59523348	86.63786044	119.4947344	126.0649148	119.8738777	Copg1	coatomer protein complex, subunit gamma 1 [Source:MGI Symbol;Acc:MGI:1858696]
ENSMUSG00000030059	14.75730926	19.27537524	13.0939683	18.45096298	21.64945826	25.75919374	8.746729238	8.636167302	9.424077891	16.90341616	16.28763598	17.76467805	Tmf1	TATA element modulatory factor 1 [Source:MGI Symbol;Acc:MGI:2684999]
ENSMUSG00000030060	49.58128203	41.00103689	58.64250559	49.47558451	41.35670443	38.07929341	354.8157927	384.6767129	322.9096125	103.4953302	109.815532	114.1144432	Hmces	5-hydroxymethylcytosine (hmC) binding, ES cell specific [Source:MGI Symbol;Acc:MGI:1914053]
[truncated: 9,325,591 more chars]
